# Supplementary material for: DMRforPairs: identifying Differentially Methylated Regions between unique samples using array based methylation profiles
Source: BMC Bioinformatics. 2014 May 15;15:141. doi: 10.1186/1471-2105-15-141 (PMC4046028; doi:10.1186/1471-2105-15-141)
Supplement: Additional file 2 — DMRforPairs output for the comparison of A431-MCF7 and NA17018-NA17105. Please start from the HTML files in each folder. Available via the BMC Bioinformatics website. [file 1471-2105-15-141-S2.zip › 1394847754114233_MOESM2_ESM/NA17105M_NA17018F/all.html]

 
 
 DMRforPairs output generated on Tue Nov 19 09:30:45 2013. Identified regions were set to contain at least 4 probes with a maximum distance of 200 bp between individual probes (n=29404). Regions in which median methylation levels (M-values) between the samples differed at least |1.4| (n=4146) (=relevant) were tested for statistical significance (significant: p 2). p.adj denotes the multiple testing corrected p-value (method:fdr). Gene symbols of overlapping transcripts are listed for the exact region and within a margin of 10000 bp of the region. 

	  
	  
		 Chr   
		 Start   
		 End   
		 Links   
		 Length   
		 n   
		 ID   
		 Class   
		 NA17105-M   
		 NA17018-F   
		 Gene.Symbol   
		 dM   
		 p   
		 p.adj  
	  
  
 20
 
  36148133
 
  36149455
 
  PDF   STATS   ENSEMBL   UCSC 
 
 1323
 
 41
 
 25416
 
 gene;tss;island
 
 0.977346
 
 0.5178004
 
 NNAT; BLCAP (margin: NNAT; PPIAP3; RP11-425M5.5; BLCAP)
 
  5.2147358
 
 1.28e-11
 
 0.0000000529
  
 
  
 7
 
  27183133
 
  27184853
 
  PDF   STATS   ENSEMBL   UCSC 
 
 1721
 
 47
 
 38347
 
 island
 
 0.452023
 
 0.9059644
 
 HOXA-AS3; HOXA3; HOXA5; HOXA6 (margin: HOXA-AS2; HOXA-AS3; HOXA3; HOXA5; HOXA6; HOXA7)
 
  3.5043451
 
 3.47e-11
 
 0.0000000719
  
 
  
 7
 
  27183369
 
  27184737
 
  PDF   STATS   ENSEMBL   UCSC 
 
 1369
 
 41
 
 37492
 
 tss
 
 0.448084
 
 0.8855557
 
 HOXA-AS3; HOXA3; HOXA5; HOXA6 (margin: HOXA-AS2; HOXA-AS3; HOXA3; HOXA5; HOXA6; HOXA7)
 
  3.1622571
 
 1.30e-09
 
 0.0000017973
  
 
  
 6
 
  31148332
 
  31148748
 
  PDF   STATS   ENSEMBL   UCSC 
 
  417
 
 16
 
 35754
 
 island
 
 0.535283
 
 0.9640636
 
 POU5F1 (margin: POU5F1; PSORS1C3)
 
  4.9834446
 
 3.33e-09
 
 0.0000034488
  
 
  
 6
 
  32551749
 
  32552670
 
  PDF   STATS   ENSEMBL   UCSC 
 
  922
 
 17
 
 34122
 
 gene;island
 
 0.062812
 
 0.9760723
 
 HLA-DRB1 (margin: HLA-DRB1)
 
  6.9892823
 
 8.31e-08
 
 0.0000689340
  
 
  
 6
 
  29893926
 
  29894341
 
  PDF   STATS   ENSEMBL   UCSC 
 
  416
 
 16
 
 34757
 
 tss;island
 
 0.021777
 
 0.4924359
 
 HLA-K; HCG4B (margin: HLA-K; HLA-U; HCG4B)
 
  5.3944350
 
 4.63e-07
 
 0.0003195879
  
 
  
 6
 
  31164743
 
  31164994
 
  PDF   STATS   ENSEMBL   UCSC 
 
  252
 
 12
 
 34803
 
 tss;island
 
 0.529376
 
 0.0106260
 
 HCG27; XXbac-BPG299F13.17 (margin: HCG27; XXbac-BPG299F13.17; XXbac-BPG299F13.14)
 
  6.2228861
 
 2.96e-06
 
 0.0017522235
  
 
  
 11
 
   2160540
 
   2161586
 
  PDF   STATS   ENSEMBL   UCSC 
 
 1047
 
 32
 
 6447
 
 gene;tss;island
 
 0.026258
 
 0.1457218
 
 IGF2-AS; IGF2; INS-IGF2 (margin: IGF2-AS; IGF2; AC132217.4; INS-IGF2; MIR483)
 
  2.6092460
 
 5.59e-06
 
 0.0028957612
  
 
  
 6
 
  32846924
 
  32847845
 
  PDF   STATS   ENSEMBL   UCSC 
 
  922
 
 27
 
 34160
 
 gene;island
 
 0.968773
 
 0.4912706
 
 PPP1R2P1 (margin: PPP1R2P1)
 
  3.9211949
 
 1.74e-05
 
 0.0080291795
  
 
  
 19
 
  52391078
 
  52391789
 
  PDF   STATS   ENSEMBL   UCSC 
 
  712
 
 13
 
 22344
 
 island
 
 0.970656
 
 0.5902092
 
 CTC-429C10.2; ZNF577; ZNF649 (margin: CTC-429C10.2; ZNF577; ZNF649)
 
  4.3996932
 
 2.67e-05
 
 0.0110819376
  
 
  
 13
 
  25670047
 
  25670238
 
  PDF   STATS   ENSEMBL   UCSC 
 
  192
 
  9
 
 11349
 
 tss
 
 0.531597
 
 0.1361886
 
 PABPC3 (margin: PABPC3)
 
  2.8557545
 
 4.11e-05
 
 0.0155042818
  
 
  
 6
 
  29599012
 
  29599390
 
  PDF   STATS   ENSEMBL   UCSC 
 
  379
 
 10
 
 33681
 
 gene;island
 
 0.522468
 
 0.9516596
 
 GABBR1 (margin: GABBR1; SUMO2P1)
 
  4.1416012
 
 7.58e-05
 
 0.0209443807
  
 
  
 19
 
  34174665
 
  34175419
 
  PDF   STATS   ENSEMBL   UCSC 
 
  755
 
 19
 
 20688
 
 tss
 
 0.029174
 
 0.4286653
 
 CHST8 (margin: CHST8)
 
  3.9832305
 
 6.75e-05
 
 0.0209443807
  
 
  
 5
 
 135415693
 
 135416613
 
  PDF   STATS   ENSEMBL   UCSC 
 
  921
 
 16
 
 33146
 
 island
 
 0.843647
 
 0.4093294
 
 VTRNA2-1 (margin: VTRNA2-1)
 
  3.7528664
 
 7.07e-05
 
 0.0209443807
  
 
  
 20
 
  13200929
 
  13200992
 
  PDF   STATS   ENSEMBL   UCSC 
 
   64
 
 10
 
 25601
 
 tss
 
 0.034365
 
 0.1740090
 
 ISM1 (margin: ISM1)
 
  3.3903740
 
 7.58e-05
 
 0.0209443807
  
 
  
 13
 
  25670047
 
  25670327
 
  PDF   STATS   ENSEMBL   UCSC 
 
  281
 
 11
 
 11609
 
 island
 
 0.505984
 
 0.1361886
 
 PABPC3 (margin: PABPC3)
 
  2.8165856
 
 8.51e-05
 
 0.0220396863
  
 
  
 19
 
  34174665
 
  34175554
 
  PDF   STATS   ENSEMBL   UCSC 
 
  890
 
 21
 
 19974
 
 gene;island
 
 0.029174
 
 0.4286653
 
 CHST8 (margin: CHST8)
 
  2.6680052
 
 9.39e-05
 
 0.0229076809
  
 
  
 20
 
  13200685
 
  13200992
 
  PDF   STATS   ENSEMBL   UCSC 
 
  308
 
 12
 
 26007
 
 island
 
 0.026418
 
 0.1161884
 
 ISM1 (margin: ISM1)
 
  3.3903740
 
 1.03e-04
 
 0.0236793538
  
 
  
 19
 
  23945472
 
  23945996
 
  PDF   STATS   ENSEMBL   UCSC 
 
  525
 
  8
 
 21849
 
 island
 
 0.014871
 
 0.9137979
 
 RP11-255H23.2; RPSAP58 (margin: RP11-255H23.2; RPSAP58; ZNF681)
 
  8.7548589
 
 1.55e-04
 
 0.0296603652
  
 
  
 5
 
   1594579
 
   1595048
 
  PDF   STATS   ENSEMBL   UCSC 
 
  470
 
  9
 
 32635
 
 island
 
 0.495912
 
 0.0131407
 
 CTD-2012J19.3; SDHAP3 (margin: CTD-2012J19.3; CTD-2012J19.2; SDHAP3; RP11-43F13.1)
 
  6.2725591
 
 1.65e-04
 
 0.0296603652
  
 
  
 20
 
  55841869
 
  55842071
 
  PDF   STATS   ENSEMBL   UCSC 
 
  203
 
  8
 
 25840
 
 tss;island
 
 0.456524
 
 0.0190137
 
 RP4-813D12.3; BMP7 (margin: RP4-813D12.3; BMP7)
 
  5.7289099
 
 1.55e-04
 
 0.0296603652
  
 
  
 6
 
  28058715
 
  28058918
 
  PDF   STATS   ENSEMBL   UCSC 
 
  204
 
  8
 
 34731
 
 tss
 
 0.495596
 
 0.9442076
 
 ZNF165; ZSCAN12P1 (margin: ZNF165; ZSCAN12P1; RP1-313I6.12)
 
  4.3722615
 
 1.55e-04
 
 0.0296603652
  
 
  
 18
 
  12254365
 
  12254690
 
  PDF   STATS   ENSEMBL   UCSC 
 
  326
 
  9
 
 19060
 
 gene
 
 0.045075
 
 0.3233203
 
 CIDEA (margin: RP11-64C12.9; CIDEA)
 
  2.7719571
 
 1.65e-04
 
 0.0296603652
  
 
  
 15
 
  74421523
 
  74421935
 
  PDF   STATS   ENSEMBL   UCSC 
 
  413
 
 12
 
 13789
 
 tss;island
 
 0.058561
 
 0.0090383
 
 ISLR2; RP11-247C2.2 (margin: ISLR2; RP11-247C2.2)
 
  3.4314793
 
 2.74e-04
 
 0.0455223165
  
 
  
 11
 
    368351
 
    369192
 
  PDF   STATS   ENSEMBL   UCSC 
 
  842
 
 24
 
 7009
 
 tss
 
 0.033820
 
 0.3225201
 
 B4GALNT4 (margin: B4GALNT4)
 
  2.8029077
 
 2.74e-04
 
 0.0455223165
  
 
  
 12
 
   9217390
 
   9217769
 
  PDF   STATS   ENSEMBL   UCSC 
 
  380
 
  8
 
 9556
 
 tss
 
 0.024435
 
 0.4924536
 
 A2M-AS1; LINC00612 (margin: A2M-AS1; LINC00612; A2M)
 
  5.6276974
 
 3.11e-04
 
 0.0495606957
  
 
  
 19
 
  52391234
 
  52391789
 
  PDF   STATS   ENSEMBL   UCSC 
 
  556
 
 10
 
 20999
 
 tss
 
 0.975093
 
 0.6137836
 
 CTC-429C10.2; ZNF577; ZNF649 (margin: CTC-429C10.2; ZNF577; ZNF649)
 
  4.5614173
 
 3.25e-04
 
 0.0498675731
  
 
  
 1
 
 229569608
 
 229570288
 
  PDF   STATS   ENSEMBL   UCSC 
 
  681
 
 14
 
 4310
 
 island
 
 0.068560
 
 0.3194111
 
 ACTA1 (margin: RP5-1068B5.1; ACTA1; RP5-1068B5.3; NUP133)
 
  2.6988347
 
 3.38e-04
 
 0.0499764971
  
 
  
 1
 
 110254662
 
 110254854
 
  PDF   STATS   ENSEMBL   UCSC 
 
  193
 
  7
 
 1810
 
 tss
 
 0.011935
 
 0.9485183
 
 Not queried
 
 10.2411497
 
 5.83e-04
 
 0.0548331102
  
 
  
 17
 
  37894014
 
  37894413
 
  PDF   STATS   ENSEMBL   UCSC 
 
  400
 
  7
 
 17316
 
 tss
 
 0.010675
 
 0.7647515
 
 Not queried
 
  8.1521652
 
 5.83e-04
 
 0.0548331102
  
 
  
 5
 
  74162621
 
  74162924
 
  PDF   STATS   ENSEMBL   UCSC 
 
  304
 
  7
 
 32127
 
 tss
 
 0.022193
 
 0.9340233
 
 Not queried
 
  6.6220625
 
 5.83e-04
 
 0.0548331102
  
 
  
 7
 
   4751840
 
   4752170
 
  PDF   STATS   ENSEMBL   UCSC 
 
  331
 
  7
 
 36842
 
 gene
 
 0.486500
 
 0.9872781
 
 Not queried
 
  6.4534127
 
 5.83e-04
 
 0.0548331102
  
 
  
 22
 
  42078217
 
  42078723
 
  PDF   STATS   ENSEMBL   UCSC 
 
  507
 
  7
 
 26883
 
 gene;island
 
 0.499550
 
 0.0106589
 
 Not queried
 
  6.0950439
 
 5.83e-04
 
 0.0548331102
  
 
  
 5
 
   1594676
 
   1595048
 
  PDF   STATS   ENSEMBL   UCSC 
 
  373
 
  8
 
 31971
 
 tss
 
 0.495503
 
 0.0152052
 
 Not queried
 
  6.0465787
 
 6.22e-04
 
 0.0548331102
  
 
  
 1
 
  67772896
 
  67773175
 
  PDF   STATS   ENSEMBL   UCSC 
 
  280
 
  8
 
 3365
 
 island
 
 0.009940
 
 0.3705543
 
 Not queried
 
  5.8201565
 
 6.22e-04
 
 0.0548331102
  
 
  
 6
 
  28226885
 
  28227093
 
  PDF   STATS   ENSEMBL   UCSC 
 
  209
 
  7
 
 34738
 
 tss
 
 0.457823
 
 0.0277058
 
 Not queried
 
  4.6872529
 
 5.83e-04
 
 0.0548331102
  
 
  
 7
 
   5111488
 
   5111669
 
  PDF   STATS   ENSEMBL   UCSC 
 
  182
 
  8
 
 37420
 
 tss;island
 
 0.474622
 
 0.0182271
 
 Not queried
 
  4.6513489
 
 6.22e-04
 
 0.0548331102
  
 
  
 21
 
  34398085
 
  34398265
 
  PDF   STATS   ENSEMBL   UCSC 
 
  181
 
  8
 
 26642
 
 island
 
 0.012381
 
 0.1706910
 
 Not queried
 
  4.5537075
 
 6.22e-04
 
 0.0548331102
  
 
  
 1
 
 111743020
 
 111743537
 
  PDF   STATS   ENSEMBL   UCSC 
 
  518
 
  7
 
 3588
 
 island
 
 0.493765
 
 0.9550531
 
 Not queried
 
  4.5447115
 
 5.83e-04
 
 0.0548331102
  
 
  
 6
 
 136871881
 
 136872119
 
  PDF   STATS   ENSEMBL   UCSC 
 
  239
 
  8
 
 35242
 
 tss
 
 0.167717
 
 0.0100143
 
 Not queried
 
  4.4926808
 
 6.22e-04
 
 0.0548331102
  
 
  
 2
 
  27665017
 
  27665306
 
  PDF   STATS   ENSEMBL   UCSC 
 
  290
 
  7
 
 24185
 
 island
 
 0.669726
 
 0.9765713
 
 Not queried
 
  4.4676767
 
 5.83e-04
 
 0.0548331102
  
 
  
 10
 
 124638756
 
 124639410
 
  PDF   STATS   ENSEMBL   UCSC 
 
  655
 
 16
 
 4799
 
 gene;tss;island
 
 0.009031
 
 0.0898760
 
 Not queried
 
  3.3954946
 
 4.67e-04
 
 0.0548331102
  
 
  
 17
 
  77758370
 
  77758850
 
  PDF   STATS   ENSEMBL   UCSC 
 
  481
 
  7
 
 16843
 
 gene;island
 
 0.682096
 
 0.9346099
 
 Not queried
 
  3.2966923
 
 5.83e-04
 
 0.0548331102
  
 
  
 22
 
  45148218
 
  45148385
 
  PDF   STATS   ENSEMBL   UCSC 
 
  168
 
  9
 
 27215
 
 tss
 
 0.011300
 
 0.1620448
 
 Not queried
 
  3.2020895
 
 4.94e-04
 
 0.0548331102
  
 
  
 18
 
  23713407
 
  23714084
 
  PDF   STATS   ENSEMBL   UCSC 
 
  678
 
 10
 
 19440
 
 island
 
 0.842550
 
 0.5055680
 
 Not queried
 
  2.9643836
 
 4.87e-04
 
 0.0548331102
  
 
  
 1
 
    854766
 
    855046
 
  PDF   STATS   ENSEMBL   UCSC 
 
  281
 
  7
 
 2379
 
 island
 
 0.316510
 
 0.7244037
 
 Not queried
 
  2.1867081
 
 5.83e-04
 
 0.0548331102
  
 
  
 6
 
  29589415
 
  29590143
 
  PDF   STATS   ENSEMBL   UCSC 
 
  729
 
 14
 
 33675
 
 gene
 
 0.298318
 
 0.7025327
 
 Not queried
 
  1.6454070
 
 4.21e-04
 
 0.0548331102
  
 
  
 7
 
  27170241
 
  27171528
 
  PDF   STATS   ENSEMBL   UCSC 
 
 1288
 
 22
 
 38345
 
 island
 
 0.873619
 
 0.2479343
 
 Not queried
 
  4.2266755
 
 8.09e-04
 
 0.0698560014
  
 
  
 17
 
  72931569
 
  72932048
 
  PDF   STATS   ENSEMBL   UCSC 
 
  480
 
 12
 
 18731
 
 island
 
 0.071701
 
 0.4133572
 
 Not queried
 
  3.6287355
 
 8.58e-04
 
 0.0725921168
  
 
  
 7
 
  27281216
 
  27281964
 
  PDF   STATS   ENSEMBL   UCSC 
 
  749
 
 14
 
 37500
 
 tss;island
 
 0.032387
 
 0.5377164
 
 Not queried
 
  3.2871315
 
 9.71e-04
 
 0.0805458728
  
 
  
 12
 
   9217390
 
   9218039
 
  PDF   STATS   ENSEMBL   UCSC 
 
  650
 
 11
 
 10235
 
 island
 
 0.025855
 
 0.4992826
 
 Not queried
 
  5.3833778
 
 1.04e-03
 
 0.0821403610
  
 
  
 22
 
  45148218
 
  45148489
 
  PDF   STATS   ENSEMBL   UCSC 
 
  272
 
 10
 
 26896
 
 gene
 
 0.012492
 
 0.1469768
 
 Not queried
 
  2.9660340
 
 1.05e-03
 
 0.0821403610
  
 
  
 1
 
  55007670
 
  55008516
 
  PDF   STATS   ENSEMBL   UCSC 
 
  847
 
 11
 
 3299
 
 island
 
 0.045250
 
 0.0204079
 
 Not queried
 
  2.0044737
 
 1.04e-03
 
 0.0821403610
  
 
  
 8
 
  86350889
 
  86351195
 
  PDF   STATS   ENSEMBL   UCSC 
 
  307
 
  7
 
 40432
 
 island
 
 0.019367
 
 0.5792245
 
 Not queried
 
  6.2835823
 
 1.17e-03
 
 0.0833132385
  
 
  
 19
 
  13124604
 
  13125281
 
  PDF   STATS   ENSEMBL   UCSC 
 
  678
 
  7
 
 19875
 
 gene;island
 
 0.008964
 
 0.2293308
 
 Not queried
 
  5.7591695
 
 1.17e-03
 
 0.0833132385
  
 
  
 11
 
    705692
 
    706100
 
  PDF   STATS   ENSEMBL   UCSC 
 
  409
 
  7
 
 7024
 
 tss;island
 
 0.046495
 
 0.6118280
 
 Not queried
 
  5.0207212
 
 1.17e-03
 
 0.0833132385
  
 
  
 19
 
  53757910
 
  53758309
 
  PDF   STATS   ENSEMBL   UCSC 
 
  400
 
  7
 
 22371
 
 island
 
 0.255299
 
 0.0133766
 
 Not queried
 
  4.9358056
 
 1.17e-03
 
 0.0833132385
  
 
  
 15
 
  69744390
 
  69745085
 
  PDF   STATS   ENSEMBL   UCSC 
 
  696
 
 12
 
 13764
 
 tss
 
 0.016066
 
 0.0044421
 
 Not queried
 
  3.1609953
 
 1.11e-03
 
 0.0833132385
  
 
  
 5
 
  74162458
 
  74162924
 
  PDF   STATS   ENSEMBL   UCSC 
 
  467
 
  9
 
 32881
 
 island
 
 0.023500
 
 0.6888514
 
 Not queried
 
  6.1357606
 
 1.23e-03
 
 0.0862637156
  
 
  
 1
 
 161993364
 
 161994363
 
  PDF   STATS   ENSEMBL   UCSC 
 
 1000
 
 13
 
 3915
 
 island
 
 0.042535
 
 0.0100612
 
 Not queried
 
  1.7061090
 
 1.25e-03
 
 0.0862637156
  
 
  
 10
 
  63809073
 
  63809170
 
  PDF   STATS   ENSEMBL   UCSC 
 
   98
 
  6
 
 4600
 
 gene
 
 0.019912
 
 0.9700297
 
 Not queried
 
 11.2311183
 
 2.16e-03
 
 0.0934794372
  
 
  
 19
 
  23945472
 
  23945761
 
  PDF   STATS   ENSEMBL   UCSC 
 
  290
 
  6
 
 20666
 
 tss
 
 0.018089
 
 0.9405765
 
 Not queried
 
  9.7164869
 
 2.16e-03
 
 0.0934794372
  
 
  
 2
 
  37571677
 
  37571901
 
  PDF   STATS   ENSEMBL   UCSC 
 
  225
 
  6
 
 24223
 
 island
 
 0.022218
 
 0.6348199
 
 Not queried
 
  7.9142517
 
 2.16e-03
 
 0.0934794372
  
 
  
 17
 
   1617102
 
   1617465
 
  PDF   STATS   ENSEMBL   UCSC 
 
  364
 
  6
 
 16318
 
 gene;island
 
 0.011185
 
 0.4151169
 
 Not queried
 
  6.2665678
 
 2.16e-03
 
 0.0934794372
  
 
  
 11
 
   2321770
 
   2322050
 
  PDF   STATS   ENSEMBL   UCSC 
 
  281
 
  9
 
 6453
 
 gene;tss
 
 0.007924
 
 0.2786334
 
 Not queried
 
  5.9779360
 
 1.85e-03
 
 0.0934794372
  
 
  
 17
 
  40575462
 
  40575582
 
  PDF   STATS   ENSEMBL   UCSC 
 
  121
 
  6
 
 17362
 
 tss
 
 0.011147
 
 0.3610249
 
 Not queried
 
  5.7697381
 
 2.16e-03
 
 0.0934794372
  
 
  
 5
 
   8457548
 
   8458089
 
  PDF   STATS   ENSEMBL   UCSC 
 
  542
 
  6
 
 32684
 
 island
 
 0.011357
 
 0.2245731
 
 Not queried
 
  5.3735231
 
 2.16e-03
 
 0.0934794372
  
 
  
 10
 
  94820892
 
  94820988
 
  PDF   STATS   ENSEMBL   UCSC 
 
   97
 
  6
 
 5233
 
 tss
 
 0.625951
 
 0.9798912
 
 Not queried
 
  5.1776571
 
 2.16e-03
 
 0.0934794372
  
 
  
 3
 
    350503
 
    351003
 
  PDF   STATS   ENSEMBL   UCSC 
 
  501
 
  6
 
 27637
 
 gene;island
 
 0.974273
 
 0.5238795
 
 Not queried
 
  5.1254069
 
 2.16e-03
 
 0.0934794372
  
 
  
 1
 
 236228598
 
 236228837
 
  PDF   STATS   ENSEMBL   UCSC 
 
  240
 
  6
 
 2339
 
 tss;island
 
 0.494590
 
 0.0239711
 
 Not queried
 
  5.0676791
 
 2.16e-03
 
 0.0934794372
  
 
  
 12
 
   6420020
 
   6420052
 
  PDF   STATS   ENSEMBL   UCSC 
 
   33
 
  6
 
 9023
 
 gene;tss;island
 
 0.283022
 
 0.0171517
 
 Not queried
 
  4.9416359
 
 2.16e-03
 
 0.0934794372
  
 
  
 6
 
  76203530
 
  76203675
 
  PDF   STATS   ENSEMBL   UCSC 
 
  146
 
  6
 
 35077
 
 tss
 
 0.439292
 
 0.8551064
 
 Not queried
 
  4.6442080
 
 2.16e-03
 
 0.0934794372
  
 
  
 1
 
 181451906
 
 181452137
 
  PDF   STATS   ENSEMBL   UCSC 
 
  232
 
  6
 
 2129
 
 tss;island
 
 0.010685
 
 0.1513533
 
 Not queried
 
  4.5011521
 
 2.16e-03
 
 0.0934794372
  
 
  
 6
 
 157931791
 
 157932180
 
  PDF   STATS   ENSEMBL   UCSC 
 
  390
 
  6
 
 34524
 
 gene
 
 0.494312
 
 0.9437816
 
 Not queried
 
  4.4177557
 
 2.16e-03
 
 0.0934794372
  
 
  
 1
 
   1266468
 
   1266781
 
  PDF   STATS   ENSEMBL   UCSC 
 
  314
 
  6
 
 2426
 
 island
 
 0.875268
 
 0.2396280
 
 Not queried
 
  4.4108652
 
 2.16e-03
 
 0.0934794372
  
 
  
 2
 
  27665017
 
  27665150
 
  PDF   STATS   ENSEMBL   UCSC 
 
  134
 
  6
 
 23270
 
 tss
 
 0.681965
 
 0.9748718
 
 Not queried
 
  4.3942236
 
 2.16e-03
 
 0.0934794372
  
 
  
 14
 
  23305548
 
  23305957
 
  PDF   STATS   ENSEMBL   UCSC 
 
  410
 
  6
 
 12703
 
 island
 
 0.093865
 
 0.6877662
 
 Not queried
 
  4.2523494
 
 2.16e-03
 
 0.0934794372
  
 
  
 6
 
 136871667
 
 136872119
 
  PDF   STATS   ENSEMBL   UCSC 
 
  453
 
 10
 
 36501
 
 island
 
 0.115338
 
 0.0100143
 
 Not queried
 
  4.1383574
 
 1.50e-03
 
 0.0934794372
  
 
  
 17
 
  72931569
 
  72931857
 
  PDF   STATS   ENSEMBL   UCSC 
 
  289
 
 10
 
 17637
 
 tss
 
 0.057973
 
 0.3615983
 
 Not queried
 
  4.0922883
 
 1.50e-03
 
 0.0934794372
  
 
  
 12
 
  25055108
 
  25055676
 
  PDF   STATS   ENSEMBL   UCSC 
 
  569
 
  8
 
 9083
 
 gene;island
 
 0.169279
 
 0.7062306
 
 Not queried
 
  3.9764033
 
 1.86e-03
 
 0.0934794372
  
 
  
 3
 
 128779498
 
 128779601
 
  PDF   STATS   ENSEMBL   UCSC 
 
  104
 
  6
 
 28576
 
 tss
 
 0.021824
 
 0.2683000
 
 Not queried
 
  3.9603548
 
 2.16e-03
 
 0.0934794372
  
 
  
 22
 
  30476089
 
  30476525
 
  PDF   STATS   ENSEMBL   UCSC 
 
  437
 
 11
 
 27404
 
 island
 
 0.115289
 
 0.6566366
 
 Not queried
 
  3.9411687
 
 1.86e-03
 
 0.0934794372
  
 
  
 1
 
   1369466
 
   1369948
 
  PDF   STATS   ENSEMBL   UCSC 
 
  483
 
  6
 
 1152
 
 tss;island
 
 0.030071
 
 0.3479041
 
 Not queried
 
  3.8633523
 
 2.16e-03
 
 0.0934794372
  
 
  
 12
 
  29650692
 
  29651041
 
  PDF   STATS   ENSEMBL   UCSC 
 
  350
 
  6
 
 9623
 
 tss
 
 0.317257
 
 0.6312738
 
 Not queried
 
  3.3778078
 
 2.16e-03
 
 0.0934794372
  
 
  
 6
 
  25652381
 
  25652407
 
  PDF   STATS   ENSEMBL   UCSC 
 
   27
 
  6
 
 34674
 
 tss
 
 0.495935
 
 0.9140510
 
 Not queried
 
  3.3236596
 
 2.16e-03
 
 0.0934794372
  
 
  
 6
 
  30078954
 
  30079662
 
  PDF   STATS   ENSEMBL   UCSC 
 
  709
 
 21
 
 33724
 
 gene
 
 0.094032
 
 0.4893740
 
 Not queried
 
  3.2414713
 
 1.48e-03
 
 0.0934794372
  
 
  
 17
 
  76037035
 
  76037562
 
  PDF   STATS   ENSEMBL   UCSC 
 
  528
 
  6
 
 16812
 
 gene;island
 
 0.659963
 
 0.9328694
 
 Not queried
 
  3.0934316
 
 2.16e-03
 
 0.0934794372
  
 
  
 2
 
 172950067
 
 172950171
 
  PDF   STATS   ENSEMBL   UCSC 
 
  105
 
  6
 
 23717
 
 tss
 
 0.007012
 
 0.0017025
 
 Not queried
 
  3.0362832
 
 2.16e-03
 
 0.0934794372
  
 
  
 7
 
  50861418
 
  50862266
 
  PDF   STATS   ENSEMBL   UCSC 
 
  849
 
 20
 
 37598
 
 tss;island
 
 0.067392
 
 0.6083027
 
 Not queried
 
  2.9908465
 
 1.59e-03
 
 0.0934794372
  
 
  
 2
 
   3642098
 
   3642967
 
  PDF   STATS   ENSEMBL   UCSC 
 
  870
 
 11
 
 24035
 
 island
 
 0.960428
 
 0.7937236
 
 Not queried
 
  2.8857211
 
 1.86e-03
 
 0.0934794372
  
 
  
 4
 
 165898825
 
 165898967
 
  PDF   STATS   ENSEMBL   UCSC 
 
  143
 
  6
 
 30666
 
 tss
 
 0.440492
 
 0.7202763
 
 Not queried
 
  2.8843316
 
 2.16e-03
 
 0.0934794372
  
 
  
 1
 
 240656217
 
 240656737
 
  PDF   STATS   ENSEMBL   UCSC 
 
  521
 
  6
 
 1106
 
 gene;island
 
 0.470493
 
 0.8323197
 
 Not queried
 
  2.7633745
 
 2.16e-03
 
 0.0934794372
  
 
  
 8
 
  68864012
 
  68864549
 
  PDF   STATS   ENSEMBL   UCSC 
 
  538
 
  9
 
 39751
 
 tss;island
 
 0.024682
 
 0.1646819
 
 Not queried
 
  2.5133379
 
 1.85e-03
 
 0.0934794372
  
 
  
 1
 
    854824
 
    855046
 
  PDF   STATS   ENSEMBL   UCSC 
 
  223
 
  6
 
 1132
 
 tss
 
 0.327425
 
 0.6559519
 
 Not queried
 
  1.9487703
 
 2.16e-03
 
 0.0934794372
  
 
  
 18
 
  12253749
 
  12254690
 
  PDF   STATS   ENSEMBL   UCSC 
 
  942
 
 19
 
 19401
 
 island
 
 0.053031
 
 0.1853589
 
 Not queried
 
  1.7155954
 
 1.99e-03
 
 0.0934794372
  
 
  
 7
 
 157567895
 
 157568392
 
  PDF   STATS   ENSEMBL   UCSC 
 
  498
 
  6
 
 37344
 
 gene;island
 
 0.392202
 
 0.6570615
 
 Not queried
 
  1.4971814
 
 2.16e-03
 
 0.0934794372
  
 
  
 17
 
  40575289
 
  40575582
 
  PDF   STATS   ENSEMBL   UCSC 
 
  294
 
  7
 
 18337
 
 island
 
 0.012102
 
 0.2966170
 
 Not queried
 
  5.1214319
 
 2.33e-03
 
 0.0974014615
  
 
  
 8
 
  23567310
 
  23567941
 
  PDF   STATS   ENSEMBL   UCSC 
 
  632
 
  7
 
 40157
 
 island
 
 0.052989
 
 0.2589032
 
 Not queried
 
  4.0115340
 
 2.33e-03
 
 0.0974014615
  
 
  
 7
 
 149569715
 
 149570367
 
  PDF   STATS   ENSEMBL   UCSC 
 
  653
 
 13
 
 37268
 
 gene;island
 
 0.016894
 
 0.4575367
 
 Not queried
 
  3.4259789
 
 2.35e-03
 
 0.0974014615
  
 
  
 10
 
    530635
 
    531584
 
  PDF   STATS   ENSEMBL   UCSC 
 
  950
 
 12
 
 4441
 
 gene;island
 
 0.234958
 
 0.5569539
 
 Not queried
 
  3.0739682
 
 2.32e-03
 
 0.0974014615
  
 
  
 6
 
  94129234
 
  94129697
 
  PDF   STATS   ENSEMBL   UCSC 
 
  464
 
 10
 
 36331
 
 island
 
 0.032464
 
 0.1218326
 
 Not queried
 
  2.3793400
 
 2.88e-03
 
 0.1170421274
  
 
  
 1
 
 186649153
 
 186649985
 
  PDF   STATS   ENSEMBL   UCSC 
 
  833
 
 13
 
 4047
 
 island
 
 0.046332
 
 0.0072640
 
 Not queried
 
  2.3383879
 
 2.87e-03
 
 0.1170421274
  
 
  
 4
 
  81119178
 
  81119473
 
  PDF   STATS   ENSEMBL   UCSC 
 
  296
 
  6
 
 30034
 
 gene;island
 
 0.034875
 
 0.8729079
 
 Not queried
 
  7.4168963
 
 4.33e-03
 
 0.1459191215
  
 
  
 17
 
  73083632
 
  73083887
 
  PDF   STATS   ENSEMBL   UCSC 
 
  256
 
  6
 
 17642
 
 tss;island
 
 0.014612
 
 0.3163723
 
 Not queried
 
  5.1933939
 
 4.33e-03
 
 0.1459191215
  
 
  
 12
 
  89748726
 
  89749142
 
  PDF   STATS   ENSEMBL   UCSC 
 
  417
 
  9
 
 10666
 
 island
 
 0.028909
 
 0.4848829
 
 Not queried
 
  5.0241402
 
 3.99e-03
 
 0.1459191215
  
 
  
 8
 
 120219902
 
 120220131
 
  PDF   STATS   ENSEMBL   UCSC 
 
  230
 
  7
 
 39885
 
 tss;island
 
 0.923056
 
 0.3317258
 
 Not queried
 
  4.9489219
 
 4.08e-03
 
 0.1459191215
  
 
  
 22
 
  30476089
 
  30476345
 
  PDF   STATS   ENSEMBL   UCSC 
 
  257
 
  9
 
 27055
 
 tss
 
 0.115289
 
 0.9223177
 
 Not queried
 
  4.9208738
 
 3.99e-03
 
 0.1459191215
  
 
  
 7
 
  44104860
 
  44105434
 
  PDF   STATS   ENSEMBL   UCSC 
 
  575
 
  6
 
 38454
 
 island
 
 0.124768
 
 0.5430846
 
 Not queried
 
  4.2541899
 
 4.33e-03
 
 0.1459191215
  
 
  
 7
 
  99775422
 
  99775558
 
  PDF   STATS   ENSEMBL   UCSC 
 
  137
 
  7
 
 37776
 
 tss;island
 
 0.150881
 
 0.8188137
 
 Not queried
 
  4.1404920
 
 4.08e-03
 
 0.1459191215
  
 
  
 1
 
 159869927
 
 159870326
 
  PDF   STATS   ENSEMBL   UCSC 
 
  400
 
  7
 
 2032
 
 tss;island
 
 0.940019
 
 0.4066345
 
 Not queried
 
  4.0336806
 
 4.08e-03
 
 0.1459191215
  
 
  
 10
 
  88728073
 
  88728361
 
  PDF   STATS   ENSEMBL   UCSC 
 
  289
 
  6
 
 5909
 
 island
 
 0.160606
 
 0.7113797
 
 Not queried
 
  3.9664500
 
 4.33e-03
 
 0.1459191215
  
 
  
 16
 
   1583810
 
   1584118
 
  PDF   STATS   ENSEMBL   UCSC 
 
  309
 
  6
 
 14574
 
 gene;tss;island
 
 0.019600
 
 0.2081214
 
 Not queried
 
  3.8346528
 
 4.33e-03
 
 0.1459191215
  
 
  
 7
 
 138720786
 
 138721065
 
  PDF   STATS   ENSEMBL   UCSC 
 
  280
 
  9
 
 37927
 
 tss;island
 
 0.018056
 
 0.3537297
 
 Not queried
 
  3.7403027
 
 3.99e-03
 
 0.1459191215
  
 
  
 22
 
  45809244
 
  45810043
 
  PDF   STATS   ENSEMBL   UCSC 
 
  800
 
 16
 
 27223
 
 tss;island
 
 0.256033
 
 0.0246571
 
 Not queried
 
  3.1529782
 
 3.92e-03
 
 0.1459191215
  
 
  
 12
 
 109162390
 
 109162748
 
  PDF   STATS   ENSEMBL   UCSC 
 
  359
 
  6
 
 10773
 
 island
 
 0.277721
 
 0.0295959
 
 Not queried
 
  2.9918148
 
 4.33e-03
 
 0.1459191215
  
 
  
 8
 
  10586678
 
  10587055
 
  PDF   STATS   ENSEMBL   UCSC 
 
  378
 
  7
 
 39199
 
 gene;island
 
 0.066544
 
 0.4429420
 
 Not queried
 
  2.7346372
 
 4.08e-03
 
 0.1459191215
  
 
  
 11
 
   7041752
 
   7041987
 
  PDF   STATS   ENSEMBL   UCSC 
 
  236
 
  6
 
 6495
 
 gene
 
 0.032949
 
 0.2007410
 
 Not queried
 
  2.6938616
 
 4.33e-03
 
 0.1459191215
  
 
  
 1
 
  31845923
 
  31846110
 
  PDF   STATS   ENSEMBL   UCSC 
 
  188
 
  6
 
 1445
 
 tss
 
 0.012407
 
 0.0580292
 
 Not queried
 
  2.3122575
 
 4.33e-03
 
 0.1459191215
  
 
  
 5
 
  87974369
 
  87974547
 
  PDF   STATS   ENSEMBL   UCSC 
 
  179
 
  7
 
 31680
 
 gene;tss;island
 
 0.491968
 
 0.0506315
 
 Not queried
 
  2.2843435
 
 4.08e-03
 
 0.1459191215
  
 
  
 14
 
 102026086
 
 102026555
 
  PDF   STATS   ENSEMBL   UCSC 
 
  470
 
 11
 
 13158
 
 island
 
 0.038088
 
 0.1289377
 
 Not queried
 
  2.0708011
 
 4.10e-03
 
 0.1459191215
  
 
  
 8
 
  63997977
 
  63998778
 
  PDF   STATS   ENSEMBL   UCSC 
 
  802
 
 11
 
 40337
 
 island
 
 0.046892
 
 0.0121773
 
 Not queried
 
  1.8981129
 
 4.10e-03
 
 0.1459191215
  
 
  
 8
 
  61193970
 
  61194292
 
  PDF   STATS   ENSEMBL   UCSC 
 
  323
 
  9
 
 39724
 
 tss
 
 0.064075
 
 0.0157552
 
 Not queried
 
  1.7649384
 
 3.99e-03
 
 0.1459191215
  
 
  
 18
 
  12253749
 
  12254566
 
  PDF   STATS   ENSEMBL   UCSC 
 
  818
 
 18
 
 19186
 
 tss
 
 0.052308
 
 0.1777130
 
 Not queried
 
  1.6192775
 
 4.16e-03
 
 0.1459191215
  
 
  
 1
 
 205818956
 
 205819609
 
  PDF   STATS   ENSEMBL   UCSC 
 
  654
 
  8
 
 4138
 
 island
 
 0.502067
 
 0.0239484
 
 Not queried
 
  6.1682762
 
 4.66e-03
 
 0.1498346615
  
 
  
 11
 
  63258509
 
  63258803
 
  PDF   STATS   ENSEMBL   UCSC 
 
  295
 
  8
 
 8369
 
 island
 
 0.008959
 
 0.0854652
 
 Not queried
 
  3.5958034
 
 4.66e-03
 
 0.1498346615
  
 
  
 6
 
  94129412
 
  94129697
 
  PDF   STATS   ENSEMBL   UCSC 
 
  286
 
  8
 
 35125
 
 tss
 
 0.032464
 
 0.1662164
 
 Not queried
 
  3.3863191
 
 4.66e-03
 
 0.1498346615
  
 
  
 19
 
  40732691
 
  40732902
 
  PDF   STATS   ENSEMBL   UCSC 
 
  212
 
  8
 
 20784
 
 tss
 
 0.249021
 
 0.6067431
 
 Not queried
 
  3.2676259
 
 4.66e-03
 
 0.1498346615
  
 
  
 6
 
  32171786
 
  32172151
 
  PDF   STATS   ENSEMBL   UCSC 
 
  366
 
  8
 
 34088
 
 gene
 
 0.255991
 
 0.4761910
 
 Not queried
 
  2.1314924
 
 4.66e-03
 
 0.1498346615
  
 
  
 4
 
 111544235
 
 111544463
 
  PDF   STATS   ENSEMBL   UCSC 
 
  229
 
  8
 
 30095
 
 gene;island
 
 0.069702
 
 0.0196600
 
 Not queried
 
  1.6038539
 
 4.66e-03
 
 0.1498346615
  
 
  
 17
 
  40489513
 
  40489785
 
  PDF   STATS   ENSEMBL   UCSC 
 
  273
 
  5
 
 16579
 
 gene
 
 0.004694
 
 0.9636622
 
 Not queried
 
 14.0378462
 
 7.94e-03
 
 0.1589602024
  
 
  
 7
 
  28452066
 
  28452289
 
  PDF   STATS   ENSEMBL   UCSC 
 
  224
 
  5
 
 36945
 
 gene;island
 
 0.989786
 
 0.0072924
 
 Not queried
 
 11.5252549
 
 7.94e-03
 
 0.1589602024
  
 
  
 2
 
 111875749
 
 111876008
 
  PDF   STATS   ENSEMBL   UCSC 
 
  260
 
  5
 
 24611
 
 island
 
 0.010095
 
 0.8522432
 
 Not queried
 
 10.1792335
 
 7.94e-03
 
 0.1589602024
  
 
  
 2
 
  37571677
 
  37571732
 
  PDF   STATS   ENSEMBL   UCSC 
 
   56
 
  5
 
 23306
 
 tss
 
 0.019335
 
 0.6384695
 
 Not queried
 
  9.3837708
 
 7.94e-03
 
 0.1589602024
  
 
  
 5
 
  68628240
 
  68628738
 
  PDF   STATS   ENSEMBL   UCSC 
 
  499
 
  5
 
 32851
 
 island
 
 0.507248
 
 0.0045282
 
 Not queried
 
  9.2522900
 
 7.94e-03
 
 0.1589602024
  
 
  
 6
 
   2891973
 
   2892152
 
  PDF   STATS   ENSEMBL   UCSC 
 
  180
 
  5
 
 33534
 
 gene;island
 
 0.962667
 
 0.0549850
 
 Not queried
 
  9.0605891
 
 7.94e-03
 
 0.1589602024
  
 
  
 5
 
 140792511
 
 140792700
 
  PDF   STATS   ENSEMBL   UCSC 
 
  190
 
  5
 
 32381
 
 tss
 
 0.467823
 
 0.0036103
 
 Not queried
 
  8.6433432
 
 7.94e-03
 
 0.1589602024
  
 
  
 5
 
 178986620
 
 178986906
 
  PDF   STATS   ENSEMBL   UCSC 
 
  287
 
  5
 
 31933
 
 gene;island
 
 0.583700
 
 0.0055649
 
 Not queried
 
  8.4790729
 
 7.94e-03
 
 0.1589602024
  
 
  
 4
 
 123747447
 
 123747750
 
  PDF   STATS   ENSEMBL   UCSC 
 
  304
 
  7
 
 30581
 
 tss;island
 
 0.012916
 
 0.7316144
 
 Not queried
 
  7.9524595
 
 6.99e-03
 
 0.1589602024
  
 
  
 12
 
  54410657
 
  54410727
 
  PDF   STATS   ENSEMBL   UCSC 
 
   71
 
  5
 
 9158
 
 gene
 
 0.467615
 
 0.0057533
 
 Not queried
 
  7.4982562
 
 7.94e-03
 
 0.1589602024
  
 
  
 19
 
  50861774
 
  50862121
 
  PDF   STATS   ENSEMBL   UCSC 
 
  348
 
  5
 
 20174
 
 gene;island
 
 0.007593
 
 0.4888662
 
 Not queried
 
  7.1183869
 
 7.94e-03
 
 0.1589602024
  
 
  
 1
 
  67772896
 
  67773044
 
  PDF   STATS   ENSEMBL   UCSC 
 
  149
 
  5
 
 1683
 
 tss
 
 0.006256
 
 0.5565591
 
 Not queried
 
  6.8363971
 
 7.94e-03
 
 0.1589602024
  
 
  
 16
 
  54965084
 
  54965142
 
  PDF   STATS   ENSEMBL   UCSC 
 
   59
 
  5
 
 15931
 
 island
 
 0.395572
 
 0.0097971
 
 Not queried
 
  6.8056915
 
 7.94e-03
 
 0.1589602024
  
 
  
 11
 
    504784
 
    504937
 
  PDF   STATS   ENSEMBL   UCSC 
 
  154
 
  5
 
 6377
 
 gene;island
 
 0.575586
 
 0.0111722
 
 Not queried
 
  6.4484180
 
 7.94e-03
 
 0.1589602024
  
 
  
 8
 
  86350889
 
  86351018
 
  PDF   STATS   ENSEMBL   UCSC 
 
  130
 
  5
 
 39795
 
 tss
 
 0.013715
 
 0.6743677
 
 Not queried
 
  6.4213889
 
 7.94e-03
 
 0.1589602024
  
 
  
 17
 
   1617291
 
   1617465
 
  PDF   STATS   ENSEMBL   UCSC 
 
  175
 
  5
 
 16993
 
 tss
 
 0.009562
 
 0.4183915
 
 Not queried
 
  6.3951308
 
 7.94e-03
 
 0.1589602024
  
 
  
 19
 
  44763979
 
  44764380
 
  PDF   STATS   ENSEMBL   UCSC 
 
  402
 
  5
 
 22106
 
 island
 
 0.007470
 
 0.5008962
 
 Not queried
 
  6.3308639
 
 7.94e-03
 
 0.1589602024
  
 
  
 1
 
 108023249
 
 108023482
 
  PDF   STATS   ENSEMBL   UCSC 
 
  234
 
  5
 
 660
 
 gene;island
 
 0.456695
 
 0.0105990
 
 Not queried
 
  6.3138337
 
 7.94e-03
 
 0.1589602024
  
 
  
 17
 
   1509883
 
   1510080
 
  PDF   STATS   ENSEMBL   UCSC 
 
  198
 
  5
 
 16316
 
 gene;island
 
 0.025488
 
 0.5173631
 
 Not queried
 
  6.1628452
 
 7.94e-03
 
 0.1589602024
  
 
  
 19
 
   3480363
 
   3480672
 
  PDF   STATS   ENSEMBL   UCSC 
 
  310
 
  5
 
 21330
 
 island
 
 0.848780
 
 0.1021668
 
 Not queried
 
  5.7671324
 
 7.94e-03
 
 0.1589602024
  
 
  
 22
 
  45809596
 
  45810043
 
  PDF   STATS   ENSEMBL   UCSC 
 
  448
 
  8
 
 26904
 
 gene
 
 0.156687
 
 0.0134295
 
 Not queried
 
  5.3368392
 
 6.99e-03
 
 0.1589602024
  
 
  
 17
 
  40715222
 
  40715281
 
  PDF   STATS   ENSEMBL   UCSC 
 
   60
 
  5
 
 16580
 
 gene;island
 
 0.071585
 
 0.8278033
 
 Not queried
 
  5.3115756
 
 7.94e-03
 
 0.1589602024
  
 
  
 1
 
 110230252
 
 110230633
 
  PDF   STATS   ENSEMBL   UCSC 
 
  382
 
  5
 
 3570
 
 island
 
 0.012832
 
 0.2712789
 
 Not queried
 
  5.2047135
 
 7.94e-03
 
 0.1589602024
  
 
  
 17
 
   2907895
 
   2908369
 
  PDF   STATS   ENSEMBL   UCSC 
 
  475
 
  5
 
 16334
 
 gene
 
 0.030995
 
 0.4715480
 
 Not queried
 
  5.1644214
 
 7.94e-03
 
 0.1589602024
  
 
  
 11
 
  61522741
 
  61522807
 
  PDF   STATS   ENSEMBL   UCSC 
 
   67
 
  5
 
 6632
 
 gene;tss;island
 
 0.559439
 
 0.9098985
 
 Not queried
 
  5.0323964
 
 7.94e-03
 
 0.1589602024
  
 
  
 10
 
 133793442
 
 133793734
 
  PDF   STATS   ENSEMBL   UCSC 
 
  293
 
  5
 
 4847
 
 gene;island
 
 0.974366
 
 0.5660057
 
 Not queried
 
  4.8201121
 
 7.94e-03
 
 0.1589602024
  
 
  
 20
 
  23549455
 
  23549525
 
  PDF   STATS   ENSEMBL   UCSC 
 
   71
 
  5
 
 25641
 
 tss
 
 0.033461
 
 0.5019344
 
 Not queried
 
  4.7611051
 
 7.94e-03
 
 0.1589602024
  
 
  
 10
 
  13748985
 
  13749625
 
  PDF   STATS   ENSEMBL   UCSC 
 
  641
 
  9
 
 4504
 
 gene
 
 0.176326
 
 0.0100498
 
 Not queried
 
  4.7313157
 
 5.64e-03
 
 0.1589602024
  
 
  
 17
 
  78800574
 
  78800806
 
  PDF   STATS   ENSEMBL   UCSC 
 
  233
 
  5
 
 16875
 
 gene;island
 
 0.645066
 
 0.1962501
 
 Not queried
 
  4.5610170
 
 7.94e-03
 
 0.1589602024
  
 
  
 1
 
   1266468
 
   1266700
 
  PDF   STATS   ENSEMBL   UCSC 
 
  233
 
  5
 
 1147
 
 tss
 
 0.886919
 
 0.2308060
 
 Not queried
 
  4.5454456
 
 7.94e-03
 
 0.1589602024
  
 
  
 3
 
 197807842
 
 197808169
 
  PDF   STATS   ENSEMBL   UCSC 
 
  328
 
  5
 
 28829
 
 tss;island
 
 0.022708
 
 0.4503911
 
 Not queried
 
  4.4928633
 
 7.94e-03
 
 0.1589602024
  
 
  
 3
 
 164914471
 
 164914621
 
  PDF   STATS   ENSEMBL   UCSC 
 
  151
 
  5
 
 28699
 
 tss
 
 0.053107
 
 0.6152631
 
 Not queried
 
  4.4845263
 
 7.94e-03
 
 0.1589602024
  
 
  
 1
 
   9884565
 
   9884732
 
  PDF   STATS   ENSEMBL   UCSC 
 
  168
 
  8
 
 1230
 
 tss;island
 
 0.068205
 
 0.4993870
 
 Not queried
 
  4.4526619
 
 6.99e-03
 
 0.1589602024
  
 
  
 8
 
  63997977
 
  63998463
 
  PDF   STATS   ENSEMBL   UCSC 
 
  487
 
  5
 
 39320
 
 gene
 
 0.055443
 
 0.0119221
 
 Not queried
 
  4.2717515
 
 7.94e-03
 
 0.1589602024
  
 
  
 11
 
 129817418
 
 129817717
 
  PDF   STATS   ENSEMBL   UCSC 
 
  300
 
  5
 
 6970
 
 gene
 
 0.498635
 
 0.0540366
 
 Not queried
 
  4.2255338
 
 7.94e-03
 
 0.1589602024
  
 
  
 1
 
 156261200
 
 156261403
 
  PDF   STATS   ENSEMBL   UCSC 
 
  204
 
  5
 
 820
 
 gene;island
 
 0.042534
 
 0.5784208
 
 Not queried
 
  4.1252895
 
 7.94e-03
 
 0.1589602024
  
 
  
 8
 
 144120335
 
 144120648
 
  PDF   STATS   ENSEMBL   UCSC 
 
  314
 
  5
 
 39943
 
 tss
 
 0.047488
 
 0.5588885
 
 Not queried
 
  4.0951858
 
 7.94e-03
 
 0.1589602024
  
 
  
 17
 
  42733527
 
  42733729
 
  PDF   STATS   ENSEMBL   UCSC 
 
  203
 
  8
 
 17412
 
 tss;island
 
 0.354247
 
 0.0630491
 
 Not queried
 
  4.0874215
 
 6.99e-03
 
 0.1589602024
  
 
  
 12
 
 110211321
 
 110211413
 
  PDF   STATS   ENSEMBL   UCSC 
 
   93
 
  5
 
 9995
 
 tss
 
 0.015344
 
 0.3419507
 
 Not queried
 
  4.0670903
 
 7.94e-03
 
 0.1589602024
  
 
  
 2
 
  43903470
 
  43904011
 
  PDF   STATS   ENSEMBL   UCSC 
 
  542
 
  8
 
 22660
 
 gene;tss
 
 0.487040
 
 0.0415703
 
 Not queried
 
  3.9888871
 
 6.99e-03
 
 0.1589602024
  
 
  
 20
 
  22566877
 
  22567170
 
  PDF   STATS   ENSEMBL   UCSC 
 
  294
 
  5
 
 25634
 
 tss;island
 
 0.030750
 
 0.3472412
 
 Not queried
 
  3.8637783
 
 7.94e-03
 
 0.1589602024
  
 
  
 1
 
   3620638
 
   3621048
 
  PDF   STATS   ENSEMBL   UCSC 
 
  411
 
  5
 
 140
 
 gene;island
 
 0.582126
 
 0.1294325
 
 Not queried
 
  3.7755481
 
 7.94e-03
 
 0.1589602024
  
 
  
 11
 
  76380921
 
  76381236
 
  PDF   STATS   ENSEMBL   UCSC 
 
  316
 
  8
 
 6837
 
 gene;island
 
 0.028251
 
 0.4276290
 
 Not queried
 
  3.7692199
 
 6.99e-03
 
 0.1589602024
  
 
  
 2
 
 223163573
 
 223163809
 
  PDF   STATS   ENSEMBL   UCSC 
 
  237
 
  5
 
 23048
 
 gene;island
 
 0.035590
 
 0.4007198
 
 Not queried
 
  3.7106825
 
 7.94e-03
 
 0.1589602024
  
 
  
 12
 
 103889772
 
 103890274
 
  PDF   STATS   ENSEMBL   UCSC 
 
  503
 
  9
 
 9952
 
 tss
 
 0.172701
 
 0.0242318
 
 Not queried
 
  3.5942682
 
 7.77e-03
 
 0.1589602024
  
 
  
 3
 
  62304470
 
  62304779
 
  PDF   STATS   ENSEMBL   UCSC 
 
  310
 
  5
 
 28424
 
 tss;island
 
 0.028231
 
 0.2808430
 
 Not queried
 
  3.5180730
 
 7.94e-03
 
 0.1589602024
  
 
  
 11
 
  46366643
 
  46367100
 
  PDF   STATS   ENSEMBL   UCSC 
 
  458
 
  8
 
 6598
 
 gene;island
 
 0.065114
 
 0.3209312
 
 Not queried
 
  3.4836681
 
 6.99e-03
 
 0.1589602024
  
 
  
 1
 
 147782116
 
 147782558
 
  PDF   STATS   ENSEMBL   UCSC 
 
  443
 
  5
 
 3702
 
 island
 
 0.488496
 
 0.8835660
 
 Not queried
 
  3.4716605
 
 7.94e-03
 
 0.1589602024
  
 
  
 11
 
   7041194
 
   7041570
 
  PDF   STATS   ENSEMBL   UCSC 
 
  377
 
  8
 
 6494
 
 gene
 
 0.183882
 
 0.0441096
 
 Not queried
 
  3.4395182
 
 6.99e-03
 
 0.1589602024
  
 
  
 12
 
  25102274
 
  25102521
 
  PDF   STATS   ENSEMBL   UCSC 
 
  248
 
  5
 
 10290
 
 island
 
 0.002678
 
 0.0136372
 
 Not queried
 
  3.3497117
 
 7.94e-03
 
 0.1589602024
  
 
  
 1
 
 153599479
 
 153599831
 
  PDF   STATS   ENSEMBL   UCSC 
 
  353
 
  5
 
 762
 
 gene;tss
 
 0.299869
 
 0.8668449
 
 Not queried
 
  3.3483387
 
 7.94e-03
 
 0.1589602024
  
 
  
 14
 
  92414151
 
  92414425
 
  PDF   STATS   ENSEMBL   UCSC 
 
  275
 
  5
 
 12562
 
 tss;island
 
 0.086907
 
 0.5081642
 
 Not queried
 
  3.2244723
 
 7.94e-03
 
 0.1589602024
  
 
  
 7
 
 145813915
 
 145814306
 
  PDF   STATS   ENSEMBL   UCSC 
 
  392
 
  5
 
 37257
 
 gene;island
 
 0.023150
 
 0.1366396
 
 Not queried
 
  3.2169324
 
 7.94e-03
 
 0.1589602024
  
 
  
 15
 
  74421739
 
  74421935
 
  PDF   STATS   ENSEMBL   UCSC 
 
  197
 
  7
 
 13416
 
 gene
 
 0.049488
 
 0.0062248
 
 Not queried
 
  3.1998197
 
 6.99e-03
 
 0.1589602024
  
 
  
 17
 
  79881468
 
  79881543
 
  PDF   STATS   ENSEMBL   UCSC 
 
   76
 
  5
 
 16929
 
 gene;tss;island
 
 0.232049
 
 0.0342844
 
 Not queried
 
  3.0975369
 
 7.94e-03
 
 0.1589602024
  
 
  
 2
 
  71503484
 
  71503883
 
  PDF   STATS   ENSEMBL   UCSC 
 
  400
 
  7
 
 24396
 
 island
 
 0.079394
 
 0.0065999
 
 Not queried
 
  3.0943422
 
 6.99e-03
 
 0.1589602024
  
 
  
 19
 
   3398706
 
   3399015
 
  PDF   STATS   ENSEMBL   UCSC 
 
  310
 
  5
 
 19715
 
 gene;island
 
 0.941854
 
 0.7375422
 
 Not queried
 
  3.0619062
 
 7.94e-03
 
 0.1589602024
  
 
  
 2
 
   3642710
 
   3642967
 
  PDF   STATS   ENSEMBL   UCSC 
 
  258
 
  5
 
 22553
 
 gene
 
 0.969974
 
 0.7826497
 
 Not queried
 
  3.0593051
 
 7.94e-03
 
 0.1589602024
  
 
  
 12
 
  99548329
 
  99548550
 
  PDF   STATS   ENSEMBL   UCSC 
 
  222
 
  5
 
 9262
 
 gene
 
 0.612051
 
 0.8946646
 
 Not queried
 
  3.0494053
 
 7.94e-03
 
 0.1589602024
  
 
  
 11
 
  20690628
 
  20691429
 
  PDF   STATS   ENSEMBL   UCSC 
 
  802
 
 12
 
 8131
 
 island
 
 0.007322
 
 0.0429836
 
 Not queried
 
  3.0260278
 
 6.81e-03
 
 0.1589602024
  
 
  
 2
 
  27505396
 
  27505593
 
  PDF   STATS   ENSEMBL   UCSC 
 
  198
 
  5
 
 23262
 
 tss
 
 0.046195
 
 0.2696030
 
 Not queried
 
  3.0052333
 
 7.94e-03
 
 0.1589602024
  
 
  
 1
 
 209848845
 
 209849185
 
  PDF   STATS   ENSEMBL   UCSC 
 
  341
 
  5
 
 986
 
 gene;island
 
 0.026664
 
 0.1864591
 
 Not queried
 
  2.8239682
 
 7.94e-03
 
 0.1589602024
  
 
  
 14
 
 104940229
 
 104940658
 
  PDF   STATS   ENSEMBL   UCSC 
 
  430
 
  5
 
 13206
 
 island
 
 0.355911
 
 0.7924069
 
 Not queried
 
  2.7890362
 
 7.94e-03
 
 0.1589602024
  
 
  
 1
 
 229569877
 
 229570288
 
  PDF   STATS   ENSEMBL   UCSC 
 
  412
 
 11
 
 2311
 
 tss
 
 0.062650
 
 0.3022816
 
 Not queried
 
  2.6624888
 
 6.63e-03
 
 0.1589602024
  
 
  
 2
 
 242749925
 
 242750102
 
  PDF   STATS   ENSEMBL   UCSC 
 
  178
 
  5
 
 23994
 
 tss
 
 0.691703
 
 0.9657436
 
 Not queried
 
  2.3873299
 
 7.94e-03
 
 0.1589602024
  
 
  
 3
 
  32859377
 
  32859445
 
  PDF   STATS   ENSEMBL   UCSC 
 
   69
 
  5
 
 28208
 
 tss
 
 0.097327
 
 0.0230958
 
 Not queried
 
  2.2458254
 
 7.94e-03
 
 0.1589602024
  
 
  
 15
 
  26108391
 
  26108412
 
  PDF   STATS   ENSEMBL   UCSC 
 
   22
 
  5
 
 13538
 
 tss
 
 0.007271
 
 0.0340858
 
 Not queried
 
  2.2338058
 
 7.94e-03
 
 0.1589602024
  
 
  
 1
 
 201368738
 
 201369031
 
  PDF   STATS   ENSEMBL   UCSC 
 
  294
 
  8
 
 2166
 
 tss
 
 0.197711
 
 0.0313311
 
 Not queried
 
  2.1959804
 
 6.99e-03
 
 0.1589602024
  
 
  
 1
 
 201368613
 
 201369031
 
  PDF   STATS   ENSEMBL   UCSC 
 
  419
 
  9
 
 4075
 
 island
 
 0.142288
 
 0.0323819
 
 Not queried
 
  2.0695451
 
 7.77e-03
 
 0.1589602024
  
 
  
 5
 
 134871645
 
 134872315
 
  PDF   STATS   ENSEMBL   UCSC 
 
  671
 
 18
 
 32305
 
 tss
 
 0.010954
 
 0.0427345
 
 Not queried
 
  2.0596760
 
 5.76e-03
 
 0.1589602024
  
 
  
 1
 
 161993675
 
 161994363
 
  PDF   STATS   ENSEMBL   UCSC 
 
  689
 
 10
 
 2056
 
 tss
 
 0.044262
 
 0.0081811
 
 Not queried
 
  2.0115237
 
 6.84e-03
 
 0.1589602024
  
 
  
 19
 
  13215041
 
  13215387
 
  PDF   STATS   ENSEMBL   UCSC 
 
  347
 
  5
 
 21644
 
 island
 
 0.027408
 
 0.0702646
 
 Not queried
 
  1.8615446
 
 7.94e-03
 
 0.1589602024
  
 
  
 3
 
   9811714
 
   9811990
 
  PDF   STATS   ENSEMBL   UCSC 
 
  277
 
  7
 
 28130
 
 tss;island
 
 0.445821
 
 0.0291129
 
 Not queried
 
  1.8061684
 
 6.99e-03
 
 0.1589602024
  
 
  
 2
 
  85107190
 
  85107488
 
  PDF   STATS   ENSEMBL   UCSC 
 
  299
 
  5
 
 22759
 
 gene;island
 
 0.009965
 
 0.0323884
 
 Not queried
 
  1.7490617
 
 7.94e-03
 
 0.1589602024
  
 
  
 6
 
  31545252
 
  31545473
 
  PDF   STATS   ENSEMBL   UCSC 
 
  222
 
  5
 
 33903
 
 gene;island
 
 0.412472
 
 0.6701955
 
 Not queried
 
  1.6599846
 
 7.94e-03
 
 0.1589602024
  
 
  
 8
 
  24771114
 
  24771645
 
  PDF   STATS   ENSEMBL   UCSC 
 
  532
 
  7
 
 39604
 
 tss;island
 
 0.299202
 
 0.0862646
 
 Not queried
 
  1.6570133
 
 6.99e-03
 
 0.1589602024
  
 
  
 4
 
  81256552
 
  81256850
 
  PDF   STATS   ENSEMBL   UCSC 
 
  299
 
  7
 
 30462
 
 tss
 
 0.006820
 
 0.0017050
 
 Not queried
 
  1.6494612
 
 6.99e-03
 
 0.1589602024
  
 
  
 4
 
   3742095
 
   3742357
 
  PDF   STATS   ENSEMBL   UCSC 
 
  263
 
  5
 
 30852
 
 island
 
 0.411660
 
 0.6692105
 
 Not queried
 
  1.5322303
 
 7.94e-03
 
 0.1589602024
  
 
  
 1
 
   3663164
 
   3663832
 
  PDF   STATS   ENSEMBL   UCSC 
 
  669
 
  6
 
 144
 
 gene
 
 0.295363
 
 0.0097385
 
 Not queried
 
  4.9667517
 
 8.66e-03
 
 0.1595382395
  
 
  
 3
 
 183543564
 
 183544049
 
  PDF   STATS   ENSEMBL   UCSC 
 
  486
 
  6
 
 28748
 
 tss;island
 
 0.685619
 
 0.0611229
 
 Not queried
 
  4.9111157
 
 8.66e-03
 
 0.1595382395
  
 
  
 2
 
 215275757
 
 215276325
 
  PDF   STATS   ENSEMBL   UCSC 
 
  569
 
  6
 
 23860
 
 tss
 
 0.004457
 
 0.1140217
 
 Not queried
 
  4.6915881
 
 8.66e-03
 
 0.1595382395
  
 
  
 1
 
  99729757
 
  99729807
 
  PDF   STATS   ENSEMBL   UCSC 
 
   51
 
  6
 
 1773
 
 tss;island
 
 0.017749
 
 0.2304625
 
 Not queried
 
  4.6888998
 
 8.66e-03
 
 0.1595382395
  
 
  
 21
 
  34398085
 
  34398226
 
  PDF   STATS   ENSEMBL   UCSC 
 
  142
 
  6
 
 26516
 
 tss
 
 0.009087
 
 0.1323411
 
 Not queried
 
  4.2937346
 
 8.66e-03
 
 0.1595382395
  
 
  
 2
 
 108602784
 
 108602979
 
  PDF   STATS   ENSEMBL   UCSC 
 
  196
 
  6
 
 23562
 
 tss
 
 0.030141
 
 0.5087433
 
 Not queried
 
  4.0590701
 
 8.66e-03
 
 0.1595382395
  
 
  
 5
 
 140480507
 
 140480967
 
  PDF   STATS   ENSEMBL   UCSC 
 
  461
 
  6
 
 31807
 
 gene;island
 
 0.312893
 
 0.7993564
 
 Not queried
 
  4.0507372
 
 8.66e-03
 
 0.1595382395
  
 
  
 17
 
    151914
 
    152350
 
  PDF   STATS   ENSEMBL   UCSC 
 
  437
 
  6
 
 16287
 
 gene;island
 
 0.067967
 
 0.5745881
 
 Not queried
 
  3.8800221
 
 8.66e-03
 
 0.1595382395
  
 
  
 10
 
 105212056
 
 105212813
 
  PDF   STATS   ENSEMBL   UCSC 
 
  758
 
 12
 
 6102
 
 island
 
 0.027339
 
 0.5248855
 
 Not queried
 
  3.7265075
 
 8.29e-03
 
 0.1595382395
  
 
  
 19
 
  50666238
 
  50666552
 
  PDF   STATS   ENSEMBL   UCSC 
 
  315
 
  6
 
 22300
 
 island
 
 0.090369
 
 0.5465316
 
 Not queried
 
  2.9998685
 
 8.66e-03
 
 0.1595382395
  
 
  
 14
 
  74705942
 
  74706113
 
  PDF   STATS   ENSEMBL   UCSC 
 
  172
 
  6
 
 12505
 
 tss
 
 0.075209
 
 0.4016325
 
 Not queried
 
  2.9726701
 
 8.66e-03
 
 0.1595382395
  
 
  
 17
 
  78417789
 
  78418076
 
  PDF   STATS   ENSEMBL   UCSC 
 
  288
 
  6
 
 18876
 
 island
 
 0.223605
 
 0.6615421
 
 Not queried
 
  2.5485699
 
 8.66e-03
 
 0.1595382395
  
 
  
 15
 
  75470777
 
  75471218
 
  PDF   STATS   ENSEMBL   UCSC 
 
  442
 
  6
 
 14325
 
 island
 
 0.138255
 
 0.0159467
 
 Not queried
 
  2.4353165
 
 8.66e-03
 
 0.1595382395
  
 
  
 4
 
 187476326
 
 187477065
 
  PDF   STATS   ENSEMBL   UCSC 
 
  740
 
 11
 
 31483
 
 island
 
 0.071298
 
 0.3663095
 
 Not queried
 
  2.4274628
 
 8.33e-03
 
 0.1595382395
  
 
  
 1
 
 221060753
 
 221061254
 
  PDF   STATS   ENSEMBL   UCSC 
 
  502
 
  6
 
 4224
 
 island
 
 0.079963
 
 0.3133908
 
 Not queried
 
  2.2645897
 
 8.66e-03
 
 0.1595382395
  
 
  
 20
 
  26188919
 
  26188997
 
  PDF   STATS   ENSEMBL   UCSC 
 
   79
 
  6
 
 25655
 
 tss
 
 0.155762
 
 0.3508526
 
 Not queried
 
  1.9006344
 
 8.66e-03
 
 0.1595382395
  
 
  
 1
 
 160039847
 
 160040667
 
  PDF   STATS   ENSEMBL   UCSC 
 
  821
 
 13
 
 3887
 
 island
 
 0.028228
 
 0.1573996
 
 Not queried
 
  1.7591108
 
 8.60e-03
 
 0.1595382395
  
 
  
 11
 
   2889809
 
   2890725
 
  PDF   STATS   ENSEMBL   UCSC 
 
  917
 
 32
 
 7078
 
 tss
 
 0.322148
 
 0.0521004
 
 Not queried
 
  1.6134135
 
 8.37e-03
 
 0.1595382395
  
 
  
 5
 
 140792511
 
 140792992
 
  PDF   STATS   ENSEMBL   UCSC 
 
  482
 
  8
 
 31832
 
 gene;island
 
 0.301042
 
 0.0068122
 
 Not queried
 
  5.3488914
 
 1.04e-02
 
 0.1806165940
  
 
  
 20
 
  21378045
 
  21378278
 
  PDF   STATS   ENSEMBL   UCSC 
 
  234
 
  8
 
 26040
 
 island
 
 0.400387
 
 0.0290619
 
 Not queried
 
  3.9353433
 
 1.04e-02
 
 0.1806165940
  
 
  
 10
 
 105212236
 
 105212813
 
  PDF   STATS   ENSEMBL   UCSC 
 
  578
 
 11
 
 5338
 
 tss
 
 0.031576
 
 0.5254705
 
 Not queried
 
  3.9273604
 
 1.04e-02
 
 0.1806165940
  
 
  
 7
 
   1022471
 
   1023156
 
  PDF   STATS   ENSEMBL   UCSC 
 
  686
 
 11
 
 38063
 
 island
 
 0.457979
 
 0.8603505
 
 Not queried
 
  3.0098392
 
 1.04e-02
 
 0.1806165940
  
 
  
 22
 
  31317764
 
  31318546
 
  PDF   STATS   ENSEMBL   UCSC 
 
  783
 
 12
 
 27420
 
 island
 
 0.031392
 
 0.4054548
 
 Not queried
 
  2.9165630
 
 1.00e-02
 
 0.1806165940
  
 
  
 14
 
  34420292
 
  34420683
 
  PDF   STATS   ENSEMBL   UCSC 
 
  392
 
  8
 
 12345
 
 tss
 
 0.015686
 
 0.0059612
 
 Not queried
 
  2.8703108
 
 1.04e-02
 
 0.1806165940
  
 
  
 7
 
 157453775
 
 157454207
 
  PDF   STATS   ENSEMBL   UCSC 
 
  433
 
  8
 
 37337
 
 gene;island
 
 0.717591
 
 0.9562647
 
 Not queried
 
  2.6855317
 
 1.04e-02
 
 0.1806165940
  
 
  
 12
 
   7260546
 
   7260888
 
  PDF   STATS   ENSEMBL   UCSC 
 
  343
 
  8
 
 9044
 
 gene;tss
 
 0.167431
 
 0.0119845
 
 Not queried
 
  2.3220771
 
 1.04e-02
 
 0.1806165940
  
 
  
 7
 
 129414627
 
 129414894
 
  PDF   STATS   ENSEMBL   UCSC 
 
  268
 
 11
 
 37894
 
 tss
 
 0.531533
 
 0.7829856
 
 Not queried
 
  1.9371322
 
 1.04e-02
 
 0.1806165940
  
 
  
 3
 
 187387555
 
 187388148
 
  PDF   STATS   ENSEMBL   UCSC 
 
  594
 
  8
 
 28077
 
 gene
 
 0.124969
 
 0.4855629
 
 Not queried
 
  1.8833305
 
 1.04e-02
 
 0.1806165940
  
 
  
 6
 
  29942951
 
  29943841
 
  PDF   STATS   ENSEMBL   UCSC 
 
  891
 
 15
 
 33703
 
 gene;island
 
 0.125717
 
 0.0123264
 
 Not queried
 
  1.8501491
 
 9.87e-03
 
 0.1806165940
  
 
  
 1
 
 160039962
 
 160040667
 
  PDF   STATS   ENSEMBL   UCSC 
 
  706
 
 11
 
 2035
 
 tss
 
 0.028228
 
 0.1870986
 
 Not queried
 
  1.7591108
 
 1.04e-02
 
 0.1806165940
  
 
  
 7
 
 129414567
 
 129414894
 
  PDF   STATS   ENSEMBL   UCSC 
 
  328
 
 12
 
 38911
 
 island
 
 0.539132
 
 0.8168730
 
 Not queried
 
  1.5015820
 
 1.00e-02
 
 0.1806165940
  
 
  
 2
 
 228029433
 
 228029856
 
  PDF   STATS   ENSEMBL   UCSC 
 
  424
 
  8
 
 23058
 
 gene;tss;island
 
 0.017290
 
 0.0350918
 
 Not queried
 
  1.4291018
 
 1.04e-02
 
 0.1806165940
  
 
  
 4
 
   4860431
 
   4860698
 
  PDF   STATS   ENSEMBL   UCSC 
 
  268
 
  9
 
 30270
 
 tss;island
 
 0.117571
 
 0.6122316
 
 Not queried
 
  4.0966798
 
 1.06e-02
 
 0.1810746987
  
 
  
 6
 
  10886946
 
  10887367
 
  PDF   STATS   ENSEMBL   UCSC 
 
  422
 
  9
 
 35442
 
 island
 
 0.036495
 
 0.0037025
 
 Not queried
 
  3.2872387
 
 1.06e-02
 
 0.1810746987
  
 
  
 4
 
  74734714
 
  74735101
 
  PDF   STATS   ENSEMBL   UCSC 
 
  388
 
  9
 
 30430
 
 tss
 
 0.020878
 
 0.3862605
 
 Not queried
 
  2.8587477
 
 1.06e-02
 
 0.1810746987
  
 
  
 2
 
  29337946
 
  29338258
 
  PDF   STATS   ENSEMBL   UCSC 
 
  313
 
  9
 
 23282
 
 tss
 
 0.015845
 
 0.0578293
 
 Not queried
 
  1.9596190
 
 1.06e-02
 
 0.1810746987
  
 
  
 1
 
  27961563
 
  27962037
 
  PDF   STATS   ENSEMBL   UCSC 
 
  475
 
  7
 
 2925
 
 island
 
 0.035234
 
 0.5355589
 
 Not queried
 
  4.8558248
 
 1.11e-02
 
 0.1821650572
  
 
  
 6
 
  26988141
 
  26988289
 
  PDF   STATS   ENSEMBL   UCSC 
 
  149
 
  7
 
 34710
 
 tss
 
 0.027023
 
 0.4341912
 
 Not queried
 
  3.3111817
 
 1.11e-02
 
 0.1821650572
  
 
  
 6
 
  28129313
 
  28129498
 
  PDF   STATS   ENSEMBL   UCSC 
 
  186
 
  7
 
 34734
 
 tss
 
 0.017455
 
 0.2015239
 
 Not queried
 
  3.3076549
 
 1.11e-02
 
 0.1821650572
  
 
  
 19
 
  15121204
 
  15121531
 
  PDF   STATS   ENSEMBL   UCSC 
 
  328
 
  7
 
 20552
 
 tss
 
 0.451378
 
 0.8462509
 
 Not queried
 
  2.9098074
 
 1.11e-02
 
 0.1821650572
  
 
  
 12
 
  54070891
 
  54071194
 
  PDF   STATS   ENSEMBL   UCSC 
 
  304
 
  7
 
 9746
 
 tss;island
 
 0.012344
 
 0.1199990
 
 Not queried
 
  2.2686824
 
 1.11e-02
 
 0.1821650572
  
 
  
 20
 
  26188908
 
  26188997
 
  PDF   STATS   ENSEMBL   UCSC 
 
   90
 
  7
 
 26067
 
 island
 
 0.166931
 
 0.3922957
 
 Not queried
 
  2.0374792
 
 1.11e-02
 
 0.1821650572
  
 
  
 10
 
  77161525
 
  77161705
 
  PDF   STATS   ENSEMBL   UCSC 
 
  181
 
  7
 
 4642
 
 gene;tss;island
 
 0.019673
 
 0.1064301
 
 Not queried
 
  1.9972093
 
 1.11e-02
 
 0.1821650572
  
 
  
 7
 
 120968877
 
 120969079
 
  PDF   STATS   ENSEMBL   UCSC 
 
  203
 
  7
 
 37861
 
 tss
 
 0.071248
 
 0.4158600
 
 Not queried
 
  1.9149680
 
 1.11e-02
 
 0.1821650572
  
 
  
 5
 
 134735544
 
 134735701
 
  PDF   STATS   ENSEMBL   UCSC 
 
  158
 
  7
 
 32303
 
 tss;island
 
 0.532128
 
 0.1718994
 
 Not queried
 
  1.8823666
 
 1.11e-02
 
 0.1821650572
  
 
  
 7
 
  28452066
 
  28452131
 
  PDF   STATS   ENSEMBL   UCSC 
 
   66
 
  4
 
 37507
 
 tss
 
 0.990161
 
 0.0226299
 
 Not queried
 
 13.3735952
 
 2.86e-02
 
 0.1830867741
  
 
  
 12
 
  25150402
 
  25150626
 
  PDF   STATS   ENSEMBL   UCSC 
 
  225
 
  4
 
 9609
 
 tss
 
 0.002884
 
 0.7435135
 
 Not queried
 
 10.9459853
 
 2.86e-02
 
 0.1830867741
  
 
  
 19
 
  36246395
 
  36246408
 
  PDF   STATS   ENSEMBL   UCSC 
 
   14
 
  4
 
 19993
 
 gene;island
 
 0.022923
 
 0.9661384
 
 Not queried
 
 10.1505494
 
 2.86e-02
 
 0.1830867741
  
 
  
 2
 
  65593761
 
  65594021
 
  PDF   STATS   ENSEMBL   UCSC 
 
  261
 
  4
 
 22709
 
 gene
 
 0.140944
 
 0.9824322
 
 Not queried
 
  9.8190186
 
 2.86e-02
 
 0.1830867741
  
 
  
 5
 
 110062384
 
 110062473
 
  PDF   STATS   ENSEMBL   UCSC 
 
   90
 
  4
 
 32204
 
 tss
 
 0.512734
 
 0.0026809
 
 Not queried
 
  9.6690668
 
 2.86e-02
 
 0.1830867741
  
 
  
 2
 
 240196769
 
 240197131
 
  PDF   STATS   ENSEMBL   UCSC 
 
  363
 
  4
 
 23112
 
 gene;island
 
 0.027964
 
 0.9387757
 
 Not queried
 
  9.6600084
 
 2.86e-02
 
 0.1830867741
  
 
  
 17
 
  38821155
 
  38821403
 
  PDF   STATS   ENSEMBL   UCSC 
 
  249
 
  4
 
 16566
 
 gene
 
 0.011024
 
 0.8064254
 
 Not queried
 
  9.6501759
 
 2.86e-02
 
 0.1830867741
  
 
  
 1
 
 205818956
 
 205819251
 
  PDF   STATS   ENSEMBL   UCSC 
 
  296
 
  4
 
 970
 
 gene
 
 0.893684
 
 0.0145689
 
 Not queried
 
  9.4102650
 
 2.86e-02
 
 0.1830867741
  
 
  
 17
 
  33759929
 
  33759986
 
  PDF   STATS   ENSEMBL   UCSC 
 
   58
 
  4
 
 17269
 
 tss
 
 0.565305
 
 0.0017409
 
 Not queried
 
  9.3556085
 
 2.86e-02
 
 0.1830867741
  
 
  
 10
 
  88295210
 
  88295591
 
  PDF   STATS   ENSEMBL   UCSC 
 
  382
 
  4
 
 5905
 
 island
 
 0.020808
 
 0.9162930
 
 Not queried
 
  9.2673245
 
 2.86e-02
 
 0.1830867741
  
 
  
 11
 
   3817692
 
   3817849
 
  PDF   STATS   ENSEMBL   UCSC 
 
  158
 
  4
 
 6480
 
 gene;tss;island
 
 0.578252
 
 0.0042910
 
 Not queried
 
  9.2278024
 
 2.86e-02
 
 0.1830867741
  
 
  
 6
 
  44265644
 
  44265696
 
  PDF   STATS   ENSEMBL   UCSC 
 
   53
 
  4
 
 35011
 
 tss;island
 
 0.003045
 
 0.4535841
 
 Not queried
 
  9.0618445
 
 2.86e-02
 
 0.1830867741
  
 
  
 10
 
  97515222
 
  97515398
 
  PDF   STATS   ENSEMBL   UCSC 
 
  177
 
  4
 
 4689
 
 gene;tss
 
 0.003970
 
 0.7209471
 
 Not queried
 
  9.0161914
 
 2.86e-02
 
 0.1830867741
  
 
  
 17
 
  37894258
 
  37894413
 
  PDF   STATS   ENSEMBL   UCSC 
 
  156
 
  4
 
 16555
 
 gene
 
 0.018304
 
 0.7741049
 
 Not queried
 
  8.4145390
 
 2.86e-02
 
 0.1830867741
  
 
  
 12
 
  49726500
 
  49726893
 
  PDF   STATS   ENSEMBL   UCSC 
 
  394
 
  4
 
 9118
 
 gene;island
 
 0.061202
 
 0.9289986
 
 Not queried
 
  8.3897404
 
 2.86e-02
 
 0.1830867741
  
 
  
 11
 
 120434710
 
 120435056
 
  PDF   STATS   ENSEMBL   UCSC 
 
  347
 
  4
 
 8896
 
 island
 
 0.024088
 
 0.9753791
 
 Not queried
 
  8.2618064
 
 2.86e-02
 
 0.1830867741
  
 
  
 19
 
    617133
 
    617432
 
  PDF   STATS   ENSEMBL   UCSC 
 
  300
 
  4
 
 21170
 
 island
 
 0.033967
 
 0.7497014
 
 Not queried
 
  8.1230432
 
 2.86e-02
 
 0.1830867741
  
 
  
 10
 
 128593922
 
 128594144
 
  PDF   STATS   ENSEMBL   UCSC 
 
  223
 
  5
 
 6246
 
 island
 
 0.066768
 
 0.9325082
 
 Not queried
 
  8.0512702
 
 1.59e-02
 
 0.1830867741
  
 
  
 22
 
  46770063
 
  46770440
 
  PDF   STATS   ENSEMBL   UCSC 
 
  378
 
  4
 
 26913
 
 gene;island
 
 0.024020
 
 0.4929873
 
 Not queried
 
  7.8031271
 
 2.86e-02
 
 0.1830867741
  
 
  
 2
 
 158731577
 
 158731796
 
  PDF   STATS   ENSEMBL   UCSC 
 
  220
 
  4
 
 22904
 
 gene
 
 0.001699
 
 0.3110054
 
 Not queried
 
  7.6994672
 
 2.86e-02
 
 0.1830867741
  
 
  
 8
 
 143407432
 
 143407817
 
  PDF   STATS   ENSEMBL   UCSC 
 
  386
 
  4
 
 39460
 
 gene;island
 
 0.923908
 
 0.0347564
 
 Not queried
 
  7.6088668
 
 2.86e-02
 
 0.1830867741
  
 
  
 21
 
  47604052
 
  47604361
 
  PDF   STATS   ENSEMBL   UCSC 
 
  310
 
  4
 
 26473
 
 gene;island
 
 0.007180
 
 0.4704738
 
 Not queried
 
  7.3173675
 
 2.86e-02
 
 0.1830867741
  
 
  
 5
 
 148521450
 
 148521623
 
  PDF   STATS   ENSEMBL   UCSC 
 
  174
 
  4
 
 31856
 
 gene;island
 
 0.589446
 
 0.0117224
 
 Not queried
 
  7.0416201
 
 2.86e-02
 
 0.1830867741
  
 
  
 1
 
 207842833
 
 207843084
 
  PDF   STATS   ENSEMBL   UCSC 
 
  252
 
  4
 
 979
 
 gene;island
 
 0.570939
 
 0.0393072
 
 Not queried
 
  6.9575180
 
 2.86e-02
 
 0.1830867741
  
 
  
 1
 
 203320386
 
 203320541
 
  PDF   STATS   ENSEMBL   UCSC 
 
  156
 
  4
 
 2192
 
 tss
 
 0.337663
 
 0.0068693
 
 Not queried
 
  6.9536704
 
 2.86e-02
 
 0.1830867741
  
 
  
 16
 
  54965084
 
  54965100
 
  PDF   STATS   ENSEMBL   UCSC 
 
   17
 
  4
 
 15238
 
 tss
 
 0.407386
 
 0.0076154
 
 Not queried
 
  6.9278831
 
 2.86e-02
 
 0.1830867741
  
 
  
 16
 
   2908157
 
   2908245
 
  PDF   STATS   ENSEMBL   UCSC 
 
   89
 
  4
 
 15623
 
 island
 
 0.583395
 
 0.0233641
 
 Not queried
 
  6.8785573
 
 2.86e-02
 
 0.1830867741
  
 
  
 1
 
  68517177
 
  68517273
 
  PDF   STATS   ENSEMBL   UCSC 
 
   97
 
  4
 
 1687
 
 tss;island
 
 0.057630
 
 0.8188631
 
 Not queried
 
  6.8449587
 
 2.86e-02
 
 0.1830867741
  
 
  
 7
 
  27170241
 
  27170394
 
  PDF   STATS   ENSEMBL   UCSC 
 
  154
 
  4
 
 36933
 
 gene
 
 0.906976
 
 0.1607960
 
 Not queried
 
  6.7531441
 
 2.86e-02
 
 0.1830867741
  
 
  
 18
 
  77724395
 
  77724453
 
  PDF   STATS   ENSEMBL   UCSC 
 
   59
 
  4
 
 19338
 
 tss;island
 
 0.004452
 
 0.2069730
 
 Not queried
 
  6.6970066
 
 2.86e-02
 
 0.1830867741
  
 
  
 5
 
 172664995
 
 172665149
 
  PDF   STATS   ENSEMBL   UCSC 
 
  155
 
  4
 
 33408
 
 island
 
 0.019835
 
 0.6341242
 
 Not queried
 
  6.6653136
 
 2.86e-02
 
 0.1830867741
  
 
  
 2
 
  74725040
 
  74725369
 
  PDF   STATS   ENSEMBL   UCSC 
 
  330
 
  4
 
 22743
 
 gene;island
 
 0.018271
 
 0.4085096
 
 Not queried
 
  6.6326987
 
 2.86e-02
 
 0.1830867741
  
 
  
 6
 
  30954872
 
  30955142
 
  PDF   STATS   ENSEMBL   UCSC 
 
  271
 
  4
 
 33845
 
 gene
 
 0.000792
 
 0.0691426
 
 Not queried
 
  6.5902972
 
 2.86e-02
 
 0.1830867741
  
 
  
 1
 
  38461540
 
  38461896
 
  PDF   STATS   ENSEMBL   UCSC 
 
  357
 
  4
 
 3081
 
 island
 
 0.576984
 
 0.0144314
 
 Not queried
 
  6.5587977
 
 2.86e-02
 
 0.1830867741
  
 
  
 10
 
  38645346
 
  38645450
 
  PDF   STATS   ENSEMBL   UCSC 
 
  105
 
  4
 
 4560
 
 gene;island
 
 0.348107
 
 0.0039324
 
 Not queried
 
  6.5555653
 
 2.86e-02
 
 0.1830867741
  
 
  
 10
 
  64565642
 
  64565772
 
  PDF   STATS   ENSEMBL   UCSC 
 
  131
 
  4
 
 4601
 
 gene;island
 
 0.017740
 
 0.6045827
 
 Not queried
 
  6.5482313
 
 2.86e-02
 
 0.1830867741
  
 
  
 19
 
  11531294
 
  11531469
 
  PDF   STATS   ENSEMBL   UCSC 
 
  176
 
  4
 
 20472
 
 tss
 
 0.016321
 
 0.3556879
 
 Not queried
 
  6.4135357
 
 2.86e-02
 
 0.1830867741
  
 
  
 10
 
  73136913
 
  73137135
 
  PDF   STATS   ENSEMBL   UCSC 
 
  223
 
  4
 
 5828
 
 island
 
 0.548947
 
 0.0244379
 
 Not queried
 
  6.3846171
 
 2.86e-02
 
 0.1830867741
  
 
  
 22
 
  24891129
 
  24891452
 
  PDF   STATS   ENSEMBL   UCSC 
 
  324
 
  8
 
 27021
 
 tss;island
 
 0.256461
 
 0.9320004
 
 Not queried
 
  6.3559364
 
 2.07e-02
 
 0.1830867741
  
 
  
 11
 
  60534911
 
  60535066
 
  PDF   STATS   ENSEMBL   UCSC 
 
  156
 
  4
 
 6622
 
 gene;island
 
 0.780722
 
 0.0675438
 
 Not queried
 
  6.1524402
 
 2.86e-02
 
 0.1830867741
  
 
  
 15
 
  74528522
 
  74528606
 
  PDF   STATS   ENSEMBL   UCSC 
 
   85
 
  4
 
 13791
 
 tss
 
 0.009907
 
 0.3859442
 
 Not queried
 
  6.1065654
 
 2.86e-02
 
 0.1830867741
  
 
  
 8
 
  25902284
 
  25902611
 
  PDF   STATS   ENSEMBL   UCSC 
 
  328
 
  6
 
 40170
 
 island
 
 0.562687
 
 0.0355912
 
 Not queried
 
  6.0580052
 
 1.52e-02
 
 0.1830867741
  
 
  
 19
 
   5838729
 
   5838999
 
  PDF   STATS   ENSEMBL   UCSC 
 
  271
 
  7
 
 19757
 
 gene
 
 0.589162
 
 0.0235604
 
 Not queried
 
  6.0319150
 
 2.62e-02
 
 0.1830867741
  
 
  
 7
 
  96654152
 
  96654198
 
  PDF   STATS   ENSEMBL   UCSC 
 
   47
 
  4
 
 37735
 
 tss
 
 0.007670
 
 0.3856614
 
 Not queried
 
  6.0292749
 
 2.86e-02
 
 0.1830867741
  
 
  
 17
 
  48049952
 
  48049979
 
  PDF   STATS   ENSEMBL   UCSC 
 
   28
 
  4
 
 16677
 
 gene;tss;island
 
 0.014207
 
 0.5747780
 
 Not queried
 
  6.0139975
 
 2.86e-02
 
 0.1830867741
  
 
  
 1
 
  31231214
 
  31231488
 
  PDF   STATS   ENSEMBL   UCSC 
 
  275
 
  4
 
 1442
 
 tss
 
 0.110274
 
 0.8751956
 
 Not queried
 
  5.9418284
 
 2.86e-02
 
 0.1830867741
  
 
  
 1
 
  19970204
 
  19970337
 
  PDF   STATS   ENSEMBL   UCSC 
 
  134
 
  4
 
 284
 
 gene;tss;island
 
 0.019627
 
 0.4917562
 
 Not queried
 
  5.8792478
 
 2.86e-02
 
 0.1830867741
  
 
  
 17
 
  38501397
 
  38501773
 
  PDF   STATS   ENSEMBL   UCSC 
 
  377
 
  4
 
 16564
 
 gene;island
 
 0.026062
 
 0.4687828
 
 Not queried
 
  5.8057967
 
 2.86e-02
 
 0.1830867741
  
 
  
 6
 
  74104388
 
  74104600
 
  PDF   STATS   ENSEMBL   UCSC 
 
  213
 
  4
 
 34388
 
 gene;island
 
 0.985075
 
 0.4831894
 
 Not queried
 
  5.7716300
 
 2.86e-02
 
 0.1830867741
  
 
  
 1
 
  40782991
 
  40783264
 
  PDF   STATS   ENSEMBL   UCSC 
 
  274
 
  7
 
 1535
 
 tss
 
 0.020585
 
 0.5042090
 
 Not queried
 
  5.7621412
 
 1.75e-02
 
 0.1830867741
  
 
  
 22
 
  50064736
 
  50065058
 
  PDF   STATS   ENSEMBL   UCSC 
 
  323
 
  4
 
 27605
 
 island
 
 0.501878
 
 0.0314037
 
 Not queried
 
  5.7566556
 
 2.86e-02
 
 0.1830867741
  
 
  
 6
 
 114177329
 
 114177535
 
  PDF   STATS   ENSEMBL   UCSC 
 
  207
 
  4
 
 35184
 
 tss;island
 
 0.001654
 
 0.0310833
 
 Not queried
 
  5.7317830
 
 2.86e-02
 
 0.1830867741
  
 
  
 17
 
    406249
 
    406501
 
  PDF   STATS   ENSEMBL   UCSC 
 
  253
 
  4
 
 17796
 
 island
 
 0.466888
 
 0.9579900
 
 Not queried
 
  5.7129551
 
 2.86e-02
 
 0.1830867741
  
 
  
 3
 
 160475035
 
 160475128
 
  PDF   STATS   ENSEMBL   UCSC 
 
   94
 
  4
 
 28017
 
 gene;island
 
 0.751198
 
 0.0991635
 
 Not queried
 
  5.6628381
 
 2.86e-02
 
 0.1830867741
  
 
  
 5
 
  54281198
 
  54281362
 
  PDF   STATS   ENSEMBL   UCSC 
 
  165
 
  4
 
 31614
 
 gene
 
 0.037765
 
 0.6573436
 
 Not queried
 
  5.5880445
 
 2.86e-02
 
 0.1830867741
  
 
  
 5
 
 134783075
 
 134783273
 
  PDF   STATS   ENSEMBL   UCSC 
 
  199
 
  4
 
 32304
 
 tss
 
 0.048225
 
 0.4280515
 
 Not queried
 
  5.5750734
 
 2.86e-02
 
 0.1830867741
  
 
  
 2
 
 176968584
 
 176968920
 
  PDF   STATS   ENSEMBL   UCSC 
 
  337
 
  4
 
 24895
 
 island
 
 0.024754
 
 0.4158494
 
 Not queried
 
  5.5362340
 
 2.86e-02
 
 0.1830867741
  
 
  
 5
 
  76010444
 
  76010658
 
  PDF   STATS   ENSEMBL   UCSC 
 
  215
 
  4
 
 32134
 
 tss;island
 
 0.281026
 
 0.0058946
 
 Not queried
 
  5.5311173
 
 2.86e-02
 
 0.1830867741
  
 
  
 13
 
 114786825
 
 114786904
 
  PDF   STATS   ENSEMBL   UCSC 
 
   80
 
  4
 
 11307
 
 gene;island
 
 0.976869
 
 0.5728202
 
 Not queried
 
  5.5295818
 
 2.86e-02
 
 0.1830867741
  
 
  
 10
 
 108924366
 
 108924560
 
  PDF   STATS   ENSEMBL   UCSC 
 
  195
 
  4
 
 5351
 
 tss;island
 
 0.571817
 
 0.0370838
 
 Not queried
 
  5.5289257
 
 2.86e-02
 
 0.1830867741
  
 
  
 1
 
  34629098
 
  34629559
 
  PDF   STATS   ENSEMBL   UCSC 
 
  462
 
  5
 
 408
 
 gene;island
 
 0.024379
 
 0.3808904
 
 Not queried
 
  5.4777791
 
 1.59e-02
 
 0.1830867741
  
 
  
 22
 
  18632404
 
  18632618
 
  PDF   STATS   ENSEMBL   UCSC 
 
  215
 
  5
 
 26949
 
 tss
 
 0.039747
 
 0.6642032
 
 Not queried
 
  5.4456720
 
 1.59e-02
 
 0.1830867741
  
 
  
 19
 
  12876846
 
  12877188
 
  PDF   STATS   ENSEMBL   UCSC 
 
  343
 
  4
 
 19866
 
 gene;island
 
 0.031259
 
 0.4443288
 
 Not queried
 
  5.3913106
 
 2.86e-02
 
 0.1830867741
  
 
  
 15
 
  74421523
 
  74421584
 
  PDF   STATS   ENSEMBL   UCSC 
 
   62
 
  4
 
 13415
 
 gene
 
 0.183881
 
 0.0119519
 
 Not queried
 
  5.3794829
 
 2.86e-02
 
 0.1830867741
  
 
  
 7
 
   2316876
 
   2317124
 
  PDF   STATS   ENSEMBL   UCSC 
 
  249
 
  6
 
 36798
 
 gene;island
 
 0.288396
 
 0.7521380
 
 Not queried
 
  5.3073506
 
 1.52e-02
 
 0.1830867741
  
 
  
 10
 
 134150451
 
 134150760
 
  PDF   STATS   ENSEMBL   UCSC 
 
  310
 
  8
 
 4862
 
 gene
 
 0.008994
 
 0.1272652
 
 Not queried
 
  5.3012308
 
 1.48e-02
 
 0.1830867741
  
 
  
 3
 
  55518091
 
  55518142
 
  PDF   STATS   ENSEMBL   UCSC 
 
   52
 
  4
 
 27839
 
 gene;island
 
 0.510060
 
 0.0194786
 
 Not queried
 
  5.2524871
 
 2.86e-02
 
 0.1830867741
  
 
  
 20
 
  21378072
 
  21378278
 
  PDF   STATS   ENSEMBL   UCSC 
 
  207
 
  7
 
 25629
 
 tss
 
 0.478938
 
 0.0250791
 
 Not queried
 
  5.2418945
 
 1.75e-02
 
 0.1830867741
  
 
  
 4
 
 154125208
 
 154125594
 
  PDF   STATS   ENSEMBL   UCSC 
 
  387
 
  6
 
 30640
 
 tss
 
 0.233898
 
 0.9133312
 
 Not queried
 
  5.2377378
 
 2.60e-02
 
 0.1830867741
  
 
  
 5
 
  54281437
 
  54281733
 
  PDF   STATS   ENSEMBL   UCSC 
 
  297
 
  4
 
 32062
 
 tss
 
 0.021359
 
 0.2787144
 
 Not queried
 
  5.2138023
 
 2.86e-02
 
 0.1830867741
  
 
  
 22
 
  37099037
 
  37099259
 
  PDF   STATS   ENSEMBL   UCSC 
 
  223
 
  4
 
 27111
 
 tss;island
 
 0.001962
 
 0.1277975
 
 Not queried
 
  5.1892328
 
 2.86e-02
 
 0.1830867741
  
 
  
 10
 
  94451351
 
  94451736
 
  PDF   STATS   ENSEMBL   UCSC 
 
  386
 
  4
 
 4676
 
 gene;island
 
 0.025563
 
 0.4395985
 
 Not queried
 
  5.1770123
 
 2.86e-02
 
 0.1830867741
  
 
  
 11
 
  45868398
 
  45868501
 
  PDF   STATS   ENSEMBL   UCSC 
 
  104
 
  4
 
 7268
 
 tss;island
 
 0.036109
 
 0.6300213
 
 Not queried
 
  5.1102337
 
 2.86e-02
 
 0.1830867741
  
 
  
 3
 
  98376390
 
  98376574
 
  PDF   STATS   ENSEMBL   UCSC 
 
  185
 
  4
 
 29316
 
 island
 
 0.138696
 
 0.0068149
 
 Not queried
 
  5.0955482
 
 2.86e-02
 
 0.1830867741
  
 
  
 8
 
  37605359
 
  37605978
 
  PDF   STATS   ENSEMBL   UCSC 
 
  620
 
  7
 
 39279
 
 gene
 
 0.593654
 
 0.0299448
 
 Not queried
 
  5.0652547
 
 2.62e-02
 
 0.1830867741
  
 
  
 3
 
   8809306
 
   8809715
 
  PDF   STATS   ENSEMBL   UCSC 
 
  410
 
  4
 
 27646
 
 gene;island
 
 0.011997
 
 0.3695701
 
 Not queried
 
  5.0594313
 
 2.86e-02
 
 0.1830867741
  
 
  
 1
 
   9788517
 
   9788769
 
  PDF   STATS   ENSEMBL   UCSC 
 
  253
 
  4
 
 205
 
 gene;island
 
 0.972305
 
 0.5121900
 
 Not queried
 
  5.0565231
 
 2.86e-02
 
 0.1830867741
  
 
  
 10
 
  94820892
 
  94821085
 
  PDF   STATS   ENSEMBL   UCSC 
 
  194
 
  7
 
 5949
 
 island
 
 0.637901
 
 0.9739686
 
 Not queried
 
  5.0561926
 
 2.62e-02
 
 0.1830867741
  
 
  
 12
 
  43946284
 
  43946518
 
  PDF   STATS   ENSEMBL   UCSC 
 
  235
 
  4
 
 9647
 
 tss;island
 
 0.048522
 
 0.4932203
 
 Not queried
 
  5.0447574
 
 2.86e-02
 
 0.1830867741
  
 
  
 19
 
  22605151
 
  22605234
 
  PDF   STATS   ENSEMBL   UCSC 
 
   84
 
  4
 
 20663
 
 tss
 
 0.029923
 
 0.4247256
 
 Not queried
 
  5.0404232
 
 2.86e-02
 
 0.1830867741
  
 
  
 7
 
  27183133
 
  27183274
 
  PDF   STATS   ENSEMBL   UCSC 
 
  142
 
  4
 
 36934
 
 gene
 
 0.512322
 
 0.9721518
 
 Not queried
 
  5.0368394
 
 2.86e-02
 
 0.1830867741
  
 
  
 1
 
 111743020
 
 111743271
 
  PDF   STATS   ENSEMBL   UCSC 
 
  252
 
  4
 
 682
 
 gene
 
 0.551104
 
 0.9709660
 
 Not queried
 
  5.0356517
 
 2.86e-02
 
 0.1830867741
  
 
  
 6
 
  33141678
 
  33142006
 
  PDF   STATS   ENSEMBL   UCSC 
 
  329
 
  7
 
 34202
 
 gene
 
 0.021523
 
 0.3298105
 
 Not queried
 
  5.0267429
 
 2.62e-02
 
 0.1830867741
  
 
  
 5
 
 140561893
 
 140562236
 
  PDF   STATS   ENSEMBL   UCSC 
 
  344
 
  6
 
 31812
 
 gene;island
 
 0.014005
 
 0.3831282
 
 Not queried
 
  5.0260323
 
 1.52e-02
 
 0.1830867741
  
 
  
 12
 
 103359273
 
 103359572
 
  PDF   STATS   ENSEMBL   UCSC 
 
  300
 
  4
 
 10730
 
 island
 
 0.047543
 
 0.4558243
 
 Not queried
 
  5.0148641
 
 2.86e-02
 
 0.1830867741
  
 
  
 3
 
  48694451
 
  48694673
 
  PDF   STATS   ENSEMBL   UCSC 
 
  223
 
  4
 
 27768
 
 gene;island
 
 0.532859
 
 0.9696640
 
 Not queried
 
  4.9980040
 
 2.86e-02
 
 0.1830867741
  
 
  
 3
 
  48413550
 
  48413707
 
  PDF   STATS   ENSEMBL   UCSC 
 
  158
 
  4
 
 28296
 
 tss
 
 0.038226
 
 0.5362540
 
 Not queried
 
  4.9461810
 
 2.86e-02
 
 0.1830867741
  
 
  
 11
 
 116700436
 
 116700556
 
  PDF   STATS   ENSEMBL   UCSC 
 
  121
 
  4
 
 7713
 
 tss
 
 0.032137
 
 0.4295356
 
 Not queried
 
  4.9285006
 
 2.86e-02
 
 0.1830867741
  
 
  
 1
 
 241520598
 
 241520941
 
  PDF   STATS   ENSEMBL   UCSC 
 
  344
 
  6
 
 2349
 
 tss;island
 
 0.015350
 
 0.4575193
 
 Not queried
 
  4.9096181
 
 2.60e-02
 
 0.1830867741
  
 
  
 17
 
   7555132
 
   7555411
 
  PDF   STATS   ENSEMBL   UCSC 
 
  280
 
  4
 
 16408
 
 gene;island
 
 0.009619
 
 0.2344496
 
 Not queried
 
  4.9060193
 
 2.86e-02
 
 0.1830867741
  
 
  
 15
 
  37179603
 
  37179779
 
  PDF   STATS   ENSEMBL   UCSC 
 
  177
 
  4
 
 13569
 
 tss;island
 
 0.021763
 
 0.2806584
 
 Not queried
 
  4.9056600
 
 2.86e-02
 
 0.1830867741
  
 
  
 4
 
 111540884
 
 111541339
 
  PDF   STATS   ENSEMBL   UCSC 
 
  456
 
  4
 
 30094
 
 gene;island
 
 0.013676
 
 0.2969051
 
 Not queried
 
  4.8954769
 
 2.86e-02
 
 0.1830867741
  
 
  
 16
 
  69141250
 
  69141478
 
  PDF   STATS   ENSEMBL   UCSC 
 
  229
 
  7
 
 14817
 
 gene;island
 
 0.008911
 
 0.2556180
 
 Not queried
 
  4.8816759
 
 1.75e-02
 
 0.1830867741
  
 
  
 19
 
  46001550
 
  46001746
 
  PDF   STATS   ENSEMBL   UCSC 
 
  197
 
  4
 
 20873
 
 tss;island
 
 0.021071
 
 0.5629363
 
 Not queried
 
  4.8637304
 
 2.86e-02
 
 0.1830867741
  
 
  
 21
 
  36259067
 
  36259460
 
  PDF   STATS   ENSEMBL   UCSC 
 
  394
 
  4
 
 26412
 
 gene;island
 
 0.571410
 
 0.0489187
 
 Not queried
 
  4.8628940
 
 2.86e-02
 
 0.1830867741
  
 
  
 7
 
   1882997
 
   1883420
 
  PDF   STATS   ENSEMBL   UCSC 
 
  424
 
  6
 
 36767
 
 gene;island
 
 0.444649
 
 0.9684212
 
 Not queried
 
  4.8528628
 
 1.52e-02
 
 0.1830867741
  
 
  
 18
 
  23713838
 
  23714084
 
  PDF   STATS   ENSEMBL   UCSC 
 
  247
 
  4
 
 19079
 
 gene
 
 0.958339
 
 0.4553496
 
 Not queried
 
  4.8518249
 
 2.86e-02
 
 0.1830867741
  
 
  
 10
 
  38691787
 
  38691801
 
  PDF   STATS   ENSEMBL   UCSC 
 
   15
 
  4
 
 5037
 
 tss;island
 
 0.386527
 
 0.0266214
 
 Not queried
 
  4.8516142
 
 2.86e-02
 
 0.1830867741
  
 
  
 6
 
  34499314
 
  34499504
 
  PDF   STATS   ENSEMBL   UCSC 
 
  191
 
  4
 
 34304
 
 gene;island
 
 0.891195
 
 0.4481749
 
 Not queried
 
  4.8132662
 
 2.86e-02
 
 0.1830867741
  
 
  
 19
 
  53758233
 
  53758309
 
  PDF   STATS   ENSEMBL   UCSC 
 
   77
 
  4
 
 21026
 
 tss
 
 0.235898
 
 0.0119834
 
 Not queried
 
  4.8107570
 
 2.86e-02
 
 0.1830867741
  
 
  
 4
 
 111551642
 
 111552040
 
  PDF   STATS   ENSEMBL   UCSC 
 
  399
 
  4
 
 30097
 
 gene;island
 
 0.027254
 
 0.3072172
 
 Not queried
 
  4.7819085
 
 2.86e-02
 
 0.1830867741
  
 
  
 4
 
 154125208
 
 154125720
 
  PDF   STATS   ENSEMBL   UCSC 
 
  513
 
  9
 
 30152
 
 gene
 
 0.649592
 
 0.9464361
 
 Not queried
 
  4.7617563
 
 1.88e-02
 
 0.1830867741
  
 
  
 21
 
  27372387
 
  27372461
 
  PDF   STATS   ENSEMBL   UCSC 
 
   75
 
  4
 
 26392
 
 gene
 
 0.033666
 
 0.4811059
 
 Not queried
 
  4.7615545
 
 2.86e-02
 
 0.1830867741
  
 
  
 8
 
 105342214
 
 105342491
 
  PDF   STATS   ENSEMBL   UCSC 
 
  278
 
  4
 
 40522
 
 island
 
 0.016720
 
 0.3133816
 
 Not queried
 
  4.7549880
 
 2.86e-02
 
 0.1830867741
  
 
  
 3
 
 112013079
 
 112013357
 
  PDF   STATS   ENSEMBL   UCSC 
 
  279
 
  4
 
 28501
 
 tss
 
 0.664826
 
 0.9812223
 
 Not queried
 
  4.7544458
 
 2.86e-02
 
 0.1830867741
  
 
  
 10
 
 125425583
 
 125425692
 
  PDF   STATS   ENSEMBL   UCSC 
 
  110
 
  5
 
 5419
 
 tss;island
 
 0.019683
 
 0.6236136
 
 Not queried
 
  4.7504753
 
 1.59e-02
 
 0.1830867741
  
 
  
 11
 
  73358518
 
  73358657
 
  PDF   STATS   ENSEMBL   UCSC 
 
  140
 
  4
 
 6822
 
 gene;tss
 
 0.351410
 
 0.0142168
 
 Not queried
 
  4.7124555
 
 2.86e-02
 
 0.1830867741
  
 
  
 12
 
  23998921
 
  23999045
 
  PDF   STATS   ENSEMBL   UCSC 
 
  125
 
  4
 
 9082
 
 gene
 
 0.467753
 
 0.9454656
 
 Not queried
 
  4.6853439
 
 2.86e-02
 
 0.1830867741
  
 
  
 1
 
  20669724
 
  20669975
 
  PDF   STATS   ENSEMBL   UCSC 
 
  252
 
  4
 
 287
 
 gene;island
 
 0.044276
 
 0.4179377
 
 Not queried
 
  4.6725470
 
 2.86e-02
 
 0.1830867741
  
 
  
 2
 
  50201382
 
  50201511
 
  PDF   STATS   ENSEMBL   UCSC 
 
  130
 
  5
 
 22675
 
 gene
 
 0.016937
 
 0.5079488
 
 Not queried
 
  4.6475245
 
 1.59e-02
 
 0.1830867741
  
 
  
 1
 
  32410413
 
  32410678
 
  PDF   STATS   ENSEMBL   UCSC 
 
  266
 
  4
 
 2979
 
 island
 
 0.029360
 
 0.3300163
 
 Not queried
 
  4.6450770
 
 2.86e-02
 
 0.1830867741
  
 
  
 20
 
  13976093
 
  13976143
 
  PDF   STATS   ENSEMBL   UCSC 
 
   51
 
  6
 
 25605
 
 tss
 
 0.965744
 
 0.3174987
 
 Not queried
 
  4.6365916
 
 2.60e-02
 
 0.1830867741
  
 
  
 2
 
  20865651
 
  20865847
 
  PDF   STATS   ENSEMBL   UCSC 
 
  197
 
  4
 
 23214
 
 tss;island
 
 0.010929
 
 0.1713343
 
 Not queried
 
  4.6167743
 
 2.86e-02
 
 0.1830867741
  
 
  
 1
 
 156696747
 
 156697041
 
  PDF   STATS   ENSEMBL   UCSC 
 
  295
 
  4
 
 828
 
 gene;island
 
 0.034085
 
 0.4764645
 
 Not queried
 
  4.5657683
 
 2.86e-02
 
 0.1830867741
  
 
  
 5
 
 140473857
 
 140474184
 
  PDF   STATS   ENSEMBL   UCSC 
 
  328
 
  6
 
 32361
 
 tss
 
 0.028922
 
 0.4164774
 
 Not queried
 
  4.5599454
 
 2.60e-02
 
 0.1830867741
  
 
  
 6
 
  24358304
 
  24358327
 
  PDF   STATS   ENSEMBL   UCSC 
 
   24
 
  4
 
 34667
 
 tss
 
 0.022145
 
 0.3741606
 
 Not queried
 
  4.5540173
 
 2.86e-02
 
 0.1830867741
  
 
  
 7
 
 143582146
 
 143582630
 
  PDF   STATS   ENSEMBL   UCSC 
 
  485
 
  4
 
 37256
 
 gene;island
 
 0.224821
 
 0.0094937
 
 Not queried
 
  4.5149598
 
 2.86e-02
 
 0.1830867741
  
 
  
 20
 
  23015899
 
  23015936
 
  PDF   STATS   ENSEMBL   UCSC 
 
   38
 
  5
 
 25635
 
 tss;island
 
 0.512118
 
 0.0368236
 
 Not queried
 
  4.5144453
 
 1.59e-02
 
 0.1830867741
  
 
  
 8
 
  72757787
 
  72758095
 
  PDF   STATS   ENSEMBL   UCSC 
 
  309
 
  4
 
 39764
 
 tss;island
 
 0.030796
 
 0.4146138
 
 Not queried
 
  4.5120747
 
 2.86e-02
 
 0.1830867741
  
 
  
 12
 
  49488554
 
  49488898
 
  PDF   STATS   ENSEMBL   UCSC 
 
  345
 
  4
 
 10385
 
 island
 
 0.011572
 
 0.1002816
 
 Not queried
 
  4.5069971
 
 2.86e-02
 
 0.1830867741
  
 
  
 19
 
  51141227
 
  51141413
 
  PDF   STATS   ENSEMBL   UCSC 
 
  187
 
  4
 
 20180
 
 gene;island
 
 0.100668
 
 0.4914495
 
 Not queried
 
  4.4857775
 
 2.86e-02
 
 0.1830867741
  
 
  
 6
 
 132945515
 
 132945592
 
  PDF   STATS   ENSEMBL   UCSC 
 
   78
 
  4
 
 35229
 
 tss
 
 0.803828
 
 0.9874499
 
 Not queried
 
  4.4688000
 
 2.86e-02
 
 0.1830867741
  
 
  
 2
 
  45397776
 
  45398036
 
  PDF   STATS   ENSEMBL   UCSC 
 
  261
 
  4
 
 24270
 
 island
 
 0.329663
 
 0.8258134
 
 Not queried
 
  4.4615329
 
 2.86e-02
 
 0.1830867741
  
 
  
 11
 
 129817591
 
 129817717
 
  PDF   STATS   ENSEMBL   UCSC 
 
  127
 
  4
 
 7814
 
 tss
 
 0.497435
 
 0.2067574
 
 Not queried
 
  4.4316431
 
 2.86e-02
 
 0.1830867741
  
 
  
 19
 
  38794554
 
  38794664
 
  PDF   STATS   ENSEMBL   UCSC 
 
  111
 
  4
 
 20749
 
 tss
 
 0.831982
 
 0.1888633
 
 Not queried
 
  4.4248145
 
 2.86e-02
 
 0.1830867741
  
 
  
 11
 
  63258712
 
  63258803
 
  PDF   STATS   ENSEMBL   UCSC 
 
   92
 
  6
 
 7375
 
 tss
 
 0.006487
 
 0.0659551
 
 Not queried
 
  4.3959312
 
 1.52e-02
 
 0.1830867741
  
 
  
 10
 
  75531971
 
  75532169
 
  PDF   STATS   ENSEMBL   UCSC 
 
  199
 
  6
 
 5856
 
 island
 
 0.120887
 
 0.0049145
 
 Not queried
 
  4.3774706
 
 1.52e-02
 
 0.1830867741
  
 
  
 3
 
 141087187
 
 141087363
 
  PDF   STATS   ENSEMBL   UCSC 
 
  177
 
  5
 
 27973
 
 gene
 
 0.964923
 
 0.5638574
 
 Not queried
 
  4.3453777
 
 1.59e-02
 
 0.1830867741
  
 
  
 5
 
 140105626
 
 140105729
 
  PDF   STATS   ENSEMBL   UCSC 
 
  104
 
  6
 
 32351
 
 tss
 
 0.006287
 
 0.1908755
 
 Not queried
 
  4.3084963
 
 2.60e-02
 
 0.1830867741
  
 
  
 17
 
   3375469
 
   3375601
 
  PDF   STATS   ENSEMBL   UCSC 
 
  133
 
  5
 
 17011
 
 tss;island
 
 0.452920
 
 0.7306629
 
 Not queried
 
  4.3062084
 
 1.59e-02
 
 0.1830867741
  
 
  
 11
 
  72929368
 
  72929553
 
  PDF   STATS   ENSEMBL   UCSC 
 
  186
 
  4
 
 6819
 
 gene
 
 0.291141
 
 0.0132261
 
 Not queried
 
  4.3021855
 
 2.86e-02
 
 0.1830867741
  
 
  
 17
 
    154410
 
    154671
 
  PDF   STATS   ENSEMBL   UCSC 
 
  262
 
  4
 
 16288
 
 gene;island
 
 0.044994
 
 0.5240917
 
 Not queried
 
  4.2945026
 
 2.86e-02
 
 0.1830867741
  
 
  
 15
 
  60296809
 
  60296996
 
  PDF   STATS   ENSEMBL   UCSC 
 
  188
 
  4
 
 13368
 
 gene;island
 
 0.413742
 
 0.0286120
 
 Not queried
 
  4.2622949
 
 2.86e-02
 
 0.1830867741
  
 
  
 3
 
  50541011
 
  50541195
 
  PDF   STATS   ENSEMBL   UCSC 
 
  185
 
  6
 
 28358
 
 tss;island
 
 0.128567
 
 0.0055968
 
 Not queried
 
  4.2609281
 
 2.60e-02
 
 0.1830867741
  
 
  
 7
 
  44104860
 
  44105166
 
  PDF   STATS   ENSEMBL   UCSC 
 
  307
 
  4
 
 36989
 
 gene
 
 0.124768
 
 0.6634237
 
 Not queried
 
  4.2541899
 
 2.86e-02
 
 0.1830867741
  
 
  
 12
 
 133022423
 
 133022955
 
  PDF   STATS   ENSEMBL   UCSC 
 
  533
 
  6
 
 11058
 
 island
 
 0.942182
 
 0.4663202
 
 Not queried
 
  4.2184654
 
 2.60e-02
 
 0.1830867741
  
 
  
 17
 
   7323540
 
   7323660
 
  PDF   STATS   ENSEMBL   UCSC 
 
  121
 
  4
 
 17080
 
 tss
 
 0.019639
 
 0.2766157
 
 Not queried
 
  4.2141447
 
 2.86e-02
 
 0.1830867741
  
 
  
 6
 
 100911949
 
 100912168
 
  PDF   STATS   ENSEMBL   UCSC 
 
  220
 
  4
 
 35140
 
 tss;island
 
 0.063734
 
 0.4268395
 
 Not queried
 
  4.2095611
 
 2.86e-02
 
 0.1830867741
  
 
  
 14
 
  61748243
 
  61748440
 
  PDF   STATS   ENSEMBL   UCSC 
 
  198
 
  4
 
 12105
 
 gene;island
 
 0.045643
 
 0.4363914
 
 Not queried
 
  4.1821509
 
 2.86e-02
 
 0.1830867741
  
 
  
 17
 
   3819339
 
   3819429
 
  PDF   STATS   ENSEMBL   UCSC 
 
   91
 
  4
 
 16349
 
 gene
 
 0.011213
 
 0.1554554
 
 Not queried
 
  4.1708948
 
 2.86e-02
 
 0.1830867741
  
 
  
 17
 
   7323540
 
   7323730
 
  PDF   STATS   ENSEMBL   UCSC 
 
  191
 
  5
 
 17946
 
 island
 
 0.021585
 
 0.2974361
 
 Not queried
 
  4.1596631
 
 1.59e-02
 
 0.1830867741
  
 
  
 6
 
  84562778
 
  84562930
 
  PDF   STATS   ENSEMBL   UCSC 
 
  153
 
  7
 
 35097
 
 tss
 
 0.017981
 
 0.2438455
 
 Not queried
 
  4.1578185
 
 1.75e-02
 
 0.1830867741
  
 
  
 3
 
 139653328
 
 139653573
 
  PDF   STATS   ENSEMBL   UCSC 
 
  246
 
  4
 
 28634
 
 tss;island
 
 0.006524
 
 0.0674136
 
 Not queried
 
  4.1025296
 
 2.86e-02
 
 0.1830867741
  
 
  
 8
 
  80523191
 
  80523461
 
  PDF   STATS   ENSEMBL   UCSC 
 
  271
 
  6
 
 40408
 
 island
 
 0.071058
 
 0.5490984
 
 Not queried
 
  4.1009150
 
 1.52e-02
 
 0.1830867741
  
 
  
 10
 
  95848649
 
  95848841
 
  PDF   STATS   ENSEMBL   UCSC 
 
  193
 
  4
 
 4685
 
 gene
 
 0.015329
 
 0.1791836
 
 Not queried
 
  4.0876629
 
 2.86e-02
 
 0.1830867741
  
 
  
 1
 
 231176985
 
 231177201
 
  PDF   STATS   ENSEMBL   UCSC 
 
  217
 
  4
 
 2320
 
 tss;island
 
 0.030851
 
 0.4045533
 
 Not queried
 
  4.0823101
 
 2.86e-02
 
 0.1830867741
  
 
  
 10
 
  50341989
 
  50342145
 
  PDF   STATS   ENSEMBL   UCSC 
 
  157
 
  4
 
 5730
 
 island
 
 0.414634
 
 0.9031739
 
 Not queried
 
  4.0663894
 
 2.86e-02
 
 0.1830867741
  
 
  
 13
 
  21900392
 
  21900591
 
  PDF   STATS   ENSEMBL   UCSC 
 
  200
 
  4
 
 11589
 
 island
 
 0.096902
 
 0.7359108
 
 Not queried
 
  4.0535574
 
 2.86e-02
 
 0.1830867741
  
 
  
 6
 
 108492642
 
 108492769
 
  PDF   STATS   ENSEMBL   UCSC 
 
  128
 
  4
 
 34439
 
 gene;island
 
 0.018495
 
 0.1343185
 
 Not queried
 
  4.0360379
 
 2.86e-02
 
 0.1830867741
  
 
  
 4
 
  46391259
 
  46391929
 
  PDF   STATS   ENSEMBL   UCSC 
 
  671
 
 13
 
 29995
 
 gene;island
 
 0.009602
 
 0.1010069
 
 Not queried
 
  4.0340972
 
 1.40e-02
 
 0.1830867741
  
 
  
 4
 
  46391414
 
  46391929
 
  PDF   STATS   ENSEMBL   UCSC 
 
  516
 
 11
 
 30372
 
 tss
 
 0.009602
 
 0.1010069
 
 Not queried
 
  4.0340972
 
 1.28e-02
 
 0.1830867741
  
 
  
 19
 
  38794554
 
  38794845
 
  PDF   STATS   ENSEMBL   UCSC 
 
  292
 
  6
 
 20014
 
 gene
 
 0.864332
 
 0.2723031
 
 Not queried
 
  4.0221234
 
 2.60e-02
 
 0.1830867741
  
 
  
 10
 
  29834018
 
  29834266
 
  PDF   STATS   ENSEMBL   UCSC 
 
  249
 
  4
 
 4547
 
 gene
 
 0.299761
 
 0.8506115
 
 Not queried
 
  4.0211681
 
 2.86e-02
 
 0.1830867741
  
 
  
 17
 
  78700906
 
  78701257
 
  PDF   STATS   ENSEMBL   UCSC 
 
  352
 
  4
 
 16871
 
 gene;island
 
 0.332682
 
 0.9167238
 
 Not queried
 
  4.0068047
 
 2.86e-02
 
 0.1830867741
  
 
  
 1
 
  47900197
 
  47900320
 
  PDF   STATS   ENSEMBL   UCSC 
 
  124
 
  4
 
 1610
 
 tss;island
 
 0.601565
 
 0.1296102
 
 Not queried
 
  4.0048889
 
 2.86e-02
 
 0.1830867741
  
 
  
 2
 
 223163725
 
 223163809
 
  PDF   STATS   ENSEMBL   UCSC 
 
   85
 
  4
 
 23916
 
 tss
 
 0.034811
 
 0.2862013
 
 Not queried
 
  3.9828466
 
 2.86e-02
 
 0.1830867741
  
 
  
 4
 
  94748617
 
  94748860
 
  PDF   STATS   ENSEMBL   UCSC 
 
  244
 
  4
 
 30500
 
 tss;island
 
 0.480003
 
 0.0576001
 
 Not queried
 
  3.9292472
 
 2.86e-02
 
 0.1830867741
  
 
  
 20
 
   1471884
 
   1472098
 
  PDF   STATS   ENSEMBL   UCSC 
 
  215
 
  4
 
 25329
 
 gene
 
 0.046458
 
 0.4230188
 
 Not queried
 
  3.9145824
 
 2.86e-02
 
 0.1830867741
  
 
  
 17
 
  50237205
 
  50237844
 
  PDF   STATS   ENSEMBL   UCSC 
 
  640
 
  9
 
 17515
 
 tss;island
 
 0.017996
 
 0.3111019
 
 Not queried
 
  3.9027449
 
 1.88e-02
 
 0.1830867741
  
 
  
 18
 
  49868101
 
  49868552
 
  PDF   STATS   ENSEMBL   UCSC 
 
  452
 
  7
 
 19116
 
 gene;island
 
 0.055344
 
 0.5090845
 
 Not queried
 
  3.8846197
 
 2.62e-02
 
 0.1830867741
  
 
  
 7
 
  23287222
 
  23287560
 
  PDF   STATS   ENSEMBL   UCSC 
 
  339
 
  4
 
 36914
 
 gene;island
 
 0.152555
 
 0.0151860
 
 Not queried
 
  3.8824713
 
 2.86e-02
 
 0.1830867741
  
 
  
 18
 
   5891948
 
   5892245
 
  PDF   STATS   ENSEMBL   UCSC 
 
  298
 
  4
 
 19376
 
 island
 
 0.114238
 
 0.6425463
 
 Not queried
 
  3.8807294
 
 2.86e-02
 
 0.1830867741
  
 
  
 15
 
  79724739
 
  79724802
 
  PDF   STATS   ENSEMBL   UCSC 
 
   64
 
  4
 
 13845
 
 tss;island
 
 0.047337
 
 0.3531323
 
 Not queried
 
  3.8666119
 
 2.86e-02
 
 0.1830867741
  
 
  
 7
 
  95402189
 
  95402528
 
  PDF   STATS   ENSEMBL   UCSC 
 
  340
 
  4
 
 37099
 
 gene;island
 
 0.037608
 
 0.3221802
 
 Not queried
 
  3.8392016
 
 2.86e-02
 
 0.1830867741
  
 
  
 11
 
  65816655
 
  65816985
 
  PDF   STATS   ENSEMBL   UCSC 
 
  331
 
  6
 
 7457
 
 tss
 
 0.099287
 
 0.7064180
 
 Not queried
 
  3.8380975
 
 2.60e-02
 
 0.1830867741
  
 
  
 21
 
  45705175
 
  45705742
 
  PDF   STATS   ENSEMBL   UCSC 
 
  568
 
 10
 
 26582
 
 tss;island
 
 0.535180
 
 0.9210059
 
 Not queried
 
  3.8354049
 
 2.32e-02
 
 0.1830867741
  
 
  
 5
 
  79330669
 
  79331135
 
  PDF   STATS   ENSEMBL   UCSC 
 
  467
 
  7
 
 32918
 
 island
 
 0.522758
 
 0.0234624
 
 Not queried
 
  3.8186070
 
 2.62e-02
 
 0.1830867741
  
 
  
 1
 
  54821853
 
  54822298
 
  PDF   STATS   ENSEMBL   UCSC 
 
  446
 
  6
 
 548
 
 gene;island
 
 0.131434
 
 0.5678403
 
 Not queried
 
  3.8040316
 
 2.60e-02
 
 0.1830867741
  
 
  
 6
 
  83073156
 
  83073335
 
  PDF   STATS   ENSEMBL   UCSC 
 
  180
 
  4
 
 34400
 
 gene;tss;island
 
 0.018892
 
 0.2987068
 
 Not queried
 
  3.7813271
 
 2.86e-02
 
 0.1830867741
  
 
  
 7
 
   4832112
 
   4832535
 
  PDF   STATS   ENSEMBL   UCSC 
 
  424
 
  5
 
 38201
 
 island
 
 0.968040
 
 0.5590610
 
 Not queried
 
  3.7715401
 
 1.59e-02
 
 0.1830867741
  
 
  
 11
 
  76381044
 
  76381236
 
  PDF   STATS   ENSEMBL   UCSC 
 
  193
 
  6
 
 7576
 
 tss
 
 0.020703
 
 0.4276290
 
 Not queried
 
  3.7692199
 
 1.52e-02
 
 0.1830867741
  
 
  
 1
 
 201857840
 
 201857955
 
  PDF   STATS   ENSEMBL   UCSC 
 
  116
 
  6
 
 942
 
 gene
 
 0.249450
 
 0.0292499
 
 Not queried
 
  3.7669695
 
 1.52e-02
 
 0.1830867741
  
 
  
 5
 
   1416793
 
   1417003
 
  PDF   STATS   ENSEMBL   UCSC 
 
  211
 
  4
 
 31538
 
 gene;island
 
 0.639431
 
 0.9423936
 
 Not queried
 
  3.7633060
 
 2.86e-02
 
 0.1830867741
  
 
  
 7
 
  27170412
 
  27171528
 
  PDF   STATS   ENSEMBL   UCSC 
 
 1117
 
 18
 
 37491
 
 tss
 
 0.872610
 
 0.2539824
 
 Not queried
 
  3.7583264
 
 1.42e-02
 
 0.1830867741
  
 
  
 10
 
 129535138
 
 129535509
 
  PDF   STATS   ENSEMBL   UCSC 
 
  372
 
  6
 
 5436
 
 tss
 
 0.013752
 
 0.1889653
 
 Not queried
 
  3.7307480
 
 2.60e-02
 
 0.1830867741
  
 
  
 10
 
  91597207
 
  91597593
 
  PDF   STATS   ENSEMBL   UCSC 
 
  387
 
  4
 
 5927
 
 island
 
 0.043749
 
 0.4905347
 
 Not queried
 
  3.7046908
 
 2.86e-02
 
 0.1830867741
  
 
  
 11
 
    637032
 
    637175
 
  PDF   STATS   ENSEMBL   UCSC 
 
  144
 
  7
 
 7021
 
 tss;island
 
 0.022499
 
 0.1574262
 
 Not queried
 
  3.7009768
 
 2.62e-02
 
 0.1830867741
  
 
  
 1
 
   1293868
 
   1294140
 
  PDF   STATS   ENSEMBL   UCSC 
 
  273
 
  4
 
 2429
 
 island
 
 0.061033
 
 0.4190615
 
 Not queried
 
  3.6949254
 
 2.86e-02
 
 0.1830867741
  
 
  
 5
 
  63461216
 
  63461930
 
  PDF   STATS   ENSEMBL   UCSC 
 
  715
 
 10
 
 32828
 
 island
 
 0.005984
 
 0.2497589
 
 Not queried
 
  3.6780591
 
 2.32e-02
 
 0.1830867741
  
 
  
 12
 
  81331457
 
  81331660
 
  PDF   STATS   ENSEMBL   UCSC 
 
  204
 
  4
 
 9227
 
 gene
 
 0.071927
 
 0.0057282
 
 Not queried
 
  3.6728279
 
 2.86e-02
 
 0.1830867741
  
 
  
 10
 
 119302050
 
 119302318
 
  PDF   STATS   ENSEMBL   UCSC 
 
  269
 
  8
 
 4785
 
 gene;island
 
 0.295870
 
 0.0127514
 
 Not queried
 
  3.6472135
 
 2.81e-02
 
 0.1830867741
  
 
  
 4
 
   1043421
 
   1043631
 
  PDF   STATS   ENSEMBL   UCSC 
 
  211
 
  5
 
 30762
 
 island
 
 0.485973
 
 0.7521422
 
 Not queried
 
  3.6349386
 
 1.59e-02
 
 0.1830867741
  
 
  
 22
 
  42095154
 
  42095536
 
  PDF   STATS   ENSEMBL   UCSC 
 
  383
 
  7
 
 27529
 
 island
 
 0.484775
 
 0.0925817
 
 Not queried
 
  3.6292560
 
 2.62e-02
 
 0.1830867741
  
 
  
 10
 
 100995599
 
 100995821
 
  PDF   STATS   ENSEMBL   UCSC 
 
  223
 
  4
 
 6005
 
 island
 
 0.108431
 
 0.0220037
 
 Not queried
 
  3.6243713
 
 2.86e-02
 
 0.1830867741
  
 
  
 13
 
  28493913
 
  28494177
 
  PDF   STATS   ENSEMBL   UCSC 
 
  265
 
  8
 
 11633
 
 island
 
 0.551103
 
 0.0625737
 
 Not queried
 
  3.6137104
 
 2.81e-02
 
 0.1830867741
  
 
  
 3
 
  15311021
 
  15311269
 
  PDF   STATS   ENSEMBL   UCSC 
 
  249
 
  4
 
 27690
 
 gene
 
 0.429156
 
 0.0694086
 
 Not queried
 
  3.5964149
 
 2.86e-02
 
 0.1830867741
  
 
  
 10
 
  15761854
 
  15762312
 
  PDF   STATS   ENSEMBL   UCSC 
 
  459
 
  8
 
 4964
 
 tss
 
 0.054852
 
 0.4355350
 
 Not queried
 
  3.5877485
 
 2.07e-02
 
 0.1830867741
  
 
  
 1
 
 175568216
 
 175568710
 
  PDF   STATS   ENSEMBL   UCSC 
 
  495
 
  4
 
 895
 
 gene;island
 
 0.012066
 
 0.0303908
 
 Not queried
 
  3.5681884
 
 2.86e-02
 
 0.1830867741
  
 
  
 6
 
  31631638
 
  31631801
 
  PDF   STATS   ENSEMBL   UCSC 
 
  164
 
  7
 
 33931
 
 gene;island
 
 0.388209
 
 0.9096620
 
 Not queried
 
  3.5557997
 
 1.75e-02
 
 0.1830867741
  
 
  
 17
 
   8702698
 
   8702896
 
  PDF   STATS   ENSEMBL   UCSC 
 
  199
 
  6
 
 17122
 
 tss
 
 0.615246
 
 0.9487782
 
 Not queried
 
  3.5513509
 
 1.52e-02
 
 0.1830867741
  
 
  
 1
 
   1897585
 
   1897959
 
  PDF   STATS   ENSEMBL   UCSC 
 
  375
 
  4
 
 51
 
 gene;island
 
 0.147175
 
 0.4683700
 
 Not queried
 
  3.5344228
 
 2.86e-02
 
 0.1830867741
  
 
  
 8
 
  22560922
 
  22561238
 
  PDF   STATS   ENSEMBL   UCSC 
 
  317
 
  5
 
 40142
 
 island
 
 0.098048
 
 0.3303944
 
 Not queried
 
  3.5337470
 
 1.59e-02
 
 0.1830867741
  
 
  
 19
 
  18548929
 
  18549274
 
  PDF   STATS   ENSEMBL   UCSC 
 
  346
 
  7
 
 20619
 
 tss
 
 0.019897
 
 0.1919573
 
 Not queried
 
  3.5309296
 
 1.75e-02
 
 0.1830867741
  
 
  
 8
 
  17271067
 
  17271119
 
  PDF   STATS   ENSEMBL   UCSC 
 
   53
 
  4
 
 39566
 
 tss;island
 
 0.094621
 
 0.5346114
 
 Not queried
 
  3.4967914
 
 2.86e-02
 
 0.1830867741
  
 
  
 7
 
 149569715
 
 149570040
 
  PDF   STATS   ENSEMBL   UCSC 
 
  326
 
  8
 
 37972
 
 tss
 
 0.016180
 
 0.5613354
 
 Not queried
 
  3.4847931
 
 2.81e-02
 
 0.1830867741
  
 
  
 1
 
 145473005
 
 145473392
 
  PDF   STATS   ENSEMBL   UCSC 
 
  388
 
  6
 
 725
 
 gene;island
 
 0.004405
 
 0.0458573
 
 Not queried
 
  3.4584551
 
 1.52e-02
 
 0.1830867741
  
 
  
 12
 
 103311088
 
 103311761
 
  PDF   STATS   ENSEMBL   UCSC 
 
  674
 
  9
 
 10726
 
 island
 
 0.020166
 
 0.1515947
 
 Not queried
 
  3.4547587
 
 1.88e-02
 
 0.1830867741
  
 
  
 8
 
  65291287
 
  65291703
 
  PDF   STATS   ENSEMBL   UCSC 
 
  417
 
  8
 
 39733
 
 tss
 
 0.063410
 
 0.3499809
 
 Not queried
 
  3.4392815
 
 2.07e-02
 
 0.1830867741
  
 
  
 19
 
  46526333
 
  46526675
 
  PDF   STATS   ENSEMBL   UCSC 
 
  343
 
  7
 
 20889
 
 tss
 
 0.038033
 
 0.4326887
 
 Not queried
 
  3.4253485
 
 2.62e-02
 
 0.1830867741
  
 
  
 5
 
  87968528
 
  87968749
 
  PDF   STATS   ENSEMBL   UCSC 
 
  222
 
  4
 
 31677
 
 gene;island
 
 0.572392
 
 0.1786603
 
 Not queried
 
  3.4135985
 
 2.86e-02
 
 0.1830867741
  
 
  
 2
 
 177001256
 
 177001426
 
  PDF   STATS   ENSEMBL   UCSC 
 
  171
 
  4
 
 24906
 
 island
 
 0.111067
 
 0.5733336
 
 Not queried
 
  3.4042581
 
 2.86e-02
 
 0.1830867741
  
 
  
 13
 
  36705446
 
  36705622
 
  PDF   STATS   ENSEMBL   UCSC 
 
  177
 
  5
 
 11665
 
 island
 
 0.019439
 
 0.0707086
 
 Not queried
 
  3.3863985
 
 1.59e-02
 
 0.1830867741
  
 
  
 1
 
  85512957
 
  85513315
 
  PDF   STATS   ENSEMBL   UCSC 
 
  359
 
  4
 
 604
 
 gene;island
 
 0.615441
 
 0.9507791
 
 Not queried
 
  3.3753286
 
 2.86e-02
 
 0.1830867741
  
 
  
 19
 
  57018848
 
  57019069
 
  PDF   STATS   ENSEMBL   UCSC 
 
  222
 
  6
 
 21099
 
 tss;island
 
 0.096676
 
 0.5049068
 
 Not queried
 
  3.3598572
 
 2.60e-02
 
 0.1830867741
  
 
  
 13
 
  61989701
 
  61990025
 
  PDF   STATS   ENSEMBL   UCSC 
 
  325
 
  8
 
 11470
 
 tss
 
 0.105035
 
 0.5444479
 
 Not queried
 
  3.3577151
 
 2.81e-02
 
 0.1830867741
  
 
  
 1
 
  60392140
 
  60392392
 
  PDF   STATS   ENSEMBL   UCSC 
 
  253
 
  4
 
 560
 
 gene
 
 0.019618
 
 0.1847350
 
 Not queried
 
  3.3487152
 
 2.86e-02
 
 0.1830867741
  
 
  
 1
 
  60392140
 
  60392567
 
  PDF   STATS   ENSEMBL   UCSC 
 
  428
 
  6
 
 3326
 
 island
 
 0.019618
 
 0.1797915
 
 Not queried
 
  3.3487152
 
 1.52e-02
 
 0.1830867741
  
 
  
 22
 
  31317764
 
  31318240
 
  PDF   STATS   ENSEMBL   UCSC 
 
  477
 
  8
 
 27070
 
 tss
 
 0.003926
 
 0.3924808
 
 Not queried
 
  3.3225626
 
 1.48e-02
 
 0.1830867741
  
 
  
 10
 
 124907409
 
 124907708
 
  PDF   STATS   ENSEMBL   UCSC 
 
  300
 
  6
 
 6217
 
 island
 
 0.271887
 
 0.0190546
 
 Not queried
 
  3.3155342
 
 1.52e-02
 
 0.1830867741
  
 
  
 3
 
 178984841
 
 178984973
 
  PDF   STATS   ENSEMBL   UCSC 
 
  133
 
  4
 
 28730
 
 tss
 
 0.125843
 
 0.0300821
 
 Not queried
 
  3.2728199
 
 2.86e-02
 
 0.1830867741
  
 
  
 17
 
  48473757
 
  48474099
 
  PDF   STATS   ENSEMBL   UCSC 
 
  343
 
  4
 
 16686
 
 gene;island
 
 0.008887
 
 0.0933201
 
 Not queried
 
  3.2724497
 
 2.86e-02
 
 0.1830867741
  
 
  
 15
 
  66947392
 
  66947617
 
  PDF   STATS   ENSEMBL   UCSC 
 
  226
 
  4
 
 14245
 
 island
 
 0.625199
 
 0.2084743
 
 Not queried
 
  3.2583591
 
 2.86e-02
 
 0.1830867741
  
 
  
 19
 
  40732518
 
  40732902
 
  PDF   STATS   ENSEMBL   UCSC 
 
  385
 
  9
 
 22017
 
 island
 
 0.100507
 
 0.5155647
 
 Not queried
 
  3.2527197
 
 1.42e-02
 
 0.1830867741
  
 
  
 11
 
  20690628
 
  20690957
 
  PDF   STATS   ENSEMBL   UCSC 
 
  330
 
  7
 
 7200
 
 tss
 
 0.017589
 
 0.2448600
 
 Not queried
 
  3.2417260
 
 2.62e-02
 
 0.1830867741
  
 
  
 2
 
 103236078
 
 103236300
 
  PDF   STATS   ENSEMBL   UCSC 
 
  223
 
  4
 
 24574
 
 island
 
 0.044063
 
 0.2343419
 
 Not queried
 
  3.2363716
 
 2.86e-02
 
 0.1830867741
  
 
  
 4
 
   5570187
 
   5570306
 
  PDF   STATS   ENSEMBL   UCSC 
 
  120
 
  4
 
 29920
 
 gene;island
 
 0.756328
 
 0.9633936
 
 Not queried
 
  3.2290837
 
 2.86e-02
 
 0.1830867741
  
 
  
 11
 
  93063516
 
  93063692
 
  PDF   STATS   ENSEMBL   UCSC 
 
  177
 
  4
 
 7619
 
 tss
 
 0.010522
 
 0.0789882
 
 Not queried
 
  3.2287560
 
 2.86e-02
 
 0.1830867741
  
 
  
 5
 
 140514711
 
 140515207
 
  PDF   STATS   ENSEMBL   UCSC 
 
  497
 
  6
 
 33212
 
 island
 
 0.040026
 
 0.3680801
 
 Not queried
 
  3.2190439
 
 1.52e-02
 
 0.1830867741
  
 
  
 13
 
  47472050
 
  47472429
 
  PDF   STATS   ENSEMBL   UCSC 
 
  380
 
 12
 
 11429
 
 tss
 
 0.392696
 
 0.7830259
 
 Not queried
 
  3.2147177
 
 2.05e-02
 
 0.1830867741
  
 
  
 4
 
  81187601
 
  81187906
 
  PDF   STATS   ENSEMBL   UCSC 
 
  306
 
  5
 
 31136
 
 island
 
 0.013711
 
 0.1160797
 
 Not queried
 
  3.2108480
 
 1.59e-02
 
 0.1830867741
  
 
  
 6
 
  99290284
 
  99290737
 
  PDF   STATS   ENSEMBL   UCSC 
 
  454
 
  5
 
 36345
 
 island
 
 0.028818
 
 0.2134832
 
 Not queried
 
  3.2047619
 
 1.59e-02
 
 0.1830867741
  
 
  
 2
 
 133426129
 
 133426418
 
  PDF   STATS   ENSEMBL   UCSC 
 
  290
 
  4
 
 22878
 
 gene;island
 
 0.061793
 
 0.3077727
 
 Not queried
 
  3.1791815
 
 2.86e-02
 
 0.1830867741
  
 
  
 12
 
 104697193
 
 104697631
 
  PDF   STATS   ENSEMBL   UCSC 
 
  439
 
 12
 
 9273
 
 gene;island
 
 0.074641
 
 0.0233017
 
 Not queried
 
  3.1788935
 
 2.42e-02
 
 0.1830867741
  
 
  
 13
 
 100626995
 
 100627366
 
  PDF   STATS   ENSEMBL   UCSC 
 
  372
 
  4
 
 11846
 
 island
 
 0.038939
 
 0.1531425
 
 Not queried
 
  3.1515476
 
 2.86e-02
 
 0.1830867741
  
 
  
 3
 
 181441345
 
 181442137
 
  PDF   STATS   ENSEMBL   UCSC 
 
  793
 
  9
 
 28044
 
 gene;island
 
 0.073266
 
 0.5428204
 
 Not queried
 
  3.1508413
 
 2.44e-02
 
 0.1830867741
  
 
  
 5
 
 140800398
 
 140800983
 
  PDF   STATS   ENSEMBL   UCSC 
 
  586
 
  9
 
 31834
 
 gene;island
 
 0.177756
 
 0.0230619
 
 Not queried
 
  3.1493344
 
 1.88e-02
 
 0.1830867741
  
 
  
 19
 
  58446745
 
  58446988
 
  PDF   STATS   ENSEMBL   UCSC 
 
  244
 
  8
 
 21129
 
 tss
 
 0.130236
 
 0.0201309
 
 Not queried
 
  3.1424041
 
 2.07e-02
 
 0.1830867741
  
 
  
 20
 
  30073209
 
  30073576
 
  PDF   STATS   ENSEMBL   UCSC 
 
  368
 
  6
 
 25660
 
 tss;island
 
 0.463661
 
 0.0683297
 
 Not queried
 
  3.1347476
 
 1.52e-02
 
 0.1830867741
  
 
  
 6
 
  29574700
 
  29575145
 
  PDF   STATS   ENSEMBL   UCSC 
 
  446
 
  7
 
 33667
 
 gene
 
 0.168012
 
 0.3890642
 
 Not queried
 
  3.1003692
 
 1.75e-02
 
 0.1830867741
  
 
  
 8
 
 145625605
 
 145625814
 
  PDF   STATS   ENSEMBL   UCSC 
 
  210
 
  4
 
 39981
 
 tss
 
 0.853023
 
 0.9818844
 
 Not queried
 
  3.0935657
 
 2.86e-02
 
 0.1830867741
  
 
  
 1
 
  40782713
 
  40783264
 
  PDF   STATS   ENSEMBL   UCSC 
 
  552
 
 10
 
 3120
 
 island
 
 0.020692
 
 0.1950846
 
 Not queried
 
  3.0872147
 
 1.85e-02
 
 0.1830867741
  
 
  
 4
 
  41869086
 
  41869611
 
  PDF   STATS   ENSEMBL   UCSC 
 
  526
 
  7
 
 31006
 
 island
 
 0.067774
 
 0.3364103
 
 Not queried
 
  3.0855695
 
 1.75e-02
 
 0.1830867741
  
 
  
 4
 
  13530923
 
  13531213
 
  PDF   STATS   ENSEMBL   UCSC 
 
  291
 
  6
 
 30931
 
 island
 
 0.116466
 
 0.5206456
 
 Not queried
 
  3.0755544
 
 2.60e-02
 
 0.1830867741
  
 
  
 19
 
  50789808
 
  50789973
 
  PDF   STATS   ENSEMBL   UCSC 
 
  166
 
  4
 
 20172
 
 gene;island
 
 0.227766
 
 0.7366940
 
 Not queried
 
  3.0750548
 
 2.86e-02
 
 0.1830867741
  
 
  
 1
 
 227505845
 
 227506021
 
  PDF   STATS   ENSEMBL   UCSC 
 
  177
 
  6
 
 2292
 
 tss;island
 
 0.020768
 
 0.1657895
 
 Not queried
 
  3.0716602
 
 2.60e-02
 
 0.1830867741
  
 
  
 10
 
  13043878
 
  13043986
 
  PDF   STATS   ENSEMBL   UCSC 
 
  109
 
  6
 
 4953
 
 tss;island
 
 0.014056
 
 0.1157171
 
 Not queried
 
  3.0372565
 
 2.60e-02
 
 0.1830867741
  
 
  
 15
 
  96875591
 
  96875830
 
  PDF   STATS   ENSEMBL   UCSC 
 
  240
 
  8
 
 13493
 
 gene;tss;island
 
 0.101365
 
 0.2722256
 
 Not queried
 
  3.0301829
 
 2.81e-02
 
 0.1830867741
  
 
  
 16
 
   3355295
 
   3355553
 
  PDF   STATS   ENSEMBL   UCSC 
 
  259
 
  4
 
 15646
 
 island
 
 0.139602
 
 0.0173491
 
 Not queried
 
  3.0195331
 
 2.86e-02
 
 0.1830867741
  
 
  
 6
 
 126071099
 
 126071538
 
  PDF   STATS   ENSEMBL   UCSC 
 
  440
 
  5
 
 34466
 
 gene;island
 
 0.041152
 
 0.0103129
 
 Not queried
 
  2.9991842
 
 1.59e-02
 
 0.1830867741
  
 
  
 19
 
  12306136
 
  12306497
 
  PDF   STATS   ENSEMBL   UCSC 
 
  362
 
  4
 
 21597
 
 island
 
 0.255375
 
 0.0366453
 
 Not queried
 
  2.9915071
 
 2.86e-02
 
 0.1830867741
  
 
  
 1
 
 186649153
 
 186649530
 
  PDF   STATS   ENSEMBL   UCSC 
 
  378
 
  6
 
 925
 
 gene
 
 0.063801
 
 0.0065812
 
 Not queried
 
  2.9898661
 
 2.60e-02
 
 0.1830867741
  
 
  
 20
 
   5891730
 
   5892091
 
  PDF   STATS   ENSEMBL   UCSC 
 
  362
 
  7
 
 25989
 
 island
 
 0.094335
 
 0.0099436
 
 Not queried
 
  2.9885540
 
 2.62e-02
 
 0.1830867741
  
 
  
 17
 
   4545074
 
   4545157
 
  PDF   STATS   ENSEMBL   UCSC 
 
   84
 
  4
 
 17027
 
 tss
 
 0.334800
 
 0.0497522
 
 Not queried
 
  2.9879022
 
 2.86e-02
 
 0.1830867741
  
 
  
 7
 
  43797839
 
  43798207
 
  PDF   STATS   ENSEMBL   UCSC 
 
  369
 
  7
 
 37558
 
 tss;island
 
 0.009984
 
 0.0625796
 
 Not queried
 
  2.9846394
 
 1.75e-02
 
 0.1830867741
  
 
  
 20
 
  51588780
 
  51588860
 
  PDF   STATS   ENSEMBL   UCSC 
 
   81
 
  4
 
 25828
 
 tss;island
 
 0.010483
 
 0.0685069
 
 Not queried
 
  2.9825650
 
 2.86e-02
 
 0.1830867741
  
 
  
 10
 
 114134878
 
 114135259
 
  PDF   STATS   ENSEMBL   UCSC 
 
  382
 
  5
 
 4761
 
 gene;tss
 
 0.009330
 
 0.0635108
 
 Not queried
 
  2.9821664
 
 1.59e-02
 
 0.1830867741
  
 
  
 10
 
 125651370
 
 125651726
 
  PDF   STATS   ENSEMBL   UCSC 
 
  357
 
  8
 
 6221
 
 island
 
 0.025220
 
 0.4460377
 
 Not queried
 
  2.9355180
 
 2.07e-02
 
 0.1830867741
  
 
  
 19
 
  30866192
 
  30866365
 
  PDF   STATS   ENSEMBL   UCSC 
 
  174
 
  4
 
 19970
 
 gene;island
 
 0.259554
 
 0.0451556
 
 Not queried
 
  2.9316933
 
 2.86e-02
 
 0.1830867741
  
 
  
 14
 
  59931922
 
  59932321
 
  PDF   STATS   ENSEMBL   UCSC 
 
  400
 
 11
 
 12892
 
 island
 
 0.006991
 
 0.0349439
 
 Not queried
 
  2.9192373
 
 2.33e-02
 
 0.1830867741
  
 
  
 2
 
 176948693
 
 176949017
 
  PDF   STATS   ENSEMBL   UCSC 
 
  325
 
  7
 
 23731
 
 tss
 
 0.251150
 
 0.8614819
 
 Not queried
 
  2.9093650
 
 2.62e-02
 
 0.1830867741
  
 
  
 2
 
 176948544
 
 176949017
 
  PDF   STATS   ENSEMBL   UCSC 
 
  474
 
  9
 
 24889
 
 island
 
 0.251150
 
 0.8357278
 
 Not queried
 
  2.9093650
 
 2.44e-02
 
 0.1830867741
  
 
  
 19
 
  46526100
 
  46526675
 
  PDF   STATS   ENSEMBL   UCSC 
 
  576
 
 10
 
 22168
 
 island
 
 0.038626
 
 0.4421039
 
 Not queried
 
  2.8985295
 
 1.47e-02
 
 0.1830867741
  
 
  
 6
 
 112575705
 
 112576119
 
  PDF   STATS   ENSEMBL   UCSC 
 
  415
 
  6
 
 36423
 
 island
 
 0.142186
 
 0.5024326
 
 Not queried
 
  2.8748515
 
 2.60e-02
 
 0.1830867741
  
 
  
 16
 
   8806359
 
   8806756
 
  PDF   STATS   ENSEMBL   UCSC 
 
  398
 
  8
 
 14652
 
 gene;tss
 
 0.067463
 
 0.3118311
 
 Not queried
 
  2.8727517
 
 2.07e-02
 
 0.1830867741
  
 
  
 14
 
  90042201
 
  90042364
 
  PDF   STATS   ENSEMBL   UCSC 
 
  164
 
  4
 
 12171
 
 gene;tss
 
 0.979529
 
 0.8646607
 
 Not queried
 
  2.8588229
 
 2.86e-02
 
 0.1830867741
  
 
  
 12
 
  25102311
 
  25102521
 
  PDF   STATS   ENSEMBL   UCSC 
 
  211
 
  4
 
 9608
 
 tss
 
 0.002829
 
 0.0189065
 
 Not queried
 
  2.8563574
 
 2.86e-02
 
 0.1830867741
  
 
  
 20
 
  36889208
 
  36889389
 
  PDF   STATS   ENSEMBL   UCSC 
 
  182
 
  7
 
 25732
 
 tss
 
 0.091445
 
 0.3858719
 
 Not queried
 
  2.8435524
 
 1.75e-02
 
 0.1830867741
  
 
  
 8
 
  12990898
 
  12991196
 
  PDF   STATS   ENSEMBL   UCSC 
 
  299
 
  7
 
 39560
 
 tss
 
 0.107552
 
 0.0085484
 
 Not queried
 
  2.8331017
 
 2.62e-02
 
 0.1830867741
  
 
  
 3
 
  36902435
 
  36902745
 
  PDF   STATS   ENSEMBL   UCSC 
 
  311
 
  4
 
 27715
 
 gene
 
 0.650937
 
 0.9386729
 
 Not queried
 
  2.8127968
 
 2.86e-02
 
 0.1830867741
  
 
  
 4
 
   4867387
 
   4867638
 
  PDF   STATS   ENSEMBL   UCSC 
 
  252
 
  4
 
 30873
 
 island
 
 0.289721
 
 0.0479401
 
 Not queried
 
  2.8000768
 
 2.86e-02
 
 0.1830867741
  
 
  
 4
 
 183064851
 
 183065224
 
  PDF   STATS   ENSEMBL   UCSC 
 
  374
 
  4
 
 30191
 
 gene;island
 
 0.090929
 
 0.0183712
 
 Not queried
 
  2.7784380
 
 2.86e-02
 
 0.1830867741
  
 
  
 1
 
 154474344
 
 154474530
 
  PDF   STATS   ENSEMBL   UCSC 
 
  187
 
  4
 
 778
 
 gene;tss;island
 
 0.269743
 
 0.0431317
 
 Not queried
 
  2.7775806
 
 2.86e-02
 
 0.1830867741
  
 
  
 2
 
 238777587
 
 238777806
 
  PDF   STATS   ENSEMBL   UCSC 
 
  220
 
  4
 
 23093
 
 gene
 
 0.007723
 
 0.0412056
 
 Not queried
 
  2.7747767
 
 2.86e-02
 
 0.1830867741
  
 
  
 13
 
  88324432
 
  88324711
 
  PDF   STATS   ENSEMBL   UCSC 
 
  280
 
  4
 
 11494
 
 tss
 
 0.020837
 
 0.1192512
 
 Not queried
 
  2.7689234
 
 2.86e-02
 
 0.1830867741
  
 
  
 17
 
   4544925
 
   4545157
 
  PDF   STATS   ENSEMBL   UCSC 
 
  233
 
  5
 
 17873
 
 island
 
 0.215835
 
 0.0428863
 
 Not queried
 
  2.7584587
 
 1.59e-02
 
 0.1830867741
  
 
  
 12
 
  34500585
 
  34500813
 
  PDF   STATS   ENSEMBL   UCSC 
 
  229
 
  4
 
 10322
 
 island
 
 0.546290
 
 0.8447644
 
 Not queried
 
  2.7537453
 
 2.86e-02
 
 0.1830867741
  
 
  
 7
 
   1748302
 
   1748766
 
  PDF   STATS   ENSEMBL   UCSC 
 
  465
 
  9
 
 37405
 
 tss
 
 0.359380
 
 0.7241534
 
 Not queried
 
  2.7524927
 
 1.88e-02
 
 0.1830867741
  
 
  
 3
 
 185911519
 
 185911885
 
  PDF   STATS   ENSEMBL   UCSC 
 
  367
 
  4
 
 28072
 
 gene;island
 
 0.034889
 
 0.3041790
 
 Not queried
 
  2.7430728
 
 2.86e-02
 
 0.1830867741
  
 
  
 15
 
  51973591
 
  51973920
 
  PDF   STATS   ENSEMBL   UCSC 
 
  330
 
  4
 
 13350
 
 gene
 
 0.409269
 
 0.0912680
 
 Not queried
 
  2.7417839
 
 2.86e-02
 
 0.1830867741
  
 
  
 17
 
  46667587
 
  46667812
 
  PDF   STATS   ENSEMBL   UCSC 
 
  226
 
  6
 
 17462
 
 tss;island
 
 0.012609
 
 0.0743953
 
 Not queried
 
  2.7293462
 
 1.52e-02
 
 0.1830867741
  
 
  
 11
 
  30038615
 
  30038797
 
  PDF   STATS   ENSEMBL   UCSC 
 
  183
 
  9
 
 7218
 
 tss;island
 
 0.116341
 
 0.4439297
 
 Not queried
 
  2.7274564
 
 1.88e-02
 
 0.1830867741
  
 
  
 1
 
 110210265
 
 110211023
 
  PDF   STATS   ENSEMBL   UCSC 
 
  759
 
 10
 
 3569
 
 island
 
 0.016292
 
 0.1647880
 
 Not queried
 
  2.7191006
 
 1.47e-02
 
 0.1830867741
  
 
  
 13
 
  88324432
 
  88324879
 
  PDF   STATS   ENSEMBL   UCSC 
 
  448
 
  5
 
 11809
 
 island
 
 0.022028
 
 0.1153146
 
 Not queried
 
  2.7092651
 
 1.59e-02
 
 0.1830867741
  
 
  
 1
 
  78354053
 
  78354406
 
  PDF   STATS   ENSEMBL   UCSC 
 
  354
 
  4
 
 3400
 
 island
 
 0.120070
 
 0.4958310
 
 Not queried
 
  2.7019465
 
 2.86e-02
 
 0.1830867741
  
 
  
 7
 
  42267269
 
  42267747
 
  PDF   STATS   ENSEMBL   UCSC 
 
  479
 
  7
 
 36976
 
 gene;island
 
 0.007495
 
 0.0583376
 
 Not queried
 
  2.6977225
 
 1.75e-02
 
 0.1830867741
  
 
  
 17
 
  72442928
 
  72443179
 
  PDF   STATS   ENSEMBL   UCSC 
 
  252
 
  5
 
 16758
 
 gene;island
 
 0.129639
 
 0.4876485
 
 Not queried
 
  2.6811077
 
 1.59e-02
 
 0.1830867741
  
 
  
 6
 
 167263025
 
 167263454
 
  PDF   STATS   ENSEMBL   UCSC 
 
  430
 
  6
 
 34555
 
 gene
 
 0.834707
 
 0.3913949
 
 Not queried
 
  2.6760943
 
 2.60e-02
 
 0.1830867741
  
 
  
 16
 
   8768488
 
   8768700
 
  PDF   STATS   ENSEMBL   UCSC 
 
  213
 
  4
 
 14651
 
 gene
 
 0.036473
 
 0.0082157
 
 Not queried
 
  2.6697511
 
 2.86e-02
 
 0.1830867741
  
 
  
 12
 
 110271439
 
 110271625
 
  PDF   STATS   ENSEMBL   UCSC 
 
  187
 
  4
 
 9996
 
 tss;island
 
 0.470323
 
 0.1238520
 
 Not queried
 
  2.6596537
 
 2.86e-02
 
 0.1830867741
  
 
  
 8
 
 105479058
 
 105479420
 
  PDF   STATS   ENSEMBL   UCSC 
 
  363
 
  7
 
 40524
 
 island
 
 0.024733
 
 0.4072998
 
 Not queried
 
  2.6524904
 
 2.62e-02
 
 0.1830867741
  
 
  
 4
 
 175135048
 
 175135997
 
  PDF   STATS   ENSEMBL   UCSC 
 
  950
 
  9
 
 31432
 
 island
 
 0.020970
 
 0.2021104
 
 Not queried
 
  2.6422687
 
 1.88e-02
 
 0.1830867741
  
 
  
 10
 
  50339928
 
  50340173
 
  PDF   STATS   ENSEMBL   UCSC 
 
  246
 
  4
 
 4582
 
 gene;island
 
 0.639948
 
 0.9081891
 
 Not queried
 
  2.6214300
 
 2.86e-02
 
 0.1830867741
  
 
  
 6
 
  33160847
 
  33161589
 
  PDF   STATS   ENSEMBL   UCSC 
 
  743
 
 22
 
 34890
 
 tss;island
 
 0.093490
 
 0.4242105
 
 Not queried
 
  2.6143188
 
 1.41e-02
 
 0.1830867741
  
 
  
 19
 
  49699879
 
  49700021
 
  PDF   STATS   ENSEMBL   UCSC 
 
  143
 
  4
 
 20147
 
 gene;island
 
 0.565025
 
 0.8611668
 
 Not queried
 
  2.6132956
 
 2.86e-02
 
 0.1830867741
  
 
  
 19
 
  15122135
 
  15122224
 
  PDF   STATS   ENSEMBL   UCSC 
 
   90
 
  4
 
 19898
 
 gene;island
 
 0.026975
 
 0.1113256
 
 Not queried
 
  2.5510477
 
 2.86e-02
 
 0.1830867741
  
 
  
 22
 
  44287876
 
  44288155
 
  PDF   STATS   ENSEMBL   UCSC 
 
  280
 
  6
 
 27206
 
 tss
 
 0.019935
 
 0.2105981
 
 Not queried
 
  2.5499477
 
 2.60e-02
 
 0.1830867741
  
 
  
 13
 
  58204028
 
  58204379
 
  PDF   STATS   ENSEMBL   UCSC 
 
  352
 
  7
 
 11765
 
 island
 
 0.052198
 
 0.3784814
 
 Not queried
 
  2.5286038
 
 1.75e-02
 
 0.1830867741
  
 
  
 6
 
 112375186
 
 112375333
 
  PDF   STATS   ENSEMBL   UCSC 
 
  148
 
  6
 
 35180
 
 tss
 
 0.283487
 
 0.6562395
 
 Not queried
 
  2.4735838
 
 1.52e-02
 
 0.1830867741
  
 
  
 2
 
  54682758
 
  54683048
 
  PDF   STATS   ENSEMBL   UCSC 
 
  291
 
  4
 
 23356
 
 tss;island
 
 0.017815
 
 0.1039624
 
 Not queried
 
  2.4676727
 
 2.86e-02
 
 0.1830867741
  
 
  
 17
 
  78388520
 
  78388593
 
  PDF   STATS   ENSEMBL   UCSC 
 
   74
 
  4
 
 16867
 
 gene;tss;island
 
 0.043786
 
 0.1509505
 
 Not queried
 
  2.4620136
 
 2.86e-02
 
 0.1830867741
  
 
  
 16
 
  56224504
 
  56225059
 
  PDF   STATS   ENSEMBL   UCSC 
 
  556
 
  6
 
 15242
 
 tss;island
 
 0.029157
 
 0.0056247
 
 Not queried
 
  2.4548705
 
 2.60e-02
 
 0.1830867741
  
 
  
 1
 
  37499309
 
  37499649
 
  PDF   STATS   ENSEMBL   UCSC 
 
  341
 
  4
 
 431
 
 gene;island
 
 0.039568
 
 0.2075014
 
 Not queried
 
  2.4418113
 
 2.86e-02
 
 0.1830867741
  
 
  
 19
 
   4769146
 
   4769240
 
  PDF   STATS   ENSEMBL   UCSC 
 
   95
 
  4
 
 19738
 
 gene
 
 0.028770
 
 0.1599287
 
 Not queried
 
  2.4371490
 
 2.86e-02
 
 0.1830867741
  
 
  
 15
 
  74659901
 
  74660566
 
  PDF   STATS   ENSEMBL   UCSC 
 
  666
 
  7
 
 13792
 
 tss;island
 
 0.439354
 
 0.7610741
 
 Not queried
 
  2.4211461
 
 1.75e-02
 
 0.1830867741
  
 
  
 10
 
 134361889
 
 134362170
 
  PDF   STATS   ENSEMBL   UCSC 
 
  282
 
  6
 
 4865
 
 gene;island
 
 0.720573
 
 0.9379951
 
 Not queried
 
  2.4119516
 
 2.60e-02
 
 0.1830867741
  
 
  
 2
 
  72376056
 
  72376239
 
  PDF   STATS   ENSEMBL   UCSC 
 
  184
 
  4
 
 23432
 
 tss;island
 
 0.003111
 
 0.0111729
 
 Not queried
 
  2.4112603
 
 2.86e-02
 
 0.1830867741
  
 
  
 11
 
  32457124
 
  32457386
 
  PDF   STATS   ENSEMBL   UCSC 
 
  263
 
  7
 
 7226
 
 tss;island
 
 0.090051
 
 0.0133918
 
 Not queried
 
  2.4063735
 
 1.75e-02
 
 0.1830867741
  
 
  
 2
 
  84743142
 
  84743743
 
  PDF   STATS   ENSEMBL   UCSC 
 
  602
 
 12
 
 24469
 
 island
 
 0.065255
 
 0.0184985
 
 Not queried
 
  2.4030055
 
 2.84e-02
 
 0.1830867741
  
 
  
 14
 
  23834710
 
  23834995
 
  PDF   STATS   ENSEMBL   UCSC 
 
  286
 
  8
 
 12718
 
 island
 
 0.011672
 
 0.0480606
 
 Not queried
 
  2.3956527
 
 2.07e-02
 
 0.1830867741
  
 
  
 22
 
  28010359
 
  28010598
 
  PDF   STATS   ENSEMBL   UCSC 
 
  240
 
  4
 
 27380
 
 island
 
 0.114863
 
 0.4472081
 
 Not queried
 
  2.3835339
 
 2.86e-02
 
 0.1830867741
  
 
  
 5
 
  78407418
 
  78407566
 
  PDF   STATS   ENSEMBL   UCSC 
 
  149
 
  6
 
 32145
 
 tss
 
 0.441072
 
 0.7441151
 
 Not queried
 
  2.3813027
 
 1.52e-02
 
 0.1830867741
  
 
  
 12
 
 103889516
 
 103890274
 
  PDF   STATS   ENSEMBL   UCSC 
 
  759
 
 11
 
 10731
 
 island
 
 0.150574
 
 0.0164043
 
 Not queried
 
  2.3687677
 
 2.81e-02
 
 0.1830867741
  
 
  
 2
 
  23850319
 
  23850586
 
  PDF   STATS   ENSEMBL   UCSC 
 
  268
 
  4
 
 22595
 
 gene;island
 
 0.949451
 
 0.7738259
 
 Not queried
 
  2.3645497
 
 2.86e-02
 
 0.1830867741
  
 
  
 1
 
 228246632
 
 228247135
 
  PDF   STATS   ENSEMBL   UCSC 
 
  504
 
  4
 
 1046
 
 gene;island
 
 0.219116
 
 0.0530374
 
 Not queried
 
  2.3128694
 
 2.86e-02
 
 0.1830867741
  
 
  
 2
 
 131046122
 
 131046551
 
  PDF   STATS   ENSEMBL   UCSC 
 
  430
 
  4
 
 24718
 
 island
 
 0.438622
 
 0.7789022
 
 Not queried
 
  2.3030522
 
 2.86e-02
 
 0.1830867741
  
 
  
 19
 
  50370992
 
  50371215
 
  PDF   STATS   ENSEMBL   UCSC 
 
  224
 
  4
 
 20966
 
 tss;island
 
 0.001660
 
 0.0058215
 
 Not queried
 
  2.3023978
 
 2.86e-02
 
 0.1830867741
  
 
  
 10
 
  73479696
 
  73479848
 
  PDF   STATS   ENSEMBL   UCSC 
 
  153
 
  4
 
 5144
 
 tss
 
 0.409236
 
 0.8161270
 
 Not queried
 
  2.2851494
 
 2.86e-02
 
 0.1830867741
  
 
  
 8
 
    599963
 
    600555
 
  PDF   STATS   ENSEMBL   UCSC 
 
  593
 
  6
 
 40003
 
 island
 
 0.753005
 
 0.9664128
 
 Not queried
 
  2.2240516
 
 2.60e-02
 
 0.1830867741
  
 
  
 16
 
    745663
 
    745765
 
  PDF   STATS   ENSEMBL   UCSC 
 
  103
 
  4
 
 14539
 
 gene;island
 
 0.090690
 
 0.0249632
 
 Not queried
 
  2.2009387
 
 2.86e-02
 
 0.1830867741
  
 
  
 13
 
 111642443
 
 111642660
 
  PDF   STATS   ENSEMBL   UCSC 
 
  218
 
  4
 
 11893
 
 island
 
 0.390952
 
 0.7327555
 
 Not queried
 
  2.1915373
 
 2.86e-02
 
 0.1830867741
  
 
  
 15
 
  26108084
 
  26108412
 
  PDF   STATS   ENSEMBL   UCSC 
 
  329
 
  7
 
 13959
 
 island
 
 0.007448
 
 0.0354872
 
 Not queried
 
  2.1739312
 
 1.75e-02
 
 0.1830867741
  
 
  
 8
 
   1094484
 
   1094904
 
  PDF   STATS   ENSEMBL   UCSC 
 
  421
 
  6
 
 40012
 
 island
 
 0.422182
 
 0.0680751
 
 Not queried
 
  2.1715270
 
 1.52e-02
 
 0.1830867741
  
 
  
 11
 
   1282842
 
   1283165
 
  PDF   STATS   ENSEMBL   UCSC 
 
  324
 
  4
 
 6407
 
 gene
 
 0.742977
 
 0.9184042
 
 Not queried
 
  2.1434053
 
 2.86e-02
 
 0.1830867741
  
 
  
 4
 
   2366485
 
   2366831
 
  PDF   STATS   ENSEMBL   UCSC 
 
  347
 
  4
 
 29889
 
 gene;island
 
 0.487525
 
 0.2605101
 
 Not queried
 
  2.1408370
 
 2.86e-02
 
 0.1830867741
  
 
  
 2
 
 172950067
 
 172950626
 
  PDF   STATS   ENSEMBL   UCSC 
 
  560
 
 10
 
 24861
 
 island
 
 0.016256
 
 0.0039084
 
 Not queried
 
  2.1172387
 
 2.32e-02
 
 0.1830867741
  
 
  
 4
 
 188916496
 
 188916875
 
  PDF   STATS   ENSEMBL   UCSC 
 
  380
 
 10
 
 30710
 
 tss
 
 0.304918
 
 0.0596189
 
 Not queried
 
  2.1163565
 
 1.47e-02
 
 0.1830867741
  
 
  
 2
 
 155554688
 
 155555053
 
  PDF   STATS   ENSEMBL   UCSC 
 
  366
 
  6
 
 23671
 
 tss
 
 0.042251
 
 0.1259365
 
 Not queried
 
  2.1103465
 
 2.60e-02
 
 0.1830867741
  
 
  
 8
 
  11560299
 
  11560851
 
  PDF   STATS   ENSEMBL   UCSC 
 
  553
 
 12
 
 39552
 
 tss;island
 
 0.047480
 
 0.2135525
 
 Not queried
 
  2.1051868
 
 2.42e-02
 
 0.1830867741
  
 
  
 5
 
  42951935
 
  42952369
 
  PDF   STATS   ENSEMBL   UCSC 
 
  435
 
  4
 
 32762
 
 island
 
 0.022741
 
 0.0562757
 
 Not queried
 
  2.0997589
 
 2.86e-02
 
 0.1830867741
  
 
  
 1
 
 154377364
 
 154378025
 
  PDF   STATS   ENSEMBL   UCSC 
 
  662
 
 12
 
 3789
 
 island
 
 0.005943
 
 0.0192423
 
 Not queried
 
  2.0892459
 
 1.73e-02
 
 0.1830867741
  
 
  
 2
 
 182521874
 
 182521926
 
  PDF   STATS   ENSEMBL   UCSC 
 
   53
 
  6
 
 23762
 
 tss;island
 
 0.009361
 
 0.0030597
 
 Not queried
 
  2.0863169
 
 2.60e-02
 
 0.1830867741
  
 
  
 6
 
 152127812
 
 152128024
 
  PDF   STATS   ENSEMBL   UCSC 
 
  213
 
  4
 
 34513
 
 gene;tss;island
 
 0.473621
 
 0.6669812
 
 Not queried
 
  2.0786454
 
 2.86e-02
 
 0.1830867741
  
 
  
 1
 
  60280322
 
  60280503
 
  PDF   STATS   ENSEMBL   UCSC 
 
  182
 
  4
 
 1659
 
 tss;island
 
 0.007889
 
 0.0337162
 
 Not queried
 
  2.0647927
 
 2.86e-02
 
 0.1830867741
  
 
  
 1
 
  24645802
 
  24646392
 
  PDF   STATS   ENSEMBL   UCSC 
 
  591
 
 13
 
 2858
 
 island
 
 0.013740
 
 0.0648931
 
 Not queried
 
  2.0618108
 
 1.20e-02
 
 0.1830867741
  
 
  
 6
 
  33180841
 
  33181565
 
  PDF   STATS   ENSEMBL   UCSC 
 
  725
 
 14
 
 35985
 
 island
 
 0.030135
 
 0.0746058
 
 Not queried
 
  2.0586344
 
 2.74e-02
 
 0.1830867741
  
 
  
 6
 
  34524095
 
  34524290
 
  PDF   STATS   ENSEMBL   UCSC 
 
  196
 
  4
 
 34920
 
 tss
 
 0.203285
 
 0.5893199
 
 Not queried
 
  2.0269656
 
 2.86e-02
 
 0.1830867741
  
 
  
 10
 
 101292403
 
 101292766
 
  PDF   STATS   ENSEMBL   UCSC 
 
  364
 
 10
 
 6012
 
 island
 
 0.015943
 
 0.0062590
 
 Not queried
 
  1.9987173
 
 1.85e-02
 
 0.1830867741
  
 
  
 6
 
  29580896
 
  29581228
 
  PDF   STATS   ENSEMBL   UCSC 
 
  333
 
  7
 
 33672
 
 gene
 
 0.189252
 
 0.6283002
 
 Not queried
 
  1.9985628
 
 2.62e-02
 
 0.1830867741
  
 
  
 4
 
 188916496
 
 188916943
 
  PDF   STATS   ENSEMBL   UCSC 
 
  448
 
 11
 
 31491
 
 island
 
 0.330975
 
 0.0666352
 
 Not queried
 
  1.9961977
 
 1.92e-02
 
 0.1830867741
  
 
  
 1
 
  26880767
 
  26881009
 
  PDF   STATS   ENSEMBL   UCSC 
 
  243
 
  4
 
 343
 
 gene;tss
 
 0.837664
 
 0.9603613
 
 Not queried
 
  1.9898809
 
 2.86e-02
 
 0.1830867741
  
 
  
 1
 
   1959442
 
   1959832
 
  PDF   STATS   ENSEMBL   UCSC 
 
  391
 
  6
 
 52
 
 gene;island
 
 0.568534
 
 0.8268992
 
 Not queried
 
  1.9863940
 
 1.52e-02
 
 0.1830867741
  
 
  
 14
 
 102026230
 
 102026555
 
  PDF   STATS   ENSEMBL   UCSC 
 
  326
 
 10
 
 12635
 
 tss
 
 0.039890
 
 0.1202916
 
 Not queried
 
  1.9854852
 
 1.47e-02
 
 0.1830867741
  
 
  
 6
 
  27463112
 
  27463347
 
  PDF   STATS   ENSEMBL   UCSC 
 
  236
 
  4
 
 35558
 
 island
 
 0.015235
 
 0.0404941
 
 Not queried
 
  1.9814794
 
 2.86e-02
 
 0.1830867741
  
 
  
 10
 
  81154142
 
  81154192
 
  PDF   STATS   ENSEMBL   UCSC 
 
   51
 
  4
 
 4652
 
 gene
 
 0.495478
 
 0.7937731
 
 Not queried
 
  1.9804491
 
 2.86e-02
 
 0.1830867741
  
 
  
 11
 
   3187271
 
   3188016
 
  PDF   STATS   ENSEMBL   UCSC 
 
  746
 
 24
 
 7092
 
 tss;island
 
 0.393021
 
 0.7006352
 
 Not queried
 
  1.9784084
 
 2.30e-02
 
 0.1830867741
  
 
  
 2
 
 108602784
 
 108603244
 
  PDF   STATS   ENSEMBL   UCSC 
 
  461
 
  9
 
 24599
 
 island
 
 0.081418
 
 0.4101483
 
 Not queried
 
  1.9639483
 
 1.88e-02
 
 0.1830867741
  
 
  
 19
 
  58864846
 
  58864929
 
  PDF   STATS   ENSEMBL   UCSC 
 
   84
 
  4
 
 20261
 
 gene;island
 
 0.769321
 
 0.9280852
 
 Not queried
 
  1.9280770
 
 2.86e-02
 
 0.1830867741
  
 
  
 6
 
    710835
 
    711405
 
  PDF   STATS   ENSEMBL   UCSC 
 
  571
 
  4
 
 35364
 
 island
 
 0.007138
 
 0.0028602
 
 Not queried
 
  1.9236772
 
 2.86e-02
 
 0.1830867741
  
 
  
 11
 
  57267101
 
  57267244
 
  PDF   STATS   ENSEMBL   UCSC 
 
  144
 
  4
 
 6614
 
 gene;island
 
 0.605447
 
 0.3352171
 
 Not queried
 
  1.9176189
 
 2.86e-02
 
 0.1830867741
  
 
  
 3
 
 132756721
 
 132757106
 
  PDF   STATS   ENSEMBL   UCSC 
 
  386
 
  9
 
 28599
 
 tss
 
 0.046028
 
 0.1339320
 
 Not queried
 
  1.9099959
 
 1.42e-02
 
 0.1830867741
  
 
  
 16
 
   2198777
 
   2199120
 
  PDF   STATS   ENSEMBL   UCSC 
 
  344
 
  4
 
 14601
 
 gene;island
 
 0.018793
 
 0.0731259
 
 Not queried
 
  1.9017796
 
 2.86e-02
 
 0.1830867741
  
 
  
 5
 
 149493008
 
 149493258
 
  PDF   STATS   ENSEMBL   UCSC 
 
  251
 
  6
 
 32424
 
 tss
 
 0.336724
 
 0.6299059
 
 Not queried
 
  1.8979918
 
 2.60e-02
 
 0.1830867741
  
 
  
 3
 
 187387555
 
 187388281
 
  PDF   STATS   ENSEMBL   UCSC 
 
  727
 
 10
 
 29759
 
 island
 
 0.116895
 
 0.4303736
 
 Not queried
 
  1.8833305
 
 1.85e-02
 
 0.1830867741
  
 
  
 6
 
  33757040
 
  33757077
 
  PDF   STATS   ENSEMBL   UCSC 
 
   38
 
  4
 
 34913
 
 tss
 
 0.006579
 
 0.0041303
 
 Not queried
 
  1.8589179
 
 2.86e-02
 
 0.1830867741
  
 
  
 10
 
  75457622
 
  75457686
 
  PDF   STATS   ENSEMBL   UCSC 
 
   65
 
  4
 
 5161
 
 tss
 
 0.855820
 
 0.9535250
 
 Not queried
 
  1.8586933
 
 2.86e-02
 
 0.1830867741
  
 
  
 11
 
  98891492
 
  98891820
 
  PDF   STATS   ENSEMBL   UCSC 
 
  329
 
  9
 
 7640
 
 tss;island
 
 0.011347
 
 0.1185692
 
 Not queried
 
  1.8536320
 
 1.42e-02
 
 0.1830867741
  
 
  
 14
 
  24803679
 
  24804342
 
  PDF   STATS   ENSEMBL   UCSC 
 
  664
 
 10
 
 12746
 
 island
 
 0.019944
 
 0.0923914
 
 Not queried
 
  1.8533722
 
 1.15e-02
 
 0.1830867741
  
 
  
 5
 
  76939333
 
  76939558
 
  PDF   STATS   ENSEMBL   UCSC 
 
  226
 
  4
 
 32904
 
 island
 
 0.032823
 
 0.1096047
 
 Not queried
 
  1.8467214
 
 2.86e-02
 
 0.1830867741
  
 
  
 17
 
  75283800
 
  75283979
 
  PDF   STATS   ENSEMBL   UCSC 
 
  180
 
  4
 
 16803
 
 gene
 
 0.648650
 
 0.8778721
 
 Not queried
 
  1.8244759
 
 2.86e-02
 
 0.1830867741
  
 
  
 5
 
 134871462
 
 134872315
 
  PDF   STATS   ENSEMBL   UCSC 
 
  854
 
 21
 
 33138
 
 island
 
 0.014745
 
 0.0377073
 
 Not queried
 
  1.7957668
 
 2.75e-02
 
 0.1830867741
  
 
  
 1
 
 151810887
 
 151810904
 
  PDF   STATS   ENSEMBL   UCSC 
 
   18
 
  4
 
 755
 
 gene;tss;island
 
 0.011861
 
 0.0478482
 
 Not queried
 
  1.7943369
 
 2.86e-02
 
 0.1830867741
  
 
  
 15
 
  65648103
 
  65648490
 
  PDF   STATS   ENSEMBL   UCSC 
 
  388
 
  4
 
 13379
 
 gene;island
 
 0.476456
 
 0.1789580
 
 Not queried
 
  1.7933043
 
 2.86e-02
 
 0.1830867741
  
 
  
 7
 
 120968877
 
 120969174
 
  PDF   STATS   ENSEMBL   UCSC 
 
  298
 
  8
 
 37203
 
 gene;island
 
 0.067727
 
 0.3832460
 
 Not queried
 
  1.7909987
 
 2.07e-02
 
 0.1830867741
  
 
  
 11
 
   2019079
 
   2019167
 
  PDF   STATS   ENSEMBL   UCSC 
 
   89
 
  5
 
 7054
 
 tss;island
 
 0.331107
 
 0.6547609
 
 Not queried
 
  1.7830111
 
 1.59e-02
 
 0.1830867741
  
 
  
 19
 
  45873596
 
  45873826
 
  PDF   STATS   ENSEMBL   UCSC 
 
  231
 
  4
 
 20093
 
 gene
 
 0.003621
 
 0.0023449
 
 Not queried
 
  1.7819760
 
 2.86e-02
 
 0.1830867741
  
 
  
 17
 
  72427538
 
  72427693
 
  PDF   STATS   ENSEMBL   UCSC 
 
  156
 
  6
 
 18718
 
 island
 
 0.026987
 
 0.1010059
 
 Not queried
 
  1.7815198
 
 2.60e-02
 
 0.1830867741
  
 
  
 2
 
 139537745
 
 139537845
 
  PDF   STATS   ENSEMBL   UCSC 
 
  101
 
  4
 
 24764
 
 island
 
 0.034089
 
 0.0799965
 
 Not queried
 
  1.7770684
 
 2.86e-02
 
 0.1830867741
  
 
  
 6
 
  44310228
 
  44310350
 
  PDF   STATS   ENSEMBL   UCSC 
 
  123
 
  6
 
 35013
 
 tss
 
 0.029705
 
 0.1438887
 
 Not queried
 
  1.7593942
 
 2.60e-02
 
 0.1830867741
  
 
  
 2
 
 172949281
 
 172949501
 
  PDF   STATS   ENSEMBL   UCSC 
 
  221
 
  4
 
 23716
 
 tss;island
 
 0.177888
 
 0.0403964
 
 Not queried
 
  1.7502867
 
 2.86e-02
 
 0.1830867741
  
 
  
 19
 
  54372324
 
  54373043
 
  PDF   STATS   ENSEMBL   UCSC 
 
  720
 
 13
 
 20209
 
 gene;island
 
 0.009573
 
 0.0305724
 
 Not queried
 
  1.7440319
 
 1.64e-02
 
 0.1830867741
  
 
  
 7
 
 150413564
 
 150413801
 
  PDF   STATS   ENSEMBL   UCSC 
 
  238
 
  4
 
 39012
 
 island
 
 0.015400
 
 0.0482461
 
 Not queried
 
  1.7283200
 
 2.86e-02
 
 0.1830867741
  
 
  
 6
 
 105584551
 
 105584780
 
  PDF   STATS   ENSEMBL   UCSC 
 
  230
 
  5
 
 35145
 
 tss;island
 
 0.122871
 
 0.0221338
 
 Not queried
 
  1.7260671
 
 1.59e-02
 
 0.1830867741
  
 
  
 11
 
   1331124
 
   1331497
 
  PDF   STATS   ENSEMBL   UCSC 
 
  374
 
  4
 
 6417
 
 gene;tss;island
 
 0.017254
 
 0.0385344
 
 Not queried
 
  1.7219765
 
 2.86e-02
 
 0.1830867741
  
 
  
 8
 
  11986813
 
  11986933
 
  PDF   STATS   ENSEMBL   UCSC 
 
  121
 
  4
 
 39209
 
 gene;tss
 
 0.596724
 
 0.8192435
 
 Not queried
 
  1.7187480
 
 2.86e-02
 
 0.1830867741
  
 
  
 5
 
    497397
 
    497639
 
  PDF   STATS   ENSEMBL   UCSC 
 
  243
 
  4
 
 31508
 
 gene;island
 
 0.268979
 
 0.4297636
 
 Not queried
 
  1.7136187
 
 2.86e-02
 
 0.1830867741
  
 
  
 8
 
   1814745
 
   1815153
 
  PDF   STATS   ENSEMBL   UCSC 
 
  409
 
  6
 
 39180
 
 gene
 
 0.474644
 
 0.6980425
 
 Not queried
 
  1.7015301
 
 2.60e-02
 
 0.1830867741
  
 
  
 7
 
   1248693
 
   1249199
 
  PDF   STATS   ENSEMBL   UCSC 
 
  507
 
  7
 
 38088
 
 island
 
 0.487127
 
 0.7738135
 
 Not queried
 
  1.6979708
 
 1.75e-02
 
 0.1830867741
  
 
  
 19
 
  58459097
 
  58459358
 
  PDF   STATS   ENSEMBL   UCSC 
 
  262
 
  8
 
 21130
 
 tss
 
 0.023160
 
 0.0070086
 
 Not queried
 
  1.6937202
 
 2.81e-02
 
 0.1830867741
  
 
  
 13
 
 113637695
 
 113637903
 
  PDF   STATS   ENSEMBL   UCSC 
 
  209
 
  4
 
 11264
 
 gene;island
 
 0.403644
 
 0.7215916
 
 Not queried
 
  1.6921137
 
 2.86e-02
 
 0.1830867741
  
 
  
 7
 
 128784416
 
 128784656
 
  PDF   STATS   ENSEMBL   UCSC 
 
  241
 
  6
 
 37890
 
 tss
 
 0.009356
 
 0.0248105
 
 Not queried
 
  1.6880117
 
 2.60e-02
 
 0.1830867741
  
 
  
 5
 
 140220686
 
 140220822
 
  PDF   STATS   ENSEMBL   UCSC 
 
  137
 
  4
 
 32354
 
 tss
 
 0.814154
 
 0.6634196
 
 Not queried
 
  1.6745301
 
 2.86e-02
 
 0.1830867741
  
 
  
 6
 
  29588894
 
  29589056
 
  PDF   STATS   ENSEMBL   UCSC 
 
  163
 
  4
 
 33674
 
 gene
 
 0.295838
 
 0.4586713
 
 Not queried
 
  1.6715444
 
 2.86e-02
 
 0.1830867741
  
 
  
 13
 
  36871646
 
  36872346
 
  PDF   STATS   ENSEMBL   UCSC 
 
  701
 
 14
 
 11668
 
 island
 
 0.071491
 
 0.2733505
 
 Not queried
 
  1.6283993
 
 2.74e-02
 
 0.1830867741
  
 
  
 17
 
   1837588
 
   1837848
 
  PDF   STATS   ENSEMBL   UCSC 
 
  261
 
  6
 
 17834
 
 island
 
 0.556131
 
 0.7298155
 
 Not queried
 
  1.6160510
 
 1.52e-02
 
 0.1830867741
  
 
  
 5
 
 178772203
 
 178772390
 
  PDF   STATS   ENSEMBL   UCSC 
 
  188
 
  4
 
 33483
 
 island
 
 0.090758
 
 0.0255323
 
 Not queried
 
  1.6075509
 
 2.86e-02
 
 0.1830867741
  
 
  
 14
 
  24640947
 
  24641201
 
  PDF   STATS   ENSEMBL   UCSC 
 
  255
 
  9
 
 12318
 
 tss;island
 
 0.035313
 
 0.1387179
 
 Not queried
 
  1.5794548
 
 1.88e-02
 
 0.1830867741
  
 
  
 19
 
  38210543
 
  38210932
 
  PDF   STATS   ENSEMBL   UCSC 
 
  390
 
  6
 
 21961
 
 island
 
 0.026777
 
 0.0107201
 
 Not queried
 
  1.5723132
 
 2.60e-02
 
 0.1830867741
  
 
  
 14
 
 105352845
 
 105353169
 
  PDF   STATS   ENSEMBL   UCSC 
 
  325
 
  4
 
 12253
 
 gene;island
 
 0.441015
 
 0.6553293
 
 Not queried
 
  1.5498550
 
 2.86e-02
 
 0.1830867741
  
 
  
 4
 
 111544271
 
 111544463
 
  PDF   STATS   ENSEMBL   UCSC 
 
  193
 
  7
 
 30549
 
 tss
 
 0.070709
 
 0.0264424
 
 Not queried
 
  1.5375798
 
 1.75e-02
 
 0.1830867741
  
 
  
 4
 
 154710353
 
 154711675
 
  PDF   STATS   ENSEMBL   UCSC 
 
 1323
 
 37
 
 30643
 
 tss
 
 0.020115
 
 0.0733571
 
 Not queried
 
  1.5272168
 
 2.13e-02
 
 0.1830867741
  
 
  
 2
 
 155554688
 
 155555414
 
  PDF   STATS   ENSEMBL   UCSC 
 
  727
 
  9
 
 24797
 
 island
 
 0.026418
 
 0.1034846
 
 Not queried
 
  1.5207507
 
 2.44e-02
 
 0.1830867741
  
 
  
 11
 
  75140541
 
  75140865
 
  PDF   STATS   ENSEMBL   UCSC 
 
  325
 
  4
 
 6836
 
 gene;island
 
 0.895614
 
 0.9587811
 
 Not queried
 
  1.5121562
 
 2.86e-02
 
 0.1830867741
  
 
  
 4
 
  94749865
 
  94750205
 
  PDF   STATS   ENSEMBL   UCSC 
 
  341
 
  7
 
 31186
 
 island
 
 0.066581
 
 0.0209156
 
 Not queried
 
  1.5118214
 
 2.62e-02
 
 0.1830867741
  
 
  
 6
 
  24877462
 
  24877729
 
  PDF   STATS   ENSEMBL   UCSC 
 
  268
 
  4
 
 33594
 
 gene
 
 0.977131
 
 0.9280579
 
 Not queried
 
  1.5025240
 
 2.86e-02
 
 0.1830867741
  
 
  
 13
 
 111281037
 
 111281479
 
  PDF   STATS   ENSEMBL   UCSC 
 
  443
 
  6
 
 11234
 
 gene;island
 
 0.879963
 
 0.9498020
 
 Not queried
 
  1.5009036
 
 1.52e-02
 
 0.1830867741
  
 
  
 19
 
  49944488
 
  49944964
 
  PDF   STATS   ENSEMBL   UCSC 
 
  477
 
  8
 
 22266
 
 island
 
 0.075683
 
 0.0192677
 
 Not queried
 
  1.5003530
 
 2.81e-02
 
 0.1830867741
  
 
  
 19
 
  58458572
 
  58459358
 
  PDF   STATS   ENSEMBL   UCSC 
 
  787
 
 12
 
 22488
 
 island
 
 0.028238
 
 0.0136535
 
 Not queried
 
  1.4966576
 
 1.73e-02
 
 0.1830867741
  
 
  
 1
 
 179711859
 
 179712361
 
  PDF   STATS   ENSEMBL   UCSC 
 
  503
 
 10
 
 4001
 
 island
 
 0.158089
 
 0.0439092
 
 Not queried
 
  1.4908799
 
 2.32e-02
 
 0.1830867741
  
 
  
 7
 
  27282233
 
  27282444
 
  PDF   STATS   ENSEMBL   UCSC 
 
  212
 
  6
 
 36943
 
 gene;island
 
 0.045666
 
 0.0207873
 
 Not queried
 
  1.4667749
 
 1.52e-02
 
 0.1830867741
  
 
  
 22
 
  44287772
 
  44288155
 
  PDF   STATS   ENSEMBL   UCSC 
 
  384
 
  8
 
 27560
 
 island
 
 0.021550
 
 0.1798303
 
 Not queried
 
  1.4604384
 
 1.48e-02
 
 0.1830867741
  
 
  
 19
 
  12997997
 
  12998135
 
  PDF   STATS   ENSEMBL   UCSC 
 
  139
 
  4
 
 21632
 
 island
 
 0.661539
 
 0.8286002
 
 Not queried
 
  1.4517119
 
 2.86e-02
 
 0.1830867741
  
 
  
 3
 
  32859377
 
  32859587
 
  PDF   STATS   ENSEMBL   UCSC 
 
  211
 
  7
 
 28968
 
 island
 
 0.097327
 
 0.0230958
 
 Not queried
 
  1.4467033
 
 1.75e-02
 
 0.1830867741
  
 
  
 4
 
 154710224
 
 154711675
 
  PDF   STATS   ENSEMBL   UCSC 
 
 1452
 
 38
 
 31377
 
 island
 
 0.019601
 
 0.0582636
 
 Not queried
 
  1.4373165
 
 1.85e-02
 
 0.1830867741
  
 
  
 6
 
  42927940
 
  42928497
 
  PDF   STATS   ENSEMBL   UCSC 
 
  558
 
 22
 
 34985
 
 tss
 
 0.063527
 
 0.3176952
 
 Not queried
 
  1.4309402
 
 1.31e-02
 
 0.1830867741
  
 
  
 5
 
  33936171
 
  33936752
 
  PDF   STATS   ENSEMBL   UCSC 
 
  582
 
 13
 
 32723
 
 island
 
 0.209477
 
 0.1119203
 
 Not queried
 
  1.4144177
 
 1.40e-02
 
 0.1830867741
  
 
  
 5
 
 140501321
 
 140501812
 
  PDF   STATS   ENSEMBL   UCSC 
 
  492
 
 10
 
 33211
 
 island
 
 0.144654
 
 0.5006071
 
 Not queried
 
  3.7247491
 
 2.88e-02
 
 0.1834529198
  
 
  
 2
 
 219905959
 
 219906468
 
  PDF   STATS   ENSEMBL   UCSC 
 
  510
 
 10
 
 25103
 
 island
 
 0.008367
 
 0.1113302
 
 Not queried
 
  3.5187332
 
 2.88e-02
 
 0.1834529198
  
 
  
 4
 
  74734714
 
  74735149
 
  PDF   STATS   ENSEMBL   UCSC 
 
  436
 
 10
 
 31097
 
 island
 
 0.018095
 
 0.3467930
 
 Not queried
 
  2.6623896
 
 2.88e-02
 
 0.1834529198
  
 
  
 4
 
 174438930
 
 174440103
 
  PDF   STATS   ENSEMBL   UCSC 
 
 1174
 
 10
 
 31426
 
 island
 
 0.105196
 
 0.3370831
 
 Not queried
 
  2.3972161
 
 2.88e-02
 
 0.1834529198
  
 
  
 15
 
  83477608
 
  83477965
 
  PDF   STATS   ENSEMBL   UCSC 
 
  358
 
  5
 
 13860
 
 tss;island
 
 0.002033
 
 0.4617145
 
 Not queried
 
  7.7627098
 
 3.17e-02
 
 0.1866936846
  
 
  
 18
 
  74499372
 
  74499584
 
  PDF   STATS   ENSEMBL   UCSC 
 
  213
 
  5
 
 19581
 
 island
 
 0.037555
 
 0.5231263
 
 Not queried
 
  6.1944565
 
 3.17e-02
 
 0.1866936846
  
 
  
 7
 
  16505589
 
  16505664
 
  PDF   STATS   ENSEMBL   UCSC 
 
   76
 
  5
 
 37452
 
 tss
 
 0.606578
 
 0.0159037
 
 Not queried
 
  6.0197990
 
 3.17e-02
 
 0.1866936846
  
 
  
 22
 
  39784481
 
  39784982
 
  PDF   STATS   ENSEMBL   UCSC 
 
  502
 
  5
 
 27504
 
 island
 
 0.121670
 
 0.0073780
 
 Not queried
 
  5.9563431
 
 3.17e-02
 
 0.1866936846
  
 
  
 7
 
  38350921
 
  38351226
 
  PDF   STATS   ENSEMBL   UCSC 
 
  306
 
  5
 
 38422
 
 island
 
 0.606805
 
 0.0251554
 
 Not queried
 
  5.9099669
 
 3.17e-02
 
 0.1866936846
  
 
  
 11
 
  68924577
 
  68924956
 
  PDF   STATS   ENSEMBL   UCSC 
 
  380
 
  5
 
 8586
 
 island
 
 0.040708
 
 0.7068889
 
 Not queried
 
  5.8091995
 
 3.17e-02
 
 0.1866936846
  
 
  
 5
 
  75380782
 
  75381070
 
  PDF   STATS   ENSEMBL   UCSC 
 
  289
 
  5
 
 31650
 
 gene;island
 
 0.262406
 
 0.0175304
 
 Not queried
 
  5.5684901
 
 3.17e-02
 
 0.1866936846
  
 
  
 6
 
  47253910
 
  47254169
 
  PDF   STATS   ENSEMBL   UCSC 
 
  260
 
  5
 
 34361
 
 gene
 
 0.155644
 
 0.9598674
 
 Not queried
 
  5.5588021
 
 3.17e-02
 
 0.1866936846
  
 
  
 4
 
  57773149
 
  57773436
 
  PDF   STATS   ENSEMBL   UCSC 
 
  288
 
  5
 
 30412
 
 tss;island
 
 0.043664
 
 0.5136327
 
 Not queried
 
  4.6833387
 
 3.17e-02
 
 0.1866936846
  
 
  
 10
 
  75531971
 
  75531995
 
  PDF   STATS   ENSEMBL   UCSC 
 
   25
 
  5
 
 5163
 
 tss
 
 0.132219
 
 0.0050752
 
 Not queried
 
  4.5891938
 
 3.17e-02
 
 0.1866936846
  
 
  
 2
 
 217560272
 
 217560403
 
  PDF   STATS   ENSEMBL   UCSC 
 
  132
 
  5
 
 23871
 
 tss;island
 
 0.136569
 
 0.0070700
 
 Not queried
 
  4.4758041
 
 3.17e-02
 
 0.1866936846
  
 
  
 5
 
 158531132
 
 158531647
 
  PDF   STATS   ENSEMBL   UCSC 
 
  516
 
  5
 
 33351
 
 island
 
 0.907852
 
 0.4984707
 
 Not queried
 
  4.2890437
 
 3.17e-02
 
 0.1866936846
  
 
  
 4
 
 144621898
 
 144621971
 
  PDF   STATS   ENSEMBL   UCSC 
 
   74
 
  5
 
 30615
 
 tss;island
 
 0.038986
 
 0.2926702
 
 Not queried
 
  4.0629214
 
 3.17e-02
 
 0.1866936846
  
 
  
 8
 
 110986685
 
 110987019
 
  PDF   STATS   ENSEMBL   UCSC 
 
  335
 
  5
 
 40542
 
 island
 
 0.386988
 
 0.0063599
 
 Not queried
 
  3.7861835
 
 3.17e-02
 
 0.1866936846
  
 
  
 8
 
    216453
 
    216788
 
  PDF   STATS   ENSEMBL   UCSC 
 
  336
 
  5
 
 40001
 
 island
 
 0.078027
 
 0.8129365
 
 Not queried
 
  3.6752829
 
 3.17e-02
 
 0.1866936846
  
 
  
 20
 
  25039658
 
  25039812
 
  PDF   STATS   ENSEMBL   UCSC 
 
  155
 
  5
 
 25644
 
 tss;island
 
 0.036501
 
 0.3214981
 
 Not queried
 
  3.6332703
 
 3.17e-02
 
 0.1866936846
  
 
  
 3
 
  42304966
 
  42305237
 
  PDF   STATS   ENSEMBL   UCSC 
 
  272
 
  5
 
 27731
 
 gene;island
 
 0.142586
 
 0.0180864
 
 Not queried
 
  3.5958447
 
 3.17e-02
 
 0.1866936846
  
 
  
 3
 
  49236800
 
  49236918
 
  PDF   STATS   ENSEMBL   UCSC 
 
  119
 
  5
 
 28324
 
 tss
 
 0.749150
 
 0.2325625
 
 Not queried
 
  3.5364244
 
 3.17e-02
 
 0.1866936846
  
 
  
 17
 
  38465281
 
  38465422
 
  PDF   STATS   ENSEMBL   UCSC 
 
  142
 
  5
 
 17329
 
 tss
 
 0.008191
 
 0.3783177
 
 Not queried
 
  3.4787903
 
 3.17e-02
 
 0.1866936846
  
 
  
 12
 
   8332808
 
   8333192
 
  PDF   STATS   ENSEMBL   UCSC 
 
  385
 
  5
 
 9049
 
 gene
 
 0.041202
 
 0.3114807
 
 Not queried
 
  3.3972512
 
 3.17e-02
 
 0.1866936846
  
 
  
 21
 
  40984760
 
  40984886
 
  PDF   STATS   ENSEMBL   UCSC 
 
  127
 
  5
 
 26552
 
 tss
 
 0.069451
 
 0.2562267
 
 Not queried
 
  3.3932587
 
 3.17e-02
 
 0.1866936846
  
 
  
 16
 
   4421445
 
   4421654
 
  PDF   STATS   ENSEMBL   UCSC 
 
  210
 
  5
 
 14638
 
 gene;tss;island
 
 0.160927
 
 0.8951612
 
 Not queried
 
  3.3491157
 
 3.17e-02
 
 0.1866936846
  
 
  
 20
 
  62795341
 
  62795807
 
  PDF   STATS   ENSEMBL   UCSC 
 
  467
 
  5
 
 25915
 
 tss
 
 0.152386
 
 0.6383937
 
 Not queried
 
  3.2969492
 
 3.17e-02
 
 0.1866936846
  
 
  
 6
 
  26103952
 
  26104165
 
  PDF   STATS   ENSEMBL   UCSC 
 
  214
 
  5
 
 34685
 
 tss
 
 0.000660
 
 0.0158735
 
 Not queried
 
  3.2208000
 
 3.17e-02
 
 0.1866936846
  
 
  
 8
 
  19460097
 
  19460243
 
  PDF   STATS   ENSEMBL   UCSC 
 
  147
 
  5
 
 39571
 
 tss
 
 0.525373
 
 0.9112071
 
 Not queried
 
  3.2206669
 
 3.17e-02
 
 0.1866936846
  
 
  
 17
 
  59534637
 
  59534998
 
  PDF   STATS   ENSEMBL   UCSC 
 
  362
 
  5
 
 16720
 
 gene;island
 
 0.043397
 
 0.0056407
 
 Not queried
 
  3.1236873
 
 3.17e-02
 
 0.1866936846
  
 
  
 16
 
  56224504
 
  56224901
 
  PDF   STATS   ENSEMBL   UCSC 
 
  398
 
  5
 
 14755
 
 gene
 
 0.044629
 
 0.0041589
 
 Not queried
 
  2.8796863
 
 3.17e-02
 
 0.1866936846
  
 
  
 7
 
  28996196
 
  28996639
 
  PDF   STATS   ENSEMBL   UCSC 
 
  444
 
  5
 
 36947
 
 gene;island
 
 0.390597
 
 0.0428360
 
 Not queried
 
  2.7736160
 
 3.17e-02
 
 0.1866936846
  
 
  
 1
 
   7913667
 
   7914143
 
  PDF   STATS   ENSEMBL   UCSC 
 
  477
 
  5
 
 1218
 
 tss
 
 0.165310
 
 0.8959520
 
 Not queried
 
  2.7360294
 
 3.17e-02
 
 0.1866936846
  
 
  
 8
 
  80803196
 
  80803526
 
  PDF   STATS   ENSEMBL   UCSC 
 
  331
 
  5
 
 40410
 
 island
 
 0.005640
 
 0.0244856
 
 Not queried
 
  2.6458797
 
 3.17e-02
 
 0.1866936846
  
 
  
 16
 
  68270129
 
  68270387
 
  PDF   STATS   ENSEMBL   UCSC 
 
  259
 
  5
 
 16041
 
 island
 
 0.018694
 
 0.2096431
 
 Not queried
 
  2.5790366
 
 3.17e-02
 
 0.1866936846
  
 
  
 18
 
  21977708
 
  21978221
 
  PDF   STATS   ENSEMBL   UCSC 
 
  514
 
  9
 
 19436
 
 island
 
 0.041679
 
 0.0109703
 
 Not queried
 
  2.5253116
 
 3.15e-02
 
 0.1866936846
  
 
  
 4
 
 187476543
 
 187477065
 
  PDF   STATS   ENSEMBL   UCSC 
 
  523
 
  9
 
 30707
 
 tss
 
 0.071298
 
 0.3663095
 
 Not queried
 
  2.4274628
 
 3.15e-02
 
 0.1866936846
  
 
  
 1
 
  27874258
 
  27874574
 
  PDF   STATS   ENSEMBL   UCSC 
 
  317
 
  5
 
 356
 
 gene;island
 
 0.598947
 
 0.9431139
 
 Not queried
 
  2.4246222
 
 3.17e-02
 
 0.1866936846
  
 
  
 1
 
  19600673
 
  19601069
 
  PDF   STATS   ENSEMBL   UCSC 
 
  397
 
  9
 
 1301
 
 tss;island
 
 0.473330
 
 0.1177154
 
 Not queried
 
  2.3915406
 
 3.15e-02
 
 0.1866936846
  
 
  
 8
 
 145625497
 
 145625814
 
  PDF   STATS   ENSEMBL   UCSC 
 
  318
 
  5
 
 39520
 
 gene;island
 
 0.789853
 
 0.9802872
 
 Not queried
 
  2.3681254
 
 3.17e-02
 
 0.1866936846
  
 
  
 11
 
  20691126
 
  20691429
 
  PDF   STATS   ENSEMBL   UCSC 
 
  304
 
  5
 
 6538
 
 gene
 
 0.006784
 
 0.0337557
 
 Not queried
 
  2.3647400
 
 3.17e-02
 
 0.1866936846
  
 
  
 10
 
 134043618
 
 134043897
 
  PDF   STATS   ENSEMBL   UCSC 
 
  280
 
  5
 
 4858
 
 gene;island
 
 0.021277
 
 0.1727682
 
 Not queried
 
  2.2696759
 
 3.17e-02
 
 0.1866936846
  
 
  
 6
 
  53530538
 
  53530628
 
  PDF   STATS   ENSEMBL   UCSC 
 
   91
 
  5
 
 35041
 
 tss
 
 0.567449
 
 0.8614295
 
 Not queried
 
  2.2124589
 
 3.17e-02
 
 0.1866936846
  
 
  
 6
 
  84562778
 
  84563098
 
  PDF   STATS   ENSEMBL   UCSC 
 
  321
 
  9
 
 36292
 
 island
 
 0.017981
 
 0.1777476
 
 Not queried
 
  2.1402716
 
 3.15e-02
 
 0.1866936846
  
 
  
 7
 
  56515510
 
  56515846
 
  PDF   STATS   ENSEMBL   UCSC 
 
  337
 
  5
 
 37022
 
 gene;island
 
 0.521952
 
 0.2003555
 
 Not queried
 
  2.1242075
 
 3.17e-02
 
 0.1866936846
  
 
  
 10
 
  73479514
 
  73479848
 
  PDF   STATS   ENSEMBL   UCSC 
 
  335
 
  5
 
 4622
 
 gene
 
 0.324592
 
 0.7857140
 
 Not queried
 
  2.1143651
 
 3.17e-02
 
 0.1866936846
  
 
  
 8
 
  41583018
 
  41583523
 
  PDF   STATS   ENSEMBL   UCSC 
 
  506
 
  5
 
 39292
 
 gene;island
 
 0.462623
 
 0.7435698
 
 Not queried
 
  2.0620085
 
 3.17e-02
 
 0.1866936846
  
 
  
 7
 
  93520074
 
  93520566
 
  PDF   STATS   ENSEMBL   UCSC 
 
  493
 
 13
 
 37715
 
 tss
 
 0.076074
 
 0.0150092
 
 Not queried
 
  1.9522330
 
 2.95e-02
 
 0.1866936846
  
 
  
 17
 
    744675
 
    744939
 
  PDF   STATS   ENSEMBL   UCSC 
 
  265
 
  5
 
 16297
 
 gene;island
 
 0.882868
 
 0.9599463
 
 Not queried
 
  1.8775980
 
 3.17e-02
 
 0.1866936846
  
 
  
 1
 
  45792557
 
  45792733
 
  PDF   STATS   ENSEMBL   UCSC 
 
  177
 
  5
 
 501
 
 gene
 
 0.016632
 
 0.0053940
 
 Not queried
 
  1.8619213
 
 3.17e-02
 
 0.1866936846
  
 
  
 4
 
 122686269
 
 122686667
 
  PDF   STATS   ENSEMBL   UCSC 
 
  399
 
  9
 
 30114
 
 gene;island
 
 0.015380
 
 0.0726511
 
 Not queried
 
  1.8572017
 
 3.15e-02
 
 0.1866936846
  
 
  
 11
 
  43596920
 
  43597273
 
  PDF   STATS   ENSEMBL   UCSC 
 
  354
 
  5
 
 8214
 
 island
 
 0.014357
 
 0.0460242
 
 Not queried
 
  1.8523778
 
 3.17e-02
 
 0.1866936846
  
 
  
 10
 
 124907409
 
 124907600
 
  PDF   STATS   ENSEMBL   UCSC 
 
  192
 
  5
 
 5417
 
 tss
 
 0.094004
 
 0.0099539
 
 Not queried
 
  1.8388990
 
 3.17e-02
 
 0.1866936846
  
 
  
 7
 
   2565944
 
   2566263
 
  PDF   STATS   ENSEMBL   UCSC 
 
  320
 
  5
 
 36805
 
 gene;island
 
 0.819087
 
 0.9591462
 
 Not queried
 
  1.8197399
 
 3.17e-02
 
 0.1866936846
  
 
  
 17
 
  19314299
 
  19314408
 
  PDF   STATS   ENSEMBL   UCSC 
 
  110
 
  5
 
 17191
 
 tss
 
 0.422713
 
 0.7339812
 
 Not queried
 
  1.7015502
 
 3.17e-02
 
 0.1866936846
  
 
  
 17
 
  15635376
 
  15635468
 
  PDF   STATS   ENSEMBL   UCSC 
 
   93
 
  5
 
 17151
 
 tss
 
 0.500917
 
 0.7299974
 
 Not queried
 
  1.4274955
 
 3.17e-02
 
 0.1866936846
  
 
  
 15
 
  25414716
 
  25414781
 
  PDF   STATS   ENSEMBL   UCSC 
 
   66
 
  5
 
 13526
 
 tss
 
 0.591736
 
 0.7783913
 
 Not queried
 
  1.4203906
 
 3.17e-02
 
 0.1866936846
  
 
  
 19
 
  58609269
 
  58610131
 
  PDF   STATS   ENSEMBL   UCSC 
 
  863
 
 15
 
 20256
 
 gene;island
 
 0.060726
 
 0.0222134
 
 Not queried
 
  1.4069783
 
 2.95e-02
 
 0.1866936846
  
 
  
 15
 
 100882020
 
 100882647
 
  PDF   STATS   ENSEMBL   UCSC 
 
  628
 
 11
 
 14507
 
 island
 
 0.160128
 
 0.0280966
 
 Not queried
 
  1.8929148
 
 3.36e-02
 
 0.1970167151
  
 
  
 8
 
  61193715
 
  61194292
 
  PDF   STATS   ENSEMBL   UCSC 
 
  578
 
 11
 
 40329
 
 island
 
 0.059467
 
 0.0153849
 
 Not queried
 
  1.6583330
 
 3.36e-02
 
 0.1970167151
  
 
  
 7
 
  95025611
 
  95026248
 
  PDF   STATS   ENSEMBL   UCSC 
 
  638
 
 20
 
 38678
 
 island
 
 0.109497
 
 0.2778207
 
 Not queried
 
  1.5308692
 
 3.50e-02
 
 0.2050200525
  
 
  
 1
 
 118727658
 
 118728226
 
  PDF   STATS   ENSEMBL   UCSC 
 
  569
 
 10
 
 3655
 
 island
 
 0.093216
 
 0.6192023
 
 Not queried
 
  2.9730820
 
 3.55e-02
 
 0.2067926195
  
 
  
 11
 
  44087367
 
  44087867
 
  PDF   STATS   ENSEMBL   UCSC 
 
  501
 
 10
 
 8221
 
 island
 
 0.012367
 
 0.0717464
 
 Not queried
 
  2.6516370
 
 3.55e-02
 
 0.2067926195
  
 
  
 4
 
 110223830
 
 110224288
 
  PDF   STATS   ENSEMBL   UCSC 
 
  459
 
 10
 
 30542
 
 tss
 
 0.042562
 
 0.2290007
 
 Not queried
 
  2.3244041
 
 3.55e-02
 
 0.2067926195
  
 
  
 5
 
  63461216
 
  63461654
 
  PDF   STATS   ENSEMBL   UCSC 
 
  439
 
  8
 
 32088
 
 tss
 
 0.039060
 
 0.3699438
 
 Not queried
 
  4.3713424
 
 3.79e-02
 
 0.2121545571
  
 
  
 12
 
  81331457
 
  81331845
 
  PDF   STATS   ENSEMBL   UCSC 
 
  389
 
  7
 
 10649
 
 island
 
 0.085153
 
 0.0064375
 
 Not queried
 
  4.3621891
 
 3.79e-02
 
 0.2121545571
  
 
  
 1
 
  38941882
 
  38942644
 
  PDF   STATS   ENSEMBL   UCSC 
 
  763
 
  7
 
 3087
 
 island
 
 0.446559
 
 0.0342856
 
 Not queried
 
  4.3343264
 
 3.79e-02
 
 0.2121545571
  
 
  
 19
 
  19648890
 
  19649144
 
  PDF   STATS   ENSEMBL   UCSC 
 
  255
 
  8
 
 21821
 
 island
 
 0.335186
 
 0.0328667
 
 Not queried
 
  4.0942664
 
 3.79e-02
 
 0.2121545571
  
 
  
 15
 
  30114295
 
  30114912
 
  PDF   STATS   ENSEMBL   UCSC 
 
  618
 
  7
 
 13977
 
 island
 
 0.088496
 
 0.0138696
 
 Not queried
 
  3.8124422
 
 3.79e-02
 
 0.2121545571
  
 
  
 13
 
  28562798
 
  28562902
 
  PDF   STATS   ENSEMBL   UCSC 
 
  105
 
  7
 
 11367
 
 tss
 
 0.781919
 
 0.9703374
 
 Not queried
 
  3.5934270
 
 3.79e-02
 
 0.2121545571
  
 
  
 22
 
  23522307
 
  23522511
 
  PDF   STATS   ENSEMBL   UCSC 
 
  205
 
  7
 
 27008
 
 tss
 
 0.578523
 
 0.0270550
 
 Not queried
 
  3.3840053
 
 3.79e-02
 
 0.2121545571
  
 
  
 8
 
  21771059
 
  21771668
 
  PDF   STATS   ENSEMBL   UCSC 
 
  610
 
  7
 
 40119
 
 island
 
 0.003647
 
 0.0185264
 
 Not queried
 
  3.3349237
 
 3.79e-02
 
 0.2121545571
  
 
  
 2
 
 162283434
 
 162284206
 
  PDF   STATS   ENSEMBL   UCSC 
 
  773
 
  8
 
 24823
 
 island
 
 0.120954
 
 0.0208398
 
 Not queried
 
  3.2803591
 
 3.79e-02
 
 0.2121545571
  
 
  
 6
 
 146350041
 
 146350748
 
  PDF   STATS   ENSEMBL   UCSC 
 
  708
 
  8
 
 34500
 
 gene;island
 
 0.030115
 
 0.4550148
 
 Not queried
 
  3.0260831
 
 3.79e-02
 
 0.2121545571
  
 
  
 14
 
  69726197
 
  69726620
 
  PDF   STATS   ENSEMBL   UCSC 
 
  424
 
  8
 
 12479
 
 tss
 
 0.274038
 
 0.0357326
 
 Not queried
 
  2.5843515
 
 3.79e-02
 
 0.2121545571
  
 
  
 6
 
  30458998
 
  30459595
 
  PDF   STATS   ENSEMBL   UCSC 
 
  598
 
  8
 
 33770
 
 gene;island
 
 0.077496
 
 0.5664437
 
 Not queried
 
  2.5593004
 
 3.79e-02
 
 0.2121545571
  
 
  
 6
 
  10886946
 
  10887047
 
  PDF   STATS   ENSEMBL   UCSC 
 
  102
 
  7
 
 34633
 
 tss
 
 0.021164
 
 0.0037025
 
 Not queried
 
  2.5121463
 
 3.79e-02
 
 0.2121545571
  
 
  
 2
 
 223164459
 
 223164925
 
  PDF   STATS   ENSEMBL   UCSC 
 
  467
 
  8
 
 23049
 
 gene;tss;island
 
 0.212004
 
 0.0306191
 
 Not queried
 
  2.4880228
 
 3.79e-02
 
 0.2121545571
  
 
  
 18
 
  21977798
 
  21978221
 
  PDF   STATS   ENSEMBL   UCSC 
 
  424
 
  8
 
 19212
 
 tss
 
 0.069647
 
 0.0181397
 
 Not queried
 
  2.4873957
 
 3.79e-02
 
 0.2121545571
  
 
  
 20
 
  37353032
 
  37353158
 
  PDF   STATS   ENSEMBL   UCSC 
 
  127
 
  7
 
 26159
 
 island
 
 0.047291
 
 0.0187041
 
 Not queried
 
  2.4028185
 
 3.79e-02
 
 0.2121545571
  
 
  
 11
 
  92702373
 
  92702663
 
  PDF   STATS   ENSEMBL   UCSC 
 
  291
 
  7
 
 7617
 
 tss;island
 
 0.210741
 
 0.5556101
 
 Not queried
 
  2.1895436
 
 3.79e-02
 
 0.2121545571
  
 
  
 12
 
 106976448
 
 106976843
 
  PDF   STATS   ENSEMBL   UCSC 
 
  396
 
  7
 
 9970
 
 tss;island
 
 0.016580
 
 0.0508069
 
 Not queried
 
  2.1804975
 
 3.79e-02
 
 0.2121545571
  
 
  
 2
 
 131673771
 
 131674221
 
  PDF   STATS   ENSEMBL   UCSC 
 
  451
 
  7
 
 23631
 
 tss
 
 0.175992
 
 0.2857955
 
 Not queried
 
  2.0937372
 
 3.79e-02
 
 0.2121545571
  
 
  
 19
 
  56988702
 
  56989102
 
  PDF   STATS   ENSEMBL   UCSC 
 
  401
 
  7
 
 22446
 
 island
 
 0.077458
 
 0.2055821
 
 Not queried
 
  2.0687482
 
 3.79e-02
 
 0.2121545571
  
 
  
 7
 
  63505584
 
  63505871
 
  PDF   STATS   ENSEMBL   UCSC 
 
  288
 
  7
 
 38522
 
 island
 
 0.108387
 
 0.3164707
 
 Not queried
 
  1.9308723
 
 3.79e-02
 
 0.2121545571
  
 
  
 2
 
 210636350
 
 210636844
 
  PDF   STATS   ENSEMBL   UCSC 
 
  495
 
  8
 
 25053
 
 island
 
 0.107441
 
 0.0227075
 
 Not queried
 
  1.8231746
 
 3.79e-02
 
 0.2121545571
  
 
  
 10
 
 134901199
 
 134901305
 
  PDF   STATS   ENSEMBL   UCSC 
 
  107
 
  7
 
 5452
 
 tss
 
 0.076554
 
 0.0236159
 
 Not queried
 
  1.7858728
 
 3.79e-02
 
 0.2121545571
  
 
  
 13
 
  98829311
 
  98829666
 
  PDF   STATS   ENSEMBL   UCSC 
 
  356
 
  8
 
 11197
 
 gene;island
 
 0.537625
 
 0.8398140
 
 Not queried
 
  1.7806779
 
 3.79e-02
 
 0.2121545571
  
 
  
 1
 
 179711859
 
 179712281
 
  PDF   STATS   ENSEMBL   UCSC 
 
  423
 
  8
 
 2120
 
 tss
 
 0.158089
 
 0.0439092
 
 Not queried
 
  1.7667161
 
 3.79e-02
 
 0.2121545571
  
 
  
 19
 
  58095424
 
  58095595
 
  PDF   STATS   ENSEMBL   UCSC 
 
  172
 
  7
 
 21120
 
 tss
 
 0.083536
 
 0.0149028
 
 Not queried
 
  1.7621150
 
 3.79e-02
 
 0.2121545571
  
 
  
 5
 
  57878953
 
  57879417
 
  PDF   STATS   ENSEMBL   UCSC 
 
  465
 
  7
 
 31623
 
 gene
 
 0.077395
 
 0.0245619
 
 Not queried
 
  1.7314158
 
 3.79e-02
 
 0.2121545571
  
 
  
 19
 
  37959744
 
  37960107
 
  PDF   STATS   ENSEMBL   UCSC 
 
  364
 
  8
 
 21956
 
 island
 
 0.021560
 
 0.0092172
 
 Not queried
 
  1.6901076
 
 3.79e-02
 
 0.2121545571
  
 
  
 6
 
  30859697
 
  30860529
 
  PDF   STATS   ENSEMBL   UCSC 
 
  833
 
 15
 
 33828
 
 gene
 
 0.955452
 
 0.8242029
 
 Not queried
 
  1.5665200
 
 3.67e-02
 
 0.2121545571
  
 
  
 19
 
  18451112
 
  18451326
 
  PDF   STATS   ENSEMBL   UCSC 
 
  215
 
  8
 
 20617
 
 tss
 
 0.012616
 
 0.0372867
 
 Not queried
 
  1.5053584
 
 3.79e-02
 
 0.2121545571
  
 
  
 22
 
  45704902
 
  45705042
 
  PDF   STATS   ENSEMBL   UCSC 
 
  141
 
  6
 
 27221
 
 tss;island
 
 0.078227
 
 0.9417578
 
 Not queried
 
  6.5866254
 
 4.11e-02
 
 0.2180389943
  
 
  
 10
 
  23216637
 
  23216847
 
  PDF   STATS   ENSEMBL   UCSC 
 
  211
 
  6
 
 4986
 
 tss
 
 0.016638
 
 0.3911388
 
 Not queried
 
  5.9986132
 
 4.11e-02
 
 0.2180389943
  
 
  
 4
 
  46126245
 
  46126448
 
  PDF   STATS   ENSEMBL   UCSC 
 
  204
 
  6
 
 30371
 
 tss
 
 0.139935
 
 0.0056398
 
 Not queried
 
  5.2793811
 
 4.11e-02
 
 0.2180389943
  
 
  
 3
 
  45077649
 
  45078075
 
  PDF   STATS   ENSEMBL   UCSC 
 
  427
 
  6
 
 29046
 
 island
 
 0.006921
 
 0.7977079
 
 Not queried
 
  5.1832864
 
 4.11e-02
 
 0.2180389943
  
 
  
 12
 
   2944286
 
   2944493
 
  PDF   STATS   ENSEMBL   UCSC 
 
  208
 
  6
 
 9491
 
 tss
 
 0.399595
 
 0.9458567
 
 Not queried
 
  5.1113920
 
 4.11e-02
 
 0.2180389943
  
 
  
 2
 
  74780172
 
  74780617
 
  PDF   STATS   ENSEMBL   UCSC 
 
  446
 
  6
 
 22750
 
 gene;island
 
 0.032229
 
 0.5529991
 
 Not queried
 
  4.9808716
 
 4.11e-02
 
 0.2180389943
  
 
  
 16
 
  28995843
 
  28996053
 
  PDF   STATS   ENSEMBL   UCSC 
 
  211
 
  6
 
 15164
 
 tss
 
 0.056829
 
 0.4669797
 
 Not queried
 
  4.2266309
 
 4.11e-02
 
 0.2180389943
  
 
  
 4
 
  25090198
 
  25090665
 
  PDF   STATS   ENSEMBL   UCSC 
 
  468
 
  6
 
 30958
 
 island
 
 0.444469
 
 0.0081244
 
 Not queried
 
  4.0409715
 
 4.11e-02
 
 0.2180389943
  
 
  
 18
 
   5197327
 
   5197559
 
  PDF   STATS   ENSEMBL   UCSC 
 
  233
 
  6
 
 19166
 
 tss;island
 
 0.034881
 
 0.5083288
 
 Not queried
 
  3.7660626
 
 4.11e-02
 
 0.2180389943
  
 
  
 5
 
 135416331
 
 135416613
 
  PDF   STATS   ENSEMBL   UCSC 
 
  283
 
  9
 
 32310
 
 tss
 
 0.921520
 
 0.4695498
 
 Not queried
 
  3.7517347
 
 4.00e-02
 
 0.2180389943
  
 
  
 6
 
  78176473
 
  78176847
 
  PDF   STATS   ENSEMBL   UCSC 
 
  375
 
  6
 
 36270
 
 island
 
 0.203778
 
 0.0244126
 
 Not queried
 
  3.4688289
 
 4.11e-02
 
 0.2180389943
  
 
  
 1
 
 154377364
 
 154377621
 
  PDF   STATS   ENSEMBL   UCSC 
 
  258
 
  6
 
 1963
 
 tss
 
 0.007779
 
 0.1079220
 
 Not queried
 
  3.4255119
 
 4.11e-02
 
 0.2180389943
  
 
  
 5
 
 178422071
 
 178422415
 
  PDF   STATS   ENSEMBL   UCSC 
 
  345
 
  6
 
 33477
 
 island
 
 0.459334
 
 0.0622419
 
 Not queried
 
  3.3290703
 
 4.11e-02
 
 0.2180389943
  
 
  
 8
 
  65291287
 
  65291730
 
  PDF   STATS   ENSEMBL   UCSC 
 
  444
 
  9
 
 40342
 
 island
 
 0.075002
 
 0.3601889
 
 Not queried
 
  3.3193992
 
 4.00e-02
 
 0.2180389943
  
 
  
 7
 
   1022471
 
   1022806
 
  PDF   STATS   ENSEMBL   UCSC 
 
  336
 
  6
 
 37392
 
 tss
 
 0.351482
 
 0.8417946
 
 Not queried
 
  3.1066868
 
 4.11e-02
 
 0.2180389943
  
 
  
 17
 
  29248604
 
  29248954
 
  PDF   STATS   ENSEMBL   UCSC 
 
  351
 
  6
 
 18183
 
 island
 
 0.009756
 
 0.0623391
 
 Not queried
 
  2.8841678
 
 4.11e-02
 
 0.2180389943
  
 
  
 15
 
  93652578
 
  93653073
 
  PDF   STATS   ENSEMBL   UCSC 
 
  496
 
  6
 
 14482
 
 island
 
 0.138207
 
 0.4800835
 
 Not queried
 
  2.8046528
 
 4.11e-02
 
 0.2180389943
  
 
  
 10
 
 118897836
 
 118898082
 
  PDF   STATS   ENSEMBL   UCSC 
 
  247
 
  6
 
 5388
 
 tss;island
 
 0.124845
 
 0.0112025
 
 Not queried
 
  2.7819576
 
 4.11e-02
 
 0.2180389943
  
 
  
 10
 
  23479993
 
  23480316
 
  PDF   STATS   ENSEMBL   UCSC 
 
  324
 
  6
 
 4987
 
 tss;island
 
 0.061272
 
 0.3489761
 
 Not queried
 
  2.7119160
 
 4.11e-02
 
 0.2180389943
  
 
  
 7
 
  27186993
 
  27187691
 
  PDF   STATS   ENSEMBL   UCSC 
 
  699
 
 11
 
 38349
 
 island
 
 0.027389
 
 0.2370978
 
 Not queried
 
  2.6609812
 
 4.00e-02
 
 0.2180389943
  
 
  
 11
 
  94501303
 
  94501718
 
  PDF   STATS   ENSEMBL   UCSC 
 
  416
 
  9
 
 8742
 
 island
 
 0.027385
 
 0.0058885
 
 Not queried
 
  2.6071414
 
 4.00e-02
 
 0.2180389943
  
 
  
 3
 
 147124363
 
 147124827
 
  PDF   STATS   ENSEMBL   UCSC 
 
  465
 
  6
 
 28651
 
 tss;island
 
 0.008399
 
 0.0478803
 
 Not queried
 
  2.5998592
 
 4.11e-02
 
 0.2180389943
  
 
  
 14
 
 101525784
 
 101526140
 
  PDF   STATS   ENSEMBL   UCSC 
 
  357
 
  6
 
 12630
 
 tss
 
 0.199593
 
 0.5623148
 
 Not queried
 
  2.5790060
 
 4.11e-02
 
 0.2180389943
  
 
  
 8
 
  21771252
 
  21771668
 
  PDF   STATS   ENSEMBL   UCSC 
 
  417
 
  6
 
 39577
 
 tss
 
 0.003702
 
 0.0234736
 
 Not queried
 
  2.5491464
 
 4.11e-02
 
 0.2180389943
  
 
  
 11
 
  46366643
 
  46366884
 
  PDF   STATS   ENSEMBL   UCSC 
 
  242
 
  6
 
 7276
 
 tss
 
 0.076800
 
 0.4784348
 
 Not queried
 
  2.5073632
 
 4.11e-02
 
 0.2180389943
  
 
  
 12
 
   8332640
 
   8333192
 
  PDF   STATS   ENSEMBL   UCSC 
 
  553
 
  9
 
 10233
 
 island
 
 0.032281
 
 0.1930658
 
 Not queried
 
  2.4291841
 
 4.00e-02
 
 0.2180389943
  
 
  
 4
 
 154216535
 
 154217214
 
  PDF   STATS   ENSEMBL   UCSC 
 
  680
 
  6
 
 30154
 
 gene;island
 
 0.694154
 
 0.9461747
 
 Not queried
 
  2.2039525
 
 4.11e-02
 
 0.2180389943
  
 
  
 4
 
  57522493
 
  57523055
 
  PDF   STATS   ENSEMBL   UCSC 
 
  563
 
  9
 
 30010
 
 gene;island
 
 0.008356
 
 0.0514101
 
 Not queried
 
  2.1646051
 
 4.00e-02
 
 0.2180389943
  
 
  
 10
 
 128077307
 
 128077376
 
  PDF   STATS   ENSEMBL   UCSC 
 
   70
 
  6
 
 5433
 
 tss;island
 
 0.402423
 
 0.0986186
 
 Not queried
 
  2.1489846
 
 4.11e-02
 
 0.2180389943
  
 
  
 5
 
 140473857
 
 140474578
 
  PDF   STATS   ENSEMBL   UCSC 
 
  722
 
  9
 
 33209
 
 island
 
 0.032266
 
 0.4031879
 
 Not queried
 
  2.1199061
 
 4.00e-02
 
 0.2180389943
  
 
  
 4
 
  94749865
 
  94750061
 
  PDF   STATS   ENSEMBL   UCSC 
 
  197
 
  6
 
 30501
 
 tss
 
 0.062675
 
 0.0182313
 
 Not queried
 
  2.1012230
 
 4.11e-02
 
 0.2180389943
  
 
  
 17
 
    747015
 
    747496
 
  PDF   STATS   ENSEMBL   UCSC 
 
  482
 
  6
 
 16298
 
 gene;island
 
 0.334713
 
 0.6323557
 
 Not queried
 
  2.0707397
 
 4.11e-02
 
 0.2180389943
  
 
  
 22
 
  24823389
 
  24823554
 
  PDF   STATS   ENSEMBL   UCSC 
 
  166
 
  6
 
 27361
 
 island
 
 0.018442
 
 0.0497011
 
 Not queried
 
  2.0343104
 
 4.11e-02
 
 0.2180389943
  
 
  
 12
 
 121416315
 
 121416547
 
  PDF   STATS   ENSEMBL   UCSC 
 
  233
 
  6
 
 10069
 
 tss
 
 0.662103
 
 0.8974876
 
 Not queried
 
  1.8623240
 
 4.11e-02
 
 0.2180389943
  
 
  
 10
 
 105678128
 
 105678266
 
  PDF   STATS   ENSEMBL   UCSC 
 
  139
 
  6
 
 5341
 
 tss
 
 0.002953
 
 0.0052784
 
 Not queried
 
  1.8588141
 
 4.11e-02
 
 0.2180389943
  
 
  
 8
 
  41166990
 
  41167278
 
  PDF   STATS   ENSEMBL   UCSC 
 
  289
 
  6
 
 39667
 
 tss;island
 
 0.475936
 
 0.1491902
 
 Not queried
 
  1.7665453
 
 4.11e-02
 
 0.2180389943
  
 
  
 8
 
  88886051
 
  88886432
 
  PDF   STATS   ENSEMBL   UCSC 
 
  382
 
  9
 
 40440
 
 island
 
 0.800661
 
 0.9569898
 
 Not queried
 
  1.7616779
 
 4.00e-02
 
 0.2180389943
  
 
  
 4
 
 187621790
 
 187622164
 
  PDF   STATS   ENSEMBL   UCSC 
 
  375
 
  6
 
 30218
 
 gene
 
 0.628168
 
 0.8237763
 
 Not queried
 
  1.7353905
 
 4.11e-02
 
 0.2180389943
  
 
  
 7
 
  56516076
 
  56516504
 
  PDF   STATS   ENSEMBL   UCSC 
 
  429
 
  9
 
 37611
 
 tss;island
 
 0.508585
 
 0.2174767
 
 Not queried
 
  1.6948909
 
 4.00e-02
 
 0.2180389943
  
 
  
 6
 
  31783315
 
  31783545
 
  PDF   STATS   ENSEMBL   UCSC 
 
  231
 
 11
 
 33968
 
 gene
 
 0.006629
 
 0.0101010
 
 Not queried
 
  1.6430226
 
 4.00e-02
 
 0.2180389943
  
 
  
 3
 
 111804830
 
 111805077
 
  PDF   STATS   ENSEMBL   UCSC 
 
  248
 
  6
 
 28499
 
 tss
 
 0.078122
 
 0.0212424
 
 Not queried
 
  1.5012270
 
 4.11e-02
 
 0.2180389943
  
 
  
 6
 
  32025886
 
  32026350
 
  PDF   STATS   ENSEMBL   UCSC 
 
  465
 
 10
 
 34022
 
 gene
 
 0.133744
 
 0.6148950
 
 Not queried
 
  2.9389555
 
 4.33e-02
 
 0.2287547702
  
 
  
 14
 
  34420102
 
  34420683
 
  PDF   STATS   ENSEMBL   UCSC 
 
  582
 
 10
 
 12772
 
 island
 
 0.021651
 
 0.0072902
 
 Not queried
 
  2.0191572
 
 4.33e-02
 
 0.2287547702
  
 
  
 21
 
  38362632
 
  38362754
 
  PDF   STATS   ENSEMBL   UCSC 
 
  123
 
  5
 
 26540
 
 tss;island
 
 0.942682
 
 0.0115774
 
 Not queried
 
  9.7561185
 
 5.56e-02
 
 0.2315877671
  
 
  
 1
 
 147012684
 
 147012802
 
  PDF   STATS   ENSEMBL   UCSC 
 
  119
 
  4
 
 1894
 
 tss
 
 0.007150
 
 0.5971200
 
 Not queried
 
  7.8598764
 
 5.71e-02
 
 0.2315877671
  
 
  
 7
 
 156741514
 
 156741808
 
  PDF   STATS   ENSEMBL   UCSC 
 
  295
 
  5
 
 38018
 
 tss;island
 
 0.748953
 
 0.0208939
 
 Not queried
 
  7.1271282
 
 5.56e-02
 
 0.2315877671
  
 
  
 8
 
  58055876
 
  58056175
 
  PDF   STATS   ENSEMBL   UCSC 
 
  300
 
  4
 
 40319
 
 island
 
 0.152164
 
 0.9166491
 
 Not queried
 
  7.0582975
 
 5.71e-02
 
 0.2315877671
  
 
  
 6
 
  32557321
 
  32557478
 
  PDF   STATS   ENSEMBL   UCSC 
 
  158
 
  5
 
 34123
 
 gene
 
 0.012535
 
 0.4154388
 
 Not queried
 
  6.6342056
 
 5.56e-02
 
 0.2315877671
  
 
  
 16
 
  51189801
 
  51190201
 
  PDF   STATS   ENSEMBL   UCSC 
 
  401
 
  5
 
 15921
 
 island
 
 0.321934
 
 0.0051882
 
 Not queried
 
  6.3905562
 
 5.56e-02
 
 0.2315877671
  
 
  
 8
 
  99984350
 
  99984763
 
  PDF   STATS   ENSEMBL   UCSC 
 
  414
 
  4
 
 40489
 
 island
 
 0.170208
 
 0.9603202
 
 Not queried
 
  6.1753446
 
 5.71e-02
 
 0.2315877671
  
 
  
 1
 
 153283628
 
 153283994
 
  PDF   STATS   ENSEMBL   UCSC 
 
  367
 
  4
 
 1940
 
 tss
 
 0.010301
 
 0.2158472
 
 Not queried
 
  5.9404463
 
 5.71e-02
 
 0.2315877671
  
 
  
 1
 
  24152579
 
  24152774
 
  PDF   STATS   ENSEMBL   UCSC 
 
  196
 
  4
 
 1350
 
 tss
 
 0.014213
 
 0.5286260
 
 Not queried
 
  5.9011291
 
 5.71e-02
 
 0.2315877671
  
 
  
 4
 
  86851225
 
  86851425
 
  PDF   STATS   ENSEMBL   UCSC 
 
  201
 
  4
 
 30048
 
 gene
 
 0.003711
 
 0.4119328
 
 Not queried
 
  5.8355275
 
 5.71e-02
 
 0.2315877671
  
 
  
 19
 
  11531294
 
  11531519
 
  PDF   STATS   ENSEMBL   UCSC 
 
  226
 
  5
 
 19840
 
 gene;island
 
 0.017772
 
 0.0795056
 
 Not queried
 
  5.7834921
 
 5.56e-02
 
 0.2315877671
  
 
  
 15
 
  60289239
 
  60289532
 
  PDF   STATS   ENSEMBL   UCSC 
 
  294
 
  4
 
 14187
 
 island
 
 0.266093
 
 0.0215595
 
 Not queried
 
  5.7802927
 
 5.71e-02
 
 0.2315877671
  
 
  
 16
 
   1133074
 
   1133378
 
  PDF   STATS   ENSEMBL   UCSC 
 
  305
 
  5
 
 15515
 
 island
 
 0.063503
 
 0.6648238
 
 Not queried
 
  5.7607478
 
 5.56e-02
 
 0.2315877671
  
 
  
 3
 
 193987426
 
 193987757
 
  PDF   STATS   ENSEMBL   UCSC 
 
  332
 
  4
 
 29785
 
 island
 
 0.022605
 
 0.5100914
 
 Not queried
 
  5.7514182
 
 5.71e-02
 
 0.2315877671
  
 
  
 7
 
 155174991
 
 155175340
 
  PDF   STATS   ENSEMBL   UCSC 
 
  350
 
  4
 
 39065
 
 island
 
 0.486428
 
 0.0273886
 
 Not queried
 
  5.7453728
 
 5.71e-02
 
 0.2315877671
  
 
  
 10
 
  70231928
 
  70232188
 
  PDF   STATS   ENSEMBL   UCSC 
 
  261
 
  5
 
 5113
 
 tss;island
 
 0.018447
 
 0.6309838
 
 Not queried
 
  5.7255724
 
 5.56e-02
 
 0.2315877671
  
 
  
 11
 
  58673453
 
  58673615
 
  PDF   STATS   ENSEMBL   UCSC 
 
  163
 
  4
 
 8298
 
 island
 
 0.854440
 
 0.0518914
 
 Not queried
 
  5.5330039
 
 5.71e-02
 
 0.2315877671
  
 
  
 1
 
 247802703
 
 247803166
 
  PDF   STATS   ENSEMBL   UCSC 
 
  464
 
  7
 
 4420
 
 island
 
 0.025863
 
 0.4915442
 
 Not queried
 
  5.3408507
 
 5.30e-02
 
 0.2315877671
  
 
  
 10
 
  93334974
 
  93335196
 
  PDF   STATS   ENSEMBL   UCSC 
 
  223
 
  4
 
 4672
 
 gene
 
 0.001477
 
 0.0341800
 
 Not queried
 
  5.2986740
 
 5.71e-02
 
 0.2315877671
  
 
  
 13
 
  76334583
 
  76334866
 
  PDF   STATS   ENSEMBL   UCSC 
 
  284
 
  4
 
 11182
 
 gene
 
 0.351713
 
 0.0104228
 
 Not queried
 
  5.2583466
 
 5.71e-02
 
 0.2315877671
  
 
  
 10
 
  49812686
 
  49813128
 
  PDF   STATS   ENSEMBL   UCSC 
 
  443
 
  4
 
 4580
 
 gene
 
 0.056221
 
 0.0168780
 
 Not queried
 
  5.2362773
 
 5.71e-02
 
 0.2315877671
  
 
  
 2
 
 134326055
 
 134326366
 
  PDF   STATS   ENSEMBL   UCSC 
 
  312
 
  5
 
 23641
 
 tss
 
 0.035473
 
 0.7882106
 
 Not queried
 
  5.1974479
 
 5.56e-02
 
 0.2315877671
  
 
  
 1
 
 151812421
 
 151812710
 
  PDF   STATS   ENSEMBL   UCSC 
 
  290
 
  4
 
 756
 
 gene;island
 
 0.036210
 
 0.3632121
 
 Not queried
 
  5.1759986
 
 5.71e-02
 
 0.2315877671
  
 
  
 2
 
 119605210
 
 119605441
 
  PDF   STATS   ENSEMBL   UCSC 
 
  232
 
  4
 
 22842
 
 gene;island
 
 0.384170
 
 0.0174242
 
 Not queried
 
  5.1486410
 
 5.71e-02
 
 0.2315877671
  
 
  
 3
 
 197401858
 
 197402207
 
  PDF   STATS   ENSEMBL   UCSC 
 
  350
 
  5
 
 28105
 
 gene;tss
 
 0.985084
 
 0.5200625
 
 Not queried
 
  5.0832980
 
 5.56e-02
 
 0.2315877671
  
 
  
 4
 
  91156040
 
  91156290
 
  PDF   STATS   ENSEMBL   UCSC 
 
  251
 
  4
 
 30060
 
 gene
 
 0.210539
 
 0.8317414
 
 Not queried
 
  5.0688803
 
 5.71e-02
 
 0.2315877671
  
 
  
 13
 
 100310162
 
 100310405
 
  PDF   STATS   ENSEMBL   UCSC 
 
  244
 
  4
 
 11205
 
 gene;island
 
 0.059154
 
 0.8741912
 
 Not queried
 
  4.9983359
 
 5.71e-02
 
 0.2315877671
  
 
  
 2
 
  74729100
 
  74729710
 
  PDF   STATS   ENSEMBL   UCSC 
 
  611
 
  7
 
 22744
 
 gene;tss;island
 
 0.037372
 
 0.5756058
 
 Not queried
 
  4.9098298
 
 5.30e-02
 
 0.2315877671
  
 
  
 20
 
   2188420
 
   2188663
 
  PDF   STATS   ENSEMBL   UCSC 
 
  244
 
  4
 
 25942
 
 island
 
 0.035850
 
 0.4261043
 
 Not queried
 
  4.8795792
 
 5.71e-02
 
 0.2315877671
  
 
  
 17
 
  19883326
 
  19883716
 
  PDF   STATS   ENSEMBL   UCSC 
 
  391
 
  4
 
 18108
 
 island
 
 0.498896
 
 0.9530463
 
 Not queried
 
  4.8444008
 
 5.71e-02
 
 0.2315877671
  
 
  
 4
 
  83956648
 
  83957095
 
  PDF   STATS   ENSEMBL   UCSC 
 
  448
 
  5
 
 30042
 
 gene
 
 0.192508
 
 0.0029961
 
 Not queried
 
  4.8338540
 
 5.56e-02
 
 0.2315877671
  
 
  
 14
 
 105830509
 
 105830859
 
  PDF   STATS   ENSEMBL   UCSC 
 
  351
 
  5
 
 12262
 
 gene;island
 
 0.545313
 
 0.0343959
 
 Not queried
 
  4.8229004
 
 5.56e-02
 
 0.2315877671
  
 
  
 7
 
 101768610
 
 101768874
 
  PDF   STATS   ENSEMBL   UCSC 
 
  265
 
  4
 
 37161
 
 gene
 
 0.170622
 
 0.7654548
 
 Not queried
 
  4.8169367
 
 5.71e-02
 
 0.2315877671
  
 
  
 16
 
  67428155
 
  67428497
 
  PDF   STATS   ENSEMBL   UCSC 
 
  343
 
  4
 
 15301
 
 tss;island
 
 0.037789
 
 0.3791329
 
 Not queried
 
  4.7765461
 
 5.71e-02
 
 0.2315877671
  
 
  
 17
 
   4486934
 
   4487125
 
  PDF   STATS   ENSEMBL   UCSC 
 
  192
 
  5
 
 17026
 
 tss;island
 
 0.558222
 
 0.9471588
 
 Not queried
 
  4.7293457
 
 5.56e-02
 
 0.2315877671
  
 
  
 6
 
  50817157
 
  50817450
 
  PDF   STATS   ENSEMBL   UCSC 
 
  294
 
  5
 
 36199
 
 island
 
 0.017360
 
 0.2985601
 
 Not queried
 
  4.7221240
 
 5.56e-02
 
 0.2315877671
  
 
  
 8
 
 105479318
 
 105479420
 
  PDF   STATS   ENSEMBL   UCSC 
 
  103
 
  4
 
 39861
 
 tss
 
 0.023804
 
 0.4697303
 
 Not queried
 
  4.6426867
 
 5.71e-02
 
 0.2315877671
  
 
  
 7
 
 158750244
 
 158750607
 
  PDF   STATS   ENSEMBL   UCSC 
 
  364
 
  4
 
 39157
 
 island
 
 0.083961
 
 0.7157174
 
 Not queried
 
  4.6287482
 
 5.71e-02
 
 0.2315877671
  
 
  
 10
 
  56561096
 
  56561213
 
  PDF   STATS   ENSEMBL   UCSC 
 
  118
 
  4
 
 5087
 
 tss
 
 0.613328
 
 0.2016158
 
 Not queried
 
  4.5996885
 
 5.71e-02
 
 0.2315877671
  
 
  
 17
 
   7341436
 
   7341641
 
  PDF   STATS   ENSEMBL   UCSC 
 
  206
 
  4
 
 17083
 
 tss;island
 
 0.133342
 
 0.8110419
 
 Not queried
 
  4.5568202
 
 5.71e-02
 
 0.2315877671
  
 
  
 6
 
  10414350
 
  10414565
 
  PDF   STATS   ENSEMBL   UCSC 
 
  216
 
  4
 
 33559
 
 gene;island
 
 0.011297
 
 0.3656582
 
 Not queried
 
  4.5409227
 
 5.71e-02
 
 0.2315877671
  
 
  
 8
 
  25902400
 
  25902611
 
  PDF   STATS   ENSEMBL   UCSC 
 
  212
 
  5
 
 39608
 
 tss
 
 0.540746
 
 0.0388134
 
 Not queried
 
  4.5342191
 
 5.56e-02
 
 0.2315877671
  
 
  
 1
 
  85514554
 
  85514708
 
  PDF   STATS   ENSEMBL   UCSC 
 
  155
 
  4
 
 1720
 
 tss;island
 
 0.082153
 
 0.0046746
 
 Not queried
 
  4.5250230
 
 5.71e-02
 
 0.2315877671
  
 
  
 6
 
  31237013
 
  31237199
 
  PDF   STATS   ENSEMBL   UCSC 
 
  187
 
  4
 
 33864
 
 gene;island
 
 0.772152
 
 0.2335941
 
 Not queried
 
  4.5204221
 
 5.71e-02
 
 0.2315877671
  
 
  
 12
 
  49942757
 
  49943340
 
  PDF   STATS   ENSEMBL   UCSC 
 
  584
 
  5
 
 9120
 
 gene;island
 
 0.066279
 
 0.3095010
 
 Not queried
 
  4.5048474
 
 5.56e-02
 
 0.2315877671
  
 
  
 6
 
  24358236
 
  24358327
 
  PDF   STATS   ENSEMBL   UCSC 
 
   92
 
  5
 
 33590
 
 gene;island
 
 0.019462
 
 0.3646849
 
 Not queried
 
  4.4701552
 
 5.56e-02
 
 0.2315877671
  
 
  
 12
 
 109728043
 
 109728241
 
  PDF   STATS   ENSEMBL   UCSC 
 
  199
 
  4
 
 9291
 
 gene;island
 
 0.201317
 
 0.6909341
 
 Not queried
 
  4.4504581
 
 5.71e-02
 
 0.2315877671
  
 
  
 20
 
   5891730
 
   5891968
 
  PDF   STATS   ENSEMBL   UCSC 
 
  239
 
  5
 
 25586
 
 tss
 
 0.171598
 
 0.0099436
 
 Not queried
 
  4.4133423
 
 5.56e-02
 
 0.2315877671
  
 
  
 11
 
  18813466
 
  18813556
 
  PDF   STATS   ENSEMBL   UCSC 
 
   91
 
  5
 
 7190
 
 tss;island
 
 0.206647
 
 0.0192811
 
 Not queried
 
  4.3957668
 
 5.56e-02
 
 0.2315877671
  
 
  
 8
 
  11555178
 
  11555548
 
  PDF   STATS   ENSEMBL   UCSC 
 
  371
 
  4
 
 40084
 
 island
 
 0.028992
 
 0.3428891
 
 Not queried
 
  4.3826064
 
 5.71e-02
 
 0.2315877671
  
 
  
 6
 
  75912410
 
  75912711
 
  PDF   STATS   ENSEMBL   UCSC 
 
  302
 
  4
 
 34394
 
 gene;island
 
 0.042643
 
 0.3364853
 
 Not queried
 
  4.3807904
 
 5.71e-02
 
 0.2315877671
  
 
  
 6
 
  36645500
 
  36645886
 
  PDF   STATS   ENSEMBL   UCSC 
 
  387
 
  5
 
 34940
 
 tss;island
 
 0.017759
 
 0.4819231
 
 Not queried
 
  4.3777137
 
 5.56e-02
 
 0.2315877671
  
 
  
 5
 
    501863
 
    502074
 
  PDF   STATS   ENSEMBL   UCSC 
 
  212
 
  4
 
 31510
 
 gene;island
 
 0.045455
 
 0.5151867
 
 Not queried
 
  4.3616946
 
 5.71e-02
 
 0.2315877671
  
 
  
 8
 
  17270347
 
  17270608
 
  PDF   STATS   ENSEMBL   UCSC 
 
  262
 
  4
 
 39216
 
 gene;island
 
 0.055807
 
 0.5020039
 
 Not queried
 
  4.3566120
 
 5.71e-02
 
 0.2315877671
  
 
  
 1
 
  27961746
 
  27962037
 
  PDF   STATS   ENSEMBL   UCSC 
 
  292
 
  5
 
 1416
 
 tss
 
 0.052100
 
 0.5620780
 
 Not queried
 
  4.3013004
 
 5.56e-02
 
 0.2315877671
  
 
  
 2
 
  71558461
 
  71558589
 
  PDF   STATS   ENSEMBL   UCSC 
 
  129
 
  4
 
 23429
 
 tss;island
 
 0.019450
 
 0.5041656
 
 Not queried
 
  4.2396151
 
 5.71e-02
 
 0.2315877671
  
 
  
 3
 
 189348936
 
 189349129
 
  PDF   STATS   ENSEMBL   UCSC 
 
  194
 
  4
 
 28782
 
 tss
 
 0.427152
 
 0.9075960
 
 Not queried
 
  4.2291450
 
 5.71e-02
 
 0.2315877671
  
 
  
 17
 
   9929698
 
   9929867
 
  PDF   STATS   ENSEMBL   UCSC 
 
  170
 
  4
 
 17133
 
 tss
 
 0.040047
 
 0.6140944
 
 Not queried
 
  4.2257335
 
 5.71e-02
 
 0.2315877671
  
 
  
 3
 
 195622522
 
 195622598
 
  PDF   STATS   ENSEMBL   UCSC 
 
   77
 
  4
 
 28096
 
 gene;tss;island
 
 0.014158
 
 0.0028555
 
 Not queried
 
  4.1495068
 
 5.71e-02
 
 0.2315877671
  
 
  
 11
 
  94883242
 
  94883516
 
  PDF   STATS   ENSEMBL   UCSC 
 
  275
 
  4
 
 8746
 
 island
 
 0.183046
 
 0.7557259
 
 Not queried
 
  4.0824864
 
 5.71e-02
 
 0.2315877671
  
 
  
 12
 
 119212160
 
 119212319
 
  PDF   STATS   ENSEMBL   UCSC 
 
  160
 
  4
 
 10871
 
 island
 
 0.007981
 
 0.3596814
 
 Not queried
 
  4.0754872
 
 5.71e-02
 
 0.2315877671
  
 
  
 18
 
  42530692
 
  42531132
 
  PDF   STATS   ENSEMBL   UCSC 
 
  441
 
  8
 
 19096
 
 gene
 
 0.880490
 
 0.5371571
 
 Not queried
 
  4.0164137
 
 4.99e-02
 
 0.2315877671
  
 
  
 7
 
  31726494
 
  31726704
 
  PDF   STATS   ENSEMBL   UCSC 
 
  211
 
  5
 
 37523
 
 tss
 
 0.566325
 
 0.7704594
 
 Not queried
 
  4.0140382
 
 5.56e-02
 
 0.2315877671
  
 
  
 11
 
  20617937
 
  20618399
 
  PDF   STATS   ENSEMBL   UCSC 
 
  463
 
  8
 
 8128
 
 island
 
 0.021672
 
 0.0939667
 
 Not queried
 
  3.9750998
 
 4.99e-02
 
 0.2315877671
  
 
  
 17
 
  40834743
 
  40835003
 
  PDF   STATS   ENSEMBL   UCSC 
 
  261
 
  4
 
 16585
 
 gene;tss;island
 
 0.009290
 
 0.0676510
 
 Not queried
 
  3.9498294
 
 5.71e-02
 
 0.2315877671
  
 
  
 12
 
  54321992
 
  54322396
 
  PDF   STATS   ENSEMBL   UCSC 
 
  405
 
  4
 
 10476
 
 island
 
 0.010275
 
 0.0763197
 
 Not queried
 
  3.8924328
 
 5.71e-02
 
 0.2315877671
  
 
  
 11
 
   1474386
 
   1474841
 
  PDF   STATS   ENSEMBL   UCSC 
 
  456
 
  5
 
 6421
 
 gene;island
 
 0.135048
 
 0.5391844
 
 Not queried
 
  3.8327815
 
 5.56e-02
 
 0.2315877671
  
 
  
 2
 
 223166909
 
 223167617
 
  PDF   STATS   ENSEMBL   UCSC 
 
  709
 
  8
 
 23050
 
 gene;island
 
 0.123163
 
 0.0159047
 
 Not queried
 
  3.8043899
 
 4.99e-02
 
 0.2315877671
  
 
  
 6
 
   5994956
 
   5995150
 
  PDF   STATS   ENSEMBL   UCSC 
 
  195
 
  4
 
 35402
 
 island
 
 0.350249
 
 0.0319115
 
 Not queried
 
  3.7677061
 
 5.71e-02
 
 0.2315877671
  
 
  
 5
 
 176520024
 
 176520502
 
  PDF   STATS   ENSEMBL   UCSC 
 
  479
 
  5
 
 31916
 
 gene;island
 
 0.628298
 
 0.9408619
 
 Not queried
 
  3.7357889
 
 5.56e-02
 
 0.2315877671
  
 
  
 1
 
  85358968
 
  85359134
 
  PDF   STATS   ENSEMBL   UCSC 
 
  167
 
  4
 
 1717
 
 tss;island
 
 0.060814
 
 0.0067531
 
 Not queried
 
  3.7125596
 
 5.71e-02
 
 0.2315877671
  
 
  
 6
 
  30510238
 
  30510459
 
  PDF   STATS   ENSEMBL   UCSC 
 
  222
 
  4
 
 33773
 
 gene
 
 0.011301
 
 0.2836384
 
 Not queried
 
  3.6965499
 
 5.71e-02
 
 0.2315877671
  
 
  
 5
 
  36607333
 
  36607417
 
  PDF   STATS   ENSEMBL   UCSC 
 
   85
 
  4
 
 31587
 
 gene;tss
 
 0.017997
 
 0.1031753
 
 Not queried
 
  3.6890303
 
 5.71e-02
 
 0.2315877671
  
 
  
 11
 
 111169284
 
 111169473
 
  PDF   STATS   ENSEMBL   UCSC 
 
  190
 
  4
 
 6897
 
 gene;tss;island
 
 0.751269
 
 0.9659356
 
 Not queried
 
  3.6862475
 
 5.71e-02
 
 0.2315877671
  
 
  
 2
 
  45237689
 
  45237929
 
  PDF   STATS   ENSEMBL   UCSC 
 
  241
 
  4
 
 23335
 
 tss;island
 
 0.033517
 
 0.3418514
 
 Not queried
 
  3.6543989
 
 5.71e-02
 
 0.2315877671
  
 
  
 2
 
 115419537
 
 115419846
 
  PDF   STATS   ENSEMBL   UCSC 
 
  310
 
  4
 
 22838
 
 gene;island
 
 0.043575
 
 0.4855960
 
 Not queried
 
  3.5993651
 
 5.71e-02
 
 0.2315877671
  
 
  
 2
 
  45159434
 
  45159663
 
  PDF   STATS   ENSEMBL   UCSC 
 
  230
 
  4
 
 24259
 
 island
 
 0.022680
 
 0.3294366
 
 Not queried
 
  3.5729726
 
 5.71e-02
 
 0.2315877671
  
 
  
 6
 
  28477957
 
  28478579
 
  PDF   STATS   ENSEMBL   UCSC 
 
  623
 
  8
 
 33632
 
 gene
 
 0.079288
 
 0.4603745
 
 Not queried
 
  3.5585162
 
 4.99e-02
 
 0.2315877671
  
 
  
 7
 
  20826128
 
  20826345
 
  PDF   STATS   ENSEMBL   UCSC 
 
  218
 
  4
 
 36910
 
 gene;island
 
 0.009959
 
 0.1200229
 
 Not queried
 
  3.5565624
 
 5.71e-02
 
 0.2315877671
  
 
  
 3
 
  55508379
 
  55508686
 
  PDF   STATS   ENSEMBL   UCSC 
 
  308
 
  4
 
 27837
 
 gene;island
 
 0.488037
 
 0.9305234
 
 Not queried
 
  3.5378539
 
 5.71e-02
 
 0.2315877671
  
 
  
 3
 
  65342546
 
  65342971
 
  PDF   STATS   ENSEMBL   UCSC 
 
  426
 
  5
 
 27852
 
 gene;island
 
 0.081258
 
 0.3936234
 
 Not queried
 
  3.5133229
 
 5.56e-02
 
 0.2315877671
  
 
  
 19
 
  18548845
 
  18549274
 
  PDF   STATS   ENSEMBL   UCSC 
 
  430
 
  8
 
 21779
 
 island
 
 0.017662
 
 0.1421337
 
 Not queried
 
  3.4635220
 
 4.99e-02
 
 0.2315877671
  
 
  
 1
 
 165325030
 
 165325231
 
  PDF   STATS   ENSEMBL   UCSC 
 
  202
 
  4
 
 2063
 
 tss
 
 0.110275
 
 0.0171501
 
 Not queried
 
  3.4581891
 
 5.71e-02
 
 0.2315877671
  
 
  
 3
 
  52001778
 
  52002169
 
  PDF   STATS   ENSEMBL   UCSC 
 
  392
 
  4
 
 28368
 
 tss
 
 0.003916
 
 0.0111635
 
 Not queried
 
  3.4427290
 
 5.71e-02
 
 0.2315877671
  
 
  
 6
 
  90597340
 
  90597591
 
  PDF   STATS   ENSEMBL   UCSC 
 
  252
 
  4
 
 36326
 
 island
 
 0.266265
 
 0.5854380
 
 Not queried
 
  3.4081995
 
 5.71e-02
 
 0.2315877671
  
 
  
 19
 
  19383613
 
  19384165
 
  PDF   STATS   ENSEMBL   UCSC 
 
  553
 
  5
 
 21810
 
 island
 
 0.577667
 
 0.0594986
 
 Not queried
 
  3.3856711
 
 5.56e-02
 
 0.2315877671
  
 
  
 5
 
 167719478
 
 167719742
 
  PDF   STATS   ENSEMBL   UCSC 
 
  265
 
  4
 
 31884
 
 gene;island
 
 0.255506
 
 0.0245031
 
 Not queried
 
  3.3698463
 
 5.71e-02
 
 0.2315877671
  
 
  
 2
 
 218898491
 
 218898788
 
  PDF   STATS   ENSEMBL   UCSC 
 
  298
 
  4
 
 25078
 
 island
 
 0.448037
 
 0.9318038
 
 Not queried
 
  3.3660964
 
 5.71e-02
 
 0.2315877671
  
 
  
 16
 
  49732224
 
  49732623
 
  PDF   STATS   ENSEMBL   UCSC 
 
  400
 
  4
 
 14745
 
 gene;island
 
 0.390415
 
 0.0339782
 
 Not queried
 
  3.3545278
 
 5.71e-02
 
 0.2315877671
  
 
  
 5
 
  92929319
 
  92929679
 
  PDF   STATS   ENSEMBL   UCSC 
 
  361
 
  5
 
 31690
 
 gene;island
 
 0.008903
 
 0.4009911
 
 Not queried
 
  3.3527447
 
 5.56e-02
 
 0.2315877671
  
 
  
 22
 
  30901707
 
  30901886
 
  PDF   STATS   ENSEMBL   UCSC 
 
  180
 
  4
 
 27064
 
 tss
 
 0.067262
 
 0.5977188
 
 Not queried
 
  3.2965247
 
 5.71e-02
 
 0.2315877671
  
 
  
 22
 
  29875578
 
  29875991
 
  PDF   STATS   ENSEMBL   UCSC 
 
  414
 
  5
 
 27049
 
 tss;island
 
 0.033919
 
 0.2298651
 
 Not queried
 
  3.2820850
 
 5.56e-02
 
 0.2315877671
  
 
  
 13
 
 113633379
 
 113633600
 
  PDF   STATS   ENSEMBL   UCSC 
 
  222
 
  4
 
 11546
 
 tss
 
 0.024793
 
 0.2773686
 
 Not queried
 
  3.2676371
 
 5.71e-02
 
 0.2315877671
  
 
  
 1
 
 153581494
 
 153581793
 
  PDF   STATS   ENSEMBL   UCSC 
 
  300
 
  5
 
 761
 
 gene
 
 0.428992
 
 0.8743217
 
 Not queried
 
  3.2198546
 
 5.56e-02
 
 0.2315877671
  
 
  
 6
 
  56111812
 
  56112696
 
  PDF   STATS   ENSEMBL   UCSC 
 
  885
 
 13
 
 36219
 
 island
 
 0.020025
 
 0.1330502
 
 Not queried
 
  3.2131731
 
 5.01e-02
 
 0.2315877671
  
 
  
 7
 
 149389669
 
 149389941
 
  PDF   STATS   ENSEMBL   UCSC 
 
  273
 
  4
 
 38999
 
 island
 
 0.629756
 
 0.9147959
 
 Not queried
 
  3.2066865
 
 5.71e-02
 
 0.2315877671
  
 
  
 7
 
 123563992
 
 123564379
 
  PDF   STATS   ENSEMBL   UCSC 
 
  388
 
  4
 
 37872
 
 tss
 
 0.032230
 
 0.3956091
 
 Not queried
 
  3.1978272
 
 5.71e-02
 
 0.2315877671
  
 
  
 1
 
  32281665
 
  32281812
 
  PDF   STATS   ENSEMBL   UCSC 
 
  148
 
  4
 
 1451
 
 tss
 
 0.446511
 
 0.0678296
 
 Not queried
 
  3.1651774
 
 5.71e-02
 
 0.2315877671
  
 
  
 17
 
  73893198
 
  73893209
 
  PDF   STATS   ENSEMBL   UCSC 
 
   12
 
  4
 
 17665
 
 tss
 
 0.012444
 
 0.1373937
 
 Not queried
 
  3.1390968
 
 5.71e-02
 
 0.2315877671
  
 
  
 1
 
  42384310
 
  42384474
 
  PDF   STATS   ENSEMBL   UCSC 
 
  165
 
  4
 
 463
 
 gene
 
 0.781784
 
 0.4284415
 
 Not queried
 
  3.1326305
 
 5.71e-02
 
 0.2315877671
  
 
  
 3
 
 183146663
 
 183146882
 
  PDF   STATS   ENSEMBL   UCSC 
 
  220
 
  4
 
 28746
 
 tss;island
 
 0.007313
 
 0.0801416
 
 Not queried
 
  3.1117824
 
 5.71e-02
 
 0.2315877671
  
 
  
 2
 
 105853494
 
 105853796
 
  PDF   STATS   ENSEMBL   UCSC 
 
  303
 
  4
 
 24583
 
 island
 
 0.881636
 
 0.5757354
 
 Not queried
 
  3.1005452
 
 5.71e-02
 
 0.2315877671
  
 
  
 1
 
    855425
 
    855649
 
  PDF   STATS   ENSEMBL   UCSC 
 
  225
 
  4
 
 1133
 
 tss;island
 
 0.332196
 
 0.7084453
 
 Not queried
 
  3.0897611
 
 5.71e-02
 
 0.2315877671
  
 
  
 16
 
  89163343
 
  89163800
 
  PDF   STATS   ENSEMBL   UCSC 
 
  458
 
  5
 
 14927
 
 gene;island
 
 0.597626
 
 0.9784122
 
 Not queried
 
  3.0740106
 
 5.56e-02
 
 0.2315877671
  
 
  
 6
 
 149772707
 
 149772892
 
  PDF   STATS   ENSEMBL   UCSC 
 
  186
 
  4
 
 34505
 
 gene;island
 
 0.626625
 
 0.9490709
 
 Not queried
 
  3.0437194
 
 5.71e-02
 
 0.2315877671
  
 
  
 19
 
  50666238
 
  50666484
 
  PDF   STATS   ENSEMBL   UCSC 
 
  247
 
  4
 
 20171
 
 gene
 
 0.158800
 
 0.5465316
 
 Not queried
 
  2.9998685
 
 5.71e-02
 
 0.2315877671
  
 
  
 10
 
   1531243
 
   1531529
 
  PDF   STATS   ENSEMBL   UCSC 
 
  287
 
  4
 
 4468
 
 gene
 
 0.227601
 
 0.0275502
 
 Not queried
 
  2.9862682
 
 5.71e-02
 
 0.2315877671
  
 
  
 13
 
  20805380
 
  20805679
 
  PDF   STATS   ENSEMBL   UCSC 
 
  300
 
  8
 
 11330
 
 tss
 
 0.770574
 
 0.3469432
 
 Not queried
 
  2.9643077
 
 4.99e-02
 
 0.2315877671
  
 
  
 16
 
   8806966
 
   8807043
 
  PDF   STATS   ENSEMBL   UCSC 
 
   78
 
  5
 
 14653
 
 gene
 
 0.032738
 
 0.2556769
 
 Not queried
 
  2.9615404
 
 5.56e-02
 
 0.2315877671
  
 
  
 2
 
  89065319
 
  89065605
 
  PDF   STATS   ENSEMBL   UCSC 
 
  287
 
  7
 
 24507
 
 island
 
 0.372448
 
 0.0377819
 
 Not queried
 
  2.9457174
 
 5.30e-02
 
 0.2315877671
  
 
  
 4
 
  81186795
 
  81187198
 
  PDF   STATS   ENSEMBL   UCSC 
 
  404
 
  5
 
 30461
 
 tss;island
 
 0.004693
 
 0.0385084
 
 Not queried
 
  2.9107668
 
 5.56e-02
 
 0.2315877671
  
 
  
 1
 
 146649635
 
 146649851
 
  PDF   STATS   ENSEMBL   UCSC 
 
  217
 
  4
 
 729
 
 gene;island
 
 0.962896
 
 0.6791366
 
 Not queried
 
  2.8922290
 
 5.71e-02
 
 0.2315877671
  
 
  
 3
 
  29322342
 
  29322474
 
  PDF   STATS   ENSEMBL   UCSC 
 
  133
 
  4
 
 28199
 
 tss
 
 0.018897
 
 0.0039822
 
 Not queried
 
  2.8901668
 
 5.71e-02
 
 0.2315877671
  
 
  
 4
 
  62382932
 
  62383240
 
  PDF   STATS   ENSEMBL   UCSC 
 
  309
 
  4
 
 30012
 
 gene;island
 
 0.551287
 
 0.0363811
 
 Not queried
 
  2.8641374
 
 5.71e-02
 
 0.2315877671
  
 
  
 19
 
  49655176
 
  49655517
 
  PDF   STATS   ENSEMBL   UCSC 
 
  342
 
  4
 
 20145
 
 gene;island
 
 0.329051
 
 0.0447399
 
 Not queried
 
  2.8565779
 
 5.71e-02
 
 0.2315877671
  
 
  
 8
 
 117950244
 
 117950454
 
  PDF   STATS   ENSEMBL   UCSC 
 
  211
 
  5
 
 39882
 
 tss
 
 0.362681
 
 0.0836967
 
 Not queried
 
  2.8346704
 
 5.56e-02
 
 0.2315877671
  
 
  
 11
 
    624457
 
    624995
 
  PDF   STATS   ENSEMBL   UCSC 
 
  539
 
  5
 
 6386
 
 gene
 
 0.497702
 
 0.8881315
 
 Not queried
 
  2.8321260
 
 5.56e-02
 
 0.2315877671
  
 
  
 7
 
 106685015
 
 106685033
 
  PDF   STATS   ENSEMBL   UCSC 
 
   19
 
  4
 
 37823
 
 tss;island
 
 0.000958
 
 0.0033418
 
 Not queried
 
  2.8313797
 
 5.71e-02
 
 0.2315877671
  
 
  
 6
 
  10695206
 
  10695389
 
  PDF   STATS   ENSEMBL   UCSC 
 
  184
 
  5
 
 33565
 
 gene
 
 0.012966
 
 0.0029640
 
 Not queried
 
  2.8050905
 
 5.56e-02
 
 0.2315877671
  
 
  
 13
 
  20717346
 
  20717597
 
  PDF   STATS   ENSEMBL   UCSC 
 
  252
 
  4
 
 11093
 
 gene;island
 
 0.714056
 
 0.9342137
 
 Not queried
 
  2.8050598
 
 5.71e-02
 
 0.2315877671
  
 
  
 2
 
 132202485
 
 132202537
 
  PDF   STATS   ENSEMBL   UCSC 
 
   53
 
  5
 
 23633
 
 tss
 
 0.923644
 
 0.6439036
 
 Not queried
 
  2.7411093
 
 5.56e-02
 
 0.2315877671
  
 
  
 1
 
 159174001
 
 159174162
 
  PDF   STATS   ENSEMBL   UCSC 
 
  162
 
  4
 
 2029
 
 tss
 
 0.101874
 
 0.4821425
 
 Not queried
 
  2.7334452
 
 5.71e-02
 
 0.2315877671
  
 
  
 6
 
 101846650
 
 101847058
 
  PDF   STATS   ENSEMBL   UCSC 
 
  409
 
 12
 
 36370
 
 island
 
 0.241133
 
 0.6061345
 
 Not queried
 
  2.7309901
 
 5.19e-02
 
 0.2315877671
  
 
  
 12
 
 120763487
 
 120763869
 
  PDF   STATS   ENSEMBL   UCSC 
 
  383
 
  5
 
 9350
 
 gene
 
 0.010707
 
 0.0435773
 
 Not queried
 
  2.7271262
 
 5.56e-02
 
 0.2315877671
  
 
  
 7
 
 111368367
 
 111368847
 
  PDF   STATS   ENSEMBL   UCSC 
 
  481
 
  4
 
 37188
 
 gene;island
 
 0.978743
 
 0.8440788
 
 Not queried
 
  2.6895038
 
 5.71e-02
 
 0.2315877671
  
 
  
 7
 
  54956202
 
  54956598
 
  PDF   STATS   ENSEMBL   UCSC 
 
  397
 
  5
 
 38501
 
 island
 
 0.016709
 
 0.1706567
 
 Not queried
 
  2.6699083
 
 5.56e-02
 
 0.2315877671
  
 
  
 3
 
 126242864
 
 126243125
 
  PDF   STATS   ENSEMBL   UCSC 
 
  262
 
  7
 
 28561
 
 tss
 
 0.162355
 
 0.0332051
 
 Not queried
 
  2.5836219
 
 5.30e-02
 
 0.2315877671
  
 
  
 2
 
  63276478
 
  63277327
 
  PDF   STATS   ENSEMBL   UCSC 
 
  850
 
 21
 
 23386
 
 tss;island
 
 0.230741
 
 0.0236931
 
 Not queried
 
  2.5748628
 
 4.90e-02
 
 0.2315877671
  
 
  
 12
 
  50344211
 
  50344475
 
  PDF   STATS   ENSEMBL   UCSC 
 
  265
 
  5
 
 9698
 
 tss
 
 0.108656
 
 0.4196181
 
 Not queried
 
  2.5686654
 
 5.56e-02
 
 0.2315877671
  
 
  
 3
 
  30647378
 
  30647988
 
  PDF   STATS   ENSEMBL   UCSC 
 
  611
 
 11
 
 28200
 
 tss
 
 0.009934
 
 0.0583963
 
 Not queried
 
  2.5675854
 
 5.57e-02
 
 0.2315877671
  
 
  
 1
 
 221052350
 
 221052729
 
  PDF   STATS   ENSEMBL   UCSC 
 
  380
 
  7
 
 2264
 
 tss
 
 0.003116
 
 0.0211087
 
 Not queried
 
  2.5429267
 
 5.30e-02
 
 0.2315877671
  
 
  
 6
 
  85474028
 
  85474595
 
  PDF   STATS   ENSEMBL   UCSC 
 
  568
 
 10
 
 35101
 
 tss;island
 
 0.463522
 
 0.0601850
 
 Not queried
 
  2.5391468
 
 5.24e-02
 
 0.2315877671
  
 
  
 13
 
 113633379
 
 113634042
 
  PDF   STATS   ENSEMBL   UCSC 
 
  664
 
  8
 
 11263
 
 gene;island
 
 0.039385
 
 0.4668478
 
 Not queried
 
  2.5320453
 
 4.99e-02
 
 0.2315877671
  
 
  
 17
 
  16256937
 
  16257223
 
  PDF   STATS   ENSEMBL   UCSC 
 
  287
 
  9
 
 17156
 
 tss;island
 
 0.316072
 
 0.0244363
 
 Not queried
 
  2.5245860
 
 5.03e-02
 
 0.2315877671
  
 
  
 6
 
  39759941
 
  39760092
 
  PDF   STATS   ENSEMBL   UCSC 
 
  152
 
  4
 
 34958
 
 tss;island
 
 0.146395
 
 0.0268831
 
 Not queried
 
  2.5016938
 
 5.71e-02
 
 0.2315877671
  
 
  
 11
 
  64115989
 
  64116279
 
  PDF   STATS   ENSEMBL   UCSC 
 
  291
 
  4
 
 6685
 
 gene;island
 
 0.768955
 
 0.9692663
 
 Not queried
 
  2.5011174
 
 5.71e-02
 
 0.2315877671
  
 
  
 16
 
   1146194
 
   1146545
 
  PDF   STATS   ENSEMBL   UCSC 
 
  352
 
  5
 
 15516
 
 island
 
 0.638660
 
 0.8113193
 
 Not queried
 
  2.5002831
 
 5.56e-02
 
 0.2315877671
  
 
  
 2
 
 161127463
 
 161127884
 
  PDF   STATS   ENSEMBL   UCSC 
 
  422
 
  5
 
 24815
 
 island
 
 0.837120
 
 0.9550362
 
 Not queried
 
  2.4906811
 
 5.56e-02
 
 0.2315877671
  
 
  
 7
 
   4850075
 
   4850345
 
  PDF   STATS   ENSEMBL   UCSC 
 
  271
 
  4
 
 36858
 
 gene;island
 
 0.522189
 
 0.8764419
 
 Not queried
 
  2.4878189
 
 5.71e-02
 
 0.2315877671
  
 
  
 4
 
 187629232
 
 187629520
 
  PDF   STATS   ENSEMBL   UCSC 
 
  289
 
  8
 
 30219
 
 gene
 
 0.656204
 
 0.8961757
 
 Not queried
 
  2.4796926
 
 4.99e-02
 
 0.2315877671
  
 
  
 16
 
  88590325
 
  88590467
 
  PDF   STATS   ENSEMBL   UCSC 
 
  143
 
  5
 
 14908
 
 gene;island
 
 0.559529
 
 0.9614388
 
 Not queried
 
  2.4611503
 
 5.56e-02
 
 0.2315877671
  
 
  
 11
 
 108408907
 
 108408981
 
  PDF   STATS   ENSEMBL   UCSC 
 
   75
 
  5
 
 6889
 
 gene
 
 0.982541
 
 0.9188900
 
 Not queried
 
  2.4460345
 
 5.56e-02
 
 0.2315877671
  
 
  
 2
 
 114048829
 
 114049041
 
  PDF   STATS   ENSEMBL   UCSC 
 
  213
 
  5
 
 22835
 
 gene;island
 
 0.067248
 
 0.4391131
 
 Not queried
 
  2.4368570
 
 5.56e-02
 
 0.2315877671
  
 
  
 1
 
 223538174
 
 223538379
 
  PDF   STATS   ENSEMBL   UCSC 
 
  206
 
  4
 
 2271
 
 tss;island
 
 0.040684
 
 0.2244590
 
 Not queried
 
  2.4066865
 
 5.71e-02
 
 0.2315877671
  
 
  
 8
 
   1501458
 
   1501670
 
  PDF   STATS   ENSEMBL   UCSC 
 
  213
 
  4
 
 39168
 
 gene;island
 
 0.543488
 
 0.8934609
 
 Not queried
 
  2.4057557
 
 5.71e-02
 
 0.2315877671
  
 
  
 11
 
  44337982
 
  44338421
 
  PDF   STATS   ENSEMBL   UCSC 
 
  440
 
  5
 
 8226
 
 island
 
 0.323426
 
 0.0418472
 
 Not queried
 
  2.3933368
 
 5.56e-02
 
 0.2315877671
  
 
  
 15
 
  99789622
 
  99789777
 
  PDF   STATS   ENSEMBL   UCSC 
 
  156
 
  4
 
 13502
 
 gene
 
 0.007294
 
 0.0375466
 
 Not queried
 
  2.3528119
 
 5.71e-02
 
 0.2315877671
  
 
  
 12
 
  85306323
 
  85306574
 
  PDF   STATS   ENSEMBL   UCSC 
 
  252
 
  4
 
 9232
 
 gene
 
 0.014529
 
 0.0856980
 
 Not queried
 
  2.3419476
 
 5.71e-02
 
 0.2315877671
  
 
  
 15
 
  33602758
 
  33602824
 
  PDF   STATS   ENSEMBL   UCSC 
 
   67
 
  4
 
 13555
 
 tss;island
 
 0.035994
 
 0.1110814
 
 Not queried
 
  2.3318736
 
 5.71e-02
 
 0.2315877671
  
 
  
 17
 
   7348316
 
   7348490
 
  PDF   STATS   ENSEMBL   UCSC 
 
  175
 
  8
 
 17950
 
 island
 
 0.007997
 
 0.0185285
 
 Not queried
 
  2.3305943
 
 4.99e-02
 
 0.2315877671
  
 
  
 10
 
   1228893
 
   1229080
 
  PDF   STATS   ENSEMBL   UCSC 
 
  188
 
  4
 
 4457
 
 gene;island
 
 0.438950
 
 0.8390931
 
 Not queried
 
  2.3221223
 
 5.71e-02
 
 0.2315877671
  
 
  
 7
 
 156812806
 
 156813602
 
  PDF   STATS   ENSEMBL   UCSC 
 
  797
 
  8
 
 39094
 
 island
 
 0.373166
 
 0.0829976
 
 Not queried
 
  2.3181889
 
 4.99e-02
 
 0.2315877671
  
 
  
 6
 
  29795815
 
  29796124
 
  PDF   STATS   ENSEMBL   UCSC 
 
  310
 
  9
 
 33696
 
 gene;island
 
 0.056361
 
 0.4262899
 
 Not queried
 
  2.3136412
 
 5.03e-02
 
 0.2315877671
  
 
  
 11
 
  32452038
 
  32452839
 
  PDF   STATS   ENSEMBL   UCSC 
 
  802
 
  8
 
 6566
 
 gene;island
 
 0.128850
 
 0.6609106
 
 Not queried
 
  2.2980321
 
 4.99e-02
 
 0.2315877671
  
 
  
 10
 
  15761192
 
  15762312
 
  PDF   STATS   ENSEMBL   UCSC 
 
 1121
 
 12
 
 5571
 
 island
 
 0.054852
 
 0.1961785
 
 Not queried
 
  2.2978572
 
 5.19e-02
 
 0.2315877671
  
 
  
 1
 
 113265410
 
 113265883
 
  PDF   STATS   ENSEMBL   UCSC 
 
  474
 
  7
 
 695
 
 gene;island
 
 0.187455
 
 0.0490725
 
 Not queried
 
  2.2880553
 
 5.30e-02
 
 0.2315877671
  
 
  
 17
 
  13505433
 
  13505799
 
  PDF   STATS   ENSEMBL   UCSC 
 
  367
 
  5
 
 17144
 
 tss;island
 
 0.011624
 
 0.0193536
 
 Not queried
 
  2.2858789
 
 5.56e-02
 
 0.2315877671
  
 
  
 5
 
 132361924
 
 132362235
 
  PDF   STATS   ENSEMBL   UCSC 
 
  312
 
  4
 
 31749
 
 gene
 
 0.007913
 
 0.0022990
 
 Not queried
 
  2.2747794
 
 5.71e-02
 
 0.2315877671
  
 
  
 11
 
  15095017
 
  15095070
 
  PDF   STATS   ENSEMBL   UCSC 
 
   54
 
  5
 
 7168
 
 tss
 
 0.202698
 
 0.0258988
 
 Not queried
 
  2.2495067
 
 5.56e-02
 
 0.2315877671
  
 
  
 7
 
   1967621
 
   1967976
 
  PDF   STATS   ENSEMBL   UCSC 
 
  356
 
  5
 
 36773
 
 gene;island
 
 0.926044
 
 0.5498361
 
 Not queried
 
  2.2486007
 
 5.56e-02
 
 0.2315877671
  
 
  
 6
 
 160679974
 
 160680213
 
  PDF   STATS   ENSEMBL   UCSC 
 
  240
 
  8
 
 35327
 
 tss
 
 0.333539
 
 0.6611111
 
 Not queried
 
  2.2436797
 
 4.99e-02
 
 0.2315877671
  
 
  
 17
 
  38375119
 
  38375510
 
  PDF   STATS   ENSEMBL   UCSC 
 
  392
 
  7
 
 17327
 
 tss;island
 
 0.007784
 
 0.0015793
 
 Not queried
 
  2.2256956
 
 5.30e-02
 
 0.2315877671
  
 
  
 19
 
  49926586
 
  49927309
 
  PDF   STATS   ENSEMBL   UCSC 
 
  724
 
  7
 
 22264
 
 island
 
 0.044329
 
 0.1391527
 
 Not queried
 
  2.2172548
 
 5.30e-02
 
 0.2315877671
  
 
  
 3
 
  44626029
 
  44626860
 
  PDF   STATS   ENSEMBL   UCSC 
 
  832
 
 11
 
 29036
 
 island
 
 0.015951
 
 0.0419183
 
 Not queried
 
  2.1780520
 
 4.73e-02
 
 0.2315877671
  
 
  
 16
 
  11350740
 
  11351330
 
  PDF   STATS   ENSEMBL   UCSC 
 
  591
 
 10
 
 15092
 
 tss;island
 
 0.012176
 
 0.0438953
 
 Not queried
 
  2.1773615
 
 5.24e-02
 
 0.2315877671
  
 
  
 15
 
  69323876
 
  69324156
 
  PDF   STATS   ENSEMBL   UCSC 
 
  281
 
  5
 
 13397
 
 gene;island
 
 0.127457
 
 0.0375231
 
 Not queried
 
  2.1769004
 
 5.56e-02
 
 0.2315877671
  
 
  
 1
 
   3157287
 
   3157510
 
  PDF   STATS   ENSEMBL   UCSC 
 
  224
 
  4
 
 111
 
 gene;island
 
 0.614771
 
 0.8318624
 
 Not queried
 
  2.1700672
 
 5.71e-02
 
 0.2315877671
  
 
  
 16
 
   2907517
 
   2907859
 
  PDF   STATS   ENSEMBL   UCSC 
 
  343
 
  4
 
 14616
 
 gene;island
 
 0.524893
 
 0.2270008
 
 Not queried
 
  2.1533895
 
 5.71e-02
 
 0.2315877671
  
 
  
 4
 
  15376072
 
  15376215
 
  PDF   STATS   ENSEMBL   UCSC 
 
  144
 
  8
 
 29958
 
 gene
 
 0.648992
 
 0.9507108
 
 Not queried
 
  2.1462917
 
 4.99e-02
 
 0.2315877671
  
 
  
 4
 
 183061830
 
 183062004
 
  PDF   STATS   ENSEMBL   UCSC 
 
  175
 
  5
 
 30188
 
 gene;island
 
 0.087876
 
 0.5096772
 
 Not queried
 
  2.1445630
 
 5.56e-02
 
 0.2315877671
  
 
  
 14
 
  91977240
 
  91977306
 
  PDF   STATS   ENSEMBL   UCSC 
 
   67
 
  4
 
 12561
 
 tss;island
 
 0.003445
 
 0.0018551
 
 Not queried
 
  2.1379096
 
 5.71e-02
 
 0.2315877671
  
 
  
 13
 
  36705475
 
  36705622
 
  PDF   STATS   ENSEMBL   UCSC 
 
  148
 
  4
 
 11391
 
 tss
 
 0.022629
 
 0.0750983
 
 Not queried
 
  2.1356163
 
 5.71e-02
 
 0.2315877671
  
 
  
 2
 
 237010048
 
 237010430
 
  PDF   STATS   ENSEMBL   UCSC 
 
  383
 
  5
 
 23088
 
 gene;island
 
 0.468082
 
 0.8218771
 
 Not queried
 
  2.1274725
 
 5.56e-02
 
 0.2315877671
  
 
  
 5
 
 134872610
 
 134872659
 
  PDF   STATS   ENSEMBL   UCSC 
 
   50
 
  4
 
 32306
 
 tss;island
 
 0.077866
 
 0.4191282
 
 Not queried
 
  2.1120854
 
 5.71e-02
 
 0.2315877671
  
 
  
 18
 
  77631625
 
  77631756
 
  PDF   STATS   ENSEMBL   UCSC 
 
  132
 
  4
 
 19143
 
 gene;island
 
 0.538380
 
 0.8230429
 
 Not queried
 
  2.1112177
 
 5.71e-02
 
 0.2315877671
  
 
  
 6
 
  32062160
 
  32062554
 
  PDF   STATS   ENSEMBL   UCSC 
 
  395
 
  5
 
 34045
 
 gene;island
 
 0.054270
 
 0.2293675
 
 Not queried
 
  2.0916098
 
 5.56e-02
 
 0.2315877671
  
 
  
 19
 
  47742558
 
  47742961
 
  PDF   STATS   ENSEMBL   UCSC 
 
  404
 
  4
 
 22189
 
 island
 
 0.038870
 
 0.1506262
 
 Not queried
 
  2.0795062
 
 5.71e-02
 
 0.2315877671
  
 
  
 7
 
  55086091
 
  55086288
 
  PDF   STATS   ENSEMBL   UCSC 
 
  198
 
  4
 
 37603
 
 tss;island
 
 0.355390
 
 0.1283766
 
 Not queried
 
  2.0749452
 
 5.71e-02
 
 0.2315877671
  
 
  
 7
 
  75188876
 
  75189413
 
  PDF   STATS   ENSEMBL   UCSC 
 
  538
 
  7
 
 37047
 
 gene;island
 
 0.794281
 
 0.9381548
 
 Not queried
 
  2.0670689
 
 5.30e-02
 
 0.2315877671
  
 
  
 5
 
 140514811
 
 140515207
 
  PDF   STATS   ENSEMBL   UCSC 
 
  397
 
  5
 
 31808
 
 gene
 
 0.056817
 
 0.2486675
 
 Not queried
 
  2.0591573
 
 5.56e-02
 
 0.2315877671
  
 
  
 13
 
  20735139
 
  20735532
 
  PDF   STATS   ENSEMBL   UCSC 
 
  394
 
  8
 
 11569
 
 island
 
 0.005249
 
 0.0018646
 
 Not queried
 
  2.0413170
 
 4.99e-02
 
 0.2315877671
  
 
  
 6
 
  28601269
 
  28601519
 
  PDF   STATS   ENSEMBL   UCSC 
 
  251
 
 14
 
 35592
 
 island
 
 0.813475
 
 0.9510254
 
 Not queried
 
  2.0407511
 
 4.97e-02
 
 0.2315877671
  
 
  
 4
 
   8087178
 
   8087448
 
  PDF   STATS   ENSEMBL   UCSC 
 
  271
 
  4
 
 29946
 
 gene;island
 
 0.145530
 
 0.5275719
 
 Not queried
 
  2.0236559
 
 5.71e-02
 
 0.2315877671
  
 
  
 19
 
  37997675
 
  37998171
 
  PDF   STATS   ENSEMBL   UCSC 
 
  497
 
 11
 
 21957
 
 island
 
 0.014403
 
 0.0023956
 
 Not queried
 
  2.0218198
 
 4.73e-02
 
 0.2315877671
  
 
  
 1
 
  16345293
 
  16345491
 
  PDF   STATS   ENSEMBL   UCSC 
 
  199
 
  4
 
 1278
 
 tss
 
 0.360077
 
 0.6272561
 
 Not queried
 
  1.9958717
 
 5.71e-02
 
 0.2315877671
  
 
  
 2
 
 210636350
 
 210636560
 
  PDF   STATS   ENSEMBL   UCSC 
 
  211
 
  5
 
 23852
 
 tss
 
 0.159382
 
 0.0201491
 
 Not queried
 
  1.9949506
 
 5.56e-02
 
 0.2315877671
  
 
  
 14
 
  77227545
 
  77227645
 
  PDF   STATS   ENSEMBL   UCSC 
 
  101
 
  4
 
 12524
 
 tss;island
 
 0.486226
 
 0.1703028
 
 Not queried
 
  1.9837352
 
 5.71e-02
 
 0.2315877671
  
 
  
 6
 
 117198428
 
 117198743
 
  PDF   STATS   ENSEMBL   UCSC 
 
  316
 
  4
 
 34453
 
 gene
 
 0.121119
 
 0.4602089
 
 Not queried
 
  1.9442479
 
 5.71e-02
 
 0.2315877671
  
 
  
 1
 
 214557246
 
 214557497
 
  PDF   STATS   ENSEMBL   UCSC 
 
  252
 
  5
 
 1006
 
 gene;island
 
 0.496582
 
 0.8339201
 
 Not queried
 
  1.9405316
 
 5.56e-02
 
 0.2315877671
  
 
  
 4
 
   1642054
 
   1642257
 
  PDF   STATS   ENSEMBL   UCSC 
 
  204
 
  4
 
 29873
 
 gene;island
 
 0.578090
 
 0.8345070
 
 Not queried
 
  1.9300558
 
 5.71e-02
 
 0.2315877671
  
 
  
 19
 
   2525231
 
   2525634
 
  PDF   STATS   ENSEMBL   UCSC 
 
  404
 
  5
 
 19690
 
 gene;island
 
 0.839606
 
 0.9608977
 
 Not queried
 
  1.9214993
 
 5.56e-02
 
 0.2315877671
  
 
  
 3
 
 157155811
 
 157156326
 
  PDF   STATS   ENSEMBL   UCSC 
 
  516
 
  4
 
 28008
 
 gene;island
 
 0.168911
 
 0.0284821
 
 Not queried
 
  1.9109978
 
 5.71e-02
 
 0.2315877671
  
 
  
 21
 
  43736736
 
  43737065
 
  PDF   STATS   ENSEMBL   UCSC 
 
  330
 
  4
 
 26566
 
 tss
 
 0.413560
 
 0.7154416
 
 Not queried
 
  1.8920047
 
 5.71e-02
 
 0.2315877671
  
 
  
 6
 
  80657412
 
  80657555
 
  PDF   STATS   ENSEMBL   UCSC 
 
  144
 
  7
 
 35083
 
 tss;island
 
 0.062462
 
 0.0179229
 
 Not queried
 
  1.8845975
 
 5.30e-02
 
 0.2315877671
  
 
  
 8
 
  23563925
 
  23564294
 
  PDF   STATS   ENSEMBL   UCSC 
 
  370
 
  8
 
 39603
 
 tss
 
 0.319307
 
 0.0744078
 
 Not queried
 
  1.8733649
 
 4.99e-02
 
 0.2315877671
  
 
  
 6
 
 170101455
 
 170101549
 
  PDF   STATS   ENSEMBL   UCSC 
 
   95
 
  4
 
 34574
 
 gene;tss;island
 
 0.010777
 
 0.0210551
 
 Not queried
 
  1.8621082
 
 5.71e-02
 
 0.2315877671
  
 
  
 17
 
  66508512
 
  66508605
 
  PDF   STATS   ENSEMBL   UCSC 
 
   94
 
  4
 
 16742
 
 gene;island
 
 0.002398
 
 0.0007576
 
 Not queried
 
  1.8558798
 
 5.71e-02
 
 0.2315877671
  
 
  
 7
 
   2774414
 
   2774654
 
  PDF   STATS   ENSEMBL   UCSC 
 
  241
 
  4
 
 36815
 
 gene;island
 
 0.236782
 
 0.5207650
 
 Not queried
 
  1.8445136
 
 5.71e-02
 
 0.2315877671
  
 
  
 1
 
 181454975
 
 181455181
 
  PDF   STATS   ENSEMBL   UCSC 
 
  207
 
  4
 
 909
 
 gene;island
 
 0.017835
 
 0.0698857
 
 Not queried
 
  1.7961410
 
 5.71e-02
 
 0.2315877671
  
 
  
 1
 
  27683345
 
  27683501
 
  PDF   STATS   ENSEMBL   UCSC 
 
  157
 
  4
 
 352
 
 gene;island
 
 0.916649
 
 0.9655393
 
 Not queried
 
  1.7958451
 
 5.71e-02
 
 0.2315877671
  
 
  
 6
 
  26103952
 
  26104560
 
  PDF   STATS   ENSEMBL   UCSC 
 
  609
 
  9
 
 35514
 
 island
 
 0.002758
 
 0.0158735
 
 Not queried
 
  1.7886086
 
 5.03e-02
 
 0.2315877671
  
 
  
 6
 
  49681299
 
  49681391
 
  PDF   STATS   ENSEMBL   UCSC 
 
   93
 
  5
 
 35027
 
 tss
 
 0.488188
 
 0.2987567
 
 Not queried
 
  1.7341260
 
 5.56e-02
 
 0.2315877671
  
 
  
 17
 
  80708279
 
  80708632
 
  PDF   STATS   ENSEMBL   UCSC 
 
  354
 
  4
 
 16956
 
 gene;island
 
 0.935662
 
 0.9847000
 
 Not queried
 
  1.7069390
 
 5.71e-02
 
 0.2315877671
  
 
  
 3
 
  45838037
 
  45838338
 
  PDF   STATS   ENSEMBL   UCSC 
 
  302
 
  8
 
 28271
 
 tss
 
 0.049079
 
 0.0211950
 
 Not queried
 
  1.6976644
 
 4.99e-02
 
 0.2315877671
  
 
  
 1
 
 111813509
 
 111813852
 
  PDF   STATS   ENSEMBL   UCSC 
 
  344
 
  4
 
 3590
 
 island
 
 0.119226
 
 0.4333552
 
 Not queried
 
  1.6915879
 
 5.71e-02
 
 0.2315877671
  
 
  
 4
 
  96470887
 
  96471143
 
  PDF   STATS   ENSEMBL   UCSC 
 
  257
 
  4
 
 30506
 
 tss;island
 
 0.007298
 
 0.0164219
 
 Not queried
 
  1.6847351
 
 5.71e-02
 
 0.2315877671
  
 
  
 4
 
 187517794
 
 187518154
 
  PDF   STATS   ENSEMBL   UCSC 
 
  361
 
  5
 
 30217
 
 gene
 
 0.739574
 
 0.9490570
 
 Not queried
 
  1.6803294
 
 5.56e-02
 
 0.2315877671
  
 
  
 1
 
   3605747
 
   3606082
 
  PDF   STATS   ENSEMBL   UCSC 
 
  336
 
  5
 
 136
 
 gene;tss;island
 
 0.725669
 
 0.3306354
 
 Not queried
 
  1.6796395
 
 5.56e-02
 
 0.2315877671
  
 
  
 11
 
   1855746
 
   1856307
 
  PDF   STATS   ENSEMBL   UCSC 
 
  562
 
  7
 
 6432
 
 gene
 
 0.170993
 
 0.3397184
 
 Not queried
 
  1.6766301
 
 5.30e-02
 
 0.2315877671
  
 
  
 16
 
    960911
 
    961353
 
  PDF   STATS   ENSEMBL   UCSC 
 
  443
 
  4
 
 14551
 
 gene;island
 
 0.498968
 
 0.8449413
 
 Not queried
 
  1.6751172
 
 5.71e-02
 
 0.2315877671
  
 
  
 14
 
 106938234
 
 106938621
 
  PDF   STATS   ENSEMBL   UCSC 
 
  388
 
 10
 
 13248
 
 island
 
 0.873004
 
 0.9687642
 
 Not queried
 
  1.6527434
 
 5.24e-02
 
 0.2315877671
  
 
  
 1
 
 227746111
 
 227746453
 
  PDF   STATS   ENSEMBL   UCSC 
 
  343
 
  4
 
 4269
 
 island
 
 0.717156
 
 0.8536863
 
 Not queried
 
  1.6458846
 
 5.71e-02
 
 0.2315877671
  
 
  
 6
 
  29626990
 
  29627395
 
  PDF   STATS   ENSEMBL   UCSC 
 
  406
 
  8
 
 33684
 
 gene
 
 0.488594
 
 0.7604528
 
 Not queried
 
  1.6330640
 
 4.99e-02
 
 0.2315877671
  
 
  
 1
 
   2359927
 
   2360074
 
  PDF   STATS   ENSEMBL   UCSC 
 
  148
 
  4
 
 2501
 
 island
 
 0.537643
 
 0.7529460
 
 Not queried
 
  1.6274957
 
 5.71e-02
 
 0.2315877671
  
 
  
 11
 
 130059549
 
 130059791
 
  PDF   STATS   ENSEMBL   UCSC 
 
  243
 
  5
 
 6977
 
 gene;island
 
 0.447162
 
 0.2718843
 
 Not queried
 
  1.6238715
 
 5.56e-02
 
 0.2315877671
  
 
  
 10
 
 135340445
 
 135341025
 
  PDF   STATS   ENSEMBL   UCSC 
 
  581
 
  9
 
 6367
 
 island
 
 0.361049
 
 0.6929544
 
 Not queried
 
  1.6182692
 
 5.03e-02
 
 0.2315877671
  
 
  
 1
 
 155006911
 
 155007323
 
  PDF   STATS   ENSEMBL   UCSC 
 
  413
 
  5
 
 788
 
 gene;tss
 
 0.319533
 
 0.5997103
 
 Not queried
 
  1.6161590
 
 5.56e-02
 
 0.2315877671
  
 
  
 8
 
  12613139
 
  12613305
 
  PDF   STATS   ENSEMBL   UCSC 
 
  167
 
  4
 
 39558
 
 tss;island
 
 0.001694
 
 0.0031260
 
 Not queried
 
  1.6149638
 
 5.71e-02
 
 0.2315877671
  
 
  
 8
 
  22960177
 
  22960619
 
  PDF   STATS   ENSEMBL   UCSC 
 
  443
 
 10
 
 40145
 
 island
 
 0.043281
 
 0.1576534
 
 Not queried
 
  1.6139747
 
 5.24e-02
 
 0.2315877671
  
 
  
 12
 
 110270919
 
 110271031
 
  PDF   STATS   ENSEMBL   UCSC 
 
  113
 
  4
 
 9296
 
 gene;island
 
 0.266165
 
 0.0736258
 
 Not queried
 
  1.6009664
 
 5.71e-02
 
 0.2315877671
  
 
  
 2
 
 220436894
 
 220436948
 
  PDF   STATS   ENSEMBL   UCSC 
 
   55
 
  4
 
 23910
 
 tss;island
 
 0.471329
 
 0.7345231
 
 Not queried
 
  1.6000300
 
 5.71e-02
 
 0.2315877671
  
 
  
 3
 
 126113327
 
 126114064
 
  PDF   STATS   ENSEMBL   UCSC 
 
  738
 
 13
 
 29432
 
 island
 
 0.077978
 
 0.3442094
 
 Not queried
 
  1.5963324
 
 4.41e-02
 
 0.2315877671
  
 
  
 12
 
   7863853
 
   7864076
 
  PDF   STATS   ENSEMBL   UCSC 
 
  224
 
  5
 
 9550
 
 tss
 
 0.479461
 
 0.7691710
 
 Not queried
 
  1.5939585
 
 5.56e-02
 
 0.2315877671
  
 
  
 17
 
  72427538
 
  72427637
 
  PDF   STATS   ENSEMBL   UCSC 
 
  100
 
  5
 
 17626
 
 tss
 
 0.027744
 
 0.1058295
 
 Not queried
 
  1.5752609
 
 5.56e-02
 
 0.2315877671
  
 
  
 17
 
   1810459
 
   1810853
 
  PDF   STATS   ENSEMBL   UCSC 
 
  395
 
  5
 
 17832
 
 island
 
 0.591030
 
 0.9212628
 
 Not queried
 
  1.5741351
 
 5.56e-02
 
 0.2315877671
  
 
  
 8
 
  23563859
 
  23564294
 
  PDF   STATS   ENSEMBL   UCSC 
 
  436
 
  9
 
 40156
 
 island
 
 0.274980
 
 0.0783536
 
 Not queried
 
  1.5708497
 
 5.03e-02
 
 0.2315877671
  
 
  
 12
 
   6881590
 
   6881629
 
  PDF   STATS   ENSEMBL   UCSC 
 
   40
 
  5
 
 9527
 
 tss;island
 
 0.314840
 
 0.4691816
 
 Not queried
 
  1.5668908
 
 5.56e-02
 
 0.2315877671
  
 
  
 5
 
 151066268
 
 151066486
 
  PDF   STATS   ENSEMBL   UCSC 
 
  219
 
  4
 
 31867
 
 gene
 
 0.046169
 
 0.0125364
 
 Not queried
 
  1.5625694
 
 5.71e-02
 
 0.2315877671
  
 
  
 7
 
 150147988
 
 150148073
 
  PDF   STATS   ENSEMBL   UCSC 
 
   86
 
  4
 
 37274
 
 gene
 
 0.026871
 
 0.0792623
 
 Not queried
 
  1.5417999
 
 5.71e-02
 
 0.2315877671
  
 
  
 6
 
 150378738
 
 150379047
 
  PDF   STATS   ENSEMBL   UCSC 
 
  310
 
  5
 
 36566
 
 island
 
 0.449181
 
 0.6711823
 
 Not queried
 
  1.5086546
 
 5.56e-02
 
 0.2315877671
  
 
  
 10
 
 123355268
 
 123355748
 
  PDF   STATS   ENSEMBL   UCSC 
 
  481
 
  4
 
 4794
 
 gene;island
 
 0.429493
 
 0.6189388
 
 Not queried
 
  1.5066415
 
 5.71e-02
 
 0.2315877671
  
 
  
 11
 
  19367501
 
  19368277
 
  PDF   STATS   ENSEMBL   UCSC 
 
  777
 
  8
 
 8120
 
 island
 
 0.006483
 
 0.0167882
 
 Not queried
 
  1.5063105
 
 4.99e-02
 
 0.2315877671
  
 
  
 3
 
  43147571
 
  43148002
 
  PDF   STATS   ENSEMBL   UCSC 
 
  432
 
  8
 
 28251
 
 tss
 
 0.034224
 
 0.0115684
 
 Not queried
 
  1.5017711
 
 4.99e-02
 
 0.2315877671
  
 
  
 1
 
  92951720
 
  92953037
 
  PDF   STATS   ENSEMBL   UCSC 
 
 1318
 
 20
 
 1752
 
 tss
 
 0.011000
 
 0.0058006
 
 Not queried
 
  1.4866599
 
 4.60e-02
 
 0.2315877671
  
 
  
 11
 
   3173868
 
   3174128
 
  PDF   STATS   ENSEMBL   UCSC 
 
  261
 
  5
 
 6475
 
 gene
 
 0.437938
 
 0.6331116
 
 Not queried
 
  1.4559935
 
 5.56e-02
 
 0.2315877671
  
 
  
 4
 
  87515184
 
  87515433
 
  PDF   STATS   ENSEMBL   UCSC 
 
  250
 
  8
 
 30482
 
 tss
 
 0.018867
 
 0.0577481
 
 Not queried
 
  1.4300877
 
 4.99e-02
 
 0.2315877671
  
 
  
 19
 
  36035831
 
  36036175
 
  PDF   STATS   ENSEMBL   UCSC 
 
  345
 
  5
 
 19985
 
 gene
 
 0.028583
 
 0.0101604
 
 Not queried
 
  1.4072375
 
 5.56e-02
 
 0.2315877671
  
 
  
 8
 
  70984199
 
  70984559
 
  PDF   STATS   ENSEMBL   UCSC 
 
  361
 
  4
 
 39758
 
 tss;island
 
 0.118963
 
 0.0465488
 
 Not queried
 
  1.4065018
 
 5.71e-02
 
 0.2315877671
  
 
  
 6
 
  29523592
 
  29524117
 
  PDF   STATS   ENSEMBL   UCSC 
 
  526
 
  8
 
 33660
 
 gene;island
 
 0.431393
 
 0.7039538
 
 Not queried
 
  1.4051896
 
 4.99e-02
 
 0.2315877671
  
 
  
 10
 
 134150451
 
 134150581
 
  PDF   STATS   ENSEMBL   UCSC 
 
  131
 
  6
 
 5448
 
 tss
 
 0.011755
 
 0.1272652
 
 Not queried
 
  5.3012308
 
 6.49e-02
 
 0.2477578846
  
 
  
 5
 
 140578896
 
 140579644
 
  PDF   STATS   ENSEMBL   UCSC 
 
  749
 
  9
 
 33223
 
 island
 
 0.061806
 
 0.5573654
 
 Not queried
 
  5.2194575
 
 6.25e-02
 
 0.2477578846
  
 
  
 17
 
  46622454
 
  46622899
 
  PDF   STATS   ENSEMBL   UCSC 
 
  446
 
  8
 
 17457
 
 tss;island
 
 0.635788
 
 0.9574357
 
 Not queried
 
  4.8326673
 
 6.50e-02
 
 0.2477578846
  
 
  
 6
 
 166876490
 
 166877038
 
  PDF   STATS   ENSEMBL   UCSC 
 
  549
 
  8
 
 34545
 
 gene
 
 0.010924
 
 0.2902401
 
 Not queried
 
  4.8234436
 
 6.50e-02
 
 0.2477578846
  
 
  
 13
 
 112720171
 
 112720565
 
  PDF   STATS   ENSEMBL   UCSC 
 
  395
 
  6
 
 11909
 
 island
 
 0.046706
 
 0.5045828
 
 Not queried
 
  4.7944405
 
 6.49e-02
 
 0.2477578846
  
 
  
 10
 
  89263900
 
  89264128
 
  PDF   STATS   ENSEMBL   UCSC 
 
  229
 
  6
 
 5202
 
 tss;island
 
 0.476123
 
 0.9812681
 
 Not queried
 
  4.5990495
 
 6.49e-02
 
 0.2477578846
  
 
  
 7
 
  31232532
 
  31232861
 
  PDF   STATS   ENSEMBL   UCSC 
 
  330
 
  6
 
 38391
 
 island
 
 0.027387
 
 0.2222159
 
 Not queried
 
  4.5936437
 
 6.49e-02
 
 0.2477578846
  
 
  
 2
 
 241497412
 
 241497846
 
  PDF   STATS   ENSEMBL   UCSC 
 
  435
 
  8
 
 23981
 
 tss;island
 
 0.526381
 
 0.0780420
 
 Not queried
 
  4.0021724
 
 6.50e-02
 
 0.2477578846
  
 
  
 8
 
  86350278
 
  86350592
 
  PDF   STATS   ENSEMBL   UCSC 
 
  315
 
  6
 
 39794
 
 tss;island
 
 0.022782
 
 0.3454082
 
 Not queried
 
  3.8531542
 
 6.49e-02
 
 0.2477578846
  
 
  
 5
 
  76934327
 
  76934696
 
  PDF   STATS   ENSEMBL   UCSC 
 
  370
 
  6
 
 32902
 
 island
 
 0.330684
 
 0.0473785
 
 Not queried
 
  3.8021768
 
 6.49e-02
 
 0.2477578846
  
 
  
 6
 
  31025933
 
  31026287
 
  PDF   STATS   ENSEMBL   UCSC 
 
  355
 
  6
 
 33849
 
 gene
 
 0.076510
 
 0.4839170
 
 Not queried
 
  3.6847892
 
 6.49e-02
 
 0.2477578846
  
 
  
 3
 
 157813327
 
 157813810
 
  PDF   STATS   ENSEMBL   UCSC 
 
  484
 
  6
 
 29616
 
 island
 
 0.483687
 
 0.0332860
 
 Not queried
 
  3.6715270
 
 6.49e-02
 
 0.2477578846
  
 
  
 17
 
  27346732
 
  27347260
 
  PDF   STATS   ENSEMBL   UCSC 
 
  529
 
  6
 
 18164
 
 island
 
 0.011890
 
 0.1769034
 
 Not queried
 
  3.5762625
 
 6.49e-02
 
 0.2477578846
  
 
  
 2
 
  20190016
 
  20190349
 
  PDF   STATS   ENSEMBL   UCSC 
 
  334
 
  9
 
 23207
 
 tss
 
 0.097668
 
 0.0297289
 
 Not queried
 
  3.4734257
 
 6.25e-02
 
 0.2477578846
  
 
  
 17
 
  78161467
 
  78161706
 
  PDF   STATS   ENSEMBL   UCSC 
 
  240
 
  6
 
 16859
 
 gene;island
 
 0.469575
 
 0.8872667
 
 Not queried
 
  3.4556045
 
 6.49e-02
 
 0.2477578846
  
 
  
 13
 
 113776873
 
 113777109
 
  PDF   STATS   ENSEMBL   UCSC 
 
  237
 
  8
 
 11548
 
 tss
 
 0.130587
 
 0.4045890
 
 Not queried
 
  3.3752746
 
 6.50e-02
 
 0.2477578846
  
 
  
 6
 
   6007259
 
   6007769
 
  PDF   STATS   ENSEMBL   UCSC 
 
  511
 
  6
 
 35407
 
 island
 
 0.166015
 
 0.0106798
 
 Not queried
 
  3.2850721
 
 6.49e-02
 
 0.2477578846
  
 
  
 19
 
  43969650
 
  43970034
 
  PDF   STATS   ENSEMBL   UCSC 
 
  385
 
  6
 
 22083
 
 island
 
 0.837238
 
 0.4221695
 
 Not queried
 
  3.2841884
 
 6.49e-02
 
 0.2477578846
  
 
  
 18
 
  31020418
 
  31021065
 
  PDF   STATS   ENSEMBL   UCSC 
 
  648
 
  8
 
 19227
 
 tss;island
 
 0.098612
 
 0.3882054
 
 Not queried
 
  3.2841435
 
 6.50e-02
 
 0.2477578846
  
 
  
 11
 
  94134446
 
  94135029
 
  PDF   STATS   ENSEMBL   UCSC 
 
  584
 
 10
 
 8737
 
 island
 
 0.349225
 
 0.0665760
 
 Not queried
 
  3.2487511
 
 6.30e-02
 
 0.2477578846
  
 
  
 19
 
  56988778
 
  56989102
 
  PDF   STATS   ENSEMBL   UCSC 
 
  325
 
  6
 
 21098
 
 tss
 
 0.067756
 
 0.2394204
 
 Not queried
 
  3.2299611
 
 6.49e-02
 
 0.2477578846
  
 
  
 22
 
  26875499
 
  26875823
 
  PDF   STATS   ENSEMBL   UCSC 
 
  325
 
  6
 
 26822
 
 gene;island
 
 0.068704
 
 0.0128369
 
 Not queried
 
  3.2219307
 
 6.49e-02
 
 0.2477578846
  
 
  
 19
 
    496239
 
    496528
 
  PDF   STATS   ENSEMBL   UCSC 
 
  290
 
  6
 
 21163
 
 island
 
 0.023423
 
 0.2507969
 
 Not queried
 
  3.2023679
 
 6.49e-02
 
 0.2477578846
  
 
  
 22
 
  42095154
 
  42095443
 
  PDF   STATS   ENSEMBL   UCSC 
 
  290
 
  6
 
 27179
 
 tss
 
 0.457559
 
 0.0896919
 
 Not queried
 
  3.1983113
 
 6.49e-02
 
 0.2477578846
  
 
  
 8
 
  49647579
 
  49647833
 
  PDF   STATS   ENSEMBL   UCSC 
 
  255
 
  6
 
 39301
 
 gene
 
 0.251334
 
 0.0274494
 
 Not queried
 
  2.9375586
 
 6.49e-02
 
 0.2477578846
  
 
  
 17
 
  73892982
 
  73893209
 
  PDF   STATS   ENSEMBL   UCSC 
 
  228
 
  6
 
 18767
 
 island
 
 0.012444
 
 0.0802483
 
 Not queried
 
  2.9269891
 
 6.49e-02
 
 0.2477578846
  
 
  
 14
 
  23834860
 
  23834995
 
  PDF   STATS   ENSEMBL   UCSC 
 
  136
 
  6
 
 12302
 
 tss
 
 0.011527
 
 0.1023300
 
 Not queried
 
  2.9204552
 
 6.49e-02
 
 0.2477578846
  
 
  
 14
 
  59932101
 
  59932321
 
  PDF   STATS   ENSEMBL   UCSC 
 
  221
 
  9
 
 12430
 
 tss
 
 0.006991
 
 0.0349439
 
 Not queried
 
  2.9192373
 
 6.25e-02
 
 0.2477578846
  
 
  
 10
 
   5708674
 
   5708881
 
  PDF   STATS   ENSEMBL   UCSC 
 
  208
 
  6
 
 4927
 
 tss;island
 
 0.003619
 
 0.0273606
 
 Not queried
 
  2.8776216
 
 6.49e-02
 
 0.2477578846
  
 
  
 20
 
  24451769
 
  24452131
 
  PDF   STATS   ENSEMBL   UCSC 
 
  363
 
  6
 
 25376
 
 gene;island
 
 0.005000
 
 0.0492387
 
 Not queried
 
  2.8606073
 
 6.49e-02
 
 0.2477578846
  
 
  
 3
 
  49236800
 
  49236946
 
  PDF   STATS   ENSEMBL   UCSC 
 
  147
 
  6
 
 27778
 
 gene;island
 
 0.788691
 
 0.3136714
 
 Not queried
 
  2.8588147
 
 6.49e-02
 
 0.2477578846
  
 
  
 11
 
  31821298
 
  31821764
 
  PDF   STATS   ENSEMBL   UCSC 
 
  467
 
  6
 
 6556
 
 gene;island
 
 0.036663
 
 0.3133859
 
 Not queried
 
  2.7851512
 
 6.49e-02
 
 0.2477578846
  
 
  
 20
 
  36888790
 
  36889389
 
  PDF   STATS   ENSEMBL   UCSC 
 
  600
 
 10
 
 26153
 
 island
 
 0.061722
 
 0.2492586
 
 Not queried
 
  2.7845123
 
 6.30e-02
 
 0.2477578846
  
 
  
 10
 
 125651526
 
 125651726
 
  PDF   STATS   ENSEMBL   UCSC 
 
  201
 
  6
 
 5420
 
 tss
 
 0.019852
 
 0.4641369
 
 Not queried
 
  2.7187928
 
 6.49e-02
 
 0.2477578846
  
 
  
 11
 
   2189562
 
   2189856
 
  PDF   STATS   ENSEMBL   UCSC 
 
  295
 
  6
 
 6452
 
 gene;island
 
 0.151828
 
 0.4996859
 
 Not queried
 
  2.6383077
 
 6.49e-02
 
 0.2477578846
  
 
  
 2
 
  45159894
 
  45160554
 
  PDF   STATS   ENSEMBL   UCSC 
 
  661
 
  8
 
 24260
 
 island
 
 0.036907
 
 0.4659900
 
 Not queried
 
  2.5912538
 
 6.50e-02
 
 0.2477578846
  
 
  
 3
 
  10149803
 
  10150139
 
  PDF   STATS   ENSEMBL   UCSC 
 
  337
 
  9
 
 28871
 
 island
 
 0.968652
 
 0.9123461
 
 Not queried
 
  2.5427612
 
 6.25e-02
 
 0.2477578846
  
 
  
 1
 
 221052350
 
 221052886
 
  PDF   STATS   ENSEMBL   UCSC 
 
  537
 
  8
 
 4221
 
 island
 
 0.003058
 
 0.0189370
 
 Not queried
 
  2.4996819
 
 6.50e-02
 
 0.2477578846
  
 
  
 6
 
 106428957
 
 106429769
 
  PDF   STATS   ENSEMBL   UCSC 
 
  813
 
  9
 
 36377
 
 island
 
 0.148291
 
 0.0416221
 
 Not queried
 
  2.4393848
 
 6.25e-02
 
 0.2477578846
  
 
  
 11
 
  26353484
 
  26353811
 
  PDF   STATS   ENSEMBL   UCSC 
 
  328
 
  8
 
 8140
 
 island
 
 0.024426
 
 0.0526714
 
 Not queried
 
  2.4315410
 
 6.50e-02
 
 0.2477578846
  
 
  
 5
 
   1268644
 
   1269309
 
  PDF   STATS   ENSEMBL   UCSC 
 
  666
 
  8
 
 31531
 
 gene;island
 
 0.961077
 
 0.7684635
 
 Not queried
 
  2.3620066
 
 6.50e-02
 
 0.2477578846
  
 
  
 4
 
   5710319
 
   5710411
 
  PDF   STATS   ENSEMBL   UCSC 
 
   93
 
  6
 
 29921
 
 gene;tss;island
 
 0.066752
 
 0.0141588
 
 Not queried
 
  2.3235219
 
 6.49e-02
 
 0.2477578846
  
 
  
 19
 
  37997675
 
  37997831
 
  PDF   STATS   ENSEMBL   UCSC 
 
  157
 
  8
 
 20740
 
 tss
 
 0.005637
 
 0.0018479
 
 Not queried
 
  2.3204139
 
 6.50e-02
 
 0.2477578846
  
 
  
 6
 
  29817963
 
  29818300
 
  PDF   STATS   ENSEMBL   UCSC 
 
  338
 
  8
 
 35638
 
 island
 
 0.046132
 
 0.0905028
 
 Not queried
 
  2.3196303
 
 6.50e-02
 
 0.2477578846
  
 
  
 4
 
  40751717
 
  40752151
 
  PDF   STATS   ENSEMBL   UCSC 
 
  435
 
  6
 
 30997
 
 island
 
 0.219481
 
 0.0409574
 
 Not queried
 
  2.1970759
 
 6.49e-02
 
 0.2477578846
  
 
  
 1
 
  25228582
 
  25229192
 
  PDF   STATS   ENSEMBL   UCSC 
 
  611
 
  9
 
 329
 
 gene;island
 
 0.734494
 
 0.9772239
 
 Not queried
 
  2.1242676
 
 6.25e-02
 
 0.2477578846
  
 
  
 1
 
 220921216
 
 220921670
 
  PDF   STATS   ENSEMBL   UCSC 
 
  455
 
  8
 
 2262
 
 tss
 
 0.051032
 
 0.2709414
 
 Not queried
 
  2.0980614
 
 6.50e-02
 
 0.2477578846
  
 
  
 10
 
 102881114
 
 102881545
 
  PDF   STATS   ENSEMBL   UCSC 
 
  432
 
  6
 
 4723
 
 gene;island
 
 0.021384
 
 0.2057506
 
 Not queried
 
  2.0604773
 
 6.49e-02
 
 0.2477578846
  
 
  
 10
 
    729479
 
    729956
 
  PDF   STATS   ENSEMBL   UCSC 
 
  478
 
  6
 
 4451
 
 gene
 
 0.444563
 
 0.7609090
 
 Not queried
 
  2.0595891
 
 6.49e-02
 
 0.2477578846
  
 
  
 11
 
  61447487
 
  61447938
 
  PDF   STATS   ENSEMBL   UCSC 
 
  452
 
  6
 
 8330
 
 island
 
 0.039408
 
 0.0050749
 
 Not queried
 
  2.0324335
 
 6.49e-02
 
 0.2477578846
  
 
  
 12
 
  89744471
 
  89744877
 
  PDF   STATS   ENSEMBL   UCSC 
 
  407
 
  8
 
 9238
 
 gene;island
 
 0.016830
 
 0.0879803
 
 Not queried
 
  1.9192747
 
 6.50e-02
 
 0.2477578846
  
 
  
 8
 
 101661767
 
 101662246
 
  PDF   STATS   ENSEMBL   UCSC 
 
  480
 
  9
 
 40498
 
 island
 
 0.040784
 
 0.0071379
 
 Not queried
 
  1.9129980
 
 6.25e-02
 
 0.2477578846
  
 
  
 10
 
 101292403
 
 101292682
 
  PDF   STATS   ENSEMBL   UCSC 
 
  280
 
  9
 
 5282
 
 tss
 
 0.012992
 
 0.0071613
 
 Not queried
 
  1.8748734
 
 6.25e-02
 
 0.2477578846
  
 
  
 14
 
  24803873
 
  24804342
 
  PDF   STATS   ENSEMBL   UCSC 
 
  470
 
  8
 
 12328
 
 tss
 
 0.019944
 
 0.1006235
 
 Not queried
 
  1.8533722
 
 6.50e-02
 
 0.2477578846
  
 
  
 14
 
  70233599
 
  70233918
 
  PDF   STATS   ENSEMBL   UCSC 
 
  320
 
 10
 
 12126
 
 gene;island
 
 0.003792
 
 0.0084055
 
 Not queried
 
  1.8431167
 
 6.30e-02
 
 0.2477578846
  
 
  
 11
 
 126873139
 
 126873689
 
  PDF   STATS   ENSEMBL   UCSC 
 
  551
 
  6
 
 8937
 
 island
 
 0.047846
 
 0.2205410
 
 Not queried
 
  1.8083705
 
 6.49e-02
 
 0.2477578846
  
 
  
 19
 
  54372324
 
  54372713
 
  PDF   STATS   ENSEMBL   UCSC 
 
  390
 
  9
 
 21043
 
 tss
 
 0.009573
 
 0.0347701
 
 Not queried
 
  1.7440319
 
 6.25e-02
 
 0.2477578846
  
 
  
 19
 
  58095424
 
  58095659
 
  PDF   STATS   ENSEMBL   UCSC 
 
  236
 
  8
 
 22475
 
 island
 
 0.078514
 
 0.0134158
 
 Not queried
 
  1.7176508
 
 6.50e-02
 
 0.2477578846
  
 
  
 20
 
  14318152
 
  14318410
 
  PDF   STATS   ENSEMBL   UCSC 
 
  259
 
  6
 
 25362
 
 gene
 
 0.009958
 
 0.0021732
 
 Not queried
 
  1.7014419
 
 6.49e-02
 
 0.2477578846
  
 
  
 5
 
 170740547
 
 170740937
 
  PDF   STATS   ENSEMBL   UCSC 
 
  391
 
  6
 
 33380
 
 island
 
 0.199828
 
 0.4517629
 
 Not queried
 
  1.6538599
 
 6.49e-02
 
 0.2477578846
  
 
  
 11
 
  31819148
 
  31819464
 
  PDF   STATS   ENSEMBL   UCSC 
 
  317
 
  6
 
 6555
 
 gene;island
 
 0.425898
 
 0.1290673
 
 Not queried
 
  1.6531958
 
 6.49e-02
 
 0.2477578846
  
 
  
 8
 
  22960177
 
  22960427
 
  PDF   STATS   ENSEMBL   UCSC 
 
  251
 
  6
 
 39596
 
 tss
 
 0.043281
 
 0.1576534
 
 Not queried
 
  1.6139747
 
 6.49e-02
 
 0.2477578846
  
 
  
 4
 
 115519436
 
 115519593
 
  PDF   STATS   ENSEMBL   UCSC 
 
  158
 
  6
 
 30558
 
 tss;island
 
 0.015226
 
 0.0455028
 
 Not queried
 
  1.4447795
 
 6.49e-02
 
 0.2477578846
  
 
  
 20
 
  43374239
 
  43374406
 
  PDF   STATS   ENSEMBL   UCSC 
 
  168
 
  8
 
 25763
 
 tss;island
 
 0.019277
 
 0.1434019
 
 Not queried
 
  1.4348460
 
 6.50e-02
 
 0.2477578846
  
 
  
 3
 
 153839743
 
 153840718
 
  PDF   STATS   ENSEMBL   UCSC 
 
  976
 
 11
 
 28000
 
 gene;island
 
 0.165598
 
 0.5299559
 
 Not queried
 
  2.9668714
 
 6.52e-02
 
 0.2483354067
  
 
  
 15
 
  26109204
 
  26109614
 
  PDF   STATS   ENSEMBL   UCSC 
 
  411
 
 12
 
 13540
 
 tss;island
 
 0.400495
 
 0.7032765
 
 Not queried
 
  1.9093800
 
 6.84e-02
 
 0.2600243423
  
 
  
 6
 
 160679391
 
 160680213
 
  PDF   STATS   ENSEMBL   UCSC 
 
  823
 
 12
 
 36612
 
 island
 
 0.503102
 
 0.6917235
 
 Not queried
 
  1.6594047
 
 6.84e-02
 
 0.2600243423
  
 
  
 6
 
 101846650
 
 101846805
 
  PDF   STATS   ENSEMBL   UCSC 
 
  156
 
  7
 
 35143
 
 tss
 
 0.118637
 
 0.9476104
 
 Not queried
 
  5.6579689
 
 7.28e-02
 
 0.2713481487
  
 
  
 20
 
  13976093
 
  13976190
 
  PDF   STATS   ENSEMBL   UCSC 
 
   98
 
  7
 
 26012
 
 island
 
 0.964378
 
 0.1845648
 
 Not queried
 
  4.6205166
 
 7.28e-02
 
 0.2713481487
  
 
  
 19
 
  19648890
 
  19649054
 
  PDF   STATS   ENSEMBL   UCSC 
 
  165
 
  7
 
 20645
 
 tss
 
 0.356053
 
 0.0417914
 
 Not queried
 
  4.6024308
 
 7.28e-02
 
 0.2713481487
  
 
  
 11
 
  43602845
 
  43602965
 
  PDF   STATS   ENSEMBL   UCSC 
 
  121
 
  7
 
 8217
 
 island
 
 0.023412
 
 0.0109467
 
 Not queried
 
  3.8291854
 
 7.28e-02
 
 0.2713481487
  
 
  
 11
 
 121986961
 
 121987203
 
  PDF   STATS   ENSEMBL   UCSC 
 
  243
 
  7
 
 6948
 
 gene;tss
 
 0.025171
 
 0.4237142
 
 Not queried
 
  3.5803092
 
 7.28e-02
 
 0.2713481487
  
 
  
 11
 
  94501303
 
  94501467
 
  PDF   STATS   ENSEMBL   UCSC 
 
  165
 
  7
 
 7632
 
 tss
 
 0.034937
 
 0.0058885
 
 Not queried
 
  3.1346055
 
 7.28e-02
 
 0.2713481487
  
 
  
 2
 
 200329486
 
 200329872
 
  PDF   STATS   ENSEMBL   UCSC 
 
  387
 
  7
 
 24992
 
 island
 
 0.017430
 
 0.1067467
 
 Not queried
 
  2.8570718
 
 7.28e-02
 
 0.2713481487
  
 
  
 1
 
 118727904
 
 118728226
 
  PDF   STATS   ENSEMBL   UCSC 
 
  323
 
  7
 
 1875
 
 tss
 
 0.054611
 
 0.5945480
 
 Not queried
 
  2.6230164
 
 7.28e-02
 
 0.2713481487
  
 
  
 7
 
  49812836
 
  49813111
 
  PDF   STATS   ENSEMBL   UCSC 
 
  276
 
  7
 
 37592
 
 tss;island
 
 0.018124
 
 0.0761940
 
 Not queried
 
  2.4510828
 
 7.28e-02
 
 0.2713481487
  
 
  
 6
 
  31276418
 
  31276797
 
  PDF   STATS   ENSEMBL   UCSC 
 
  380
 
  7
 
 35764
 
 island
 
 0.580301
 
 0.0343728
 
 Not queried
 
  2.3869498
 
 7.28e-02
 
 0.2713481487
  
 
  
 16
 
  58498456
 
  58498754
 
  PDF   STATS   ENSEMBL   UCSC 
 
  299
 
  7
 
 15274
 
 tss
 
 0.120966
 
 0.0118378
 
 Not queried
 
  2.2447329
 
 7.28e-02
 
 0.2713481487
  
 
  
 4
 
  57522689
 
  57523055
 
  PDF   STATS   ENSEMBL   UCSC 
 
  367
 
  7
 
 30411
 
 tss
 
 0.008356
 
 0.0514101
 
 Not queried
 
  2.1646051
 
 7.28e-02
 
 0.2713481487
  
 
  
 19
 
  37959744
 
  37959961
 
  PDF   STATS   ENSEMBL   UCSC 
 
  218
 
  7
 
 20739
 
 tss
 
 0.019369
 
 0.0075376
 
 Not queried
 
  2.1516467
 
 7.28e-02
 
 0.2713481487
  
 
  
 18
 
  51750950
 
  51751391
 
  PDF   STATS   ENSEMBL   UCSC 
 
  442
 
  7
 
 19522
 
 island
 
 0.005750
 
 0.0024286
 
 Not queried
 
  2.1303276
 
 7.28e-02
 
 0.2713481487
  
 
  
 17
 
  79304498
 
  79304683
 
  PDF   STATS   ENSEMBL   UCSC 
 
  186
 
  7
 
 17737
 
 tss
 
 0.305353
 
 0.5774052
 
 Not queried
 
  2.0265235
 
 7.28e-02
 
 0.2713481487
  
 
  
 16
 
  56691551
 
  56691849
 
  PDF   STATS   ENSEMBL   UCSC 
 
  299
 
  7
 
 15250
 
 tss
 
 0.006276
 
 0.0305222
 
 Not queried
 
  2.0141844
 
 7.28e-02
 
 0.2713481487
  
 
  
 15
 
  39871808
 
  39872186
 
  PDF   STATS   ENSEMBL   UCSC 
 
  379
 
  7
 
 13576
 
 tss;island
 
 0.386703
 
 0.7069689
 
 Not queried
 
  2.0033086
 
 7.28e-02
 
 0.2713481487
  
 
  
 1
 
  45792540
 
  45792733
 
  PDF   STATS   ENSEMBL   UCSC 
 
  194
 
  7
 
 3198
 
 island
 
 0.016632
 
 0.0053940
 
 Not queried
 
  1.8619213
 
 7.28e-02
 
 0.2713481487
  
 
  
 1
 
 119531625
 
 119532116
 
  PDF   STATS   ENSEMBL   UCSC 
 
  492
 
  7
 
 716
 
 gene
 
 0.054377
 
 0.1418638
 
 Not queried
 
  1.7902012
 
 7.28e-02
 
 0.2713481487
  
 
  
 3
 
 135969387
 
 135969981
 
  PDF   STATS   ENSEMBL   UCSC 
 
  595
 
  7
 
 27959
 
 gene;island
 
 0.031146
 
 0.0057009
 
 Not queried
 
  1.6508736
 
 7.28e-02
 
 0.2713481487
  
 
  
 12
 
  57588031
 
  57588243
 
  PDF   STATS   ENSEMBL   UCSC 
 
  213
 
  7
 
 9811
 
 tss
 
 0.488030
 
 0.7961411
 
 Not queried
 
  1.6239880
 
 7.28e-02
 
 0.2713481487
  
 
  
 2
 
  38152187
 
  38152364
 
  PDF   STATS   ENSEMBL   UCSC 
 
  178
 
  7
 
 23308
 
 tss;island
 
 0.028567
 
 0.0587901
 
 Not queried
 
  1.6114218
 
 7.28e-02
 
 0.2713481487
  
 
  
 19
 
   3606835
 
   3607269
 
  PDF   STATS   ENSEMBL   UCSC 
 
  435
 
  7
 
 20348
 
 tss
 
 0.113927
 
 0.1548680
 
 Not queried
 
  1.4247427
 
 7.28e-02
 
 0.2713481487
  
 
  
 1
 
 247171234
 
 247171838
 
  PDF   STATS   ENSEMBL   UCSC 
 
  605
 
 10
 
 4411
 
 island
 
 0.054074
 
 0.0148760
 
 Not queried
 
  2.5855762
 
 7.53e-02
 
 0.2795801356
  
 
  
 1
 
   3663164
 
   3664084
 
  PDF   STATS   ENSEMBL   UCSC 
 
  921
 
 10
 
 2570
 
 island
 
 0.047230
 
 0.0078903
 
 Not queried
 
  2.2499103
 
 7.53e-02
 
 0.2795801356
  
 
  
 12
 
  22093960
 
  22094750
 
  PDF   STATS   ENSEMBL   UCSC 
 
  791
 
 10
 
 10281
 
 island
 
 0.092075
 
 0.0245975
 
 Not queried
 
  1.5628535
 
 7.53e-02
 
 0.2795801356
  
 
  
 6
 
  31275551
 
  31275881
 
  PDF   STATS   ENSEMBL   UCSC 
 
  331
 
 11
 
 35762
 
 island
 
 0.476808
 
 0.6845095
 
 Not queried
 
  2.6514387
 
 7.59e-02
 
 0.2811835917
  
 
  
 2
 
  27712555
 
  27713092
 
  PDF   STATS   ENSEMBL   UCSC 
 
  538
 
 11
 
 24187
 
 island
 
 0.005316
 
 0.0167768
 
 Not queried
 
  2.0173151
 
 7.59e-02
 
 0.2811835917
  
 
  
 5
 
    191127
 
    191806
 
  PDF   STATS   ENSEMBL   UCSC 
 
  680
 
 11
 
 32573
 
 island
 
 0.161198
 
 0.0482724
 
 Not queried
 
  1.4109916
 
 7.59e-02
 
 0.2811835917
  
 
  
 17
 
   8702369
 
   8702896
 
  PDF   STATS   ENSEMBL   UCSC 
 
  528
 
  9
 
 18008
 
 island
 
 0.675907
 
 0.9567168
 
 Not queried
 
  3.0320807
 
 7.70e-02
 
 0.2825346647
  
 
  
 13
 
  38443634
 
  38444227
 
  PDF   STATS   ENSEMBL   UCSC 
 
  594
 
  9
 
 11678
 
 island
 
 0.031350
 
 0.1410408
 
 Not queried
 
  2.9003858
 
 7.70e-02
 
 0.2825346647
  
 
  
 15
 
  96873850
 
  96874052
 
  PDF   STATS   ENSEMBL   UCSC 
 
  203
 
  9
 
 13492
 
 gene;tss;island
 
 0.060289
 
 0.2298941
 
 Not queried
 
  2.5043616
 
 7.70e-02
 
 0.2825346647
  
 
  
 14
 
  96180576
 
  96181044
 
  PDF   STATS   ENSEMBL   UCSC 
 
  469
 
  9
 
 12590
 
 tss
 
 0.043242
 
 0.1738057
 
 Not queried
 
  2.4168352
 
 7.70e-02
 
 0.2825346647
  
 
  
 11
 
  44087367
 
  44087808
 
  PDF   STATS   ENSEMBL   UCSC 
 
  442
 
  9
 
 7259
 
 tss
 
 0.015183
 
 0.0375473
 
 Not queried
 
  2.2661823
 
 7.70e-02
 
 0.2825346647
  
 
  
 2
 
  84743142
 
  84743573
 
  PDF   STATS   ENSEMBL   UCSC 
 
  432
 
  9
 
 23475
 
 tss
 
 0.046898
 
 0.0185170
 
 Not queried
 
  2.2493785
 
 7.70e-02
 
 0.2825346647
  
 
  
 15
 
  95869918
 
  95870475
 
  PDF   STATS   ENSEMBL   UCSC 
 
  558
 
  9
 
 14484
 
 island
 
 0.059046
 
 0.1767330
 
 Not queried
 
  2.0210336
 
 7.70e-02
 
 0.2825346647
  
 
  
 12
 
  10875874
 
  10876242
 
  PDF   STATS   ENSEMBL   UCSC 
 
  369
 
  9
 
 10242
 
 island
 
 0.003221
 
 0.0016369
 
 Not queried
 
  1.9506943
 
 7.70e-02
 
 0.2825346647
  
 
  
 3
 
 140770213
 
 140770984
 
  PDF   STATS   ENSEMBL   UCSC 
 
  772
 
 14
 
 29543
 
 island
 
 0.009634
 
 0.0484192
 
 Not queried
 
  1.6348469
 
 7.67e-02
 
 0.2825346647
  
 
  
 10
 
 134901199
 
 134901689
 
  PDF   STATS   ENSEMBL   UCSC 
 
  491
 
  9
 
 6332
 
 island
 
 0.076554
 
 0.0236159
 
 Not queried
 
  1.4681376
 
 7.70e-02
 
 0.2825346647
  
 
  
 5
 
 178593785
 
 178594649
 
  PDF   STATS   ENSEMBL   UCSC 
 
  865
 
  9
 
 31929
 
 gene;island
 
 0.072024
 
 0.2156889
 
 Not queried
 
  1.4595105
 
 7.70e-02
 
 0.2825346647
  
 
  
 6
 
  29407834
 
  29408666
 
  PDF   STATS   ENSEMBL   UCSC 
 
  833
 
 13
 
 33656
 
 gene
 
 0.184880
 
 0.6001960
 
 Not queried
 
  1.6839086
 
 8.11e-02
 
 0.2974420553
  
 
  
 3
 
  10857688
 
  10857800
 
  PDF   STATS   ENSEMBL   UCSC 
 
  113
 
  8
 
 28153
 
 tss;island
 
 0.255645
 
 0.5182897
 
 Not queried
 
  2.7893547
 
 8.30e-02
 
 0.3028612233
  
 
  
 11
 
    624457
 
    625147
 
  PDF   STATS   ENSEMBL   UCSC 
 
  691
 
  8
 
 7869
 
 island
 
 0.541067
 
 0.8818389
 
 Not queried
 
  2.2115773
 
 8.30e-02
 
 0.3028612233
  
 
  
 20
 
  30062970
 
  30063249
 
  PDF   STATS   ENSEMBL   UCSC 
 
  280
 
  8
 
 26071
 
 island
 
 0.068107
 
 0.0207092
 
 Not queried
 
  1.8840291
 
 8.30e-02
 
 0.3028612233
  
 
  
 8
 
  33457226
 
  33457822
 
  PDF   STATS   ENSEMBL   UCSC 
 
  597
 
  8
 
 40215
 
 island
 
 0.055377
 
 0.0160468
 
 Not queried
 
  1.7735759
 
 8.30e-02
 
 0.3028612233
  
 
  
 2
 
 171571533
 
 171571941
 
  PDF   STATS   ENSEMBL   UCSC 
 
  409
 
  8
 
 23709
 
 tss;island
 
 0.036043
 
 0.0206971
 
 Not queried
 
  1.5921149
 
 8.30e-02
 
 0.3028612233
  
 
  
 17
 
  29297380
 
  29297873
 
  PDF   STATS   ENSEMBL   UCSC 
 
  494
 
 14
 
 17245
 
 tss;island
 
 0.003779
 
 0.0164551
 
 Not queried
 
  1.6149542
 
 8.49e-02
 
 0.3096829949
  
 
  
 3
 
  71833543
 
  71833907
 
  PDF   STATS   ENSEMBL   UCSC 
 
  365
 
  5
 
 27860
 
 gene;island
 
 0.092742
 
 0.0041900
 
 Not queried
 
  6.1846047
 
 9.52e-02
 
 0.3104222821
  
 
  
 8
 
  22223852
 
  22224272
 
  PDF   STATS   ENSEMBL   UCSC 
 
  421
 
  6
 
 39585
 
 tss;island
 
 0.965579
 
 0.3667395
 
 Not queried
 
  5.5310784
 
 9.31e-02
 
 0.3104222821
  
 
  
 18
 
  74535840
 
  74536113
 
  PDF   STATS   ENSEMBL   UCSC 
 
  274
 
  5
 
 19328
 
 tss
 
 0.556322
 
 0.0373539
 
 Not queried
 
  5.3611383
 
 9.52e-02
 
 0.3104222821
  
 
  
 3
 
 155394109
 
 155394428
 
  PDF   STATS   ENSEMBL   UCSC 
 
  320
 
  5
 
 28004
 
 gene;tss
 
 0.019622
 
 0.4311865
 
 Not queried
 
  5.2440670
 
 9.52e-02
 
 0.3104222821
  
 
  
 5
 
 140613740
 
 140613884
 
  PDF   STATS   ENSEMBL   UCSC 
 
  145
 
  5
 
 32368
 
 tss
 
 0.020419
 
 0.3064247
 
 Not queried
 
  5.1984726
 
 9.52e-02
 
 0.3104222821
  
 
  
 8
 
  95962352
 
  95962463
 
  PDF   STATS   ENSEMBL   UCSC 
 
  112
 
  5
 
 39821
 
 tss;island
 
 0.009554
 
 0.1993835
 
 Not queried
 
  5.0427654
 
 9.52e-02
 
 0.3104222821
  
 
  
 12
 
 133016214
 
 133016787
 
  PDF   STATS   ENSEMBL   UCSC 
 
  574
 
  6
 
 11055
 
 island
 
 0.054808
 
 0.9120153
 
 Not queried
 
  4.9814655
 
 9.31e-02
 
 0.3104222821
  
 
  
 16
 
   3225355
 
   3225686
 
  PDF   STATS   ENSEMBL   UCSC 
 
  332
 
  5
 
 15639
 
 island
 
 0.010129
 
 0.2240548
 
 Not queried
 
  4.8246316
 
 9.52e-02
 
 0.3104222821
  
 
  
 6
 
  29596840
 
  29597113
 
  PDF   STATS   ENSEMBL   UCSC 
 
  274
 
  5
 
 33679
 
 gene;tss;island
 
 0.003409
 
 0.0320932
 
 Not queried
 
  4.6215982
 
 9.52e-02
 
 0.3104222821
  
 
  
 1
 
  29587088
 
  29587490
 
  PDF   STATS   ENSEMBL   UCSC 
 
  403
 
  5
 
 377
 
 gene;island
 
 0.270422
 
 0.6472787
 
 Not queried
 
  4.3535818
 
 9.52e-02
 
 0.3104222821
  
 
  
 2
 
  66667039
 
  66667101
 
  PDF   STATS   ENSEMBL   UCSC 
 
   63
 
  5
 
 22711
 
 gene
 
 0.012587
 
 0.1830191
 
 Not queried
 
  4.2409951
 
 9.52e-02
 
 0.3104222821
  
 
  
 7
 
 150652948
 
 150653195
 
  PDF   STATS   ENSEMBL   UCSC 
 
  248
 
  5
 
 37984
 
 tss
 
 0.026037
 
 0.1906653
 
 Not queried
 
  4.2208483
 
 9.52e-02
 
 0.3104222821
  
 
  
 6
 
  31237664
 
  31238036
 
  PDF   STATS   ENSEMBL   UCSC 
 
  373
 
  5
 
 33865
 
 gene;island
 
 0.820402
 
 0.4674876
 
 Not queried
 
  4.2031424
 
 9.52e-02
 
 0.3104222821
  
 
  
 1
 
  91190891
 
  91191254
 
  PDF   STATS   ENSEMBL   UCSC 
 
  364
 
  5
 
 3462
 
 island
 
 0.132433
 
 0.4287169
 
 Not queried
 
  4.1210459
 
 9.52e-02
 
 0.3104222821
  
 
  
 13
 
  98869706
 
  98870144
 
  PDF   STATS   ENSEMBL   UCSC 
 
  439
 
  5
 
 11198
 
 gene
 
 0.628782
 
 0.9535963
 
 Not queried
 
  4.0669244
 
 9.52e-02
 
 0.3104222821
  
 
  
 7
 
  76029648
 
  76029881
 
  PDF   STATS   ENSEMBL   UCSC 
 
  234
 
  5
 
 37057
 
 gene;island
 
 0.808568
 
 0.9832735
 
 Not queried
 
  3.8008185
 
 9.52e-02
 
 0.3104222821
  
 
  
 17
 
  78161467
 
  78161572
 
  PDF   STATS   ENSEMBL   UCSC 
 
  106
 
  5
 
 17722
 
 tss
 
 0.458785
 
 0.9001853
 
 Not queried
 
  3.6217913
 
 9.52e-02
 
 0.3104222821
  
 
  
 8
 
  28174814
 
  28175021
 
  PDF   STATS   ENSEMBL   UCSC 
 
  208
 
  5
 
 39261
 
 gene
 
 0.770122
 
 0.4701871
 
 Not queried
 
  3.6116236
 
 9.52e-02
 
 0.3104222821
  
 
  
 5
 
 140723331
 
 140723583
 
  PDF   STATS   ENSEMBL   UCSC 
 
  253
 
  6
 
 32373
 
 tss
 
 0.349565
 
 0.0177258
 
 Not queried
 
  3.5873424
 
 9.31e-02
 
 0.3104222821
  
 
  
 8
 
  27468981
 
  27469338
 
  PDF   STATS   ENSEMBL   UCSC 
 
  358
 
  5
 
 39257
 
 gene;tss;island
 
 0.020508
 
 0.1857063
 
 Not queried
 
  3.5745465
 
 9.52e-02
 
 0.3104222821
  
 
  
 1
 
  47915553
 
  47915951
 
  PDF   STATS   ENSEMBL   UCSC 
 
  399
 
  6
 
 3241
 
 island
 
 0.018629
 
 0.3780236
 
 Not queried
 
  3.4815464
 
 9.31e-02
 
 0.3104222821
  
 
  
 11
 
  43602845
 
  43602920
 
  PDF   STATS   ENSEMBL   UCSC 
 
   76
 
  6
 
 7254
 
 tss
 
 0.021060
 
 0.0074671
 
 Not queried
 
  3.3709655
 
 9.31e-02
 
 0.3104222821
  
 
  
 12
 
 106974832
 
 106975246
 
  PDF   STATS   ENSEMBL   UCSC 
 
  415
 
  5
 
 10751
 
 island
 
 0.036412
 
 0.2969122
 
 Not queried
 
  3.3558852
 
 9.52e-02
 
 0.3104222821
  
 
  
 17
 
   9694619
 
   9695137
 
  PDF   STATS   ENSEMBL   UCSC 
 
  519
 
  6
 
 17130
 
 tss
 
 0.137268
 
 0.5104770
 
 Not queried
 
  3.3396720
 
 9.31e-02
 
 0.3104222821
  
 
  
 20
 
  11871282
 
  11871396
 
  PDF   STATS   ENSEMBL   UCSC 
 
  115
 
  5
 
 25600
 
 tss
 
 0.248713
 
 0.0188459
 
 Not queried
 
  3.3258084
 
 9.52e-02
 
 0.3104222821
  
 
  
 5
 
 178422128
 
 178422415
 
  PDF   STATS   ENSEMBL   UCSC 
 
  288
 
  5
 
 32546
 
 tss
 
 0.376135
 
 0.0575165
 
 Not queried
 
  3.3083793
 
 9.52e-02
 
 0.3104222821
  
 
  
 10
 
  42863444
 
  42863594
 
  PDF   STATS   ENSEMBL   UCSC 
 
  151
 
  5
 
 5680
 
 island
 
 0.258469
 
 0.0270393
 
 Not queried
 
  3.2861170
 
 9.52e-02
 
 0.3104222821
  
 
  
 7
 
   1329387
 
   1329592
 
  PDF   STATS   ENSEMBL   UCSC 
 
  206
 
  5
 
 38097
 
 island
 
 0.012470
 
 0.4757445
 
 Not queried
 
  3.2535815
 
 9.52e-02
 
 0.3104222821
  
 
  
 6
 
  29079939
 
  29080437
 
  PDF   STATS   ENSEMBL   UCSC 
 
  499
 
  5
 
 33650
 
 gene
 
 0.066728
 
 0.4689336
 
 Not queried
 
  3.1754233
 
 9.52e-02
 
 0.3104222821
  
 
  
 10
 
    695844
 
    695900
 
  PDF   STATS   ENSEMBL   UCSC 
 
   57
 
  5
 
 4910
 
 tss
 
 0.487210
 
 0.8927664
 
 Not queried
 
  3.1534377
 
 9.52e-02
 
 0.3104222821
  
 
  
 17
 
  46871708
 
  46871905
 
  PDF   STATS   ENSEMBL   UCSC 
 
  198
 
  5
 
 17473
 
 tss
 
 0.451132
 
 0.8696867
 
 Not queried
 
  3.1217641
 
 9.52e-02
 
 0.3104222821
  
 
  
 19
 
  50553682
 
  50553907
 
  PDF   STATS   ENSEMBL   UCSC 
 
  226
 
  5
 
 20971
 
 tss
 
 0.013263
 
 0.1089239
 
 Not queried
 
  3.1193164
 
 9.52e-02
 
 0.3104222821
  
 
  
 12
 
  81110706
 
  81111012
 
  PDF   STATS   ENSEMBL   UCSC 
 
  307
 
  5
 
 10648
 
 island
 
 0.096599
 
 0.5430759
 
 Not queried
 
  3.0801984
 
 9.52e-02
 
 0.3104222821
  
 
  
 1
 
 166916762
 
 166917267
 
  PDF   STATS   ENSEMBL   UCSC 
 
  506
 
  5
 
 872
 
 gene;island
 
 0.136823
 
 0.0219466
 
 Not queried
 
  3.0765717
 
 9.52e-02
 
 0.3104222821
  
 
  
 19
 
  53832311
 
  53832670
 
  PDF   STATS   ENSEMBL   UCSC 
 
  360
 
  5
 
 22374
 
 island
 
 0.558452
 
 0.1341545
 
 Not queried
 
  3.0424334
 
 9.52e-02
 
 0.3104222821
  
 
  
 17
 
  79495268
 
  79495348
 
  PDF   STATS   ENSEMBL   UCSC 
 
   81
 
  5
 
 17740
 
 tss
 
 0.881945
 
 0.5287471
 
 Not queried
 
  3.0361396
 
 9.52e-02
 
 0.3104222821
  
 
  
 13
 
  38443950
 
  38444227
 
  PDF   STATS   ENSEMBL   UCSC 
 
  278
 
  6
 
 11401
 
 tss
 
 0.040362
 
 0.1381480
 
 Not queried
 
  3.0083693
 
 9.31e-02
 
 0.3104222821
  
 
  
 1
 
  17746286
 
  17746597
 
  PDF   STATS   ENSEMBL   UCSC 
 
  312
 
  6
 
 266
 
 gene;island
 
 0.953764
 
 0.4738686
 
 Not queried
 
  2.9864004
 
 9.31e-02
 
 0.3104222821
  
 
  
 7
 
 114055074
 
 114055210
 
  PDF   STATS   ENSEMBL   UCSC 
 
  137
 
  6
 
 37192
 
 gene
 
 0.286109
 
 0.6217738
 
 Not queried
 
  2.9496767
 
 9.31e-02
 
 0.3104222821
  
 
  
 4
 
 111397134
 
 111397220
 
  PDF   STATS   ENSEMBL   UCSC 
 
   87
 
  5
 
 30548
 
 tss
 
 0.459156
 
 0.8564763
 
 Not queried
 
  2.9289814
 
 9.52e-02
 
 0.3104222821
  
 
  
 5
 
  32710456
 
  32710882
 
  PDF   STATS   ENSEMBL   UCSC 
 
  427
 
  6
 
 32008
 
 tss;island
 
 0.288472
 
 0.0219898
 
 Not queried
 
  2.9257164
 
 9.31e-02
 
 0.3104222821
  
 
  
 22
 
  24823389
 
  24823519
 
  PDF   STATS   ENSEMBL   UCSC 
 
  131
 
  5
 
 27019
 
 tss
 
 0.016175
 
 0.0569364
 
 Not queried
 
  2.8994921
 
 9.52e-02
 
 0.3104222821
  
 
  
 17
 
   6796745
 
   6797119
 
  PDF   STATS   ENSEMBL   UCSC 
 
  375
 
  5
 
 16378
 
 gene;island
 
 0.286622
 
 0.5248991
 
 Not queried
 
  2.8974398
 
 9.52e-02
 
 0.3104222821
  
 
  
 8
 
  33457483
 
  33457822
 
  PDF   STATS   ENSEMBL   UCSC 
 
  340
 
  6
 
 39642
 
 tss
 
 0.096896
 
 0.0117201
 
 Not queried
 
  2.8781884
 
 9.31e-02
 
 0.3104222821
  
 
  
 11
 
  63871228
 
  63871343
 
  PDF   STATS   ENSEMBL   UCSC 
 
  116
 
  5
 
 6663
 
 gene;tss
 
 0.169922
 
 0.6777335
 
 Not queried
 
  2.8451308
 
 9.52e-02
 
 0.3104222821
  
 
  
 11
 
  17498514
 
  17498619
 
  PDF   STATS   ENSEMBL   UCSC 
 
  106
 
  6
 
 7176
 
 tss
 
 0.049513
 
 0.1155710
 
 Not queried
 
  2.8095599
 
 9.31e-02
 
 0.3104222821
  
 
  
 6
 
  33943520
 
  33943929
 
  PDF   STATS   ENSEMBL   UCSC 
 
  410
 
  5
 
 36048
 
 island
 
 0.070865
 
 0.2483905
 
 Not queried
 
  2.7972694
 
 9.52e-02
 
 0.3104222821
  
 
  
 7
 
  95401692
 
  95401856
 
  PDF   STATS   ENSEMBL   UCSC 
 
  165
 
  5
 
 38683
 
 island
 
 0.014207
 
 0.0297380
 
 Not queried
 
  2.7671712
 
 9.52e-02
 
 0.3104222821
  
 
  
 16
 
   8768425
 
   8768700
 
  PDF   STATS   ENSEMBL   UCSC 
 
  276
 
  5
 
 15685
 
 island
 
 0.035745
 
 0.0065167
 
 Not queried
 
  2.7388049
 
 9.52e-02
 
 0.3104222821
  
 
  
 16
 
   1060367
 
   1060765
 
  PDF   STATS   ENSEMBL   UCSC 
 
  399
 
  5
 
 15506
 
 island
 
 0.096046
 
 0.1694025
 
 Not queried
 
  2.7387517
 
 9.52e-02
 
 0.3104222821
  
 
  
 12
 
 103311462
 
 103311761
 
  PDF   STATS   ENSEMBL   UCSC 
 
  300
 
  6
 
 9951
 
 tss
 
 0.015379
 
 0.0560934
 
 Not queried
 
  2.6804617
 
 9.31e-02
 
 0.3104222821
  
 
  
 6
 
  32297615
 
  32297861
 
  PDF   STATS   ENSEMBL   UCSC 
 
  247
 
  5
 
 34101
 
 gene
 
 0.825668
 
 0.9801835
 
 Not queried
 
  2.6521827
 
 9.52e-02
 
 0.3104222821
  
 
  
 14
 
  69951720
 
  69952104
 
  PDF   STATS   ENSEMBL   UCSC 
 
  385
 
  5
 
 12125
 
 gene;island
 
 0.260396
 
 0.5286518
 
 Not queried
 
  2.6400181
 
 9.52e-02
 
 0.3104222821
  
 
  
 20
 
  61866902
 
  61867533
 
  PDF   STATS   ENSEMBL   UCSC 
 
  632
 
 10
 
 26346
 
 island
 
 0.234251
 
 0.6747862
 
 Not queried
 
  2.6389507
 
 8.92e-02
 
 0.3104222821
  
 
  
 14
 
  96670628
 
  96671104
 
  PDF   STATS   ENSEMBL   UCSC 
 
  477
 
 10
 
 12592
 
 tss
 
 0.060094
 
 0.4271878
 
 Not queried
 
  2.6262520
 
 8.92e-02
 
 0.3104222821
  
 
  
 12
 
  64784483
 
  64784686
 
  PDF   STATS   ENSEMBL   UCSC 
 
  204
 
  5
 
 9841
 
 tss;island
 
 0.019824
 
 0.1090484
 
 Not queried
 
  2.6137007
 
 9.52e-02
 
 0.3104222821
  
 
  
 12
 
 103344336
 
 103344685
 
  PDF   STATS   ENSEMBL   UCSC 
 
  350
 
  5
 
 10727
 
 island
 
 0.011664
 
 0.1041506
 
 Not queried
 
  2.6122145
 
 9.52e-02
 
 0.3104222821
  
 
  
 7
 
 157982161
 
 157982589
 
  PDF   STATS   ENSEMBL   UCSC 
 
  429
 
  5
 
 37361
 
 gene;island
 
 0.050835
 
 0.2413452
 
 Not queried
 
  2.5728990
 
 9.52e-02
 
 0.3104222821
  
 
  
 2
 
  45029543
 
  45030072
 
  PDF   STATS   ENSEMBL   UCSC 
 
  530
 
  6
 
 24257
 
 island
 
 0.039261
 
 0.2692720
 
 Not queried
 
  2.5329829
 
 9.31e-02
 
 0.3104222821
  
 
  
 11
 
  14993378
 
  14993818
 
  PDF   STATS   ENSEMBL   UCSC 
 
  441
 
  6
 
 6520
 
 gene
 
 0.097303
 
 0.4085192
 
 Not queried
 
  2.5264605
 
 9.31e-02
 
 0.3104222821
  
 
  
 1
 
 205290869
 
 205291215
 
  PDF   STATS   ENSEMBL   UCSC 
 
  347
 
  5
 
 4126
 
 island
 
 0.048680
 
 0.2106659
 
 Not queried
 
  2.5063453
 
 9.52e-02
 
 0.3104222821
  
 
  
 6
 
  44355135
 
  44355287
 
  PDF   STATS   ENSEMBL   UCSC 
 
  153
 
  6
 
 35014
 
 tss;island
 
 0.001748
 
 0.0053898
 
 Not queried
 
  2.4577518
 
 9.31e-02
 
 0.3104222821
  
 
  
 11
 
  69933927
 
  69934128
 
  PDF   STATS   ENSEMBL   UCSC 
 
  202
 
  5
 
 6786
 
 gene;island
 
 0.051572
 
 0.7081570
 
 Not queried
 
  2.4567804
 
 9.52e-02
 
 0.3104222821
  
 
  
 1
 
 197887955
 
 197888512
 
  PDF   STATS   ENSEMBL   UCSC 
 
  558
 
  6
 
 931
 
 gene;island
 
 0.197446
 
 0.0170297
 
 Not queried
 
  2.4543565
 
 9.31e-02
 
 0.3104222821
  
 
  
 17
 
   4337082
 
   4337202
 
  PDF   STATS   ENSEMBL   UCSC 
 
  121
 
  5
 
 17023
 
 tss
 
 0.011567
 
 0.0665275
 
 Not queried
 
  2.4331331
 
 9.52e-02
 
 0.3104222821
  
 
  
 14
 
 104690040
 
 104690244
 
  PDF   STATS   ENSEMBL   UCSC 
 
  205
 
  5
 
 13204
 
 island
 
 0.202987
 
 0.0334864
 
 Not queried
 
  2.4102053
 
 9.52e-02
 
 0.3104222821
  
 
  
 19
 
  15121204
 
  15121596
 
  PDF   STATS   ENSEMBL   UCSC 
 
  393
 
  9
 
 21691
 
 island
 
 0.447423
 
 0.8079015
 
 Not queried
 
  2.3557961
 
 9.39e-02
 
 0.3104222821
  
 
  
 15
 
  26026152
 
  26026414
 
  PDF   STATS   ENSEMBL   UCSC 
 
  263
 
  6
 
 13260
 
 gene
 
 0.360181
 
 0.6919758
 
 Not queried
 
  2.3534989
 
 9.31e-02
 
 0.3104222821
  
 
  
 5
 
  78365647
 
  78365801
 
  PDF   STATS   ENSEMBL   UCSC 
 
  155
 
  5
 
 31660
 
 gene;tss;island
 
 0.546893
 
 0.1859017
 
 Not queried
 
  2.3270673
 
 9.52e-02
 
 0.3104222821
  
 
  
 1
 
  47184738
 
  47185236
 
  PDF   STATS   ENSEMBL   UCSC 
 
  499
 
  5
 
 1605
 
 tss
 
 0.002003
 
 0.0076806
 
 Not queried
 
  2.3124937
 
 9.52e-02
 
 0.3104222821
  
 
  
 4
 
 170678860
 
 170679082
 
  PDF   STATS   ENSEMBL   UCSC 
 
  223
 
  5
 
 30176
 
 gene
 
 0.005115
 
 0.0012807
 
 Not queried
 
  2.3108859
 
 9.52e-02
 
 0.3104222821
  
 
  
 19
 
  12203029
 
  12203349
 
  PDF   STATS   ENSEMBL   UCSC 
 
  321
 
  5
 
 21592
 
 island
 
 0.023803
 
 0.0064804
 
 Not queried
 
  2.3027970
 
 9.52e-02
 
 0.3104222821
  
 
  
 12
 
  53183773
 
  53184086
 
  PDF   STATS   ENSEMBL   UCSC 
 
  314
 
  5
 
 9140
 
 gene;island
 
 0.025678
 
 0.1393825
 
 Not queried
 
  2.2970656
 
 9.52e-02
 
 0.3104222821
  
 
  
 6
 
  33241226
 
  33241770
 
  PDF   STATS   ENSEMBL   UCSC 
 
  545
 
  5
 
 34245
 
 gene;island
 
 0.063201
 
 0.4635804
 
 Not queried
 
  2.2792101
 
 9.52e-02
 
 0.3104222821
  
 
  
 12
 
 123351548
 
 123351868
 
  PDF   STATS   ENSEMBL   UCSC 
 
  321
 
  5
 
 9374
 
 gene;island
 
 0.967463
 
 0.9092432
 
 Not queried
 
  2.2721173
 
 9.52e-02
 
 0.3104222821
  
 
  
 2
 
   3642098
 
   3642634
 
  PDF   STATS   ENSEMBL   UCSC 
 
  537
 
  6
 
 23163
 
 tss
 
 0.959802
 
 0.7949537
 
 Not queried
 
  2.2622350
 
 9.31e-02
 
 0.3104222821
  
 
  
 4
 
 187111841
 
 187112145
 
  PDF   STATS   ENSEMBL   UCSC 
 
  305
 
  5
 
 30705
 
 tss;island
 
 0.006144
 
 0.0014688
 
 Not queried
 
  2.2412771
 
 9.52e-02
 
 0.3104222821
  
 
  
 5
 
  95768997
 
  95769079
 
  PDF   STATS   ENSEMBL   UCSC 
 
   83
 
  6
 
 32189
 
 tss
 
 0.403526
 
 0.1761539
 
 Not queried
 
  2.2309695
 
 9.31e-02
 
 0.3104222821
  
 
  
 13
 
  36871646
 
  36871962
 
  PDF   STATS   ENSEMBL   UCSC 
 
  317
 
  6
 
 11129
 
 gene
 
 0.050261
 
 0.2166580
 
 Not queried
 
  2.2238911
 
 9.31e-02
 
 0.3104222821
  
 
  
 10
 
  71892694
 
  71892787
 
  PDF   STATS   ENSEMBL   UCSC 
 
   94
 
  6
 
 5130
 
 tss
 
 0.006243
 
 0.1497056
 
 Not queried
 
  2.2118264
 
 9.31e-02
 
 0.3104222821
  
 
  
 19
 
  58520847
 
  58521273
 
  PDF   STATS   ENSEMBL   UCSC 
 
  427
 
  6
 
 22490
 
 island
 
 0.102892
 
 0.0366759
 
 Not queried
 
  2.1921434
 
 9.31e-02
 
 0.3104222821
  
 
  
 3
 
  64430634
 
  64431038
 
  PDF   STATS   ENSEMBL   UCSC 
 
  405
 
  5
 
 29261
 
 island
 
 0.036546
 
 0.1710696
 
 Not queried
 
  2.1751511
 
 9.52e-02
 
 0.3104222821
  
 
  
 6
 
 105400884
 
 105401339
 
  PDF   STATS   ENSEMBL   UCSC 
 
  456
 
  5
 
 36373
 
 island
 
 0.389981
 
 0.0503490
 
 Not queried
 
  2.1745710
 
 9.52e-02
 
 0.3104222821
  
 
  
 10
 
  72163475
 
  72163814
 
  PDF   STATS   ENSEMBL   UCSC 
 
  340
 
  5
 
 5134
 
 tss
 
 0.011915
 
 0.0024626
 
 Not queried
 
  2.1490348
 
 9.52e-02
 
 0.3104222821
  
 
  
 7
 
 121955208
 
 121955894
 
  PDF   STATS   ENSEMBL   UCSC 
 
  687
 
  6
 
 38873
 
 island
 
 0.012057
 
 0.0554851
 
 Not queried
 
  2.1450270
 
 9.31e-02
 
 0.3104222821
  
 
  
 12
 
 131443231
 
 131443486
 
  PDF   STATS   ENSEMBL   UCSC 
 
  256
 
  6
 
 9421
 
 gene
 
 0.826439
 
 0.9643836
 
 Not queried
 
  2.1017578
 
 9.31e-02
 
 0.3104222821
  
 
  
 14
 
  65016517
 
  65016602
 
  PDF   STATS   ENSEMBL   UCSC 
 
   86
 
  5
 
 12460
 
 tss;island
 
 0.031029
 
 0.0530443
 
 Not queried
 
  2.0782949
 
 9.52e-02
 
 0.3104222821
  
 
  
 11
 
  67442067
 
  67442408
 
  PDF   STATS   ENSEMBL   UCSC 
 
  342
 
  6
 
 6764
 
 gene
 
 0.288648
 
 0.6266378
 
 Not queried
 
  2.0771339
 
 9.31e-02
 
 0.3104222821
  
 
  
 12
 
  82152229
 
  82152625
 
  PDF   STATS   ENSEMBL   UCSC 
 
  397
 
  5
 
 9229
 
 gene;island
 
 0.020127
 
 0.1590574
 
 Not queried
 
  2.0659216
 
 9.52e-02
 
 0.3104222821
  
 
  
 4
 
 186435443
 
 186435667
 
  PDF   STATS   ENSEMBL   UCSC 
 
  225
 
  6
 
 30210
 
 gene
 
 0.628932
 
 0.8811745
 
 Not queried
 
  2.0644090
 
 9.31e-02
 
 0.3104222821
  
 
  
 1
 
  78511600
 
  78512129
 
  PDF   STATS   ENSEMBL   UCSC 
 
  530
 
  5
 
 595
 
 gene
 
 0.019036
 
 0.0589176
 
 Not queried
 
  2.0629246
 
 9.52e-02
 
 0.3104222821
  
 
  
 16
 
  80966033
 
  80966491
 
  PDF   STATS   ENSEMBL   UCSC 
 
  459
 
  6
 
 16118
 
 island
 
 0.071428
 
 0.0118588
 
 Not queried
 
  2.0535453
 
 9.31e-02
 
 0.3104222821
  
 
  
 2
 
 182545485
 
 182545934
 
  PDF   STATS   ENSEMBL   UCSC 
 
  450
 
  9
 
 23763
 
 tss;island
 
 0.126450
 
 0.0137518
 
 Not queried
 
  2.0486817
 
 9.39e-02
 
 0.3104222821
  
 
  
 18
 
  42643730
 
  42643936
 
  PDF   STATS   ENSEMBL   UCSC 
 
  207
 
  5
 
 19097
 
 gene;island
 
 0.974794
 
 0.9127336
 
 Not queried
 
  2.0129905
 
 9.52e-02
 
 0.3104222821
  
 
  
 14
 
  57261601
 
  57262177
 
  PDF   STATS   ENSEMBL   UCSC 
 
  577
 
  5
 
 12874
 
 island
 
 0.119282
 
 0.2884768
 
 Not queried
 
  2.0119961
 
 9.52e-02
 
 0.3104222821
  
 
  
 2
 
   9533573
 
   9533887
 
  PDF   STATS   ENSEMBL   UCSC 
 
  315
 
  5
 
 22563
 
 gene;island
 
 0.768865
 
 0.9294884
 
 Not queried
 
  2.0035170
 
 9.52e-02
 
 0.3104222821
  
 
  
 17
 
  27045043
 
  27045176
 
  PDF   STATS   ENSEMBL   UCSC 
 
  134
 
  5
 
 16500
 
 gene
 
 0.020503
 
 0.0742646
 
 Not queried
 
  1.9775758
 
 9.52e-02
 
 0.3104222821
  
 
  
 6
 
  31683051
 
  31683131
 
  PDF   STATS   ENSEMBL   UCSC 
 
   81
 
  5
 
 34831
 
 tss
 
 0.278949
 
 0.6078311
 
 Not queried
 
  1.9402463
 
 9.52e-02
 
 0.3104222821
  
 
  
 8
 
 143823734
 
 143824001
 
  PDF   STATS   ENSEMBL   UCSC 
 
  268
 
  5
 
 40642
 
 island
 
 0.450400
 
 0.7522281
 
 Not queried
 
  1.9391753
 
 9.52e-02
 
 0.3104222821
  
 
  
 2
 
 220378996
 
 220379260
 
  PDF   STATS   ENSEMBL   UCSC 
 
  265
 
  9
 
 23036
 
 gene;island
 
 0.009555
 
 0.0227615
 
 Not queried
 
  1.9389787
 
 9.39e-02
 
 0.3104222821
  
 
  
 13
 
  98829521
 
  98829666
 
  PDF   STATS   ENSEMBL   UCSC 
 
  146
 
  5
 
 11508
 
 tss
 
 0.473666
 
 0.8191273
 
 Not queried
 
  1.9230504
 
 9.52e-02
 
 0.3104222821
  
 
  
 6
 
  28186797
 
  28186923
 
  PDF   STATS   ENSEMBL   UCSC 
 
  127
 
  6
 
 34735
 
 tss
 
 0.016732
 
 0.0505087
 
 Not queried
 
  1.8788129
 
 9.31e-02
 
 0.3104222821
  
 
  
 8
 
  42911287
 
  42911370
 
  PDF   STATS   ENSEMBL   UCSC 
 
   84
 
  5
 
 39684
 
 tss
 
 0.000901
 
 0.0032045
 
 Not queried
 
  1.8338803
 
 9.52e-02
 
 0.3104222821
  
 
  
 13
 
 111522755
 
 111522985
 
  PDF   STATS   ENSEMBL   UCSC 
 
  231
 
  5
 
 11540
 
 tss
 
 0.645199
 
 0.9104279
 
 Not queried
 
  1.8286299
 
 9.52e-02
 
 0.3104222821
  
 
  
 7
 
  63505584
 
  63505811
 
  PDF   STATS   ENSEMBL   UCSC 
 
  228
 
  6
 
 37613
 
 tss
 
 0.069717
 
 0.2963209
 
 Not queried
 
  1.8235987
 
 9.31e-02
 
 0.3104222821
  
 
  
 11
 
   1506936
 
   1507347
 
  PDF   STATS   ENSEMBL   UCSC 
 
  412
 
  6
 
 6423
 
 gene
 
 0.495356
 
 0.8137552
 
 Not queried
 
  1.7806653
 
 9.31e-02
 
 0.3104222821
  
 
  
 1
 
 228473704
 
 228473980
 
  PDF   STATS   ENSEMBL   UCSC 
 
  277
 
  5
 
 1054
 
 gene;island
 
 0.178439
 
 0.5230743
 
 Not queried
 
  1.7793898
 
 9.52e-02
 
 0.3104222821
  
 
  
 18
 
  44557635
 
  44557819
 
  PDF   STATS   ENSEMBL   UCSC 
 
  185
 
  5
 
 19105
 
 gene;tss;island
 
 0.362254
 
 0.8108857
 
 Not queried
 
  1.7665731
 
 9.52e-02
 
 0.3104222821
  
 
  
 11
 
  63852804
 
  63853037
 
  PDF   STATS   ENSEMBL   UCSC 
 
  234
 
  5
 
 6662
 
 gene;island
 
 0.924688
 
 0.9805733
 
 Not queried
 
  1.7661687
 
 9.52e-02
 
 0.3104222821
  
 
  
 12
 
  95945082
 
  95945384
 
  PDF   STATS   ENSEMBL   UCSC 
 
  303
 
  5
 
 10689
 
 island
 
 0.673580
 
 0.8773202
 
 Not queried
 
  1.7639438
 
 9.52e-02
 
 0.3104222821
  
 
  
 1
 
 206786000
 
 206786222
 
  PDF   STATS   ENSEMBL   UCSC 
 
  223
 
  6
 
 2215
 
 tss;island
 
 0.903707
 
 0.5346124
 
 Not queried
 
  1.7572686
 
 9.31e-02
 
 0.3104222821
  
 
  
 2
 
  79220029
 
  79220528
 
  PDF   STATS   ENSEMBL   UCSC 
 
  500
 
  5
 
 24460
 
 island
 
 0.020564
 
 0.0581392
 
 Not queried
 
  1.7466560
 
 9.52e-02
 
 0.3104222821
  
 
  
 1
 
   3104999
 
   3105326
 
  PDF   STATS   ENSEMBL   UCSC 
 
  328
 
  5
 
 107
 
 gene;island
 
 0.328052
 
 0.5736146
 
 Not queried
 
  1.7150999
 
 9.52e-02
 
 0.3104222821
  
 
  
 7
 
 157700872
 
 157701262
 
  PDF   STATS   ENSEMBL   UCSC 
 
  391
 
  5
 
 37354
 
 gene;island
 
 0.269667
 
 0.5465449
 
 Not queried
 
  1.7101311
 
 9.52e-02
 
 0.3104222821
  
 
  
 6
 
 112575850
 
 112576119
 
  PDF   STATS   ENSEMBL   UCSC 
 
  270
 
  5
 
 35183
 
 tss
 
 0.204222
 
 0.4897964
 
 Not queried
 
  1.7029860
 
 9.52e-02
 
 0.3104222821
  
 
  
 5
 
   5303295
 
   5303564
 
  PDF   STATS   ENSEMBL   UCSC 
 
  270
 
  5
 
 31553
 
 gene;island
 
 0.806112
 
 0.5990253
 
 Not queried
 
  1.7011209
 
 9.52e-02
 
 0.3104222821
  
 
  
 8
 
   1364427
 
   1364689
 
  PDF   STATS   ENSEMBL   UCSC 
 
  263
 
  5
 
 40023
 
 island
 
 0.554491
 
 0.6840260
 
 Not queried
 
  1.7008843
 
 9.52e-02
 
 0.3104222821
  
 
  
 3
 
  45837898
 
  45838338
 
  PDF   STATS   ENSEMBL   UCSC 
 
  441
 
 10
 
 29051
 
 island
 
 0.056656
 
 0.0277113
 
 Not queried
 
  1.6976644
 
 8.92e-02
 
 0.3104222821
  
 
  
 1
 
  32827707
 
  32828191
 
  PDF   STATS   ENSEMBL   UCSC 
 
  485
 
  9
 
 395
 
 gene;tss;island
 
 0.058992
 
 0.1761777
 
 Not queried
 
  1.6775940
 
 9.39e-02
 
 0.3104222821
  
 
  
 6
 
  13274151
 
  13274354
 
  PDF   STATS   ENSEMBL   UCSC 
 
  204
 
  5
 
 33575
 
 gene
 
 0.003993
 
 0.0197653
 
 Not queried
 
  1.6692270
 
 9.52e-02
 
 0.3104222821
  
 
  
 19
 
   5507274
 
   5507540
 
  PDF   STATS   ENSEMBL   UCSC 
 
  267
 
  5
 
 21405
 
 island
 
 0.700655
 
 0.9439743
 
 Not queried
 
  1.6590552
 
 9.52e-02
 
 0.3104222821
  
 
  
 11
 
  70961596
 
  70961902
 
  PDF   STATS   ENSEMBL   UCSC 
 
  307
 
  5
 
 8616
 
 island
 
 0.111143
 
 0.0122260
 
 Not queried
 
  1.5756572
 
 9.52e-02
 
 0.3104222821
  
 
  
 16
 
   2765620
 
   2765900
 
  PDF   STATS   ENSEMBL   UCSC 
 
  281
 
  5
 
 14610
 
 gene;island
 
 0.067689
 
 0.0212775
 
 Not queried
 
  1.5531543
 
 9.52e-02
 
 0.3104222821
  
 
  
 10
 
  21783445
 
  21783930
 
  PDF   STATS   ENSEMBL   UCSC 
 
  486
 
  5
 
 4525
 
 gene
 
 0.004842
 
 0.0169939
 
 Not queried
 
  1.5403828
 
 9.52e-02
 
 0.3104222821
  
 
  
 10
 
  71811927
 
  71812332
 
  PDF   STATS   ENSEMBL   UCSC 
 
  406
 
  9
 
 5129
 
 tss;island
 
 0.123161
 
 0.0313450
 
 Not queried
 
  1.5375897
 
 9.39e-02
 
 0.3104222821
  
 
  
 8
 
  88886301
 
  88886432
 
  PDF   STATS   ENSEMBL   UCSC 
 
  132
 
  6
 
 39801
 
 tss
 
 0.674475
 
 0.9611853
 
 Not queried
 
  1.5363966
 
 9.31e-02
 
 0.3104222821
  
 
  
 4
 
   4868723
 
   4869172
 
  PDF   STATS   ENSEMBL   UCSC 
 
  450
 
  6
 
 30874
 
 island
 
 0.041555
 
 0.0201637
 
 Not queried
 
  1.5064299
 
 9.31e-02
 
 0.3104222821
  
 
  
 22
 
  44258572
 
  44258712
 
  PDF   STATS   ENSEMBL   UCSC 
 
  141
 
  5
 
 27205
 
 tss;island
 
 0.164561
 
 0.0803602
 
 Not queried
 
  1.4841998
 
 9.52e-02
 
 0.3104222821
  
 
  
 5
 
 140810051
 
 140811312
 
  PDF   STATS   ENSEMBL   UCSC 
 
 1262
 
 16
 
 31836
 
 gene;island
 
 0.028637
 
 0.0095387
 
 Not queried
 
  1.4641808
 
 8.65e-02
 
 0.3104222821
  
 
  
 11
 
  61447487
 
  61447866
 
  PDF   STATS   ENSEMBL   UCSC 
 
  380
 
  5
 
 7336
 
 tss
 
 0.034387
 
 0.0046789
 
 Not queried
 
  1.4634985
 
 9.52e-02
 
 0.3104222821
  
 
  
 1
 
  38230769
 
  38230887
 
  PDF   STATS   ENSEMBL   UCSC 
 
  119
 
  6
 
 3073
 
 island
 
 0.030313
 
 0.1148596
 
 Not queried
 
  1.4418922
 
 9.31e-02
 
 0.3104222821
  
 
  
 3
 
 136537607
 
 136537863
 
  PDF   STATS   ENSEMBL   UCSC 
 
  257
 
  9
 
 29508
 
 island
 
 0.046786
 
 0.0155327
 
 Not queried
 
  1.4207085
 
 9.39e-02
 
 0.3104222821
  
 
  
 8
 
   1106401
 
   1106743
 
  PDF   STATS   ENSEMBL   UCSC 
 
  343
 
  5
 
 40014
 
 island
 
 0.315230
 
 0.5651591
 
 Not queried
 
  1.4119525
 
 9.52e-02
 
 0.3104222821
  
 
  
 19
 
  38754529
 
  38754930
 
  PDF   STATS   ENSEMBL   UCSC 
 
  402
 
  5
 
 20748
 
 tss
 
 0.032536
 
 0.0758652
 
 Not queried
 
  1.4093842
 
 9.52e-02
 
 0.3104222821
  
 
  
 3
 
  11058836
 
  11059050
 
  PDF   STATS   ENSEMBL   UCSC 
 
  215
 
  6
 
 27665
 
 gene;island
 
 0.312104
 
 0.5860570
 
 Not queried
 
  1.4041403
 
 9.31e-02
 
 0.3104222821
  
 
  
 12
 
  13256639
 
  13256869
 
  PDF   STATS   ENSEMBL   UCSC 
 
  231
 
  4
 
 9581
 
 tss;island
 
 0.008945
 
 0.6121293
 
 Not queried
 
  7.4517711
 
 1.14e-01
 
 0.3123457953
  
 
  
 17
 
  53800225
 
  53800554
 
  PDF   STATS   ENSEMBL   UCSC 
 
  330
 
  4
 
 17519
 
 tss
 
 0.455268
 
 0.0237989
 
 Not queried
 
  6.4703660
 
 1.14e-01
 
 0.3123457953
  
 
  
 13
 
  25506131
 
  25506383
 
  PDF   STATS   ENSEMBL   UCSC 
 
  253
 
  4
 
 11107
 
 gene;island
 
 0.781167
 
 0.1542156
 
 Not queried
 
  6.2199085
 
 1.14e-01
 
 0.3123457953
  
 
  
 16
 
   2286370
 
   2286786
 
  PDF   STATS   ENSEMBL   UCSC 
 
  417
 
  4
 
 15599
 
 island
 
 0.039669
 
 0.7518968
 
 Not queried
 
  6.1764090
 
 1.14e-01
 
 0.3123457953
  
 
  
 17
 
   8056950
 
   8057058
 
  PDF   STATS   ENSEMBL   UCSC 
 
  109
 
  4
 
 17110
 
 tss;island
 
 0.006821
 
 0.3169140
 
 Not queried
 
  6.0803359
 
 1.14e-01
 
 0.3123457953
  
 
  
 2
 
 232260116
 
 232260305
 
  PDF   STATS   ENSEMBL   UCSC 
 
  190
 
  4
 
 23939
 
 tss;island
 
 0.010783
 
 0.2456547
 
 Not queried
 
  5.6530188
 
 1.14e-01
 
 0.3123457953
  
 
  
 5
 
  54528100
 
  54528369
 
  PDF   STATS   ENSEMBL   UCSC 
 
  270
 
  4
 
 31615
 
 gene;island
 
 0.344857
 
 0.0152431
 
 Not queried
 
  5.6004650
 
 1.14e-01
 
 0.3123457953
  
 
  
 5
 
    218073
 
    218200
 
  PDF   STATS   ENSEMBL   UCSC 
 
  128
 
  4
 
 31498
 
 gene;tss;island
 
 0.120301
 
 0.0076117
 
 Not queried
 
  5.4378369
 
 1.14e-01
 
 0.3123457953
  
 
  
 2
 
 223169352
 
 223169609
 
  PDF   STATS   ENSEMBL   UCSC 
 
  258
 
  4
 
 23052
 
 gene;island
 
 0.679054
 
 0.0154962
 
 Not queried
 
  5.2398511
 
 1.14e-01
 
 0.3123457953
  
 
  
 14
 
 100625027
 
 100625253
 
  PDF   STATS   ENSEMBL   UCSC 
 
  227
 
  4
 
 12206
 
 gene;island
 
 0.132817
 
 0.7360527
 
 Not queried
 
  5.1968591
 
 1.14e-01
 
 0.3123457953
  
 
  
 6
 
  30173936
 
  30174261
 
  PDF   STATS   ENSEMBL   UCSC 
 
  326
 
  7
 
 33750
 
 gene
 
 0.038350
 
 0.5739863
 
 Not queried
 
  5.1600517
 
 9.73e-02
 
 0.3123457953
  
 
  
 11
 
 110583882
 
 110584082
 
  PDF   STATS   ENSEMBL   UCSC 
 
  201
 
  4
 
 7680
 
 tss;island
 
 0.116571
 
 0.0080829
 
 Not queried
 
  5.1332065
 
 1.14e-01
 
 0.3123457953
  
 
  
 1
 
 151689979
 
 151690075
 
  PDF   STATS   ENSEMBL   UCSC 
 
   97
 
  4
 
 1930
 
 tss
 
 0.070568
 
 0.6105439
 
 Not queried
 
  5.1261948
 
 1.14e-01
 
 0.3123457953
  
 
  
 11
 
  49579997
 
  49580074
 
  PDF   STATS   ENSEMBL   UCSC 
 
   78
 
  4
 
 7295
 
 tss
 
 0.116313
 
 0.6179595
 
 Not queried
 
  5.1121574
 
 1.14e-01
 
 0.3123457953
  
 
  
 19
 
   7735239
 
   7735510
 
  PDF   STATS   ENSEMBL   UCSC 
 
  272
 
  4
 
 21461
 
 island
 
 0.007351
 
 0.1123205
 
 Not queried
 
  4.9360556
 
 1.14e-01
 
 0.3123457953
  
 
  
 2
 
 208890341
 
 208890496
 
  PDF   STATS   ENSEMBL   UCSC 
 
  156
 
  4
 
 23845
 
 tss
 
 0.001161
 
 0.0147307
 
 Not queried
 
  4.7590829
 
 1.14e-01
 
 0.3123457953
  
 
  
 17
 
  46831306
 
  46831742
 
  PDF   STATS   ENSEMBL   UCSC 
 
  437
 
  4
 
 18508
 
 island
 
 0.330166
 
 0.0144688
 
 Not queried
 
  4.6214808
 
 1.14e-01
 
 0.3123457953
  
 
  
 10
 
  97200911
 
  97201172
 
  PDF   STATS   ENSEMBL   UCSC 
 
  262
 
  4
 
 4688
 
 gene
 
 0.354050
 
 0.8986871
 
 Not queried
 
  4.5925442
 
 1.14e-01
 
 0.3123457953
  
 
  
 14
 
 101036192
 
 101036470
 
  PDF   STATS   ENSEMBL   UCSC 
 
  279
 
  4
 
 12605
 
 tss;island
 
 0.420754
 
 0.0213335
 
 Not queried
 
  4.5699268
 
 1.14e-01
 
 0.3123457953
  
 
  
 3
 
  53190593
 
  53190763
 
  PDF   STATS   ENSEMBL   UCSC 
 
  171
 
  4
 
 29198
 
 island
 
 0.355074
 
 0.0181910
 
 Not queried
 
  4.5500490
 
 1.14e-01
 
 0.3123457953
  
 
  
 10
 
  77164987
 
  77165293
 
  PDF   STATS   ENSEMBL   UCSC 
 
  307
 
  4
 
 4644
 
 gene;island
 
 0.956553
 
 0.7227986
 
 Not queried
 
  4.5187378
 
 1.14e-01
 
 0.3123457953
  
 
  
 11
 
 118550443
 
 118550644
 
  PDF   STATS   ENSEMBL   UCSC 
 
  202
 
  4
 
 7737
 
 tss
 
 0.016582
 
 0.2279150
 
 Not queried
 
  4.4827727
 
 1.14e-01
 
 0.3123457953
  
 
  
 3
 
 193587490
 
 193587939
 
  PDF   STATS   ENSEMBL   UCSC 
 
  450
 
  4
 
 29778
 
 island
 
 0.126042
 
 0.5526444
 
 Not queried
 
  4.4493052
 
 1.14e-01
 
 0.3123457953
  
 
  
 3
 
 184279593
 
 184279728
 
  PDF   STATS   ENSEMBL   UCSC 
 
  136
 
  4
 
 28064
 
 gene
 
 0.207339
 
 0.0065175
 
 Not queried
 
  4.3679246
 
 1.14e-01
 
 0.3123457953
  
 
  
 6
 
  10398494
 
  10398754
 
  PDF   STATS   ENSEMBL   UCSC 
 
  261
 
  4
 
 33556
 
 gene;island
 
 0.112681
 
 0.0158238
 
 Not queried
 
  4.3625756
 
 1.14e-01
 
 0.3123457953
  
 
  
 20
 
  22559305
 
  22559456
 
  PDF   STATS   ENSEMBL   UCSC 
 
  152
 
  4
 
 25632
 
 tss;island
 
 0.077749
 
 0.6203961
 
 Not queried
 
  4.3550840
 
 1.14e-01
 
 0.3123457953
  
 
  
 1
 
   6265071
 
   6265417
 
  PDF   STATS   ENSEMBL   UCSC 
 
  347
 
  4
 
 1200
 
 tss;island
 
 0.405909
 
 0.0141605
 
 Not queried
 
  4.2841561
 
 1.14e-01
 
 0.3123457953
  
 
  
 2
 
 191207820
 
 191208171
 
  PDF   STATS   ENSEMBL   UCSC 
 
  352
 
  4
 
 23785
 
 tss
 
 0.490906
 
 0.0193727
 
 Not queried
 
  4.2615178
 
 1.14e-01
 
 0.3123457953
  
 
  
 3
 
 126007325
 
 126007563
 
  PDF   STATS   ENSEMBL   UCSC 
 
  239
 
  4
 
 29429
 
 island
 
 0.026836
 
 0.5248609
 
 Not queried
 
  4.2400834
 
 1.14e-01
 
 0.3123457953
  
 
  
 17
 
    202588
 
    202759
 
  PDF   STATS   ENSEMBL   UCSC 
 
  172
 
  4
 
 16978
 
 tss;island
 
 0.030016
 
 0.3091529
 
 Not queried
 
  4.2104974
 
 1.14e-01
 
 0.3123457953
  
 
  
 4
 
 113970617
 
 113970745
 
  PDF   STATS   ENSEMBL   UCSC 
 
  129
 
  4
 
 30555
 
 tss
 
 0.657963
 
 0.0342533
 
 Not queried
 
  4.1744485
 
 1.14e-01
 
 0.3123457953
  
 
  
 19
 
  13134771
 
  13134830
 
  PDF   STATS   ENSEMBL   UCSC 
 
   60
 
  4
 
 19876
 
 gene;island
 
 0.056978
 
 0.3671905
 
 Not queried
 
  4.1638323
 
 1.14e-01
 
 0.3123457953
  
 
  
 2
 
  38304720
 
  38304804
 
  PDF   STATS   ENSEMBL   UCSC 
 
   85
 
  4
 
 23310
 
 tss;island
 
 0.012217
 
 0.3227624
 
 Not queried
 
  4.1459229
 
 1.14e-01
 
 0.3123457953
  
 
  
 11
 
  64405764
 
  64406051
 
  PDF   STATS   ENSEMBL   UCSC 
 
  288
 
  4
 
 6691
 
 gene;island
 
 0.774279
 
 0.0307032
 
 Not queried
 
  4.0747224
 
 1.14e-01
 
 0.3123457953
  
 
  
 1
 
  29460817
 
  29461070
 
  PDF   STATS   ENSEMBL   UCSC 
 
  254
 
  4
 
 2959
 
 island
 
 0.868522
 
 0.4591063
 
 Not queried
 
  3.9937854
 
 1.14e-01
 
 0.3123457953
  
 
  
 19
 
   3369702
 
   3369820
 
  PDF   STATS   ENSEMBL   UCSC 
 
  119
 
  4
 
 19714
 
 gene;island
 
 0.014301
 
 0.3843609
 
 Not queried
 
  3.9647442
 
 1.14e-01
 
 0.3123457953
  
 
  
 10
 
  94825547
 
  94825848
 
  PDF   STATS   ENSEMBL   UCSC 
 
  302
 
  4
 
 4679
 
 gene;island
 
 0.117326
 
 0.0189496
 
 Not queried
 
  3.9537149
 
 1.14e-01
 
 0.3123457953
  
 
  
 5
 
   1174426
 
   1174666
 
  PDF   STATS   ENSEMBL   UCSC 
 
  241
 
  4
 
 32611
 
 island
 
 0.064733
 
 0.5128787
 
 Not queried
 
  3.9530685
 
 1.14e-01
 
 0.3123457953
  
 
  
 2
 
 220299484
 
 220299672
 
  PDF   STATS   ENSEMBL   UCSC 
 
  189
 
  7
 
 23906
 
 tss
 
 0.208188
 
 0.0410133
 
 Not queried
 
  3.9075403
 
 9.73e-02
 
 0.3123457953
  
 
  
 19
 
  48897863
 
  48898160
 
  PDF   STATS   ENSEMBL   UCSC 
 
  298
 
  4
 
 22214
 
 island
 
 0.090583
 
 0.0176275
 
 Not queried
 
  3.8788571
 
 1.14e-01
 
 0.3123457953
  
 
  
 15
 
  45028083
 
  45028270
 
  PDF   STATS   ENSEMBL   UCSC 
 
  188
 
  4
 
 13642
 
 tss
 
 0.014021
 
 0.1522310
 
 Not queried
 
  3.8068567
 
 1.14e-01
 
 0.3123457953
  
 
  
 6
 
 127796989
 
 127797286
 
  PDF   STATS   ENSEMBL   UCSC 
 
  298
 
  4
 
 34470
 
 gene;island
 
 0.523373
 
 0.8893552
 
 Not queried
 
  3.7825430
 
 1.14e-01
 
 0.3123457953
  
 
  
 17
 
  39969264
 
  39969397
 
  PDF   STATS   ENSEMBL   UCSC 
 
  134
 
  4
 
 16572
 
 gene;tss;island
 
 0.154001
 
 0.6875490
 
 Not queried
 
  3.7320693
 
 1.14e-01
 
 0.3123457953
  
 
  
 5
 
  76936361
 
  76936793
 
  PDF   STATS   ENSEMBL   UCSC 
 
  433
 
  4
 
 32903
 
 island
 
 0.345499
 
 0.0349317
 
 Not queried
 
  3.7218189
 
 1.14e-01
 
 0.3123457953
  
 
  
 19
 
  45720771
 
  45721076
 
  PDF   STATS   ENSEMBL   UCSC 
 
  306
 
  4
 
 20089
 
 gene;island
 
 0.010775
 
 0.2216448
 
 Not queried
 
  3.7128430
 
 1.14e-01
 
 0.3123457953
  
 
  
 7
 
   1083072
 
   1083473
 
  PDF   STATS   ENSEMBL   UCSC 
 
  402
 
  4
 
 36740
 
 gene;island
 
 0.940180
 
 0.6756889
 
 Not queried
 
  3.7025951
 
 1.14e-01
 
 0.3123457953
  
 
  
 3
 
 101498198
 
 101498279
 
  PDF   STATS   ENSEMBL   UCSC 
 
   82
 
  4
 
 28480
 
 tss
 
 0.007655
 
 0.0014265
 
 Not queried
 
  3.6830749
 
 1.14e-01
 
 0.3123457953
  
 
  
 2
 
 236772900
 
 236773260
 
  PDF   STATS   ENSEMBL   UCSC 
 
  361
 
  4
 
 23083
 
 gene;island
 
 0.250828
 
 0.5793988
 
 Not queried
 
  3.6574384
 
 1.14e-01
 
 0.3123457953
  
 
  
 6
 
  33134045
 
  33134325
 
  PDF   STATS   ENSEMBL   UCSC 
 
  281
 
  4
 
 34196
 
 gene
 
 0.031177
 
 0.2592363
 
 Not queried
 
  3.6147836
 
 1.14e-01
 
 0.3123457953
  
 
  
 12
 
 117797056
 
 117797385
 
  PDF   STATS   ENSEMBL   UCSC 
 
  330
 
  4
 
 9338
 
 gene;island
 
 0.030130
 
 0.4064809
 
 Not queried
 
  3.5755323
 
 1.14e-01
 
 0.3123457953
  
 
  
 1
 
  94884039
 
  94884189
 
  PDF   STATS   ENSEMBL   UCSC 
 
  151
 
  4
 
 642
 
 gene;island
 
 0.005226
 
 0.0000791
 
 Not queried
 
  3.5606566
 
 1.14e-01
 
 0.3123457953
  
 
  
 19
 
  45927563
 
  45927696
 
  PDF   STATS   ENSEMBL   UCSC 
 
  134
 
  4
 
 20871
 
 tss;island
 
 0.007057
 
 0.0768458
 
 Not queried
 
  3.5586123
 
 1.14e-01
 
 0.3123457953
  
 
  
 19
 
  58545001
 
  58545333
 
  PDF   STATS   ENSEMBL   UCSC 
 
  333
 
  8
 
 21131
 
 tss;island
 
 0.099317
 
 0.4381719
 
 Not queried
 
  3.5539831
 
 1.05e-01
 
 0.3123457953
  
 
  
 8
 
  21881998
 
  21882104
 
  PDF   STATS   ENSEMBL   UCSC 
 
  107
 
  4
 
 39578
 
 tss;island
 
 0.015063
 
 0.2540440
 
 Not queried
 
  3.5081424
 
 1.14e-01
 
 0.3123457953
  
 
  
 17
 
  78865087
 
  78865755
 
  PDF   STATS   ENSEMBL   UCSC 
 
  669
 
  7
 
 16881
 
 gene;island
 
 0.064827
 
 0.6768798
 
 Not queried
 
  3.4603924
 
 9.73e-02
 
 0.3123457953
  
 
  
 13
 
  21520203
 
  21520519
 
  PDF   STATS   ENSEMBL   UCSC 
 
  317
 
  4
 
 11582
 
 island
 
 0.301211
 
 0.0285169
 
 Not queried
 
  3.4571413
 
 1.14e-01
 
 0.3123457953
  
 
  
 11
 
 115374969
 
 115375226
 
  PDF   STATS   ENSEMBL   UCSC 
 
  258
 
  4
 
 6913
 
 gene
 
 0.094766
 
 0.5002125
 
 Not queried
 
  3.4353353
 
 1.14e-01
 
 0.3123457953
  
 
  
 21
 
  39644216
 
  39644354
 
  PDF   STATS   ENSEMBL   UCSC 
 
  139
 
  4
 
 26426
 
 gene;tss
 
 0.271289
 
 0.7190326
 
 Not queried
 
  3.4273895
 
 1.14e-01
 
 0.3123457953
  
 
  
 4
 
  40751717
 
  40751845
 
  PDF   STATS   ENSEMBL   UCSC 
 
  129
 
  4
 
 30360
 
 tss
 
 0.381311
 
 0.0460266
 
 Not queried
 
  3.4089116
 
 1.14e-01
 
 0.3123457953
  
 
  
 20
 
  61637360
 
  61637856
 
  PDF   STATS   ENSEMBL   UCSC 
 
  497
 
  4
 
 25496
 
 gene;island
 
 0.643545
 
 0.0642121
 
 Not queried
 
  3.4059850
 
 1.14e-01
 
 0.3123457953
  
 
  
 4
 
  81118188
 
  81118588
 
  PDF   STATS   ENSEMBL   UCSC 
 
  401
 
  4
 
 30033
 
 gene;tss;island
 
 0.010490
 
 0.0983330
 
 Not queried
 
  3.3758816
 
 1.14e-01
 
 0.3123457953
  
 
  
 14
 
  61188077
 
  61188431
 
  PDF   STATS   ENSEMBL   UCSC 
 
  355
 
  4
 
 12104
 
 gene;island
 
 0.008757
 
 0.3057612
 
 Not queried
 
  3.3634835
 
 1.14e-01
 
 0.3123457953
  
 
  
 8
 
 120428418
 
 120428692
 
  PDF   STATS   ENSEMBL   UCSC 
 
  275
 
  4
 
 40558
 
 island
 
 0.013354
 
 0.1398503
 
 Not queried
 
  3.3474137
 
 1.14e-01
 
 0.3123457953
  
 
  
 20
 
  11871282
 
  11871743
 
  PDF   STATS   ENSEMBL   UCSC 
 
  462
 
  7
 
 26006
 
 island
 
 0.248713
 
 0.0188459
 
 Not queried
 
  3.3258084
 
 9.73e-02
 
 0.3123457953
  
 
  
 11
 
 124747075
 
 124747263
 
  PDF   STATS   ENSEMBL   UCSC 
 
  189
 
  4
 
 6955
 
 gene;island
 
 0.672895
 
 0.3462173
 
 Not queried
 
  3.3230607
 
 1.14e-01
 
 0.3123457953
  
 
  
 19
 
  36247867
 
  36248077
 
  PDF   STATS   ENSEMBL   UCSC 
 
  211
 
  4
 
 20713
 
 tss;island
 
 0.023944
 
 0.3909136
 
 Not queried
 
  3.3176271
 
 1.14e-01
 
 0.3123457953
  
 
  
 1
 
 220864119
 
 220864321
 
  PDF   STATS   ENSEMBL   UCSC 
 
  203
 
  4
 
 1019
 
 gene;island
 
 0.136764
 
 0.0162523
 
 Not queried
 
  3.3054924
 
 1.14e-01
 
 0.3123457953
  
 
  
 11
 
  61283865
 
  61284134
 
  PDF   STATS   ENSEMBL   UCSC 
 
  270
 
  4
 
 6629
 
 gene;island
 
 0.009199
 
 0.1344169
 
 Not queried
 
  3.2970480
 
 1.14e-01
 
 0.3123457953
  
 
  
 4
 
  74719046
 
  74719289
 
  PDF   STATS   ENSEMBL   UCSC 
 
  244
 
  4
 
 30022
 
 gene
 
 0.417780
 
 0.0427439
 
 Not queried
 
  3.2900845
 
 1.14e-01
 
 0.3123457953
  
 
  
 4
 
  55095282
 
  55095529
 
  PDF   STATS   ENSEMBL   UCSC 
 
  248
 
  4
 
 30004
 
 gene
 
 0.216729
 
 0.0019075
 
 Not queried
 
  3.2893425
 
 1.14e-01
 
 0.3123457953
  
 
  
 2
 
  55450577
 
  55450874
 
  PDF   STATS   ENSEMBL   UCSC 
 
  298
 
  4
 
 22686
 
 gene;island
 
 0.273282
 
 0.0446650
 
 Not queried
 
  3.2751159
 
 1.14e-01
 
 0.3123457953
  
 
  
 2
 
 223162666
 
 223162800
 
  PDF   STATS   ENSEMBL   UCSC 
 
  135
 
  4
 
 23915
 
 tss
 
 0.349657
 
 0.0130669
 
 Not queried
 
  3.2653891
 
 1.14e-01
 
 0.3123457953
  
 
  
 3
 
 139346734
 
 139346955
 
  PDF   STATS   ENSEMBL   UCSC 
 
  222
 
  4
 
 27970
 
 gene
 
 0.124782
 
 0.4512945
 
 Not queried
 
  3.2325391
 
 1.14e-01
 
 0.3123457953
  
 
  
 17
 
    185102
 
    185263
 
  PDF   STATS   ENSEMBL   UCSC 
 
  162
 
  4
 
 16292
 
 gene;island
 
 0.282092
 
 0.7210152
 
 Not queried
 
  3.2185554
 
 1.14e-01
 
 0.3123457953
  
 
  
 6
 
  28226885
 
  28227482
 
  PDF   STATS   ENSEMBL   UCSC 
 
  598
 
 11
 
 35585
 
 island
 
 0.760262
 
 0.1261724
 
 Not queried
 
  3.2155257
 
 1.01e-01
 
 0.3123457953
  
 
  
 18
 
  12287173
 
  12287561
 
  PDF   STATS   ENSEMBL   UCSC 
 
  389
 
  4
 
 19402
 
 island
 
 0.150209
 
 0.4381731
 
 Not queried
 
  3.1979329
 
 1.14e-01
 
 0.3123457953
  
 
  
 7
 
   1783946
 
   1784169
 
  PDF   STATS   ENSEMBL   UCSC 
 
  224
 
  4
 
 36766
 
 gene;island
 
 0.445826
 
 0.8944046
 
 Not queried
 
  3.1755838
 
 1.14e-01
 
 0.3123457953
  
 
  
 5
 
 175224563
 
 175224721
 
  PDF   STATS   ENSEMBL   UCSC 
 
  159
 
  4
 
 31908
 
 gene;island
 
 0.017810
 
 0.0899433
 
 Not queried
 
  3.1272910
 
 1.14e-01
 
 0.3123457953
  
 
  
 3
 
  44626492
 
  44626860
 
  PDF   STATS   ENSEMBL   UCSC 
 
  369
 
  4
 
 27740
 
 gene
 
 0.019088
 
 0.1860929
 
 Not queried
 
  3.0866621
 
 1.14e-01
 
 0.3123457953
  
 
  
 10
 
  88149210
 
  88149632
 
  PDF   STATS   ENSEMBL   UCSC 
 
  423
 
  4
 
 5903
 
 island
 
 0.028078
 
 0.3121494
 
 Not queried
 
  3.0630372
 
 1.14e-01
 
 0.3123457953
  
 
  
 20
 
  51589616
 
  51589985
 
  PDF   STATS   ENSEMBL   UCSC 
 
  370
 
  4
 
 25457
 
 gene;island
 
 0.025588
 
 0.0997219
 
 Not queried
 
  3.0510306
 
 1.14e-01
 
 0.3123457953
  
 
  
 17
 
  79495268
 
  79495664
 
  PDF   STATS   ENSEMBL   UCSC 
 
  397
 
  7
 
 18927
 
 island
 
 0.881945
 
 0.5287471
 
 Not queried
 
  3.0361396
 
 9.73e-02
 
 0.3123457953
  
 
  
 2
 
  88427581
 
  88427827
 
  PDF   STATS   ENSEMBL   UCSC 
 
  247
 
  4
 
 23499
 
 tss
 
 0.150518
 
 0.4325552
 
 Not queried
 
  3.0294267
 
 1.14e-01
 
 0.3123457953
  
 
  
 17
 
  73084263
 
  73084494
 
  PDF   STATS   ENSEMBL   UCSC 
 
  232
 
  4
 
 16764
 
 gene;island
 
 0.057081
 
 0.3457477
 
 Not queried
 
  3.0201976
 
 1.14e-01
 
 0.3123457953
  
 
  
 5
 
 172661803
 
 172662130
 
  PDF   STATS   ENSEMBL   UCSC 
 
  328
 
  4
 
 31904
 
 gene
 
 0.042999
 
 0.0104252
 
 Not queried
 
  3.0127401
 
 1.14e-01
 
 0.3123457953
  
 
  
 8
 
 103750821
 
 103751006
 
  PDF   STATS   ENSEMBL   UCSC 
 
  186
 
  4
 
 40513
 
 island
 
 0.560228
 
 0.1280501
 
 Not queried
 
  2.9955805
 
 1.14e-01
 
 0.3123457953
  
 
  
 13
 
  20805094
 
  20805679
 
  PDF   STATS   ENSEMBL   UCSC 
 
  586
 
 10
 
 11096
 
 gene;island
 
 0.761518
 
 0.4580431
 
 Not queried
 
  2.9442779
 
 1.05e-01
 
 0.3123457953
  
 
  
 11
 
   1463541
 
   1463662
 
  PDF   STATS   ENSEMBL   UCSC 
 
  122
 
  4
 
 6419
 
 gene;island
 
 0.018674
 
 0.2592572
 
 Not queried
 
  2.9287810
 
 1.14e-01
 
 0.3123457953
  
 
  
 12
 
  71314587
 
  71314752
 
  PDF   STATS   ENSEMBL   UCSC 
 
  166
 
  4
 
 9869
 
 tss
 
 0.017652
 
 0.0912462
 
 Not queried
 
  2.9241069
 
 1.14e-01
 
 0.3123457953
  
 
  
 1
 
 243646235
 
 243646402
 
  PDF   STATS   ENSEMBL   UCSC 
 
  168
 
  4
 
 1112
 
 gene;island
 
 0.061032
 
 0.4198476
 
 Not queried
 
  2.9220316
 
 1.14e-01
 
 0.3123457953
  
 
  
 4
 
  77341841
 
  77342104
 
  PDF   STATS   ENSEMBL   UCSC 
 
  264
 
  4
 
 31117
 
 island
 
 0.518399
 
 0.9023114
 
 Not queried
 
  2.9212089
 
 1.14e-01
 
 0.3123457953
  
 
  
 12
 
  81110749
 
  81111012
 
  PDF   STATS   ENSEMBL   UCSC 
 
  264
 
  4
 
 9226
 
 gene
 
 0.148809
 
 0.6113221
 
 Not queried
 
  2.9104541
 
 1.14e-01
 
 0.3123457953
  
 
  
 10
 
    652260
 
    652597
 
  PDF   STATS   ENSEMBL   UCSC 
 
  338
 
  4
 
 4445
 
 gene;island
 
 0.160202
 
 0.6129444
 
 Not queried
 
  2.9082896
 
 1.14e-01
 
 0.3123457953
  
 
  
 7
 
   6655711
 
   6655973
 
  PDF   STATS   ENSEMBL   UCSC 
 
  263
 
  4
 
 36887
 
 gene;island
 
 0.014053
 
 0.2923783
 
 Not queried
 
  2.9069999
 
 1.14e-01
 
 0.3123457953
  
 
  
 10
 
 118900324
 
 118900492
 
  PDF   STATS   ENSEMBL   UCSC 
 
  169
 
  4
 
 6173
 
 island
 
 0.042582
 
 0.1807114
 
 Not queried
 
  2.8958247
 
 1.14e-01
 
 0.3123457953
  
 
  
 11
 
   1706894
 
   1707163
 
  PDF   STATS   ENSEMBL   UCSC 
 
  270
 
  4
 
 6428
 
 gene;tss
 
 0.070491
 
 0.3407529
 
 Not queried
 
  2.8831970
 
 1.14e-01
 
 0.3123457953
  
 
  
 12
 
   7167584
 
   7167900
 
  PDF   STATS   ENSEMBL   UCSC 
 
  317
 
  4
 
 9543
 
 tss
 
 0.262939
 
 0.7941673
 
 Not queried
 
  2.8724542
 
 1.14e-01
 
 0.3123457953
  
 
  
 6
 
 153452724
 
 153452954
 
  PDF   STATS   ENSEMBL   UCSC 
 
  231
 
  4
 
 35310
 
 tss;island
 
 0.388419
 
 0.0516849
 
 Not queried
 
  2.8518279
 
 1.14e-01
 
 0.3123457953
  
 
  
 2
 
 242716388
 
 242716801
 
  PDF   STATS   ENSEMBL   UCSC 
 
  414
 
  4
 
 23145
 
 gene
 
 0.438128
 
 0.8711536
 
 Not queried
 
  2.8392809
 
 1.14e-01
 
 0.3123457953
  
 
  
 16
 
   1146277
 
   1146545
 
  PDF   STATS   ENSEMBL   UCSC 
 
  269
 
  4
 
 14988
 
 tss
 
 0.512512
 
 0.8782966
 
 Not queried
 
  2.8349818
 
 1.14e-01
 
 0.3123457953
  
 
  
 19
 
   4531638
 
   4531961
 
  PDF   STATS   ENSEMBL   UCSC 
 
  324
 
  4
 
 19735
 
 gene;island
 
 0.413688
 
 0.0506504
 
 Not queried
 
  2.8261973
 
 1.14e-01
 
 0.3123457953
  
 
  
 1
 
  18956762
 
  18957023
 
  PDF   STATS   ENSEMBL   UCSC 
 
  262
 
  4
 
 1295
 
 tss;island
 
 0.030912
 
 0.1469318
 
 Not queried
 
  2.8104438
 
 1.14e-01
 
 0.3123457953
  
 
  
 7
 
 103630013
 
 103630093
 
  PDF   STATS   ENSEMBL   UCSC 
 
   81
 
  4
 
 37813
 
 tss;island
 
 0.011619
 
 0.1414593
 
 Not queried
 
  2.8021109
 
 1.14e-01
 
 0.3123457953
  
 
  
 4
 
 155663689
 
 155664311
 
  PDF   STATS   ENSEMBL   UCSC 
 
  623
 
  7
 
 30646
 
 tss
 
 0.038651
 
 0.1386183
 
 Not queried
 
  2.7931415
 
 9.73e-02
 
 0.3123457953
  
 
  
 19
 
  41698814
 
  41699065
 
  PDF   STATS   ENSEMBL   UCSC 
 
  252
 
  9
 
 20802
 
 tss;island
 
 0.060469
 
 0.3086603
 
 Not queried
 
  2.7931127
 
 1.13e-01
 
 0.3123457953
  
 
  
 20
 
   2731102
 
   2731560
 
  PDF   STATS   ENSEMBL   UCSC 
 
  459
 
  4
 
 25337
 
 gene;island
 
 0.038043
 
 0.2859799
 
 Not queried
 
  2.7855834
 
 1.14e-01
 
 0.3123457953
  
 
  
 10
 
  38691306
 
  38691563
 
  PDF   STATS   ENSEMBL   UCSC 
 
  258
 
  4
 
 4561
 
 gene;island
 
 0.335967
 
 0.0346915
 
 Not queried
 
  2.7432267
 
 1.14e-01
 
 0.3123457953
  
 
  
 19
 
  54850444
 
  54850645
 
  PDF   STATS   ENSEMBL   UCSC 
 
  202
 
  4
 
 21059
 
 tss
 
 0.672483
 
 0.2119643
 
 Not queried
 
  2.7091715
 
 1.14e-01
 
 0.3123457953
  
 
  
 2
 
  38300537
 
  38300885
 
  PDF   STATS   ENSEMBL   UCSC 
 
  349
 
  4
 
 22650
 
 gene;island
 
 0.945788
 
 0.3753242
 
 Not queried
 
  2.7037638
 
 1.14e-01
 
 0.3123457953
  
 
  
 2
 
  75147006
 
  75147288
 
  PDF   STATS   ENSEMBL   UCSC 
 
  283
 
  4
 
 24453
 
 island
 
 0.158684
 
 0.0252248
 
 Not queried
 
  2.6832386
 
 1.14e-01
 
 0.3123457953
  
 
  
 2
 
   3699195
 
   3699353
 
  PDF   STATS   ENSEMBL   UCSC 
 
  159
 
  4
 
 24039
 
 island
 
 0.190095
 
 0.0393927
 
 Not queried
 
  2.6739825
 
 1.14e-01
 
 0.3123457953
  
 
  
 4
 
    780856
 
    781272
 
  PDF   STATS   ENSEMBL   UCSC 
 
  417
 
  4
 
 29838
 
 gene;island
 
 0.089342
 
 0.4721470
 
 Not queried
 
  2.6596922
 
 1.14e-01
 
 0.3123457953
  
 
  
 16
 
   3079708
 
   3079953
 
  PDF   STATS   ENSEMBL   UCSC 
 
  246
 
  4
 
 14622
 
 gene;island
 
 0.022235
 
 0.1763880
 
 Not queried
 
  2.6389496
 
 1.14e-01
 
 0.3123457953
  
 
  
 19
 
  18496756
 
  18496953
 
  PDF   STATS   ENSEMBL   UCSC 
 
  198
 
  4
 
 20618
 
 tss
 
 0.259132
 
 0.0287304
 
 Not queried
 
  2.6327736
 
 1.14e-01
 
 0.3123457953
  
 
  
 5
 
 180486604
 
 180486879
 
  PDF   STATS   ENSEMBL   UCSC 
 
  276
 
  4
 
 31949
 
 gene;island
 
 0.303824
 
 0.0626534
 
 Not queried
 
  2.6263736
 
 1.14e-01
 
 0.3123457953
  
 
  
 11
 
  63828569
 
  63828762
 
  PDF   STATS   ENSEMBL   UCSC 
 
  194
 
  4
 
 6661
 
 gene
 
 0.011875
 
 0.0894900
 
 Not queried
 
  2.6207374
 
 1.14e-01
 
 0.3123457953
  
 
  
 7
 
  50800426
 
  50800620
 
  PDF   STATS   ENSEMBL   UCSC 
 
  195
 
  4
 
 37013
 
 gene;tss
 
 0.249887
 
 0.6573329
 
 Not queried
 
  2.6179652
 
 1.14e-01
 
 0.3123457953
  
 
  
 10
 
 125853676
 
 125853893
 
  PDF   STATS   ENSEMBL   UCSC 
 
  218
 
  4
 
 6225
 
 island
 
 0.094549
 
 0.3784362
 
 Not queried
 
  2.6173179
 
 1.14e-01
 
 0.3123457953
  
 
  
 6
 
 149285724
 
 149285963
 
  PDF   STATS   ENSEMBL   UCSC 
 
  240
 
  4
 
 34503
 
 gene
 
 0.944393
 
 0.6258820
 
 Not queried
 
  2.5967444
 
 1.14e-01
 
 0.3123457953
  
 
  
 19
 
  11530021
 
  11530065
 
  PDF   STATS   ENSEMBL   UCSC 
 
   45
 
  4
 
 20471
 
 tss
 
 0.005982
 
 0.0409693
 
 Not queried
 
  2.5883836
 
 1.14e-01
 
 0.3123457953
  
 
  
 11
 
    320732
 
    320889
 
  PDF   STATS   ENSEMBL   UCSC 
 
  158
 
  4
 
 6370
 
 gene
 
 0.050066
 
 0.3162403
 
 Not queried
 
  2.5707119
 
 1.14e-01
 
 0.3123457953
  
 
  
 1
 
   3100143
 
   3100497
 
  PDF   STATS   ENSEMBL   UCSC 
 
  355
 
  4
 
 106
 
 gene;island
 
 0.073123
 
 0.5601795
 
 Not queried
 
  2.5653144
 
 1.14e-01
 
 0.3123457953
  
 
  
 11
 
    320732
 
    320956
 
  PDF   STATS   ENSEMBL   UCSC 
 
  225
 
  7
 
 7842
 
 island
 
 0.028990
 
 0.2756238
 
 Not queried
 
  2.5579050
 
 9.73e-02
 
 0.3123457953
  
 
  
 13
 
  40746370
 
  40746648
 
  PDF   STATS   ENSEMBL   UCSC 
 
  279
 
  4
 
 11685
 
 island
 
 0.386967
 
 0.8056389
 
 Not queried
 
  2.5482323
 
 1.14e-01
 
 0.3123457953
  
 
  
 16
 
  67427183
 
  67427391
 
  PDF   STATS   ENSEMBL   UCSC 
 
  209
 
  4
 
 14798
 
 gene;island
 
 0.064904
 
 0.3693196
 
 Not queried
 
  2.5321764
 
 1.14e-01
 
 0.3123457953
  
 
  
 7
 
   1018384
 
   1018603
 
  PDF   STATS   ENSEMBL   UCSC 
 
  220
 
  4
 
 38062
 
 island
 
 0.787156
 
 0.9650794
 
 Not queried
 
  2.5206036
 
 1.14e-01
 
 0.3123457953
  
 
  
 8
 
   1792023
 
   1792249
 
  PDF   STATS   ENSEMBL   UCSC 
 
  227
 
  4
 
 39178
 
 gene
 
 0.441138
 
 0.8302538
 
 Not queried
 
  2.5044162
 
 1.14e-01
 
 0.3123457953
  
 
  
 7
 
 157414294
 
 157414580
 
  PDF   STATS   ENSEMBL   UCSC 
 
  287
 
  4
 
 37333
 
 gene;island
 
 0.789301
 
 0.9586676
 
 Not queried
 
  2.4935892
 
 1.14e-01
 
 0.3123457953
  
 
  
 22
 
  17602561
 
  17602681
 
  PDF   STATS   ENSEMBL   UCSC 
 
  121
 
  4
 
 26941
 
 tss;island
 
 0.044335
 
 0.2601731
 
 Not queried
 
  2.4732281
 
 1.14e-01
 
 0.3123457953
  
 
  
 1
 
  89357908
 
  89358043
 
  PDF   STATS   ENSEMBL   UCSC 
 
  136
 
  4
 
 1737
 
 tss;island
 
 0.878085
 
 0.9789418
 
 Not queried
 
  2.4726036
 
 1.14e-01
 
 0.3123457953
  
 
  
 20
 
  52789956
 
  52790141
 
  PDF   STATS   ENSEMBL   UCSC 
 
  186
 
  4
 
 25458
 
 gene;island
 
 0.090433
 
 0.0126912
 
 Not queried
 
  2.4259165
 
 1.14e-01
 
 0.3123457953
  
 
  
 10
 
  92631135
 
  92631706
 
  PDF   STATS   ENSEMBL   UCSC 
 
  572
 
 12
 
 5219
 
 tss
 
 0.010630
 
 0.0026497
 
 Not queried
 
  2.3983566
 
 1.14e-01
 
 0.3123457953
  
 
  
 20
 
  43160245
 
  43160301
 
  PDF   STATS   ENSEMBL   UCSC 
 
   57
 
  4
 
 25762
 
 tss;island
 
 0.011777
 
 0.0866389
 
 Not queried
 
  2.3976835
 
 1.14e-01
 
 0.3123457953
  
 
  
 11
 
  62314820
 
  62315119
 
  PDF   STATS   ENSEMBL   UCSC 
 
  300
 
  4
 
 7345
 
 tss;island
 
 0.420437
 
 0.6351212
 
 Not queried
 
  2.3908547
 
 1.14e-01
 
 0.3123457953
  
 
  
 4
 
  55524145
 
  55524400
 
  PDF   STATS   ENSEMBL   UCSC 
 
  256
 
  4
 
 30005
 
 gene;island
 
 0.014639
 
 0.0619903
 
 Not queried
 
  2.3908137
 
 1.14e-01
 
 0.3123457953
  
 
  
 17
 
   7342585
 
   7342894
 
  PDF   STATS   ENSEMBL   UCSC 
 
  310
 
  4
 
 17949
 
 island
 
 0.046318
 
 0.3328364
 
 Not queried
 
  2.3643206
 
 1.14e-01
 
 0.3123457953
  
 
  
 2
 
 242497960
 
 242498098
 
  PDF   STATS   ENSEMBL   UCSC 
 
  139
 
  4
 
 23991
 
 tss;island
 
 0.183523
 
 0.4949677
 
 Not queried
 
  2.3509309
 
 1.14e-01
 
 0.3123457953
  
 
  
 19
 
  13616871
 
  13617094
 
  PDF   STATS   ENSEMBL   UCSC 
 
  224
 
  4
 
 19882
 
 gene;island
 
 0.155332
 
 0.0282120
 
 Not queried
 
  2.3478221
 
 1.14e-01
 
 0.3123457953
  
 
  
 15
 
  65368982
 
  65369137
 
  PDF   STATS   ENSEMBL   UCSC 
 
  156
 
  4
 
 13738
 
 tss
 
 0.777180
 
 0.6133794
 
 Not queried
 
  2.3321934
 
 1.14e-01
 
 0.3123457953
  
 
  
 2
 
 131594925
 
 131595321
 
  PDF   STATS   ENSEMBL   UCSC 
 
  397
 
  4
 
 24724
 
 island
 
 0.170714
 
 0.0410445
 
 Not queried
 
  2.3036627
 
 1.14e-01
 
 0.3123457953
  
 
  
 6
 
  29580340
 
  29580367
 
  PDF   STATS   ENSEMBL   UCSC 
 
   28
 
  4
 
 33671
 
 gene
 
 0.674273
 
 0.9120232
 
 Not queried
 
  2.3023786
 
 1.14e-01
 
 0.3123457953
  
 
  
 12
 
  51236582
 
  51236693
 
  PDF   STATS   ENSEMBL   UCSC 
 
  112
 
  4
 
 9708
 
 tss
 
 0.510146
 
 0.8258437
 
 Not queried
 
  2.2939295
 
 1.14e-01
 
 0.3123457953
  
 
  
 17
 
  42081810
 
  42082036
 
  PDF   STATS   ENSEMBL   UCSC 
 
  227
 
  8
 
 17397
 
 tss;island
 
 0.166181
 
 0.0580383
 
 Not queried
 
  2.2911446
 
 1.05e-01
 
 0.3123457953
  
 
  
 19
 
  12167119
 
  12167313
 
  PDF   STATS   ENSEMBL   UCSC 
 
  195
 
  4
 
 21590
 
 island
 
 0.660318
 
 0.2585724
 
 Not queried
 
  2.2812418
 
 1.14e-01
 
 0.3123457953
  
 
  
 4
 
 174443242
 
 174443567
 
  PDF   STATS   ENSEMBL   UCSC 
 
  326
 
  4
 
 31427
 
 island
 
 0.120550
 
 0.3213410
 
 Not queried
 
  2.2562827
 
 1.14e-01
 
 0.3123457953
  
 
  
 7
 
 100493710
 
 100493849
 
  PDF   STATS   ENSEMBL   UCSC 
 
  140
 
  4
 
 37795
 
 tss
 
 0.004864
 
 0.0301463
 
 Not queried
 
  2.2550003
 
 1.14e-01
 
 0.3123457953
  
 
  
 16
 
    979898
 
    980146
 
  PDF   STATS   ENSEMBL   UCSC 
 
  249
 
  4
 
 14555
 
 gene;island
 
 0.633402
 
 0.9114010
 
 Not queried
 
  2.2361926
 
 1.14e-01
 
 0.3123457953
  
 
  
 9
 
  99382084
 
  99382235
 
  PDF   STATS   ENSEMBL   UCSC 
 
  152
 
  4
 
 40917
 
 island
 
 0.012614
 
 0.0267356
 
 Not queried
 
  2.2275760
 
 1.14e-01
 
 0.3123457953
  
 
  
 16
 
    959749
 
    960144
 
  PDF   STATS   ENSEMBL   UCSC 
 
  396
 
  4
 
 14550
 
 gene;island
 
 0.817712
 
 0.9584398
 
 Not queried
 
  2.2249031
 
 1.14e-01
 
 0.3123457953
  
 
  
 11
 
  66045351
 
  66045718
 
  PDF   STATS   ENSEMBL   UCSC 
 
  368
 
  9
 
 8501
 
 island
 
 0.003566
 
 0.0054310
 
 Not queried
 
  2.2138681
 
 1.13e-01
 
 0.3123457953
  
 
  
 18
 
  48085671
 
  48085844
 
  PDF   STATS   ENSEMBL   UCSC 
 
  174
 
  4
 
 19275
 
 tss;island
 
 0.028170
 
 0.1581170
 
 Not queried
 
  2.2064182
 
 1.14e-01
 
 0.3123457953
  
 
  
 22
 
  19701734
 
  19701871
 
  PDF   STATS   ENSEMBL   UCSC 
 
  138
 
  4
 
 26963
 
 tss;island
 
 0.021544
 
 0.0058340
 
 Not queried
 
  2.1840825
 
 1.14e-01
 
 0.3123457953
  
 
  
 19
 
  35329860
 
  35330243
 
  PDF   STATS   ENSEMBL   UCSC 
 
  384
 
  4
 
 21892
 
 island
 
 0.162774
 
 0.4734748
 
 Not queried
 
  2.1820582
 
 1.14e-01
 
 0.3123457953
  
 
  
 1
 
   8085604
 
   8085795
 
  PDF   STATS   ENSEMBL   UCSC 
 
  192
 
  4
 
 192
 
 gene;island
 
 0.142693
 
 0.0182648
 
 Not queried
 
  2.1784183
 
 1.14e-01
 
 0.3123457953
  
 
  
 16
 
  68270252
 
  68270387
 
  PDF   STATS   ENSEMBL   UCSC 
 
  136
 
  4
 
 15319
 
 tss
 
 0.043555
 
 0.1525232
 
 Not queried
 
  2.1722076
 
 1.14e-01
 
 0.3123457953
  
 
  
 2
 
 164593221
 
 164593498
 
  PDF   STATS   ENSEMBL   UCSC 
 
  278
 
  4
 
 23691
 
 tss;island
 
 0.032324
 
 0.1757761
 
 Not queried
 
  2.1672467
 
 1.14e-01
 
 0.3123457953
  
 
  
 15
 
  53097649
 
  53097840
 
  PDF   STATS   ENSEMBL   UCSC 
 
  192
 
  4
 
 14154
 
 island
 
 0.020573
 
 0.1154236
 
 Not queried
 
  2.1563704
 
 1.14e-01
 
 0.3123457953
  
 
  
 8
 
  41519026
 
  41519399
 
  PDF   STATS   ENSEMBL   UCSC 
 
  374
 
  4
 
 39290
 
 gene;tss
 
 0.900895
 
 0.9607751
 
 Not queried
 
  2.1496033
 
 1.14e-01
 
 0.3123457953
  
 
  
 1
 
 112531263
 
 112531653
 
  PDF   STATS   ENSEMBL   UCSC 
 
  391
 
  4
 
 689
 
 gene
 
 0.022813
 
 0.3138943
 
 Not queried
 
  2.1482435
 
 1.14e-01
 
 0.3123457953
  
 
  
 16
 
  86600309
 
  86600499
 
  PDF   STATS   ENSEMBL   UCSC 
 
  191
 
  4
 
 15404
 
 tss;island
 
 0.022933
 
 0.0776261
 
 Not queried
 
  2.1446293
 
 1.14e-01
 
 0.3123457953
  
 
  
 10
 
  50604330
 
  50604569
 
  PDF   STATS   ENSEMBL   UCSC 
 
  240
 
  4
 
 5733
 
 island
 
 0.012674
 
 0.0541104
 
 Not queried
 
  2.1375899
 
 1.14e-01
 
 0.3123457953
  
 
  
 14
 
  37641134
 
  37641726
 
  PDF   STATS   ENSEMBL   UCSC 
 
  593
 
  8
 
 12069
 
 gene;island
 
 0.118342
 
 0.2721263
 
 Not queried
 
  2.1237394
 
 1.05e-01
 
 0.3123457953
  
 
  
 12
 
 132865182
 
 132865474
 
  PDF   STATS   ENSEMBL   UCSC 
 
  293
 
  4
 
 9445
 
 gene;island
 
 0.471402
 
 0.7936891
 
 Not queried
 
  2.1015057
 
 1.14e-01
 
 0.3123457953
  
 
  
 11
 
   1774722
 
   1775039
 
  PDF   STATS   ENSEMBL   UCSC 
 
  318
 
  4
 
 6431
 
 gene;island
 
 0.676555
 
 0.9147124
 
 Not queried
 
  2.0895999
 
 1.14e-01
 
 0.3123457953
  
 
  
 19
 
  19322527
 
  19322902
 
  PDF   STATS   ENSEMBL   UCSC 
 
  376
 
  8
 
 21808
 
 island
 
 0.008900
 
 0.1008369
 
 Not queried
 
  2.0892394
 
 1.05e-01
 
 0.3123457953
  
 
  
 16
 
  88747591
 
  88747919
 
  PDF   STATS   ENSEMBL   UCSC 
 
  329
 
  4
 
 14911
 
 gene;island
 
 0.464832
 
 0.1795408
 
 Not queried
 
  2.0338503
 
 1.14e-01
 
 0.3123457953
  
 
  
 2
 
 176981919
 
 176982342
 
  PDF   STATS   ENSEMBL   UCSC 
 
  424
 
  4
 
 22936
 
 gene;island
 
 0.071679
 
 0.3791765
 
 Not queried
 
  2.0329923
 
 1.14e-01
 
 0.3123457953
  
 
  
 6
 
 169951905
 
 169952299
 
  PDF   STATS   ENSEMBL   UCSC 
 
  395
 
  4
 
 34569
 
 gene
 
 0.942031
 
 0.9821104
 
 Not queried
 
  2.0197873
 
 1.14e-01
 
 0.3123457953
  
 
  
 5
 
   1093580
 
   1093797
 
  PDF   STATS   ENSEMBL   UCSC 
 
  218
 
  4
 
 31523
 
 gene;island
 
 0.963393
 
 0.8794946
 
 Not queried
 
  2.0139992
 
 1.14e-01
 
 0.3123457953
  
 
  
 5
 
   1342124
 
   1342200
 
  PDF   STATS   ENSEMBL   UCSC 
 
   77
 
  4
 
 31535
 
 gene;island
 
 0.059199
 
 0.2345177
 
 Not queried
 
  2.0077083
 
 1.14e-01
 
 0.3123457953
  
 
  
 19
 
    407758
 
    408233
 
  PDF   STATS   ENSEMBL   UCSC 
 
  476
 
  4
 
 19625
 
 gene;island
 
 0.617548
 
 0.9301426
 
 Not queried
 
  1.9956779
 
 1.14e-01
 
 0.3123457953
  
 
  
 1
 
  22919873
 
  22920169
 
  PDF   STATS   ENSEMBL   UCSC 
 
  297
 
  4
 
 307
 
 gene;island
 
 0.141827
 
 0.6711594
 
 Not queried
 
  1.9934751
 
 1.14e-01
 
 0.3123457953
  
 
  
 10
 
  15130387
 
  15130767
 
  PDF   STATS   ENSEMBL   UCSC 
 
  381
 
  4
 
 4511
 
 gene
 
 0.048964
 
 0.0280726
 
 Not queried
 
  1.9799541
 
 1.14e-01
 
 0.3123457953
  
 
  
 18
 
  44259787
 
  44260091
 
  PDF   STATS   ENSEMBL   UCSC 
 
  305
 
  4
 
 19104
 
 gene;island
 
 0.688430
 
 0.9017504
 
 Not queried
 
  1.9781798
 
 1.14e-01
 
 0.3123457953
  
 
  
 19
 
  38210837
 
  38210932
 
  PDF   STATS   ENSEMBL   UCSC 
 
   96
 
  4
 
 20744
 
 tss
 
 0.032341
 
 0.0107201
 
 Not queried
 
  1.9633383
 
 1.14e-01
 
 0.3123457953
  
 
  
 14
 
 103739856
 
 103740085
 
  PDF   STATS   ENSEMBL   UCSC 
 
  230
 
  4
 
 13185
 
 island
 
 0.038005
 
 0.2042497
 
 Not queried
 
  1.9616949
 
 1.14e-01
 
 0.3123457953
  
 
  
 10
 
 100991959
 
 100992222
 
  PDF   STATS   ENSEMBL   UCSC 
 
  264
 
  4
 
 4705
 
 gene;island
 
 0.175816
 
 0.0444393
 
 Not queried
 
  1.9353488
 
 1.14e-01
 
 0.3123457953
  
 
  
 17
 
  62658750
 
  62658919
 
  PDF   STATS   ENSEMBL   UCSC 
 
  170
 
  4
 
 17585
 
 tss;island
 
 0.055250
 
 0.0141636
 
 Not queried
 
  1.9333201
 
 1.14e-01
 
 0.3123457953
  
 
  
 6
 
 166907748
 
 166908071
 
  PDF   STATS   ENSEMBL   UCSC 
 
  324
 
  4
 
 34547
 
 gene
 
 0.349993
 
 0.6439449
 
 Not queried
 
  1.9309174
 
 1.14e-01
 
 0.3123457953
  
 
  
 1
 
   2984218
 
   2984275
 
  PDF   STATS   ENSEMBL   UCSC 
 
   58
 
  4
 
 96
 
 gene
 
 0.235662
 
 0.6433476
 
 Not queried
 
  1.9225145
 
 1.14e-01
 
 0.3123457953
  
 
  
 20
 
  53092781
 
  53093152
 
  PDF   STATS   ENSEMBL   UCSC 
 
  372
 
  4
 
 25459
 
 gene;island
 
 0.053121
 
 0.0114995
 
 Not queried
 
  1.9140550
 
 1.14e-01
 
 0.3123457953
  
 
  
 17
 
  48172598
 
  48172668
 
  PDF   STATS   ENSEMBL   UCSC 
 
   71
 
  4
 
 17492
 
 tss;island
 
 0.005535
 
 0.0027489
 
 Not queried
 
  1.9052323
 
 1.14e-01
 
 0.3123457953
  
 
  
 2
 
  73429084
 
  73429539
 
  PDF   STATS   ENSEMBL   UCSC 
 
  456
 
  8
 
 24412
 
 island
 
 0.124353
 
 0.0358634
 
 Not queried
 
  1.8992970
 
 1.05e-01
 
 0.3123457953
  
 
  
 8
 
  24771273
 
  24771645
 
  PDF   STATS   ENSEMBL   UCSC 
 
  373
 
  4
 
 39245
 
 gene
 
 0.354380
 
 0.0960490
 
 Not queried
 
  1.8970073
 
 1.14e-01
 
 0.3123457953
  
 
  
 1
 
 214557726
 
 214557928
 
  PDF   STATS   ENSEMBL   UCSC 
 
  203
 
  4
 
 1007
 
 gene;island
 
 0.831901
 
 0.9728915
 
 Not queried
 
  1.8818916
 
 1.14e-01
 
 0.3123457953
  
 
  
 22
 
  17083412
 
  17083727
 
  PDF   STATS   ENSEMBL   UCSC 
 
  316
 
  4
 
 26778
 
 gene;island
 
 0.042168
 
 0.1337405
 
 Not queried
 
  1.8678144
 
 1.14e-01
 
 0.3123457953
  
 
  
 10
 
  28034669
 
  28034856
 
  PDF   STATS   ENSEMBL   UCSC 
 
  188
 
  7
 
 5634
 
 island
 
 0.109395
 
 0.3444235
 
 Not queried
 
  1.8567066
 
 9.73e-02
 
 0.3123457953
  
 
  
 6
 
  33140660
 
  33141305
 
  PDF   STATS   ENSEMBL   UCSC 
 
  646
 
  9
 
 34201
 
 gene
 
 0.021133
 
 0.3734163
 
 Not queried
 
  1.8427386
 
 1.13e-01
 
 0.3123457953
  
 
  
 17
 
  42091713
 
  42091909
 
  PDF   STATS   ENSEMBL   UCSC 
 
  197
 
  4
 
 16603
 
 gene;island
 
 0.237335
 
 0.0087139
 
 Not queried
 
  1.8373878
 
 1.14e-01
 
 0.3123457953
  
 
  
 6
 
  30883762
 
  30884124
 
  PDF   STATS   ENSEMBL   UCSC 
 
  363
 
  4
 
 33839
 
 gene;island
 
 0.673190
 
 0.9228009
 
 Not queried
 
  1.8205573
 
 1.14e-01
 
 0.3123457953
  
 
  
 17
 
  70120182
 
  70120541
 
  PDF   STATS   ENSEMBL   UCSC 
 
  360
 
  4
 
 16744
 
 gene;island
 
 0.670321
 
 0.8574427
 
 Not queried
 
  1.8203281
 
 1.14e-01
 
 0.3123457953
  
 
  
 13
 
  20735236
 
  20735532
 
  PDF   STATS   ENSEMBL   UCSC 
 
  297
 
  7
 
 11327
 
 tss
 
 0.004664
 
 0.0018097
 
 Not queried
 
  1.8080290
 
 9.73e-02
 
 0.3123457953
  
 
  
 11
 
  43601969
 
  43602179
 
  PDF   STATS   ENSEMBL   UCSC 
 
  211
 
  4
 
 7253
 
 tss;island
 
 0.026131
 
 0.0765721
 
 Not queried
 
  1.7946997
 
 1.14e-01
 
 0.3123457953
  
 
  
 14
 
  75389066
 
  75389280
 
  PDF   STATS   ENSEMBL   UCSC 
 
  215
 
  4
 
 13009
 
 island
 
 0.743246
 
 0.9406826
 
 Not queried
 
  1.7839679
 
 1.14e-01
 
 0.3123457953
  
 
  
 22
 
  50493126
 
  50493212
 
  PDF   STATS   ENSEMBL   UCSC 
 
   87
 
  4
 
 27243
 
 tss
 
 0.731736
 
 0.9456839
 
 Not queried
 
  1.7828923
 
 1.14e-01
 
 0.3123457953
  
 
  
 14
 
 106938234
 
 106938451
 
  PDF   STATS   ENSEMBL   UCSC 
 
  218
 
  7
 
 12676
 
 tss
 
 0.942402
 
 0.9729162
 
 Not queried
 
  1.7678248
 
 9.73e-02
 
 0.3123457953
  
 
  
 11
 
 108408950
 
 108408981
 
  PDF   STATS   ENSEMBL   UCSC 
 
   32
 
  4
 
 7673
 
 tss
 
 0.983349
 
 0.9236018
 
 Not queried
 
  1.7626667
 
 1.14e-01
 
 0.3123457953
  
 
  
 12
 
  31744043
 
  31744436
 
  PDF   STATS   ENSEMBL   UCSC 
 
  394
 
  4
 
 9627
 
 tss
 
 0.009055
 
 0.0178849
 
 Not queried
 
  1.7611629
 
 1.14e-01
 
 0.3123457953
  
 
  
 16
 
    963814
 
    963994
 
  PDF   STATS   ENSEMBL   UCSC 
 
  181
 
  4
 
 14552
 
 gene;island
 
 0.579272
 
 0.8196708
 
 Not queried
 
  1.7444972
 
 1.14e-01
 
 0.3123457953
  
 
  
 18
 
  76552701
 
  76552971
 
  PDF   STATS   ENSEMBL   UCSC 
 
  271
 
  4
 
 19592
 
 island
 
 0.675918
 
 0.9084694
 
 Not queried
 
  1.7425530
 
 1.14e-01
 
 0.3123457953
  
 
  
 3
 
  50540137
 
  50540261
 
  PDF   STATS   ENSEMBL   UCSC 
 
  125
 
  4
 
 27811
 
 gene;island
 
 0.010417
 
 0.0532816
 
 Not queried
 
  1.7346170
 
 1.14e-01
 
 0.3123457953
  
 
  
 4
 
  47034181
 
  47034428
 
  PDF   STATS   ENSEMBL   UCSC 
 
  248
 
  4
 
 29996
 
 gene;island
 
 0.042973
 
 0.1116331
 
 Not queried
 
  1.7244345
 
 1.14e-01
 
 0.3123457953
  
 
  
 1
 
  60539559
 
  60539675
 
  PDF   STATS   ENSEMBL   UCSC 
 
  117
 
  4
 
 1660
 
 tss
 
 0.047965
 
 0.3499515
 
 Not queried
 
  1.7175378
 
 1.14e-01
 
 0.3123457953
  
 
  
 4
 
 128554125
 
 128554327
 
  PDF   STATS   ENSEMBL   UCSC 
 
  203
 
  4
 
 30124
 
 gene
 
 0.013269
 
 0.0048268
 
 Not queried
 
  1.7152704
 
 1.14e-01
 
 0.3123457953
  
 
  
 15
 
  89312795
 
  89313222
 
  PDF   STATS   ENSEMBL   UCSC 
 
  428
 
  7
 
 14421
 
 island
 
 0.071192
 
 0.4384446
 
 Not queried
 
  1.7147362
 
 9.73e-02
 
 0.3123457953
  
 
  
 15
 
  27019248
 
  27019448
 
  PDF   STATS   ENSEMBL   UCSC 
 
  201
 
  4
 
 13541
 
 tss;island
 
 0.018179
 
 0.0469065
 
 Not queried
 
  1.6836394
 
 1.14e-01
 
 0.3123457953
  
 
  
 11
 
  46414401
 
  46414626
 
  PDF   STATS   ENSEMBL   UCSC 
 
  226
 
  4
 
 8251
 
 island
 
 0.010284
 
 0.0050767
 
 Not queried
 
  1.6829847
 
 1.14e-01
 
 0.3123457953
  
 
  
 6
 
  30080189
 
  30080782
 
  PDF   STATS   ENSEMBL   UCSC 
 
  594
 
 10
 
 33725
 
 gene
 
 0.499614
 
 0.8781150
 
 Not queried
 
  1.6744988
 
 1.05e-01
 
 0.3123457953
  
 
  
 22
 
  30901532
 
  30901886
 
  PDF   STATS   ENSEMBL   UCSC 
 
  355
 
  7
 
 27413
 
 island
 
 0.100078
 
 0.5519746
 
 Not queried
 
  1.6481763
 
 9.73e-02
 
 0.3123457953
  
 
  
 12
 
 133179887
 
 133180238
 
  PDF   STATS   ENSEMBL   UCSC 
 
  352
 
  4
 
 11065
 
 island
 
 0.589702
 
 0.4101703
 
 Not queried
 
  1.6449185
 
 1.14e-01
 
 0.3123457953
  
 
  
 3
 
  68981852
 
  68982098
 
  PDF   STATS   ENSEMBL   UCSC 
 
  247
 
  9
 
 28437
 
 tss;island
 
 0.058628
 
 0.0300646
 
 Not queried
 
  1.6398591
 
 1.13e-01
 
 0.3123457953
  
 
  
 16
 
  55542668
 
  55542751
 
  PDF   STATS   ENSEMBL   UCSC 
 
   84
 
  4
 
 15240
 
 tss
 
 0.541110
 
 0.7808951
 
 Not queried
 
  1.6324991
 
 1.14e-01
 
 0.3123457953
  
 
  
 11
 
  33183108
 
  33183473
 
  PDF   STATS   ENSEMBL   UCSC 
 
  366
 
  7
 
 7232
 
 tss
 
 0.001650
 
 0.0008811
 
 Not queried
 
  1.6300295
 
 9.73e-02
 
 0.3123457953
  
 
  
 8
 
 122654023
 
 122654328
 
  PDF   STATS   ENSEMBL   UCSC 
 
  306
 
  4
 
 39415
 
 gene;tss;island
 
 0.001079
 
 0.0066885
 
 Not queried
 
  1.6291939
 
 1.14e-01
 
 0.3123457953
  
 
  
 1
 
 177133536
 
 177133737
 
  PDF   STATS   ENSEMBL   UCSC 
 
  202
 
  4
 
 897
 
 gene;island
 
 0.016803
 
 0.0407187
 
 Not queried
 
  1.6269059
 
 1.14e-01
 
 0.3123457953
  
 
  
 14
 
 105635706
 
 105635769
 
  PDF   STATS   ENSEMBL   UCSC 
 
   64
 
  4
 
 12667
 
 tss
 
 0.085190
 
 0.0297904
 
 Not queried
 
  1.6135006
 
 1.14e-01
 
 0.3123457953
  
 
  
 2
 
  27712613
 
  27713092
 
  PDF   STATS   ENSEMBL   UCSC 
 
  480
 
 10
 
 23271
 
 tss
 
 0.006120
 
 0.0256674
 
 Not queried
 
  1.6021195
 
 1.05e-01
 
 0.3123457953
  
 
  
 19
 
  49106849
 
  49107139
 
  PDF   STATS   ENSEMBL   UCSC 
 
  291
 
  4
 
 20129
 
 gene;island
 
 0.893000
 
 0.9579269
 
 Not queried
 
  1.5996488
 
 1.14e-01
 
 0.3123457953
  
 
  
 16
 
    891260
 
    891400
 
  PDF   STATS   ENSEMBL   UCSC 
 
  141
 
  4
 
 15493
 
 island
 
 0.816418
 
 0.9555346
 
 Not queried
 
  1.5960735
 
 1.14e-01
 
 0.3123457953
  
 
  
 17
 
  45908849
 
  45908985
 
  PDF   STATS   ENSEMBL   UCSC 
 
  137
 
  4
 
 17445
 
 tss;island
 
 0.001831
 
 0.0034994
 
 Not queried
 
  1.5862546
 
 1.14e-01
 
 0.3123457953
  
 
  
 20
 
  23471627
 
  23471737
 
  PDF   STATS   ENSEMBL   UCSC 
 
  111
 
  4
 
 25640
 
 tss
 
 0.420201
 
 0.7238814
 
 Not queried
 
  1.5860274
 
 1.14e-01
 
 0.3123457953
  
 
  
 17
 
  39743164
 
  39743427
 
  PDF   STATS   ENSEMBL   UCSC 
 
  264
 
  4
 
 17341
 
 tss
 
 0.405378
 
 0.6266914
 
 Not queried
 
  1.5829028
 
 1.14e-01
 
 0.3123457953
  
 
  
 10
 
  38146893
 
  38147123
 
  PDF   STATS   ENSEMBL   UCSC 
 
  231
 
  4
 
 5033
 
 tss;island
 
 0.045949
 
 0.0122500
 
 Not queried
 
  1.5703623
 
 1.14e-01
 
 0.3123457953
  
 
  
 16
 
    216450
 
    216757
 
  PDF   STATS   ENSEMBL   UCSC 
 
  308
 
  4
 
 14523
 
 gene;island
 
 0.036276
 
 0.1161318
 
 Not queried
 
  1.5690932
 
 1.14e-01
 
 0.3123457953
  
 
  
 1
 
  39956641
 
  39956807
 
  PDF   STATS   ENSEMBL   UCSC 
 
  167
 
  4
 
 1522
 
 tss;island
 
 0.190040
 
 0.0226243
 
 Not queried
 
  1.5589448
 
 1.14e-01
 
 0.3123457953
  
 
  
 11
 
  89224288
 
  89224799
 
  PDF   STATS   ENSEMBL   UCSC 
 
  512
 
 10
 
 6857
 
 gene;island
 
 0.071967
 
 0.0225441
 
 Not queried
 
  1.5560783
 
 1.05e-01
 
 0.3123457953
  
 
  
 11
 
   2828214
 
   2828778
 
  PDF   STATS   ENSEMBL   UCSC 
 
  565
 
  7
 
 6467
 
 gene;island
 
 0.231517
 
 0.5134463
 
 Not queried
 
  1.5345461
 
 9.73e-02
 
 0.3123457953
  
 
  
 7
 
  64734093
 
  64734749
 
  PDF   STATS   ENSEMBL   UCSC 
 
  657
 
  7
 
 38527
 
 island
 
 0.043718
 
 0.0092132
 
 Not queried
 
  1.5075798
 
 9.73e-02
 
 0.3123457953
  
 
  
 7
 
 158319029
 
 158319204
 
  PDF   STATS   ENSEMBL   UCSC 
 
  176
 
  4
 
 37377
 
 gene;island
 
 0.399448
 
 0.7051042
 
 Not queried
 
  1.5008941
 
 1.14e-01
 
 0.3123457953
  
 
  
 7
 
 128784416
 
 128784712
 
  PDF   STATS   ENSEMBL   UCSC 
 
  297
 
  7
 
 38905
 
 island
 
 0.009150
 
 0.0244624
 
 Not queried
 
  1.4876337
 
 9.73e-02
 
 0.3123457953
  
 
  
 15
 
  93615439
 
  93615718
 
  PDF   STATS   ENSEMBL   UCSC 
 
  280
 
  4
 
 13489
 
 gene;island
 
 0.001099
 
 0.0025650
 
 Not queried
 
  1.4844343
 
 1.14e-01
 
 0.3123457953
  
 
  
 14
 
  27066404
 
  27066771
 
  PDF   STATS   ENSEMBL   UCSC 
 
  368
 
  4
 
 12052
 
 gene;island
 
 0.048525
 
 0.0174840
 
 Not queried
 
  1.4842452
 
 1.14e-01
 
 0.3123457953
  
 
  
 20
 
  25129126
 
  25129400
 
  PDF   STATS   ENSEMBL   UCSC 
 
  275
 
  4
 
 25378
 
 gene
 
 0.039669
 
 0.1209305
 
 Not queried
 
  1.4835829
 
 1.14e-01
 
 0.3123457953
  
 
  
 11
 
   3253659
 
   3254504
 
  PDF   STATS   ENSEMBL   UCSC 
 
  846
 
 14
 
 7094
 
 tss
 
 0.655817
 
 0.7885070
 
 Not queried
 
  1.4810638
 
 1.04e-01
 
 0.3123457953
  
 
  
 14
 
  56232985
 
  56233178
 
  PDF   STATS   ENSEMBL   UCSC 
 
  194
 
  4
 
 12090
 
 gene
 
 0.563237
 
 0.9143382
 
 Not queried
 
  1.4743592
 
 1.14e-01
 
 0.3123457953
  
 
  
 3
 
 145878963
 
 145879277
 
  PDF   STATS   ENSEMBL   UCSC 
 
  315
 
  4
 
 27982
 
 gene
 
 0.048203
 
 0.0271433
 
 Not queried
 
  1.4701500
 
 1.14e-01
 
 0.3123457953
  
 
  
 10
 
 105344723
 
 105344807
 
  PDF   STATS   ENSEMBL   UCSC 
 
   85
 
  4
 
 4744
 
 gene;island
 
 0.437152
 
 0.2396080
 
 Not queried
 
  1.4683823
 
 1.14e-01
 
 0.3123457953
  
 
  
 14
 
  60559007
 
  60559200
 
  PDF   STATS   ENSEMBL   UCSC 
 
  194
 
  4
 
 12100
 
 gene;island
 
 0.004621
 
 0.0121580
 
 Not queried
 
  1.4671100
 
 1.14e-01
 
 0.3123457953
  
 
  
 6
 
   6004207
 
   6004464
 
  PDF   STATS   ENSEMBL   UCSC 
 
  258
 
  4
 
 33544
 
 gene;island
 
 0.107414
 
 0.0532324
 
 Not queried
 
  1.4603911
 
 1.14e-01
 
 0.3123457953
  
 
  
 17
 
  44060252
 
  44060502
 
  PDF   STATS   ENSEMBL   UCSC 
 
  251
 
  4
 
 16642
 
 gene;island
 
 0.525803
 
 0.8237045
 
 Not queried
 
  1.4579624
 
 1.14e-01
 
 0.3123457953
  
 
  
 20
 
    825050
 
    825798
 
  PDF   STATS   ENSEMBL   UCSC 
 
  749
 
 10
 
 25324
 
 gene;island
 
 0.014375
 
 0.0369399
 
 Not queried
 
  1.4572174
 
 1.05e-01
 
 0.3123457953
  
 
  
 8
 
  49833685
 
  49833956
 
  PDF   STATS   ENSEMBL   UCSC 
 
  272
 
  4
 
 39302
 
 gene;island
 
 0.059268
 
 0.0095251
 
 Not queried
 
  1.4551530
 
 1.14e-01
 
 0.3123457953
  
 
  
 1
 
  68629000
 
  68629133
 
  PDF   STATS   ENSEMBL   UCSC 
 
  134
 
  4
 
 575
 
 gene;island
 
 0.739661
 
 0.5518637
 
 Not queried
 
  1.4236516
 
 1.14e-01
 
 0.3123457953
  
 
  
 11
 
  12398874
 
  12399109
 
  PDF   STATS   ENSEMBL   UCSC 
 
  236
 
  7
 
 7154
 
 tss
 
 0.050502
 
 0.0827144
 
 Not queried
 
  1.4206283
 
 9.73e-02
 
 0.3123457953
  
 
  
 7
 
   8301971
 
   8302301
 
  PDF   STATS   ENSEMBL   UCSC 
 
  331
 
  8
 
 37438
 
 tss;island
 
 0.046914
 
 0.1094109
 
 Not queried
 
  1.4037099
 
 1.05e-01
 
 0.3123457953
  
 
  
 11
 
   2481857
 
   2482594
 
  PDF   STATS   ENSEMBL   UCSC 
 
  738
 
 15
 
 7076
 
 tss
 
 0.159465
 
 0.3971130
 
 Not queried
 
  1.7035883
 
 1.16e-01
 
 0.3167640946
  
 
  
 6
 
  10415597
 
  10416090
 
  PDF   STATS   ENSEMBL   UCSC 
 
  494
 
 11
 
 33561
 
 gene;tss;island
 
 0.547082
 
 0.3076797
 
 Not queried
 
  1.8043464
 
 1.16e-01
 
 0.3175184142
  
 
  
 2
 
  63277672
 
  63277944
 
  PDF   STATS   ENSEMBL   UCSC 
 
  273
 
 11
 
 23387
 
 tss
 
 0.005495
 
 0.0027708
 
 Not queried
 
  1.6854309
 
 1.16e-01
 
 0.3175184142
  
 
  
 11
 
 118842126
 
 118842650
 
  PDF   STATS   ENSEMBL   UCSC 
 
  525
 
 10
 
 8864
 
 island
 
 0.943276
 
 0.5611051
 
 Not queried
 
  2.6433457
 
 1.23e-01
 
 0.3348527312
  
 
  
 20
 
  34359544
 
  34359976
 
  PDF   STATS   ENSEMBL   UCSC 
 
  433
 
 10
 
 26131
 
 island
 
 0.003258
 
 0.0092285
 
 Not queried
 
  2.0863347
 
 1.23e-01
 
 0.3348527312
  
 
  
 19
 
   1438017
 
   1438306
 
  PDF   STATS   ENSEMBL   UCSC 
 
  290
 
 10
 
 20305
 
 tss
 
 0.017648
 
 0.0924066
 
 Not queried
 
  1.6987558
 
 1.23e-01
 
 0.3348527312
  
 
  
 5
 
 140578896
 
 140579303
 
  PDF   STATS   ENSEMBL   UCSC 
 
  408
 
  7
 
 32366
 
 tss
 
 0.012741
 
 0.9148090
 
 Not queried
 
  5.4205924
 
 1.28e-01
 
 0.3397986247
  
 
  
 18
 
  74535840
 
  74536226
 
  PDF   STATS   ENSEMBL   UCSC 
 
  387
 
  6
 
 19582
 
 island
 
 0.585095
 
 0.0504722
 
 Not queried
 
  5.3613171
 
 1.32e-01
 
 0.3397986247
  
 
  
 6
 
  32181764
 
  32182326
 
  PDF   STATS   ENSEMBL   UCSC 
 
  563
 
  6
 
 34093
 
 gene
 
 0.027788
 
 0.4495004
 
 Not queried
 
  5.3217485
 
 1.32e-01
 
 0.3397986247
  
 
  
 11
 
 101454676
 
 101454996
 
  PDF   STATS   ENSEMBL   UCSC 
 
  321
 
  7
 
 7643
 
 tss
 
 0.056417
 
 0.4534735
 
 Not queried
 
  4.8953236
 
 1.28e-01
 
 0.3397986247
  
 
  
 10
 
  18629497
 
  18629677
 
  PDF   STATS   ENSEMBL   UCSC 
 
  181
 
  6
 
 4522
 
 gene
 
 0.466567
 
 0.9369457
 
 Not queried
 
  4.1971002
 
 1.32e-01
 
 0.3397986247
  
 
  
 2
 
  63275868
 
  63276268
 
  PDF   STATS   ENSEMBL   UCSC 
 
  401
 
  6
 
 23385
 
 tss;island
 
 0.423178
 
 0.0401711
 
 Not queried
 
  4.1395685
 
 1.32e-01
 
 0.3397986247
  
 
  
 19
 
  55691730
 
  55692003
 
  PDF   STATS   ENSEMBL   UCSC 
 
  274
 
  7
 
 21072
 
 tss;island
 
 0.185237
 
 0.0351534
 
 Not queried
 
  4.1039580
 
 1.28e-01
 
 0.3397986247
  
 
  
 14
 
 104314434
 
 104314734
 
  PDF   STATS   ENSEMBL   UCSC 
 
  301
 
  6
 
 12654
 
 tss;island
 
 0.020896
 
 0.0010466
 
 Not queried
 
  3.9174243
 
 1.32e-01
 
 0.3397986247
  
 
  
 21
 
  43198283
 
  43198797
 
  PDF   STATS   ENSEMBL   UCSC 
 
  515
 
  6
 
 26694
 
 island
 
 0.069966
 
 0.4170170
 
 Not queried
 
  3.9062947
 
 1.32e-01
 
 0.3397986247
  
 
  
 7
 
 157179773
 
 157180124
 
  PDF   STATS   ENSEMBL   UCSC 
 
  352
 
  6
 
 37320
 
 gene
 
 0.971951
 
 0.5703864
 
 Not queried
 
  3.8708355
 
 1.32e-01
 
 0.3397986247
  
 
  
 5
 
  43000303
 
  43000890
 
  PDF   STATS   ENSEMBL   UCSC 
 
  588
 
  6
 
 32764
 
 island
 
 0.297950
 
 0.7019960
 
 Not queried
 
  3.7385462
 
 1.32e-01
 
 0.3397986247
  
 
  
 2
 
 219906245
 
 219906468
 
  PDF   STATS   ENSEMBL   UCSC 
 
  224
 
  7
 
 23889
 
 tss
 
 0.010265
 
 0.0977714
 
 Not queried
 
  3.6387830
 
 1.28e-01
 
 0.3397986247
  
 
  
 7
 
 153584416
 
 153584873
 
  PDF   STATS   ENSEMBL   UCSC 
 
  458
 
  7
 
 39053
 
 island
 
 0.214607
 
 0.5445993
 
 Not queried
 
  3.5888067
 
 1.28e-01
 
 0.3397986247
  
 
  
 12
 
 115122028
 
 115122304
 
  PDF   STATS   ENSEMBL   UCSC 
 
  277
 
  6
 
 10036
 
 tss;island
 
 0.164730
 
 0.0410793
 
 Not queried
 
  3.5703618
 
 1.32e-01
 
 0.3397986247
  
 
  
 6
 
  33386967
 
  33387205
 
  PDF   STATS   ENSEMBL   UCSC 
 
  239
 
  6
 
 34908
 
 tss;island
 
 0.647067
 
 0.9553141
 
 Not queried
 
  3.5680267
 
 1.32e-01
 
 0.3397986247
  
 
  
 1
 
  91182534
 
  91183051
 
  PDF   STATS   ENSEMBL   UCSC 
 
  518
 
  7
 
 3458
 
 island
 
 0.221207
 
 0.5677069
 
 Not queried
 
  3.5631785
 
 1.28e-01
 
 0.3397986247
  
 
  
 7
 
   2059796
 
   2060179
 
  PDF   STATS   ENSEMBL   UCSC 
 
  384
 
  6
 
 36786
 
 gene;island
 
 0.503158
 
 0.9258758
 
 Not queried
 
  3.5162198
 
 1.32e-01
 
 0.3397986247
  
 
  
 10
 
    695844
 
    696063
 
  PDF   STATS   ENSEMBL   UCSC 
 
  220
 
  6
 
 4449
 
 gene
 
 0.438396
 
 0.7636201
 
 Not queried
 
  3.4922069
 
 1.32e-01
 
 0.3397986247
  
 
  
 6
 
  56112392
 
  56112696
 
  PDF   STATS   ENSEMBL   UCSC 
 
  305
 
  8
 
 35044
 
 tss
 
 0.017206
 
 0.3490909
 
 Not queried
 
  3.4170327
 
 1.30e-01
 
 0.3397986247
  
 
  
 2
 
  43398079
 
  43398339
 
  PDF   STATS   ENSEMBL   UCSC 
 
  261
 
  6
 
 24248
 
 island
 
 0.497167
 
 0.9489853
 
 Not queried
 
  3.1159645
 
 1.32e-01
 
 0.3397986247
  
 
  
 5
 
 145713607
 
 145713882
 
  PDF   STATS   ENSEMBL   UCSC 
 
  276
 
  6
 
 33279
 
 island
 
 0.015629
 
 0.3816110
 
 Not queried
 
  3.1086948
 
 1.32e-01
 
 0.3397986247
  
 
  
 11
 
  70672740
 
  70672986
 
  PDF   STATS   ENSEMBL   UCSC 
 
  247
 
  7
 
 6797
 
 gene;island
 
 0.030969
 
 0.3579024
 
 Not queried
 
  3.0357612
 
 1.28e-01
 
 0.3397986247
  
 
  
 1
 
 200011684
 
 200011988
 
  PDF   STATS   ENSEMBL   UCSC 
 
  305
 
  6
 
 934
 
 gene;island
 
 0.102862
 
 0.0108638
 
 Not queried
 
  2.9995193
 
 1.32e-01
 
 0.3397986247
  
 
  
 2
 
  30144547
 
  30144838
 
  PDF   STATS   ENSEMBL   UCSC 
 
  292
 
  6
 
 23283
 
 tss
 
 0.012349
 
 0.0045441
 
 Not queried
 
  2.8875527
 
 1.32e-01
 
 0.3397986247
  
 
  
 10
 
  77542302
 
  77542488
 
  PDF   STATS   ENSEMBL   UCSC 
 
  187
 
  7
 
 5177
 
 tss
 
 0.896352
 
 0.3628286
 
 Not queried
 
  2.8567180
 
 1.28e-01
 
 0.3397986247
  
 
  
 17
 
   1548881
 
   1549434
 
  PDF   STATS   ENSEMBL   UCSC 
 
  554
 
  6
 
 17821
 
 island
 
 0.248519
 
 0.8381442
 
 Not queried
 
  2.8172140
 
 1.32e-01
 
 0.3397986247
  
 
  
 22
 
  45809244
 
  45809454
 
  PDF   STATS   ENSEMBL   UCSC 
 
  211
 
  6
 
 26903
 
 gene
 
 0.583575
 
 0.2374703
 
 Not queried
 
  2.7924196
 
 1.32e-01
 
 0.3397986247
  
 
  
 13
 
 114880761
 
 114881194
 
  PDF   STATS   ENSEMBL   UCSC 
 
  434
 
  6
 
 11316
 
 gene;island
 
 0.556425
 
 0.9015543
 
 Not queried
 
  2.7838574
 
 1.32e-01
 
 0.3397986247
  
 
  
 1
 
 247171403
 
 247171838
 
  PDF   STATS   ENSEMBL   UCSC 
 
  436
 
  7
 
 2365
 
 tss
 
 0.102150
 
 0.0149454
 
 Not queried
 
  2.7594971
 
 1.28e-01
 
 0.3397986247
  
 
  
 4
 
   7637856
 
   7638280
 
  PDF   STATS   ENSEMBL   UCSC 
 
  425
 
  6
 
 29940
 
 gene
 
 0.426288
 
 0.5453953
 
 Not queried
 
  2.7087880
 
 1.32e-01
 
 0.3397986247
  
 
  
 8
 
  19459996
 
  19460243
 
  PDF   STATS   ENSEMBL   UCSC 
 
  248
 
  6
 
 39221
 
 gene;island
 
 0.529596
 
 0.9155166
 
 Not queried
 
  2.7036221
 
 1.32e-01
 
 0.3397986247
  
 
  
 20
 
  61866902
 
  61867218
 
  PDF   STATS   ENSEMBL   UCSC 
 
  317
 
  8
 
 25891
 
 tss
 
 0.234251
 
 0.6747862
 
 Not queried
 
  2.6389507
 
 1.30e-01
 
 0.3397986247
  
 
  
 8
 
  67344553
 
  67344665
 
  PDF   STATS   ENSEMBL   UCSC 
 
  113
 
  6
 
 39742
 
 tss
 
 0.011661
 
 0.0852933
 
 Not queried
 
  2.5706166
 
 1.32e-01
 
 0.3397986247
  
 
  
 10
 
  95326801
 
  95327325
 
  PDF   STATS   ENSEMBL   UCSC 
 
  525
 
  6
 
 4682
 
 gene;island
 
 0.165107
 
 0.0466165
 
 Not queried
 
  2.5503245
 
 1.32e-01
 
 0.3397986247
  
 
  
 4
 
   4387697
 
   4387917
 
  PDF   STATS   ENSEMBL   UCSC 
 
  221
 
  6
 
 30267
 
 tss;island
 
 0.143151
 
 0.0130273
 
 Not queried
 
  2.5501652
 
 1.32e-01
 
 0.3397986247
  
 
  
 3
 
  10149918
 
  10150139
 
  PDF   STATS   ENSEMBL   UCSC 
 
  222
 
  7
 
 28142
 
 tss
 
 0.954410
 
 0.9123461
 
 Not queried
 
  2.5427612
 
 1.28e-01
 
 0.3397986247
  
 
  
 7
 
   5938193
 
   5938293
 
  PDF   STATS   ENSEMBL   UCSC 
 
  101
 
  6
 
 37428
 
 tss;island
 
 0.002997
 
 0.0069024
 
 Not queried
 
  2.4610943
 
 1.32e-01
 
 0.3397986247
  
 
  
 11
 
 101454626
 
 101454996
 
  PDF   STATS   ENSEMBL   UCSC 
 
  371
 
  8
 
 8756
 
 island
 
 0.060003
 
 0.3457013
 
 Not queried
 
  2.4089203
 
 1.30e-01
 
 0.3397986247
  
 
  
 2
 
  14772312
 
  14772734
 
  PDF   STATS   ENSEMBL   UCSC 
 
  423
 
  7
 
 23195
 
 tss;island
 
 0.060575
 
 0.1856801
 
 Not queried
 
  2.3970642
 
 1.28e-01
 
 0.3397986247
  
 
  
 12
 
   5603131
 
   5603261
 
  PDF   STATS   ENSEMBL   UCSC 
 
  131
 
  6
 
 9019
 
 gene;tss;island
 
 0.185839
 
 0.4083781
 
 Not queried
 
  2.3900737
 
 1.32e-01
 
 0.3397986247
  
 
  
 3
 
 121902442
 
 121902622
 
  PDF   STATS   ENSEMBL   UCSC 
 
  181
 
  7
 
 29396
 
 island
 
 0.295385
 
 0.4866874
 
 Not queried
 
  2.3742055
 
 1.28e-01
 
 0.3397986247
  
 
  
 19
 
   1275121
 
   1275466
 
  PDF   STATS   ENSEMBL   UCSC 
 
  346
 
  6
 
 20299
 
 tss;island
 
 0.043809
 
 0.0054162
 
 Not queried
 
  2.3359241
 
 1.32e-01
 
 0.3397986247
  
 
  
 11
 
 114069864
 
 114070210
 
  PDF   STATS   ENSEMBL   UCSC 
 
  347
 
  6
 
 6911
 
 gene;island
 
 0.537097
 
 0.7947402
 
 Not queried
 
  2.3297671
 
 1.32e-01
 
 0.3397986247
  
 
  
 2
 
 119599459
 
 119600002
 
  PDF   STATS   ENSEMBL   UCSC 
 
  544
 
  6
 
 24644
 
 island
 
 0.063512
 
 0.0173191
 
 Not queried
 
  2.3291470
 
 1.32e-01
 
 0.3397986247
  
 
  
 2
 
 132202427
 
 132202537
 
  PDF   STATS   ENSEMBL   UCSC 
 
  111
 
  6
 
 24732
 
 island
 
 0.938573
 
 0.6792436
 
 Not queried
 
  2.3232503
 
 1.32e-01
 
 0.3397986247
  
 
  
 5
 
  57755773
 
  57756026
 
  PDF   STATS   ENSEMBL   UCSC 
 
  254
 
  8
 
 32815
 
 island
 
 0.008161
 
 0.0140553
 
 Not queried
 
  2.2995560
 
 1.30e-01
 
 0.3397986247
  
 
  
 5
 
 170736021
 
 170736277
 
  PDF   STATS   ENSEMBL   UCSC 
 
  257
 
  7
 
 32486
 
 tss
 
 0.358178
 
 0.1378274
 
 Not queried
 
  2.2682380
 
 1.28e-01
 
 0.3397986247
  
 
  
 4
 
  77172617
 
  77172841
 
  PDF   STATS   ENSEMBL   UCSC 
 
  225
 
  6
 
 30447
 
 tss
 
 0.008970
 
 0.0038725
 
 Not queried
 
  2.2087409
 
 1.32e-01
 
 0.3397986247
  
 
  
 8
 
  67344553
 
  67344720
 
  PDF   STATS   ENSEMBL   UCSC 
 
  168
 
  7
 
 40354
 
 island
 
 0.007639
 
 0.0787184
 
 Not queried
 
  2.2082289
 
 1.28e-01
 
 0.3397986247
  
 
  
 16
 
  58498456
 
  58498818
 
  PDF   STATS   ENSEMBL   UCSC 
 
  363
 
  8
 
 14772
 
 gene;island
 
 0.085940
 
 0.0137775
 
 Not queried
 
  2.1996798
 
 1.30e-01
 
 0.3397986247
  
 
  
 6
 
  32365491
 
  32366096
 
  PDF   STATS   ENSEMBL   UCSC 
 
  606
 
  7
 
 34107
 
 gene
 
 0.125887
 
 0.0142202
 
 Not queried
 
  2.1949765
 
 1.28e-01
 
 0.3397986247
  
 
  
 14
 
 105444724
 
 105445253
 
  PDF   STATS   ENSEMBL   UCSC 
 
  530
 
  8
 
 12663
 
 tss
 
 0.015087
 
 0.0803697
 
 Not queried
 
  2.1772501
 
 1.30e-01
 
 0.3397986247
  
 
  
 11
 
  67183078
 
  67183616
 
  PDF   STATS   ENSEMBL   UCSC 
 
  539
 
  8
 
 8545
 
 island
 
 0.018898
 
 0.2600868
 
 Not queried
 
  2.1631248
 
 1.30e-01
 
 0.3397986247
  
 
  
 20
 
  44993182
 
  44993436
 
  PDF   STATS   ENSEMBL   UCSC 
 
  255
 
  7
 
 25790
 
 tss
 
 0.116040
 
 0.0172185
 
 Not queried
 
  2.1615165
 
 1.28e-01
 
 0.3397986247
  
 
  
 11
 
  31827593
 
  31828040
 
  PDF   STATS   ENSEMBL   UCSC 
 
  448
 
  7
 
 6561
 
 gene;island
 
 0.183850
 
 0.0346008
 
 Not queried
 
  2.1589524
 
 1.28e-01
 
 0.3397986247
  
 
  
 18
 
  32957329
 
  32957683
 
  PDF   STATS   ENSEMBL   UCSC 
 
  355
 
  8
 
 19237
 
 tss;island
 
 0.058621
 
 0.4845186
 
 Not queried
 
  2.1251502
 
 1.30e-01
 
 0.3397986247
  
 
  
 7
 
 130126153
 
 130126637
 
  PDF   STATS   ENSEMBL   UCSC 
 
  485
 
  6
 
 37223
 
 gene
 
 0.026178
 
 0.0716954
 
 Not queried
 
  2.1233094
 
 1.32e-01
 
 0.3397986247
  
 
  
 15
 
  73076635
 
  73077046
 
  PDF   STATS   ENSEMBL   UCSC 
 
  412
 
  6
 
 13780
 
 tss;island
 
 0.312048
 
 0.0622330
 
 Not queried
 
  2.1184458
 
 1.32e-01
 
 0.3397986247
  
 
  
 19
 
  49926748
 
  49927309
 
  PDF   STATS   ENSEMBL   UCSC 
 
  562
 
  6
 
 20944
 
 tss
 
 0.046810
 
 0.1458620
 
 Not queried
 
  2.0867540
 
 1.32e-01
 
 0.3397986247
  
 
  
 6
 
  33135753
 
  33136061
 
  PDF   STATS   ENSEMBL   UCSC 
 
  309
 
  7
 
 34198
 
 gene
 
 0.596140
 
 0.8168719
 
 Not queried
 
  2.0598807
 
 1.28e-01
 
 0.3397986247
  
 
  
 7
 
  50633725
 
  50634252
 
  PDF   STATS   ENSEMBL   UCSC 
 
  528
 
  6
 
 37595
 
 tss
 
 0.146953
 
 0.4032683
 
 Not queried
 
  1.9922215
 
 1.32e-01
 
 0.3397986247
  
 
  
 1
 
  21044685
 
  21044945
 
  PDF   STATS   ENSEMBL   UCSC 
 
  261
 
  6
 
 1318
 
 tss;island
 
 0.026846
 
 0.2123271
 
 Not queried
 
  1.9503629
 
 1.32e-01
 
 0.3397986247
  
 
  
 17
 
  79905152
 
  79905319
 
  PDF   STATS   ENSEMBL   UCSC 
 
  168
 
  7
 
 17762
 
 tss
 
 0.669460
 
 0.9145035
 
 Not queried
 
  1.9129984
 
 1.28e-01
 
 0.3397986247
  
 
  
 10
 
 131356942
 
 131357256
 
  PDF   STATS   ENSEMBL   UCSC 
 
  315
 
  6
 
 4827
 
 gene
 
 0.684864
 
 0.8683282
 
 Not queried
 
  1.9081491
 
 1.32e-01
 
 0.3397986247
  
 
  
 1
 
  47696212
 
  47696788
 
  PDF   STATS   ENSEMBL   UCSC 
 
  577
 
  6
 
 1607
 
 tss;island
 
 0.070647
 
 0.0150852
 
 Not queried
 
  1.8960373
 
 1.32e-01
 
 0.3397986247
  
 
  
 6
 
   1598854
 
   1599401
 
  PDF   STATS   ENSEMBL   UCSC 
 
  548
 
  6
 
 35370
 
 island
 
 0.493415
 
 0.8987920
 
 Not queried
 
  1.8630846
 
 1.32e-01
 
 0.3397986247
  
 
  
 7
 
   8473234
 
   8473578
 
  PDF   STATS   ENSEMBL   UCSC 
 
  345
 
  6
 
 37439
 
 tss;island
 
 0.027300
 
 0.0896942
 
 Not queried
 
  1.8284335
 
 1.32e-01
 
 0.3397986247
  
 
  
 11
 
  50257496
 
  50257979
 
  PDF   STATS   ENSEMBL   UCSC 
 
  484
 
  7
 
 8279
 
 island
 
 0.664115
 
 0.8059195
 
 Not queried
 
  1.8277788
 
 1.28e-01
 
 0.3397986247
  
 
  
 11
 
  64067685
 
  64067868
 
  PDF   STATS   ENSEMBL   UCSC 
 
  184
 
  7
 
 8403
 
 island
 
 0.020195
 
 0.0838877
 
 Not queried
 
  1.8110894
 
 1.28e-01
 
 0.3397986247
  
 
  
 2
 
  72079276
 
  72079609
 
  PDF   STATS   ENSEMBL   UCSC 
 
  334
 
  6
 
 24402
 
 island
 
 0.187642
 
 0.4266485
 
 Not queried
 
  1.8003923
 
 1.32e-01
 
 0.3397986247
  
 
  
 2
 
 232790974
 
 232791126
 
  PDF   STATS   ENSEMBL   UCSC 
 
  153
 
  6
 
 23946
 
 tss;island
 
 0.033863
 
 0.0153290
 
 Not queried
 
  1.7858055
 
 1.32e-01
 
 0.3397986247
  
 
  
 8
 
 143859669
 
 143859990
 
  PDF   STATS   ENSEMBL   UCSC 
 
  322
 
  7
 
 39938
 
 tss;island
 
 0.368854
 
 0.5738676
 
 Not queried
 
  1.7594063
 
 1.28e-01
 
 0.3397986247
  
 
  
 6
 
 142409939
 
 142410235
 
  PDF   STATS   ENSEMBL   UCSC 
 
  297
 
  8
 
 35260
 
 tss
 
 0.161760
 
 0.0465878
 
 Not queried
 
  1.7086683
 
 1.30e-01
 
 0.3397986247
  
 
  
 19
 
  50553682
 
  50554067
 
  PDF   STATS   ENSEMBL   UCSC 
 
  386
 
  7
 
 22299
 
 island
 
 0.013263
 
 0.0751992
 
 Not queried
 
  1.7021394
 
 1.28e-01
 
 0.3397986247
  
 
  
 7
 
  54609587
 
  54610320
 
  PDF   STATS   ENSEMBL   UCSC 
 
  734
 
 12
 
 38498
 
 island
 
 0.022204
 
 0.1106090
 
 Not queried
 
  1.7014273
 
 1.28e-01
 
 0.3397986247
  
 
  
 3
 
 158390510
 
 158390821
 
  PDF   STATS   ENSEMBL   UCSC 
 
  312
 
  7
 
 28686
 
 tss
 
 0.032922
 
 0.2825048
 
 Not queried
 
  1.6701691
 
 1.28e-01
 
 0.3397986247
  
 
  
 4
 
  55095148
 
  55095529
 
  PDF   STATS   ENSEMBL   UCSC 
 
  382
 
  8
 
 31055
 
 island
 
 0.049378
 
 0.0099997
 
 Not queried
 
  1.6496189
 
 1.30e-01
 
 0.3397986247
  
 
  
 10
 
 135340445
 
 135340850
 
  PDF   STATS   ENSEMBL   UCSC 
 
  406
 
  7
 
 5465
 
 tss
 
 0.361049
 
 0.6929544
 
 Not queried
 
  1.6182692
 
 1.28e-01
 
 0.3397986247
  
 
  
 2
 
 133174035
 
 133174168
 
  PDF   STATS   ENSEMBL   UCSC 
 
  134
 
  6
 
 24741
 
 island
 
 0.018527
 
 0.0587871
 
 Not queried
 
  1.6144054
 
 1.32e-01
 
 0.3397986247
  
 
  
 10
 
     95198
 
     95398
 
  PDF   STATS   ENSEMBL   UCSC 
 
  201
 
  6
 
 4908
 
 tss
 
 0.231801
 
 0.5262028
 
 Not queried
 
  1.5968843
 
 1.32e-01
 
 0.3397986247
  
 
  
 20
 
  18568287
 
  18568549
 
  PDF   STATS   ENSEMBL   UCSC 
 
  263
 
  7
 
 25620
 
 tss
 
 0.004565
 
 0.0018213
 
 Not queried
 
  1.5425559
 
 1.28e-01
 
 0.3397986247
  
 
  
 12
 
  10183167
 
  10183267
 
  PDF   STATS   ENSEMBL   UCSC 
 
  101
 
  6
 
 9559
 
 tss
 
 0.916443
 
 0.9743593
 
 Not queried
 
  1.5121147
 
 1.32e-01
 
 0.3397986247
  
 
  
 11
 
  64885222
 
  64885558
 
  PDF   STATS   ENSEMBL   UCSC 
 
  337
 
  8
 
 7415
 
 tss
 
 0.006582
 
 0.0028725
 
 Not queried
 
  1.4943556
 
 1.30e-01
 
 0.3397986247
  
 
  
 17
 
  16593426
 
  16593613
 
  PDF   STATS   ENSEMBL   UCSC 
 
  188
 
  6
 
 17161
 
 tss;island
 
 0.496981
 
 0.2020077
 
 Not queried
 
  1.4823372
 
 1.32e-01
 
 0.3397986247
  
 
  
 17
 
  73874443
 
  73874790
 
  PDF   STATS   ENSEMBL   UCSC 
 
  348
 
  7
 
 18765
 
 island
 
 0.060081
 
 0.0129582
 
 Not queried
 
  1.4728856
 
 1.28e-01
 
 0.3397986247
  
 
  
 17
 
  76506489
 
  76506855
 
  PDF   STATS   ENSEMBL   UCSC 
 
  367
 
  8
 
 16823
 
 gene
 
 0.741228
 
 0.8951445
 
 Not queried
 
  1.4402198
 
 1.30e-01
 
 0.3397986247
  
 
  
 5
 
  59189649
 
  59189934
 
  PDF   STATS   ENSEMBL   UCSC 
 
  286
 
  8
 
 32079
 
 tss
 
 0.105468
 
 0.3384295
 
 Not queried
 
  1.4123949
 
 1.30e-01
 
 0.3397986247
  
 
  
 5
 
 145316063
 
 145316416
 
  PDF   STATS   ENSEMBL   UCSC 
 
  354
 
  8
 
 33277
 
 island
 
 0.029734
 
 0.0582972
 
 Not queried
 
  1.4076950
 
 1.30e-01
 
 0.3397986247
  
 
  
 2
 
  20189711
 
  20190349
 
  PDF   STATS   ENSEMBL   UCSC 
 
  639
 
 11
 
 24106
 
 island
 
 0.073096
 
 0.0251274
 
 Not queried
 
  1.6905168
 
 1.33e-01
 
 0.3420544733
  
 
  
 12
 
 132896199
 
 132896939
 
  PDF   STATS   ENSEMBL   UCSC 
 
  741
 
  9
 
 9447
 
 gene;island
 
 0.541334
 
 0.8478458
 
 Not queried
 
  2.1934353
 
 1.36e-01
 
 0.3491249184
  
 
  
 8
 
  23261618
 
  23262159
 
  PDF   STATS   ENSEMBL   UCSC 
 
  542
 
  9
 
 40151
 
 island
 
 0.031657
 
 0.1645760
 
 Not queried
 
  1.5544883
 
 1.36e-01
 
 0.3491249184
  
 
  
 6
 
  29796351
 
  29796770
 
  PDF   STATS   ENSEMBL   UCSC 
 
  420
 
 15
 
 33697
 
 gene;island
 
 0.402293
 
 0.5486822
 
 Not queried
 
  1.4327282
 
 1.37e-01
 
 0.3516378542
  
 
  
 3
 
  30647378
 
  30648098
 
  PDF   STATS   ENSEMBL   UCSC 
 
  721
 
 13
 
 28957
 
 island
 
 0.014097
 
 0.0504448
 
 Not queried
 
  2.2382313
 
 1.39e-01
 
 0.3560599333
  
 
  
 13
 
  36920644
 
  36921174
 
  PDF   STATS   ENSEMBL   UCSC 
 
  531
 
 13
 
 11131
 
 gene;island
 
 0.004275
 
 0.0072987
 
 Not queried
 
  1.4549556
 
 1.39e-01
 
 0.3560599333
  
 
  
 6
 
  31322577
 
  31322996
 
  PDF   STATS   ENSEMBL   UCSC 
 
  420
 
  5
 
 33867
 
 gene;island
 
 0.012559
 
 0.4097707
 
 Not queried
 
  6.5376871
 
 1.51e-01
 
 0.3618000441
  
 
  
 18
 
  21719263
 
  21719433
 
  PDF   STATS   ENSEMBL   UCSC 
 
  171
 
  5
 
 19210
 
 tss
 
 0.013548
 
 0.5238099
 
 Not queried
 
  6.2518224
 
 1.51e-01
 
 0.3618000441
  
 
  
 2
 
 219848258
 
 219848580
 
  PDF   STATS   ENSEMBL   UCSC 
 
  323
 
  5
 
 23022
 
 gene;island
 
 0.287540
 
 0.0101754
 
 Not queried
 
  6.0692760
 
 1.51e-01
 
 0.3618000441
  
 
  
 8
 
  37605552
 
  37605978
 
  PDF   STATS   ENSEMBL   UCSC 
 
  427
 
  5
 
 39646
 
 tss
 
 0.599871
 
 0.0299448
 
 Not queried
 
  5.9299419
 
 1.51e-01
 
 0.3618000441
  
 
  
 7
 
  27265004
 
  27265522
 
  PDF   STATS   ENSEMBL   UCSC 
 
  519
 
  5
 
 38364
 
 island
 
 0.074195
 
 0.2193581
 
 Not queried
 
  5.2079819
 
 1.51e-01
 
 0.3618000441
  
 
  
 11
 
  31848347
 
  31848828
 
  PDF   STATS   ENSEMBL   UCSC 
 
  482
 
  5
 
 8170
 
 island
 
 0.142804
 
 0.0250653
 
 Not queried
 
  5.0262157
 
 1.51e-01
 
 0.3618000441
  
 
  
 6
 
 152126736
 
 152126938
 
  PDF   STATS   ENSEMBL   UCSC 
 
  203
 
  5
 
 34512
 
 gene;island
 
 0.270620
 
 0.5778432
 
 Not queried
 
  4.8718095
 
 1.51e-01
 
 0.3618000441
  
 
  
 11
 
 113746456
 
 113746831
 
  PDF   STATS   ENSEMBL   UCSC 
 
  376
 
  5
 
 7706
 
 tss;island
 
 0.110392
 
 0.0053712
 
 Not queried
 
  4.5708826
 
 1.51e-01
 
 0.3618000441
  
 
  
 3
 
 181437145
 
 181437712
 
  PDF   STATS   ENSEMBL   UCSC 
 
  568
 
  5
 
 28043
 
 gene;island
 
 0.003133
 
 0.2850011
 
 Not queried
 
  4.5189274
 
 1.51e-01
 
 0.3618000441
  
 
  
 14
 
  96152464
 
  96152706
 
  PDF   STATS   ENSEMBL   UCSC 
 
  243
 
  5
 
 12589
 
 tss
 
 0.514913
 
 0.9577324
 
 Not queried
 
  4.4779915
 
 1.51e-01
 
 0.3618000441
  
 
  
 10
 
    468051
 
    468412
 
  PDF   STATS   ENSEMBL   UCSC 
 
  362
 
  5
 
 4439
 
 gene;island
 
 0.047484
 
 0.7374656
 
 Not queried
 
  4.3777021
 
 1.51e-01
 
 0.3618000441
  
 
  
 17
 
  35300448
 
  35300874
 
  PDF   STATS   ENSEMBL   UCSC 
 
  427
 
  5
 
 18238
 
 island
 
 0.053014
 
 0.6592834
 
 Not queried
 
  4.3171017
 
 1.51e-01
 
 0.3618000441
  
 
  
 1
 
 210110998
 
 210111162
 
  PDF   STATS   ENSEMBL   UCSC 
 
  165
 
  5
 
 2235
 
 tss;island
 
 0.060473
 
 0.0089126
 
 Not queried
 
  4.2636542
 
 1.51e-01
 
 0.3618000441
  
 
  
 5
 
 142065671
 
 142065819
 
  PDF   STATS   ENSEMBL   UCSC 
 
  149
 
  5
 
 32398
 
 tss
 
 0.027041
 
 0.3670222
 
 Not queried
 
  4.1596394
 
 1.51e-01
 
 0.3618000441
  
 
  
 2
 
 200468445
 
 200469026
 
  PDF   STATS   ENSEMBL   UCSC 
 
  582
 
  5
 
 24994
 
 island
 
 0.441209
 
 0.8163402
 
 Not queried
 
  4.0674152
 
 1.51e-01
 
 0.3618000441
  
 
  
 15
 
  90437628
 
  90437891
 
  PDF   STATS   ENSEMBL   UCSC 
 
  264
 
  5
 
 13893
 
 tss
 
 0.172879
 
 0.0138283
 
 Not queried
 
  3.9029461
 
 1.51e-01
 
 0.3618000441
  
 
  
 1
 
  74663583
 
  74663827
 
  PDF   STATS   ENSEMBL   UCSC 
 
  245
 
  5
 
 583
 
 gene
 
 0.079821
 
 0.0021672
 
 Not queried
 
  3.8772487
 
 1.51e-01
 
 0.3618000441
  
 
  
 5
 
 132155233
 
 132155555
 
  PDF   STATS   ENSEMBL   UCSC 
 
  323
 
  5
 
 33101
 
 island
 
 0.471365
 
 0.0611205
 
 Not queried
 
  3.7852489
 
 1.51e-01
 
 0.3618000441
  
 
  
 3
 
 128205143
 
 128205496
 
  PDF   STATS   ENSEMBL   UCSC 
 
  354
 
  5
 
 27936
 
 gene;island
 
 0.029275
 
 0.1053049
 
 Not queried
 
  3.7553198
 
 1.51e-01
 
 0.3618000441
  
 
  
 17
 
   9929528
 
   9929867
 
  PDF   STATS   ENSEMBL   UCSC 
 
  340
 
  5
 
 16440
 
 gene
 
 0.053440
 
 0.6254988
 
 Not queried
 
  3.6931038
 
 1.51e-01
 
 0.3618000441
  
 
  
 11
 
   5617703
 
   5617926
 
  PDF   STATS   ENSEMBL   UCSC 
 
  224
 
  5
 
 6485
 
 gene
 
 0.019773
 
 0.0367787
 
 Not queried
 
  3.6358871
 
 1.51e-01
 
 0.3618000441
  
 
  
 22
 
  38218945
 
  38219400
 
  PDF   STATS   ENSEMBL   UCSC 
 
  456
 
  5
 
 27475
 
 island
 
 0.061730
 
 0.4372888
 
 Not queried
 
  3.5631111
 
 1.51e-01
 
 0.3618000441
  
 
  
 4
 
  85402870
 
  85403409
 
  PDF   STATS   ENSEMBL   UCSC 
 
  540
 
  5
 
 31158
 
 island
 
 0.060851
 
 0.4270387
 
 Not queried
 
  3.5356525
 
 1.51e-01
 
 0.3618000441
  
 
  
 1
 
  32708234
 
  32708485
 
  PDF   STATS   ENSEMBL   UCSC 
 
  252
 
  5
 
 1456
 
 tss;island
 
 0.055494
 
 0.4538287
 
 Not queried
 
  3.4998693
 
 1.51e-01
 
 0.3618000441
  
 
  
 8
 
 142517341
 
 142517553
 
  PDF   STATS   ENSEMBL   UCSC 
 
  213
 
  5
 
 39930
 
 tss
 
 0.033302
 
 0.1309394
 
 Not queried
 
  3.4979756
 
 1.51e-01
 
 0.3618000441
  
 
  
 1
 
 150947832
 
 150948030
 
  PDF   STATS   ENSEMBL   UCSC 
 
  199
 
  5
 
 1916
 
 tss;island
 
 0.732467
 
 0.0300704
 
 Not queried
 
  3.4885148
 
 1.51e-01
 
 0.3618000441
  
 
  
 1
 
  50893469
 
  50893984
 
  PDF   STATS   ENSEMBL   UCSC 
 
  516
 
  5
 
 3254
 
 island
 
 0.497596
 
 0.0477150
 
 Not queried
 
  3.4479253
 
 1.51e-01
 
 0.3618000441
  
 
  
 2
 
   1286216
 
   1286641
 
  PDF   STATS   ENSEMBL   UCSC 
 
  426
 
  5
 
 22528
 
 gene;island
 
 0.549962
 
 0.6042107
 
 Not queried
 
  3.4325950
 
 1.51e-01
 
 0.3618000441
  
 
  
 11
 
  31825605
 
  31825969
 
  PDF   STATS   ENSEMBL   UCSC 
 
  365
 
  5
 
 6560
 
 gene;island
 
 0.018203
 
 0.1383361
 
 Not queried
 
  3.3431448
 
 1.51e-01
 
 0.3618000441
  
 
  
 6
 
 166891617
 
 166891959
 
  PDF   STATS   ENSEMBL   UCSC 
 
  343
 
  5
 
 34546
 
 gene
 
 0.453685
 
 0.9082311
 
 Not queried
 
  3.3375776
 
 1.51e-01
 
 0.3618000441
  
 
  
 8
 
   9763552
 
   9764073
 
  PDF   STATS   ENSEMBL   UCSC 
 
  522
 
  5
 
 40066
 
 island
 
 0.251502
 
 0.0021723
 
 Not queried
 
  3.3337846
 
 1.51e-01
 
 0.3618000441
  
 
  
 17
 
  45949677
 
  45949878
 
  PDF   STATS   ENSEMBL   UCSC 
 
  202
 
  5
 
 18464
 
 island
 
 0.554480
 
 0.8047263
 
 Not queried
 
  3.2650056
 
 1.51e-01
 
 0.3618000441
  
 
  
 11
 
  17229487
 
  17229731
 
  PDF   STATS   ENSEMBL   UCSC 
 
  245
 
  5
 
 8095
 
 island
 
 0.000279
 
 0.0038103
 
 Not queried
 
  3.2170317
 
 1.51e-01
 
 0.3618000441
  
 
  
 17
 
  79420145
 
  79420436
 
  PDF   STATS   ENSEMBL   UCSC 
 
  292
 
  5
 
 16911
 
 gene;island
 
 0.059488
 
 0.3692773
 
 Not queried
 
  3.2098960
 
 1.51e-01
 
 0.3618000441
  
 
  
 13
 
 113104883
 
 113105341
 
  PDF   STATS   ENSEMBL   UCSC 
 
  459
 
  5
 
 11920
 
 island
 
 0.336563
 
 0.8075437
 
 Not queried
 
  3.2061007
 
 1.51e-01
 
 0.3618000441
  
 
  
 7
 
  16460965
 
  16461244
 
  PDF   STATS   ENSEMBL   UCSC 
 
  280
 
  5
 
 37451
 
 tss
 
 0.235574
 
 0.0266022
 
 Not queried
 
  3.1966773
 
 1.51e-01
 
 0.3618000441
  
 
  
 6
 
  29570507
 
  29570781
 
  PDF   STATS   ENSEMBL   UCSC 
 
  275
 
  5
 
 33663
 
 gene
 
 0.251345
 
 0.4621757
 
 Not queried
 
  3.1683048
 
 1.51e-01
 
 0.3618000441
  
 
  
 1
 
  98510865
 
  98511258
 
  PDF   STATS   ENSEMBL   UCSC 
 
  394
 
  5
 
 3513
 
 island
 
 0.013544
 
 0.1609140
 
 Not queried
 
  3.1503290
 
 1.51e-01
 
 0.3618000441
  
 
  
 2
 
  97505275
 
  97505787
 
  PDF   STATS   ENSEMBL   UCSC 
 
  513
 
  5
 
 22787
 
 gene;island
 
 0.666745
 
 0.9779356
 
 Not queried
 
  3.1419825
 
 1.51e-01
 
 0.3618000441
  
 
  
 5
 
  41509849
 
  41510116
 
  PDF   STATS   ENSEMBL   UCSC 
 
  268
 
  5
 
 31599
 
 gene;island
 
 0.044750
 
 0.5973028
 
 Not queried
 
  3.1264669
 
 1.51e-01
 
 0.3618000441
  
 
  
 1
 
  15256054
 
  15256222
 
  PDF   STATS   ENSEMBL   UCSC 
 
  169
 
  5
 
 240
 
 gene;tss
 
 0.050273
 
 0.4664632
 
 Not queried
 
  3.1025409
 
 1.51e-01
 
 0.3618000441
  
 
  
 8
 
 110704026
 
 110704289
 
  PDF   STATS   ENSEMBL   UCSC 
 
  264
 
  5
 
 39874
 
 tss
 
 0.007865
 
 0.0410854
 
 Not queried
 
  3.0986503
 
 1.51e-01
 
 0.3618000441
  
 
  
 17
 
  33760230
 
  33760527
 
  PDF   STATS   ENSEMBL   UCSC 
 
  298
 
  5
 
 17270
 
 tss
 
 0.264130
 
 0.0293236
 
 Not queried
 
  3.0643946
 
 1.51e-01
 
 0.3618000441
  
 
  
 3
 
 191048308
 
 191048439
 
  PDF   STATS   ENSEMBL   UCSC 
 
  132
 
  5
 
 28084
 
 gene;island
 
 0.001807
 
 0.0055487
 
 Not queried
 
  3.0314492
 
 1.51e-01
 
 0.3618000441
  
 
  
 12
 
 119616616
 
 119616913
 
  PDF   STATS   ENSEMBL   UCSC 
 
  298
 
  5
 
 9340
 
 gene
 
 0.034877
 
 0.0067880
 
 Not queried
 
  3.0240357
 
 1.51e-01
 
 0.3618000441
  
 
  
 5
 
 135265753
 
 135265937
 
  PDF   STATS   ENSEMBL   UCSC 
 
  185
 
  5
 
 32308
 
 tss
 
 0.015248
 
 0.0950287
 
 Not queried
 
  2.9848937
 
 1.51e-01
 
 0.3618000441
  
 
  
 11
 
  18230754
 
  18231021
 
  PDF   STATS   ENSEMBL   UCSC 
 
  268
 
  5
 
 6526
 
 gene
 
 0.537989
 
 0.9017393
 
 Not queried
 
  2.9784066
 
 1.51e-01
 
 0.3618000441
  
 
  
 19
 
    496239
 
    496428
 
  PDF   STATS   ENSEMBL   UCSC 
 
  190
 
  5
 
 20273
 
 tss
 
 0.021964
 
 0.0819689
 
 Not queried
 
  2.9335544
 
 1.51e-01
 
 0.3618000441
  
 
  
 16
 
  69141250
 
  69141437
 
  PDF   STATS   ENSEMBL   UCSC 
 
  188
 
  5
 
 15326
 
 tss
 
 0.011495
 
 0.2011220
 
 Not queried
 
  2.9315752
 
 1.51e-01
 
 0.3618000441
  
 
  
 6
 
  30122947
 
  30123226
 
  PDF   STATS   ENSEMBL   UCSC 
 
  280
 
  5
 
 33730
 
 gene
 
 0.154383
 
 0.4952480
 
 Not queried
 
  2.8776459
 
 1.51e-01
 
 0.3618000441
  
 
  
 5
 
  16784775
 
  16785135
 
  PDF   STATS   ENSEMBL   UCSC 
 
  361
 
  5
 
 31570
 
 gene
 
 0.184098
 
 0.6081389
 
 Not queried
 
  2.8612401
 
 1.51e-01
 
 0.3618000441
  
 
  
 11
 
  67183148
 
  67183616
 
  PDF   STATS   ENSEMBL   UCSC 
 
  469
 
  5
 
 6753
 
 gene
 
 0.081083
 
 0.5308419
 
 Not queried
 
  2.7792598
 
 1.51e-01
 
 0.3618000441
  
 
  
 12
 
 104697193
 
 104697545
 
  PDF   STATS   ENSEMBL   UCSC 
 
  353
 
 10
 
 9959
 
 tss
 
 0.061978
 
 0.0239481
 
 Not queried
 
  2.7760759
 
 1.43e-01
 
 0.3618000441
  
 
  
 16
 
  29625133
 
  29625259
 
  PDF   STATS   ENSEMBL   UCSC 
 
  127
 
  5
 
 15166
 
 tss;island
 
 0.004801
 
 0.0106568
 
 Not queried
 
  2.7492631
 
 1.51e-01
 
 0.3618000441
  
 
  
 11
 
    393938
 
    394289
 
  PDF   STATS   ENSEMBL   UCSC 
 
  352
 
  5
 
 7847
 
 island
 
 0.443709
 
 0.6670112
 
 Not queried
 
  2.7203472
 
 1.51e-01
 
 0.3618000441
  
 
  
 8
 
  26724744
 
  26725033
 
  PDF   STATS   ENSEMBL   UCSC 
 
  290
 
  5
 
 40177
 
 island
 
 0.009539
 
 0.0474909
 
 Not queried
 
  2.6805635
 
 1.51e-01
 
 0.3618000441
  
 
  
 12
 
  54393966
 
  54394212
 
  PDF   STATS   ENSEMBL   UCSC 
 
  247
 
  5
 
 9157
 
 gene;island
 
 0.029785
 
 0.1950794
 
 Not queried
 
  2.5810850
 
 1.51e-01
 
 0.3618000441
  
 
  
 8
 
  23385905
 
  23386187
 
  PDF   STATS   ENSEMBL   UCSC 
 
  283
 
  5
 
 39602
 
 tss;island
 
 0.004843
 
 0.4735073
 
 Not queried
 
  2.5666648
 
 1.51e-01
 
 0.3618000441
  
 
  
 8
 
  65488640
 
  65489065
 
  PDF   STATS   ENSEMBL   UCSC 
 
  426
 
  5
 
 39322
 
 gene;island
 
 0.111812
 
 0.0106453
 
 Not queried
 
  2.4965513
 
 1.51e-01
 
 0.3618000441
  
 
  
 16
 
   3414983
 
   3415311
 
  PDF   STATS   ENSEMBL   UCSC 
 
  329
 
  5
 
 15647
 
 island
 
 0.007182
 
 0.1895461
 
 Not queried
 
  2.4695408
 
 1.51e-01
 
 0.3618000441
  
 
  
 5
 
  50265272
 
  50265767
 
  PDF   STATS   ENSEMBL   UCSC 
 
  496
 
  5
 
 32786
 
 island
 
 0.037838
 
 0.1620725
 
 Not queried
 
  2.4572889
 
 1.51e-01
 
 0.3618000441
  
 
  
 18
 
  52626476
 
  52626848
 
  PDF   STATS   ENSEMBL   UCSC 
 
  373
 
  5
 
 19285
 
 tss;island
 
 0.086313
 
 0.0207855
 
 Not queried
 
  2.4321878
 
 1.51e-01
 
 0.3618000441
  
 
  
 1
 
 247275735
 
 247276096
 
  PDF   STATS   ENSEMBL   UCSC 
 
  362
 
  5
 
 2367
 
 tss
 
 0.238521
 
 0.6272460
 
 Not queried
 
  2.3905979
 
 1.51e-01
 
 0.3618000441
  
 
  
 2
 
 224904626
 
 224904837
 
  PDF   STATS   ENSEMBL   UCSC 
 
  212
 
  5
 
 23924
 
 tss;island
 
 0.021635
 
 0.1649503
 
 Not queried
 
  2.3735811
 
 1.51e-01
 
 0.3618000441
  
 
  
 7
 
 155601876
 
 155602259
 
  PDF   STATS   ENSEMBL   UCSC 
 
  384
 
  5
 
 37312
 
 gene;island
 
 0.025247
 
 0.0113338
 
 Not queried
 
  2.3669211
 
 1.51e-01
 
 0.3618000441
  
 
  
 7
 
 157366933
 
 157367159
 
  PDF   STATS   ENSEMBL   UCSC 
 
  227
 
  5
 
 37328
 
 gene;island
 
 0.342701
 
 0.7258505
 
 Not queried
 
  2.3467602
 
 1.51e-01
 
 0.3618000441
  
 
  
 3
 
  58563609
 
  58563693
 
  PDF   STATS   ENSEMBL   UCSC 
 
   85
 
  5
 
 28419
 
 tss
 
 0.051822
 
 0.1739978
 
 Not queried
 
  2.2952662
 
 1.51e-01
 
 0.3618000441
  
 
  
 22
 
  33196328
 
  33196627
 
  PDF   STATS   ENSEMBL   UCSC 
 
  300
 
  5
 
 26850
 
 gene;tss;island
 
 0.166764
 
 0.5415209
 
 Not queried
 
  2.2714990
 
 1.51e-01
 
 0.3618000441
  
 
  
 20
 
  17549599
 
  17549955
 
  PDF   STATS   ENSEMBL   UCSC 
 
  357
 
  5
 
 25608
 
 tss;island
 
 0.187214
 
 0.0751218
 
 Not queried
 
  2.2653395
 
 1.51e-01
 
 0.3618000441
  
 
  
 19
 
  12228348
 
  12228618
 
  PDF   STATS   ENSEMBL   UCSC 
 
  271
 
  5
 
 21593
 
 island
 
 0.761009
 
 0.9413005
 
 Not queried
 
  2.2126575
 
 1.51e-01
 
 0.3618000441
  
 
  
 15
 
 102264738
 
 102264772
 
  PDF   STATS   ENSEMBL   UCSC 
 
   35
 
  5
 
 13936
 
 tss;island
 
 0.000653
 
 0.0029676
 
 Not queried
 
  2.2098444
 
 1.51e-01
 
 0.3618000441
  
 
  
 11
 
 117947459
 
 117947657
 
  PDF   STATS   ENSEMBL   UCSC 
 
  199
 
  5
 
 7725
 
 tss
 
 0.444355
 
 0.6383892
 
 Not queried
 
  2.2007856
 
 1.51e-01
 
 0.3618000441
  
 
  
 7
 
  44579245
 
  44579475
 
  PDF   STATS   ENSEMBL   UCSC 
 
  231
 
  5
 
 36993
 
 gene
 
 0.274819
 
 0.6754242
 
 Not queried
 
  2.1969650
 
 1.51e-01
 
 0.3618000441
  
 
  
 6
 
  11111894
 
  11112237
 
  PDF   STATS   ENSEMBL   UCSC 
 
  344
 
  5
 
 33569
 
 gene
 
 0.863359
 
 0.5301021
 
 Not queried
 
  2.1941814
 
 1.51e-01
 
 0.3618000441
  
 
  
 10
 
 101282572
 
 101282883
 
  PDF   STATS   ENSEMBL   UCSC 
 
  312
 
  5
 
 6009
 
 island
 
 0.616440
 
 0.8450342
 
 Not queried
 
  2.1739712
 
 1.51e-01
 
 0.3618000441
  
 
  
 2
 
 170335723
 
 170335894
 
  PDF   STATS   ENSEMBL   UCSC 
 
  172
 
  5
 
 23702
 
 tss;island
 
 0.055545
 
 0.4272432
 
 Not queried
 
  2.1698963
 
 1.51e-01
 
 0.3618000441
  
 
  
 10
 
    334430
 
    334773
 
  PDF   STATS   ENSEMBL   UCSC 
 
  344
 
  5
 
 4432
 
 gene
 
 0.423948
 
 0.5293270
 
 Not queried
 
  2.1093483
 
 1.51e-01
 
 0.3618000441
  
 
  
 1
 
 209848306
 
 209848577
 
  PDF   STATS   ENSEMBL   UCSC 
 
  272
 
  5
 
 2231
 
 tss;island
 
 0.038318
 
 0.2054520
 
 Not queried
 
  2.1079386
 
 1.51e-01
 
 0.3618000441
  
 
  
 13
 
 102068234
 
 102068650
 
  PDF   STATS   ENSEMBL   UCSC 
 
  417
 
  5
 
 11212
 
 gene;island
 
 0.038962
 
 0.2876562
 
 Not queried
 
  2.0550213
 
 1.51e-01
 
 0.3618000441
  
 
  
 19
 
  45975694
 
  45976195
 
  PDF   STATS   ENSEMBL   UCSC 
 
  502
 
  5
 
 20098
 
 gene;island
 
 0.039785
 
 0.2341775
 
 Not queried
 
  2.0452856
 
 1.51e-01
 
 0.3618000441
  
 
  
 2
 
 242754333
 
 242754756
 
  PDF   STATS   ENSEMBL   UCSC 
 
  424
 
  5
 
 23147
 
 gene;island
 
 0.669520
 
 0.9485087
 
 Not queried
 
  2.0444823
 
 1.51e-01
 
 0.3618000441
  
 
  
 7
 
  96632294
 
  96632841
 
  PDF   STATS   ENSEMBL   UCSC 
 
  548
 
  5
 
 37101
 
 gene;island
 
 0.666707
 
 0.1175437
 
 Not queried
 
  1.9974756
 
 1.51e-01
 
 0.3618000441
  
 
  
 18
 
  55102849
 
  55102925
 
  PDF   STATS   ENSEMBL   UCSC 
 
   77
 
  5
 
 19532
 
 island
 
 0.020801
 
 0.0969011
 
 Not queried
 
  1.9798646
 
 1.51e-01
 
 0.3618000441
  
 
  
 6
 
  33156779
 
  33157222
 
  PDF   STATS   ENSEMBL   UCSC 
 
  444
 
 10
 
 34213
 
 gene;island
 
 0.224646
 
 0.5127815
 
 Not queried
 
  1.9785832
 
 1.43e-01
 
 0.3618000441
  
 
  
 14
 
  45431486
 
  45431885
 
  PDF   STATS   ENSEMBL   UCSC 
 
  400
 
  5
 
 12073
 
 gene
 
 0.011616
 
 0.0033594
 
 Not queried
 
  1.9768323
 
 1.51e-01
 
 0.3618000441
  
 
  
 3
 
 127174705
 
 127175109
 
  PDF   STATS   ENSEMBL   UCSC 
 
  405
 
  5
 
 29443
 
 island
 
 0.019081
 
 0.2724310
 
 Not queried
 
  1.9424914
 
 1.51e-01
 
 0.3618000441
  
 
  
 1
 
 183387128
 
 183387564
 
  PDF   STATS   ENSEMBL   UCSC 
 
  437
 
  5
 
 916
 
 gene
 
 0.055477
 
 0.0220820
 
 Not queried
 
  1.9293994
 
 1.51e-01
 
 0.3618000441
  
 
  
 2
 
  98329691
 
  98330020
 
  PDF   STATS   ENSEMBL   UCSC 
 
  330
 
  5
 
 23531
 
 tss
 
 0.034523
 
 0.2067331
 
 Not queried
 
  1.9224576
 
 1.51e-01
 
 0.3618000441
  
 
  
 17
 
  79393463
 
  79393718
 
  PDF   STATS   ENSEMBL   UCSC 
 
  256
 
  5
 
 16908
 
 gene;island
 
 0.503865
 
 0.7851212
 
 Not queried
 
  1.8476436
 
 1.51e-01
 
 0.3618000441
  
 
  
 7
 
 157333405
 
 157333949
 
  PDF   STATS   ENSEMBL   UCSC 
 
  545
 
  5
 
 37324
 
 gene
 
 0.600708
 
 0.8835025
 
 Not queried
 
  1.8365330
 
 1.51e-01
 
 0.3618000441
  
 
  
 11
 
  50257633
 
  50257979
 
  PDF   STATS   ENSEMBL   UCSC 
 
  347
 
  5
 
 7296
 
 tss
 
 0.614782
 
 0.7275173
 
 Not queried
 
  1.8277788
 
 1.51e-01
 
 0.3618000441
  
 
  
 20
 
   2361431
 
   2361548
 
  PDF   STATS   ENSEMBL   UCSC 
 
  118
 
  5
 
 25543
 
 tss
 
 0.465697
 
 0.7532331
 
 Not queried
 
  1.8213132
 
 1.51e-01
 
 0.3618000441
  
 
  
 2
 
 191207820
 
 191208232
 
  PDF   STATS   ENSEMBL   UCSC 
 
  413
 
  5
 
 24961
 
 island
 
 0.084018
 
 0.0243049
 
 Not queried
 
  1.8034009
 
 1.51e-01
 
 0.3618000441
  
 
  
 11
 
 121970613
 
 121970768
 
  PDF   STATS   ENSEMBL   UCSC 
 
  156
 
  5
 
 7769
 
 tss
 
 0.815645
 
 0.9406248
 
 Not queried
 
  1.7975224
 
 1.51e-01
 
 0.3618000441
  
 
  
 1
 
 114447334
 
 114447605
 
  PDF   STATS   ENSEMBL   UCSC 
 
  272
 
  5
 
 699
 
 gene
 
 0.006483
 
 0.0164463
 
 Not queried
 
  1.7753760
 
 1.51e-01
 
 0.3618000441
  
 
  
 8
 
  63161579
 
  63161676
 
  PDF   STATS   ENSEMBL   UCSC 
 
   98
 
  5
 
 39318
 
 gene;island
 
 0.079013
 
 0.0102500
 
 Not queried
 
  1.7688893
 
 1.51e-01
 
 0.3618000441
  
 
  
 5
 
   7826845
 
   7827133
 
  PDF   STATS   ENSEMBL   UCSC 
 
  289
 
  5
 
 31555
 
 gene
 
 0.156455
 
 0.3920735
 
 Not queried
 
  1.7567510
 
 1.51e-01
 
 0.3618000441
  
 
  
 20
 
  61051317
 
  61052060
 
  PDF   STATS   ENSEMBL   UCSC 
 
  744
 
 18
 
 25872
 
 tss;island
 
 0.018981
 
 0.0336903
 
 Not queried
 
  1.7399288
 
 1.43e-01
 
 0.3618000441
  
 
  
 14
 
  23398313
 
  23398654
 
  PDF   STATS   ENSEMBL   UCSC 
 
  342
 
  5
 
 12019
 
 gene
 
 0.000940
 
 0.0040751
 
 Not queried
 
  1.7385510
 
 1.51e-01
 
 0.3618000441
  
 
  
 4
 
  77355817
 
  77356250
 
  PDF   STATS   ENSEMBL   UCSC 
 
  434
 
  5
 
 30449
 
 tss
 
 0.406627
 
 0.5959410
 
 Not queried
 
  1.7017370
 
 1.51e-01
 
 0.3618000441
  
 
  
 6
 
  69345836
 
  69346248
 
  PDF   STATS   ENSEMBL   UCSC 
 
  413
 
  5
 
 34379
 
 gene;island
 
 0.014336
 
 0.0271453
 
 Not queried
 
  1.6350809
 
 1.51e-01
 
 0.3618000441
  
 
  
 17
 
  37895771
 
  37896175
 
  PDF   STATS   ENSEMBL   UCSC 
 
  405
 
  5
 
 16556
 
 gene
 
 0.494825
 
 0.8634164
 
 Not queried
 
  1.6224296
 
 1.51e-01
 
 0.3618000441
  
 
  
 13
 
 103052144
 
 103052560
 
  PDF   STATS   ENSEMBL   UCSC 
 
  417
 
  5
 
 11215
 
 gene;island
 
 0.042030
 
 0.1203582
 
 Not queried
 
  1.6221052
 
 1.51e-01
 
 0.3618000441
  
 
  
 8
 
  49890360
 
  49890873
 
  PDF   STATS   ENSEMBL   UCSC 
 
  514
 
  5
 
 40288
 
 island
 
 0.752072
 
 0.8940682
 
 Not queried
 
  1.6082244
 
 1.51e-01
 
 0.3618000441
  
 
  
 11
 
 117352692
 
 117352939
 
  PDF   STATS   ENSEMBL   UCSC 
 
  248
 
  5
 
 6920
 
 gene;island
 
 0.642081
 
 0.8963945
 
 Not queried
 
  1.5849862
 
 1.51e-01
 
 0.3618000441
  
 
  
 14
 
  81421983
 
  81422160
 
  PDF   STATS   ENSEMBL   UCSC 
 
  178
 
  5
 
 12161
 
 gene;island
 
 0.200536
 
 0.5009834
 
 Not queried
 
  1.5416732
 
 1.51e-01
 
 0.3618000441
  
 
  
 12
 
  75785089
 
  75785295
 
  PDF   STATS   ENSEMBL   UCSC 
 
  207
 
  5
 
 9222
 
 gene;island
 
 0.002679
 
 0.0055901
 
 Not queried
 
  1.5405345
 
 1.51e-01
 
 0.3618000441
  
 
  
 1
 
   2918373
 
   2918752
 
  PDF   STATS   ENSEMBL   UCSC 
 
  380
 
  5
 
 2525
 
 island
 
 0.400248
 
 0.5859395
 
 Not queried
 
  1.5181853
 
 1.51e-01
 
 0.3618000441
  
 
  
 11
 
 130416857
 
 130417168
 
  PDF   STATS   ENSEMBL   UCSC 
 
  312
 
  5
 
 8963
 
 island
 
 0.442166
 
 0.6254598
 
 Not queried
 
  1.5093707
 
 1.51e-01
 
 0.3618000441
  
 
  
 17
 
   8661887
 
   8662011
 
  PDF   STATS   ENSEMBL   UCSC 
 
  125
 
  5
 
 17121
 
 tss
 
 0.477414
 
 0.6294408
 
 Not queried
 
  1.4719671
 
 1.51e-01
 
 0.3618000441
  
 
  
 1
 
  46152302
 
  46152494
 
  PDF   STATS   ENSEMBL   UCSC 
 
  193
 
  5
 
 1589
 
 tss
 
 0.003761
 
 0.0032501
 
 Not queried
 
  1.4716714
 
 1.51e-01
 
 0.3618000441
  
 
  
 2
 
 131673771
 
 131674687
 
  PDF   STATS   ENSEMBL   UCSC 
 
  917
 
 11
 
 24725
 
 island
 
 0.253113
 
 0.4803829
 
 Not queried
 
  1.6279060
 
 1.51e-01
 
 0.3627442585
  
 
  
 1
 
  32801964
 
  32802312
 
  PDF   STATS   ENSEMBL   UCSC 
 
  349
 
  8
 
 1458
 
 tss;island
 
 0.063611
 
 0.3766846
 
 Not queried
 
  3.6218997
 
 1.61e-01
 
 0.3814043454
  
 
  
 1
 
 207277018
 
 207277581
 
  PDF   STATS   ENSEMBL   UCSC 
 
  564
 
  8
 
 2219
 
 tss
 
 0.563654
 
 0.9144842
 
 Not queried
 
  3.1661488
 
 1.61e-01
 
 0.3814043454
  
 
  
 11
 
 122852412
 
 122852698
 
  PDF   STATS   ENSEMBL   UCSC 
 
  287
 
  8
 
 7775
 
 tss
 
 0.043201
 
 0.1965441
 
 Not queried
 
  2.8184821
 
 1.61e-01
 
 0.3814043454
  
 
  
 17
 
  11501503
 
  11501663
 
  PDF   STATS   ENSEMBL   UCSC 
 
  161
 
  8
 
 17137
 
 tss;island
 
 0.093546
 
 0.2188429
 
 Not queried
 
  2.5353840
 
 1.61e-01
 
 0.3814043454
  
 
  
 11
 
  94134646
 
  94135029
 
  PDF   STATS   ENSEMBL   UCSC 
 
  384
 
  8
 
 7629
 
 tss
 
 0.244520
 
 0.0665760
 
 Not queried
 
  2.4108005
 
 1.61e-01
 
 0.3814043454
  
 
  
 4
 
 155663529
 
 155664311
 
  PDF   STATS   ENSEMBL   UCSC 
 
  783
 
  8
 
 31386
 
 island
 
 0.031154
 
 0.1181505
 
 Not queried
 
  2.3967314
 
 1.61e-01
 
 0.3814043454
  
 
  
 6
 
  26183508
 
  26184245
 
  PDF   STATS   ENSEMBL   UCSC 
 
  738
 
 12
 
 35521
 
 island
 
 0.046651
 
 0.0192424
 
 Not queried
 
  2.2606249
 
 1.60e-01
 
 0.3814043454
  
 
  
 8
 
  22422657
 
  22423091
 
  PDF   STATS   ENSEMBL   UCSC 
 
  435
 
  8
 
 39234
 
 gene;tss;island
 
 0.013601
 
 0.0729163
 
 Not queried
 
  2.1786435
 
 1.61e-01
 
 0.3814043454
  
 
  
 6
 
 117197952
 
 117198281
 
  PDF   STATS   ENSEMBL   UCSC 
 
  330
 
  8
 
 35194
 
 tss
 
 0.075964
 
 0.0177783
 
 Not queried
 
  2.0572410
 
 1.61e-01
 
 0.3814043454
  
 
  
 12
 
  49393027
 
  49393408
 
  PDF   STATS   ENSEMBL   UCSC 
 
  382
 
  8
 
 10381
 
 island
 
 0.018932
 
 0.0532970
 
 Not queried
 
  1.7272748
 
 1.61e-01
 
 0.3814043454
  
 
  
 14
 
  70233599
 
  70233819
 
  PDF   STATS   ENSEMBL   UCSC 
 
  221
 
  8
 
 12483
 
 tss
 
 0.005621
 
 0.0093011
 
 Not queried
 
  1.6562356
 
 1.61e-01
 
 0.3814043454
  
 
  
 17
 
   4802692
 
   4803053
 
  PDF   STATS   ENSEMBL   UCSC 
 
  362
 
  8
 
 16359
 
 gene;island
 
 0.021502
 
 0.0126353
 
 Not queried
 
  1.5272651
 
 1.61e-01
 
 0.3814043454
  
 
  
 2
 
  31805915
 
  31806551
 
  PDF   STATS   ENSEMBL   UCSC 
 
  637
 
  8
 
 24205
 
 island
 
 0.617906
 
 0.4528895
 
 Not queried
 
  1.4491871
 
 1.61e-01
 
 0.3814043454
  
 
  
 7
 
   1351539
 
   1352002
 
  PDF   STATS   ENSEMBL   UCSC 
 
  464
 
  8
 
 38098
 
 island
 
 0.710303
 
 0.9255634
 
 Not queried
 
  1.4171632
 
 1.61e-01
 
 0.3814043454
  
 
  
 11
 
  65554042
 
  65554410
 
  PDF   STATS   ENSEMBL   UCSC 
 
  369
 
  8
 
 7441
 
 tss;island
 
 0.024382
 
 0.0120805
 
 Not queried
 
  1.4130266
 
 1.61e-01
 
 0.3814043454
  
 
  
 4
 
 146403866
 
 146404134
 
  PDF   STATS   ENSEMBL   UCSC 
 
  269
 
  8
 
 30146
 
 gene;island
 
 0.028988
 
 0.0014241
 
 Not queried
 
  1.4052924
 
 1.61e-01
 
 0.3814043454
  
 
  
 10
 
  18629497
 
  18629602
 
  PDF   STATS   ENSEMBL   UCSC 
 
  106
 
  4
 
 4974
 
 tss
 
 0.178035
 
 0.9308506
 
 Not queried
 
  7.8250209
 
 2.00e-01
 
 0.3826488233
  
 
  
 10
 
  38692145
 
  38692317
 
  PDF   STATS   ENSEMBL   UCSC 
 
  173
 
  4
 
 5038
 
 tss;island
 
 0.266319
 
 0.0129665
 
 Not queried
 
  6.9911931
 
 2.00e-01
 
 0.3826488233
  
 
  
 6
 
  32031395
 
  32031820
 
  PDF   STATS   ENSEMBL   UCSC 
 
  426
 
  4
 
 34027
 
 gene
 
 0.036104
 
 0.6808349
 
 Not queried
 
  6.0921318
 
 2.00e-01
 
 0.3826488233
  
 
  
 5
 
 178986131
 
 178986412
 
  PDF   STATS   ENSEMBL   UCSC 
 
  282
 
  4
 
 31932
 
 gene;tss;island
 
 0.542875
 
 0.0575700
 
 Not queried
 
  5.9429089
 
 2.00e-01
 
 0.3826488233
  
 
  
 3
 
 194119861
 
 194120150
 
  PDF   STATS   ENSEMBL   UCSC 
 
  290
 
  4
 
 29788
 
 island
 
 0.762812
 
 0.0474323
 
 Not queried
 
  5.7620747
 
 2.00e-01
 
 0.3826488233
  
 
  
 7
 
  37959686
 
  37959966
 
  PDF   STATS   ENSEMBL   UCSC 
 
  281
 
  4
 
 37543
 
 tss;island
 
 0.112338
 
 0.8866424
 
 Not queried
 
  5.6260583
 
 2.00e-01
 
 0.3826488233
  
 
  
 1
 
   1104257
 
   1104366
 
  PDF   STATS   ENSEMBL   UCSC 
 
  110
 
  4
 
 1141
 
 tss
 
 0.011158
 
 0.3980179
 
 Not queried
 
  5.5056717
 
 2.00e-01
 
 0.3826488233
  
 
  
 18
 
  70211774
 
  70211974
 
  PDF   STATS   ENSEMBL   UCSC 
 
  201
 
  6
 
 19318
 
 tss;island
 
 0.448068
 
 0.0199522
 
 Not queried
 
  5.2218287
 
 1.80e-01
 
 0.3826488233
  
 
  
 11
 
    843897
 
    844400
 
  PDF   STATS   ENSEMBL   UCSC 
 
  504
 
  8
 
 7033
 
 tss
 
 0.037768
 
 0.5299714
 
 Not queried
 
  5.1451996
 
 1.95e-01
 
 0.3826488233
  
 
  
 7
 
  48019388
 
  48019713
 
  PDF   STATS   ENSEMBL   UCSC 
 
  326
 
  4
 
 37590
 
 tss
 
 0.162706
 
 0.8216916
 
 Not queried
 
  5.0068079
 
 2.00e-01
 
 0.3826488233
  
 
  
 7
 
 142583555
 
 142583836
 
  PDF   STATS   ENSEMBL   UCSC 
 
  282
 
  4
 
 37948
 
 tss
 
 0.012480
 
 0.2072602
 
 Not queried
 
  4.8995836
 
 2.00e-01
 
 0.3826488233
  
 
  
 14
 
  51339072
 
  51339216
 
  PDF   STATS   ENSEMBL   UCSC 
 
  145
 
  4
 
 12078
 
 gene;island
 
 0.045052
 
 0.5146187
 
 Not queried
 
  4.7619007
 
 2.00e-01
 
 0.3826488233
  
 
  
 1
 
 110210265
 
 110210641
 
  PDF   STATS   ENSEMBL   UCSC 
 
  377
 
  4
 
 1809
 
 tss
 
 0.036149
 
 0.5720959
 
 Not queried
 
  4.6969730
 
 2.00e-01
 
 0.3826488233
  
 
  
 10
 
   3824387
 
   3824687
 
  PDF   STATS   ENSEMBL   UCSC 
 
  301
 
  4
 
 4476
 
 gene;island
 
 0.027255
 
 0.5177853
 
 Not queried
 
  4.4825836
 
 2.00e-01
 
 0.3826488233
  
 
  
 14
 
 100069535
 
 100069840
 
  PDF   STATS   ENSEMBL   UCSC 
 
  306
 
  4
 
 12204
 
 gene;island
 
 0.360339
 
 0.9276096
 
 Not queried
 
  4.3991893
 
 2.00e-01
 
 0.3826488233
  
 
  
 7
 
 154003215
 
 154003309
 
  PDF   STATS   ENSEMBL   UCSC 
 
   95
 
  4
 
 37303
 
 gene;island
 
 0.101762
 
 0.6972957
 
 Not queried
 
  4.3704097
 
 2.00e-01
 
 0.3826488233
  
 
  
 12
 
 125412894
 
 125413164
 
  PDF   STATS   ENSEMBL   UCSC 
 
  271
 
  4
 
 10979
 
 island
 
 0.035964
 
 0.3342672
 
 Not queried
 
  4.3095668
 
 2.00e-01
 
 0.3826488233
  
 
  
 10
 
  85985296
 
  85985448
 
  PDF   STATS   ENSEMBL   UCSC 
 
  153
 
  4
 
 5193
 
 tss
 
 0.070780
 
 0.7874621
 
 Not queried
 
  4.3080062
 
 2.00e-01
 
 0.3826488233
  
 
  
 17
 
  80330638
 
  80330901
 
  PDF   STATS   ENSEMBL   UCSC 
 
  264
 
  4
 
 18988
 
 island
 
 0.050327
 
 0.5391216
 
 Not queried
 
  4.2648506
 
 2.00e-01
 
 0.3826488233
  
 
  
 1
 
  91182534
 
  91182777
 
  PDF   STATS   ENSEMBL   UCSC 
 
  244
 
  4
 
 625
 
 gene
 
 0.142601
 
 0.5012195
 
 Not queried
 
  4.2619337
 
 2.00e-01
 
 0.3826488233
  
 
  
 12
 
 119419106
 
 119419331
 
  PDF   STATS   ENSEMBL   UCSC 
 
  226
 
  4
 
 10049
 
 tss;island
 
 0.018381
 
 0.0047018
 
 Not queried
 
  4.1916902
 
 2.00e-01
 
 0.3826488233
  
 
  
 17
 
  46657346
 
  46657656
 
  PDF   STATS   ENSEMBL   UCSC 
 
  311
 
  4
 
 17461
 
 tss
 
 0.098104
 
 0.6394960
 
 Not queried
 
  4.1806786
 
 2.00e-01
 
 0.3826488233
  
 
  
 21
 
  34444104
 
  34444382
 
  PDF   STATS   ENSEMBL   UCSC 
 
  279
 
  4
 
 26402
 
 gene;island
 
 0.009564
 
 0.0260145
 
 Not queried
 
  4.1618304
 
 2.00e-01
 
 0.3826488233
  
 
  
 1
 
   3477168
 
   3477442
 
  PDF   STATS   ENSEMBL   UCSC 
 
  275
 
  4
 
 130
 
 gene
 
 0.025479
 
 0.2553422
 
 Not queried
 
  4.1567412
 
 2.00e-01
 
 0.3826488233
  
 
  
 3
 
 191048361
 
 191048439
 
  PDF   STATS   ENSEMBL   UCSC 
 
   79
 
  4
 
 28789
 
 tss
 
 0.001126
 
 0.0197534
 
 Not queried
 
  4.1555046
 
 2.00e-01
 
 0.3826488233
  
 
  
 11
 
 102826469
 
 102826680
 
  PDF   STATS   ENSEMBL   UCSC 
 
  212
 
  4
 
 7650
 
 tss
 
 0.130413
 
 0.8811823
 
 Not queried
 
  4.1473002
 
 2.00e-01
 
 0.3826488233
  
 
  
 5
 
 142066121
 
 142066398
 
  PDF   STATS   ENSEMBL   UCSC 
 
  278
 
  4
 
 31844
 
 gene;tss
 
 0.016096
 
 0.3024416
 
 Not queried
 
  4.1454037
 
 2.00e-01
 
 0.3826488233
  
 
  
 16
 
  29296614
 
  29296797
 
  PDF   STATS   ENSEMBL   UCSC 
 
  184
 
  4
 
 15801
 
 island
 
 0.544783
 
 0.1144939
 
 Not queried
 
  4.1377360
 
 2.00e-01
 
 0.3826488233
  
 
  
 6
 
  33739406
 
  33739653
 
  PDF   STATS   ENSEMBL   UCSC 
 
  248
 
  4
 
 34298
 
 gene;island
 
 0.070155
 
 0.5359286
 
 Not queried
 
  4.1373491
 
 2.00e-01
 
 0.3826488233
  
 
  
 19
 
  15344046
 
  15344364
 
  PDF   STATS   ENSEMBL   UCSC 
 
  319
 
  4
 
 20558
 
 tss;island
 
 0.038474
 
 0.3137285
 
 Not queried
 
  4.1363798
 
 2.00e-01
 
 0.3826488233
  
 
  
 1
 
 202778443
 
 202778869
 
  PDF   STATS   ENSEMBL   UCSC 
 
  427
 
  4
 
 2181
 
 tss;island
 
 0.513659
 
 0.9258786
 
 Not queried
 
  4.1098922
 
 2.00e-01
 
 0.3826488233
  
 
  
 15
 
  30114814
 
  30114912
 
  PDF   STATS   ENSEMBL   UCSC 
 
   99
 
  4
 
 13547
 
 tss
 
 0.356001
 
 0.0381178
 
 Not queried
 
  4.0772678
 
 2.00e-01
 
 0.3826488233
  
 
  
 17
 
  77311577
 
  77311812
 
  PDF   STATS   ENSEMBL   UCSC 
 
  236
 
  4
 
 16839
 
 gene
 
 0.080093
 
 0.4243768
 
 Not queried
 
  4.0337303
 
 2.00e-01
 
 0.3826488233
  
 
  
 5
 
 140749735
 
 140749834
 
  PDF   STATS   ENSEMBL   UCSC 
 
  100
 
  4
 
 31824
 
 gene;tss;island
 
 0.047133
 
 0.4521275
 
 Not queried
 
  4.0172760
 
 2.00e-01
 
 0.3826488233
  
 
  
 12
 
  52585786
 
  52585892
 
  PDF   STATS   ENSEMBL   UCSC 
 
  107
 
  4
 
 9724
 
 tss
 
 0.015878
 
 0.3925901
 
 Not queried
 
  3.9821590
 
 2.00e-01
 
 0.3826488233
  
 
  
 17
 
   9940004
 
   9940321
 
  PDF   STATS   ENSEMBL   UCSC 
 
  318
 
  4
 
 16441
 
 gene
 
 0.486281
 
 0.8820323
 
 Not queried
 
  3.9763590
 
 2.00e-01
 
 0.3826488233
  
 
  
 10
 
 119295619
 
 119295875
 
  PDF   STATS   ENSEMBL   UCSC 
 
  257
 
  4
 
 4783
 
 gene;island
 
 0.510507
 
 0.0397518
 
 Not queried
 
  3.9015693
 
 2.00e-01
 
 0.3826488233
  
 
  
 5
 
 175108269
 
 175108429
 
  PDF   STATS   ENSEMBL   UCSC 
 
  161
 
  4
 
 31907
 
 gene;tss
 
 0.030800
 
 0.4051231
 
 Not queried
 
  3.8858399
 
 2.00e-01
 
 0.3826488233
  
 
  
 2
 
 237076164
 
 237076497
 
  PDF   STATS   ENSEMBL   UCSC 
 
  334
 
  4
 
 23089
 
 gene;island
 
 0.025000
 
 0.2260679
 
 Not queried
 
  3.8510259
 
 2.00e-01
 
 0.3826488233
  
 
  
 6
 
 159290283
 
 159290589
 
  PDF   STATS   ENSEMBL   UCSC 
 
  307
 
  4
 
 36600
 
 island
 
 0.007667
 
 0.0783309
 
 Not queried
 
  3.8346028
 
 2.00e-01
 
 0.3826488233
  
 
  
 13
 
 113776873
 
 113776964
 
  PDF   STATS   ENSEMBL   UCSC 
 
   92
 
  4
 
 11957
 
 island
 
 0.256018
 
 0.7088707
 
 Not queried
 
  3.8189529
 
 2.00e-01
 
 0.3826488233
  
 
  
 7
 
  54610022
 
  54610320
 
  PDF   STATS   ENSEMBL   UCSC 
 
  299
 
  4
 
 37017
 
 gene
 
 0.040272
 
 0.2893149
 
 Not queried
 
  3.7472747
 
 2.00e-01
 
 0.3826488233
  
 
  
 8
 
  80523191
 
  80523364
 
  PDF   STATS   ENSEMBL   UCSC 
 
  174
 
  4
 
 39780
 
 tss
 
 0.081583
 
 0.4500296
 
 Not queried
 
  3.7368353
 
 2.00e-01
 
 0.3826488233
  
 
  
 17
 
   7117125
 
   7117310
 
  PDF   STATS   ENSEMBL   UCSC 
 
  186
 
  4
 
 16383
 
 gene;island
 
 0.069625
 
 0.5451692
 
 Not queried
 
  3.7232531
 
 2.00e-01
 
 0.3826488233
  
 
  
 2
 
 120189867
 
 120190297
 
  PDF   STATS   ENSEMBL   UCSC 
 
  431
 
  4
 
 22846
 
 gene;island
 
 0.042452
 
 0.3765866
 
 Not queried
 
  3.7212804
 
 2.00e-01
 
 0.3826488233
  
 
  
 1
 
  65731689
 
  65731886
 
  PDF   STATS   ENSEMBL   UCSC 
 
  198
 
  4
 
 568
 
 gene;island
 
 0.013867
 
 0.1030364
 
 Not queried
 
  3.7028309
 
 2.00e-01
 
 0.3826488233
  
 
  
 1
 
 182921660
 
 182921857
 
  PDF   STATS   ENSEMBL   UCSC 
 
  198
 
  4
 
 912
 
 gene;island
 
 0.876215
 
 0.3363280
 
 Not queried
 
  3.6841193
 
 2.00e-01
 
 0.3826488233
  
 
  
 6
 
  33138421
 
  33138669
 
  PDF   STATS   ENSEMBL   UCSC 
 
  249
 
  4
 
 34200
 
 gene
 
 0.245873
 
 0.8021846
 
 Not queried
 
  3.6774101
 
 2.00e-01
 
 0.3826488233
  
 
  
 6
 
  31794278
 
  31794519
 
  PDF   STATS   ENSEMBL   UCSC 
 
  242
 
  4
 
 34840
 
 tss;island
 
 0.031943
 
 0.4542175
 
 Not queried
 
  3.6554216
 
 2.00e-01
 
 0.3826488233
  
 
  
 17
 
  68128754
 
  68128923
 
  PDF   STATS   ENSEMBL   UCSC 
 
  170
 
  4
 
 16743
 
 gene
 
 0.339581
 
 0.8093192
 
 Not queried
 
  3.6392431
 
 2.00e-01
 
 0.3826488233
  
 
  
 10
 
  42863508
 
  42863594
 
  PDF   STATS   ENSEMBL   UCSC 
 
   87
 
  4
 
 5039
 
 tss
 
 0.275602
 
 0.0267964
 
 Not queried
 
  3.6337507
 
 2.00e-01
 
 0.3826488233
  
 
  
 11
 
   7695165
 
   7695528
 
  PDF   STATS   ENSEMBL   UCSC 
 
  364
 
  7
 
 7121
 
 tss;island
 
 0.047491
 
 0.3776778
 
 Not queried
 
  3.6181266
 
 1.65e-01
 
 0.3826488233
  
 
  
 19
 
  35940425
 
  35940593
 
  PDF   STATS   ENSEMBL   UCSC 
 
  169
 
  4
 
 20703
 
 tss
 
 0.628681
 
 0.9454819
 
 Not queried
 
  3.5977487
 
 2.00e-01
 
 0.3826488233
  
 
  
 7
 
 150652702
 
 150653195
 
  PDF   STATS   ENSEMBL   UCSC 
 
  494
 
  8
 
 37279
 
 gene;island
 
 0.038011
 
 0.2229411
 
 Not queried
 
  3.5916242
 
 1.95e-01
 
 0.3826488233
  
 
  
 15
 
  26327718
 
  26328092
 
  PDF   STATS   ENSEMBL   UCSC 
 
  375
 
  4
 
 13962
 
 island
 
 0.027222
 
 0.3772468
 
 Not queried
 
  3.5420731
 
 2.00e-01
 
 0.3826488233
  
 
  
 13
 
  49549440
 
  49549665
 
  PDF   STATS   ENSEMBL   UCSC 
 
  226
 
  4
 
 11437
 
 tss;island
 
 0.006744
 
 0.1433550
 
 Not queried
 
  3.5344185
 
 2.00e-01
 
 0.3826488233
  
 
  
 2
 
 137748366
 
 137748460
 
  PDF   STATS   ENSEMBL   UCSC 
 
   95
 
  4
 
 23651
 
 tss
 
 0.023032
 
 0.1586009
 
 Not queried
 
  3.4958919
 
 2.00e-01
 
 0.3826488233
  
 
  
 19
 
  42806146
 
  42806265
 
  PDF   STATS   ENSEMBL   UCSC 
 
  120
 
  7
 
 20823
 
 tss
 
 0.037594
 
 0.2489556
 
 Not queried
 
  3.4923680
 
 1.65e-01
 
 0.3826488233
  
 
  
 2
 
 176952696
 
 176952930
 
  PDF   STATS   ENSEMBL   UCSC 
 
  235
 
  4
 
 24890
 
 island
 
 0.046260
 
 0.2868037
 
 Not queried
 
  3.4527660
 
 2.00e-01
 
 0.3826488233
  
 
  
 4
 
 118006405
 
 118006832
 
  PDF   STATS   ENSEMBL   UCSC 
 
  428
 
  9
 
 31264
 
 island
 
 0.206485
 
 0.0434089
 
 Not queried
 
  3.4437756
 
 1.90e-01
 
 0.3826488233
  
 
  
 14
 
  65005951
 
  65006359
 
  PDF   STATS   ENSEMBL   UCSC 
 
  409
 
 10
 
 12458
 
 tss;island
 
 0.091199
 
 0.5319735
 
 Not queried
 
  3.4314239
 
 1.65e-01
 
 0.3826488233
  
 
  
 6
 
  35732234
 
  35732480
 
  PDF   STATS   ENSEMBL   UCSC 
 
  247
 
  4
 
 36075
 
 island
 
 0.296577
 
 0.0243639
 
 Not queried
 
  3.4132705
 
 2.00e-01
 
 0.3826488233
  
 
  
 15
 
  42500502
 
  42500843
 
  PDF   STATS   ENSEMBL   UCSC 
 
  342
 
  6
 
 13614
 
 tss
 
 0.016822
 
 0.2240995
 
 Not queried
 
  3.3976218
 
 1.80e-01
 
 0.3826488233
  
 
  
 6
 
 166418799
 
 166419138
 
  PDF   STATS   ENSEMBL   UCSC 
 
  340
 
  4
 
 36624
 
 island
 
 0.102080
 
 0.4007129
 
 Not queried
 
  3.3572028
 
 2.00e-01
 
 0.3826488233
  
 
  
 6
 
  32410247
 
  32410459
 
  PDF   STATS   ENSEMBL   UCSC 
 
  213
 
  6
 
 34113
 
 gene
 
 0.733020
 
 0.9822975
 
 Not queried
 
  3.3441759
 
 1.80e-01
 
 0.3826488233
  
 
  
 8
 
   6662963
 
   6663401
 
  PDF   STATS   ENSEMBL   UCSC 
 
  439
 
  4
 
 40052
 
 island
 
 0.196506
 
 0.4973700
 
 Not queried
 
  3.2822283
 
 2.00e-01
 
 0.3826488233
  
 
  
 5
 
 138861832
 
 138861941
 
  PDF   STATS   ENSEMBL   UCSC 
 
  110
 
  4
 
 31777
 
 gene
 
 0.036097
 
 0.3106130
 
 Not queried
 
  3.2658031
 
 2.00e-01
 
 0.3826488233
  
 
  
 10
 
  72648543
 
  72648693
 
  PDF   STATS   ENSEMBL   UCSC 
 
  151
 
  4
 
 5140
 
 tss
 
 0.004727
 
 0.0078073
 
 Not queried
 
  3.2565092
 
 2.00e-01
 
 0.3826488233
  
 
  
 6
 
  30135449
 
  30135671
 
  PDF   STATS   ENSEMBL   UCSC 
 
  223
 
  4
 
 33737
 
 gene
 
 0.056603
 
 0.4247008
 
 Not queried
 
  3.2526753
 
 2.00e-01
 
 0.3826488233
  
 
  
 8
 
 143580770
 
 143581070
 
  PDF   STATS   ENSEMBL   UCSC 
 
  301
 
  4
 
 39463
 
 gene;island
 
 0.912833
 
 0.4944840
 
 Not queried
 
  3.2119633
 
 2.00e-01
 
 0.3826488233
  
 
  
 8
 
  93114150
 
  93114437
 
  PDF   STATS   ENSEMBL   UCSC 
 
  288
 
  4
 
 40449
 
 island
 
 0.071322
 
 0.3781465
 
 Not queried
 
  3.2088412
 
 2.00e-01
 
 0.3826488233
  
 
  
 4
 
  55093834
 
  55094011
 
  PDF   STATS   ENSEMBL   UCSC 
 
  178
 
  4
 
 30400
 
 tss
 
 0.204852
 
 0.0073430
 
 Not queried
 
  3.2071307
 
 2.00e-01
 
 0.3826488233
  
 
  
 5
 
  72746710
 
  72746990
 
  PDF   STATS   ENSEMBL   UCSC 
 
  281
 
  4
 
 32874
 
 island
 
 0.211885
 
 0.0129545
 
 Not queried
 
  3.2008123
 
 2.00e-01
 
 0.3826488233
  
 
  
 8
 
  77585222
 
  77585448
 
  PDF   STATS   ENSEMBL   UCSC 
 
  227
 
  4
 
 39340
 
 gene
 
 0.297623
 
 0.0376278
 
 Not queried
 
  3.1893271
 
 2.00e-01
 
 0.3826488233
  
 
  
 2
 
  63274857
 
  63275178
 
  PDF   STATS   ENSEMBL   UCSC 
 
  322
 
  4
 
 23384
 
 tss
 
 0.181711
 
 0.0354269
 
 Not queried
 
  3.1667230
 
 2.00e-01
 
 0.3826488233
  
 
  
 5
 
 149535646
 
 149535765
 
  PDF   STATS   ENSEMBL   UCSC 
 
  120
 
  4
 
 32425
 
 tss
 
 0.022575
 
 0.2264305
 
 Not queried
 
  3.1663175
 
 2.00e-01
 
 0.3826488233
  
 
  
 12
 
 113909932
 
 113910034
 
  PDF   STATS   ENSEMBL   UCSC 
 
  103
 
  6
 
 10030
 
 tss;island
 
 0.009513
 
 0.1641742
 
 Not queried
 
  3.1657366
 
 1.80e-01
 
 0.3826488233
  
 
  
 16
 
  87447700
 
  87448114
 
  PDF   STATS   ENSEMBL   UCSC 
 
  415
 
  4
 
 14890
 
 gene;island
 
 0.533056
 
 0.9457984
 
 Not queried
 
  3.1432236
 
 2.00e-01
 
 0.3826488233
  
 
  
 11
 
 118842126
 
 118842413
 
  PDF   STATS   ENSEMBL   UCSC 
 
  288
 
  7
 
 7742
 
 tss
 
 0.955158
 
 0.5619925
 
 Not queried
 
  3.1401343
 
 1.65e-01
 
 0.3826488233
  
 
  
 17
 
  80084554
 
  80084751
 
  PDF   STATS   ENSEMBL   UCSC 
 
  198
 
  4
 
 16939
 
 gene;island
 
 0.519052
 
 0.9751829
 
 Not queried
 
  3.1400841
 
 2.00e-01
 
 0.3826488233
  
 
  
 11
 
   2176877
 
   2177018
 
  PDF   STATS   ENSEMBL   UCSC 
 
  142
 
  4
 
 6451
 
 gene;island
 
 0.350977
 
 0.7937380
 
 Not queried
 
  3.1338522
 
 2.00e-01
 
 0.3826488233
  
 
  
 12
 
 106533667
 
 106533903
 
  PDF   STATS   ENSEMBL   UCSC 
 
  237
 
  6
 
 10747
 
 island
 
 0.270738
 
 0.0476903
 
 Not queried
 
  3.1240471
 
 1.80e-01
 
 0.3826488233
  
 
  
 13
 
  92051400
 
  92051786
 
  PDF   STATS   ENSEMBL   UCSC 
 
  387
 
  4
 
 11189
 
 gene;island
 
 0.082483
 
 0.5059692
 
 Not queried
 
  3.1100513
 
 2.00e-01
 
 0.3826488233
  
 
  
 7
 
  93519220
 
  93519621
 
  PDF   STATS   ENSEMBL   UCSC 
 
  402
 
  4
 
 37090
 
 gene;island
 
 0.017189
 
 0.1457377
 
 Not queried
 
  3.0913437
 
 2.00e-01
 
 0.3826488233
  
 
  
 1
 
   7111184
 
   7111367
 
  PDF   STATS   ENSEMBL   UCSC 
 
  184
 
  4
 
 185
 
 gene
 
 0.466286
 
 0.6631403
 
 Not queried
 
  3.0863882
 
 2.00e-01
 
 0.3826488233
  
 
  
 1
 
  65991461
 
  65991765
 
  PDF   STATS   ENSEMBL   UCSC 
 
  305
 
  4
 
 571
 
 gene;island
 
 0.055427
 
 0.3235456
 
 Not queried
 
  3.0440962
 
 2.00e-01
 
 0.3826488233
  
 
  
 19
 
  19383613
 
  19383976
 
  PDF   STATS   ENSEMBL   UCSC 
 
  364
 
  4
 
 19949
 
 gene
 
 0.522827
 
 0.1735715
 
 Not queried
 
  3.0271442
 
 2.00e-01
 
 0.3826488233
  
 
  
 4
 
  52942997
 
  52943247
 
  PDF   STATS   ENSEMBL   UCSC 
 
  251
 
  4
 
 30001
 
 gene;island
 
 0.024595
 
 0.1708308
 
 Not queried
 
  3.0265066
 
 2.00e-01
 
 0.3826488233
  
 
  
 17
 
  46641708
 
  46642104
 
  PDF   STATS   ENSEMBL   UCSC 
 
  397
 
  4
 
 16652
 
 gene;island
 
 0.910287
 
 0.5354375
 
 Not queried
 
  3.0250133
 
 2.00e-01
 
 0.3826488233
  
 
  
 17
 
  16119344
 
  16119456
 
  PDF   STATS   ENSEMBL   UCSC 
 
  113
 
  4
 
 17154
 
 tss;island
 
 0.119434
 
 0.0278802
 
 Not queried
 
  3.0090763
 
 2.00e-01
 
 0.3826488233
  
 
  
 1
 
   1475644
 
   1475742
 
  PDF   STATS   ENSEMBL   UCSC 
 
   99
 
  4
 
 2444
 
 island
 
 0.007740
 
 0.0472580
 
 Not queried
 
  2.9987363
 
 2.00e-01
 
 0.3826488233
  
 
  
 7
 
 100199952
 
 100200009
 
  PDF   STATS   ENSEMBL   UCSC 
 
   58
 
  4
 
 37144
 
 gene
 
 0.572182
 
 0.3226436
 
 Not queried
 
  2.9915570
 
 2.00e-01
 
 0.3826488233
  
 
  
 3
 
 124931627
 
 124931746
 
  PDF   STATS   ENSEMBL   UCSC 
 
  120
 
  4
 
 28550
 
 tss
 
 0.337487
 
 0.0442416
 
 Not queried
 
  2.9814452
 
 2.00e-01
 
 0.3826488233
  
 
  
 3
 
 170746029
 
 170746291
 
  PDF   STATS   ENSEMBL   UCSC 
 
  263
 
  6
 
 29661
 
 island
 
 0.089067
 
 0.0207267
 
 Not queried
 
  2.9803151
 
 1.80e-01
 
 0.3826488233
  
 
  
 8
 
 144242029
 
 144242448
 
  PDF   STATS   ENSEMBL   UCSC 
 
  420
 
  6
 
 39944
 
 tss;island
 
 0.040099
 
 0.2454499
 
 Not queried
 
  2.9775991
 
 1.80e-01
 
 0.3826488233
  
 
  
 17
 
  33787402
 
  33787739
 
  PDF   STATS   ENSEMBL   UCSC 
 
  338
 
  4
 
 18215
 
 island
 
 0.044184
 
 0.3216334
 
 Not queried
 
  2.9542119
 
 2.00e-01
 
 0.3826488233
  
 
  
 16
 
  18995157
 
  18995637
 
  PDF   STATS   ENSEMBL   UCSC 
 
  481
 
  6
 
 15732
 
 island
 
 0.032336
 
 0.0099943
 
 Not queried
 
  2.9262872
 
 1.80e-01
 
 0.3826488233
  
 
  
 10
 
 134751598
 
 134751800
 
  PDF   STATS   ENSEMBL   UCSC 
 
  203
 
  4
 
 4882
 
 gene
 
 0.491085
 
 0.8769495
 
 Not queried
 
  2.8942825
 
 2.00e-01
 
 0.3826488233
  
 
  
 13
 
 112668130
 
 112668501
 
  PDF   STATS   ENSEMBL   UCSC 
 
  372
 
  6
 
 11906
 
 island
 
 0.541126
 
 0.8977076
 
 Not queried
 
  2.8830501
 
 1.80e-01
 
 0.3826488233
  
 
  
 11
 
   1328419
 
   1328622
 
  PDF   STATS   ENSEMBL   UCSC 
 
  204
 
  4
 
 6415
 
 gene;island
 
 0.926341
 
 0.5773456
 
 Not queried
 
  2.8823078
 
 2.00e-01
 
 0.3826488233
  
 
  
 13
 
  79181285
 
  79181613
 
  PDF   STATS   ENSEMBL   UCSC 
 
  329
 
  4
 
 11795
 
 island
 
 0.047554
 
 0.2648847
 
 Not queried
 
  2.8617833
 
 2.00e-01
 
 0.3826488233
  
 
  
 2
 
 220362124
 
 220362242
 
  PDF   STATS   ENSEMBL   UCSC 
 
  119
 
  4
 
 23907
 
 tss
 
 0.044168
 
 0.4139588
 
 Not queried
 
  2.8563717
 
 2.00e-01
 
 0.3826488233
  
 
  
 17
 
  41985144
 
  41985408
 
  PDF   STATS   ENSEMBL   UCSC 
 
  265
 
  4
 
 17396
 
 tss
 
 0.244841
 
 0.0133478
 
 Not queried
 
  2.8560641
 
 2.00e-01
 
 0.3826488233
  
 
  
 17
 
  46799479
 
  46800028
 
  PDF   STATS   ENSEMBL   UCSC 
 
  550
 
  9
 
 17469
 
 tss;island
 
 0.472348
 
 0.0913897
 
 Not queried
 
  2.8551737
 
 1.90e-01
 
 0.3826488233
  
 
  
 1
 
   2040968
 
   2041228
 
  PDF   STATS   ENSEMBL   UCSC 
 
  261
 
  4
 
 60
 
 gene;island
 
 0.411277
 
 0.7409765
 
 Not queried
 
  2.8179110
 
 2.00e-01
 
 0.3826488233
  
 
  
 4
 
  85414016
 
  85414194
 
  PDF   STATS   ENSEMBL   UCSC 
 
  179
 
  4
 
 31159
 
 island
 
 0.219917
 
 0.0340369
 
 Not queried
 
  2.8156101
 
 2.00e-01
 
 0.3826488233
  
 
  
 3
 
 147128998
 
 147129278
 
  PDF   STATS   ENSEMBL   UCSC 
 
  281
 
  4
 
 27989
 
 gene;island
 
 0.488049
 
 0.1165188
 
 Not queried
 
  2.8127445
 
 2.00e-01
 
 0.3826488233
  
 
  
 7
 
  27127448
 
  27127752
 
  PDF   STATS   ENSEMBL   UCSC 
 
  305
 
  4
 
 38334
 
 island
 
 0.566560
 
 0.1958705
 
 Not queried
 
  2.8107294
 
 2.00e-01
 
 0.3826488233
  
 
  
 7
 
 158379132
 
 158379348
 
  PDF   STATS   ENSEMBL   UCSC 
 
  217
 
  4
 
 37379
 
 gene;island
 
 0.158827
 
 0.6910585
 
 Not queried
 
  2.8085239
 
 2.00e-01
 
 0.3826488233
  
 
  
 19
 
  54876728
 
  54876795
 
  PDF   STATS   ENSEMBL   UCSC 
 
   68
 
  4
 
 21060
 
 tss
 
 0.083578
 
 0.3616424
 
 Not queried
 
  2.8074721
 
 2.00e-01
 
 0.3826488233
  
 
  
 11
 
  65123257
 
  65123609
 
  PDF   STATS   ENSEMBL   UCSC 
 
  353
 
  4
 
 6706
 
 gene;island
 
 0.670690
 
 0.2804171
 
 Not queried
 
  2.7844914
 
 2.00e-01
 
 0.3826488233
  
 
  
 8
 
 116681611
 
 116681744
 
  PDF   STATS   ENSEMBL   UCSC 
 
  134
 
  4
 
 39878
 
 tss;island
 
 0.026443
 
 0.0053167
 
 Not queried
 
  2.7838224
 
 2.00e-01
 
 0.3826488233
  
 
  
 15
 
  91423337
 
  91423574
 
  PDF   STATS   ENSEMBL   UCSC 
 
  238
 
  4
 
 13477
 
 gene;island
 
 0.959332
 
 0.6790939
 
 Not queried
 
  2.7827558
 
 2.00e-01
 
 0.3826488233
  
 
  
 10
 
  43047134
 
  43047371
 
  PDF   STATS   ENSEMBL   UCSC 
 
  238
 
  4
 
 4565
 
 gene;island
 
 0.550448
 
 0.9114319
 
 Not queried
 
  2.7709597
 
 2.00e-01
 
 0.3826488233
  
 
  
 21
 
  44088823
 
  44089142
 
  PDF   STATS   ENSEMBL   UCSC 
 
  320
 
  4
 
 26439
 
 gene;island
 
 0.703637
 
 0.9571283
 
 Not queried
 
  2.7633549
 
 2.00e-01
 
 0.3826488233
  
 
  
 1
 
  35395541
 
  35395837
 
  PDF   STATS   ENSEMBL   UCSC 
 
  297
 
  4
 
 3030
 
 island
 
 0.102812
 
 0.0070113
 
 Not queried
 
  2.7421289
 
 2.00e-01
 
 0.3826488233
  
 
  
 20
 
  37353032
 
  37353096
 
  PDF   STATS   ENSEMBL   UCSC 
 
   65
 
  4
 
 25738
 
 tss
 
 0.079979
 
 0.0161984
 
 Not queried
 
  2.7300117
 
 2.00e-01
 
 0.3826488233
  
 
  
 22
 
  31318349
 
  31318546
 
  PDF   STATS   ENSEMBL   UCSC 
 
  198
 
  4
 
 26839
 
 gene
 
 0.063605
 
 0.4078735
 
 Not queried
 
  2.7289630
 
 2.00e-01
 
 0.3826488233
  
 
  
 15
 
  24123232
 
  24123421
 
  PDF   STATS   ENSEMBL   UCSC 
 
  190
 
  4
 
 13949
 
 island
 
 0.333782
 
 0.1185486
 
 Not queried
 
  2.7228205
 
 2.00e-01
 
 0.3826488233
  
 
  
 14
 
 105936300
 
 105936715
 
  PDF   STATS   ENSEMBL   UCSC 
 
  416
 
  4
 
 12265
 
 gene;island
 
 0.755449
 
 0.9825063
 
 Not queried
 
  2.7220960
 
 2.00e-01
 
 0.3826488233
  
 
  
 1
 
  26233332
 
  26233709
 
  PDF   STATS   ENSEMBL   UCSC 
 
  378
 
 11
 
 1380
 
 tss;island
 
 0.419663
 
 0.5929551
 
 Not queried
 
  2.7203489
 
 1.71e-01
 
 0.3826488233
  
 
  
 6
 
  31513571
 
  31513886
 
  PDF   STATS   ENSEMBL   UCSC 
 
  316
 
  4
 
 33897
 
 gene;tss;island
 
 0.321999
 
 0.7930431
 
 Not queried
 
  2.7173596
 
 2.00e-01
 
 0.3826488233
  
 
  
 20
 
  23030102
 
  23030533
 
  PDF   STATS   ENSEMBL   UCSC 
 
  432
 
  7
 
 26048
 
 island
 
 0.010473
 
 0.0450709
 
 Not queried
 
  2.7082180
 
 1.65e-01
 
 0.3826488233
  
 
  
 3
 
  49395574
 
  49395745
 
  PDF   STATS   ENSEMBL   UCSC 
 
  172
 
  4
 
 27781
 
 gene
 
 0.009032
 
 0.0535444
 
 Not queried
 
  2.6870193
 
 2.00e-01
 
 0.3826488233
  
 
  
 20
 
  62151993
 
  62152128
 
  PDF   STATS   ENSEMBL   UCSC 
 
  136
 
  4
 
 25896
 
 tss
 
 0.001100
 
 0.0047149
 
 Not queried
 
  2.6854437
 
 2.00e-01
 
 0.3826488233
  
 
  
 5
 
  72594461
 
  72594851
 
  PDF   STATS   ENSEMBL   UCSC 
 
  391
 
  4
 
 32868
 
 island
 
 0.034780
 
 0.2155144
 
 Not queried
 
  2.6802312
 
 2.00e-01
 
 0.3826488233
  
 
  
 13
 
  28493913
 
  28494161
 
  PDF   STATS   ENSEMBL   UCSC 
 
  249
 
  6
 
 11365
 
 tss
 
 0.447738
 
 0.0738360
 
 Not queried
 
  2.6797794
 
 1.80e-01
 
 0.3826488233
  
 
  
 12
 
 132923339
 
 132923603
 
  PDF   STATS   ENSEMBL   UCSC 
 
  265
 
  4
 
 11045
 
 island
 
 0.042006
 
 0.1285374
 
 Not queried
 
  2.6758414
 
 2.00e-01
 
 0.3826488233
  
 
  
 10
 
  91405265
 
  91405300
 
  PDF   STATS   ENSEMBL   UCSC 
 
   36
 
  4
 
 5216
 
 tss;island
 
 0.116460
 
 0.0142254
 
 Not queried
 
  2.6743096
 
 2.00e-01
 
 0.3826488233
  
 
  
 11
 
  46354803
 
  46355283
 
  PDF   STATS   ENSEMBL   UCSC 
 
  481
 
  4
 
 6597
 
 gene
 
 0.012620
 
 0.2538289
 
 Not queried
 
  2.6703169
 
 2.00e-01
 
 0.3826488233
  
 
  
 20
 
    327607
 
    327811
 
  PDF   STATS   ENSEMBL   UCSC 
 
  205
 
  4
 
 25321
 
 gene;island
 
 0.015880
 
 0.0028658
 
 Not queried
 
  2.6657180
 
 2.00e-01
 
 0.3826488233
  
 
  
 19
 
  15162515
 
  15162971
 
  PDF   STATS   ENSEMBL   UCSC 
 
  457
 
  4
 
 20553
 
 tss
 
 0.050238
 
 0.1819012
 
 Not queried
 
  2.6639489
 
 2.00e-01
 
 0.3826488233
  
 
  
 10
 
 133000130
 
 133000471
 
  PDF   STATS   ENSEMBL   UCSC 
 
  342
 
  6
 
 4843
 
 gene
 
 0.280006
 
 0.6512461
 
 Not queried
 
  2.6593208
 
 1.80e-01
 
 0.3826488233
  
 
  
 7
 
  34873912
 
  34874196
 
  PDF   STATS   ENSEMBL   UCSC 
 
  285
 
  4
 
 36962
 
 gene
 
 0.383490
 
 0.7952754
 
 Not queried
 
  2.6564939
 
 2.00e-01
 
 0.3826488233
  
 
  
 1
 
   3394709
 
   3395053
 
  PDF   STATS   ENSEMBL   UCSC 
 
  345
 
  4
 
 126
 
 gene;island
 
 0.439035
 
 0.7344071
 
 Not queried
 
  2.6540090
 
 2.00e-01
 
 0.3826488233
  
 
  
 11
 
   8102311
 
   8102510
 
  PDF   STATS   ENSEMBL   UCSC 
 
  200
 
  4
 
 6496
 
 gene;tss;island
 
 0.012618
 
 0.0338738
 
 Not queried
 
  2.6502234
 
 2.00e-01
 
 0.3826488233
  
 
  
 1
 
  55504649
 
  55505189
 
  PDF   STATS   ENSEMBL   UCSC 
 
  541
 
 10
 
 1651
 
 tss
 
 0.423977
 
 0.0515203
 
 Not queried
 
  2.6437036
 
 1.65e-01
 
 0.3826488233
  
 
  
 3
 
 168863860
 
 168864313
 
  PDF   STATS   ENSEMBL   UCSC 
 
  454
 
  7
 
 28020
 
 gene;island
 
 0.019983
 
 0.1209651
 
 Not queried
 
  2.6352420
 
 1.65e-01
 
 0.3826488233
  
 
  
 17
 
  37754160
 
  37754424
 
  PDF   STATS   ENSEMBL   UCSC 
 
  265
 
  4
 
 18277
 
 island
 
 0.085326
 
 0.5357091
 
 Not queried
 
  2.6301172
 
 2.00e-01
 
 0.3826488233
  
 
  
 1
 
 155100382
 
 155100511
 
  PDF   STATS   ENSEMBL   UCSC 
 
  130
 
  4
 
 791
 
 gene
 
 0.031607
 
 0.3116428
 
 Not queried
 
  2.6270525
 
 2.00e-01
 
 0.3826488233
  
 
  
 17
 
  32906394
 
  32906622
 
  PDF   STATS   ENSEMBL   UCSC 
 
  229
 
  4
 
 17260
 
 tss
 
 0.004474
 
 0.0176603
 
 Not queried
 
  2.6156357
 
 2.00e-01
 
 0.3826488233
  
 
  
 5
 
 168728149
 
 168728270
 
  PDF   STATS   ENSEMBL   UCSC 
 
  122
 
  4
 
 32480
 
 tss
 
 0.080306
 
 0.0079159
 
 Not queried
 
  2.6126390
 
 2.00e-01
 
 0.3826488233
  
 
  
 8
 
   1960217
 
   1960466
 
  PDF   STATS   ENSEMBL   UCSC 
 
  250
 
  4
 
 40046
 
 island
 
 0.227819
 
 0.6685937
 
 Not queried
 
  2.6122034
 
 2.00e-01
 
 0.3826488233
  
 
  
 5
 
 140305947
 
 140306249
 
  PDF   STATS   ENSEMBL   UCSC 
 
  303
 
  8
 
 32357
 
 tss
 
 0.121438
 
 0.0561376
 
 Not queried
 
  2.6017825
 
 1.95e-01
 
 0.3826488233
  
 
  
 1
 
 110672769
 
 110673319
 
  PDF   STATS   ENSEMBL   UCSC 
 
  551
 
  6
 
 3575
 
 island
 
 0.058464
 
 0.2687691
 
 Not queried
 
  2.5854304
 
 1.80e-01
 
 0.3826488233
  
 
  
 8
 
 124219425
 
 124219725
 
  PDF   STATS   ENSEMBL   UCSC 
 
  301
 
  4
 
 39419
 
 gene;island
 
 0.562113
 
 0.8482687
 
 Not queried
 
  2.5762549
 
 2.00e-01
 
 0.3826488233
  
 
  
 13
 
  79170923
 
  79171230
 
  PDF   STATS   ENSEMBL   UCSC 
 
  308
 
  4
 
 11793
 
 island
 
 0.156723
 
 0.0616192
 
 Not queried
 
  2.5722857
 
 2.00e-01
 
 0.3826488233
  
 
  
 6
 
  29631227
 
  29631447
 
  PDF   STATS   ENSEMBL   UCSC 
 
  221
 
  4
 
 33685
 
 gene
 
 0.407657
 
 0.7034022
 
 Not queried
 
  2.5721041
 
 2.00e-01
 
 0.3826488233
  
 
  
 19
 
  14229237
 
  14229581
 
  PDF   STATS   ENSEMBL   UCSC 
 
  345
 
  4
 
 20540
 
 tss;island
 
 0.035709
 
 0.2267326
 
 Not queried
 
  2.5637530
 
 2.00e-01
 
 0.3826488233
  
 
  
 11
 
  64059307
 
  64059594
 
  PDF   STATS   ENSEMBL   UCSC 
 
  288
 
  4
 
 6678
 
 gene;island
 
 0.003536
 
 0.0054689
 
 Not queried
 
  2.5623084
 
 2.00e-01
 
 0.3826488233
  
 
  
 3
 
  46506104
 
  46506374
 
  PDF   STATS   ENSEMBL   UCSC 
 
  271
 
  4
 
 27749
 
 gene
 
 0.240380
 
 0.0190717
 
 Not queried
 
  2.5590534
 
 2.00e-01
 
 0.3826488233
  
 
  
 2
 
  55277876
 
  55278269
 
  PDF   STATS   ENSEMBL   UCSC 
 
  394
 
  4
 
 23358
 
 tss;island
 
 0.008082
 
 0.0033385
 
 Not queried
 
  2.5572132
 
 2.00e-01
 
 0.3826488233
  
 
  
 13
 
 111089028
 
 111089384
 
  PDF   STATS   ENSEMBL   UCSC 
 
  357
 
  4
 
 11229
 
 gene;island
 
 0.524251
 
 0.8644930
 
 Not queried
 
  2.5537353
 
 2.00e-01
 
 0.3826488233
  
 
  
 17
 
  11501999
 
  11502254
 
  PDF   STATS   ENSEMBL   UCSC 
 
  256
 
  4
 
 16445
 
 gene;island
 
 0.037860
 
 0.2753104
 
 Not queried
 
  2.5433043
 
 2.00e-01
 
 0.3826488233
  
 
  
 15
 
 100533202
 
 100533336
 
  PDF   STATS   ENSEMBL   UCSC 
 
  135
 
  4
 
 13504
 
 gene;island
 
 0.046239
 
 0.3359695
 
 Not queried
 
  2.5400430
 
 2.00e-01
 
 0.3826488233
  
 
  
 10
 
 126694147
 
 126694517
 
  PDF   STATS   ENSEMBL   UCSC 
 
  371
 
  4
 
 4810
 
 gene
 
 0.436017
 
 0.6500064
 
 Not queried
 
  2.5361906
 
 2.00e-01
 
 0.3826488233
  
 
  
 14
 
 103388656
 
 103389022
 
  PDF   STATS   ENSEMBL   UCSC 
 
  367
 
  6
 
 13180
 
 island
 
 0.610187
 
 0.8982798
 
 Not queried
 
  2.5331712
 
 1.80e-01
 
 0.3826488233
  
 
  
 11
 
  64051699
 
  64051814
 
  PDF   STATS   ENSEMBL   UCSC 
 
  116
 
  6
 
 6673
 
 gene;tss;island
 
 0.060043
 
 0.0136480
 
 Not queried
 
  2.5276565
 
 1.80e-01
 
 0.3826488233
  
 
  
 16
 
  49316845
 
  49317169
 
  PDF   STATS   ENSEMBL   UCSC 
 
  325
 
  4
 
 15226
 
 tss
 
 0.017759
 
 0.2232500
 
 Not queried
 
  2.5261755
 
 2.00e-01
 
 0.3826488233
  
 
  
 8
 
 144820683
 
 144820908
 
  PDF   STATS   ENSEMBL   UCSC 
 
  226
 
  4
 
 40685
 
 island
 
 0.253137
 
 0.6071820
 
 Not queried
 
  2.5231022
 
 2.00e-01
 
 0.3826488233
  
 
  
 10
 
 124895638
 
 124895886
 
  PDF   STATS   ENSEMBL   UCSC 
 
  249
 
  4
 
 4802
 
 gene
 
 0.014682
 
 0.0035043
 
 Not queried
 
  2.5164538
 
 2.00e-01
 
 0.3826488233
  
 
  
 18
 
   7011217
 
   7011463
 
  PDF   STATS   ENSEMBL   UCSC 
 
  247
 
  4
 
 19051
 
 gene;island
 
 0.492370
 
 0.8785936
 
 Not queried
 
  2.4996501
 
 2.00e-01
 
 0.3826488233
  
 
  
 2
 
 220412406
 
 220412606
 
  PDF   STATS   ENSEMBL   UCSC 
 
  201
 
  4
 
 23038
 
 gene;island
 
 0.529309
 
 0.7342977
 
 Not queried
 
  2.4929196
 
 2.00e-01
 
 0.3826488233
  
 
  
 10
 
  81679675
 
  81679852
 
  PDF   STATS   ENSEMBL   UCSC 
 
  178
 
  4
 
 5184
 
 tss
 
 0.658993
 
 0.8854888
 
 Not queried
 
  2.4928989
 
 2.00e-01
 
 0.3826488233
  
 
  
 3
 
 194014481
 
 194014928
 
  PDF   STATS   ENSEMBL   UCSC 
 
  448
 
  6
 
 29786
 
 island
 
 0.423510
 
 0.8401943
 
 Not queried
 
  2.4919508
 
 1.80e-01
 
 0.3826488233
  
 
  
 8
 
  11559039
 
  11559488
 
  PDF   STATS   ENSEMBL   UCSC 
 
  450
 
  4
 
 40085
 
 island
 
 0.050359
 
 0.0249222
 
 Not queried
 
  2.4899653
 
 2.00e-01
 
 0.3826488233
  
 
  
 11
 
 101918304
 
 101918710
 
  PDF   STATS   ENSEMBL   UCSC 
 
  407
 
  4
 
 6876
 
 gene
 
 0.060978
 
 0.2725801
 
 Not queried
 
  2.4849511
 
 2.00e-01
 
 0.3826488233
  
 
  
 6
 
 170571686
 
 170572016
 
  PDF   STATS   ENSEMBL   UCSC 
 
  331
 
  8
 
 35354
 
 tss
 
 0.451802
 
 0.5009088
 
 Not queried
 
  2.4826499
 
 1.95e-01
 
 0.3826488233
  
 
  
 12
 
  50677228
 
  50677281
 
  PDF   STATS   ENSEMBL   UCSC 
 
   54
 
  4
 
 9128
 
 gene
 
 0.006374
 
 0.0046811
 
 Not queried
 
  2.4779583
 
 2.00e-01
 
 0.3826488233
  
 
  
 15
 
  47476133
 
  47476482
 
  PDF   STATS   ENSEMBL   UCSC 
 
  350
 
  6
 
 14118
 
 island
 
 0.061104
 
 0.1778840
 
 Not queried
 
  2.4778066
 
 1.80e-01
 
 0.3826488233
  
 
  
 1
 
   7022041
 
   7022284
 
  PDF   STATS   ENSEMBL   UCSC 
 
  244
 
  4
 
 183
 
 gene;island
 
 0.197626
 
 0.5446307
 
 Not queried
 
  2.4723334
 
 2.00e-01
 
 0.3826488233
  
 
  
 16
 
  67232180
 
  67232557
 
  PDF   STATS   ENSEMBL   UCSC 
 
  378
 
  4
 
 14793
 
 gene;tss;island
 
 0.694923
 
 0.4717267
 
 Not queried
 
  2.4680605
 
 2.00e-01
 
 0.3826488233
  
 
  
 1
 
 161697052
 
 161697281
 
  PDF   STATS   ENSEMBL   UCSC 
 
  230
 
  4
 
 855
 
 gene;island
 
 0.004079
 
 0.0008139
 
 Not queried
 
  2.4670702
 
 2.00e-01
 
 0.3826488233
  
 
  
 6
 
  50818685
 
  50819236
 
  PDF   STATS   ENSEMBL   UCSC 
 
  552
 
  4
 
 36200
 
 island
 
 0.007350
 
 0.0307307
 
 Not queried
 
  2.4637402
 
 2.00e-01
 
 0.3826488233
  
 
  
 19
 
  54024649
 
  54024834
 
  PDF   STATS   ENSEMBL   UCSC 
 
  186
 
  4
 
 20205
 
 gene;island
 
 0.231225
 
 0.0556079
 
 Not queried
 
  2.4553183
 
 2.00e-01
 
 0.3826488233
  
 
  
 12
 
  58011764
 
  58011875
 
  PDF   STATS   ENSEMBL   UCSC 
 
  112
 
  4
 
 10567
 
 island
 
 0.419482
 
 0.8086863
 
 Not queried
 
  2.4546202
 
 2.00e-01
 
 0.3826488233
  
 
  
 5
 
 178684416
 
 178684658
 
  PDF   STATS   ENSEMBL   UCSC 
 
  243
 
  4
 
 31931
 
 gene;island
 
 0.532573
 
 0.8428093
 
 Not queried
 
  2.4536749
 
 2.00e-01
 
 0.3826488233
  
 
  
 1
 
  50798944
 
  50799316
 
  PDF   STATS   ENSEMBL   UCSC 
 
  373
 
  4
 
 3251
 
 island
 
 0.011136
 
 0.0525112
 
 Not queried
 
  2.4524617
 
 2.00e-01
 
 0.3826488233
  
 
  
 13
 
 112728799
 
 112728964
 
  PDF   STATS   ENSEMBL   UCSC 
 
  166
 
  4
 
 11914
 
 island
 
 0.012198
 
 0.0425340
 
 Not queried
 
  2.4507906
 
 2.00e-01
 
 0.3826488233
  
 
  
 10
 
 124894943
 
 124895886
 
  PDF   STATS   ENSEMBL   UCSC 
 
  944
 
 13
 
 6215
 
 island
 
 0.043127
 
 0.0112991
 
 Not queried
 
  2.4423127
 
 1.69e-01
 
 0.3826488233
  
 
  
 20
 
    591076
 
    591400
 
  PDF   STATS   ENSEMBL   UCSC 
 
  325
 
  6
 
 25528
 
 tss;island
 
 0.134742
 
 0.5015485
 
 Not queried
 
  2.4266168
 
 1.80e-01
 
 0.3826488233
  
 
  
 2
 
 176977220
 
 176977458
 
  PDF   STATS   ENSEMBL   UCSC 
 
  239
 
  4
 
 24898
 
 island
 
 0.051615
 
 0.3324358
 
 Not queried
 
  2.4264818
 
 2.00e-01
 
 0.3826488233
  
 
  
 11
 
    695873
 
    695896
 
  PDF   STATS   ENSEMBL   UCSC 
 
   24
 
  4
 
 6388
 
 gene;tss;island
 
 0.001820
 
 0.0042941
 
 Not queried
 
  2.3996987
 
 2.00e-01
 
 0.3826488233
  
 
  
 7
 
 158109339
 
 158109694
 
  PDF   STATS   ENSEMBL   UCSC 
 
  356
 
  4
 
 37367
 
 gene;island
 
 0.586084
 
 0.8617274
 
 Not queried
 
  2.3949641
 
 2.00e-01
 
 0.3826488233
  
 
  
 12
 
  14720726
 
  14721288
 
  PDF   STATS   ENSEMBL   UCSC 
 
  563
 
 10
 
 10264
 
 island
 
 0.571683
 
 0.2938349
 
 Not queried
 
  2.3770191
 
 1.90e-01
 
 0.3826488233
  
 
  
 7
 
   1135747
 
   1136023
 
  PDF   STATS   ENSEMBL   UCSC 
 
  277
 
  4
 
 36750
 
 gene;island
 
 0.814985
 
 0.9573961
 
 Not queried
 
  2.3758231
 
 2.00e-01
 
 0.3826488233
  
 
  
 13
 
  84456127
 
  84456486
 
  PDF   STATS   ENSEMBL   UCSC 
 
  360
 
  6
 
 11186
 
 gene
 
 0.137638
 
 0.0308672
 
 Not queried
 
  2.3602001
 
 1.80e-01
 
 0.3826488233
  
 
  
 10
 
  23983496
 
  23983538
 
  PDF   STATS   ENSEMBL   UCSC 
 
   43
 
  4
 
 4989
 
 tss
 
 0.019765
 
 0.1594757
 
 Not queried
 
  2.3588007
 
 2.00e-01
 
 0.3826488233
  
 
  
 6
 
  31622318
 
  31622439
 
  PDF   STATS   ENSEMBL   UCSC 
 
  122
 
  6
 
 34824
 
 tss;island
 
 0.913873
 
 0.7537636
 
 Not queried
 
  2.3539913
 
 1.80e-01
 
 0.3826488233
  
 
  
 16
 
  85362963
 
  85363209
 
  PDF   STATS   ENSEMBL   UCSC 
 
  247
 
  4
 
 16154
 
 island
 
 0.145055
 
 0.4400410
 
 Not queried
 
  2.3480210
 
 2.00e-01
 
 0.3826488233
  
 
  
 8
 
  72459908
 
  72460135
 
  PDF   STATS   ENSEMBL   UCSC 
 
  228
 
  4
 
 40379
 
 island
 
 0.051699
 
 0.2022811
 
 Not queried
 
  2.3433032
 
 2.00e-01
 
 0.3826488233
  
 
  
 2
 
    620044
 
    620310
 
  PDF   STATS   ENSEMBL   UCSC 
 
  267
 
  4
 
 24008
 
 island
 
 0.259969
 
 0.6291734
 
 Not queried
 
  2.3431512
 
 2.00e-01
 
 0.3826488233
  
 
  
 15
 
  99193929
 
  99194172
 
  PDF   STATS   ENSEMBL   UCSC 
 
  244
 
  4
 
 13495
 
 gene;island
 
 0.036045
 
 0.0138735
 
 Not queried
 
  2.3397147
 
 2.00e-01
 
 0.3826488233
  
 
  
 7
 
  45927735
 
  45928326
 
  PDF   STATS   ENSEMBL   UCSC 
 
  592
 
  8
 
 38477
 
 island
 
 0.030099
 
 0.2631327
 
 Not queried
 
  2.3291332
 
 1.95e-01
 
 0.3826488233
  
 
  
 5
 
  15499895
 
  15500270
 
  PDF   STATS   ENSEMBL   UCSC 
 
  376
 
  4
 
 31994
 
 tss;island
 
 0.008922
 
 0.0569322
 
 Not queried
 
  2.3200657
 
 2.00e-01
 
 0.3826488233
  
 
  
 17
 
  36997420
 
  36997740
 
  PDF   STATS   ENSEMBL   UCSC 
 
  321
 
  7
 
 18261
 
 island
 
 0.565503
 
 0.9234333
 
 Not queried
 
  2.3176451
 
 1.65e-01
 
 0.3826488233
  
 
  
 15
 
  91478847
 
  91479184
 
  PDF   STATS   ENSEMBL   UCSC 
 
  338
 
  4
 
 13480
 
 gene;island
 
 0.025551
 
 0.2712620
 
 Not queried
 
  2.3118336
 
 2.00e-01
 
 0.3826488233
  
 
  
 5
 
  19988592
 
  19988944
 
  PDF   STATS   ENSEMBL   UCSC 
 
  353
 
  9
 
 32000
 
 tss
 
 0.022448
 
 0.1554178
 
 Not queried
 
  2.2878440
 
 1.90e-01
 
 0.3826488233
  
 
  
 2
 
 113956255
 
 113956634
 
  PDF   STATS   ENSEMBL   UCSC 
 
  380
 
  4
 
 22833
 
 gene;island
 
 0.742591
 
 0.4761905
 
 Not queried
 
  2.2842634
 
 2.00e-01
 
 0.3826488233
  
 
  
 20
 
   1716270
 
   1716632
 
  PDF   STATS   ENSEMBL   UCSC 
 
  363
 
  4
 
 25939
 
 island
 
 0.960920
 
 0.7525547
 
 Not queried
 
  2.2834086
 
 2.00e-01
 
 0.3826488233
  
 
  
 7
 
  77428232
 
  77428337
 
  PDF   STATS   ENSEMBL   UCSC 
 
  106
 
  4
 
 37061
 
 gene
 
 0.001741
 
 0.0046484
 
 Not queried
 
  2.2795059
 
 2.00e-01
 
 0.3826488233
  
 
  
 2
 
   1746509
 
   1746846
 
  PDF   STATS   ENSEMBL   UCSC 
 
  338
 
  4
 
 22536
 
 gene;island
 
 0.019176
 
 0.0044970
 
 Not queried
 
  2.2738781
 
 2.00e-01
 
 0.3826488233
  
 
  
 3
 
  11034749
 
  11035079
 
  PDF   STATS   ENSEMBL   UCSC 
 
  331
 
  4
 
 27664
 
 gene;island
 
 0.057142
 
 0.2868259
 
 Not queried
 
  2.2738440
 
 2.00e-01
 
 0.3826488233
  
 
  
 5
 
 131892242
 
 131892723
 
  PDF   STATS   ENSEMBL   UCSC 
 
  482
 
  7
 
 33093
 
 island
 
 0.007403
 
 0.0300324
 
 Not queried
 
  2.2716250
 
 1.65e-01
 
 0.3826488233
  
 
  
 6
 
 108436115
 
 108436519
 
  PDF   STATS   ENSEMBL   UCSC 
 
  405
 
  4
 
 36390
 
 island
 
 0.009737
 
 0.0458079
 
 Not queried
 
  2.2594216
 
 2.00e-01
 
 0.3826488233
  
 
  
 19
 
   3671903
 
   3672411
 
  PDF   STATS   ENSEMBL   UCSC 
 
  509
 
  4
 
 19719
 
 gene;island
 
 0.051896
 
 0.1345629
 
 Not queried
 
  2.2580043
 
 2.00e-01
 
 0.3826488233
  
 
  
 5
 
 135364827
 
 135365012
 
  PDF   STATS   ENSEMBL   UCSC 
 
  186
 
  4
 
 31761
 
 gene;island
 
 0.299426
 
 0.5659787
 
 Not queried
 
  2.2548288
 
 2.00e-01
 
 0.3826488233
  
 
  
 4
 
 164087672
 
 164088022
 
  PDF   STATS   ENSEMBL   UCSC 
 
  351
 
  4
 
 30166
 
 gene
 
 0.004986
 
 0.0020024
 
 Not queried
 
  2.2535742
 
 2.00e-01
 
 0.3826488233
  
 
  
 2
 
 133175192
 
 133175397
 
  PDF   STATS   ENSEMBL   UCSC 
 
  206
 
  4
 
 22875
 
 gene;island
 
 0.313894
 
 0.7541624
 
 Not queried
 
  2.2464499
 
 2.00e-01
 
 0.3826488233
  
 
  
 1
 
    848379
 
    848609
 
  PDF   STATS   ENSEMBL   UCSC 
 
  231
 
  4
 
 2378
 
 island
 
 0.217945
 
 0.4279101
 
 Not queried
 
  2.2283461
 
 2.00e-01
 
 0.3826488233
  
 
  
 11
 
    811545
 
    811669
 
  PDF   STATS   ENSEMBL   UCSC 
 
  125
 
  4
 
 7030
 
 tss
 
 0.801168
 
 0.9515020
 
 Not queried
 
  2.2270322
 
 2.00e-01
 
 0.3826488233
  
 
  
 1
 
  95392925
 
  95393138
 
  PDF   STATS   ENSEMBL   UCSC 
 
  214
 
  4
 
 1767
 
 tss;island
 
 0.088006
 
 0.0397724
 
 Not queried
 
  2.2265487
 
 2.00e-01
 
 0.3826488233
  
 
  
 11
 
 115024362
 
 115024626
 
  PDF   STATS   ENSEMBL   UCSC 
 
  265
 
  4
 
 8826
 
 island
 
 0.569389
 
 0.8451969
 
 Not queried
 
  2.2232091
 
 2.00e-01
 
 0.3826488233
  
 
  
 20
 
  61809557
 
  61809841
 
  PDF   STATS   ENSEMBL   UCSC 
 
  285
 
  7
 
 25889
 
 tss
 
 0.063572
 
 0.2519511
 
 Not queried
 
  2.2187832
 
 1.65e-01
 
 0.3826488233
  
 
  
 1
 
  42618891
 
  42619087
 
  PDF   STATS   ENSEMBL   UCSC 
 
  197
 
  4
 
 1546
 
 tss
 
 0.353901
 
 0.6021706
 
 Not queried
 
  2.2141848
 
 2.00e-01
 
 0.3826488233
  
 
  
 17
 
   6797466
 
   6797771
 
  PDF   STATS   ENSEMBL   UCSC 
 
  306
 
  4
 
 16379
 
 gene;island
 
 0.037121
 
 0.2010040
 
 Not queried
 
  2.2114862
 
 2.00e-01
 
 0.3826488233
  
 
  
 3
 
  48700375
 
  48700498
 
  PDF   STATS   ENSEMBL   UCSC 
 
  124
 
  6
 
 28305
 
 tss
 
 0.079189
 
 0.0178739
 
 Not queried
 
  2.2057136
 
 1.80e-01
 
 0.3826488233
  
 
  
 3
 
  40428713
 
  40429014
 
  PDF   STATS   ENSEMBL   UCSC 
 
  302
 
  4
 
 27727
 
 gene
 
 0.050220
 
 0.3417998
 
 Not queried
 
  2.2053520
 
 2.00e-01
 
 0.3826488233
  
 
  
 16
 
  66400320
 
  66400411
 
  PDF   STATS   ENSEMBL   UCSC 
 
   92
 
  4
 
 15279
 
 tss
 
 0.224469
 
 0.5372098
 
 Not queried
 
  2.2028146
 
 2.00e-01
 
 0.3826488233
  
 
  
 17
 
  46698881
 
  46699155
 
  PDF   STATS   ENSEMBL   UCSC 
 
  275
 
  4
 
 16661
 
 gene;island
 
 0.252180
 
 0.6846486
 
 Not queried
 
  2.1856348
 
 2.00e-01
 
 0.3826488233
  
 
  
 20
 
  62688573
 
  62688896
 
  PDF   STATS   ENSEMBL   UCSC 
 
  324
 
  4
 
 25516
 
 gene;island
 
 0.137279
 
 0.3888901
 
 Not queried
 
  2.1841526
 
 2.00e-01
 
 0.3826488233
  
 
  
 20
 
  55904633
 
  55904875
 
  PDF   STATS   ENSEMBL   UCSC 
 
  243
 
  7
 
 26279
 
 island
 
 0.629064
 
 0.8873675
 
 Not queried
 
  2.1818385
 
 1.65e-01
 
 0.3826488233
  
 
  
 1
 
  25868969
 
  25869196
 
  PDF   STATS   ENSEMBL   UCSC 
 
  228
 
  4
 
 1370
 
 tss;island
 
 0.076452
 
 0.5139389
 
 Not queried
 
  2.1804820
 
 2.00e-01
 
 0.3826488233
  
 
  
 20
 
  34359544
 
  34359917
 
  PDF   STATS   ENSEMBL   UCSC 
 
  374
 
  9
 
 25711
 
 tss
 
 0.004329
 
 0.0094883
 
 Not queried
 
  2.1679912
 
 1.61e-01
 
 0.3826488233
  
 
  
 1
 
   9775755
 
   9776133
 
  PDF   STATS   ENSEMBL   UCSC 
 
  379
 
  4
 
 204
 
 gene;island
 
 0.961975
 
 0.9002175
 
 Not queried
 
  2.1639281
 
 2.00e-01
 
 0.3826488233
  
 
  
 10
 
 112256517
 
 112256825
 
  PDF   STATS   ENSEMBL   UCSC 
 
  309
 
  4
 
 5356
 
 tss;island
 
 0.472212
 
 0.8236765
 
 Not queried
 
  2.1585002
 
 2.00e-01
 
 0.3826488233
  
 
  
 11
 
  61335071
 
  61335269
 
  PDF   STATS   ENSEMBL   UCSC 
 
  199
 
  4
 
 6631
 
 gene;island
 
 0.074132
 
 0.0231800
 
 Not queried
 
  2.1549455
 
 2.00e-01
 
 0.3826488233
  
 
  
 14
 
  36983404
 
  36983949
 
  PDF   STATS   ENSEMBL   UCSC 
 
  546
 
  7
 
 12357
 
 tss;island
 
 0.022031
 
 0.1993156
 
 Not queried
 
  2.1542670
 
 1.65e-01
 
 0.3826488233
  
 
  
 16
 
   2198395
 
   2198504
 
  PDF   STATS   ENSEMBL   UCSC 
 
  110
 
  4
 
 15018
 
 tss;island
 
 0.008353
 
 0.0490011
 
 Not queried
 
  2.1503040
 
 2.00e-01
 
 0.3826488233
  
 
  
 14
 
 101830869
 
 101831332
 
  PDF   STATS   ENSEMBL   UCSC 
 
  464
 
  4
 
 13155
 
 island
 
 0.369489
 
 0.7105475
 
 Not queried
 
  2.1480361
 
 2.00e-01
 
 0.3826488233
  
 
  
 19
 
  12780830
 
  12781150
 
  PDF   STATS   ENSEMBL   UCSC 
 
  321
 
  4
 
 19861
 
 gene;tss;island
 
 0.007535
 
 0.0520090
 
 Not queried
 
  2.1337456
 
 2.00e-01
 
 0.3826488233
  
 
  
 5
 
 113697330
 
 113697649
 
  PDF   STATS   ENSEMBL   UCSC 
 
  320
 
  4
 
 32221
 
 tss;island
 
 0.015022
 
 0.0053236
 
 Not queried
 
  2.1322724
 
 2.00e-01
 
 0.3826488233
  
 
  
 7
 
 138552716
 
 138552825
 
  PDF   STATS   ENSEMBL   UCSC 
 
  110
 
  4
 
 37239
 
 gene;island
 
 0.820632
 
 0.9727796
 
 Not queried
 
  2.1190989
 
 2.00e-01
 
 0.3826488233
  
 
  
 17
 
  46800992
 
  46801314
 
  PDF   STATS   ENSEMBL   UCSC 
 
  323
 
  4
 
 16663
 
 gene;tss;island
 
 0.019784
 
 0.1061188
 
 Not queried
 
  2.1146176
 
 2.00e-01
 
 0.3826488233
  
 
  
 17
 
  43222106
 
  43222258
 
  PDF   STATS   ENSEMBL   UCSC 
 
  153
 
  4
 
 18426
 
 island
 
 0.213571
 
 0.5630675
 
 Not queried
 
  2.1143438
 
 2.00e-01
 
 0.3826488233
  
 
  
 14
 
 101520369
 
 101520662
 
  PDF   STATS   ENSEMBL   UCSC 
 
  294
 
  4
 
 12628
 
 tss
 
 0.480577
 
 0.7517739
 
 Not queried
 
  2.1090432
 
 2.00e-01
 
 0.3826488233
  
 
  
 1
 
 223316476
 
 223316927
 
  PDF   STATS   ENSEMBL   UCSC 
 
  452
 
  6
 
 4234
 
 island
 
 0.001549
 
 0.0034145
 
 Not queried
 
  2.1082726
 
 1.80e-01
 
 0.3826488233
  
 
  
 17
 
  78157897
 
  78158248
 
  PDF   STATS   ENSEMBL   UCSC 
 
  352
 
  4
 
 16858
 
 gene;island
 
 0.212289
 
 0.5356309
 
 Not queried
 
  2.1043018
 
 2.00e-01
 
 0.3826488233
  
 
  
 12
 
  52845930
 
  52846088
 
  PDF   STATS   ENSEMBL   UCSC 
 
  159
 
  4
 
 9728
 
 tss
 
 0.096368
 
 0.3825011
 
 Not queried
 
  2.1016721
 
 2.00e-01
 
 0.3826488233
  
 
  
 5
 
  34838922
 
  34839241
 
  PDF   STATS   ENSEMBL   UCSC 
 
  320
 
  8
 
 32013
 
 tss
 
 0.020926
 
 0.0091559
 
 Not queried
 
  2.0987274
 
 1.95e-01
 
 0.3826488233
  
 
  
 1
 
   2415199
 
   2415453
 
  PDF   STATS   ENSEMBL   UCSC 
 
  255
 
  4
 
 86
 
 gene;island
 
 0.227781
 
 0.5409351
 
 Not queried
 
  2.0910383
 
 2.00e-01
 
 0.3826488233
  
 
  
 6
 
  28497278
 
  28497370
 
  PDF   STATS   ENSEMBL   UCSC 
 
   93
 
  4
 
 33635
 
 gene
 
 0.385030
 
 0.7025517
 
 Not queried
 
  2.0903610
 
 2.00e-01
 
 0.3826488233
  
 
  
 7
 
 138483046
 
 138483314
 
  PDF   STATS   ENSEMBL   UCSC 
 
  269
 
  4
 
 37238
 
 gene;tss
 
 0.318246
 
 0.7162874
 
 Not queried
 
  2.0817783
 
 2.00e-01
 
 0.3826488233
  
 
  
 19
 
   9609335
 
   9609704
 
  PDF   STATS   ENSEMBL   UCSC 
 
  370
 
  8
 
 20425
 
 tss
 
 0.084098
 
 0.3939870
 
 Not queried
 
  2.0728562
 
 1.95e-01
 
 0.3826488233
  
 
  
 2
 
  63281069
 
  63281383
 
  PDF   STATS   ENSEMBL   UCSC 
 
  315
 
  4
 
 22704
 
 gene;island
 
 0.037587
 
 0.1677395
 
 Not queried
 
  2.0648844
 
 2.00e-01
 
 0.3826488233
  
 
  
 7
 
 157669880
 
 157670171
 
  PDF   STATS   ENSEMBL   UCSC 
 
  292
 
  4
 
 37349
 
 gene;island
 
 0.039631
 
 0.2512375
 
 Not queried
 
  2.0638632
 
 2.00e-01
 
 0.3826488233
  
 
  
 14
 
 103674159
 
 103674342
 
  PDF   STATS   ENSEMBL   UCSC 
 
  184
 
  4
 
 13184
 
 island
 
 0.474697
 
 0.1587206
 
 Not queried
 
  2.0598162
 
 2.00e-01
 
 0.3826488233
  
 
  
 4
 
   8307757
 
   8308063
 
  PDF   STATS   ENSEMBL   UCSC 
 
  307
 
  4
 
 29950
 
 gene;island
 
 0.784095
 
 0.9373300
 
 Not queried
 
  2.0566867
 
 2.00e-01
 
 0.3826488233
  
 
  
 16
 
  28075183
 
  28075295
 
  PDF   STATS   ENSEMBL   UCSC 
 
  113
 
  4
 
 15153
 
 tss;island
 
 0.031364
 
 0.1247061
 
 Not queried
 
  2.0561190
 
 2.00e-01
 
 0.3826488233
  
 
  
 17
 
  79077169
 
  79077463
 
  PDF   STATS   ENSEMBL   UCSC 
 
  295
 
  4
 
 16893
 
 gene;island
 
 0.540369
 
 0.8194218
 
 Not queried
 
  2.0561031
 
 2.00e-01
 
 0.3826488233
  
 
  
 22
 
  45125218
 
  45125666
 
  PDF   STATS   ENSEMBL   UCSC 
 
  449
 
  4
 
 26895
 
 gene;island
 
 0.478851
 
 0.1826172
 
 Not queried
 
  2.0533395
 
 2.00e-01
 
 0.3826488233
  
 
  
 11
 
 128761146
 
 128761211
 
  PDF   STATS   ENSEMBL   UCSC 
 
   66
 
  6
 
 7807
 
 tss;island
 
 0.010309
 
 0.0048350
 
 Not queried
 
  2.0528001
 
 1.80e-01
 
 0.3826488233
  
 
  
 8
 
 144824625
 
 144824658
 
  PDF   STATS   ENSEMBL   UCSC 
 
   34
 
  4
 
 40686
 
 island
 
 0.524899
 
 0.8216730
 
 Not queried
 
  2.0525787
 
 2.00e-01
 
 0.3826488233
  
 
  
 5
 
  38445522
 
  38445614
 
  PDF   STATS   ENSEMBL   UCSC 
 
   93
 
  4
 
 32029
 
 tss
 
 0.684528
 
 0.9023920
 
 Not queried
 
  2.0516966
 
 2.00e-01
 
 0.3826488233
  
 
  
 1
 
 239550177
 
 239550578
 
  PDF   STATS   ENSEMBL   UCSC 
 
  402
 
  6
 
 4371
 
 island
 
 0.016908
 
 0.0594496
 
 Not queried
 
  2.0501067
 
 1.80e-01
 
 0.3826488233
  
 
  
 19
 
  57049530
 
  57049857
 
  PDF   STATS   ENSEMBL   UCSC 
 
  328
 
  4
 
 21100
 
 tss;island
 
 0.009678
 
 0.0335468
 
 Not queried
 
  2.0458885
 
 2.00e-01
 
 0.3826488233
  
 
  
 7
 
   4921827
 
   4922196
 
  PDF   STATS   ENSEMBL   UCSC 
 
  370
 
  4
 
 36864
 
 gene;island
 
 0.139399
 
 0.2545897
 
 Not queried
 
  2.0457534
 
 2.00e-01
 
 0.3826488233
  
 
  
 15
 
  45406534
 
  45406939
 
  PDF   STATS   ENSEMBL   UCSC 
 
  406
 
  6
 
 13326
 
 gene
 
 0.024136
 
 0.0708683
 
 Not queried
 
  2.0446785
 
 1.80e-01
 
 0.3826488233
  
 
  
 8
 
 144069457
 
 144069888
 
  PDF   STATS   ENSEMBL   UCSC 
 
  432
 
  6
 
 39470
 
 gene
 
 0.458425
 
 0.8325015
 
 Not queried
 
  2.0412581
 
 1.80e-01
 
 0.3826488233
  
 
  
 17
 
   6358363
 
   6358599
 
  PDF   STATS   ENSEMBL   UCSC 
 
  237
 
  4
 
 16373
 
 gene;island
 
 0.279115
 
 0.4731571
 
 Not queried
 
  2.0378788
 
 2.00e-01
 
 0.3826488233
  
 
  
 13
 
 112322853
 
 112323195
 
  PDF   STATS   ENSEMBL   UCSC 
 
  343
 
  4
 
 11902
 
 island
 
 0.547088
 
 0.8224090
 
 Not queried
 
  2.0371887
 
 2.00e-01
 
 0.3826488233
  
 
  
 22
 
  43583726
 
  43583855
 
  PDF   STATS   ENSEMBL   UCSC 
 
  130
 
  4
 
 27203
 
 tss;island
 
 0.002907
 
 0.0084545
 
 Not queried
 
  2.0314157
 
 2.00e-01
 
 0.3826488233
  
 
  
 3
 
 123603306
 
 123603618
 
  PDF   STATS   ENSEMBL   UCSC 
 
  313
 
  6
 
 28547
 
 tss;island
 
 0.016252
 
 0.0340170
 
 Not queried
 
  2.0261185
 
 1.80e-01
 
 0.3826488233
  
 
  
 5
 
 122426096
 
 122426400
 
  PDF   STATS   ENSEMBL   UCSC 
 
  305
 
  4
 
 31729
 
 gene;island
 
 0.214461
 
 0.0502888
 
 Not queried
 
  2.0183579
 
 2.00e-01
 
 0.3826488233
  
 
  
 1
 
  76540438
 
  76540641
 
  PDF   STATS   ENSEMBL   UCSC 
 
  204
 
  4
 
 591
 
 gene;island
 
 0.035708
 
 0.0105034
 
 Not queried
 
  2.0170026
 
 2.00e-01
 
 0.3826488233
  
 
  
 2
 
   3704363
 
   3704793
 
  PDF   STATS   ENSEMBL   UCSC 
 
  431
 
  7
 
 23164
 
 tss
 
 0.074164
 
 0.8879860
 
 Not queried
 
  2.0102992
 
 1.65e-01
 
 0.3826488233
  
 
  
 14
 
 106857188
 
 106857655
 
  PDF   STATS   ENSEMBL   UCSC 
 
  468
 
  4
 
 13247
 
 island
 
 0.874849
 
 0.9697678
 
 Not queried
 
  2.0090324
 
 2.00e-01
 
 0.3826488233
  
 
  
 2
 
 224866411
 
 224866636
 
  PDF   STATS   ENSEMBL   UCSC 
 
  226
 
  6
 
 23054
 
 gene
 
 0.638052
 
 0.8973144
 
 Not queried
 
  2.0067612
 
 1.80e-01
 
 0.3826488233
  
 
  
 4
 
  55093612
 
  55094011
 
  PDF   STATS   ENSEMBL   UCSC 
 
  400
 
  6
 
 31054
 
 island
 
 0.204852
 
 0.0101228
 
 Not queried
 
  1.9960121
 
 1.80e-01
 
 0.3826488233
  
 
  
 17
 
  22191704
 
  22191979
 
  PDF   STATS   ENSEMBL   UCSC 
 
  276
 
  4
 
 18131
 
 island
 
 0.330859
 
 0.6620744
 
 Not queried
 
  1.9937404
 
 2.00e-01
 
 0.3826488233
  
 
  
 6
 
  26987575
 
  26988289
 
  PDF   STATS   ENSEMBL   UCSC 
 
  715
 
 11
 
 33615
 
 gene;island
 
 0.141098
 
 0.4341912
 
 Not queried
 
  1.9931464
 
 1.71e-01
 
 0.3826488233
  
 
  
 16
 
  86912525
 
  86912782
 
  PDF   STATS   ENSEMBL   UCSC 
 
  258
 
  4
 
 16178
 
 island
 
 0.045312
 
 0.1958081
 
 Not queried
 
  1.9918374
 
 2.00e-01
 
 0.3826488233
  
 
  
 21
 
  45232531
 
  45232601
 
  PDF   STATS   ENSEMBL   UCSC 
 
   71
 
  4
 
 26579
 
 tss
 
 0.546439
 
 0.8086574
 
 Not queried
 
  1.9862007
 
 2.00e-01
 
 0.3826488233
  
 
  
 17
 
  56355299
 
  56355431
 
  PDF   STATS   ENSEMBL   UCSC 
 
  133
 
  4
 
 16709
 
 gene;island
 
 0.632258
 
 0.8865600
 
 Not queried
 
  1.9815935
 
 2.00e-01
 
 0.3826488233
  
 
  
 20
 
  46415306
 
  46415859
 
  PDF   STATS   ENSEMBL   UCSC 
 
  554
 
 12
 
 25802
 
 tss;island
 
 0.022024
 
 0.2907706
 
 Not queried
 
  1.9732380
 
 1.78e-01
 
 0.3826488233
  
 
  
 11
 
   6341842
 
   6341908
 
  PDF   STATS   ENSEMBL   UCSC 
 
   67
 
  7
 
 7106
 
 tss
 
 0.012216
 
 0.0335103
 
 Not queried
 
  1.9701614
 
 1.65e-01
 
 0.3826488233
  
 
  
 1
 
 118166013
 
 118166402
 
  PDF   STATS   ENSEMBL   UCSC 
 
  390
 
  4
 
 712
 
 gene
 
 0.601917
 
 0.8251253
 
 Not queried
 
  1.9699048
 
 2.00e-01
 
 0.3826488233
  
 
  
 5
 
 149545907
 
 149546087
 
  PDF   STATS   ENSEMBL   UCSC 
 
  181
 
  4
 
 32426
 
 tss;island
 
 0.720236
 
 0.9427034
 
 Not queried
 
  1.9698354
 
 2.00e-01
 
 0.3826488233
  
 
  
 19
 
  18902081
 
  18902221
 
  PDF   STATS   ENSEMBL   UCSC 
 
  141
 
  6
 
 21794
 
 island
 
 0.232504
 
 0.4916602
 
 Not queried
 
  1.9677400
 
 1.80e-01
 
 0.3826488233
  
 
  
 17
 
  27045043
 
  27045302
 
  PDF   STATS   ENSEMBL   UCSC 
 
  260
 
  6
 
 17218
 
 tss;island
 
 0.019216
 
 0.0696603
 
 Not queried
 
  1.9665676
 
 1.80e-01
 
 0.3826488233
  
 
  
 11
 
  10326100
 
  10326342
 
  PDF   STATS   ENSEMBL   UCSC 
 
  243
 
  4
 
 7142
 
 tss;island
 
 0.028337
 
 0.0766347
 
 Not queried
 
  1.9644638
 
 2.00e-01
 
 0.3826488233
  
 
  
 16
 
  62069806
 
  62070365
 
  PDF   STATS   ENSEMBL   UCSC 
 
  560
 
 11
 
 15985
 
 island
 
 0.237570
 
 0.0805091
 
 Not queried
 
  1.9638824
 
 1.93e-01
 
 0.3826488233
  
 
  
 11
 
   3601010
 
   3601214
 
  PDF   STATS   ENSEMBL   UCSC 
 
  205
 
  4
 
 7998
 
 island
 
 0.460884
 
 0.7276309
 
 Not queried
 
  1.9588350
 
 2.00e-01
 
 0.3826488233
  
 
  
 11
 
  64067685
 
  64067858
 
  PDF   STATS   ENSEMBL   UCSC 
 
  174
 
  6
 
 7394
 
 tss
 
 0.019558
 
 0.0845352
 
 Not queried
 
  1.9520597
 
 1.80e-01
 
 0.3826488233
  
 
  
 16
 
  89557017
 
  89557420
 
  PDF   STATS   ENSEMBL   UCSC 
 
  404
 
  8
 
 15422
 
 tss;island
 
 0.005774
 
 0.0020228
 
 Not queried
 
  1.9482760
 
 1.95e-01
 
 0.3826488233
  
 
  
 5
 
 168195173
 
 168195460
 
  PDF   STATS   ENSEMBL   UCSC 
 
  288
 
  4
 
 31886
 
 gene
 
 0.142621
 
 0.3706011
 
 Not queried
 
  1.9481989
 
 2.00e-01
 
 0.3826488233
  
 
  
 11
 
 134336336
 
 134336476
 
  PDF   STATS   ENSEMBL   UCSC 
 
  141
 
  4
 
 8988
 
 island
 
 0.122970
 
 0.2076573
 
 Not queried
 
  1.9464390
 
 2.00e-01
 
 0.3826488233
  
 
  
 3
 
  62363922
 
  62364390
 
  PDF   STATS   ENSEMBL   UCSC 
 
  469
 
  6
 
 29251
 
 island
 
 0.068207
 
 0.1487169
 
 Not queried
 
  1.9455335
 
 1.80e-01
 
 0.3826488233
  
 
  
 3
 
  71633826
 
  71634044
 
  PDF   STATS   ENSEMBL   UCSC 
 
  219
 
  4
 
 28442
 
 tss;island
 
 0.015010
 
 0.0028965
 
 Not queried
 
  1.9453619
 
 2.00e-01
 
 0.3826488233
  
 
  
 7
 
 117864720
 
 117865112
 
  PDF   STATS   ENSEMBL   UCSC 
 
  393
 
  4
 
 37202
 
 gene
 
 0.612353
 
 0.9218055
 
 Not queried
 
  1.9453280
 
 2.00e-01
 
 0.3826488233
  
 
  
 1
 
 145826877
 
 145827062
 
  PDF   STATS   ENSEMBL   UCSC 
 
  186
 
  4
 
 728
 
 gene;island
 
 0.002695
 
 0.0018718
 
 Not queried
 
  1.9450942
 
 2.00e-01
 
 0.3826488233
  
 
  
 11
 
  94502349
 
  94502879
 
  PDF   STATS   ENSEMBL   UCSC 
 
  531
 
  8
 
 6869
 
 gene;island
 
 0.178995
 
 0.0266060
 
 Not queried
 
  1.9419292
 
 1.95e-01
 
 0.3826488233
  
 
  
 3
 
 150690183
 
 150690456
 
  PDF   STATS   ENSEMBL   UCSC 
 
  274
 
  6
 
 28664
 
 tss
 
 0.472651
 
 0.7477817
 
 Not queried
 
  1.9351931
 
 1.80e-01
 
 0.3826488233
  
 
  
 14
 
  94088174
 
  94088604
 
  PDF   STATS   ENSEMBL   UCSC 
 
  431
 
  4
 
 12191
 
 gene
 
 0.504780
 
 0.8643646
 
 Not queried
 
  1.9236328
 
 2.00e-01
 
 0.3826488233
  
 
  
 21
 
  37442104
 
  37442476
 
  PDF   STATS   ENSEMBL   UCSC 
 
  373
 
  4
 
 26668
 
 island
 
 0.035904
 
 0.0084622
 
 Not queried
 
  1.9233162
 
 2.00e-01
 
 0.3826488233
  
 
  
 15
 
  85113863
 
  85114479
 
  PDF   STATS   ENSEMBL   UCSC 
 
  617
 
  8
 
 14401
 
 island
 
 0.059852
 
 0.0131265
 
 Not queried
 
  1.9225031
 
 1.95e-01
 
 0.3826488233
  
 
  
 11
 
  65657704
 
  65657849
 
  PDF   STATS   ENSEMBL   UCSC 
 
  146
 
  6
 
 7450
 
 tss
 
 0.238271
 
 0.0179440
 
 Not queried
 
  1.9141002
 
 1.80e-01
 
 0.3826488233
  
 
  
 19
 
  54371775
 
  54372062
 
  PDF   STATS   ENSEMBL   UCSC 
 
  288
 
  4
 
 20208
 
 gene;tss;island
 
 0.002634
 
 0.0041618
 
 Not queried
 
  1.9137504
 
 2.00e-01
 
 0.3826488233
  
 
  
 13
 
  50570018
 
  50570250
 
  PDF   STATS   ENSEMBL   UCSC 
 
  233
 
  4
 
 11167
 
 gene;tss;island
 
 0.005574
 
 0.0192182
 
 Not queried
 
  1.9132046
 
 2.00e-01
 
 0.3826488233
  
 
  
 14
 
  96180319
 
  96181044
 
  PDF   STATS   ENSEMBL   UCSC 
 
  726
 
 11
 
 13113
 
 island
 
 0.014896
 
 0.1478445
 
 Not queried
 
  1.9095916
 
 1.93e-01
 
 0.3826488233
  
 
  
 5
 
   2753746
 
   2754322
 
  PDF   STATS   ENSEMBL   UCSC 
 
  577
 
 10
 
 31550
 
 gene;island
 
 0.012552
 
 0.0374693
 
 Not queried
 
  1.8759414
 
 1.90e-01
 
 0.3826488233
  
 
  
 8
 
 116679763
 
 116680127
 
  PDF   STATS   ENSEMBL   UCSC 
 
  365
 
  4
 
 39404
 
 gene;island
 
 0.086707
 
 0.0107011
 
 Not queried
 
  1.8758143
 
 2.00e-01
 
 0.3826488233
  
 
  
 1
 
  99126913
 
  99127446
 
  PDF   STATS   ENSEMBL   UCSC 
 
  534
 
  6
 
 3516
 
 island
 
 0.012565
 
 0.0519440
 
 Not queried
 
  1.8753703
 
 1.80e-01
 
 0.3826488233
  
 
  
 1
 
 236557473
 
 236557758
 
  PDF   STATS   ENSEMBL   UCSC 
 
  286
 
  4
 
 2343
 
 tss;island
 
 0.016643
 
 0.0521670
 
 Not queried
 
  1.8739920
 
 2.00e-01
 
 0.3826488233
  
 
  
 13
 
 114793125
 
 114793396
 
  PDF   STATS   ENSEMBL   UCSC 
 
  272
 
  4
 
 11308
 
 gene
 
 0.862385
 
 0.5574913
 
 Not queried
 
  1.8663169
 
 2.00e-01
 
 0.3826488233
  
 
  
 17
 
  39822429
 
  39822735
 
  PDF   STATS   ENSEMBL   UCSC 
 
  307
 
  4
 
 18312
 
 island
 
 0.003539
 
 0.0023870
 
 Not queried
 
  1.8647085
 
 2.00e-01
 
 0.3826488233
  
 
  
 12
 
 132905611
 
 132906010
 
  PDF   STATS   ENSEMBL   UCSC 
 
  400
 
  4
 
 11042
 
 island
 
 0.062144
 
 0.0387345
 
 Not queried
 
  1.8608550
 
 2.00e-01
 
 0.3826488233
  
 
  
 1
 
 186649559
 
 186649985
 
  PDF   STATS   ENSEMBL   UCSC 
 
  427
 
  7
 
 2146
 
 tss
 
 0.021140
 
 0.0109766
 
 Not queried
 
  1.8608029
 
 1.65e-01
 
 0.3826488233
  
 
  
 5
 
  34838922
 
  34839352
 
  PDF   STATS   ENSEMBL   UCSC 
 
  431
 
  9
 
 32726
 
 island
 
 0.019203
 
 0.0088598
 
 Not queried
 
  1.8602645
 
 1.90e-01
 
 0.3826488233
  
 
  
 12
 
 132976722
 
 132977140
 
  PDF   STATS   ENSEMBL   UCSC 
 
  419
 
  4
 
 11049
 
 island
 
 0.239597
 
 0.0575342
 
 Not queried
 
  1.8527547
 
 2.00e-01
 
 0.3826488233
  
 
  
 12
 
  57410518
 
  57410788
 
  PDF   STATS   ENSEMBL   UCSC 
 
  271
 
  4
 
 9806
 
 tss
 
 0.436808
 
 0.7309983
 
 Not queried
 
  1.8492478
 
 2.00e-01
 
 0.3826488233
  
 
  
 6
 
 170562191
 
 170562720
 
  PDF   STATS   ENSEMBL   UCSC 
 
  530
 
  4
 
 36683
 
 island
 
 0.463732
 
 0.7143391
 
 Not queried
 
  1.8482535
 
 2.00e-01
 
 0.3826488233
  
 
  
 21
 
  15645649
 
  15645988
 
  PDF   STATS   ENSEMBL   UCSC 
 
  340
 
  4
 
 26487
 
 tss
 
 0.368855
 
 0.7494067
 
 Not queried
 
  1.8451590
 
 2.00e-01
 
 0.3826488233
  
 
  
 1
 
  47184735
 
  47185236
 
  PDF   STATS   ENSEMBL   UCSC 
 
  502
 
  6
 
 3227
 
 island
 
 0.002800
 
 0.0084091
 
 Not queried
 
  1.8424228
 
 1.80e-01
 
 0.3826488233
  
 
  
 15
 
  89910632
 
  89910860
 
  PDF   STATS   ENSEMBL   UCSC 
 
  229
 
  4
 
 13886
 
 tss;island
 
 0.104032
 
 0.3103732
 
 Not queried
 
  1.8232052
 
 2.00e-01
 
 0.3826488233
  
 
  
 8
 
 144965352
 
 144965577
 
  PDF   STATS   ENSEMBL   UCSC 
 
  226
 
  4
 
 40696
 
 island
 
 0.130054
 
 0.3492451
 
 Not queried
 
  1.8219015
 
 2.00e-01
 
 0.3826488233
  
 
  
 15
 
  99250795
 
  99251007
 
  PDF   STATS   ENSEMBL   UCSC 
 
  213
 
  4
 
 13496
 
 gene;island
 
 0.608671
 
 0.8591555
 
 Not queried
 
  1.8127379
 
 2.00e-01
 
 0.3826488233
  
 
  
 8
 
  70983567
 
  70983760
 
  PDF   STATS   ENSEMBL   UCSC 
 
  194
 
  4
 
 39757
 
 tss
 
 0.216746
 
 0.0728431
 
 Not queried
 
  1.8087381
 
 2.00e-01
 
 0.3826488233
  
 
  
 3
 
 184529753
 
 184529967
 
  PDF   STATS   ENSEMBL   UCSC 
 
  215
 
  6
 
 29734
 
 island
 
 0.003610
 
 0.0070596
 
 Not queried
 
  1.7998085
 
 1.80e-01
 
 0.3826488233
  
 
  
 8
 
  25042340
 
  25042630
 
  PDF   STATS   ENSEMBL   UCSC 
 
  291
 
  4
 
 39248
 
 gene;island
 
 0.005618
 
 0.0328772
 
 Not queried
 
  1.7986987
 
 2.00e-01
 
 0.3826488233
  
 
  
 14
 
 101532886
 
 101533127
 
  PDF   STATS   ENSEMBL   UCSC 
 
  242
 
  4
 
 13154
 
 island
 
 0.386514
 
 0.6136881
 
 Not queried
 
  1.7913819
 
 2.00e-01
 
 0.3826488233
  
 
  
 11
 
   1104295
 
   1104527
 
  PDF   STATS   ENSEMBL   UCSC 
 
  233
 
  4
 
 7903
 
 island
 
 0.728508
 
 0.9542502
 
 Not queried
 
  1.7868368
 
 2.00e-01
 
 0.3826488233
  
 
  
 1
 
  27240319
 
  27240514
 
  PDF   STATS   ENSEMBL   UCSC 
 
  196
 
  4
 
 347
 
 gene
 
 0.457996
 
 0.7339096
 
 Not queried
 
  1.7836151
 
 2.00e-01
 
 0.3826488233
  
 
  
 5
 
  57755773
 
  57755881
 
  PDF   STATS   ENSEMBL   UCSC 
 
  109
 
  4
 
 31622
 
 gene
 
 0.010178
 
 0.0351831
 
 Not queried
 
  1.7679729
 
 2.00e-01
 
 0.3826488233
  
 
  
 7
 
   3019159
 
   3019382
 
  PDF   STATS   ENSEMBL   UCSC 
 
  224
 
  4
 
 36821
 
 gene
 
 0.034878
 
 0.1160977
 
 Not queried
 
  1.7670612
 
 2.00e-01
 
 0.3826488233
  
 
  
 14
 
  50328815
 
  50328966
 
  PDF   STATS   ENSEMBL   UCSC 
 
  152
 
  4
 
 12826
 
 island
 
 0.001721
 
 0.0095070
 
 Not queried
 
  1.7635612
 
 2.00e-01
 
 0.3826488233
  
 
  
 3
 
  50313996
 
  50314344
 
  PDF   STATS   ENSEMBL   UCSC 
 
  349
 
  4
 
 27799
 
 gene;island
 
 0.909612
 
 0.5980592
 
 Not queried
 
  1.7616669
 
 2.00e-01
 
 0.3826488233
  
 
  
 11
 
 128760367
 
 128760609
 
  PDF   STATS   ENSEMBL   UCSC 
 
  243
 
  4
 
 7806
 
 tss;island
 
 0.079791
 
 0.0260460
 
 Not queried
 
  1.7333589
 
 2.00e-01
 
 0.3826488233
  
 
  
 3
 
  52812367
 
  52812520
 
  PDF   STATS   ENSEMBL   UCSC 
 
  154
 
  4
 
 27825
 
 gene;tss
 
 0.367853
 
 0.7088191
 
 Not queried
 
  1.7308090
 
 2.00e-01
 
 0.3826488233
  
 
  
 3
 
  23244051
 
  23244125
 
  PDF   STATS   ENSEMBL   UCSC 
 
   75
 
  4
 
 28183
 
 tss;island
 
 0.030586
 
 0.1331221
 
 Not queried
 
  1.7292038
 
 2.00e-01
 
 0.3826488233
  
 
  
 18
 
  33877021
 
  33877274
 
  PDF   STATS   ENSEMBL   UCSC 
 
  254
 
  4
 
 19244
 
 tss;island
 
 0.004255
 
 0.0130927
 
 Not queried
 
  1.7261923
 
 2.00e-01
 
 0.3826488233
  
 
  
 11
 
  31839314
 
  31839947
 
  PDF   STATS   ENSEMBL   UCSC 
 
  634
 
  9
 
 8167
 
 island
 
 0.511753
 
 0.1100228
 
 Not queried
 
  1.7199569
 
 1.90e-01
 
 0.3826488233
  
 
  
 6
 
  30860866
 
  30860960
 
  PDF   STATS   ENSEMBL   UCSC 
 
   95
 
  4
 
 33829
 
 gene
 
 0.918512
 
 0.7731777
 
 Not queried
 
  1.7188149
 
 2.00e-01
 
 0.3826488233
  
 
  
 3
 
  10334717
 
  10334760
 
  PDF   STATS   ENSEMBL   UCSC 
 
   44
 
  4
 
 27659
 
 gene;tss
 
 0.036199
 
 0.1064040
 
 Not queried
 
  1.7185844
 
 2.00e-01
 
 0.3826488233
  
 
  
 16
 
  86588418
 
  86588622
 
  PDF   STATS   ENSEMBL   UCSC 
 
  205
 
  4
 
 14886
 
 gene;tss;island
 
 0.004291
 
 0.0203149
 
 Not queried
 
  1.7174983
 
 2.00e-01
 
 0.3826488233
  
 
  
 5
 
 174162510
 
 174162907
 
  PDF   STATS   ENSEMBL   UCSC 
 
  398
 
  4
 
 33417
 
 island
 
 0.049606
 
 0.0272298
 
 Not queried
 
  1.7170030
 
 2.00e-01
 
 0.3826488233
  
 
  
 2
 
 233320486
 
 233320796
 
  PDF   STATS   ENSEMBL   UCSC 
 
  311
 
  4
 
 23949
 
 tss
 
 0.047108
 
 0.1787090
 
 Not queried
 
  1.6950442
 
 2.00e-01
 
 0.3826488233
  
 
  
 2
 
 214016774
 
 214017273
 
  PDF   STATS   ENSEMBL   UCSC 
 
  500
 
  4
 
 23858
 
 tss;island
 
 0.009067
 
 0.0196127
 
 Not queried
 
  1.6928537
 
 2.00e-01
 
 0.3826488233
  
 
  
 5
 
 175200401
 
 175200576
 
  PDF   STATS   ENSEMBL   UCSC 
 
  176
 
  4
 
 33420
 
 island
 
 0.095791
 
 0.0339945
 
 Not queried
 
  1.6882709
 
 2.00e-01
 
 0.3826488233
  
 
  
 2
 
 242751796
 
 242752080
 
  PDF   STATS   ENSEMBL   UCSC 
 
  285
 
  4
 
 23146
 
 gene;island
 
 0.376664
 
 0.6077898
 
 Not queried
 
  1.6853032
 
 2.00e-01
 
 0.3826488233
  
 
  
 6
 
  31845413
 
  31845879
 
  PDF   STATS   ENSEMBL   UCSC 
 
  467
 
  6
 
 33979
 
 gene
 
 0.520646
 
 0.6657655
 
 Not queried
 
  1.6777924
 
 1.80e-01
 
 0.3826488233
  
 
  
 16
 
    751312
 
    751603
 
  PDF   STATS   ENSEMBL   UCSC 
 
  292
 
  4
 
 14540
 
 gene;island
 
 0.029343
 
 0.0961720
 
 Not queried
 
  1.6770149
 
 2.00e-01
 
 0.3826488233
  
 
  
 4
 
  81124484
 
  81124767
 
  PDF   STATS   ENSEMBL   UCSC 
 
  284
 
  4
 
 30035
 
 gene;island
 
 0.037372
 
 0.1415738
 
 Not queried
 
  1.6685948
 
 2.00e-01
 
 0.3826488233
  
 
  
 17
 
  21356245
 
  21356372
 
  PDF   STATS   ENSEMBL   UCSC 
 
  128
 
  4
 
 18121
 
 island
 
 0.270824
 
 0.5413516
 
 Not queried
 
  1.6649768
 
 2.00e-01
 
 0.3826488233
  
 
  
 1
 
   3824232
 
   3824447
 
  PDF   STATS   ENSEMBL   UCSC 
 
  216
 
  4
 
 151
 
 gene;island
 
 0.809980
 
 0.9650583
 
 Not queried
 
  1.6595738
 
 2.00e-01
 
 0.3826488233
  
 
  
 10
 
 135061171
 
 135061360
 
  PDF   STATS   ENSEMBL   UCSC 
 
  190
 
  6
 
 5457
 
 tss
 
 0.583522
 
 0.6760661
 
 Not queried
 
  1.6585169
 
 1.80e-01
 
 0.3826488233
  
 
  
 19
 
  46932069
 
  46932308
 
  PDF   STATS   ENSEMBL   UCSC 
 
  240
 
  4
 
 22172
 
 island
 
 0.116569
 
 0.0253889
 
 Not queried
 
  1.6583669
 
 2.00e-01
 
 0.3826488233
  
 
  
 20
 
  61870691
 
  61871067
 
  PDF   STATS   ENSEMBL   UCSC 
 
  377
 
  4
 
 25498
 
 gene;island
 
 0.665260
 
 0.8892425
 
 Not queried
 
  1.6582913
 
 2.00e-01
 
 0.3826488233
  
 
  
 1
 
   3623319
 
   3623605
 
  PDF   STATS   ENSEMBL   UCSC 
 
  287
 
  4
 
 141
 
 gene;island
 
 0.948857
 
 0.8790048
 
 Not queried
 
  1.6538652
 
 2.00e-01
 
 0.3826488233
  
 
  
 5
 
 138609219
 
 138609700
 
  PDF   STATS   ENSEMBL   UCSC 
 
  482
 
  6
 
 32328
 
 tss
 
 0.007056
 
 0.0251916
 
 Not queried
 
  1.6465010
 
 1.80e-01
 
 0.3826488233
  
 
  
 8
 
  10928985
 
  10929210
 
  PDF   STATS   ENSEMBL   UCSC 
 
  226
 
  4
 
 39203
 
 gene;island
 
 0.680160
 
 0.9095087
 
 Not queried
 
  1.6428089
 
 2.00e-01
 
 0.3826488233
  
 
  
 20
 
   3643863
 
   3644300
 
  PDF   STATS   ENSEMBL   UCSC 
 
  438
 
  6
 
 25972
 
 island
 
 0.450334
 
 0.8022348
 
 Not queried
 
  1.6425562
 
 1.80e-01
 
 0.3826488233
  
 
  
 17
 
  46688182
 
  46688329
 
  PDF   STATS   ENSEMBL   UCSC 
 
  148
 
  4
 
 16659
 
 gene
 
 0.001361
 
 0.0017871
 
 Not queried
 
  1.6343663
 
 2.00e-01
 
 0.3826488233
  
 
  
 11
 
 120894635
 
 120894801
 
  PDF   STATS   ENSEMBL   UCSC 
 
  167
 
  4
 
 7765
 
 tss
 
 0.012953
 
 0.0042195
 
 Not queried
 
  1.6229947
 
 2.00e-01
 
 0.3826488233
  
 
  
 14
 
  74705942
 
  74706336
 
  PDF   STATS   ENSEMBL   UCSC 
 
  395
 
  8
 
 12997
 
 island
 
 0.144956
 
 0.3424674
 
 Not queried
 
  1.6214522
 
 1.95e-01
 
 0.3826488233
  
 
  
 19
 
  41858816
 
  41859304
 
  PDF   STATS   ENSEMBL   UCSC 
 
  489
 
  4
 
 20057
 
 gene;island
 
 0.005006
 
 0.0028399
 
 Not queried
 
  1.6202819
 
 2.00e-01
 
 0.3826488233
  
 
  
 15
 
  69853608
 
  69854024
 
  PDF   STATS   ENSEMBL   UCSC 
 
  417
 
  4
 
 13765
 
 tss
 
 0.150136
 
 0.3784376
 
 Not queried
 
  1.6051961
 
 2.00e-01
 
 0.3826488233
  
 
  
 8
 
  85096835
 
  85097429
 
  PDF   STATS   ENSEMBL   UCSC 
 
  595
 
  9
 
 39352
 
 gene;island
 
 0.023785
 
 0.0433944
 
 Not queried
 
  1.6020771
 
 1.90e-01
 
 0.3826488233
  
 
  
 11
 
  16631866
 
  16632088
 
  PDF   STATS   ENSEMBL   UCSC 
 
  223
 
  4
 
 8090
 
 island
 
 0.623185
 
 0.3920192
 
 Not queried
 
  1.5999070
 
 2.00e-01
 
 0.3826488233
  
 
  
 14
 
  23235611
 
  23235724
 
  PDF   STATS   ENSEMBL   UCSC 
 
  114
 
  4
 
 12287
 
 tss
 
 0.007022
 
 0.0017416
 
 Not queried
 
  1.5990288
 
 2.00e-01
 
 0.3826488233
  
 
  
 13
 
  35515865
 
  35516223
 
  PDF   STATS   ENSEMBL   UCSC 
 
  359
 
  4
 
 11389
 
 tss
 
 0.016825
 
 0.0054476
 
 Not queried
 
  1.5928100
 
 2.00e-01
 
 0.3826488233
  
 
  
 4
 
  80246713
 
  80247088
 
  PDF   STATS   ENSEMBL   UCSC 
 
  376
 
  4
 
 30032
 
 gene
 
 0.208028
 
 0.4359019
 
 Not queried
 
  1.5909603
 
 2.00e-01
 
 0.3826488233
  
 
  
 14
 
  89628141
 
  89628422
 
  PDF   STATS   ENSEMBL   UCSC 
 
  282
 
  4
 
 12169
 
 gene;island
 
 0.979459
 
 0.9252491
 
 Not queried
 
  1.5866008
 
 2.00e-01
 
 0.3826488233
  
 
  
 5
 
   2008188
 
   2008364
 
  PDF   STATS   ENSEMBL   UCSC 
 
  177
 
  4
 
 32650
 
 island
 
 0.656038
 
 0.3570834
 
 Not queried
 
  1.5786621
 
 2.00e-01
 
 0.3826488233
  
 
  
 6
 
  24646213
 
  24646782
 
  PDF   STATS   ENSEMBL   UCSC 
 
  570
 
  8
 
 35494
 
 island
 
 0.014530
 
 0.0047086
 
 Not queried
 
  1.5759317
 
 1.95e-01
 
 0.3826488233
  
 
  
 8
 
  58907018
 
  58907091
 
  PDF   STATS   ENSEMBL   UCSC 
 
   74
 
  4
 
 39718
 
 tss
 
 0.007467
 
 0.0201738
 
 Not queried
 
  1.5737911
 
 2.00e-01
 
 0.3826488233
  
 
  
 11
 
  66059064
 
  66059164
 
  PDF   STATS   ENSEMBL   UCSC 
 
  101
 
  4
 
 7463
 
 tss;island
 
 0.275378
 
 0.5311161
 
 Not queried
 
  1.5733938
 
 2.00e-01
 
 0.3826488233
  
 
  
 11
 
 103908255
 
 103908576
 
  PDF   STATS   ENSEMBL   UCSC 
 
  322
 
  4
 
 6879
 
 gene;island
 
 0.507063
 
 0.7906644
 
 Not queried
 
  1.5710836
 
 2.00e-01
 
 0.3826488233
  
 
  
 7
 
   5535582
 
   5535934
 
  PDF   STATS   ENSEMBL   UCSC 
 
  353
 
  7
 
 37423
 
 tss
 
 0.723852
 
 0.9689442
 
 Not queried
 
  1.5710180
 
 1.65e-01
 
 0.3826488233
  
 
  
 6
 
  10419684
 
  10420079
 
  PDF   STATS   ENSEMBL   UCSC 
 
  396
 
  8
 
 35432
 
 island
 
 0.287516
 
 0.0893138
 
 Not queried
 
  1.5619167
 
 1.95e-01
 
 0.3826488233
  
 
  
 2
 
  23840537
 
  23840763
 
  PDF   STATS   ENSEMBL   UCSC 
 
  227
 
  4
 
 22594
 
 gene
 
 0.933156
 
 0.7497132
 
 Not queried
 
  1.5600705
 
 2.00e-01
 
 0.3826488233
  
 
  
 11
 
  15136059
 
  15136688
 
  PDF   STATS   ENSEMBL   UCSC 
 
  630
 
  9
 
 6521
 
 gene;island
 
 0.084600
 
 0.3242095
 
 Not queried
 
  1.5579949
 
 1.90e-01
 
 0.3826488233
  
 
  
 4
 
 164253207
 
 164253656
 
  PDF   STATS   ENSEMBL   UCSC 
 
  450
 
  4
 
 30167
 
 gene
 
 0.090709
 
 0.0216878
 
 Not queried
 
  1.5566249
 
 2.00e-01
 
 0.3826488233
  
 
  
 8
 
 127569581
 
 127569677
 
  PDF   STATS   ENSEMBL   UCSC 
 
   97
 
  4
 
 39426
 
 gene;island
 
 0.061277
 
 0.2761344
 
 Not queried
 
  1.5549985
 
 2.00e-01
 
 0.3826488233
  
 
  
 3
 
  69788259
 
  69788442
 
  PDF   STATS   ENSEMBL   UCSC 
 
  184
 
  4
 
 28441
 
 tss;island
 
 0.006195
 
 0.0034266
 
 Not queried
 
  1.5479735
 
 2.00e-01
 
 0.3826488233
  
 
  
 20
 
  18568287
 
  18568595
 
  PDF   STATS   ENSEMBL   UCSC 
 
  309
 
  9
 
 26030
 
 island
 
 0.004565
 
 0.0018213
 
 Not queried
 
  1.5425559
 
 1.61e-01
 
 0.3826488233
  
 
  
 1
 
  39269995
 
  39270310
 
  PDF   STATS   ENSEMBL   UCSC 
 
  316
 
  4
 
 3090
 
 island
 
 0.032091
 
 0.0846199
 
 Not queried
 
  1.5390633
 
 2.00e-01
 
 0.3826488233
  
 
  
 5
 
   1409773
 
   1410090
 
  PDF   STATS   ENSEMBL   UCSC 
 
  318
 
  4
 
 31537
 
 gene
 
 0.157161
 
 0.3787684
 
 Not queried
 
  1.5308279
 
 2.00e-01
 
 0.3826488233
  
 
  
 19
 
   5245891
 
   5246048
 
  PDF   STATS   ENSEMBL   UCSC 
 
  158
 
  4
 
 19748
 
 gene;island
 
 0.544990
 
 0.7882884
 
 Not queried
 
  1.5302228
 
 2.00e-01
 
 0.3826488233
  
 
  
 5
 
 128300967
 
 128301185
 
  PDF   STATS   ENSEMBL   UCSC 
 
  219
 
  6
 
 32260
 
 tss
 
 0.009455
 
 0.0214262
 
 Not queried
 
  1.5251326
 
 1.80e-01
 
 0.3826488233
  
 
  
 11
 
   1083178
 
   1083509
 
  PDF   STATS   ENSEMBL   UCSC 
 
  332
 
  6
 
 6402
 
 gene;island
 
 0.351816
 
 0.5159970
 
 Not queried
 
  1.5197277
 
 1.80e-01
 
 0.3826488233
  
 
  
 20
 
  49620366
 
  49620679
 
  PDF   STATS   ENSEMBL   UCSC 
 
  314
 
  4
 
 25453
 
 gene;island
 
 0.173429
 
 0.3859913
 
 Not queried
 
  1.5183860
 
 2.00e-01
 
 0.3826488233
  
 
  
 4
 
 166795260
 
 166795913
 
  PDF   STATS   ENSEMBL   UCSC 
 
  654
 
  9
 
 30173
 
 gene;island
 
 0.015088
 
 0.0478614
 
 Not queried
 
  1.5183451
 
 1.90e-01
 
 0.3826488233
  
 
  
 19
 
   4785298
 
   4785605
 
  PDF   STATS   ENSEMBL   UCSC 
 
  308
 
  4
 
 21388
 
 island
 
 0.068124
 
 0.4465156
 
 Not queried
 
  1.5181592
 
 2.00e-01
 
 0.3826488233
  
 
  
 2
 
 168572517
 
 168572814
 
  PDF   STATS   ENSEMBL   UCSC 
 
  298
 
  4
 
 24834
 
 island
 
 0.667078
 
 0.9296042
 
 Not queried
 
  1.5172115
 
 2.00e-01
 
 0.3826488233
  
 
  
 5
 
   1950701
 
   1951028
 
  PDF   STATS   ENSEMBL   UCSC 
 
  328
 
  4
 
 32648
 
 island
 
 0.707081
 
 0.8732240
 
 Not queried
 
  1.5147988
 
 2.00e-01
 
 0.3826488233
  
 
  
 22
 
  30642820
 
  30643063
 
  PDF   STATS   ENSEMBL   UCSC 
 
  244
 
  4
 
 27056
 
 tss
 
 0.031326
 
 0.0074707
 
 Not queried
 
  1.5110390
 
 2.00e-01
 
 0.3826488233
  
 
  
 16
 
  82660206
 
  82660505
 
  PDF   STATS   ENSEMBL   UCSC 
 
  300
 
  9
 
 15385
 
 tss
 
 0.396685
 
 0.0846439
 
 Not queried
 
  1.5101783
 
 1.61e-01
 
 0.3826488233
  
 
  
 10
 
 133947518
 
 133947916
 
  PDF   STATS   ENSEMBL   UCSC 
 
  399
 
  4
 
 4850
 
 gene;island
 
 0.245135
 
 0.6195486
 
 Not queried
 
  1.5100128
 
 2.00e-01
 
 0.3826488233
  
 
  
 12
 
 125223502
 
 125223679
 
  PDF   STATS   ENSEMBL   UCSC 
 
  178
 
  4
 
 10976
 
 island
 
 0.839916
 
 0.9159065
 
 Not queried
 
  1.5092421
 
 2.00e-01
 
 0.3826488233
  
 
  
 4
 
  54969963
 
  54970254
 
  PDF   STATS   ENSEMBL   UCSC 
 
  292
 
  4
 
 31052
 
 island
 
 0.254845
 
 0.1102809
 
 Not queried
 
  1.5054683
 
 2.00e-01
 
 0.3826488233
  
 
  
 19
 
   4153364
 
   4153603
 
  PDF   STATS   ENSEMBL   UCSC 
 
  240
 
  4
 
 20360
 
 tss
 
 0.068926
 
 0.3156329
 
 Not queried
 
  1.5014368
 
 2.00e-01
 
 0.3826488233
  
 
  
 1
 
 228652338
 
 228652581
 
  PDF   STATS   ENSEMBL   UCSC 
 
  244
 
  4
 
 4302
 
 island
 
 0.011689
 
 0.0359338
 
 Not queried
 
  1.5005026
 
 2.00e-01
 
 0.3826488233
  
 
  
 14
 
  37135384
 
  37135671
 
  PDF   STATS   ENSEMBL   UCSC 
 
  288
 
  4
 
 12068
 
 gene;island
 
 0.160627
 
 0.0659419
 
 Not queried
 
  1.4962546
 
 2.00e-01
 
 0.3826488233
  
 
  
 16
 
   3243304
 
   3243552
 
  PDF   STATS   ENSEMBL   UCSC 
 
  249
 
  4
 
 15641
 
 island
 
 0.959182
 
 0.8087573
 
 Not queried
 
  1.4959841
 
 2.00e-01
 
 0.3826488233
  
 
  
 1
 
 166890293
 
 166890436
 
  PDF   STATS   ENSEMBL   UCSC 
 
  144
 
  4
 
 871
 
 gene;island
 
 0.272893
 
 0.1410157
 
 Not queried
 
  1.4925503
 
 2.00e-01
 
 0.3826488233
  
 
  
 3
 
 167966505
 
 167967220
 
  PDF   STATS   ENSEMBL   UCSC 
 
  716
 
  6
 
 28705
 
 tss;island
 
 0.019935
 
 0.0129735
 
 Not queried
 
  1.4823613
 
 1.80e-01
 
 0.3826488233
  
 
  
 1
 
 180992098
 
 180992117
 
  PDF   STATS   ENSEMBL   UCSC 
 
   20
 
  4
 
 2127
 
 tss
 
 0.001121
 
 0.0006708
 
 Not queried
 
  1.4725514
 
 2.00e-01
 
 0.3826488233
  
 
  
 1
 
   1148973
 
   1149211
 
  PDF   STATS   ENSEMBL   UCSC 
 
  239
 
  4
 
 21
 
 gene;island
 
 0.029832
 
 0.1012860
 
 Not queried
 
  1.4710932
 
 2.00e-01
 
 0.3826488233
  
 
  
 8
 
 143459774
 
 143460209
 
  PDF   STATS   ENSEMBL   UCSC 
 
  436
 
  6
 
 39462
 
 gene
 
 0.827984
 
 0.9266823
 
 Not queried
 
  1.4703194
 
 1.80e-01
 
 0.3826488233
  
 
  
 14
 
 105444384
 
 105445253
 
  PDF   STATS   ENSEMBL   UCSC 
 
  870
 
 10
 
 13224
 
 island
 
 0.015487
 
 0.0646524
 
 Not queried
 
  1.4667444
 
 1.90e-01
 
 0.3826488233
  
 
  
 4
 
  10686394
 
  10686610
 
  PDF   STATS   ENSEMBL   UCSC 
 
  217
 
  4
 
 30301
 
 tss
 
 0.016735
 
 0.0339472
 
 Not queried
 
  1.4602029
 
 2.00e-01
 
 0.3826488233
  
 
  
 6
 
 167370215
 
 167370276
 
  PDF   STATS   ENSEMBL   UCSC 
 
   62
 
  4
 
 35340
 
 tss;island
 
 0.001946
 
 0.0059997
 
 Not queried
 
  1.4563639
 
 2.00e-01
 
 0.3826488233
  
 
  
 7
 
 157791721
 
 157792100
 
  PDF   STATS   ENSEMBL   UCSC 
 
  380
 
  4
 
 37356
 
 gene
 
 0.421483
 
 0.6934692
 
 Not queried
 
  1.4444447
 
 2.00e-01
 
 0.3826488233
  
 
  
 3
 
  63263752
 
  63263832
 
  PDF   STATS   ENSEMBL   UCSC 
 
   81
 
  4
 
 28430
 
 tss;island
 
 0.018991
 
 0.0771020
 
 Not queried
 
  1.4438142
 
 2.00e-01
 
 0.3826488233
  
 
  
 17
 
   7328520
 
   7328921
 
  PDF   STATS   ENSEMBL   UCSC 
 
  402
 
  6
 
 17081
 
 tss
 
 0.279460
 
 0.4931755
 
 Not queried
 
  1.4434998
 
 1.80e-01
 
 0.3826488233
  
 
  
 1
 
 183387128
 
 183387751
 
  PDF   STATS   ENSEMBL   UCSC 
 
  624
 
  6
 
 4031
 
 island
 
 0.050074
 
 0.0178258
 
 Not queried
 
  1.4414122
 
 1.80e-01
 
 0.3826488233
  
 
  
 2
 
 183942982
 
 183943203
 
  PDF   STATS   ENSEMBL   UCSC 
 
  222
 
  4
 
 23769
 
 tss
 
 0.016802
 
 0.0720272
 
 Not queried
 
  1.4397361
 
 2.00e-01
 
 0.3826488233
  
 
  
 19
 
   5455176
 
   5455386
 
  PDF   STATS   ENSEMBL   UCSC 
 
  211
 
  4
 
 20376
 
 tss
 
 0.595355
 
 0.7993355
 
 Not queried
 
  1.4389114
 
 2.00e-01
 
 0.3826488233
  
 
  
 13
 
  28674876
 
  28675056
 
  PDF   STATS   ENSEMBL   UCSC 
 
  181
 
  4
 
 11368
 
 tss
 
 0.011387
 
 0.0246506
 
 Not queried
 
  1.4357943
 
 2.00e-01
 
 0.3826488233
  
 
  
 7
 
   4304779
 
   4305061
 
  PDF   STATS   ENSEMBL   UCSC 
 
  283
 
  4
 
 36838
 
 gene;island
 
 0.359329
 
 0.6048750
 
 Not queried
 
  1.4329978
 
 2.00e-01
 
 0.3826488233
  
 
  
 21
 
  46340776
 
  46341067
 
  PDF   STATS   ENSEMBL   UCSC 
 
  292
 
  7
 
 26460
 
 gene
 
 0.011155
 
 0.0297641
 
 Not queried
 
  1.4327251
 
 1.65e-01
 
 0.3826488233
  
 
  
 2
 
 242499273
 
 242499542
 
  PDF   STATS   ENSEMBL   UCSC 
 
  270
 
  4
 
 23136
 
 gene;island
 
 0.557243
 
 0.4745764
 
 Not queried
 
  1.4323707
 
 2.00e-01
 
 0.3826488233
  
 
  
 10
 
  28034792
 
  28034856
 
  PDF   STATS   ENSEMBL   UCSC 
 
   65
 
  6
 
 5004
 
 tss
 
 0.099747
 
 0.3413192
 
 Not queried
 
  1.4313906
 
 1.80e-01
 
 0.3826488233
  
 
  
 7
 
  70597458
 
  70597778
 
  PDF   STATS   ENSEMBL   UCSC 
 
  321
 
  6
 
 37627
 
 tss
 
 0.250653
 
 0.1453060
 
 Not queried
 
  1.4258044
 
 1.80e-01
 
 0.3826488233
  
 
  
 22
 
  48977177
 
  48977448
 
  PDF   STATS   ENSEMBL   UCSC 
 
  272
 
  4
 
 26919
 
 gene;island
 
 0.496340
 
 0.7375622
 
 Not queried
 
  1.4214457
 
 2.00e-01
 
 0.3826488233
  
 
  
 7
 
  98099806
 
  98100419
 
  PDF   STATS   ENSEMBL   UCSC 
 
  614
 
  7
 
 38713
 
 island
 
 0.110714
 
 0.0256934
 
 Not queried
 
  1.4191331
 
 1.65e-01
 
 0.3826488233
  
 
  
 7
 
 157447022
 
 157447249
 
  PDF   STATS   ENSEMBL   UCSC 
 
  228
 
  4
 
 37335
 
 gene;island
 
 0.931057
 
 0.9635898
 
 Not queried
 
  1.4132421
 
 2.00e-01
 
 0.3826488233
  
 
  
 11
 
  47359839
 
  47360291
 
  PDF   STATS   ENSEMBL   UCSC 
 
  453
 
  6
 
 6606
 
 gene;island
 
 0.556343
 
 0.7094522
 
 Not queried
 
  1.4102397
 
 1.80e-01
 
 0.3826488233
  
 
  
 20
 
   4702381
 
   4702538
 
  PDF   STATS   ENSEMBL   UCSC 
 
  158
 
  4
 
 25575
 
 tss
 
 0.616359
 
 0.8038331
 
 Not queried
 
  1.4093707
 
 2.00e-01
 
 0.3826488233
  
 
  
 19
 
  36035831
 
  36036387
 
  PDF   STATS   ENSEMBL   UCSC 
 
  557
 
  7
 
 20707
 
 tss
 
 0.018647
 
 0.0101604
 
 Not queried
 
  1.4072375
 
 1.65e-01
 
 0.3826488233
  
 
  
 19
 
  41882232
 
  41882327
 
  PDF   STATS   ENSEMBL   UCSC 
 
   96
 
  4
 
 20059
 
 gene;tss;island
 
 0.020995
 
 0.0504424
 
 Not queried
 
  1.4063569
 
 2.00e-01
 
 0.3826488233
  
 
  
 15
 
  91427821
 
  91428056
 
  PDF   STATS   ENSEMBL   UCSC 
 
  236
 
  5
 
 13478
 
 gene;tss;island
 
 0.012881
 
 0.1703054
 
 Not queried
 
  6.2270055
 
 2.22e-01
 
 0.3935758299
  
 
  
 17
 
  17109570
 
  17110353
 
  PDF   STATS   ENSEMBL   UCSC 
 
  784
 
 14
 
 18060
 
 island
 
 0.016359
 
 0.4901897
 
 Not queried
 
  4.8192938
 
 2.10e-01
 
 0.3935758299
  
 
  
 22
 
  45073031
 
  45073165
 
  PDF   STATS   ENSEMBL   UCSC 
 
  135
 
  5
 
 26893
 
 gene
 
 0.008589
 
 0.2915127
 
 Not queried
 
  4.7221284
 
 2.22e-01
 
 0.3935758299
  
 
  
 3
 
  42947263
 
  42947565
 
  PDF   STATS   ENSEMBL   UCSC 
 
  303
 
  5
 
 28250
 
 tss
 
 0.181297
 
 0.5223723
 
 Not queried
 
  4.4843531
 
 2.22e-01
 
 0.3935758299
  
 
  
 6
 
  27647713
 
  27648057
 
  PDF   STATS   ENSEMBL   UCSC 
 
  345
 
  5
 
 35560
 
 island
 
 0.086451
 
 0.0294615
 
 Not queried
 
  4.3774324
 
 2.22e-01
 
 0.3935758299
  
 
  
 14
 
  69256677
 
  69256977
 
  PDF   STATS   ENSEMBL   UCSC 
 
  301
 
  5
 
 12121
 
 gene;island
 
 0.004557
 
 0.0146637
 
 Not queried
 
  4.2654751
 
 2.22e-01
 
 0.3935758299
  
 
  
 20
 
   1294301
 
   1294376
 
  PDF   STATS   ENSEMBL   UCSC 
 
   76
 
  5
 
 25328
 
 gene;island
 
 0.003802
 
 0.0192056
 
 Not queried
 
  4.2579894
 
 2.22e-01
 
 0.3935758299
  
 
  
 6
 
  27259010
 
  27259237
 
  PDF   STATS   ENSEMBL   UCSC 
 
  228
 
  5
 
 35553
 
 island
 
 0.522824
 
 0.0400349
 
 Not queried
 
  4.2385788
 
 2.22e-01
 
 0.3935758299
  
 
  
 10
 
    636076
 
    636390
 
  PDF   STATS   ENSEMBL   UCSC 
 
  315
 
  5
 
 4444
 
 gene
 
 0.046241
 
 0.1281579
 
 Not queried
 
  3.9480335
 
 2.22e-01
 
 0.3935758299
  
 
  
 6
 
  31513198
 
  31513306
 
  PDF   STATS   ENSEMBL   UCSC 
 
  109
 
  5
 
 33896
 
 gene;tss;island
 
 0.023645
 
 0.4710397
 
 Not queried
 
  3.9471680
 
 2.22e-01
 
 0.3935758299
  
 
  
 6
 
  32073324
 
  32073700
 
  PDF   STATS   ENSEMBL   UCSC 
 
  377
 
  5
 
 34048
 
 gene
 
 0.479689
 
 0.6848821
 
 Not queried
 
  3.9435048
 
 2.22e-01
 
 0.3935758299
  
 
  
 5
 
  79330669
 
  79330959
 
  PDF   STATS   ENSEMBL   UCSC 
 
  291
 
  5
 
 32148
 
 tss
 
 0.522758
 
 0.0234624
 
 Not queried
 
  3.8186070
 
 2.22e-01
 
 0.3935758299
  
 
  
 16
 
  18995157
 
  18995532
 
  PDF   STATS   ENSEMBL   UCSC 
 
  376
 
  5
 
 15112
 
 tss
 
 0.044428
 
 0.0113492
 
 Not queried
 
  3.7362517
 
 2.22e-01
 
 0.3935758299
  
 
  
 12
 
 106533840
 
 106533903
 
  PDF   STATS   ENSEMBL   UCSC 
 
   64
 
  5
 
 9966
 
 tss
 
 0.248463
 
 0.0290661
 
 Not queried
 
  3.6951852
 
 2.22e-01
 
 0.3935758299
  
 
  
 1
 
 202183264
 
 202183605
 
  PDF   STATS   ENSEMBL   UCSC 
 
  342
 
  5
 
 945
 
 gene;island
 
 0.155809
 
 0.4957891
 
 Not queried
 
  3.6498809
 
 2.22e-01
 
 0.3935758299
  
 
  
 6
 
  33397511
 
  33397959
 
  PDF   STATS   ENSEMBL   UCSC 
 
  449
 
  5
 
 34289
 
 gene;island
 
 0.027653
 
 0.0728588
 
 Not queried
 
  3.5903729
 
 2.22e-01
 
 0.3935758299
  
 
  
 11
 
  65816463
 
  65816985
 
  PDF   STATS   ENSEMBL   UCSC 
 
  523
 
 10
 
 8497
 
 island
 
 0.105831
 
 0.5137191
 
 Not queried
 
  3.3847085
 
 2.18e-01
 
 0.3935758299
  
 
  
 10
 
  95517382
 
  95517562
 
  PDF   STATS   ENSEMBL   UCSC 
 
  181
 
  5
 
 5242
 
 tss
 
 0.026427
 
 0.0031403
 
 Not queried
 
  3.3729999
 
 2.22e-01
 
 0.3935758299
  
 
  
 10
 
  43846281
 
  43846705
 
  PDF   STATS   ENSEMBL   UCSC 
 
  425
 
  5
 
 5694
 
 island
 
 0.772136
 
 0.2703340
 
 Not queried
 
  3.3611356
 
 2.22e-01
 
 0.3935758299
  
 
  
 4
 
 104640489
 
 104640833
 
  PDF   STATS   ENSEMBL   UCSC 
 
  345
 
  5
 
 30078
 
 gene;island
 
 0.047137
 
 0.3812198
 
 Not queried
 
  3.3606443
 
 2.22e-01
 
 0.3935758299
  
 
  
 5
 
 149546331
 
 149546471
 
  PDF   STATS   ENSEMBL   UCSC 
 
  141
 
  5
 
 33304
 
 island
 
 0.678275
 
 0.9646446
 
 Not queried
 
  3.2924389
 
 2.22e-01
 
 0.3935758299
  
 
  
 20
 
  23030343
 
  23030533
 
  PDF   STATS   ENSEMBL   UCSC 
 
  191
 
  5
 
 25636
 
 tss
 
 0.007082
 
 0.0172442
 
 Not queried
 
  3.1978175
 
 2.22e-01
 
 0.3935758299
  
 
  
 10
 
 135039948
 
 135040250
 
  PDF   STATS   ENSEMBL   UCSC 
 
  303
 
  5
 
 6345
 
 island
 
 0.070367
 
 0.4247312
 
 Not queried
 
  3.1763887
 
 2.22e-01
 
 0.3935758299
  
 
  
 5
 
 162930177
 
 162930325
 
  PDF   STATS   ENSEMBL   UCSC 
 
  149
 
  5
 
 33365
 
 island
 
 0.017820
 
 0.0056229
 
 Not queried
 
  3.1634598
 
 2.22e-01
 
 0.3935758299
  
 
  
 2
 
 242808427
 
 242808907
 
  PDF   STATS   ENSEMBL   UCSC 
 
  481
 
  5
 
 25312
 
 island
 
 0.138288
 
 0.5336316
 
 Not queried
 
  3.0927595
 
 2.22e-01
 
 0.3935758299
  
 
  
 6
 
  87647370
 
  87647644
 
  PDF   STATS   ENSEMBL   UCSC 
 
  275
 
  5
 
 34408
 
 gene;island
 
 0.057945
 
 0.3851937
 
 Not queried
 
  3.0807045
 
 2.22e-01
 
 0.3935758299
  
 
  
 22
 
  26875552
 
  26875823
 
  PDF   STATS   ENSEMBL   UCSC 
 
  272
 
  5
 
 27033
 
 tss
 
 0.083770
 
 0.0218832
 
 Not queried
 
  3.0768793
 
 2.22e-01
 
 0.3935758299
  
 
  
 16
 
  86546628
 
  86546979
 
  PDF   STATS   ENSEMBL   UCSC 
 
  352
 
  5
 
 14884
 
 gene;island
 
 0.371148
 
 0.0472291
 
 Not queried
 
  3.0653097
 
 2.22e-01
 
 0.3935758299
  
 
  
 17
 
  75789279
 
  75789529
 
  PDF   STATS   ENSEMBL   UCSC 
 
  251
 
  5
 
 18812
 
 island
 
 0.506372
 
 0.7702039
 
 Not queried
 
  3.0440209
 
 2.22e-01
 
 0.3935758299
  
 
  
 7
 
   1022841
 
   1023156
 
  PDF   STATS   ENSEMBL   UCSC 
 
  316
 
  5
 
 36730
 
 gene
 
 0.704845
 
 0.9108346
 
 Not queried
 
  3.0098392
 
 2.22e-01
 
 0.3935758299
  
 
  
 8
 
  16859295
 
  16859671
 
  PDF   STATS   ENSEMBL   UCSC 
 
  377
 
  5
 
 39214
 
 gene
 
 0.070643
 
 0.4472376
 
 Not queried
 
  2.9645102
 
 2.22e-01
 
 0.3935758299
  
 
  
 20
 
  30605656
 
  30606095
 
  PDF   STATS   ENSEMBL   UCSC 
 
  440
 
  5
 
 25387
 
 gene;island
 
 0.046617
 
 0.0289826
 
 Not queried
 
  2.9620816
 
 2.22e-01
 
 0.3935758299
  
 
  
 6
 
  30460244
 
  30460798
 
  PDF   STATS   ENSEMBL   UCSC 
 
  555
 
  7
 
 33771
 
 gene;island
 
 0.532756
 
 0.9459607
 
 Not queried
 
  2.8739428
 
 2.09e-01
 
 0.3935758299
  
 
  
 4
 
  74718978
 
  74719289
 
  PDF   STATS   ENSEMBL   UCSC 
 
  312
 
  5
 
 31096
 
 island
 
 0.314276
 
 0.0591095
 
 Not queried
 
  2.8668797
 
 2.22e-01
 
 0.3935758299
  
 
  
 7
 
  56297490
 
  56297714
 
  PDF   STATS   ENSEMBL   UCSC 
 
  225
 
  5
 
 38513
 
 island
 
 0.109067
 
 0.0906536
 
 Not queried
 
  2.8432259
 
 2.22e-01
 
 0.3935758299
  
 
  
 8
 
 133573739
 
 133574073
 
  PDF   STATS   ENSEMBL   UCSC 
 
  335
 
  5
 
 39919
 
 tss
 
 0.003771
 
 0.1937665
 
 Not queried
 
  2.8085773
 
 2.22e-01
 
 0.3935758299
  
 
  
 12
 
  14720805
 
  14721288
 
  PDF   STATS   ENSEMBL   UCSC 
 
  484
 
  9
 
 9584
 
 tss
 
 0.887066
 
 0.4882384
 
 Not queried
 
  2.7890280
 
 2.22e-01
 
 0.3935758299
  
 
  
 16
 
  56554066
 
  56554313
 
  PDF   STATS   ENSEMBL   UCSC 
 
  248
 
  5
 
 15245
 
 tss
 
 0.230353
 
 0.9056949
 
 Not queried
 
  2.7213610
 
 2.22e-01
 
 0.3935758299
  
 
  
 6
 
  32810551
 
  32810833
 
  PDF   STATS   ENSEMBL   UCSC 
 
  283
 
  5
 
 34150
 
 gene;island
 
 0.089046
 
 0.2779585
 
 Not queried
 
  2.6759930
 
 2.22e-01
 
 0.3935758299
  
 
  
 12
 
 129337674
 
 129337910
 
  PDF   STATS   ENSEMBL   UCSC 
 
  237
 
  5
 
 10116
 
 tss;island
 
 0.025085
 
 0.4109776
 
 Not queried
 
  2.6391412
 
 2.22e-01
 
 0.3935758299
  
 
  
 11
 
  27015656
 
  27015991
 
  PDF   STATS   ENSEMBL   UCSC 
 
  336
 
  5
 
 6541
 
 gene
 
 0.120775
 
 0.4554488
 
 Not queried
 
  2.6065390
 
 2.22e-01
 
 0.3935758299
  
 
  
 7
 
   1280040
 
   1280640
 
  PDF   STATS   ENSEMBL   UCSC 
 
  601
 
  5
 
 38094
 
 island
 
 0.014360
 
 0.1141960
 
 Not queried
 
  2.5848599
 
 2.22e-01
 
 0.3935758299
  
 
  
 20
 
  35972924
 
  35973098
 
  PDF   STATS   ENSEMBL   UCSC 
 
  175
 
  5
 
 25726
 
 tss;island
 
 0.171898
 
 0.4578843
 
 Not queried
 
  2.5801553
 
 2.22e-01
 
 0.3935758299
  
 
  
 12
 
  93836052
 
  93836653
 
  PDF   STATS   ENSEMBL   UCSC 
 
  602
 
  9
 
 9915
 
 tss
 
 0.004833
 
 0.0034309
 
 Not queried
 
  2.5795035
 
 2.22e-01
 
 0.3935758299
  
 
  
 17
 
   9478950
 
   9479224
 
  PDF   STATS   ENSEMBL   UCSC 
 
  275
 
  5
 
 16436
 
 gene
 
 0.003881
 
 0.0094686
 
 Not queried
 
  2.5372104
 
 2.22e-01
 
 0.3935758299
  
 
  
 4
 
 174452835
 
 174453287
 
  PDF   STATS   ENSEMBL   UCSC 
 
  453
 
  5
 
 30181
 
 gene;island
 
 0.017926
 
 0.2769595
 
 Not queried
 
  2.4992828
 
 2.22e-01
 
 0.3935758299
  
 
  
 19
 
  13976610
 
  13976956
 
  PDF   STATS   ENSEMBL   UCSC 
 
  347
 
  5
 
 21660
 
 island
 
 0.009371
 
 0.0214785
 
 Not queried
 
  2.4929141
 
 2.22e-01
 
 0.3935758299
  
 
  
 8
 
 145925708
 
 145926089
 
  PDF   STATS   ENSEMBL   UCSC 
 
  382
 
  5
 
 40746
 
 island
 
 0.378131
 
 0.2338769
 
 Not queried
 
  2.4789050
 
 2.22e-01
 
 0.3935758299
  
 
  
 5
 
  98104747
 
  98105150
 
  PDF   STATS   ENSEMBL   UCSC 
 
  404
 
  5
 
 32981
 
 island
 
 0.017657
 
 0.0046316
 
 Not queried
 
  2.4734926
 
 2.22e-01
 
 0.3935758299
  
 
  
 2
 
  74669349
 
  74669516
 
  PDF   STATS   ENSEMBL   UCSC 
 
  168
 
  5
 
 23454
 
 tss;island
 
 0.559135
 
 0.7124505
 
 Not queried
 
  2.4702430
 
 2.22e-01
 
 0.3935758299
  
 
  
 17
 
  40086714
 
  40086869
 
  PDF   STATS   ENSEMBL   UCSC 
 
  156
 
  5
 
 17349
 
 tss
 
 0.006188
 
 0.0047420
 
 Not queried
 
  2.4646483
 
 2.22e-01
 
 0.3935758299
  
 
  
 6
 
  25652381
 
  25652815
 
  PDF   STATS   ENSEMBL   UCSC 
 
  435
 
 10
 
 35499
 
 island
 
 0.465571
 
 0.8122825
 
 Not queried
 
  2.4641980
 
 2.18e-01
 
 0.3935758299
  
 
  
 11
 
  44972672
 
  44973218
 
  PDF   STATS   ENSEMBL   UCSC 
 
  547
 
 10
 
 7263
 
 tss
 
 0.118308
 
 0.4868528
 
 Not queried
 
  2.4562624
 
 2.18e-01
 
 0.3935758299
  
 
  
 15
 
 101926307
 
 101926872
 
  PDF   STATS   ENSEMBL   UCSC 
 
  566
 
  5
 
 13509
 
 gene
 
 0.433577
 
 0.5801094
 
 Not queried
 
  2.4535796
 
 2.22e-01
 
 0.3935758299
  
 
  
 11
 
  44972574
 
  44973218
 
  PDF   STATS   ENSEMBL   UCSC 
 
  645
 
 11
 
 8229
 
 island
 
 0.084422
 
 0.4760670
 
 Not queried
 
  2.4434258
 
 2.17e-01
 
 0.3935758299
  
 
  
 15
 
  37403088
 
  37403304
 
  PDF   STATS   ENSEMBL   UCSC 
 
  217
 
  5
 
 14015
 
 island
 
 0.047988
 
 0.5030541
 
 Not queried
 
  2.4151620
 
 2.22e-01
 
 0.3935758299
  
 
  
 17
 
   7486551
 
   7486961
 
  PDF   STATS   ENSEMBL   UCSC 
 
  411
 
  9
 
 17090
 
 tss
 
 0.365900
 
 0.5155269
 
 Not queried
 
  2.4103386
 
 2.22e-01
 
 0.3935758299
  
 
  
 5
 
   1076587
 
   1076892
 
  PDF   STATS   ENSEMBL   UCSC 
 
  306
 
  5
 
 31518
 
 gene;island
 
 0.896603
 
 0.6215861
 
 Not queried
 
  2.3916349
 
 2.22e-01
 
 0.3935758299
  
 
  
 11
 
 134262988
 
 134263349
 
  PDF   STATS   ENSEMBL   UCSC 
 
  362
 
  5
 
 7001
 
 gene
 
 0.139983
 
 0.4378882
 
 Not queried
 
  2.3803460
 
 2.22e-01
 
 0.3935758299
  
 
  
 12
 
  50297477
 
  50297945
 
  PDF   STATS   ENSEMBL   UCSC 
 
  469
 
  7
 
 10406
 
 island
 
 0.037908
 
 0.1294793
 
 Not queried
 
  2.3686351
 
 2.09e-01
 
 0.3935758299
  
 
  
 3
 
 170746029
 
 170746265
 
  PDF   STATS   ENSEMBL   UCSC 
 
  237
 
  5
 
 28717
 
 tss
 
 0.035161
 
 0.0080118
 
 Not queried
 
  2.3268487
 
 2.22e-01
 
 0.3935758299
  
 
  
 11
 
  67350153
 
  67350663
 
  PDF   STATS   ENSEMBL   UCSC 
 
  511
 
  7
 
 7497
 
 tss;island
 
 0.584071
 
 0.4779931
 
 Not queried
 
  2.3204247
 
 2.09e-01
 
 0.3935758299
  
 
  
 11
 
 128562003
 
 128562408
 
  PDF   STATS   ENSEMBL   UCSC 
 
  406
 
  7
 
 7802
 
 tss;island
 
 0.048567
 
 0.0955358
 
 Not queried
 
  2.2916816
 
 2.09e-01
 
 0.3935758299
  
 
  
 19
 
  48908102
 
  48908557
 
  PDF   STATS   ENSEMBL   UCSC 
 
  456
 
  5
 
 20123
 
 gene;island
 
 0.544515
 
 0.8693372
 
 Not queried
 
  2.2848969
 
 2.22e-01
 
 0.3935758299
  
 
  
 1
 
  47902085
 
  47902371
 
  PDF   STATS   ENSEMBL   UCSC 
 
  287
 
  5
 
 518
 
 gene;island
 
 0.011032
 
 0.0056193
 
 Not queried
 
  2.2590309
 
 2.22e-01
 
 0.3935758299
  
 
  
 2
 
  30144376
 
  30144838
 
  PDF   STATS   ENSEMBL   UCSC 
 
  463
 
  7
 
 24199
 
 island
 
 0.013654
 
 0.0051273
 
 Not queried
 
  2.2584136
 
 2.09e-01
 
 0.3935758299
  
 
  
 6
 
  32784097
 
  32784676
 
  PDF   STATS   ENSEMBL   UCSC 
 
  580
 
  5
 
 34135
 
 gene
 
 0.006897
 
 0.0022263
 
 Not queried
 
  2.2556290
 
 2.22e-01
 
 0.3935758299
  
 
  
 3
 
 157824148
 
 157824283
 
  PDF   STATS   ENSEMBL   UCSC 
 
  136
 
  5
 
 28682
 
 tss;island
 
 0.039689
 
 0.0074809
 
 Not queried
 
  2.2507295
 
 2.22e-01
 
 0.3935758299
  
 
  
 6
 
  10419835
 
  10420079
 
  PDF   STATS   ENSEMBL   UCSC 
 
  245
 
  7
 
 34625
 
 tss
 
 0.380276
 
 0.0641276
 
 Not queried
 
  2.2501948
 
 2.09e-01
 
 0.3935758299
  
 
  
 3
 
 120003547
 
 120003884
 
  PDF   STATS   ENSEMBL   UCSC 
 
  338
 
  5
 
 29387
 
 island
 
 0.406864
 
 0.5333307
 
 Not queried
 
  2.2376783
 
 2.22e-01
 
 0.3935758299
  
 
  
 15
 
  65368982
 
  65369213
 
  PDF   STATS   ENSEMBL   UCSC 
 
  232
 
  5
 
 14226
 
 island
 
 0.867640
 
 0.6429829
 
 Not queried
 
  2.2301204
 
 2.22e-01
 
 0.3935758299
  
 
  
 7
 
 156795767
 
 156796145
 
  PDF   STATS   ENSEMBL   UCSC 
 
  379
 
  5
 
 39090
 
 island
 
 0.343458
 
 0.1553366
 
 Not queried
 
  2.2279573
 
 2.22e-01
 
 0.3935758299
  
 
  
 16
 
   2653222
 
   2653306
 
  PDF   STATS   ENSEMBL   UCSC 
 
   85
 
  7
 
 15032
 
 tss
 
 0.009520
 
 0.0076578
 
 Not queried
 
  2.2234841
 
 2.09e-01
 
 0.3935758299
  
 
  
 6
 
   5851644
 
   5851994
 
  PDF   STATS   ENSEMBL   UCSC 
 
  351
 
  5
 
 35401
 
 island
 
 0.087955
 
 0.2642338
 
 Not queried
 
  2.1798504
 
 2.22e-01
 
 0.3935758299
  
 
  
 5
 
  87971794
 
  87972265
 
  PDF   STATS   ENSEMBL   UCSC 
 
  472
 
  7
 
 31679
 
 gene;island
 
 0.025241
 
 0.0108808
 
 Not queried
 
  2.1759476
 
 2.09e-01
 
 0.3935758299
  
 
  
 7
 
 150210742
 
 150211152
 
  PDF   STATS   ENSEMBL   UCSC 
 
  411
 
  5
 
 37980
 
 tss
 
 0.544294
 
 0.6900520
 
 Not queried
 
  2.1606674
 
 2.22e-01
 
 0.3935758299
  
 
  
 14
 
  37666810
 
  37666994
 
  PDF   STATS   ENSEMBL   UCSC 
 
  185
 
  5
 
 12361
 
 tss;island
 
 0.033375
 
 0.1746561
 
 Not queried
 
  2.1572825
 
 2.22e-01
 
 0.3935758299
  
 
  
 5
 
 137225191
 
 137225509
 
  PDF   STATS   ENSEMBL   UCSC 
 
  319
 
  5
 
 31763
 
 gene;island
 
 0.002248
 
 0.0026534
 
 Not queried
 
  2.1568530
 
 2.22e-01
 
 0.3935758299
  
 
  
 5
 
 135266509
 
 135266896
 
  PDF   STATS   ENSEMBL   UCSC 
 
  388
 
  5
 
 31760
 
 gene;island
 
 0.185549
 
 0.1299760
 
 Not queried
 
  2.1541930
 
 2.22e-01
 
 0.3935758299
  
 
  
 22
 
  29834388
 
  29834697
 
  PDF   STATS   ENSEMBL   UCSC 
 
  310
 
  5
 
 26832
 
 gene
 
 0.131315
 
 0.5786169
 
 Not queried
 
  2.1522900
 
 2.22e-01
 
 0.3935758299
  
 
  
 2
 
 128051810
 
 128051925
 
  PDF   STATS   ENSEMBL   UCSC 
 
  116
 
  5
 
 23611
 
 tss
 
 0.008757
 
 0.0062341
 
 Not queried
 
  2.1508435
 
 2.22e-01
 
 0.3935758299
  
 
  
 11
 
  84634409
 
  84634735
 
  PDF   STATS   ENSEMBL   UCSC 
 
  327
 
  5
 
 6851
 
 gene
 
 0.446178
 
 0.8272360
 
 Not queried
 
  2.1470776
 
 2.22e-01
 
 0.3935758299
  
 
  
 5
 
  72715731
 
  72716122
 
  PDF   STATS   ENSEMBL   UCSC 
 
  392
 
  5
 
 32872
 
 island
 
 0.016098
 
 0.0253329
 
 Not queried
 
  2.1450218
 
 2.22e-01
 
 0.3935758299
  
 
  
 11
 
  59578351
 
  59578540
 
  PDF   STATS   ENSEMBL   UCSC 
 
  190
 
  5
 
 7319
 
 tss
 
 0.006566
 
 0.0735545
 
 Not queried
 
  2.1443442
 
 2.22e-01
 
 0.3935758299
  
 
  
 12
 
 132862800
 
 132863118
 
  PDF   STATS   ENSEMBL   UCSC 
 
  319
 
  5
 
 9443
 
 gene;island
 
 0.063178
 
 0.1351692
 
 Not queried
 
  2.1346217
 
 2.22e-01
 
 0.3935758299
  
 
  
 2
 
  73429084
 
  73429371
 
  PDF   STATS   ENSEMBL   UCSC 
 
  288
 
  7
 
 23437
 
 tss
 
 0.157638
 
 0.0404895
 
 Not queried
 
  2.1221394
 
 2.09e-01
 
 0.3935758299
  
 
  
 19
 
  58728390
 
  58728865
 
  PDF   STATS   ENSEMBL   UCSC 
 
  476
 
  5
 
 22498
 
 island
 
 0.777132
 
 0.4538132
 
 Not queried
 
  2.0696410
 
 2.22e-01
 
 0.3935758299
  
 
  
 18
 
  13611370
 
  13611489
 
  PDF   STATS   ENSEMBL   UCSC 
 
  120
 
  5
 
 19062
 
 gene;tss
 
 0.004980
 
 0.0571277
 
 Not queried
 
  2.0626241
 
 2.22e-01
 
 0.3935758299
  
 
  
 17
 
  46669455
 
  46669674
 
  PDF   STATS   ENSEMBL   UCSC 
 
  220
 
  7
 
 17463
 
 tss
 
 0.169650
 
 0.6050237
 
 Not queried
 
  2.0613223
 
 2.09e-01
 
 0.3935758299
  
 
  
 10
 
 132909113
 
 132909470
 
  PDF   STATS   ENSEMBL   UCSC 
 
  358
 
  5
 
 4840
 
 gene
 
 0.264510
 
 0.5990727
 
 Not queried
 
  2.0569120
 
 2.22e-01
 
 0.3935758299
  
 
  
 3
 
  39323202
 
  39323539
 
  PDF   STATS   ENSEMBL   UCSC 
 
  338
 
  5
 
 28233
 
 tss
 
 0.108439
 
 0.3780588
 
 Not queried
 
  2.0529848
 
 2.22e-01
 
 0.3935758299
  
 
  
 12
 
 115104274
 
 115104983
 
  PDF   STATS   ENSEMBL   UCSC 
 
  710
 
  7
 
 10844
 
 island
 
 0.028962
 
 0.4652791
 
 Not queried
 
  2.0517678
 
 2.09e-01
 
 0.3935758299
  
 
  
 5
 
   1394296
 
   1394633
 
  PDF   STATS   ENSEMBL   UCSC 
 
  338
 
  5
 
 31536
 
 gene
 
 0.061907
 
 0.5325649
 
 Not queried
 
  2.0215033
 
 2.22e-01
 
 0.3935758299
  
 
  
 5
 
   3605783
 
   3606081
 
  PDF   STATS   ENSEMBL   UCSC 
 
  299
 
  5
 
 32668
 
 island
 
 0.179798
 
 0.0666601
 
 Not queried
 
  2.0188136
 
 2.22e-01
 
 0.3935758299
  
 
  
 17
 
  36003216
 
  36003443
 
  PDF   STATS   ENSEMBL   UCSC 
 
  228
 
  5
 
 16536
 
 gene
 
 0.002751
 
 0.0049066
 
 Not queried
 
  1.9886477
 
 2.22e-01
 
 0.3935758299
  
 
  
 1
 
 228403127
 
 228403515
 
  PDF   STATS   ENSEMBL   UCSC 
 
  389
 
  5
 
 1050
 
 gene;island
 
 0.906939
 
 0.9502267
 
 Not queried
 
  1.9882350
 
 2.22e-01
 
 0.3935758299
  
 
  
 10
 
 103985730
 
 103986128
 
  PDF   STATS   ENSEMBL   UCSC 
 
  399
 
  7
 
 5318
 
 tss
 
 0.005845
 
 0.0159470
 
 Not queried
 
  1.9878180
 
 2.09e-01
 
 0.3935758299
  
 
  
 6
 
 112575212
 
 112575482
 
  PDF   STATS   ENSEMBL   UCSC 
 
  271
 
  5
 
 34450
 
 gene;island
 
 0.031979
 
 0.1157297
 
 Not queried
 
  1.9825537
 
 2.22e-01
 
 0.3935758299
  
 
  
 6
 
  33161377
 
  33161589
 
  PDF   STATS   ENSEMBL   UCSC 
 
  213
 
  5
 
 34217
 
 gene
 
 0.460392
 
 0.5272291
 
 Not queried
 
  1.9650520
 
 2.22e-01
 
 0.3935758299
  
 
  
 1
 
 230492737
 
 230493133
 
  PDF   STATS   ENSEMBL   UCSC 
 
  397
 
  5
 
 1072
 
 gene;island
 
 0.542553
 
 0.8466562
 
 Not queried
 
  1.9375434
 
 2.22e-01
 
 0.3935758299
  
 
  
 11
 
  27741916
 
  27742454
 
  PDF   STATS   ENSEMBL   UCSC 
 
  539
 
  9
 
 6547
 
 gene;tss;island
 
 0.154067
 
 0.1563225
 
 Not queried
 
  1.9321041
 
 2.22e-01
 
 0.3935758299
  
 
  
 11
 
 111847692
 
 111847984
 
  PDF   STATS   ENSEMBL   UCSC 
 
  293
 
  5
 
 7695
 
 tss
 
 0.013809
 
 0.0319009
 
 Not queried
 
  1.9318212
 
 2.22e-01
 
 0.3935758299
  
 
  
 15
 
  43785364
 
  43785537
 
  PDF   STATS   ENSEMBL   UCSC 
 
  174
 
  7
 
 13316
 
 gene;tss;island
 
 0.003032
 
 0.0054954
 
 Not queried
 
  1.9179438
 
 2.09e-01
 
 0.3935758299
  
 
  
 8
 
 101661918
 
 101662246
 
  PDF   STATS   ENSEMBL   UCSC 
 
  329
 
  7
 
 39844
 
 tss
 
 0.084620
 
 0.0071379
 
 Not queried
 
  1.9129980
 
 2.09e-01
 
 0.3935758299
  
 
  
 14
 
  77732283
 
  77732641
 
  PDF   STATS   ENSEMBL   UCSC 
 
  359
 
  5
 
 12150
 
 gene
 
 0.203222
 
 0.5179161
 
 Not queried
 
  1.9001318
 
 2.22e-01
 
 0.3935758299
  
 
  
 1
 
 211306986
 
 211307408
 
  PDF   STATS   ENSEMBL   UCSC 
 
  423
 
  5
 
 993
 
 gene;island
 
 0.013732
 
 0.0366259
 
 Not queried
 
  1.8974170
 
 2.22e-01
 
 0.3935758299
  
 
  
 4
 
    298681
 
    299370
 
  PDF   STATS   ENSEMBL   UCSC 
 
  690
 
  9
 
 30715
 
 island
 
 0.500183
 
 0.3106543
 
 Not queried
 
  1.8962997
 
 2.22e-01
 
 0.3935758299
  
 
  
 22
 
  23522307
 
  23522748
 
  PDF   STATS   ENSEMBL   UCSC 
 
  442
 
  9
 
 27349
 
 island
 
 0.172053
 
 0.0270550
 
 Not queried
 
  1.8852723
 
 2.22e-01
 
 0.3935758299
  
 
  
 12
 
  54343506
 
  54343835
 
  PDF   STATS   ENSEMBL   UCSC 
 
  330
 
  5
 
 10480
 
 island
 
 0.024140
 
 0.1336765
 
 Not queried
 
  1.8812960
 
 2.22e-01
 
 0.3935758299
  
 
  
 8
 
  19796299
 
  19796561
 
  PDF   STATS   ENSEMBL   UCSC 
 
  263
 
  5
 
 39574
 
 tss
 
 0.190963
 
 0.0303151
 
 Not queried
 
  1.8681138
 
 2.22e-01
 
 0.3935758299
  
 
  
 4
 
 148402399
 
 148402899
 
  PDF   STATS   ENSEMBL   UCSC 
 
  501
 
  5
 
 30150
 
 gene;island
 
 0.090257
 
 0.2785738
 
 Not queried
 
  1.8662345
 
 2.22e-01
 
 0.3935758299
  
 
  
 10
 
  71892593
 
  71892787
 
  PDF   STATS   ENSEMBL   UCSC 
 
  195
 
  7
 
 5812
 
 island
 
 0.004685
 
 0.0270801
 
 Not queried
 
  1.8594142
 
 2.09e-01
 
 0.3935758299
  
 
  
 1
 
 109941043
 
 109941201
 
  PDF   STATS   ENSEMBL   UCSC 
 
  159
 
  5
 
 1802
 
 tss;island
 
 0.523749
 
 0.0596369
 
 Not queried
 
  1.8556105
 
 2.22e-01
 
 0.3935758299
  
 
  
 1
 
 228291486
 
 228291705
 
  PDF   STATS   ENSEMBL   UCSC 
 
  220
 
  5
 
 2297
 
 tss;island
 
 0.981224
 
 0.8149215
 
 Not queried
 
  1.8523353
 
 2.22e-01
 
 0.3935758299
  
 
  
 11
 
  89867637
 
  89867809
 
  PDF   STATS   ENSEMBL   UCSC 
 
  173
 
  5
 
 7615
 
 tss
 
 0.011756
 
 0.0035716
 
 Not queried
 
  1.8242027
 
 2.22e-01
 
 0.3935758299
  
 
  
 10
 
  88024529
 
  88024673
 
  PDF   STATS   ENSEMBL   UCSC 
 
  145
 
  7
 
 4659
 
 gene;island
 
 0.139245
 
 0.3890921
 
 Not queried
 
  1.8213047
 
 2.09e-01
 
 0.3935758299
  
 
  
 20
 
  30406997
 
  30407145
 
  PDF   STATS   ENSEMBL   UCSC 
 
  149
 
  5
 
 25667
 
 tss
 
 0.508836
 
 0.8150419
 
 Not queried
 
  1.8132038
 
 2.22e-01
 
 0.3935758299
  
 
  
 4
 
 174427388
 
 174428191
 
  PDF   STATS   ENSEMBL   UCSC 
 
  804
 
  9
 
 31424
 
 island
 
 0.017714
 
 0.0435752
 
 Not queried
 
  1.8004759
 
 2.22e-01
 
 0.3935758299
  
 
  
 5
 
 145316210
 
 145316416
 
  PDF   STATS   ENSEMBL   UCSC 
 
  207
 
  5
 
 31849
 
 gene
 
 0.036536
 
 0.0977517
 
 Not queried
 
  1.7985348
 
 2.22e-01
 
 0.3935758299
  
 
  
 3
 
 140770213
 
 140770683
 
  PDF   STATS   ENSEMBL   UCSC 
 
  471
 
 11
 
 28637
 
 tss
 
 0.007307
 
 0.0294311
 
 Not queried
 
  1.7678304
 
 2.17e-01
 
 0.3935758299
  
 
  
 12
 
 125399699
 
 125399964
 
  PDF   STATS   ENSEMBL   UCSC 
 
  266
 
  5
 
 10110
 
 tss;island
 
 0.010623
 
 0.0029807
 
 Not queried
 
  1.7623007
 
 2.22e-01
 
 0.3935758299
  
 
  
 7
 
   4901337
 
   4901798
 
  PDF   STATS   ENSEMBL   UCSC 
 
  462
 
  7
 
 36862
 
 gene;island
 
 0.668782
 
 0.4343964
 
 Not queried
 
  1.7500768
 
 2.09e-01
 
 0.3935758299
  
 
  
 15
 
  90456248
 
  90456686
 
  PDF   STATS   ENSEMBL   UCSC 
 
  439
 
  5
 
 13894
 
 tss
 
 0.035608
 
 0.3630170
 
 Not queried
 
  1.7392580
 
 2.22e-01
 
 0.3935758299
  
 
  
 17
 
  62018877
 
  62019438
 
  PDF   STATS   ENSEMBL   UCSC 
 
  562
 
  5
 
 16730
 
 gene;island
 
 0.589905
 
 0.8681906
 
 Not queried
 
  1.7361019
 
 2.22e-01
 
 0.3935758299
  
 
  
 10
 
   1451381
 
   1451648
 
  PDF   STATS   ENSEMBL   UCSC 
 
  268
 
  5
 
 4464
 
 gene
 
 0.674036
 
 0.8785177
 
 Not queried
 
  1.7318052
 
 2.22e-01
 
 0.3935758299
  
 
  
 6
 
 122793073
 
 122793338
 
  PDF   STATS   ENSEMBL   UCSC 
 
  266
 
  5
 
 34462
 
 gene
 
 0.002256
 
 0.0042148
 
 Not queried
 
  1.7316466
 
 2.22e-01
 
 0.3935758299
  
 
  
 17
 
  17465162
 
  17465476
 
  PDF   STATS   ENSEMBL   UCSC 
 
  315
 
  5
 
 16464
 
 gene;island
 
 0.480938
 
 0.7422868
 
 Not queried
 
  1.7306962
 
 2.22e-01
 
 0.3935758299
  
 
  
 2
 
 176987161
 
 176987411
 
  PDF   STATS   ENSEMBL   UCSC 
 
  251
 
  5
 
 23737
 
 tss
 
 0.438248
 
 0.1227829
 
 Not queried
 
  1.7294872
 
 2.22e-01
 
 0.3935758299
  
 
  
 7
 
 150974273
 
 150974554
 
  PDF   STATS   ENSEMBL   UCSC 
 
  282
 
  7
 
 37999
 
 tss;island
 
 0.030855
 
 0.0112445
 
 Not queried
 
  1.7267160
 
 2.09e-01
 
 0.3935758299
  
 
  
 10
 
  82116203
 
  82116596
 
  PDF   STATS   ENSEMBL   UCSC 
 
  394
 
 10
 
 4654
 
 gene;tss;island
 
 0.038268
 
 0.0686148
 
 Not queried
 
  1.7221475
 
 2.18e-01
 
 0.3935758299
  
 
  
 10
 
 103043991
 
 103044385
 
  PDF   STATS   ENSEMBL   UCSC 
 
  395
 
  5
 
 6065
 
 island
 
 0.113561
 
 0.0420769
 
 Not queried
 
  1.7048482
 
 2.22e-01
 
 0.3935758299
  
 
  
 14
 
 101512592
 
 101512826
 
  PDF   STATS   ENSEMBL   UCSC 
 
  235
 
  5
 
 12627
 
 tss
 
 0.355222
 
 0.7770768
 
 Not queried
 
  1.6988714
 
 2.22e-01
 
 0.3935758299
  
 
  
 5
 
 140810051
 
 140810137
 
  PDF   STATS   ENSEMBL   UCSC 
 
   87
 
  5
 
 32383
 
 tss
 
 0.013391
 
 0.0041203
 
 Not queried
 
  1.6985656
 
 2.22e-01
 
 0.3935758299
  
 
  
 10
 
   3823758
 
   3824016
 
  PDF   STATS   ENSEMBL   UCSC 
 
  259
 
  5
 
 4475
 
 gene;island
 
 0.494849
 
 0.5648704
 
 Not queried
 
  1.6953121
 
 2.22e-01
 
 0.3935758299
  
 
  
 4
 
 146403866
 
 146403948
 
  PDF   STATS   ENSEMBL   UCSC 
 
   83
 
  5
 
 30620
 
 tss
 
 0.054237
 
 0.0014999
 
 Not queried
 
  1.6908958
 
 2.22e-01
 
 0.3935758299
  
 
  
 19
 
  49198900
 
  49199234
 
  PDF   STATS   ENSEMBL   UCSC 
 
  335
 
  5
 
 22230
 
 island
 
 0.182484
 
 0.4165967
 
 Not queried
 
  1.6777965
 
 2.22e-01
 
 0.3935758299
  
 
  
 15
 
  74494781
 
  74494909
 
  PDF   STATS   ENSEMBL   UCSC 
 
  129
 
  5
 
 13420
 
 gene
 
 0.392872
 
 0.6675646
 
 Not queried
 
  1.6692110
 
 2.22e-01
 
 0.3935758299
  
 
  
 2
 
 176988203
 
 176988580
 
  PDF   STATS   ENSEMBL   UCSC 
 
  378
 
  5
 
 22937
 
 gene;island
 
 0.054867
 
 0.6988130
 
 Not queried
 
  1.6605155
 
 2.22e-01
 
 0.3935758299
  
 
  
 2
 
   5836111
 
   5836366
 
  PDF   STATS   ENSEMBL   UCSC 
 
  256
 
  5
 
 22558
 
 gene;island
 
 0.016714
 
 0.0041611
 
 Not queried
 
  1.6423702
 
 2.22e-01
 
 0.3935758299
  
 
  
 10
 
  16561202
 
  16561605
 
  PDF   STATS   ENSEMBL   UCSC 
 
  404
 
  5
 
 4515
 
 gene;island
 
 0.468438
 
 0.5327622
 
 Not queried
 
  1.6418726
 
 2.22e-01
 
 0.3935758299
  
 
  
 13
 
 114085424
 
 114085732
 
  PDF   STATS   ENSEMBL   UCSC 
 
  309
 
  5
 
 11285
 
 gene;island
 
 0.746358
 
 0.8888950
 
 Not queried
 
  1.6299326
 
 2.22e-01
 
 0.3935758299
  
 
  
 10
 
 102809954
 
 102810157
 
  PDF   STATS   ENSEMBL   UCSC 
 
  204
 
  5
 
 6048
 
 island
 
 0.009638
 
 0.0486468
 
 Not queried
 
  1.6261847
 
 2.22e-01
 
 0.3935758299
  
 
  
 10
 
 134977981
 
 134978147
 
  PDF   STATS   ENSEMBL   UCSC 
 
  167
 
  5
 
 4887
 
 gene;island
 
 0.301777
 
 0.6562851
 
 Not queried
 
  1.6246050
 
 2.22e-01
 
 0.3935758299
  
 
  
 11
 
 102187898
 
 102188180
 
  PDF   STATS   ENSEMBL   UCSC 
 
  283
 
  7
 
 7647
 
 tss
 
 0.001289
 
 0.0050936
 
 Not queried
 
  1.6235532
 
 2.09e-01
 
 0.3935758299
  
 
  
 5
 
  37834672
 
  37835168
 
  PDF   STATS   ENSEMBL   UCSC 
 
  497
 
  7
 
 31590
 
 gene;island
 
 0.091218
 
 0.0228805
 
 Not queried
 
  1.6167965
 
 2.09e-01
 
 0.3935758299
  
 
  
 11
 
  63827695
 
  63828346
 
  PDF   STATS   ENSEMBL   UCSC 
 
  652
 
  5
 
 6660
 
 gene
 
 0.211995
 
 0.5249339
 
 Not queried
 
  1.5944516
 
 2.22e-01
 
 0.3935758299
  
 
  
 2
 
 209010890
 
 209011053
 
  PDF   STATS   ENSEMBL   UCSC 
 
  164
 
  5
 
 23847
 
 tss
 
 0.244850
 
 0.4849821
 
 Not queried
 
  1.5924246
 
 2.22e-01
 
 0.3935758299
  
 
  
 17
 
  34062219
 
  34062573
 
  PDF   STATS   ENSEMBL   UCSC 
 
  355
 
  5
 
 16524
 
 gene;island
 
 0.690677
 
 0.9130124
 
 Not queried
 
  1.5650900
 
 2.22e-01
 
 0.3935758299
  
 
  
 14
 
 101498206
 
 101498317
 
  PDF   STATS   ENSEMBL   UCSC 
 
  112
 
  5
 
 12623
 
 tss
 
 0.669605
 
 0.9089989
 
 Not queried
 
  1.5537975
 
 2.22e-01
 
 0.3935758299
  
 
  
 10
 
 103985730
 
 103986268
 
  PDF   STATS   ENSEMBL   UCSC 
 
  539
 
  9
 
 6080
 
 island
 
 0.014656
 
 0.0180248
 
 Not queried
 
  1.5487062
 
 2.22e-01
 
 0.3935758299
  
 
  
 13
 
  28674720
 
  28675056
 
  PDF   STATS   ENSEMBL   UCSC 
 
  337
 
  5
 
 11637
 
 island
 
 0.008420
 
 0.0209545
 
 Not queried
 
  1.5442942
 
 2.22e-01
 
 0.3935758299
  
 
  
 16
 
   1728187
 
   1728262
 
  PDF   STATS   ENSEMBL   UCSC 
 
   76
 
  5
 
 15001
 
 tss
 
 0.003376
 
 0.0003365
 
 Not queried
 
  1.5416333
 
 2.22e-01
 
 0.3935758299
  
 
  
 4
 
   7436701
 
   7437156
 
  PDF   STATS   ENSEMBL   UCSC 
 
  456
 
  5
 
 30292
 
 tss
 
 0.044072
 
 0.3291283
 
 Not queried
 
  1.5410172
 
 2.22e-01
 
 0.3935758299
  
 
  
 6
 
  33165404
 
  33165899
 
  PDF   STATS   ENSEMBL   UCSC 
 
  496
 
  9
 
 34219
 
 gene;island
 
 0.631216
 
 0.9201609
 
 Not queried
 
  1.5337454
 
 2.22e-01
 
 0.3935758299
  
 
  
 4
 
   1496822
 
   1497223
 
  PDF   STATS   ENSEMBL   UCSC 
 
  402
 
  5
 
 30787
 
 island
 
 0.426106
 
 0.6735733
 
 Not queried
 
  1.5091110
 
 2.22e-01
 
 0.3935758299
  
 
  
 6
 
 161575229
 
 161575667
 
  PDF   STATS   ENSEMBL   UCSC 
 
  439
 
  7
 
 34537
 
 gene
 
 0.848917
 
 0.9028214
 
 Not queried
 
  1.5001200
 
 2.09e-01
 
 0.3935758299
  
 
  
 6
 
 126070013
 
 126070499
 
  PDF   STATS   ENSEMBL   UCSC 
 
  487
 
  7
 
 35211
 
 tss;island
 
 0.009430
 
 0.0037202
 
 Not queried
 
  1.4915619
 
 2.09e-01
 
 0.3935758299
  
 
  
 5
 
 178322737
 
 178322856
 
  PDF   STATS   ENSEMBL   UCSC 
 
  120
 
  5
 
 32544
 
 tss
 
 0.060236
 
 0.0824277
 
 Not queried
 
  1.4898843
 
 2.22e-01
 
 0.3935758299
  
 
  
 19
 
   3687751
 
   3688176
 
  PDF   STATS   ENSEMBL   UCSC 
 
  426
 
  5
 
 19721
 
 gene;island
 
 0.011822
 
 0.0145177
 
 Not queried
 
  1.4880428
 
 2.22e-01
 
 0.3935758299
  
 
  
 17
 
  56064295
 
  56064928
 
  PDF   STATS   ENSEMBL   UCSC 
 
  634
 
  7
 
 16706
 
 gene;island
 
 0.003290
 
 0.0107299
 
 Not queried
 
  1.4870620
 
 2.09e-01
 
 0.3935758299
  
 
  
 2
 
 176994142
 
 176994448
 
  PDF   STATS   ENSEMBL   UCSC 
 
  307
 
  5
 
 23739
 
 tss
 
 0.059107
 
 0.2091567
 
 Not queried
 
  1.4824323
 
 2.22e-01
 
 0.3935758299
  
 
  
 14
 
 101123263
 
 101123867
 
  PDF   STATS   ENSEMBL   UCSC 
 
  605
 
  7
 
 13140
 
 island
 
 0.526809
 
 0.8013707
 
 Not queried
 
  1.4793927
 
 2.09e-01
 
 0.3935758299
  
 
  
 16
 
  23765810
 
  23766116
 
  PDF   STATS   ENSEMBL   UCSC 
 
  307
 
  5
 
 15769
 
 island
 
 0.270799
 
 0.1668973
 
 Not queried
 
  1.4689504
 
 2.22e-01
 
 0.3935758299
  
 
  
 15
 
  99789622
 
  99789855
 
  PDF   STATS   ENSEMBL   UCSC 
 
  234
 
  5
 
 14503
 
 island
 
 0.006067
 
 0.0329515
 
 Not queried
 
  1.4674485
 
 2.22e-01
 
 0.3935758299
  
 
  
 12
 
  10876053
 
  10876242
 
  PDF   STATS   ENSEMBL   UCSC 
 
  190
 
  7
 
 9563
 
 tss
 
 0.001761
 
 0.0015209
 
 Not queried
 
  1.4669869
 
 2.09e-01
 
 0.3935758299
  
 
  
 14
 
  23235611
 
  23235738
 
  PDF   STATS   ENSEMBL   UCSC 
 
  128
 
  5
 
 12700
 
 island
 
 0.005792
 
 0.0016283
 
 Not queried
 
  1.4552579
 
 2.22e-01
 
 0.3935758299
  
 
  
 19
 
  57741988
 
  57742423
 
  PDF   STATS   ENSEMBL   UCSC 
 
  436
 
  9
 
 21107
 
 tss
 
 0.925233
 
 0.7853748
 
 Not queried
 
  1.4543585
 
 2.22e-01
 
 0.3935758299
  
 
  
 1
 
  63989080
 
  63989278
 
  PDF   STATS   ENSEMBL   UCSC 
 
  199
 
  5
 
 565
 
 gene
 
 0.006232
 
 0.0034583
 
 Not queried
 
  1.4528939
 
 2.22e-01
 
 0.3935758299
  
 
  
 13
 
  92001440
 
  92001959
 
  PDF   STATS   ENSEMBL   UCSC 
 
  520
 
  5
 
 11188
 
 gene;tss;island
 
 0.003461
 
 0.0010040
 
 Not queried
 
  1.4525072
 
 2.22e-01
 
 0.3935758299
  
 
  
 21
 
  43823604
 
  43823863
 
  PDF   STATS   ENSEMBL   UCSC 
 
  260
 
  5
 
 26568
 
 tss
 
 0.910401
 
 0.7145502
 
 Not queried
 
  1.4397501
 
 2.22e-01
 
 0.3935758299
  
 
  
 16
 
  85517170
 
  85517684
 
  PDF   STATS   ENSEMBL   UCSC 
 
  515
 
  7
 
 16156
 
 island
 
 0.470846
 
 0.7427880
 
 Not queried
 
  1.4370390
 
 2.09e-01
 
 0.3935758299
  
 
  
 17
 
  42019637
 
  42019987
 
  PDF   STATS   ENSEMBL   UCSC 
 
  351
 
  5
 
 18382
 
 island
 
 0.580505
 
 0.6797953
 
 Not queried
 
  1.4305893
 
 2.22e-01
 
 0.3935758299
  
 
  
 6
 
  44695195
 
  44695536
 
  PDF   STATS   ENSEMBL   UCSC 
 
  342
 
  5
 
 36180
 
 island
 
 0.851604
 
 0.7140631
 
 Not queried
 
  1.4298011
 
 2.22e-01
 
 0.3935758299
  
 
  
 16
 
   1493343
 
   1493859
 
  PDF   STATS   ENSEMBL   UCSC 
 
  517
 
  5
 
 14571
 
 gene;island
 
 0.930494
 
 0.6780917
 
 Not queried
 
  1.4280272
 
 2.22e-01
 
 0.3935758299
  
 
  
 10
 
 103603722
 
 103604019
 
  PDF   STATS   ENSEMBL   UCSC 
 
  298
 
  5
 
 5312
 
 tss
 
 0.088622
 
 0.0221892
 
 Not queried
 
  1.4148738
 
 2.22e-01
 
 0.3935758299
  
 
  
 1
 
 190447232
 
 190447640
 
  PDF   STATS   ENSEMBL   UCSC 
 
  409
 
  5
 
 2147
 
 tss
 
 0.016353
 
 0.0018712
 
 Not queried
 
  1.4101740
 
 2.22e-01
 
 0.3935758299
  
 
  
 17
 
  32483560
 
  32484259
 
  PDF   STATS   ENSEMBL   UCSC 
 
  700
 
 13
 
 18200
 
 island
 
 0.040385
 
 0.2445831
 
 Not queried
 
  2.3759424
 
 2.23e-01
 
 0.3936710914
  
 
  
 13
 
 102068989
 
 102069296
 
  PDF   STATS   ENSEMBL   UCSC 
 
  308
 
  8
 
 11519
 
 tss;island
 
 0.217888
 
 0.0194661
 
 Not queried
 
  3.9599921
 
 2.34e-01
 
 0.4128374386
  
 
  
 5
 
 138628911
 
 138629210
 
  PDF   STATS   ENSEMBL   UCSC 
 
  300
 
  8
 
 31772
 
 gene;tss;island
 
 0.012451
 
 0.0015043
 
 Not queried
 
  3.8890981
 
 2.34e-01
 
 0.4128374386
  
 
  
 11
 
  57519282
 
  57519659
 
  PDF   STATS   ENSEMBL   UCSC 
 
  378
 
  8
 
 7310
 
 tss;island
 
 0.167328
 
 0.6865073
 
 Not queried
 
  2.5107935
 
 2.34e-01
 
 0.4128374386
  
 
  
 5
 
 172672684
 
 172673349
 
  PDF   STATS   ENSEMBL   UCSC 
 
  666
 
  8
 
 33410
 
 island
 
 0.070328
 
 0.2505509
 
 Not queried
 
  2.3894434
 
 2.34e-01
 
 0.4128374386
  
 
  
 19
 
  37157776
 
  37157995
 
  PDF   STATS   ENSEMBL   UCSC 
 
  220
 
  8
 
 20729
 
 tss
 
 0.008310
 
 0.0030423
 
 Not queried
 
  1.9337333
 
 2.34e-01
 
 0.4128374386
  
 
  
 11
 
 129939665
 
 129939958
 
  PDF   STATS   ENSEMBL   UCSC 
 
  294
 
  8
 
 8954
 
 island
 
 0.003124
 
 0.0133581
 
 Not queried
 
  1.7522569
 
 2.34e-01
 
 0.4128374386
  
 
  
 15
 
  91537812
 
  91538344
 
  PDF   STATS   ENSEMBL   UCSC 
 
  533
 
  8
 
 13909
 
 tss
 
 0.004777
 
 0.0113703
 
 Not queried
 
  1.7430328
 
 2.34e-01
 
 0.4128374386
  
 
  
 6
 
 150463775
 
 150464265
 
  PDF   STATS   ENSEMBL   UCSC 
 
  491
 
  8
 
 36568
 
 island
 
 0.046512
 
 0.0248429
 
 Not queried
 
  1.5475838
 
 2.34e-01
 
 0.4128374386
  
 
  
 18
 
   5544031
 
   5544237
 
  PDF   STATS   ENSEMBL   UCSC 
 
  207
 
  8
 
 19171
 
 tss
 
 0.022920
 
 0.0818604
 
 Not queried
 
  1.5185578
 
 2.34e-01
 
 0.4128374386
  
 
  
 3
 
  48700269
 
  48700498
 
  PDF   STATS   ENSEMBL   UCSC 
 
  230
 
  8
 
 29095
 
 island
 
 0.029456
 
 0.0139085
 
 Not queried
 
  1.4016363
 
 2.34e-01
 
 0.4128374386
  
 
  
 10
 
  47083283
 
  47083504
 
  PDF   STATS   ENSEMBL   UCSC 
 
  222
 
  8
 
 5063
 
 tss
 
 0.084251
 
 0.0217117
 
 Not queried
 
  1.4008647
 
 2.34e-01
 
 0.4128374386
  
 
  
 1
 
  59043173
 
  59043370
 
  PDF   STATS   ENSEMBL   UCSC 
 
  198
 
  6
 
 1654
 
 tss
 
 0.408430
 
 0.9753593
 
 Not queried
 
  5.4408718
 
 2.40e-01
 
 0.4133265075
  
 
  
 7
 
 153584582
 
 153584873
 
  PDF   STATS   ENSEMBL   UCSC 
 
  292
 
  6
 
 37301
 
 gene
 
 0.297042
 
 0.6974766
 
 Not queried
 
  3.6206208
 
 2.40e-01
 
 0.4133265075
  
 
  
 2
 
 220282914
 
 220283214
 
  PDF   STATS   ENSEMBL   UCSC 
 
  301
 
  6
 
 25123
 
 island
 
 0.080477
 
 0.4228122
 
 Not queried
 
  3.2488508
 
 2.40e-01
 
 0.4133265075
  
 
  
 5
 
 172655841
 
 172656166
 
  PDF   STATS   ENSEMBL   UCSC 
 
  326
 
  6
 
 33405
 
 island
 
 0.136688
 
 0.0172213
 
 Not queried
 
  3.1679816
 
 2.40e-01
 
 0.4133265075
  
 
  
 14
 
  89028798
 
  89029224
 
  PDF   STATS   ENSEMBL   UCSC 
 
  427
 
  6
 
 12545
 
 tss;island
 
 0.078839
 
 0.5668370
 
 Not queried
 
  3.0078832
 
 2.40e-01
 
 0.4133265075
  
 
  
 3
 
  27766009
 
  27766382
 
  PDF   STATS   ENSEMBL   UCSC 
 
  374
 
  6
 
 28953
 
 island
 
 0.210773
 
 0.0464318
 
 Not queried
 
  2.8455302
 
 2.40e-01
 
 0.4133265075
  
 
  
 16
 
  30485383
 
  30485966
 
  PDF   STATS   ENSEMBL   UCSC 
 
  584
 
  6
 
 14721
 
 gene;island
 
 0.816019
 
 0.9133375
 
 Not queried
 
  2.6987900
 
 2.40e-01
 
 0.4133265075
  
 
  
 2
 
  51259693
 
  51260003
 
  PDF   STATS   ENSEMBL   UCSC 
 
  311
 
  6
 
 23349
 
 tss
 
 0.013117
 
 0.0048512
 
 Not queried
 
  2.6863808
 
 2.40e-01
 
 0.4133265075
  
 
  
 5
 
 151304770
 
 151305143
 
  PDF   STATS   ENSEMBL   UCSC 
 
  374
 
  6
 
 32445
 
 tss;island
 
 0.019517
 
 0.2324845
 
 Not queried
 
  2.6323532
 
 2.40e-01
 
 0.4133265075
  
 
  
 5
 
 142065539
 
 142065819
 
  PDF   STATS   ENSEMBL   UCSC 
 
  281
 
  6
 
 31843
 
 gene
 
 0.034353
 
 0.2259026
 
 Not queried
 
  2.6259264
 
 2.40e-01
 
 0.4133265075
  
 
  
 10
 
 102756795
 
 102756923
 
  PDF   STATS   ENSEMBL   UCSC 
 
  129
 
  6
 
 5301
 
 tss
 
 0.003667
 
 0.0055906
 
 Not queried
 
  2.5432082
 
 2.40e-01
 
 0.4133265075
  
 
  
 1
 
  15271830
 
  15272326
 
  PDF   STATS   ENSEMBL   UCSC 
 
  497
 
  6
 
 241
 
 gene;tss
 
 0.254002
 
 0.5285203
 
 Not queried
 
  2.3018208
 
 2.40e-01
 
 0.4133265075
  
 
  
 6
 
  46459694
 
  46460003
 
  PDF   STATS   ENSEMBL   UCSC 
 
  310
 
  6
 
 36186
 
 island
 
 0.177104
 
 0.0623318
 
 Not queried
 
  2.2872403
 
 2.40e-01
 
 0.4133265075
  
 
  
 1
 
 201617847
 
 201618284
 
  PDF   STATS   ENSEMBL   UCSC 
 
  438
 
  6
 
 940
 
 gene;island
 
 0.172463
 
 0.5816383
 
 Not queried
 
  2.2654593
 
 2.40e-01
 
 0.4133265075
  
 
  
 4
 
  54457737
 
  54458246
 
  PDF   STATS   ENSEMBL   UCSC 
 
  510
 
  6
 
 30397
 
 tss
 
 0.121072
 
 0.0624863
 
 Not queried
 
  2.1981305
 
 2.40e-01
 
 0.4133265075
  
 
  
 10
 
  86001154
 
  86001418
 
  PDF   STATS   ENSEMBL   UCSC 
 
  265
 
  6
 
 5896
 
 island
 
 0.204161
 
 0.4056413
 
 Not queried
 
  2.1864426
 
 2.40e-01
 
 0.4133265075
  
 
  
 8
 
 120868172
 
 120868512
 
  PDF   STATS   ENSEMBL   UCSC 
 
  341
 
  6
 
 39888
 
 tss;island
 
 0.002912
 
 0.0004384
 
 Not queried
 
  2.1139798
 
 2.40e-01
 
 0.4133265075
  
 
  
 1
 
 111682944
 
 111683426
 
  PDF   STATS   ENSEMBL   UCSC 
 
  483
 
  6
 
 681
 
 gene;tss;island
 
 0.010113
 
 0.0026909
 
 Not queried
 
  2.0817570
 
 2.40e-01
 
 0.4133265075
  
 
  
 4
 
 118006747
 
 118006832
 
  PDF   STATS   ENSEMBL   UCSC 
 
   86
 
  6
 
 30559
 
 tss
 
 0.155771
 
 0.0382333
 
 Not queried
 
  2.0751487
 
 2.40e-01
 
 0.4133265075
  
 
  
 22
 
  38054505
 
  38054892
 
  PDF   STATS   ENSEMBL   UCSC 
 
  388
 
  6
 
 27466
 
 island
 
 0.004561
 
 0.0111429
 
 Not queried
 
  1.9893038
 
 2.40e-01
 
 0.4133265075
  
 
  
 7
 
   1407330
 
   1407571
 
  PDF   STATS   ENSEMBL   UCSC 
 
  242
 
  6
 
 38100
 
 island
 
 0.398310
 
 0.6138212
 
 Not queried
 
  1.9870364
 
 2.40e-01
 
 0.4133265075
  
 
  
 7
 
 155325552
 
 155326102
 
  PDF   STATS   ENSEMBL   UCSC 
 
  551
 
  6
 
 37310
 
 gene;island
 
 0.233230
 
 0.0355060
 
 Not queried
 
  1.9452031
 
 2.40e-01
 
 0.4133265075
  
 
  
 1
 
  71513494
 
  71513789
 
  PDF   STATS   ENSEMBL   UCSC 
 
  296
 
  6
 
 1693
 
 tss;island
 
 0.214708
 
 0.0274660
 
 Not queried
 
  1.9411408
 
 2.40e-01
 
 0.4133265075
  
 
  
 17
 
   4802692
 
   4802906
 
  PDF   STATS   ENSEMBL   UCSC 
 
  215
 
  6
 
 17033
 
 tss
 
 0.021502
 
 0.0106465
 
 Not queried
 
  1.9359626
 
 2.40e-01
 
 0.4133265075
  
 
  
 13
 
 113642457
 
 113642784
 
  PDF   STATS   ENSEMBL   UCSC 
 
  328
 
  6
 
 11265
 
 gene
 
 0.691641
 
 0.9018924
 
 Not queried
 
  1.8887384
 
 2.40e-01
 
 0.4133265075
  
 
  
 6
 
 152702330
 
 152702660
 
  PDF   STATS   ENSEMBL   UCSC 
 
  331
 
  6
 
 34516
 
 gene
 
 0.578251
 
 0.7498369
 
 Not queried
 
  1.8803725
 
 2.40e-01
 
 0.4133265075
  
 
  
 7
 
   1095416
 
   1095720
 
  PDF   STATS   ENSEMBL   UCSC 
 
  305
 
  6
 
 36742
 
 gene;island
 
 0.546825
 
 0.4516949
 
 Not queried
 
  1.8747429
 
 2.40e-01
 
 0.4133265075
  
 
  
 11
 
 133938676
 
 133939064
 
  PDF   STATS   ENSEMBL   UCSC 
 
  389
 
  6
 
 8974
 
 island
 
 0.025519
 
 0.0133647
 
 Not queried
 
  1.8191157
 
 2.40e-01
 
 0.4133265075
  
 
  
 6
 
  32632106
 
  32632338
 
  PDF   STATS   ENSEMBL   UCSC 
 
  233
 
  6
 
 34125
 
 gene;island
 
 0.238172
 
 0.4229016
 
 Not queried
 
  1.8188120
 
 2.40e-01
 
 0.4133265075
  
 
  
 2
 
 197457277
 
 197457430
 
  PDF   STATS   ENSEMBL   UCSC 
 
  154
 
  6
 
 24975
 
 island
 
 0.018179
 
 0.0434348
 
 Not queried
 
  1.7861314
 
 2.40e-01
 
 0.4133265075
  
 
  
 10
 
 102997782
 
 102998117
 
  PDF   STATS   ENSEMBL   UCSC 
 
  336
 
  6
 
 4728
 
 gene;island
 
 0.201032
 
 0.0497788
 
 Not queried
 
  1.7595979
 
 2.40e-01
 
 0.4133265075
  
 
  
 10
 
  50816917
 
  50817306
 
  PDF   STATS   ENSEMBL   UCSC 
 
  390
 
  6
 
 5073
 
 tss;island
 
 0.014067
 
 0.0353188
 
 Not queried
 
  1.7511018
 
 2.40e-01
 
 0.4133265075
  
 
  
 21
 
  46424715
 
  46424976
 
  PDF   STATS   ENSEMBL   UCSC 
 
  262
 
  6
 
 26591
 
 tss;island
 
 0.292517
 
 0.6818980
 
 Not queried
 
  1.7217187
 
 2.40e-01
 
 0.4133265075
  
 
  
 1
 
 203456691
 
 203457150
 
  PDF   STATS   ENSEMBL   UCSC 
 
  460
 
  6
 
 952
 
 gene;island
 
 0.004401
 
 0.0182840
 
 Not queried
 
  1.7152214
 
 2.40e-01
 
 0.4133265075
  
 
  
 5
 
   1089507
 
   1089800
 
  PDF   STATS   ENSEMBL   UCSC 
 
  294
 
  6
 
 31522
 
 gene;island
 
 0.933616
 
 0.6746886
 
 Not queried
 
  1.7114603
 
 2.40e-01
 
 0.4133265075
  
 
  
 7
 
  28319343
 
  28319676
 
  PDF   STATS   ENSEMBL   UCSC 
 
  334
 
  6
 
 37504
 
 tss
 
 0.518468
 
 0.6638618
 
 Not queried
 
  1.6804115
 
 2.40e-01
 
 0.4133265075
  
 
  
 20
 
  61162000
 
  61162112
 
  PDF   STATS   ENSEMBL   UCSC 
 
  113
 
  6
 
 25877
 
 tss
 
 0.250623
 
 0.4740069
 
 Not queried
 
  1.6713535
 
 2.40e-01
 
 0.4133265075
  
 
  
 22
 
  18531028
 
  18531591
 
  PDF   STATS   ENSEMBL   UCSC 
 
  564
 
  6
 
 27282
 
 island
 
 0.526946
 
 0.7784972
 
 Not queried
 
  1.6398113
 
 2.40e-01
 
 0.4133265075
  
 
  
 5
 
 140743780
 
 140744155
 
  PDF   STATS   ENSEMBL   UCSC 
 
  376
 
  6
 
 31823
 
 gene;island
 
 0.500021
 
 0.7084960
 
 Not queried
 
  1.6271368
 
 2.40e-01
 
 0.4133265075
  
 
  
 15
 
  67417607
 
  67417964
 
  PDF   STATS   ENSEMBL   UCSC 
 
  358
 
  6
 
 13392
 
 gene;tss
 
 0.921387
 
 0.5672181
 
 Not queried
 
  1.6269886
 
 2.40e-01
 
 0.4133265075
  
 
  
 1
 
  60539362
 
  60539675
 
  PDF   STATS   ENSEMBL   UCSC 
 
  314
 
  6
 
 3327
 
 island
 
 0.047965
 
 0.3123748
 
 Not queried
 
  1.6265057
 
 2.40e-01
 
 0.4133265075
  
 
  
 12
 
   4554786
 
   4554971
 
  PDF   STATS   ENSEMBL   UCSC 
 
  186
 
  6
 
 9498
 
 tss
 
 0.696877
 
 0.8551511
 
 Not queried
 
  1.5898724
 
 2.40e-01
 
 0.4133265075
  
 
  
 12
 
  85673860
 
  85674022
 
  PDF   STATS   ENSEMBL   UCSC 
 
  163
 
  6
 
 9901
 
 tss
 
 0.087387
 
 0.0305350
 
 Not queried
 
  1.5758998
 
 2.40e-01
 
 0.4133265075
  
 
  
 13
 
  25861182
 
  25861849
 
  PDF   STATS   ENSEMBL   UCSC 
 
  668
 
  6
 
 11611
 
 island
 
 0.002223
 
 0.0086433
 
 Not queried
 
  1.5521300
 
 2.40e-01
 
 0.4133265075
  
 
  
 5
 
 176873332
 
 176873656
 
  PDF   STATS   ENSEMBL   UCSC 
 
  325
 
  6
 
 32528
 
 tss
 
 0.003212
 
 0.0124995
 
 Not queried
 
  1.5462360
 
 2.40e-01
 
 0.4133265075
  
 
  
 7
 
   4308120
 
   4308449
 
  PDF   STATS   ENSEMBL   UCSC 
 
  330
 
  6
 
 36839
 
 gene;island
 
 0.367527
 
 0.5334327
 
 Not queried
 
  1.5449767
 
 2.40e-01
 
 0.4133265075
  
 
  
 1
 
  32930375
 
  32930544
 
  PDF   STATS   ENSEMBL   UCSC 
 
  170
 
  6
 
 1461
 
 tss;island
 
 0.022654
 
 0.0292741
 
 Not queried
 
  1.5416380
 
 2.40e-01
 
 0.4133265075
  
 
  
 7
 
   2499915
 
   2500200
 
  PDF   STATS   ENSEMBL   UCSC 
 
  286
 
  6
 
 38158
 
 island
 
 0.423974
 
 0.6433618
 
 Not queried
 
  1.4980990
 
 2.40e-01
 
 0.4133265075
  
 
  
 10
 
  75173098
 
  75173760
 
  PDF   STATS   ENSEMBL   UCSC 
 
  663
 
  6
 
 4634
 
 gene
 
 0.008041
 
 0.0038971
 
 Not queried
 
  1.4891156
 
 2.40e-01
 
 0.4133265075
  
 
  
 6
 
  13924355
 
  13924708
 
  PDF   STATS   ENSEMBL   UCSC 
 
  354
 
  6
 
 34647
 
 tss;island
 
 0.372135
 
 0.6290747
 
 Not queried
 
  1.4888018
 
 2.40e-01
 
 0.4133265075
  
 
  
 11
 
   1074682
 
   1074873
 
  PDF   STATS   ENSEMBL   UCSC 
 
  192
 
  6
 
 7038
 
 tss
 
 0.506712
 
 0.6923000
 
 Not queried
 
  1.4795659
 
 2.40e-01
 
 0.4133265075
  
 
  
 6
 
  43655599
 
  43655795
 
  PDF   STATS   ENSEMBL   UCSC 
 
  197
 
  6
 
 35005
 
 tss
 
 0.021555
 
 0.0041579
 
 Not queried
 
  1.4757712
 
 2.40e-01
 
 0.4133265075
  
 
  
 2
 
   1926366
 
   1926907
 
  PDF   STATS   ENSEMBL   UCSC 
 
  542
 
  6
 
 22541
 
 gene
 
 0.090198
 
 0.2031822
 
 Not queried
 
  1.4614751
 
 2.40e-01
 
 0.4133265075
  
 
  
 8
 
  82644373
 
  82644668
 
  PDF   STATS   ENSEMBL   UCSC 
 
  296
 
  6
 
 39786
 
 tss
 
 0.017761
 
 0.0059419
 
 Not queried
 
  1.4484777
 
 2.40e-01
 
 0.4133265075
  
 
  
 11
 
  15095017
 
  15095178
 
  PDF   STATS   ENSEMBL   UCSC 
 
  162
 
  6
 
 8082
 
 island
 
 0.135921
 
 0.0280108
 
 Not queried
 
  1.4105908
 
 2.40e-01
 
 0.4133265075
  
 
  
 7
 
  24796981
 
  24797884
 
  PDF   STATS   ENSEMBL   UCSC 
 
  904
 
 12
 
 38318
 
 island
 
 0.131219
 
 0.6970204
 
 Not queried
 
  2.1756592
 
 2.42e-01
 
 0.4149845107
  
 
  
 2
 
 131512651
 
 131513216
 
  PDF   STATS   ENSEMBL   UCSC 
 
  566
 
 12
 
 23629
 
 tss;island
 
 0.124054
 
 0.3834580
 
 Not queried
 
  2.1289528
 
 2.42e-01
 
 0.4149845107
  
 
  
 10
 
 123353298
 
 123353956
 
  PDF   STATS   ENSEMBL   UCSC 
 
  659
 
 12
 
 4793
 
 gene;island
 
 0.684553
 
 0.8153415
 
 Not queried
 
  1.4223923
 
 2.42e-01
 
 0.4149845107
  
 
  
 7
 
  24797102
 
  24797884
 
  PDF   STATS   ENSEMBL   UCSC 
 
  783
 
 11
 
 37478
 
 tss
 
 0.176079
 
 0.8381729
 
 Not queried
 
  2.5695071
 
 2.43e-01
 
 0.4165716700
  
 
  
 2
 
   6635591
 
   6636516
 
  PDF   STATS   ENSEMBL   UCSC 
 
  926
 
 11
 
 24043
 
 island
 
 0.772497
 
 0.8915564
 
 Not queried
 
  1.6368982
 
 2.43e-01
 
 0.4165716700
  
 
  
 11
 
  18230491
 
  18231021
 
  PDF   STATS   ENSEMBL   UCSC 
 
  531
 
 10
 
 8107
 
 island
 
 0.542638
 
 0.7781336
 
 Not queried
 
  2.6396328
 
 2.47e-01
 
 0.4242889032
  
 
  
 5
 
 170736021
 
 170736572
 
  PDF   STATS   ENSEMBL   UCSC 
 
  552
 
 10
 
 33379
 
 island
 
 0.192912
 
 0.1272363
 
 Not queried
 
  1.5099273
 
 2.47e-01
 
 0.4242889032
  
 
  
 19
 
   2702863
 
   2703086
 
  PDF   STATS   ENSEMBL   UCSC 
 
  224
 
 10
 
 20329
 
 tss;island
 
 0.008707
 
 0.0026313
 
 Not queried
 
  1.4179423
 
 2.47e-01
 
 0.4242889032
  
 
  
 1
 
  85527430
 
  85527943
 
  PDF   STATS   ENSEMBL   UCSC 
 
  514
 
  7
 
 1721
 
 tss
 
 0.068178
 
 0.0063141
 
 Not queried
 
  4.5922427
 
 2.59e-01
 
 0.4381244265
  
 
  
 3
 
  42947263
 
  42947771
 
  PDF   STATS   ENSEMBL   UCSC 
 
  509
 
  7
 
 29023
 
 island
 
 0.026679
 
 0.1609467
 
 Not queried
 
  4.2516309
 
 2.59e-01
 
 0.4381244265
  
 
  
 19
 
  47617042
 
  47617301
 
  PDF   STATS   ENSEMBL   UCSC 
 
  260
 
  7
 
 20902
 
 tss;island
 
 0.015233
 
 0.0040454
 
 Not queried
 
  3.5950381
 
 2.59e-01
 
 0.4381244265
  
 
  
 7
 
  29186153
 
  29186347
 
  PDF   STATS   ENSEMBL   UCSC 
 
  195
 
  7
 
 37510
 
 tss
 
 0.033840
 
 0.0193550
 
 Not queried
 
  3.1607620
 
 2.59e-01
 
 0.4381244265
  
 
  
 1
 
   1104257
 
   1104526
 
  PDF   STATS   ENSEMBL   UCSC 
 
  270
 
  7
 
 2410
 
 island
 
 0.129184
 
 0.5517690
 
 Not queried
 
  3.0533989
 
 2.59e-01
 
 0.4381244265
  
 
  
 8
 
  41504566
 
  41505042
 
  PDF   STATS   ENSEMBL   UCSC 
 
  477
 
  7
 
 40247
 
 island
 
 0.272223
 
 0.1138229
 
 Not queried
 
  3.0305523
 
 2.59e-01
 
 0.4381244265
  
 
  
 11
 
  31837104
 
  31837607
 
  PDF   STATS   ENSEMBL   UCSC 
 
  504
 
  7
 
 6563
 
 gene;island
 
 0.058747
 
 0.0338451
 
 Not queried
 
  2.7974401
 
 2.59e-01
 
 0.4381244265
  
 
  
 2
 
  63274476
 
  63275178
 
  PDF   STATS   ENSEMBL   UCSC 
 
  703
 
  7
 
 24335
 
 island
 
 0.151371
 
 0.0343217
 
 Not queried
 
  2.6249518
 
 2.59e-01
 
 0.4381244265
  
 
  
 12
 
 106751060
 
 106751428
 
  PDF   STATS   ENSEMBL   UCSC 
 
  369
 
  7
 
 9968
 
 tss;island
 
 0.002288
 
 0.0037429
 
 Not queried
 
  2.5329235
 
 2.59e-01
 
 0.4381244265
  
 
  
 6
 
  26183508
 
  26183899
 
  PDF   STATS   ENSEMBL   UCSC 
 
  392
 
  9
 
 34691
 
 tss
 
 0.019488
 
 0.0182204
 
 Not queried
 
  2.5150682
 
 2.58e-01
 
 0.4381244265
  
 
  
 19
 
  39694477
 
  39695237
 
  PDF   STATS   ENSEMBL   UCSC 
 
  761
 
  9
 
 21990
 
 island
 
 0.156392
 
 0.3870774
 
 Not queried
 
  2.4287140
 
 2.58e-01
 
 0.4381244265
  
 
  
 7
 
  27153580
 
  27153944
 
  PDF   STATS   ENSEMBL   UCSC 
 
  365
 
  7
 
 36930
 
 gene;island
 
 0.839740
 
 0.9500709
 
 Not queried
 
  2.2466197
 
 2.59e-01
 
 0.4381244265
  
 
  
 3
 
 154042288
 
 154042778
 
  PDF   STATS   ENSEMBL   UCSC 
 
  491
 
  9
 
 28668
 
 tss
 
 0.012358
 
 0.0276448
 
 Not queried
 
  2.2154292
 
 2.58e-01
 
 0.4381244265
  
 
  
 10
 
 134838014
 
 134838656
 
  PDF   STATS   ENSEMBL   UCSC 
 
  643
 
  7
 
 6326
 
 island
 
 0.261896
 
 0.3737896
 
 Not queried
 
  2.1563335
 
 2.59e-01
 
 0.4381244265
  
 
  
 1
 
 165324679
 
 165325231
 
  PDF   STATS   ENSEMBL   UCSC 
 
  553
 
  7
 
 3927
 
 island
 
 0.105957
 
 0.0264925
 
 Not queried
 
  2.1416820
 
 2.59e-01
 
 0.4381244265
  
 
  
 7
 
 157467883
 
 157468443
 
  PDF   STATS   ENSEMBL   UCSC 
 
  561
 
  7
 
 37339
 
 gene
 
 0.556969
 
 0.5872824
 
 Not queried
 
  2.1157615
 
 2.59e-01
 
 0.4381244265
  
 
  
 3
 
 187870621
 
 187871034
 
  PDF   STATS   ENSEMBL   UCSC 
 
  414
 
  7
 
 28780
 
 tss;island
 
 0.400495
 
 0.6164564
 
 Not queried
 
  2.0433805
 
 2.59e-01
 
 0.4381244265
  
 
  
 12
 
  49371987
 
  49372474
 
  PDF   STATS   ENSEMBL   UCSC 
 
  488
 
  9
 
 10380
 
 island
 
 0.139749
 
 0.0417224
 
 Not queried
 
  2.0074668
 
 2.58e-01
 
 0.4381244265
  
 
  
 6
 
 150463775
 
 150464124
 
  PDF   STATS   ENSEMBL   UCSC 
 
  350
 
  7
 
 35296
 
 tss
 
 0.054533
 
 0.0243810
 
 Not queried
 
  1.8897487
 
 2.59e-01
 
 0.4381244265
  
 
  
 17
 
  40932199
 
  40932746
 
  PDF   STATS   ENSEMBL   UCSC 
 
  548
 
  9
 
 18353
 
 island
 
 0.054793
 
 0.4925054
 
 Not queried
 
  1.8886272
 
 2.58e-01
 
 0.4381244265
  
 
  
 10
 
  38382849
 
  38383250
 
  PDF   STATS   ENSEMBL   UCSC 
 
  402
 
  9
 
 5036
 
 tss
 
 0.010948
 
 0.0041865
 
 Not queried
 
  1.8770299
 
 2.58e-01
 
 0.4381244265
  
 
  
 2
 
 232651086
 
 232651190
 
  PDF   STATS   ENSEMBL   UCSC 
 
  105
 
  7
 
 25188
 
 island
 
 0.002624
 
 0.0013041
 
 Not queried
 
  1.8724473
 
 2.59e-01
 
 0.4381244265
  
 
  
 20
 
  42543034
 
  42543446
 
  PDF   STATS   ENSEMBL   UCSC 
 
  413
 
  7
 
 25752
 
 tss;island
 
 0.032186
 
 0.0195695
 
 Not queried
 
  1.8617114
 
 2.59e-01
 
 0.4381244265
  
 
  
 7
 
 157320088
 
 157320662
 
  PDF   STATS   ENSEMBL   UCSC 
 
  575
 
  7
 
 39106
 
 island
 
 0.218449
 
 0.4338374
 
 Not queried
 
  1.8471076
 
 2.59e-01
 
 0.4381244265
  
 
  
 14
 
 101923155
 
 101923702
 
  PDF   STATS   ENSEMBL   UCSC 
 
  548
 
  7
 
 13157
 
 island
 
 0.048938
 
 0.0868360
 
 Not queried
 
  1.7345566
 
 2.59e-01
 
 0.4381244265
  
 
  
 11
 
  31839521
 
  31839947
 
  PDF   STATS   ENSEMBL   UCSC 
 
  427
 
  7
 
 7224
 
 tss
 
 0.511753
 
 0.1100228
 
 Not queried
 
  1.7199569
 
 2.59e-01
 
 0.4381244265
  
 
  
 2
 
   3063115
 
   3063671
 
  PDF   STATS   ENSEMBL   UCSC 
 
  557
 
  9
 
 24025
 
 island
 
 0.524099
 
 0.8205761
 
 Not queried
 
  1.6897910
 
 2.58e-01
 
 0.4381244265
  
 
  
 2
 
 190445477
 
 190446181
 
  PDF   STATS   ENSEMBL   UCSC 
 
  705
 
  7
 
 24955
 
 island
 
 0.016070
 
 0.0516899
 
 Not queried
 
  1.6850424
 
 2.59e-01
 
 0.4381244265
  
 
  
 20
 
  55107833
 
  55108228
 
  PDF   STATS   ENSEMBL   UCSC 
 
  396
 
  7
 
 25838
 
 tss
 
 0.597362
 
 0.8263823
 
 Not queried
 
  1.6558962
 
 2.59e-01
 
 0.4381244265
  
 
  
 13
 
  84456127
 
  84456722
 
  PDF   STATS   ENSEMBL   UCSC 
 
  596
 
  9
 
 11806
 
 island
 
 0.066354
 
 0.0369046
 
 Not queried
 
  1.5819526
 
 2.58e-01
 
 0.4381244265
  
 
  
 12
 
 132900274
 
 132901088
 
  PDF   STATS   ENSEMBL   UCSC 
 
  815
 
  7
 
 9448
 
 gene;island
 
 0.470661
 
 0.5935848
 
 Not queried
 
  1.5614823
 
 2.59e-01
 
 0.4381244265
  
 
  
 10
 
     95030
 
     95398
 
  PDF   STATS   ENSEMBL   UCSC 
 
  369
 
  7
 
 4429
 
 gene;island
 
 0.250066
 
 0.5534574
 
 Not queried
 
  1.4788688
 
 2.59e-01
 
 0.4381244265
  
 
  
 12
 
  49582874
 
  49583112
 
  PDF   STATS   ENSEMBL   UCSC 
 
  239
 
  7
 
 9684
 
 tss
 
 0.011851
 
 0.0061522
 
 Not queried
 
  1.4658978
 
 2.59e-01
 
 0.4381244265
  
 
  
 3
 
  44626029
 
  44626453
 
  PDF   STATS   ENSEMBL   UCSC 
 
  425
 
  7
 
 28257
 
 tss
 
 0.015951
 
 0.0419183
 
 Not queried
 
  1.4358765
 
 2.59e-01
 
 0.4381244265
  
 
  
 14
 
 105154557
 
 105155327
 
  PDF   STATS   ENSEMBL   UCSC 
 
  771
 
  7
 
 12660
 
 tss;island
 
 0.675261
 
 0.9098583
 
 Not queried
 
  1.4103886
 
 2.59e-01
 
 0.4381244265
  
 
  
 6
 
 168418276
 
 168418527
 
  PDF   STATS   ENSEMBL   UCSC 
 
  252
 
  7
 
 35346
 
 tss
 
 0.192324
 
 0.3800862
 
 Not queried
 
  1.4101920
 
 2.59e-01
 
 0.4381244265
  
 
  
 11
 
    843897
 
    844686
 
  PDF   STATS   ENSEMBL   UCSC 
 
  790
 
 11
 
 6393
 
 gene;island
 
 0.022883
 
 0.4512571
 
 Not queried
 
  3.8758189
 
 2.70e-01
 
 0.4563244066
  
 
  
 6
 
  28973318
 
  28973524
 
  PDF   STATS   ENSEMBL   UCSC 
 
  207
 
 11
 
 34746
 
 tss
 
 0.012767
 
 0.0253975
 
 Not queried
 
  1.8046648
 
 2.70e-01
 
 0.4563244066
  
 
  
 6
 
  33043072
 
  33043220
 
  PDF   STATS   ENSEMBL   UCSC 
 
  149
 
  4
 
 34888
 
 tss
 
 0.002132
 
 0.3019556
 
 Not queried
 
  6.6278086
 
 3.43e-01
 
 0.4672865596
  
 
  
 17
 
  53800041
 
  53800554
 
  PDF   STATS   ENSEMBL   UCSC 
 
  514
 
  5
 
 16701
 
 gene
 
 0.492922
 
 0.0387231
 
 Not queried
 
  6.4307896
 
 3.10e-01
 
 0.4672865596
  
 
  
 17
 
  37856133
 
  37856230
 
  PDF   STATS   ENSEMBL   UCSC 
 
   98
 
  4
 
 17314
 
 tss
 
 0.455261
 
 0.0266922
 
 Not queried
 
  5.8587140
 
 3.43e-01
 
 0.4672865596
  
 
  
 1
 
 167090972
 
 167091161
 
  PDF   STATS   ENSEMBL   UCSC 
 
  190
 
  4
 
 873
 
 gene;island
 
 0.012627
 
 0.6329090
 
 Not queried
 
  5.8128990
 
 3.43e-01
 
 0.4672865596
  
 
  
 7
 
 114561804
 
 114562063
 
  PDF   STATS   ENSEMBL   UCSC 
 
  260
 
  5
 
 37847
 
 tss;island
 
 0.565096
 
 0.9722679
 
 Not queried
 
  5.5117249
 
 3.10e-01
 
 0.4672865596
  
 
  
 13
 
 108866993
 
 108867154
 
  PDF   STATS   ENSEMBL   UCSC 
 
  162
 
  4
 
 11221
 
 gene;island
 
 0.892977
 
 0.4940247
 
 Not queried
 
  5.1873484
 
 3.43e-01
 
 0.4672865596
  
 
  
 12
 
 119772354
 
 119772421
 
  PDF   STATS   ENSEMBL   UCSC 
 
   68
 
  4
 
 10050
 
 tss
 
 0.011164
 
 0.3469812
 
 Not queried
 
  5.1054373
 
 3.43e-01
 
 0.4672865596
  
 
  
 1
 
 154539974
 
 154540159
 
  PDF   STATS   ENSEMBL   UCSC 
 
  186
 
  4
 
 1967
 
 tss
 
 0.040628
 
 0.5109139
 
 Not queried
 
  5.0947582
 
 3.43e-01
 
 0.4672865596
  
 
  
 2
 
  66809780
 
  66809985
 
  PDF   STATS   ENSEMBL   UCSC 
 
  206
 
  4
 
 24358
 
 island
 
 0.005937
 
 0.2516030
 
 Not queried
 
  5.0697280
 
 3.43e-01
 
 0.4672865596
  
 
  
 12
 
 119772354
 
 119772577
 
  PDF   STATS   ENSEMBL   UCSC 
 
  224
 
  5
 
 10874
 
 island
 
 0.017043
 
 0.5616242
 
 Not queried
 
  4.9226255
 
 3.10e-01
 
 0.4672865596
  
 
  
 8
 
 144654887
 
 144655030
 
  PDF   STATS   ENSEMBL   UCSC 
 
  144
 
  4
 
 40672
 
 island
 
 0.160018
 
 0.8568316
 
 Not queried
 
  4.8781021
 
 3.43e-01
 
 0.4672865596
  
 
  
 11
 
  66444632
 
  66444806
 
  PDF   STATS   ENSEMBL   UCSC 
 
  175
 
  4
 
 6735
 
 gene;island
 
 0.031232
 
 0.0039076
 
 Not queried
 
  4.7738755
 
 3.43e-01
 
 0.4672865596
  
 
  
 4
 
   4866047
 
   4866192
 
  PDF   STATS   ENSEMBL   UCSC 
 
  146
 
  4
 
 30871
 
 island
 
 0.815303
 
 0.0199716
 
 Not queried
 
  4.7701144
 
 3.43e-01
 
 0.4672865596
  
 
  
 11
 
  64058878
 
  64059057
 
  PDF   STATS   ENSEMBL   UCSC 
 
  180
 
  4
 
 6677
 
 gene
 
 0.098692
 
 0.2654192
 
 Not queried
 
  4.5019930
 
 3.43e-01
 
 0.4672865596
  
 
  
 11
 
  78673073
 
  78673290
 
  PDF   STATS   ENSEMBL   UCSC 
 
  218
 
  4
 
 6847
 
 gene;island
 
 0.112673
 
 0.0156897
 
 Not queried
 
  4.3743230
 
 3.43e-01
 
 0.4672865596
  
 
  
 2
 
 165812079
 
 165812297
 
  PDF   STATS   ENSEMBL   UCSC 
 
  219
 
  4
 
 23694
 
 tss
 
 0.037549
 
 0.6399491
 
 Not queried
 
  4.3672582
 
 3.43e-01
 
 0.4672865596
  
 
  
 15
 
  40674878
 
  40674910
 
  PDF   STATS   ENSEMBL   UCSC 
 
   33
 
  4
 
 13588
 
 tss
 
 0.298435
 
 0.7076900
 
 Not queried
 
  4.3422861
 
 3.43e-01
 
 0.4672865596
  
 
  
 14
 
  57274550
 
  57274900
 
  PDF   STATS   ENSEMBL   UCSC 
 
  351
 
  5
 
 12091
 
 gene;island
 
 0.095191
 
 0.0301228
 
 Not queried
 
  4.2847446
 
 3.10e-01
 
 0.4672865596
  
 
  
 14
 
  92790072
 
  92790364
 
  PDF   STATS   ENSEMBL   UCSC 
 
  293
 
  4
 
 12181
 
 gene;island
 
 0.005971
 
 0.1646884
 
 Not queried
 
  4.2700307
 
 3.43e-01
 
 0.4672865596
  
 
  
 1
 
 210501936
 
 210502156
 
  PDF   STATS   ENSEMBL   UCSC 
 
  221
 
  5
 
 991
 
 gene;tss;island
 
 0.044014
 
 0.0102948
 
 Not queried
 
  4.1727595
 
 3.10e-01
 
 0.4672865596
  
 
  
 1
 
 100111713
 
 100111949
 
  PDF   STATS   ENSEMBL   UCSC 
 
  237
 
  4
 
 648
 
 gene
 
 0.006698
 
 0.3070426
 
 Not queried
 
  4.1717407
 
 3.43e-01
 
 0.4672865596
  
 
  
 17
 
  46657274
 
  46657656
 
  PDF   STATS   ENSEMBL   UCSC 
 
  383
 
  5
 
 18482
 
 island
 
 0.065422
 
 0.5492457
 
 Not queried
 
  4.1681024
 
 3.10e-01
 
 0.4672865596
  
 
  
 10
 
  27541298
 
  27541418
 
  PDF   STATS   ENSEMBL   UCSC 
 
  121
 
  4
 
 5001
 
 tss
 
 0.945614
 
 0.5740699
 
 Not queried
 
  4.1447838
 
 3.43e-01
 
 0.4672865596
  
 
  
 2
 
  38892846
 
  38892958
 
  PDF   STATS   ENSEMBL   UCSC 
 
  113
 
  4
 
 23312
 
 tss
 
 0.048212
 
 0.6300073
 
 Not queried
 
  4.1345167
 
 3.43e-01
 
 0.4672865596
  
 
  
 17
 
  46659751
 
  46660002
 
  PDF   STATS   ENSEMBL   UCSC 
 
  252
 
  4
 
 18483
 
 island
 
 0.097834
 
 0.4481147
 
 Not queried
 
  4.1066071
 
 3.43e-01
 
 0.4672865596
  
 
  
 5
 
 140613740
 
 140614106
 
  PDF   STATS   ENSEMBL   UCSC 
 
  367
 
  8
 
 33226
 
 island
 
 0.021037
 
 0.2591885
 
 Not queried
 
  4.0585146
 
 3.28e-01
 
 0.4672865596
  
 
  
 19
 
  39785679
 
  39786136
 
  PDF   STATS   ENSEMBL   UCSC 
 
  458
 
  4
 
 20768
 
 tss
 
 0.294433
 
 0.6238974
 
 Not queried
 
  4.0138298
 
 3.43e-01
 
 0.4672865596
  
 
  
 8
 
  97169621
 
  97170097
 
  PDF   STATS   ENSEMBL   UCSC 
 
  477
 
  5
 
 39366
 
 gene;island
 
 0.224333
 
 0.0128583
 
 Not queried
 
  3.9964705
 
 3.10e-01
 
 0.4672865596
  
 
  
 2
 
 154336045
 
 154336401
 
  PDF   STATS   ENSEMBL   UCSC 
 
  357
 
  5
 
 23669
 
 tss;island
 
 0.035778
 
 0.6328532
 
 Not queried
 
  3.9547187
 
 3.10e-01
 
 0.4672865596
  
 
  
 1
 
 233462575
 
 233462803
 
  PDF   STATS   ENSEMBL   UCSC 
 
  229
 
  5
 
 2326
 
 tss;island
 
 0.655854
 
 0.5343208
 
 Not queried
 
  3.9033325
 
 3.10e-01
 
 0.4672865596
  
 
  
 1
 
  62660556
 
  62660861
 
  PDF   STATS   ENSEMBL   UCSC 
 
  306
 
  4
 
 562
 
 gene
 
 0.032150
 
 0.3760301
 
 Not queried
 
  3.8916816
 
 3.43e-01
 
 0.4672865596
  
 
  
 6
 
  33143721
 
  33143807
 
  PDF   STATS   ENSEMBL   UCSC 
 
   87
 
  4
 
 34204
 
 gene
 
 0.261299
 
 0.6283601
 
 Not queried
 
  3.7854758
 
 3.43e-01
 
 0.4672865596
  
 
  
 11
 
  65408429
 
  65408566
 
  PDF   STATS   ENSEMBL   UCSC 
 
  138
 
  4
 
 6714
 
 gene;island
 
 0.010906
 
 0.2196516
 
 Not queried
 
  3.7842647
 
 3.43e-01
 
 0.4672865596
  
 
  
 18
 
   3447454
 
   3447713
 
  PDF   STATS   ENSEMBL   UCSC 
 
  260
 
  4
 
 19040
 
 gene;island
 
 0.018586
 
 0.0957538
 
 Not queried
 
  3.7441975
 
 3.43e-01
 
 0.4672865596
  
 
  
 17
 
  56232260
 
  56232499
 
  PDF   STATS   ENSEMBL   UCSC 
 
  240
 
  4
 
 17532
 
 tss
 
 0.008439
 
 0.1930153
 
 Not queried
 
  3.6966744
 
 3.43e-01
 
 0.4672865596
  
 
  
 17
 
  79031376
 
  79031712
 
  PDF   STATS   ENSEMBL   UCSC 
 
  337
 
  5
 
 16890
 
 gene
 
 0.028216
 
 0.3014919
 
 Not queried
 
  3.6794624
 
 3.10e-01
 
 0.4672865596
  
 
  
 1
 
  46951291
 
  46951683
 
  PDF   STATS   ENSEMBL   UCSC 
 
  393
 
  6
 
 3219
 
 island
 
 0.259827
 
 0.0539460
 
 Not queried
 
  3.6591432
 
 3.10e-01
 
 0.4672865596
  
 
  
 7
 
  55640396
 
  55640714
 
  PDF   STATS   ENSEMBL   UCSC 
 
  319
 
  4
 
 37604
 
 tss;island
 
 0.046584
 
 0.0211315
 
 Not queried
 
  3.6011830
 
 3.43e-01
 
 0.4672865596
  
 
  
 8
 
  35092687
 
  35092876
 
  PDF   STATS   ENSEMBL   UCSC 
 
  190
 
  4
 
 39643
 
 tss;island
 
 0.057986
 
 0.7298806
 
 Not queried
 
  3.5932784
 
 3.43e-01
 
 0.4672865596
  
 
  
 5
 
  66124395
 
  66124588
 
  PDF   STATS   ENSEMBL   UCSC 
 
  194
 
  5
 
 32102
 
 tss
 
 0.002604
 
 0.0124195
 
 Not queried
 
  3.5876013
 
 3.10e-01
 
 0.4672865596
  
 
  
 1
 
  39284267
 
  39284609
 
  PDF   STATS   ENSEMBL   UCSC 
 
  343
 
  4
 
 3092
 
 island
 
 0.016437
 
 0.2738193
 
 Not queried
 
  3.5836212
 
 3.43e-01
 
 0.4672865596
  
 
  
 15
 
  90437601
 
  90437891
 
  PDF   STATS   ENSEMBL   UCSC 
 
  291
 
  6
 
 14443
 
 island
 
 0.169234
 
 0.0110290
 
 Not queried
 
  3.5835288
 
 3.10e-01
 
 0.4672865596
  
 
  
 1
 
  91195159
 
  91195683
 
  PDF   STATS   ENSEMBL   UCSC 
 
  525
 
  5
 
 3465
 
 island
 
 0.228807
 
 0.0715302
 
 Not queried
 
  3.5687826
 
 3.10e-01
 
 0.4672865596
  
 
  
 1
 
  11752109
 
  11752494
 
  PDF   STATS   ENSEMBL   UCSC 
 
  386
 
  4
 
 226
 
 gene;tss;island
 
 0.018022
 
 0.2666639
 
 Not queried
 
  3.5473751
 
 3.43e-01
 
 0.4672865596
  
 
  
 7
 
   1708566
 
   1708892
 
  PDF   STATS   ENSEMBL   UCSC 
 
  327
 
  4
 
 38122
 
 island
 
 0.437782
 
 0.0691259
 
 Not queried
 
  3.5232593
 
 3.43e-01
 
 0.4672865596
  
 
  
 1
 
  21835945
 
  21836326
 
  PDF   STATS   ENSEMBL   UCSC 
 
  382
 
  4
 
 297
 
 gene;island
 
 0.246608
 
 0.0329425
 
 Not queried
 
  3.5199990
 
 3.43e-01
 
 0.4672865596
  
 
  
 4
 
   1800154
 
   1800448
 
  PDF   STATS   ENSEMBL   UCSC 
 
  295
 
  4
 
 29875
 
 gene;island
 
 0.233785
 
 0.6175343
 
 Not queried
 
  3.5066262
 
 3.43e-01
 
 0.4672865596
  
 
  
 15
 
  79382548
 
  79383167
 
  PDF   STATS   ENSEMBL   UCSC 
 
  620
 
  7
 
 13447
 
 gene;island
 
 0.018426
 
 0.0168678
 
 Not queried
 
  3.4808704
 
 3.18e-01
 
 0.4672865596
  
 
  
 16
 
   1480790
 
   1480992
 
  PDF   STATS   ENSEMBL   UCSC 
 
  203
 
  4
 
 15542
 
 island
 
 0.537876
 
 0.0792839
 
 Not queried
 
  3.4774135
 
 3.43e-01
 
 0.4672865596
  
 
  
 11
 
  31822243
 
  31822526
 
  PDF   STATS   ENSEMBL   UCSC 
 
  284
 
  4
 
 6557
 
 gene;island
 
 0.703707
 
 0.3298915
 
 Not queried
 
  3.4618754
 
 3.43e-01
 
 0.4672865596
  
 
  
 20
 
  54824316
 
  54824646
 
  PDF   STATS   ENSEMBL   UCSC 
 
  331
 
  4
 
 25460
 
 gene;island
 
 0.032035
 
 0.3896165
 
 Not queried
 
  3.4347252
 
 3.43e-01
 
 0.4672865596
  
 
  
 15
 
  25425326
 
  25425615
 
  PDF   STATS   ENSEMBL   UCSC 
 
  290
 
  4
 
 13527
 
 tss
 
 0.137366
 
 0.3158142
 
 Not queried
 
  3.4154570
 
 3.43e-01
 
 0.4672865596
  
 
  
 20
 
  44540794
 
  44541153
 
  PDF   STATS   ENSEMBL   UCSC 
 
  360
 
  5
 
 25783
 
 tss
 
 0.019293
 
 0.2967710
 
 Not queried
 
  3.4031438
 
 3.10e-01
 
 0.4672865596
  
 
  
 11
 
  22359188
 
  22359658
 
  PDF   STATS   ENSEMBL   UCSC 
 
  471
 
  7
 
 7202
 
 tss;island
 
 0.105813
 
 0.0552101
 
 Not queried
 
  3.3854893
 
 3.18e-01
 
 0.4672865596
  
 
  
 2
 
  74153086
 
  74153317
 
  PDF   STATS   ENSEMBL   UCSC 
 
  232
 
  4
 
 23447
 
 tss;island
 
 0.799919
 
 0.9688409
 
 Not queried
 
  3.3626096
 
 3.43e-01
 
 0.4672865596
  
 
  
 10
 
  72141375
 
  72141625
 
  PDF   STATS   ENSEMBL   UCSC 
 
  251
 
  4
 
 4618
 
 gene;island
 
 0.499075
 
 0.1608556
 
 Not queried
 
  3.3310373
 
 3.43e-01
 
 0.4672865596
  
 
  
 1
 
 202172778
 
 202172912
 
  PDF   STATS   ENSEMBL   UCSC 
 
  135
 
  4
 
 944
 
 gene
 
 0.339670
 
 0.0278806
 
 Not queried
 
  3.3297804
 
 3.43e-01
 
 0.4672865596
  
 
  
 7
 
  27134109
 
  27134369
 
  PDF   STATS   ENSEMBL   UCSC 
 
  261
 
  4
 
 36926
 
 gene;island
 
 0.518766
 
 0.9322212
 
 Not queried
 
  3.3290203
 
 3.43e-01
 
 0.4672865596
  
 
  
 10
 
  30721971
 
  30722286
 
  PDF   STATS   ENSEMBL   UCSC 
 
  316
 
  4
 
 5012
 
 tss;island
 
 0.532196
 
 0.0362266
 
 Not queried
 
  3.3240295
 
 3.43e-01
 
 0.4672865596
  
 
  
 7
 
  43151725
 
  43152125
 
  PDF   STATS   ENSEMBL   UCSC 
 
  401
 
  6
 
 37555
 
 tss
 
 0.033469
 
 0.5502725
 
 Not queried
 
  3.2829981
 
 3.10e-01
 
 0.4672865596
  
 
  
 2
 
  80530701
 
  80530948
 
  PDF   STATS   ENSEMBL   UCSC 
 
  248
 
  4
 
 22755
 
 gene;island
 
 0.013212
 
 0.1801262
 
 Not queried
 
  3.2829079
 
 3.43e-01
 
 0.4672865596
  
 
  
 10
 
 124906421
 
 124906930
 
  PDF   STATS   ENSEMBL   UCSC 
 
  510
 
  6
 
 5416
 
 tss;island
 
 0.092780
 
 0.0125162
 
 Not queried
 
  3.2632354
 
 3.10e-01
 
 0.4672865596
  
 
  
 2
 
 220299484
 
 220299900
 
  PDF   STATS   ENSEMBL   UCSC 
 
  417
 
  9
 
 25124
 
 island
 
 0.100604
 
 0.0410133
 
 Not queried
 
  3.2556915
 
 3.40e-01
 
 0.4672865596
  
 
  
 4
 
  13538730
 
  13539255
 
  PDF   STATS   ENSEMBL   UCSC 
 
  526
 
  7
 
 30932
 
 island
 
 0.175480
 
 0.2001467
 
 Not queried
 
  3.2500824
 
 3.18e-01
 
 0.4672865596
  
 
  
 1
 
  82268588
 
  82268801
 
  PDF   STATS   ENSEMBL   UCSC 
 
  214
 
  4
 
 598
 
 gene;island
 
 0.015403
 
 0.2946156
 
 Not queried
 
  3.2496032
 
 3.43e-01
 
 0.4672865596
  
 
  
 7
 
  99766376
 
  99766665
 
  PDF   STATS   ENSEMBL   UCSC 
 
  290
 
  5
 
 37774
 
 tss
 
 0.154572
 
 0.6764655
 
 Not queried
 
  3.1990158
 
 3.10e-01
 
 0.4672865596
  
 
  
 17
 
  41739130
 
  41739326
 
  PDF   STATS   ENSEMBL   UCSC 
 
  197
 
  4
 
 17392
 
 tss
 
 0.172413
 
 0.4886118
 
 Not queried
 
  3.1947197
 
 3.43e-01
 
 0.4672865596
  
 
  
 14
 
  90168304
 
  90168492
 
  PDF   STATS   ENSEMBL   UCSC 
 
  189
 
  4
 
 13067
 
 island
 
 0.528003
 
 0.1400913
 
 Not queried
 
  3.1932985
 
 3.43e-01
 
 0.4672865596
  
 
  
 19
 
  12946231
 
  12946465
 
  PDF   STATS   ENSEMBL   UCSC 
 
  235
 
  6
 
 20515
 
 tss
 
 0.143776
 
 0.0392447
 
 Not queried
 
  3.1868902
 
 3.10e-01
 
 0.4672865596
  
 
  
 15
 
  58723657
 
  58724041
 
  PDF   STATS   ENSEMBL   UCSC 
 
  385
 
  4
 
 13698
 
 tss
 
 0.446383
 
 0.8760834
 
 Not queried
 
  3.1812270
 
 3.43e-01
 
 0.4672865596
  
 
  
 17
 
  58216297
 
  58216651
 
  PDF   STATS   ENSEMBL   UCSC 
 
  355
 
  4
 
 18618
 
 island
 
 0.443435
 
 0.0811534
 
 Not queried
 
  3.1744987
 
 3.43e-01
 
 0.4672865596
  
 
  
 22
 
  38218945
 
  38219336
 
  PDF   STATS   ENSEMBL   UCSC 
 
  392
 
  4
 
 27126
 
 tss
 
 0.157393
 
 0.5301157
 
 Not queried
 
  3.1687503
 
 3.43e-01
 
 0.4672865596
  
 
  
 2
 
 220174201
 
 220174251
 
  PDF   STATS   ENSEMBL   UCSC 
 
   51
 
  4
 
 23904
 
 tss;island
 
 0.061714
 
 0.2258709
 
 Not queried
 
  3.1614054
 
 3.43e-01
 
 0.4672865596
  
 
  
 17
 
   1202015
 
   1202252
 
  PDF   STATS   ENSEMBL   UCSC 
 
  238
 
  4
 
 16312
 
 gene
 
 0.457696
 
 0.0888748
 
 Not queried
 
  3.1611107
 
 3.43e-01
 
 0.4672865596
  
 
  
 11
 
 119227592
 
 119227876
 
  PDF   STATS   ENSEMBL   UCSC 
 
  285
 
  4
 
 6939
 
 gene;island
 
 0.016618
 
 0.0716363
 
 Not queried
 
  3.1332629
 
 3.43e-01
 
 0.4672865596
  
 
  
 2
 
 109647956
 
 109648208
 
  PDF   STATS   ENSEMBL   UCSC 
 
  253
 
  4
 
 24604
 
 island
 
 0.015818
 
 0.2917285
 
 Not queried
 
  3.1327712
 
 3.43e-01
 
 0.4672865596
  
 
  
 2
 
 176986261
 
 176986659
 
  PDF   STATS   ENSEMBL   UCSC 
 
  399
 
  4
 
 23736
 
 tss;island
 
 0.572285
 
 0.2527560
 
 Not queried
 
  3.1249468
 
 3.43e-01
 
 0.4672865596
  
 
  
 3
 
  42705828
 
  42706106
 
  PDF   STATS   ENSEMBL   UCSC 
 
  279
 
  4
 
 27735
 
 gene
 
 0.346372
 
 0.0373939
 
 Not queried
 
  3.1178348
 
 3.43e-01
 
 0.4672865596
  
 
  
 11
 
  16632311
 
  16632724
 
  PDF   STATS   ENSEMBL   UCSC 
 
  414
 
  4
 
 8091
 
 island
 
 0.214565
 
 0.3810090
 
 Not queried
 
  3.1137160
 
 3.43e-01
 
 0.4672865596
  
 
  
 19
 
  51231557
 
  51232007
 
  PDF   STATS   ENSEMBL   UCSC 
 
  451
 
  5
 
 22322
 
 island
 
 0.364422
 
 0.6508143
 
 Not queried
 
  3.1106793
 
 3.10e-01
 
 0.4672865596
  
 
  
 1
 
   6639570
 
   6639762
 
  PDF   STATS   ENSEMBL   UCSC 
 
  193
 
  4
 
 179
 
 gene
 
 0.022654
 
 0.2939859
 
 Not queried
 
  3.1102119
 
 3.43e-01
 
 0.4672865596
  
 
  
 1
 
 180201002
 
 180201319
 
  PDF   STATS   ENSEMBL   UCSC 
 
  318
 
  4
 
 905
 
 gene;island
 
 0.247612
 
 0.0161127
 
 Not queried
 
  3.0868826
 
 3.43e-01
 
 0.4672865596
  
 
  
 14
 
  77279317
 
  77279508
 
  PDF   STATS   ENSEMBL   UCSC 
 
  192
 
  4
 
 12525
 
 tss
 
 0.019999
 
 0.1231417
 
 Not queried
 
  3.0796243
 
 3.43e-01
 
 0.4672865596
  
 
  
 6
 
 168435914
 
 168436099
 
  PDF   STATS   ENSEMBL   UCSC 
 
  186
 
  4
 
 34558
 
 gene;island
 
 0.202853
 
 0.5737327
 
 Not queried
 
  3.0667669
 
 3.43e-01
 
 0.4672865596
  
 
  
 2
 
 235860416
 
 235860554
 
  PDF   STATS   ENSEMBL   UCSC 
 
  139
 
  5
 
 23963
 
 tss;island
 
 0.003347
 
 0.0218814
 
 Not queried
 
  3.0603730
 
 3.10e-01
 
 0.4672865596
  
 
  
 14
 
 105116395
 
 105116681
 
  PDF   STATS   ENSEMBL   UCSC 
 
  287
 
  4
 
 13210
 
 island
 
 0.008708
 
 0.0560845
 
 Not queried
 
  3.0381109
 
 3.43e-01
 
 0.4672865596
  
 
  
 1
 
 205181033
 
 205181261
 
  PDF   STATS   ENSEMBL   UCSC 
 
  229
 
  4
 
 2202
 
 tss;island
 
 0.045024
 
 0.0068030
 
 Not queried
 
  3.0335030
 
 3.43e-01
 
 0.4672865596
  
 
  
 10
 
 116164598
 
 116164955
 
  PDF   STATS   ENSEMBL   UCSC 
 
  358
 
  6
 
 5377
 
 tss;island
 
 0.531697
 
 0.2301978
 
 Not queried
 
  3.0308075
 
 3.10e-01
 
 0.4672865596
  
 
  
 10
 
 114043123
 
 114043435
 
  PDF   STATS   ENSEMBL   UCSC 
 
  313
 
  5
 
 5364
 
 tss
 
 0.037647
 
 0.2884384
 
 Not queried
 
  3.0297746
 
 3.10e-01
 
 0.4672865596
  
 
  
 1
 
  63249693
 
  63249793
 
  PDF   STATS   ENSEMBL   UCSC 
 
  101
 
  4
 
 1667
 
 tss
 
 0.000480
 
 0.0145815
 
 Not queried
 
  3.0248157
 
 3.43e-01
 
 0.4672865596
  
 
  
 3
 
  55517496
 
  55517853
 
  PDF   STATS   ENSEMBL   UCSC 
 
  358
 
  4
 
 27838
 
 gene;island
 
 0.007897
 
 0.1784096
 
 Not queried
 
  3.0242071
 
 3.43e-01
 
 0.4672865596
  
 
  
 12
 
 124990897
 
 124991139
 
  PDF   STATS   ENSEMBL   UCSC 
 
  243
 
  4
 
 9399
 
 gene
 
 0.214400
 
 0.7424805
 
 Not queried
 
  3.0101146
 
 3.43e-01
 
 0.4672865596
  
 
  
 1
 
 247578859
 
 247579312
 
  PDF   STATS   ENSEMBL   UCSC 
 
  454
 
  4
 
 2368
 
 tss
 
 0.020402
 
 0.4114587
 
 Not queried
 
  3.0020364
 
 3.43e-01
 
 0.4672865596
  
 
  
 7
 
  32111001
 
  32111453
 
  PDF   STATS   ENSEMBL   UCSC 
 
  453
 
  7
 
 37524
 
 tss
 
 0.104076
 
 0.4828719
 
 Not queried
 
  2.9798743
 
 3.18e-01
 
 0.4672865596
  
 
  
 2
 
 160654866
 
 160655081
 
  PDF   STATS   ENSEMBL   UCSC 
 
  216
 
  4
 
 23682
 
 tss;island
 
 0.503513
 
 0.1355615
 
 Not queried
 
  2.9711486
 
 3.43e-01
 
 0.4672865596
  
 
  
 7
 
   1198522
 
   1198977
 
  PDF   STATS   ENSEMBL   UCSC 
 
  456
 
  5
 
 36752
 
 gene;island
 
 0.060816
 
 0.4282235
 
 Not queried
 
  2.9636299
 
 3.10e-01
 
 0.4672865596
  
 
  
 1
 
  59042745
 
  59043370
 
  PDF   STATS   ENSEMBL   UCSC 
 
  626
 
 10
 
 3314
 
 island
 
 0.369821
 
 0.4224662
 
 Not queried
 
  2.9601249
 
 2.80e-01
 
 0.4672865596
  
 
  
 18
 
  67069959
 
  67070461
 
  PDF   STATS   ENSEMBL   UCSC 
 
  503
 
  6
 
 19123
 
 gene;island
 
 0.057877
 
 0.3145806
 
 Not queried
 
  2.9536644
 
 3.10e-01
 
 0.4672865596
  
 
  
 17
 
  47113407
 
  47113596
 
  PDF   STATS   ENSEMBL   UCSC 
 
  190
 
  4
 
 16671
 
 gene;island
 
 0.303094
 
 0.5897038
 
 Not queried
 
  2.9415301
 
 3.43e-01
 
 0.4672865596
  
 
  
 7
 
   4848683
 
   4848939
 
  PDF   STATS   ENSEMBL   UCSC 
 
  257
 
  4
 
 36857
 
 gene;island
 
 0.310457
 
 0.0189786
 
 Not queried
 
  2.9374443
 
 3.43e-01
 
 0.4672865596
  
 
  
 20
 
  44540777
 
  44541153
 
  PDF   STATS   ENSEMBL   UCSC 
 
  377
 
  6
 
 26209
 
 island
 
 0.016897
 
 0.2302050
 
 Not queried
 
  2.9329471
 
 3.10e-01
 
 0.4672865596
  
 
  
 16
 
  85832091
 
  85832378
 
  PDF   STATS   ENSEMBL   UCSC 
 
  288
 
  5
 
 14881
 
 gene;tss;island
 
 0.048875
 
 0.0074871
 
 Not queried
 
  2.9283798
 
 3.10e-01
 
 0.4672865596
  
 
  
 14
 
 105616988
 
 105617308
 
  PDF   STATS   ENSEMBL   UCSC 
 
  321
 
  4
 
 12256
 
 gene;island
 
 0.408094
 
 0.6868628
 
 Not queried
 
  2.9240302
 
 3.43e-01
 
 0.4672865596
  
 
  
 1
 
 153651886
 
 153652247
 
  PDF   STATS   ENSEMBL   UCSC 
 
  362
 
  4
 
 765
 
 gene;island
 
 0.110120
 
 0.3605126
 
 Not queried
 
  2.9193405
 
 3.43e-01
 
 0.4672865596
  
 
  
 15
 
  60294120
 
  60294517
 
  PDF   STATS   ENSEMBL   UCSC 
 
  398
 
  4
 
 14188
 
 island
 
 0.061486
 
 0.3139806
 
 Not queried
 
  2.9144554
 
 3.43e-01
 
 0.4672865596
  
 
  
 3
 
  64211155
 
  64211659
 
  PDF   STATS   ENSEMBL   UCSC 
 
  505
 
  5
 
 28433
 
 tss
 
 0.510164
 
 0.5489353
 
 Not queried
 
  2.9119567
 
 3.10e-01
 
 0.4672865596
  
 
  
 7
 
  95063967
 
  95064225
 
  PDF   STATS   ENSEMBL   UCSC 
 
  259
 
  4
 
 37097
 
 gene
 
 0.015171
 
 0.1734971
 
 Not queried
 
  2.8976193
 
 3.43e-01
 
 0.4672865596
  
 
  
 5
 
  63985282
 
  63985489
 
  PDF   STATS   ENSEMBL   UCSC 
 
  208
 
  4
 
 32090
 
 tss;island
 
 0.144657
 
 0.4276363
 
 Not queried
 
  2.8893270
 
 3.43e-01
 
 0.4672865596
  
 
  
 2
 
   1879820
 
   1880131
 
  PDF   STATS   ENSEMBL   UCSC 
 
  312
 
  4
 
 22539
 
 gene
 
 0.415716
 
 0.7664347
 
 Not queried
 
  2.8861660
 
 3.43e-01
 
 0.4672865596
  
 
  
 19
 
  16394366
 
  16394774
 
  PDF   STATS   ENSEMBL   UCSC 
 
  409
 
  5
 
 21716
 
 island
 
 0.048301
 
 0.3150751
 
 Not queried
 
  2.8820350
 
 3.10e-01
 
 0.4672865596
  
 
  
 1
 
  24645802
 
  24645853
 
  PDF   STATS   ENSEMBL   UCSC 
 
   52
 
  4
 
 1358
 
 tss
 
 0.009698
 
 0.1234896
 
 Not queried
 
  2.8692084
 
 3.43e-01
 
 0.4672865596
  
 
  
 6
 
 100442105
 
 100442151
 
  PDF   STATS   ENSEMBL   UCSC 
 
   47
 
  4
 
 35138
 
 tss
 
 0.350790
 
 0.0420067
 
 Not queried
 
  2.8674322
 
 3.43e-01
 
 0.4672865596
  
 
  
 7
 
  32110988
 
  32111453
 
  PDF   STATS   ENSEMBL   UCSC 
 
  466
 
  8
 
 38393
 
 island
 
 0.080096
 
 0.4801985
 
 Not queried
 
  2.8649195
 
 3.28e-01
 
 0.4672865596
  
 
  
 5
 
 123987423
 
 123987747
 
  PDF   STATS   ENSEMBL   UCSC 
 
  325
 
  5
 
 31733
 
 gene;island
 
 0.006901
 
 0.0099487
 
 Not queried
 
  2.8477793
 
 3.10e-01
 
 0.4672865596
  
 
  
 5
 
  68710808
 
  68710912
 
  PDF   STATS   ENSEMBL   UCSC 
 
  105
 
  6
 
 32110
 
 tss;island
 
 0.022447
 
 0.1705138
 
 Not queried
 
  2.8468156
 
 3.10e-01
 
 0.4672865596
  
 
  
 1
 
  86622395
 
  86622737
 
  PDF   STATS   ENSEMBL   UCSC 
 
  343
 
  6
 
 1725
 
 tss;island
 
 0.007640
 
 0.0263222
 
 Not queried
 
  2.8354055
 
 3.10e-01
 
 0.4672865596
  
 
  
 5
 
  76925380
 
  76925623
 
  PDF   STATS   ENSEMBL   UCSC 
 
  244
 
  4
 
 31655
 
 gene;island
 
 0.185293
 
 0.0471344
 
 Not queried
 
  2.8328504
 
 3.43e-01
 
 0.4672865596
  
 
  
 11
 
 116371188
 
 116371487
 
  PDF   STATS   ENSEMBL   UCSC 
 
  300
 
  4
 
 8832
 
 island
 
 0.045321
 
 0.2673187
 
 Not queried
 
  2.8309667
 
 3.43e-01
 
 0.4672865596
  
 
  
 6
 
 168426063
 
 168426413
 
  PDF   STATS   ENSEMBL   UCSC 
 
  351
 
  5
 
 34557
 
 gene
 
 0.127598
 
 0.6312924
 
 Not queried
 
  2.8292651
 
 3.10e-01
 
 0.4672865596
  
 
  
 17
 
  46871582
 
  46871905
 
  PDF   STATS   ENSEMBL   UCSC 
 
  324
 
  6
 
 16667
 
 gene
 
 0.493072
 
 0.6916913
 
 Not queried
 
  2.8288448
 
 3.10e-01
 
 0.4672865596
  
 
  
 1
 
 200860363
 
 200860682
 
  PDF   STATS   ENSEMBL   UCSC 
 
  320
 
  4
 
 4069
 
 island
 
 0.005878
 
 0.1128146
 
 Not queried
 
  2.8240989
 
 3.43e-01
 
 0.4672865596
  
 
  
 17
 
  79225573
 
  79225974
 
  PDF   STATS   ENSEMBL   UCSC 
 
  402
 
  4
 
 16902
 
 gene
 
 0.385128
 
 0.0801736
 
 Not queried
 
  2.7984086
 
 3.43e-01
 
 0.4672865596
  
 
  
 8
 
  18541446
 
  18541627
 
  PDF   STATS   ENSEMBL   UCSC 
 
  182
 
  5
 
 39219
 
 gene
 
 0.046108
 
 0.2514736
 
 Not queried
 
  2.7973377
 
 3.10e-01
 
 0.4672865596
  
 
  
 7
 
 121950924
 
 121951055
 
  PDF   STATS   ENSEMBL   UCSC 
 
  132
 
  4
 
 38872
 
 island
 
 0.094822
 
 0.4335543
 
 Not queried
 
  2.7885313
 
 3.43e-01
 
 0.4672865596
  
 
  
 4
 
  41694736
 
  41694847
 
  PDF   STATS   ENSEMBL   UCSC 
 
  112
 
  4
 
 29992
 
 gene
 
 0.661637
 
 0.9405007
 
 Not queried
 
  2.7872756
 
 3.43e-01
 
 0.4672865596
  
 
  
 6
 
 134213992
 
 134214307
 
  PDF   STATS   ENSEMBL   UCSC 
 
  316
 
  4
 
 34480
 
 gene;island
 
 0.058514
 
 0.4697679
 
 Not queried
 
  2.7863193
 
 3.43e-01
 
 0.4672865596
  
 
  
 7
 
  88424985
 
  88425148
 
  PDF   STATS   ENSEMBL   UCSC 
 
  164
 
  6
 
 37079
 
 gene
 
 0.571694
 
 0.8756798
 
 Not queried
 
  2.7705750
 
 3.10e-01
 
 0.4672865596
  
 
  
 12
 
 131303016
 
 131303247
 
  PDF   STATS   ENSEMBL   UCSC 
 
  232
 
  4
 
 9418
 
 gene;island
 
 0.647196
 
 0.3889138
 
 Not queried
 
  2.7692374
 
 3.43e-01
 
 0.4672865596
  
 
  
 5
 
  57755923
 
  57756026
 
  PDF   STATS   ENSEMBL   UCSC 
 
  104
 
  4
 
 32076
 
 tss
 
 0.004790
 
 0.0113263
 
 Not queried
 
  2.7507677
 
 3.43e-01
 
 0.4672865596
  
 
  
 10
 
  27541213
 
  27541418
 
  PDF   STATS   ENSEMBL   UCSC 
 
  206
 
  5
 
 5627
 
 island
 
 0.982419
 
 0.5989667
 
 Not queried
 
  2.7421103
 
 3.10e-01
 
 0.4672865596
  
 
  
 13
 
  43148017
 
  43148436
 
  PDF   STATS   ENSEMBL   UCSC 
 
  420
 
  5
 
 11146
 
 gene;island
 
 0.022899
 
 0.1409463
 
 Not queried
 
  2.7399223
 
 3.10e-01
 
 0.4672865596
  
 
  
 17
 
  79920395
 
  79920755
 
  PDF   STATS   ENSEMBL   UCSC 
 
  361
 
  5
 
 18962
 
 island
 
 0.602677
 
 0.9091583
 
 Not queried
 
  2.7305513
 
 3.10e-01
 
 0.4672865596
  
 
  
 14
 
 104928289
 
 104928675
 
  PDF   STATS   ENSEMBL   UCSC 
 
  387
 
  4
 
 13205
 
 island
 
 0.176725
 
 0.0204336
 
 Not queried
 
  2.7297512
 
 3.43e-01
 
 0.4672865596
  
 
  
 2
 
 239228826
 
 239229182
 
  PDF   STATS   ENSEMBL   UCSC 
 
  357
 
  4
 
 23974
 
 tss
 
 0.004390
 
 0.0552233
 
 Not queried
 
  2.7294343
 
 3.43e-01
 
 0.4672865596
  
 
  
 19
 
  18902117
 
  18902221
 
  PDF   STATS   ENSEMBL   UCSC 
 
  105
 
  4
 
 20628
 
 tss
 
 0.105101
 
 0.3071746
 
 Not queried
 
  2.7176905
 
 3.43e-01
 
 0.4672865596
  
 
  
 2
 
 176961552
 
 176961907
 
  PDF   STATS   ENSEMBL   UCSC 
 
  356
 
  4
 
 24892
 
 island
 
 0.003977
 
 0.0278223
 
 Not queried
 
  2.7101570
 
 3.43e-01
 
 0.4672865596
  
 
  
 11
 
 130029961
 
 130030459
 
  PDF   STATS   ENSEMBL   UCSC 
 
  499
 
  4
 
 6976
 
 gene;island
 
 0.015465
 
 0.1246543
 
 Not queried
 
  2.7098630
 
 3.43e-01
 
 0.4672865596
  
 
  
 7
 
 136553088
 
 136553327
 
  PDF   STATS   ENSEMBL   UCSC 
 
  240
 
  7
 
 37922
 
 tss;island
 
 0.004973
 
 0.0208930
 
 Not queried
 
  2.7058287
 
 3.18e-01
 
 0.4672865596
  
 
  
 1
 
 205819406
 
 205819609
 
  PDF   STATS   ENSEMBL   UCSC 
 
  204
 
  4
 
 2210
 
 tss
 
 0.406962
 
 0.0321711
 
 Not queried
 
  2.7005414
 
 3.43e-01
 
 0.4672865596
  
 
  
 11
 
 123430575
 
 123430949
 
  PDF   STATS   ENSEMBL   UCSC 
 
  375
 
  4
 
 6950
 
 gene
 
 0.016057
 
 0.2595990
 
 Not queried
 
  2.6977706
 
 3.43e-01
 
 0.4672865596
  
 
  
 16
 
  55794456
 
  55794910
 
  PDF   STATS   ENSEMBL   UCSC 
 
  455
 
  5
 
 15938
 
 island
 
 0.302379
 
 0.2139369
 
 Not queried
 
  2.6976466
 
 3.10e-01
 
 0.4672865596
  
 
  
 17
 
  46114615
 
  46114933
 
  PDF   STATS   ENSEMBL   UCSC 
 
  319
 
  6
 
 17450
 
 tss
 
 0.027797
 
 0.0824910
 
 Not queried
 
  2.6969019
 
 3.10e-01
 
 0.4672865596
  
 
  
 12
 
  70132987
 
  70133067
 
  PDF   STATS   ENSEMBL   UCSC 
 
   81
 
  5
 
 9213
 
 gene;tss;island
 
 0.006055
 
 0.0037489
 
 Not queried
 
  2.6953407
 
 3.10e-01
 
 0.4672865596
  
 
  
 17
 
  35289351
 
  35289875
 
  PDF   STATS   ENSEMBL   UCSC 
 
  525
 
  5
 
 18234
 
 island
 
 0.034741
 
 0.1103071
 
 Not queried
 
  2.6921103
 
 3.10e-01
 
 0.4672865596
  
 
  
 4
 
  21305805
 
  21306051
 
  PDF   STATS   ENSEMBL   UCSC 
 
  247
 
  4
 
 29965
 
 gene;tss
 
 0.079444
 
 0.4025088
 
 Not queried
 
  2.6785436
 
 3.43e-01
 
 0.4672865596
  
 
  
 6
 
  32005786
 
  32006079
 
  PDF   STATS   ENSEMBL   UCSC 
 
  294
 
  4
 
 34860
 
 tss
 
 0.080074
 
 0.4246125
 
 Not queried
 
  2.6776938
 
 3.43e-01
 
 0.4672865596
  
 
  
 19
 
  49016564
 
  49016934
 
  PDF   STATS   ENSEMBL   UCSC 
 
  371
 
  6
 
 20917
 
 tss;island
 
 0.214004
 
 0.0141683
 
 Not queried
 
  2.6767313
 
 3.10e-01
 
 0.4672865596
  
 
  
 15
 
  96887824
 
  96888024
 
  PDF   STATS   ENSEMBL   UCSC 
 
  201
 
  5
 
 14489
 
 island
 
 0.019642
 
 0.0482741
 
 Not queried
 
  2.6589893
 
 3.10e-01
 
 0.4672865596
  
 
  
 6
 
  37616410
 
  37616803
 
  PDF   STATS   ENSEMBL   UCSC 
 
  394
 
  5
 
 34323
 
 gene;island
 
 0.178338
 
 0.0757104
 
 Not queried
 
  2.6546127
 
 3.10e-01
 
 0.4672865596
  
 
  
 6
 
  28481660
 
  28482191
 
  PDF   STATS   ENSEMBL   UCSC 
 
  532
 
  6
 
 33634
 
 gene
 
 0.117375
 
 0.4553744
 
 Not queried
 
  2.6525525
 
 3.10e-01
 
 0.4672865596
  
 
  
 22
 
  36877697
 
  36877737
 
  PDF   STATS   ENSEMBL   UCSC 
 
   41
 
  4
 
 27108
 
 tss
 
 0.000899
 
 0.0047143
 
 Not queried
 
  2.6435448
 
 3.43e-01
 
 0.4672865596
  
 
  
 5
 
 154070144
 
 154070385
 
  PDF   STATS   ENSEMBL   UCSC 
 
  242
 
  4
 
 33335
 
 island
 
 0.976051
 
 0.7699479
 
 Not queried
 
  2.6424260
 
 3.43e-01
 
 0.4672865596
  
 
  
 4
 
 187646301
 
 187646446
 
  PDF   STATS   ENSEMBL   UCSC 
 
  146
 
  4
 
 30709
 
 tss;island
 
 0.085068
 
 0.0107697
 
 Not queried
 
  2.6414102
 
 3.43e-01
 
 0.4672865596
  
 
  
 3
 
  52828583
 
  52828680
 
  PDF   STATS   ENSEMBL   UCSC 
 
   98
 
  4
 
 28393
 
 tss
 
 0.377794
 
 0.6396259
 
 Not queried
 
  2.6395291
 
 3.43e-01
 
 0.4672865596
  
 
  
 6
 
  32549139
 
  32549631
 
  PDF   STATS   ENSEMBL   UCSC 
 
  493
 
  5
 
 34121
 
 gene;island
 
 0.649069
 
 0.9371347
 
 Not queried
 
  2.6167457
 
 3.10e-01
 
 0.4672865596
  
 
  
 11
 
   2193041
 
   2193293
 
  PDF   STATS   ENSEMBL   UCSC 
 
  253
 
  4
 
 7063
 
 tss
 
 0.220433
 
 0.6300158
 
 Not queried
 
  2.6159063
 
 3.43e-01
 
 0.4672865596
  
 
  
 19
 
  37825307
 
  37825446
 
  PDF   STATS   ENSEMBL   UCSC 
 
  140
 
  6
 
 20736
 
 tss;island
 
 0.415411
 
 0.2107143
 
 Not queried
 
  2.6118824
 
 3.10e-01
 
 0.4672865596
  
 
  
 3
 
 121264538
 
 121264849
 
  PDF   STATS   ENSEMBL   UCSC 
 
  312
 
  5
 
 27912
 
 gene
 
 0.004760
 
 0.0106002
 
 Not queried
 
  2.6076036
 
 3.10e-01
 
 0.4672865596
  
 
  
 10
 
   1517083
 
   1517340
 
  PDF   STATS   ENSEMBL   UCSC 
 
  258
 
  5
 
 4467
 
 gene;island
 
 0.139948
 
 0.3999584
 
 Not queried
 
  2.5958246
 
 3.10e-01
 
 0.4672865596
  
 
  
 5
 
  36606102
 
  36606446
 
  PDF   STATS   ENSEMBL   UCSC 
 
  345
 
  5
 
 32019
 
 tss
 
 0.015053
 
 0.0034310
 
 Not queried
 
  2.5918510
 
 3.10e-01
 
 0.4672865596
  
 
  
 11
 
  74442547
 
  74442900
 
  PDF   STATS   ENSEMBL   UCSC 
 
  354
 
  4
 
 7561
 
 tss;island
 
 0.029475
 
 0.1355857
 
 Not queried
 
  2.5860231
 
 3.43e-01
 
 0.4672865596
  
 
  
 3
 
  46933782
 
  46933972
 
  PDF   STATS   ENSEMBL   UCSC 
 
  191
 
  4
 
 27757
 
 gene;island
 
 0.024316
 
 0.0453671
 
 Not queried
 
  2.5804511
 
 3.43e-01
 
 0.4672865596
  
 
  
 14
 
 105104840
 
 105105198
 
  PDF   STATS   ENSEMBL   UCSC 
 
  359
 
  4
 
 13209
 
 island
 
 0.369464
 
 0.7762412
 
 Not queried
 
  2.5798634
 
 3.43e-01
 
 0.4672865596
  
 
  
 15
 
  68724690
 
  68725002
 
  PDF   STATS   ENSEMBL   UCSC 
 
  313
 
  4
 
 13759
 
 tss;island
 
 0.056614
 
 0.2550204
 
 Not queried
 
  2.5713514
 
 3.43e-01
 
 0.4672865596
  
 
  
 15
 
  68851385
 
  68851827
 
  PDF   STATS   ENSEMBL   UCSC 
 
  443
 
  5
 
 14259
 
 island
 
 0.007837
 
 0.0063835
 
 Not queried
 
  2.5708295
 
 3.10e-01
 
 0.4672865596
  
 
  
 2
 
 175205716
 
 175205852
 
  PDF   STATS   ENSEMBL   UCSC 
 
  137
 
  4
 
 24875
 
 island
 
 0.031393
 
 0.1844833
 
 Not queried
 
  2.5663269
 
 3.43e-01
 
 0.4672865596
  
 
  
 5
 
 171057072
 
 171057339
 
  PDF   STATS   ENSEMBL   UCSC 
 
  268
 
  5
 
 33383
 
 island
 
 0.314930
 
 0.1177605
 
 Not queried
 
  2.5607025
 
 3.10e-01
 
 0.4672865596
  
 
  
 16
 
  85878940
 
  85879145
 
  PDF   STATS   ENSEMBL   UCSC 
 
  206
 
  4
 
 16166
 
 island
 
 0.650906
 
 0.8498712
 
 Not queried
 
  2.5500879
 
 3.43e-01
 
 0.4672865596
  
 
  
 15
 
  75639163
 
  75639433
 
  PDF   STATS   ENSEMBL   UCSC 
 
  271
 
  4
 
 14328
 
 island
 
 0.079444
 
 0.4186265
 
 Not queried
 
  2.5474593
 
 3.43e-01
 
 0.4672865596
  
 
  
 5
 
 158689508
 
 158689727
 
  PDF   STATS   ENSEMBL   UCSC 
 
  220
 
  4
 
 32461
 
 tss;island
 
 0.169879
 
 0.6508837
 
 Not queried
 
  2.5466271
 
 3.43e-01
 
 0.4672865596
  
 
  
 11
 
 120973085
 
 120973274
 
  PDF   STATS   ENSEMBL   UCSC 
 
  190
 
  4
 
 7766
 
 tss
 
 0.368009
 
 0.7408096
 
 Not queried
 
  2.5457464
 
 3.43e-01
 
 0.4672865596
  
 
  
 12
 
   1725788
 
   1726076
 
  PDF   STATS   ENSEMBL   UCSC 
 
  289
 
  6
 
 9485
 
 tss
 
 0.112196
 
 0.6017566
 
 Not queried
 
  2.5379237
 
 3.10e-01
 
 0.4672865596
  
 
  
 1
 
 155265026
 
 155265378
 
  PDF   STATS   ENSEMBL   UCSC 
 
  353
 
  4
 
 800
 
 gene;island
 
 0.491717
 
 0.1030736
 
 Not queried
 
  2.5378208
 
 3.43e-01
 
 0.4672865596
  
 
  
 14
 
 103388656
 
 103388859
 
  PDF   STATS   ENSEMBL   UCSC 
 
  204
 
  4
 
 12647
 
 tss
 
 0.725962
 
 0.9010361
 
 Not queried
 
  2.5331712
 
 3.43e-01
 
 0.4672865596
  
 
  
 2
 
 105484357
 
 105484823
 
  PDF   STATS   ENSEMBL   UCSC 
 
  467
 
  4
 
 24580
 
 island
 
 0.488615
 
 0.1408323
 
 Not queried
 
  2.5255420
 
 3.43e-01
 
 0.4672865596
  
 
  
 1
 
  40204285
 
  40204479
 
  PDF   STATS   ENSEMBL   UCSC 
 
  195
 
  4
 
 1528
 
 tss
 
 0.012815
 
 0.0552693
 
 Not queried
 
  2.5239137
 
 3.43e-01
 
 0.4672865596
  
 
  
 15
 
  88798331
 
  88798666
 
  PDF   STATS   ENSEMBL   UCSC 
 
  336
 
  4
 
 13464
 
 gene;island
 
 0.015429
 
 0.2349007
 
 Not queried
 
  2.5218466
 
 3.43e-01
 
 0.4672865596
  
 
  
 11
 
 134176598
 
 134176888
 
  PDF   STATS   ENSEMBL   UCSC 
 
  291
 
  5
 
 6996
 
 gene
 
 0.179390
 
 0.6400339
 
 Not queried
 
  2.5193545
 
 3.10e-01
 
 0.4672865596
  
 
  
 22
 
  37252282
 
  37252652
 
  PDF   STATS   ENSEMBL   UCSC 
 
  371
 
  4
 
 27460
 
 island
 
 0.012910
 
 0.0412111
 
 Not queried
 
  2.5112412
 
 3.43e-01
 
 0.4672865596
  
 
  
 1
 
 207669922
 
 207670205
 
  PDF   STATS   ENSEMBL   UCSC 
 
  284
 
  4
 
 978
 
 gene;island
 
 0.151272
 
 0.0244846
 
 Not queried
 
  2.4982230
 
 3.43e-01
 
 0.4672865596
  
 
  
 6
 
 167136206
 
 167136432
 
  PDF   STATS   ENSEMBL   UCSC 
 
  227
 
  4
 
 34554
 
 gene
 
 0.220989
 
 0.6049366
 
 Not queried
 
  2.4969403
 
 3.43e-01
 
 0.4672865596
  
 
  
 4
 
   1407592
 
   1407989
 
  PDF   STATS   ENSEMBL   UCSC 
 
  398
 
  4
 
 30786
 
 island
 
 0.413352
 
 0.6378061
 
 Not queried
 
  2.4949821
 
 3.43e-01
 
 0.4672865596
  
 
  
 22
 
  51221675
 
  51221773
 
  PDF   STATS   ENSEMBL   UCSC 
 
   99
 
  4
 
 26935
 
 gene;tss;island
 
 0.167406
 
 0.0157013
 
 Not queried
 
  2.4915316
 
 3.43e-01
 
 0.4672865596
  
 
  
 2
 
 112917127
 
 112917424
 
  PDF   STATS   ENSEMBL   UCSC 
 
  298
 
  4
 
 22827
 
 gene;island
 
 0.477260
 
 0.6303392
 
 Not queried
 
  2.4894351
 
 3.43e-01
 
 0.4672865596
  
 
  
 2
 
 157291759
 
 157292127
 
  PDF   STATS   ENSEMBL   UCSC 
 
  369
 
  6
 
 23674
 
 tss;island
 
 0.090812
 
 0.6223004
 
 Not queried
 
  2.4819206
 
 3.10e-01
 
 0.4672865596
  
 
  
 2
 
  31806042
 
  31806551
 
  PDF   STATS   ENSEMBL   UCSC 
 
  510
 
  6
 
 23289
 
 tss
 
 0.779428
 
 0.4528895
 
 Not queried
 
  2.4644304
 
 3.10e-01
 
 0.4672865596
  
 
  
 6
 
  10384975
 
  10385489
 
  PDF   STATS   ENSEMBL   UCSC 
 
  515
 
  6
 
 35423
 
 island
 
 0.209734
 
 0.0222228
 
 Not queried
 
  2.4621930
 
 3.10e-01
 
 0.4672865596
  
 
  
 11
 
 122852284
 
 122852698
 
  PDF   STATS   ENSEMBL   UCSC 
 
  415
 
  9
 
 8904
 
 island
 
 0.049781
 
 0.2107363
 
 Not queried
 
  2.4614740
 
 2.97e-01
 
 0.4672865596
  
 
  
 1
 
 117753591
 
 117753741
 
  PDF   STATS   ENSEMBL   UCSC 
 
  151
 
  4
 
 1870
 
 tss
 
 0.211275
 
 0.5609836
 
 Not queried
 
  2.4589366
 
 3.43e-01
 
 0.4672865596
  
 
  
 5
 
 134363324
 
 134363973
 
  PDF   STATS   ENSEMBL   UCSC 
 
  650
 
  7
 
 33129
 
 island
 
 0.250561
 
 0.0843992
 
 Not queried
 
  2.4442774
 
 3.18e-01
 
 0.4672865596
  
 
  
 10
 
 124894943
 
 124895500
 
  PDF   STATS   ENSEMBL   UCSC 
 
  558
 
  9
 
 5415
 
 tss
 
 0.108226
 
 0.0219478
 
 Not queried
 
  2.4423127
 
 2.97e-01
 
 0.4672865596
  
 
  
 19
 
  33764284
 
  33764550
 
  PDF   STATS   ENSEMBL   UCSC 
 
  267
 
  4
 
 21873
 
 island
 
 0.281618
 
 0.6851568
 
 Not queried
 
  2.4421883
 
 3.43e-01
 
 0.4672865596
  
 
  
 6
 
   8102871
 
   8103092
 
  PDF   STATS   ENSEMBL   UCSC 
 
  222
 
  8
 
 34619
 
 tss
 
 0.029157
 
 0.0058591
 
 Not queried
 
  2.4419240
 
 3.28e-01
 
 0.4672865596
  
 
  
 8
 
  19796299
 
  19796591
 
  PDF   STATS   ENSEMBL   UCSC 
 
  293
 
  6
 
 40114
 
 island
 
 0.179155
 
 0.0274353
 
 Not queried
 
  2.4381577
 
 3.10e-01
 
 0.4672865596
  
 
  
 3
 
 124931535
 
 124931746
 
  PDF   STATS   ENSEMBL   UCSC 
 
  212
 
  5
 
 29419
 
 island
 
 0.292674
 
 0.0510194
 
 Not queried
 
  2.4285428
 
 3.10e-01
 
 0.4672865596
  
 
  
 7
 
  27153636
 
  27153944
 
  PDF   STATS   ENSEMBL   UCSC 
 
  309
 
  5
 
 37489
 
 tss
 
 0.678560
 
 0.9649139
 
 Not queried
 
  2.4190262
 
 3.10e-01
 
 0.4672865596
  
 
  
 5
 
 148928885
 
 148929784
 
  PDF   STATS   ENSEMBL   UCSC 
 
  900
 
  8
 
 31858
 
 gene;island
 
 0.085766
 
 0.0040202
 
 Not queried
 
  2.4071296
 
 2.79e-01
 
 0.4672865596
  
 
  
 3
 
 147131069
 
 147131332
 
  PDF   STATS   ENSEMBL   UCSC 
 
  264
 
  4
 
 27990
 
 gene;island
 
 0.161157
 
 0.0274946
 
 Not queried
 
  2.4036884
 
 3.43e-01
 
 0.4672865596
  
 
  
 11
 
 118478236
 
 118478329
 
  PDF   STATS   ENSEMBL   UCSC 
 
   94
 
  4
 
 6931
 
 gene;island
 
 0.042988
 
 0.2391585
 
 Not queried
 
  2.4035751
 
 3.43e-01
 
 0.4672865596
  
 
  
 12
 
  98850691
 
  98851021
 
  PDF   STATS   ENSEMBL   UCSC 
 
  331
 
  6
 
 10703
 
 island
 
 0.015007
 
 0.1627761
 
 Not queried
 
  2.4030522
 
 3.10e-01
 
 0.4672865596
  
 
  
 6
 
 166908379
 
 166908811
 
  PDF   STATS   ENSEMBL   UCSC 
 
  433
 
  5
 
 34548
 
 gene
 
 0.346421
 
 0.8092010
 
 Not queried
 
  2.3955765
 
 3.10e-01
 
 0.4672865596
  
 
  
 6
 
 133563764
 
 133564066
 
  PDF   STATS   ENSEMBL   UCSC 
 
  303
 
  4
 
 34477
 
 gene;island
 
 0.046992
 
 0.1380449
 
 Not queried
 
  2.3948408
 
 3.43e-01
 
 0.4672865596
  
 
  
 3
 
  52091484
 
  52091829
 
  PDF   STATS   ENSEMBL   UCSC 
 
  346
 
  4
 
 28371
 
 tss;island
 
 0.022669
 
 0.0156699
 
 Not queried
 
  2.3830501
 
 3.43e-01
 
 0.4672865596
  
 
  
 3
 
 195384522
 
 195384752
 
  PDF   STATS   ENSEMBL   UCSC 
 
  231
 
  6
 
 28805
 
 tss;island
 
 0.147007
 
 0.0122928
 
 Not queried
 
  2.3798944
 
 3.10e-01
 
 0.4672865596
  
 
  
 8
 
 104512858
 
 104513186
 
  PDF   STATS   ENSEMBL   UCSC 
 
  329
 
  4
 
 40521
 
 island
 
 0.032200
 
 0.0153260
 
 Not queried
 
  2.3770631
 
 3.43e-01
 
 0.4672865596
  
 
  
 2
 
 234776807
 
 234777085
 
  PDF   STATS   ENSEMBL   UCSC 
 
  279
 
  5
 
 25212
 
 island
 
 0.013930
 
 0.0526091
 
 Not queried
 
  2.3753092
 
 3.10e-01
 
 0.4672865596
  
 
  
 16
 
   1968576
 
   1968709
 
  PDF   STATS   ENSEMBL   UCSC 
 
  134
 
  4
 
 15009
 
 tss;island
 
 0.097408
 
 0.4936292
 
 Not queried
 
  2.3743421
 
 3.43e-01
 
 0.4672865596
  
 
  
 18
 
  44337664
 
  44338147
 
  PDF   STATS   ENSEMBL   UCSC 
 
  484
 
  6
 
 19259
 
 tss;island
 
 0.029478
 
 0.0645835
 
 Not queried
 
  2.3651277
 
 3.10e-01
 
 0.4672865596
  
 
  
 11
 
  61322742
 
  61323413
 
  PDF   STATS   ENSEMBL   UCSC 
 
  672
 
  6
 
 6630
 
 gene;island
 
 0.063580
 
 0.3341927
 
 Not queried
 
  2.3638952
 
 3.10e-01
 
 0.4672865596
  
 
  
 8
 
  20054372
 
  20054699
 
  PDF   STATS   ENSEMBL   UCSC 
 
  328
 
  8
 
 39575
 
 tss
 
 0.046857
 
 0.0176910
 
 Not queried
 
  2.3637396
 
 3.28e-01
 
 0.4672865596
  
 
  
 16
 
    420490
 
    420755
 
  PDF   STATS   ENSEMBL   UCSC 
 
  266
 
  4
 
 15453
 
 island
 
 0.000211
 
 0.0074683
 
 Not queried
 
  2.3435527
 
 3.43e-01
 
 0.4672865596
  
 
  
 12
 
  30948636
 
  30948794
 
  PDF   STATS   ENSEMBL   UCSC 
 
  159
 
  5
 
 10308
 
 island
 
 0.019159
 
 0.0896267
 
 Not queried
 
  2.3386042
 
 3.10e-01
 
 0.4672865596
  
 
  
 11
 
  64058550
 
  64059057
 
  PDF   STATS   ENSEMBL   UCSC 
 
  508
 
  6
 
 8401
 
 island
 
 0.125574
 
 0.2654192
 
 Not queried
 
  2.3379310
 
 3.10e-01
 
 0.4672865596
  
 
  
 1
 
 156182601
 
 156182774
 
  PDF   STATS   ENSEMBL   UCSC 
 
  174
 
  4
 
 2010
 
 tss
 
 0.001203
 
 0.0076596
 
 Not queried
 
  2.3254034
 
 3.43e-01
 
 0.4672865596
  
 
  
 5
 
   1876168
 
   1876397
 
  PDF   STATS   ENSEMBL   UCSC 
 
  230
 
  5
 
 32643
 
 island
 
 0.041730
 
 0.2670145
 
 Not queried
 
  2.3218197
 
 3.10e-01
 
 0.4672865596
  
 
  
 1
 
  60280716
 
  60280868
 
  PDF   STATS   ENSEMBL   UCSC 
 
  153
 
  4
 
 559
 
 gene;island
 
 0.024878
 
 0.1522178
 
 Not queried
 
  2.3152551
 
 3.43e-01
 
 0.4672865596
  
 
  
 3
 
 129694392
 
 129694683
 
  PDF   STATS   ENSEMBL   UCSC 
 
  292
 
  4
 
 27945
 
 gene;island
 
 0.061146
 
 0.1730350
 
 Not queried
 
  2.3121917
 
 3.43e-01
 
 0.4672865596
  
 
  
 8
 
  20054372
 
  20054703
 
  PDF   STATS   ENSEMBL   UCSC 
 
  332
 
  9
 
 40115
 
 island
 
 0.042902
 
 0.0076583
 
 Not queried
 
  2.3083527
 
 2.97e-01
 
 0.4672865596
  
 
  
 19
 
  41531634
 
  41531976
 
  PDF   STATS   ENSEMBL   UCSC 
 
  343
 
  4
 
 22044
 
 island
 
 0.249320
 
 0.4956716
 
 Not queried
 
  2.3059822
 
 3.43e-01
 
 0.4672865596
  
 
  
 22
 
  30938383
 
  30938784
 
  PDF   STATS   ENSEMBL   UCSC 
 
  402
 
  4
 
 27414
 
 island
 
 0.086682
 
 0.3833000
 
 Not queried
 
  2.2996051
 
 3.43e-01
 
 0.4672865596
  
 
  
 10
 
 133956824
 
 133957095
 
  PDF   STATS   ENSEMBL   UCSC 
 
  272
 
  4
 
 4856
 
 gene;island
 
 0.081871
 
 0.3600594
 
 Not queried
 
  2.2965902
 
 3.43e-01
 
 0.4672865596
  
 
  
 1
 
 155035008
 
 155035530
 
  PDF   STATS   ENSEMBL   UCSC 
 
  523
 
  5
 
 1978
 
 tss;island
 
 0.024216
 
 0.0451894
 
 Not queried
 
  2.2947646
 
 3.10e-01
 
 0.4672865596
  
 
  
 12
 
 132637734
 
 132638107
 
  PDF   STATS   ENSEMBL   UCSC 
 
  374
 
  4
 
 11025
 
 island
 
 0.553749
 
 0.7826907
 
 Not queried
 
  2.2916037
 
 3.43e-01
 
 0.4672865596
  
 
  
 4
 
  37961862
 
  37962205
 
  PDF   STATS   ENSEMBL   UCSC 
 
  344
 
  4
 
 29975
 
 gene
 
 0.829249
 
 0.9779467
 
 Not queried
 
  2.2909591
 
 3.43e-01
 
 0.4672865596
  
 
  
 10
 
  17271006
 
  17271303
 
  PDF   STATS   ENSEMBL   UCSC 
 
  298
 
  5
 
 4518
 
 gene;island
 
 0.001654
 
 0.0007974
 
 Not queried
 
  2.2893006
 
 3.10e-01
 
 0.4672865596
  
 
  
 4
 
   7755730
 
   7755806
 
  PDF   STATS   ENSEMBL   UCSC 
 
   77
 
  4
 
 30293
 
 tss
 
 0.073800
 
 0.2668002
 
 Not queried
 
  2.2859323
 
 3.43e-01
 
 0.4672865596
  
 
  
 11
 
 132948042
 
 132948244
 
  PDF   STATS   ENSEMBL   UCSC 
 
  203
 
  4
 
 6984
 
 gene;island
 
 0.275655
 
 0.7923465
 
 Not queried
 
  2.2852386
 
 3.43e-01
 
 0.4672865596
  
 
  
 1
 
  41889271
 
  41889625
 
  PDF   STATS   ENSEMBL   UCSC 
 
  355
 
  4
 
 3139
 
 island
 
 0.494341
 
 0.8918063
 
 Not queried
 
  2.2837237
 
 3.43e-01
 
 0.4672865596
  
 
  
 6
 
 144607911
 
 144608270
 
  PDF   STATS   ENSEMBL   UCSC 
 
  360
 
  4
 
 36537
 
 island
 
 0.016330
 
 0.0639548
 
 Not queried
 
  2.2836511
 
 3.43e-01
 
 0.4672865596
  
 
  
 13
 
 112692119
 
 112692382
 
  PDF   STATS   ENSEMBL   UCSC 
 
  264
 
  4
 
 11907
 
 island
 
 0.177470
 
 0.5072312
 
 Not queried
 
  2.2797789
 
 3.43e-01
 
 0.4672865596
  
 
  
 6
 
   1384491
 
   1384780
 
  PDF   STATS   ENSEMBL   UCSC 
 
  290
 
  4
 
 35366
 
 island
 
 0.012896
 
 0.0898859
 
 Not queried
 
  2.2744146
 
 3.43e-01
 
 0.4672865596
  
 
  
 15
 
  74420511
 
  74420890
 
  PDF   STATS   ENSEMBL   UCSC 
 
  380
 
  4
 
 13414
 
 gene;tss;island
 
 0.216305
 
 0.0161838
 
 Not queried
 
  2.2736350
 
 3.43e-01
 
 0.4672865596
  
 
  
 11
 
  18477153
 
  18477753
 
  PDF   STATS   ENSEMBL   UCSC 
 
  601
 
  9
 
 8111
 
 island
 
 0.852075
 
 0.5435392
 
 Not queried
 
  2.2718823
 
 2.97e-01
 
 0.4672865596
  
 
  
 16
 
  49316845
 
  49317262
 
  PDF   STATS   ENSEMBL   UCSC 
 
  418
 
  5
 
 15907
 
 island
 
 0.031534
 
 0.0940601
 
 Not queried
 
  2.2702947
 
 3.10e-01
 
 0.4672865596
  
 
  
 6
 
 108488751
 
 108489064
 
  PDF   STATS   ENSEMBL   UCSC 
 
  314
 
  4
 
 34438
 
 gene;island
 
 0.046108
 
 0.3524716
 
 Not queried
 
  2.2668983
 
 3.43e-01
 
 0.4672865596
  
 
  
 1
 
  98515041
 
  98515272
 
  PDF   STATS   ENSEMBL   UCSC 
 
  232
 
  4
 
 3515
 
 island
 
 0.014148
 
 0.0064386
 
 Not queried
 
  2.2584053
 
 3.43e-01
 
 0.4672865596
  
 
  
 12
 
    568798
 
    569221
 
  PDF   STATS   ENSEMBL   UCSC 
 
  424
 
  5
 
 9479
 
 tss;island
 
 0.134860
 
 0.0519740
 
 Not queried
 
  2.2456234
 
 3.10e-01
 
 0.4672865596
  
 
  
 8
 
 144367072
 
 144367553
 
  PDF   STATS   ENSEMBL   UCSC 
 
  482
 
  4
 
 40651
 
 island
 
 0.512824
 
 0.8200622
 
 Not queried
 
  2.2420601
 
 3.43e-01
 
 0.4672865596
  
 
  
 20
 
  44098223
 
  44098409
 
  PDF   STATS   ENSEMBL   UCSC 
 
  187
 
  7
 
 26200
 
 island
 
 0.126234
 
 0.1518501
 
 Not queried
 
  2.2418844
 
 3.18e-01
 
 0.4672865596
  
 
  
 20
 
   2796729
 
   2796872
 
  PDF   STATS   ENSEMBL   UCSC 
 
  144
 
  4
 
 25552
 
 tss
 
 0.254124
 
 0.5918625
 
 Not queried
 
  2.2390483
 
 3.43e-01
 
 0.4672865596
  
 
  
 22
 
  29426511
 
  29426832
 
  PDF   STATS   ENSEMBL   UCSC 
 
  322
 
  4
 
 26829
 
 gene;island
 
 0.007202
 
 0.0621209
 
 Not queried
 
  2.2321780
 
 3.43e-01
 
 0.4672865596
  
 
  
 15
 
  80543353
 
  80543685
 
  PDF   STATS   ENSEMBL   UCSC 
 
  333
 
  5
 
 14374
 
 island
 
 0.216735
 
 0.6076228
 
 Not queried
 
  2.2308543
 
 3.10e-01
 
 0.4672865596
  
 
  
 19
 
  49259215
 
  49259452
 
  PDF   STATS   ENSEMBL   UCSC 
 
  238
 
  4
 
 20927
 
 tss;island
 
 0.023174
 
 0.2019228
 
 Not queried
 
  2.2308065
 
 3.43e-01
 
 0.4672865596
  
 
  
 4
 
  57410776
 
  57411219
 
  PDF   STATS   ENSEMBL   UCSC 
 
  444
 
  5
 
 31069
 
 island
 
 0.009018
 
 0.0422662
 
 Not queried
 
  2.2303749
 
 3.10e-01
 
 0.4672865596
  
 
  
 6
 
  31459698
 
  31460088
 
  PDF   STATS   ENSEMBL   UCSC 
 
  391
 
  6
 
 35773
 
 island
 
 0.027191
 
 0.0120101
 
 Not queried
 
  2.2301264
 
 3.10e-01
 
 0.4672865596
  
 
  
 6
 
 108444791
 
 108444920
 
  PDF   STATS   ENSEMBL   UCSC 
 
  130
 
  4
 
 36392
 
 island
 
 0.541542
 
 0.3118909
 
 Not queried
 
  2.2280861
 
 3.43e-01
 
 0.4672865596
  
 
  
 1
 
 113216774
 
 113217043
 
  PDF   STATS   ENSEMBL   UCSC 
 
  270
 
  5
 
 1837
 
 tss
 
 0.025774
 
 0.0117046
 
 Not queried
 
  2.2269693
 
 3.10e-01
 
 0.4672865596
  
 
  
 11
 
  58830191
 
  58830610
 
  PDF   STATS   ENSEMBL   UCSC 
 
  420
 
  4
 
 8300
 
 island
 
 0.795788
 
 0.5427661
 
 Not queried
 
  2.2263355
 
 3.43e-01
 
 0.4672865596
  
 
  
 6
 
 167031273
 
 167031411
 
  PDF   STATS   ENSEMBL   UCSC 
 
  139
 
  4
 
 34549
 
 gene;island
 
 0.848252
 
 0.9542949
 
 Not queried
 
  2.2249946
 
 3.43e-01
 
 0.4672865596
  
 
  
 1
 
 173639044
 
 173639135
 
  PDF   STATS   ENSEMBL   UCSC 
 
   92
 
  4
 
 2097
 
 tss;island
 
 0.464771
 
 0.1812384
 
 Not queried
 
  2.2245081
 
 3.43e-01
 
 0.4672865596
  
 
  
 11
 
 107730432
 
 107730551
 
  PDF   STATS   ENSEMBL   UCSC 
 
  120
 
  4
 
 7665
 
 tss;island
 
 0.004257
 
 0.0131558
 
 Not queried
 
  2.2183599
 
 3.43e-01
 
 0.4672865596
  
 
  
 6
 
  40345376
 
  40345751
 
  PDF   STATS   ENSEMBL   UCSC 
 
  376
 
  4
 
 34961
 
 tss
 
 0.415097
 
 0.6647332
 
 Not queried
 
  2.2164308
 
 3.43e-01
 
 0.4672865596
  
 
  
 10
 
 102890886
 
 102891080
 
  PDF   STATS   ENSEMBL   UCSC 
 
  195
 
  8
 
 5303
 
 tss;island
 
 0.054699
 
 0.0314488
 
 Not queried
 
  2.2152402
 
 2.79e-01
 
 0.4672865596
  
 
  
 2
 
 234847554
 
 234847856
 
  PDF   STATS   ENSEMBL   UCSC 
 
  303
 
  4
 
 23079
 
 gene;island
 
 0.156535
 
 0.0136238
 
 Not queried
 
  2.2151156
 
 3.43e-01
 
 0.4672865596
  
 
  
 19
 
  44952417
 
  44952618
 
  PDF   STATS   ENSEMBL   UCSC 
 
  202
 
  4
 
 20083
 
 gene
 
 0.038431
 
 0.1263621
 
 Not queried
 
  2.2144599
 
 3.43e-01
 
 0.4672865596
  
 
  
 16
 
    680872
 
    681057
 
  PDF   STATS   ENSEMBL   UCSC 
 
  186
 
  4
 
 15465
 
 island
 
 0.446212
 
 0.0477619
 
 Not queried
 
  2.2121231
 
 3.43e-01
 
 0.4672865596
  
 
  
 3
 
 138067350
 
 138067409
 
  PDF   STATS   ENSEMBL   UCSC 
 
   60
 
  4
 
 28622
 
 tss
 
 0.007718
 
 0.0015335
 
 Not queried
 
  2.2119223
 
 3.43e-01
 
 0.4672865596
  
 
  
 17
 
  46114574
 
  46114933
 
  PDF   STATS   ENSEMBL   UCSC 
 
  360
 
  7
 
 16649
 
 gene;island
 
 0.033107
 
 0.1354282
 
 Not queried
 
  2.2112946
 
 3.18e-01
 
 0.4672865596
  
 
  
 12
 
  50349173
 
  50349522
 
  PDF   STATS   ENSEMBL   UCSC 
 
  350
 
  4
 
 9126
 
 gene;island
 
 0.144249
 
 0.5020661
 
 Not queried
 
  2.1939046
 
 3.43e-01
 
 0.4672865596
  
 
  
 11
 
    417540
 
    417643
 
  PDF   STATS   ENSEMBL   UCSC 
 
  104
 
  4
 
 7011
 
 tss;island
 
 0.597724
 
 0.2951342
 
 Not queried
 
  2.1938341
 
 3.43e-01
 
 0.4672865596
  
 
  
 1
 
   2324205
 
   2324396
 
  PDF   STATS   ENSEMBL   UCSC 
 
  192
 
  4
 
 81
 
 gene;tss;island
 
 0.960961
 
 0.8453853
 
 Not queried
 
  2.1904708
 
 3.43e-01
 
 0.4672865596
  
 
  
 12
 
 130918514
 
 130918994
 
  PDF   STATS   ENSEMBL   UCSC 
 
  481
 
  5
 
 9414
 
 gene
 
 0.534178
 
 0.7017579
 
 Not queried
 
  2.1839310
 
 3.10e-01
 
 0.4672865596
  
 
  
 6
 
  29601398
 
  29601705
 
  PDF   STATS   ENSEMBL   UCSC 
 
  308
 
  6
 
 34750
 
 tss;island
 
 0.546702
 
 0.8102783
 
 Not queried
 
  2.1797495
 
 3.10e-01
 
 0.4672865596
  
 
  
 6
 
  24646254
 
  24646782
 
  PDF   STATS   ENSEMBL   UCSC 
 
  529
 
  7
 
 34670
 
 tss
 
 0.018025
 
 0.0054877
 
 Not queried
 
  2.1795168
 
 3.18e-01
 
 0.4672865596
  
 
  
 8
 
  25905478
 
  25905811
 
  PDF   STATS   ENSEMBL   UCSC 
 
  334
 
  4
 
 40171
 
 island
 
 0.335054
 
 0.6178983
 
 Not queried
 
  2.1776058
 
 3.43e-01
 
 0.4672865596
  
 
  
 6
 
  99292170
 
  99292438
 
  PDF   STATS   ENSEMBL   UCSC 
 
  269
 
  4
 
 36347
 
 island
 
 0.103296
 
 0.0234357
 
 Not queried
 
  2.1738811
 
 3.43e-01
 
 0.4672865596
  
 
  
 5
 
 140305947
 
 140306458
 
  PDF   STATS   ENSEMBL   UCSC 
 
  512
 
 10
 
 31804
 
 gene;island
 
 0.121438
 
 0.0936216
 
 Not queried
 
  2.1720943
 
 2.80e-01
 
 0.4672865596
  
 
  
 10
 
 119312691
 
 119313075
 
  PDF   STATS   ENSEMBL   UCSC 
 
  385
 
  4
 
 6183
 
 island
 
 0.264122
 
 0.0543216
 
 Not queried
 
  2.1719052
 
 3.43e-01
 
 0.4672865596
  
 
  
 5
 
  41870796
 
  41871189
 
  PDF   STATS   ENSEMBL   UCSC 
 
  394
 
  9
 
 32038
 
 tss
 
 0.017950
 
 0.2441021
 
 Not queried
 
  2.1705691
 
 2.97e-01
 
 0.4672865596
  
 
  
 5
 
 135265753
 
 135266135
 
  PDF   STATS   ENSEMBL   UCSC 
 
  383
 
  6
 
 33142
 
 island
 
 0.038047
 
 0.0800113
 
 Not queried
 
  2.1694378
 
 3.10e-01
 
 0.4672865596
  
 
  
 3
 
   8811437
 
   8811758
 
  PDF   STATS   ENSEMBL   UCSC 
 
  322
 
  4
 
 28122
 
 tss
 
 0.872177
 
 0.7026152
 
 Not queried
 
  2.1664265
 
 3.43e-01
 
 0.4672865596
  
 
  
 3
 
  52811297
 
  52811637
 
  PDF   STATS   ENSEMBL   UCSC 
 
  341
 
  4
 
 28390
 
 tss
 
 0.360600
 
 0.6940198
 
 Not queried
 
  2.1661955
 
 3.43e-01
 
 0.4672865596
  
 
  
 5
 
 140186484
 
 140186983
 
  PDF   STATS   ENSEMBL   UCSC 
 
  500
 
  6
 
 31795
 
 gene;island
 
 0.201562
 
 0.6490287
 
 Not queried
 
  2.1648812
 
 3.10e-01
 
 0.4672865596
  
 
  
 1
 
  34632573
 
  34632658
 
  PDF   STATS   ENSEMBL   UCSC 
 
   86
 
  4
 
 1476
 
 tss;island
 
 0.032235
 
 0.1315593
 
 Not queried
 
  2.1648597
 
 3.43e-01
 
 0.4672865596
  
 
  
 17
 
  10741502
 
  10741610
 
  PDF   STATS   ENSEMBL   UCSC 
 
  109
 
  4
 
 17136
 
 tss
 
 0.696469
 
 0.9147984
 
 Not queried
 
  2.1585794
 
 3.43e-01
 
 0.4672865596
  
 
  
 19
 
  57610536
 
  57610870
 
  PDF   STATS   ENSEMBL   UCSC 
 
  335
 
  4
 
 22456
 
 island
 
 0.175656
 
 0.5281522
 
 Not queried
 
  2.1548474
 
 3.43e-01
 
 0.4672865596
  
 
  
 8
 
  99961768
 
  99962118
 
  PDF   STATS   ENSEMBL   UCSC 
 
  351
 
  4
 
 39375
 
 gene;island
 
 0.286597
 
 0.5322123
 
 Not queried
 
  2.1497231
 
 3.43e-01
 
 0.4672865596
  
 
  
 6
 
  10883834
 
  10884314
 
  PDF   STATS   ENSEMBL   UCSC 
 
  481
 
  6
 
 35441
 
 island
 
 0.636838
 
 0.4105571
 
 Not queried
 
  2.1280582
 
 3.10e-01
 
 0.4672865596
  
 
  
 11
 
 118401536
 
 118401801
 
  PDF   STATS   ENSEMBL   UCSC 
 
  266
 
  4
 
 7734
 
 tss
 
 0.002707
 
 0.0037170
 
 Not queried
 
  2.1154573
 
 3.43e-01
 
 0.4672865596
  
 
  
 11
 
  64703415
 
  64703818
 
  PDF   STATS   ENSEMBL   UCSC 
 
  404
 
  5
 
 7409
 
 tss
 
 0.430469
 
 0.6263208
 
 Not queried
 
  2.1135153
 
 3.10e-01
 
 0.4672865596
  
 
  
 19
 
   1401576
 
   1401781
 
  PDF   STATS   ENSEMBL   UCSC 
 
  206
 
  4
 
 20304
 
 tss
 
 0.102313
 
 0.0227106
 
 Not queried
 
  2.1130834
 
 3.43e-01
 
 0.4672865596
  
 
  
 2
 
 176943669
 
 176943914
 
  PDF   STATS   ENSEMBL   UCSC 
 
  246
 
  4
 
 24886
 
 island
 
 0.326741
 
 0.0608433
 
 Not queried
 
  2.1111514
 
 3.43e-01
 
 0.4672865596
  
 
  
 19
 
  58695006
 
  58695164
 
  PDF   STATS   ENSEMBL   UCSC 
 
  159
 
  4
 
 20258
 
 gene;island
 
 0.016512
 
 0.0065461
 
 Not queried
 
  2.1069084
 
 3.43e-01
 
 0.4672865596
  
 
  
 19
 
  49198900
 
  49199217
 
  PDF   STATS   ENSEMBL   UCSC 
 
  318
 
  4
 
 20923
 
 tss
 
 0.334459
 
 0.5102684
 
 Not queried
 
  2.1049489
 
 3.43e-01
 
 0.4672865596
  
 
  
 6
 
  25930894
 
  25931205
 
  PDF   STATS   ENSEMBL   UCSC 
 
  312
 
  5
 
 34676
 
 tss
 
 0.591929
 
 0.9180470
 
 Not queried
 
  2.1039100
 
 3.10e-01
 
 0.4672865596
  
 
  
 14
 
 102504851
 
 102505062
 
  PDF   STATS   ENSEMBL   UCSC 
 
  212
 
  4
 
 12222
 
 gene
 
 0.795198
 
 0.9821636
 
 Not queried
 
  2.1024831
 
 3.43e-01
 
 0.4672865596
  
 
  
 6
 
  74103959
 
  74104137
 
  PDF   STATS   ENSEMBL   UCSC 
 
  179
 
  4
 
 35069
 
 tss;island
 
 0.607796
 
 0.9115497
 
 Not queried
 
  2.0982045
 
 3.43e-01
 
 0.4672865596
  
 
  
 14
 
  95826570
 
  95826997
 
  PDF   STATS   ENSEMBL   UCSC 
 
  428
 
  5
 
 13110
 
 island
 
 0.608986
 
 0.4342987
 
 Not queried
 
  2.0897789
 
 3.10e-01
 
 0.4672865596
  
 
  
 1
 
  18691778
 
  18692019
 
  PDF   STATS   ENSEMBL   UCSC 
 
  242
 
  5
 
 271
 
 gene;island
 
 0.176437
 
 0.4759527
 
 Not queried
 
  2.0860391
 
 3.10e-01
 
 0.4672865596
  
 
  
 10
 
  99338056
 
  99338240
 
  PDF   STATS   ENSEMBL   UCSC 
 
  185
 
  4
 
 4698
 
 gene;island
 
 0.870931
 
 0.9648793
 
 Not queried
 
  2.0775971
 
 3.43e-01
 
 0.4672865596
  
 
  
 3
 
  23987430
 
  23987467
 
  PDF   STATS   ENSEMBL   UCSC 
 
   38
 
  4
 
 27703
 
 gene;tss;island
 
 0.002651
 
 0.0093602
 
 Not queried
 
  2.0752089
 
 3.43e-01
 
 0.4672865596
  
 
  
 19
 
  44220122
 
  44220337
 
  PDF   STATS   ENSEMBL   UCSC 
 
  216
 
  4
 
 22093
 
 island
 
 0.743933
 
 0.5106224
 
 Not queried
 
  2.0732524
 
 3.43e-01
 
 0.4672865596
  
 
  
 19
 
  42509801
 
  42510093
 
  PDF   STATS   ENSEMBL   UCSC 
 
  293
 
  4
 
 20063
 
 gene;island
 
 0.184139
 
 0.4754262
 
 Not queried
 
  2.0681979
 
 3.43e-01
 
 0.4672865596
  
 
  
 16
 
    725416
 
    725581
 
  PDF   STATS   ENSEMBL   UCSC 
 
  166
 
  4
 
 14975
 
 tss
 
 0.010630
 
 0.0335754
 
 Not queried
 
  2.0647046
 
 3.43e-01
 
 0.4672865596
  
 
  
 1
 
   2100169
 
   2100435
 
  PDF   STATS   ENSEMBL   UCSC 
 
  267
 
  4
 
 66
 
 gene
 
 0.517585
 
 0.7332220
 
 Not queried
 
  2.0640799
 
 3.43e-01
 
 0.4672865596
  
 
  
 8
 
 102236522
 
 102236831
 
  PDF   STATS   ENSEMBL   UCSC 
 
  310
 
  4
 
 40505
 
 island
 
 0.522079
 
 0.7943313
 
 Not queried
 
  2.0637976
 
 3.43e-01
 
 0.4672865596
  
 
  
 12
 
  91347805
 
  91348242
 
  PDF   STATS   ENSEMBL   UCSC 
 
  438
 
  4
 
 9240
 
 gene;island
 
 0.535316
 
 0.7498978
 
 Not queried
 
  2.0609856
 
 3.43e-01
 
 0.4672865596
  
 
  
 14
 
 101908561
 
 101909021
 
  PDF   STATS   ENSEMBL   UCSC 
 
  461
 
  6
 
 13156
 
 island
 
 0.238603
 
 0.5907211
 
 Not queried
 
  2.0601939
 
 3.10e-01
 
 0.4672865596
  
 
  
 8
 
  22960461
 
  22960619
 
  PDF   STATS   ENSEMBL   UCSC 
 
  159
 
  4
 
 39239
 
 gene
 
 0.042317
 
 0.2128241
 
 Not queried
 
  2.0589708
 
 3.43e-01
 
 0.4672865596
  
 
  
 10
 
 134735893
 
 134736248
 
  PDF   STATS   ENSEMBL   UCSC 
 
  356
 
  5
 
 6318
 
 island
 
 0.841673
 
 0.9070316
 
 Not queried
 
  2.0575420
 
 3.10e-01
 
 0.4672865596
  
 
  
 1
 
  21996199
 
  21996376
 
  PDF   STATS   ENSEMBL   UCSC 
 
  178
 
  4
 
 1326
 
 tss;island
 
 0.016573
 
 0.0893301
 
 Not queried
 
  2.0564954
 
 3.43e-01
 
 0.4672865596
  
 
  
 4
 
 113970617
 
 113970935
 
  PDF   STATS   ENSEMBL   UCSC 
 
  319
 
  6
 
 30103
 
 gene
 
 0.657963
 
 0.1285238
 
 Not queried
 
  2.0557969
 
 3.10e-01
 
 0.4672865596
  
 
  
 7
 
   4869981
 
   4870162
 
  PDF   STATS   ENSEMBL   UCSC 
 
  182
 
  4
 
 36859
 
 gene
 
 0.425787
 
 0.1323347
 
 Not queried
 
  2.0521510
 
 3.43e-01
 
 0.4672865596
  
 
  
 19
 
    863054
 
    863244
 
  PDF   STATS   ENSEMBL   UCSC 
 
  191
 
  4
 
 19635
 
 gene;island
 
 0.276542
 
 0.0273721
 
 Not queried
 
  2.0519222
 
 3.43e-01
 
 0.4672865596
  
 
  
 12
 
  85673200
 
  85673347
 
  PDF   STATS   ENSEMBL   UCSC 
 
  148
 
  4
 
 9900
 
 tss;island
 
 0.046019
 
 0.0974175
 
 Not queried
 
  2.0469976
 
 3.43e-01
 
 0.4672865596
  
 
  
 6
 
 168533375
 
 168533689
 
  PDF   STATS   ENSEMBL   UCSC 
 
  315
 
  4
 
 36659
 
 island
 
 0.380227
 
 0.0730777
 
 Not queried
 
  2.0445274
 
 3.43e-01
 
 0.4672865596
  
 
  
 14
 
  93170534
 
  93170970
 
  PDF   STATS   ENSEMBL   UCSC 
 
  437
 
  4
 
 12183
 
 gene
 
 0.008838
 
 0.0040834
 
 Not queried
 
  2.0441793
 
 3.43e-01
 
 0.4672865596
  
 
  
 22
 
  46731652
 
  46731750
 
  PDF   STATS   ENSEMBL   UCSC 
 
   99
 
  4
 
 26912
 
 gene;island
 
 0.003472
 
 0.0010393
 
 Not queried
 
  2.0425519
 
 3.43e-01
 
 0.4672865596
  
 
  
 7
 
  99766203
 
  99766665
 
  PDF   STATS   ENSEMBL   UCSC 
 
  463
 
  6
 
 38753
 
 island
 
 0.152203
 
 0.5441352
 
 Not queried
 
  2.0338215
 
 3.10e-01
 
 0.4672865596
  
 
  
 19
 
  18496756
 
  18497201
 
  PDF   STATS   ENSEMBL   UCSC 
 
  446
 
  8
 
 21778
 
 island
 
 0.353694
 
 0.0562590
 
 Not queried
 
  2.0329689
 
 2.79e-01
 
 0.4672865596
  
 
  
 10
 
 118386951
 
 118387518
 
  PDF   STATS   ENSEMBL   UCSC 
 
  568
 
  5
 
 4776
 
 gene;island
 
 0.402633
 
 0.0766412
 
 Not queried
 
  2.0237869
 
 3.10e-01
 
 0.4672865596
  
 
  
 2
 
 237120069
 
 237120313
 
  PDF   STATS   ENSEMBL   UCSC 
 
  245
 
  4
 
 23090
 
 gene;island
 
 0.416691
 
 0.6071160
 
 Not queried
 
  2.0233639
 
 3.43e-01
 
 0.4672865596
  
 
  
 16
 
  84423876
 
  84424282
 
  PDF   STATS   ENSEMBL   UCSC 
 
  407
 
  4
 
 14869
 
 gene
 
 0.690273
 
 0.8976316
 
 Not queried
 
  2.0227703
 
 3.43e-01
 
 0.4672865596
  
 
  
 5
 
  87963342
 
  87963592
 
  PDF   STATS   ENSEMBL   UCSC 
 
  251
 
  4
 
 31676
 
 gene
 
 0.026117
 
 0.0913248
 
 Not queried
 
  2.0119288
 
 3.43e-01
 
 0.4672865596
  
 
  
 19
 
  38924045
 
  38924330
 
  PDF   STATS   ENSEMBL   UCSC 
 
  286
 
  7
 
 20755
 
 tss
 
 0.083490
 
 0.3009042
 
 Not queried
 
  1.9976656
 
 3.18e-01
 
 0.4672865596
  
 
  
 19
 
  16830613
 
  16830749
 
  PDF   STATS   ENSEMBL   UCSC 
 
  137
 
  4
 
 20575
 
 tss
 
 0.574857
 
 0.0942908
 
 Not queried
 
  1.9953759
 
 3.43e-01
 
 0.4672865596
  
 
  
 22
 
  21398553
 
  21398740
 
  PDF   STATS   ENSEMBL   UCSC 
 
  188
 
  4
 
 26992
 
 tss;island
 
 0.209213
 
 0.6496377
 
 Not queried
 
  1.9919407
 
 3.43e-01
 
 0.4672865596
  
 
  
 5
 
  95768915
 
  95769079
 
  PDF   STATS   ENSEMBL   UCSC 
 
  165
 
  7
 
 32974
 
 island
 
 0.294900
 
 0.1921766
 
 Not queried
 
  1.9881841
 
 3.18e-01
 
 0.4672865596
  
 
  
 7
 
 134464242
 
 134464411
 
  PDF   STATS   ENSEMBL   UCSC 
 
  170
 
  4
 
 37231
 
 gene
 
 0.504183
 
 0.8016365
 
 Not queried
 
  1.9765045
 
 3.43e-01
 
 0.4672865596
  
 
  
 6
 
  29815787
 
  29815902
 
  PDF   STATS   ENSEMBL   UCSC 
 
  116
 
  4
 
 35637
 
 island
 
 0.059363
 
 0.0146186
 
 Not queried
 
  1.9734585
 
 3.43e-01
 
 0.4672865596
  
 
  
 2
 
  27256009
 
  27256285
 
  PDF   STATS   ENSEMBL   UCSC 
 
  277
 
  4
 
 22619
 
 gene;island
 
 0.003140
 
 0.0023572
 
 Not queried
 
  1.9719473
 
 3.43e-01
 
 0.4672865596
  
 
  
 2
 
  74875227
 
  74875548
 
  PDF   STATS   ENSEMBL   UCSC 
 
  322
 
  8
 
 23465
 
 tss;island
 
 0.531092
 
 0.3321873
 
 Not queried
 
  1.9696407
 
 2.79e-01
 
 0.4672865596
  
 
  
 8
 
  85094742
 
  85094923
 
  PDF   STATS   ENSEMBL   UCSC 
 
  182
 
  4
 
 39787
 
 tss;island
 
 0.053784
 
 0.0123299
 
 Not queried
 
  1.9672277
 
 3.43e-01
 
 0.4672865596
  
 
  
 22
 
  18484303
 
  18484586
 
  PDF   STATS   ENSEMBL   UCSC 
 
  284
 
  4
 
 26782
 
 gene;island
 
 0.001622
 
 0.0108866
 
 Not queried
 
  1.9525437
 
 3.43e-01
 
 0.4672865596
  
 
  
 5
 
  27038707
 
  27038836
 
  PDF   STATS   ENSEMBL   UCSC 
 
  130
 
  4
 
 32003
 
 tss
 
 0.240399
 
 0.5459505
 
 Not queried
 
  1.9477319
 
 3.43e-01
 
 0.4672865596
  
 
  
 7
 
  65878734
 
  65879115
 
  PDF   STATS   ENSEMBL   UCSC 
 
  382
 
  4
 
 38534
 
 island
 
 0.044069
 
 0.0066514
 
 Not queried
 
  1.9446938
 
 3.43e-01
 
 0.4672865596
  
 
  
 7
 
   4838902
 
   4839249
 
  PDF   STATS   ENSEMBL   UCSC 
 
  348
 
  5
 
 36856
 
 gene
 
 0.725797
 
 0.9106134
 
 Not queried
 
  1.9390198
 
 3.10e-01
 
 0.4672865596
  
 
  
 2
 
  89065319
 
  89065379
 
  PDF   STATS   ENSEMBL   UCSC 
 
   61
 
  4
 
 23504
 
 tss
 
 0.205164
 
 0.1159613
 
 Not queried
 
  1.9362456
 
 3.43e-01
 
 0.4672865596
  
 
  
 8
 
  98290148
 
  98290601
 
  PDF   STATS   ENSEMBL   UCSC 
 
  454
 
  9
 
 40476
 
 island
 
 0.172124
 
 0.0517428
 
 Not queried
 
  1.9340912
 
 2.97e-01
 
 0.4672865596
  
 
  
 13
 
 113119625
 
 113119864
 
  PDF   STATS   ENSEMBL   UCSC 
 
  240
 
  4
 
 11922
 
 island
 
 0.808388
 
 0.9247676
 
 Not queried
 
  1.9312717
 
 3.43e-01
 
 0.4672865596
  
 
  
 1
 
 215256254
 
 215256978
 
  PDF   STATS   ENSEMBL   UCSC 
 
  725
 
  9
 
 1009
 
 gene;island
 
 0.017198
 
 0.0082082
 
 Not queried
 
  1.9306657
 
 3.40e-01
 
 0.4672865596
  
 
  
 4
 
  96761264
 
  96761796
 
  PDF   STATS   ENSEMBL   UCSC 
 
  533
 
  5
 
 30064
 
 gene
 
 0.564679
 
 0.8012380
 
 Not queried
 
  1.9182411
 
 3.10e-01
 
 0.4672865596
  
 
  
 12
 
  68647289
 
  68647590
 
  PDF   STATS   ENSEMBL   UCSC 
 
  302
 
  6
 
 9853
 
 tss
 
 0.751035
 
 0.9372766
 
 Not queried
 
  1.9168630
 
 3.10e-01
 
 0.4672865596
  
 
  
 6
 
  17102310
 
  17102678
 
  PDF   STATS   ENSEMBL   UCSC 
 
  369
 
  6
 
 35473
 
 island
 
 0.067495
 
 0.2304071
 
 Not queried
 
  1.9165859
 
 3.10e-01
 
 0.4672865596
  
 
  
 5
 
   1286042
 
   1286354
 
  PDF   STATS   ENSEMBL   UCSC 
 
  313
 
  4
 
 31534
 
 gene;island
 
 0.959752
 
 0.8553669
 
 Not queried
 
  1.9143480
 
 3.43e-01
 
 0.4672865596
  
 
  
 11
 
  76092311
 
  76092535
 
  PDF   STATS   ENSEMBL   UCSC 
 
  225
 
  4
 
 7574
 
 tss;island
 
 0.002992
 
 0.0030843
 
 Not queried
 
  1.9130348
 
 3.43e-01
 
 0.4672865596
  
 
  
 5
 
  87985809
 
  87986067
 
  PDF   STATS   ENSEMBL   UCSC 
 
  259
 
  4
 
 32949
 
 island
 
 0.080803
 
 0.0226861
 
 Not queried
 
  1.9127369
 
 3.43e-01
 
 0.4672865596
  
 
  
 11
 
    330872
 
    331179
 
  PDF   STATS   ENSEMBL   UCSC 
 
  308
 
  4
 
 7843
 
 island
 
 0.557176
 
 0.7790861
 
 Not queried
 
  1.9125473
 
 3.43e-01
 
 0.4672865596
  
 
  
 5
 
 100238655
 
 100238983
 
  PDF   STATS   ENSEMBL   UCSC 
 
  329
 
  4
 
 31702
 
 gene
 
 0.000173
 
 0.0010354
 
 Not queried
 
  1.9108189
 
 3.43e-01
 
 0.4672865596
  
 
  
 8
 
 110703888
 
 110704289
 
  PDF   STATS   ENSEMBL   UCSC 
 
  402
 
  6
 
 40541
 
 island
 
 0.011671
 
 0.0872041
 
 Not queried
 
  1.9046066
 
 3.10e-01
 
 0.4672865596
  
 
  
 4
 
   4405844
 
   4406200
 
  PDF   STATS   ENSEMBL   UCSC 
 
  357
 
  6
 
 29916
 
 gene
 
 0.529348
 
 0.6738702
 
 Not queried
 
  1.9016819
 
 3.10e-01
 
 0.4672865596
  
 
  
 4
 
 187509603
 
 187510183
 
  PDF   STATS   ENSEMBL   UCSC 
 
  581
 
  5
 
 30216
 
 gene
 
 0.466449
 
 0.8344066
 
 Not queried
 
  1.9012322
 
 3.10e-01
 
 0.4672865596
  
 
  
 14
 
  99664107
 
  99664344
 
  PDF   STATS   ENSEMBL   UCSC 
 
  238
 
  5
 
 12201
 
 gene
 
 0.212224
 
 0.6629196
 
 Not queried
 
  1.8940121
 
 3.10e-01
 
 0.4672865596
  
 
  
 4
 
  57547347
 
  57547699
 
  PDF   STATS   ENSEMBL   UCSC 
 
  353
 
  4
 
 30011
 
 gene
 
 0.270131
 
 0.3905145
 
 Not queried
 
  1.8889888
 
 3.43e-01
 
 0.4672865596
  
 
  
 7
 
  20838339
 
  20838631
 
  PDF   STATS   ENSEMBL   UCSC 
 
  293
 
  5
 
 38296
 
 island
 
 0.173723
 
 0.0133535
 
 Not queried
 
  1.8861823
 
 3.10e-01
 
 0.4672865596
  
 
  
 10
 
   8101307
 
   8101566
 
  PDF   STATS   ENSEMBL   UCSC 
 
  260
 
  4
 
 4490
 
 gene;island
 
 0.918740
 
 0.3210888
 
 Not queried
 
  1.8780180
 
 3.43e-01
 
 0.4672865596
  
 
  
 14
 
  85996101
 
  85996393
 
  PDF   STATS   ENSEMBL   UCSC 
 
  293
 
  8
 
 12542
 
 tss
 
 0.057501
 
 0.1967738
 
 Not queried
 
  1.8679777
 
 2.79e-01
 
 0.4672865596
  
 
  
 10
 
  97889373
 
  97889448
 
  PDF   STATS   ENSEMBL   UCSC 
 
   76
 
  4
 
 5253
 
 tss
 
 0.486244
 
 0.9451948
 
 Not queried
 
  1.8645821
 
 3.43e-01
 
 0.4672865596
  
 
  
 11
 
  84634581
 
  84634735
 
  PDF   STATS   ENSEMBL   UCSC 
 
  155
 
  4
 
 7601
 
 tss
 
 0.659709
 
 0.8904612
 
 Not queried
 
  1.8636017
 
 3.43e-01
 
 0.4672865596
  
 
  
 14
 
  77737495
 
  77737800
 
  PDF   STATS   ENSEMBL   UCSC 
 
  306
 
  6
 
 13032
 
 island
 
 0.361521
 
 0.0550492
 
 Not queried
 
  1.8625142
 
 3.10e-01
 
 0.4672865596
  
 
  
 8
 
  49231559
 
  49231786
 
  PDF   STATS   ENSEMBL   UCSC 
 
  228
 
  4
 
 40280
 
 island
 
 0.015225
 
 0.0496185
 
 Not queried
 
  1.8615720
 
 3.43e-01
 
 0.4672865596
  
 
  
 8
 
 142452378
 
 142452509
 
  PDF   STATS   ENSEMBL   UCSC 
 
  132
 
  4
 
 39454
 
 gene;island
 
 0.718241
 
 0.9007904
 
 Not queried
 
  1.8606387
 
 3.43e-01
 
 0.4672865596
  
 
  
 3
 
 184529753
 
 184529876
 
  PDF   STATS   ENSEMBL   UCSC 
 
  124
 
  5
 
 28764
 
 tss
 
 0.002187
 
 0.0070356
 
 Not queried
 
  1.8588633
 
 3.10e-01
 
 0.4672865596
  
 
  
 2
 
  97202260
 
  97202435
 
  PDF   STATS   ENSEMBL   UCSC 
 
  176
 
  4
 
 23519
 
 tss
 
 0.001626
 
 0.0060287
 
 Not queried
 
  1.8582312
 
 3.43e-01
 
 0.4672865596
  
 
  
 13
 
  51640297
 
  51640442
 
  PDF   STATS   ENSEMBL   UCSC 
 
  146
 
  4
 
 11450
 
 tss
 
 0.019940
 
 0.0855513
 
 Not queried
 
  1.8571878
 
 3.43e-01
 
 0.4672865596
  
 
  
 7
 
 150216872
 
 150217081
 
  PDF   STATS   ENSEMBL   UCSC 
 
  210
 
  4
 
 37275
 
 gene
 
 0.500586
 
 0.8722164
 
 Not queried
 
  1.8536069
 
 3.43e-01
 
 0.4672865596
  
 
  
 15
 
  45422325
 
  45422553
 
  PDF   STATS   ENSEMBL   UCSC 
 
  229
 
  4
 
 13328
 
 gene;tss;island
 
 0.444123
 
 0.6630927
 
 Not queried
 
  1.8528942
 
 3.43e-01
 
 0.4672865596
  
 
  
 15
 
  43398302
 
  43398581
 
  PDF   STATS   ENSEMBL   UCSC 
 
  280
 
  6
 
 13622
 
 tss
 
 0.006909
 
 0.0024242
 
 Not queried
 
  1.8527636
 
 3.10e-01
 
 0.4672865596
  
 
  
 16
 
  79633452
 
  79633901
 
  PDF   STATS   ENSEMBL   UCSC 
 
  450
 
  4
 
 14851
 
 gene;island
 
 0.248118
 
 0.0995400
 
 Not queried
 
  1.8518636
 
 3.43e-01
 
 0.4672865596
  
 
  
 2
 
 207506975
 
 207507096
 
  PDF   STATS   ENSEMBL   UCSC 
 
  122
 
  4
 
 23838
 
 tss
 
 0.096460
 
 0.2328940
 
 Not queried
 
  1.8513196
 
 3.43e-01
 
 0.4672865596
  
 
  
 16
 
  88575243
 
  88575557
 
  PDF   STATS   ENSEMBL   UCSC 
 
  315
 
  4
 
 14907
 
 gene;island
 
 0.728737
 
 0.9345652
 
 Not queried
 
  1.8508321
 
 3.43e-01
 
 0.4672865596
  
 
  
 10
 
  38382849
 
  38383525
 
  PDF   STATS   ENSEMBL   UCSC 
 
  677
 
 11
 
 5673
 
 island
 
 0.017106
 
 0.0065924
 
 Not queried
 
  1.8506960
 
 3.32e-01
 
 0.4672865596
  
 
  
 19
 
   7911120
 
   7911362
 
  PDF   STATS   ENSEMBL   UCSC 
 
  243
 
  5
 
 20409
 
 tss
 
 0.403862
 
 0.5895586
 
 Not queried
 
  1.8464247
 
 3.10e-01
 
 0.4672865596
  
 
  
 19
 
  42891265
 
  42891428
 
  PDF   STATS   ENSEMBL   UCSC 
 
  164
 
  4
 
 20071
 
 gene;island
 
 0.025582
 
 0.1804435
 
 Not queried
 
  1.8452558
 
 3.43e-01
 
 0.4672865596
  
 
  
 16
 
  85171054
 
  85171295
 
  PDF   STATS   ENSEMBL   UCSC 
 
  242
 
  4
 
 16150
 
 island
 
 0.019453
 
 0.1229229
 
 Not queried
 
  1.8439188
 
 3.43e-01
 
 0.4672865596
  
 
  
 8
 
  41518004
 
  41518139
 
  PDF   STATS   ENSEMBL   UCSC 
 
  136
 
  4
 
 39289
 
 gene
 
 0.799872
 
 0.8989271
 
 Not queried
 
  1.8380454
 
 3.43e-01
 
 0.4672865596
  
 
  
 19
 
  57352014
 
  57352074
 
  PDF   STATS   ENSEMBL   UCSC 
 
   61
 
  4
 
 20242
 
 gene
 
 0.952955
 
 0.7325450
 
 Not queried
 
  1.8367354
 
 3.43e-01
 
 0.4672865596
  
 
  
 5
 
 115297690
 
 115298231
 
  PDF   STATS   ENSEMBL   UCSC 
 
  542
 
  6
 
 33029
 
 island
 
 0.382904
 
 0.0580617
 
 Not queried
 
  1.8314980
 
 3.10e-01
 
 0.4672865596
  
 
  
 14
 
 102554826
 
 102554977
 
  PDF   STATS   ENSEMBL   UCSC 
 
  152
 
  4
 
 12226
 
 gene;tss;island
 
 0.074109
 
 0.2476712
 
 Not queried
 
  1.8314559
 
 3.43e-01
 
 0.4672865596
  
 
  
 3
 
 150690183
 
 150690585
 
  PDF   STATS   ENSEMBL   UCSC 
 
  403
 
  7
 
 27996
 
 gene
 
 0.497193
 
 0.8496373
 
 Not queried
 
  1.8310298
 
 3.18e-01
 
 0.4672865596
  
 
  
 11
 
  57232331
 
  57232742
 
  PDF   STATS   ENSEMBL   UCSC 
 
  412
 
  5
 
 6613
 
 gene;island
 
 0.805244
 
 0.5297770
 
 Not queried
 
  1.8178778
 
 3.10e-01
 
 0.4672865596
  
 
  
 17
 
  48072565
 
  48072833
 
  PDF   STATS   ENSEMBL   UCSC 
 
  269
 
  4
 
 18537
 
 island
 
 0.010590
 
 0.0540234
 
 Not queried
 
  1.8155088
 
 3.43e-01
 
 0.4672865596
  
 
  
 15
 
  25434030
 
  25434533
 
  PDF   STATS   ENSEMBL   UCSC 
 
  504
 
  7
 
 13531
 
 tss
 
 0.271933
 
 0.3835864
 
 Not queried
 
  1.8145914
 
 3.18e-01
 
 0.4672865596
  
 
  
 13
 
  31773806
 
  31773954
 
  PDF   STATS   ENSEMBL   UCSC 
 
  149
 
  4
 
 11379
 
 tss;island
 
 0.019906
 
 0.3699127
 
 Not queried
 
  1.8113872
 
 3.43e-01
 
 0.4672865596
  
 
  
 7
 
 140179446
 
 140179608
 
  PDF   STATS   ENSEMBL   UCSC 
 
  163
 
  4
 
 37938
 
 tss;island
 
 0.022343
 
 0.0055727
 
 Not queried
 
  1.8095250
 
 3.43e-01
 
 0.4672865596
  
 
  
 18
 
  10414355
 
  10414556
 
  PDF   STATS   ENSEMBL   UCSC 
 
  202
 
  4
 
 19389
 
 island
 
 0.700827
 
 0.9419741
 
 Not queried
 
  1.8094780
 
 3.43e-01
 
 0.4672865596
  
 
  
 16
 
    875881
 
    876216
 
  PDF   STATS   ENSEMBL   UCSC 
 
  336
 
  4
 
 15492
 
 island
 
 0.567528
 
 0.8890019
 
 Not queried
 
  1.8092433
 
 3.43e-01
 
 0.4672865596
  
 
  
 4
 
 187422005
 
 187422119
 
  PDF   STATS   ENSEMBL   UCSC 
 
  115
 
  5
 
 31481
 
 island
 
 0.781438
 
 0.8954278
 
 Not queried
 
  1.8060746
 
 3.10e-01
 
 0.4672865596
  
 
  
 15
 
  23932370
 
  23932412
 
  PDF   STATS   ENSEMBL   UCSC 
 
   43
 
  4
 
 13254
 
 gene;island
 
 0.339253
 
 0.5319708
 
 Not queried
 
  1.8055784
 
 3.43e-01
 
 0.4672865596
  
 
  
 1
 
 228509380
 
 228509762
 
  PDF   STATS   ENSEMBL   UCSC 
 
  383
 
  5
 
 1056
 
 gene;island
 
 0.396701
 
 0.5274892
 
 Not queried
 
  1.8037178
 
 3.10e-01
 
 0.4672865596
  
 
  
 18
 
  24445134
 
  24445318
 
  PDF   STATS   ENSEMBL   UCSC 
 
  185
 
  4
 
 19082
 
 gene;island
 
 0.189835
 
 0.4069941
 
 Not queried
 
  1.7998007
 
 3.43e-01
 
 0.4672865596
  
 
  
 15
 
  31195612
 
  31195982
 
  PDF   STATS   ENSEMBL   UCSC 
 
  371
 
  8
 
 13549
 
 tss
 
 0.033922
 
 0.0121498
 
 Not queried
 
  1.7971874
 
 2.79e-01
 
 0.4672865596
  
 
  
 6
 
  31589593
 
  31589994
 
  PDF   STATS   ENSEMBL   UCSC 
 
  402
 
  5
 
 33909
 
 gene;tss;island
 
 0.334684
 
 0.4499689
 
 Not queried
 
  1.7964980
 
 3.10e-01
 
 0.4672865596
  
 
  
 11
 
  36422377
 
  36422615
 
  PDF   STATS   ENSEMBL   UCSC 
 
  239
 
  5
 
 6585
 
 gene
 
 0.281281
 
 0.1246554
 
 Not queried
 
  1.7945734
 
 3.10e-01
 
 0.4672865596
  
 
  
 13
 
  37248078
 
  37248423
 
  PDF   STATS   ENSEMBL   UCSC 
 
  346
 
  4
 
 11133
 
 gene;island
 
 0.071313
 
 0.1571770
 
 Not queried
 
  1.7937387
 
 3.43e-01
 
 0.4672865596
  
 
  
 1
 
 226410871
 
 226411270
 
  PDF   STATS   ENSEMBL   UCSC 
 
  400
 
  8
 
 2288
 
 tss;island
 
 0.050233
 
 0.2854047
 
 Not queried
 
  1.7912483
 
 2.79e-01
 
 0.4672865596
  
 
  
 1
 
 205290905
 
 205291215
 
  PDF   STATS   ENSEMBL   UCSC 
 
  311
 
  4
 
 2204
 
 tss
 
 0.080803
 
 0.1478778
 
 Not queried
 
  1.7878672
 
 3.43e-01
 
 0.4672865596
  
 
  
 7
 
 158936508
 
 158936739
 
  PDF   STATS   ENSEMBL   UCSC 
 
  232
 
  4
 
 37385
 
 gene;island
 
 0.030126
 
 0.0107116
 
 Not queried
 
  1.7862965
 
 3.43e-01
 
 0.4672865596
  
 
  
 20
 
  25566178
 
  25566563
 
  PDF   STATS   ENSEMBL   UCSC 
 
  386
 
  7
 
 25652
 
 tss;island
 
 0.074853
 
 0.0423922
 
 Not queried
 
  1.7812927
 
 3.18e-01
 
 0.4672865596
  
 
  
 7
 
 154863176
 
 154863381
 
  PDF   STATS   ENSEMBL   UCSC 
 
  206
 
  4
 
 37305
 
 gene;island
 
 0.256657
 
 0.4852402
 
 Not queried
 
  1.7802929
 
 3.43e-01
 
 0.4672865596
  
 
  
 11
 
  74870268
 
  74870741
 
  PDF   STATS   ENSEMBL   UCSC 
 
  474
 
  5
 
 6831
 
 gene;tss
 
 0.825803
 
 0.9132805
 
 Not queried
 
  1.7801001
 
 3.10e-01
 
 0.4672865596
  
 
  
 13
 
  33780205
 
  33780362
 
  PDF   STATS   ENSEMBL   UCSC 
 
  158
 
  4
 
 11386
 
 tss
 
 0.025838
 
 0.0096066
 
 Not queried
 
  1.7779554
 
 3.43e-01
 
 0.4672865596
  
 
  
 16
 
  88547533
 
  88547861
 
  PDF   STATS   ENSEMBL   UCSC 
 
  329
 
  4
 
 14903
 
 gene
 
 0.856837
 
 0.9534248
 
 Not queried
 
  1.7727482
 
 3.43e-01
 
 0.4672865596
  
 
  
 2
 
 240302585
 
 240302855
 
  PDF   STATS   ENSEMBL   UCSC 
 
  271
 
  4
 
 23118
 
 gene
 
 0.029434
 
 0.1852496
 
 Not queried
 
  1.7726793
 
 3.43e-01
 
 0.4672865596
  
 
  
 16
 
  82044738
 
  82045078
 
  PDF   STATS   ENSEMBL   UCSC 
 
  341
 
  5
 
 14857
 
 gene
 
 0.042743
 
 0.0189652
 
 Not queried
 
  1.7677628
 
 3.10e-01
 
 0.4672865596
  
 
  
 1
 
  20813574
 
  20813666
 
  PDF   STATS   ENSEMBL   UCSC 
 
   93
 
  4
 
 1313
 
 tss;island
 
 0.123202
 
 0.0347390
 
 Not queried
 
  1.7641187
 
 3.43e-01
 
 0.4672865596
  
 
  
 1
 
   3306999
 
   3307215
 
  PDF   STATS   ENSEMBL   UCSC 
 
  217
 
  4
 
 122
 
 gene;island
 
 0.736343
 
 0.8683705
 
 Not queried
 
  1.7624648
 
 3.43e-01
 
 0.4672865596
  
 
  
 12
 
   5018715
 
   5019066
 
  PDF   STATS   ENSEMBL   UCSC 
 
  352
 
  9
 
 9502
 
 tss
 
 0.031339
 
 0.0201291
 
 Not queried
 
  1.7621913
 
 3.40e-01
 
 0.4672865596
  
 
  
 17
 
     96392
 
     96796
 
  PDF   STATS   ENSEMBL   UCSC 
 
  405
 
  6
 
 16285
 
 gene;island
 
 0.554628
 
 0.7280863
 
 Not queried
 
  1.7592670
 
 3.10e-01
 
 0.4672865596
  
 
  
 18
 
  11851233
 
  11851347
 
  PDF   STATS   ENSEMBL   UCSC 
 
  115
 
  6
 
 19184
 
 tss
 
 0.005811
 
 0.0025104
 
 Not queried
 
  1.7576217
 
 3.10e-01
 
 0.4672865596
  
 
  
 12
 
  81471311
 
  81471757
 
  PDF   STATS   ENSEMBL   UCSC 
 
  447
 
  7
 
 9894
 
 tss
 
 0.025681
 
 0.0524423
 
 Not queried
 
  1.7540530
 
 3.18e-01
 
 0.4672865596
  
 
  
 11
 
 129939817
 
 129939958
 
  PDF   STATS   ENSEMBL   UCSC 
 
  142
 
  4
 
 6972
 
 gene
 
 0.001338
 
 0.0123683
 
 Not queried
 
  1.7522569
 
 3.43e-01
 
 0.4672865596
  
 
  
 17
 
  42147905
 
  42148070
 
  PDF   STATS   ENSEMBL   UCSC 
 
  166
 
  7
 
 17400
 
 tss
 
 0.004271
 
 0.0108313
 
 Not queried
 
  1.7513815
 
 3.18e-01
 
 0.4672865596
  
 
  
 5
 
 140165938
 
 140166213
 
  PDF   STATS   ENSEMBL   UCSC 
 
  276
 
  4
 
 31793
 
 gene;island
 
 0.300974
 
 0.5882800
 
 Not queried
 
  1.7503765
 
 3.43e-01
 
 0.4672865596
  
 
  
 3
 
  77147070
 
  77147364
 
  PDF   STATS   ENSEMBL   UCSC 
 
  295
 
  5
 
 27862
 
 gene;island
 
 0.373978
 
 0.7050930
 
 Not queried
 
  1.7501391
 
 3.10e-01
 
 0.4672865596
  
 
  
 2
 
  74757141
 
  74757221
 
  PDF   STATS   ENSEMBL   UCSC 
 
   81
 
  5
 
 22749
 
 gene;tss;island
 
 0.002843
 
 0.0040924
 
 Not queried
 
  1.7486994
 
 3.10e-01
 
 0.4672865596
  
 
  
 2
 
 240031454
 
 240031700
 
  PDF   STATS   ENSEMBL   UCSC 
 
  247
 
  4
 
 23105
 
 gene;island
 
 0.916871
 
 0.6861846
 
 Not queried
 
  1.7484400
 
 3.43e-01
 
 0.4672865596
  
 
  
 11
 
 122850490
 
 122850741
 
  PDF   STATS   ENSEMBL   UCSC 
 
  252
 
  4
 
 6949
 
 gene;island
 
 0.097688
 
 0.2827586
 
 Not queried
 
  1.7476850
 
 3.43e-01
 
 0.4672865596
  
 
  
 10
 
    444914
 
    445215
 
  PDF   STATS   ENSEMBL   UCSC 
 
  302
 
  4
 
 4437
 
 gene;island
 
 0.766901
 
 0.8856956
 
 Not queried
 
  1.7448497
 
 3.43e-01
 
 0.4672865596
  
 
  
 11
 
   6292311
 
   6292615
 
  PDF   STATS   ENSEMBL   UCSC 
 
  305
 
  4
 
 6489
 
 gene;island
 
 0.801710
 
 0.9558057
 
 Not queried
 
  1.7444454
 
 3.43e-01
 
 0.4672865596
  
 
  
 22
 
  39323204
 
  39323648
 
  PDF   STATS   ENSEMBL   UCSC 
 
  445
 
  4
 
 27498
 
 island
 
 0.286197
 
 0.0187000
 
 Not queried
 
  1.7419352
 
 3.43e-01
 
 0.4672865596
  
 
  
 4
 
 155661691
 
 155661929
 
  PDF   STATS   ENSEMBL   UCSC 
 
  239
 
  4
 
 31384
 
 island
 
 0.803106
 
 0.9241524
 
 Not queried
 
  1.7412793
 
 3.43e-01
 
 0.4672865596
  
 
  
 7
 
 157291868
 
 157292182
 
  PDF   STATS   ENSEMBL   UCSC 
 
  315
 
  4
 
 39104
 
 island
 
 0.269117
 
 0.5820509
 
 Not queried
 
  1.7388664
 
 3.43e-01
 
 0.4672865596
  
 
  
 6
 
  31510729
 
  31511014
 
  PDF   STATS   ENSEMBL   UCSC 
 
  286
 
  4
 
 34812
 
 tss;island
 
 0.919339
 
 0.9734423
 
 Not queried
 
  1.7360702
 
 3.43e-01
 
 0.4672865596
  
 
  
 6
 
  32299502
 
  32299676
 
  PDF   STATS   ENSEMBL   UCSC 
 
  175
 
  4
 
 34102
 
 gene
 
 0.749047
 
 0.5423570
 
 Not queried
 
  1.7349988
 
 3.43e-01
 
 0.4672865596
  
 
  
 8
 
  42911287
 
  42911648
 
  PDF   STATS   ENSEMBL   UCSC 
 
  362
 
  7
 
 40263
 
 island
 
 0.002224
 
 0.0036584
 
 Not queried
 
  1.7252491
 
 3.18e-01
 
 0.4672865596
  
 
  
 7
 
  92463254
 
  92463390
 
  PDF   STATS   ENSEMBL   UCSC 
 
  137
 
  6
 
 37710
 
 tss
 
 0.005400
 
 0.0119505
 
 Not queried
 
  1.7233972
 
 3.10e-01
 
 0.4672865596
  
 
  
 6
 
  39902288
 
  39902693
 
  PDF   STATS   ENSEMBL   UCSC 
 
  406
 
  9
 
 36113
 
 island
 
 0.095115
 
 0.0148999
 
 Not queried
 
  1.7192007
 
 3.40e-01
 
 0.4672865596
  
 
  
 6
 
 169224886
 
 169225041
 
  PDF   STATS   ENSEMBL   UCSC 
 
  156
 
  4
 
 36667
 
 island
 
 0.580485
 
 0.8297044
 
 Not queried
 
  1.7164989
 
 3.43e-01
 
 0.4672865596
  
 
  
 1
 
  47489282
 
  47489543
 
  PDF   STATS   ENSEMBL   UCSC 
 
  262
 
  4
 
 514
 
 gene
 
 0.101010
 
 0.0410871
 
 Not queried
 
  1.7134251
 
 3.43e-01
 
 0.4672865596
  
 
  
 1
 
 116380946
 
 116381082
 
  PDF   STATS   ENSEMBL   UCSC 
 
  137
 
  4
 
 705
 
 gene;island
 
 0.154066
 
 0.0367831
 
 Not queried
 
  1.7130498
 
 3.43e-01
 
 0.4672865596
  
 
  
 2
 
 218712695
 
 218712871
 
  PDF   STATS   ENSEMBL   UCSC 
 
  177
 
  4
 
 23011
 
 gene
 
 0.400442
 
 0.5060889
 
 Not queried
 
  1.7040000
 
 3.43e-01
 
 0.4672865596
  
 
  
 6
 
  33389072
 
  33389172
 
  PDF   STATS   ENSEMBL   UCSC 
 
  101
 
  4
 
 34283
 
 gene;island
 
 0.659542
 
 0.8723565
 
 Not queried
 
  1.7028802
 
 3.43e-01
 
 0.4672865596
  
 
  
 8
 
 134202337
 
 134202370
 
  PDF   STATS   ENSEMBL   UCSC 
 
   34
 
  4
 
 39921
 
 tss
 
 0.638182
 
 0.8561514
 
 Not queried
 
  1.7027201
 
 3.43e-01
 
 0.4672865596
  
 
  
 2
 
  27373014
 
  27373412
 
  PDF   STATS   ENSEMBL   UCSC 
 
  399
 
  5
 
 22621
 
 gene;island
 
 0.164548
 
 0.7628913
 
 Not queried
 
  1.7019417
 
 3.10e-01
 
 0.4672865596
  
 
  
 21
 
  46890849
 
  46891093
 
  PDF   STATS   ENSEMBL   UCSC 
 
  245
 
  4
 
 26469
 
 gene;island
 
 0.438902
 
 0.6920077
 
 Not queried
 
  1.7013952
 
 3.43e-01
 
 0.4672865596
  
 
  
 8
 
  98290229
 
  98290601
 
  PDF   STATS   ENSEMBL   UCSC 
 
  373
 
  8
 
 39829
 
 tss
 
 0.174629
 
 0.0765677
 
 Not queried
 
  1.6978273
 
 3.28e-01
 
 0.4672865596
  
 
  
 1
 
 149871404
 
 149871658
 
  PDF   STATS   ENSEMBL   UCSC 
 
  255
 
  4
 
 734
 
 gene;island
 
 0.028577
 
 0.0835171
 
 Not queried
 
  1.6959648
 
 3.43e-01
 
 0.4672865596
  
 
  
 17
 
  42422247
 
  42422442
 
  PDF   STATS   ENSEMBL   UCSC 
 
  196
 
  6
 
 17409
 
 tss
 
 0.134374
 
 0.0191112
 
 Not queried
 
  1.6954059
 
 3.10e-01
 
 0.4672865596
  
 
  
 10
 
  88391658
 
  88392088
 
  PDF   STATS   ENSEMBL   UCSC 
 
  431
 
  4
 
 5907
 
 island
 
 0.333510
 
 0.1284515
 
 Not queried
 
  1.6945634
 
 3.43e-01
 
 0.4672865596
  
 
  
 7
 
 158220708
 
 158221076
 
  PDF   STATS   ENSEMBL   UCSC 
 
  369
 
  5
 
 37370
 
 gene;island
 
 0.469444
 
 0.7410748
 
 Not queried
 
  1.6939553
 
 3.10e-01
 
 0.4672865596
  
 
  
 8
 
 121824330
 
 121824929
 
  PDF   STATS   ENSEMBL   UCSC 
 
  600
 
  9
 
 39891
 
 tss
 
 0.004932
 
 0.0077509
 
 Not queried
 
  1.6937573
 
 2.97e-01
 
 0.4672865596
  
 
  
 6
 
  43211208
 
  43211491
 
  PDF   STATS   ENSEMBL   UCSC 
 
  284
 
  5
 
 36158
 
 island
 
 0.697656
 
 0.3658020
 
 Not queried
 
  1.6936139
 
 3.10e-01
 
 0.4672865596
  
 
  
 12
 
  54785171
 
  54785320
 
  PDF   STATS   ENSEMBL   UCSC 
 
  150
 
  4
 
 9763
 
 tss
 
 0.036611
 
 0.0067099
 
 Not queried
 
  1.6908842
 
 3.43e-01
 
 0.4672865596
  
 
  
 19
 
  39694993
 
  39695237
 
  PDF   STATS   ENSEMBL   UCSC 
 
  245
 
  5
 
 20767
 
 tss
 
 0.466815
 
 0.7383369
 
 Not queried
 
  1.6889302
 
 3.10e-01
 
 0.4672865596
  
 
  
 21
 
  17961737
 
  17962124
 
  PDF   STATS   ENSEMBL   UCSC 
 
  388
 
  4
 
 26388
 
 gene;tss
 
 0.441118
 
 0.7442956
 
 Not queried
 
  1.6848627
 
 3.43e-01
 
 0.4672865596
  
 
  
 7
 
  76026853
 
  76027101
 
  PDF   STATS   ENSEMBL   UCSC 
 
  249
 
  4
 
 37056
 
 gene;island
 
 0.024130
 
 0.0096519
 
 Not queried
 
  1.6841566
 
 3.43e-01
 
 0.4672865596
  
 
  
 15
 
  55880894
 
  55881246
 
  PDF   STATS   ENSEMBL   UCSC 
 
  353
 
  9
 
 14160
 
 island
 
 0.182576
 
 0.0594019
 
 Not queried
 
  1.6822378
 
 2.97e-01
 
 0.4672865596
  
 
  
 2
 
 162094696
 
 162094867
 
  PDF   STATS   ENSEMBL   UCSC 
 
  172
 
  4
 
 24819
 
 island
 
 0.012246
 
 0.0039023
 
 Not queried
 
  1.6743663
 
 3.43e-01
 
 0.4672865596
  
 
  
 7
 
 156811252
 
 156811655
 
  PDF   STATS   ENSEMBL   UCSC 
 
  404
 
  6
 
 39093
 
 island
 
 0.186143
 
 0.0227578
 
 Not queried
 
  1.6676265
 
 3.10e-01
 
 0.4672865596
  
 
  
 15
 
  45315362
 
  45315438
 
  PDF   STATS   ENSEMBL   UCSC 
 
   77
 
  4
 
 13325
 
 gene
 
 0.004452
 
 0.0030882
 
 Not queried
 
  1.6666243
 
 3.43e-01
 
 0.4672865596
  
 
  
 7
 
  19183563
 
  19183880
 
  PDF   STATS   ENSEMBL   UCSC 
 
  318
 
  4
 
 38285
 
 island
 
 0.006425
 
 0.0126180
 
 Not queried
 
  1.6645906
 
 3.43e-01
 
 0.4672865596
  
 
  
 15
 
  79103007
 
  79103221
 
  PDF   STATS   ENSEMBL   UCSC 
 
  215
 
  4
 
 13441
 
 gene;island
 
 0.025552
 
 0.0133088
 
 Not queried
 
  1.6628777
 
 3.43e-01
 
 0.4672865596
  
 
  
 13
 
  33780078
 
  33780362
 
  PDF   STATS   ENSEMBL   UCSC 
 
  285
 
  5
 
 11125
 
 gene
 
 0.014214
 
 0.0086548
 
 Not queried
 
  1.6622095
 
 3.10e-01
 
 0.4672865596
  
 
  
 6
 
  30955358
 
  30955425
 
  PDF   STATS   ENSEMBL   UCSC 
 
   68
 
  5
 
 33846
 
 gene
 
 0.046663
 
 0.1393236
 
 Not queried
 
  1.6562277
 
 3.10e-01
 
 0.4672865596
  
 
  
 18
 
  31803454
 
  31803836
 
  PDF   STATS   ENSEMBL   UCSC 
 
  383
 
  6
 
 19228
 
 tss;island
 
 0.015265
 
 0.0620067
 
 Not queried
 
  1.6546432
 
 3.10e-01
 
 0.4672865596
  
 
  
 14
 
  75642944
 
  75643321
 
  PDF   STATS   ENSEMBL   UCSC 
 
  378
 
  4
 
 12145
 
 gene
 
 0.009359
 
 0.0022373
 
 Not queried
 
  1.6513694
 
 3.43e-01
 
 0.4672865596
  
 
  
 17
 
   7348316
 
   7348399
 
  PDF   STATS   ENSEMBL   UCSC 
 
   84
 
  6
 
 17084
 
 tss
 
 0.008871
 
 0.0167352
 
 Not queried
 
  1.6497521
 
 3.10e-01
 
 0.4672865596
  
 
  
 7
 
  16460779
 
  16461244
 
  PDF   STATS   ENSEMBL   UCSC 
 
  466
 
  6
 
 38274
 
 island
 
 0.239927
 
 0.0303239
 
 Not queried
 
  1.6489802
 
 3.10e-01
 
 0.4672865596
  
 
  
 20
 
   3644097
 
   3644300
 
  PDF   STATS   ENSEMBL   UCSC 
 
  204
 
  4
 
 25566
 
 tss
 
 0.401709
 
 0.8022348
 
 Not queried
 
  1.6425562
 
 3.43e-01
 
 0.4672865596
  
 
  
 7
 
 151136940
 
 151137192
 
  PDF   STATS   ENSEMBL   UCSC 
 
  253
 
  5
 
 39040
 
 island
 
 0.112871
 
 0.0345011
 
 Not queried
 
  1.6405012
 
 3.10e-01
 
 0.4672865596
  
 
  
 8
 
 117950244
 
 117950504
 
  PDF   STATS   ENSEMBL   UCSC 
 
  261
 
  7
 
 40553
 
 island
 
 0.362681
 
 0.2056712
 
 Not queried
 
  1.6341523
 
 3.18e-01
 
 0.4672865596
  
 
  
 16
 
  30023400
 
  30023780
 
  PDF   STATS   ENSEMBL   UCSC 
 
  381
 
  4
 
 15176
 
 tss;island
 
 0.289602
 
 0.5575527
 
 Not queried
 
  1.6332832
 
 3.43e-01
 
 0.4672865596
  
 
  
 5
 
   3854465
 
   3854732
 
  PDF   STATS   ENSEMBL   UCSC 
 
  268
 
  5
 
 32670
 
 island
 
 0.548181
 
 0.8376611
 
 Not queried
 
  1.6279461
 
 3.10e-01
 
 0.4672865596
  
 
  
 1
 
   2984218
 
   2984869
 
  PDF   STATS   ENSEMBL   UCSC 
 
  652
 
 10
 
 2528
 
 island
 
 0.156891
 
 0.4039699
 
 Not queried
 
  1.6255730
 
 3.15e-01
 
 0.4672865596
  
 
  
 19
 
  52873173
 
  52873624
 
  PDF   STATS   ENSEMBL   UCSC 
 
  452
 
  4
 
 20194
 
 gene
 
 0.068617
 
 0.0367662
 
 Not queried
 
  1.6252361
 
 3.43e-01
 
 0.4672865596
  
 
  
 19
 
  49925720
 
  49926070
 
  PDF   STATS   ENSEMBL   UCSC 
 
  351
 
  4
 
 20149
 
 gene;island
 
 0.047504
 
 0.2519362
 
 Not queried
 
  1.6227332
 
 3.43e-01
 
 0.4672865596
  
 
  
 11
 
  67442126
 
  67442408
 
  PDF   STATS   ENSEMBL   UCSC 
 
  283
 
  4
 
 7501
 
 tss
 
 0.268444
 
 0.4490576
 
 Not queried
 
  1.6226352
 
 3.43e-01
 
 0.4672865596
  
 
  
 6
 
  39760416
 
  39760833
 
  PDF   STATS   ENSEMBL   UCSC 
 
  418
 
  5
 
 36112
 
 island
 
 0.100659
 
 0.0361678
 
 Not queried
 
  1.6190526
 
 3.10e-01
 
 0.4672865596
  
 
  
 20
 
  61435829
 
  61436024
 
  PDF   STATS   ENSEMBL   UCSC 
 
  196
 
  6
 
 25881
 
 tss;island
 
 0.001770
 
 0.0079167
 
 Not queried
 
  1.6190366
 
 3.10e-01
 
 0.4672865596
  
 
  
 16
 
   2264855
 
   2264884
 
  PDF   STATS   ENSEMBL   UCSC 
 
   30
 
  4
 
 15022
 
 tss;island
 
 0.005456
 
 0.0046416
 
 Not queried
 
  1.6189700
 
 3.43e-01
 
 0.4672865596
  
 
  
 1
 
  86888585
 
  86888728
 
  PDF   STATS   ENSEMBL   UCSC 
 
  144
 
  4
 
 1727
 
 tss
 
 0.429740
 
 0.6624540
 
 Not queried
 
  1.6138246
 
 3.43e-01
 
 0.4672865596
  
 
  
 4
 
 141264393
 
 141264721
 
  PDF   STATS   ENSEMBL   UCSC 
 
  329
 
  5
 
 30136
 
 gene
 
 0.004265
 
 0.0525323
 
 Not queried
 
  1.6102036
 
 3.10e-01
 
 0.4672865596
  
 
  
 10
 
 105239085
 
 105239205
 
  PDF   STATS   ENSEMBL   UCSC 
 
  121
 
  4
 
 5339
 
 tss
 
 0.762195
 
 0.8893549
 
 Not queried
 
  1.6059848
 
 3.43e-01
 
 0.4672865596
  
 
  
 6
 
  29797211
 
  29797420
 
  PDF   STATS   ENSEMBL   UCSC 
 
  210
 
  4
 
 33698
 
 gene;island
 
 0.342904
 
 0.5516839
 
 Not queried
 
  1.6019483
 
 3.43e-01
 
 0.4672865596
  
 
  
 2
 
 241807708
 
 241808113
 
  PDF   STATS   ENSEMBL   UCSC 
 
  406
 
  6
 
 23982
 
 tss
 
 0.239073
 
 0.3317992
 
 Not queried
 
  1.6008690
 
 3.10e-01
 
 0.4672865596
  
 
  
 16
 
  55542668
 
  55543166
 
  PDF   STATS   ENSEMBL   UCSC 
 
  499
 
  9
 
 15936
 
 island
 
 0.074553
 
 0.5686716
 
 Not queried
 
  1.5997556
 
 2.97e-01
 
 0.4672865596
  
 
  
 16
 
   1598866
 
   1599150
 
  PDF   STATS   ENSEMBL   UCSC 
 
  285
 
  4
 
 14576
 
 gene;island
 
 0.596112
 
 0.7375993
 
 Not queried
 
  1.5977860
 
 3.43e-01
 
 0.4672865596
  
 
  
 3
 
  45066580
 
  45066971
 
  PDF   STATS   ENSEMBL   UCSC 
 
  392
 
  4
 
 28266
 
 tss
 
 0.896176
 
 0.7056556
 
 Not queried
 
  1.5944662
 
 3.43e-01
 
 0.4672865596
  
 
  
 16
 
  20360206
 
  20360497
 
  PDF   STATS   ENSEMBL   UCSC 
 
  292
 
  4
 
 14683
 
 gene;island
 
 0.299516
 
 0.4492679
 
 Not queried
 
  1.5943819
 
 3.43e-01
 
 0.4672865596
  
 
  
 10
 
 123353500
 
 123353956
 
  PDF   STATS   ENSEMBL   UCSC 
 
  457
 
  8
 
 5404
 
 tss
 
 0.351142
 
 0.5732475
 
 Not queried
 
  1.5934314
 
 2.79e-01
 
 0.4672865596
  
 
  
 16
 
    103423
 
    103592
 
  PDF   STATS   ENSEMBL   UCSC 
 
  170
 
  4
 
 14521
 
 gene
 
 0.111802
 
 0.0308726
 
 Not queried
 
  1.5930529
 
 3.43e-01
 
 0.4672865596
  
 
  
 6
 
  43230558
 
  43231063
 
  PDF   STATS   ENSEMBL   UCSC 
 
  506
 
  5
 
 34346
 
 gene;island
 
 0.740533
 
 0.8945480
 
 Not queried
 
  1.5915250
 
 3.10e-01
 
 0.4672865596
  
 
  
 20
 
  43150917
 
  43151161
 
  PDF   STATS   ENSEMBL   UCSC 
 
  245
 
  4
 
 25761
 
 tss;island
 
 0.001545
 
 0.0007211
 
 Not queried
 
  1.5907780
 
 3.43e-01
 
 0.4672865596
  
 
  
 15
 
  42289618
 
  42289939
 
  PDF   STATS   ENSEMBL   UCSC 
 
  322
 
  4
 
 13306
 
 gene
 
 0.545830
 
 0.7577666
 
 Not queried
 
  1.5904429
 
 3.43e-01
 
 0.4672865596
  
 
  
 17
 
  43503394
 
  43503638
 
  PDF   STATS   ENSEMBL   UCSC 
 
  245
 
  4
 
 17430
 
 tss;island
 
 0.248393
 
 0.5929308
 
 Not queried
 
  1.5890214
 
 3.43e-01
 
 0.4672865596
  
 
  
 1
 
  33722125
 
  33722135
 
  PDF   STATS   ENSEMBL   UCSC 
 
   11
 
  4
 
 1472
 
 tss;island
 
 0.007664
 
 0.0042798
 
 Not queried
 
  1.5887738
 
 3.43e-01
 
 0.4672865596
  
 
  
 4
 
  40752692
 
  40752947
 
  PDF   STATS   ENSEMBL   UCSC 
 
  256
 
  4
 
 29988
 
 gene;island
 
 0.424982
 
 0.1691628
 
 Not queried
 
  1.5868740
 
 3.43e-01
 
 0.4672865596
  
 
  
 12
 
 121454133
 
 121454451
 
  PDF   STATS   ENSEMBL   UCSC 
 
  319
 
  8
 
 10905
 
 island
 
 0.004157
 
 0.0018619
 
 Not queried
 
  1.5853581
 
 3.28e-01
 
 0.4672865596
  
 
  
 13
 
 110918331
 
 110918683
 
  PDF   STATS   ENSEMBL   UCSC 
 
  353
 
  4
 
 11227
 
 gene;island
 
 0.520169
 
 0.8565828
 
 Not queried
 
  1.5833090
 
 3.43e-01
 
 0.4672865596
  
 
  
 7
 
  99067116
 
  99067368
 
  PDF   STATS   ENSEMBL   UCSC 
 
  253
 
  5
 
 38733
 
 island
 
 0.305976
 
 0.4635624
 
 Not queried
 
  1.5830792
 
 3.10e-01
 
 0.4672865596
  
 
  
 14
 
 105044984
 
 105045347
 
  PDF   STATS   ENSEMBL   UCSC 
 
  364
 
  4
 
 12658
 
 tss;island
 
 0.233221
 
 0.3713450
 
 Not queried
 
  1.5829222
 
 3.43e-01
 
 0.4672865596
  
 
  
 1
 
 156889826
 
 156890244
 
  PDF   STATS   ENSEMBL   UCSC 
 
  419
 
  5
 
 2026
 
 tss;island
 
 0.405767
 
 0.0745629
 
 Not queried
 
  1.5827580
 
 3.10e-01
 
 0.4672865596
  
 
  
 20
 
    709016
 
    709392
 
  PDF   STATS   ENSEMBL   UCSC 
 
  377
 
  4
 
 25928
 
 island
 
 0.125488
 
 0.0311598
 
 Not queried
 
  1.5817703
 
 3.43e-01
 
 0.4672865596
  
 
  
 3
 
  49756301
 
  49757476
 
  PDF   STATS   ENSEMBL   UCSC 
 
 1176
 
 12
 
 27788
 
 gene;island
 
 0.140171
 
 0.3716208
 
 Not queried
 
  1.5799488
 
 2.91e-01
 
 0.4672865596
  
 
  
 10
 
 111216646
 
 111216962
 
  PDF   STATS   ENSEMBL   UCSC 
 
  317
 
  5
 
 6122
 
 island
 
 0.236717
 
 0.0671713
 
 Not queried
 
  1.5785822
 
 3.10e-01
 
 0.4672865596
  
 
  
 19
 
   7852051
 
   7852238
 
  PDF   STATS   ENSEMBL   UCSC 
 
  188
 
  6
 
 20407
 
 tss;island
 
 0.337932
 
 0.6912196
 
 Not queried
 
  1.5785484
 
 3.10e-01
 
 0.4672865596
  
 
  
 17
 
  19651881
 
  19652056
 
  PDF   STATS   ENSEMBL   UCSC 
 
  176
 
  4
 
 17195
 
 tss;island
 
 0.421379
 
 0.2177605
 
 Not queried
 
  1.5764019
 
 3.43e-01
 
 0.4672865596
  
 
  
 6
 
 170615736
 
 170615780
 
  PDF   STATS   ENSEMBL   UCSC 
 
   45
 
  5
 
 35356
 
 tss
 
 0.001525
 
 0.0030563
 
 Not queried
 
  1.5728130
 
 3.10e-01
 
 0.4672865596
  
 
  
 16
 
  89002930
 
  89003235
 
  PDF   STATS   ENSEMBL   UCSC 
 
  306
 
  4
 
 14925
 
 gene;island
 
 0.049320
 
 0.1657764
 
 Not queried
 
  1.5692414
 
 3.43e-01
 
 0.4672865596
  
 
  
 2
 
 223290012
 
 223290515
 
  PDF   STATS   ENSEMBL   UCSC 
 
  504
 
  4
 
 23053
 
 gene;island
 
 0.005273
 
 0.0147836
 
 Not queried
 
  1.5657432
 
 3.43e-01
 
 0.4672865596
  
 
  
 20
 
  45523098
 
  45523187
 
  PDF   STATS   ENSEMBL   UCSC 
 
   90
 
  4
 
 25798
 
 tss
 
 0.005030
 
 0.0203687
 
 Not queried
 
  1.5643003
 
 3.43e-01
 
 0.4672865596
  
 
  
 13
 
 113140125
 
 113140323
 
  PDF   STATS   ENSEMBL   UCSC 
 
  199
 
  4
 
 11247
 
 gene;island
 
 0.858169
 
 0.9404600
 
 Not queried
 
  1.5639920
 
 3.43e-01
 
 0.4672865596
  
 
  
 1
 
  44063475
 
  44063744
 
  PDF   STATS   ENSEMBL   UCSC 
 
  270
 
  4
 
 482
 
 gene;island
 
 0.624604
 
 0.9155978
 
 Not queried
 
  1.5626158
 
 3.43e-01
 
 0.4672865596
  
 
  
 8
 
   2046752
 
   2047010
 
  PDF   STATS   ENSEMBL   UCSC 
 
  259
 
  4
 
 39188
 
 gene;island
 
 0.668643
 
 0.8512175
 
 Not queried
 
  1.5612363
 
 3.43e-01
 
 0.4672865596
  
 
  
 19
 
  19322527
 
  19322778
 
  PDF   STATS   ENSEMBL   UCSC 
 
  252
 
  6
 
 20639
 
 tss
 
 0.008801
 
 0.0236959
 
 Not queried
 
  1.5610312
 
 3.10e-01
 
 0.4672865596
  
 
  
 4
 
   1052372
 
   1052717
 
  PDF   STATS   ENSEMBL   UCSC 
 
  346
 
  4
 
 30766
 
 island
 
 0.391802
 
 0.5614881
 
 Not queried
 
  1.5604873
 
 3.43e-01
 
 0.4672865596
  
 
  
 7
 
 134855640
 
 134855774
 
  PDF   STATS   ENSEMBL   UCSC 
 
  135
 
  8
 
 37916
 
 tss
 
 0.001306
 
 0.0061692
 
 Not queried
 
  1.5587149
 
 3.28e-01
 
 0.4672865596
  
 
  
 7
 
  50800837
 
  50801423
 
  PDF   STATS   ENSEMBL   UCSC 
 
  587
 
  8
 
 37014
 
 gene;tss
 
 0.458144
 
 0.7675301
 
 Not queried
 
  1.5565051
 
 2.79e-01
 
 0.4672865596
  
 
  
 1
 
 223888869
 
 223889141
 
  PDF   STATS   ENSEMBL   UCSC 
 
  273
 
  4
 
 2273
 
 tss
 
 0.542113
 
 0.7479012
 
 Not queried
 
  1.5551039
 
 3.43e-01
 
 0.4672865596
  
 
  
 12
 
   7023195
 
   7023527
 
  PDF   STATS   ENSEMBL   UCSC 
 
  333
 
  9
 
 9534
 
 tss;island
 
 0.407433
 
 0.0179502
 
 Not queried
 
  1.5516075
 
 2.97e-01
 
 0.4672865596
  
 
  
 6
 
  32025416
 
  32025657
 
  PDF   STATS   ENSEMBL   UCSC 
 
  242
 
  4
 
 34021
 
 gene
 
 0.113787
 
 0.3286048
 
 Not queried
 
  1.5495138
 
 3.43e-01
 
 0.4672865596
  
 
  
 12
 
  66582582
 
  66582805
 
  PDF   STATS   ENSEMBL   UCSC 
 
  224
 
  4
 
 9848
 
 tss;island
 
 0.331261
 
 0.0829447
 
 Not queried
 
  1.5466737
 
 3.43e-01
 
 0.4672865596
  
 
  
 8
 
 140716970
 
 140717372
 
  PDF   STATS   ENSEMBL   UCSC 
 
  403
 
  6
 
 40606
 
 island
 
 0.046790
 
 0.3058538
 
 Not queried
 
  1.5464029
 
 3.10e-01
 
 0.4672865596
  
 
  
 12
 
  51236582
 
  51236768
 
  PDF   STATS   ENSEMBL   UCSC 
 
  187
 
  6
 
 10418
 
 island
 
 0.526950
 
 0.8258437
 
 Not queried
 
  1.5452259
 
 3.10e-01
 
 0.4672865596
  
 
  
 8
 
  48100868
 
  48100957
 
  PDF   STATS   ENSEMBL   UCSC 
 
   90
 
  4
 
 40270
 
 island
 
 0.264169
 
 0.6908387
 
 Not queried
 
  1.5434232
 
 3.43e-01
 
 0.4672865596
  
 
  
 4
 
  89299733
 
  89300321
 
  PDF   STATS   ENSEMBL   UCSC 
 
  589
 
  7
 
 31173
 
 island
 
 0.009337
 
 0.0247208
 
 Not queried
 
  1.5419242
 
 3.18e-01
 
 0.4672865596
  
 
  
 2
 
  79739863
 
  79739940
 
  PDF   STATS   ENSEMBL   UCSC 
 
   78
 
  4
 
 23472
 
 tss;island
 
 0.032239
 
 0.0943497
 
 Not queried
 
  1.5414332
 
 3.43e-01
 
 0.4672865596
  
 
  
 12
 
  48577720
 
  48578223
 
  PDF   STATS   ENSEMBL   UCSC 
 
  504
 
  4
 
 9111
 
 gene;island
 
 0.073045
 
 0.0153400
 
 Not queried
 
  1.5405428
 
 3.43e-01
 
 0.4672865596
  
 
  
 14
 
  90526639
 
  90526912
 
  PDF   STATS   ENSEMBL   UCSC 
 
  274
 
  4
 
 12551
 
 tss;island
 
 0.148133
 
 0.0178751
 
 Not queried
 
  1.5404732
 
 3.43e-01
 
 0.4672865596
  
 
  
 4
 
   5890358
 
   5890430
 
  PDF   STATS   ENSEMBL   UCSC 
 
   73
 
  6
 
 30277
 
 tss
 
 0.022719
 
 0.0096651
 
 Not queried
 
  1.5355804
 
 3.10e-01
 
 0.4672865596
  
 
  
 15
 
  41803428
 
  41803850
 
  PDF   STATS   ENSEMBL   UCSC 
 
  423
 
  4
 
 13304
 
 gene;island
 
 0.298159
 
 0.0486081
 
 Not queried
 
  1.5354255
 
 3.43e-01
 
 0.4672865596
  
 
  
 19
 
  35739448
 
  35739762
 
  PDF   STATS   ENSEMBL   UCSC 
 
  315
 
  5
 
 21900
 
 island
 
 0.007669
 
 0.0095460
 
 Not queried
 
  1.5320049
 
 3.10e-01
 
 0.4672865596
  
 
  
 10
 
  27547536
 
  27547965
 
  PDF   STATS   ENSEMBL   UCSC 
 
  430
 
  6
 
 5628
 
 island
 
 0.049606
 
 0.0373044
 
 Not queried
 
  1.5300959
 
 3.10e-01
 
 0.4672865596
  
 
  
 10
 
  29827500
 
  29827742
 
  PDF   STATS   ENSEMBL   UCSC 
 
  243
 
  7
 
 4546
 
 gene
 
 0.921847
 
 0.9403986
 
 Not queried
 
  1.5277349
 
 3.18e-01
 
 0.4672865596
  
 
  
 4
 
 140374776
 
 140375014
 
  PDF   STATS   ENSEMBL   UCSC 
 
  239
 
  4
 
 31318
 
 island
 
 0.011884
 
 0.0087259
 
 Not queried
 
  1.5270110
 
 3.43e-01
 
 0.4672865596
  
 
  
 17
 
  40428524
 
  40428550
 
  PDF   STATS   ENSEMBL   UCSC 
 
   27
 
  4
 
 17359
 
 tss;island
 
 0.005994
 
 0.0179351
 
 Not queried
 
  1.5211908
 
 3.43e-01
 
 0.4672865596
  
 
  
 10
 
   5541568
 
   5541823
 
  PDF   STATS   ENSEMBL   UCSC 
 
  256
 
  4
 
 4925
 
 tss
 
 0.201106
 
 0.4613629
 
 Not queried
 
  1.5209465
 
 3.43e-01
 
 0.4672865596
  
 
  
 16
 
   1128689
 
   1128865
 
  PDF   STATS   ENSEMBL   UCSC 
 
  177
 
  5
 
 14987
 
 tss;island
 
 0.734262
 
 0.8492595
 
 Not queried
 
  1.5190507
 
 3.10e-01
 
 0.4672865596
  
 
  
 7
 
 152591099
 
 152592038
 
  PDF   STATS   ENSEMBL   UCSC 
 
  940
 
 10
 
 39052
 
 island
 
 0.275002
 
 0.5713940
 
 Not queried
 
  1.5175583
 
 3.15e-01
 
 0.4672865596
  
 
  
 14
 
  73925368
 
  73925622
 
  PDF   STATS   ENSEMBL   UCSC 
 
  255
 
  4
 
 12496
 
 tss
 
 0.021521
 
 0.0188485
 
 Not queried
 
  1.5171126
 
 3.43e-01
 
 0.4672865596
  
 
  
 13
 
  95357638
 
  95357832
 
  PDF   STATS   ENSEMBL   UCSC 
 
  195
 
  4
 
 11821
 
 island
 
 0.034597
 
 0.0630413
 
 Not queried
 
  1.5135923
 
 3.43e-01
 
 0.4672865596
  
 
  
 11
 
    788406
 
    788714
 
  PDF   STATS   ENSEMBL   UCSC 
 
  309
 
  4
 
 6390
 
 gene;island
 
 0.871941
 
 0.9532300
 
 Not queried
 
  1.5105389
 
 3.43e-01
 
 0.4672865596
  
 
  
 6
 
  39760416
 
  39760776
 
  PDF   STATS   ENSEMBL   UCSC 
 
  361
 
  4
 
 34959
 
 tss
 
 0.095558
 
 0.0234253
 
 Not queried
 
  1.5093042
 
 3.43e-01
 
 0.4672865596
  
 
  
 8
 
  91803657
 
  91803805
 
  PDF   STATS   ENSEMBL   UCSC 
 
  149
 
  5
 
 39807
 
 tss;island
 
 0.226028
 
 0.3880801
 
 Not queried
 
  1.5082600
 
 3.10e-01
 
 0.4672865596
  
 
  
 4
 
 183063397
 
 183063637
 
  PDF   STATS   ENSEMBL   UCSC 
 
  241
 
  4
 
 30190
 
 gene;island
 
 0.038671
 
 0.0066590
 
 Not queried
 
  1.5062852
 
 3.43e-01
 
 0.4672865596
  
 
  
 16
 
  84256036
 
  84256337
 
  PDF   STATS   ENSEMBL   UCSC 
 
  302
 
  4
 
 14866
 
 gene;island
 
 0.336196
 
 0.5437979
 
 Not queried
 
  1.5053429
 
 3.43e-01
 
 0.4672865596
  
 
  
 1
 
 154965419
 
 154965572
 
  PDF   STATS   ENSEMBL   UCSC 
 
  154
 
  4
 
 787
 
 gene;tss
 
 0.718726
 
 0.9572602
 
 Not queried
 
  1.5009085
 
 3.43e-01
 
 0.4672865596
  
 
  
 6
 
 100911687
 
 100911746
 
  PDF   STATS   ENSEMBL   UCSC 
 
   60
 
  6
 
 35139
 
 tss
 
 0.140058
 
 0.3819300
 
 Not queried
 
  1.4994359
 
 3.10e-01
 
 0.4672865596
  
 
  
 14
 
  65347138
 
  65347518
 
  PDF   STATS   ENSEMBL   UCSC 
 
  381
 
  4
 
 12943
 
 island
 
 0.042436
 
 0.0236433
 
 Not queried
 
  1.4990313
 
 3.43e-01
 
 0.4672865596
  
 
  
 19
 
  47925344
 
  47925618
 
  PDF   STATS   ENSEMBL   UCSC 
 
  275
 
  5
 
 22194
 
 island
 
 0.013596
 
 0.0086676
 
 Not queried
 
  1.4985209
 
 3.10e-01
 
 0.4672865596
  
 
  
 15
 
  76004985
 
  76005171
 
  PDF   STATS   ENSEMBL   UCSC 
 
  187
 
  4
 
 13432
 
 gene
 
 0.218176
 
 0.3204939
 
 Not queried
 
  1.4939917
 
 3.43e-01
 
 0.4672865596
  
 
  
 13
 
  20710941
 
  20711179
 
  PDF   STATS   ENSEMBL   UCSC 
 
  239
 
  4
 
 11566
 
 island
 
 0.051780
 
 0.0110849
 
 Not queried
 
  1.4928495
 
 3.43e-01
 
 0.4672865596
  
 
  
 2
 
  44395749
 
  44395983
 
  PDF   STATS   ENSEMBL   UCSC 
 
  235
 
  6
 
 23331
 
 tss
 
 0.018829
 
 0.0091933
 
 Not queried
 
  1.4926776
 
 3.10e-01
 
 0.4672865596
  
 
  
 16
 
   1088364
 
   1088734
 
  PDF   STATS   ENSEMBL   UCSC 
 
  371
 
  4
 
 15510
 
 island
 
 0.488889
 
 0.8143758
 
 Not queried
 
  1.4902842
 
 3.43e-01
 
 0.4672865596
  
 
  
 6
 
  27258460
 
  27258767
 
  PDF   STATS   ENSEMBL   UCSC 
 
  308
 
  6
 
 35552
 
 island
 
 0.580771
 
 0.2717177
 
 Not queried
 
  1.4871986
 
 3.10e-01
 
 0.4672865596
  
 
  
 10
 
   7661611
 
   7661796
 
  PDF   STATS   ENSEMBL   UCSC 
 
  186
 
  5
 
 4484
 
 gene
 
 0.627117
 
 0.8726270
 
 Not queried
 
  1.4859233
 
 3.10e-01
 
 0.4672865596
  
 
  
 2
 
 201171255
 
 201171347
 
  PDF   STATS   ENSEMBL   UCSC 
 
   93
 
  5
 
 23808
 
 tss
 
 0.015146
 
 0.0008757
 
 Not queried
 
  1.4843873
 
 3.10e-01
 
 0.4672865596
  
 
  
 3
 
  26664115
 
  26664404
 
  PDF   STATS   ENSEMBL   UCSC 
 
  290
 
  5
 
 28945
 
 island
 
 0.034622
 
 0.0506999
 
 Not queried
 
  1.4828942
 
 3.10e-01
 
 0.4672865596
  
 
  
 21
 
  34442160
 
  34442674
 
  PDF   STATS   ENSEMBL   UCSC 
 
  515
 
 10
 
 26643
 
 island
 
 0.024228
 
 0.0129998
 
 Not queried
 
  1.4825960
 
 3.15e-01
 
 0.4672865596
  
 
  
 18
 
  10588980
 
  10589360
 
  PDF   STATS   ENSEMBL   UCSC 
 
  381
 
  5
 
 19392
 
 island
 
 0.900586
 
 0.9509761
 
 Not queried
 
  1.4825304
 
 3.10e-01
 
 0.4672865596
  
 
  
 7
 
 157444235
 
 157444501
 
  PDF   STATS   ENSEMBL   UCSC 
 
  267
 
  5
 
 37334
 
 gene;island
 
 0.464058
 
 0.7595352
 
 Not queried
 
  1.4823385
 
 3.10e-01
 
 0.4672865596
  
 
  
 5
 
 154091976
 
 154092426
 
  PDF   STATS   ENSEMBL   UCSC 
 
  451
 
  6
 
 32450
 
 tss
 
 0.829944
 
 0.9232076
 
 Not queried
 
  1.4821224
 
 3.10e-01
 
 0.4672865596
  
 
  
 6
 
  29274521
 
  29275337
 
  PDF   STATS   ENSEMBL   UCSC 
 
  817
 
 12
 
 33651
 
 gene
 
 0.291221
 
 0.3615302
 
 Not queried
 
  1.4769661
 
 3.19e-01
 
 0.4672865596
  
 
  
 21
 
  46359575
 
  46359716
 
  PDF   STATS   ENSEMBL   UCSC 
 
  142
 
  4
 
 26461
 
 gene;tss;island
 
 0.001674
 
 0.0044663
 
 Not queried
 
  1.4733060
 
 3.43e-01
 
 0.4672865596
  
 
  
 19
 
   5822318
 
   5822630
 
  PDF   STATS   ENSEMBL   UCSC 
 
  313
 
  4
 
 20383
 
 tss;island
 
 0.261361
 
 0.6348993
 
 Not queried
 
  1.4706879
 
 3.43e-01
 
 0.4672865596
  
 
  
 6
 
  52009019
 
  52009126
 
  PDF   STATS   ENSEMBL   UCSC 
 
  108
 
  4
 
 35029
 
 tss
 
 0.406063
 
 0.7265184
 
 Not queried
 
  1.4682423
 
 3.43e-01
 
 0.4672865596
  
 
  
 15
 
  43531947
 
  43532243
 
  PDF   STATS   ENSEMBL   UCSC 
 
  297
 
  4
 
 13314
 
 gene
 
 0.172636
 
 0.5344091
 
 Not queried
 
  1.4682199
 
 3.43e-01
 
 0.4672865596
  
 
  
 5
 
 133451237
 
 133451474
 
  PDF   STATS   ENSEMBL   UCSC 
 
  238
 
  7
 
 32287
 
 tss
 
 0.012380
 
 0.0225059
 
 Not queried
 
  1.4652987
 
 3.18e-01
 
 0.4672865596
  
 
  
 22
 
  36806414
 
  36806655
 
  PDF   STATS   ENSEMBL   UCSC 
 
  242
 
  4
 
 27452
 
 island
 
 0.546771
 
 0.3053379
 
 Not queried
 
  1.4647924
 
 3.43e-01
 
 0.4672865596
  
 
  
 2
 
    906044
 
    906427
 
  PDF   STATS   ENSEMBL   UCSC 
 
  384
 
  4
 
 23156
 
 tss
 
 0.685553
 
 0.8553213
 
 Not queried
 
  1.4642091
 
 3.43e-01
 
 0.4672865596
  
 
  
 17
 
  33701321
 
  33701529
 
  PDF   STATS   ENSEMBL   UCSC 
 
  209
 
  4
 
 17268
 
 tss;island
 
 0.015628
 
 0.0339934
 
 Not queried
 
  1.4620489
 
 3.43e-01
 
 0.4672865596
  
 
  
 17
 
   7486996
 
   7487593
 
  PDF   STATS   ENSEMBL   UCSC 
 
  598
 
  5
 
 16403
 
 gene
 
 0.005903
 
 0.0028136
 
 Not queried
 
  1.4612520
 
 3.10e-01
 
 0.4672865596
  
 
  
 7
 
  16793463
 
  16793736
 
  PDF   STATS   ENSEMBL   UCSC 
 
  274
 
  4
 
 36906
 
 gene
 
 0.089252
 
 0.0361802
 
 Not queried
 
  1.4592850
 
 3.43e-01
 
 0.4672865596
  
 
  
 1
 
 155904084
 
 155904158
 
  PDF   STATS   ENSEMBL   UCSC 
 
   75
 
  4
 
 809
 
 gene
 
 0.002366
 
 0.0075573
 
 Not queried
 
  1.4582020
 
 3.43e-01
 
 0.4672865596
  
 
  
 6
 
 125475016
 
 125475385
 
  PDF   STATS   ENSEMBL   UCSC 
 
  370
 
  8
 
 34465
 
 gene
 
 0.011325
 
 0.0207548
 
 Not queried
 
  1.4562927
 
 3.28e-01
 
 0.4672865596
  
 
  
 6
 
 108487063
 
 108487183
 
  PDF   STATS   ENSEMBL   UCSC 
 
  121
 
  6
 
 35158
 
 tss;island
 
 0.088953
 
 0.0326959
 
 Not queried
 
  1.4553839
 
 3.10e-01
 
 0.4672865596
  
 
  
 13
 
  91826885
 
  91827042
 
  PDF   STATS   ENSEMBL   UCSC 
 
  158
 
  4
 
 11812
 
 island
 
 0.669580
 
 0.8938454
 
 Not queried
 
  1.4549132
 
 3.43e-01
 
 0.4672865596
  
 
  
 7
 
  38670804
 
  38671001
 
  PDF   STATS   ENSEMBL   UCSC 
 
  198
 
  6
 
 36972
 
 gene;island
 
 0.043646
 
 0.0253980
 
 Not queried
 
  1.4503065
 
 3.10e-01
 
 0.4672865596
  
 
  
 1
 
 117487269
 
 117487644
 
  PDF   STATS   ENSEMBL   UCSC 
 
  376
 
  4
 
 710
 
 gene;island
 
 0.949963
 
 0.7389178
 
 Not queried
 
  1.4490246
 
 3.43e-01
 
 0.4672865596
  
 
  
 12
 
 101603835
 
 101604258
 
  PDF   STATS   ENSEMBL   UCSC 
 
  424
 
  8
 
 10717
 
 island
 
 0.128156
 
 0.0466159
 
 Not queried
 
  1.4485592
 
 2.79e-01
 
 0.4672865596
  
 
  
 22
 
  20004356
 
  20004382
 
  PDF   STATS   ENSEMBL   UCSC 
 
   27
 
  6
 
 26971
 
 tss;island
 
 0.003893
 
 0.0022209
 
 Not queried
 
  1.4464327
 
 3.10e-01
 
 0.4672865596
  
 
  
 7
 
  50849639
 
  50849931
 
  PDF   STATS   ENSEMBL   UCSC 
 
  293
 
  4
 
 37015
 
 gene;island
 
 0.764613
 
 0.4614707
 
 Not queried
 
  1.4452055
 
 3.43e-01
 
 0.4672865596
  
 
  
 14
 
  76452112
 
  76452459
 
  PDF   STATS   ENSEMBL   UCSC 
 
  348
 
  4
 
 12148
 
 gene
 
 0.009232
 
 0.0040705
 
 Not queried
 
  1.4431189
 
 3.43e-01
 
 0.4672865596
  
 
  
 7
 
  75795931
 
  75796235
 
  PDF   STATS   ENSEMBL   UCSC 
 
  305
 
  4
 
 38594
 
 island
 
 0.008364
 
 0.0474171
 
 Not queried
 
  1.4429951
 
 3.43e-01
 
 0.4672865596
  
 
  
 6
 
  32301073
 
  32301513
 
  PDF   STATS   ENSEMBL   UCSC 
 
  441
 
  4
 
 34103
 
 gene
 
 0.657437
 
 0.8325990
 
 Not queried
 
  1.4406450
 
 3.43e-01
 
 0.4672865596
  
 
  
 6
 
  32039796
 
  32039999
 
  PDF   STATS   ENSEMBL   UCSC 
 
  204
 
  7
 
 34031
 
 gene
 
 0.317170
 
 0.4462107
 
 Not queried
 
  1.4395665
 
 3.18e-01
 
 0.4672865596
  
 
  
 5
 
 175223225
 
 175223497
 
  PDF   STATS   ENSEMBL   UCSC 
 
  273
 
  4
 
 32508
 
 tss
 
 0.171729
 
 0.0105323
 
 Not queried
 
  1.4373486
 
 3.43e-01
 
 0.4672865596
  
 
  
 8
 
  17013409
 
  17013756
 
  PDF   STATS   ENSEMBL   UCSC 
 
  348
 
  4
 
 39564
 
 tss;island
 
 0.013687
 
 0.0064362
 
 Not queried
 
  1.4369379
 
 3.43e-01
 
 0.4672865596
  
 
  
 3
 
 128294970
 
 128295141
 
  PDF   STATS   ENSEMBL   UCSC 
 
  172
 
  6
 
 28570
 
 tss
 
 0.278478
 
 0.4425791
 
 Not queried
 
  1.4343506
 
 3.10e-01
 
 0.4672865596
  
 
  
 14
 
  32670315
 
  32670656
 
  PDF   STATS   ENSEMBL   UCSC 
 
  342
 
  4
 
 12770
 
 island
 
 0.001605
 
 0.0024774
 
 Not queried
 
  1.4342783
 
 3.43e-01
 
 0.4672865596
  
 
  
 2
 
 136875897
 
 136876188
 
  PDF   STATS   ENSEMBL   UCSC 
 
  292
 
  7
 
 23649
 
 tss;island
 
 0.003498
 
 0.0029000
 
 Not queried
 
  1.4336952
 
 3.18e-01
 
 0.4672865596
  
 
  
 15
 
  29407792
 
  29408145
 
  PDF   STATS   ENSEMBL   UCSC 
 
  354
 
  4
 
 13268
 
 gene;island
 
 0.067447
 
 0.3652578
 
 Not queried
 
  1.4307458
 
 3.43e-01
 
 0.4672865596
  
 
  
 18
 
  71958904
 
  71959170
 
  PDF   STATS   ENSEMBL   UCSC 
 
  267
 
  4
 
 19126
 
 gene
 
 0.008520
 
 0.0920562
 
 Not queried
 
  1.4292477
 
 3.43e-01
 
 0.4672865596
  
 
  
 3
 
 192444985
 
 192445380
 
  PDF   STATS   ENSEMBL   UCSC 
 
  396
 
  5
 
 28087
 
 gene
 
 0.408919
 
 0.0812078
 
 Not queried
 
  1.4288532
 
 3.10e-01
 
 0.4672865596
  
 
  
 11
 
 115530932
 
 115531299
 
  PDF   STATS   ENSEMBL   UCSC 
 
  368
 
  5
 
 8830
 
 island
 
 0.027817
 
 0.0079496
 
 Not queried
 
  1.4254678
 
 3.10e-01
 
 0.4672865596
  
 
  
 2
 
  27341795
 
  27341901
 
  PDF   STATS   ENSEMBL   UCSC 
 
  107
 
  4
 
 22620
 
 gene;tss;island
 
 0.027516
 
 0.0105474
 
 Not queried
 
  1.4235612
 
 3.43e-01
 
 0.4672865596
  
 
  
 19
 
  53141620
 
  53141793
 
  PDF   STATS   ENSEMBL   UCSC 
 
  174
 
  4
 
 20198
 
 gene;island
 
 0.050262
 
 0.0254702
 
 Not queried
 
  1.4187733
 
 3.43e-01
 
 0.4672865596
  
 
  
 7
 
  22705331
 
  22705607
 
  PDF   STATS   ENSEMBL   UCSC 
 
  277
 
  4
 
 38301
 
 island
 
 0.768806
 
 0.9783582
 
 Not queried
 
  1.4164719
 
 3.43e-01
 
 0.4672865596
  
 
  
 4
 
 159131324
 
 159131785
 
  PDF   STATS   ENSEMBL   UCSC 
 
  462
 
  7
 
 31396
 
 island
 
 0.179342
 
 0.0223667
 
 Not queried
 
  1.4147013
 
 3.18e-01
 
 0.4672865596
  
 
  
 5
 
 168271613
 
 168272051
 
  PDF   STATS   ENSEMBL   UCSC 
 
  439
 
  7
 
 31887
 
 gene
 
 0.910435
 
 0.9707438
 
 Not queried
 
  1.4143263
 
 3.18e-01
 
 0.4672865596
  
 
  
 19
 
  48216390
 
  48216502
 
  PDF   STATS   ENSEMBL   UCSC 
 
  113
 
  5
 
 20908
 
 tss
 
 0.007241
 
 0.0380320
 
 Not queried
 
  1.4137955
 
 3.10e-01
 
 0.4672865596
  
 
  
 6
 
   7585711
 
   7585896
 
  PDF   STATS   ENSEMBL   UCSC 
 
  186
 
  4
 
 33553
 
 gene
 
 0.742103
 
 0.9058017
 
 Not queried
 
  1.4133905
 
 3.43e-01
 
 0.4672865596
  
 
  
 10
 
  44788627
 
  44789077
 
  PDF   STATS   ENSEMBL   UCSC 
 
  451
 
  5
 
 5706
 
 island
 
 0.200488
 
 0.3441300
 
 Not queried
 
  1.4087394
 
 3.10e-01
 
 0.4672865596
  
 
  
 14
 
  62228873
 
  62229059
 
  PDF   STATS   ENSEMBL   UCSC 
 
  187
 
  6
 
 12445
 
 tss
 
 0.009257
 
 0.0026083
 
 Not queried
 
  1.4049673
 
 3.10e-01
 
 0.4672865596
  
 
  
 7
 
   1039875
 
   1040260
 
  PDF   STATS   ENSEMBL   UCSC 
 
  386
 
  4
 
 36731
 
 gene;island
 
 0.209147
 
 0.5178229
 
 Not queried
 
  1.4024250
 
 3.43e-01
 
 0.4672865596
  
 
  
 2
 
 208635549
 
 208636363
 
  PDF   STATS   ENSEMBL   UCSC 
 
  815
 
 10
 
 25044
 
 island
 
 0.952530
 
 0.2397046
 
 Not queried
 
  3.4195209
 
 3.53e-01
 
 0.4803603739
  
 
  
 19
 
  42806146
 
  42806556
 
  PDF   STATS   ENSEMBL   UCSC 
 
  411
 
 10
 
 20067
 
 gene;island
 
 0.029879
 
 0.1383191
 
 Not queried
 
  1.9893462
 
 3.53e-01
 
 0.4803603739
  
 
  
 7
 
 116164422
 
 116164836
 
  PDF   STATS   ENSEMBL   UCSC 
 
  415
 
 11
 
 37850
 
 tss
 
 0.034177
 
 0.0226336
 
 Not queried
 
  1.7216508
 
 3.65e-01
 
 0.4973948256
  
 
  
 8
 
  57350735
 
  57351185
 
  PDF   STATS   ENSEMBL   UCSC 
 
  451
 
  7
 
 40313
 
 island
 
 0.644513
 
 0.5476801
 
 Not queried
 
  2.9347533
 
 3.83e-01
 
 0.5168893301
  
 
  
 1
 
  22140769
 
  22141400
 
  PDF   STATS   ENSEMBL   UCSC 
 
  632
 
  7
 
 301
 
 gene;island
 
 0.050378
 
 0.5128944
 
 Not queried
 
  2.8693331
 
 3.83e-01
 
 0.5168893301
  
 
  
 15
 
  52404887
 
  52405162
 
  PDF   STATS   ENSEMBL   UCSC 
 
  276
 
  8
 
 14147
 
 island
 
 0.615563
 
 0.1819962
 
 Not queried
 
  2.7420430
 
 3.82e-01
 
 0.5168893301
  
 
  
 19
 
  12946093
 
  12946465
 
  PDF   STATS   ENSEMBL   UCSC 
 
  373
 
  8
 
 21625
 
 island
 
 0.186375
 
 0.0413733
 
 Not queried
 
  2.6206186
 
 3.82e-01
 
 0.5168893301
  
 
  
 10
 
  29698152
 
  29698470
 
  PDF   STATS   ENSEMBL   UCSC 
 
  319
 
  8
 
 5008
 
 tss;island
 
 0.011361
 
 0.0050132
 
 Not queried
 
  2.6103419
 
 3.82e-01
 
 0.5168893301
  
 
  
 1
 
  34642000
 
  34642519
 
  PDF   STATS   ENSEMBL   UCSC 
 
  520
 
  8
 
 1477
 
 tss
 
 0.631119
 
 0.4891831
 
 Not queried
 
  2.3762282
 
 3.82e-01
 
 0.5168893301
  
 
  
 7
 
  15726348
 
  15726821
 
  PDF   STATS   ENSEMBL   UCSC 
 
  474
 
  7
 
 37450
 
 tss
 
 0.007130
 
 0.0097541
 
 Not queried
 
  2.2404597
 
 3.83e-01
 
 0.5168893301
  
 
  
 3
 
 189838996
 
 189839473
 
  PDF   STATS   ENSEMBL   UCSC 
 
  478
 
  7
 
 28081
 
 gene;tss;island
 
 0.876566
 
 0.6098152
 
 Not queried
 
  2.2229652
 
 3.83e-01
 
 0.5168893301
  
 
  
 10
 
  86001241
 
  86001560
 
  PDF   STATS   ENSEMBL   UCSC 
 
  320
 
  7
 
 5194
 
 tss
 
 0.031742
 
 0.1157977
 
 Not queried
 
  1.9978662
 
 3.83e-01
 
 0.5168893301
  
 
  
 11
 
 113184936
 
 113185247
 
  PDF   STATS   ENSEMBL   UCSC 
 
  312
 
  8
 
 7702
 
 tss
 
 0.014096
 
 0.0644177
 
 Not queried
 
  1.9513611
 
 3.82e-01
 
 0.5168893301
  
 
  
 6
 
 143832933
 
 143833251
 
  PDF   STATS   ENSEMBL   UCSC 
 
  319
 
  7
 
 35266
 
 tss
 
 0.518884
 
 0.1022071
 
 Not queried
 
  1.9280694
 
 3.83e-01
 
 0.5168893301
  
 
  
 19
 
  11199851
 
  11200146
 
  PDF   STATS   ENSEMBL   UCSC 
 
  296
 
  7
 
 21550
 
 island
 
 0.002034
 
 0.0034037
 
 Not queried
 
  1.7011578
 
 3.83e-01
 
 0.5168893301
  
 
  
 6
 
 100903561
 
 100904055
 
  PDF   STATS   ENSEMBL   UCSC 
 
  495
 
  7
 
 34431
 
 gene;island
 
 0.090723
 
 0.0069902
 
 Not queried
 
  1.6352069
 
 3.83e-01
 
 0.5168893301
  
 
  
 22
 
  19743759
 
  19744078
 
  PDF   STATS   ENSEMBL   UCSC 
 
  320
 
  7
 
 26965
 
 tss
 
 0.374111
 
 0.5248242
 
 Not queried
 
  1.5838801
 
 3.83e-01
 
 0.5168893301
  
 
  
 6
 
 127840517
 
 127840755
 
  PDF   STATS   ENSEMBL   UCSC 
 
  239
 
  8
 
 35217
 
 tss
 
 0.053568
 
 0.1629892
 
 Not queried
 
  1.5712084
 
 3.82e-01
 
 0.5168893301
  
 
  
 6
 
 143832855
 
 143833251
 
  PDF   STATS   ENSEMBL   UCSC 
 
  397
 
  8
 
 36529
 
 island
 
 0.302293
 
 0.0777072
 
 Not queried
 
  1.5685415
 
 3.82e-01
 
 0.5168893301
  
 
  
 11
 
 115374969
 
 115375432
 
  PDF   STATS   ENSEMBL   UCSC 
 
  464
 
  8
 
 8828
 
 island
 
 0.049250
 
 0.1215208
 
 Not queried
 
  1.5683461
 
 3.82e-01
 
 0.5168893301
  
 
  
 10
 
  79793353
 
  79793501
 
  PDF   STATS   ENSEMBL   UCSC 
 
  149
 
  7
 
 5179
 
 tss
 
 0.015386
 
 0.0052087
 
 Not queried
 
  1.5231980
 
 3.83e-01
 
 0.5168893301
  
 
  
 6
 
 152125861
 
 152126441
 
  PDF   STATS   ENSEMBL   UCSC 
 
  581
 
  8
 
 34511
 
 gene;tss;island
 
 0.615735
 
 0.7408235
 
 Not queried
 
  1.4927996
 
 3.82e-01
 
 0.5168893301
  
 
  
 6
 
  30130109
 
  30130458
 
  PDF   STATS   ENSEMBL   UCSC 
 
  350
 
  7
 
 34764
 
 tss
 
 0.728575
 
 0.8822096
 
 Not queried
 
  1.4871924
 
 3.83e-01
 
 0.5168893301
  
 
  
 13
 
 114312504
 
 114312763
 
  PDF   STATS   ENSEMBL   UCSC 
 
  260
 
  7
 
 11554
 
 tss
 
 0.471664
 
 0.7228183
 
 Not queried
 
  1.4845330
 
 3.83e-01
 
 0.5168893301
  
 
  
 6
 
 169629684
 
 169630013
 
  PDF   STATS   ENSEMBL   UCSC 
 
  330
 
  7
 
 34566
 
 gene;island
 
 0.846557
 
 0.8533990
 
 Not queried
 
  1.4582809
 
 3.83e-01
 
 0.5168893301
  
 
  
 6
 
  55444061
 
  55444488
 
  PDF   STATS   ENSEMBL   UCSC 
 
  428
 
  7
 
 35043
 
 tss
 
 0.124939
 
 0.3019253
 
 Not queried
 
  1.4515803
 
 3.83e-01
 
 0.5168893301
  
 
  
 22
 
  19743759
 
  19744239
 
  PDF   STATS   ENSEMBL   UCSC 
 
  481
 
  8
 
 27304
 
 island
 
 0.312195
 
 0.5137342
 
 Not queried
 
  1.4424126
 
 3.82e-01
 
 0.5168893301
  
 
  
 10
 
 102415991
 
 102416715
 
  PDF   STATS   ENSEMBL   UCSC 
 
  725
 
  7
 
 6028
 
 island
 
 0.080828
 
 0.5208127
 
 Not queried
 
  1.4305206
 
 3.83e-01
 
 0.5168893301
  
 
  
 19
 
  41510026
 
  41510285
 
  PDF   STATS   ENSEMBL   UCSC 
 
  260
 
  7
 
 20052
 
 gene
 
 0.689868
 
 0.8471915
 
 Not queried
 
  1.4023144
 
 3.83e-01
 
 0.5168893301
  
 
  
 15
 
  53086630
 
  53087599
 
  PDF   STATS   ENSEMBL   UCSC 
 
  970
 
  9
 
 14153
 
 island
 
 0.410182
 
 0.1773317
 
 Not queried
 
  2.0568544
 
 3.87e-01
 
 0.5209559659
  
 
  
 7
 
  82792053
 
  82792411
 
  PDF   STATS   ENSEMBL   UCSC 
 
  359
 
  9
 
 38625
 
 island
 
 0.002022
 
 0.0099143
 
 Not queried
 
  1.9120602
 
 3.87e-01
 
 0.5209559659
  
 
  
 4
 
   2935692
 
   2936284
 
  PDF   STATS   ENSEMBL   UCSC 
 
  593
 
  9
 
 29896
 
 gene;island
 
 0.004902
 
 0.0062769
 
 Not queried
 
  1.6396499
 
 3.87e-01
 
 0.5209559659
  
 
  
 1
 
   2984245
 
   2984869
 
  PDF   STATS   ENSEMBL   UCSC 
 
  625
 
  9
 
 1178
 
 tss
 
 0.181684
 
 0.4235885
 
 Not queried
 
  1.5242351
 
 3.87e-01
 
 0.5209559659
  
 
  
 5
 
 161274307
 
 161275138
 
  PDF   STATS   ENSEMBL   UCSC 
 
  832
 
  9
 
 31879
 
 gene;tss
 
 0.006532
 
 0.0296635
 
 Not queried
 
  1.4269512
 
 3.87e-01
 
 0.5209559659
  
 
  
 11
 
  61519687
 
  61520258
 
  PDF   STATS   ENSEMBL   UCSC 
 
  572
 
  6
 
 8331
 
 island
 
 0.230066
 
 0.0163608
 
 Not queried
 
  4.0679597
 
 3.94e-01
 
 0.5236526859
  
 
  
 2
 
  26401640
 
  26402098
 
  PDF   STATS   ENSEMBL   UCSC 
 
  459
 
  6
 
 22607
 
 gene;island
 
 0.215096
 
 0.0246505
 
 Not queried
 
  3.8557063
 
 3.94e-01
 
 0.5236526859
  
 
  
 1
 
  28994850
 
  28995195
 
  PDF   STATS   ENSEMBL   UCSC 
 
  346
 
  6
 
 1435
 
 tss
 
 0.002796
 
 0.0017698
 
 Not queried
 
  2.7928188
 
 3.94e-01
 
 0.5236526859
  
 
  
 19
 
  48948963
 
  48949463
 
  PDF   STATS   ENSEMBL   UCSC 
 
  501
 
  6
 
 22217
 
 island
 
 0.007990
 
 0.0017458
 
 Not queried
 
  2.7276440
 
 3.94e-01
 
 0.5236526859
  
 
  
 12
 
   6421437
 
   6421806
 
  PDF   STATS   ENSEMBL   UCSC 
 
  370
 
  6
 
 9024
 
 gene;tss;island
 
 0.595732
 
 0.8786544
 
 Not queried
 
  2.5233147
 
 3.94e-01
 
 0.5236526859
  
 
  
 17
 
  37307911
 
  37308278
 
  PDF   STATS   ENSEMBL   UCSC 
 
  368
 
  6
 
 17303
 
 tss;island
 
 0.024714
 
 0.0053116
 
 Not queried
 
  2.3535562
 
 3.94e-01
 
 0.5236526859
  
 
  
 4
 
 166299786
 
 166300051
 
  PDF   STATS   ENSEMBL   UCSC 
 
  266
 
  6
 
 30670
 
 tss;island
 
 0.007627
 
 0.0129834
 
 Not queried
 
  2.3403325
 
 3.94e-01
 
 0.5236526859
  
 
  
 6
 
 161695210
 
 161695561
 
  PDF   STATS   ENSEMBL   UCSC 
 
  352
 
  6
 
 35328
 
 tss
 
 0.221149
 
 0.0227276
 
 Not queried
 
  2.3030674
 
 3.94e-01
 
 0.5236526859
  
 
  
 12
 
 107349499
 
 107349539
 
  PDF   STATS   ENSEMBL   UCSC 
 
   41
 
  6
 
 9972
 
 tss
 
 0.004174
 
 0.0055862
 
 Not queried
 
  2.2797407
 
 3.94e-01
 
 0.5236526859
  
 
  
 2
 
 162272107
 
 162272604
 
  PDF   STATS   ENSEMBL   UCSC 
 
  498
 
  6
 
 23687
 
 tss
 
 0.036863
 
 0.0521284
 
 Not queried
 
  2.2253757
 
 3.94e-01
 
 0.5236526859
  
 
  
 7
 
  14028690
 
  14029085
 
  PDF   STATS   ENSEMBL   UCSC 
 
  396
 
  6
 
 36898
 
 gene;island
 
 0.005513
 
 0.0199296
 
 Not queried
 
  2.1446206
 
 3.94e-01
 
 0.5236526859
  
 
  
 5
 
  39425374
 
  39425549
 
  PDF   STATS   ENSEMBL   UCSC 
 
  176
 
  6
 
 32033
 
 tss
 
 0.126382
 
 0.0315732
 
 Not queried
 
  2.1237474
 
 3.94e-01
 
 0.5236526859
  
 
  
 19
 
  49622361
 
  49622717
 
  PDF   STATS   ENSEMBL   UCSC 
 
  357
 
  6
 
 20941
 
 tss;island
 
 0.006717
 
 0.0059772
 
 Not queried
 
  2.0197456
 
 3.94e-01
 
 0.5236526859
  
 
  
 1
 
 180600890
 
 180601141
 
  PDF   STATS   ENSEMBL   UCSC 
 
  252
 
  6
 
 2126
 
 tss;island
 
 0.003498
 
 0.0087014
 
 Not queried
 
  1.9913271
 
 3.94e-01
 
 0.5236526859
  
 
  
 7
 
 151421620
 
 151422150
 
  PDF   STATS   ENSEMBL   UCSC 
 
  531
 
  6
 
 37296
 
 gene;island
 
 0.580765
 
 0.7888995
 
 Not queried
 
  1.9779194
 
 3.94e-01
 
 0.5236526859
  
 
  
 8
 
  82024462
 
  82024860
 
  PDF   STATS   ENSEMBL   UCSC 
 
  399
 
  6
 
 39783
 
 tss;island
 
 0.008255
 
 0.0099803
 
 Not queried
 
  1.9397945
 
 3.94e-01
 
 0.5236526859
  
 
  
 2
 
 228736089
 
 228736324
 
  PDF   STATS   ENSEMBL   UCSC 
 
  236
 
  6
 
 23932
 
 tss
 
 0.012946
 
 0.0406914
 
 Not queried
 
  1.7916249
 
 3.94e-01
 
 0.5236526859
  
 
  
 20
 
  44098223
 
  44098387
 
  PDF   STATS   ENSEMBL   UCSC 
 
  165
 
  6
 
 25776
 
 tss
 
 0.140976
 
 0.1813798
 
 Not queried
 
  1.7825463
 
 3.94e-01
 
 0.5236526859
  
 
  
 19
 
  43979316
 
  43979739
 
  PDF   STATS   ENSEMBL   UCSC 
 
  424
 
  6
 
 20074
 
 gene;island
 
 0.041944
 
 0.1107018
 
 Not queried
 
  1.7788234
 
 3.94e-01
 
 0.5236526859
  
 
  
 14
 
 104551867
 
 104552397
 
  PDF   STATS   ENSEMBL   UCSC 
 
  531
 
  6
 
 13199
 
 island
 
 0.610496
 
 0.8373741
 
 Not queried
 
  1.7769299
 
 3.94e-01
 
 0.5236526859
  
 
  
 17
 
  42247565
 
  42248062
 
  PDF   STATS   ENSEMBL   UCSC 
 
  498
 
  6
 
 17403
 
 tss
 
 0.443762
 
 0.3065741
 
 Not queried
 
  1.7724086
 
 3.94e-01
 
 0.5236526859
  
 
  
 7
 
 134576049
 
 134576386
 
  PDF   STATS   ENSEMBL   UCSC 
 
  338
 
  6
 
 37233
 
 gene
 
 0.076116
 
 0.3859200
 
 Not queried
 
  1.7534795
 
 3.94e-01
 
 0.5236526859
  
 
  
 1
 
  40204285
 
  40204847
 
  PDF   STATS   ENSEMBL   UCSC 
 
  563
 
  6
 
 3107
 
 island
 
 0.007233
 
 0.0123840
 
 Not queried
 
  1.7362856
 
 3.94e-01
 
 0.5236526859
  
 
  
 6
 
 100054469
 
 100054615
 
  PDF   STATS   ENSEMBL   UCSC 
 
  147
 
  6
 
 35137
 
 tss
 
 0.485078
 
 0.2796131
 
 Not queried
 
  1.7031805
 
 3.94e-01
 
 0.5236526859
  
 
  
 6
 
  32489553
 
  32489991
 
  PDF   STATS   ENSEMBL   UCSC 
 
  439
 
  6
 
 34115
 
 gene;island
 
 0.893596
 
 0.6980464
 
 Not queried
 
  1.6994869
 
 3.94e-01
 
 0.5236526859
  
 
  
 11
 
  22214333
 
  22214726
 
  PDF   STATS   ENSEMBL   UCSC 
 
  394
 
  6
 
 8132
 
 island
 
 0.011745
 
 0.0150928
 
 Not queried
 
  1.6785179
 
 3.94e-01
 
 0.5236526859
  
 
  
 10
 
  88024553
 
  88024673
 
  PDF   STATS   ENSEMBL   UCSC 
 
  121
 
  6
 
 5196
 
 tss
 
 0.111615
 
 0.3737576
 
 Not queried
 
  1.6595925
 
 3.94e-01
 
 0.5236526859
  
 
  
 12
 
 117176113
 
 117176563
 
  PDF   STATS   ENSEMBL   UCSC 
 
  451
 
  6
 
 9329
 
 gene
 
 0.005023
 
 0.0073080
 
 Not queried
 
  1.6492697
 
 3.94e-01
 
 0.5236526859
  
 
  
 2
 
 222437040
 
 222437130
 
  PDF   STATS   ENSEMBL   UCSC 
 
   91
 
  6
 
 23913
 
 tss
 
 0.009210
 
 0.0046018
 
 Not queried
 
  1.6436369
 
 3.94e-01
 
 0.5236526859
  
 
  
 1
 
  11795746
 
  11796079
 
  PDF   STATS   ENSEMBL   UCSC 
 
  334
 
 10
 
 1249
 
 tss
 
 0.022372
 
 0.2148243
 
 Not queried
 
  1.5948832
 
 3.93e-01
 
 0.5236526859
  
 
  
 4
 
 140586921
 
 140587179
 
  PDF   STATS   ENSEMBL   UCSC 
 
  259
 
  6
 
 30132
 
 gene
 
 0.020444
 
 0.0117572
 
 Not queried
 
  1.5531161
 
 3.94e-01
 
 0.5236526859
  
 
  
 7
 
 157691177
 
 157691684
 
  PDF   STATS   ENSEMBL   UCSC 
 
  508
 
  6
 
 37351
 
 gene;island
 
 0.146390
 
 0.2970764
 
 Not queried
 
  1.5510841
 
 3.94e-01
 
 0.5236526859
  
 
  
 7
 
  75932840
 
  75932977
 
  PDF   STATS   ENSEMBL   UCSC 
 
  138
 
  6
 
 37053
 
 gene;island
 
 0.004235
 
 0.0063283
 
 Not queried
 
  1.5425568
 
 3.94e-01
 
 0.5236526859
  
 
  
 1
 
  77747875
 
  77748221
 
  PDF   STATS   ENSEMBL   UCSC 
 
  347
 
  6
 
 1705
 
 tss
 
 0.039941
 
 0.0151330
 
 Not queried
 
  1.5146298
 
 3.94e-01
 
 0.5236526859
  
 
  
 16
 
  67926607
 
  67927170
 
  PDF   STATS   ENSEMBL   UCSC 
 
  564
 
  6
 
 15316
 
 tss
 
 0.010292
 
 0.0147605
 
 Not queried
 
  1.5131690
 
 3.94e-01
 
 0.5236526859
  
 
  
 19
 
  51165207
 
  51165845
 
  PDF   STATS   ENSEMBL   UCSC 
 
  639
 
  6
 
 20181
 
 gene;island
 
 0.635574
 
 0.7738421
 
 Not queried
 
  1.5077014
 
 3.94e-01
 
 0.5236526859
  
 
  
 15
 
  55881075
 
  55881246
 
  PDF   STATS   ENSEMBL   UCSC 
 
  172
 
  6
 
 13684
 
 tss
 
 0.306524
 
 0.0678728
 
 Not queried
 
  1.5074665
 
 3.94e-01
 
 0.5236526859
  
 
  
 1
 
  43424882
 
  43425412
 
  PDF   STATS   ENSEMBL   UCSC 
 
  531
 
  6
 
 1555
 
 tss
 
 0.008334
 
 0.0047621
 
 Not queried
 
  1.5012620
 
 3.94e-01
 
 0.5236526859
  
 
  
 11
 
  45230723
 
  45231326
 
  PDF   STATS   ENSEMBL   UCSC 
 
  604
 
  6
 
 6592
 
 gene;island
 
 0.507884
 
 0.6536042
 
 Not queried
 
  1.4770744
 
 3.94e-01
 
 0.5236526859
  
 
  
 1
 
  94703079
 
  94703507
 
  PDF   STATS   ENSEMBL   UCSC 
 
  429
 
 10
 
 3499
 
 island
 
 0.029608
 
 0.0180700
 
 Not queried
 
  1.4563619
 
 3.93e-01
 
 0.5236526859
  
 
  
 17
 
  79875985
 
  79876287
 
  PDF   STATS   ENSEMBL   UCSC 
 
  303
 
  6
 
 18954
 
 island
 
 0.010624
 
 0.0022474
 
 Not queried
 
  1.4445309
 
 3.94e-01
 
 0.5236526859
  
 
  
 4
 
  74847758
 
  74848016
 
  PDF   STATS   ENSEMBL   UCSC 
 
  259
 
  6
 
 30431
 
 tss
 
 0.384563
 
 0.6089371
 
 Not queried
 
  1.4243090
 
 3.94e-01
 
 0.5236526859
  
 
  
 2
 
  27193211
 
  27193496
 
  PDF   STATS   ENSEMBL   UCSC 
 
  286
 
  6
 
 23248
 
 tss
 
 0.002727
 
 0.0051713
 
 Not queried
 
  1.4189222
 
 3.94e-01
 
 0.5236526859
  
 
  
 10
 
 129535138
 
 129535968
 
  PDF   STATS   ENSEMBL   UCSC 
 
  831
 
 11
 
 6249
 
 island
 
 0.047058
 
 0.2171388
 
 Not queried
 
  2.1240173
 
 4.01e-01
 
 0.5327651490
  
 
  
 19
 
  11353961
 
  11354401
 
  PDF   STATS   ENSEMBL   UCSC 
 
  441
 
  5
 
 19832
 
 gene;island
 
 0.576828
 
 0.1551352
 
 Not queried
 
  5.3964126
 
 4.21e-01
 
 0.5404252807
  
 
  
 14
 
  53418879
 
  53419111
 
  PDF   STATS   ENSEMBL   UCSC 
 
  233
 
  5
 
 12402
 
 tss;island
 
 0.017951
 
 0.5144407
 
 Not queried
 
  4.8516552
 
 4.21e-01
 
 0.5404252807
  
 
  
 2
 
 127413363
 
 127413530
 
  PDF   STATS   ENSEMBL   UCSC 
 
  168
 
  5
 
 23608
 
 tss;island
 
 0.054603
 
 0.6023570
 
 Not queried
 
  4.2785690
 
 4.21e-01
 
 0.5404252807
  
 
  
 7
 
  48019205
 
  48019713
 
  PDF   STATS   ENSEMBL   UCSC 
 
  509
 
  5
 
 38481
 
 island
 
 0.038628
 
 0.6851785
 
 Not queried
 
  3.7303551
 
 4.21e-01
 
 0.5404252807
  
 
  
 5
 
 131892242
 
 131892523
 
  PDF   STATS   ENSEMBL   UCSC 
 
  282
 
  5
 
 32272
 
 tss
 
 0.007403
 
 0.4551062
 
 Not queried
 
  3.6560254
 
 4.21e-01
 
 0.5404252807
  
 
  
 9
 
 137030463
 
 137030823
 
  PDF   STATS   ENSEMBL   UCSC 
 
  361
 
  5
 
 40853
 
 tss;island
 
 0.018871
 
 0.2175678
 
 Not queried
 
  3.4291385
 
 4.21e-01
 
 0.5404252807
  
 
  
 10
 
  95361132
 
  95361386
 
  PDF   STATS   ENSEMBL   UCSC 
 
  255
 
  5
 
 5239
 
 tss;island
 
 0.315542
 
 0.0832746
 
 Not queried
 
  3.3915593
 
 4.21e-01
 
 0.5404252807
  
 
  
 6
 
 110736772
 
 110737053
 
  PDF   STATS   ENSEMBL   UCSC 
 
  282
 
  5
 
 35169
 
 tss
 
 0.041832
 
 0.0527786
 
 Not queried
 
  3.2571161
 
 4.21e-01
 
 0.5404252807
  
 
  
 2
 
  97171137
 
  97171449
 
  PDF   STATS   ENSEMBL   UCSC 
 
  313
 
  5
 
 22781
 
 gene;island
 
 0.012691
 
 0.2584532
 
 Not queried
 
  3.1916260
 
 4.21e-01
 
 0.5404252807
  
 
  
 2
 
 219861012
 
 219861150
 
  PDF   STATS   ENSEMBL   UCSC 
 
  139
 
  5
 
 25101
 
 island
 
 0.012309
 
 0.0036412
 
 Not queried
 
  3.0441930
 
 4.21e-01
 
 0.5404252807
  
 
  
 18
 
  77793182
 
  77793554
 
  PDF   STATS   ENSEMBL   UCSC 
 
  373
 
  5
 
 19340
 
 tss;island
 
 0.003679
 
 0.0067487
 
 Not queried
 
  3.0299247
 
 4.21e-01
 
 0.5404252807
  
 
  
 6
 
  32018906
 
  32019556
 
  PDF   STATS   ENSEMBL   UCSC 
 
  651
 
  5
 
 34017
 
 gene
 
 0.048981
 
 0.2209213
 
 Not queried
 
  3.0283103
 
 4.21e-01
 
 0.5404252807
  
 
  
 15
 
  67813132
 
  67813371
 
  PDF   STATS   ENSEMBL   UCSC 
 
  240
 
  5
 
 13756
 
 tss;island
 
 0.003570
 
 0.0046130
 
 Not queried
 
  2.9260172
 
 4.21e-01
 
 0.5404252807
  
 
  
 7
 
  88425077
 
  88425148
 
  PDF   STATS   ENSEMBL   UCSC 
 
   72
 
  5
 
 37693
 
 tss
 
 0.687849
 
 0.9054183
 
 Not queried
 
  2.8198869
 
 4.21e-01
 
 0.5404252807
  
 
  
 6
 
 100442002
 
 100442151
 
  PDF   STATS   ENSEMBL   UCSC 
 
  150
 
  5
 
 36361
 
 island
 
 0.269554
 
 0.0396720
 
 Not queried
 
  2.8125338
 
 4.21e-01
 
 0.5404252807
  
 
  
 10
 
 101874829
 
 101875138
 
  PDF   STATS   ENSEMBL   UCSC 
 
  310
 
  5
 
 6018
 
 island
 
 0.631686
 
 0.9168938
 
 Not queried
 
  2.7589024
 
 4.21e-01
 
 0.5404252807
  
 
  
 4
 
   8136996
 
   8137155
 
  PDF   STATS   ENSEMBL   UCSC 
 
  160
 
  5
 
 29947
 
 gene
 
 0.446253
 
 0.5755021
 
 Not queried
 
  2.7224966
 
 4.21e-01
 
 0.5404252807
  
 
  
 20
 
  35169260
 
  35169609
 
  PDF   STATS   ENSEMBL   UCSC 
 
  350
 
  5
 
 25716
 
 tss;island
 
 0.091356
 
 0.2278898
 
 Not queried
 
  2.7124267
 
 4.21e-01
 
 0.5404252807
  
 
  
 6
 
  85482570
 
  85483055
 
  PDF   STATS   ENSEMBL   UCSC 
 
  486
 
  5
 
 36298
 
 island
 
 0.321699
 
 0.2147192
 
 Not queried
 
  2.6551371
 
 4.21e-01
 
 0.5404252807
  
 
  
 21
 
  42688468
 
  42688650
 
  PDF   STATS   ENSEMBL   UCSC 
 
  183
 
  5
 
 26555
 
 tss
 
 0.026122
 
 0.0504250
 
 Not queried
 
  2.6509796
 
 4.21e-01
 
 0.5404252807
  
 
  
 7
 
  45927735
 
  45927947
 
  PDF   STATS   ENSEMBL   UCSC 
 
  213
 
  5
 
 37586
 
 tss
 
 0.093613
 
 0.4889669
 
 Not queried
 
  2.6202936
 
 4.21e-01
 
 0.5404252807
  
 
  
 16
 
   1070957
 
   1071402
 
  PDF   STATS   ENSEMBL   UCSC 
 
  446
 
  5
 
 15508
 
 island
 
 0.417148
 
 0.5679185
 
 Not queried
 
  2.6089855
 
 4.21e-01
 
 0.5404252807
  
 
  
 3
 
 147142102
 
 147142415
 
  PDF   STATS   ENSEMBL   UCSC 
 
  314
 
  5
 
 29576
 
 island
 
 0.036478
 
 0.2752495
 
 Not queried
 
  2.5599032
 
 4.21e-01
 
 0.5404252807
  
 
  
 22
 
  31536476
 
  31536784
 
  PDF   STATS   ENSEMBL   UCSC 
 
  309
 
  5
 
 27075
 
 tss
 
 0.030401
 
 0.1194275
 
 Not queried
 
  2.3761116
 
 4.21e-01
 
 0.5404252807
  
 
  
 12
 
 121342197
 
 121342435
 
  PDF   STATS   ENSEMBL   UCSC 
 
  239
 
  5
 
 10068
 
 tss;island
 
 0.011835
 
 0.0081462
 
 Not queried
 
  2.3613229
 
 4.21e-01
 
 0.5404252807
  
 
  
 2
 
  26468086
 
  26468406
 
  PDF   STATS   ENSEMBL   UCSC 
 
  321
 
  5
 
 22609
 
 gene;tss;island
 
 0.003437
 
 0.0052212
 
 Not queried
 
  2.3528515
 
 4.21e-01
 
 0.5404252807
  
 
  
 7
 
 152456579
 
 152456815
 
  PDF   STATS   ENSEMBL   UCSC 
 
  237
 
  5
 
 38007
 
 tss;island
 
 0.003538
 
 0.0073717
 
 Not queried
 
  2.3437336
 
 4.21e-01
 
 0.5404252807
  
 
  
 22
 
  50968297
 
  50968516
 
  PDF   STATS   ENSEMBL   UCSC 
 
  220
 
  5
 
 27258
 
 tss
 
 0.014551
 
 0.0041809
 
 Not queried
 
  2.3089882
 
 4.21e-01
 
 0.5404252807
  
 
  
 17
 
  56232260
 
  56232674
 
  PDF   STATS   ENSEMBL   UCSC 
 
  415
 
  5
 
 18594
 
 island
 
 0.014781
 
 0.3302219
 
 Not queried
 
  2.3059221
 
 4.21e-01
 
 0.5404252807
  
 
  
 10
 
  99695981
 
  99696260
 
  PDF   STATS   ENSEMBL   UCSC 
 
  280
 
  5
 
 4701
 
 gene
 
 0.448856
 
 0.7980857
 
 Not queried
 
  2.2934970
 
 4.21e-01
 
 0.5404252807
  
 
  
 17
 
  43047405
 
  43047971
 
  PDF   STATS   ENSEMBL   UCSC 
 
  567
 
  5
 
 18418
 
 island
 
 0.009422
 
 0.0102568
 
 Not queried
 
  2.2541210
 
 4.21e-01
 
 0.5404252807
  
 
  
 12
 
   8995557
 
   8995965
 
  PDF   STATS   ENSEMBL   UCSC 
 
  409
 
  5
 
 9050
 
 gene
 
 0.741236
 
 0.9407724
 
 Not queried
 
  2.2312488
 
 4.21e-01
 
 0.5404252807
  
 
  
 12
 
   2788627
 
   2788847
 
  PDF   STATS   ENSEMBL   UCSC 
 
  221
 
  5
 
 9005
 
 gene;island
 
 0.693635
 
 0.8569039
 
 Not queried
 
  2.1989840
 
 4.21e-01
 
 0.5404252807
  
 
  
 1
 
  94703343
 
  94703507
 
  PDF   STATS   ENSEMBL   UCSC 
 
  165
 
  5
 
 1763
 
 tss
 
 0.105000
 
 0.0394629
 
 Not queried
 
  2.1977596
 
 4.21e-01
 
 0.5404252807
  
 
  
 7
 
  98248852
 
  98249103
 
  PDF   STATS   ENSEMBL   UCSC 
 
  252
 
  5
 
 37114
 
 gene;island
 
 0.055753
 
 0.0361054
 
 Not queried
 
  2.1844779
 
 4.21e-01
 
 0.5404252807
  
 
  
 6
 
  32725075
 
  32725438
 
  PDF   STATS   ENSEMBL   UCSC 
 
  364
 
  5
 
 34131
 
 gene
 
 0.382679
 
 0.4704989
 
 Not queried
 
  2.1774197
 
 4.21e-01
 
 0.5404252807
  
 
  
 12
 
 133186599
 
 133187174
 
  PDF   STATS   ENSEMBL   UCSC 
 
  576
 
  5
 
 11066
 
 island
 
 0.469969
 
 0.1667485
 
 Not queried
 
  2.1660572
 
 4.21e-01
 
 0.5404252807
  
 
  
 1
 
 155948550
 
 155949012
 
  PDF   STATS   ENSEMBL   UCSC 
 
  463
 
  5
 
 2008
 
 tss;island
 
 0.014550
 
 0.0119415
 
 Not queried
 
  2.1628273
 
 4.21e-01
 
 0.5404252807
  
 
  
 19
 
  52599190
 
  52599329
 
  PDF   STATS   ENSEMBL   UCSC 
 
  140
 
  5
 
 21006
 
 tss
 
 0.037107
 
 0.0091362
 
 Not queried
 
  2.1561592
 
 4.21e-01
 
 0.5404252807
  
 
  
 1
 
  55088984
 
  55089372
 
  PDF   STATS   ENSEMBL   UCSC 
 
  389
 
  5
 
 549
 
 gene
 
 0.580170
 
 0.6898542
 
 Not queried
 
  2.1504773
 
 4.21e-01
 
 0.5404252807
  
 
  
 10
 
 102987993
 
 102988529
 
  PDF   STATS   ENSEMBL   UCSC 
 
  537
 
  5
 
 4726
 
 gene;tss;island
 
 0.068803
 
 0.1683915
 
 Not queried
 
  2.1363885
 
 4.21e-01
 
 0.5404252807
  
 
  
 17
 
  40936498
 
  40936820
 
  PDF   STATS   ENSEMBL   UCSC 
 
  323
 
  5
 
 16588
 
 gene;island
 
 0.021050
 
 0.1561701
 
 Not queried
 
  2.1340729
 
 4.21e-01
 
 0.5404252807
  
 
  
 1
 
 160312929
 
 160313057
 
  PDF   STATS   ENSEMBL   UCSC 
 
  129
 
  5
 
 2038
 
 tss
 
 0.006777
 
 0.0017536
 
 Not queried
 
  2.1339718
 
 4.21e-01
 
 0.5404252807
  
 
  
 17
 
  74137399
 
  74137522
 
  PDF   STATS   ENSEMBL   UCSC 
 
  124
 
  5
 
 17672
 
 tss
 
 0.000236
 
 0.0029575
 
 Not queried
 
  2.1262372
 
 4.21e-01
 
 0.5404252807
  
 
  
 19
 
  15083795
 
  15083916
 
  PDF   STATS   ENSEMBL   UCSC 
 
  122
 
  5
 
 20551
 
 tss
 
 0.373523
 
 0.4972341
 
 Not queried
 
  2.1189057
 
 4.21e-01
 
 0.5404252807
  
 
  
 11
 
 118401536
 
 118401878
 
  PDF   STATS   ENSEMBL   UCSC 
 
  343
 
  5
 
 8858
 
 island
 
 0.002953
 
 0.0039406
 
 Not queried
 
  2.0659347
 
 4.21e-01
 
 0.5404252807
  
 
  
 17
 
  42431627
 
  42432165
 
  PDF   STATS   ENSEMBL   UCSC 
 
  539
 
  5
 
 16617
 
 gene;island
 
 0.004949
 
 0.0270820
 
 Not queried
 
  2.0392581
 
 4.21e-01
 
 0.5404252807
  
 
  
 7
 
  45066738
 
  45067057
 
  PDF   STATS   ENSEMBL   UCSC 
 
  320
 
  5
 
 36997
 
 gene;tss
 
 0.022754
 
 0.0232528
 
 Not queried
 
  1.9928395
 
 4.21e-01
 
 0.5404252807
  
 
  
 1
 
 211667297
 
 211667751
 
  PDF   STATS   ENSEMBL   UCSC 
 
  455
 
  5
 
 2239
 
 tss
 
 0.634581
 
 0.7423326
 
 Not queried
 
  1.9785076
 
 4.21e-01
 
 0.5404252807
  
 
  
 2
 
 120282191
 
 120282327
 
  PDF   STATS   ENSEMBL   UCSC 
 
  137
 
  5
 
 23595
 
 tss
 
 0.860998
 
 0.9604909
 
 Not queried
 
  1.9744148
 
 4.21e-01
 
 0.5404252807
  
 
  
 5
 
 115297690
 
 115298079
 
  PDF   STATS   ENSEMBL   UCSC 
 
  390
 
  5
 
 32232
 
 tss
 
 0.551634
 
 0.0414134
 
 Not queried
 
  1.9740022
 
 4.21e-01
 
 0.5404252807
  
 
  
 14
 
  93798983
 
  93799331
 
  PDF   STATS   ENSEMBL   UCSC 
 
  349
 
  5
 
 12188
 
 gene
 
 0.003617
 
 0.0013198
 
 Not queried
 
  1.9537033
 
 4.21e-01
 
 0.5404252807
  
 
  
 2
 
  48132755
 
  48133053
 
  PDF   STATS   ENSEMBL   UCSC 
 
  299
 
  5
 
 24286
 
 island
 
 0.003085
 
 0.0065989
 
 Not queried
 
  1.9399613
 
 4.21e-01
 
 0.5404252807
  
 
  
 20
 
  55904633
 
  55904768
 
  PDF   STATS   ENSEMBL   UCSC 
 
  136
 
  5
 
 25841
 
 tss
 
 0.752291
 
 0.9467671
 
 Not queried
 
  1.9330429
 
 4.21e-01
 
 0.5404252807
  
 
  
 22
 
  36784250
 
  36784627
 
  PDF   STATS   ENSEMBL   UCSC 
 
  378
 
  5
 
 27107
 
 tss;island
 
 0.005793
 
 0.0006386
 
 Not queried
 
  1.8961238
 
 4.21e-01
 
 0.5404252807
  
 
  
 4
 
 187810446
 
 187810783
 
  PDF   STATS   ENSEMBL   UCSC 
 
  338
 
  5
 
 31487
 
 island
 
 0.573393
 
 0.9278655
 
 Not queried
 
  1.8836094
 
 4.21e-01
 
 0.5404252807
  
 
  
 4
 
 176922556
 
 176923081
 
  PDF   STATS   ENSEMBL   UCSC 
 
  526
 
  5
 
 30185
 
 gene;island
 
 0.015106
 
 0.0081440
 
 Not queried
 
  1.8709818
 
 4.21e-01
 
 0.5404252807
  
 
  
 1
 
 206680459
 
 206680868
 
  PDF   STATS   ENSEMBL   UCSC 
 
  410
 
  5
 
 2212
 
 tss
 
 0.006145
 
 0.0208515
 
 Not queried
 
  1.8647177
 
 4.21e-01
 
 0.5404252807
  
 
  
 18
 
  19476805
 
  19477059
 
  PDF   STATS   ENSEMBL   UCSC 
 
  255
 
  5
 
 19420
 
 island
 
 0.513000
 
 0.6348109
 
 Not queried
 
  1.8519570
 
 4.21e-01
 
 0.5404252807
  
 
  
 19
 
    663355
 
    663602
 
  PDF   STATS   ENSEMBL   UCSC 
 
  248
 
  5
 
 20277
 
 tss;island
 
 0.004359
 
 0.0106079
 
 Not queried
 
  1.8513220
 
 4.21e-01
 
 0.5404252807
  
 
  
 6
 
 167276275
 
 167276650
 
  PDF   STATS   ENSEMBL   UCSC 
 
  376
 
  5
 
 35339
 
 tss;island
 
 0.036784
 
 0.0179026
 
 Not queried
 
  1.8379510
 
 4.21e-01
 
 0.5404252807
  
 
  
 1
 
 202927535
 
 202927977
 
  PDF   STATS   ENSEMBL   UCSC 
 
  443
 
  5
 
 2186
 
 tss
 
 0.019869
 
 0.0232619
 
 Not queried
 
  1.8232111
 
 4.21e-01
 
 0.5404252807
  
 
  
 6
 
  30112423
 
  30112776
 
  PDF   STATS   ENSEMBL   UCSC 
 
  354
 
  5
 
 33727
 
 gene
 
 0.552994
 
 0.6623090
 
 Not queried
 
  1.8082745
 
 4.21e-01
 
 0.5404252807
  
 
  
 6
 
  31276088
 
  31276212
 
  PDF   STATS   ENSEMBL   UCSC 
 
  125
 
  5
 
 35763
 
 island
 
 0.088168
 
 0.1788566
 
 Not queried
 
  1.7991068
 
 4.21e-01
 
 0.5404252807
  
 
  
 20
 
  44258197
 
  44258437
 
  PDF   STATS   ENSEMBL   UCSC 
 
  241
 
  5
 
 25431
 
 gene
 
 0.091807
 
 0.4450900
 
 Not queried
 
  1.7942820
 
 4.21e-01
 
 0.5404252807
  
 
  
 11
 
  44341361
 
  44341831
 
  PDF   STATS   ENSEMBL   UCSC 
 
  471
 
  5
 
 8227
 
 island
 
 0.059698
 
 0.1751281
 
 Not queried
 
  1.7883141
 
 4.21e-01
 
 0.5404252807
  
 
  
 10
 
  85324700
 
  85325062
 
  PDF   STATS   ENSEMBL   UCSC 
 
  363
 
  5
 
 5892
 
 island
 
 0.590998
 
 0.8728752
 
 Not queried
 
  1.7839264
 
 4.21e-01
 
 0.5404252807
  
 
  
 13
 
 112724221
 
 112724583
 
  PDF   STATS   ENSEMBL   UCSC 
 
  363
 
  5
 
 11246
 
 gene;island
 
 0.457268
 
 0.2132542
 
 Not queried
 
  1.7803893
 
 4.21e-01
 
 0.5404252807
  
 
  
 19
 
   4566400
 
   4566677
 
  PDF   STATS   ENSEMBL   UCSC 
 
  278
 
  5
 
 21381
 
 island
 
 0.539492
 
 0.5707105
 
 Not queried
 
  1.7776927
 
 4.21e-01
 
 0.5404252807
  
 
  
 21
 
  44496158
 
  44496378
 
  PDF   STATS   ENSEMBL   UCSC 
 
  221
 
  5
 
 26573
 
 tss
 
 0.006615
 
 0.0150632
 
 Not queried
 
  1.7758109
 
 4.21e-01
 
 0.5404252807
  
 
  
 1
 
   7887199
 
   7887560
 
  PDF   STATS   ENSEMBL   UCSC 
 
  362
 
  5
 
 190
 
 gene;island
 
 0.757511
 
 0.9134019
 
 Not queried
 
  1.7756655
 
 4.21e-01
 
 0.5404252807
  
 
  
 4
 
  22389706
 
  22390126
 
  PDF   STATS   ENSEMBL   UCSC 
 
  421
 
  5
 
 29967
 
 gene
 
 0.569250
 
 0.8650862
 
 Not queried
 
  1.7749973
 
 4.21e-01
 
 0.5404252807
  
 
  
 11
 
  66725860
 
  66725961
 
  PDF   STATS   ENSEMBL   UCSC 
 
  102
 
  5
 
 7482
 
 tss
 
 0.045125
 
 0.0189259
 
 Not queried
 
  1.7506233
 
 4.21e-01
 
 0.5404252807
  
 
  
 22
 
  44708761
 
  44708908
 
  PDF   STATS   ENSEMBL   UCSC 
 
  148
 
  5
 
 27212
 
 tss
 
 0.202476
 
 0.4086259
 
 Not queried
 
  1.7201868
 
 4.21e-01
 
 0.5404252807
  
 
  
 11
 
  67432681
 
  67433133
 
  PDF   STATS   ENSEMBL   UCSC 
 
  453
 
  5
 
 6763
 
 gene;island
 
 0.203495
 
 0.5626587
 
 Not queried
 
  1.7176764
 
 4.21e-01
 
 0.5404252807
  
 
  
 3
 
  48471575
 
  48471901
 
  PDF   STATS   ENSEMBL   UCSC 
 
  327
 
  5
 
 28297
 
 tss;island
 
 0.542671
 
 0.4882588
 
 Not queried
 
  1.6924862
 
 4.21e-01
 
 0.5404252807
  
 
  
 2
 
 217363243
 
 217363465
 
  PDF   STATS   ENSEMBL   UCSC 
 
  223
 
  5
 
 23869
 
 tss
 
 0.002887
 
 0.0018564
 
 Not queried
 
  1.6854711
 
 4.21e-01
 
 0.5404252807
  
 
  
 6
 
  33144795
 
  33145182
 
  PDF   STATS   ENSEMBL   UCSC 
 
  388
 
  5
 
 34205
 
 gene
 
 0.365399
 
 0.7579557
 
 Not queried
 
  1.6786311
 
 4.21e-01
 
 0.5404252807
  
 
  
 5
 
   1257034
 
   1257221
 
  PDF   STATS   ENSEMBL   UCSC 
 
  188
 
  5
 
 31530
 
 gene;island
 
 0.793292
 
 0.5466133
 
 Not queried
 
  1.6671164
 
 4.21e-01
 
 0.5404252807
  
 
  
 17
 
  48785310
 
  48785602
 
  PDF   STATS   ENSEMBL   UCSC 
 
  293
 
  5
 
 17505
 
 tss
 
 0.021007
 
 0.0078438
 
 Not queried
 
  1.6616441
 
 4.21e-01
 
 0.5404252807
  
 
  
 1
 
  47489195
 
  47489543
 
  PDF   STATS   ENSEMBL   UCSC 
 
  349
 
  5
 
 3228
 
 island
 
 0.095692
 
 0.0367792
 
 Not queried
 
  1.6415192
 
 4.21e-01
 
 0.5404252807
  
 
  
 4
 
  41867415
 
  41867562
 
  PDF   STATS   ENSEMBL   UCSC 
 
  148
 
  5
 
 31005
 
 island
 
 0.020640
 
 0.1137083
 
 Not queried
 
  1.6235898
 
 4.21e-01
 
 0.5404252807
  
 
  
 20
 
  13765341
 
  13765530
 
  PDF   STATS   ENSEMBL   UCSC 
 
  190
 
  5
 
 25360
 
 gene
 
 0.004460
 
 0.0004637
 
 Not queried
 
  1.6162427
 
 4.21e-01
 
 0.5404252807
  
 
  
 1
 
  33816020
 
  33816159
 
  PDF   STATS   ENSEMBL   UCSC 
 
  140
 
  5
 
 404
 
 gene;tss;island
 
 0.007098
 
 0.0105188
 
 Not queried
 
  1.6142770
 
 4.21e-01
 
 0.5404252807
  
 
  
 9
 
 124262344
 
 124262699
 
  PDF   STATS   ENSEMBL   UCSC 
 
  356
 
  5
 
 40835
 
 tss;island
 
 0.107304
 
 0.0329016
 
 Not queried
 
  1.5891479
 
 4.21e-01
 
 0.5404252807
  
 
  
 2
 
 106681831
 
 106681998
 
  PDF   STATS   ENSEMBL   UCSC 
 
  168
 
  5
 
 23556
 
 tss;island
 
 0.390784
 
 0.1124596
 
 Not queried
 
  1.5862580
 
 4.21e-01
 
 0.5404252807
  
 
  
 15
 
  79574773
 
  79575048
 
  PDF   STATS   ENSEMBL   UCSC 
 
  276
 
  5
 
 13843
 
 tss;island
 
 0.011688
 
 0.0185231
 
 Not queried
 
  1.5717988
 
 4.21e-01
 
 0.5404252807
  
 
  
 7
 
  87936225
 
  87936414
 
  PDF   STATS   ENSEMBL   UCSC 
 
  190
 
  5
 
 37691
 
 tss
 
 0.036441
 
 0.0459499
 
 Not queried
 
  1.5454032
 
 4.21e-01
 
 0.5404252807
  
 
  
 20
 
  43029468
 
  43029895
 
  PDF   STATS   ENSEMBL   UCSC 
 
  428
 
  5
 
 25759
 
 tss
 
 0.261912
 
 0.4275095
 
 Not queried
 
  1.5278933
 
 4.21e-01
 
 0.5404252807
  
 
  
 6
 
  27782484
 
  27782658
 
  PDF   STATS   ENSEMBL   UCSC 
 
  175
 
  5
 
 34720
 
 tss;island
 
 0.007316
 
 0.0045772
 
 Not queried
 
  1.5262981
 
 4.21e-01
 
 0.5404252807
  
 
  
 13
 
  20532610
 
  20532768
 
  PDF   STATS   ENSEMBL   UCSC 
 
  159
 
  5
 
 11326
 
 tss
 
 0.005521
 
 0.0040927
 
 Not queried
 
  1.5222563
 
 4.21e-01
 
 0.5404252807
  
 
  
 5
 
 169724590
 
 169724803
 
  PDF   STATS   ENSEMBL   UCSC 
 
  214
 
  5
 
 31889
 
 gene
 
 0.008420
 
 0.0198473
 
 Not queried
 
  1.5181483
 
 4.21e-01
 
 0.5404252807
  
 
  
 13
 
  52419332
 
  52419483
 
  PDF   STATS   ENSEMBL   UCSC 
 
  152
 
  5
 
 11454
 
 tss
 
 0.131747
 
 0.0570990
 
 Not queried
 
  1.5149699
 
 4.21e-01
 
 0.5404252807
  
 
  
 11
 
 120894635
 
 120894841
 
  PDF   STATS   ENSEMBL   UCSC 
 
  207
 
  5
 
 8897
 
 island
 
 0.016789
 
 0.0051368
 
 Not queried
 
  1.5071707
 
 4.21e-01
 
 0.5404252807
  
 
  
 6
 
  29573014
 
  29573377
 
  PDF   STATS   ENSEMBL   UCSC 
 
  364
 
  5
 
 33666
 
 gene
 
 0.448437
 
 0.7813951
 
 Not queried
 
  1.4824625
 
 4.21e-01
 
 0.5404252807
  
 
  
 14
 
 101123263
 
 101123599
 
  PDF   STATS   ENSEMBL   UCSC 
 
  337
 
  5
 
 12606
 
 tss
 
 0.701154
 
 0.8013707
 
 Not queried
 
  1.4793927
 
 4.21e-01
 
 0.5404252807
  
 
  
 5
 
   7869257
 
   7869428
 
  PDF   STATS   ENSEMBL   UCSC 
 
  172
 
  5
 
 31558
 
 gene
 
 0.002569
 
 0.0069708
 
 Not queried
 
  1.4667668
 
 4.21e-01
 
 0.5404252807
  
 
  
 1
 
 156084490
 
 156084750
 
  PDF   STATS   ENSEMBL   UCSC 
 
  261
 
  5
 
 814
 
 gene
 
 0.004857
 
 0.0037934
 
 Not queried
 
  1.4611802
 
 4.21e-01
 
 0.5404252807
  
 
  
 1
 
   4832352
 
   4832593
 
  PDF   STATS   ENSEMBL   UCSC 
 
  242
 
  5
 
 153
 
 gene;island
 
 0.533579
 
 0.6081855
 
 Not queried
 
  1.4605520
 
 4.21e-01
 
 0.5404252807
  
 
  
 7
 
  80548695
 
  80548752
 
  PDF   STATS   ENSEMBL   UCSC 
 
   58
 
  5
 
 37675
 
 tss;island
 
 0.018082
 
 0.0781476
 
 Not queried
 
  1.4430803
 
 4.21e-01
 
 0.5404252807
  
 
  
 5
 
 157170413
 
 157170601
 
  PDF   STATS   ENSEMBL   UCSC 
 
  189
 
  5
 
 32458
 
 tss;island
 
 0.002139
 
 0.0027530
 
 Not queried
 
  1.4425783
 
 4.21e-01
 
 0.5404252807
  
 
  
 2
 
 241938159
 
 241938193
 
  PDF   STATS   ENSEMBL   UCSC 
 
   35
 
  5
 
 23983
 
 tss
 
 0.007660
 
 0.0032372
 
 Not queried
 
  1.4339440
 
 4.21e-01
 
 0.5404252807
  
 
  
 10
 
  63213339
 
  63213510
 
  PDF   STATS   ENSEMBL   UCSC 
 
  172
 
  5
 
 5097
 
 tss
 
 0.107972
 
 0.4735550
 
 Not queried
 
  1.4226529
 
 4.21e-01
 
 0.5404252807
  
 
  
 10
 
  80733663
 
  80734012
 
  PDF   STATS   ENSEMBL   UCSC 
 
  350
 
  5
 
 4648
 
 gene;island
 
 0.018666
 
 0.0057314
 
 Not queried
 
  1.4197273
 
 4.21e-01
 
 0.5404252807
  
 
  
 8
 
 124194847
 
 124195192
 
  PDF   STATS   ENSEMBL   UCSC 
 
  346
 
  5
 
 39417
 
 gene
 
 0.529235
 
 0.7488696
 
 Not queried
 
  1.4147898
 
 4.21e-01
 
 0.5404252807
  
 
  
 5
 
  37834909
 
  37835168
 
  PDF   STATS   ENSEMBL   UCSC 
 
  260
 
  5
 
 32025
 
 tss
 
 0.015303
 
 0.0112806
 
 Not queried
 
  1.4147538
 
 4.21e-01
 
 0.5404252807
  
 
  
 20
 
  62151993
 
  62152146
 
  PDF   STATS   ENSEMBL   UCSC 
 
  154
 
  5
 
 26354
 
 island
 
 0.001655
 
 0.0047575
 
 Not queried
 
  1.4033526
 
 4.21e-01
 
 0.5404252807
  
 
  
 11
 
 131779469
 
 131780499
 
  PDF   STATS   ENSEMBL   UCSC 
 
 1031
 
 18
 
 6978
 
 gene;tss;island
 
 0.037313
 
 0.2762719
 
 Not queried
 
  1.4461120
 
 4.24e-01
 
 0.5451667310
  
 
  
 10
 
  70321243
 
  70321959
 
  PDF   STATS   ENSEMBL   UCSC 
 
  717
 
 10
 
 4610
 
 gene;island
 
 0.022200
 
 0.1731352
 
 Not queried
 
  2.7029331
 
 4.36e-01
 
 0.5589686471
  
 
  
 20
 
  61809557
 
  61809932
 
  PDF   STATS   ENSEMBL   UCSC 
 
  376
 
  9
 
 26343
 
 island
 
 0.063572
 
 0.0769251
 
 Not queried
 
  2.0164172
 
 4.36e-01
 
 0.5589686471
  
 
  
 10
 
 104535792
 
 104536121
 
  PDF   STATS   ENSEMBL   UCSC 
 
  330
 
  9
 
 4738
 
 gene
 
 0.277101
 
 0.0968229
 
 Not queried
 
  1.9601896
 
 4.36e-01
 
 0.5589686471
  
 
  
 12
 
  12965884
 
  12966464
 
  PDF   STATS   ENSEMBL   UCSC 
 
  581
 
  9
 
 10256
 
 island
 
 0.005232
 
 0.0024193
 
 Not queried
 
  1.8215567
 
 4.36e-01
 
 0.5589686471
  
 
  
 5
 
 176778888
 
 176779229
 
  PDF   STATS   ENSEMBL   UCSC 
 
  342
 
  9
 
 32524
 
 tss
 
 0.001469
 
 0.0037723
 
 Not queried
 
  1.6582319
 
 4.36e-01
 
 0.5589686471
  
 
  
 15
 
  31195612
 
  31196075
 
  PDF   STATS   ENSEMBL   UCSC 
 
  464
 
  9
 
 13980
 
 island
 
 0.029916
 
 0.0096563
 
 Not queried
 
  1.6077622
 
 4.36e-01
 
 0.5589686471
  
 
  
 19
 
  51487109
 
  51487594
 
  PDF   STATS   ENSEMBL   UCSC 
 
  486
 
  9
 
 20988
 
 tss
 
 0.295903
 
 0.1600472
 
 Not queried
 
  1.5230690
 
 4.36e-01
 
 0.5589686471
  
 
  
 11
 
  36615733
 
  36616365
 
  PDF   STATS   ENSEMBL   UCSC 
 
  633
 
  9
 
 6586
 
 gene
 
 0.056648
 
 0.1365319
 
 Not queried
 
  1.4066854
 
 4.36e-01
 
 0.5589686471
  
 
  
 10
 
 131927345
 
 131927554
 
  PDF   STATS   ENSEMBL   UCSC 
 
  210
 
  4
 
 6272
 
 island
 
 0.256337
 
 0.5959698
 
 Not queried
 
  4.3571106
 
 4.86e-01
 
 0.5640816327
  
 
  
 20
 
   1294311
 
   1294376
 
  PDF   STATS   ENSEMBL   UCSC 
 
   66
 
  4
 
 25537
 
 tss
 
 0.004419
 
 0.0185784
 
 Not queried
 
  4.2819461
 
 4.86e-01
 
 0.5640816327
  
 
  
 1
 
  50574383
 
  50574591
 
  PDF   STATS   ENSEMBL   UCSC 
 
  209
 
  4
 
 524
 
 gene;tss
 
 0.203875
 
 0.3750304
 
 Not queried
 
  4.2041032
 
 4.86e-01
 
 0.5640816327
  
 
  
 9
 
 133589177
 
 133589654
 
  PDF   STATS   ENSEMBL   UCSC 
 
  478
 
  4
 
 40961
 
 island
 
 0.620226
 
 0.0945145
 
 Not queried
 
  4.1912734
 
 4.86e-01
 
 0.5640816327
  
 
  
 11
 
  75139390
 
  75139736
 
  PDF   STATS   ENSEMBL   UCSC 
 
  347
 
  4
 
 6835
 
 gene;island
 
 0.497799
 
 0.0584431
 
 Not queried
 
  4.1872825
 
 4.86e-01
 
 0.5640816327
  
 
  
 17
 
  77776428
 
  77776716
 
  PDF   STATS   ENSEMBL   UCSC 
 
  289
 
  4
 
 18843
 
 island
 
 0.010428
 
 0.0216421
 
 Not queried
 
  4.1163614
 
 4.86e-01
 
 0.5640816327
  
 
  
 15
 
  79164541
 
  79164980
 
  PDF   STATS   ENSEMBL   UCSC 
 
  440
 
  4
 
 13839
 
 tss;island
 
 0.295594
 
 0.0197964
 
 Not queried
 
  4.0555815
 
 4.86e-01
 
 0.5640816327
  
 
  
 3
 
   3885955
 
   3886273
 
  PDF   STATS   ENSEMBL   UCSC 
 
  319
 
  4
 
 27640
 
 gene
 
 0.008473
 
 0.1282886
 
 Not queried
 
  4.0172101
 
 4.86e-01
 
 0.5640816327
  
 
  
 19
 
  17791074
 
  17791354
 
  PDF   STATS   ENSEMBL   UCSC 
 
  281
 
  4
 
 19919
 
 gene;island
 
 0.027428
 
 0.0091032
 
 Not queried
 
  3.8129938
 
 4.86e-01
 
 0.5640816327
  
 
  
 19
 
  39694477
 
  39694831
 
  PDF   STATS   ENSEMBL   UCSC 
 
  355
 
  4
 
 20026
 
 gene
 
 0.053589
 
 0.1006625
 
 Not queried
 
  3.7263739
 
 4.86e-01
 
 0.5640816327
  
 
  
 5
 
 112849156
 
 112849285
 
  PDF   STATS   ENSEMBL   UCSC 
 
  130
 
  4
 
 32220
 
 tss
 
 0.005403
 
 0.1567673
 
 Not queried
 
  3.7131652
 
 4.86e-01
 
 0.5640816327
  
 
  
 7
 
  12610355
 
  12610833
 
  PDF   STATS   ENSEMBL   UCSC 
 
  479
 
  6
 
 36896
 
 gene
 
 0.033826
 
 0.0871635
 
 Not queried
 
  3.6027548
 
 4.85e-01
 
 0.5640816327
  
 
  
 17
 
  72438885
 
  72439217
 
  PDF   STATS   ENSEMBL   UCSC 
 
  333
 
  4
 
 16757
 
 gene
 
 0.078851
 
 0.2521795
 
 Not queried
 
  3.5524555
 
 4.86e-01
 
 0.5640816327
  
 
  
 19
 
  51529846
 
  51529999
 
  PDF   STATS   ENSEMBL   UCSC 
 
  154
 
  4
 
 20189
 
 gene
 
 0.231626
 
 0.4876721
 
 Not queried
 
  3.4258529
 
 4.86e-01
 
 0.5640816327
  
 
  
 7
 
  96649956
 
  96650668
 
  PDF   STATS   ENSEMBL   UCSC 
 
  713
 
  8
 
 37105
 
 gene;island
 
 0.015460
 
 0.0216470
 
 Not queried
 
  3.3981469
 
 4.42e-01
 
 0.5640816327
  
 
  
 11
 
   2029309
 
   2029688
 
  PDF   STATS   ENSEMBL   UCSC 
 
  380
 
  4
 
 7948
 
 island
 
 0.405414
 
 0.7116371
 
 Not queried
 
  3.3834073
 
 4.86e-01
 
 0.5640816327
  
 
  
 4
 
   2956627
 
   2957353
 
  PDF   STATS   ENSEMBL   UCSC 
 
  727
 
  6
 
 29897
 
 gene;island
 
 0.807849
 
 0.9760357
 
 Not queried
 
  3.3798162
 
 4.85e-01
 
 0.5640816327
  
 
  
 8
 
  68864769
 
  68864964
 
  PDF   STATS   ENSEMBL   UCSC 
 
  196
 
  4
 
 39328
 
 gene;island
 
 0.012225
 
 0.2578596
 
 Not queried
 
  3.2534677
 
 4.86e-01
 
 0.5640816327
  
 
  
 1
 
  35220224
 
  35220710
 
  PDF   STATS   ENSEMBL   UCSC 
 
  487
 
  6
 
 1478
 
 tss
 
 0.235004
 
 0.4929670
 
 Not queried
 
  3.2261894
 
 4.85e-01
 
 0.5640816327
  
 
  
 15
 
  89438795
 
  89438964
 
  PDF   STATS   ENSEMBL   UCSC 
 
  170
 
  4
 
 13882
 
 tss
 
 0.021979
 
 0.2056080
 
 Not queried
 
  3.1706893
 
 4.86e-01
 
 0.5640816327
  
 
  
 6
 
  32977400
 
  32977708
 
  PDF   STATS   ENSEMBL   UCSC 
 
  309
 
  4
 
 34886
 
 tss
 
 0.496310
 
 0.3063218
 
 Not queried
 
  3.1420846
 
 4.86e-01
 
 0.5640816327
  
 
  
 5
 
    181726
 
    182028
 
  PDF   STATS   ENSEMBL   UCSC 
 
  303
 
  4
 
 31496
 
 gene;island
 
 0.386328
 
 0.6009033
 
 Not queried
 
  3.1297997
 
 4.86e-01
 
 0.5640816327
  
 
  
 2
 
 232765174
 
 232765416
 
  PDF   STATS   ENSEMBL   UCSC 
 
  243
 
  4
 
 25189
 
 island
 
 0.707781
 
 0.0735826
 
 Not queried
 
  3.1082250
 
 4.86e-01
 
 0.5640816327
  
 
  
 19
 
  16939845
 
  16940140
 
  PDF   STATS   ENSEMBL   UCSC 
 
  296
 
  4
 
 20576
 
 tss
 
 0.003853
 
 0.0096927
 
 Not queried
 
  3.0777135
 
 4.86e-01
 
 0.5640816327
  
 
  
 17
 
  20687681
 
  20687912
 
  PDF   STATS   ENSEMBL   UCSC 
 
  232
 
  4
 
 18110
 
 island
 
 0.609177
 
 0.3486135
 
 Not queried
 
  3.0682352
 
 4.86e-01
 
 0.5640816327
  
 
  
 17
 
  64188403
 
  64188644
 
  PDF   STATS   ENSEMBL   UCSC 
 
  242
 
  4
 
 17595
 
 tss;island
 
 0.021051
 
 0.0071959
 
 Not queried
 
  3.0598319
 
 4.86e-01
 
 0.5640816327
  
 
  
 22
 
  32767005
 
  32767162
 
  PDF   STATS   ENSEMBL   UCSC 
 
  158
 
  4
 
 27091
 
 tss
 
 0.613961
 
 0.8685027
 
 Not queried
 
  3.0154898
 
 4.86e-01
 
 0.5640816327
  
 
  
 1
 
 223316634
 
 223316927
 
  PDF   STATS   ENSEMBL   UCSC 
 
  294
 
  4
 
 2269
 
 tss
 
 0.003709
 
 0.0143718
 
 Not queried
 
  3.0100924
 
 4.86e-01
 
 0.5640816327
  
 
  
 7
 
 127744210
 
 127744617
 
  PDF   STATS   ENSEMBL   UCSC 
 
  408
 
  4
 
 38890
 
 island
 
 0.025671
 
 0.0980616
 
 Not queried
 
  2.9927954
 
 4.86e-01
 
 0.5640816327
  
 
  
 6
 
  30076659
 
  30076980
 
  PDF   STATS   ENSEMBL   UCSC 
 
  322
 
  4
 
 33723
 
 gene
 
 0.156978
 
 0.3289770
 
 Not queried
 
  2.9889636
 
 4.86e-01
 
 0.5640816327
  
 
  
 11
 
 126872615
 
 126872863
 
  PDF   STATS   ENSEMBL   UCSC 
 
  249
 
  4
 
 8936
 
 island
 
 0.562546
 
 0.0689540
 
 Not queried
 
  2.9622494
 
 4.86e-01
 
 0.5640816327
  
 
  
 8
 
  24813866
 
  24814137
 
  PDF   STATS   ENSEMBL   UCSC 
 
  272
 
  4
 
 40162
 
 island
 
 0.010504
 
 0.0307879
 
 Not queried
 
  2.9608647
 
 4.86e-01
 
 0.5640816327
  
 
  
 6
 
  31800885
 
  31801070
 
  PDF   STATS   ENSEMBL   UCSC 
 
  186
 
  4
 
 35850
 
 island
 
 0.410065
 
 0.6323439
 
 Not queried
 
  2.9420429
 
 4.86e-01
 
 0.5640816327
  
 
  
 16
 
  49499006
 
  49499385
 
  PDF   STATS   ENSEMBL   UCSC 
 
  380
 
  4
 
 15908
 
 island
 
 0.321423
 
 0.6207172
 
 Not queried
 
  2.9403105
 
 4.86e-01
 
 0.5640816327
  
 
  
 2
 
 105478656
 
 105479054
 
  PDF   STATS   ENSEMBL   UCSC 
 
  399
 
  4
 
 24579
 
 island
 
 0.128924
 
 0.0292300
 
 Not queried
 
  2.9214295
 
 4.86e-01
 
 0.5640816327
  
 
  
 1
 
   2288167
 
   2288463
 
  PDF   STATS   ENSEMBL   UCSC 
 
  297
 
  4
 
 77
 
 gene
 
 0.361261
 
 0.5813857
 
 Not queried
 
  2.8957610
 
 4.86e-01
 
 0.5640816327
  
 
  
 5
 
 110847639
 
 110848106
 
  PDF   STATS   ENSEMBL   UCSC 
 
  468
 
  4
 
 31709
 
 gene
 
 0.005538
 
 0.0187524
 
 Not queried
 
  2.8886132
 
 4.86e-01
 
 0.5640816327
  
 
  
 1
 
  15250209
 
  15250388
 
  PDF   STATS   ENSEMBL   UCSC 
 
  180
 
  4
 
 239
 
 gene;tss;island
 
 0.058313
 
 0.2994698
 
 Not queried
 
  2.8830977
 
 4.86e-01
 
 0.5640816327
  
 
  
 1
 
  22470218
 
  22470343
 
  PDF   STATS   ENSEMBL   UCSC 
 
  126
 
  4
 
 1331
 
 tss;island
 
 0.015056
 
 0.2099592
 
 Not queried
 
  2.8632027
 
 4.86e-01
 
 0.5640816327
  
 
  
 6
 
  33132943
 
  33133083
 
  PDF   STATS   ENSEMBL   UCSC 
 
  141
 
  4
 
 34194
 
 gene;island
 
 0.441884
 
 0.7029882
 
 Not queried
 
  2.8544576
 
 4.86e-01
 
 0.5640816327
  
 
  
 14
 
  65569751
 
  65570008
 
  PDF   STATS   ENSEMBL   UCSC 
 
  258
 
  4
 
 12467
 
 tss;island
 
 0.001185
 
 0.0030038
 
 Not queried
 
  2.8397722
 
 4.86e-01
 
 0.5640816327
  
 
  
 22
 
  50968250
 
  50968516
 
  PDF   STATS   ENSEMBL   UCSC 
 
  267
 
  6
 
 27629
 
 island
 
 0.015136
 
 0.0079119
 
 Not queried
 
  2.8230756
 
 4.85e-01
 
 0.5640816327
  
 
  
 22
 
  51038379
 
  51038723
 
  PDF   STATS   ENSEMBL   UCSC 
 
  345
 
  4
 
 27262
 
 tss;island
 
 0.304639
 
 0.0552868
 
 Not queried
 
  2.8139218
 
 4.86e-01
 
 0.5640816327
  
 
  
 6
 
   4775063
 
   4775222
 
  PDF   STATS   ENSEMBL   UCSC 
 
  160
 
  4
 
 33541
 
 gene;island
 
 0.058020
 
 0.1985458
 
 Not queried
 
  2.8043444
 
 4.86e-01
 
 0.5640816327
  
 
  
 1
 
 204951031
 
 204951336
 
  PDF   STATS   ENSEMBL   UCSC 
 
  306
 
  4
 
 961
 
 gene;island
 
 0.244757
 
 0.4479910
 
 Not queried
 
  2.7633459
 
 4.86e-01
 
 0.5640816327
  
 
  
 1
 
  50886782
 
  50886969
 
  PDF   STATS   ENSEMBL   UCSC 
 
  188
 
  4
 
 525
 
 gene;island
 
 0.270326
 
 0.0509012
 
 Not queried
 
  2.7609274
 
 4.86e-01
 
 0.5640816327
  
 
  
 15
 
  51385812
 
  51385970
 
  PDF   STATS   ENSEMBL   UCSC 
 
  159
 
  4
 
 13348
 
 gene;island
 
 0.001523
 
 0.0055494
 
 Not queried
 
  2.7566142
 
 4.86e-01
 
 0.5640816327
  
 
  
 7
 
  44279049
 
  44279273
 
  PDF   STATS   ENSEMBL   UCSC 
 
  225
 
  4
 
 36992
 
 gene;island
 
 0.186311
 
 0.4695392
 
 Not queried
 
  2.7511037
 
 4.86e-01
 
 0.5640816327
  
 
  
 7
 
 158656429
 
 158656677
 
  PDF   STATS   ENSEMBL   UCSC 
 
  249
 
  4
 
 37380
 
 gene;island
 
 0.010792
 
 0.0016173
 
 Not queried
 
  2.7305202
 
 4.86e-01
 
 0.5640816327
  
 
  
 6
 
  29527870
 
  29528119
 
  PDF   STATS   ENSEMBL   UCSC 
 
  250
 
  4
 
 34748
 
 tss
 
 0.481707
 
 0.6903221
 
 Not queried
 
  2.7107858
 
 4.86e-01
 
 0.5640816327
  
 
  
 14
 
  52455805
 
  52456142
 
  PDF   STATS   ENSEMBL   UCSC 
 
  338
 
  4
 
 12394
 
 tss;island
 
 0.007331
 
 0.0059271
 
 Not queried
 
  2.7062910
 
 4.86e-01
 
 0.5640816327
  
 
  
 12
 
  46767665
 
  46768016
 
  PDF   STATS   ENSEMBL   UCSC 
 
  352
 
  6
 
 9658
 
 tss;island
 
 0.376921
 
 0.7629516
 
 Not queried
 
  2.6933741
 
 4.85e-01
 
 0.5640816327
  
 
  
 6
 
 106433792
 
 106434623
 
  PDF   STATS   ENSEMBL   UCSC 
 
  832
 
  8
 
 36378
 
 island
 
 0.156155
 
 0.0268787
 
 Not queried
 
  2.6732550
 
 4.42e-01
 
 0.5640816327
  
 
  
 1
 
  29240533
 
  29240705
 
  PDF   STATS   ENSEMBL   UCSC 
 
  173
 
  4
 
 374
 
 gene;tss;island
 
 0.672687
 
 0.2620181
 
 Not queried
 
  2.6711582
 
 4.86e-01
 
 0.5640816327
  
 
  
 11
 
  18230491
 
  18230799
 
  PDF   STATS   ENSEMBL   UCSC 
 
  309
 
  7
 
 7181
 
 tss
 
 0.547286
 
 0.8258070
 
 Not queried
 
  2.6706902
 
 4.56e-01
 
 0.5640816327
  
 
  
 19
 
  29455596
 
  29455913
 
  PDF   STATS   ENSEMBL   UCSC 
 
  318
 
  4
 
 20668
 
 tss
 
 0.029829
 
 0.3270932
 
 Not queried
 
  2.6503478
 
 4.86e-01
 
 0.5640816327
  
 
  
 9
 
  98784231
 
  98784441
 
  PDF   STATS   ENSEMBL   UCSC 
 
  211
 
  4
 
 40824
 
 tss;island
 
 0.026267
 
 0.2310009
 
 Not queried
 
  2.6297424
 
 4.86e-01
 
 0.5640816327
  
 
  
 14
 
  64319089
 
  64319306
 
  PDF   STATS   ENSEMBL   UCSC 
 
  218
 
  4
 
 12452
 
 tss;island
 
 0.422250
 
 0.4365582
 
 Not queried
 
  2.6229443
 
 4.86e-01
 
 0.5640816327
  
 
  
 2
 
 132558939
 
 132559214
 
  PDF   STATS   ENSEMBL   UCSC 
 
  276
 
  4
 
 22872
 
 gene
 
 0.328854
 
 0.5087209
 
 Not queried
 
  2.6212660
 
 4.86e-01
 
 0.5640816327
  
 
  
 11
 
  70257035
 
  70257280
 
  PDF   STATS   ENSEMBL   UCSC 
 
  246
 
  4
 
 6789
 
 gene;island
 
 0.629318
 
 0.0752362
 
 Not queried
 
  2.6005338
 
 4.86e-01
 
 0.5640816327
  
 
  
 6
 
  70991156
 
  70991283
 
  PDF   STATS   ENSEMBL   UCSC 
 
  128
 
  4
 
 34382
 
 gene;island
 
 0.375437
 
 0.6584076
 
 Not queried
 
  2.5850896
 
 4.86e-01
 
 0.5640816327
  
 
  
 18
 
  13801010
 
  13801271
 
  PDF   STATS   ENSEMBL   UCSC 
 
  262
 
  4
 
 19411
 
 island
 
 0.023820
 
 0.0448497
 
 Not queried
 
  2.5820546
 
 4.86e-01
 
 0.5640816327
  
 
  
 6
 
  30458519
 
  30458730
 
  PDF   STATS   ENSEMBL   UCSC 
 
  212
 
  4
 
 33769
 
 gene;island
 
 0.032566
 
 0.0752740
 
 Not queried
 
  2.5736440
 
 4.86e-01
 
 0.5640816327
  
 
  
 5
 
 140718283
 
 140718405
 
  PDF   STATS   ENSEMBL   UCSC 
 
  123
 
  4
 
 31816
 
 gene;island
 
 0.012394
 
 0.0646590
 
 Not queried
 
  2.5620872
 
 4.86e-01
 
 0.5640816327
  
 
  
 12
 
    678853
 
    679287
 
  PDF   STATS   ENSEMBL   UCSC 
 
  435
 
  4
 
 8996
 
 gene;island
 
 0.009031
 
 0.0195039
 
 Not queried
 
  2.5489156
 
 4.86e-01
 
 0.5640816327
  
 
  
 12
 
  79258313
 
  79258438
 
  PDF   STATS   ENSEMBL   UCSC 
 
  126
 
  4
 
 9225
 
 gene;tss
 
 0.007463
 
 0.0437722
 
 Not queried
 
  2.5208070
 
 4.86e-01
 
 0.5640816327
  
 
  
 1
 
 152487844
 
 152488368
 
  PDF   STATS   ENSEMBL   UCSC 
 
  525
 
  8
 
 758
 
 gene;island
 
 0.097672
 
 0.4485084
 
 Not queried
 
  2.5161100
 
 4.42e-01
 
 0.5640816327
  
 
  
 6
 
   7911240
 
   7911480
 
  PDF   STATS   ENSEMBL   UCSC 
 
  241
 
  4
 
 34617
 
 tss;island
 
 0.002912
 
 0.0178085
 
 Not queried
 
  2.5001369
 
 4.86e-01
 
 0.5640816327
  
 
  
 20
 
  31780882
 
  31781393
 
  PDF   STATS   ENSEMBL   UCSC 
 
  512
 
  7
 
 25680
 
 tss
 
 0.013543
 
 0.0182476
 
 Not queried
 
  2.4913221
 
 4.56e-01
 
 0.5640816327
  
 
  
 19
 
  11199851
 
  11199965
 
  PDF   STATS   ENSEMBL   UCSC 
 
  115
 
  6
 
 20464
 
 tss
 
 0.001274
 
 0.0037361
 
 Not queried
 
  2.4746899
 
 4.85e-01
 
 0.5640816327
  
 
  
 16
 
    202311
 
    202542
 
  PDF   STATS   ENSEMBL   UCSC 
 
  232
 
  4
 
 14961
 
 tss;island
 
 0.203292
 
 0.4525171
 
 Not queried
 
  2.4722308
 
 4.86e-01
 
 0.5640816327
  
 
  
 12
 
  85305677
 
  85306021
 
  PDF   STATS   ENSEMBL   UCSC 
 
  345
 
  4
 
 9231
 
 gene;island
 
 0.019523
 
 0.1345911
 
 Not queried
 
  2.4574755
 
 4.86e-01
 
 0.5640816327
  
 
  
 13
 
  25946657
 
  25947159
 
  PDF   STATS   ENSEMBL   UCSC 
 
  503
 
  4
 
 11109
 
 gene;island
 
 0.316825
 
 0.6119701
 
 Not queried
 
  2.4475603
 
 4.86e-01
 
 0.5640816327
  
 
  
 2
 
 145281942
 
 145282262
 
  PDF   STATS   ENSEMBL   UCSC 
 
  321
 
  4
 
 24771
 
 island
 
 0.052275
 
 0.0216455
 
 Not queried
 
  2.4448125
 
 4.86e-01
 
 0.5640816327
  
 
  
 5
 
 139944046
 
 139944115
 
  PDF   STATS   ENSEMBL   UCSC 
 
   70
 
  4
 
 31787
 
 gene
 
 0.004161
 
 0.0006765
 
 Not queried
 
  2.4405393
 
 4.86e-01
 
 0.5640816327
  
 
  
 2
 
 176993017
 
 176993320
 
  PDF   STATS   ENSEMBL   UCSC 
 
  304
 
  6
 
 23738
 
 tss;island
 
 0.024281
 
 0.2123277
 
 Not queried
 
  2.4378116
 
 4.85e-01
 
 0.5640816327
  
 
  
 16
 
  67562424
 
  67562672
 
  PDF   STATS   ENSEMBL   UCSC 
 
  249
 
  4
 
 15305
 
 tss;island
 
 0.030328
 
 0.1496989
 
 Not queried
 
  2.4208153
 
 4.86e-01
 
 0.5640816327
  
 
  
 6
 
 134209935
 
 134210257
 
  PDF   STATS   ENSEMBL   UCSC 
 
  323
 
  6
 
 35232
 
 tss
 
 0.039457
 
 0.3151910
 
 Not queried
 
  2.4142250
 
 4.85e-01
 
 0.5640816327
  
 
  
 10
 
  27609163
 
  27609333
 
  PDF   STATS   ENSEMBL   UCSC 
 
  171
 
  4
 
 5629
 
 island
 
 0.628307
 
 0.9364240
 
 Not queried
 
  2.3877553
 
 4.86e-01
 
 0.5640816327
  
 
  
 12
 
 107711872
 
 107712210
 
  PDF   STATS   ENSEMBL   UCSC 
 
  339
 
  6
 
 10761
 
 island
 
 0.015648
 
 0.0044481
 
 Not queried
 
  2.3797173
 
 4.85e-01
 
 0.5640816327
  
 
  
 1
 
    805338
 
    805554
 
  PDF   STATS   ENSEMBL   UCSC 
 
  217
 
  4
 
 1
 
 gene;island
 
 0.284768
 
 0.6287204
 
 Not queried
 
  2.3660271
 
 4.86e-01
 
 0.5640816327
  
 
  
 3
 
  64672542
 
  64672750
 
  PDF   STATS   ENSEMBL   UCSC 
 
  209
 
  4
 
 27851
 
 gene;island
 
 0.031098
 
 0.0095976
 
 Not queried
 
  2.3529802
 
 4.86e-01
 
 0.5640816327
  
 
  
 1
 
  16073336
 
  16073520
 
  PDF   STATS   ENSEMBL   UCSC 
 
  185
 
  4
 
 251
 
 gene;island
 
 0.736475
 
 0.9614973
 
 Not queried
 
  2.3478164
 
 4.86e-01
 
 0.5640816327
  
 
  
 6
 
  32178603
 
  32178775
 
  PDF   STATS   ENSEMBL   UCSC 
 
  173
 
  4
 
 34091
 
 gene
 
 0.545648
 
 0.8555073
 
 Not queried
 
  2.3438856
 
 4.86e-01
 
 0.5640816327
  
 
  
 2
 
 233390712
 
 233390859
 
  PDF   STATS   ENSEMBL   UCSC 
 
  148
 
  4
 
 23950
 
 tss
 
 0.028899
 
 0.3447050
 
 Not queried
 
  2.3384613
 
 4.86e-01
 
 0.5640816327
  
 
  
 21
 
  47064079
 
  47064462
 
  PDF   STATS   ENSEMBL   UCSC 
 
  384
 
  4
 
 26762
 
 island
 
 0.066667
 
 0.3104941
 
 Not queried
 
  2.3317531
 
 4.86e-01
 
 0.5640816327
  
 
  
 8
 
  25041651
 
  25041796
 
  PDF   STATS   ENSEMBL   UCSC 
 
  146
 
  4
 
 39606
 
 tss;island
 
 0.048215
 
 0.2439939
 
 Not queried
 
  2.3251996
 
 4.86e-01
 
 0.5640816327
  
 
  
 2
 
  38763130
 
  38763533
 
  PDF   STATS   ENSEMBL   UCSC 
 
  404
 
  4
 
 24230
 
 island
 
 0.022687
 
 0.0095600
 
 Not queried
 
  2.3137283
 
 4.86e-01
 
 0.5640816327
  
 
  
 1
 
  89357317
 
  89357426
 
  PDF   STATS   ENSEMBL   UCSC 
 
  110
 
  4
 
 1736
 
 tss
 
 0.001187
 
 0.0039948
 
 Not queried
 
  2.3055408
 
 4.86e-01
 
 0.5640816327
  
 
  
 6
 
 170190914
 
 170191082
 
  PDF   STATS   ENSEMBL   UCSC 
 
  169
 
  4
 
 34580
 
 gene;island
 
 0.605697
 
 0.7699543
 
 Not queried
 
  2.2976433
 
 4.86e-01
 
 0.5640816327
  
 
  
 4
 
   7940375
 
   7940579
 
  PDF   STATS   ENSEMBL   UCSC 
 
  205
 
  4
 
 29943
 
 gene;island
 
 0.147430
 
 0.0361974
 
 Not queried
 
  2.2818895
 
 4.86e-01
 
 0.5640816327
  
 
  
 1
 
   6419606
 
   6419906
 
  PDF   STATS   ENSEMBL   UCSC 
 
  301
 
  4
 
 170
 
 gene;tss
 
 0.006649
 
 0.1346306
 
 Not queried
 
  2.2800342
 
 4.86e-01
 
 0.5640816327
  
 
  
 13
 
  47126300
 
  47126551
 
  PDF   STATS   ENSEMBL   UCSC 
 
  252
 
  4
 
 11427
 
 tss;island
 
 0.439919
 
 0.2704923
 
 Not queried
 
  2.2794127
 
 4.86e-01
 
 0.5640816327
  
 
  
 11
 
  18477379
 
  18477753
 
  PDF   STATS   ENSEMBL   UCSC 
 
  375
 
  4
 
 6530
 
 gene
 
 0.891719
 
 0.7360134
 
 Not queried
 
  2.2757720
 
 4.86e-01
 
 0.5640816327
  
 
  
 17
 
  74868561
 
  74868725
 
  PDF   STATS   ENSEMBL   UCSC 
 
  165
 
  4
 
 16798
 
 gene;tss;island
 
 0.010349
 
 0.0260256
 
 Not queried
 
  2.2637400
 
 4.86e-01
 
 0.5640816327
  
 
  
 11
 
  65409760
 
  65410126
 
  PDF   STATS   ENSEMBL   UCSC 
 
  367
 
  4
 
 6715
 
 gene;island
 
 0.966808
 
 0.7309103
 
 Not queried
 
  2.2561698
 
 4.86e-01
 
 0.5640816327
  
 
  
 7
 
 141438205
 
 141438468
 
  PDF   STATS   ENSEMBL   UCSC 
 
  264
 
  4
 
 37252
 
 gene
 
 0.004090
 
 0.0022067
 
 Not queried
 
  2.2193843
 
 4.86e-01
 
 0.5640816327
  
 
  
 19
 
  11494980
 
  11495295
 
  PDF   STATS   ENSEMBL   UCSC 
 
  316
 
  4
 
 21566
 
 island
 
 0.381964
 
 0.0512136
 
 Not queried
 
  2.2169771
 
 4.86e-01
 
 0.5640816327
  
 
  
 1
 
  95006322
 
  95006977
 
  PDF   STATS   ENSEMBL   UCSC 
 
  656
 
  6
 
 643
 
 gene;island
 
 0.644840
 
 0.7562703
 
 Not queried
 
  2.2096813
 
 4.85e-01
 
 0.5640816327
  
 
  
 17
 
  36997420
 
  36997627
 
  PDF   STATS   ENSEMBL   UCSC 
 
  208
 
  4
 
 16542
 
 gene
 
 0.560683
 
 0.9525755
 
 Not queried
 
  2.1977183
 
 4.86e-01
 
 0.5640816327
  
 
  
 18
 
  63417605
 
  63417783
 
  PDF   STATS   ENSEMBL   UCSC 
 
  179
 
  4
 
 19122
 
 gene;tss;island
 
 0.031992
 
 0.1461462
 
 Not queried
 
  2.1940384
 
 4.86e-01
 
 0.5640816327
  
 
  
 3
 
 184243086
 
 184243411
 
  PDF   STATS   ENSEMBL   UCSC 
 
  326
 
  4
 
 29729
 
 island
 
 0.003756
 
 0.0582942
 
 Not queried
 
  2.1916788
 
 4.86e-01
 
 0.5640816327
  
 
  
 11
 
  20044428
 
  20044639
 
  PDF   STATS   ENSEMBL   UCSC 
 
  212
 
  4
 
 6536
 
 gene
 
 0.530973
 
 0.8696106
 
 Not queried
 
  2.1888775
 
 4.86e-01
 
 0.5640816327
  
 
  
 8
 
  88884985
 
  88885254
 
  PDF   STATS   ENSEMBL   UCSC 
 
  270
 
  4
 
 39355
 
 gene;island
 
 0.180071
 
 0.5007843
 
 Not queried
 
  2.1843986
 
 4.86e-01
 
 0.5640816327
  
 
  
 17
 
   6347867
 
   6348200
 
  PDF   STATS   ENSEMBL   UCSC 
 
  334
 
  6
 
 16372
 
 gene
 
 0.346826
 
 0.0201331
 
 Not queried
 
  2.1767197
 
 4.85e-01
 
 0.5640816327
  
 
  
 8
 
 144387646
 
 144387935
 
  PDF   STATS   ENSEMBL   UCSC 
 
  290
 
  4
 
 40653
 
 island
 
 0.563774
 
 0.8541781
 
 Not queried
 
  2.1718998
 
 4.86e-01
 
 0.5640816327
  
 
  
 3
 
 123167507
 
 123167770
 
  PDF   STATS   ENSEMBL   UCSC 
 
  264
 
  4
 
 28545
 
 tss;island
 
 0.220817
 
 0.0230532
 
 Not queried
 
  2.1545667
 
 4.86e-01
 
 0.5640816327
  
 
  
 3
 
  50283852
 
  50284305
 
  PDF   STATS   ENSEMBL   UCSC 
 
  454
 
  4
 
 27797
 
 gene;tss
 
 0.145245
 
 0.0196805
 
 Not queried
 
  2.1463046
 
 4.86e-01
 
 0.5640816327
  
 
  
 6
 
  30644681
 
  30644832
 
  PDF   STATS   ENSEMBL   UCSC 
 
  152
 
  4
 
 33799
 
 gene;island
 
 0.841471
 
 0.6575656
 
 Not queried
 
  2.1211667
 
 4.86e-01
 
 0.5640816327
  
 
  
 8
 
 110656982
 
 110657197
 
  PDF   STATS   ENSEMBL   UCSC 
 
  216
 
  6
 
 39400
 
 gene;island
 
 0.002576
 
 0.0016554
 
 Not queried
 
  2.1159527
 
 4.85e-01
 
 0.5640816327
  
 
  
 2
 
 235859315
 
 235859792
 
  PDF   STATS   ENSEMBL   UCSC 
 
  478
 
  4
 
 23962
 
 tss;island
 
 0.094492
 
 0.3375256
 
 Not queried
 
  2.1092284
 
 4.86e-01
 
 0.5640816327
  
 
  
 5
 
  54518469
 
  54519307
 
  PDF   STATS   ENSEMBL   UCSC 
 
  839
 
  7
 
 32800
 
 island
 
 0.047573
 
 0.0241057
 
 Not queried
 
  2.1036057
 
 4.56e-01
 
 0.5640816327
  
 
  
 2
 
  54342584
 
  54342961
 
  PDF   STATS   ENSEMBL   UCSC 
 
  378
 
  4
 
 22682
 
 gene;island
 
 0.001841
 
 0.0029276
 
 Not queried
 
  2.1030324
 
 4.86e-01
 
 0.5640816327
  
 
  
 3
 
  69915189
 
  69915449
 
  PDF   STATS   ENSEMBL   UCSC 
 
  261
 
  4
 
 27856
 
 gene
 
 0.086543
 
 0.2457305
 
 Not queried
 
  2.1016910
 
 4.86e-01
 
 0.5640816327
  
 
  
 4
 
  74864596
 
  74864725
 
  PDF   STATS   ENSEMBL   UCSC 
 
  130
 
  4
 
 30432
 
 tss;island
 
 0.091118
 
 0.3762809
 
 Not queried
 
  2.0904702
 
 4.86e-01
 
 0.5640816327
  
 
  
 5
 
  83678796
 
  83679364
 
  PDF   STATS   ENSEMBL   UCSC 
 
  569
 
  6
 
 31671
 
 gene;island
 
 0.005226
 
 0.0994709
 
 Not queried
 
  2.0884650
 
 4.85e-01
 
 0.5640816327
  
 
  
 6
 
  30295772
 
  30296049
 
  PDF   STATS   ENSEMBL   UCSC 
 
  278
 
  4
 
 33760
 
 gene;tss;island
 
 0.851052
 
 0.9796837
 
 Not queried
 
  2.0861008
 
 4.86e-01
 
 0.5640816327
  
 
  
 10
 
 131767960
 
 131768149
 
  PDF   STATS   ENSEMBL   UCSC 
 
  190
 
  4
 
 6268
 
 island
 
 0.273958
 
 0.0385239
 
 Not queried
 
  2.0840479
 
 4.86e-01
 
 0.5640816327
  
 
  
 19
 
  49340489
 
  49340765
 
  PDF   STATS   ENSEMBL   UCSC 
 
  277
 
  4
 
 20138
 
 gene;tss;island
 
 0.052480
 
 0.0293126
 
 Not queried
 
  2.0821918
 
 4.86e-01
 
 0.5640816327
  
 
  
 1
 
 162792092
 
 162792613
 
  PDF   STATS   ENSEMBL   UCSC 
 
  522
 
  6
 
 3921
 
 island
 
 0.275288
 
 0.0272740
 
 Not queried
 
  2.0637124
 
 4.85e-01
 
 0.5640816327
  
 
  
 1
 
  43250691
 
  43251253
 
  PDF   STATS   ENSEMBL   UCSC 
 
  563
 
  6
 
 3151
 
 island
 
 0.450425
 
 0.0769018
 
 Not queried
 
  2.0616055
 
 4.85e-01
 
 0.5640816327
  
 
  
 7
 
  18126902
 
  18127112
 
  PDF   STATS   ENSEMBL   UCSC 
 
  211
 
  4
 
 38280
 
 island
 
 0.345258
 
 0.7608913
 
 Not queried
 
  2.0605580
 
 4.86e-01
 
 0.5640816327
  
 
  
 22
 
  32600722
 
  32601274
 
  PDF   STATS   ENSEMBL   UCSC 
 
  553
 
  4
 
 27089
 
 tss
 
 0.409130
 
 0.6777338
 
 Not queried
 
  2.0518940
 
 4.86e-01
 
 0.5640816327
  
 
  
 17
 
  34838488
 
  34838831
 
  PDF   STATS   ENSEMBL   UCSC 
 
  344
 
  4
 
 18226
 
 island
 
 0.372556
 
 0.1393394
 
 Not queried
 
  2.0499025
 
 4.86e-01
 
 0.5640816327
  
 
  
 22
 
  47081634
 
  47082044
 
  PDF   STATS   ENSEMBL   UCSC 
 
  411
 
  4
 
 26915
 
 gene;island
 
 0.811746
 
 0.9707444
 
 Not queried
 
  2.0471502
 
 4.86e-01
 
 0.5640816327
  
 
  
 11
 
 130029478
 
 130029615
 
  PDF   STATS   ENSEMBL   UCSC 
 
  138
 
  4
 
 7817
 
 tss
 
 0.231744
 
 0.1369278
 
 Not queried
 
  2.0468037
 
 4.86e-01
 
 0.5640816327
  
 
  
 12
 
 121570677
 
 121571068
 
  PDF   STATS   ENSEMBL   UCSC 
 
  392
 
  4
 
 9362
 
 gene
 
 0.051569
 
 0.0277278
 
 Not queried
 
  2.0369613
 
 4.86e-01
 
 0.5640816327
  
 
  
 22
 
  21386798
 
  21387058
 
  PDF   STATS   ENSEMBL   UCSC 
 
  261
 
  8
 
 27330
 
 island
 
 0.128035
 
 0.0262249
 
 Not queried
 
  2.0272382
 
 4.42e-01
 
 0.5640816327
  
 
  
 3
 
  26663551
 
  26663885
 
  PDF   STATS   ENSEMBL   UCSC 
 
  335
 
  6
 
 28194
 
 tss;island
 
 0.109079
 
 0.3296150
 
 Not queried
 
  2.0185634
 
 4.85e-01
 
 0.5640816327
  
 
  
 3
 
 133392794
 
 133393240
 
  PDF   STATS   ENSEMBL   UCSC 
 
  447
 
  4
 
 29490
 
 island
 
 0.271582
 
 0.4833957
 
 Not queried
 
  2.0160667
 
 4.86e-01
 
 0.5640816327
  
 
  
 12
 
  46123553
 
  46123850
 
  PDF   STATS   ENSEMBL   UCSC 
 
  298
 
  4
 
 10345
 
 island
 
 0.009375
 
 0.0072977
 
 Not queried
 
  2.0015854
 
 4.86e-01
 
 0.5640816327
  
 
  
 5
 
 140797172
 
 140797286
 
  PDF   STATS   ENSEMBL   UCSC 
 
  115
 
  4
 
 31833
 
 gene;island
 
 0.025098
 
 0.0903849
 
 Not queried
 
  1.9997617
 
 4.86e-01
 
 0.5640816327
  
 
  
 16
 
  54318044
 
  54318314
 
  PDF   STATS   ENSEMBL   UCSC 
 
  271
 
  4
 
 14751
 
 gene;island
 
 0.033533
 
 0.2020581
 
 Not queried
 
  1.9931527
 
 4.86e-01
 
 0.5640816327
  
 
  
 12
 
  54409207
 
  54409770
 
  PDF   STATS   ENSEMBL   UCSC 
 
  564
 
 12
 
 9753
 
 tss;island
 
 0.005438
 
 0.0109554
 
 Not queried
 
  1.9924049
 
 4.43e-01
 
 0.5640816327
  
 
  
 1
 
   8086588
 
   8086959
 
  PDF   STATS   ENSEMBL   UCSC 
 
  372
 
  4
 
 1220
 
 tss;island
 
 0.116567
 
 0.3539901
 
 Not queried
 
  1.9915501
 
 4.86e-01
 
 0.5640816327
  
 
  
 2
 
 186603398
 
 186603639
 
  PDF   STATS   ENSEMBL   UCSC 
 
  242
 
  4
 
 24947
 
 island
 
 0.040808
 
 0.0985260
 
 Not queried
 
  1.9834072
 
 4.86e-01
 
 0.5640816327
  
 
  
 1
 
 117664976
 
 117665186
 
  PDF   STATS   ENSEMBL   UCSC 
 
  211
 
  4
 
 1869
 
 tss;island
 
 0.520311
 
 0.2272376
 
 Not queried
 
  1.9816128
 
 4.86e-01
 
 0.5640816327
  
 
  
 5
 
   1253833
 
   1254048
 
  PDF   STATS   ENSEMBL   UCSC 
 
  216
 
  4
 
 31529
 
 gene;island
 
 0.838629
 
 0.4377513
 
 Not queried
 
  1.9808676
 
 4.86e-01
 
 0.5640816327
  
 
  
 22
 
  19712623
 
  19712750
 
  PDF   STATS   ENSEMBL   UCSC 
 
  128
 
  4
 
 27302
 
 island
 
 0.659141
 
 0.8430542
 
 Not queried
 
  1.9790568
 
 4.86e-01
 
 0.5640816327
  
 
  
 1
 
 156022836
 
 156023044
 
  PDF   STATS   ENSEMBL   UCSC 
 
  209
 
  4
 
 812
 
 gene;island
 
 0.003933
 
 0.0039620
 
 Not queried
 
  1.9723494
 
 4.86e-01
 
 0.5640816327
  
 
  
 19
 
  11465809
 
  11466020
 
  PDF   STATS   ENSEMBL   UCSC 
 
  212
 
  4
 
 20469
 
 tss
 
 0.030276
 
 0.0216669
 
 Not queried
 
  1.9661192
 
 4.86e-01
 
 0.5640816327
  
 
  
 3
 
 197463810
 
 197464306
 
  PDF   STATS   ENSEMBL   UCSC 
 
  497
 
  7
 
 28823
 
 tss
 
 0.001835
 
 0.0016086
 
 Not queried
 
  1.9613947
 
 4.56e-01
 
 0.5640816327
  
 
  
 10
 
 126289835
 
 126290192
 
  PDF   STATS   ENSEMBL   UCSC 
 
  358
 
  4
 
 4807
 
 gene
 
 0.045040
 
 0.2204544
 
 Not queried
 
  1.9558631
 
 4.86e-01
 
 0.5640816327
  
 
  
 13
 
  27333803
 
  27334259
 
  PDF   STATS   ENSEMBL   UCSC 
 
  457
 
  4
 
 11113
 
 gene;island
 
 0.028303
 
 0.0644026
 
 Not queried
 
  1.9541423
 
 4.86e-01
 
 0.5640816327
  
 
  
 5
 
  68485086
 
  68485282
 
  PDF   STATS   ENSEMBL   UCSC 
 
  197
 
  4
 
 32106
 
 tss
 
 0.008264
 
 0.1440488
 
 Not queried
 
  1.9498651
 
 4.86e-01
 
 0.5640816327
  
 
  
 7
 
 157502370
 
 157502620
 
  PDF   STATS   ENSEMBL   UCSC 
 
  251
 
  4
 
 37340
 
 gene;island
 
 0.248548
 
 0.4086604
 
 Not queried
 
  1.9359463
 
 4.86e-01
 
 0.5640816327
  
 
  
 15
 
  85114131
 
  85114479
 
  PDF   STATS   ENSEMBL   UCSC 
 
  349
 
  6
 
 13867
 
 tss
 
 0.014012
 
 0.0106960
 
 Not queried
 
  1.9225031
 
 4.85e-01
 
 0.5640816327
  
 
  
 15
 
  81616310
 
  81616489
 
  PDF   STATS   ENSEMBL   UCSC 
 
  180
 
  4
 
 13451
 
 gene
 
 0.002737
 
 0.0032577
 
 Not queried
 
  1.9059049
 
 4.86e-01
 
 0.5640816327
  
 
  
 1
 
  46767565
 
  46767971
 
  PDF   STATS   ENSEMBL   UCSC 
 
  407
 
  4
 
 511
 
 gene;island
 
 0.005071
 
 0.0060037
 
 Not queried
 
  1.9023588
 
 4.86e-01
 
 0.5640816327
  
 
  
 3
 
  72788183
 
  72788482
 
  PDF   STATS   ENSEMBL   UCSC 
 
  300
 
  4
 
 29282
 
 island
 
 0.028150
 
 0.0474910
 
 Not queried
 
  1.8991635
 
 4.86e-01
 
 0.5640816327
  
 
  
 19
 
  41018757
 
  41018880
 
  PDF   STATS   ENSEMBL   UCSC 
 
  124
 
  4
 
 20043
 
 gene;island
 
 0.332625
 
 0.1421107
 
 Not queried
 
  1.8987120
 
 4.86e-01
 
 0.5640816327
  
 
  
 22
 
  46659317
 
  46659595
 
  PDF   STATS   ENSEMBL   UCSC 
 
  279
 
  4
 
 27232
 
 tss;island
 
 0.815085
 
 0.9345517
 
 Not queried
 
  1.8951312
 
 4.86e-01
 
 0.5640816327
  
 
  
 15
 
  73735509
 
  73735694
 
  PDF   STATS   ENSEMBL   UCSC 
 
  186
 
  4
 
 13412
 
 gene
 
 0.610617
 
 0.7840795
 
 Not queried
 
  1.8913154
 
 4.86e-01
 
 0.5640816327
  
 
  
 20
 
  62037503
 
  62037758
 
  PDF   STATS   ENSEMBL   UCSC 
 
  256
 
  4
 
 26352
 
 island
 
 0.497636
 
 0.7951329
 
 Not queried
 
  1.8782886
 
 4.86e-01
 
 0.5640816327
  
 
  
 20
 
   9819697
 
   9819820
 
  PDF   STATS   ENSEMBL   UCSC 
 
  124
 
  6
 
 25593
 
 tss;island
 
 0.024536
 
 0.0128838
 
 Not queried
 
  1.8780558
 
 4.85e-01
 
 0.5640816327
  
 
  
 6
 
  99276697
 
  99277015
 
  PDF   STATS   ENSEMBL   UCSC 
 
  319
 
  4
 
 36341
 
 island
 
 0.251051
 
 0.0063921
 
 Not queried
 
  1.8740542
 
 4.86e-01
 
 0.5640816327
  
 
  
 5
 
 177659919
 
 177660036
 
  PDF   STATS   ENSEMBL   UCSC 
 
  118
 
  4
 
 32539
 
 tss;island
 
 0.020673
 
 0.0906450
 
 Not queried
 
  1.8706182
 
 4.86e-01
 
 0.5640816327
  
 
  
 5
 
   5287980
 
   5288296
 
  PDF   STATS   ENSEMBL   UCSC 
 
  317
 
  6
 
 31552
 
 gene
 
 0.745754
 
 0.7781827
 
 Not queried
 
  1.8654305
 
 4.85e-01
 
 0.5640816327
  
 
  
 12
 
  52627272
 
  52627576
 
  PDF   STATS   ENSEMBL   UCSC 
 
  305
 
  4
 
 9135
 
 gene;island
 
 0.086764
 
 0.3699897
 
 Not queried
 
  1.8638874
 
 4.86e-01
 
 0.5640816327
  
 
  
 17
 
  73642404
 
  73642628
 
  PDF   STATS   ENSEMBL   UCSC 
 
  225
 
  6
 
 17658
 
 tss
 
 0.064437
 
 0.0204908
 
 Not queried
 
  1.8598313
 
 4.85e-01
 
 0.5640816327
  
 
  
 17
 
  17109651
 
  17110353
 
  PDF   STATS   ENSEMBL   UCSC 
 
  703
 
 12
 
 17162
 
 tss
 
 0.379419
 
 0.6042759
 
 Not queried
 
  1.8578554
 
 4.43e-01
 
 0.5640816327
  
 
  
 8
 
  42751637
 
  42751762
 
  PDF   STATS   ENSEMBL   UCSC 
 
  126
 
  4
 
 39299
 
 gene;tss;island
 
 0.010777
 
 0.0083481
 
 Not queried
 
  1.8537500
 
 4.86e-01
 
 0.5640816327
  
 
  
 11
 
 128556611
 
 128556992
 
  PDF   STATS   ENSEMBL   UCSC 
 
  382
 
  4
 
 8939
 
 island
 
 0.039086
 
 0.0155537
 
 Not queried
 
  1.8511844
 
 4.86e-01
 
 0.5640816327
  
 
  
 11
 
  79148183
 
  79148548
 
  PDF   STATS   ENSEMBL   UCSC 
 
  366
 
  4
 
 6849
 
 gene;island
 
 0.041789
 
 0.1015135
 
 Not queried
 
  1.8489810
 
 4.86e-01
 
 0.5640816327
  
 
  
 19
 
  41106155
 
  41106351
 
  PDF   STATS   ENSEMBL   UCSC 
 
  197
 
  4
 
 20046
 
 gene;tss;island
 
 0.008111
 
 0.0120234
 
 Not queried
 
  1.8464189
 
 4.86e-01
 
 0.5640816327
  
 
  
 11
 
  72982929
 
  72983097
 
  PDF   STATS   ENSEMBL   UCSC 
 
  169
 
  4
 
 6820
 
 gene;tss
 
 0.888310
 
 0.4418872
 
 Not queried
 
  1.8439709
 
 4.86e-01
 
 0.5640816327
  
 
  
 19
 
  10735482
 
  10735594
 
  PDF   STATS   ENSEMBL   UCSC 
 
  113
 
  4
 
 19817
 
 gene;tss;island
 
 0.920014
 
 0.6214295
 
 Not queried
 
  1.8410825
 
 4.86e-01
 
 0.5640816327
  
 
  
 15
 
  53075421
 
  53075877
 
  PDF   STATS   ENSEMBL   UCSC 
 
  457
 
  6
 
 13357
 
 gene;island
 
 0.326869
 
 0.0422130
 
 Not queried
 
  1.8401212
 
 4.85e-01
 
 0.5640816327
  
 
  
 10
 
  72201484
 
  72201741
 
  PDF   STATS   ENSEMBL   UCSC 
 
  258
 
  6
 
 5135
 
 tss
 
 0.287560
 
 0.2084869
 
 Not queried
 
  1.8374411
 
 4.85e-01
 
 0.5640816327
  
 
  
 6
 
  99278991
 
  99279108
 
  PDF   STATS   ENSEMBL   UCSC 
 
  118
 
  4
 
 36342
 
 island
 
 0.081619
 
 0.2129319
 
 Not queried
 
  1.8364347
 
 4.86e-01
 
 0.5640816327
  
 
  
 17
 
  41832753
 
  41833324
 
  PDF   STATS   ENSEMBL   UCSC 
 
  572
 
  6
 
 16598
 
 gene;island
 
 0.083519
 
 0.1316803
 
 Not queried
 
  1.8300691
 
 4.85e-01
 
 0.5640816327
  
 
  
 6
 
  99283553
 
  99284027
 
  PDF   STATS   ENSEMBL   UCSC 
 
  475
 
  4
 
 34420
 
 gene;island
 
 0.118845
 
 0.1668224
 
 Not queried
 
  1.8300654
 
 4.86e-01
 
 0.5640816327
  
 
  
 11
 
   2593857
 
   2594153
 
  PDF   STATS   ENSEMBL   UCSC 
 
  297
 
  4
 
 6463
 
 gene;island
 
 0.324374
 
 0.4709794
 
 Not queried
 
  1.8277525
 
 4.86e-01
 
 0.5640816327
  
 
  
 3
 
  49907414
 
  49907692
 
  PDF   STATS   ENSEMBL   UCSC 
 
  279
 
  6
 
 28338
 
 tss
 
 0.034503
 
 0.1669478
 
 Not queried
 
  1.8227419
 
 4.85e-01
 
 0.5640816327
  
 
  
 19
 
  20162642
 
  20163138
 
  PDF   STATS   ENSEMBL   UCSC 
 
  497
 
  7
 
 21833
 
 island
 
 0.017399
 
 0.0054683
 
 Not queried
 
  1.8213285
 
 4.56e-01
 
 0.5640816327
  
 
  
 17
 
  79412583
 
  79412788
 
  PDF   STATS   ENSEMBL   UCSC 
 
  206
 
  4
 
 16910
 
 gene;island
 
 0.535508
 
 0.7217751
 
 Not queried
 
  1.8205545
 
 4.86e-01
 
 0.5640816327
  
 
  
 10
 
  98946673
 
  98946943
 
  PDF   STATS   ENSEMBL   UCSC 
 
  271
 
  4
 
 5258
 
 tss;island
 
 0.499068
 
 0.7029724
 
 Not queried
 
  1.8198446
 
 4.86e-01
 
 0.5640816327
  
 
  
 20
 
  56803778
 
  56804327
 
  PDF   STATS   ENSEMBL   UCSC 
 
  550
 
  4
 
 26287
 
 island
 
 0.048081
 
 0.0946797
 
 Not queried
 
  1.8154281
 
 4.86e-01
 
 0.5640816327
  
 
  
 17
 
  79632335
 
  79632662
 
  PDF   STATS   ENSEMBL   UCSC 
 
  328
 
  4
 
 17744
 
 tss
 
 0.924178
 
 0.5626375
 
 Not queried
 
  1.8135666
 
 4.86e-01
 
 0.5640816327
  
 
  
 6
 
  42145851
 
  42146143
 
  PDF   STATS   ENSEMBL   UCSC 
 
  293
 
  4
 
 34338
 
 gene;island
 
 0.420015
 
 0.0662211
 
 Not queried
 
  1.8101651
 
 4.86e-01
 
 0.5640816327
  
 
  
 11
 
   2183708
 
   2183864
 
  PDF   STATS   ENSEMBL   UCSC 
 
  157
 
  4
 
 7062
 
 tss;island
 
 0.551106
 
 0.8090281
 
 Not queried
 
  1.8096536
 
 4.86e-01
 
 0.5640816327
  
 
  
 5
 
  83679941
 
  83680326
 
  PDF   STATS   ENSEMBL   UCSC 
 
  386
 
  4
 
 31672
 
 gene;island
 
 0.031451
 
 0.1010048
 
 Not queried
 
  1.8037358
 
 4.86e-01
 
 0.5640816327
  
 
  
 1
 
 183385831
 
 183386237
 
  PDF   STATS   ENSEMBL   UCSC 
 
  407
 
  4
 
 915
 
 gene;island
 
 0.124721
 
 0.3605279
 
 Not queried
 
  1.8018780
 
 4.86e-01
 
 0.5640816327
  
 
  
 12
 
 113587240
 
 113587690
 
  PDF   STATS   ENSEMBL   UCSC 
 
  451
 
  6
 
 10827
 
 island
 
 0.079638
 
 0.3508295
 
 Not queried
 
  1.8017356
 
 4.85e-01
 
 0.5640816327
  
 
  
 8
 
   1365502
 
   1365906
 
  PDF   STATS   ENSEMBL   UCSC 
 
  405
 
  4
 
 40024
 
 island
 
 0.341273
 
 0.5012772
 
 Not queried
 
  1.7995277
 
 4.86e-01
 
 0.5640816327
  
 
  
 14
 
  94255361
 
  94255513
 
  PDF   STATS   ENSEMBL   UCSC 
 
  153
 
  4
 
 12576
 
 tss;island
 
 0.022678
 
 0.0425318
 
 Not queried
 
  1.7960073
 
 4.86e-01
 
 0.5640816327
  
 
  
 11
 
   2323801
 
   2323938
 
  PDF   STATS   ENSEMBL   UCSC 
 
  138
 
  4
 
 6455
 
 gene;tss
 
 0.028306
 
 0.1069675
 
 Not queried
 
  1.7958284
 
 4.86e-01
 
 0.5640816327
  
 
  
 17
 
  72208213
 
  72208504
 
  PDF   STATS   ENSEMBL   UCSC 
 
  292
 
  4
 
 16748
 
 gene;tss;island
 
 0.476178
 
 0.7604797
 
 Not queried
 
  1.7873048
 
 4.86e-01
 
 0.5640816327
  
 
  
 8
 
 124214953
 
 124215152
 
  PDF   STATS   ENSEMBL   UCSC 
 
  200
 
  4
 
 39418
 
 gene
 
 0.474803
 
 0.7502735
 
 Not queried
 
  1.7852112
 
 4.86e-01
 
 0.5640816327
  
 
  
 1
 
  27189270
 
  27189440
 
  PDF   STATS   ENSEMBL   UCSC 
 
  171
 
  4
 
 1399
 
 tss;island
 
 0.491915
 
 0.0938689
 
 Not queried
 
  1.7840869
 
 4.86e-01
 
 0.5640816327
  
 
  
 11
 
 134147143
 
 134147634
 
  PDF   STATS   ENSEMBL   UCSC 
 
  492
 
  4
 
 6994
 
 gene;island
 
 0.015154
 
 0.3378675
 
 Not queried
 
  1.7840207
 
 4.86e-01
 
 0.5640816327
  
 
  
 3
 
  49907297
 
  49907692
 
  PDF   STATS   ENSEMBL   UCSC 
 
  396
 
  7
 
 29132
 
 island
 
 0.035726
 
 0.1155753
 
 Not queried
 
  1.7834365
 
 4.56e-01
 
 0.5640816327
  
 
  
 7
 
   1278142
 
   1278508
 
  PDF   STATS   ENSEMBL   UCSC 
 
  367
 
  4
 
 38093
 
 island
 
 0.074833
 
 0.2232519
 
 Not queried
 
  1.7772011
 
 4.86e-01
 
 0.5640816327
  
 
  
 14
 
  62229147
 
  62229330
 
  PDF   STATS   ENSEMBL   UCSC 
 
  184
 
  4
 
 12108
 
 gene
 
 0.012309
 
 0.0190787
 
 Not queried
 
  1.7735778
 
 4.86e-01
 
 0.5640816327
  
 
  
 13
 
 115079815
 
 115079979
 
  PDF   STATS   ENSEMBL   UCSC 
 
  165
 
  4
 
 12002
 
 island
 
 0.001078
 
 0.0033042
 
 Not queried
 
  1.7666036
 
 4.86e-01
 
 0.5640816327
  
 
  
 19
 
  13985513
 
  13985688
 
  PDF   STATS   ENSEMBL   UCSC 
 
  176
 
  4
 
 19885
 
 gene
 
 0.632594
 
 0.7821732
 
 Not queried
 
  1.7659434
 
 4.86e-01
 
 0.5640816327
  
 
  
 17
 
   8090466
 
   8090696
 
  PDF   STATS   ENSEMBL   UCSC 
 
  231
 
  4
 
 17995
 
 island
 
 0.007110
 
 0.0045152
 
 Not queried
 
  1.7614611
 
 4.86e-01
 
 0.5640816327
  
 
  
 5
 
 168174689
 
 168174935
 
  PDF   STATS   ENSEMBL   UCSC 
 
  247
 
  4
 
 31885
 
 gene
 
 0.417254
 
 0.6873336
 
 Not queried
 
  1.7533408
 
 4.86e-01
 
 0.5640816327
  
 
  
 10
 
 118033581
 
 118034031
 
  PDF   STATS   ENSEMBL   UCSC 
 
  451
 
  4
 
 5383
 
 tss;island
 
 0.027019
 
 0.0415705
 
 Not queried
 
  1.7516378
 
 4.86e-01
 
 0.5640816327
  
 
  
 1
 
   3426236
 
   3426532
 
  PDF   STATS   ENSEMBL   UCSC 
 
  297
 
  4
 
 127
 
 gene;island
 
 0.351326
 
 0.7245160
 
 Not queried
 
  1.7506502
 
 4.86e-01
 
 0.5640816327
  
 
  
 2
 
   6121909
 
   6122107
 
  PDF   STATS   ENSEMBL   UCSC 
 
  199
 
  4
 
 23165
 
 tss
 
 0.038584
 
 0.0180359
 
 Not queried
 
  1.7476492
 
 4.86e-01
 
 0.5640816327
  
 
  
 12
 
   5018715
 
   5019091
 
  PDF   STATS   ENSEMBL   UCSC 
 
  377
 
 10
 
 10180
 
 island
 
 0.028015
 
 0.0344179
 
 Not queried
 
  1.7403809
 
 4.81e-01
 
 0.5640816327
  
 
  
 17
 
  29159410
 
  29159713
 
  PDF   STATS   ENSEMBL   UCSC 
 
  304
 
  4
 
 16509
 
 gene;island
 
 0.000870
 
 0.0022094
 
 Not queried
 
  1.7364636
 
 4.86e-01
 
 0.5640816327
  
 
  
 5
 
 130330787
 
 130331091
 
  PDF   STATS   ENSEMBL   UCSC 
 
  305
 
  4
 
 33076
 
 island
 
 0.280539
 
 0.5918140
 
 Not queried
 
  1.7352371
 
 4.86e-01
 
 0.5640816327
  
 
  
 14
 
  23589066
 
  23589419
 
  PDF   STATS   ENSEMBL   UCSC 
 
  354
 
  4
 
 12297
 
 tss
 
 0.343921
 
 0.8301865
 
 Not queried
 
  1.7330868
 
 4.86e-01
 
 0.5640816327
  
 
  
 5
 
    480033
 
    480412
 
  PDF   STATS   ENSEMBL   UCSC 
 
  380
 
  4
 
 31506
 
 gene;island
 
 0.737933
 
 0.8110294
 
 Not queried
 
  1.7323900
 
 4.86e-01
 
 0.5640816327
  
 
  
 3
 
 185826362
 
 185826590
 
  PDF   STATS   ENSEMBL   UCSC 
 
  229
 
  4
 
 28071
 
 gene;island
 
 0.007523
 
 0.0032581
 
 Not queried
 
  1.7297463
 
 4.86e-01
 
 0.5640816327
  
 
  
 12
 
  13197360
 
  13197827
 
  PDF   STATS   ENSEMBL   UCSC 
 
  468
 
  4
 
 9063
 
 gene
 
 0.003882
 
 0.0022492
 
 Not queried
 
  1.7287257
 
 4.86e-01
 
 0.5640816327
  
 
  
 6
 
 160783785
 
 160784044
 
  PDF   STATS   ENSEMBL   UCSC 
 
  260
 
  4
 
 34535
 
 gene;island
 
 0.333180
 
 0.5998887
 
 Not queried
 
  1.7268610
 
 4.86e-01
 
 0.5640816327
  
 
  
 11
 
  67203360
 
  67203661
 
  PDF   STATS   ENSEMBL   UCSC 
 
  302
 
  4
 
 6755
 
 gene;island
 
 0.250840
 
 0.4374744
 
 Not queried
 
  1.7265718
 
 4.86e-01
 
 0.5640816327
  
 
  
 7
 
 102985332
 
 102985587
 
  PDF   STATS   ENSEMBL   UCSC 
 
  256
 
  4
 
 37812
 
 tss
 
 0.496223
 
 0.6764612
 
 Not queried
 
  1.7257211
 
 4.86e-01
 
 0.5640816327
  
 
  
 20
 
    749264
 
    749620
 
  PDF   STATS   ENSEMBL   UCSC 
 
  357
 
  6
 
 25529
 
 tss
 
 0.055311
 
 0.0950342
 
 Not queried
 
  1.7232157
 
 4.85e-01
 
 0.5640816327
  
 
  
 19
 
  57683687
 
  57683885
 
  PDF   STATS   ENSEMBL   UCSC 
 
  199
 
  4
 
 22459
 
 island
 
 0.095000
 
 0.0420101
 
 Not queried
 
  1.7183137
 
 4.86e-01
 
 0.5640816327
  
 
  
 4
 
  83483204
 
  83483487
 
  PDF   STATS   ENSEMBL   UCSC 
 
  284
 
  6
 
 30467
 
 tss;island
 
 0.082663
 
 0.0320432
 
 Not queried
 
  1.7177263
 
 4.85e-01
 
 0.5640816327
  
 
  
 5
 
  41510742
 
  41510851
 
  PDF   STATS   ENSEMBL   UCSC 
 
  110
 
  4
 
 32037
 
 tss
 
 0.171907
 
 0.0554017
 
 Not queried
 
  1.7170998
 
 4.86e-01
 
 0.5640816327
  
 
  
 6
 
 143999465
 
 143999715
 
  PDF   STATS   ENSEMBL   UCSC 
 
  251
 
  4
 
 34497
 
 gene;island
 
 0.050084
 
 0.0072726
 
 Not queried
 
  1.7158278
 
 4.86e-01
 
 0.5640816327
  
 
  
 2
 
  10916238
 
  10916392
 
  PDF   STATS   ENSEMBL   UCSC 
 
  155
 
  4
 
 22570
 
 gene
 
 0.722911
 
 0.9591740
 
 Not queried
 
  1.7124503
 
 4.86e-01
 
 0.5640816327
  
 
  
 10
 
   1095446
 
   1095578
 
  PDF   STATS   ENSEMBL   UCSC 
 
  133
 
  4
 
 4913
 
 tss;island
 
 0.017587
 
 0.0054466
 
 Not queried
 
  1.7092800
 
 4.86e-01
 
 0.5640816327
  
 
  
 6
 
  11232933
 
  11233125
 
  PDF   STATS   ENSEMBL   UCSC 
 
  193
 
  4
 
 34636
 
 tss
 
 0.003450
 
 0.0017765
 
 Not queried
 
  1.7079015
 
 4.86e-01
 
 0.5640816327
  
 
  
 7
 
 109600399
 
 109600733
 
  PDF   STATS   ENSEMBL   UCSC 
 
  335
 
  4
 
 37837
 
 tss
 
 0.134877
 
 0.4096239
 
 Not queried
 
  1.7028994
 
 4.86e-01
 
 0.5640816327
  
 
  
 4
 
  20702045
 
  20702377
 
  PDF   STATS   ENSEMBL   UCSC 
 
  333
 
  4
 
 29964
 
 gene
 
 0.004226
 
 0.0014937
 
 Not queried
 
  1.7026780
 
 4.86e-01
 
 0.5640816327
  
 
  
 2
 
 142889283
 
 142889392
 
  PDF   STATS   ENSEMBL   UCSC 
 
  110
 
  4
 
 23653
 
 tss;island
 
 0.407117
 
 0.7376485
 
 Not queried
 
  1.6971022
 
 4.86e-01
 
 0.5640816327
  
 
  
 7
 
   4911022
 
   4911224
 
  PDF   STATS   ENSEMBL   UCSC 
 
  203
 
  4
 
 36863
 
 gene
 
 0.209745
 
 0.4387077
 
 Not queried
 
  1.6922137
 
 4.86e-01
 
 0.5640816327
  
 
  
 5
 
 140734543
 
 140734758
 
  PDF   STATS   ENSEMBL   UCSC 
 
  216
 
  4
 
 32375
 
 tss
 
 0.167934
 
 0.4279464
 
 Not queried
 
  1.6892038
 
 4.86e-01
 
 0.5640816327
  
 
  
 8
 
  63776508
 
  63776762
 
  PDF   STATS   ENSEMBL   UCSC 
 
  255
 
  4
 
 39319
 
 gene
 
 0.736104
 
 0.5185909
 
 Not queried
 
  1.6867298
 
 4.86e-01
 
 0.5640816327
  
 
  
 3
 
 140769610
 
 140770013
 
  PDF   STATS   ENSEMBL   UCSC 
 
  404
 
  4
 
 28636
 
 tss;island
 
 0.039906
 
 0.0588921
 
 Not queried
 
  1.6840801
 
 4.86e-01
 
 0.5640816327
  
 
  
 1
 
  53608037
 
  53608280
 
  PDF   STATS   ENSEMBL   UCSC 
 
  244
 
  4
 
 537
 
 gene
 
 0.375922
 
 0.5857823
 
 Not queried
 
  1.6823127
 
 4.86e-01
 
 0.5640816327
  
 
  
 14
 
  21493964
 
  21494269
 
  PDF   STATS   ENSEMBL   UCSC 
 
  306
 
  6
 
 12280
 
 tss;island
 
 0.482364
 
 0.0849839
 
 Not queried
 
  1.6766231
 
 4.85e-01
 
 0.5640816327
  
 
  
 20
 
  30795553
 
  30795610
 
  PDF   STATS   ENSEMBL   UCSC 
 
   58
 
  6
 
 25673
 
 tss;island
 
 0.000803
 
 0.0024303
 
 Not queried
 
  1.6764815
 
 4.85e-01
 
 0.5640816327
  
 
  
 16
 
    783597
 
    783916
 
  PDF   STATS   ENSEMBL   UCSC 
 
  320
 
  4
 
 14543
 
 gene;island
 
 0.657425
 
 0.8308091
 
 Not queried
 
  1.6748437
 
 4.86e-01
 
 0.5640816327
  
 
  
 1
 
 228112806
 
 228113207
 
  PDF   STATS   ENSEMBL   UCSC 
 
  402
 
  6
 
 1044
 
 gene;island
 
 0.423476
 
 0.4505143
 
 Not queried
 
  1.6747236
 
 4.85e-01
 
 0.5640816327
  
 
  
 11
 
   2950725
 
   2950754
 
  PDF   STATS   ENSEMBL   UCSC 
 
   30
 
  4
 
 7087
 
 tss
 
 0.012418
 
 0.0095879
 
 Not queried
 
  1.6726266
 
 4.86e-01
 
 0.5640816327
  
 
  
 3
 
 158519410
 
 158519645
 
  PDF   STATS   ENSEMBL   UCSC 
 
  236
 
  6
 
 28688
 
 tss
 
 0.023381
 
 0.0110608
 
 Not queried
 
  1.6721619
 
 4.85e-01
 
 0.5640816327
  
 
  
 19
 
   1650224
 
   1650452
 
  PDF   STATS   ENSEMBL   UCSC 
 
  229
 
  4
 
 19660
 
 gene;island
 
 0.498450
 
 0.7138461
 
 Not queried
 
  1.6622364
 
 4.86e-01
 
 0.5640816327
  
 
  
 7
 
  25893749
 
  25894005
 
  PDF   STATS   ENSEMBL   UCSC 
 
  257
 
  4
 
 38323
 
 island
 
 0.054444
 
 0.2865402
 
 Not queried
 
  1.6569211
 
 4.86e-01
 
 0.5640816327
  
 
  
 3
 
 126707103
 
 126707501
 
  PDF   STATS   ENSEMBL   UCSC 
 
  399
 
  4
 
 29438
 
 island
 
 0.647708
 
 0.8648309
 
 Not queried
 
  1.6552351
 
 4.86e-01
 
 0.5640816327
  
 
  
 16
 
  67219152
 
  67219218
 
  PDF   STATS   ENSEMBL   UCSC 
 
   67
 
  4
 
 14792
 
 gene;tss;island
 
 0.478653
 
 0.3030164
 
 Not queried
 
  1.6545048
 
 4.86e-01
 
 0.5640816327
  
 
  
 1
 
    713921
 
    714177
 
  PDF   STATS   ENSEMBL   UCSC 
 
  257
 
  6
 
 2376
 
 island
 
 0.013496
 
 0.0097380
 
 Not queried
 
  1.6539419
 
 4.85e-01
 
 0.5640816327
  
 
  
 6
 
  30882641
 
  30882708
 
  PDF   STATS   ENSEMBL   UCSC 
 
   68
 
  4
 
 33837
 
 gene;island
 
 0.109780
 
 0.1702758
 
 Not queried
 
  1.6486602
 
 4.86e-01
 
 0.5640816327
  
 
  
 9
 
  34370781
 
  34370894
 
  PDF   STATS   ENSEMBL   UCSC 
 
  114
 
  4
 
 40760
 
 gene;island
 
 0.803533
 
 0.9665674
 
 Not queried
 
  1.6469891
 
 4.86e-01
 
 0.5640816327
  
 
  
 5
 
  72530105
 
  72530245
 
  PDF   STATS   ENSEMBL   UCSC 
 
  141
 
  4
 
 32867
 
 island
 
 0.019250
 
 0.0624046
 
 Not queried
 
  1.6370303
 
 4.86e-01
 
 0.5640816327
  
 
  
 6
 
 111136481
 
 111136640
 
  PDF   STATS   ENSEMBL   UCSC 
 
  160
 
  4
 
 35171
 
 tss
 
 0.003147
 
 0.0033969
 
 Not queried
 
  1.6356578
 
 4.86e-01
 
 0.5640816327
  
 
  
 12
 
  93096622
 
  93096819
 
  PDF   STATS   ENSEMBL   UCSC 
 
  198
 
  4
 
 9913
 
 tss
 
 0.006525
 
 0.0055683
 
 Not queried
 
  1.6352854
 
 4.86e-01
 
 0.5640816327
  
 
  
 4
 
  77997182
 
  77997201
 
  PDF   STATS   ENSEMBL   UCSC 
 
   20
 
  4
 
 30452
 
 tss;island
 
 0.002788
 
 0.0024533
 
 Not queried
 
  1.6272831
 
 4.86e-01
 
 0.5640816327
  
 
  
 22
 
  30116919
 
  30117159
 
  PDF   STATS   ENSEMBL   UCSC 
 
  241
 
  4
 
 26834
 
 gene;island
 
 0.447388
 
 0.2127058
 
 Not queried
 
  1.6245846
 
 4.86e-01
 
 0.5640816327
  
 
  
 3
 
  38664022
 
  38664451
 
  PDF   STATS   ENSEMBL   UCSC 
 
  430
 
  4
 
 27722
 
 gene
 
 0.232488
 
 0.3453286
 
 Not queried
 
  1.6215795
 
 4.86e-01
 
 0.5640816327
  
 
  
 13
 
 111773936
 
 111774127
 
  PDF   STATS   ENSEMBL   UCSC 
 
  192
 
  4
 
 11242
 
 gene
 
 0.672408
 
 0.8373891
 
 Not queried
 
  1.6209692
 
 4.86e-01
 
 0.5640816327
  
 
  
 19
 
  35085512
 
  35085677
 
  PDF   STATS   ENSEMBL   UCSC 
 
  166
 
  4
 
 20693
 
 tss
 
 0.508526
 
 0.7718167
 
 Not queried
 
  1.6079274
 
 4.86e-01
 
 0.5640816327
  
 
  
 10
 
 132910868
 
 132911152
 
  PDF   STATS   ENSEMBL   UCSC 
 
  285
 
  4
 
 4841
 
 gene
 
 0.451153
 
 0.6717339
 
 Not queried
 
  1.6070250
 
 4.86e-01
 
 0.5640816327
  
 
  
 8
 
  18666531
 
  18666598
 
  PDF   STATS   ENSEMBL   UCSC 
 
   68
 
  4
 
 39220
 
 gene;tss
 
 0.285265
 
 0.4283312
 
 Not queried
 
  1.6054804
 
 4.86e-01
 
 0.5640816327
  
 
  
 8
 
 144653855
 
 144653975
 
  PDF   STATS   ENSEMBL   UCSC 
 
  121
 
  4
 
 39484
 
 gene;island
 
 0.012274
 
 0.0185735
 
 Not queried
 
  1.6016500
 
 4.86e-01
 
 0.5640816327
  
 
  
 7
 
  48495072
 
  48495333
 
  PDF   STATS   ENSEMBL   UCSC 
 
  262
 
  4
 
 37008
 
 gene;island
 
 0.553786
 
 0.7216637
 
 Not queried
 
  1.5996871
 
 4.86e-01
 
 0.5640816327
  
 
  
 12
 
  57569768
 
  57569940
 
  PDF   STATS   ENSEMBL   UCSC 
 
  173
 
  4
 
 9183
 
 gene;island
 
 0.493196
 
 0.8487071
 
 Not queried
 
  1.5983886
 
 4.86e-01
 
 0.5640816327
  
 
  
 2
 
 223155841
 
 223156136
 
  PDF   STATS   ENSEMBL   UCSC 
 
  296
 
  4
 
 23044
 
 gene;island
 
 0.207621
 
 0.0518450
 
 Not queried
 
  1.5963415
 
 4.86e-01
 
 0.5640816327
  
 
  
 14
 
  29254530
 
  29255119
 
  PDF   STATS   ENSEMBL   UCSC 
 
  590
 
  6
 
 12055
 
 gene;island
 
 0.178437
 
 0.0726748
 
 Not queried
 
  1.5922020
 
 4.85e-01
 
 0.5640816327
  
 
  
 2
 
  11924984
 
  11925375
 
  PDF   STATS   ENSEMBL   UCSC 
 
  392
 
  4
 
 22575
 
 gene;island
 
 0.478383
 
 0.3126958
 
 Not queried
 
  1.5902937
 
 4.86e-01
 
 0.5640816327
  
 
  
 12
 
  49110427
 
  49110646
 
  PDF   STATS   ENSEMBL   UCSC 
 
  220
 
  4
 
 9113
 
 gene
 
 0.005141
 
 0.0077721
 
 Not queried
 
  1.5859862
 
 4.86e-01
 
 0.5640816327
  
 
  
 1
 
 206729034
 
 206729258
 
  PDF   STATS   ENSEMBL   UCSC 
 
  225
 
  4
 
 971
 
 gene;tss;island
 
 0.378528
 
 0.6699104
 
 Not queried
 
  1.5831364
 
 4.86e-01
 
 0.5640816327
  
 
  
 17
 
  15686294
 
  15686473
 
  PDF   STATS   ENSEMBL   UCSC 
 
  180
 
  4
 
 18043
 
 island
 
 0.053614
 
 0.1354625
 
 Not queried
 
  1.5703018
 
 4.86e-01
 
 0.5640816327
  
 
  
 2
 
 109746691
 
 109746754
 
  PDF   STATS   ENSEMBL   UCSC 
 
   64
 
  4
 
 22823
 
 gene;tss;island
 
 0.803016
 
 0.5890672
 
 Not queried
 
  1.5679375
 
 4.86e-01
 
 0.5640816327
  
 
  
 1
 
  39281613
 
  39282003
 
  PDF   STATS   ENSEMBL   UCSC 
 
  391
 
  4
 
 3091
 
 island
 
 0.039820
 
 0.0130205
 
 Not queried
 
  1.5672098
 
 4.86e-01
 
 0.5640816327
  
 
  
 12
 
 104609609
 
 104610064
 
  PDF   STATS   ENSEMBL   UCSC 
 
  456
 
  4
 
 9271
 
 gene
 
 0.113607
 
 0.0375411
 
 Not queried
 
  1.5629248
 
 4.86e-01
 
 0.5640816327
  
 
  
 11
 
  82444946
 
  82445170
 
  PDF   STATS   ENSEMBL   UCSC 
 
  225
 
  4
 
 7594
 
 tss
 
 0.176924
 
 0.3822536
 
 Not queried
 
  1.5602631
 
 4.86e-01
 
 0.5640816327
  
 
  
 21
 
  45773189
 
  45773400
 
  PDF   STATS   ENSEMBL   UCSC 
 
  212
 
  4
 
 26584
 
 tss
 
 0.028778
 
 0.2094148
 
 Not queried
 
  1.5569509
 
 4.86e-01
 
 0.5640816327
  
 
  
 11
 
 113953462
 
 113953838
 
  PDF   STATS   ENSEMBL   UCSC 
 
  377
 
  4
 
 6910
 
 gene;island
 
 0.015509
 
 0.1320434
 
 Not queried
 
  1.5557471
 
 4.86e-01
 
 0.5640816327
  
 
  
 14
 
  77737661
 
  77737800
 
  PDF   STATS   ENSEMBL   UCSC 
 
  140
 
  4
 
 12529
 
 tss
 
 0.371830
 
 0.2705120
 
 Not queried
 
  1.5464485
 
 4.86e-01
 
 0.5640816327
  
 
  
 2
 
  71205052
 
  71205564
 
  PDF   STATS   ENSEMBL   UCSC 
 
  513
 
  6
 
 23424
 
 tss
 
 0.044843
 
 0.0123538
 
 Not queried
 
  1.5438305
 
 4.85e-01
 
 0.5640816327
  
 
  
 16
 
    711015
 
    711235
 
  PDF   STATS   ENSEMBL   UCSC 
 
  221
 
  4
 
 14535
 
 gene;island
 
 0.120263
 
 0.3100079
 
 Not queried
 
  1.5396964
 
 4.86e-01
 
 0.5640816327
  
 
  
 2
 
 112944644
 
 112944999
 
  PDF   STATS   ENSEMBL   UCSC 
 
  356
 
  4
 
 22828
 
 gene;island
 
 0.881126
 
 0.9267742
 
 Not queried
 
  1.5391141
 
 4.86e-01
 
 0.5640816327
  
 
  
 12
 
   6716557
 
   6716597
 
  PDF   STATS   ENSEMBL   UCSC 
 
   41
 
  4
 
 9518
 
 tss;island
 
 0.009914
 
 0.0041204
 
 Not queried
 
  1.5348720
 
 4.86e-01
 
 0.5640816327
  
 
  
 8
 
  23082233
 
  23082634
 
  PDF   STATS   ENSEMBL   UCSC 
 
  402
 
  4
 
 39240
 
 gene
 
 0.006254
 
 0.0090790
 
 Not queried
 
  1.5296781
 
 4.86e-01
 
 0.5640816327
  
 
  
 12
 
 101603835
 
 101603996
 
  PDF   STATS   ENSEMBL   UCSC 
 
  162
 
  4
 
 9267
 
 gene
 
 0.140394
 
 0.0370625
 
 Not queried
 
  1.5256277
 
 4.86e-01
 
 0.5640816327
  
 
  
 6
 
 108496716
 
 108497141
 
  PDF   STATS   ENSEMBL   UCSC 
 
  426
 
  4
 
 34441
 
 gene;island
 
 0.063030
 
 0.2494617
 
 Not queried
 
  1.5255428
 
 4.86e-01
 
 0.5640816327
  
 
  
 4
 
  90031538
 
  90031969
 
  PDF   STATS   ENSEMBL   UCSC 
 
  432
 
  4
 
 31177
 
 island
 
 0.329812
 
 0.0191679
 
 Not queried
 
  1.5202773
 
 4.86e-01
 
 0.5640816327
  
 
  
 11
 
  65547881
 
  65548028
 
  PDF   STATS   ENSEMBL   UCSC 
 
  148
 
  6
 
 7440
 
 tss
 
 0.009439
 
 0.0072226
 
 Not queried
 
  1.5184258
 
 4.85e-01
 
 0.5640816327
  
 
  
 13
 
 113663206
 
 113663538
 
  PDF   STATS   ENSEMBL   UCSC 
 
  333
 
  4
 
 11268
 
 gene;island
 
 0.887724
 
 0.9583249
 
 Not queried
 
  1.5167465
 
 4.86e-01
 
 0.5640816327
  
 
  
 4
 
 174451954
 
 174452159
 
  PDF   STATS   ENSEMBL   UCSC 
 
  206
 
  4
 
 30180
 
 gene;tss;island
 
 0.049725
 
 0.1573947
 
 Not queried
 
  1.5162248
 
 4.86e-01
 
 0.5640816327
  
 
  
 5
 
 171711430
 
 171711562
 
  PDF   STATS   ENSEMBL   UCSC 
 
  133
 
  4
 
 32492
 
 tss;island
 
 0.002711
 
 0.0018095
 
 Not queried
 
  1.5147422
 
 4.86e-01
 
 0.5640816327
  
 
  
 1
 
 150898437
 
 150898761
 
  PDF   STATS   ENSEMBL   UCSC 
 
  325
 
  8
 
 1914
 
 tss;island
 
 0.007350
 
 0.0108492
 
 Not queried
 
  1.5098376
 
 4.42e-01
 
 0.5640816327
  
 
  
 1
 
  27817084
 
  27817404
 
  PDF   STATS   ENSEMBL   UCSC 
 
  321
 
  4
 
 1414
 
 tss;island
 
 0.043834
 
 0.0201731
 
 Not queried
 
  1.5072888
 
 4.86e-01
 
 0.5640816327
  
 
  
 15
 
  90319420
 
  90319584
 
  PDF   STATS   ENSEMBL   UCSC 
 
  165
 
  4
 
 13892
 
 tss
 
 0.125766
 
 0.2931458
 
 Not queried
 
  1.5065816
 
 4.86e-01
 
 0.5640816327
  
 
  
 19
 
  13365923
 
  13366101
 
  PDF   STATS   ENSEMBL   UCSC 
 
  179
 
  4
 
 19880
 
 gene;island
 
 0.935064
 
 0.9600017
 
 Not queried
 
  1.5023033
 
 4.86e-01
 
 0.5640816327
  
 
  
 16
 
   6068831
 
   6069047
 
  PDF   STATS   ENSEMBL   UCSC 
 
  217
 
  4
 
 15080
 
 tss
 
 0.030967
 
 0.0594160
 
 Not queried
 
  1.4957224
 
 4.86e-01
 
 0.5640816327
  
 
  
 3
 
  50376000
 
  50376475
 
  PDF   STATS   ENSEMBL   UCSC 
 
  476
 
  6
 
 27806
 
 gene;tss;island
 
 0.549080
 
 0.7158266
 
 Not queried
 
  1.4931689
 
 4.85e-01
 
 0.5640816327
  
 
  
 19
 
  18451410
 
  18451650
 
  PDF   STATS   ENSEMBL   UCSC 
 
  241
 
  4
 
 19933
 
 gene
 
 0.009268
 
 0.0056198
 
 Not queried
 
  1.4928643
 
 4.86e-01
 
 0.5640816327
  
 
  
 17
 
   8027830
 
   8027961
 
  PDF   STATS   ENSEMBL   UCSC 
 
  132
 
  4
 
 17108
 
 tss;island
 
 0.023412
 
 0.0149956
 
 Not queried
 
  1.4927282
 
 4.86e-01
 
 0.5640816327
  
 
  
 3
 
 185655795
 
 185656289
 
  PDF   STATS   ENSEMBL   UCSC 
 
  495
 
  6
 
 29744
 
 island
 
 0.013540
 
 0.0231935
 
 Not queried
 
  1.4919540
 
 4.85e-01
 
 0.5640816327
  
 
  
 4
 
  54232368
 
  54232638
 
  PDF   STATS   ENSEMBL   UCSC 
 
  271
 
  4
 
 30394
 
 tss
 
 0.003184
 
 0.0084904
 
 Not queried
 
  1.4916888
 
 4.86e-01
 
 0.5640816327
  
 
  
 1
 
   6484616
 
   6484980
 
  PDF   STATS   ENSEMBL   UCSC 
 
  365
 
  4
 
 2605
 
 island
 
 0.323861
 
 0.0673099
 
 Not queried
 
  1.4898077
 
 4.86e-01
 
 0.5640816327
  
 
  
 7
 
 121651766
 
 121652024
 
  PDF   STATS   ENSEMBL   UCSC 
 
  259
 
  4
 
 37204
 
 gene
 
 0.006618
 
 0.0201150
 
 Not queried
 
  1.4867358
 
 4.86e-01
 
 0.5640816327
  
 
  
 16
 
  89440878
 
  89441217
 
  PDF   STATS   ENSEMBL   UCSC 
 
  340
 
  4
 
 14942
 
 gene;island
 
 0.966104
 
 0.8455548
 
 Not queried
 
  1.4853191
 
 4.86e-01
 
 0.5640816327
  
 
  
 6
 
  30124214
 
  30124299
 
  PDF   STATS   ENSEMBL   UCSC 
 
   86
 
  4
 
 33731
 
 gene
 
 0.539759
 
 0.7524369
 
 Not queried
 
  1.4826138
 
 4.86e-01
 
 0.5640816327
  
 
  
 7
 
  36700744
 
  36701016
 
  PDF   STATS   ENSEMBL   UCSC 
 
  273
 
  4
 
 36966
 
 gene
 
 0.254250
 
 0.4621867
 
 Not queried
 
  1.4811558
 
 4.86e-01
 
 0.5640816327
  
 
  
 6
 
   3445256
 
   3445330
 
  PDF   STATS   ENSEMBL   UCSC 
 
   75
 
  4
 
 33537
 
 gene;tss
 
 0.267669
 
 0.4253230
 
 Not queried
 
  1.4797021
 
 4.86e-01
 
 0.5640816327
  
 
  
 15
 
  78631512
 
  78631652
 
  PDF   STATS   ENSEMBL   UCSC 
 
  141
 
  4
 
 13833
 
 tss;island
 
 0.495456
 
 0.6371056
 
 Not queried
 
  1.4788365
 
 4.86e-01
 
 0.5640816327
  
 
  
 16
 
   2479181
 
   2479406
 
  PDF   STATS   ENSEMBL   UCSC 
 
  226
 
  4
 
 15603
 
 island
 
 0.002507
 
 0.0078826
 
 Not queried
 
  1.4784801
 
 4.86e-01
 
 0.5640816327
  
 
  
 17
 
  76564972
 
  76565232
 
  PDF   STATS   ENSEMBL   UCSC 
 
  261
 
  4
 
 16824
 
 gene
 
 0.111065
 
 0.2705387
 
 Not queried
 
  1.4777201
 
 4.86e-01
 
 0.5640816327
  
 
  
 6
 
  32119616
 
  32119691
 
  PDF   STATS   ENSEMBL   UCSC 
 
   76
 
  4
 
 34059
 
 gene
 
 0.100237
 
 0.1603067
 
 Not queried
 
  1.4760835
 
 4.86e-01
 
 0.5640816327
  
 
  
 17
 
   7909776
 
   7910040
 
  PDF   STATS   ENSEMBL   UCSC 
 
  265
 
  4
 
 16423
 
 gene;island
 
 0.717170
 
 0.9203362
 
 Not queried
 
  1.4727377
 
 4.86e-01
 
 0.5640816327
  
 
  
 16
 
    731772
 
    732216
 
  PDF   STATS   ENSEMBL   UCSC 
 
  445
 
  4
 
 14537
 
 gene;island
 
 0.736006
 
 0.9092615
 
 Not queried
 
  1.4718429
 
 4.86e-01
 
 0.5640816327
  
 
  
 13
 
 113120945
 
 113121280
 
  PDF   STATS   ENSEMBL   UCSC 
 
  336
 
  4
 
 11923
 
 island
 
 0.838592
 
 0.9336456
 
 Not queried
 
  1.4712211
 
 4.86e-01
 
 0.5640816327
  
 
  
 4
 
 183721565
 
 183721778
 
  PDF   STATS   ENSEMBL   UCSC 
 
  214
 
  4
 
 30193
 
 gene;island
 
 0.651527
 
 0.8944040
 
 Not queried
 
  1.4705368
 
 4.86e-01
 
 0.5640816327
  
 
  
 5
 
  95159365
 
  95159639
 
  PDF   STATS   ENSEMBL   UCSC 
 
  275
 
  4
 
 32187
 
 tss
 
 0.070576
 
 0.4502314
 
 Not queried
 
  1.4668276
 
 4.86e-01
 
 0.5640816327
  
 
  
 17
 
  46894191
 
  46894465
 
  PDF   STATS   ENSEMBL   UCSC 
 
  275
 
  4
 
 16668
 
 gene
 
 0.024477
 
 0.0113202
 
 Not queried
 
  1.4662675
 
 4.86e-01
 
 0.5640816327
  
 
  
 17
 
  32483882
 
  32484259
 
  PDF   STATS   ENSEMBL   UCSC 
 
  378
 
 10
 
 17258
 
 tss
 
 0.026378
 
 0.0913390
 
 Not queried
 
  1.4607511
 
 4.81e-01
 
 0.5640816327
  
 
  
 5
 
 140254793
 
 140255069
 
  PDF   STATS   ENSEMBL   UCSC 
 
  277
 
  4
 
 31802
 
 gene;island
 
 0.110291
 
 0.2927317
 
 Not queried
 
  1.4606439
 
 4.86e-01
 
 0.5640816327
  
 
  
 22
 
  17956453
 
  17956560
 
  PDF   STATS   ENSEMBL   UCSC 
 
  108
 
  4
 
 26943
 
 tss
 
 0.902672
 
 0.7194816
 
 Not queried
 
  1.4579805
 
 4.86e-01
 
 0.5640816327
  
 
  
 8
 
  25898191
 
  25898539
 
  PDF   STATS   ENSEMBL   UCSC 
 
  349
 
  4
 
 39250
 
 gene;island
 
 0.039931
 
 0.1061560
 
 Not queried
 
  1.4563795
 
 4.86e-01
 
 0.5640816327
  
 
  
 19
 
  17137685
 
  17137714
 
  PDF   STATS   ENSEMBL   UCSC 
 
   30
 
  4
 
 20578
 
 tss
 
 0.002796
 
 0.0122255
 
 Not queried
 
  1.4537877
 
 4.86e-01
 
 0.5640816327
  
 
  
 5
 
 178368071
 
 178368205
 
  PDF   STATS   ENSEMBL   UCSC 
 
  135
 
  6
 
 32545
 
 tss
 
 0.365431
 
 0.1778000
 
 Not queried
 
  1.4503030
 
 4.85e-01
 
 0.5640816327
  
 
  
 8
 
  65284438
 
  65284519
 
  PDF   STATS   ENSEMBL   UCSC 
 
   82
 
  4
 
 40340
 
 island
 
 0.012882
 
 0.0719369
 
 Not queried
 
  1.4499479
 
 4.86e-01
 
 0.5640816327
  
 
  
 17
 
  46827458
 
  46827626
 
  PDF   STATS   ENSEMBL   UCSC 
 
  169
 
  4
 
 18507
 
 island
 
 0.102544
 
 0.0070173
 
 Not queried
 
  1.4441757
 
 4.86e-01
 
 0.5640816327
  
 
  
 2
 
  60812505
 
  60812753
 
  PDF   STATS   ENSEMBL   UCSC 
 
  249
 
  4
 
 24322
 
 island
 
 0.622732
 
 0.8225839
 
 Not queried
 
  1.4422157
 
 4.86e-01
 
 0.5640816327
  
 
  
 19
 
  54466187
 
  54466724
 
  PDF   STATS   ENSEMBL   UCSC 
 
  538
 
  6
 
 22391
 
 island
 
 0.026752
 
 0.0945828
 
 Not queried
 
  1.4418473
 
 4.85e-01
 
 0.5640816327
  
 
  
 2
 
  46707626
 
  46707844
 
  PDF   STATS   ENSEMBL   UCSC 
 
  219
 
  4
 
 22667
 
 gene;island
 
 0.848625
 
 0.9339356
 
 Not queried
 
  1.4409263
 
 4.86e-01
 
 0.5640816327
  
 
  
 14
 
  55907299
 
  55907501
 
  PDF   STATS   ENSEMBL   UCSC 
 
  203
 
  7
 
 12414
 
 tss
 
 0.523643
 
 0.7181888
 
 Not queried
 
  1.4378333
 
 4.56e-01
 
 0.5640816327
  
 
  
 8
 
   1821246
 
   1821603
 
  PDF   STATS   ENSEMBL   UCSC 
 
  358
 
  4
 
 39181
 
 gene;island
 
 0.898669
 
 0.9294502
 
 Not queried
 
  1.4354428
 
 4.86e-01
 
 0.5640816327
  
 
  
 3
 
 101498198
 
 101498416
 
  PDF   STATS   ENSEMBL   UCSC 
 
  219
 
  6
 
 27882
 
 gene;island
 
 0.007655
 
 0.0021009
 
 Not queried
 
  1.4316262
 
 4.85e-01
 
 0.5640816327
  
 
  
 14
 
  24801616
 
  24802150
 
  PDF   STATS   ENSEMBL   UCSC 
 
  535
 
  4
 
 12047
 
 gene;island
 
 0.330763
 
 0.7215773
 
 Not queried
 
  1.4310582
 
 4.86e-01
 
 0.5640816327
  
 
  
 4
 
  76650351
 
  76650666
 
  PDF   STATS   ENSEMBL   UCSC 
 
  316
 
  4
 
 30025
 
 gene;island
 
 0.007422
 
 0.0044851
 
 Not queried
 
  1.4291994
 
 4.86e-01
 
 0.5640816327
  
 
  
 9
 
 124029872
 
 124030048
 
  PDF   STATS   ENSEMBL   UCSC 
 
  177
 
  4
 
 40834
 
 tss
 
 0.593765
 
 0.9110425
 
 Not queried
 
  1.4285874
 
 4.86e-01
 
 0.5640816327
  
 
  
 15
 
  59949760
 
  59950097
 
  PDF   STATS   ENSEMBL   UCSC 
 
  338
 
  8
 
 13706
 
 tss;island
 
 0.010563
 
 0.0115979
 
 Not queried
 
  1.4284721
 
 4.42e-01
 
 0.5640816327
  
 
  
 10
 
  88730324
 
  88730599
 
  PDF   STATS   ENSEMBL   UCSC 
 
  276
 
  4
 
 4664
 
 gene;island
 
 0.031482
 
 0.0854891
 
 Not queried
 
  1.4260452
 
 4.86e-01
 
 0.5640816327
  
 
  
 14
 
  37125292
 
  37125997
 
  PDF   STATS   ENSEMBL   UCSC 
 
  706
 
  8
 
 12360
 
 tss
 
 0.310439
 
 0.2258883
 
 Not queried
 
  1.4233945
 
 4.42e-01
 
 0.5640816327
  
 
  
 2
 
  74753691
 
  74753946
 
  PDF   STATS   ENSEMBL   UCSC 
 
  256
 
  4
 
 23462
 
 tss;island
 
 0.128613
 
 0.5449061
 
 Not queried
 
  1.4197219
 
 4.86e-01
 
 0.5640816327
  
 
  
 11
 
   4629034
 
   4629432
 
  PDF   STATS   ENSEMBL   UCSC 
 
  399
 
  4
 
 6484
 
 gene
 
 0.018507
 
 0.0059650
 
 Not queried
 
  1.4187646
 
 4.86e-01
 
 0.5640816327
  
 
  
 18
 
  13726491
 
  13726506
 
  PDF   STATS   ENSEMBL   UCSC 
 
   16
 
  4
 
 19063
 
 gene
 
 0.003567
 
 0.0018042
 
 Not queried
 
  1.4155155
 
 4.86e-01
 
 0.5640816327
  
 
  
 3
 
   9594151
 
   9594385
 
  PDF   STATS   ENSEMBL   UCSC 
 
  235
 
  4
 
 27653
 
 gene;island
 
 0.150050
 
 0.2561690
 
 Not queried
 
  1.4122600
 
 4.86e-01
 
 0.5640816327
  
 
  
 17
 
  80289500
 
  80289701
 
  PDF   STATS   ENSEMBL   UCSC 
 
  202
 
  4
 
 16943
 
 gene;island
 
 0.551007
 
 0.7125547
 
 Not queried
 
  1.4106399
 
 4.86e-01
 
 0.5640816327
  
 
  
 10
 
  18999145
 
  18999425
 
  PDF   STATS   ENSEMBL   UCSC 
 
  281
 
  4
 
 5587
 
 island
 
 0.870556
 
 0.9203482
 
 Not queried
 
  1.4102758
 
 4.86e-01
 
 0.5640816327
  
 
  
 8
 
   1404885
 
   1405138
 
  PDF   STATS   ENSEMBL   UCSC 
 
  254
 
  4
 
 40025
 
 island
 
 0.457200
 
 0.5885661
 
 Not queried
 
  1.4095116
 
 4.86e-01
 
 0.5640816327
  
 
  
 19
 
  33571484
 
  33571768
 
  PDF   STATS   ENSEMBL   UCSC 
 
  285
 
  4
 
 20681
 
 tss
 
 0.112795
 
 0.4003822
 
 Not queried
 
  1.4077344
 
 4.86e-01
 
 0.5640816327
  
 
  
 16
 
   3142645
 
   3143250
 
  PDF   STATS   ENSEMBL   UCSC 
 
  606
 
  8
 
 15636
 
 island
 
 0.590895
 
 0.7512143
 
 Not queried
 
  1.4060739
 
 4.42e-01
 
 0.5640816327
  
 
  
 2
 
 121554672
 
 121554817
 
  PDF   STATS   ENSEMBL   UCSC 
 
  146
 
  4
 
 23600
 
 tss
 
 0.376429
 
 0.4969653
 
 Not queried
 
  1.4014680
 
 4.86e-01
 
 0.5640816327
  
 
  
 13
 
  46425866
 
  46426263
 
  PDF   STATS   ENSEMBL   UCSC 
 
  398
 
  9
 
 11424
 
 tss
 
 0.689481
 
 0.1173224
 
 Not queried
 
  2.7422772
 
 4.89e-01
 
 0.5677586445
  
 
  
 16
 
  62070072
 
  62070365
 
  PDF   STATS   ENSEMBL   UCSC 
 
  294
 
  9
 
 15278
 
 tss
 
 0.237570
 
 0.0947658
 
 Not queried
 
  1.9180839
 
 4.89e-01
 
 0.5677586445
  
 
  
 8
 
 145028093
 
 145028505
 
  PDF   STATS   ENSEMBL   UCSC 
 
  413
 
  9
 
 39967
 
 tss
 
 0.075565
 
 0.3091700
 
 Not queried
 
  1.8798668
 
 4.89e-01
 
 0.5677586445
  
 
  
 3
 
  32509018
 
  32509543
 
  PDF   STATS   ENSEMBL   UCSC 
 
  526
 
  9
 
 28964
 
 island
 
 0.048102
 
 0.0359746
 
 Not queried
 
  1.5835655
 
 4.89e-01
 
 0.5677586445
  
 
  
 17
 
  46507549
 
  46507895
 
  PDF   STATS   ENSEMBL   UCSC 
 
  347
 
  8
 
 18472
 
 island
 
 0.093462
 
 0.0687748
 
 Not queried
 
  2.1705822
 
 5.05e-01
 
 0.5852592101
  
 
  
 6
 
  39902296
 
  39902693
 
  PDF   STATS   ENSEMBL   UCSC 
 
  398
 
  8
 
 34960
 
 tss
 
 0.160021
 
 0.0230110
 
 Not queried
 
  1.9054109
 
 5.05e-01
 
 0.5852592101
  
 
  
 8
 
 109095782
 
 109096151
 
  PDF   STATS   ENSEMBL   UCSC 
 
  370
 
  8
 
 40530
 
 island
 
 0.310175
 
 0.1083795
 
 Not queried
 
  1.6973727
 
 5.05e-01
 
 0.5852592101
  
 
  
 16
 
   2892518
 
   2892994
 
  PDF   STATS   ENSEMBL   UCSC 
 
  477
 
  8
 
 15620
 
 island
 
 0.525370
 
 0.4220178
 
 Not queried
 
  1.5491284
 
 5.05e-01
 
 0.5852592101
  
 
  
 12
 
 121163597
 
 121164371
 
  PDF   STATS   ENSEMBL   UCSC 
 
  775
 
  8
 
 9358
 
 gene
 
 0.079598
 
 0.1171663
 
 Not queried
 
  1.4815757
 
 5.05e-01
 
 0.5852592101
  
 
  
 4
 
   7941764
 
   7942031
 
  PDF   STATS   ENSEMBL   UCSC 
 
  268
 
  8
 
 30294
 
 tss;island
 
 0.345957
 
 0.7195233
 
 Not queried
 
  1.4381099
 
 5.05e-01
 
 0.5852592101
  
 
  
 11
 
  20181877
 
  20182122
 
  PDF   STATS   ENSEMBL   UCSC 
 
  246
 
  7
 
 7195
 
 tss
 
 0.189189
 
 0.0959135
 
 Not queried
 
  2.8326614
 
 5.35e-01
 
 0.6176455124
  
 
  
 10
 
 102279690
 
 102279954
 
  PDF   STATS   ENSEMBL   UCSC 
 
  265
 
  7
 
 5294
 
 tss;island
 
 0.488329
 
 0.1328599
 
 Not queried
 
  2.6332931
 
 5.35e-01
 
 0.6176455124
  
 
  
 12
 
 107349499
 
 107349641
 
  PDF   STATS   ENSEMBL   UCSC 
 
  143
 
  7
 
 10757
 
 island
 
 0.004750
 
 0.0057917
 
 Not queried
 
  2.2711810
 
 5.35e-01
 
 0.6176455124
  
 
  
 17
 
  32905966
 
  32906622
 
  PDF   STATS   ENSEMBL   UCSC 
 
  657
 
  7
 
 18201
 
 island
 
 0.005154
 
 0.0092495
 
 Not queried
 
  2.2661481
 
 5.35e-01
 
 0.6176455124
  
 
  
 17
 
  46507690
 
  46507895
 
  PDF   STATS   ENSEMBL   UCSC 
 
  206
 
  7
 
 17455
 
 tss
 
 0.122736
 
 0.0931558
 
 Not queried
 
  2.0870641
 
 5.35e-01
 
 0.6176455124
  
 
  
 22
 
  21386885
 
  21387058
 
  PDF   STATS   ENSEMBL   UCSC 
 
  174
 
  7
 
 26991
 
 tss
 
 0.187027
 
 0.0339212
 
 Not queried
 
  2.0415750
 
 5.35e-01
 
 0.6176455124
  
 
  
 17
 
  15244970
 
  15245237
 
  PDF   STATS   ENSEMBL   UCSC 
 
  268
 
  7
 
 17150
 
 tss
 
 0.005577
 
 0.0085946
 
 Not queried
 
  1.8005429
 
 5.35e-01
 
 0.6176455124
  
 
  
 12
 
  56882314
 
  56882693
 
  PDF   STATS   ENSEMBL   UCSC 
 
  380
 
  7
 
 9800
 
 tss;island
 
 0.042968
 
 0.0839836
 
 Not queried
 
  1.7644374
 
 5.35e-01
 
 0.6176455124
  
 
  
 11
 
  93474820
 
  93475054
 
  PDF   STATS   ENSEMBL   UCSC 
 
  235
 
  7
 
 6862
 
 gene
 
 0.021157
 
 0.0181498
 
 Not queried
 
  1.6461035
 
 5.35e-01
 
 0.6176455124
  
 
  
 3
 
  62861055
 
  62861311
 
  PDF   STATS   ENSEMBL   UCSC 
 
  257
 
  7
 
 29252
 
 island
 
 0.045186
 
 0.0448052
 
 Not queried
 
  1.5392641
 
 5.35e-01
 
 0.6176455124
  
 
  
 2
 
  85131951
 
  85132663
 
  PDF   STATS   ENSEMBL   UCSC 
 
  713
 
  7
 
 23478
 
 tss
 
 0.004128
 
 0.0085602
 
 Not queried
 
  1.4792781
 
 5.35e-01
 
 0.6176455124
  
 
  
 2
 
 213400697
 
 213401333
 
  PDF   STATS   ENSEMBL   UCSC 
 
  637
 
  5
 
 23005
 
 gene;island
 
 0.023302
 
 0.0148530
 
 Not queried
 
  4.5758352
 
 5.48e-01
 
 0.6179718485
  
 
  
 19
 
  41768003
 
  41768357
 
  PDF   STATS   ENSEMBL   UCSC 
 
  355
 
  5
 
 20803
 
 tss
 
 0.012012
 
 0.3758975
 
 Not queried
 
  4.1589444
 
 5.48e-01
 
 0.6179718485
  
 
  
 14
 
  53258566
 
  53258684
 
  PDF   STATS   ENSEMBL   UCSC 
 
  119
 
  5
 
 12400
 
 tss;island
 
 0.651294
 
 0.0157519
 
 Not queried
 
  3.7983467
 
 5.48e-01
 
 0.6179718485
  
 
  
 19
 
  39523309
 
  39523626
 
  PDF   STATS   ENSEMBL   UCSC 
 
  318
 
  5
 
 20764
 
 tss
 
 0.566968
 
 0.5661579
 
 Not queried
 
  3.6000008
 
 5.48e-01
 
 0.6179718485
  
 
  
 3
 
  46792023
 
  46792462
 
  PDF   STATS   ENSEMBL   UCSC 
 
  440
 
  5
 
 29060
 
 island
 
 0.535143
 
 0.7289656
 
 Not queried
 
  3.3938811
 
 5.48e-01
 
 0.6179718485
  
 
  
 22
 
  50968250
 
  50968343
 
  PDF   STATS   ENSEMBL   UCSC 
 
   94
 
  5
 
 26931
 
 gene
 
 0.015722
 
 0.0041809
 
 Not queried
 
  3.3371630
 
 5.48e-01
 
 0.6179718485
  
 
  
 10
 
 119293800
 
 119294332
 
  PDF   STATS   ENSEMBL   UCSC 
 
  533
 
  5
 
 4782
 
 gene;island
 
 0.158614
 
 0.5220398
 
 Not queried
 
  3.2643863
 
 5.48e-01
 
 0.6179718485
  
 
  
 6
 
 110798012
 
 110798323
 
  PDF   STATS   ENSEMBL   UCSC 
 
  312
 
  5
 
 35170
 
 tss;island
 
 0.525172
 
 0.6558192
 
 Not queried
 
  3.2093259
 
 5.48e-01
 
 0.6179718485
  
 
  
 7
 
  23636744
 
  23636910
 
  PDF   STATS   ENSEMBL   UCSC 
 
  167
 
  5
 
 37474
 
 tss
 
 0.017983
 
 0.0185138
 
 Not queried
 
  3.0734409
 
 5.48e-01
 
 0.6179718485
  
 
  
 1
 
 200992902
 
 200993311
 
  PDF   STATS   ENSEMBL   UCSC 
 
  410
 
  5
 
 2163
 
 tss
 
 0.020736
 
 0.0098914
 
 Not queried
 
  3.0272106
 
 5.48e-01
 
 0.6179718485
  
 
  
 15
 
  78933630
 
  78933902
 
  PDF   STATS   ENSEMBL   UCSC 
 
  273
 
  5
 
 13838
 
 tss;island
 
 0.173589
 
 0.0264398
 
 Not queried
 
  2.9531810
 
 5.48e-01
 
 0.6179718485
  
 
  
 20
 
  43439387
 
  43439688
 
  PDF   STATS   ENSEMBL   UCSC 
 
  302
 
  5
 
 25764
 
 tss;island
 
 0.008111
 
 0.1287547
 
 Not queried
 
  2.9117396
 
 5.48e-01
 
 0.6179718485
  
 
  
 1
 
  16553267
 
  16553549
 
  PDF   STATS   ENSEMBL   UCSC 
 
  283
 
  5
 
 2740
 
 island
 
 0.758189
 
 0.5187543
 
 Not queried
 
  2.7860565
 
 5.48e-01
 
 0.6179718485
  
 
  
 11
 
  67033594
 
  67033801
 
  PDF   STATS   ENSEMBL   UCSC 
 
  208
 
  5
 
 7484
 
 tss;island
 
 0.007747
 
 0.0028289
 
 Not queried
 
  2.7513706
 
 5.48e-01
 
 0.6179718485
  
 
  
 7
 
 106505688
 
 106505961
 
  PDF   STATS   ENSEMBL   UCSC 
 
  274
 
  5
 
 38825
 
 island
 
 0.011752
 
 0.0043983
 
 Not queried
 
  2.6555477
 
 5.48e-01
 
 0.6179718485
  
 
  
 17
 
  49243257
 
  49243640
 
  PDF   STATS   ENSEMBL   UCSC 
 
  384
 
  5
 
 16695
 
 gene;tss;island
 
 0.012667
 
 0.0184425
 
 Not queried
 
  2.5681245
 
 5.48e-01
 
 0.6179718485
  
 
  
 3
 
 193922037
 
 193922406
 
  PDF   STATS   ENSEMBL   UCSC 
 
  370
 
  5
 
 29784
 
 island
 
 0.050964
 
 0.4858026
 
 Not queried
 
  2.5621744
 
 5.48e-01
 
 0.6179718485
  
 
  
 12
 
  69753119
 
  69753455
 
  PDF   STATS   ENSEMBL   UCSC 
 
  337
 
  5
 
 9860
 
 tss
 
 0.003325
 
 0.0019630
 
 Not queried
 
  2.5140705
 
 5.48e-01
 
 0.6179718485
  
 
  
 10
 
  43697817
 
  43698141
 
  PDF   STATS   ENSEMBL   UCSC 
 
  325
 
  5
 
 4568
 
 gene;island
 
 0.005352
 
 0.0190274
 
 Not queried
 
  2.4075516
 
 5.48e-01
 
 0.6179718485
  
 
  
 17
 
  62084205
 
  62084359
 
  PDF   STATS   ENSEMBL   UCSC 
 
  155
 
  5
 
 16731
 
 gene
 
 0.557219
 
 0.8686408
 
 Not queried
 
  2.3925128
 
 5.48e-01
 
 0.6179718485
  
 
  
 3
 
  27765232
 
  27765546
 
  PDF   STATS   ENSEMBL   UCSC 
 
  315
 
  5
 
 28952
 
 island
 
 0.075664
 
 0.0414450
 
 Not queried
 
  2.3758388
 
 5.48e-01
 
 0.6179718485
  
 
  
 20
 
  62574023
 
  62574273
 
  PDF   STATS   ENSEMBL   UCSC 
 
  251
 
  5
 
 25514
 
 gene;tss;island
 
 0.550443
 
 0.8352042
 
 Not queried
 
  2.3384799
 
 5.48e-01
 
 0.6179718485
  
 
  
 11
 
  63775071
 
  63775612
 
  PDF   STATS   ENSEMBL   UCSC 
 
  542
 
  5
 
 6658
 
 gene;island
 
 0.115649
 
 0.0902568
 
 Not queried
 
  2.2692638
 
 5.48e-01
 
 0.6179718485
  
 
  
 10
 
  97850047
 
  97850343
 
  PDF   STATS   ENSEMBL   UCSC 
 
  297
 
  5
 
 5971
 
 island
 
 0.002785
 
 0.0053435
 
 Not queried
 
  2.2668512
 
 5.48e-01
 
 0.6179718485
  
 
  
 4
 
  53727714
 
  53728038
 
  PDF   STATS   ENSEMBL   UCSC 
 
  325
 
  5
 
 30393
 
 tss;island
 
 0.905061
 
 0.7210437
 
 Not queried
 
  2.1539185
 
 5.48e-01
 
 0.6179718485
  
 
  
 3
 
  50242325
 
  50242754
 
  PDF   STATS   ENSEMBL   UCSC 
 
  430
 
  5
 
 29138
 
 island
 
 0.008018
 
 0.0428411
 
 Not queried
 
  2.1272987
 
 5.48e-01
 
 0.6179718485
  
 
  
 14
 
 104195181
 
 104195666
 
  PDF   STATS   ENSEMBL   UCSC 
 
  486
 
  5
 
 12243
 
 gene;island
 
 0.918334
 
 0.9612446
 
 Not queried
 
  2.0734385
 
 5.48e-01
 
 0.6179718485
  
 
  
 1
 
  43855376
 
  43855471
 
  PDF   STATS   ENSEMBL   UCSC 
 
   96
 
  5
 
 478
 
 gene
 
 0.002739
 
 0.0069994
 
 Not queried
 
  2.0214075
 
 5.48e-01
 
 0.6179718485
  
 
  
 16
 
  66955873
 
  66956242
 
  PDF   STATS   ENSEMBL   UCSC 
 
  370
 
  5
 
 14784
 
 gene;island
 
 0.672210
 
 0.8948975
 
 Not queried
 
  2.0058393
 
 5.48e-01
 
 0.6179718485
  
 
  
 17
 
  75277207
 
  75277435
 
  PDF   STATS   ENSEMBL   UCSC 
 
  229
 
  5
 
 17695
 
 tss;island
 
 0.003099
 
 0.0012535
 
 Not queried
 
  1.9993773
 
 5.48e-01
 
 0.6179718485
  
 
  
 1
 
  24514218
 
  24514557
 
  PDF   STATS   ENSEMBL   UCSC 
 
  340
 
  5
 
 1357
 
 tss;island
 
 0.026862
 
 0.0259515
 
 Not queried
 
  1.9953544
 
 5.48e-01
 
 0.6179718485
  
 
  
 18
 
  44099028
 
  44099340
 
  PDF   STATS   ENSEMBL   UCSC 
 
  313
 
  5
 
 19103
 
 gene
 
 0.019416
 
 0.0864335
 
 Not queried
 
  1.9716540
 
 5.48e-01
 
 0.6179718485
  
 
  
 11
 
 130029478
 
 130029684
 
  PDF   STATS   ENSEMBL   UCSC 
 
  207
 
  5
 
 8957
 
 island
 
 0.118856
 
 0.1073045
 
 Not queried
 
  1.9677077
 
 5.48e-01
 
 0.6179718485
  
 
  
 19
 
  44860658
 
  44861241
 
  PDF   STATS   ENSEMBL   UCSC 
 
  584
 
  5
 
 22108
 
 island
 
 0.533758
 
 0.3245240
 
 Not queried
 
  1.9211444
 
 5.48e-01
 
 0.6179718485
  
 
  
 7
 
  92672812
 
  92673176
 
  PDF   STATS   ENSEMBL   UCSC 
 
  365
 
  5
 
 38665
 
 island
 
 0.482642
 
 0.3556937
 
 Not queried
 
  1.9029032
 
 5.48e-01
 
 0.6179718485
  
 
  
 17
 
  10560609
 
  10560928
 
  PDF   STATS   ENSEMBL   UCSC 
 
  320
 
  5
 
 17134
 
 tss
 
 0.921918
 
 0.9345011
 
 Not queried
 
  1.8995742
 
 5.48e-01
 
 0.6179718485
  
 
  
 16
 
   1384626
 
   1384908
 
  PDF   STATS   ENSEMBL   UCSC 
 
  283
 
  5
 
 15529
 
 island
 
 0.234448
 
 0.0723550
 
 Not queried
 
  1.8687570
 
 5.48e-01
 
 0.6179718485
  
 
  
 16
 
  86411222
 
  86411456
 
  PDF   STATS   ENSEMBL   UCSC 
 
  235
 
  5
 
 16169
 
 island
 
 0.427156
 
 0.6985670
 
 Not queried
 
  1.8446049
 
 5.48e-01
 
 0.6179718485
  
 
  
 19
 
   2427708
 
   2428122
 
  PDF   STATS   ENSEMBL   UCSC 
 
  415
 
  5
 
 21284
 
 island
 
 0.006919
 
 0.0075437
 
 Not queried
 
  1.8162728
 
 5.48e-01
 
 0.6179718485
  
 
  
 17
 
  75451779
 
  75452100
 
  PDF   STATS   ENSEMBL   UCSC 
 
  322
 
  5
 
 16810
 
 gene
 
 0.658531
 
 0.9279821
 
 Not queried
 
  1.8129996
 
 5.48e-01
 
 0.6179718485
  
 
  
 6
 
 166571782
 
 166572045
 
  PDF   STATS   ENSEMBL   UCSC 
 
  264
 
  5
 
 34543
 
 gene;island
 
 0.697891
 
 0.7357168
 
 Not queried
 
  1.7861701
 
 5.48e-01
 
 0.6179718485
  
 
  
 12
 
 107711872
 
 107712194
 
  PDF   STATS   ENSEMBL   UCSC 
 
  323
 
  5
 
 9975
 
 tss
 
 0.010686
 
 0.0027662
 
 Not queried
 
  1.7768629
 
 5.48e-01
 
 0.6179718485
  
 
  
 19
 
  15284853
 
  15285108
 
  PDF   STATS   ENSEMBL   UCSC 
 
  256
 
  5
 
 19901
 
 gene;island
 
 0.667393
 
 0.8723853
 
 Not queried
 
  1.7732387
 
 5.48e-01
 
 0.6179718485
  
 
  
 8
 
  65489863
 
  65489939
 
  PDF   STATS   ENSEMBL   UCSC 
 
   77
 
  5
 
 39734
 
 tss;island
 
 0.060441
 
 0.0119509
 
 Not queried
 
  1.7608021
 
 5.48e-01
 
 0.6179718485
  
 
  
 18
 
  21852313
 
  21852436
 
  PDF   STATS   ENSEMBL   UCSC 
 
  124
 
  5
 
 19211
 
 tss
 
 0.005776
 
 0.0151450
 
 Not queried
 
  1.7550719
 
 5.48e-01
 
 0.6179718485
  
 
  
 11
 
  31845148
 
  31845639
 
  PDF   STATS   ENSEMBL   UCSC 
 
  492
 
  5
 
 8168
 
 island
 
 0.035110
 
 0.0044182
 
 Not queried
 
  1.7260057
 
 5.48e-01
 
 0.6179718485
  
 
  
 1
 
  47082691
 
  47082869
 
  PDF   STATS   ENSEMBL   UCSC 
 
  179
 
  5
 
 1603
 
 tss;island
 
 0.053474
 
 0.0486333
 
 Not queried
 
  1.7175582
 
 5.48e-01
 
 0.6179718485
  
 
  
 10
 
 134729942
 
 134730258
 
  PDF   STATS   ENSEMBL   UCSC 
 
  317
 
  5
 
 6317
 
 island
 
 0.768864
 
 0.4945439
 
 Not queried
 
  1.7100252
 
 5.48e-01
 
 0.6179718485
  
 
  
 17
 
  47091978
 
  47092272
 
  PDF   STATS   ENSEMBL   UCSC 
 
  295
 
  5
 
 16670
 
 gene;island
 
 0.742150
 
 0.4857384
 
 Not queried
 
  1.7095630
 
 5.48e-01
 
 0.6179718485
  
 
  
 17
 
  79876118
 
  79876287
 
  PDF   STATS   ENSEMBL   UCSC 
 
  170
 
  5
 
 17757
 
 tss
 
 0.010669
 
 0.0031477
 
 Not queried
 
  1.7049214
 
 5.48e-01
 
 0.6179718485
  
 
  
 13
 
  95655083
 
  95655579
 
  PDF   STATS   ENSEMBL   UCSC 
 
  497
 
  5
 
 11824
 
 island
 
 0.150777
 
 0.0167115
 
 Not queried
 
  1.7040521
 
 5.48e-01
 
 0.6179718485
  
 
  
 3
 
 197463746
 
 197464306
 
  PDF   STATS   ENSEMBL   UCSC 
 
  561
 
  9
 
 28106
 
 gene;island
 
 0.006316
 
 0.0020195
 
 Not queried
 
  1.7014361
 
 5.46e-01
 
 0.6179718485
  
 
  
 11
 
  66314211
 
  66314378
 
  PDF   STATS   ENSEMBL   UCSC 
 
  168
 
  5
 
 7473
 
 tss;island
 
 0.036790
 
 0.0084230
 
 Not queried
 
  1.6610855
 
 5.48e-01
 
 0.6179718485
  
 
  
 3
 
 120460879
 
 120461349
 
  PDF   STATS   ENSEMBL   UCSC 
 
  471
 
  5
 
 27910
 
 gene
 
 0.002682
 
 0.0010189
 
 Not queried
 
  1.6594876
 
 5.48e-01
 
 0.6179718485
  
 
  
 20
 
  61810348
 
  61810902
 
  PDF   STATS   ENSEMBL   UCSC 
 
  555
 
  5
 
 26344
 
 island
 
 0.037364
 
 0.0131455
 
 Not queried
 
  1.6543422
 
 5.48e-01
 
 0.6179718485
  
 
  
 4
 
  80247171
 
  80247281
 
  PDF   STATS   ENSEMBL   UCSC 
 
  111
 
  5
 
 30456
 
 tss
 
 0.825114
 
 0.8698521
 
 Not queried
 
  1.6527393
 
 5.48e-01
 
 0.6179718485
  
 
  
 17
 
  17480829
 
  17481059
 
  PDF   STATS   ENSEMBL   UCSC 
 
  231
 
  5
 
 16465
 
 gene;tss
 
 0.639154
 
 0.8295058
 
 Not queried
 
  1.6390872
 
 5.48e-01
 
 0.6179718485
  
 
  
 7
 
  97841334
 
  97841513
 
  PDF   STATS   ENSEMBL   UCSC 
 
  180
 
  5
 
 37743
 
 tss
 
 0.938607
 
 0.5764505
 
 Not queried
 
  1.6362998
 
 5.48e-01
 
 0.6179718485
  
 
  
 10
 
   1509675
 
   1510077
 
  PDF   STATS   ENSEMBL   UCSC 
 
  403
 
  5
 
 4465
 
 gene;island
 
 0.451686
 
 0.7083001
 
 Not queried
 
  1.6348309
 
 5.48e-01
 
 0.6179718485
  
 
  
 2
 
 187713902
 
 187714279
 
  PDF   STATS   ENSEMBL   UCSC 
 
  378
 
  9
 
 23775
 
 tss
 
 0.059675
 
 0.0207510
 
 Not queried
 
  1.6315022
 
 5.46e-01
 
 0.6179718485
  
 
  
 6
 
  33159353
 
  33159593
 
  PDF   STATS   ENSEMBL   UCSC 
 
  241
 
  5
 
 34215
 
 gene;island
 
 0.415787
 
 0.4424883
 
 Not queried
 
  1.6128979
 
 5.48e-01
 
 0.6179718485
  
 
  
 16
 
  55542921
 
  55543166
 
  PDF   STATS   ENSEMBL   UCSC 
 
  246
 
  5
 
 14753
 
 gene
 
 0.036465
 
 0.0477288
 
 Not queried
 
  1.5997556
 
 5.48e-01
 
 0.6179718485
  
 
  
 16
 
  10674326
 
  10674468
 
  PDF   STATS   ENSEMBL   UCSC 
 
  143
 
  5
 
 14660
 
 gene
 
 0.005134
 
 0.0040592
 
 Not queried
 
  1.5944302
 
 5.48e-01
 
 0.6179718485
  
 
  
 20
 
   9494838
 
   9495199
 
  PDF   STATS   ENSEMBL   UCSC 
 
  362
 
  5
 
 25592
 
 tss
 
 0.012678
 
 0.0075208
 
 Not queried
 
  1.5714106
 
 5.48e-01
 
 0.6179718485
  
 
  
 19
 
  55417361
 
  55417500
 
  PDF   STATS   ENSEMBL   UCSC 
 
  140
 
  5
 
 21066
 
 tss
 
 0.087583
 
 0.3331102
 
 Not queried
 
  1.5665707
 
 5.48e-01
 
 0.6179718485
  
 
  
 19
 
  13985408
 
  13985688
 
  PDF   STATS   ENSEMBL   UCSC 
 
  281
 
  5
 
 21661
 
 island
 
 0.504071
 
 0.6385437
 
 Not queried
 
  1.5611295
 
 5.48e-01
 
 0.6179718485
  
 
  
 10
 
 134049464
 
 134049802
 
  PDF   STATS   ENSEMBL   UCSC 
 
  339
 
  5
 
 4859
 
 gene
 
 0.864461
 
 0.8584882
 
 Not queried
 
  1.5562841
 
 5.48e-01
 
 0.6179718485
  
 
  
 16
 
  67678593
 
  67678846
 
  PDF   STATS   ENSEMBL   UCSC 
 
  254
 
  5
 
 15307
 
 tss;island
 
 0.012875
 
 0.0097638
 
 Not queried
 
  1.5485600
 
 5.48e-01
 
 0.6179718485
  
 
  
 4
 
 184922382
 
 184922646
 
  PDF   STATS   ENSEMBL   UCSC 
 
  265
 
  5
 
 30197
 
 gene
 
 0.403547
 
 0.5988744
 
 Not queried
 
  1.5455982
 
 5.48e-01
 
 0.6179718485
  
 
  
 17
 
  74099707
 
  74100207
 
  PDF   STATS   ENSEMBL   UCSC 
 
  501
 
 13
 
 17671
 
 tss;island
 
 0.006254
 
 0.0054015
 
 Not queried
 
  1.5416122
 
 5.45e-01
 
 0.6179718485
  
 
  
 4
 
  46911291
 
  46911550
 
  PDF   STATS   ENSEMBL   UCSC 
 
  260
 
  5
 
 30374
 
 tss
 
 0.503274
 
 0.8571869
 
 Not queried
 
  1.5309687
 
 5.48e-01
 
 0.6179718485
  
 
  
 14
 
  73602752
 
  73602980
 
  PDF   STATS   ENSEMBL   UCSC 
 
  229
 
  5
 
 12495
 
 tss;island
 
 0.035022
 
 0.0148698
 
 Not queried
 
  1.5234612
 
 5.48e-01
 
 0.6179718485
  
 
  
 18
 
    596703
 
    596943
 
  PDF   STATS   ENSEMBL   UCSC 
 
  241
 
  5
 
 19152
 
 tss
 
 0.012391
 
 0.0251542
 
 Not queried
 
  1.5038700
 
 5.48e-01
 
 0.6179718485
  
 
  
 14
 
  35099471
 
  35099711
 
  PDF   STATS   ENSEMBL   UCSC 
 
  241
 
  5
 
 12347
 
 tss
 
 0.119855
 
 0.0293947
 
 Not queried
 
  1.5026033
 
 5.48e-01
 
 0.6179718485
  
 
  
 2
 
    468737
 
    469214
 
  PDF   STATS   ENSEMBL   UCSC 
 
  478
 
  5
 
 24003
 
 island
 
 0.456086
 
 0.7848009
 
 Not queried
 
  1.4950629
 
 5.48e-01
 
 0.6179718485
  
 
  
 17
 
  45772333
 
  45772523
 
  PDF   STATS   ENSEMBL   UCSC 
 
  191
 
  5
 
 17443
 
 tss
 
 0.006531
 
 0.0017316
 
 Not queried
 
  1.4798863
 
 5.48e-01
 
 0.6179718485
  
 
  
 6
 
  32172354
 
  32172871
 
  PDF   STATS   ENSEMBL   UCSC 
 
  518
 
  5
 
 34089
 
 gene
 
 0.213213
 
 0.2714793
 
 Not queried
 
  1.4591197
 
 5.48e-01
 
 0.6179718485
  
 
  
 7
 
  18067508
 
  18067537
 
  PDF   STATS   ENSEMBL   UCSC 
 
   30
 
  5
 
 37457
 
 tss
 
 0.978732
 
 0.9728710
 
 Not queried
 
  1.4503381
 
 5.48e-01
 
 0.6179718485
  
 
  
 16
 
  30905695
 
  30905875
 
  PDF   STATS   ENSEMBL   UCSC 
 
  181
 
  5
 
 15197
 
 tss;island
 
 0.090110
 
 0.0641778
 
 Not queried
 
  1.4318272
 
 5.48e-01
 
 0.6179718485
  
 
  
 8
 
  85095275
 
  85095697
 
  PDF   STATS   ENSEMBL   UCSC 
 
  423
 
  5
 
 39788
 
 tss;island
 
 0.028763
 
 0.0219028
 
 Not queried
 
  1.4272809
 
 5.48e-01
 
 0.6179718485
  
 
  
 6
 
  37225949
 
  37226214
 
  PDF   STATS   ENSEMBL   UCSC 
 
  266
 
  5
 
 34321
 
 gene;tss;island
 
 0.001606
 
 0.0016975
 
 Not queried
 
  1.4256939
 
 5.48e-01
 
 0.6179718485
  
 
  
 1
 
   1992046
 
   1992687
 
  PDF   STATS   ENSEMBL   UCSC 
 
  642
 
  5
 
 54
 
 gene;island
 
 0.542900
 
 0.7346494
 
 Not queried
 
  1.4197045
 
 5.48e-01
 
 0.6179718485
  
 
  
 11
 
    393328
 
    393648
 
  PDF   STATS   ENSEMBL   UCSC 
 
  321
 
  5
 
 7010
 
 tss;island
 
 0.309728
 
 0.5428290
 
 Not queried
 
  1.4089841
 
 5.48e-01
 
 0.6179718485
  
 
  
 11
 
  70048796
 
  70049203
 
  PDF   STATS   ENSEMBL   UCSC 
 
  408
 
 11
 
 7526
 
 tss;island
 
 0.068922
 
 0.0920677
 
 Not queried
 
  1.4589626
 
 5.62e-01
 
 0.6339017597
  
 
  
 15
 
  90294471
 
  90294776
 
  PDF   STATS   ENSEMBL   UCSC 
 
  306
 
  8
 
 14441
 
 island
 
 0.024987
 
 0.0524074
 
 Not queried
 
  2.0530744
 
 5.74e-01
 
 0.6460388787
  
 
  
 1
 
 153643479
 
 153643822
 
  PDF   STATS   ENSEMBL   UCSC 
 
  344
 
  8
 
 1949
 
 tss
 
 0.004012
 
 0.0065817
 
 Not queried
 
  2.0318594
 
 5.74e-01
 
 0.6460388787
  
 
  
 7
 
  27224092
 
  27224795
 
  PDF   STATS   ENSEMBL   UCSC 
 
  704
 
  8
 
 36941
 
 gene
 
 0.024501
 
 0.0435094
 
 Not queried
 
  1.6910029
 
 5.74e-01
 
 0.6460388787
  
 
  
 4
 
  47033126
 
  47033634
 
  PDF   STATS   ENSEMBL   UCSC 
 
  509
 
  8
 
 31022
 
 island
 
 0.035472
 
 0.0195677
 
 Not queried
 
  1.5907481
 
 5.74e-01
 
 0.6460388787
  
 
  
 8
 
  74659776
 
  74660304
 
  PDF   STATS   ENSEMBL   UCSC 
 
  529
 
  8
 
 39769
 
 tss;island
 
 0.022201
 
 0.0175204
 
 Not queried
 
  1.5546133
 
 5.74e-01
 
 0.6460388787
  
 
  
 7
 
   1272255
 
   1272559
 
  PDF   STATS   ENSEMBL   UCSC 
 
  305
 
  8
 
 37398
 
 tss
 
 0.110116
 
 0.1921847
 
 Not queried
 
  1.5025709
 
 5.74e-01
 
 0.6460388787
  
 
  
 18
 
  47792708
 
  47793175
 
  PDF   STATS   ENSEMBL   UCSC 
 
  468
 
  8
 
 19508
 
 island
 
 0.016454
 
 0.0275478
 
 Not queried
 
  1.4602402
 
 5.74e-01
 
 0.6460388787
  
 
  
 6
 
  17282284
 
  17282859
 
  PDF   STATS   ENSEMBL   UCSC 
 
  576
 
 10
 
 33587
 
 gene;tss;island
 
 0.107877
 
 0.2304046
 
 Not queried
 
  2.1781164
 
 5.79e-01
 
 0.6514968922
  
 
  
 15
 
  40674878
 
  40675029
 
  PDF   STATS   ENSEMBL   UCSC 
 
  152
 
  6
 
 14035
 
 island
 
 0.028424
 
 0.3568408
 
 Not queried
 
  4.1154325
 
 5.89e-01
 
 0.6570484697
  
 
  
 2
 
 198650603
 
 198651076
 
  PDF   STATS   ENSEMBL   UCSC 
 
  474
 
  6
 
 23802
 
 tss;island
 
 0.014038
 
 0.0302761
 
 Not queried
 
  3.0483908
 
 5.89e-01
 
 0.6570484697
  
 
  
 2
 
 109065127
 
 109065510
 
  PDF   STATS   ENSEMBL   UCSC 
 
  384
 
  6
 
 23564
 
 tss
 
 0.011806
 
 0.0668655
 
 Not queried
 
  2.9028507
 
 5.89e-01
 
 0.6570484697
  
 
  
 7
 
  95064691
 
  95064926
 
  PDF   STATS   ENSEMBL   UCSC 
 
  236
 
  6
 
 37728
 
 tss;island
 
 0.049465
 
 0.0224180
 
 Not queried
 
  2.8253507
 
 5.89e-01
 
 0.6570484697
  
 
  
 2
 
  38978654
 
  38978896
 
  PDF   STATS   ENSEMBL   UCSC 
 
  243
 
  6
 
 23313
 
 tss
 
 0.019159
 
 0.0215297
 
 Not queried
 
  2.7969623
 
 5.89e-01
 
 0.6570484697
  
 
  
 20
 
  54919037
 
  54919410
 
  PDF   STATS   ENSEMBL   UCSC 
 
  374
 
  6
 
 26271
 
 island
 
 0.012426
 
 0.0733906
 
 Not queried
 
  2.6904540
 
 5.89e-01
 
 0.6570484697
  
 
  
 16
 
  46603015
 
  46603321
 
  PDF   STATS   ENSEMBL   UCSC 
 
  307
 
  6
 
 15218
 
 tss
 
 0.789595
 
 0.6006238
 
 Not queried
 
  2.4988651
 
 5.89e-01
 
 0.6570484697
  
 
  
 14
 
 101530343
 
 101530856
 
  PDF   STATS   ENSEMBL   UCSC 
 
  514
 
  6
 
 12633
 
 tss;island
 
 0.183086
 
 0.3710628
 
 Not queried
 
  2.2715056
 
 5.89e-01
 
 0.6570484697
  
 
  
 19
 
   3869086
 
   3869599
 
  PDF   STATS   ENSEMBL   UCSC 
 
  514
 
  6
 
 20353
 
 tss;island
 
 0.190991
 
 0.4252011
 
 Not queried
 
  2.1849834
 
 5.89e-01
 
 0.6570484697
  
 
  
 4
 
 122685352
 
 122685675
 
  PDF   STATS   ENSEMBL   UCSC 
 
  324
 
  6
 
 30113
 
 gene;tss;island
 
 0.224906
 
 0.2559040
 
 Not queried
 
  2.1455370
 
 5.89e-01
 
 0.6570484697
  
 
  
 15
 
  51973460
 
  51973920
 
  PDF   STATS   ENSEMBL   UCSC 
 
  461
 
  6
 
 14140
 
 island
 
 0.409269
 
 0.0959105
 
 Not queried
 
  2.0668274
 
 5.89e-01
 
 0.6570484697
  
 
  
 15
 
  90294568
 
  90294776
 
  PDF   STATS   ENSEMBL   UCSC 
 
  209
 
  6
 
 13891
 
 tss
 
 0.035780
 
 0.1210127
 
 Not queried
 
  2.0530744
 
 5.89e-01
 
 0.6570484697
  
 
  
 19
 
  57306631
 
  57307081
 
  PDF   STATS   ENSEMBL   UCSC 
 
  451
 
  6
 
 20239
 
 gene;island
 
 0.190490
 
 0.3400953
 
 Not queried
 
  1.8983641
 
 5.89e-01
 
 0.6570484697
  
 
  
 8
 
  25909171
 
  25909599
 
  PDF   STATS   ENSEMBL   UCSC 
 
  429
 
  6
 
 40172
 
 island
 
 0.060144
 
 0.1074702
 
 Not queried
 
  1.8978591
 
 5.89e-01
 
 0.6570484697
  
 
  
 6
 
  33215649
 
  33215868
 
  PDF   STATS   ENSEMBL   UCSC 
 
  220
 
  6
 
 35986
 
 island
 
 0.055725
 
 0.1468743
 
 Not queried
 
  1.8640394
 
 5.89e-01
 
 0.6570484697
  
 
  
 1
 
  16563580
 
  16563954
 
  PDF   STATS   ENSEMBL   UCSC 
 
  375
 
  6
 
 2741
 
 island
 
 0.002182
 
 0.0039208
 
 Not queried
 
  1.8089684
 
 5.89e-01
 
 0.6570484697
  
 
  
 7
 
  39989474
 
  39989867
 
  PDF   STATS   ENSEMBL   UCSC 
 
  394
 
  6
 
 37548
 
 tss
 
 0.002798
 
 0.0056767
 
 Not queried
 
  1.7755610
 
 5.89e-01
 
 0.6570484697
  
 
  
 11
 
  36615733
 
  36616048
 
  PDF   STATS   ENSEMBL   UCSC 
 
  316
 
  6
 
 7250
 
 tss
 
 0.053299
 
 0.5450694
 
 Not queried
 
  1.7731805
 
 5.89e-01
 
 0.6570484697
  
 
  
 3
 
 101546486
 
 101546859
 
  PDF   STATS   ENSEMBL   UCSC 
 
  374
 
  6
 
 27883
 
 gene
 
 0.001327
 
 0.0011716
 
 Not queried
 
  1.7358355
 
 5.89e-01
 
 0.6570484697
  
 
  
 7
 
 123673331
 
 123673690
 
  PDF   STATS   ENSEMBL   UCSC 
 
  360
 
  6
 
 38879
 
 island
 
 0.078619
 
 0.0428412
 
 Not queried
 
  1.7067405
 
 5.89e-01
 
 0.6570484697
  
 
  
 1
 
  89357197
 
  89357426
 
  PDF   STATS   ENSEMBL   UCSC 
 
  230
 
  6
 
 3446
 
 island
 
 0.002212
 
 0.0023029
 
 Not queried
 
  1.6997537
 
 5.89e-01
 
 0.6570484697
  
 
  
 17
 
  80185943
 
  80186336
 
  PDF   STATS   ENSEMBL   UCSC 
 
  394
 
  6
 
 17769
 
 tss;island
 
 0.014302
 
 0.0495647
 
 Not queried
 
  1.6980368
 
 5.89e-01
 
 0.6570484697
  
 
  
 19
 
   9420290
 
   9420631
 
  PDF   STATS   ENSEMBL   UCSC 
 
  342
 
  6
 
 21495
 
 island
 
 0.001629
 
 0.0042662
 
 Not queried
 
  1.6021886
 
 5.89e-01
 
 0.6570484697
  
 
  
 14
 
  36989287
 
  36989588
 
  PDF   STATS   ENSEMBL   UCSC 
 
  302
 
  6
 
 12358
 
 tss;island
 
 0.032556
 
 0.0550841
 
 Not queried
 
  1.5950840
 
 5.89e-01
 
 0.6570484697
  
 
  
 16
 
   2892766
 
   2892994
 
  PDF   STATS   ENSEMBL   UCSC 
 
  229
 
  6
 
 15038
 
 tss
 
 0.525370
 
 0.1983996
 
 Not queried
 
  1.5491284
 
 5.89e-01
 
 0.6570484697
  
 
  
 7
 
    544525
 
    544976
 
  PDF   STATS   ENSEMBL   UCSC 
 
  452
 
  6
 
 36706
 
 gene
 
 0.790630
 
 0.8970978
 
 Not queried
 
  1.5359387
 
 5.89e-01
 
 0.6570484697
  
 
  
 15
 
  33360195
 
  33360485
 
  PDF   STATS   ENSEMBL   UCSC 
 
  291
 
  6
 
 13554
 
 tss
 
 0.061093
 
 0.0479924
 
 Not queried
 
  1.5255083
 
 5.89e-01
 
 0.6570484697
  
 
  
 17
 
  74137336
 
  74137522
 
  PDF   STATS   ENSEMBL   UCSC 
 
  187
 
  6
 
 18774
 
 island
 
 0.003879
 
 0.0055274
 
 Not queried
 
  1.5061932
 
 5.89e-01
 
 0.6570484697
  
 
  
 17
 
  48785255
 
  48785602
 
  PDF   STATS   ENSEMBL   UCSC 
 
  348
 
  6
 
 18559
 
 island
 
 0.016746
 
 0.0069435
 
 Not queried
 
  1.4870585
 
 5.89e-01
 
 0.6570484697
  
 
  
 11
 
  17373103
 
  17373302
 
  PDF   STATS   ENSEMBL   UCSC 
 
  200
 
  6
 
 7174
 
 tss;island
 
 0.048818
 
 0.0468460
 
 Not queried
 
  1.4854394
 
 5.89e-01
 
 0.6570484697
  
 
  
 8
 
 114447055
 
 114447871
 
  PDF   STATS   ENSEMBL   UCSC 
 
  817
 
  6
 
 39402
 
 gene;island
 
 0.003015
 
 0.0033010
 
 Not queried
 
  1.4580406
 
 5.89e-01
 
 0.6570484697
  
 
  
 5
 
  44388799
 
  44389062
 
  PDF   STATS   ENSEMBL   UCSC 
 
  264
 
  6
 
 32052
 
 tss
 
 0.029112
 
 0.1110427
 
 Not queried
 
  1.4265132
 
 5.89e-01
 
 0.6570484697
  
 
  
 11
 
 118436807
 
 118437133
 
  PDF   STATS   ENSEMBL   UCSC 
 
  327
 
  9
 
 7735
 
 tss
 
 0.003320
 
 0.0102620
 
 Not queried
 
  2.2283755
 
 6.05e-01
 
 0.6747992494
  
 
  
 2
 
 177012117
 
 177012706
 
  PDF   STATS   ENSEMBL   UCSC 
 
  590
 
  7
 
 24907
 
 island
 
 0.191349
 
 0.1433182
 
 Not queried
 
  2.3407207
 
 6.20e-01
 
 0.6899391537
  
 
  
 11
 
  86085623
 
  86086101
 
  PDF   STATS   ENSEMBL   UCSC 
 
  479
 
  7
 
 8711
 
 island
 
 0.102191
 
 0.0550710
 
 Not queried
 
  1.9816954
 
 6.20e-01
 
 0.6899391537
  
 
  
 2
 
 133428422
 
 133428993
 
  PDF   STATS   ENSEMBL   UCSC 
 
  572
 
  7
 
 22879
 
 gene
 
 0.183745
 
 0.4667998
 
 Not queried
 
  1.9595513
 
 6.20e-01
 
 0.6899391537
  
 
  
 3
 
 113160430
 
 113160821
 
  PDF   STATS   ENSEMBL   UCSC 
 
  392
 
  7
 
 28506
 
 tss
 
 0.506058
 
 0.1779857
 
 Not queried
 
  1.9110093
 
 6.20e-01
 
 0.6899391537
  
 
  
 15
 
  52404977
 
  52405162
 
  PDF   STATS   ENSEMBL   UCSC 
 
  186
 
  7
 
 13677
 
 tss
 
 0.620366
 
 0.2554756
 
 Not queried
 
  1.7956867
 
 6.20e-01
 
 0.6899391537
  
 
  
 10
 
  72201417
 
  72201741
 
  PDF   STATS   ENSEMBL   UCSC 
 
  325
 
  7
 
 5819
 
 island
 
 0.132182
 
 0.1140739
 
 Not queried
 
  1.7840361
 
 6.20e-01
 
 0.6899391537
  
 
  
 17
 
  14206774
 
  14207036
 
  PDF   STATS   ENSEMBL   UCSC 
 
  263
 
  7
 
 16450
 
 gene;tss;island
 
 0.015863
 
 0.0687744
 
 Not queried
 
  1.6845745
 
 6.20e-01
 
 0.6899391537
  
 
  
 3
 
 133748115
 
 133748809
 
  PDF   STATS   ENSEMBL   UCSC 
 
  695
 
  7
 
 27953
 
 gene;island
 
 0.016254
 
 0.0187591
 
 Not queried
 
  1.5217253
 
 6.20e-01
 
 0.6899391537
  
 
  
 1
 
  29062689
 
  29063076
 
  PDF   STATS   ENSEMBL   UCSC 
 
  388
 
  7
 
 1436
 
 tss;island
 
 0.006550
 
 0.0073647
 
 Not queried
 
  1.4215736
 
 6.20e-01
 
 0.6899391537
  
 
  
 6
 
  33039396
 
  33039804
 
  PDF   STATS   ENSEMBL   UCSC 
 
  409
 
  7
 
 34182
 
 gene
 
 0.006133
 
 0.0095460
 
 Not queried
 
  1.4075210
 
 6.20e-01
 
 0.6899391537
  
 
  
 8
 
 145027948
 
 145028505
 
  PDF   STATS   ENSEMBL   UCSC 
 
  558
 
 10
 
 39504
 
 gene;island
 
 0.284765
 
 0.2611669
 
 Not queried
 
  1.4406035
 
 6.31e-01
 
 0.7014147777
  
 
  
 19
 
  34918900
 
  34919411
 
  PDF   STATS   ENSEMBL   UCSC 
 
  512
 
  8
 
 21886
 
 island
 
 0.002442
 
 0.0028556
 
 Not queried
 
  2.5326486
 
 6.45e-01
 
 0.7177393779
  
 
  
 5
 
 114516315
 
 114516422
 
  PDF   STATS   ENSEMBL   UCSC 
 
  108
 
  4
 
 32223
 
 tss
 
 0.410756
 
 0.0354266
 
 Not queried
 
  6.6880116
 
 6.86e-01
 
 0.7223604052
  
 
  
 15
 
  90455635
 
  90455799
 
  PDF   STATS   ENSEMBL   UCSC 
 
  165
 
  4
 
 13475
 
 gene;island
 
 0.015269
 
 0.0198965
 
 Not queried
 
  4.9969969
 
 6.86e-01
 
 0.7223604052
  
 
  
 18
 
  32398024
 
  32398287
 
  PDF   STATS   ENSEMBL   UCSC 
 
  264
 
  4
 
 19086
 
 gene;tss
 
 0.215588
 
 0.4662638
 
 Not queried
 
  4.6461984
 
 6.86e-01
 
 0.7223604052
  
 
  
 5
 
  79378709
 
  79378955
 
  PDF   STATS   ENSEMBL   UCSC 
 
  247
 
  4
 
 31662
 
 gene
 
 0.497981
 
 0.0187632
 
 Not queried
 
  4.2722597
 
 6.86e-01
 
 0.7223604052
  
 
  
 2
 
 105470558
 
 105470794
 
  PDF   STATS   ENSEMBL   UCSC 
 
  237
 
  4
 
 23550
 
 tss;island
 
 0.125208
 
 0.0189009
 
 Not queried
 
  4.2615648
 
 6.86e-01
 
 0.7223604052
  
 
  
 4
 
  71337658
 
  71337873
 
  PDF   STATS   ENSEMBL   UCSC 
 
  216
 
  4
 
 30016
 
 gene
 
 0.374758
 
 0.6244061
 
 Not queried
 
  3.9951738
 
 6.86e-01
 
 0.7223604052
  
 
  
 10
 
 115998843
 
 115999009
 
  PDF   STATS   ENSEMBL   UCSC 
 
  167
 
  4
 
 5376
 
 tss
 
 0.008597
 
 0.0021103
 
 Not queried
 
  3.9337169
 
 6.86e-01
 
 0.7223604052
  
 
  
 6
 
  29705815
 
  29706048
 
  PDF   STATS   ENSEMBL   UCSC 
 
  234
 
  4
 
 33689
 
 gene
 
 0.253532
 
 0.0267591
 
 Not queried
 
  3.7954979
 
 6.86e-01
 
 0.7223604052
  
 
  
 17
 
   8647944
 
   8648193
 
  PDF   STATS   ENSEMBL   UCSC 
 
  250
 
  5
 
 18007
 
 island
 
 0.721660
 
 0.9789607
 
 Not queried
 
  3.7486231
 
 6.90e-01
 
 0.7223604052
  
 
  
 15
 
  31621629
 
  31621843
 
  PDF   STATS   ENSEMBL   UCSC 
 
  215
 
  4
 
 13273
 
 gene;island
 
 0.142527
 
 0.4588002
 
 Not queried
 
  3.6780978
 
 6.86e-01
 
 0.7223604052
  
 
  
 11
 
  46615333
 
  46615693
 
  PDF   STATS   ENSEMBL   UCSC 
 
  361
 
  4
 
 8252
 
 island
 
 0.010758
 
 0.0071118
 
 Not queried
 
  3.6135061
 
 6.86e-01
 
 0.7223604052
  
 
  
 16
 
  84681507
 
  84681729
 
  PDF   STATS   ENSEMBL   UCSC 
 
  223
 
  4
 
 15393
 
 tss;island
 
 0.264722
 
 0.6276138
 
 Not queried
 
  3.4946437
 
 6.86e-01
 
 0.7223604052
  
 
  
 12
 
  25538177
 
  25538380
 
  PDF   STATS   ENSEMBL   UCSC 
 
  204
 
  4
 
 10292
 
 island
 
 0.106589
 
 0.3004271
 
 Not queried
 
  3.3906179
 
 6.86e-01
 
 0.7223604052
  
 
  
 11
 
 111385338
 
 111385496
 
  PDF   STATS   ENSEMBL   UCSC 
 
  159
 
  5
 
 7687
 
 tss
 
 0.022049
 
 0.0137907
 
 Not queried
 
  3.3651660
 
 6.90e-01
 
 0.7223604052
  
 
  
 13
 
  20138761
 
  20139220
 
  PDF   STATS   ENSEMBL   UCSC 
 
  460
 
  5
 
 11559
 
 island
 
 0.137979
 
 0.5330424
 
 Not queried
 
  3.3114118
 
 6.90e-01
 
 0.7223604052
  
 
  
 11
 
  62621178
 
  62621406
 
  PDF   STATS   ENSEMBL   UCSC 
 
  229
 
  4
 
 6649
 
 gene;tss;island
 
 0.013512
 
 0.0073728
 
 Not queried
 
  3.1627985
 
 6.86e-01
 
 0.7223604052
  
 
  
 16
 
  56677634
 
  56677855
 
  PDF   STATS   ENSEMBL   UCSC 
 
  222
 
  4
 
 14763
 
 gene
 
 0.080407
 
 0.4252559
 
 Not queried
 
  3.0821466
 
 6.86e-01
 
 0.7223604052
  
 
  
 2
 
 182850059
 
 182850441
 
  PDF   STATS   ENSEMBL   UCSC 
 
  383
 
  4
 
 23764
 
 tss
 
 0.031851
 
 0.6128378
 
 Not queried
 
  3.0336603
 
 6.86e-01
 
 0.7223604052
  
 
  
 8
 
  41504884
 
  41505042
 
  PDF   STATS   ENSEMBL   UCSC 
 
  159
 
  5
 
 39671
 
 tss
 
 0.272223
 
 0.0607937
 
 Not queried
 
  3.0305523
 
 6.90e-01
 
 0.7223604052
  
 
  
 11
 
 117296936
 
 117297328
 
  PDF   STATS   ENSEMBL   UCSC 
 
  393
 
  4
 
 8847
 
 island
 
 0.027087
 
 0.5855665
 
 Not queried
 
  2.9709973
 
 6.86e-01
 
 0.7223604052
  
 
  
 10
 
 129534107
 
 129534411
 
  PDF   STATS   ENSEMBL   UCSC 
 
  305
 
  4
 
 5435
 
 tss
 
 0.201693
 
 0.0115884
 
 Not queried
 
  2.9389028
 
 6.86e-01
 
 0.7223604052
  
 
  
 1
 
 214162545
 
 214162854
 
  PDF   STATS   ENSEMBL   UCSC 
 
  310
 
  5
 
 1004
 
 gene;island
 
 0.035173
 
 0.0694327
 
 Not queried
 
  2.8337792
 
 6.90e-01
 
 0.7223604052
  
 
  
 16
 
  28948092
 
  28948319
 
  PDF   STATS   ENSEMBL   UCSC 
 
  228
 
  4
 
 14708
 
 gene;island
 
 0.798091
 
 0.3111220
 
 Not queried
 
  2.8218181
 
 6.86e-01
 
 0.7223604052
  
 
  
 1
 
  59369792
 
  59369954
 
  PDF   STATS   ENSEMBL   UCSC 
 
  163
 
  4
 
 3321
 
 island
 
 0.020760
 
 0.0586502
 
 Not queried
 
  2.7508294
 
 6.86e-01
 
 0.7223604052
  
 
  
 1
 
  10856466
 
  10856739
 
  PDF   STATS   ENSEMBL   UCSC 
 
  274
 
  5
 
 2675
 
 island
 
 0.134248
 
 0.0502371
 
 Not queried
 
  2.7309783
 
 6.90e-01
 
 0.7223604052
  
 
  
 3
 
  21792248
 
  21792684
 
  PDF   STATS   ENSEMBL   UCSC 
 
  437
 
  4
 
 27700
 
 gene
 
 0.042981
 
 0.0226073
 
 Not queried
 
  2.7147323
 
 6.86e-01
 
 0.7223604052
  
 
  
 5
 
    523430
 
    523794
 
  PDF   STATS   ENSEMBL   UCSC 
 
  365
 
  4
 
 31511
 
 gene;island
 
 0.033056
 
 0.0291063
 
 Not queried
 
  2.7027267
 
 6.86e-01
 
 0.7223604052
  
 
  
 1
 
 156051324
 
 156051681
 
  PDF   STATS   ENSEMBL   UCSC 
 
  358
 
  4
 
 813
 
 gene
 
 0.009125
 
 0.0559349
 
 Not queried
 
  2.6735797
 
 6.86e-01
 
 0.7223604052
  
 
  
 16
 
  88038949
 
  88039257
 
  PDF   STATS   ENSEMBL   UCSC 
 
  309
 
  4
 
 14897
 
 gene;island
 
 0.739312
 
 0.5072577
 
 Not queried
 
  2.6658544
 
 6.86e-01
 
 0.7223604052
  
 
  
 16
 
  89390609
 
  89390968
 
  PDF   STATS   ENSEMBL   UCSC 
 
  360
 
  4
 
 14938
 
 gene
 
 0.025052
 
 0.3469676
 
 Not queried
 
  2.6642103
 
 6.86e-01
 
 0.7223604052
  
 
  
 17
 
  35014412
 
  35014676
 
  PDF   STATS   ENSEMBL   UCSC 
 
  265
 
  4
 
 18232
 
 island
 
 0.026460
 
 0.2203379
 
 Not queried
 
  2.6510883
 
 6.86e-01
 
 0.7223604052
  
 
  
 3
 
  64670459
 
  64670640
 
  PDF   STATS   ENSEMBL   UCSC 
 
  182
 
  4
 
 27850
 
 gene;island
 
 0.442801
 
 0.2592019
 
 Not queried
 
  2.5976964
 
 6.86e-01
 
 0.7223604052
  
 
  
 2
 
 182549353
 
 182549604
 
  PDF   STATS   ENSEMBL   UCSC 
 
  252
 
  5
 
 24939
 
 island
 
 0.096290
 
 0.4937362
 
 Not queried
 
  2.5879700
 
 6.90e-01
 
 0.7223604052
  
 
  
 1
 
 119543883
 
 119544329
 
  PDF   STATS   ENSEMBL   UCSC 
 
  447
 
  5
 
 3661
 
 island
 
 0.089517
 
 0.0697744
 
 Not queried
 
  2.5741137
 
 6.90e-01
 
 0.7223604052
  
 
  
 7
 
 126893237
 
 126893362
 
  PDF   STATS   ENSEMBL   UCSC 
 
  126
 
  4
 
 37875
 
 tss;island
 
 0.067972
 
 0.1003687
 
 Not queried
 
  2.5428084
 
 6.86e-01
 
 0.7223604052
  
 
  
 17
 
  53342593
 
  53342740
 
  PDF   STATS   ENSEMBL   UCSC 
 
  148
 
  4
 
 16700
 
 gene;island
 
 0.020060
 
 0.0092028
 
 Not queried
 
  2.5310362
 
 6.86e-01
 
 0.7223604052
  
 
  
 5
 
   3595728
 
   3595977
 
  PDF   STATS   ENSEMBL   UCSC 
 
  250
 
  4
 
 31977
 
 tss;island
 
 0.496714
 
 0.1095521
 
 Not queried
 
  2.5001996
 
 6.86e-01
 
 0.7223604052
  
 
  
 12
 
 123757860
 
 123758062
 
  PDF   STATS   ENSEMBL   UCSC 
 
  203
 
  4
 
 10099
 
 tss;island
 
 0.979664
 
 0.7697336
 
 Not queried
 
  2.4922574
 
 6.86e-01
 
 0.7223604052
  
 
  
 19
 
  14550846
 
  14550999
 
  PDF   STATS   ENSEMBL   UCSC 
 
  154
 
  4
 
 19892
 
 gene;tss;island
 
 0.114997
 
 0.0074450
 
 Not queried
 
  2.4905487
 
 6.86e-01
 
 0.7223604052
  
 
  
 17
 
  46656543
 
  46656690
 
  PDF   STATS   ENSEMBL   UCSC 
 
  148
 
  4
 
 17460
 
 tss;island
 
 0.525402
 
 0.2772957
 
 Not queried
 
  2.4897125
 
 6.86e-01
 
 0.7223604052
  
 
  
 4
 
   8412369
 
   8412795
 
  PDF   STATS   ENSEMBL   UCSC 
 
  427
 
  4
 
 29952
 
 gene
 
 0.518274
 
 0.7071051
 
 Not queried
 
  2.4867395
 
 6.86e-01
 
 0.7223604052
  
 
  
 1
 
 164290179
 
 164290275
 
  PDF   STATS   ENSEMBL   UCSC 
 
   97
 
  4
 
 3924
 
 island
 
 0.070561
 
 0.2172307
 
 Not queried
 
  2.4768565
 
 6.86e-01
 
 0.7223604052
  
 
  
 3
 
  35720994
 
  35721284
 
  PDF   STATS   ENSEMBL   UCSC 
 
  291
 
  5
 
 27712
 
 gene;tss
 
 0.169669
 
 0.0167726
 
 Not queried
 
  2.4742498
 
 6.90e-01
 
 0.7223604052
  
 
  
 16
 
  58497714
 
  58497815
 
  PDF   STATS   ENSEMBL   UCSC 
 
  102
 
  4
 
 14771
 
 gene;tss;island
 
 0.000790
 
 0.0047393
 
 Not queried
 
  2.4596741
 
 6.86e-01
 
 0.7223604052
  
 
  
 7
 
 150211761
 
 150211939
 
  PDF   STATS   ENSEMBL   UCSC 
 
  179
 
  5
 
 37981
 
 tss
 
 0.012873
 
 0.0170158
 
 Not queried
 
  2.4576374
 
 6.90e-01
 
 0.7223604052
  
 
  
 5
 
   1201359
 
   1201713
 
  PDF   STATS   ENSEMBL   UCSC 
 
  355
 
 10
 
 32613
 
 island
 
 0.463140
 
 0.4898382
 
 Not queried
 
  2.4397322
 
 6.84e-01
 
 0.7223604052
  
 
  
 15
 
 101629063
 
 101629375
 
  PDF   STATS   ENSEMBL   UCSC 
 
  313
 
  4
 
 14514
 
 island
 
 0.084007
 
 0.1845655
 
 Not queried
 
  2.4239079
 
 6.86e-01
 
 0.7223604052
  
 
  
 6
 
  50808627
 
  50808813
 
  PDF   STATS   ENSEMBL   UCSC 
 
  187
 
  4
 
 34366
 
 gene;island
 
 0.067105
 
 0.0523690
 
 Not queried
 
  2.3665364
 
 6.86e-01
 
 0.7223604052
  
 
  
 1
 
    917642
 
    917892
 
  PDF   STATS   ENSEMBL   UCSC 
 
  251
 
  5
 
 1136
 
 tss;island
 
 0.050058
 
 0.2125513
 
 Not queried
 
  2.3648690
 
 6.90e-01
 
 0.7223604052
  
 
  
 6
 
 168613133
 
 168613387
 
  PDF   STATS   ENSEMBL   UCSC 
 
  255
 
  4
 
 36660
 
 island
 
 0.542335
 
 0.7643234
 
 Not queried
 
  2.3563684
 
 6.86e-01
 
 0.7223604052
  
 
  
 7
 
 158279694
 
 158279891
 
  PDF   STATS   ENSEMBL   UCSC 
 
  198
 
  4
 
 37376
 
 gene;island
 
 0.373791
 
 0.4885707
 
 Not queried
 
  2.3512332
 
 6.86e-01
 
 0.7223604052
  
 
  
 9
 
 117267242
 
 117267672
 
  PDF   STATS   ENSEMBL   UCSC 
 
  431
 
  5
 
 40780
 
 gene;island
 
 0.008081
 
 0.0349022
 
 Not queried
 
  2.3428189
 
 6.90e-01
 
 0.7223604052
  
 
  
 11
 
  44116911
 
  44117063
 
  PDF   STATS   ENSEMBL   UCSC 
 
  153
 
  5
 
 7260
 
 tss
 
 0.048107
 
 0.0281401
 
 Not queried
 
  2.3398764
 
 6.90e-01
 
 0.7223604052
  
 
  
 1
 
  91182128
 
  91182305
 
  PDF   STATS   ENSEMBL   UCSC 
 
  178
 
  4
 
 624
 
 gene;island
 
 0.240231
 
 0.4773231
 
 Not queried
 
  2.3303510
 
 6.86e-01
 
 0.7223604052
  
 
  
 19
 
  49631042
 
  49631297
 
  PDF   STATS   ENSEMBL   UCSC 
 
  256
 
  4
 
 20143
 
 gene;island
 
 0.047248
 
 0.0142515
 
 Not queried
 
  2.2761249
 
 6.86e-01
 
 0.7223604052
  
 
  
 5
 
 122434853
 
 122435202
 
  PDF   STATS   ENSEMBL   UCSC 
 
  350
 
  4
 
 31732
 
 gene;island
 
 0.053461
 
 0.1759999
 
 Not queried
 
  2.2613180
 
 6.86e-01
 
 0.7223604052
  
 
  
 20
 
  10200138
 
  10200617
 
  PDF   STATS   ENSEMBL   UCSC 
 
  480
 
  5
 
 25357
 
 gene;island
 
 0.007760
 
 0.0133065
 
 Not queried
 
  2.2486337
 
 6.90e-01
 
 0.7223604052
  
 
  
 14
 
  99736387
 
  99736559
 
  PDF   STATS   ENSEMBL   UCSC 
 
  173
 
  4
 
 12202
 
 gene;island
 
 0.015627
 
 0.0243118
 
 Not queried
 
  2.2385208
 
 6.86e-01
 
 0.7223604052
  
 
  
 8
 
 143781297
 
 143781406
 
  PDF   STATS   ENSEMBL   UCSC 
 
  110
 
  4
 
 39933
 
 tss;island
 
 0.085669
 
 0.0879656
 
 Not queried
 
  2.2156736
 
 6.86e-01
 
 0.7223604052
  
 
  
 4
 
  47033126
 
  47033274
 
  PDF   STATS   ENSEMBL   UCSC 
 
  149
 
  5
 
 30376
 
 tss
 
 0.071627
 
 0.0497760
 
 Not queried
 
  2.1973741
 
 6.90e-01
 
 0.7223604052
  
 
  
 16
 
  84029457
 
  84029838
 
  PDF   STATS   ENSEMBL   UCSC 
 
  382
 
  5
 
 14862
 
 gene;island
 
 0.025733
 
 0.0268502
 
 Not queried
 
  2.1929282
 
 6.90e-01
 
 0.7223604052
  
 
  
 12
 
  59313279
 
  59313517
 
  PDF   STATS   ENSEMBL   UCSC 
 
  239
 
  4
 
 9198
 
 gene;island
 
 0.005035
 
 0.0023348
 
 Not queried
 
  2.1850414
 
 6.86e-01
 
 0.7223604052
  
 
  
 8
 
 145133526
 
 145133899
 
  PDF   STATS   ENSEMBL   UCSC 
 
  374
 
  4
 
 39509
 
 gene
 
 0.011102
 
 0.0186937
 
 Not queried
 
  2.1798954
 
 6.86e-01
 
 0.7223604052
  
 
  
 5
 
 113697861
 
 113697946
 
  PDF   STATS   ENSEMBL   UCSC 
 
   86
 
  4
 
 32222
 
 tss
 
 0.126186
 
 0.0508916
 
 Not queried
 
  2.1696779
 
 6.86e-01
 
 0.7223604052
  
 
  
 1
 
  19639298
 
  19639494
 
  PDF   STATS   ENSEMBL   UCSC 
 
  197
 
  4
 
 282
 
 gene;tss;island
 
 0.032820
 
 0.2153276
 
 Not queried
 
  2.1208724
 
 6.86e-01
 
 0.7223604052
  
 
  
 15
 
  72489416
 
  72489828
 
  PDF   STATS   ENSEMBL   UCSC 
 
  413
 
  4
 
 13408
 
 gene;island
 
 0.140460
 
 0.4055419
 
 Not queried
 
  2.1192443
 
 6.86e-01
 
 0.7223604052
  
 
  
 10
 
  71336773
 
  71337104
 
  PDF   STATS   ENSEMBL   UCSC 
 
  332
 
  4
 
 5807
 
 island
 
 0.013686
 
 0.0167100
 
 Not queried
 
  2.1185495
 
 6.86e-01
 
 0.7223604052
  
 
  
 10
 
 129533998
 
 129534411
 
  PDF   STATS   ENSEMBL   UCSC 
 
  414
 
  5
 
 6248
 
 island
 
 0.298415
 
 0.0194293
 
 Not queried
 
  2.1026132
 
 6.90e-01
 
 0.7223604052
  
 
  
 1
 
  24307153
 
  24307535
 
  PDF   STATS   ENSEMBL   UCSC 
 
  383
 
  5
 
 1354
 
 tss;island
 
 0.005897
 
 0.0249386
 
 Not queried
 
  2.1011983
 
 6.90e-01
 
 0.7223604052
  
 
  
 3
 
 157821919
 
 157822072
 
  PDF   STATS   ENSEMBL   UCSC 
 
  154
 
  4
 
 28011
 
 gene;island
 
 0.036300
 
 0.2387128
 
 Not queried
 
  2.0991341
 
 6.86e-01
 
 0.7223604052
  
 
  
 3
 
  42846907
 
  42847116
 
  PDF   STATS   ENSEMBL   UCSC 
 
  210
 
  4
 
 28249
 
 tss;island
 
 0.789597
 
 0.9417809
 
 Not queried
 
  2.0948616
 
 6.86e-01
 
 0.7223604052
  
 
  
 3
 
 172167445
 
 172167810
 
  PDF   STATS   ENSEMBL   UCSC 
 
  366
 
  5
 
 29667
 
 island
 
 0.234779
 
 0.2208060
 
 Not queried
 
  2.0939091
 
 6.90e-01
 
 0.7223604052
  
 
  
 18
 
  44099053
 
  44099340
 
  PDF   STATS   ENSEMBL   UCSC 
 
  288
 
  4
 
 19257
 
 tss
 
 0.016006
 
 0.0704488
 
 Not queried
 
  2.0878920
 
 6.86e-01
 
 0.7223604052
  
 
  
 15
 
  76016056
 
  76016306
 
  PDF   STATS   ENSEMBL   UCSC 
 
  251
 
  4
 
 13818
 
 tss
 
 0.111841
 
 0.3393808
 
 Not queried
 
  2.0843852
 
 6.86e-01
 
 0.7223604052
  
 
  
 1
 
  41827960
 
  41828276
 
  PDF   STATS   ENSEMBL   UCSC 
 
  317
 
  4
 
 3137
 
 island
 
 0.011489
 
 0.0509377
 
 Not queried
 
  2.0820438
 
 6.86e-01
 
 0.7223604052
  
 
  
 3
 
   4508494
 
   4508875
 
  PDF   STATS   ENSEMBL   UCSC 
 
  382
 
  4
 
 27642
 
 gene
 
 0.000764
 
 0.0011242
 
 Not queried
 
  2.0742757
 
 6.86e-01
 
 0.7223604052
  
 
  
 17
 
  61514690
 
  61515010
 
  PDF   STATS   ENSEMBL   UCSC 
 
  321
 
  4
 
 16725
 
 gene;island
 
 0.761078
 
 0.9509752
 
 Not queried
 
  2.0623472
 
 6.86e-01
 
 0.7223604052
  
 
  
 4
 
    619162
 
    619314
 
  PDF   STATS   ENSEMBL   UCSC 
 
  153
 
  4
 
 30226
 
 tss
 
 0.312025
 
 0.4527222
 
 Not queried
 
  2.0584353
 
 6.86e-01
 
 0.7223604052
  
 
  
 2
 
  45161107
 
  45161510
 
  PDF   STATS   ENSEMBL   UCSC 
 
  404
 
  4
 
 24261
 
 island
 
 0.032286
 
 0.0802245
 
 Not queried
 
  2.0562674
 
 6.86e-01
 
 0.7223604052
  
 
  
 19
 
  54106789
 
  54106910
 
  PDF   STATS   ENSEMBL   UCSC 
 
  122
 
  4
 
 21032
 
 tss
 
 0.179169
 
 0.7259045
 
 Not queried
 
  2.0509984
 
 6.86e-01
 
 0.7223604052
  
 
  
 8
 
 146024438
 
 146024786
 
  PDF   STATS   ENSEMBL   UCSC 
 
  349
 
  4
 
 39530
 
 gene;island
 
 0.005985
 
 0.0132274
 
 Not queried
 
  2.0351572
 
 6.86e-01
 
 0.7223604052
  
 
  
 1
 
 181452618
 
 181452839
 
  PDF   STATS   ENSEMBL   UCSC 
 
  222
 
  4
 
 4019
 
 island
 
 0.204332
 
 0.2828037
 
 Not queried
 
  2.0302970
 
 6.86e-01
 
 0.7223604052
  
 
  
 3
 
  49170599
 
  49170849
 
  PDF   STATS   ENSEMBL   UCSC 
 
  251
 
  5
 
 28320
 
 tss
 
 0.152634
 
 0.0723897
 
 Not queried
 
  2.0297355
 
 6.90e-01
 
 0.7223604052
  
 
  
 6
 
 160388924
 
 160389126
 
  PDF   STATS   ENSEMBL   UCSC 
 
  203
 
  4
 
 35325
 
 tss;island
 
 0.960219
 
 0.7794676
 
 Not queried
 
  2.0163089
 
 6.86e-01
 
 0.7223604052
  
 
  
 1
 
 155294935
 
 155295267
 
  PDF   STATS   ENSEMBL   UCSC 
 
  333
 
  4
 
 805
 
 gene;tss;island
 
 0.038679
 
 0.0238238
 
 Not queried
 
  2.0123797
 
 6.86e-01
 
 0.7223604052
  
 
  
 1
 
  45769308
 
  45769557
 
  PDF   STATS   ENSEMBL   UCSC 
 
  250
 
  5
 
 1582
 
 tss
 
 0.002697
 
 0.0055029
 
 Not queried
 
  1.9949753
 
 6.90e-01
 
 0.7223604052
  
 
  
 22
 
  38863905
 
  38864244
 
  PDF   STATS   ENSEMBL   UCSC 
 
  340
 
  5
 
 27490
 
 island
 
 0.048483
 
 0.0116921
 
 Not queried
 
  1.9928673
 
 6.90e-01
 
 0.7223604052
  
 
  
 10
 
 101290958
 
 101291397
 
  PDF   STATS   ENSEMBL   UCSC 
 
  440
 
  4
 
 6011
 
 island
 
 0.124484
 
 0.3033880
 
 Not queried
 
  1.9816295
 
 6.86e-01
 
 0.7223604052
  
 
  
 11
 
 107460901
 
 107461225
 
  PDF   STATS   ENSEMBL   UCSC 
 
  325
 
  4
 
 7662
 
 tss;island
 
 0.022791
 
 0.0273348
 
 Not queried
 
  1.9794480
 
 6.86e-01
 
 0.7223604052
  
 
  
 11
 
  77791290
 
  77791508
 
  PDF   STATS   ENSEMBL   UCSC 
 
  219
 
  4
 
 7589
 
 tss
 
 0.761491
 
 0.3736713
 
 Not queried
 
  1.9696518
 
 6.86e-01
 
 0.7223604052
  
 
  
 17
 
  29032782
 
  29033187
 
  PDF   STATS   ENSEMBL   UCSC 
 
  406
 
  4
 
 18179
 
 island
 
 0.092352
 
 0.2551416
 
 Not queried
 
  1.9657733
 
 6.86e-01
 
 0.7223604052
  
 
  
 6
 
  30126161
 
  30126535
 
  PDF   STATS   ENSEMBL   UCSC 
 
  375
 
  4
 
 33733
 
 gene
 
 0.371910
 
 0.8792458
 
 Not queried
 
  1.9631754
 
 6.86e-01
 
 0.7223604052
  
 
  
 11
 
   1854174
 
   1854368
 
  PDF   STATS   ENSEMBL   UCSC 
 
  195
 
  4
 
 7046
 
 tss
 
 0.018490
 
 0.3608698
 
 Not queried
 
  1.9603320
 
 6.86e-01
 
 0.7223604052
  
 
  
 15
 
  50716705
 
  50716929
 
  PDF   STATS   ENSEMBL   UCSC 
 
  225
 
  4
 
 13347
 
 gene;island
 
 0.006775
 
 0.0033423
 
 Not queried
 
  1.9584192
 
 6.86e-01
 
 0.7223604052
  
 
  
 6
 
  31324916
 
  31324972
 
  PDF   STATS   ENSEMBL   UCSC 
 
   57
 
  5
 
 33870
 
 gene
 
 0.002524
 
 0.0057183
 
 Not queried
 
  1.9515516
 
 6.90e-01
 
 0.7223604052
  
 
  
 10
 
 119293083
 
 119293550
 
  PDF   STATS   ENSEMBL   UCSC 
 
  468
 
  5
 
 4781
 
 gene;island
 
 0.013958
 
 0.1169555
 
 Not queried
 
  1.9501418
 
 6.90e-01
 
 0.7223604052
  
 
  
 4
 
    380157
 
    380529
 
  PDF   STATS   ENSEMBL   UCSC 
 
  373
 
  4
 
 30717
 
 island
 
 0.294166
 
 0.6111464
 
 Not queried
 
  1.9233094
 
 6.86e-01
 
 0.7223604052
  
 
  
 11
 
  32459428
 
  32459954
 
  PDF   STATS   ENSEMBL   UCSC 
 
  527
 
  4
 
 6569
 
 gene;island
 
 0.279749
 
 0.0503974
 
 Not queried
 
  1.9190223
 
 6.86e-01
 
 0.7223604052
  
 
  
 17
 
  61042718
 
  61043072
 
  PDF   STATS   ENSEMBL   UCSC 
 
  355
 
  4
 
 18639
 
 island
 
 0.539329
 
 0.9161967
 
 Not queried
 
  1.9178694
 
 6.86e-01
 
 0.7223604052
  
 
  
 22
 
  45072455
 
  45072724
 
  PDF   STATS   ENSEMBL   UCSC 
 
  270
 
  4
 
 26892
 
 gene
 
 0.076629
 
 0.2372083
 
 Not queried
 
  1.9090174
 
 6.86e-01
 
 0.7223604052
  
 
  
 4
 
 102268799
 
 102268957
 
  PDF   STATS   ENSEMBL   UCSC 
 
  159
 
  5
 
 30517
 
 tss
 
 0.007129
 
 0.0078840
 
 Not queried
 
  1.8928606
 
 6.90e-01
 
 0.7223604052
  
 
  
 3
 
 147140754
 
 147140930
 
  PDF   STATS   ENSEMBL   UCSC 
 
  177
 
  4
 
 29575
 
 island
 
 0.273214
 
 0.0395355
 
 Not queried
 
  1.8922488
 
 6.86e-01
 
 0.7223604052
  
 
  
 16
 
  50099438
 
  50099780
 
  PDF   STATS   ENSEMBL   UCSC 
 
  343
 
  4
 
 15228
 
 tss
 
 0.275058
 
 0.0251630
 
 Not queried
 
  1.8896227
 
 6.86e-01
 
 0.7223604052
  
 
  
 8
 
  62200380
 
  62200518
 
  PDF   STATS   ENSEMBL   UCSC 
 
  139
 
  4
 
 39727
 
 tss
 
 0.037520
 
 0.2694527
 
 Not queried
 
  1.8882920
 
 6.86e-01
 
 0.7223604052
  
 
  
 1
 
  16563727
 
  16563954
 
  PDF   STATS   ENSEMBL   UCSC 
 
  228
 
  5
 
 1281
 
 tss
 
 0.002719
 
 0.0040388
 
 Not queried
 
  1.8740145
 
 6.90e-01
 
 0.7223604052
  
 
  
 17
 
  72462164
 
  72462441
 
  PDF   STATS   ENSEMBL   UCSC 
 
  278
 
  4
 
 17627
 
 tss
 
 0.026344
 
 0.1427795
 
 Not queried
 
  1.8670593
 
 6.86e-01
 
 0.7223604052
  
 
  
 17
 
  74864041
 
  74864302
 
  PDF   STATS   ENSEMBL   UCSC 
 
  262
 
  4
 
 17690
 
 tss;island
 
 0.112547
 
 0.0719962
 
 Not queried
 
  1.8591272
 
 6.86e-01
 
 0.7223604052
  
 
  
 17
 
   4648949
 
   4649333
 
  PDF   STATS   ENSEMBL   UCSC 
 
  385
 
  4
 
 16358
 
 gene
 
 0.513959
 
 0.5084802
 
 Not queried
 
  1.8503753
 
 6.86e-01
 
 0.7223604052
  
 
  
 6
 
  27101633
 
  27101941
 
  PDF   STATS   ENSEMBL   UCSC 
 
  309
 
  4
 
 34712
 
 tss;island
 
 0.004137
 
 0.0033723
 
 Not queried
 
  1.8364892
 
 6.86e-01
 
 0.7223604052
  
 
  
 6
 
  26458189
 
  26458489
 
  PDF   STATS   ENSEMBL   UCSC 
 
  301
 
  4
 
 33612
 
 gene
 
 0.010731
 
 0.0060300
 
 Not queried
 
  1.8333933
 
 6.86e-01
 
 0.7223604052
  
 
  
 22
 
  32599511
 
  32599648
 
  PDF   STATS   ENSEMBL   UCSC 
 
  138
 
  5
 
 26846
 
 gene;tss
 
 0.537350
 
 0.5458295
 
 Not queried
 
  1.8209162
 
 6.90e-01
 
 0.7223604052
  
 
  
 1
 
  14027378
 
  14027581
 
  PDF   STATS   ENSEMBL   UCSC 
 
  204
 
  4
 
 236
 
 gene;island
 
 0.014714
 
 0.0119940
 
 Not queried
 
  1.8138034
 
 6.86e-01
 
 0.7223604052
  
 
  
 16
 
  88815867
 
  88816022
 
  PDF   STATS   ENSEMBL   UCSC 
 
  156
 
  4
 
 14916
 
 gene;island
 
 0.772644
 
 0.9643065
 
 Not queried
 
  1.8106565
 
 6.86e-01
 
 0.7223604052
  
 
  
 19
 
  57337294
 
  57337648
 
  PDF   STATS   ENSEMBL   UCSC 
 
  355
 
  4
 
 20240
 
 gene;tss
 
 0.058603
 
 0.3060058
 
 Not queried
 
  1.8078154
 
 6.86e-01
 
 0.7223604052
  
 
  
 5
 
  38259135
 
  38259398
 
  PDF   STATS   ENSEMBL   UCSC 
 
  264
 
  4
 
 31594
 
 gene;island
 
 0.163367
 
 0.0420373
 
 Not queried
 
  1.8029601
 
 6.86e-01
 
 0.7223604052
  
 
  
 2
 
 158114069
 
 158114300
 
  PDF   STATS   ENSEMBL   UCSC 
 
  232
 
  4
 
 23676
 
 tss
 
 0.332850
 
 0.0522101
 
 Not queried
 
  1.7897323
 
 6.86e-01
 
 0.7223604052
  
 
  
 12
 
  66123127
 
  66123496
 
  PDF   STATS   ENSEMBL   UCSC 
 
  370
 
  4
 
 10600
 
 island
 
 0.380490
 
 0.1222140
 
 Not queried
 
  1.7790426
 
 6.86e-01
 
 0.7223604052
  
 
  
 14
 
 104551867
 
 104552034
 
  PDF   STATS   ENSEMBL   UCSC 
 
  168
 
  4
 
 12656
 
 tss
 
 0.352593
 
 0.6474712
 
 Not queried
 
  1.7769299
 
 6.86e-01
 
 0.7223604052
  
 
  
 3
 
 184032711
 
 184033015
 
  PDF   STATS   ENSEMBL   UCSC 
 
  305
 
  4
 
 28061
 
 gene;island
 
 0.006698
 
 0.0018366
 
 Not queried
 
  1.7762335
 
 6.86e-01
 
 0.7223604052
  
 
  
 11
 
  44330731
 
  44331108
 
  PDF   STATS   ENSEMBL   UCSC 
 
  378
 
  4
 
 6590
 
 gene;island
 
 0.116237
 
 0.0155645
 
 Not queried
 
  1.7707094
 
 6.86e-01
 
 0.7223604052
  
 
  
 15
 
  37402488
 
  37402723
 
  PDF   STATS   ENSEMBL   UCSC 
 
  236
 
  4
 
 14014
 
 island
 
 0.444090
 
 0.0564171
 
 Not queried
 
  1.7706096
 
 6.86e-01
 
 0.7223604052
  
 
  
 1
 
 115396277
 
 115396629
 
  PDF   STATS   ENSEMBL   UCSC 
 
  353
 
  4
 
 1856
 
 tss;island
 
 0.628995
 
 0.8334914
 
 Not queried
 
  1.7691761
 
 6.86e-01
 
 0.7223604052
  
 
  
 7
 
   8480586
 
   8481036
 
  PDF   STATS   ENSEMBL   UCSC 
 
  451
 
  4
 
 36891
 
 gene;island
 
 0.226905
 
 0.1786723
 
 Not queried
 
  1.7688416
 
 6.86e-01
 
 0.7223604052
  
 
  
 7
 
  72935613
 
  72935712
 
  PDF   STATS   ENSEMBL   UCSC 
 
  100
 
  4
 
 37036
 
 gene;island
 
 0.019728
 
 0.0438160
 
 Not queried
 
  1.7666893
 
 6.86e-01
 
 0.7223604052
  
 
  
 6
 
 148881126
 
 148881461
 
  PDF   STATS   ENSEMBL   UCSC 
 
  336
 
  4
 
 36551
 
 island
 
 0.460081
 
 0.2497338
 
 Not queried
 
  1.7625851
 
 6.86e-01
 
 0.7223604052
  
 
  
 7
 
  29519297
 
  29519450
 
  PDF   STATS   ENSEMBL   UCSC 
 
  154
 
  5
 
 37511
 
 tss
 
 0.250872
 
 0.3611472
 
 Not queried
 
  1.7570869
 
 6.90e-01
 
 0.7223604052
  
 
  
 6
 
  50803820
 
  50803945
 
  PDF   STATS   ENSEMBL   UCSC 
 
  126
 
  4
 
 34365
 
 gene
 
 0.051853
 
 0.2900521
 
 Not queried
 
  1.7535865
 
 6.86e-01
 
 0.7223604052
  
 
  
 5
 
 134209784
 
 134210024
 
  PDF   STATS   ENSEMBL   UCSC 
 
  241
 
  4
 
 31753
 
 gene;island
 
 0.024524
 
 0.0204960
 
 Not queried
 
  1.7477541
 
 6.86e-01
 
 0.7223604052
  
 
  
 17
 
  61988667
 
  61988811
 
  PDF   STATS   ENSEMBL   UCSC 
 
  145
 
  4
 
 17576
 
 tss
 
 0.454891
 
 0.4434571
 
 Not queried
 
  1.7456346
 
 6.86e-01
 
 0.7223604052
  
 
  
 19
 
  15530606
 
  15530870
 
  PDF   STATS   ENSEMBL   UCSC 
 
  265
 
  4
 
 20561
 
 tss;island
 
 0.301177
 
 0.6759827
 
 Not queried
 
  1.7409504
 
 6.86e-01
 
 0.7223604052
  
 
  
 15
 
  25513277
 
  25513654
 
  PDF   STATS   ENSEMBL   UCSC 
 
  378
 
  4
 
 13536
 
 tss
 
 0.370627
 
 0.2322563
 
 Not queried
 
  1.7368760
 
 6.86e-01
 
 0.7223604052
  
 
  
 5
 
 140614369
 
 140614765
 
  PDF   STATS   ENSEMBL   UCSC 
 
  397
 
  4
 
 31815
 
 gene;island
 
 0.017645
 
 0.1027415
 
 Not queried
 
  1.7307711
 
 6.86e-01
 
 0.7223604052
  
 
  
 13
 
 112895431
 
 112895794
 
  PDF   STATS   ENSEMBL   UCSC 
 
  364
 
  5
 
 11918
 
 island
 
 0.317027
 
 0.6300337
 
 Not queried
 
  1.7300134
 
 6.90e-01
 
 0.7223604052
  
 
  
 11
 
  67188491
 
  67188871
 
  PDF   STATS   ENSEMBL   UCSC 
 
  381
 
  5
 
 6754
 
 gene;island
 
 0.002737
 
 0.0025524
 
 Not queried
 
  1.7281921
 
 6.90e-01
 
 0.7223604052
  
 
  
 1
 
 103574469
 
 103574619
 
  PDF   STATS   ENSEMBL   UCSC 
 
  151
 
  4
 
 1785
 
 tss
 
 0.593408
 
 0.3742547
 
 Not queried
 
  1.7131437
 
 6.86e-01
 
 0.7223604052
  
 
  
 10
 
   1511024
 
   1511277
 
  PDF   STATS   ENSEMBL   UCSC 
 
  254
 
  4
 
 4466
 
 gene
 
 0.323313
 
 0.6060477
 
 Not queried
 
  1.7007124
 
 6.86e-01
 
 0.7223604052
  
 
  
 19
 
  12868527
 
  12868938
 
  PDF   STATS   ENSEMBL   UCSC 
 
  412
 
  5
 
 19865
 
 gene;island
 
 0.040217
 
 0.0127706
 
 Not queried
 
  1.6951801
 
 6.90e-01
 
 0.7223604052
  
 
  
 5
 
  73980834
 
  73980966
 
  PDF   STATS   ENSEMBL   UCSC 
 
  133
 
  5
 
 32125
 
 tss
 
 0.005882
 
 0.0039400
 
 Not queried
 
  1.6782057
 
 6.90e-01
 
 0.7223604052
  
 
  
 11
 
   1221398
 
   1221812
 
  PDF   STATS   ENSEMBL   UCSC 
 
  415
 
  4
 
 7906
 
 island
 
 0.608778
 
 0.7938220
 
 Not queried
 
  1.6641486
 
 6.86e-01
 
 0.7223604052
  
 
  
 5
 
 140551968
 
 140552163
 
  PDF   STATS   ENSEMBL   UCSC 
 
  196
 
  4
 
 32364
 
 tss
 
 0.040504
 
 0.0426696
 
 Not queried
 
  1.6614128
 
 6.86e-01
 
 0.7223604052
  
 
  
 11
 
  67803771
 
  67804112
 
  PDF   STATS   ENSEMBL   UCSC 
 
  342
 
  4
 
 6767
 
 gene;island
 
 0.753358
 
 0.9514486
 
 Not queried
 
  1.6592721
 
 6.86e-01
 
 0.7223604052
  
 
  
 16
 
    928778
 
    929101
 
  PDF   STATS   ENSEMBL   UCSC 
 
  324
 
  4
 
 14549
 
 gene;island
 
 0.717464
 
 0.7674792
 
 Not queried
 
  1.6509372
 
 6.86e-01
 
 0.7223604052
  
 
  
 6
 
  28317687
 
  28317796
 
  PDF   STATS   ENSEMBL   UCSC 
 
  110
 
  4
 
 33631
 
 gene
 
 0.009072
 
 0.0046274
 
 Not queried
 
  1.6507149
 
 6.86e-01
 
 0.7223604052
  
 
  
 1
 
 230777685
 
 230777875
 
  PDF   STATS   ENSEMBL   UCSC 
 
  191
 
  4
 
 2316
 
 tss;island
 
 0.154502
 
 0.0204816
 
 Not queried
 
  1.6481430
 
 6.86e-01
 
 0.7223604052
  
 
  
 12
 
  54360131
 
  54360511
 
  PDF   STATS   ENSEMBL   UCSC 
 
  381
 
  4
 
 9156
 
 gene;island
 
 0.347041
 
 0.4297930
 
 Not queried
 
  1.6467720
 
 6.86e-01
 
 0.7223604052
  
 
  
 10
 
  72200612
 
  72201008
 
  PDF   STATS   ENSEMBL   UCSC 
 
  397
 
  4
 
 4619
 
 gene;island
 
 0.011116
 
 0.0813750
 
 Not queried
 
  1.6417948
 
 6.86e-01
 
 0.7223604052
  
 
  
 7
 
  50132541
 
  50132764
 
  PDF   STATS   ENSEMBL   UCSC 
 
  224
 
  4
 
 37010
 
 gene
 
 0.020572
 
 0.0158032
 
 Not queried
 
  1.6375557
 
 6.86e-01
 
 0.7223604052
  
 
  
 19
 
  52772664
 
  52772724
 
  PDF   STATS   ENSEMBL   UCSC 
 
   61
 
  4
 
 21010
 
 tss
 
 0.019215
 
 0.0093625
 
 Not queried
 
  1.6365958
 
 6.86e-01
 
 0.7223604052
  
 
  
 5
 
  63802106
 
  63802412
 
  PDF   STATS   ENSEMBL   UCSC 
 
  307
 
  5
 
 32089
 
 tss
 
 0.131210
 
 0.0181527
 
 Not queried
 
  1.6365552
 
 6.90e-01
 
 0.7223604052
  
 
  
 10
 
  71176719
 
  71176853
 
  PDF   STATS   ENSEMBL   UCSC 
 
  135
 
  4
 
 5124
 
 tss
 
 0.464679
 
 0.5799152
 
 Not queried
 
  1.6357932
 
 6.86e-01
 
 0.7223604052
  
 
  
 6
 
 159462964
 
 159463300
 
  PDF   STATS   ENSEMBL   UCSC 
 
  337
 
  4
 
 34532
 
 gene
 
 0.787936
 
 0.9720642
 
 Not queried
 
  1.6339970
 
 6.86e-01
 
 0.7223604052
  
 
  
 1
 
 110163175
 
 110163427
 
  PDF   STATS   ENSEMBL   UCSC 
 
  253
 
  4
 
 670
 
 gene;island
 
 0.017637
 
 0.0475135
 
 Not queried
 
  1.6318695
 
 6.86e-01
 
 0.7223604052
  
 
  
 4
 
  84376750
 
  84376998
 
  PDF   STATS   ENSEMBL   UCSC 
 
  249
 
  5
 
 30045
 
 gene
 
 0.002863
 
 0.0087887
 
 Not queried
 
  1.6309411
 
 6.90e-01
 
 0.7223604052
  
 
  
 12
 
 109826364
 
 109826496
 
  PDF   STATS   ENSEMBL   UCSC 
 
  133
 
  4
 
 9991
 
 tss
 
 0.943809
 
 0.9513428
 
 Not queried
 
  1.6243782
 
 6.86e-01
 
 0.7223604052
  
 
  
 6
 
  32804435
 
  32804677
 
  PDF   STATS   ENSEMBL   UCSC 
 
  243
 
  4
 
 34145
 
 gene;island
 
 0.557786
 
 0.6779277
 
 Not queried
 
  1.6241871
 
 6.86e-01
 
 0.7223604052
  
 
  
 12
 
 120426529
 
 120426784
 
  PDF   STATS   ENSEMBL   UCSC 
 
  256
 
  4
 
 10054
 
 tss;island
 
 0.010290
 
 0.0060968
 
 Not queried
 
  1.6227192
 
 6.86e-01
 
 0.7223604052
  
 
  
 19
 
  48949047
 
  48949463
 
  PDF   STATS   ENSEMBL   UCSC 
 
  417
 
  5
 
 20125
 
 gene
 
 0.007526
 
 0.0027882
 
 Not queried
 
  1.6168353
 
 6.90e-01
 
 0.7223604052
  
 
  
 17
 
   3558202
 
   3558426
 
  PDF   STATS   ENSEMBL   UCSC 
 
  225
 
  4
 
 16342
 
 gene
 
 0.938250
 
 0.9793887
 
 Not queried
 
  1.6167056
 
 6.86e-01
 
 0.7223604052
  
 
  
 15
 
  89089914
 
  89090195
 
  PDF   STATS   ENSEMBL   UCSC 
 
  282
 
  4
 
 13880
 
 tss
 
 0.033950
 
 0.0091001
 
 Not queried
 
  1.6105655
 
 6.86e-01
 
 0.7223604052
  
 
  
 2
 
 124782117
 
 124782263
 
  PDF   STATS   ENSEMBL   UCSC 
 
  147
 
  4
 
 23606
 
 tss;island
 
 0.324011
 
 0.5943983
 
 Not queried
 
  1.6098334
 
 6.86e-01
 
 0.7223604052
  
 
  
 12
 
  54376019
 
  54376241
 
  PDF   STATS   ENSEMBL   UCSC 
 
  223
 
  4
 
 10485
 
 island
 
 0.287527
 
 0.2558260
 
 Not queried
 
  1.6074628
 
 6.86e-01
 
 0.7223604052
  
 
  
 10
 
  88296171
 
  88296605
 
  PDF   STATS   ENSEMBL   UCSC 
 
  435
 
  4
 
 5906
 
 island
 
 0.186183
 
 0.1675723
 
 Not queried
 
  1.6071510
 
 6.86e-01
 
 0.7223604052
  
 
  
 4
 
 159093841
 
 159094034
 
  PDF   STATS   ENSEMBL   UCSC 
 
  194
 
  4
 
 30162
 
 gene
 
 0.003620
 
 0.0029554
 
 Not queried
 
  1.6061089
 
 6.86e-01
 
 0.7223604052
  
 
  
 6
 
  39895360
 
  39895717
 
  PDF   STATS   ENSEMBL   UCSC 
 
  358
 
  5
 
 34326
 
 gene
 
 0.356723
 
 0.6275579
 
 Not queried
 
  1.6038212
 
 6.90e-01
 
 0.7223604052
  
 
  
 1
 
   2273591
 
   2273958
 
  PDF   STATS   ENSEMBL   UCSC 
 
  368
 
  4
 
 76
 
 gene;island
 
 0.243297
 
 0.4251328
 
 Not queried
 
  1.5979369
 
 6.86e-01
 
 0.7223604052
  
 
  
 10
 
  48413742
 
  48413956
 
  PDF   STATS   ENSEMBL   UCSC 
 
  215
 
  4
 
 4577
 
 gene;island
 
 0.501642
 
 0.6338418
 
 Not queried
 
  1.5961995
 
 6.86e-01
 
 0.7223604052
  
 
  
 14
 
  54430382
 
  54430831
 
  PDF   STATS   ENSEMBL   UCSC 
 
  450
 
  4
 
 12854
 
 island
 
 0.008788
 
 0.1020782
 
 Not queried
 
  1.5921016
 
 6.86e-01
 
 0.7223604052
  
 
  
 7
 
  98245716
 
  98246006
 
  PDF   STATS   ENSEMBL   UCSC 
 
  291
 
  4
 
 37747
 
 tss;island
 
 0.242813
 
 0.2155416
 
 Not queried
 
  1.5914301
 
 6.86e-01
 
 0.7223604052
  
 
  
 1
 
 116184219
 
 116184423
 
  PDF   STATS   ENSEMBL   UCSC 
 
  205
 
  4
 
 1859
 
 tss;island
 
 0.141068
 
 0.4281782
 
 Not queried
 
  1.5894408
 
 6.86e-01
 
 0.7223604052
  
 
  
 5
 
  92916862
 
  92917125
 
  PDF   STATS   ENSEMBL   UCSC 
 
  264
 
  4
 
 32959
 
 island
 
 0.011379
 
 0.0144358
 
 Not queried
 
  1.5873543
 
 6.86e-01
 
 0.7223604052
  
 
  
 4
 
    618114
 
    618549
 
  PDF   STATS   ENSEMBL   UCSC 
 
  436
 
  5
 
 30225
 
 tss;island
 
 0.167817
 
 0.6198074
 
 Not queried
 
  1.5839154
 
 6.90e-01
 
 0.7223604052
  
 
  
 1
 
  21044031
 
  21044207
 
  PDF   STATS   ENSEMBL   UCSC 
 
  177
 
  4
 
 294
 
 gene
 
 0.139701
 
 0.0230850
 
 Not queried
 
  1.5779619
 
 6.86e-01
 
 0.7223604052
  
 
  
 2
 
 236826207
 
 236826485
 
  PDF   STATS   ENSEMBL   UCSC 
 
  279
 
  5
 
 23084
 
 gene
 
 0.444953
 
 0.5626523
 
 Not queried
 
  1.5732704
 
 6.90e-01
 
 0.7223604052
  
 
  
 2
 
 133402827
 
 133403009
 
  PDF   STATS   ENSEMBL   UCSC 
 
  183
 
  4
 
 22877
 
 gene;island
 
 0.795915
 
 0.9414569
 
 Not queried
 
  1.5732558
 
 6.86e-01
 
 0.7223604052
  
 
  
 19
 
  14591033
 
  14591345
 
  PDF   STATS   ENSEMBL   UCSC 
 
  313
 
  5
 
 19894
 
 gene;island
 
 0.045884
 
 0.3373916
 
 Not queried
 
  1.5692645
 
 6.90e-01
 
 0.7223604052
  
 
  
 7
 
 157561593
 
 157561925
 
  PDF   STATS   ENSEMBL   UCSC 
 
  333
 
  5
 
 37343
 
 gene;island
 
 0.474082
 
 0.7066988
 
 Not queried
 
  1.5692258
 
 6.90e-01
 
 0.7223604052
  
 
  
 10
 
  75757809
 
  75757817
 
  PDF   STATS   ENSEMBL   UCSC 
 
    9
 
  4
 
 5167
 
 tss
 
 0.005766
 
 0.0023344
 
 Not queried
 
  1.5655289
 
 6.86e-01
 
 0.7223604052
  
 
  
 8
 
  11203882
 
  11204132
 
  PDF   STATS   ENSEMBL   UCSC 
 
  251
 
  4
 
 39204
 
 gene;island
 
 0.636315
 
 0.5242486
 
 Not queried
 
  1.5631560
 
 6.86e-01
 
 0.7223604052
  
 
  
 8
 
  71581111
 
  71581445
 
  PDF   STATS   ENSEMBL   UCSC 
 
  335
 
  4
 
 39333
 
 gene
 
 0.006272
 
 0.0059194
 
 Not queried
 
  1.5627537
 
 6.86e-01
 
 0.7223604052
  
 
  
 2
 
 235406275
 
 235406540
 
  PDF   STATS   ENSEMBL   UCSC 
 
  266
 
  4
 
 23961
 
 tss;island
 
 0.511484
 
 0.7175599
 
 Not queried
 
  1.5615278
 
 6.86e-01
 
 0.7223604052
  
 
  
 1
 
 151966792
 
 151967023
 
  PDF   STATS   ENSEMBL   UCSC 
 
  232
 
  5
 
 1935
 
 tss;island
 
 0.069073
 
 0.0434220
 
 Not queried
 
  1.5597499
 
 6.90e-01
 
 0.7223604052
  
 
  
 12
 
  64784070
 
  64784252
 
  PDF   STATS   ENSEMBL   UCSC 
 
  183
 
  4
 
 9203
 
 gene;island
 
 0.330510
 
 0.1360359
 
 Not queried
 
  1.5584406
 
 6.86e-01
 
 0.7223604052
  
 
  
 6
 
 168920172
 
 168920331
 
  PDF   STATS   ENSEMBL   UCSC 
 
  160
 
  4
 
 34561
 
 gene;island
 
 0.642838
 
 0.7164699
 
 Not queried
 
  1.5578537
 
 6.86e-01
 
 0.7223604052
  
 
  
 4
 
   8153998
 
   8154405
 
  PDF   STATS   ENSEMBL   UCSC 
 
  408
 
  5
 
 29948
 
 gene;island
 
 0.849063
 
 0.8003628
 
 Not queried
 
  1.5573478
 
 6.90e-01
 
 0.7223604052
  
 
  
 13
 
  98605770
 
  98605892
 
  PDF   STATS   ENSEMBL   UCSC 
 
  123
 
  4
 
 11507
 
 tss
 
 0.595759
 
 0.7217355
 
 Not queried
 
  1.5549894
 
 6.86e-01
 
 0.7223604052
  
 
  
 2
 
  88849457
 
  88849830
 
  PDF   STATS   ENSEMBL   UCSC 
 
  374
 
  4
 
 24503
 
 island
 
 0.543745
 
 0.6334495
 
 Not queried
 
  1.5451891
 
 6.86e-01
 
 0.7223604052
  
 
  
 16
 
  88937115
 
  88937386
 
  PDF   STATS   ENSEMBL   UCSC 
 
  272
 
  5
 
 16226
 
 island
 
 0.296307
 
 0.0858042
 
 Not queried
 
  1.5433305
 
 6.90e-01
 
 0.7223604052
  
 
  
 17
 
  80291982
 
  80292252
 
  PDF   STATS   ENSEMBL   UCSC 
 
  271
 
  4
 
 17773
 
 tss;island
 
 0.477117
 
 0.6125284
 
 Not queried
 
  1.5411376
 
 6.86e-01
 
 0.7223604052
  
 
  
 1
 
   1998716
 
   1999183
 
  PDF   STATS   ENSEMBL   UCSC 
 
  468
 
  4
 
 55
 
 gene
 
 0.667227
 
 0.9229309
 
 Not queried
 
  1.5391684
 
 6.86e-01
 
 0.7223604052
  
 
  
 8
 
 108510286
 
 108510343
 
  PDF   STATS   ENSEMBL   UCSC 
 
   58
 
  4
 
 39865
 
 tss
 
 0.015780
 
 0.0089436
 
 Not queried
 
  1.5357471
 
 6.86e-01
 
 0.7223604052
  
 
  
 8
 
  96037118
 
  96037199
 
  PDF   STATS   ENSEMBL   UCSC 
 
   82
 
  5
 
 39822
 
 tss;island
 
 0.007217
 
 0.0097894
 
 Not queried
 
  1.5354036
 
 6.90e-01
 
 0.7223604052
  
 
  
 11
 
 118754280
 
 118754535
 
  PDF   STATS   ENSEMBL   UCSC 
 
  256
 
  5
 
 7739
 
 tss
 
 0.005439
 
 0.0040741
 
 Not queried
 
  1.5323543
 
 6.90e-01
 
 0.7223604052
  
 
  
 12
 
  96882885
 
  96883863
 
  PDF   STATS   ENSEMBL   UCSC 
 
  979
 
  9
 
 10700
 
 island
 
 0.033325
 
 0.3290218
 
 Not queried
 
  1.5104921
 
 6.66e-01
 
 0.7223604052
  
 
  
 9
 
 138670609
 
 138670828
 
  PDF   STATS   ENSEMBL   UCSC 
 
  220
 
  4
 
 40792
 
 gene;island
 
 0.436660
 
 0.5277164
 
 Not queried
 
  1.5075929
 
 6.86e-01
 
 0.7223604052
  
 
  
 1
 
  18806517
 
  18806982
 
  PDF   STATS   ENSEMBL   UCSC 
 
  466
 
  4
 
 1294
 
 tss;island
 
 0.302028
 
 0.5349676
 
 Not queried
 
  1.5056285
 
 6.86e-01
 
 0.7223604052
  
 
  
 2
 
 220041926
 
 220042205
 
  PDF   STATS   ENSEMBL   UCSC 
 
  280
 
  4
 
 23892
 
 tss;island
 
 0.001340
 
 0.0031188
 
 Not queried
 
  1.5037782
 
 6.86e-01
 
 0.7223604052
  
 
  
 10
 
  20105179
 
  20105641
 
  PDF   STATS   ENSEMBL   UCSC 
 
  463
 
  5
 
 5588
 
 island
 
 0.032925
 
 0.3882682
 
 Not queried
 
  1.5013149
 
 6.90e-01
 
 0.7223604052
  
 
  
 11
 
  67169500
 
  67169560
 
  PDF   STATS   ENSEMBL   UCSC 
 
   61
 
  4
 
 7490
 
 tss
 
 0.019396
 
 0.0127071
 
 Not queried
 
  1.5011748
 
 6.86e-01
 
 0.7223604052
  
 
  
 1
 
 228464049
 
 228464394
 
  PDF   STATS   ENSEMBL   UCSC 
 
  346
 
  4
 
 1052
 
 gene;island
 
 0.479038
 
 0.7050606
 
 Not queried
 
  1.5009068
 
 6.86e-01
 
 0.7223604052
  
 
  
 1
 
  47082037
 
  47082211
 
  PDF   STATS   ENSEMBL   UCSC 
 
  175
 
  4
 
 1602
 
 tss
 
 0.006960
 
 0.0074439
 
 Not queried
 
  1.4993430
 
 6.86e-01
 
 0.7223604052
  
 
  
 12
 
  48722990
 
  48723360
 
  PDF   STATS   ENSEMBL   UCSC 
 
  371
 
  4
 
 9112
 
 gene;island
 
 0.670567
 
 0.8186257
 
 Not queried
 
  1.4977984
 
 6.86e-01
 
 0.7223604052
  
 
  
 21
 
  43528734
 
  43528949
 
  PDF   STATS   ENSEMBL   UCSC 
 
  216
 
  4
 
 26435
 
 gene;tss;island
 
 0.489292
 
 0.1918909
 
 Not queried
 
  1.4974117
 
 6.86e-01
 
 0.7223604052
  
 
  
 21
 
  40817540
 
  40817657
 
  PDF   STATS   ENSEMBL   UCSC 
 
  118
 
  4
 
 26551
 
 tss
 
 0.001813
 
 0.0046743
 
 Not queried
 
  1.4848932
 
 6.86e-01
 
 0.7223604052
  
 
  
 17
 
  30244184
 
  30244630
 
  PDF   STATS   ENSEMBL   UCSC 
 
  447
 
  4
 
 18191
 
 island
 
 0.631405
 
 0.4257294
 
 Not queried
 
  1.4840317
 
 6.86e-01
 
 0.7223604052
  
 
  
 2
 
 162930666
 
 162931003
 
  PDF   STATS   ENSEMBL   UCSC 
 
  338
 
  4
 
 22911
 
 gene
 
 0.014854
 
 0.0094031
 
 Not queried
 
  1.4753462
 
 6.86e-01
 
 0.7223604052
  
 
  
 6
 
  33158503
 
  33158597
 
  PDF   STATS   ENSEMBL   UCSC 
 
   95
 
  4
 
 34214
 
 gene;island
 
 0.573614
 
 0.7555737
 
 Not queried
 
  1.4693167
 
 6.86e-01
 
 0.7223604052
  
 
  
 13
 
  53425635
 
  53426032
 
  PDF   STATS   ENSEMBL   UCSC 
 
  398
 
  5
 
 11764
 
 island
 
 0.003455
 
 0.0116660
 
 Not queried
 
  1.4687296
 
 6.90e-01
 
 0.7223604052
  
 
  
 17
 
  80200634
 
  80200974
 
  PDF   STATS   ENSEMBL   UCSC 
 
  341
 
  4
 
 18978
 
 island
 
 0.862812
 
 0.9685401
 
 Not queried
 
  1.4641823
 
 6.86e-01
 
 0.7223604052
  
 
  
 12
 
  52400530
 
  52400667
 
  PDF   STATS   ENSEMBL   UCSC 
 
  138
 
  4
 
 9722
 
 tss;island
 
 0.002536
 
 0.0040654
 
 Not queried
 
  1.4621370
 
 6.86e-01
 
 0.7223604052
  
 
  
 17
 
  58677502
 
  58677584
 
  PDF   STATS   ENSEMBL   UCSC 
 
   83
 
  4
 
 18625
 
 island
 
 0.011032
 
 0.0047564
 
 Not queried
 
  1.4620722
 
 6.86e-01
 
 0.7223604052
  
 
  
 6
 
 134158982
 
 134159412
 
  PDF   STATS   ENSEMBL   UCSC 
 
  431
 
  5
 
 34478
 
 gene;island
 
 0.004665
 
 0.0040842
 
 Not queried
 
  1.4589427
 
 6.90e-01
 
 0.7223604052
  
 
  
 11
 
   2883529
 
   2883912
 
  PDF   STATS   ENSEMBL   UCSC 
 
  384
 
  4
 
 7974
 
 island
 
 0.414326
 
 0.5945703
 
 Not queried
 
  1.4574156
 
 6.86e-01
 
 0.7223604052
  
 
  
 2
 
 242212422
 
 242212528
 
  PDF   STATS   ENSEMBL   UCSC 
 
  107
 
  5
 
 23133
 
 gene;tss;island
 
 0.007516
 
 0.0180498
 
 Not queried
 
  1.4413589
 
 6.90e-01
 
 0.7223604052
  
 
  
 2
 
 105858730
 
 105859083
 
  PDF   STATS   ENSEMBL   UCSC 
 
  354
 
  4
 
 22813
 
 gene;island
 
 0.672949
 
 0.7292338
 
 Not queried
 
  1.4397971
 
 6.86e-01
 
 0.7223604052
  
 
  
 1
 
 152161237
 
 152161683
 
  PDF   STATS   ENSEMBL   UCSC 
 
  447
 
  4
 
 3760
 
 island
 
 0.043360
 
 0.3777941
 
 Not queried
 
  1.4382953
 
 6.86e-01
 
 0.7223604052
  
 
  
 11
 
  65337543
 
  65337913
 
  PDF   STATS   ENSEMBL   UCSC 
 
  371
 
  5
 
 7432
 
 tss
 
 0.002050
 
 0.0055576
 
 Not queried
 
  1.4366707
 
 6.90e-01
 
 0.7223604052
  
 
  
 21
 
  45560663
 
  45560984
 
  PDF   STATS   ENSEMBL   UCSC 
 
  322
 
  4
 
 26452
 
 gene;island
 
 0.630546
 
 0.7321486
 
 Not queried
 
  1.4364504
 
 6.86e-01
 
 0.7223604052
  
 
  
 1
 
   1356565
 
   1356902
 
  PDF   STATS   ENSEMBL   UCSC 
 
  338
 
  4
 
 2437
 
 island
 
 0.480187
 
 0.6390244
 
 Not queried
 
  1.4359518
 
 6.86e-01
 
 0.7223604052
  
 
  
 4
 
  30721997
 
  30722274
 
  PDF   STATS   ENSEMBL   UCSC 
 
  278
 
  4
 
 30970
 
 island
 
 0.208366
 
 0.1329757
 
 Not queried
 
  1.4353865
 
 6.86e-01
 
 0.7223604052
  
 
  
 11
 
    798350
 
    798458
 
  PDF   STATS   ENSEMBL   UCSC 
 
  109
 
  4
 
 7881
 
 island
 
 0.011990
 
 0.0109324
 
 Not queried
 
  1.4308788
 
 6.86e-01
 
 0.7223604052
  
 
  
 4
 
   1020943
 
   1021291
 
  PDF   STATS   ENSEMBL   UCSC 
 
  349
 
  4
 
 30759
 
 island
 
 0.692779
 
 0.7996747
 
 Not queried
 
  1.4301296
 
 6.86e-01
 
 0.7223604052
  
 
  
 19
 
  19651867
 
  19652066
 
  PDF   STATS   ENSEMBL   UCSC 
 
  200
 
  4
 
 19955
 
 gene;island
 
 0.132241
 
 0.1872807
 
 Not queried
 
  1.4291021
 
 6.86e-01
 
 0.7223604052
  
 
  
 6
 
  32057053
 
  32057341
 
  PDF   STATS   ENSEMBL   UCSC 
 
  289
 
  4
 
 34042
 
 gene;island
 
 0.310767
 
 0.5661905
 
 Not queried
 
  1.4280535
 
 6.86e-01
 
 0.7223604052
  
 
  
 12
 
 123319894
 
 123319981
 
  PDF   STATS   ENSEMBL   UCSC 
 
   88
 
  4
 
 10090
 
 tss
 
 0.012611
 
 0.0218331
 
 Not queried
 
  1.4279748
 
 6.86e-01
 
 0.7223604052
  
 
  
 1
 
  26605913
 
  26606110
 
  PDF   STATS   ENSEMBL   UCSC 
 
  198
 
  4
 
 1388
 
 tss;island
 
 0.113943
 
 0.0527164
 
 Not queried
 
  1.4250647
 
 6.86e-01
 
 0.7223604052
  
 
  
 5
 
 177412586
 
 177412718
 
  PDF   STATS   ENSEMBL   UCSC 
 
  133
 
  4
 
 33462
 
 island
 
 0.142418
 
 0.1649053
 
 Not queried
 
  1.4240527
 
 6.86e-01
 
 0.7223604052
  
 
  
 17
 
   1549098
 
   1549434
 
  PDF   STATS   ENSEMBL   UCSC 
 
  337
 
  4
 
 16989
 
 tss
 
 0.438392
 
 0.8442088
 
 Not queried
 
  1.4194744
 
 6.86e-01
 
 0.7223604052
  
 
  
 11
 
 129939665
 
 129939713
 
  PDF   STATS   ENSEMBL   UCSC 
 
   49
 
  4
 
 7816
 
 tss
 
 0.009789
 
 0.0133581
 
 Not queried
 
  1.4142363
 
 6.86e-01
 
 0.7223604052
  
 
  
 7
 
  29725137
 
  29725196
 
  PDF   STATS   ENSEMBL   UCSC 
 
   60
 
  4
 
 37513
 
 tss;island
 
 0.003741
 
 0.0020454
 
 Not queried
 
  1.4130071
 
 6.86e-01
 
 0.7223604052
  
 
  
 15
 
  40633194
 
  40633416
 
  PDF   STATS   ENSEMBL   UCSC 
 
  223
 
  4
 
 13586
 
 tss
 
 0.496786
 
 0.0903798
 
 Not queried
 
  1.4122789
 
 6.86e-01
 
 0.7223604052
  
 
  
 4
 
 174459743
 
 174460124
 
  PDF   STATS   ENSEMBL   UCSC 
 
  382
 
  4
 
 30182
 
 gene;island
 
 0.242621
 
 0.0545611
 
 Not queried
 
  1.4039036
 
 6.86e-01
 
 0.7223604052
  
 
  
 19
 
  49660986
 
  49661083
 
  PDF   STATS   ENSEMBL   UCSC 
 
   98
 
  4
 
 22257
 
 island
 
 0.014077
 
 0.0204678
 
 Not queried
 
  1.4032584
 
 6.86e-01
 
 0.7223604052
  
 
  
 21
 
  46812996
 
  46813298
 
  PDF   STATS   ENSEMBL   UCSC 
 
  303
 
  4
 
 26753
 
 island
 
 0.541063
 
 0.6589324
 
 Not queried
 
  1.4028684
 
 6.86e-01
 
 0.7223604052
  
 
  
 7
 
 155326713
 
 155326803
 
  PDF   STATS   ENSEMBL   UCSC 
 
   91
 
  4
 
 38012
 
 tss;island
 
 0.017807
 
 0.0440460
 
 Not queried
 
  1.4024839
 
 6.86e-01
 
 0.7223604052
  
 
  
 1
 
   1309161
 
   1309641
 
  PDF   STATS   ENSEMBL   UCSC 
 
  481
 
  4
 
 33
 
 gene;island
 
 0.589247
 
 0.7460055
 
 Not queried
 
  1.4002344
 
 6.86e-01
 
 0.7223604052
  
 
  
 1
 
 229406360
 
 229406711
 
  PDF   STATS   ENSEMBL   UCSC 
 
  352
 
  6
 
 2310
 
 tss;island
 
 0.618205
 
 0.3115503
 
 Not queried
 
  2.8015921
 
 6.99e-01
 
 0.7290267580
  
 
  
 5
 
 114516141
 
 114516422
 
  PDF   STATS   ENSEMBL   UCSC 
 
  282
 
  6
 
 33019
 
 island
 
 0.031740
 
 0.0354266
 
 Not queried
 
  2.6745508
 
 6.99e-01
 
 0.7290267580
  
 
  
 15
 
  65689058
 
  65689433
 
  PDF   STATS   ENSEMBL   UCSC 
 
  376
 
  6
 
 13380
 
 gene;island
 
 0.031253
 
 0.0230401
 
 Not queried
 
  2.1107370
 
 6.99e-01
 
 0.7290267580
  
 
  
 10
 
 104211296
 
 104211488
 
  PDF   STATS   ENSEMBL   UCSC 
 
  193
 
  6
 
 6089
 
 island
 
 0.362178
 
 0.4686381
 
 Not queried
 
  2.0527979
 
 6.99e-01
 
 0.7290267580
  
 
  
 3
 
  62861071
 
  62861311
 
  PDF   STATS   ENSEMBL   UCSC 
 
  241
 
  6
 
 28428
 
 tss
 
 0.040649
 
 0.0337872
 
 Not queried
 
  2.0343040
 
 6.99e-01
 
 0.7290267580
  
 
  
 4
 
   2795105
 
   2795501
 
  PDF   STATS   ENSEMBL   UCSC 
 
  397
 
  6
 
 29893
 
 gene;island
 
 0.007113
 
 0.0220613
 
 Not queried
 
  1.8615271
 
 6.99e-01
 
 0.7290267580
  
 
  
 5
 
   1645268
 
   1645802
 
  PDF   STATS   ENSEMBL   UCSC 
 
  535
 
  6
 
 32638
 
 island
 
 0.118910
 
 0.3439365
 
 Not queried
 
  1.8020966
 
 6.99e-01
 
 0.7290267580
  
 
  
 22
 
  19710880
 
  19711051
 
  PDF   STATS   ENSEMBL   UCSC 
 
  172
 
  6
 
 26788
 
 gene;tss;island
 
 0.007788
 
 0.0097924
 
 Not queried
 
  1.6850754
 
 6.99e-01
 
 0.7290267580
  
 
  
 20
 
  53091600
 
  53092259
 
  PDF   STATS   ENSEMBL   UCSC 
 
  660
 
  6
 
 25831
 
 tss
 
 0.072733
 
 0.0156724
 
 Not queried
 
  1.6361048
 
 6.99e-01
 
 0.7290267580
  
 
  
 6
 
  52284632
 
  52284958
 
  PDF   STATS   ENSEMBL   UCSC 
 
  327
 
  6
 
 35032
 
 tss
 
 0.003368
 
 0.0052754
 
 Not queried
 
  1.6113998
 
 6.99e-01
 
 0.7290267580
  
 
  
 7
 
 100199764
 
 100200009
 
  PDF   STATS   ENSEMBL   UCSC 
 
  246
 
  6
 
 38770
 
 island
 
 0.759082
 
 0.5270757
 
 Not queried
 
  1.5926214
 
 6.99e-01
 
 0.7290267580
  
 
  
 4
 
   7069843
 
   7070051
 
  PDF   STATS   ENSEMBL   UCSC 
 
  209
 
  6
 
 30290
 
 tss
 
 0.012311
 
 0.0035354
 
 Not queried
 
  1.5150468
 
 6.99e-01
 
 0.7290267580
  
 
  
 19
 
  54676948
 
  54677232
 
  PDF   STATS   ENSEMBL   UCSC 
 
  285
 
  6
 
 21055
 
 tss
 
 0.227455
 
 0.0951465
 
 Not queried
 
  1.4771241
 
 6.99e-01
 
 0.7290267580
  
 
  
 3
 
  33260732
 
  33261204
 
  PDF   STATS   ENSEMBL   UCSC 
 
  473
 
  7
 
 28212
 
 tss;island
 
 0.076077
 
 0.0778799
 
 Not queried
 
  2.3873885
 
 7.10e-01
 
 0.7390730975
  
 
  
 5
 
 134181484
 
 134181707
 
  PDF   STATS   ENSEMBL   UCSC 
 
  224
 
  7
 
 31752
 
 gene;island
 
 0.003010
 
 0.0029341
 
 Not queried
 
  1.6753136
 
 7.10e-01
 
 0.7390730975
  
 
  
 20
 
  39995539
 
  39995747
 
  PDF   STATS   ENSEMBL   UCSC 
 
  209
 
  7
 
 25746
 
 tss;island
 
 0.040089
 
 0.0268830
 
 Not queried
 
  1.5419808
 
 7.10e-01
 
 0.7390730975
  
 
  
 14
 
 105767366
 
 105767639
 
  PDF   STATS   ENSEMBL   UCSC 
 
  274
 
  7
 
 12670
 
 tss;island
 
 0.003269
 
 0.0061061
 
 Not queried
 
  1.5036940
 
 7.10e-01
 
 0.7390730975
  
 
  
 4
 
  66536186
 
  66536772
 
  PDF   STATS   ENSEMBL   UCSC 
 
  587
 
  7
 
 30417
 
 tss;island
 
 0.007378
 
 0.0256086
 
 Not queried
 
  1.5022890
 
 7.10e-01
 
 0.7390730975
  
 
  
 12
 
  52695197
 
  52695623
 
  PDF   STATS   ENSEMBL   UCSC 
 
  427
 
  7
 
 9726
 
 tss
 
 0.426838
 
 0.7443484
 
 Not queried
 
  1.4971192
 
 7.10e-01
 
 0.7390730975
  
 
  
 8
 
 109095918
 
 109096151
 
  PDF   STATS   ENSEMBL   UCSC 
 
  234
 
  7
 
 39866
 
 tss
 
 0.439271
 
 0.1656072
 
 Not queried
 
  1.4760508
 
 7.10e-01
 
 0.7390730975
  
 
  
 2
 
 132439861
 
 132440298
 
  PDF   STATS   ENSEMBL   UCSC 
 
  438
 
  7
 
 24737
 
 island
 
 0.051002
 
 0.0508964
 
 Not queried
 
  1.4540568
 
 7.10e-01
 
 0.7390730975
  
 
  
 2
 
 241938159
 
 241938436
 
  PDF   STATS   ENSEMBL   UCSC 
 
  278
 
  7
 
 25283
 
 island
 
 0.007660
 
 0.0079186
 
 Not queried
 
  1.4339440
 
 7.10e-01
 
 0.7390730975
  
 
  
 5
 
   1201359
 
   1201698
 
  PDF   STATS   ENSEMBL   UCSC 
 
  340
 
  9
 
 31967
 
 tss
 
 0.463418
 
 0.5544233
 
 Not queried
 
  2.3295551
 
 7.30e-01
 
 0.7597603748
  
 
  
 19
 
   1401118
 
   1401781
 
  PDF   STATS   ENSEMBL   UCSC 
 
  664
 
  9
 
 21222
 
 island
 
 0.010237
 
 0.0158888
 
 Not queried
 
  1.6707109
 
 7.96e-01
 
 0.8275104336
  
 
  
 1
 
 153643319
 
 153643822
 
  PDF   STATS   ENSEMBL   UCSC 
 
  504
 
  9
 
 3766
 
 island
 
 0.006951
 
 0.0087503
 
 Not queried
 
  1.6675098
 
 7.96e-01
 
 0.8275104336
  
 
  
 6
 
  75915730
 
  75915885
 
  PDF   STATS   ENSEMBL   UCSC 
 
  156
 
  9
 
 35074
 
 tss;island
 
 0.082749
 
 0.1456539
 
 Not queried
 
  1.4005706
 
 7.96e-01
 
 0.8275104336
  
 
  
 5
 
 140562562
 
 140563400
 
  PDF   STATS   ENSEMBL   UCSC 
 
  839
 
  8
 
 31813
 
 gene;island
 
 0.226750
 
 0.2349964
 
 Not queried
 
  2.0500477
 
 7.98e-01
 
 0.8290400976
  
 
  
 2
 
  42396143
 
  42396458
 
  PDF   STATS   ENSEMBL   UCSC 
 
  316
 
  8
 
 23319
 
 tss;island
 
 0.014713
 
 0.0514026
 
 Not queried
 
  1.4526571
 
 7.98e-01
 
 0.8290400976
  
 
  
 4
 
    206112
 
    206562
 
  PDF   STATS   ENSEMBL   UCSC 
 
  451
 
  8
 
 30714
 
 island
 
 0.355295
 
 0.4012904
 
 Not queried
 
  1.4473054
 
 7.98e-01
 
 0.8290400976
  
 
  
 7
 
  14029285
 
  14029701
 
  PDF   STATS   ENSEMBL   UCSC 
 
  417
 
  8
 
 36899
 
 gene;island
 
 0.014668
 
 0.0141362
 
 Not queried
 
  1.4176711
 
 7.98e-01
 
 0.8290400976
  
 
  
 19
 
  14016603
 
  14016822
 
  PDF   STATS   ENSEMBL   UCSC 
 
  220
 
  7
 
 19887
 
 gene
 
 0.022725
 
 0.1228958
 
 Not queried
 
  2.2437767
 
 8.05e-01
 
 0.8347790563
  
 
  
 19
 
  34918900
 
  34919248
 
  PDF   STATS   ENSEMBL   UCSC 
 
  349
 
  7
 
 20692
 
 tss
 
 0.003519
 
 0.0013785
 
 Not queried
 
  2.1094456
 
 8.05e-01
 
 0.8347790563
  
 
  
 10
 
 120925308
 
 120925632
 
  PDF   STATS   ENSEMBL   UCSC 
 
  325
 
  7
 
 5399
 
 tss
 
 0.004929
 
 0.0074475
 
 Not queried
 
  1.6825131
 
 8.05e-01
 
 0.8347790563
  
 
  
 1
 
 223899685
 
 223899998
 
  PDF   STATS   ENSEMBL   UCSC 
 
  314
 
  7
 
 1025
 
 gene;tss;island
 
 0.482488
 
 0.4827912
 
 Not queried
 
  1.6649963
 
 8.05e-01
 
 0.8347790563
  
 
  
 3
 
  49967462
 
  49967713
 
  PDF   STATS   ENSEMBL   UCSC 
 
  252
 
  6
 
 28340
 
 tss
 
 0.240927
 
 0.4677453
 
 Not queried
 
  2.3015917
 
 8.18e-01
 
 0.8469867211
  
 
  
 6
 
  41343210
 
  41343830
 
  PDF   STATS   ENSEMBL   UCSC 
 
  621
 
  6
 
 36120
 
 island
 
 0.018414
 
 0.0392064
 
 Not queried
 
  2.1595639
 
 8.18e-01
 
 0.8469867211
  
 
  
 22
 
  19949873
 
  19950040
 
  PDF   STATS   ENSEMBL   UCSC 
 
  168
 
  6
 
 26970
 
 tss
 
 0.296361
 
 0.6026653
 
 Not queried
 
  2.0985811
 
 8.18e-01
 
 0.8469867211
  
 
  
 18
 
  47792917
 
  47793175
 
  PDF   STATS   ENSEMBL   UCSC 
 
  259
 
  6
 
 19274
 
 tss
 
 0.009137
 
 0.0186194
 
 Not queried
 
  1.8926842
 
 8.18e-01
 
 0.8469867211
  
 
  
 5
 
 140529499
 
 140529819
 
  PDF   STATS   ENSEMBL   UCSC 
 
  321
 
  6
 
 32363
 
 tss;island
 
 0.086443
 
 0.1504914
 
 Not queried
 
  1.8537045
 
 8.18e-01
 
 0.8469867211
  
 
  
 1
 
  53392492
 
  53392881
 
  PDF   STATS   ENSEMBL   UCSC 
 
  390
 
  6
 
 1634
 
 tss
 
 0.035837
 
 0.3646636
 
 Not queried
 
  1.6811515
 
 8.18e-01
 
 0.8469867211
  
 
  
 6
 
  32634245
 
  32634508
 
  PDF   STATS   ENSEMBL   UCSC 
 
  264
 
  6
 
 35922
 
 island
 
 0.017910
 
 0.0188413
 
 Not queried
 
  1.5879479
 
 8.18e-01
 
 0.8469867211
  
 
  
 20
 
  22565881
 
  22566289
 
  PDF   STATS   ENSEMBL   UCSC 
 
  409
 
  6
 
 25633
 
 tss;island
 
 0.033777
 
 0.0477360
 
 Not queried
 
  1.5164130
 
 8.18e-01
 
 0.8469867211
  
 
  
 11
 
  61519687
 
  61520084
 
  PDF   STATS   ENSEMBL   UCSC 
 
  398
 
  5
 
 7337
 
 tss
 
 0.168782
 
 0.0221014
 
 Not queried
 
  3.9510369
 
 8.41e-01
 
 0.8635565145
  
 
  
 2
 
  63283806
 
  63284132
 
  PDF   STATS   ENSEMBL   UCSC 
 
  327
 
  5
 
 22705
 
 gene;island
 
 0.007541
 
 0.0058027
 
 Not queried
 
  2.5345540
 
 8.41e-01
 
 0.8635565145
  
 
  
 20
 
  45179226
 
  45179413
 
  PDF   STATS   ENSEMBL   UCSC 
 
  188
 
  5
 
 25793
 
 tss
 
 0.510187
 
 0.4486714
 
 Not queried
 
  2.4187100
 
 8.41e-01
 
 0.8635565145
  
 
  
 2
 
 178976981
 
 178977094
 
  PDF   STATS   ENSEMBL   UCSC 
 
  114
 
  5
 
 23753
 
 tss
 
 0.020174
 
 0.0060979
 
 Not queried
 
  2.4070499
 
 8.41e-01
 
 0.8635565145
  
 
  
 8
 
  42234724
 
  42234975
 
  PDF   STATS   ENSEMBL   UCSC 
 
  252
 
  5
 
 39678
 
 tss
 
 0.166024
 
 0.4783922
 
 Not queried
 
  2.2040891
 
 8.41e-01
 
 0.8635565145
  
 
  
 2
 
 119600348
 
 119600627
 
  PDF   STATS   ENSEMBL   UCSC 
 
  280
 
  5
 
 22840
 
 gene;island
 
 0.085682
 
 0.1225383
 
 Not queried
 
  2.0277123
 
 8.41e-01
 
 0.8635565145
  
 
  
 12
 
 115105220
 
 115105710
 
  PDF   STATS   ENSEMBL   UCSC 
 
  491
 
  5
 
 10845
 
 island
 
 0.039170
 
 0.1356963
 
 Not queried
 
  1.9443248
 
 8.41e-01
 
 0.8635565145
  
 
  
 1
 
 150254280
 
 150254546
 
  PDF   STATS   ENSEMBL   UCSC 
 
  267
 
  5
 
 1905
 
 tss;island
 
 0.020837
 
 0.0341387
 
 Not queried
 
  1.9201454
 
 8.41e-01
 
 0.8635565145
  
 
  
 20
 
   1165065
 
   1165435
 
  PDF   STATS   ENSEMBL   UCSC 
 
  371
 
  5
 
 25933
 
 island
 
 0.550017
 
 0.4843414
 
 Not queried
 
  1.9089988
 
 8.41e-01
 
 0.8635565145
  
 
  
 3
 
  62305003
 
  62305285
 
  PDF   STATS   ENSEMBL   UCSC 
 
  283
 
  5
 
 28425
 
 tss;island
 
 0.203835
 
 0.1294322
 
 Not queried
 
  1.9068674
 
 8.41e-01
 
 0.8635565145
  
 
  
 7
 
 121956502
 
 121956890
 
  PDF   STATS   ENSEMBL   UCSC 
 
  389
 
  5
 
 38874
 
 island
 
 0.009693
 
 0.0339019
 
 Not queried
 
  1.8876004
 
 8.41e-01
 
 0.8635565145
  
 
  
 8
 
  53477881
 
  53478102
 
  PDF   STATS   ENSEMBL   UCSC 
 
  222
 
  5
 
 40293
 
 island
 
 0.094092
 
 0.0850991
 
 Not queried
 
  1.8787287
 
 8.41e-01
 
 0.8635565145
  
 
  
 1
 
   2046506
 
   2046928
 
  PDF   STATS   ENSEMBL   UCSC 
 
  423
 
  5
 
 61
 
 gene;island
 
 0.621083
 
 0.7438763
 
 Not queried
 
  1.8557744
 
 8.41e-01
 
 0.8635565145
  
 
  
 12
 
 113917395
 
 113917737
 
  PDF   STATS   ENSEMBL   UCSC 
 
  343
 
  5
 
 10835
 
 island
 
 0.029910
 
 0.0164595
 
 Not queried
 
  1.8062351
 
 8.41e-01
 
 0.8635565145
  
 
  
 12
 
 133463607
 
 133464074
 
  PDF   STATS   ENSEMBL   UCSC 
 
  468
 
  5
 
 9469
 
 gene;island
 
 0.024006
 
 0.0219735
 
 Not queried
 
  1.7674523
 
 8.41e-01
 
 0.8635565145
  
 
  
 12
 
  54346784
 
  54347242
 
  PDF   STATS   ENSEMBL   UCSC 
 
  459
 
  5
 
 10481
 
 island
 
 0.035339
 
 0.1591735
 
 Not queried
 
  1.7608065
 
 8.41e-01
 
 0.8635565145
  
 
  
 12
 
  53496842
 
  53497147
 
  PDF   STATS   ENSEMBL   UCSC 
 
  306
 
  5
 
 9733
 
 tss
 
 0.025601
 
 0.1481273
 
 Not queried
 
  1.6896527
 
 8.41e-01
 
 0.8635565145
  
 
  
 8
 
 144790187
 
 144790410
 
  PDF   STATS   ENSEMBL   UCSC 
 
  224
 
  5
 
 40681
 
 island
 
 0.582268
 
 0.8182489
 
 Not queried
 
  1.6846589
 
 8.41e-01
 
 0.8635565145
  
 
  
 7
 
   4243286
 
   4243734
 
  PDF   STATS   ENSEMBL   UCSC 
 
  449
 
  5
 
 36834
 
 gene
 
 0.783010
 
 0.6980930
 
 Not queried
 
  1.6745910
 
 8.41e-01
 
 0.8635565145
  
 
  
 1
 
  17231486
 
  17231793
 
  PDF   STATS   ENSEMBL   UCSC 
 
  308
 
  5
 
 2750
 
 island
 
 0.004369
 
 0.0034223
 
 Not queried
 
  1.6617825
 
 8.41e-01
 
 0.8635565145
  
 
  
 1
 
  42921582
 
  42921899
 
  PDF   STATS   ENSEMBL   UCSC 
 
  318
 
  5
 
 465
 
 gene
 
 0.013771
 
 0.0146973
 
 Not queried
 
  1.5857617
 
 8.41e-01
 
 0.8635565145
  
 
  
 16
 
  74331461
 
  74331660
 
  PDF   STATS   ENSEMBL   UCSC 
 
  200
 
  5
 
 14840
 
 gene;island
 
 0.125896
 
 0.1247919
 
 Not queried
 
  1.5806109
 
 8.41e-01
 
 0.8635565145
  
 
  
 8
 
 110657174
 
 110657197
 
  PDF   STATS   ENSEMBL   UCSC 
 
   24
 
  5
 
 39873
 
 tss
 
 0.001345
 
 0.0032085
 
 Not queried
 
  1.5734988
 
 8.41e-01
 
 0.8635565145
  
 
  
 13
 
 111367171
 
 111367259
 
  PDF   STATS   ENSEMBL   UCSC 
 
   89
 
  5
 
 11240
 
 gene;tss;island
 
 0.008566
 
 0.0216853
 
 Not queried
 
  1.5671306
 
 8.41e-01
 
 0.8635565145
  
 
  
 5
 
  65222276
 
  65222522
 
  PDF   STATS   ENSEMBL   UCSC 
 
  247
 
  5
 
 32840
 
 island
 
 0.002963
 
 0.0072061
 
 Not queried
 
  1.5638968
 
 8.41e-01
 
 0.8635565145
  
 
  
 11
 
  82444798
 
  82445170
 
  PDF   STATS   ENSEMBL   UCSC 
 
  373
 
  5
 
 8698
 
 island
 
 0.127372
 
 0.2931931
 
 Not queried
 
  1.5140739
 
 8.41e-01
 
 0.8635565145
  
 
  
 1
 
  98511523
 
  98511908
 
  PDF   STATS   ENSEMBL   UCSC 
 
  386
 
  5
 
 3514
 
 island
 
 0.028644
 
 0.0774009
 
 Not queried
 
  1.5011552
 
 8.41e-01
 
 0.8635565145
  
 
  
 1
 
  26394201
 
  26394593
 
  PDF   STATS   ENSEMBL   UCSC 
 
  393
 
  5
 
 1382
 
 tss
 
 0.274436
 
 0.5619599
 
 Not queried
 
  1.4934077
 
 8.41e-01
 
 0.8635565145
  
 
  
 18
 
  59560365
 
  59560495
 
  PDF   STATS   ENSEMBL   UCSC 
 
  131
 
  5
 
 19301
 
 tss
 
 0.026083
 
 0.0611366
 
 Not queried
 
  1.4618213
 
 8.41e-01
 
 0.8635565145
  
 
  
 1
 
 151371944
 
 151372335
 
  PDF   STATS   ENSEMBL   UCSC 
 
  392
 
  5
 
 3745
 
 island
 
 0.002110
 
 0.0052985
 
 Not queried
 
  1.4482268
 
 8.41e-01
 
 0.8635565145
  
 
  
 2
 
 229046325
 
 229046785
 
  PDF   STATS   ENSEMBL   UCSC 
 
  461
 
  5
 
 25170
 
 island
 
 0.140716
 
 0.2110473
 
 Not queried
 
  1.4473419
 
 8.41e-01
 
 0.8635565145
  
 
  
 17
 
    146404
 
    146926
 
  PDF   STATS   ENSEMBL   UCSC 
 
  523
 
  5
 
 16286
 
 gene
 
 0.307926
 
 0.4073764
 
 Not queried
 
  1.4276205
 
 8.41e-01
 
 0.8635565145
  
 
  
 19
 
  15618961
 
  15619355
 
  PDF   STATS   ENSEMBL   UCSC 
 
  395
 
  5
 
 21709
 
 island
 
 0.032841
 
 0.0792637
 
 Not queried
 
  1.4191737
 
 8.41e-01
 
 0.8635565145
  
 
  
 10
 
 124578028
 
 124578544
 
  PDF   STATS   ENSEMBL   UCSC 
 
  517
 
  5
 
 6210
 
 island
 
 0.351666
 
 0.5091883
 
 Not queried
 
  1.4136889
 
 8.41e-01
 
 0.8635565145
  
 
  
 14
 
  29234890
 
  29235469
 
  PDF   STATS   ENSEMBL   UCSC 
 
  580
 
 11
 
 12335
 
 tss;island
 
 0.079030
 
 0.2028200
 
 Not queried
 
  1.9918755
 
 8.47e-01
 
 0.8692219522
  
 
  
 1
 
  52521668
 
  52521894
 
  PDF   STATS   ENSEMBL   UCSC 
 
  227
 
  4
 
 1628
 
 tss;island
 
 0.002389
 
 0.0017682
 
 Not queried
 
  3.6510658
 
 8.86e-01
 
 0.8941250130
  
 
  
 5
 
  83017000
 
  83017184
 
  PDF   STATS   ENSEMBL   UCSC 
 
  185
 
  4
 
 32160
 
 tss;island
 
 0.728577
 
 0.4729371
 
 Not queried
 
  3.4880323
 
 8.86e-01
 
 0.8941250130
  
 
  
 2
 
     47150
 
     47452
 
  PDF   STATS   ENSEMBL   UCSC 
 
  303
 
  4
 
 23151
 
 tss;island
 
 0.303830
 
 0.0592925
 
 Not queried
 
  2.9318538
 
 8.86e-01
 
 0.8941250130
  
 
  
 16
 
  53468112
 
  53468205
 
  PDF   STATS   ENSEMBL   UCSC 
 
   94
 
  4
 
 15235
 
 tss
 
 0.107969
 
 0.1996397
 
 Not queried
 
  2.7150475
 
 8.86e-01
 
 0.8941250130
  
 
  
 7
 
 154684327
 
 154684562
 
  PDF   STATS   ENSEMBL   UCSC 
 
  236
 
  4
 
 37304
 
 gene;island
 
 0.588014
 
 0.7324579
 
 Not queried
 
  2.7058374
 
 8.86e-01
 
 0.8941250130
  
 
  
 16
 
  83171068
 
  83171314
 
  PDF   STATS   ENSEMBL   UCSC 
 
  247
 
  4
 
 14860
 
 gene
 
 0.339118
 
 0.0320173
 
 Not queried
 
  2.6840132
 
 8.86e-01
 
 0.8941250130
  
 
  
 5
 
 142077712
 
 142078020
 
  PDF   STATS   ENSEMBL   UCSC 
 
  309
 
  4
 
 32400
 
 tss
 
 0.022167
 
 0.2579818
 
 Not queried
 
  2.6459144
 
 8.86e-01
 
 0.8941250130
  
 
  
 8
 
  65292217
 
  65292498
 
  PDF   STATS   ENSEMBL   UCSC 
 
  282
 
  4
 
 40343
 
 island
 
 0.523981
 
 0.1036686
 
 Not queried
 
  2.5269122
 
 8.86e-01
 
 0.8941250130
  
 
  
 18
 
   3498854
 
   3499253
 
  PDF   STATS   ENSEMBL   UCSC 
 
  400
 
  4
 
 19044
 
 gene;island
 
 0.424270
 
 0.3375687
 
 Not queried
 
  2.4192914
 
 8.86e-01
 
 0.8941250130
  
 
  
 17
 
  79503642
 
  79503877
 
  PDF   STATS   ENSEMBL   UCSC 
 
  236
 
  4
 
 16914
 
 gene;island
 
 0.024893
 
 0.1643360
 
 Not queried
 
  2.3427172
 
 8.86e-01
 
 0.8941250130
  
 
  
 1
 
   7023495
 
   7023864
 
  PDF   STATS   ENSEMBL   UCSC 
 
  370
 
  4
 
 184
 
 gene;island
 
 0.119176
 
 0.0446165
 
 Not queried
 
  2.2741518
 
 8.86e-01
 
 0.8941250130
  
 
  
 1
 
 156215418
 
 156215727
 
  PDF   STATS   ENSEMBL   UCSC 
 
  310
 
  4
 
 818
 
 gene;island
 
 0.024103
 
 0.0231470
 
 Not queried
 
  2.2579104
 
 8.86e-01
 
 0.8941250130
  
 
  
 19
 
  51524658
 
  51524828
 
  PDF   STATS   ENSEMBL   UCSC 
 
  171
 
  4
 
 20991
 
 tss;island
 
 0.126573
 
 0.0176172
 
 Not queried
 
  2.2230842
 
 8.86e-01
 
 0.8941250130
  
 
  
 19
 
  41317837
 
  41318235
 
  PDF   STATS   ENSEMBL   UCSC 
 
  399
 
  4
 
 22043
 
 island
 
 0.028557
 
 0.1319943
 
 Not queried
 
  2.2230010
 
 8.86e-01
 
 0.8941250130
  
 
  
 3
 
 184972080
 
 184972153
 
  PDF   STATS   ENSEMBL   UCSC 
 
   74
 
  4
 
 28766
 
 tss;island
 
 0.283849
 
 0.4331436
 
 Not queried
 
  2.2193981
 
 8.86e-01
 
 0.8941250130
  
 
  
 8
 
  48100081
 
  48100186
 
  PDF   STATS   ENSEMBL   UCSC 
 
  106
 
  4
 
 40269
 
 island
 
 0.015737
 
 0.0141942
 
 Not queried
 
  2.1788637
 
 8.86e-01
 
 0.8941250130
  
 
  
 14
 
 102094825
 
 102095139
 
  PDF   STATS   ENSEMBL   UCSC 
 
  315
 
  4
 
 13161
 
 island
 
 0.040883
 
 0.0430369
 
 Not queried
 
  2.1267680
 
 8.86e-01
 
 0.8941250130
  
 
  
 5
 
 139040820
 
 139041014
 
  PDF   STATS   ENSEMBL   UCSC 
 
  195
 
  4
 
 31778
 
 gene;island
 
 0.012660
 
 0.0129514
 
 Not queried
 
  2.1200177
 
 8.86e-01
 
 0.8941250130
  
 
  
 19
 
  55314879
 
  55315034
 
  PDF   STATS   ENSEMBL   UCSC 
 
  156
 
  4
 
 21065
 
 tss
 
 0.298899
 
 0.4717482
 
 Not queried
 
  2.0923344
 
 8.86e-01
 
 0.8941250130
  
 
  
 1
 
  51433840
 
  51434014
 
  PDF   STATS   ENSEMBL   UCSC 
 
  175
 
  4
 
 1618
 
 tss;island
 
 0.005299
 
 0.0027346
 
 Not queried
 
  2.0893105
 
 8.86e-01
 
 0.8941250130
  
 
  
 7
 
   1902804
 
   1903159
 
  PDF   STATS   ENSEMBL   UCSC 
 
  356
 
  4
 
 36770
 
 gene;island
 
 0.726993
 
 0.9525028
 
 Not queried
 
  2.0694534
 
 8.86e-01
 
 0.8941250130
  
 
  
 14
 
  68067023
 
  68067294
 
  PDF   STATS   ENSEMBL   UCSC 
 
  272
 
  4
 
 12473
 
 tss
 
 0.024361
 
 0.0270307
 
 Not queried
 
  2.0448578
 
 8.86e-01
 
 0.8941250130
  
 
  
 2
 
 171784945
 
 171785124
 
  PDF   STATS   ENSEMBL   UCSC 
 
  180
 
  4
 
 23710
 
 tss;island
 
 0.743451
 
 0.6884419
 
 Not queried
 
  2.0343689
 
 8.86e-01
 
 0.8941250130
  
 
  
 1
 
  16259191
 
  16259499
 
  PDF   STATS   ENSEMBL   UCSC 
 
  309
 
  4
 
 256
 
 gene;island
 
 0.799862
 
 0.9738319
 
 Not queried
 
  2.0171585
 
 8.86e-01
 
 0.8941250130
  
 
  
 4
 
  84377120
 
  84377400
 
  PDF   STATS   ENSEMBL   UCSC 
 
  281
 
  4
 
 30046
 
 gene
 
 0.002189
 
 0.0028095
 
 Not queried
 
  1.9503351
 
 8.86e-01
 
 0.8941250130
  
 
  
 6
 
  10882927
 
  10883105
 
  PDF   STATS   ENSEMBL   UCSC 
 
  179
 
  4
 
 34632
 
 tss;island
 
 0.370985
 
 0.1954796
 
 Not queried
 
  1.9362762
 
 8.86e-01
 
 0.8941250130
  
 
  
 6
 
  26020672
 
  26020714
 
  PDF   STATS   ENSEMBL   UCSC 
 
   43
 
  4
 
 34678
 
 tss;island
 
 0.000546
 
 0.0020977
 
 Not queried
 
  1.9281980
 
 8.86e-01
 
 0.8941250130
  
 
  
 5
 
 139135602
 
 139135961
 
  PDF   STATS   ENSEMBL   UCSC 
 
  360
 
  4
 
 33178
 
 island
 
 0.023920
 
 0.0401517
 
 Not queried
 
  1.9045002
 
 8.86e-01
 
 0.8941250130
  
 
  
 19
 
   7562249
 
   7562555
 
  PDF   STATS   ENSEMBL   UCSC 
 
  307
 
  4
 
 21450
 
 island
 
 0.392704
 
 0.2581591
 
 Not queried
 
  1.8822050
 
 8.86e-01
 
 0.8941250130
  
 
  
 20
 
  31870775
 
  31870935
 
  PDF   STATS   ENSEMBL   UCSC 
 
  161
 
  4
 
 25681
 
 tss
 
 0.137513
 
 0.0388358
 
 Not queried
 
  1.8661508
 
 8.86e-01
 
 0.8941250130
  
 
  
 10
 
 134755601
 
 134755955
 
  PDF   STATS   ENSEMBL   UCSC 
 
  355
 
  4
 
 4883
 
 gene;island
 
 0.047796
 
 0.0604170
 
 Not queried
 
  1.8589189
 
 8.86e-01
 
 0.8941250130
  
 
  
 17
 
  79868964
 
  79869136
 
  PDF   STATS   ENSEMBL   UCSC 
 
  173
 
  4
 
 16927
 
 gene;island
 
 0.006008
 
 0.0043972
 
 Not queried
 
  1.8436516
 
 8.86e-01
 
 0.8941250130
  
 
  
 5
 
 153863417
 
 153863826
 
  PDF   STATS   ENSEMBL   UCSC 
 
  410
 
  4
 
 33333
 
 island
 
 0.197720
 
 0.0629747
 
 Not queried
 
  1.8249795
 
 8.86e-01
 
 0.8941250130
  
 
  
 16
 
   3989076
 
   3989432
 
  PDF   STATS   ENSEMBL   UCSC 
 
  357
 
  4
 
 15657
 
 island
 
 0.074236
 
 0.0077266
 
 Not queried
 
  1.8129295
 
 8.86e-01
 
 0.8941250130
  
 
  
 6
 
 135501674
 
 135501969
 
  PDF   STATS   ENSEMBL   UCSC 
 
  296
 
  4
 
 35237
 
 tss;island
 
 0.008885
 
 0.0010404
 
 Not queried
 
  1.8040115
 
 8.86e-01
 
 0.8941250130
  
 
  
 2
 
 229046406
 
 229046785
 
  PDF   STATS   ENSEMBL   UCSC 
 
  380
 
  4
 
 23933
 
 tss
 
 0.235960
 
 0.2805691
 
 Not queried
 
  1.7677759
 
 8.86e-01
 
 0.8941250130
  
 
  
 1
 
  39546511
 
  39546688
 
  PDF   STATS   ENSEMBL   UCSC 
 
  178
 
  4
 
 1520
 
 tss;island
 
 0.564070
 
 0.3614233
 
 Not queried
 
  1.7224573
 
 8.86e-01
 
 0.8941250130
  
 
  
 11
 
  86085623
 
  86085757
 
  PDF   STATS   ENSEMBL   UCSC 
 
  135
 
  4
 
 7609
 
 tss
 
 0.161909
 
 0.0540376
 
 Not queried
 
  1.7117861
 
 8.86e-01
 
 0.8941250130
  
 
  
 7
 
 123673549
 
 123673690
 
  PDF   STATS   ENSEMBL   UCSC 
 
  142
 
  4
 
 37873
 
 tss
 
 0.077381
 
 0.0723958
 
 Not queried
 
  1.7067405
 
 8.86e-01
 
 0.8941250130
  
 
  
 3
 
  39425775
 
  39426101
 
  PDF   STATS   ENSEMBL   UCSC 
 
  327
 
  4
 
 27725
 
 gene;island
 
 0.009439
 
 0.0187756
 
 Not queried
 
  1.7025806
 
 8.86e-01
 
 0.8941250130
  
 
  
 15
 
  65360366
 
  65360486
 
  PDF   STATS   ENSEMBL   UCSC 
 
  121
 
  4
 
 14225
 
 island
 
 0.043904
 
 0.2252410
 
 Not queried
 
  1.7006804
 
 8.86e-01
 
 0.8941250130
  
 
  
 16
 
  67876973
 
  67877413
 
  PDF   STATS   ENSEMBL   UCSC 
 
  441
 
  4
 
 14803
 
 gene;island
 
 0.042795
 
 0.0451095
 
 Not queried
 
  1.6978290
 
 8.86e-01
 
 0.8941250130
  
 
  
 4
 
 185938720
 
 185938989
 
  PDF   STATS   ENSEMBL   UCSC 
 
  270
 
  4
 
 30698
 
 tss;island
 
 0.015349
 
 0.0086446
 
 Not queried
 
  1.6939292
 
 8.86e-01
 
 0.8941250130
  
 
  
 3
 
 171175713
 
 171176016
 
  PDF   STATS   ENSEMBL   UCSC 
 
  304
 
  4
 
 28031
 
 gene;island
 
 0.045041
 
 0.1765733
 
 Not queried
 
  1.6856420
 
 8.86e-01
 
 0.8941250130
  
 
  
 1
 
  46958475
 
  46958856
 
  PDF   STATS   ENSEMBL   UCSC 
 
  382
 
  4
 
 3220
 
 island
 
 0.094186
 
 0.0872342
 
 Not queried
 
  1.6341316
 
 8.86e-01
 
 0.8941250130
  
 
  
 19
 
  51531297
 
  51531484
 
  PDF   STATS   ENSEMBL   UCSC 
 
  188
 
  4
 
 20992
 
 tss;island
 
 0.044374
 
 0.0926669
 
 Not queried
 
  1.6330715
 
 8.86e-01
 
 0.8941250130
  
 
  
 12
 
  12223661
 
  12223989
 
  PDF   STATS   ENSEMBL   UCSC 
 
  329
 
  4
 
 9567
 
 tss
 
 0.507922
 
 0.5244277
 
 Not queried
 
  1.5982005
 
 8.86e-01
 
 0.8941250130
  
 
  
 2
 
 203499669
 
 203499802
 
  PDF   STATS   ENSEMBL   UCSC 
 
  134
 
  4
 
 23826
 
 tss;island
 
 0.012250
 
 0.0086901
 
 Not queried
 
  1.5770850
 
 8.86e-01
 
 0.8941250130
  
 
  
 1
 
   2391121
 
   2391347
 
  PDF   STATS   ENSEMBL   UCSC 
 
  227
 
  4
 
 2503
 
 island
 
 0.607213
 
 0.6577949
 
 Not queried
 
  1.5759109
 
 8.86e-01
 
 0.8941250130
  
 
  
 1
 
 236445668
 
 236445970
 
  PDF   STATS   ENSEMBL   UCSC 
 
  303
 
  4
 
 2342
 
 tss;island
 
 0.008587
 
 0.0146609
 
 Not queried
 
  1.5523155
 
 8.86e-01
 
 0.8941250130
  
 
  
 11
 
  17565744
 
  17565885
 
  PDF   STATS   ENSEMBL   UCSC 
 
  142
 
  4
 
 6525
 
 gene
 
 0.284984
 
 0.0413574
 
 Not queried
 
  1.5467853
 
 8.86e-01
 
 0.8941250130
  
 
  
 15
 
  68723690
 
  68724065
 
  PDF   STATS   ENSEMBL   UCSC 
 
  376
 
  4
 
 13396
 
 gene;island
 
 0.029782
 
 0.0296511
 
 Not queried
 
  1.5334683
 
 8.86e-01
 
 0.8941250130
  
 
  
 11
 
  57414401
 
  57414626
 
  PDF   STATS   ENSEMBL   UCSC 
 
  226
 
  4
 
 6615
 
 gene;island
 
 0.312824
 
 0.5100594
 
 Not queried
 
  1.5260771
 
 8.86e-01
 
 0.8941250130
  
 
  
 14
 
  57263993
 
  57264287
 
  PDF   STATS   ENSEMBL   UCSC 
 
  295
 
  4
 
 12875
 
 island
 
 0.216194
 
 0.0787654
 
 Not queried
 
  1.5146417
 
 8.86e-01
 
 0.8941250130
  
 
  
 6
 
  31782675
 
  31782761
 
  PDF   STATS   ENSEMBL   UCSC 
 
   87
 
  4
 
 33967
 
 gene
 
 0.003158
 
 0.0161408
 
 Not queried
 
  1.5010585
 
 8.86e-01
 
 0.8941250130
  
 
  
 16
 
  31548640
 
  31548908
 
  PDF   STATS   ENSEMBL   UCSC 
 
  269
 
  4
 
 15881
 
 island
 
 0.544134
 
 0.4855602
 
 Not queried
 
  1.5004950
 
 8.86e-01
 
 0.8941250130
  
 
  
 7
 
  50628718
 
  50629067
 
  PDF   STATS   ENSEMBL   UCSC 
 
  350
 
  4
 
 37012
 
 gene
 
 0.148880
 
 0.3298638
 
 Not queried
 
  1.4997263
 
 8.86e-01
 
 0.8941250130
  
 
  
 11
 
  66511804
 
  66512052
 
  PDF   STATS   ENSEMBL   UCSC 
 
  249
 
  4
 
 7480
 
 tss;island
 
 0.005839
 
 0.0076777
 
 Not queried
 
  1.4985198
 
 8.86e-01
 
 0.8941250130
  
 
  
 19
 
  17377962
 
  17378213
 
  PDF   STATS   ENSEMBL   UCSC 
 
  252
 
  4
 
 20583
 
 tss
 
 0.019630
 
 0.0138786
 
 Not queried
 
  1.4690046
 
 8.86e-01
 
 0.8941250130
  
 
  
 11
 
   1274018
 
   1274269
 
  PDF   STATS   ENSEMBL   UCSC 
 
  252
 
  4
 
 6406
 
 gene;island
 
 0.618844
 
 0.6427314
 
 Not queried
 
  1.4629949
 
 8.86e-01
 
 0.8941250130
  
 
  
 1
 
 111022652
 
 111022914
 
  PDF   STATS   ENSEMBL   UCSC 
 
  263
 
  4
 
 1816
 
 tss
 
 0.091135
 
 0.2317514
 
 Not queried
 
  1.4580081
 
 8.86e-01
 
 0.8941250130
  
 
  
 1
 
   8938701
 
   8938754
 
  PDF   STATS   ENSEMBL   UCSC 
 
   54
 
  4
 
 198
 
 gene
 
 0.002770
 
 0.0041118
 
 Not queried
 
  1.4501780
 
 8.86e-01
 
 0.8941250130
  
 
  
 21
 
  38593636
 
  38593754
 
  PDF   STATS   ENSEMBL   UCSC 
 
  119
 
  4
 
 26678
 
 island
 
 0.003741
 
 0.0153592
 
 Not queried
 
  1.4434360
 
 8.86e-01
 
 0.8941250130
  
 
  
 15
 
  34610829
 
  34611069
 
  PDF   STATS   ENSEMBL   UCSC 
 
  241
 
  4
 
 13277
 
 gene
 
 0.003161
 
 0.0054743
 
 Not queried
 
  1.4366641
 
 8.86e-01
 
 0.8941250130
  
 
  
 2
 
 171671480
 
 171671795
 
  PDF   STATS   ENSEMBL   UCSC 
 
  316
 
  4
 
 24849
 
 island
 
 0.212698
 
 0.2004900
 
 Not queried
 
  1.4136467
 
 8.86e-01
 
 0.8941250130
  
 
  
 4
 
  85422998
 
  85423302
 
  PDF   STATS   ENSEMBL   UCSC 
 
  305
 
  4
 
 31162
 
 island
 
 0.030936
 
 0.0766657
 
 Not queried
 
  1.4096414
 
 8.86e-01
 
 0.8941250130
  
 
  
 20
 
  42574332
 
  42574362
 
  PDF   STATS   ENSEMBL   UCSC 
 
   31
 
  4
 
 25423
 
 gene;tss
 
 0.878759
 
 0.9003424
 
 Not queried
 
  1.4055219
 
 8.86e-01
 
 0.8941250130
  
 
  
 2
 
 239228826
 
 239229395
 
  PDF   STATS   ENSEMBL   UCSC 
 
  570
 
  7
 
 25244
 
 island
 
 0.004386
 
 0.0025213
 
 Not queried
 
  2.1446325
 
 9.02e-01
 
 0.9091904204
  
 
  
 1
 
 246670726
 
 246670940
 
  PDF   STATS   ENSEMBL   UCSC 
 
  215
 
  7
 
 2362
 
 tss;island
 
 0.010834
 
 0.0021820
 
 Not queried
 
  1.8491093
 
 9.02e-01
 
 0.9091904204
  
 
  
 3
 
 138725153
 
 138725425
 
  PDF   STATS   ENSEMBL   UCSC 
 
  273
 
  7
 
 28627
 
 tss;island
 
 0.940352
 
 0.8682768
 
 Not queried
 
  1.5284853
 
 9.02e-01
 
 0.9091904204
  
 
  
 11
 
  15136059
 
  15136475
 
  PDF   STATS   ENSEMBL   UCSC 
 
  417
 
  7
 
 7169
 
 tss
 
 0.290035
 
 0.3028075
 
 Not queried
 
  1.4607781
 
 9.02e-01
 
 0.9091904204
  
 
  
 19
 
   1238119
 
   1238538
 
  PDF   STATS   ENSEMBL   UCSC 
 
  420
 
  6
 
 20294
 
 tss;island
 
 0.075356
 
 0.0346337
 
 Not queried
 
  1.9831816
 
 9.37e-01
 
 0.9445195058
  
 
  
 8
 
  70378380
 
  70378760
 
  PDF   STATS   ENSEMBL   UCSC 
 
  381
 
  6
 
 39752
 
 tss
 
 0.005704
 
 0.0047013
 
 Not queried
 
  1.4140637
 
 9.37e-01
 
 0.9445195058
  
 
  
 1
 
 166853278
 
 166853812
 
  PDF   STATS   ENSEMBL   UCSC 
 
  535
 
  6
 
 3940
 
 island
 
 0.141660
 
 0.5108695
 
 Not queried
 
  1.4009569
 
 9.37e-01
 
 0.9445195058
  
 
  
 20
 
  30618874
 
  30619244
 
  PDF   STATS   ENSEMBL   UCSC 
 
  371
 
  5
 
 25388
 
 gene;island
 
 0.319723
 
 0.3822562
 
 Not queried
 
  2.7455884
 
 1.00e+00
 
 1.0000000000
  
 
  
 16
 
  51187388
 
  51187807
 
  PDF   STATS   ENSEMBL   UCSC 
 
  420
 
  5
 
 15920
 
 island
 
 0.126862
 
 0.0519300
 
 Not queried
 
  2.5595784
 
 1.00e+00
 
 1.0000000000
  
 
  
 3
 
  38388371
 
  38388808
 
  PDF   STATS   ENSEMBL   UCSC 
 
  438
 
  5
 
 27719
 
 gene
 
 0.035975
 
 0.0311888
 
 Not queried
 
  2.3333939
 
 1.00e+00
 
 1.0000000000
  
 
  
 11
 
 118135094
 
 118135407
 
  PDF   STATS   ENSEMBL   UCSC 
 
  314
 
  7
 
 7729
 
 tss
 
 0.120378
 
 0.4720755
 
 Not queried
 
  2.0369597
 
 1.00e+00
 
 1.0000000000
  
 
  
 1
 
  45452509
 
  45452605
 
  PDF   STATS   ENSEMBL   UCSC 
 
   97
 
  4
 
 1578
 
 tss
 
 0.275807
 
 0.0569409
 
 Not queried
 
  2.0245556
 
 1.00e+00
 
 1.0000000000
  
 
  
 11
 
  73498584
 
  73499134
 
  PDF   STATS   ENSEMBL   UCSC 
 
  551
 
  5
 
 8644
 
 island
 
 0.019097
 
 0.0090291
 
 Not queried
 
  1.9625367
 
 1.00e+00
 
 1.0000000000
  
 
  
 6
 
  33360495
 
  33360836
 
  PDF   STATS   ENSEMBL   UCSC 
 
  342
 
  5
 
 34272
 
 gene;island
 
 0.076133
 
 0.0206651
 
 Not queried
 
  1.9560222
 
 1.00e+00
 
 1.0000000000
  
 
  
 17
 
  75243296
 
  75243679
 
  PDF   STATS   ENSEMBL   UCSC 
 
  384
 
  4
 
 18803
 
 island
 
 0.016991
 
 0.0172077
 
 Not queried
 
  1.9333352
 
 1.00e+00
 
 1.0000000000
  
 
  
 13
 
 113540400
 
 113540631
 
  PDF   STATS   ENSEMBL   UCSC 
 
  232
 
  5
 
 11262
 
 gene;island
 
 0.386447
 
 0.5351819
 
 Not queried
 
  1.9104490
 
 1.00e+00
 
 1.0000000000
  
 
  
 5
 
 137475177
 
 137475532
 
  PDF   STATS   ENSEMBL   UCSC 
 
  356
 
  4
 
 32313
 
 tss
 
 0.014759
 
 0.1309918
 
 Not queried
 
  1.9052634
 
 1.00e+00
 
 1.0000000000
  
 
  
 10
 
 104211319
 
 104211488
 
  PDF   STATS   ENSEMBL   UCSC 
 
  170
 
  5
 
 5325
 
 tss
 
 0.576781
 
 0.4559739
 
 Not queried
 
  1.8906039
 
 1.00e+00
 
 1.0000000000
  
 
  
 5
 
 156696185
 
 156696520
 
  PDF   STATS   ENSEMBL   UCSC 
 
  336
 
  4
 
 31874
 
 gene;island
 
 0.112000
 
 0.1753423
 
 Not queried
 
  1.8783766
 
 1.00e+00
 
 1.0000000000
  
 
  
 1
 
  47695138
 
  47695475
 
  PDF   STATS   ENSEMBL   UCSC 
 
  338
 
  4
 
 3230
 
 island
 
 0.059152
 
 0.0566000
 
 Not queried
 
  1.8484303
 
 1.00e+00
 
 1.0000000000
  
 
  
 11
 
  32458656
 
  32459057
 
  PDF   STATS   ENSEMBL   UCSC 
 
  402
 
  5
 
 6568
 
 gene;island
 
 0.125520
 
 0.0461392
 
 Not queried
 
  1.8083467
 
 1.00e+00
 
 1.0000000000
  
 
  
 22
 
  19949873
 
  19950166
 
  PDF   STATS   ENSEMBL   UCSC 
 
  294
 
  7
 
 26794
 
 gene
 
 0.416448
 
 0.5344807
 
 Not queried
 
  1.7732491
 
 1.00e+00
 
 1.0000000000
  
 
  
 12
 
 108955167
 
 108955348
 
  PDF   STATS   ENSEMBL   UCSC 
 
  182
 
  6
 
 9982
 
 tss;island
 
 0.000809
 
 0.0012391
 
 Not queried
 
  1.7473758
 
 1.00e+00
 
 1.0000000000
  
 
  
 1
 
 117529478
 
 117530009
 
  PDF   STATS   ENSEMBL   UCSC 
 
  532
 
  5
 
 711
 
 gene
 
 0.680912
 
 0.9573233
 
 Not queried
 
  1.7205117
 
 1.00e+00
 
 1.0000000000
  
 
  
 17
 
  37856133
 
  37856786
 
  PDF   STATS   ENSEMBL   UCSC 
 
  654
 
  9
 
 16553
 
 gene;island
 
 0.153808
 
 0.0328318
 
 Not queried
 
  1.7197835
 
 1.00e+00
 
 1.0000000000
  
 
  
 11
 
  17743610
 
  17743946
 
  PDF   STATS   ENSEMBL   UCSC 
 
  337
 
  5
 
 8104
 
 island
 
 0.032096
 
 0.0999297
 
 Not queried
 
  1.7171937
 
 1.00e+00
 
 1.0000000000
  
 
  
 11
 
  20181725
 
  20182122
 
  PDF   STATS   ENSEMBL   UCSC 
 
  398
 
  8
 
 8124
 
 island
 
 0.185624
 
 0.3204307
 
 Not queried
 
  1.6606833
 
 1.00e+00
 
 1.0000000000
  
 
  
 7
 
   1277521
 
   1277790
 
  PDF   STATS   ENSEMBL   UCSC 
 
  270
 
  4
 
 38092
 
 island
 
 0.292693
 
 0.2282559
 
 Not queried
 
  1.6590611
 
 1.00e+00
 
 1.0000000000
  
 
  
 11
 
  33891541
 
  33891924
 
  PDF   STATS   ENSEMBL   UCSC 
 
  384
 
  5
 
 6579
 
 gene;tss;island
 
 0.026037
 
 0.0258473
 
 Not queried
 
  1.6378023
 
 1.00e+00
 
 1.0000000000
  
 
  
 3
 
  46924768
 
  46925181
 
  PDF   STATS   ENSEMBL   UCSC 
 
  414
 
  4
 
 27756
 
 gene;island
 
 0.038351
 
 0.0082836
 
 Not queried
 
  1.6318993
 
 1.00e+00
 
 1.0000000000
  
 
  
 7
 
    193229
 
    193732
 
  PDF   STATS   ENSEMBL   UCSC 
 
  504
 
  5
 
 36701
 
 gene;island
 
 0.129964
 
 0.1777295
 
 Not queried
 
  1.6277657
 
 1.00e+00
 
 1.0000000000
  
 
  
 5
 
 110427798
 
 110427863
 
  PDF   STATS   ENSEMBL   UCSC 
 
   66
 
  4
 
 32207
 
 tss
 
 0.011894
 
 0.0192052
 
 Not queried
 
  1.6116952
 
 1.00e+00
 
 1.0000000000
  
 
  
 3
 
 139257713
 
 139257907
 
  PDF   STATS   ENSEMBL   UCSC 
 
  195
 
  5
 
 27969
 
 gene;island
 
 0.435385
 
 0.2028965
 
 Not queried
 
  1.5990398
 
 1.00e+00
 
 1.0000000000
  
 
  
 11
 
 118134801
 
 118134959
 
  PDF   STATS   ENSEMBL   UCSC 
 
  159
 
  4
 
 6927
 
 gene
 
 0.033305
 
 0.0279764
 
 Not queried
 
  1.5955711
 
 1.00e+00
 
 1.0000000000
  
 
  
 6
 
   6004686
 
   6004897
 
  PDF   STATS   ENSEMBL   UCSC 
 
  212
 
  4
 
 33545
 
 gene;island
 
 0.094257
 
 0.1277059
 
 Not queried
 
  1.5912024
 
 1.00e+00
 
 1.0000000000
  
 
  
 1
 
  12538341
 
  12538678
 
  PDF   STATS   ENSEMBL   UCSC 
 
  338
 
  4
 
 233
 
 gene
 
 0.328798
 
 0.4550101
 
 Not queried
 
  1.5807261
 
 1.00e+00
 
 1.0000000000
  
 
  
 7
 
   4901628
 
   4901798
 
  PDF   STATS   ENSEMBL   UCSC 
 
  171
 
  4
 
 37417
 
 tss
 
 0.572535
 
 0.4919895
 
 Not queried
 
  1.5360260
 
 1.00e+00
 
 1.0000000000
  
 
  
 5
 
   1298511
 
   1298965
 
  PDF   STATS   ENSEMBL   UCSC 
 
  455
 
  6
 
 32625
 
 island
 
 0.568223
 
 0.6092532
 
 Not queried
 
  1.5245275
 
 1.00e+00
 
 1.0000000000
  
 
  
 5
 
 153857789
 
 153858102
 
  PDF   STATS   ENSEMBL   UCSC 
 
  314
 
  6
 
 33329
 
 island
 
 0.066140
 
 0.0408608
 
 Not queried
 
  1.4659611
 
 1.00e+00
 
 1.0000000000
  
 
  
 19
 
   1503042
 
   1503812
 
  ENSEMBL   UCSC 
 
  771
 
  7
 
 21229
 
 island
 
 0.086675
 
 0.0682116
 
 Not queried
 
  1.3999028
 
       NA
 
           NA
  
 
  
 1
 
 228404763
 
 228404947
 
  ENSEMBL   UCSC 
 
  185
 
  4
 
 1051
 
 gene;island
 
 0.871263
 
 0.9403076
 
 Not queried
 
  1.3997345
 
       NA
 
           NA
  
 
  
 19
 
  51293367
 
  51293677
 
  ENSEMBL   UCSC 
 
  311
 
  6
 
 22323
 
 island
 
 0.774266
 
 0.8171248
 
 Not queried
 
  1.3991811
 
       NA
 
           NA
  
 
  
 1
 
   4715467
 
   4715615
 
  ENSEMBL   UCSC 
 
  149
 
  4
 
 152
 
 gene;island
 
 0.047909
 
 0.0199737
 
 Not queried
 
  1.3988324
 
       NA
 
           NA
  
 
  
 6
 
 112041290
 
 112041490
 
  ENSEMBL   UCSC 
 
  201
 
  4
 
 35178
 
 tss
 
 0.668109
 
 0.8156859
 
 Not queried
 
  1.3982512
 
       NA
 
           NA
  
 
  
 6
 
  32054441
 
  32055738
 
  ENSEMBL   UCSC 
 
 1298
 
 29
 
 34040
 
 gene;island
 
 0.021414
 
 0.2456839
 
 Not queried
 
  1.3977630
 
       NA
 
           NA
  
 
  
 19
 
  48216390
 
  48216613
 
  ENSEMBL   UCSC 
 
  224
 
  6
 
 22200
 
 island
 
 0.006985
 
 0.0282834
 
 Not queried
 
  1.3977628
 
       NA
 
           NA
  
 
  
 10
 
 112432175
 
 112432418
 
  ENSEMBL   UCSC 
 
  244
 
  4
 
 4756
 
 gene;island
 
 0.008055
 
 0.0211019
 
 Not queried
 
  1.3972898
 
       NA
 
           NA
  
 
  
 1
 
 214360574
 
 214360753
 
  ENSEMBL   UCSC 
 
  180
 
  4
 
 4197
 
 island
 
 0.018801
 
 0.2561589
 
 Not queried
 
  1.3972620
 
       NA
 
           NA
  
 
  
 6
 
  41215806
 
  41216090
 
  ENSEMBL   UCSC 
 
  285
 
  4
 
 34968
 
 tss
 
 0.302034
 
 0.4941598
 
 Not queried
 
  1.3969502
 
       NA
 
           NA
  
 
  
 1
 
 233121957
 
 233122135
 
  ENSEMBL   UCSC 
 
  179
 
  4
 
 1083
 
 gene;island
 
 0.832052
 
 0.9437888
 
 Not queried
 
  1.3969457
 
       NA
 
           NA
  
 
  
 20
 
  62318426
 
  62318588
 
  ENSEMBL   UCSC 
 
  163
 
  4
 
 25507
 
 gene;island
 
 0.341323
 
 0.3948958
 
 Not queried
 
  1.3967662
 
       NA
 
           NA
  
 
  
 1
 
  92495720
 
  92495877
 
  ENSEMBL   UCSC 
 
  158
 
  4
 
 632
 
 gene;island
 
 0.111304
 
 0.0534339
 
 Not queried
 
  1.3964649
 
       NA
 
           NA
  
 
  
 5
 
 177764003
 
 177764408
 
  ENSEMBL   UCSC 
 
  406
 
  4
 
 31927
 
 gene;island
 
 0.403971
 
 0.5841215
 
 Not queried
 
  1.3963463
 
       NA
 
           NA
  
 
  
 10
 
  12171245
 
  12171454
 
  ENSEMBL   UCSC 
 
  210
 
  4
 
 4950
 
 tss;island
 
 0.014182
 
 0.0261021
 
 Not queried
 
  1.3960544
 
       NA
 
           NA
  
 
  
 8
 
 114450065
 
 114450347
 
  ENSEMBL   UCSC 
 
  283
 
  4
 
 39877
 
 tss
 
 0.020553
 
 0.0117411
 
 Not queried
 
  1.3958657
 
       NA
 
           NA
  
 
  
 5
 
  89705234
 
  89705579
 
  ENSEMBL   UCSC 
 
  346
 
  4
 
 31684
 
 gene
 
 0.008264
 
 0.0041409
 
 Not queried
 
  1.3957857
 
       NA
 
           NA
  
 
  
 19
 
  44302666
 
  44303058
 
  ENSEMBL   UCSC 
 
  393
 
  4
 
 20079
 
 gene;island
 
 0.122839
 
 0.2554745
 
 Not queried
 
  1.3953466
 
       NA
 
           NA
  
 
  
 3
 
 140951194
 
 140951447
 
  ENSEMBL   UCSC 
 
  254
 
  4
 
 27972
 
 gene;island
 
 0.035978
 
 0.0172839
 
 Not queried
 
  1.3950510
 
       NA
 
           NA
  
 
  
 1
 
 235813272
 
 235813452
 
  ENSEMBL   UCSC 
 
  181
 
  4
 
 1093
 
 gene;island
 
 0.010045
 
 0.0092439
 
 Not queried
 
  1.3942621
 
       NA
 
           NA
  
 
  
 13
 
  25320388
 
  25320824
 
  ENSEMBL   UCSC 
 
  437
 
  4
 
 11605
 
 island
 
 0.122126
 
 0.3274360
 
 Not queried
 
  1.3940257
 
       NA
 
           NA
  
 
  
 6
 
  32920962
 
  32921233
 
  ENSEMBL   UCSC 
 
  272
 
  5
 
 34882
 
 tss
 
 0.004352
 
 0.0023819
 
 Not queried
 
  1.3935177
 
       NA
 
           NA
  
 
  
 6
 
  25278956
 
  25279165
 
  ENSEMBL   UCSC 
 
  210
 
  6
 
 34673
 
 tss;island
 
 0.017442
 
 0.0957632
 
 Not queried
 
  1.3927519
 
       NA
 
           NA
  
 
  
 2
 
 129076335
 
 129076394
 
  ENSEMBL   UCSC 
 
   60
 
  5
 
 23623
 
 tss;island
 
 0.030841
 
 0.0769298
 
 Not queried
 
  1.3926570
 
       NA
 
           NA
  
 
  
 6
 
  32184829
 
  32185340
 
  ENSEMBL   UCSC 
 
  512
 
  9
 
 34096
 
 gene
 
 0.337480
 
 0.6240903
 
 Not queried
 
  1.3925110
 
       NA
 
           NA
  
 
  
 19
 
   7746796
 
   7747220
 
  ENSEMBL   UCSC 
 
  425
 
  4
 
 19785
 
 gene;island
 
 0.552715
 
 0.4600854
 
 Not queried
 
  1.3923930
 
       NA
 
           NA
  
 
  
 7
 
  20816184
 
  20816695
 
  ENSEMBL   UCSC 
 
  512
 
  5
 
 38290
 
 island
 
 0.118490
 
 0.0249515
 
 Not queried
 
  1.3923535
 
       NA
 
           NA
  
 
  
 17
 
  73629082
 
  73629463
 
  ENSEMBL   UCSC 
 
  382
 
  6
 
 16772
 
 gene;tss;island
 
 0.236573
 
 0.5817189
 
 Not queried
 
  1.3920871
 
       NA
 
           NA
  
 
  
 19
 
  15292440
 
  15292775
 
  ENSEMBL   UCSC 
 
  336
 
  5
 
 19902
 
 gene;island
 
 0.640428
 
 0.7440262
 
 Not queried
 
  1.3920750
 
       NA
 
           NA
  
 
  
 14
 
 103367306
 
 103367632
 
  ENSEMBL   UCSC 
 
  327
 
  4
 
 12234
 
 gene
 
 0.203039
 
 0.1354920
 
 Not queried
 
  1.3917470
 
       NA
 
           NA
  
 
  
 12
 
  54763081
 
  54763433
 
  ENSEMBL   UCSC 
 
  353
 
  4
 
 9165
 
 gene;island
 
 0.915470
 
 0.6884335
 
 Not queried
 
  1.3913253
 
       NA
 
           NA
  
 
  
 12
 
  54366760
 
  54366875
 
  ENSEMBL   UCSC 
 
  116
 
  4
 
 9749
 
 tss
 
 0.074043
 
 0.2774927
 
 Not queried
 
  1.3913111
 
       NA
 
           NA
  
 
  
 12
 
   1973208
 
   1973452
 
  ENSEMBL   UCSC 
 
  245
 
  5
 
 9001
 
 gene;island
 
 0.707698
 
 0.9067408
 
 Not queried
 
  1.3911440
 
       NA
 
           NA
  
 
  
 1
 
    901685
 
    901799
 
  ENSEMBL   UCSC 
 
  115
 
  4
 
 1135
 
 tss
 
 0.015798
 
 0.0233322
 
 Not queried
 
  1.3910051
 
       NA
 
           NA
  
 
  
 7
 
 150778201
 
 150778340
 
  ENSEMBL   UCSC 
 
  140
 
  5
 
 37286
 
 gene
 
 0.068990
 
 0.2354054
 
 Not queried
 
  1.3909318
 
       NA
 
           NA
  
 
  
 4
 
  81256552
 
  81257269
 
  ENSEMBL   UCSC 
 
  718
 
 12
 
 31137
 
 island
 
 0.011193
 
 0.0053372
 
 Not queried
 
  1.3908439
 
       NA
 
           NA
  
 
  
 11
 
  67176914
 
  67177296
 
  ENSEMBL   UCSC 
 
  383
 
  6
 
 6752
 
 gene;island
 
 0.041394
 
 0.1235351
 
 Not queried
 
  1.3904812
 
       NA
 
           NA
  
 
  
 14
 
 105119383
 
 105119774
 
  ENSEMBL   UCSC 
 
  392
 
  4
 
 13211
 
 island
 
 0.934253
 
 0.9787227
 
 Not queried
 
  1.3903703
 
       NA
 
           NA
  
 
  
 4
 
    646293
 
    646577
 
  ENSEMBL   UCSC 
 
  285
 
  5
 
 29829
 
 gene;tss;island
 
 0.665929
 
 0.9020073
 
 Not queried
 
  1.3903360
 
       NA
 
           NA
  
 
  
 2
 
  85640762
 
  85641254
 
  ENSEMBL   UCSC 
 
  493
 
  6
 
 24478
 
 island
 
 0.118322
 
 0.0167642
 
 Not queried
 
  1.3900442
 
       NA
 
           NA
  
 
  
 19
 
  14228565
 
  14228823
 
  ENSEMBL   UCSC 
 
  259
 
  7
 
 20539
 
 tss
 
 0.006998
 
 0.0056713
 
 Not queried
 
  1.3894370
 
       NA
 
           NA
  
 
  
 1
 
  65532705
 
  65532761
 
  ENSEMBL   UCSC 
 
   57
 
  4
 
 3351
 
 island
 
 0.005402
 
 0.0011396
 
 Not queried
 
  1.3890801
 
       NA
 
           NA
  
 
  
 7
 
   4169306
 
   4169752
 
  ENSEMBL   UCSC 
 
  447
 
  6
 
 36829
 
 gene;island
 
 0.681540
 
 0.8509138
 
 Not queried
 
  1.3890331
 
       NA
 
           NA
  
 
  
 2
 
 109065127
 
 109066028
 
  ENSEMBL   UCSC 
 
  902
 
 10
 
 24600
 
 island
 
 0.007842
 
 0.0113564
 
 Not queried
 
  1.3887577
 
       NA
 
           NA
  
 
  
 17
 
  17740275
 
  17740468
 
  ENSEMBL   UCSC 
 
  194
 
  6
 
 18073
 
 island
 
 0.009612
 
 0.0040918
 
 Not queried
 
  1.3886421
 
       NA
 
           NA
  
 
  
 2
 
  18742004
 
  18742235
 
  ENSEMBL   UCSC 
 
  232
 
  5
 
 23204
 
 tss
 
 0.015749
 
 0.0116497
 
 Not queried
 
  1.3884261
 
       NA
 
           NA
  
 
  
 11
 
  65308245
 
  65308645
 
  ENSEMBL   UCSC 
 
  401
 
  4
 
 6709
 
 gene;island
 
 0.005241
 
 0.0048228
 
 Not queried
 
  1.3884179
 
       NA
 
           NA
  
 
  
 10
 
 131989161
 
 131989849
 
  ENSEMBL   UCSC 
 
  689
 
  8
 
 6274
 
 island
 
 0.771146
 
 0.9622618
 
 Not queried
 
  1.3879688
 
       NA
 
           NA
  
 
  
 2
 
  63277672
 
  63278211
 
  ENSEMBL   UCSC 
 
  540
 
 15
 
 24338
 
 island
 
 0.005477
 
 0.0026662
 
 Not queried
 
  1.3877157
 
       NA
 
           NA
  
 
  
 10
 
  99530944
 
  99531416
 
  ENSEMBL   UCSC 
 
  473
 
  5
 
 4700
 
 gene;island
 
 0.052851
 
 0.0193281
 
 Not queried
 
  1.3876523
 
       NA
 
           NA
  
 
  
 6
 
  49755009
 
  49755190
 
  ENSEMBL   UCSC 
 
  182
 
  4
 
 35028
 
 tss
 
 0.167648
 
 0.3087020
 
 Not queried
 
  1.3876188
 
       NA
 
           NA
  
 
  
 7
 
  99680278
 
  99680324
 
  ENSEMBL   UCSC 
 
   47
 
  5
 
 37766
 
 tss;island
 
 0.006843
 
 0.0073945
 
 Not queried
 
  1.3872874
 
       NA
 
           NA
  
 
  
 4
 
  91048416
 
  91048584
 
  ENSEMBL   UCSC 
 
  169
 
  6
 
 30498
 
 tss;island
 
 0.011595
 
 0.0047785
 
 Not queried
 
  1.3872507
 
       NA
 
           NA
  
 
  
 7
 
   5527927
 
   5528266
 
  ENSEMBL   UCSC 
 
  340
 
  4
 
 36879
 
 gene;island
 
 0.868038
 
 0.7704495
 
 Not queried
 
  1.3868525
 
       NA
 
           NA
  
 
  
 3
 
  50243021
 
  50243323
 
  ENSEMBL   UCSC 
 
  303
 
  4
 
 27795
 
 gene;island
 
 0.080572
 
 0.0257041
 
 Not queried
 
  1.3868307
 
       NA
 
           NA
  
 
  
 11
 
  27743258
 
  27743664
 
  ENSEMBL   UCSC 
 
  407
 
  8
 
 7215
 
 tss;island
 
 0.030148
 
 0.0469720
 
 Not queried
 
  1.3867320
 
       NA
 
           NA
  
 
  
 1
 
 150337032
 
 150337482
 
  ENSEMBL   UCSC 
 
  451
 
  6
 
 739
 
 gene
 
 0.010676
 
 0.0025630
 
 Not queried
 
  1.3865494
 
       NA
 
           NA
  
 
  
 3
 
  62355884
 
  62356323
 
  ENSEMBL   UCSC 
 
  440
 
  4
 
 27845
 
 gene;island
 
 0.042549
 
 0.0427732
 
 Not queried
 
  1.3857937
 
       NA
 
           NA
  
 
  
 22
 
  50492970
 
  50493212
 
  ENSEMBL   UCSC 
 
  243
 
  5
 
 27611
 
 island
 
 0.567370
 
 0.9419769
 
 Not queried
 
  1.3856437
 
       NA
 
           NA
  
 
  
 22
 
  22901830
 
  22902237
 
  ENSEMBL   UCSC 
 
  408
 
  6
 
 26808
 
 gene
 
 0.247904
 
 0.5822840
 
 Not queried
 
  1.3855726
 
       NA
 
           NA
  
 
  
 21
 
  46077454
 
  46077731
 
  ENSEMBL   UCSC 
 
  278
 
  6
 
 26459
 
 gene;tss
 
 0.687959
 
 0.6036963
 
 Not queried
 
  1.3854889
 
       NA
 
           NA
  
 
  
 2
 
  38978557
 
  38978896
 
  ENSEMBL   UCSC 
 
  340
 
  7
 
 24233
 
 island
 
 0.017403
 
 0.0148079
 
 Not queried
 
  1.3853981
 
       NA
 
           NA
  
 
  
 11
 
  34937341
 
  34937665
 
  ENSEMBL   UCSC 
 
  325
 
  7
 
 7243
 
 tss
 
 0.011841
 
 0.0059266
 
 Not queried
 
  1.3850805
 
       NA
 
           NA
  
 
  
 10
 
  23463015
 
  23463377
 
  ENSEMBL   UCSC 
 
  363
 
  4
 
 5608
 
 island
 
 0.177793
 
 0.3518164
 
 Not queried
 
  1.3849751
 
       NA
 
           NA
  
 
  
 14
 
  94423801
 
  94423950
 
  ENSEMBL   UCSC 
 
  150
 
  4
 
 12579
 
 tss
 
 0.704156
 
 0.4793747
 
 Not queried
 
  1.3847751
 
       NA
 
           NA
  
 
  
 7
 
  73118452
 
  73118748
 
  ENSEMBL   UCSC 
 
  297
 
  4
 
 37041
 
 gene;island
 
 0.373031
 
 0.1135253
 
 Not queried
 
  1.3846607
 
       NA
 
           NA
  
 
  
 20
 
  49547693
 
  49547986
 
  ENSEMBL   UCSC 
 
  294
 
  4
 
 25822
 
 tss;island
 
 0.020627
 
 0.0138321
 
 Not queried
 
  1.3836515
 
       NA
 
           NA
  
 
  
 12
 
 125028166
 
 125028339
 
  ENSEMBL   UCSC 
 
  174
 
  4
 
 10975
 
 island
 
 0.779241
 
 0.5880199
 
 Not queried
 
  1.3834897
 
       NA
 
           NA
  
 
  
 17
 
  76227227
 
  76227386
 
  ENSEMBL   UCSC 
 
  160
 
  4
 
 17702
 
 tss
 
 0.434363
 
 0.5273876
 
 Not queried
 
  1.3834077
 
       NA
 
           NA
  
 
  
 2
 
 241389021
 
 241389258
 
  ENSEMBL   UCSC 
 
  238
 
  4
 
 23120
 
 gene;island
 
 0.571195
 
 0.7533551
 
 Not queried
 
  1.3828257
 
       NA
 
           NA
  
 
  
 2
 
  48132877
 
  48133053
 
  ENSEMBL   UCSC 
 
  177
 
  4
 
 23345
 
 tss
 
 0.002822
 
 0.0051179
 
 Not queried
 
  1.3828112
 
       NA
 
           NA
  
 
  
 11
 
 108422663
 
 108423325
 
  ENSEMBL   UCSC 
 
  663
 
  8
 
 6890
 
 gene
 
 0.909808
 
 0.7437618
 
 Not queried
 
  1.3826356
 
       NA
 
           NA
  
 
  
 11
 
 108422936
 
 108423325
 
  ENSEMBL   UCSC 
 
  390
 
  6
 
 7674
 
 tss
 
 0.879074
 
 0.7437618
 
 Not queried
 
  1.3826356
 
       NA
 
           NA
  
 
  
 11
 
 133800146
 
 133800473
 
  ENSEMBL   UCSC 
 
  328
 
  4
 
 6985
 
 gene;island
 
 0.479107
 
 0.6452920
 
 Not queried
 
  1.3826016
 
       NA
 
           NA
  
 
  
 17
 
   7486551
 
   7487593
 
  ENSEMBL   UCSC 
 
 1043
 
 14
 
 17959
 
 island
 
 0.019458
 
 0.2212571
 
 Not queried
 
  1.3825655
 
       NA
 
           NA
  
 
  
 14
 
  97499908
 
  97500345
 
  ENSEMBL   UCSC 
 
  438
 
  4
 
 13122
 
 island
 
 0.097701
 
 0.1256151
 
 Not queried
 
  1.3825234
 
       NA
 
           NA
  
 
  
 14
 
 101488345
 
 101488488
 
  ENSEMBL   UCSC 
 
  144
 
  4
 
 12618
 
 tss
 
 0.115125
 
 0.2535612
 
 Not queried
 
  1.3822409
 
       NA
 
           NA
  
 
  
 14
 
  24540174
 
  24540452
 
  ENSEMBL   UCSC 
 
  279
 
  4
 
 12310
 
 tss
 
 0.393186
 
 0.5551418
 
 Not queried
 
  1.3819044
 
       NA
 
           NA
  
 
  
 3
 
  46607965
 
  46608179
 
  ENSEMBL   UCSC 
 
  215
 
  6
 
 27750
 
 gene;island
 
 0.059314
 
 0.3448456
 
 Not queried
 
  1.3818563
 
       NA
 
           NA
  
 
  
 2
 
 164204628
 
 164205343
 
  ENSEMBL   UCSC 
 
  716
 
  7
 
 24827
 
 island
 
 0.512010
 
 0.9186135
 
 Not queried
 
  1.3815671
 
       NA
 
           NA
  
 
  
 1
 
 113216774
 
 113217181
 
  ENSEMBL   UCSC 
 
  408
 
  6
 
 3606
 
 island
 
 0.020113
 
 0.0099582
 
 Not queried
 
  1.3812644
 
       NA
 
           NA
  
 
  
 15
 
  83240550
 
  83240791
 
  ENSEMBL   UCSC 
 
  242
 
  7
 
 13453
 
 gene
 
 0.036511
 
 0.1931845
 
 Not queried
 
  1.3808206
 
       NA
 
           NA
  
 
  
 20
 
  60119508
 
  60119664
 
  ENSEMBL   UCSC 
 
  157
 
  4
 
 25481
 
 gene;island
 
 0.525776
 
 0.6285610
 
 Not queried
 
  1.3808044
 
       NA
 
           NA
  
 
  
 8
 
   1906312
 
   1906498
 
  ENSEMBL   UCSC 
 
  187
 
  4
 
 39186
 
 gene
 
 0.643202
 
 0.8093136
 
 Not queried
 
  1.3805748
 
       NA
 
           NA
  
 
  
 19
 
  59086971
 
  59087367
 
  ENSEMBL   UCSC 
 
  397
 
  4
 
 20268
 
 gene;island
 
 0.012042
 
 0.0043334
 
 Not queried
 
  1.3805472
 
       NA
 
           NA
  
 
  
 19
 
  57630034
 
  57630742
 
  ENSEMBL   UCSC 
 
  709
 
 16
 
 21104
 
 tss;island
 
 0.636751
 
 0.8981408
 
 Not queried
 
  1.3803746
 
       NA
 
           NA
  
 
  
 10
 
  99790593
 
  99790910
 
  ENSEMBL   UCSC 
 
  318
 
  4
 
 5275
 
 tss
 
 0.242965
 
 0.0940437
 
 Not queried
 
  1.3797888
 
       NA
 
           NA
  
 
  
 15
 
  32933661
 
  32933835
 
  ENSEMBL   UCSC 
 
  175
 
  4
 
 13553
 
 tss
 
 0.915138
 
 0.7177575
 
 Not queried
 
  1.3794757
 
       NA
 
           NA
  
 
  
 5
 
  81047184
 
  81047249
 
  ENSEMBL   UCSC 
 
   66
 
  4
 
 32155
 
 tss;island
 
 0.037921
 
 0.0214742
 
 Not queried
 
  1.3794524
 
       NA
 
           NA
  
 
  
 1
 
  16369395
 
  16369631
 
  ENSEMBL   UCSC 
 
  237
 
  4
 
 1280
 
 tss
 
 0.175386
 
 0.3505622
 
 Not queried
 
  1.3792883
 
       NA
 
           NA
  
 
  
 17
 
  72969277
 
  72969503
 
  ENSEMBL   UCSC 
 
  227
 
  4
 
 17639
 
 tss;island
 
 0.317371
 
 0.4891227
 
 Not queried
 
  1.3792360
 
       NA
 
           NA
  
 
  
 18
 
  46064816
 
  46065292
 
  ENSEMBL   UCSC 
 
  477
 
  6
 
 19266
 
 tss;island
 
 0.057841
 
 0.1509066
 
 Not queried
 
  1.3791893
 
       NA
 
           NA
  
 
  
 5
 
 171766423
 
 171766855
 
  ENSEMBL   UCSC 
 
  433
 
  5
 
 31893
 
 gene;island
 
 0.513024
 
 0.5217595
 
 Not queried
 
  1.3791562
 
       NA
 
           NA
  
 
  
 1
 
   3077798
 
   3078206
 
  ENSEMBL   UCSC 
 
  409
 
  4
 
 105
 
 gene;island
 
 0.042602
 
 0.1110865
 
 Not queried
 
  1.3789856
 
       NA
 
           NA
  
 
  
 3
 
  13691967
 
  13692212
 
  ENSEMBL   UCSC 
 
  246
 
  7
 
 28166
 
 tss
 
 0.013820
 
 0.0162093
 
 Not queried
 
  1.3789651
 
       NA
 
           NA
  
 
  
 10
 
  15130785
 
  15131036
 
  ENSEMBL   UCSC 
 
  252
 
  6
 
 4961
 
 tss
 
 0.026500
 
 0.0165523
 
 Not queried
 
  1.3780525
 
       NA
 
           NA
  
 
  
 10
 
  15130387
 
  15131036
 
  ENSEMBL   UCSC 
 
  650
 
 10
 
 5567
 
 island
 
 0.040069
 
 0.0221216
 
 Not queried
 
  1.3780525
 
       NA
 
           NA
  
 
  
 13
 
 112547341
 
 112547653
 
  ENSEMBL   UCSC 
 
  313
 
  4
 
 11903
 
 island
 
 0.082634
 
 0.0191541
 
 Not queried
 
  1.3779230
 
       NA
 
           NA
  
 
  
 7
 
   2563478
 
   2563909
 
  ENSEMBL   UCSC 
 
  432
 
  5
 
 36804
 
 gene;island
 
 0.936930
 
 0.5249680
 
 Not queried
 
  1.3778294
 
       NA
 
           NA
  
 
  
 1
 
  46859671
 
  46859845
 
  ENSEMBL   UCSC 
 
  175
 
  6
 
 1600
 
 tss;island
 
 0.410316
 
 0.7805564
 
 Not queried
 
  1.3777272
 
       NA
 
           NA
  
 
  
 15
 
  78914083
 
  78914439
 
  ENSEMBL   UCSC 
 
  357
 
  4
 
 13837
 
 tss;island
 
 0.832235
 
 0.9161846
 
 Not queried
 
  1.3776752
 
       NA
 
           NA
  
 
  
 11
 
  67417958
 
  67418113
 
  ENSEMBL   UCSC 
 
  156
 
  4
 
 6762
 
 gene
 
 0.007361
 
 0.0172282
 
 Not queried
 
  1.3771459
 
       NA
 
           NA
  
 
  
 17
 
  78032288
 
  78032540
 
  ENSEMBL   UCSC 
 
  253
 
  5
 
 16853
 
 gene;island
 
 0.484617
 
 0.6761081
 
 Not queried
 
  1.3767347
 
       NA
 
           NA
  
 
  
 2
 
 132152814
 
 132152960
 
  ENSEMBL   UCSC 
 
  147
 
  4
 
 24731
 
 island
 
 0.645537
 
 0.7681287
 
 Not queried
 
  1.3766350
 
       NA
 
           NA
  
 
  
 5
 
  33936171
 
  33936462
 
  ENSEMBL   UCSC 
 
  292
 
 10
 
 32010
 
 tss
 
 0.202560
 
 0.1099474
 
 Not queried
 
  1.3764432
 
       NA
 
           NA
  
 
  
 3
 
 126113327
 
 126113743
 
  ENSEMBL   UCSC 
 
  417
 
 10
 
 28558
 
 tss
 
 0.061117
 
 0.2878711
 
 Not queried
 
  1.3758403
 
       NA
 
           NA
  
 
  
 6
 
  33147086
 
  33147259
 
  ENSEMBL   UCSC 
 
  174
 
  4
 
 34207
 
 gene
 
 0.430884
 
 0.6212835
 
 Not queried
 
  1.3756978
 
       NA
 
           NA
  
 
  
 19
 
  58220657
 
  58220837
 
  ENSEMBL   UCSC 
 
  181
 
  6
 
 21124
 
 tss
 
 0.046430
 
 0.0256806
 
 Not queried
 
  1.3755078
 
       NA
 
           NA
  
 
  
 8
 
 121824218
 
 121824929
 
  ENSEMBL   UCSC 
 
  712
 
 10
 
 40565
 
 island
 
 0.005238
 
 0.0082861
 
 Not queried
 
  1.3753928
 
       NA
 
           NA
  
 
  
 5
 
   2754753
 
   2755158
 
  ENSEMBL   UCSC 
 
  406
 
  4
 
 31551
 
 gene;island
 
 0.015159
 
 0.0310639
 
 Not queried
 
  1.3753710
 
       NA
 
           NA
  
 
  
 19
 
   1383340
 
   1383493
 
  ENSEMBL   UCSC 
 
  154
 
  4
 
 20302
 
 tss;island
 
 0.009698
 
 0.0047494
 
 Not queried
 
  1.3752953
 
       NA
 
           NA
  
 
  
 1
 
 165599451
 
 165599927
 
  ENSEMBL   UCSC 
 
  477
 
  5
 
 2067
 
 tss;island
 
 0.010902
 
 0.0036331
 
 Not queried
 
  1.3738727
 
       NA
 
           NA
  
 
  
 6
 
 167032331
 
 167032502
 
  ENSEMBL   UCSC 
 
  172
 
  4
 
 34550
 
 gene;island
 
 0.610534
 
 0.8172791
 
 Not queried
 
  1.3738409
 
       NA
 
           NA
  
 
  
 6
 
  30920763
 
  30921007
 
  ENSEMBL   UCSC 
 
  245
 
  7
 
 33843
 
 gene;island
 
 0.340519
 
 0.9140966
 
 Not queried
 
  1.3737644
 
       NA
 
           NA
  
 
  
 1
 
  84972303
 
  84972490
 
  ENSEMBL   UCSC 
 
  188
 
  5
 
 602
 
 gene;tss;island
 
 0.020953
 
 0.0073166
 
 Not queried
 
  1.3737411
 
       NA
 
           NA
  
 
  
 15
 
  90039409
 
  90039794
 
  ENSEMBL   UCSC 
 
  386
 
  4
 
 13470
 
 gene
 
 0.093027
 
 0.0317495
 
 Not queried
 
  1.3728644
 
       NA
 
           NA
  
 
  
 19
 
  15236599
 
  15236813
 
  ENSEMBL   UCSC 
 
  215
 
  6
 
 20555
 
 tss
 
 0.009680
 
 0.0020117
 
 Not queried
 
  1.3726722
 
       NA
 
           NA
  
 
  
 12
 
 105379893
 
 105379979
 
  ENSEMBL   UCSC 
 
   87
 
  4
 
 9961
 
 tss
 
 0.005425
 
 0.0019942
 
 Not queried
 
  1.3725375
 
       NA
 
           NA
  
 
  
 4
 
  41258790
 
  41259044
 
  ENSEMBL   UCSC 
 
  255
 
  5
 
 31001
 
 island
 
 0.084670
 
 0.0321474
 
 Not queried
 
  1.3721516
 
       NA
 
           NA
  
 
  
 7
 
 157849615
 
 157849794
 
  ENSEMBL   UCSC 
 
  180
 
  5
 
 37358
 
 gene
 
 0.512811
 
 0.8208537
 
 Not queried
 
  1.3718041
 
       NA
 
           NA
  
 
  
 12
 
  57472580
 
  57472858
 
  ENSEMBL   UCSC 
 
  279
 
  9
 
 9807
 
 tss
 
 0.007052
 
 0.0020361
 
 Not queried
 
  1.3717471
 
       NA
 
           NA
  
 
  
 1
 
   3207371
 
   3207588
 
  ENSEMBL   UCSC 
 
  218
 
  5
 
 117
 
 gene;island
 
 0.555154
 
 0.7628960
 
 Not queried
 
  1.3717123
 
       NA
 
           NA
  
 
  
 2
 
   1497815
 
   1498138
 
  ENSEMBL   UCSC 
 
  324
 
  5
 
 22531
 
 gene;island
 
 0.198209
 
 0.4423383
 
 Not queried
 
  1.3716819
 
       NA
 
           NA
  
 
  
 7
 
  27196296
 
  27196555
 
  ENSEMBL   UCSC 
 
  260
 
  7
 
 37494
 
 tss
 
 0.039803
 
 0.0170646
 
 Not queried
 
  1.3714472
 
       NA
 
           NA
  
 
  
 3
 
  39851641
 
  39851931
 
  ENSEMBL   UCSC 
 
  291
 
  4
 
 27726
 
 gene;island
 
 0.026083
 
 0.0666112
 
 Not queried
 
  1.3713595
 
       NA
 
           NA
  
 
  
 6
 
  99272871
 
  99273830
 
  ENSEMBL   UCSC 
 
  960
 
 12
 
 36340
 
 island
 
 0.050162
 
 0.0265621
 
 Not queried
 
  1.3710664
 
       NA
 
           NA
  
 
  
 5
 
 148758847
 
 148759090
 
  ENSEMBL   UCSC 
 
  244
 
  6
 
 32417
 
 tss
 
 0.381475
 
 0.5524746
 
 Not queried
 
  1.3710451
 
       NA
 
           NA
  
 
  
 13
 
  96328811
 
  96328951
 
  ENSEMBL   UCSC 
 
  141
 
  4
 
 11504
 
 tss;island
 
 0.003419
 
 0.0024938
 
 Not queried
 
  1.3704760
 
       NA
 
           NA
  
 
  
 8
 
  25314954
 
  25315063
 
  ENSEMBL   UCSC 
 
  110
 
  4
 
 39249
 
 gene;island
 
 0.017828
 
 0.0121988
 
 Not queried
 
  1.3703560
 
       NA
 
           NA
  
 
  
 1
 
 220267790
 
 220268095
 
  ENSEMBL   UCSC 
 
  306
 
  4
 
 1017
 
 gene;island
 
 0.118004
 
 0.0436520
 
 Not queried
 
  1.3697654
 
       NA
 
           NA
  
 
  
 19
 
  45737483
 
  45737880
 
  ENSEMBL   UCSC 
 
  398
 
  8
 
 20864
 
 tss;island
 
 0.960351
 
 0.9789713
 
 Not queried
 
  1.3693533
 
       NA
 
           NA
  
 
  
 10
 
 133449506
 
 133449715
 
  ENSEMBL   UCSC 
 
  210
 
  4
 
 6277
 
 island
 
 0.630474
 
 0.8657621
 
 Not queried
 
  1.3690809
 
       NA
 
           NA
  
 
  
 5
 
  38445522
 
  38445916
 
  ENSEMBL   UCSC 
 
  395
 
  7
 
 31595
 
 gene
 
 0.722733
 
 0.8568942
 
 Not queried
 
  1.3690182
 
       NA
 
           NA
  
 
  
 11
 
 129992297
 
 129992368
 
  ENSEMBL   UCSC 
 
   72
 
  4
 
 6973
 
 gene;island
 
 0.356601
 
 0.5608052
 
 Not queried
 
  1.3689670
 
       NA
 
           NA
  
 
  
 21
 
  39493025
 
  39493391
 
  ENSEMBL   UCSC 
 
  367
 
  5
 
 26425
 
 gene
 
 0.754305
 
 0.8659454
 
 Not queried
 
  1.3689223
 
       NA
 
           NA
  
 
  
 3
 
  63850039
 
  63850464
 
  ENSEMBL   UCSC 
 
  426
 
  6
 
 28431
 
 tss;island
 
 0.003616
 
 0.0057984
 
 Not queried
 
  1.3689187
 
       NA
 
           NA
  
 
  
 4
 
 186732837
 
 186733060
 
  ENSEMBL   UCSC 
 
  224
 
  7
 
 30212
 
 gene;tss
 
 0.002196
 
 0.0073786
 
 Not queried
 
  1.3682815
 
       NA
 
           NA
  
 
  
 19
 
  12917086
 
  12917312
 
  ENSEMBL   UCSC 
 
  227
 
  6
 
 20513
 
 tss
 
 0.021093
 
 0.0162370
 
 Not queried
 
  1.3679728
 
       NA
 
           NA
  
 
  
 2
 
  43453251
 
  43453558
 
  ENSEMBL   UCSC 
 
  308
 
  5
 
 22657
 
 gene;tss;island
 
 0.002619
 
 0.0043980
 
 Not queried
 
  1.3677477
 
       NA
 
           NA
  
 
  
 18
 
  55019621
 
  55019711
 
  ENSEMBL   UCSC 
 
   91
 
  4
 
 19289
 
 tss
 
 0.139168
 
 0.0530177
 
 Not queried
 
  1.3672615
 
       NA
 
           NA
  
 
  
 5
 
  37838290
 
  37838871
 
  ENSEMBL   UCSC 
 
  582
 
  6
 
 31592
 
 gene;island
 
 0.018479
 
 0.0205256
 
 Not queried
 
  1.3664600
 
       NA
 
           NA
  
 
  
 11
 
  60738971
 
  60739019
 
  ENSEMBL   UCSC 
 
   49
 
  4
 
 7327
 
 tss
 
 0.020460
 
 0.0077587
 
 Not queried
 
  1.3662541
 
       NA
 
           NA
  
 
  
 12
 
  49504641
 
  49505053
 
  ENSEMBL   UCSC 
 
  413
 
  7
 
 10386
 
 island
 
 0.016834
 
 0.0054509
 
 Not queried
 
  1.3661968
 
       NA
 
           NA
  
 
  
 12
 
  52701963
 
  52702318
 
  ENSEMBL   UCSC 
 
  356
 
  4
 
 9137
 
 gene;island
 
 0.005492
 
 0.0257000
 
 Not queried
 
  1.3657022
 
       NA
 
           NA
  
 
  
 14
 
  53620162
 
  53620401
 
  ENSEMBL   UCSC 
 
  240
 
  5
 
 12403
 
 tss;island
 
 0.007226
 
 0.0048582
 
 Not queried
 
  1.3653939
 
       NA
 
           NA
  
 
  
 7
 
 150644459
 
 150644866
 
  ENSEMBL   UCSC 
 
  408
 
  5
 
 37278
 
 gene;island
 
 0.366053
 
 0.4167434
 
 Not queried
 
  1.3650826
 
       NA
 
           NA
  
 
  
 10
 
 131671373
 
 131671830
 
  ENSEMBL   UCSC 
 
  458
 
  6
 
 4832
 
 gene
 
 0.414809
 
 0.5097571
 
 Not queried
 
  1.3650799
 
       NA
 
           NA
  
 
  
 6
 
  32038747
 
  32038958
 
  ENSEMBL   UCSC 
 
  212
 
  4
 
 34030
 
 gene
 
 0.293667
 
 0.4017884
 
 Not queried
 
  1.3648785
 
       NA
 
           NA
  
 
  
 11
 
  76858648
 
  76859056
 
  ENSEMBL   UCSC 
 
  409
 
  6
 
 6839
 
 gene;island
 
 0.095640
 
 0.0144547
 
 Not queried
 
  1.3646856
 
       NA
 
           NA
  
 
  
 6
 
  29572219
 
  29572379
 
  ENSEMBL   UCSC 
 
  161
 
  6
 
 33665
 
 gene
 
 0.485458
 
 0.6947907
 
 Not queried
 
  1.3646267
 
       NA
 
           NA
  
 
  
 5
 
 180622072
 
 180622643
 
  ENSEMBL   UCSC 
 
  572
 
  5
 
 31950
 
 gene;island
 
 0.930892
 
 0.9554787
 
 Not queried
 
  1.3641827
 
       NA
 
           NA
  
 
  
 2
 
 149634369
 
 149634726
 
  ENSEMBL   UCSC 
 
  358
 
  5
 
 22894
 
 gene;island
 
 0.048951
 
 0.0127437
 
 Not queried
 
  1.3641204
 
       NA
 
           NA
  
 
  
 6
 
 112041092
 
 112041490
 
  ENSEMBL   UCSC 
 
  399
 
  5
 
 34448
 
 gene
 
 0.869218
 
 0.8629066
 
 Not queried
 
  1.3639047
 
       NA
 
           NA
  
 
  
 1
 
   3191481
 
   3191883
 
  ENSEMBL   UCSC 
 
  403
 
  6
 
 116
 
 gene;island
 
 0.542558
 
 0.6783898
 
 Not queried
 
  1.3637970
 
       NA
 
           NA
  
 
  
 1
 
 167189429
 
 167189846
 
  ENSEMBL   UCSC 
 
  418
 
  5
 
 2074
 
 tss;island
 
 0.004636
 
 0.0022513
 
 Not queried
 
  1.3637712
 
       NA
 
           NA
  
 
  
 4
 
 126237325
 
 126237513
 
  ENSEMBL   UCSC 
 
  189
 
  6
 
 30585
 
 tss
 
 0.014710
 
 0.0041833
 
 Not queried
 
  1.3636275
 
       NA
 
           NA
  
 
  
 22
 
  30868060
 
  30868226
 
  ENSEMBL   UCSC 
 
  167
 
  4
 
 27063
 
 tss
 
 0.190499
 
 0.3858719
 
 Not queried
 
  1.3632667
 
       NA
 
           NA
  
 
  
 11
 
   2481857
 
   2482929
 
  ENSEMBL   UCSC 
 
 1073
 
 17
 
 6460
 
 gene
 
 0.257851
 
 0.4190148
 
 Not queried
 
  1.3625190
 
       NA
 
           NA
  
 
  
 19
 
  42440001
 
  42440198
 
  ENSEMBL   UCSC 
 
  198
 
  4
 
 22061
 
 island
 
 0.397205
 
 0.3501977
 
 Not queried
 
  1.3617383
 
       NA
 
           NA
  
 
  
 8
 
  20107455
 
  20107758
 
  ENSEMBL   UCSC 
 
  304
 
  4
 
 39222
 
 gene;island
 
 0.507665
 
 0.7643581
 
 Not queried
 
  1.3616622
 
       NA
 
           NA
  
 
  
 20
 
  30062970
 
  30063038
 
  ENSEMBL   UCSC 
 
   69
 
  6
 
 25659
 
 tss
 
 0.068107
 
 0.0342958
 
 Not queried
 
  1.3613476
 
       NA
 
           NA
  
 
  
 20
 
   5451661
 
   5451815
 
  ENSEMBL   UCSC 
 
  155
 
  6
 
 25582
 
 tss
 
 0.466422
 
 0.7135659
 
 Not queried
 
  1.3612676
 
       NA
 
           NA
  
 
  
 15
 
  83776269
 
  83776446
 
  ENSEMBL   UCSC 
 
  178
 
  5
 
 14394
 
 island
 
 0.006185
 
 0.0043800
 
 Not queried
 
  1.3610974
 
       NA
 
           NA
  
 
  
 11
 
 113143664
 
 113143807
 
  ENSEMBL   UCSC 
 
  144
 
  4
 
 6908
 
 gene
 
 0.789504
 
 0.3098162
 
 Not queried
 
  1.3609553
 
       NA
 
           NA
  
 
  
 14
 
  29243173
 
  29243762
 
  ENSEMBL   UCSC 
 
  590
 
  7
 
 12053
 
 gene;island
 
 0.173014
 
 0.0337095
 
 Not queried
 
  1.3607953
 
       NA
 
           NA
  
 
  
 19
 
   7911120
 
   7911551
 
  ENSEMBL   UCSC 
 
  432
 
  6
 
 19786
 
 gene;island
 
 0.416822
 
 0.6498551
 
 Not queried
 
  1.3602529
 
       NA
 
           NA
  
 
  
 2
 
 121494701
 
 121495233
 
  ENSEMBL   UCSC 
 
  533
 
  5
 
 24670
 
 island
 
 0.078351
 
 0.1038474
 
 Not queried
 
  1.3601573
 
       NA
 
           NA
  
 
  
 17
 
   8066184
 
   8066251
 
  ENSEMBL   UCSC 
 
   68
 
  4
 
 16427
 
 gene;island
 
 0.008994
 
 0.0121485
 
 Not queried
 
  1.3601338
 
       NA
 
           NA
  
 
  
 2
 
  75938118
 
  75938438
 
  ENSEMBL   UCSC 
 
  321
 
  9
 
 23471
 
 tss
 
 0.037594
 
 0.0215371
 
 Not queried
 
  1.3595896
 
       NA
 
           NA
  
 
  
 6
 
  99790960
 
  99791250
 
  ENSEMBL   UCSC 
 
  291
 
  4
 
 34422
 
 gene
 
 0.684874
 
 0.8429775
 
 Not queried
 
  1.3595183
 
       NA
 
           NA
  
 
  
 7
 
  79081666
 
  79081807
 
  ENSEMBL   UCSC 
 
  142
 
  4
 
 37065
 
 gene;island
 
 0.036523
 
 0.0663622
 
 Not queried
 
  1.3593580
 
       NA
 
           NA
  
 
  
 10
 
  21783344
 
  21783930
 
  ENSEMBL   UCSC 
 
  587
 
  6
 
 5589
 
 island
 
 0.006467
 
 0.0244678
 
 Not queried
 
  1.3593150
 
       NA
 
           NA
  
 
  
 6
 
  41472382
 
  41472657
 
  ENSEMBL   UCSC 
 
  276
 
  4
 
 36123
 
 island
 
 0.060628
 
 0.0169630
 
 Not queried
 
  1.3592232
 
       NA
 
           NA
  
 
  
 7
 
  73241729
 
  73242028
 
  ENSEMBL   UCSC 
 
  300
 
  4
 
 38570
 
 island
 
 0.022231
 
 0.0515051
 
 Not queried
 
  1.3591337
 
       NA
 
           NA
  
 
  
 5
 
 159343148
 
 159343549
 
  ENSEMBL   UCSC 
 
  402
 
  6
 
 32464
 
 tss;island
 
 0.219909
 
 0.0328903
 
 Not queried
 
  1.3590121
 
       NA
 
           NA
  
 
  
 15
 
  30917918
 
  30918065
 
  ENSEMBL   UCSC 
 
  148
 
  4
 
 13548
 
 tss;island
 
 0.001212
 
 0.0012944
 
 Not queried
 
  1.3583945
 
       NA
 
           NA
  
 
  
 16
 
  28270490
 
  28270754
 
  ENSEMBL   UCSC 
 
  265
 
  4
 
 15785
 
 island
 
 0.676212
 
 0.8401697
 
 Not queried
 
  1.3583857
 
       NA
 
           NA
  
 
  
 16
 
  10275934
 
  10276119
 
  ENSEMBL   UCSC 
 
  186
 
  4
 
 15086
 
 tss
 
 0.021906
 
 0.0165107
 
 Not queried
 
  1.3583145
 
       NA
 
           NA
  
 
  
 1
 
  24151609
 
  24151885
 
  ENSEMBL   UCSC 
 
  277
 
  4
 
 322
 
 gene
 
 0.002595
 
 0.0020353
 
 Not queried
 
  1.3581703
 
       NA
 
           NA
  
 
  
 17
 
  48911983
 
  48912556
 
  ENSEMBL   UCSC 
 
  574
 
 12
 
 17508
 
 tss
 
 0.535511
 
 0.8276068
 
 Not queried
 
  1.3577249
 
       NA
 
           NA
  
 
  
 4
 
 128801645
 
 128802013
 
  ENSEMBL   UCSC 
 
  369
 
  8
 
 30588
 
 tss
 
 0.009763
 
 0.0073417
 
 Not queried
 
  1.3570502
 
       NA
 
           NA
  
 
  
 11
 
  12398874
 
  12399176
 
  ENSEMBL   UCSC 
 
  303
 
  8
 
 8066
 
 island
 
 0.052236
 
 0.0910702
 
 Not queried
 
  1.3569357
 
       NA
 
           NA
  
 
  
 2
 
 239542239
 
 239542460
 
  ENSEMBL   UCSC 
 
  222
 
  4
 
 25247
 
 island
 
 0.804877
 
 0.8964828
 
 Not queried
 
  1.3568991
 
       NA
 
           NA
  
 
  
 1
 
 231297516
 
 231297737
 
  ENSEMBL   UCSC 
 
  222
 
  4
 
 2321
 
 tss;island
 
 0.064636
 
 0.0709418
 
 Not queried
 
  1.3565894
 
       NA
 
           NA
  
 
  
 15
 
  76196328
 
  76196799
 
  ENSEMBL   UCSC 
 
  472
 
  5
 
 13433
 
 gene
 
 0.006169
 
 0.0157116
 
 Not queried
 
  1.3563643
 
       NA
 
           NA
  
 
  
 5
 
 175792454
 
 175792498
 
  ENSEMBL   UCSC 
 
   45
 
  4
 
 32510
 
 tss
 
 0.007999
 
 0.0071322
 
 Not queried
 
  1.3561955
 
       NA
 
           NA
  
 
  
 20
 
   2633258
 
   2633362
 
  ENSEMBL   UCSC 
 
  105
 
  4
 
 25334
 
 gene
 
 0.018308
 
 0.0370414
 
 Not queried
 
  1.3560768
 
       NA
 
           NA
  
 
  
 18
 
  61144150
 
  61144339
 
  ENSEMBL   UCSC 
 
  190
 
  4
 
 19121
 
 gene
 
 0.608518
 
 0.7291336
 
 Not queried
 
  1.3559936
 
       NA
 
           NA
  
 
  
 16
 
   1221825
 
   1222188
 
  ENSEMBL   UCSC 
 
  364
 
  4
 
 14557
 
 gene;island
 
 0.402957
 
 0.6303474
 
 Not queried
 
  1.3558846
 
       NA
 
           NA
  
 
  
 14
 
  69341430
 
  69341905
 
  ENSEMBL   UCSC 
 
  476
 
  4
 
 12123
 
 gene;island
 
 0.965020
 
 0.9842334
 
 Not queried
 
  1.3556839
 
       NA
 
           NA
  
 
  
 16
 
   2986767
 
   2987035
 
  ENSEMBL   UCSC 
 
  269
 
  4
 
 14617
 
 gene
 
 0.649283
 
 0.8218985
 
 Not queried
 
  1.3556691
 
       NA
 
           NA
  
 
  
 5
 
 139283051
 
 139283351
 
  ENSEMBL   UCSC 
 
  301
 
  5
 
 31782
 
 gene;island
 
 0.020936
 
 0.0082514
 
 Not queried
 
  1.3556148
 
       NA
 
           NA
  
 
  
 1
 
 171810105
 
 171810570
 
  ENSEMBL   UCSC 
 
  466
 
  8
 
 2093
 
 tss;island
 
 0.020859
 
 0.0415014
 
 Not queried
 
  1.3556091
 
       NA
 
           NA
  
 
  
 6
 
  29555799
 
  29556415
 
  ENSEMBL   UCSC 
 
  617
 
  8
 
 33662
 
 gene
 
 0.040532
 
 0.2994025
 
 Not queried
 
  1.3552143
 
       NA
 
           NA
  
 
  
 11
 
  66488919
 
  66489022
 
  ENSEMBL   UCSC 
 
  104
 
  4
 
 7479
 
 tss
 
 0.873135
 
 0.9136642
 
 Not queried
 
  1.3551645
 
       NA
 
           NA
  
 
  
 1
 
 208132388
 
 208132787
 
  ENSEMBL   UCSC 
 
  400
 
  4
 
 4158
 
 island
 
 0.014599
 
 0.0278446
 
 Not queried
 
  1.3551477
 
       NA
 
           NA
  
 
  
 11
 
 121970496
 
 121970768
 
  ENSEMBL   UCSC 
 
  273
 
  6
 
 6946
 
 gene
 
 0.807136
 
 0.9397076
 
 Not queried
 
  1.3546650
 
       NA
 
           NA
  
 
  
 8
 
 135724771
 
 135725096
 
  ENSEMBL   UCSC 
 
  326
 
  4
 
 39434
 
 gene;island
 
 0.003626
 
 0.0017050
 
 Not queried
 
  1.3544101
 
       NA
 
           NA
  
 
  
 2
 
 121104029
 
 121104400
 
  ENSEMBL   UCSC 
 
  372
 
  4
 
 22849
 
 gene;island
 
 0.036322
 
 0.0204348
 
 Not queried
 
  1.3543941
 
       NA
 
           NA
  
 
  
 11
 
   1154976
 
   1155278
 
  ENSEMBL   UCSC 
 
  303
 
  4
 
 7904
 
 island
 
 0.700560
 
 0.8858065
 
 Not queried
 
  1.3542509
 
       NA
 
           NA
  
 
  
 13
 
  38923912
 
  38924138
 
  ENSEMBL   UCSC 
 
  227
 
  5
 
 11679
 
 island
 
 0.001605
 
 0.0014378
 
 Not queried
 
  1.3541547
 
       NA
 
           NA
  
 
  
 2
 
 131485262
 
 131485486
 
  ENSEMBL   UCSC 
 
  225
 
  4
 
 23628
 
 tss
 
 0.004253
 
 0.0026316
 
 Not queried
 
  1.3534099
 
       NA
 
           NA
  
 
  
 20
 
   3149249
 
   3149495
 
  ENSEMBL   UCSC 
 
  247
 
  6
 
 25560
 
 tss;island
 
 0.377197
 
 0.6535469
 
 Not queried
 
  1.3533158
 
       NA
 
           NA
  
 
  
 1
 
  92414221
 
  92414520
 
  ENSEMBL   UCSC 
 
  300
 
  4
 
 1744
 
 tss;island
 
 0.542503
 
 0.4961203
 
 Not queried
 
  1.3532223
 
       NA
 
           NA
  
 
  
 5
 
  38557085
 
  38557634
 
  ENSEMBL   UCSC 
 
  550
 
  7
 
 31596
 
 gene;tss;island
 
 0.028958
 
 0.0102908
 
 Not queried
 
  1.3530670
 
       NA
 
           NA
  
 
  
 4
 
  17782853
 
  17783502
 
  ENSEMBL   UCSC 
 
  650
 
 11
 
 30949
 
 island
 
 0.120210
 
 0.0388773
 
 Not queried
 
  1.3529777
 
       NA
 
           NA
  
 
  
 8
 
  41654331
 
  41654618
 
  ENSEMBL   UCSC 
 
  288
 
  5
 
 39293
 
 gene;island
 
 0.777349
 
 0.8605714
 
 Not queried
 
  1.3526578
 
       NA
 
           NA
  
 
  
 16
 
  82044738
 
  82045222
 
  ENSEMBL   UCSC 
 
  485
 
  9
 
 16127
 
 island
 
 0.042743
 
 0.0185918
 
 Not queried
 
  1.3526244
 
       NA
 
           NA
  
 
  
 17
 
  38573776
 
  38574080
 
  ENSEMBL   UCSC 
 
  305
 
  5
 
 16565
 
 gene
 
 0.001388
 
 0.0008623
 
 Not queried
 
  1.3521164
 
       NA
 
           NA
  
 
  
 22
 
  38201496
 
  38201848
 
  ENSEMBL   UCSC 
 
  353
 
  4
 
 26864
 
 gene;island
 
 0.065996
 
 0.1684329
 
 Not queried
 
  1.3519602
 
       NA
 
           NA
  
 
  
 6
 
  31439037
 
  31439148
 
  ENSEMBL   UCSC 
 
  112
 
  4
 
 33878
 
 gene
 
 0.789135
 
 0.9069217
 
 Not queried
 
  1.3519242
 
       NA
 
           NA
  
 
  
 12
 
   2986348
 
   2987138
 
  ENSEMBL   UCSC 
 
  791
 
  9
 
 9492
 
 tss;island
 
 0.004167
 
 0.0129890
 
 Not queried
 
  1.3513989
 
       NA
 
           NA
  
 
  
 16
 
  84853583
 
  84853871
 
  ENSEMBL   UCSC 
 
  289
 
  5
 
 16146
 
 island
 
 0.103136
 
 0.0445380
 
 Not queried
 
  1.3508461
 
       NA
 
           NA
  
 
  
 7
 
   1269693
 
   1270134
 
  ENSEMBL   UCSC 
 
  442
 
  4
 
 38090
 
 island
 
 0.092120
 
 0.1305531
 
 Not queried
 
  1.3497433
 
       NA
 
           NA
  
 
  
 3
 
  44753865
 
  44754131
 
  ENSEMBL   UCSC 
 
  267
 
  8
 
 28260
 
 tss
 
 0.274387
 
 0.4860162
 
 Not queried
 
  1.3496899
 
       NA
 
           NA
  
 
  
 1
 
 153508511
 
 153508713
 
  ENSEMBL   UCSC 
 
  203
 
  6
 
 760
 
 gene
 
 0.001399
 
 0.0037711
 
 Not queried
 
  1.3496229
 
       NA
 
           NA
  
 
  
 2
 
  86426669
 
  86426930
 
  ENSEMBL   UCSC 
 
  262
 
  4
 
 22768
 
 gene
 
 0.006127
 
 0.0045111
 
 Not queried
 
  1.3492169
 
       NA
 
           NA
  
 
  
 10
 
   1245261
 
   1245586
 
  ENSEMBL   UCSC 
 
  326
 
  4
 
 4458
 
 gene
 
 0.109389
 
 0.5949035
 
 Not queried
 
  1.3490768
 
       NA
 
           NA
  
 
  
 4
 
  21699209
 
  21699534
 
  ENSEMBL   UCSC 
 
  326
 
  7
 
 29966
 
 gene
 
 0.115163
 
 0.4706962
 
 Not queried
 
  1.3487025
 
       NA
 
           NA
  
 
  
 16
 
  31580889
 
  31581309
 
  ENSEMBL   UCSC 
 
  421
 
  6
 
 15215
 
 tss;island
 
 0.199647
 
 0.1152347
 
 Not queried
 
  1.3486629
 
       NA
 
           NA
  
 
  
 13
 
 109779684
 
 109779925
 
  ENSEMBL   UCSC 
 
  242
 
  4
 
 11225
 
 gene;island
 
 0.774853
 
 0.9035280
 
 Not queried
 
  1.3484540
 
       NA
 
           NA
  
 
  
 12
 
  14133129
 
  14133426
 
  ENSEMBL   UCSC 
 
  298
 
  6
 
 9582
 
 tss
 
 0.032581
 
 0.0214044
 
 Not queried
 
  1.3483775
 
       NA
 
           NA
  
 
  
 12
 
 117319785
 
 117320316
 
  ENSEMBL   UCSC 
 
  532
 
  7
 
 10041
 
 tss;island
 
 0.070310
 
 0.0704975
 
 Not queried
 
  1.3480319
 
       NA
 
           NA
  
 
  
 16
 
   2569911
 
   2569967
 
  ENSEMBL   UCSC 
 
   57
 
  4
 
 14606
 
 gene;tss;island
 
 0.767378
 
 0.9677118
 
 Not queried
 
  1.3477799
 
       NA
 
           NA
  
 
  
 16
 
  23313148
 
  23313559
 
  ENSEMBL   UCSC 
 
  412
 
  8
 
 15137
 
 tss;island
 
 0.041856
 
 0.0054002
 
 Not queried
 
  1.3477092
 
       NA
 
           NA
  
 
  
 1
 
 111218079
 
 111218287
 
  ENSEMBL   UCSC 
 
  209
 
  4
 
 1819
 
 tss;island
 
 0.482155
 
 0.4502351
 
 Not queried
 
  1.3474801
 
       NA
 
           NA
  
 
  
 13
 
  96204493
 
  96204978
 
  ENSEMBL   UCSC 
 
  486
 
 10
 
 11193
 
 gene;island
 
 0.021665
 
 0.0102426
 
 Not queried
 
  1.3472019
 
       NA
 
           NA
  
 
  
 1
 
  32253906
 
  32254320
 
  ENSEMBL   UCSC 
 
  415
 
  5
 
 2978
 
 island
 
 0.007211
 
 0.0025707
 
 Not queried
 
  1.3465756
 
       NA
 
           NA
  
 
  
 10
 
  81075012
 
  81075241
 
  ENSEMBL   UCSC 
 
  230
 
  4
 
 4649
 
 gene
 
 0.866637
 
 0.8836373
 
 Not queried
 
  1.3463506
 
       NA
 
           NA
  
 
  
 12
 
 133018735
 
 133019135
 
  ENSEMBL   UCSC 
 
  401
 
  4
 
 11056
 
 island
 
 0.550067
 
 0.7092178
 
 Not queried
 
  1.3463190
 
       NA
 
           NA
  
 
  
 19
 
   2250901
 
   2251067
 
  ENSEMBL   UCSC 
 
  167
 
  4
 
 19682
 
 gene;island
 
 0.165537
 
 0.2049758
 
 Not queried
 
  1.3461332
 
       NA
 
           NA
  
 
  
 11
 
  44324759
 
  44325029
 
  ENSEMBL   UCSC 
 
  271
 
  5
 
 6589
 
 gene;island
 
 0.089370
 
 0.3495578
 
 Not queried
 
  1.3459830
 
       NA
 
           NA
  
 
  
 17
 
  48133890
 
  48134226
 
  ENSEMBL   UCSC 
 
  337
 
  4
 
 16679
 
 gene;island
 
 0.005693
 
 0.0035232
 
 Not queried
 
  1.3458167
 
       NA
 
           NA
  
 
  
 17
 
  43974804
 
  43975063
 
  ENSEMBL   UCSC 
 
  260
 
  5
 
 16641
 
 gene;island
 
 0.040667
 
 0.0164313
 
 Not queried
 
  1.3457711
 
       NA
 
           NA
  
 
  
 8
 
  26047780
 
  26048065
 
  ENSEMBL   UCSC 
 
  286
 
  5
 
 40173
 
 island
 
 0.673671
 
 0.4316542
 
 Not queried
 
  1.3455499
 
       NA
 
           NA
  
 
  
 3
 
  13921803
 
  13921889
 
  ENSEMBL   UCSC 
 
   87
 
  4
 
 28167
 
 tss;island
 
 0.020616
 
 0.0904066
 
 Not queried
 
  1.3454999
 
       NA
 
           NA
  
 
  
 2
 
 171568984
 
 171569381
 
  ENSEMBL   UCSC 
 
  398
 
  6
 
 22917
 
 gene
 
 0.205810
 
 0.0808188
 
 Not queried
 
  1.3454959
 
       NA
 
           NA
  
 
  
 2
 
 197457393
 
 197457430
 
  ENSEMBL   UCSC 
 
   38
 
  5
 
 23794
 
 tss
 
 0.025079
 
 0.0608281
 
 Not queried
 
  1.3454913
 
       NA
 
           NA
  
 
  
 11
 
  69830436
 
  69830611
 
  ENSEMBL   UCSC 
 
  176
 
  4
 
 8602
 
 island
 
 0.336046
 
 0.0598876
 
 Not queried
 
  1.3453742
 
       NA
 
           NA
  
 
  
 16
 
  25228569
 
  25228709
 
  ENSEMBL   UCSC 
 
  141
 
  4
 
 14699
 
 gene
 
 0.784126
 
 0.8631170
 
 Not queried
 
  1.3449867
 
       NA
 
           NA
  
 
  
 15
 
  78556423
 
  78556534
 
  ENSEMBL   UCSC 
 
  112
 
  4
 
 13831
 
 tss;island
 
 0.806163
 
 0.9005791
 
 Not queried
 
  1.3448892
 
       NA
 
           NA
  
 
  
 14
 
  65239327
 
  65239653
 
  ENSEMBL   UCSC 
 
  327
 
  4
 
 12115
 
 gene;island
 
 0.470368
 
 0.6684259
 
 Not queried
 
  1.3448592
 
       NA
 
           NA
  
 
  
 5
 
    893015
 
    893076
 
  ENSEMBL   UCSC 
 
   62
 
  6
 
 31515
 
 gene;tss;island
 
 0.003627
 
 0.0048752
 
 Not queried
 
  1.3447711
 
       NA
 
           NA
  
 
  
 14
 
  55738748
 
  55739068
 
  ENSEMBL   UCSC 
 
  321
 
  5
 
 12088
 
 gene;island
 
 0.009127
 
 0.0057698
 
 Not queried
 
  1.3444546
 
       NA
 
           NA
  
 
  
 10
 
  61468548
 
  61468757
 
  ENSEMBL   UCSC 
 
  210
 
  4
 
 4597
 
 gene;island
 
 0.004196
 
 0.0137741
 
 Not queried
 
  1.3443643
 
       NA
 
           NA
  
 
  
 12
 
  12419752
 
  12420135
 
  ENSEMBL   UCSC 
 
  384
 
  6
 
 10245
 
 island
 
 0.021081
 
 0.0086118
 
 Not queried
 
  1.3439975
 
       NA
 
           NA
  
 
  
 17
 
  37784694
 
  37784895
 
  ENSEMBL   UCSC 
 
  202
 
  4
 
 16549
 
 gene;island
 
 0.602579
 
 0.8460472
 
 Not queried
 
  1.3439645
 
       NA
 
           NA
  
 
  
 12
 
  71552188
 
  71552569
 
  ENSEMBL   UCSC 
 
  382
 
  4
 
 9870
 
 tss
 
 0.688944
 
 0.5093221
 
 Not queried
 
  1.3439249
 
       NA
 
           NA
  
 
  
 6
 
  30113696
 
  30114039
 
  ENSEMBL   UCSC 
 
  344
 
 10
 
 33728
 
 gene
 
 0.671301
 
 0.7182652
 
 Not queried
 
  1.3439121
 
       NA
 
           NA
  
 
  
 7
 
   4118583
 
   4118868
 
  ENSEMBL   UCSC 
 
  286
 
  5
 
 36826
 
 gene
 
 0.477080
 
 0.6333599
 
 Not queried
 
  1.3438641
 
       NA
 
           NA
  
 
  
 2
 
 136289456
 
 136289752
 
  ENSEMBL   UCSC 
 
  297
 
  4
 
 22883
 
 gene;tss;island
 
 0.007814
 
 0.0046048
 
 Not queried
 
  1.3438251
 
       NA
 
           NA
  
 
  
 12
 
  50366280
 
  50366593
 
  ENSEMBL   UCSC 
 
  314
 
  4
 
 9699
 
 tss
 
 0.317655
 
 0.6378545
 
 Not queried
 
  1.3437923
 
       NA
 
           NA
  
 
  
 7
 
  27185136
 
  27185512
 
  ENSEMBL   UCSC 
 
  377
 
  4
 
 36935
 
 gene;island
 
 0.476664
 
 0.6855159
 
 Not queried
 
  1.3437058
 
       NA
 
           NA
  
 
  
 19
 
  34988751
 
  34988911
 
  ENSEMBL   UCSC 
 
  161
 
  4
 
 19976
 
 gene;island
 
 0.829856
 
 0.9419251
 
 Not queried
 
  1.3434487
 
       NA
 
           NA
  
 
  
 16
 
  53468112
 
  53468350
 
  ENSEMBL   UCSC 
 
  239
 
  5
 
 15925
 
 island
 
 0.015361
 
 0.0087453
 
 Not queried
 
  1.3434099
 
       NA
 
           NA
  
 
  
 1
 
  42846740
 
  42847055
 
  ENSEMBL   UCSC 
 
  316
 
  4
 
 464
 
 gene;island
 
 0.019937
 
 0.0055693
 
 Not queried
 
  1.3433746
 
       NA
 
           NA
  
 
  
 12
 
  53297384
 
  53298027
 
  ENSEMBL   UCSC 
 
  644
 
  7
 
 9141
 
 gene;island
 
 0.028281
 
 0.1254607
 
 Not queried
 
  1.3425259
 
       NA
 
           NA
  
 
  
 18
 
  33647525
 
  33647664
 
  ENSEMBL   UCSC 
 
  140
 
  5
 
 19240
 
 tss
 
 0.000270
 
 0.0004541
 
 Not queried
 
  1.3422418
 
       NA
 
           NA
  
 
  
 10
 
 133795558
 
 133795885
 
  ENSEMBL   UCSC 
 
  328
 
  4
 
 5443
 
 tss;island
 
 0.008739
 
 0.0033176
 
 Not queried
 
  1.3416090
 
       NA
 
           NA
  
 
  
 5
 
 176449842
 
 176450179
 
  ENSEMBL   UCSC 
 
  338
 
  4
 
 31914
 
 gene
 
 0.007271
 
 0.0040084
 
 Not queried
 
  1.3414135
 
       NA
 
           NA
  
 
  
 6
 
  29711249
 
  29711588
 
  ENSEMBL   UCSC 
 
  340
 
  4
 
 33690
 
 gene
 
 0.689387
 
 0.8085222
 
 Not queried
 
  1.3414059
 
       NA
 
           NA
  
 
  
 1
 
  24438703
 
  24439075
 
  ENSEMBL   UCSC 
 
  373
 
  5
 
 1355
 
 tss
 
 0.556629
 
 0.3083328
 
 Not queried
 
  1.3413828
 
       NA
 
           NA
  
 
  
 11
 
  24518149
 
  24518448
 
  ENSEMBL   UCSC 
 
  300
 
  9
 
 7205
 
 tss;island
 
 0.089813
 
 0.0523886
 
 Not queried
 
  1.3413324
 
       NA
 
           NA
  
 
  
 20
 
  46414765
 
  46414999
 
  ENSEMBL   UCSC 
 
  235
 
  6
 
 25442
 
 gene;island
 
 0.007239
 
 0.0213639
 
 Not queried
 
  1.3412680
 
       NA
 
           NA
  
 
  
 1
 
  87617710
 
  87617972
 
  ENSEMBL   UCSC 
 
  263
 
  4
 
 614
 
 gene;island
 
 0.039743
 
 0.1276778
 
 Not queried
 
  1.3410746
 
       NA
 
           NA
  
 
  
 11
 
  59578167
 
  59578540
 
  ENSEMBL   UCSC 
 
  374
 
  7
 
 8309
 
 island
 
 0.006566
 
 0.0735545
 
 Not queried
 
  1.3410492
 
       NA
 
           NA
  
 
  
 19
 
  22817176
 
  22817496
 
  ENSEMBL   UCSC 
 
  321
 
  4
 
 19965
 
 gene
 
 0.194964
 
 0.0810877
 
 Not queried
 
  1.3409779
 
       NA
 
           NA
  
 
  
 18
 
  72124660
 
  72124791
 
  ENSEMBL   UCSC 
 
  132
 
  5
 
 19322
 
 tss;island
 
 0.002507
 
 0.0054160
 
 Not queried
 
  1.3405478
 
       NA
 
           NA
  
 
  
 11
 
  93466719
 
  93467095
 
  ENSEMBL   UCSC 
 
  377
 
  4
 
 7622
 
 tss
 
 0.717437
 
 0.9166640
 
 Not queried
 
  1.3404150
 
       NA
 
           NA
  
 
  
 20
 
  50179151
 
  50179192
 
  ENSEMBL   UCSC 
 
   42
 
  4
 
 26256
 
 island
 
 0.024691
 
 0.0105734
 
 Not queried
 
  1.3403371
 
       NA
 
           NA
  
 
  
 8
 
  16859295
 
  16860121
 
  ENSEMBL   UCSC 
 
  827
 
 10
 
 40100
 
 island
 
 0.459391
 
 0.5958535
 
 Not queried
 
  1.3401799
 
       NA
 
           NA
  
 
  
 1
 
 202090849
 
 202091175
 
  ENSEMBL   UCSC 
 
  327
 
  5
 
 2174
 
 tss
 
 0.058874
 
 0.3557334
 
 Not queried
 
  1.3401596
 
       NA
 
           NA
  
 
  
 17
 
  48545805
 
  48546620
 
  ENSEMBL   UCSC 
 
  816
 
 12
 
 16687
 
 gene;island
 
 0.072268
 
 0.2036410
 
 Not queried
 
  1.3400755
 
       NA
 
           NA
  
 
  
 4
 
 107956955
 
 107957430
 
  ENSEMBL   UCSC 
 
  476
 
  5
 
 30082
 
 gene;island
 
 0.014943
 
 0.0043807
 
 Not queried
 
  1.3400222
 
       NA
 
           NA
  
 
  
 4
 
  99182242
 
  99182422
 
  ENSEMBL   UCSC 
 
  181
 
  4
 
 30509
 
 tss
 
 0.001526
 
 0.0136218
 
 Not queried
 
  1.3393859
 
       NA
 
           NA
  
 
  
 7
 
 100026182
 
 100026469
 
  ENSEMBL   UCSC 
 
  288
 
  7
 
 37780
 
 tss;island
 
 0.008860
 
 0.0043736
 
 Not queried
 
  1.3390921
 
       NA
 
           NA
  
 
  
 19
 
  58951599
 
  58951885
 
  ENSEMBL   UCSC 
 
  287
 
  8
 
 21143
 
 tss
 
 0.003803
 
 0.0061944
 
 Not queried
 
  1.3385919
 
       NA
 
           NA
  
 
  
 8
 
  55533655
 
  55534103
 
  ENSEMBL   UCSC 
 
  449
 
  7
 
 39306
 
 gene;island
 
 0.623401
 
 0.8067548
 
 Not queried
 
  1.3383254
 
       NA
 
           NA
  
 
  
 1
 
  91184126
 
  91184501
 
  ENSEMBL   UCSC 
 
  376
 
  4
 
 3459
 
 island
 
 0.072441
 
 0.1681792
 
 Not queried
 
  1.3382315
 
       NA
 
           NA
  
 
  
 4
 
    206112
 
    206339
 
  ENSEMBL   UCSC 
 
  228
 
  6
 
 30222
 
 tss
 
 0.220012
 
 0.2814439
 
 Not queried
 
  1.3382270
 
       NA
 
           NA
  
 
  
 12
 
 132939657
 
 132939992
 
  ENSEMBL   UCSC 
 
  336
 
  4
 
 11047
 
 island
 
 0.885482
 
 0.9577037
 
 Not queried
 
  1.3376552
 
       NA
 
           NA
  
 
  
 6
 
  10882238
 
  10882336
 
  ENSEMBL   UCSC 
 
   99
 
  5
 
 34631
 
 tss
 
 0.008204
 
 0.0379555
 
 Not queried
 
  1.3376364
 
       NA
 
           NA
  
 
  
 1
 
  21930180
 
  21930336
 
  ENSEMBL   UCSC 
 
  157
 
  5
 
 299
 
 gene;island
 
 0.708925
 
 0.8708513
 
 Not queried
 
  1.3375337
 
       NA
 
           NA
  
 
  
 7
 
  72349822
 
  72349862
 
  ENSEMBL   UCSC 
 
   41
 
  4
 
 37630
 
 tss
 
 0.004331
 
 0.0030153
 
 Not queried
 
  1.3367561
 
       NA
 
           NA
  
 
  
 12
 
 110939771
 
 110939833
 
  ENSEMBL   UCSC 
 
   63
 
  4
 
 9302
 
 gene
 
 0.004386
 
 0.0035625
 
 Not queried
 
  1.3364413
 
       NA
 
           NA
  
 
  
 11
 
  67250046
 
  67250445
 
  ENSEMBL   UCSC 
 
  400
 
  9
 
 7495
 
 tss
 
 0.005760
 
 0.0088130
 
 Not queried
 
  1.3364405
 
       NA
 
           NA
  
 
  
 3
 
  13114343
 
  13114803
 
  ENSEMBL   UCSC 
 
  461
 
  6
 
 28901
 
 island
 
 0.015354
 
 0.0247931
 
 Not queried
 
  1.3362553
 
       NA
 
           NA
  
 
  
 1
 
  25291905
 
  25292412
 
  ENSEMBL   UCSC 
 
  508
 
 11
 
 1366
 
 tss
 
 0.023109
 
 0.0732276
 
 Not queried
 
  1.3360332
 
       NA
 
           NA
  
 
  
 4
 
  15471214
 
  15471377
 
  ENSEMBL   UCSC 
 
  164
 
  4
 
 30309
 
 tss
 
 0.004855
 
 0.0196201
 
 Not queried
 
  1.3357854
 
       NA
 
           NA
  
 
  
 6
 
 168106055
 
 168106326
 
  ENSEMBL   UCSC 
 
  272
 
  4
 
 36645
 
 island
 
 0.722523
 
 0.7922689
 
 Not queried
 
  1.3355938
 
       NA
 
           NA
  
 
  
 1
 
 110081561
 
 110081965
 
  ENSEMBL   UCSC 
 
  405
 
  4
 
 1806
 
 tss
 
 0.435145
 
 0.6317223
 
 Not queried
 
  1.3353798
 
       NA
 
           NA
  
 
  
 12
 
  81471311
 
  81472177
 
  ENSEMBL   UCSC 
 
  867
 
 13
 
 10650
 
 island
 
 0.028670
 
 0.0720478
 
 Not queried
 
  1.3352177
 
       NA
 
           NA
  
 
  
 5
 
 140530028
 
 140530606
 
  ENSEMBL   UCSC 
 
  579
 
  5
 
 31809
 
 gene;island
 
 0.430187
 
 0.6621149
 
 Not queried
 
  1.3351088
 
       NA
 
           NA
  
 
  
 3
 
 138763852
 
 138764193
 
  ENSEMBL   UCSC 
 
  342
 
  7
 
 28629
 
 tss
 
 0.849192
 
 0.3073040
 
 Not queried
 
  1.3348563
 
       NA
 
           NA
  
 
  
 1
 
   1099563
 
   1099651
 
  ENSEMBL   UCSC 
 
   89
 
  4
 
 2406
 
 island
 
 0.112454
 
 0.2007860
 
 Not queried
 
  1.3345789
 
       NA
 
           NA
  
 
  
 1
 
  47690795
 
  47691301
 
  ENSEMBL   UCSC 
 
  507
 
  5
 
 515
 
 gene;island
 
 0.028943
 
 0.0174587
 
 Not queried
 
  1.3345650
 
       NA
 
           NA
  
 
  
 14
 
  23298687
 
  23299085
 
  ENSEMBL   UCSC 
 
  399
 
  9
 
 12288
 
 tss
 
 0.004845
 
 0.0104240
 
 Not queried
 
  1.3343222
 
       NA
 
           NA
  
 
  
 7
 
 112758734
 
 112758954
 
  ENSEMBL   UCSC 
 
  221
 
  6
 
 37846
 
 tss
 
 0.008079
 
 0.0032857
 
 Not queried
 
  1.3342666
 
       NA
 
           NA
  
 
  
 13
 
 100150711
 
 100151066
 
  ENSEMBL   UCSC 
 
  356
 
  4
 
 11838
 
 island
 
 0.010442
 
 0.0072484
 
 Not queried
 
  1.3341668
 
       NA
 
           NA
  
 
  
 2
 
 207506480
 
 207506675
 
  ENSEMBL   UCSC 
 
  196
 
  4
 
 23837
 
 tss;island
 
 0.122885
 
 0.3615690
 
 Not queried
 
  1.3339058
 
       NA
 
           NA
  
 
  
 1
 
  44482771
 
  44483296
 
  ENSEMBL   UCSC 
 
  526
 
  5
 
 486
 
 gene
 
 0.494410
 
 0.7136505
 
 Not queried
 
  1.3334890
 
       NA
 
           NA
  
 
  
 1
 
  28906210
 
  28906538
 
  ENSEMBL   UCSC 
 
  329
 
  5
 
 368
 
 gene;tss;island
 
 0.203953
 
 0.5748445
 
 Not queried
 
  1.3333950
 
       NA
 
           NA
  
 
  
 5
 
 156351010
 
 156351266
 
  ENSEMBL   UCSC 
 
  257
 
  4
 
 31872
 
 gene
 
 0.610492
 
 0.7980630
 
 Not queried
 
  1.3332718
 
       NA
 
           NA
  
 
  
 2
 
  97524767
 
  97525009
 
  ENSEMBL   UCSC 
 
  243
 
  4
 
 23525
 
 tss;island
 
 0.788464
 
 0.9569233
 
 Not queried
 
  1.3330202
 
       NA
 
           NA
  
 
  
 10
 
  77871958
 
  77872458
 
  ENSEMBL   UCSC 
 
  501
 
  5
 
 4646
 
 gene;island
 
 0.898622
 
 0.8413447
 
 Not queried
 
  1.3329322
 
       NA
 
           NA
  
 
  
 11
 
  27721222
 
  27721350
 
  ENSEMBL   UCSC 
 
  129
 
  5
 
 7211
 
 tss
 
 0.035842
 
 0.0146173
 
 Not queried
 
  1.3328031
 
       NA
 
           NA
  
 
  
 10
 
 126782326
 
 126782592
 
  ENSEMBL   UCSC 
 
  267
 
  6
 
 4811
 
 gene
 
 0.632204
 
 0.7021401
 
 Not queried
 
  1.3326510
 
       NA
 
           NA
  
 
  
 2
 
 113735377
 
 113735503
 
  ENSEMBL   UCSC 
 
  127
 
  4
 
 23581
 
 tss
 
 0.427057
 
 0.4977037
 
 Not queried
 
  1.3325177
 
       NA
 
           NA
  
 
  
 6
 
   4079342
 
   4079704
 
  ENSEMBL   UCSC 
 
  363
 
  7
 
 33539
 
 gene;tss;island
 
 0.470497
 
 0.5954213
 
 Not queried
 
  1.3324210
 
       NA
 
           NA
  
 
  
 5
 
  89705234
 
  89706015
 
  ENSEMBL   UCSC 
 
  782
 
 12
 
 32952
 
 island
 
 0.006738
 
 0.0032171
 
 Not queried
 
  1.3320702
 
       NA
 
           NA
  
 
  
 6
 
  30121774
 
  30122593
 
  ENSEMBL   UCSC 
 
  820
 
 18
 
 33729
 
 gene
 
 0.456314
 
 0.7094385
 
 Not queried
 
  1.3318221
 
       NA
 
           NA
  
 
  
 17
 
   6899522
 
   6899888
 
  ENSEMBL   UCSC 
 
  367
 
  5
 
 16380
 
 gene
 
 0.915073
 
 0.5954290
 
 Not queried
 
  1.3316057
 
       NA
 
           NA
  
 
  
 20
 
  61162000
 
  61162216
 
  ENSEMBL   UCSC 
 
  217
 
  7
 
 25491
 
 gene
 
 0.352556
 
 0.4537939
 
 Not queried
 
  1.3314236
 
       NA
 
           NA
  
 
  
 11
 
  69813290
 
  69813451
 
  ENSEMBL   UCSC 
 
  162
 
  4
 
 8601
 
 island
 
 0.832415
 
 0.9033484
 
 Not queried
 
  1.3312672
 
       NA
 
           NA
  
 
  
 4
 
 100574455
 
 100574653
 
  ENSEMBL   UCSC 
 
  199
 
  4
 
 31199
 
 island
 
 0.586636
 
 0.7588353
 
 Not queried
 
  1.3308322
 
       NA
 
           NA
  
 
  
 7
 
    207605
 
    207774
 
  ENSEMBL   UCSC 
 
  170
 
  4
 
 36703
 
 gene;island
 
 0.693018
 
 0.8361191
 
 Not queried
 
  1.3300534
 
       NA
 
           NA
  
 
  
 17
 
   6756633
 
   6756891
 
  ENSEMBL   UCSC 
 
  259
 
  5
 
 17055
 
 tss
 
 0.386454
 
 0.6004849
 
 Not queried
 
  1.3299360
 
       NA
 
           NA
  
 
  
 10
 
 115998843
 
 115999174
 
  ENSEMBL   UCSC 
 
  332
 
  6
 
 6152
 
 island
 
 0.010752
 
 0.0032602
 
 Not queried
 
  1.3292052
 
       NA
 
           NA
  
 
  
 10
 
 102986525
 
 102986973
 
  ENSEMBL   UCSC 
 
  449
 
  6
 
 6059
 
 island
 
 0.248545
 
 0.0489270
 
 Not queried
 
  1.3291694
 
       NA
 
           NA
  
 
  
 19
 
  17877419
 
  17877846
 
  ENSEMBL   UCSC 
 
  428
 
  6
 
 19921
 
 gene;island
 
 0.199996
 
 0.4403127
 
 Not queried
 
  1.3281339
 
       NA
 
           NA
  
 
  
 6
 
  41410759
 
  41411128
 
  ENSEMBL   UCSC 
 
  370
 
  4
 
 36121
 
 island
 
 0.208829
 
 0.4405369
 
 Not queried
 
  1.3280331
 
       NA
 
           NA
  
 
  
 3
 
 136537607
 
 136537856
 
  ENSEMBL   UCSC 
 
  250
 
  8
 
 28614
 
 tss
 
 0.043608
 
 0.0153464
 
 Not queried
 
  1.3278532
 
       NA
 
           NA
  
 
  
 12
 
 106978920
 
 106979229
 
  ENSEMBL   UCSC 
 
  310
 
  4
 
 9280
 
 gene;island
 
 0.007390
 
 0.0071922
 
 Not queried
 
  1.3277048
 
       NA
 
           NA
  
 
  
 6
 
  32184296
 
  32184498
 
  ENSEMBL   UCSC 
 
  203
 
  4
 
 34095
 
 gene
 
 0.378027
 
 0.6341835
 
 Not queried
 
  1.3276696
 
       NA
 
           NA
  
 
  
 5
 
  41925120
 
  41925229
 
  ENSEMBL   UCSC 
 
  110
 
  5
 
 32040
 
 tss
 
 0.011412
 
 0.0082246
 
 Not queried
 
  1.3274570
 
       NA
 
           NA
  
 
  
 4
 
  37246688
 
  37247022
 
  ENSEMBL   UCSC 
 
  335
 
  4
 
 30973
 
 island
 
 0.025122
 
 0.0239852
 
 Not queried
 
  1.3270709
 
       NA
 
           NA
  
 
  
 22
 
  38054505
 
  38054735
 
  ENSEMBL   UCSC 
 
  231
 
  5
 
 27119
 
 tss
 
 0.004791
 
 0.0123459
 
 Not queried
 
  1.3270185
 
       NA
 
           NA
  
 
  
 17
 
  40932199
 
  40932572
 
  ENSEMBL   UCSC 
 
  374
 
  8
 
 17374
 
 tss
 
 0.141796
 
 0.5078082
 
 Not queried
 
  1.3266239
 
       NA
 
           NA
  
 
  
 4
 
 154713235
 
 154713496
 
  ENSEMBL   UCSC 
 
  262
 
  4
 
 31378
 
 island
 
 0.276310
 
 0.3014708
 
 Not queried
 
  1.3261402
 
       NA
 
           NA
  
 
  
 20
 
  47896769
 
  47897451
 
  ENSEMBL   UCSC 
 
  683
 
  7
 
 25447
 
 gene;island
 
 0.011318
 
 0.0187084
 
 Not queried
 
  1.3259477
 
       NA
 
           NA
  
 
  
 3
 
 190105524
 
 190105821
 
  ENSEMBL   UCSC 
 
  298
 
  8
 
 28785
 
 tss
 
 0.460128
 
 0.7338645
 
 Not queried
 
  1.3257845
 
       NA
 
           NA
  
 
  
 15
 
  42500107
 
  42500843
 
  ENSEMBL   UCSC 
 
  737
 
 11
 
 14072
 
 island
 
 0.021582
 
 0.0707369
 
 Not queried
 
  1.3251057
 
       NA
 
           NA
  
 
  
 12
 
  82153109
 
  82153464
 
  ENSEMBL   UCSC 
 
  356
 
  7
 
 9895
 
 tss
 
 0.103427
 
 0.0767642
 
 Not queried
 
  1.3247954
 
       NA
 
           NA
  
 
  
 12
 
  82153008
 
  82153464
 
  ENSEMBL   UCSC 
 
  457
 
  9
 
 10652
 
 island
 
 0.103427
 
 0.0767642
 
 Not queried
 
  1.3247954
 
       NA
 
           NA
  
 
  
 1
 
 228395145
 
 228395450
 
  ENSEMBL   UCSC 
 
  306
 
  4
 
 2302
 
 tss
 
 0.382861
 
 0.5863074
 
 Not queried
 
  1.3241651
 
       NA
 
           NA
  
 
  
 22
 
  49376354
 
  49376628
 
  ENSEMBL   UCSC 
 
  275
 
  4
 
 27602
 
 island
 
 0.526609
 
 0.7024462
 
 Not queried
 
  1.3235367
 
       NA
 
           NA
  
 
  
 4
 
 120221986
 
 120222239
 
  ENSEMBL   UCSC 
 
  254
 
  9
 
 30109
 
 gene;tss;island
 
 0.007178
 
 0.0055835
 
 Not queried
 
  1.3233225
 
       NA
 
           NA
  
 
  
 12
 
  48722474
 
  48722754
 
  ENSEMBL   UCSC 
 
  281
 
  4
 
 9670
 
 tss;island
 
 0.374954
 
 0.6239214
 
 Not queried
 
  1.3230349
 
       NA
 
           NA
  
 
  
 21
 
  40752339
 
  40752891
 
  ENSEMBL   UCSC 
 
  553
 
  5
 
 26427
 
 gene
 
 0.021089
 
 0.0083721
 
 Not queried
 
  1.3229878
 
       NA
 
           NA
  
 
  
 10
 
 112836471
 
 112836785
 
  ENSEMBL   UCSC 
 
  315
 
  7
 
 5363
 
 tss;island
 
 0.004878
 
 0.0163537
 
 Not queried
 
  1.3229523
 
       NA
 
           NA
  
 
  
 17
 
  74463439
 
  74463585
 
  ENSEMBL   UCSC 
 
  147
 
  4
 
 17679
 
 tss
 
 0.539558
 
 0.7918875
 
 Not queried
 
  1.3229161
 
       NA
 
           NA
  
 
  
 1
 
  78149330
 
  78149566
 
  ENSEMBL   UCSC 
 
  237
 
  5
 
 1707
 
 tss;island
 
 0.006650
 
 0.0044028
 
 Not queried
 
  1.3229032
 
       NA
 
           NA
  
 
  
 18
 
  45457585
 
  45457604
 
  ENSEMBL   UCSC 
 
   20
 
  4
 
 19265
 
 tss;island
 
 0.005977
 
 0.0074180
 
 Not queried
 
  1.3228487
 
       NA
 
           NA
  
 
  
 19
 
  58038573
 
  58038689
 
  ENSEMBL   UCSC 
 
  117
 
  6
 
 21118
 
 tss
 
 0.008146
 
 0.0188038
 
 Not queried
 
  1.3224021
 
       NA
 
           NA
  
 
  
 19
 
  58038573
 
  58038856
 
  ENSEMBL   UCSC 
 
  284
 
  8
 
 22473
 
 island
 
 0.008146
 
 0.0132972
 
 Not queried
 
  1.3224021
 
       NA
 
           NA
  
 
  
 2
 
  63284481
 
  63284784
 
  ENSEMBL   UCSC 
 
  304
 
  5
 
 24341
 
 island
 
 0.033434
 
 0.0677365
 
 Not queried
 
  1.3218693
 
       NA
 
           NA
  
 
  
 7
 
 157412628
 
 157413057
 
  ENSEMBL   UCSC 
 
  430
 
  5
 
 37332
 
 gene;island
 
 0.579072
 
 0.6116761
 
 Not queried
 
  1.3215474
 
       NA
 
           NA
  
 
  
 19
 
  36427426
 
  36427779
 
  ENSEMBL   UCSC 
 
  354
 
  5
 
 20718
 
 tss;island
 
 0.019034
 
 0.0196263
 
 Not queried
 
  1.3212470
 
       NA
 
           NA
  
 
  
 11
 
  10715175
 
  10715445
 
  ENSEMBL   UCSC 
 
  271
 
  7
 
 6510
 
 gene
 
 0.378086
 
 0.7017001
 
 Not queried
 
  1.3212401
 
       NA
 
           NA
  
 
  
 7
 
  94953653
 
  94953877
 
  ENSEMBL   UCSC 
 
  225
 
  5
 
 37096
 
 gene
 
 0.759796
 
 0.5190337
 
 Not queried
 
  1.3206850
 
       NA
 
           NA
  
 
  
 8
 
 145025059
 
 145025178
 
  ENSEMBL   UCSC 
 
  120
 
  5
 
 39966
 
 tss
 
 0.098612
 
 0.2035250
 
 Not queried
 
  1.3205021
 
       NA
 
           NA
  
 
  
 2
 
 178976981
 
 178977341
 
  ENSEMBL   UCSC 
 
  361
 
  7
 
 24926
 
 island
 
 0.005811
 
 0.0060979
 
 Not queried
 
  1.3201656
 
       NA
 
           NA
  
 
  
 3
 
  13678918
 
  13679259
 
  ENSEMBL   UCSC 
 
  342
 
  4
 
 27683
 
 gene;island
 
 0.518898
 
 0.7201796
 
 Not queried
 
  1.3199200
 
       NA
 
           NA
  
 
  
 17
 
  77030221
 
  77030418
 
  ENSEMBL   UCSC 
 
  198
 
  4
 
 17712
 
 tss
 
 0.287107
 
 0.5202208
 
 Not queried
 
  1.3197596
 
       NA
 
           NA
  
 
  
 19
 
   7587289
 
   7587465
 
  ENSEMBL   UCSC 
 
  177
 
  5
 
 20399
 
 tss
 
 0.001959
 
 0.0059026
 
 Not queried
 
  1.3197320
 
       NA
 
           NA
  
 
  
 6
 
  32179862
 
  32180305
 
  ENSEMBL   UCSC 
 
  444
 
  6
 
 34092
 
 gene
 
 0.402688
 
 0.6109645
 
 Not queried
 
  1.3195395
 
       NA
 
           NA
  
 
  
 6
 
  28753834
 
  28754198
 
  ENSEMBL   UCSC 
 
  365
 
  5
 
 35594
 
 island
 
 0.079841
 
 0.1767412
 
 Not queried
 
  1.3192815
 
       NA
 
           NA
  
 
  
 12
 
  25539423
 
  25539889
 
  ENSEMBL   UCSC 
 
  467
 
  5
 
 10293
 
 island
 
 0.002870
 
 0.0019916
 
 Not queried
 
  1.3191759
 
       NA
 
           NA
  
 
  
 11
 
   2919689
 
   2919808
 
  ENSEMBL   UCSC 
 
  120
 
  4
 
 6468
 
 gene;tss;island
 
 0.133863
 
 0.0907728
 
 Not queried
 
  1.3191604
 
       NA
 
           NA
  
 
  
 19
 
   1851750
 
   1852165
 
  ENSEMBL   UCSC 
 
  416
 
  5
 
 21249
 
 island
 
 0.061686
 
 0.0399802
 
 Not queried
 
  1.3191519
 
       NA
 
           NA
  
 
  
 17
 
   5019310
 
   5019989
 
  ENSEMBL   UCSC 
 
  680
 
  7
 
 16364
 
 gene;island
 
 0.701741
 
 0.8511294
 
 Not queried
 
  1.3190657
 
       NA
 
           NA
  
 
  
 14
 
 105612753
 
 105613102
 
  ENSEMBL   UCSC 
 
  350
 
  4
 
 12255
 
 gene;island
 
 0.603995
 
 0.7851802
 
 Not queried
 
  1.3188549
 
       NA
 
           NA
  
 
  
 11
 
 118214762
 
 118214927
 
  ENSEMBL   UCSC 
 
  166
 
  4
 
 7730
 
 tss
 
 0.578993
 
 0.6371537
 
 Not queried
 
  1.3186497
 
       NA
 
           NA
  
 
  
 8
 
  11565277
 
  11565725
 
  ENSEMBL   UCSC 
 
  449
 
  4
 
 39205
 
 gene;island
 
 0.131781
 
 0.0561271
 
 Not queried
 
  1.3184003
 
       NA
 
           NA
  
 
  
 3
 
  99536614
 
  99536649
 
  ENSEMBL   UCSC 
 
   36
 
  5
 
 28464
 
 tss
 
 0.004375
 
 0.0044859
 
 Not queried
 
  1.3183427
 
       NA
 
           NA
  
 
  
 19
 
  46296257
 
  46296285
 
  ENSEMBL   UCSC 
 
   29
 
  4
 
 20880
 
 tss;island
 
 0.003765
 
 0.0046473
 
 Not queried
 
  1.3182795
 
       NA
 
           NA
  
 
  
 15
 
  78913474
 
  78913820
 
  ENSEMBL   UCSC 
 
  347
 
  5
 
 14357
 
 island
 
 0.225805
 
 0.1044838
 
 Not queried
 
  1.3180718
 
       NA
 
           NA
  
 
  
 5
 
    851101
 
    851458
 
  ENSEMBL   UCSC 
 
  358
 
  6
 
 31962
 
 tss;island
 
 0.620274
 
 0.7310588
 
 Not queried
 
  1.3179556
 
       NA
 
           NA
  
 
  
 2
 
 176973688
 
 176974060
 
  ENSEMBL   UCSC 
 
  373
 
  5
 
 22935
 
 gene;island
 
 0.345368
 
 0.0681370
 
 Not queried
 
  1.3175156
 
       NA
 
           NA
  
 
  
 6
 
    292329
 
    292596
 
  ENSEMBL   UCSC 
 
  268
 
  4
 
 33529
 
 gene;island
 
 0.052280
 
 0.0218303
 
 Not queried
 
  1.3169532
 
       NA
 
           NA
  
 
  
 19
 
   5250376
 
   5250655
 
  ENSEMBL   UCSC 
 
  280
 
  4
 
 19749
 
 gene;island
 
 0.638361
 
 0.6682989
 
 Not queried
 
  1.3164635
 
       NA
 
           NA
  
 
  
 8
 
  97171668
 
  97172012
 
  ENSEMBL   UCSC 
 
  345
 
  4
 
 39367
 
 gene;island
 
 0.118596
 
 0.3097341
 
 Not queried
 
  1.3159348
 
       NA
 
           NA
  
 
  
 15
 
  45421578
 
  45422054
 
  ENSEMBL   UCSC 
 
  477
 
  8
 
 13327
 
 gene
 
 0.007600
 
 0.0626528
 
 Not queried
 
  1.3158619
 
       NA
 
           NA
  
 
  
 5
 
 108745243
 
 108745575
 
  ENSEMBL   UCSC 
 
  333
 
  4
 
 31705
 
 gene;island
 
 0.008932
 
 0.0045740
 
 Not queried
 
  1.3157699
 
       NA
 
           NA
  
 
  
 8
 
 145026305
 
 145026613
 
  ENSEMBL   UCSC 
 
  309
 
  4
 
 39503
 
 gene;island
 
 0.001443
 
 0.0057776
 
 Not queried
 
  1.3149824
 
       NA
 
           NA
  
 
  
 7
 
  24323559
 
  24323799
 
  ENSEMBL   UCSC 
 
  241
 
  6
 
 37477
 
 tss
 
 0.026701
 
 0.0108448
 
 Not queried
 
  1.3149675
 
       NA
 
           NA
  
 
  
 1
 
 245316781
 
 245317174
 
  ENSEMBL   UCSC 
 
  394
 
  5
 
 4401
 
 island
 
 0.021689
 
 0.0238765
 
 Not queried
 
  1.3145567
 
       NA
 
           NA
  
 
  
 1
 
 179544476
 
 179544765
 
  ENSEMBL   UCSC 
 
  290
 
  4
 
 904
 
 gene;island
 
 0.347934
 
 0.4645234
 
 Not queried
 
  1.3142785
 
       NA
 
           NA
  
 
  
 22
 
  24181212
 
  24181270
 
  ENSEMBL   UCSC 
 
   59
 
  4
 
 27012
 
 tss
 
 0.004670
 
 0.0158873
 
 Not queried
 
  1.3140305
 
       NA
 
           NA
  
 
  
 22
 
  24180995
 
  24181270
 
  ENSEMBL   UCSC 
 
  276
 
  6
 
 27354
 
 island
 
 0.004670
 
 0.0158873
 
 Not queried
 
  1.3140305
 
       NA
 
           NA
  
 
  
 16
 
  67926607
 
  67927351
 
  ENSEMBL   UCSC 
 
  745
 
  7
 
 16034
 
 island
 
 0.008311
 
 0.0091139
 
 Not queried
 
  1.3138402
 
       NA
 
           NA
  
 
  
 9
 
    841340
 
    841636
 
  ENSEMBL   UCSC 
 
  297
 
  5
 
 40801
 
 tss;island
 
 0.013229
 
 0.0207864
 
 Not queried
 
  1.3132840
 
       NA
 
           NA
  
 
  
 17
 
  41277694
 
  41279022
 
  ENSEMBL   UCSC 
 
 1329
 
 28
 
 16595
 
 gene
 
 0.544481
 
 0.8628551
 
 Not queried
 
  1.3131938
 
       NA
 
           NA
  
 
  
 4
 
  54931064
 
  54931210
 
  ENSEMBL   UCSC 
 
  147
 
  4
 
 30398
 
 tss;island
 
 0.002074
 
 0.0071739
 
 Not queried
 
  1.3131592
 
       NA
 
           NA
  
 
  
 3
 
 157261004
 
 157261273
 
  ENSEMBL   UCSC 
 
  270
 
  8
 
 29615
 
 island
 
 0.095657
 
 0.0264348
 
 Not queried
 
  1.3129755
 
       NA
 
           NA
  
 
  
 12
 
  12878752
 
  12878810
 
  ENSEMBL   UCSC 
 
   59
 
  4
 
 9576
 
 tss
 
 0.003789
 
 0.0026063
 
 Not queried
 
  1.3129291
 
       NA
 
           NA
  
 
  
 12
 
  12878752
 
  12878887
 
  ENSEMBL   UCSC 
 
  136
 
  6
 
 10254
 
 island
 
 0.002221
 
 0.0025580
 
 Not queried
 
  1.3129291
 
       NA
 
           NA
  
 
  
 5
 
 135188170
 
 135188477
 
  ENSEMBL   UCSC 
 
  308
 
  4
 
 31759
 
 gene;island
 
 0.556725
 
 0.6757144
 
 Not queried
 
  1.3127551
 
       NA
 
           NA
  
 
  
 1
 
 156646293
 
 156646746
 
  ENSEMBL   UCSC 
 
  454
 
  5
 
 827
 
 gene;island
 
 0.032762
 
 0.0324094
 
 Not queried
 
  1.3120201
 
       NA
 
           NA
  
 
  
 3
 
  42623511
 
  42623862
 
  ENSEMBL   UCSC 
 
  352
 
  5
 
 28244
 
 tss
 
 0.006182
 
 0.0083629
 
 Not queried
 
  1.3118036
 
       NA
 
           NA
  
 
  
 10
 
 106113175
 
 106113447
 
  ENSEMBL   UCSC 
 
  273
 
  5
 
 5349
 
 tss
 
 0.026960
 
 0.0050901
 
 Not queried
 
  1.3116794
 
       NA
 
           NA
  
 
  
 17
 
   7153814
 
   7154245
 
  ENSEMBL   UCSC 
 
  432
 
  4
 
 16387
 
 gene;island
 
 0.212779
 
 0.1324541
 
 Not queried
 
  1.3115801
 
       NA
 
           NA
  
 
  
 17
 
  80455521
 
  80455872
 
  ENSEMBL   UCSC 
 
  352
 
  5
 
 18998
 
 island
 
 0.007254
 
 0.0305718
 
 Not queried
 
  1.3114069
 
       NA
 
           NA
  
 
  
 2
 
  11485384
 
  11485561
 
  ENSEMBL   UCSC 
 
  178
 
  4
 
 23189
 
 tss;island
 
 0.054428
 
 0.1193491
 
 Not queried
 
  1.3113821
 
       NA
 
           NA
  
 
  
 21
 
  34442160
 
  34442377
 
  ENSEMBL   UCSC 
 
  218
 
  7
 
 26517
 
 tss
 
 0.032952
 
 0.0135514
 
 Not queried
 
  1.3105072
 
       NA
 
           NA
  
 
  
 5
 
  16936808
 
  16936927
 
  ENSEMBL   UCSC 
 
  120
 
  4
 
 31998
 
 tss;island
 
 0.025208
 
 0.0627149
 
 Not queried
 
  1.3103696
 
       NA
 
           NA
  
 
  
 11
 
 114309932
 
 114310057
 
  ENSEMBL   UCSC 
 
  126
 
  7
 
 7708
 
 tss
 
 0.007542
 
 0.0037448
 
 Not queried
 
  1.3102269
 
       NA
 
           NA
  
 
  
 11
 
   2466409
 
   2466909
 
  ENSEMBL   UCSC 
 
  501
 
  6
 
 6458
 
 gene;island
 
 0.038163
 
 0.0429309
 
 Not queried
 
  1.3101421
 
       NA
 
           NA
  
 
  
 16
 
  23160906
 
  23161212
 
  ENSEMBL   UCSC 
 
  307
 
  4
 
 15135
 
 tss;island
 
 0.028070
 
 0.0135809
 
 Not queried
 
  1.3100949
 
       NA
 
           NA
  
 
  
 12
 
  49393166
 
  49393408
 
  ENSEMBL   UCSC 
 
  243
 
  7
 
 9680
 
 tss
 
 0.025404
 
 0.0459443
 
 Not queried
 
  1.3099799
 
       NA
 
           NA
  
 
  
 16
 
   1383433
 
   1383595
 
  ENSEMBL   UCSC 
 
  163
 
  4
 
 14990
 
 tss;island
 
 0.044641
 
 0.0524501
 
 Not queried
 
  1.3097977
 
       NA
 
           NA
  
 
  
 17
 
  79410470
 
  79410752
 
  ENSEMBL   UCSC 
 
  283
 
  5
 
 16909
 
 gene;island
 
 0.578579
 
 0.7195463
 
 Not queried
 
  1.3093028
 
       NA
 
           NA
  
 
  
 16
 
  24267226
 
  24267559
 
  ENSEMBL   UCSC 
 
  334
 
  4
 
 14697
 
 gene;island
 
 0.076003
 
 0.0295978
 
 Not queried
 
  1.3092312
 
       NA
 
           NA
  
 
  
 1
 
  93913424
 
  93913524
 
  ENSEMBL   UCSC 
 
  101
 
  4
 
 1758
 
 tss;island
 
 0.069435
 
 0.0362315
 
 Not queried
 
  1.3089364
 
       NA
 
           NA
  
 
  
 6
 
   1515524
 
   1515797
 
  ENSEMBL   UCSC 
 
  274
 
  4
 
 35368
 
 island
 
 0.733260
 
 0.8872240
 
 Not queried
 
  1.3089299
 
       NA
 
           NA
  
 
  
 11
 
   2162183
 
   2162930
 
  ENSEMBL   UCSC 
 
  748
 
 19
 
 6448
 
 gene;island
 
 0.046115
 
 0.0784362
 
 Not queried
 
  1.3089205
 
       NA
 
           NA
  
 
  
 6
 
  74063982
 
  74064594
 
  ENSEMBL   UCSC 
 
  613
 
  8
 
 35066
 
 tss;island
 
 0.553117
 
 0.8255242
 
 Not queried
 
  1.3088668
 
       NA
 
           NA
  
 
  
 10
 
  28822348
 
  28822500
 
  ENSEMBL   UCSC 
 
  153
 
  5
 
 4545
 
 gene;island
 
 0.003789
 
 0.0035488
 
 Not queried
 
  1.3086610
 
       NA
 
           NA
  
 
  
 1
 
 156357839
 
 156358408
 
  ENSEMBL   UCSC 
 
  570
 
 10
 
 3854
 
 island
 
 0.038194
 
 0.2686633
 
 Not queried
 
  1.3084116
 
       NA
 
           NA
  
 
  
 7
 
  33944213
 
  33944420
 
  ENSEMBL   UCSC 
 
  208
 
  4
 
 37533
 
 tss;island
 
 0.002201
 
 0.0088479
 
 Not queried
 
  1.3083107
 
       NA
 
           NA
  
 
  
 6
 
 101846872
 
 101847058
 
  ENSEMBL   UCSC 
 
  187
 
  5
 
 34432
 
 gene
 
 0.261900
 
 0.5142956
 
 Not queried
 
  1.3079413
 
       NA
 
           NA
  
 
  
 11
 
   6341717
 
   6341908
 
  ENSEMBL   UCSC 
 
  192
 
  8
 
 8013
 
 island
 
 0.018328
 
 0.0396399
 
 Not queried
 
  1.3075341
 
       NA
 
           NA
  
 
  
 14
 
  95234658
 
  95235127
 
  ENSEMBL   UCSC 
 
  470
 
  7
 
 12196
 
 gene;island
 
 0.036316
 
 0.0097915
 
 Not queried
 
  1.3072035
 
       NA
 
           NA
  
 
  
 19
 
  37958346
 
  37958752
 
  ENSEMBL   UCSC 
 
  407
 
  7
 
 20738
 
 tss;island
 
 0.026483
 
 0.0236157
 
 Not queried
 
  1.3071846
 
       NA
 
           NA
  
 
  
 10
 
   8095173
 
   8096818
 
  ENSEMBL   UCSC 
 
 1646
 
 43
 
 4942
 
 tss
 
 0.007458
 
 0.0149099
 
 Not queried
 
  1.3070972
 
       NA
 
           NA
  
 
  
 12
 
 104443925
 
 104444136
 
  ENSEMBL   UCSC 
 
  212
 
  8
 
 9956
 
 tss
 
 0.024331
 
 0.1142040
 
 Not queried
 
  1.3069725
 
       NA
 
           NA
  
 
  
 5
 
   1641593
 
   1641876
 
  ENSEMBL   UCSC 
 
  284
 
  6
 
 32637
 
 island
 
 0.467805
 
 0.0381323
 
 Not queried
 
  1.3067081
 
       NA
 
           NA
  
 
  
 10
 
 127464440
 
 127464762
 
  ENSEMBL   UCSC 
 
  323
 
  6
 
 5430
 
 tss
 
 0.516124
 
 0.7168597
 
 Not queried
 
  1.3062463
 
       NA
 
           NA
  
 
  
 13
 
  45885173
 
  45885599
 
  ENSEMBL   UCSC 
 
  427
 
  6
 
 11709
 
 island
 
 0.019755
 
 0.0070372
 
 Not queried
 
  1.3059167
 
       NA
 
           NA
  
 
  
 1
 
   6304272
 
   6304499
 
  ENSEMBL   UCSC 
 
  228
 
  4
 
 165
 
 gene
 
 0.246307
 
 0.1482807
 
 Not queried
 
  1.3052286
 
       NA
 
           NA
  
 
  
 1
 
 169336845
 
 169337140
 
  ENSEMBL   UCSC 
 
  296
 
  6
 
 879
 
 gene
 
 0.010032
 
 0.0049781
 
 Not queried
 
  1.3050291
 
       NA
 
           NA
  
 
  
 17
 
  20811860
 
  20812310
 
  ENSEMBL   UCSC 
 
  451
 
  5
 
 18113
 
 island
 
 0.260546
 
 0.0354532
 
 Not queried
 
  1.3047099
 
       NA
 
           NA
  
 
  
 5
 
 179588440
 
 179588838
 
  ENSEMBL   UCSC 
 
  399
 
  4
 
 31940
 
 gene;island
 
 0.580672
 
 0.7219967
 
 Not queried
 
  1.3043704
 
       NA
 
           NA
  
 
  
 8
 
 143761503
 
 143761811
 
  ENSEMBL   UCSC 
 
  309
 
  6
 
 39932
 
 tss
 
 0.111687
 
 0.3808937
 
 Not queried
 
  1.3043694
 
       NA
 
           NA
  
 
  
 6
 
 168197625
 
 168198163
 
  ENSEMBL   UCSC 
 
  539
 
  7
 
 35343
 
 tss
 
 0.454011
 
 0.0837671
 
 Not queried
 
  1.3043544
 
       NA
 
           NA
  
 
  
 15
 
  78632670
 
  78632861
 
  ENSEMBL   UCSC 
 
  192
 
  5
 
 13438
 
 gene
 
 0.037403
 
 0.0974514
 
 Not queried
 
  1.3040383
 
       NA
 
           NA
  
 
  
 1
 
 220263017
 
 220263189
 
  ENSEMBL   UCSC 
 
  173
 
  4
 
 1016
 
 gene
 
 0.006060
 
 0.0024280
 
 Not queried
 
  1.3040186
 
       NA
 
           NA
  
 
  
 10
 
 102027447
 
 102027787
 
  ENSEMBL   UCSC 
 
  341
 
  8
 
 5289
 
 tss
 
 0.005728
 
 0.0026126
 
 Not queried
 
  1.3038973
 
       NA
 
           NA
  
 
  
 7
 
 135413023
 
 135413232
 
  ENSEMBL   UCSC 
 
  210
 
  5
 
 37920
 
 tss
 
 0.870220
 
 0.9283427
 
 Not queried
 
  1.3037978
 
       NA
 
           NA
  
 
  
 2
 
 201980504
 
 201980867
 
  ENSEMBL   UCSC 
 
  364
 
  7
 
 23814
 
 tss
 
 0.021725
 
 0.0846817
 
 Not queried
 
  1.3034802
 
       NA
 
           NA
  
 
  
 15
 
  25307169
 
  25307422
 
  ENSEMBL   UCSC 
 
  254
 
  4
 
 13522
 
 tss
 
 0.693432
 
 0.8971573
 
 Not queried
 
  1.3032700
 
       NA
 
           NA
  
 
  
 11
 
    315751
 
    316456
 
  ENSEMBL   UCSC 
 
  706
 
  6
 
 7841
 
 island
 
 0.973385
 
 0.9000983
 
 Not queried
 
  1.3029715
 
       NA
 
           NA
  
 
  
 7
 
 153749736
 
 153749759
 
  ENSEMBL   UCSC 
 
   24
 
  4
 
 37302
 
 gene;tss;island
 
 0.198792
 
 0.4287996
 
 Not queried
 
  1.3027699
 
       NA
 
           NA
  
 
  
 17
 
  11923892
 
  11924127
 
  ENSEMBL   UCSC 
 
  236
 
  7
 
 17139
 
 tss;island
 
 0.002572
 
 0.0014297
 
 Not queried
 
  1.3027009
 
       NA
 
           NA
  
 
  
 4
 
 117520432
 
 117520758
 
  ENSEMBL   UCSC 
 
  327
 
  4
 
 31263
 
 island
 
 0.520689
 
 0.7288285
 
 Not queried
 
  1.3025559
 
       NA
 
           NA
  
 
  
 3
 
  46608025
 
  46608179
 
  ENSEMBL   UCSC 
 
  155
 
  5
 
 28276
 
 tss
 
 0.022287
 
 0.4627800
 
 Not queried
 
  1.3024819
 
       NA
 
           NA
  
 
  
 8
 
  38326358
 
  38326730
 
  ENSEMBL   UCSC 
 
  373
 
  6
 
 39658
 
 tss
 
 0.767491
 
 0.9515522
 
 Not queried
 
  1.3024334
 
       NA
 
           NA
  
 
  
 2
 
 220196530
 
 220196755
 
  ENSEMBL   UCSC 
 
  226
 
  4
 
 23033
 
 gene;island
 
 0.546525
 
 0.3626953
 
 Not queried
 
  1.3023127
 
       NA
 
           NA
  
 
  
 7
 
  45613676
 
  45613825
 
  ENSEMBL   UCSC 
 
  150
 
  4
 
 37584
 
 tss;island
 
 0.018983
 
 0.0147382
 
 Not queried
 
  1.3021594
 
       NA
 
           NA
  
 
  
 14
 
  81407988
 
  81408275
 
  ENSEMBL   UCSC 
 
  288
 
  4
 
 13047
 
 island
 
 0.003387
 
 0.0047245
 
 Not queried
 
  1.3016670
 
       NA
 
           NA
  
 
  
 13
 
  88328251
 
  88328471
 
  ENSEMBL   UCSC 
 
  221
 
  4
 
 11187
 
 gene;island
 
 0.422596
 
 0.7762302
 
 Not queried
 
  1.3010955
 
       NA
 
           NA
  
 
  
 1
 
  41981735
 
  41982115
 
  ENSEMBL   UCSC 
 
  381
 
  6
 
 462
 
 gene;island
 
 0.022284
 
 0.0419241
 
 Not queried
 
  1.3009977
 
       NA
 
           NA
  
 
  
 16
 
  75150744
 
  75150880
 
  ENSEMBL   UCSC 
 
  137
 
  6
 
 15358
 
 tss
 
 0.330312
 
 0.2452144
 
 Not queried
 
  1.3008566
 
       NA
 
           NA
  
 
  
 16
 
  75150456
 
  75150880
 
  ENSEMBL   UCSC 
 
  425
 
  8
 
 16096
 
 island
 
 0.330312
 
 0.2878194
 
 Not queried
 
  1.3008566
 
       NA
 
           NA
  
 
  
 13
 
  36920660
 
  36921174
 
  ENSEMBL   UCSC 
 
  515
 
 12
 
 11394
 
 tss
 
 0.003278
 
 0.0061448
 
 Not queried
 
  1.2999576
 
       NA
 
           NA
  
 
  
 1
 
 248903300
 
 248903467
 
  ENSEMBL   UCSC 
 
  168
 
  4
 
 2373
 
 tss
 
 0.879132
 
 0.7571621
 
 Not queried
 
  1.2998799
 
       NA
 
           NA
  
 
  
 19
 
   2302783
 
   2303032
 
  ENSEMBL   UCSC 
 
  250
 
  4
 
 19686
 
 gene;island
 
 0.038056
 
 0.0635694
 
 Not queried
 
  1.2996395
 
       NA
 
           NA
  
 
  
 11
 
 133994642
 
 133995182
 
  ENSEMBL   UCSC 
 
  541
 
  5
 
 6989
 
 gene;island
 
 0.048199
 
 0.2153963
 
 Not queried
 
  1.2994018
 
       NA
 
           NA
  
 
  
 17
 
     52763
 
     52989
 
  ENSEMBL   UCSC 
 
  227
 
  4
 
 17784
 
 island
 
 0.666618
 
 0.7189973
 
 Not queried
 
  1.2992223
 
       NA
 
           NA
  
 
  
 5
 
  65892993
 
  65893538
 
  ENSEMBL   UCSC 
 
  546
 
  5
 
 31635
 
 gene;island
 
 0.026571
 
 0.0120840
 
 Not queried
 
  1.2991809
 
       NA
 
           NA
  
 
  
 13
 
 113106260
 
 113106473
 
  ENSEMBL   UCSC 
 
  214
 
  4
 
 11921
 
 island
 
 0.549208
 
 0.7485449
 
 Not queried
 
  1.2989946
 
       NA
 
           NA
  
 
  
 5
 
    958808
 
    959141
 
  ENSEMBL   UCSC 
 
  334
 
  4
 
 32599
 
 island
 
 0.909657
 
 0.7061104
 
 Not queried
 
  1.2988680
 
       NA
 
           NA
  
 
  
 8
 
  21916635
 
  21917015
 
  ENSEMBL   UCSC 
 
  381
 
  5
 
 39225
 
 gene;island
 
 0.449214
 
 0.4423953
 
 Not queried
 
  1.2988324
 
       NA
 
           NA
  
 
  
 8
 
   9911482
 
   9911765
 
  ENSEMBL   UCSC 
 
  284
 
  9
 
 39546
 
 tss;island
 
 0.004466
 
 0.0073731
 
 Not queried
 
  1.2982563
 
       NA
 
           NA
  
 
  
 11
 
  58731357
 
  58731634
 
  ENSEMBL   UCSC 
 
  278
 
  4
 
 8299
 
 island
 
 0.891939
 
 0.9420770
 
 Not queried
 
  1.2982469
 
       NA
 
           NA
  
 
  
 2
 
 106886503
 
 106886729
 
  ENSEMBL   UCSC 
 
  227
 
  4
 
 24591
 
 island
 
 0.010814
 
 0.0239214
 
 Not queried
 
  1.2979385
 
       NA
 
           NA
  
 
  
 1
 
  28286348
 
  28286411
 
  ENSEMBL   UCSC 
 
   64
 
  4
 
 1423
 
 tss;island
 
 0.005072
 
 0.0202499
 
 Not queried
 
  1.2979344
 
       NA
 
           NA
  
 
  
 10
 
  93805441
 
  93805870
 
  ENSEMBL   UCSC 
 
  430
 
  5
 
 5936
 
 island
 
 0.902526
 
 0.9593377
 
 Not queried
 
  1.2978198
 
       NA
 
           NA
  
 
  
 10
 
 134756164
 
 134756707
 
  ENSEMBL   UCSC 
 
  544
 
  6
 
 5451
 
 tss;island
 
 0.145250
 
 0.0756264
 
 Not queried
 
  1.2977950
 
       NA
 
           NA
  
 
  
 7
 
  96634480
 
  96634914
 
  ENSEMBL   UCSC 
 
  435
 
  5
 
 37102
 
 gene;tss;island
 
 0.128163
 
 0.1515206
 
 Not queried
 
  1.2975245
 
       NA
 
           NA
  
 
  
 6
 
  18264719
 
  18264854
 
  ENSEMBL   UCSC 
 
  136
 
  4
 
 35480
 
 island
 
 0.010691
 
 0.0073708
 
 Not queried
 
  1.2975089
 
       NA
 
           NA
  
 
  
 19
 
  48896922
 
  48897279
 
  ENSEMBL   UCSC 
 
  358
 
  6
 
 20914
 
 tss;island
 
 0.004917
 
 0.0120484
 
 Not queried
 
  1.2974305
 
       NA
 
           NA
  
 
  
 2
 
 170220773
 
 170221103
 
  ENSEMBL   UCSC 
 
  331
 
  4
 
 24838
 
 island
 
 0.011200
 
 0.0057037
 
 Not queried
 
  1.2968992
 
       NA
 
           NA
  
 
  
 5
 
  77072222
 
  77072483
 
  ENSEMBL   UCSC 
 
  262
 
  5
 
 32139
 
 tss
 
 0.016400
 
 0.0099781
 
 Not queried
 
  1.2968315
 
       NA
 
           NA
  
 
  
 2
 
  87088796
 
  87088843
 
  ENSEMBL   UCSC 
 
   48
 
  4
 
 22773
 
 gene;island
 
 0.055423
 
 0.1848878
 
 Not queried
 
  1.2968108
 
       NA
 
           NA
  
 
  
 11
 
  18727552
 
  18727949
 
  ENSEMBL   UCSC 
 
  398
 
  4
 
 6532
 
 gene;island
 
 0.038851
 
 0.0306607
 
 Not queried
 
  1.2962371
 
       NA
 
           NA
  
 
  
 12
 
  57588031
 
  57588350
 
  ENSEMBL   UCSC 
 
  320
 
  8
 
 9187
 
 gene
 
 0.471596
 
 0.7082008
 
 Not queried
 
  1.2960915
 
       NA
 
           NA
  
 
  
 1
 
 248020331
 
 248020436
 
  ENSEMBL   UCSC 
 
  106
 
  4
 
 2371
 
 tss
 
 0.054654
 
 0.0666422
 
 Not queried
 
  1.2955261
 
       NA
 
           NA
  
 
  
 4
 
 174255082
 
 174255105
 
  ENSEMBL   UCSC 
 
   24
 
  4
 
 30178
 
 gene;tss;island
 
 0.010235
 
 0.0078656
 
 Not queried
 
  1.2953104
 
       NA
 
           NA
  
 
  
 1
 
 247275326
 
 247276096
 
  ENSEMBL   UCSC 
 
  771
 
 10
 
 4414
 
 island
 
 0.098803
 
 0.3393392
 
 Not queried
 
  1.2952035
 
       NA
 
           NA
  
 
  
 19
 
  31640642
 
  31640711
 
  ENSEMBL   UCSC 
 
   70
 
  5
 
 20673
 
 tss
 
 0.940422
 
 0.9747974
 
 Not queried
 
  1.2950194
 
       NA
 
           NA
  
 
  
 7
 
  47694695
 
  47694837
 
  ENSEMBL   UCSC 
 
  143
 
  5
 
 37589
 
 tss
 
 0.195620
 
 0.3368110
 
 Not queried
 
  1.2948935
 
       NA
 
           NA
  
 
  
 19
 
  54927528
 
  54928185
 
  ENSEMBL   UCSC 
 
  658
 
  5
 
 20221
 
 gene;island
 
 0.820410
 
 0.1462263
 
 Not queried
 
  1.2946039
 
       NA
 
           NA
  
 
  
 5
 
  35617687
 
  35617969
 
  ENSEMBL   UCSC 
 
  283
 
  8
 
 32015
 
 tss
 
 0.153203
 
 0.0814606
 
 Not queried
 
  1.2943207
 
       NA
 
           NA
  
 
  
 8
 
  11561283
 
  11561620
 
  ENSEMBL   UCSC 
 
  338
 
  9
 
 39553
 
 tss
 
 0.118913
 
 0.0444367
 
 Not queried
 
  1.2939269
 
       NA
 
           NA
  
 
  
 2
 
 171570700
 
 171571151
 
  ENSEMBL   UCSC 
 
  452
 
  6
 
 23708
 
 tss;island
 
 0.045220
 
 0.0271420
 
 Not queried
 
  1.2936643
 
       NA
 
           NA
  
 
  
 3
 
  51534186
 
  51534298
 
  ENSEMBL   UCSC 
 
  113
 
  4
 
 28364
 
 tss
 
 0.016946
 
 0.0142512
 
 Not queried
 
  1.2932588
 
       NA
 
           NA
  
 
  
 3
 
  51533805
 
  51534298
 
  ENSEMBL   UCSC 
 
  494
 
  6
 
 29167
 
 island
 
 0.007695
 
 0.0050141
 
 Not queried
 
  1.2932588
 
       NA
 
           NA
  
 
  
 13
 
  36872048
 
  36872346
 
  ENSEMBL   UCSC 
 
  299
 
  8
 
 11393
 
 tss
 
 0.081248
 
 0.4114969
 
 Not queried
 
  1.2928969
 
       NA
 
           NA
  
 
  
 19
 
  14359671
 
  14359902
 
  ENSEMBL   UCSC 
 
  232
 
  4
 
 21677
 
 island
 
 0.007403
 
 0.0137578
 
 Not queried
 
  1.2927982
 
       NA
 
           NA
  
 
  
 10
 
  94455543
 
  94455895
 
  ENSEMBL   UCSC 
 
  353
 
  4
 
 5946
 
 island
 
 0.986903
 
 0.9701428
 
 Not queried
 
  1.2927105
 
       NA
 
           NA
  
 
  
 14
 
  61788314
 
  61788345
 
  ENSEMBL   UCSC 
 
   32
 
  4
 
 12443
 
 tss;island
 
 0.006177
 
 0.0080667
 
 Not queried
 
  1.2926806
 
       NA
 
           NA
  
 
  
 20
 
  45523098
 
  45523294
 
  ENSEMBL   UCSC 
 
  197
 
  5
 
 26222
 
 island
 
 0.004708
 
 0.0195857
 
 Not queried
 
  1.2925681
 
       NA
 
           NA
  
 
  
 12
 
  58012960
 
  58013687
 
  ENSEMBL   UCSC 
 
  728
 
 20
 
 9822
 
 tss;island
 
 0.073304
 
 0.0491217
 
 Not queried
 
  1.2924078
 
       NA
 
           NA
  
 
  
 11
 
  10326549
 
  10326589
 
  ENSEMBL   UCSC 
 
   41
 
  6
 
 7143
 
 tss
 
 0.006437
 
 0.0116519
 
 Not queried
 
  1.2923700
 
       NA
 
           NA
  
 
  
 19
 
  55851237
 
  55851365
 
  ENSEMBL   UCSC 
 
  129
 
  4
 
 20227
 
 gene
 
 0.006871
 
 0.0067002
 
 Not queried
 
  1.2923056
 
       NA
 
           NA
  
 
  
 12
 
  81471867
 
  81472177
 
  ENSEMBL   UCSC 
 
  311
 
  6
 
 9228
 
 gene
 
 0.116654
 
 0.2804903
 
 Not queried
 
  1.2922905
 
       NA
 
           NA
  
 
  
 11
 
 124932892
 
 124933200
 
  ENSEMBL   UCSC 
 
  309
 
  6
 
 8921
 
 island
 
 0.004759
 
 0.0041671
 
 Not queried
 
  1.2922752
 
       NA
 
           NA
  
 
  
 17
 
  46805785
 
  46806056
 
  ENSEMBL   UCSC 
 
  272
 
  4
 
 16666
 
 gene
 
 0.052219
 
 0.0518192
 
 Not queried
 
  1.2922221
 
       NA
 
           NA
  
 
  
 17
 
  76921829
 
  76922138
 
  ENSEMBL   UCSC 
 
  310
 
  4
 
 17708
 
 tss;island
 
 0.011566
 
 0.0220153
 
 Not queried
 
  1.2919359
 
       NA
 
           NA
  
 
  
 6
 
  28501816
 
  28501916
 
  ENSEMBL   UCSC 
 
  101
 
  5
 
 33636
 
 gene
 
 0.605204
 
 0.9038194
 
 Not queried
 
  1.2919283
 
       NA
 
           NA
  
 
  
 15
 
  93633145
 
  93633408
 
  ENSEMBL   UCSC 
 
  264
 
  5
 
 13916
 
 tss;island
 
 0.020160
 
 0.0090888
 
 Not queried
 
  1.2918574
 
       NA
 
           NA
  
 
  
 12
 
 131303478
 
 131303774
 
  ENSEMBL   UCSC 
 
  297
 
  4
 
 9419
 
 gene;island
 
 0.045161
 
 0.0184454
 
 Not queried
 
  1.2914381
 
       NA
 
           NA
  
 
  
 15
 
  83240578
 
  83240791
 
  ENSEMBL   UCSC 
 
  214
 
  6
 
 13857
 
 tss
 
 0.038683
 
 0.1415229
 
 Not queried
 
  1.2912436
 
       NA
 
           NA
  
 
  
 11
 
  47587211
 
  47587672
 
  ENSEMBL   UCSC 
 
  462
 
  4
 
 6609
 
 gene;island
 
 0.010474
 
 0.0104605
 
 Not queried
 
  1.2910523
 
       NA
 
           NA
  
 
  
 2
 
 200323017
 
 200323432
 
  ENSEMBL   UCSC 
 
  416
 
  5
 
 22974
 
 gene;island
 
 0.109492
 
 0.0210709
 
 Not queried
 
  1.2905027
 
       NA
 
           NA
  
 
  
 12
 
  64062384
 
  64062724
 
  ENSEMBL   UCSC 
 
  341
 
  8
 
 9837
 
 tss
 
 0.027065
 
 0.1296690
 
 Not queried
 
  1.2899412
 
       NA
 
           NA
  
 
  
 11
 
  16628281
 
  16628944
 
  ENSEMBL   UCSC 
 
  664
 
  6
 
 8089
 
 island
 
 0.047774
 
 0.0436243
 
 Not queried
 
  1.2898773
 
       NA
 
           NA
  
 
  
 6
 
  46702683
 
  46703670
 
  ENSEMBL   UCSC 
 
  988
 
 19
 
 36189
 
 island
 
 0.008609
 
 0.0717289
 
 Not queried
 
  1.2897707
 
       NA
 
           NA
  
 
  
 5
 
 180646608
 
 180646976
 
  ENSEMBL   UCSC 
 
  369
 
  4
 
 33521
 
 island
 
 0.809825
 
 0.9561576
 
 Not queried
 
  1.2897274
 
       NA
 
           NA
  
 
  
 17
 
  35294472
 
  35294491
 
  ENSEMBL   UCSC 
 
   20
 
  4
 
 17284
 
 tss
 
 0.001855
 
 0.0030670
 
 Not queried
 
  1.2895130
 
       NA
 
           NA
  
 
  
 12
 
  21680220
 
  21681006
 
  ENSEMBL   UCSC 
 
  787
 
  8
 
 9079
 
 gene;island
 
 0.019122
 
 0.0205884
 
 Not queried
 
  1.2891929
 
       NA
 
           NA
  
 
  
 16
 
  73126608
 
  73127100
 
  ENSEMBL   UCSC 
 
  493
 
  5
 
 14838
 
 gene
 
 0.638450
 
 0.9269593
 
 Not queried
 
  1.2884466
 
       NA
 
           NA
  
 
  
 6
 
 127835990
 
 127836159
 
  ENSEMBL   UCSC 
 
  170
 
  4
 
 34471
 
 gene;island
 
 0.095303
 
 0.2199420
 
 Not queried
 
  1.2881253
 
       NA
 
           NA
  
 
  
 3
 
  46853850
 
  46854311
 
  ENSEMBL   UCSC 
 
  462
 
  7
 
 29061
 
 island
 
 0.104187
 
 0.0602310
 
 Not queried
 
  1.2880843
 
       NA
 
           NA
  
 
  
 16
 
    967929
 
    968265
 
  ENSEMBL   UCSC 
 
  337
 
  6
 
 14554
 
 gene;island
 
 0.868445
 
 0.9433354
 
 Not queried
 
  1.2880071
 
       NA
 
           NA
  
 
  
 21
 
  34100294
 
  34100315
 
  ENSEMBL   UCSC 
 
   22
 
  5
 
 26400
 
 gene
 
 0.004919
 
 0.0054017
 
 Not queried
 
  1.2879188
 
       NA
 
           NA
  
 
  
 1
 
 150241550
 
 150241941
 
  ENSEMBL   UCSC 
 
  392
 
  8
 
 1904
 
 tss
 
 0.005827
 
 0.0051036
 
 Not queried
 
  1.2877572
 
       NA
 
           NA
  
 
  
 21
 
  46221599
 
  46221955
 
  ENSEMBL   UCSC 
 
  357
 
  6
 
 26740
 
 island
 
 0.005880
 
 0.0056463
 
 Not queried
 
  1.2870216
 
       NA
 
           NA
  
 
  
 2
 
 172950219
 
 172950626
 
  ENSEMBL   UCSC 
 
  408
 
  4
 
 22922
 
 gene
 
 0.057218
 
 0.0289995
 
 Not queried
 
  1.2869389
 
       NA
 
           NA
  
 
  
 15
 
  91537654
 
  91538344
 
  ENSEMBL   UCSC 
 
  691
 
 10
 
 14465
 
 island
 
 0.002983
 
 0.0094332
 
 Not queried
 
  1.2865037
 
       NA
 
           NA
  
 
  
 3
 
  48130799
 
  48130927
 
  ENSEMBL   UCSC 
 
  129
 
  6
 
 28292
 
 tss;island
 
 0.005466
 
 0.0021472
 
 Not queried
 
  1.2863968
 
       NA
 
           NA
  
 
  
 4
 
   6303534
 
   6304042
 
  ENSEMBL   UCSC 
 
  509
 
  4
 
 29927
 
 gene;island
 
 0.742600
 
 0.8110316
 
 Not queried
 
  1.2861498
 
       NA
 
           NA
  
 
  
 16
 
  69220667
 
  69220955
 
  ENSEMBL   UCSC 
 
  289
 
  4
 
 15328
 
 tss;island
 
 0.013134
 
 0.0061351
 
 Not queried
 
  1.2860920
 
       NA
 
           NA
  
 
  
 4
 
 177190277
 
 177190421
 
  ENSEMBL   UCSC 
 
  145
 
  4
 
 30689
 
 tss
 
 0.857065
 
 0.9331341
 
 Not queried
 
  1.2860853
 
       NA
 
           NA
  
 
  
 11
 
  70917159
 
  70917533
 
  ENSEMBL   UCSC 
 
  375
 
  4
 
 6800
 
 gene
 
 0.565979
 
 0.5154158
 
 Not queried
 
  1.2860779
 
       NA
 
           NA
  
 
  
 15
 
  90456194
 
  90456686
 
  ENSEMBL   UCSC 
 
  493
 
  6
 
 14445
 
 island
 
 0.033863
 
 0.2408894
 
 Not queried
 
  1.2858715
 
       NA
 
           NA
  
 
  
 15
 
  41624609
 
  41624793
 
  ENSEMBL   UCSC 
 
  185
 
  4
 
 13302
 
 gene
 
 0.005718
 
 0.0041467
 
 Not queried
 
  1.2856173
 
       NA
 
           NA
  
 
  
 19
 
  36485938
 
  36485972
 
  ENSEMBL   UCSC 
 
   35
 
  4
 
 20719
 
 tss
 
 0.005421
 
 0.0048321
 
 Not queried
 
  1.2854457
 
       NA
 
           NA
  
 
  
 5
 
    537349
 
    537906
 
  ENSEMBL   UCSC 
 
  558
 
  7
 
 32589
 
 island
 
 0.632787
 
 0.8459189
 
 Not queried
 
  1.2852634
 
       NA
 
           NA
  
 
  
 8
 
  49293379
 
  49293600
 
  ENSEMBL   UCSC 
 
  222
 
  4
 
 40282
 
 island
 
 0.034516
 
 0.1120132
 
 Not queried
 
  1.2852568
 
       NA
 
           NA
  
 
  
 1
 
 220921855
 
 220922217
 
  ENSEMBL   UCSC 
 
  363
 
  6
 
 1020
 
 gene
 
 0.324752
 
 0.1574340
 
 Not queried
 
  1.2842917
 
       NA
 
           NA
  
 
  
 16
 
  88765071
 
  88765295
 
  ENSEMBL   UCSC 
 
  225
 
  4
 
 14913
 
 gene;island
 
 0.922480
 
 0.7415412
 
 Not queried
 
  1.2841669
 
       NA
 
           NA
  
 
  
 2
 
  29337946
 
  29338432
 
  ENSEMBL   UCSC 
 
  487
 
 10
 
 24198
 
 island
 
 0.015844
 
 0.0561077
 
 Not queried
 
  1.2841507
 
       NA
 
           NA
  
 
  
 4
 
  76555772
 
  76556042
 
  ENSEMBL   UCSC 
 
  271
 
  9
 
 30439
 
 tss
 
 0.012308
 
 0.0969098
 
 Not queried
 
  1.2840740
 
       NA
 
           NA
  
 
  
 4
 
 114681544
 
 114681880
 
  ENSEMBL   UCSC 
 
  337
 
  4
 
 30105
 
 gene;island
 
 0.005561
 
 0.0025043
 
 Not queried
 
  1.2840328
 
       NA
 
           NA
  
 
  
 17
 
  78152051
 
  78152466
 
  ENSEMBL   UCSC 
 
  416
 
  6
 
 18863
 
 island
 
 0.847597
 
 0.9079554
 
 Not queried
 
  1.2839295
 
       NA
 
           NA
  
 
  
 8
 
  21966036
 
  21966491
 
  ENSEMBL   UCSC 
 
  456
 
  4
 
 39227
 
 gene;island
 
 0.133288
 
 0.0595007
 
 Not queried
 
  1.2831335
 
       NA
 
           NA
  
 
  
 19
 
   1271019
 
   1271371
 
  ENSEMBL   UCSC 
 
  353
 
  7
 
 19651
 
 gene;tss;island
 
 0.964196
 
 0.9389582
 
 Not queried
 
  1.2828765
 
       NA
 
           NA
  
 
  
 17
 
   5402883
 
   5403516
 
  ENSEMBL   UCSC 
 
  634
 
  6
 
 16368
 
 gene;island
 
 0.653371
 
 0.4992593
 
 Not queried
 
  1.2826340
 
       NA
 
           NA
  
 
  
 4
 
  41646293
 
  41646672
 
  ENSEMBL   UCSC 
 
  380
 
  4
 
 29991
 
 gene;island
 
 0.114876
 
 0.2246476
 
 Not queried
 
  1.2826001
 
       NA
 
           NA
  
 
  
 11
 
  34535085
 
  34535281
 
  ENSEMBL   UCSC 
 
  197
 
  4
 
 6581
 
 gene
 
 0.646490
 
 0.7771035
 
 Not queried
 
  1.2823146
 
       NA
 
           NA
  
 
  
 1
 
 153030514
 
 153030751
 
  ENSEMBL   UCSC 
 
  238
 
  4
 
 1939
 
 tss
 
 0.186058
 
 0.2968122
 
 Not queried
 
  1.2821938
 
       NA
 
           NA
  
 
  
 12
 
 132689907
 
 132690782
 
  ENSEMBL   UCSC 
 
  876
 
 11
 
 9434
 
 gene;island
 
 0.138525
 
 0.0434100
 
 Not queried
 
  1.2820990
 
       NA
 
           NA
  
 
  
 5
 
    500202
 
    500565
 
  ENSEMBL   UCSC 
 
  364
 
  5
 
 31509
 
 gene;island
 
 0.471294
 
 0.7507971
 
 Not queried
 
  1.2819191
 
       NA
 
           NA
  
 
  
 4
 
    995849
 
    996280
 
  ENSEMBL   UCSC 
 
  432
 
  5
 
 29855
 
 gene;island
 
 0.073054
 
 0.1332885
 
 Not queried
 
  1.2818667
 
       NA
 
           NA
  
 
  
 6
 
  30865889
 
  30866037
 
  ENSEMBL   UCSC 
 
  149
 
  6
 
 33830
 
 gene
 
 0.952136
 
 0.8847664
 
 Not queried
 
  1.2817230
 
       NA
 
           NA
  
 
  
 4
 
  17513197
 
  17513834
 
  ENSEMBL   UCSC 
 
  638
 
  5
 
 29961
 
 gene
 
 0.025965
 
 0.0145854
 
 Not queried
 
  1.2814408
 
       NA
 
           NA
  
 
  
 6
 
  13574842
 
  13574897
 
  ENSEMBL   UCSC 
 
   56
 
  4
 
 33577
 
 gene;island
 
 0.004979
 
 0.0031170
 
 Not queried
 
  1.2812669
 
       NA
 
           NA
  
 
  
 7
 
  12443880
 
  12444290
 
  ENSEMBL   UCSC 
 
  411
 
  7
 
 37444
 
 tss
 
 0.432169
 
 0.1090684
 
 Not queried
 
  1.2811744
 
       NA
 
           NA
  
 
  
 1
 
  26516478
 
  26516623
 
  ENSEMBL   UCSC 
 
  146
 
  4
 
 1386
 
 tss
 
 0.455327
 
 0.5778645
 
 Not queried
 
  1.2808707
 
       NA
 
           NA
  
 
  
 1
 
   3239916
 
   3240227
 
  ENSEMBL   UCSC 
 
  312
 
  4
 
 120
 
 gene;island
 
 0.248321
 
 0.0695345
 
 Not queried
 
  1.2803833
 
       NA
 
           NA
  
 
  
 7
 
 100612725
 
 100612891
 
  ENSEMBL   UCSC 
 
  167
 
  4
 
 37796
 
 tss
 
 0.245627
 
 0.4309975
 
 Not queried
 
  1.2800388
 
       NA
 
           NA
  
 
  
 1
 
 207669003
 
 207669334
 
  ENSEMBL   UCSC 
 
  332
 
  5
 
 2223
 
 tss;island
 
 0.439358
 
 0.5974477
 
 Not queried
 
  1.2798919
 
       NA
 
           NA
  
 
  
 16
 
  56651283
 
  56651456
 
  ENSEMBL   UCSC 
 
  174
 
  4
 
 15948
 
 island
 
 0.033188
 
 0.0578641
 
 Not queried
 
  1.2798415
 
       NA
 
           NA
  
 
  
 20
 
  61718852
 
  61719188
 
  ENSEMBL   UCSC 
 
  337
 
  5
 
 26341
 
 island
 
 0.495881
 
 0.8187961
 
 Not queried
 
  1.2797347
 
       NA
 
           NA
  
 
  
 8
 
  23386415
 
  23386623
 
  ENSEMBL   UCSC 
 
  209
 
  4
 
 39242
 
 gene;island
 
 0.004014
 
 0.0034780
 
 Not queried
 
  1.2793436
 
       NA
 
           NA
  
 
  
 2
 
 152956034
 
 152956135
 
  ENSEMBL   UCSC 
 
  102
 
  4
 
 23663
 
 tss;island
 
 0.007837
 
 0.0020859
 
 Not queried
 
  1.2790649
 
       NA
 
           NA
  
 
  
 8
 
  28747299
 
  28747613
 
  ENSEMBL   UCSC 
 
  315
 
  4
 
 39265
 
 gene
 
 0.005882
 
 0.0029833
 
 Not queried
 
  1.2789222
 
       NA
 
           NA
  
 
  
 1
 
   1177886
 
   1178245
 
  ENSEMBL   UCSC 
 
  360
 
  5
 
 23
 
 gene
 
 0.244397
 
 0.4763535
 
 Not queried
 
  1.2788344
 
       NA
 
           NA
  
 
  
 5
 
  37839829
 
  37840158
 
  ENSEMBL   UCSC 
 
  330
 
  7
 
 32027
 
 tss
 
 0.015959
 
 0.1125748
 
 Not queried
 
  1.2785674
 
       NA
 
           NA
  
 
  
 5
 
  37839684
 
  37840158
 
  ENSEMBL   UCSC 
 
  475
 
  9
 
 32741
 
 island
 
 0.019726
 
 0.1125748
 
 Not queried
 
  1.2785674
 
       NA
 
           NA
  
 
  
 15
 
  93632611
 
  93632896
 
  ENSEMBL   UCSC 
 
  286
 
  5
 
 13915
 
 tss;island
 
 0.022262
 
 0.0067949
 
 Not queried
 
  1.2783848
 
       NA
 
           NA
  
 
  
 11
 
  73020351
 
  73020729
 
  ENSEMBL   UCSC 
 
  379
 
  5
 
 6821
 
 gene;island
 
 0.060384
 
 0.2657776
 
 Not queried
 
  1.2782107
 
       NA
 
           NA
  
 
  
 11
 
  26353484
 
  26353630
 
  ENSEMBL   UCSC 
 
  147
 
  5
 
 7206
 
 tss
 
 0.028610
 
 0.0475895
 
 Not queried
 
  1.2780817
 
       NA
 
           NA
  
 
  
 15
 
  84047906
 
  84048308
 
  ENSEMBL   UCSC 
 
  403
 
  5
 
 14399
 
 island
 
 0.029266
 
 0.0727292
 
 Not queried
 
  1.2779577
 
       NA
 
           NA
  
 
  
 19
 
  55866087
 
  55866412
 
  ENSEMBL   UCSC 
 
  326
 
  9
 
 22419
 
 island
 
 0.144061
 
 0.0171873
 
 Not queried
 
  1.2779420
 
       NA
 
           NA
  
 
  
 4
 
   4228571
 
   4228650
 
  ENSEMBL   UCSC 
 
   80
 
  4
 
 30857
 
 island
 
 0.569863
 
 0.7356416
 
 Not queried
 
  1.2778451
 
       NA
 
           NA
  
 
  
 19
 
  57741988
 
  57742444
 
  ENSEMBL   UCSC 
 
  457
 
 10
 
 22461
 
 island
 
 0.946134
 
 0.8435240
 
 Not queried
 
  1.2775661
 
       NA
 
           NA
  
 
  
 15
 
  65281804
 
  65281963
 
  ENSEMBL   UCSC 
 
  160
 
  6
 
 13376
 
 gene;island
 
 0.003749
 
 0.0085315
 
 Not queried
 
  1.2770393
 
       NA
 
           NA
  
 
  
 15
 
  83776769
 
  83777082
 
  ENSEMBL   UCSC 
 
  314
 
  5
 
 13457
 
 gene;island
 
 0.034060
 
 0.0599370
 
 Not queried
 
  1.2769105
 
       NA
 
           NA
  
 
  
 2
 
  74347215
 
  74347776
 
  ENSEMBL   UCSC 
 
  562
 
  5
 
 24424
 
 island
 
 0.007168
 
 0.0245790
 
 Not queried
 
  1.2763600
 
       NA
 
           NA
  
 
  
 16
 
  84853665
 
  84853871
 
  ENSEMBL   UCSC 
 
  207
 
  4
 
 14872
 
 gene
 
 0.100059
 
 0.0467605
 
 Not queried
 
  1.2761849
 
       NA
 
           NA
  
 
  
 22
 
  17178585
 
  17178751
 
  ENSEMBL   UCSC 
 
  167
 
  4
 
 27268
 
 island
 
 0.584846
 
 0.8568167
 
 Not queried
 
  1.2759665
 
       NA
 
           NA
  
 
  
 8
 
 110374294
 
 110374675
 
  ENSEMBL   UCSC 
 
  382
 
  6
 
 39870
 
 tss
 
 0.051167
 
 0.0409011
 
 Not queried
 
  1.2759571
 
       NA
 
           NA
  
 
  
 8
 
 110374294
 
 110374866
 
  ENSEMBL   UCSC 
 
  573
 
  8
 
 40536
 
 island
 
 0.051167
 
 0.0426565
 
 Not queried
 
  1.2759571
 
       NA
 
           NA
  
 
  
 7
 
  19812479
 
  19813525
 
  ENSEMBL   UCSC 
 
 1047
 
 15
 
 37463
 
 tss
 
 0.498295
 
 0.0650204
 
 Not queried
 
  1.2758341
 
       NA
 
           NA
  
 
  
 4
 
 165118829
 
 165118938
 
  ENSEMBL   UCSC 
 
  110
 
  4
 
 30170
 
 gene
 
 0.311475
 
 0.4095756
 
 Not queried
 
  1.2757709
 
       NA
 
           NA
  
 
  
 2
 
  97535892
 
  97536287
 
  ENSEMBL   UCSC 
 
  396
 
  7
 
 23526
 
 tss;island
 
 0.010595
 
 0.0177790
 
 Not queried
 
  1.2748671
 
       NA
 
           NA
  
 
  
 17
 
  79099711
 
  79100287
 
  ENSEMBL   UCSC 
 
  577
 
 10
 
 16894
 
 gene;tss;island
 
 0.683339
 
 0.8764086
 
 Not queried
 
  1.2742055
 
       NA
 
           NA
  
 
  
 19
 
  44716462
 
  44716642
 
  ENSEMBL   UCSC 
 
  181
 
  5
 
 20846
 
 tss
 
 0.001020
 
 0.0010913
 
 Not queried
 
  1.2737213
 
       NA
 
           NA
  
 
  
 19
 
  58609730
 
  58610131
 
  ENSEMBL   UCSC 
 
  402
 
 10
 
 21133
 
 tss
 
 0.075770
 
 0.0296047
 
 Not queried
 
  1.2734797
 
       NA
 
           NA
  
 
  
 10
 
  99329961
 
  99330203
 
  ENSEMBL   UCSC 
 
  243
 
  5
 
 4697
 
 gene;island
 
 0.674366
 
 0.7719619
 
 Not queried
 
  1.2729824
 
       NA
 
           NA
  
 
  
 6
 
  32141584
 
  32142236
 
  ENSEMBL   UCSC 
 
  653
 
 10
 
 34070
 
 gene
 
 0.922316
 
 0.9755438
 
 Not queried
 
  1.2729738
 
       NA
 
           NA
  
 
  
 11
 
  22454152
 
  22454661
 
  ENSEMBL   UCSC 
 
  510
 
  6
 
 8134
 
 island
 
 0.258194
 
 0.2985683
 
 Not queried
 
  1.2727785
 
       NA
 
           NA
  
 
  
 3
 
  69812798
 
  69812973
 
  ENSEMBL   UCSC 
 
  176
 
  4
 
 27855
 
 gene
 
 0.574068
 
 0.7033953
 
 Not queried
 
  1.2727743
 
       NA
 
           NA
  
 
  
 1
 
  36042986
 
  36043084
 
  ENSEMBL   UCSC 
 
   99
 
  4
 
 422
 
 gene;island
 
 0.062399
 
 0.0224075
 
 Not queried
 
  1.2725331
 
       NA
 
           NA
  
 
  
 2
 
 201390524
 
 201390889
 
  ENSEMBL   UCSC 
 
  366
 
  4
 
 25002
 
 island
 
 0.021218
 
 0.0122823
 
 Not queried
 
  1.2723761
 
       NA
 
           NA
  
 
  
 12
 
  56359158
 
  56359450
 
  ENSEMBL   UCSC 
 
  293
 
  4
 
 9171
 
 gene;tss;island
 
 0.827188
 
 0.6408523
 
 Not queried
 
  1.2720948
 
       NA
 
           NA
  
 
  
 7
 
  51096557
 
  51096971
 
  ENSEMBL   UCSC 
 
  415
 
  6
 
 37016
 
 gene
 
 0.169375
 
 0.4066084
 
 Not queried
 
  1.2720288
 
       NA
 
           NA
  
 
  
 19
 
  52004992
 
  52005188
 
  ENSEMBL   UCSC 
 
  197
 
  6
 
 20996
 
 tss
 
 0.040537
 
 0.0108503
 
 Not queried
 
  1.2714224
 
       NA
 
           NA
  
 
  
 16
 
   1110844
 
   1111220
 
  ENSEMBL   UCSC 
 
  377
 
  4
 
 15512
 
 island
 
 0.541150
 
 0.8274649
 
 Not queried
 
  1.2711211
 
       NA
 
           NA
  
 
  
 14
 
  50329613
 
  50329901
 
  ENSEMBL   UCSC 
 
  289
 
  4
 
 12827
 
 island
 
 0.001948
 
 0.0032842
 
 Not queried
 
  1.2707967
 
       NA
 
           NA
  
 
  
 1
 
  63783375
 
  63783576
 
  ENSEMBL   UCSC 
 
  202
 
  4
 
 3340
 
 island
 
 0.040392
 
 0.0663043
 
 Not queried
 
  1.2702351
 
       NA
 
           NA
  
 
  
 6
 
 111408745
 
 111408765
 
  ENSEMBL   UCSC 
 
   21
 
  5
 
 35175
 
 tss
 
 0.022866
 
 0.0122242
 
 Not queried
 
  1.2699517
 
       NA
 
           NA
  
 
  
 3
 
 129275285
 
 129275655
 
  ENSEMBL   UCSC 
 
  371
 
  4
 
 27942
 
 gene
 
 0.686484
 
 0.8603549
 
 Not queried
 
  1.2696638
 
       NA
 
           NA
  
 
  
 1
 
 207082828
 
 207083129
 
  ENSEMBL   UCSC 
 
  302
 
  4
 
 974
 
 gene;island
 
 0.042268
 
 0.0582168
 
 Not queried
 
  1.2696577
 
       NA
 
           NA
  
 
  
 1
 
 117213980
 
 117214228
 
  ENSEMBL   UCSC 
 
  249
 
  4
 
 1865
 
 tss;island
 
 0.954521
 
 0.9117844
 
 Not queried
 
  1.2696148
 
       NA
 
           NA
  
 
  
 17
 
  15164106
 
  15164292
 
  ENSEMBL   UCSC 
 
  187
 
  6
 
 17148
 
 tss
 
 0.008104
 
 0.0132233
 
 Not queried
 
  1.2695835
 
       NA
 
           NA
  
 
  
 11
 
  18034662
 
  18034920
 
  ENSEMBL   UCSC 
 
  259
 
  6
 
 7179
 
 tss
 
 0.001547
 
 0.0062393
 
 Not queried
 
  1.2695736
 
       NA
 
           NA
  
 
  
 2
 
  88752056
 
  88752322
 
  ENSEMBL   UCSC 
 
  267
 
  5
 
 23500
 
 tss;island
 
 0.060853
 
 0.0263503
 
 Not queried
 
  1.2695244
 
       NA
 
           NA
  
 
  
 20
 
  20433062
 
  20433328
 
  ENSEMBL   UCSC 
 
  267
 
  4
 
 25373
 
 gene;island
 
 0.020086
 
 0.0658448
 
 Not queried
 
  1.2693259
 
       NA
 
           NA
  
 
  
 1
 
  34631324
 
  34631613
 
  ENSEMBL   UCSC 
 
  290
 
  4
 
 1475
 
 tss;island
 
 0.046678
 
 0.0644153
 
 Not queried
 
  1.2691105
 
       NA
 
           NA
  
 
  
 11
 
   3186792
 
   3187032
 
  ENSEMBL   UCSC 
 
  241
 
  6
 
 7091
 
 tss;island
 
 0.091625
 
 0.2569861
 
 Not queried
 
  1.2686120
 
       NA
 
           NA
  
 
  
 7
 
  26897597
 
  26897714
 
  ENSEMBL   UCSC 
 
  118
 
  4
 
 36924
 
 gene
 
 0.187948
 
 0.1307368
 
 Not queried
 
  1.2683354
 
       NA
 
           NA
  
 
  
 6
 
  10747564
 
  10747898
 
  ENSEMBL   UCSC 
 
  335
 
  7
 
 34630
 
 tss;island
 
 0.009404
 
 0.0038595
 
 Not queried
 
  1.2681885
 
       NA
 
           NA
  
 
  
 6
 
  43456984
 
  43457274
 
  ENSEMBL   UCSC 
 
  291
 
  4
 
 34348
 
 gene;tss
 
 0.861624
 
 0.7212398
 
 Not queried
 
  1.2677746
 
       NA
 
           NA
  
 
  
 10
 
  46993502
 
  46993586
 
  ENSEMBL   UCSC 
 
   85
 
  4
 
 5718
 
 island
 
 0.193201
 
 0.3415148
 
 Not queried
 
  1.2677057
 
       NA
 
           NA
  
 
  
 15
 
  59042242
 
  59042352
 
  ENSEMBL   UCSC 
 
  111
 
  4
 
 13699
 
 tss;island
 
 0.002843
 
 0.0063003
 
 Not queried
 
  1.2676799
 
       NA
 
           NA
  
 
  
 2
 
  70121099
 
  70121414
 
  ENSEMBL   UCSC 
 
  316
 
  5
 
 22719
 
 gene
 
 0.014236
 
 0.0107133
 
 Not queried
 
  1.2673456
 
       NA
 
           NA
  
 
  
 5
 
 140430569
 
 140430962
 
  ENSEMBL   UCSC 
 
  394
 
  6
 
 32360
 
 tss
 
 0.071169
 
 0.0515777
 
 Not queried
 
  1.2669962
 
       NA
 
           NA
  
 
  
 19
 
  50249464
 
  50249927
 
  ENSEMBL   UCSC 
 
  464
 
  6
 
 20163
 
 gene;island
 
 0.583440
 
 0.1452771
 
 Not queried
 
  1.2662947
 
       NA
 
           NA
  
 
  
 7
 
  36022276
 
  36022841
 
  ENSEMBL   UCSC 
 
  566
 
  5
 
 38413
 
 island
 
 0.521070
 
 0.6809493
 
 Not queried
 
  1.2662042
 
       NA
 
           NA
  
 
  
 22
 
  39712956
 
  39713086
 
  ENSEMBL   UCSC 
 
  131
 
  6
 
 26876
 
 gene;tss;island
 
 0.940931
 
 0.9660798
 
 Not queried
 
  1.2661848
 
       NA
 
           NA
  
 
  
 13
 
  36788776
 
  36789025
 
  ENSEMBL   UCSC 
 
  250
 
  6
 
 11392
 
 tss
 
 0.709435
 
 0.8783295
 
 Not queried
 
  1.2654381
 
       NA
 
           NA
  
 
  
 6
 
  24583477
 
  24583848
 
  ENSEMBL   UCSC 
 
  372
 
  5
 
 33592
 
 gene
 
 0.018560
 
 0.6707686
 
 Not queried
 
  1.2652220
 
       NA
 
           NA
  
 
  
 19
 
  37157565
 
  37157995
 
  ENSEMBL   UCSC 
 
  431
 
 11
 
 21944
 
 island
 
 0.011214
 
 0.0040744
 
 Not queried
 
  1.2651528
 
       NA
 
           NA
  
 
  
 13
 
  96204493
 
  96204917
 
  ENSEMBL   UCSC 
 
  425
 
  9
 
 11502
 
 tss
 
 0.012276
 
 0.0099613
 
 Not queried
 
  1.2650210
 
       NA
 
           NA
  
 
  
 10
 
    327132
 
    327612
 
  ENSEMBL   UCSC 
 
  481
 
  6
 
 4431
 
 gene
 
 0.705579
 
 0.8517476
 
 Not queried
 
  1.2648995
 
       NA
 
           NA
  
 
  
 20
 
  25128681
 
  25128807
 
  ENSEMBL   UCSC 
 
  127
 
  6
 
 25377
 
 gene;tss;island
 
 0.690698
 
 0.8396271
 
 Not queried
 
  1.2646699
 
       NA
 
           NA
  
 
  
 1
 
  13909522
 
  13909821
 
  ENSEMBL   UCSC 
 
  300
 
  4
 
 1260
 
 tss;island
 
 0.254411
 
 0.5506703
 
 Not queried
 
  1.2644525
 
       NA
 
           NA
  
 
  
 14
 
  92788914
 
  92789193
 
  ENSEMBL   UCSC 
 
  280
 
  4
 
 12566
 
 tss;island
 
 0.108476
 
 0.0487073
 
 Not queried
 
  1.2644489
 
       NA
 
           NA
  
 
  
 14
 
 101526485
 
 101527144
 
  ENSEMBL   UCSC 
 
  660
 
 10
 
 12631
 
 tss
 
 0.557662
 
 0.7059332
 
 Not queried
 
  1.2644069
 
       NA
 
           NA
  
 
  
 5
 
 140098174
 
 140098496
 
  ENSEMBL   UCSC 
 
  323
 
  6
 
 32350
 
 tss
 
 0.567148
 
 0.6389778
 
 Not queried
 
  1.2641405
 
       NA
 
           NA
  
 
  
 13
 
  41496623
 
  41496979
 
  ENSEMBL   UCSC 
 
  357
 
  4
 
 11407
 
 tss;island
 
 0.944189
 
 0.9757816
 
 Not queried
 
  1.2632028
 
       NA
 
           NA
  
 
  
 6
 
  32906460
 
  32906923
 
  ENSEMBL   UCSC 
 
  464
 
  7
 
 34162
 
 gene
 
 0.503904
 
 0.6797274
 
 Not queried
 
  1.2626095
 
       NA
 
           NA
  
 
  
 1
 
  36916159
 
  36916744
 
  ENSEMBL   UCSC 
 
  586
 
 11
 
 1497
 
 tss
 
 0.012859
 
 0.0086728
 
 Not queried
 
  1.2619085
 
       NA
 
           NA
  
 
  
 7
 
  19184257
 
  19184555
 
  ENSEMBL   UCSC 
 
  299
 
  4
 
 38286
 
 island
 
 0.218493
 
 0.0511282
 
 Not queried
 
  1.2613785
 
       NA
 
           NA
  
 
  
 11
 
  66045351
 
  66045564
 
  ENSEMBL   UCSC 
 
  214
 
  8
 
 7461
 
 tss
 
 0.003845
 
 0.0055582
 
 Not queried
 
  1.2612785
 
       NA
 
           NA
  
 
  
 16
 
  86370962
 
  86371442
 
  ENSEMBL   UCSC 
 
  481
 
  6
 
 14883
 
 gene
 
 0.376169
 
 0.5686720
 
 Not queried
 
  1.2609621
 
       NA
 
           NA
  
 
  
 10
 
  85954312
 
  85954336
 
  ENSEMBL   UCSC 
 
   25
 
  4
 
 5192
 
 tss;island
 
 0.003886
 
 0.0076335
 
 Not queried
 
  1.2608752
 
       NA
 
           NA
  
 
  
 6
 
  32011506
 
  32011687
 
  ENSEMBL   UCSC 
 
  182
 
  8
 
 34012
 
 gene
 
 0.377549
 
 0.6050996
 
 Not queried
 
  1.2608708
 
       NA
 
           NA
  
 
  
 19
 
  45146828
 
  45147078
 
  ENSEMBL   UCSC 
 
  251
 
  7
 
 20851
 
 tss;island
 
 0.042562
 
 0.1145653
 
 Not queried
 
  1.2607091
 
       NA
 
           NA
  
 
  
 1
 
   4842472
 
   4842898
 
  ENSEMBL   UCSC 
 
  427
 
  4
 
 154
 
 gene
 
 0.495621
 
 0.7156746
 
 Not queried
 
  1.2602375
 
       NA
 
           NA
  
 
  
 17
 
  62340717
 
  62340798
 
  ENSEMBL   UCSC 
 
   82
 
  4
 
 17581
 
 tss
 
 0.008613
 
 0.0121521
 
 Not queried
 
  1.2600989
 
       NA
 
           NA
  
 
  
 3
 
 131753858
 
 131754273
 
  ENSEMBL   UCSC 
 
  416
 
  6
 
 28596
 
 tss;island
 
 0.005430
 
 0.0133395
 
 Not queried
 
  1.2596659
 
       NA
 
           NA
  
 
  
 6
 
 168936486
 
 168936684
 
  ENSEMBL   UCSC 
 
  199
 
  4
 
 34562
 
 gene;island
 
 0.567102
 
 0.7942233
 
 Not queried
 
  1.2596294
 
       NA
 
           NA
  
 
  
 14
 
  57046206
 
  57046475
 
  ENSEMBL   UCSC 
 
  270
 
  7
 
 12417
 
 tss;island
 
 0.004536
 
 0.0085866
 
 Not queried
 
  1.2592729
 
       NA
 
           NA
  
 
  
 1
 
   6639570
 
   6639959
 
  ENSEMBL   UCSC 
 
  390
 
  8
 
 1211
 
 tss
 
 0.005472
 
 0.0246448
 
 Not queried
 
  1.2591945
 
       NA
 
           NA
  
 
  
 17
 
   7197878
 
   7198087
 
  ENSEMBL   UCSC 
 
  210
 
  7
 
 17072
 
 tss
 
 0.012469
 
 0.0164607
 
 Not queried
 
  1.2591746
 
       NA
 
           NA
  
 
  
 17
 
   7197775
 
   7198087
 
  ENSEMBL   UCSC 
 
  313
 
  9
 
 17933
 
 island
 
 0.016394
 
 0.0374389
 
 Not queried
 
  1.2591746
 
       NA
 
           NA
  
 
  
 1
 
  20811132
 
  20811408
 
  ENSEMBL   UCSC 
 
  277
 
  4
 
 288
 
 gene;island
 
 0.033842
 
 0.0521244
 
 Not queried
 
  1.2581797
 
       NA
 
           NA
  
 
  
 6
 
  42110740
 
  42111005
 
  ENSEMBL   UCSC 
 
  266
 
  5
 
 34978
 
 tss;island
 
 0.855673
 
 0.6523716
 
 Not queried
 
  1.2580453
 
       NA
 
           NA
  
 
  
 13
 
  80054976
 
  80055241
 
  ENSEMBL   UCSC 
 
  266
 
  8
 
 11491
 
 tss;island
 
 0.003367
 
 0.0032330
 
 Not queried
 
  1.2574003
 
       NA
 
           NA
  
 
  
 3
 
 123679768
 
 123680219
 
  ENSEMBL   UCSC 
 
  452
 
  4
 
 27919
 
 gene;island
 
 0.005993
 
 0.0064332
 
 Not queried
 
  1.2570995
 
       NA
 
           NA
  
 
  
 1
 
 231155682
 
 231155983
 
  ENSEMBL   UCSC 
 
  302
 
 10
 
 2319
 
 tss
 
 0.626114
 
 0.8144372
 
 Not queried
 
  1.2570802
 
       NA
 
           NA
  
 
  
 2
 
   3260576
 
   3261155
 
  ENSEMBL   UCSC 
 
  580
 
  5
 
 22543
 
 gene
 
 0.937791
 
 0.9726677
 
 Not queried
 
  1.2568587
 
       NA
 
           NA
  
 
  
 7
 
  29519297
 
  29519656
 
  ENSEMBL   UCSC 
 
  360
 
  8
 
 36949
 
 gene
 
 0.257595
 
 0.3026092
 
 Not queried
 
  1.2566724
 
       NA
 
           NA
  
 
  
 6
 
  32634245
 
  32634362
 
  ENSEMBL   UCSC 
 
  118
 
  5
 
 34127
 
 gene
 
 0.027184
 
 0.0296564
 
 Not queried
 
  1.2566352
 
       NA
 
           NA
  
 
  
 11
 
  62414022
 
  62414275
 
  ENSEMBL   UCSC 
 
  254
 
  8
 
 8346
 
 island
 
 0.007509
 
 0.0070135
 
 Not queried
 
  1.2565921
 
       NA
 
           NA
  
 
  
 15
 
  25981027
 
  25981391
 
  ENSEMBL   UCSC 
 
  365
 
  5
 
 13259
 
 gene;island
 
 0.700121
 
 0.7620242
 
 Not queried
 
  1.2560566
 
       NA
 
           NA
  
 
  
 2
 
 217363243
 
 217363620
 
  ENSEMBL   UCSC 
 
  378
 
  6
 
 25074
 
 island
 
 0.002479
 
 0.0023898
 
 Not queried
 
  1.2552788
 
       NA
 
           NA
  
 
  
 19
 
  49993125
 
  49993217
 
  ENSEMBL   UCSC 
 
   93
 
  6
 
 20154
 
 gene;tss;island
 
 0.528873
 
 0.7238942
 
 Not queried
 
  1.2551425
 
       NA
 
           NA
  
 
  
 5
 
   1666974
 
   1667258
 
  ENSEMBL   UCSC 
 
  285
 
  4
 
 32639
 
 island
 
 0.049686
 
 0.0180333
 
 Not queried
 
  1.2549720
 
       NA
 
           NA
  
 
  
 22
 
  39529123
 
  39529323
 
  ENSEMBL   UCSC 
 
  201
 
  5
 
 26873
 
 gene
 
 0.798020
 
 0.8482681
 
 Not queried
 
  1.2547265
 
       NA
 
           NA
  
 
  
 4
 
 155413392
 
 155413789
 
  ENSEMBL   UCSC 
 
  398
 
  4
 
 30644
 
 tss;island
 
 0.273146
 
 0.3025205
 
 Not queried
 
  1.2546900
 
       NA
 
           NA
  
 
  
 10
 
  45719751
 
  45720134
 
  ENSEMBL   UCSC 
 
  384
 
  5
 
 5711
 
 island
 
 0.955490
 
 0.9758159
 
 Not queried
 
  1.2543845
 
       NA
 
           NA
  
 
  
 4
 
 122618147
 
 122618170
 
  ENSEMBL   UCSC 
 
   24
 
  4
 
 30571
 
 tss
 
 0.009644
 
 0.0086940
 
 Not queried
 
  1.2543241
 
       NA
 
           NA
  
 
  
 13
 
  36919674
 
  36919960
 
  ENSEMBL   UCSC 
 
  287
 
  5
 
 11130
 
 gene;island
 
 0.004943
 
 0.0193016
 
 Not queried
 
  1.2541094
 
       NA
 
           NA
  
 
  
 1
 
 210407473
 
 210407973
 
  ENSEMBL   UCSC 
 
  501
 
  9
 
 990
 
 gene;tss;island
 
 0.026637
 
 0.0584546
 
 Not queried
 
  1.2540849
 
       NA
 
           NA
  
 
  
 6
 
 170400942
 
 170401305
 
  ENSEMBL   UCSC 
 
  364
 
  4
 
 36678
 
 island
 
 0.570160
 
 0.6683310
 
 Not queried
 
  1.2539835
 
       NA
 
           NA
  
 
  
 7
 
 158059704
 
 158060003
 
  ENSEMBL   UCSC 
 
  300
 
  5
 
 37363
 
 gene;island
 
 0.752380
 
 0.8523115
 
 Not queried
 
  1.2531210
 
       NA
 
           NA
  
 
  
 17
 
  16255560
 
  16255884
 
  ENSEMBL   UCSC 
 
  325
 
  4
 
 16458
 
 gene;island
 
 0.008021
 
 0.0251052
 
 Not queried
 
  1.2530090
 
       NA
 
           NA
  
 
  
 17
 
  46810540
 
  46810934
 
  ENSEMBL   UCSC 
 
  395
 
  6
 
 18505
 
 island
 
 0.028131
 
 0.0957756
 
 Not queried
 
  1.2527965
 
       NA
 
           NA
  
 
  
 19
 
  15051936
 
  15052282
 
  ENSEMBL   UCSC 
 
  347
 
  4
 
 20550
 
 tss
 
 0.722937
 
 0.8588288
 
 Not queried
 
  1.2526072
 
       NA
 
           NA
  
 
  
 10
 
  57390536
 
  57391055
 
  ENSEMBL   UCSC 
 
  520
 
  4
 
 5755
 
 island
 
 0.046593
 
 0.2090382
 
 Not queried
 
  1.2524096
 
       NA
 
           NA
  
 
  
 4
 
   7395388
 
   7395612
 
  ENSEMBL   UCSC 
 
  225
 
  4
 
 29936
 
 gene
 
 0.469019
 
 0.7196175
 
 Not queried
 
  1.2515106
 
       NA
 
           NA
  
 
  
 6
 
  56111812
 
  56112291
 
  ENSEMBL   UCSC 
 
  480
 
  5
 
 34375
 
 gene
 
 0.022972
 
 0.0517557
 
 Not queried
 
  1.2514178
 
       NA
 
           NA
  
 
  
 5
 
 179636283
 
 179636617
 
  ENSEMBL   UCSC 
 
  335
 
  4
 
 32559
 
 tss;island
 
 0.658471
 
 0.5082006
 
 Not queried
 
  1.2511552
 
       NA
 
           NA
  
 
  
 12
 
   2613703
 
   2613948
 
  ENSEMBL   UCSC 
 
  246
 
  5
 
 9003
 
 gene
 
 0.509375
 
 0.6524127
 
 Not queried
 
  1.2507581
 
       NA
 
           NA
  
 
  
 7
 
  98633110
 
  98633538
 
  ENSEMBL   UCSC 
 
  429
 
  4
 
 37122
 
 gene;island
 
 0.977280
 
 0.9717603
 
 Not queried
 
  1.2506983
 
       NA
 
           NA
  
 
  
 6
 
 146919854
 
 146920295
 
  ENSEMBL   UCSC 
 
  442
 
 10
 
 36545
 
 island
 
 0.056812
 
 0.0191021
 
 Not queried
 
  1.2502915
 
       NA
 
           NA
  
 
  
 1
 
  17085860
 
  17086071
 
  ENSEMBL   UCSC 
 
  212
 
  4
 
 262
 
 gene;island
 
 0.243224
 
 0.4956177
 
 Not queried
 
  1.2501736
 
       NA
 
           NA
  
 
  
 9
 
 139743586
 
 139743802
 
  ENSEMBL   UCSC 
 
  217
 
  4
 
 40795
 
 gene;island
 
 0.007912
 
 0.0073557
 
 Not queried
 
  1.2501420
 
       NA
 
           NA
  
 
  
 13
 
  37574527
 
  37574825
 
  ENSEMBL   UCSC 
 
  299
 
  8
 
 11399
 
 tss
 
 0.003022
 
 0.0009585
 
 Not queried
 
  1.2500937
 
       NA
 
           NA
  
 
  
 13
 
  37574527
 
  37575268
 
  ENSEMBL   UCSC 
 
  742
 
 12
 
 11676
 
 island
 
 0.004579
 
 0.0017703
 
 Not queried
 
  1.2500937
 
       NA
 
           NA
  
 
  
 11
 
  63753020
 
  63753251
 
  ENSEMBL   UCSC 
 
  232
 
  6
 
 7382
 
 tss
 
 0.183984
 
 0.0418117
 
 Not queried
 
  1.2500238
 
       NA
 
           NA
  
 
  
 10
 
  23982350
 
  23982579
 
  ENSEMBL   UCSC 
 
  230
 
  4
 
 4988
 
 tss;island
 
 0.012946
 
 0.0359581
 
 Not queried
 
  1.2498748
 
       NA
 
           NA
  
 
  
 11
 
   1847227
 
   1847357
 
  ENSEMBL   UCSC 
 
  131
 
  4
 
 7934
 
 island
 
 0.555807
 
 0.7060553
 
 Not queried
 
  1.2491506
 
       NA
 
           NA
  
 
  
 3
 
  98452812
 
  98453086
 
  ENSEMBL   UCSC 
 
  275
 
  4
 
 27873
 
 gene;island
 
 0.003645
 
 0.0072611
 
 Not queried
 
  1.2488384
 
       NA
 
           NA
  
 
  
 19
 
  45417526
 
  45417814
 
  ENSEMBL   UCSC 
 
  289
 
  5
 
 20856
 
 tss
 
 0.569863
 
 0.6942452
 
 Not queried
 
  1.2481697
 
       NA
 
           NA
  
 
  
 7
 
   4872926
 
   4873549
 
  ENSEMBL   UCSC 
 
  624
 
  6
 
 36860
 
 gene;island
 
 0.407463
 
 0.5170712
 
 Not queried
 
  1.2481697
 
       NA
 
           NA
  
 
  
 17
 
  32907636
 
  32907731
 
  ENSEMBL   UCSC 
 
   96
 
  4
 
 17261
 
 tss
 
 0.195038
 
 0.0458964
 
 Not queried
 
  1.2480244
 
       NA
 
           NA
  
 
  
 3
 
  62861544
 
  62861925
 
  ENSEMBL   UCSC 
 
  382
 
  4
 
 28429
 
 tss;island
 
 0.165575
 
 0.5610102
 
 Not queried
 
  1.2477670
 
       NA
 
           NA
  
 
  
 8
 
  67454546
 
  67454892
 
  ENSEMBL   UCSC 
 
  347
 
  5
 
 40356
 
 island
 
 0.499594
 
 0.9122876
 
 Not queried
 
  1.2476995
 
       NA
 
           NA
  
 
  
 15
 
  42371511
 
  42371967
 
  ENSEMBL   UCSC 
 
  457
 
  7
 
 13307
 
 gene;island
 
 0.654490
 
 0.4885842
 
 Not queried
 
  1.2476435
 
       NA
 
           NA
  
 
  
 18
 
  19780587
 
  19780878
 
  ENSEMBL   UCSC 
 
  292
 
  4
 
 19069
 
 gene;island
 
 0.606089
 
 0.8002624
 
 Not queried
 
  1.2472733
 
       NA
 
           NA
  
 
  
 17
 
  72580901
 
  72581014
 
  ENSEMBL   UCSC 
 
  114
 
  4
 
 17628
 
 tss
 
 0.507295
 
 0.7080451
 
 Not queried
 
  1.2460853
 
       NA
 
           NA
  
 
  
 4
 
  37688031
 
  37688612
 
  ENSEMBL   UCSC 
 
  582
 
  8
 
 30342
 
 tss;island
 
 0.004338
 
 0.0058663
 
 Not queried
 
  1.2454715
 
       NA
 
           NA
  
 
  
 2
 
  48982509
 
  48982957
 
  ENSEMBL   UCSC 
 
  449
 
  6
 
 24290
 
 island
 
 0.502617
 
 0.1311634
 
 Not queried
 
  1.2452547
 
       NA
 
           NA
  
 
  
 4
 
  83293766
 
  83294101
 
  ENSEMBL   UCSC 
 
  336
 
  5
 
 30038
 
 gene;island
 
 0.005078
 
 0.0006734
 
 Not queried
 
  1.2444636
 
       NA
 
           NA
  
 
  
 11
 
   9335544
 
   9335706
 
  ENSEMBL   UCSC 
 
  163
 
  4
 
 6503
 
 gene;island
 
 0.020036
 
 0.0100421
 
 Not queried
 
  1.2442888
 
       NA
 
           NA
  
 
  
 19
 
  18496984
 
  18497201
 
  ENSEMBL   UCSC 
 
  218
 
  4
 
 19934
 
 gene
 
 0.678085
 
 0.4541483
 
 Not queried
 
  1.2441652
 
       NA
 
           NA
  
 
  
 20
 
   4795836
 
   4796176
 
  ENSEMBL   UCSC 
 
  341
 
  7
 
 25350
 
 gene;tss
 
 0.006408
 
 0.0029214
 
 Not queried
 
  1.2440323
 
       NA
 
           NA
  
 
  
 11
 
  65266482
 
  65266561
 
  ENSEMBL   UCSC 
 
   80
 
  4
 
 6708
 
 gene;island
 
 0.006380
 
 0.0169704
 
 Not queried
 
  1.2439349
 
       NA
 
           NA
  
 
  
 17
 
  74099707
 
  74099835
 
  ENSEMBL   UCSC 
 
  129
 
  4
 
 16785
 
 gene
 
 0.005319
 
 0.0050211
 
 Not queried
 
  1.2438399
 
       NA
 
           NA
  
 
  
 8
 
 143373411
 
 143373735
 
  ENSEMBL   UCSC 
 
  325
 
  4
 
 39457
 
 gene
 
 0.659336
 
 0.9043487
 
 Not queried
 
  1.2438378
 
       NA
 
           NA
  
 
  
 17
 
   4890669
 
   4890696
 
  ENSEMBL   UCSC 
 
   28
 
  4
 
 17037
 
 tss
 
 0.007621
 
 0.0033081
 
 Not queried
 
  1.2436349
 
       NA
 
           NA
  
 
  
 7
 
 140103545
 
 140103783
 
  ENSEMBL   UCSC 
 
  239
 
  7
 
 37937
 
 tss
 
 0.083411
 
 0.0649459
 
 Not queried
 
  1.2435083
 
       NA
 
           NA
  
 
  
 5
 
 121187445
 
 121187598
 
  ENSEMBL   UCSC 
 
  154
 
  5
 
 32241
 
 tss;island
 
 0.471265
 
 0.6783598
 
 Not queried
 
  1.2434076
 
       NA
 
           NA
  
 
  
 14
 
  89020645
 
  89021109
 
  ENSEMBL   UCSC 
 
  465
 
  5
 
 12167
 
 gene
 
 0.035432
 
 0.0155276
 
 Not queried
 
  1.2429506
 
       NA
 
           NA
  
 
  
 16
 
  12997467
 
  12997684
 
  ENSEMBL   UCSC 
 
  218
 
  4
 
 14673
 
 gene;island
 
 0.059670
 
 0.0380047
 
 Not queried
 
  1.2427223
 
       NA
 
           NA
  
 
  
 13
 
  31479935
 
  31480304
 
  ENSEMBL   UCSC 
 
  370
 
  6
 
 11376
 
 tss
 
 0.287389
 
 0.5995818
 
 Not queried
 
  1.2425839
 
       NA
 
           NA
  
 
  
 12
 
  54582795
 
  54583029
 
  ENSEMBL   UCSC 
 
  235
 
  7
 
 9758
 
 tss
 
 0.004503
 
 0.0018240
 
 Not queried
 
  1.2423996
 
       NA
 
           NA
  
 
  
 2
 
 152830505
 
 152830665
 
  ENSEMBL   UCSC 
 
  161
 
  5
 
 22897
 
 gene;tss
 
 0.562013
 
 0.4589518
 
 Not queried
 
  1.2421591
 
       NA
 
           NA
  
 
  
 10
 
 126303581
 
 126303795
 
  ENSEMBL   UCSC 
 
  215
 
  4
 
 6231
 
 island
 
 0.923684
 
 0.9639643
 
 Not queried
 
  1.2421189
 
       NA
 
           NA
  
 
  
 17
 
  16120399
 
  16120465
 
  ENSEMBL   UCSC 
 
   67
 
  5
 
 17155
 
 tss
 
 0.011647
 
 0.0056448
 
 Not queried
 
  1.2420708
 
       NA
 
           NA
  
 
  
 13
 
  33589962
 
  33590495
 
  ENSEMBL   UCSC 
 
  534
 
 11
 
 11385
 
 tss;island
 
 0.043479
 
 0.0144087
 
 Not queried
 
  1.2418070
 
       NA
 
           NA
  
 
  
 20
 
  47896769
 
  47897207
 
  ENSEMBL   UCSC 
 
  439
 
  5
 
 25807
 
 tss
 
 0.062083
 
 0.0187084
 
 Not queried
 
  1.2418003
 
       NA
 
           NA
  
 
  
 3
 
 126748542
 
 126748873
 
  ENSEMBL   UCSC 
 
  332
 
  4
 
 27931
 
 gene;island
 
 0.721266
 
 0.8689322
 
 Not queried
 
  1.2417940
 
       NA
 
           NA
  
 
  
 16
 
  23568302
 
  23568683
 
  ENSEMBL   UCSC 
 
  382
 
  6
 
 14692
 
 gene
 
 0.003356
 
 0.0047503
 
 Not queried
 
  1.2414426
 
       NA
 
           NA
  
 
  
 6
 
 170605952
 
 170606506
 
  ENSEMBL   UCSC 
 
  555
 
  6
 
 36692
 
 island
 
 0.084104
 
 0.0202406
 
 Not queried
 
  1.2414326
 
       NA
 
           NA
  
 
  
 4
 
  55095148
 
  55095261
 
  ENSEMBL   UCSC 
 
  114
 
  4
 
 30401
 
 tss
 
 0.049150
 
 0.0341770
 
 Not queried
 
  1.2413214
 
       NA
 
           NA
  
 
  
 18
 
  47018588
 
  47018976
 
  ENSEMBL   UCSC 
 
  389
 
  6
 
 19271
 
 tss;island
 
 0.007683
 
 0.0078460
 
 Not queried
 
  1.2410244
 
       NA
 
           NA
  
 
  
 11
 
   2293552
 
   2293665
 
  ENSEMBL   UCSC 
 
  114
 
  6
 
 7065
 
 tss;island
 
 0.274110
 
 0.5741689
 
 Not queried
 
  1.2409452
 
       NA
 
           NA
  
 
  
 22
 
  25615317
 
  25615557
 
  ENSEMBL   UCSC 
 
  241
 
  8
 
 27029
 
 tss
 
 0.454026
 
 0.8627465
 
 Not queried
 
  1.2408834
 
       NA
 
           NA
  
 
  
 3
 
 157221198
 
 157221329
 
  ENSEMBL   UCSC 
 
  132
 
  4
 
 28010
 
 gene;tss
 
 0.499386
 
 0.6749902
 
 Not queried
 
  1.2398219
 
       NA
 
           NA
  
 
  
 17
 
  73642404
 
  73642773
 
  ENSEMBL   UCSC 
 
  370
 
  7
 
 16773
 
 gene
 
 0.097440
 
 0.0366244
 
 Not queried
 
  1.2398070
 
       NA
 
           NA
  
 
  
 8
 
   1921165
 
   1921906
 
  ENSEMBL   UCSC 
 
  742
 
  7
 
 39536
 
 tss;island
 
 0.011271
 
 0.0096709
 
 Not queried
 
  1.2397814
 
       NA
 
           NA
  
 
  
 13
 
  20437712
 
  20437969
 
  ENSEMBL   UCSC 
 
  258
 
  5
 
 11563
 
 island
 
 0.010865
 
 0.0106238
 
 Not queried
 
  1.2396092
 
       NA
 
           NA
  
 
  
 2
 
 220109985
 
 220110125
 
  ENSEMBL   UCSC 
 
  141
 
  5
 
 23027
 
 gene;tss;island
 
 0.006202
 
 0.0090261
 
 Not queried
 
  1.2394966
 
       NA
 
           NA
  
 
  
 19
 
  56915403
 
  56915658
 
  ENSEMBL   UCSC 
 
  256
 
  6
 
 21097
 
 tss
 
 0.040009
 
 0.0318824
 
 Not queried
 
  1.2390039
 
       NA
 
           NA
  
 
  
 10
 
   1205563
 
   1205942
 
  ENSEMBL   UCSC 
 
  380
 
  8
 
 5489
 
 island
 
 0.776935
 
 0.9120297
 
 Not queried
 
  1.2385329
 
       NA
 
           NA
  
 
  
 2
 
 177017331
 
 177017797
 
  ENSEMBL   UCSC 
 
  467
 
  6
 
 22939
 
 gene;island
 
 0.107109
 
 0.1763571
 
 Not queried
 
  1.2384941
 
       NA
 
           NA
  
 
  
 6
 
  10411781
 
  10412486
 
  ENSEMBL   UCSC 
 
  706
 
  8
 
 33557
 
 gene;island
 
 0.009039
 
 0.0649249
 
 Not queried
 
  1.2381741
 
       NA
 
           NA
  
 
  
 7
 
  19812327
 
  19813525
 
  ENSEMBL   UCSC 
 
 1199
 
 16
 
 38289
 
 island
 
 0.471991
 
 0.0836274
 
 Not queried
 
  1.2373082
 
       NA
 
           NA
  
 
  
 5
 
 132948251
 
 132948515
 
  ENSEMBL   UCSC 
 
  265
 
  4
 
 32284
 
 tss
 
 0.014524
 
 0.0071919
 
 Not queried
 
  1.2371610
 
       NA
 
           NA
  
 
  
 6
 
  13873924
 
  13874251
 
  ENSEMBL   UCSC 
 
  328
 
  4
 
 35459
 
 island
 
 0.013279
 
 0.0294182
 
 Not queried
 
  1.2363917
 
       NA
 
           NA
  
 
  
 14
 
  24911599
 
  24911994
 
  ENSEMBL   UCSC 
 
  396
 
  4
 
 12051
 
 gene
 
 0.010506
 
 0.0045634
 
 Not queried
 
  1.2360633
 
       NA
 
           NA
  
 
  
 5
 
  63802106
 
  63802993
 
  ENSEMBL   UCSC 
 
  888
 
 10
 
 32829
 
 island
 
 0.066782
 
 0.0173660
 
 Not queried
 
  1.2359505
 
       NA
 
           NA
  
 
  
 2
 
  12858298
 
  12858655
 
  ENSEMBL   UCSC 
 
  358
 
  5
 
 22577
 
 gene;island
 
 0.084101
 
 0.0167871
 
 Not queried
 
  1.2358667
 
       NA
 
           NA
  
 
  
 6
 
 154831692
 
 154831780
 
  ENSEMBL   UCSC 
 
   89
 
  4
 
 36586
 
 island
 
 0.030349
 
 0.0160269
 
 Not queried
 
  1.2358271
 
       NA
 
           NA
  
 
  
 11
 
  66083541
 
  66083766
 
  ENSEMBL   UCSC 
 
  226
 
  4
 
 6729
 
 gene;island
 
 0.856603
 
 0.8773008
 
 Not queried
 
  1.2353180
 
       NA
 
           NA
  
 
  
 20
 
  60502022
 
  60502248
 
  ENSEMBL   UCSC 
 
  227
 
  4
 
 25482
 
 gene;island
 
 0.727388
 
 0.8761488
 
 Not queried
 
  1.2348451
 
       NA
 
           NA
  
 
  
 7
 
  75956931
 
  75957376
 
  ENSEMBL   UCSC 
 
  446
 
  7
 
 37054
 
 gene;island
 
 0.266943
 
 0.1108666
 
 Not queried
 
  1.2346562
 
       NA
 
           NA
  
 
  
 6
 
  32061084
 
  32061478
 
  ENSEMBL   UCSC 
 
  395
 
  4
 
 34044
 
 gene;island
 
 0.114727
 
 0.2366923
 
 Not queried
 
  1.2342097
 
       NA
 
           NA
  
 
  
 6
 
  46703193
 
  46703670
 
  ENSEMBL   UCSC 
 
  478
 
 15
 
 35022
 
 tss
 
 0.015972
 
 0.1222268
 
 Not queried
 
  1.2341902
 
       NA
 
           NA
  
 
  
 6
 
  86158972
 
  86159251
 
  ENSEMBL   UCSC 
 
  280
 
  6
 
 35102
 
 tss
 
 0.038394
 
 0.2670990
 
 Not queried
 
  1.2340146
 
       NA
 
           NA
  
 
  
 8
 
 146176323
 
 146176538
 
  ENSEMBL   UCSC 
 
  216
 
  6
 
 39996
 
 tss
 
 0.034895
 
 0.0140200
 
 Not queried
 
  1.2338999
 
       NA
 
           NA
  
 
  
 11
 
 134254573
 
 134254936
 
  ENSEMBL   UCSC 
 
  364
 
  6
 
 6999
 
 gene;island
 
 0.301219
 
 0.5077562
 
 Not queried
 
  1.2337679
 
       NA
 
           NA
  
 
  
 1
 
 145715571
 
 145715719
 
  ENSEMBL   UCSC 
 
  149
 
  4
 
 1891
 
 tss;island
 
 0.563957
 
 0.6728613
 
 Not queried
 
  1.2337541
 
       NA
 
           NA
  
 
  
 6
 
  27064705
 
  27065003
 
  ENSEMBL   UCSC 
 
  299
 
  4
 
 35545
 
 island
 
 0.006530
 
 0.0213237
 
 Not queried
 
  1.2335617
 
       NA
 
           NA
  
 
  
 2
 
 236840170
 
 236840562
 
  ENSEMBL   UCSC 
 
  393
 
  5
 
 23085
 
 gene
 
 0.788732
 
 0.8765120
 
 Not queried
 
  1.2333943
 
       NA
 
           NA
  
 
  
 6
 
 166582159
 
 166582585
 
  ENSEMBL   UCSC 
 
  427
 
  9
 
 35334
 
 tss;island
 
 0.031018
 
 0.0186652
 
 Not queried
 
  1.2328382
 
       NA
 
           NA
  
 
  
 1
 
   4221331
 
   4221616
 
  ENSEMBL   UCSC 
 
  286
 
  5
 
 2582
 
 island
 
 0.591964
 
 0.7055969
 
 Not queried
 
  1.2325800
 
       NA
 
           NA
  
 
  
 19
 
  40324850
 
  40325128
 
  ENSEMBL   UCSC 
 
  279
 
  8
 
 20778
 
 tss
 
 0.030295
 
 0.0122429
 
 Not queried
 
  1.2323619
 
       NA
 
           NA
  
 
  
 22
 
  29711916
 
  29712139
 
  ENSEMBL   UCSC 
 
  224
 
  6
 
 27047
 
 tss;island
 
 0.004427
 
 0.0185953
 
 Not queried
 
  1.2321409
 
       NA
 
           NA
  
 
  
 17
 
   7297857
 
   7298077
 
  ENSEMBL   UCSC 
 
  221
 
  4
 
 17078
 
 tss
 
 0.007757
 
 0.0039031
 
 Not queried
 
  1.2321099
 
       NA
 
           NA
  
 
  
 12
 
 132929654
 
 132930230
 
  ENSEMBL   UCSC 
 
  577
 
  6
 
 11046
 
 island
 
 0.464903
 
 0.5448094
 
 Not queried
 
  1.2317538
 
       NA
 
           NA
  
 
  
 2
 
 242784034
 
 242784150
 
  ENSEMBL   UCSC 
 
  117
 
  4
 
 25307
 
 island
 
 0.918825
 
 0.9676776
 
 Not queried
 
  1.2311749
 
       NA
 
           NA
  
 
  
 14
 
  39583276
 
  39583547
 
  ENSEMBL   UCSC 
 
  272
 
  5
 
 12804
 
 island
 
 0.002291
 
 0.0030365
 
 Not queried
 
  1.2310110
 
       NA
 
           NA
  
 
  
 2
 
  73404524
 
  73404999
 
  ENSEMBL   UCSC 
 
  476
 
  4
 
 24411
 
 island
 
 0.024531
 
 0.0147139
 
 Not queried
 
  1.2309975
 
       NA
 
           NA
  
 
  
 10
 
  72971961
 
  72972207
 
  ENSEMBL   UCSC 
 
  247
 
  8
 
 5141
 
 tss;island
 
 0.026451
 
 0.0082388
 
 Not queried
 
  1.2303732
 
       NA
 
           NA
  
 
  
 15
 
  62989935
 
  62990138
 
  ENSEMBL   UCSC 
 
  204
 
  5
 
 13370
 
 gene;island
 
 0.751534
 
 0.8766952
 
 Not queried
 
  1.2298375
 
       NA
 
           NA
  
 
  
 5
 
 112824614
 
 112824920
 
  ENSEMBL   UCSC 
 
  307
 
  6
 
 32219
 
 tss
 
 0.385489
 
 0.5211493
 
 Not queried
 
  1.2296785
 
       NA
 
           NA
  
 
  
 13
 
  36788667
 
  36789025
 
  ENSEMBL   UCSC 
 
  359
 
  8
 
 11667
 
 island
 
 0.658256
 
 0.7892252
 
 Not queried
 
  1.2291795
 
       NA
 
           NA
  
 
  
 2
 
 176032513
 
 176032603
 
  ENSEMBL   UCSC 
 
   91
 
  7
 
 23727
 
 tss
 
 0.006990
 
 0.0030619
 
 Not queried
 
  1.2289009
 
       NA
 
           NA
  
 
  
 13
 
 113121632
 
 113121958
 
  ENSEMBL   UCSC 
 
  327
 
  4
 
 11924
 
 island
 
 0.765589
 
 0.8722634
 
 Not queried
 
  1.2286301
 
       NA
 
           NA
  
 
  
 19
 
  58400494
 
  58400574
 
  ENSEMBL   UCSC 
 
   81
 
  4
 
 21127
 
 tss
 
 0.086190
 
 0.0369280
 
 Not queried
 
  1.2282499
 
       NA
 
           NA
  
 
  
 12
 
  49582842
 
  49583112
 
  ENSEMBL   UCSC 
 
  271
 
  8
 
 10388
 
 island
 
 0.015339
 
 0.0061832
 
 Not queried
 
  1.2273955
 
       NA
 
           NA
  
 
  
 12
 
 133526451
 
 133526768
 
  ENSEMBL   UCSC 
 
  318
 
  4
 
 9470
 
 gene
 
 0.900019
 
 0.9676500
 
 Not queried
 
  1.2268971
 
       NA
 
           NA
  
 
  
 2
 
 219849133
 
 219849307
 
  ENSEMBL   UCSC 
 
  175
 
  4
 
 23023
 
 gene;island
 
 0.025389
 
 0.0117867
 
 Not queried
 
  1.2267496
 
       NA
 
           NA
  
 
  
 2
 
    264904
 
    265069
 
  ENSEMBL   UCSC 
 
  166
 
  4
 
 22526
 
 gene
 
 0.007379
 
 0.0028816
 
 Not queried
 
  1.2264197
 
       NA
 
           NA
  
 
  
 16
 
  15595854
 
  15596423
 
  ENSEMBL   UCSC 
 
  570
 
  9
 
 14675
 
 gene
 
 0.487732
 
 0.8424604
 
 Not queried
 
  1.2262479
 
       NA
 
           NA
  
 
  
 5
 
  76114373
 
  76114933
 
  ENSEMBL   UCSC 
 
  561
 
  9
 
 32895
 
 island
 
 0.038123
 
 0.0142066
 
 Not queried
 
  1.2257192
 
       NA
 
           NA
  
 
  
 2
 
 169658881
 
 169659182
 
  ENSEMBL   UCSC 
 
  302
 
  6
 
 22913
 
 gene
 
 0.473989
 
 0.6873780
 
 Not queried
 
  1.2254706
 
       NA
 
           NA
  
 
  
 17
 
  37821447
 
  37821536
 
  ENSEMBL   UCSC 
 
   90
 
  4
 
 17312
 
 tss
 
 0.732387
 
 0.7202244
 
 Not queried
 
  1.2247651
 
       NA
 
           NA
  
 
  
 1
 
  34175169
 
  34175343
 
  ENSEMBL   UCSC 
 
  175
 
  4
 
 407
 
 gene
 
 0.300447
 
 0.5174854
 
 Not queried
 
  1.2247500
 
       NA
 
           NA
  
 
  
 10
 
  65028929
 
  65029168
 
  ENSEMBL   UCSC 
 
  240
 
  5
 
 4604
 
 gene
 
 0.001219
 
 0.0023812
 
 Not queried
 
  1.2245806
 
       NA
 
           NA
  
 
  
 1
 
  17381800
 
  17382130
 
  ENSEMBL   UCSC 
 
  331
 
  4
 
 1290
 
 tss;island
 
 0.332914
 
 0.5193346
 
 Not queried
 
  1.2244123
 
       NA
 
           NA
  
 
  
 2
 
 130691738
 
 130691956
 
  ENSEMBL   UCSC 
 
  219
 
  5
 
 24713
 
 island
 
 0.037310
 
 0.0950186
 
 Not queried
 
  1.2243272
 
       NA
 
           NA
  
 
  
 1
 
  46598727
 
  46598774
 
  ENSEMBL   UCSC 
 
   48
 
  4
 
 1593
 
 tss;island
 
 0.007031
 
 0.0068622
 
 Not queried
 
  1.2242886
 
       NA
 
           NA
  
 
  
 14
 
  79744991
 
  79745592
 
  ENSEMBL   UCSC 
 
  602
 
  7
 
 12159
 
 gene;tss;island
 
 0.072262
 
 0.0223449
 
 Not queried
 
  1.2241567
 
       NA
 
           NA
  
 
  
 12
 
   6419409
 
   6419750
 
  ENSEMBL   UCSC 
 
  342
 
  6
 
 9506
 
 tss;island
 
 0.744213
 
 0.4265779
 
 Not queried
 
  1.2241135
 
       NA
 
           NA
  
 
  
 5
 
 134181484
 
 134181607
 
  ENSEMBL   UCSC 
 
  124
 
  6
 
 32299
 
 tss
 
 0.003159
 
 0.0027796
 
 Not queried
 
  1.2239907
 
       NA
 
           NA
  
 
  
 11
 
  10715175
 
  10715767
 
  ENSEMBL   UCSC 
 
  593
 
 11
 
 7147
 
 tss
 
 0.315549
 
 0.5765010
 
 Not queried
 
  1.2237375
 
       NA
 
           NA
  
 
  
 6
 
  30312580
 
  30312866
 
  ENSEMBL   UCSC 
 
  287
 
  7
 
 34770
 
 tss
 
 0.012161
 
 0.0407119
 
 Not queried
 
  1.2232875
 
       NA
 
           NA
  
 
  
 3
 
  14597418
 
  14597729
 
  ENSEMBL   UCSC 
 
  312
 
  4
 
 28913
 
 island
 
 0.199348
 
 0.4481712
 
 Not queried
 
  1.2231157
 
       NA
 
           NA
  
 
  
 3
 
  39447891
 
  39448280
 
  ENSEMBL   UCSC 
 
  390
 
 11
 
 28234
 
 tss;island
 
 0.003978
 
 0.0023082
 
 Not queried
 
  1.2222819
 
       NA
 
           NA
  
 
  
 2
 
  11750735
 
  11751071
 
  ENSEMBL   UCSC 
 
  337
 
  4
 
 22572
 
 gene;island
 
 0.801660
 
 0.9586945
 
 Not queried
 
  1.2216359
 
       NA
 
           NA
  
 
  
 16
 
  14724253
 
  14724468
 
  ENSEMBL   UCSC 
 
  216
 
  4
 
 15103
 
 tss;island
 
 0.006540
 
 0.0090679
 
 Not queried
 
  1.2215998
 
       NA
 
           NA
  
 
  
 22
 
  31643997
 
  31644282
 
  ENSEMBL   UCSC 
 
  286
 
  4
 
 26841
 
 gene;tss
 
 0.612444
 
 0.2284310
 
 Not queried
 
  1.2215191
 
       NA
 
           NA
  
 
  
 12
 
  54089240
 
  54089497
 
  ENSEMBL   UCSC 
 
  258
 
  4
 
 10471
 
 island
 
 0.021001
 
 0.0229797
 
 Not queried
 
  1.2214584
 
       NA
 
           NA
  
 
  
 1
 
  44398868
 
  44399069
 
  ENSEMBL   UCSC 
 
  202
 
  4
 
 3172
 
 island
 
 0.047468
 
 0.0409612
 
 Not queried
 
  1.2213382
 
       NA
 
           NA
  
 
  
 7
 
  95025736
 
  95026248
 
  ENSEMBL   UCSC 
 
  513
 
 19
 
 37726
 
 tss
 
 0.096442
 
 0.2709027
 
 Not queried
 
  1.2212271
 
       NA
 
           NA
  
 
  
 10
 
   8095121
 
   8095363
 
  ENSEMBL   UCSC 
 
  243
 
  4
 
 4488
 
 gene
 
 0.010468
 
 0.0200720
 
 Not queried
 
  1.2209962
 
       NA
 
           NA
  
 
  
 3
 
  13895992
 
  13896256
 
  ENSEMBL   UCSC 
 
  265
 
  5
 
 27685
 
 gene;island
 
 0.424005
 
 0.7438252
 
 Not queried
 
  1.2205215
 
       NA
 
           NA
  
 
  
 17
 
  51899837
 
  51900115
 
  ENSEMBL   UCSC 
 
  279
 
  6
 
 17516
 
 tss
 
 0.410644
 
 0.5871036
 
 Not queried
 
  1.2196589
 
       NA
 
           NA
  
 
  
 10
 
  57387511
 
  57387715
 
  ENSEMBL   UCSC 
 
  205
 
  4
 
 5753
 
 island
 
 0.334418
 
 0.1721217
 
 Not queried
 
  1.2196445
 
       NA
 
           NA
  
 
  
 12
 
  42538806
 
  42538901
 
  ENSEMBL   UCSC 
 
   96
 
  7
 
 9641
 
 tss
 
 0.002978
 
 0.0041287
 
 Not queried
 
  1.2195215
 
       NA
 
           NA
  
 
  
 5
 
 176937350
 
 176937527
 
  ENSEMBL   UCSC 
 
  178
 
  4
 
 32531
 
 tss
 
 0.016673
 
 0.0100931
 
 Not queried
 
  1.2192711
 
       NA
 
           NA
  
 
  
 2
 
 241395181
 
 241395351
 
  ENSEMBL   UCSC 
 
  171
 
  4
 
 23121
 
 gene;tss;island
 
 0.037964
 
 0.0136461
 
 Not queried
 
  1.2187868
 
       NA
 
           NA
  
 
  
 1
 
 172113756
 
 172114068
 
  ENSEMBL   UCSC 
 
  313
 
  4
 
 885
 
 gene
 
 0.946093
 
 0.9776784
 
 Not queried
 
  1.2185060
 
       NA
 
           NA
  
 
  
 6
 
 100054469
 
 100055173
 
  ENSEMBL   UCSC 
 
  705
 
 11
 
 36357
 
 island
 
 0.429988
 
 0.1065370
 
 Not queried
 
  1.2184724
 
       NA
 
           NA
  
 
  
 7
 
 105221985
 
 105222180
 
  ENSEMBL   UCSC 
 
  196
 
  7
 
 37819
 
 tss
 
 0.037695
 
 0.0146593
 
 Not queried
 
  1.2180483
 
       NA
 
           NA
  
 
  
 19
 
    450940
 
    451320
 
  ENSEMBL   UCSC 
 
  381
 
  4
 
 19627
 
 gene;island
 
 0.787108
 
 0.8639216
 
 Not queried
 
  1.2178842
 
       NA
 
           NA
  
 
  
 3
 
 125075895
 
 125076480
 
  ENSEMBL   UCSC 
 
  586
 
  5
 
 27926
 
 gene;island
 
 0.011005
 
 0.0039253
 
 Not queried
 
  1.2176827
 
       NA
 
           NA
  
 
  
 8
 
 145024637
 
 145025178
 
  ENSEMBL   UCSC 
 
  542
 
  9
 
 39502
 
 gene;island
 
 0.589060
 
 0.7037688
 
 Not queried
 
  1.2176361
 
       NA
 
           NA
  
 
  
 4
 
 106816089
 
 106816564
 
  ENSEMBL   UCSC 
 
  476
 
  5
 
 30532
 
 tss;island
 
 0.033487
 
 0.0200632
 
 Not queried
 
  1.2175274
 
       NA
 
           NA
  
 
  
 2
 
   1544321
 
   1544474
 
  ENSEMBL   UCSC 
 
  154
 
  4
 
 22533
 
 gene;island
 
 0.444655
 
 0.6875451
 
 Not queried
 
  1.2174880
 
       NA
 
           NA
  
 
  
 10
 
 134971594
 
 134972063
 
  ENSEMBL   UCSC 
 
  470
 
  5
 
 6337
 
 island
 
 0.531319
 
 0.7246450
 
 Not queried
 
  1.2174121
 
       NA
 
           NA
  
 
  
 2
 
 175199259
 
 175199828
 
  ENSEMBL   UCSC 
 
  570
 
 12
 
 24874
 
 island
 
 0.107004
 
 0.0369089
 
 Not queried
 
  1.2173835
 
       NA
 
           NA
  
 
  
 4
 
  89205891
 
  89206201
 
  ENSEMBL   UCSC 
 
  311
 
  7
 
 30491
 
 tss
 
 0.002464
 
 0.0064183
 
 Not queried
 
  1.2170117
 
       NA
 
           NA
  
 
  
 1
 
 234743527
 
 234743830
 
  ENSEMBL   UCSC 
 
  304
 
  4
 
 1087
 
 gene;island
 
 0.002361
 
 0.0059182
 
 Not queried
 
  1.2166851
 
       NA
 
           NA
  
 
  
 6
 
  43044771
 
  43045187
 
  ENSEMBL   UCSC 
 
  417
 
  4
 
 34342
 
 gene;island
 
 0.046520
 
 0.1030225
 
 Not queried
 
  1.2166535
 
       NA
 
           NA
  
 
  
 15
 
  56035371
 
  56035616
 
  ENSEMBL   UCSC 
 
  246
 
  6
 
 13685
 
 tss;island
 
 0.186584
 
 0.4802745
 
 Not queried
 
  1.2165380
 
       NA
 
           NA
  
 
  
 8
 
 143915776
 
 143916206
 
  ENSEMBL   UCSC 
 
  431
 
  7
 
 39941
 
 tss
 
 0.499228
 
 0.8593349
 
 Not queried
 
  1.2162342
 
       NA
 
           NA
  
 
  
 11
 
 112831501
 
 112831938
 
  ENSEMBL   UCSC 
 
  438
 
  8
 
 7701
 
 tss
 
 0.132069
 
 0.2744940
 
 Not queried
 
  1.2161411
 
       NA
 
           NA
  
 
  
 5
 
 140736762
 
 140737111
 
  ENSEMBL   UCSC 
 
  350
 
  5
 
 31821
 
 gene;island
 
 0.520587
 
 0.3939665
 
 Not queried
 
  1.2160525
 
       NA
 
           NA
  
 
  
 19
 
  12250778
 
  12251131
 
  ENSEMBL   UCSC 
 
  354
 
  4
 
 19852
 
 gene
 
 0.008607
 
 0.0044449
 
 Not queried
 
  1.2156835
 
       NA
 
           NA
  
 
  
 5
 
  45695467
 
  45695922
 
  ENSEMBL   UCSC 
 
  456
 
  4
 
 31607
 
 gene;island
 
 0.177183
 
 0.4768052
 
 Not queried
 
  1.2156749
 
       NA
 
           NA
  
 
  
 20
 
  30028062
 
  30028292
 
  ENSEMBL   UCSC 
 
  231
 
  6
 
 25657
 
 tss
 
 0.535292
 
 0.7986727
 
 Not queried
 
  1.2153510
 
       NA
 
           NA
  
 
  
 8
 
 145770785
 
 145771145
 
  ENSEMBL   UCSC 
 
  361
 
  4
 
 39527
 
 gene;island
 
 0.607011
 
 0.8594279
 
 Not queried
 
  1.2151703
 
       NA
 
           NA
  
 
  
 22
 
  25201891
 
  25202163
 
  ENSEMBL   UCSC 
 
  273
 
  8
 
 27367
 
 island
 
 0.007701
 
 0.0267949
 
 Not queried
 
  1.2148495
 
       NA
 
           NA
  
 
  
 2
 
 121411889
 
 121412432
 
  ENSEMBL   UCSC 
 
  544
 
  5
 
 24668
 
 island
 
 0.310770
 
 0.8830205
 
 Not queried
 
  1.2144677
 
       NA
 
           NA
  
 
  
 8
 
   1441386
 
   1441539
 
  ENSEMBL   UCSC 
 
  154
 
  4
 
 40026
 
 island
 
 0.564472
 
 0.7581835
 
 Not queried
 
  1.2141850
 
       NA
 
           NA
  
 
  
 18
 
  44562019
 
  44562136
 
  ENSEMBL   UCSC 
 
  118
 
  6
 
 19106
 
 gene;tss;island
 
 0.692030
 
 0.9630397
 
 Not queried
 
  1.2139619
 
       NA
 
           NA
  
 
  
 2
 
  54952078
 
  54952487
 
  ENSEMBL   UCSC 
 
  410
 
  5
 
 24304
 
 island
 
 0.008427
 
 0.0033295
 
 Not queried
 
  1.2138162
 
       NA
 
           NA
  
 
  
 12
 
  52695197
 
  52695728
 
  ENSEMBL   UCSC 
 
  532
 
  8
 
 10440
 
 island
 
 0.541385
 
 0.7196362
 
 Not queried
 
  1.2138027
 
       NA
 
           NA
  
 
  
 6
 
  34664699
 
  34665156
 
  ENSEMBL   UCSC 
 
  458
 
  6
 
 34921
 
 tss;island
 
 0.003142
 
 0.0062320
 
 Not queried
 
  1.2136989
 
       NA
 
           NA
  
 
  
 2
 
 204400159
 
 204400358
 
  ENSEMBL   UCSC 
 
  200
 
  6
 
 23831
 
 tss
 
 0.014128
 
 0.0121338
 
 Not queried
 
  1.2136816
 
       NA
 
           NA
  
 
  
 3
 
 138658243
 
 138659021
 
  ENSEMBL   UCSC 
 
  779
 
  8
 
 29529
 
 island
 
 0.042922
 
 0.0185773
 
 Not queried
 
  1.2136229
 
       NA
 
           NA
  
 
  
 19
 
  57631118
 
  57631478
 
  ENSEMBL   UCSC 
 
  361
 
  9
 
 21105
 
 tss;island
 
 0.745198
 
 0.8940689
 
 Not queried
 
  1.2134524
 
       NA
 
           NA
  
 
  
 6
 
  32822857
 
  32823292
 
  ENSEMBL   UCSC 
 
  436
 
  5
 
 34157
 
 gene;island
 
 0.001601
 
 0.0015547
 
 Not queried
 
  1.2132545
 
       NA
 
           NA
  
 
  
 22
 
  19776050
 
  19776431
 
  ENSEMBL   UCSC 
 
  382
 
  4
 
 26790
 
 gene;island
 
 0.941290
 
 0.9731374
 
 Not queried
 
  1.2132291
 
       NA
 
           NA
  
 
  
 1
 
  43770523
 
  43770899
 
  ENSEMBL   UCSC 
 
  377
 
  5
 
 475
 
 gene;island
 
 0.902170
 
 0.9625819
 
 Not queried
 
  1.2131004
 
       NA
 
           NA
  
 
  
 11
 
  76839129
 
  76839217
 
  ENSEMBL   UCSC 
 
   89
 
  4
 
 7583
 
 tss
 
 0.082626
 
 0.3095805
 
 Not queried
 
  1.2129928
 
       NA
 
           NA
  
 
  
 6
 
  84419189
 
  84419360
 
  ENSEMBL   UCSC 
 
  172
 
  9
 
 35096
 
 tss;island
 
 0.060528
 
 0.1308285
 
 Not queried
 
  1.2129621
 
       NA
 
           NA
  
 
  
 17
 
  77955167
 
  77955530
 
  ENSEMBL   UCSC 
 
  364
 
  4
 
 16848
 
 gene;island
 
 0.692014
 
 0.8659772
 
 Not queried
 
  1.2128840
 
       NA
 
           NA
  
 
  
 3
 
 154146398
 
 154146871
 
  ENSEMBL   UCSC 
 
  474
 
  5
 
 28002
 
 gene;island
 
 0.061395
 
 0.0241178
 
 Not queried
 
  1.2128771
 
       NA
 
           NA
  
 
  
 8
 
  33580365
 
  33580539
 
  ENSEMBL   UCSC 
 
  175
 
  4
 
 40216
 
 island
 
 0.284892
 
 0.4386959
 
 Not queried
 
  1.2127960
 
       NA
 
           NA
  
 
  
 2
 
 120281354
 
 120282327
 
  ENSEMBL   UCSC 
 
  974
 
 11
 
 24657
 
 island
 
 0.182732
 
 0.5066552
 
 Not queried
 
  1.2126374
 
       NA
 
           NA
  
 
  
 19
 
  45754942
 
  45755260
 
  ENSEMBL   UCSC 
 
  319
 
  4
 
 20090
 
 gene
 
 0.014617
 
 0.0153870
 
 Not queried
 
  1.2125023
 
       NA
 
           NA
  
 
  
 11
 
 124932892
 
 124933008
 
  ENSEMBL   UCSC 
 
  117
 
  5
 
 7789
 
 tss
 
 0.004438
 
 0.0050747
 
 Not queried
 
  1.2124621
 
       NA
 
           NA
  
 
  
 1
 
  63795636
 
  63795970
 
  ENSEMBL   UCSC 
 
  335
 
  5
 
 3344
 
 island
 
 0.045845
 
 0.0349063
 
 Not queried
 
  1.2122085
 
       NA
 
           NA
  
 
  
 12
 
  14927294
 
  14927542
 
  ENSEMBL   UCSC 
 
  249
 
  4
 
 9065
 
 gene
 
 0.007171
 
 0.0060570
 
 Not queried
 
  1.2116445
 
       NA
 
           NA
  
 
  
 19
 
   4007860
 
   4008209
 
  ENSEMBL   UCSC 
 
  350
 
  4
 
 19729
 
 gene;island
 
 0.006197
 
 0.0034308
 
 Not queried
 
  1.2112950
 
       NA
 
           NA
  
 
  
 19
 
  44529223
 
  44529477
 
  ENSEMBL   UCSC 
 
  255
 
  7
 
 20840
 
 tss
 
 0.017186
 
 0.0262210
 
 Not queried
 
  1.2112125
 
       NA
 
           NA
  
 
  
 8
 
  67686767
 
  67687060
 
  ENSEMBL   UCSC 
 
  294
 
  5
 
 39325
 
 gene;tss;island
 
 0.018555
 
 0.0363696
 
 Not queried
 
  1.2109370
 
       NA
 
           NA
  
 
  
 11
 
  10326549
 
  10326672
 
  ENSEMBL   UCSC 
 
  124
 
  7
 
 8055
 
 island
 
 0.007280
 
 0.0137725
 
 Not queried
 
  1.2108883
 
       NA
 
           NA
  
 
  
 5
 
  65017248
 
  65017496
 
  ENSEMBL   UCSC 
 
  249
 
  4
 
 31632
 
 gene;tss;island
 
 0.022495
 
 0.0101661
 
 Not queried
 
  1.2107787
 
       NA
 
           NA
  
 
  
 22
 
  43820888
 
  43821227
 
  ENSEMBL   UCSC 
 
  340
 
  4
 
 26889
 
 gene;island
 
 0.749283
 
 0.9050276
 
 Not queried
 
  1.2106497
 
       NA
 
           NA
  
 
  
 1
 
  92951355
 
  92953037
 
  ENSEMBL   UCSC 
 
 1683
 
 24
 
 3486
 
 island
 
 0.009438
 
 0.0056930
 
 Not queried
 
  1.2103001
 
       NA
 
           NA
  
 
  
 8
 
 144373269
 
 144373452
 
  ENSEMBL   UCSC 
 
  184
 
  6
 
 39946
 
 tss
 
 0.025150
 
 0.0115707
 
 Not queried
 
  1.2102900
 
       NA
 
           NA
  
 
  
 17
 
  27169869
 
  27170225
 
  ENSEMBL   UCSC 
 
  357
 
  9
 
 17224
 
 tss;island
 
 0.036572
 
 0.0172026
 
 Not queried
 
  1.2097882
 
       NA
 
           NA
  
 
  
 10
 
 120925093
 
 120925632
 
  ENSEMBL   UCSC 
 
  540
 
 10
 
 6192
 
 island
 
 0.006505
 
 0.0086666
 
 Not queried
 
  1.2096567
 
       NA
 
           NA
  
 
  
 7
 
  63667212
 
  63667572
 
  ENSEMBL   UCSC 
 
  361
 
  6
 
 37614
 
 tss
 
 0.491165
 
 0.6713738
 
 Not queried
 
  1.2096324
 
       NA
 
           NA
  
 
  
 17
 
  77982906
 
  77983156
 
  ENSEMBL   UCSC 
 
  251
 
  4
 
 16849
 
 gene;island
 
 0.700108
 
 0.8759838
 
 Not queried
 
  1.2094820
 
       NA
 
           NA
  
 
  
 8
 
   1785833
 
   1786322
 
  ENSEMBL   UCSC 
 
  490
 
  6
 
 39176
 
 gene
 
 0.421059
 
 0.7901336
 
 Not queried
 
  1.2094685
 
       NA
 
           NA
  
 
  
 12
 
  57579382
 
  57579596
 
  ENSEMBL   UCSC 
 
  215
 
  4
 
 9185
 
 gene;island
 
 0.854099
 
 0.9305440
 
 Not queried
 
  1.2093118
 
       NA
 
           NA
  
 
  
 1
 
 237947246
 
 237947620
 
  ENSEMBL   UCSC 
 
  375
 
  5
 
 1104
 
 gene;island
 
 0.422811
 
 0.6287389
 
 Not queried
 
  1.2091450
 
       NA
 
           NA
  
 
  
 11
 
 130298544
 
 130298880
 
  ENSEMBL   UCSC 
 
  337
 
  8
 
 7819
 
 tss
 
 0.020506
 
 0.0690734
 
 Not queried
 
  1.2090961
 
       NA
 
           NA
  
 
  
 3
 
 129261768
 
 129262055
 
  ENSEMBL   UCSC 
 
  288
 
  4
 
 28587
 
 tss
 
 0.289168
 
 0.4362880
 
 Not queried
 
  1.2088849
 
       NA
 
           NA
  
 
  
 5
 
  10563358
 
  10563856
 
  ENSEMBL   UCSC 
 
  499
 
  4
 
 31990
 
 tss;island
 
 0.014375
 
 0.0175174
 
 Not queried
 
  1.2088229
 
       NA
 
           NA
  
 
  
 11
 
  67980776
 
  67980803
 
  ENSEMBL   UCSC 
 
   28
 
  4
 
 8567
 
 island
 
 0.009868
 
 0.0095512
 
 Not queried
 
  1.2085873
 
       NA
 
           NA
  
 
  
 12
 
  83080681
 
  83080838
 
  ENSEMBL   UCSC 
 
  158
 
  4
 
 9897
 
 tss;island
 
 0.004380
 
 0.0023765
 
 Not queried
 
  1.2085000
 
       NA
 
           NA
  
 
  
 15
 
  72977851
 
  72978371
 
  ENSEMBL   UCSC 
 
  521
 
  5
 
 13410
 
 gene;tss;island
 
 0.003796
 
 0.0037666
 
 Not queried
 
  1.2083208
 
       NA
 
           NA
  
 
  
 9
 
  74764263
 
  74764504
 
  ENSEMBL   UCSC 
 
  242
 
  4
 
 40893
 
 island
 
 0.066321
 
 0.1189178
 
 Not queried
 
  1.2082171
 
       NA
 
           NA
  
 
  
 1
 
  15127680
 
  15127907
 
  ENSEMBL   UCSC 
 
  228
 
  4
 
 238
 
 gene;island
 
 0.451181
 
 0.5597050
 
 Not queried
 
  1.2081081
 
       NA
 
           NA
  
 
  
 19
 
  51220202
 
  51220537
 
  ENSEMBL   UCSC 
 
  336
 
  7
 
 20983
 
 tss
 
 0.356438
 
 0.5298739
 
 Not queried
 
  1.2080046
 
       NA
 
           NA
  
 
  
 19
 
  42348327
 
  42349127
 
  ENSEMBL   UCSC 
 
  801
 
 13
 
 20814
 
 tss;island
 
 0.794434
 
 0.8939041
 
 Not queried
 
  1.2077889
 
       NA
 
           NA
  
 
  
 16
 
  70323412
 
  70323915
 
  ENSEMBL   UCSC 
 
  504
 
  9
 
 15339
 
 tss
 
 0.002047
 
 0.0017836
 
 Not queried
 
  1.2075597
 
       NA
 
           NA
  
 
  
 7
 
 100421296
 
 100421510
 
  ENSEMBL   UCSC 
 
  215
 
  5
 
 37147
 
 gene;island
 
 0.707373
 
 0.8620180
 
 Not queried
 
  1.2074668
 
       NA
 
           NA
  
 
  
 1
 
   2063799
 
   2064216
 
  ENSEMBL   UCSC 
 
  418
 
  5
 
 63
 
 gene;island
 
 0.488036
 
 0.0631719
 
 Not queried
 
  1.2073440
 
       NA
 
           NA
  
 
  
 10
 
   1205563
 
   1205673
 
  ENSEMBL   UCSC 
 
  111
 
  6
 
 4915
 
 tss
 
 0.835614
 
 0.9478115
 
 Not queried
 
  1.2072849
 
       NA
 
           NA
  
 
  
 2
 
 233321600
 
 233321900
 
  ENSEMBL   UCSC 
 
  301
 
  4
 
 23062
 
 gene;island
 
 0.607044
 
 0.6884535
 
 Not queried
 
  1.2065090
 
       NA
 
           NA
  
 
  
 15
 
  66678782
 
  66679174
 
  ENSEMBL   UCSC 
 
  393
 
  8
 
 13748
 
 tss;island
 
 0.007878
 
 0.0047098
 
 Not queried
 
  1.2063688
 
       NA
 
           NA
  
 
  
 11
 
 134231621
 
 134232313
 
  ENSEMBL   UCSC 
 
  693
 
  7
 
 6998
 
 gene;island
 
 0.802397
 
 0.8922084
 
 Not queried
 
  1.2061169
 
       NA
 
           NA
  
 
  
 4
 
   6107021
 
   6107339
 
  ENSEMBL   UCSC 
 
  319
 
  5
 
 29925
 
 gene;island
 
 0.941375
 
 0.6740329
 
 Not queried
 
  1.2057633
 
       NA
 
           NA
  
 
  
 22
 
  50528179
 
  50528333
 
  ENSEMBL   UCSC 
 
  155
 
  7
 
 27246
 
 tss;island
 
 0.108282
 
 0.6495868
 
 Not queried
 
  1.2057089
 
       NA
 
           NA
  
 
  
 6
 
  31698047
 
  31698226
 
  ENSEMBL   UCSC 
 
  180
 
  7
 
 34833
 
 tss
 
 0.009355
 
 0.0339304
 
 Not queried
 
  1.2054883
 
       NA
 
           NA
  
 
  
 8
 
  19540210
 
  19540734
 
  ENSEMBL   UCSC 
 
  525
 
  8
 
 39572
 
 tss
 
 0.574553
 
 0.7064170
 
 Not queried
 
  1.2051270
 
       NA
 
           NA
  
 
  
 15
 
  65579119
 
  65579557
 
  ENSEMBL   UCSC 
 
  439
 
  7
 
 13742
 
 tss
 
 0.002112
 
 0.0046785
 
 Not queried
 
  1.2050036
 
       NA
 
           NA
  
 
  
 12
 
   1100377
 
   1100388
 
  ENSEMBL   UCSC 
 
   12
 
  4
 
 9482
 
 tss
 
 0.003880
 
 0.0066018
 
 Not queried
 
  1.2049116
 
       NA
 
           NA
  
 
  
 1
 
  88928168
 
  88928839
 
  ENSEMBL   UCSC 
 
  672
 
  6
 
 3443
 
 island
 
 0.220033
 
 0.3172347
 
 Not queried
 
  1.2048500
 
       NA
 
           NA
  
 
  
 1
 
 232941468
 
 232941775
 
  ENSEMBL   UCSC 
 
  308
 
  4
 
 1081
 
 gene;island
 
 0.453414
 
 0.0909337
 
 Not queried
 
  1.2047740
 
       NA
 
           NA
  
 
  
 1
 
  26758512
 
  26758790
 
  ENSEMBL   UCSC 
 
  279
 
  7
 
 1393
 
 tss
 
 0.005486
 
 0.0029587
 
 Not queried
 
  1.2046670
 
       NA
 
           NA
  
 
  
 12
 
   3600019
 
   3600317
 
  ENSEMBL   UCSC 
 
  299
 
  6
 
 9494
 
 tss;island
 
 0.018267
 
 0.0307482
 
 Not queried
 
  1.2046099
 
       NA
 
           NA
  
 
  
 1
 
   8271918
 
   8272277
 
  ENSEMBL   UCSC 
 
  360
 
  5
 
 2634
 
 island
 
 0.096691
 
 0.4590920
 
 Not queried
 
  1.2044643
 
       NA
 
           NA
  
 
  
 5
 
  63257499
 
  63257941
 
  ENSEMBL   UCSC 
 
  443
 
 10
 
 32827
 
 island
 
 0.046380
 
 0.1132165
 
 Not queried
 
  1.2043964
 
       NA
 
           NA
  
 
  
 6
 
  31650735
 
  31651362
 
  ENSEMBL   UCSC 
 
  628
 
 21
 
 35822
 
 island
 
 0.927653
 
 0.9712942
 
 Not queried
 
  1.2042723
 
       NA
 
           NA
  
 
  
 19
 
  33864279
 
  33864478
 
  ENSEMBL   UCSC 
 
  200
 
  8
 
 20684
 
 tss;island
 
 0.005136
 
 0.0042486
 
 Not queried
 
  1.2041748
 
       NA
 
           NA
  
 
  
 11
 
 120038521
 
 120039029
 
  ENSEMBL   UCSC 
 
  509
 
  5
 
 8889
 
 island
 
 0.717172
 
 0.8663955
 
 Not queried
 
  1.2038460
 
       NA
 
           NA
  
 
  
 11
 
  19736150
 
  19736333
 
  ENSEMBL   UCSC 
 
  184
 
  5
 
 6535
 
 gene;tss;island
 
 0.008396
 
 0.0070606
 
 Not queried
 
  1.2036075
 
       NA
 
           NA
  
 
  
 2
 
 176032401
 
 176032603
 
  ENSEMBL   UCSC 
 
  203
 
  8
 
 22933
 
 gene;island
 
 0.007335
 
 0.0030901
 
 Not queried
 
  1.2030069
 
       NA
 
           NA
  
 
  
 10
 
 133948124
 
 133948645
 
  ENSEMBL   UCSC 
 
  522
 
  6
 
 4851
 
 gene;island
 
 0.691838
 
 0.8405117
 
 Not queried
 
  1.2024445
 
       NA
 
           NA
  
 
  
 12
 
  85673860
 
  85674694
 
  ENSEMBL   UCSC 
 
  835
 
 11
 
 10658
 
 island
 
 0.072391
 
 0.0296182
 
 Not queried
 
  1.2024339
 
       NA
 
           NA
  
 
  
 1
 
 219347252
 
 219347458
 
  ENSEMBL   UCSC 
 
  207
 
  5
 
 1013
 
 gene
 
 0.010221
 
 0.0044630
 
 Not queried
 
  1.2021622
 
       NA
 
           NA
  
 
  
 19
 
  16435203
 
  16435500
 
  ENSEMBL   UCSC 
 
  298
 
  4
 
 20570
 
 tss
 
 0.020559
 
 0.0726253
 
 Not queried
 
  1.2020976
 
       NA
 
           NA
  
 
  
 17
 
  15244887
 
  15245237
 
  ENSEMBL   UCSC 
 
  351
 
  8
 
 18040
 
 island
 
 0.009637
 
 0.0140775
 
 Not queried
 
  1.2020528
 
       NA
 
           NA
  
 
  
 12
 
 121890163
 
 121890503
 
  ENSEMBL   UCSC 
 
  341
 
  4
 
 9368
 
 gene;island
 
 0.960238
 
 0.8296414
 
 Not queried
 
  1.2017688
 
       NA
 
           NA
  
 
  
 19
 
   9609152
 
   9609704
 
  ENSEMBL   UCSC 
 
  553
 
 10
 
 21499
 
 island
 
 0.084098
 
 0.2341836
 
 Not queried
 
  1.2015323
 
       NA
 
           NA
  
 
  
 1
 
  44056769
 
  44057099
 
  ENSEMBL   UCSC 
 
  331
 
  4
 
 481
 
 gene;island
 
 0.782950
 
 0.8925501
 
 Not queried
 
  1.2007301
 
       NA
 
           NA
  
 
  
 1
 
  76540110
 
  76540236
 
  ENSEMBL   UCSC 
 
  127
 
  5
 
 1702
 
 tss;island
 
 0.013727
 
 0.0078395
 
 Not queried
 
  1.2004269
 
       NA
 
           NA
  
 
  
 17
 
  33700180
 
  33700295
 
  ENSEMBL   UCSC 
 
  116
 
  4
 
 16519
 
 gene;island
 
 0.011237
 
 0.0092053
 
 Not queried
 
  1.2003627
 
       NA
 
           NA
  
 
  
 14
 
 101359793
 
 101360236
 
  ENSEMBL   UCSC 
 
  444
 
 11
 
 12615
 
 tss
 
 0.362718
 
 0.5602351
 
 Not queried
 
  1.2002724
 
       NA
 
           NA
  
 
  
 19
 
    571107
 
    571416
 
  ENSEMBL   UCSC 
 
  310
 
  5
 
 20274
 
 tss;island
 
 0.012626
 
 0.0057278
 
 Not queried
 
  1.2001554
 
       NA
 
           NA
  
 
  
 1
 
   3163341
 
   3163710
 
  ENSEMBL   UCSC 
 
  370
 
  4
 
 113
 
 gene;island
 
 0.273651
 
 0.4348865
 
 Not queried
 
  1.2000597
 
       NA
 
           NA
  
 
  
 11
 
 102187898
 
 102188539
 
  ENSEMBL   UCSC 
 
  642
 
 11
 
 8761
 
 island
 
 0.001289
 
 0.0050936
 
 Not queried
 
  1.2000580
 
       NA
 
           NA
  
 
  
 17
 
  80251443
 
  80251697
 
  ENSEMBL   UCSC 
 
  255
 
  4
 
 18982
 
 island
 
 0.699863
 
 0.5554675
 
 Not queried
 
  1.1998932
 
       NA
 
           NA
  
 
  
 4
 
  40817953
 
  40818214
 
  ENSEMBL   UCSC 
 
  262
 
  4
 
 29989
 
 gene
 
 0.946721
 
 0.9775928
 
 Not queried
 
  1.1997192
 
       NA
 
           NA
  
 
  
 21
 
  44037277
 
  44037470
 
  ENSEMBL   UCSC 
 
  194
 
  4
 
 26704
 
 island
 
 0.695338
 
 0.7997656
 
 Not queried
 
  1.1994871
 
       NA
 
           NA
  
 
  
 2
 
 207506975
 
 207507163
 
  ENSEMBL   UCSC 
 
  189
 
  5
 
 25036
 
 island
 
 0.132859
 
 0.2631150
 
 Not queried
 
  1.1994839
 
       NA
 
           NA
  
 
  
 20
 
  61002595
 
  61002866
 
  ENSEMBL   UCSC 
 
  272
 
  6
 
 25870
 
 tss
 
 0.240038
 
 0.3981879
 
 Not queried
 
  1.1993035
 
       NA
 
           NA
  
 
  
 17
 
  22051978
 
  22052191
 
  ENSEMBL   UCSC 
 
  214
 
  4
 
 18130
 
 island
 
 0.437031
 
 0.6068402
 
 Not queried
 
  1.1984127
 
       NA
 
           NA
  
 
  
 20
 
   6750617
 
   6750989
 
  ENSEMBL   UCSC 
 
  373
 
  5
 
 25354
 
 gene;island
 
 0.106820
 
 0.0501018
 
 Not queried
 
  1.1982561
 
       NA
 
           NA
  
 
  
 5
 
 172483354
 
 172483448
 
  ENSEMBL   UCSC 
 
   95
 
  4
 
 31901
 
 gene;island
 
 0.012318
 
 0.0065225
 
 Not queried
 
  1.1982197
 
       NA
 
           NA
  
 
  
 8
 
  58907018
 
  58907387
 
  ENSEMBL   UCSC 
 
  370
 
  6
 
 40321
 
 island
 
 0.007467
 
 0.0201738
 
 Not queried
 
  1.1976521
 
       NA
 
           NA
  
 
  
 1
 
 110210684
 
 110211023
 
  ENSEMBL   UCSC 
 
  340
 
  6
 
 673
 
 gene
 
 0.015286
 
 0.1052940
 
 Not queried
 
  1.1972263
 
       NA
 
           NA
  
 
  
 4
 
 132896266
 
 132896736
 
  ENSEMBL   UCSC 
 
  471
 
  5
 
 31305
 
 island
 
 0.687668
 
 0.7349003
 
 Not queried
 
  1.1970885
 
       NA
 
           NA
  
 
  
 12
 
   1609245
 
   1609549
 
  ENSEMBL   UCSC 
 
  305
 
  8
 
 9483
 
 tss
 
 0.345479
 
 0.4144115
 
 Not queried
 
  1.1970793
 
       NA
 
           NA
  
 
  
 6
 
 168393930
 
 168394198
 
  ENSEMBL   UCSC 
 
  269
 
  5
 
 36653
 
 island
 
 0.676613
 
 0.8363862
 
 Not queried
 
  1.1970089
 
       NA
 
           NA
  
 
  
 17
 
  45401148
 
  45401312
 
  ENSEMBL   UCSC 
 
  165
 
  4
 
 17441
 
 tss
 
 0.016603
 
 0.0137772
 
 Not queried
 
  1.1967390
 
       NA
 
           NA
  
 
  
 13
 
 108518152
 
 108518621
 
  ENSEMBL   UCSC 
 
  470
 
  6
 
 11220
 
 gene;island
 
 0.052864
 
 0.3044936
 
 Not queried
 
  1.1967121
 
       NA
 
           NA
  
 
  
 5
 
  41870746
 
  41871189
 
  ENSEMBL   UCSC 
 
  444
 
 10
 
 32755
 
 island
 
 0.014873
 
 0.1473889
 
 Not queried
 
  1.1962381
 
       NA
 
           NA
  
 
  
 17
 
  80668611
 
  80668944
 
  ENSEMBL   UCSC 
 
  334
 
  5
 
 19006
 
 island
 
 0.532491
 
 0.6385054
 
 Not queried
 
  1.1961230
 
       NA
 
           NA
  
 
  
 6
 
 153304236
 
 153304588
 
  ENSEMBL   UCSC 
 
  353
 
  7
 
 35307
 
 tss
 
 0.002071
 
 0.0042074
 
 Not queried
 
  1.1957655
 
       NA
 
           NA
  
 
  
 6
 
  31508106
 
  31508318
 
  ENSEMBL   UCSC 
 
  213
 
  6
 
 33894
 
 gene;island
 
 0.038359
 
 0.1313205
 
 Not queried
 
  1.1956340
 
       NA
 
           NA
  
 
  
 16
 
   3306524
 
   3307037
 
  ENSEMBL   UCSC 
 
  514
 
  4
 
 15643
 
 island
 
 0.240944
 
 0.3808718
 
 Not queried
 
  1.1951772
 
       NA
 
           NA
  
 
  
 3
 
  54157557
 
  54157900
 
  ENSEMBL   UCSC 
 
  344
 
  5
 
 27835
 
 gene;island
 
 0.006557
 
 0.0011236
 
 Not queried
 
  1.1951311
 
       NA
 
           NA
  
 
  
 16
 
   1179514
 
   1179941
 
  ENSEMBL   UCSC 
 
  428
 
  5
 
 15517
 
 island
 
 0.889705
 
 0.9486600
 
 Not queried
 
  1.1949723
 
       NA
 
           NA
  
 
  
 12
 
  98987143
 
  98987659
 
  ENSEMBL   UCSC 
 
  517
 
  8
 
 10706
 
 island
 
 0.002803
 
 0.0027778
 
 Not queried
 
  1.1946211
 
       NA
 
           NA
  
 
  
 11
 
   3253581
 
   3254504
 
  ENSEMBL   UCSC 
 
  924
 
 15
 
 7996
 
 island
 
 0.650550
 
 0.7546577
 
 Not queried
 
  1.1944898
 
       NA
 
           NA
  
 
  
 16
 
  89686618
 
  89687052
 
  ENSEMBL   UCSC 
 
  435
 
  5
 
 14947
 
 gene
 
 0.242116
 
 0.2855051
 
 Not queried
 
  1.1942074
 
       NA
 
           NA
  
 
  
 21
 
  44496012
 
  44496378
 
  ENSEMBL   UCSC 
 
  367
 
  6
 
 26713
 
 island
 
 0.007888
 
 0.0138011
 
 Not queried
 
  1.1942000
 
       NA
 
           NA
  
 
  
 16
 
   3450775
 
   3451006
 
  ENSEMBL   UCSC 
 
  232
 
  5
 
 14625
 
 gene
 
 0.000265
 
 0.0011010
 
 Not queried
 
  1.1938191
 
       NA
 
           NA
  
 
  
 8
 
 146277200
 
 146277480
 
  ENSEMBL   UCSC 
 
  281
 
  4
 
 39999
 
 tss;island
 
 0.018712
 
 0.0256684
 
 Not queried
 
  1.1935078
 
       NA
 
           NA
  
 
  
 7
 
 135433610
 
 135433790
 
  ENSEMBL   UCSC 
 
  181
 
  6
 
 37921
 
 tss
 
 0.452595
 
 0.9025587
 
 Not queried
 
  1.1934958
 
       NA
 
           NA
  
 
  
 1
 
  43919949
 
  43920264
 
  ENSEMBL   UCSC 
 
  316
 
  8
 
 1563
 
 tss
 
 0.018514
 
 0.0112002
 
 Not queried
 
  1.1928002
 
       NA
 
           NA
  
 
  
 4
 
 110223040
 
 110224288
 
  ENSEMBL   UCSC 
 
 1249
 
 20
 
 31235
 
 island
 
 0.046926
 
 0.1440251
 
 Not queried
 
  1.1916261
 
       NA
 
           NA
  
 
  
 17
 
  78152051
 
  78152232
 
  ENSEMBL   UCSC 
 
  182
 
  4
 
 17721
 
 tss
 
 0.852086
 
 0.9118334
 
 Not queried
 
  1.1916136
 
       NA
 
           NA
  
 
  
 5
 
 134363516
 
 134363973
 
  ENSEMBL   UCSC 
 
  458
 
  6
 
 31756
 
 gene
 
 0.251248
 
 0.1374660
 
 Not queried
 
  1.1915011
 
       NA
 
           NA
  
 
  
 11
 
   1036714
 
   1036938
 
  ENSEMBL   UCSC 
 
  225
 
  6
 
 7036
 
 tss
 
 0.072306
 
 0.1540231
 
 Not queried
 
  1.1914198
 
       NA
 
           NA
  
 
  
 11
 
  33182787
 
  33183473
 
  ENSEMBL   UCSC 
 
  687
 
 12
 
 8188
 
 island
 
 0.003199
 
 0.0014649
 
 Not queried
 
  1.1912899
 
       NA
 
           NA
  
 
  
 11
 
  50227079
 
  50227265
 
  ENSEMBL   UCSC 
 
  187
 
  4
 
 8278
 
 island
 
 0.687044
 
 0.9282837
 
 Not queried
 
  1.1911766
 
       NA
 
           NA
  
 
  
 9
 
 100615084
 
 100615357
 
  ENSEMBL   UCSC 
 
  274
 
  4
 
 40825
 
 tss;island
 
 0.053759
 
 0.0217634
 
 Not queried
 
  1.1911535
 
       NA
 
           NA
  
 
  
 5
 
   1100096
 
   1100544
 
  ENSEMBL   UCSC 
 
  449
 
  4
 
 31524
 
 gene;island
 
 0.983815
 
 0.9634498
 
 Not queried
 
  1.1910516
 
       NA
 
           NA
  
 
  
 3
 
  50336673
 
  50336875
 
  ENSEMBL   UCSC 
 
  203
 
  8
 
 27800
 
 gene
 
 0.003450
 
 0.0039064
 
 Not queried
 
  1.1908851
 
       NA
 
           NA
  
 
  
 19
 
  46438607
 
  46438944
 
  ENSEMBL   UCSC 
 
  338
 
  4
 
 22166
 
 island
 
 0.533943
 
 0.6891695
 
 Not queried
 
  1.1906711
 
       NA
 
           NA
  
 
  
 17
 
   9478950
 
   9479335
 
  ENSEMBL   UCSC 
 
  386
 
  6
 
 17126
 
 tss;island
 
 0.006284
 
 0.0077325
 
 Not queried
 
  1.1905673
 
       NA
 
           NA
  
 
  
 2
 
 161265247
 
 161265366
 
  ENSEMBL   UCSC 
 
  120
 
  4
 
 22908
 
 gene;island
 
 0.944425
 
 0.9756202
 
 Not queried
 
  1.1904729
 
       NA
 
           NA
  
 
  
 7
 
  90226284
 
  90226609
 
  ENSEMBL   UCSC 
 
  326
 
  6
 
 38650
 
 island
 
 0.017076
 
 0.0040415
 
 Not queried
 
  1.1902212
 
       NA
 
           NA
  
 
  
 19
 
   2858854
 
   2859153
 
  ENSEMBL   UCSC 
 
  300
 
  4
 
 21307
 
 island
 
 0.922663
 
 0.7067615
 
 Not queried
 
  1.1898151
 
       NA
 
           NA
  
 
  
 14
 
  65346490
 
  65346875
 
  ENSEMBL   UCSC 
 
  386
 
  6
 
 12942
 
 island
 
 0.023244
 
 0.0112292
 
 Not queried
 
  1.1895956
 
       NA
 
           NA
  
 
  
 3
 
  62362660
 
  62363248
 
  ENSEMBL   UCSC 
 
  589
 
  5
 
 29250
 
 island
 
 0.843913
 
 0.2518508
 
 Not queried
 
  1.1894722
 
       NA
 
           NA
  
 
  
 2
 
  11774142
 
  11774474
 
  ENSEMBL   UCSC 
 
  333
 
  5
 
 22573
 
 gene;island
 
 0.520849
 
 0.8737043
 
 Not queried
 
  1.1893038
 
       NA
 
           NA
  
 
  
 6
 
  31545836
 
  31546085
 
  ENSEMBL   UCSC 
 
  250
 
  4
 
 33904
 
 gene;island
 
 0.548453
 
 0.6277269
 
 Not queried
 
  1.1892342
 
       NA
 
           NA
  
 
  
 2
 
 239799720
 
 239799954
 
  ENSEMBL   UCSC 
 
  235
 
  4
 
 23099
 
 gene
 
 0.515004
 
 0.6511156
 
 Not queried
 
  1.1892047
 
       NA
 
           NA
  
 
  
 1
 
 152733025
 
 152733395
 
  ENSEMBL   UCSC 
 
  371
 
  6
 
 759
 
 gene;island
 
 0.443346
 
 0.6736265
 
 Not queried
 
  1.1886690
 
       NA
 
           NA
  
 
  
 4
 
   7593370
 
   7593585
 
  ENSEMBL   UCSC 
 
  216
 
  4
 
 29939
 
 gene;island
 
 0.498285
 
 0.7079224
 
 Not queried
 
  1.1883989
 
       NA
 
           NA
  
 
  
 1
 
  64058883
 
  64059052
 
  ENSEMBL   UCSC 
 
  170
 
  4
 
 3346
 
 island
 
 0.014636
 
 0.0063542
 
 Not queried
 
  1.1881044
 
       NA
 
           NA
  
 
  
 10
 
   1415977
 
   1416224
 
  ENSEMBL   UCSC 
 
  248
 
  4
 
 4461
 
 gene;island
 
 0.855789
 
 0.9318362
 
 Not queried
 
  1.1880754
 
       NA
 
           NA
  
 
  
 5
 
  63802491
 
  63802993
 
  ENSEMBL   UCSC 
 
  503
 
  5
 
 31628
 
 gene
 
 0.023145
 
 0.0122756
 
 Not queried
 
  1.1880318
 
       NA
 
           NA
  
 
  
 3
 
 111804830
 
 111805408
 
  ENSEMBL   UCSC 
 
  579
 
 12
 
 29357
 
 island
 
 0.040620
 
 0.0180983
 
 Not queried
 
  1.1875304
 
       NA
 
           NA
  
 
  
 10
 
  81145959
 
  81146193
 
  ENSEMBL   UCSC 
 
  235
 
  4
 
 4651
 
 gene;island
 
 0.774293
 
 0.8963262
 
 Not queried
 
  1.1874767
 
       NA
 
           NA
  
 
  
 8
 
 124253706
 
 124253829
 
  ENSEMBL   UCSC 
 
  124
 
  4
 
 39897
 
 tss
 
 0.036962
 
 0.0217386
 
 Not queried
 
  1.1874458
 
       NA
 
           NA
  
 
  
 13
 
  25861182
 
  25861655
 
  ENSEMBL   UCSC 
 
  474
 
  5
 
 11108
 
 gene
 
 0.002887
 
 0.0112577
 
 Not queried
 
  1.1871705
 
       NA
 
           NA
  
 
  
 12
 
    298151
 
    298484
 
  ENSEMBL   UCSC 
 
  334
 
  4
 
 10141
 
 island
 
 0.224415
 
 0.4127253
 
 Not queried
 
  1.1870783
 
       NA
 
           NA
  
 
  
 14
 
 101348035
 
 101348236
 
  ENSEMBL   UCSC 
 
  202
 
  5
 
 12211
 
 gene;tss;island
 
 0.781111
 
 0.8865395
 
 Not queried
 
  1.1865052
 
       NA
 
           NA
  
 
  
 2
 
  36582038
 
  36582467
 
  ENSEMBL   UCSC 
 
  430
 
  5
 
 23301
 
 tss;island
 
 0.470426
 
 0.2806862
 
 Not queried
 
  1.1864328
 
       NA
 
           NA
  
 
  
 19
 
  49220102
 
  49220485
 
  ENSEMBL   UCSC 
 
  384
 
  6
 
 20135
 
 gene;island
 
 0.651043
 
 0.8891392
 
 Not queried
 
  1.1862906
 
       NA
 
           NA
  
 
  
 11
 
  18477153
 
  18477303
 
  ENSEMBL   UCSC 
 
  151
 
  5
 
 7185
 
 tss
 
 0.622861
 
 0.5092031
 
 Not queried
 
  1.1862644
 
       NA
 
           NA
  
 
  
 2
 
  97173713
 
  97174391
 
  ENSEMBL   UCSC 
 
  679
 
 12
 
 24525
 
 island
 
 0.011216
 
 0.0344151
 
 Not queried
 
  1.1860113
 
       NA
 
           NA
  
 
  
 12
 
  12509705
 
  12509963
 
  ENSEMBL   UCSC 
 
  259
 
  6
 
 9057
 
 gene
 
 0.002966
 
 0.0043779
 
 Not queried
 
  1.1859628
 
       NA
 
           NA
  
 
  
 2
 
 201980504
 
 201980910
 
  ENSEMBL   UCSC 
 
  407
 
  8
 
 25007
 
 island
 
 0.015712
 
 0.0525537
 
 Not queried
 
  1.1858073
 
       NA
 
           NA
  
 
  
 11
 
  64884962
 
  64885558
 
  ENSEMBL   UCSC 
 
  597
 
 11
 
 8438
 
 island
 
 0.005300
 
 0.0030205
 
 Not queried
 
  1.1853297
 
       NA
 
           NA
  
 
  
 4
 
  87515184
 
  87515700
 
  ENSEMBL   UCSC 
 
  517
 
 12
 
 31164
 
 island
 
 0.019422
 
 0.0444461
 
 Not queried
 
  1.1852559
 
       NA
 
           NA
  
 
  
 17
 
  74721824
 
  74722097
 
  ENSEMBL   UCSC 
 
  274
 
  6
 
 16793
 
 gene;tss;island
 
 0.043937
 
 0.0782897
 
 Not queried
 
  1.1852021
 
       NA
 
           NA
  
 
  
 11
 
  27527925
 
  27528142
 
  ENSEMBL   UCSC 
 
  218
 
  4
 
 6542
 
 gene;tss;island
 
 0.004950
 
 0.0156667
 
 Not queried
 
  1.1851889
 
       NA
 
           NA
  
 
  
 7
 
   4254699
 
   4255011
 
  ENSEMBL   UCSC 
 
  313
 
  4
 
 36835
 
 gene
 
 0.356074
 
 0.5556220
 
 Not queried
 
  1.1850601
 
       NA
 
           NA
  
 
  
 16
 
  89883185
 
  89883359
 
  ENSEMBL   UCSC 
 
  175
 
  8
 
 15431
 
 tss;island
 
 0.008188
 
 0.0105638
 
 Not queried
 
  1.1849700
 
       NA
 
           NA
  
 
  
 1
 
 156561646
 
 156561947
 
  ENSEMBL   UCSC 
 
  302
 
  4
 
 824
 
 gene;island
 
 0.035073
 
 0.1145930
 
 Not queried
 
  1.1843154
 
       NA
 
           NA
  
 
  
 6
 
  30579288
 
  30579650
 
  ENSEMBL   UCSC 
 
  363
 
  4
 
 33784
 
 gene
 
 0.983459
 
 0.9708312
 
 Not queried
 
  1.1841456
 
       NA
 
           NA
  
 
  
 17
 
    710582
 
    710977
 
  ENSEMBL   UCSC 
 
  396
 
  6
 
 16296
 
 gene;island
 
 0.774325
 
 0.8279759
 
 Not queried
 
  1.1839994
 
       NA
 
           NA
  
 
  
 16
 
  85116332
 
  85116513
 
  ENSEMBL   UCSC 
 
  182
 
  4
 
 14875
 
 gene
 
 0.050906
 
 0.0767814
 
 Not queried
 
  1.1836941
 
       NA
 
           NA
  
 
  
 5
 
 177558089
 
 177558283
 
  ENSEMBL   UCSC 
 
  195
 
  4
 
 31924
 
 gene
 
 0.015868
 
 0.0139799
 
 Not queried
 
  1.1835467
 
       NA
 
           NA
  
 
  
 6
 
 168388195
 
 168388388
 
  ENSEMBL   UCSC 
 
  194
 
  4
 
 36651
 
 island
 
 0.600419
 
 0.7770069
 
 Not queried
 
  1.1834724
 
       NA
 
           NA
  
 
  
 2
 
 177028606
 
 177028714
 
  ENSEMBL   UCSC 
 
  109
 
  4
 
 23742
 
 tss
 
 0.007537
 
 0.0084398
 
 Not queried
 
  1.1830402
 
       NA
 
           NA
  
 
  
 17
 
  80179899
 
  80180166
 
  ENSEMBL   UCSC 
 
  268
 
  4
 
 18975
 
 island
 
 0.674163
 
 0.7966526
 
 Not queried
 
  1.1825421
 
       NA
 
           NA
  
 
  
 20
 
   4721490
 
   4721766
 
  ENSEMBL   UCSC 
 
  277
 
  4
 
 25576
 
 tss
 
 0.028705
 
 0.0039306
 
 Not queried
 
  1.1824694
 
       NA
 
           NA
  
 
  
 2
 
 237080267
 
 237080535
 
  ENSEMBL   UCSC 
 
  269
 
  4
 
 25226
 
 island
 
 0.032188
 
 0.1468426
 
 Not queried
 
  1.1821152
 
       NA
 
           NA
  
 
  
 17
 
  75471193
 
  75471308
 
  ENSEMBL   UCSC 
 
  116
 
  4
 
 17699
 
 tss
 
 0.006222
 
 0.0132146
 
 Not queried
 
  1.1820883
 
       NA
 
           NA
  
 
  
 8
 
  77592946
 
  77593623
 
  ENSEMBL   UCSC 
 
  678
 
  8
 
 39342
 
 gene;island
 
 0.193881
 
 0.0464622
 
 Not queried
 
  1.1819611
 
       NA
 
           NA
  
 
  
 15
 
  45421578
 
  45422095
 
  ENSEMBL   UCSC 
 
  518
 
 11
 
 13646
 
 tss;island
 
 0.005454
 
 0.0328874
 
 Not queried
 
  1.1819333
 
       NA
 
           NA
  
 
  
 6
 
  32027379
 
  32027666
 
  ENSEMBL   UCSC 
 
  288
 
  7
 
 34024
 
 gene
 
 0.394797
 
 0.5009334
 
 Not queried
 
  1.1818036
 
       NA
 
           NA
  
 
  
 20
 
  30406997
 
  30407388
 
  ENSEMBL   UCSC 
 
  392
 
  9
 
 26080
 
 island
 
 0.515119
 
 0.7951919
 
 Not queried
 
  1.1817522
 
       NA
 
           NA
  
 
  
 12
 
  16757954
 
  16758267
 
  ENSEMBL   UCSC 
 
  314
 
  6
 
 9071
 
 gene
 
 0.321960
 
 0.1374252
 
 Not queried
 
  1.1817508
 
       NA
 
           NA
  
 
  
 1
 
 154934627
 
 154934734
 
  ENSEMBL   UCSC 
 
  108
 
  4
 
 1971
 
 tss;island
 
 0.004006
 
 0.0092109
 
 Not queried
 
  1.1811930
 
       NA
 
           NA
  
 
  
 22
 
  41763093
 
  41763370
 
  ENSEMBL   UCSC 
 
  278
 
  7
 
 27170
 
 tss
 
 0.041550
 
 0.1598893
 
 Not queried
 
  1.1811176
 
       NA
 
           NA
  
 
  
 1
 
  91176405
 
  91176810
 
  ENSEMBL   UCSC 
 
  406
 
  5
 
 3456
 
 island
 
 0.074057
 
 0.0162514
 
 Not queried
 
  1.1810899
 
       NA
 
           NA
  
 
  
 2
 
    496713
 
    497417
 
  ENSEMBL   UCSC 
 
  705
 
  7
 
 24004
 
 island
 
 0.546877
 
 0.6914535
 
 Not queried
 
  1.1808633
 
       NA
 
           NA
  
 
  
 4
 
 155702172
 
 155702411
 
  ENSEMBL   UCSC 
 
  240
 
  7
 
 30648
 
 tss
 
 0.577255
 
 0.9228904
 
 Not queried
 
  1.1807680
 
       NA
 
           NA
  
 
  
 15
 
  69744390
 
  69745373
 
  ENSEMBL   UCSC 
 
  984
 
 14
 
 14266
 
 island
 
 0.015680
 
 0.0047402
 
 Not queried
 
  1.1794424
 
       NA
 
           NA
  
 
  
 19
 
  13985408
 
  13985587
 
  ENSEMBL   UCSC 
 
  180
 
  4
 
 20531
 
 tss
 
 0.632594
 
 0.7821732
 
 Not queried
 
  1.1793312
 
       NA
 
           NA
  
 
  
 1
 
 181382639
 
 181382934
 
  ENSEMBL   UCSC 
 
  296
 
  4
 
 4017
 
 island
 
 0.553975
 
 0.4221317
 
 Not queried
 
  1.1793098
 
       NA
 
           NA
  
 
  
 19
 
    913499
 
    913714
 
  ENSEMBL   UCSC 
 
  216
 
  4
 
 20284
 
 tss;island
 
 0.004761
 
 0.0041122
 
 Not queried
 
  1.1789551
 
       NA
 
           NA
  
 
  
 11
 
 134216065
 
 134216220
 
  ENSEMBL   UCSC 
 
  156
 
  4
 
 6997
 
 gene;island
 
 0.553133
 
 0.7313481
 
 Not queried
 
  1.1789544
 
       NA
 
           NA
  
 
  
 18
 
  52495404
 
  52495796
 
  ENSEMBL   UCSC 
 
  393
 
  8
 
 19284
 
 tss
 
 0.069155
 
 0.0472507
 
 Not queried
 
  1.1787808
 
       NA
 
           NA
  
 
  
 1
 
 115300693
 
 115300877
 
  ENSEMBL   UCSC 
 
  185
 
  5
 
 1853
 
 tss
 
 0.010311
 
 0.0027371
 
 Not queried
 
  1.1787239
 
       NA
 
           NA
  
 
  
 17
 
  46804123
 
  46804414
 
  ENSEMBL   UCSC 
 
  292
 
  4
 
 16665
 
 gene;island
 
 0.047111
 
 0.0219332
 
 Not queried
 
  1.1782538
 
       NA
 
           NA
  
 
  
 13
 
  20797356
 
  20797693
 
  ENSEMBL   UCSC 
 
  338
 
  5
 
 11095
 
 gene
 
 0.793302
 
 0.9253905
 
 Not queried
 
  1.1778810
 
       NA
 
           NA
  
 
  
 18
 
  21718735
 
  21718979
 
  ENSEMBL   UCSC 
 
  245
 
 10
 
 19209
 
 tss;island
 
 0.023679
 
 0.0115321
 
 Not queried
 
  1.1777490
 
       NA
 
           NA
  
 
  
 5
 
 110848437
 
 110848688
 
  ENSEMBL   UCSC 
 
  252
 
  4
 
 32208
 
 tss;island
 
 0.005988
 
 0.0110770
 
 Not queried
 
  1.1776939
 
       NA
 
           NA
  
 
  
 21
 
  34602027
 
  34602157
 
  ENSEMBL   UCSC 
 
  131
 
  5
 
 26518
 
 tss;island
 
 0.003506
 
 0.0044352
 
 Not queried
 
  1.1773930
 
       NA
 
           NA
  
 
  
 10
 
   5406890
 
   5406971
 
  ENSEMBL   UCSC 
 
   82
 
  6
 
 4922
 
 tss
 
 0.391495
 
 0.6300658
 
 Not queried
 
  1.1772923
 
       NA
 
           NA
  
 
  
 8
 
 101118377
 
 101118548
 
  ENSEMBL   UCSC 
 
  172
 
  4
 
 39839
 
 tss;island
 
 0.185872
 
 0.1135050
 
 Not queried
 
  1.1772498
 
       NA
 
           NA
  
 
  
 1
 
  68299388
 
  68299511
 
  ENSEMBL   UCSC 
 
  124
 
  5
 
 1685
 
 tss;island
 
 0.392491
 
 0.4447220
 
 Not queried
 
  1.1770284
 
       NA
 
           NA
  
 
  
 19
 
  54485321
 
  54485509
 
  ENSEMBL   UCSC 
 
  189
 
  6
 
 20213
 
 gene;tss;island
 
 0.245053
 
 0.4448057
 
 Not queried
 
  1.1767819
 
       NA
 
           NA
  
 
  
 14
 
 101198681
 
 101198811
 
  ENSEMBL   UCSC 
 
  131
 
  4
 
 12208
 
 gene;island
 
 0.871050
 
 0.9395320
 
 Not queried
 
  1.1767062
 
       NA
 
           NA
  
 
  
 7
 
  99595095
 
  99595458
 
  ENSEMBL   UCSC 
 
  364
 
  5
 
 38742
 
 island
 
 0.062180
 
 0.1559614
 
 Not queried
 
  1.1766679
 
       NA
 
           NA
  
 
  
 13
 
 113739251
 
 113739577
 
  ENSEMBL   UCSC 
 
  327
 
  4
 
 11275
 
 gene;island
 
 0.426813
 
 0.6288947
 
 Not queried
 
  1.1766230
 
       NA
 
           NA
  
 
  
 8
 
   1051510
 
   1051814
 
  ENSEMBL   UCSC 
 
  305
 
  5
 
 40011
 
 island
 
 0.284676
 
 0.5007726
 
 Not queried
 
  1.1765196
 
       NA
 
           NA
  
 
  
 15
 
  61521674
 
  61522112
 
  ENSEMBL   UCSC 
 
  439
 
  7
 
 13713
 
 tss;island
 
 0.013274
 
 0.0329152
 
 Not queried
 
  1.1763668
 
       NA
 
           NA
  
 
  
 6
 
  40567317
 
  40567579
 
  ENSEMBL   UCSC 
 
  263
 
  4
 
 36114
 
 island
 
 0.346522
 
 0.5382160
 
 Not queried
 
  1.1763229
 
       NA
 
           NA
  
 
  
 15
 
  63115816
 
  63116127
 
  ENSEMBL   UCSC 
 
  312
 
  6
 
 13371
 
 gene;tss
 
 0.474946
 
 0.6106920
 
 Not queried
 
  1.1756858
 
       NA
 
           NA
  
 
  
 6
 
 125420676
 
 125421084
 
  ENSEMBL   UCSC 
 
  409
 
  4
 
 36457
 
 island
 
 0.017974
 
 0.0364737
 
 Not queried
 
  1.1754043
 
       NA
 
           NA
  
 
  
 5
 
 133451237
 
 133451625
 
  ENSEMBL   UCSC 
 
  389
 
  8
 
 31750
 
 gene;island
 
 0.020122
 
 0.1085799
 
 Not queried
 
  1.1753096
 
       NA
 
           NA
  
 
  
 2
 
 176971820
 
 176972068
 
  ENSEMBL   UCSC 
 
  249
 
  7
 
 23734
 
 tss
 
 0.101011
 
 0.2392038
 
 Not queried
 
  1.1751352
 
       NA
 
           NA
  
 
  
 8
 
  11725943
 
  11726286
 
  ENSEMBL   UCSC 
 
  344
 
  4
 
 39556
 
 tss;island
 
 0.055717
 
 0.0396703
 
 Not queried
 
  1.1748186
 
       NA
 
           NA
  
 
  
 5
 
  57878745
 
  57879417
 
  ENSEMBL   UCSC 
 
  673
 
 12
 
 32817
 
 island
 
 0.064409
 
 0.0251455
 
 Not queried
 
  1.1742588
 
       NA
 
           NA
  
 
  
 17
 
  79107083
 
  79107259
 
  ENSEMBL   UCSC 
 
  177
 
  4
 
 16895
 
 gene;island
 
 0.480719
 
 0.5496504
 
 Not queried
 
  1.1741499
 
       NA
 
           NA
  
 
  
 6
 
   6588693
 
   6588931
 
  ENSEMBL   UCSC 
 
  239
 
  4
 
 34610
 
 tss
 
 0.002340
 
 0.0012756
 
 Not queried
 
  1.1741341
 
       NA
 
           NA
  
 
  
 13
 
 100624207
 
 100624373
 
  ENSEMBL   UCSC 
 
  167
 
  7
 
 11515
 
 tss
 
 0.507768
 
 0.1744772
 
 Not queried
 
  1.1740491
 
       NA
 
           NA
  
 
  
 13
 
 100623942
 
 100624373
 
  ENSEMBL   UCSC 
 
  432
 
  9
 
 11845
 
 island
 
 0.332282
 
 0.1549173
 
 Not queried
 
  1.1740491
 
       NA
 
           NA
  
 
  
 19
 
  18314906
 
  18315256
 
  ENSEMBL   UCSC 
 
  351
 
  6
 
 20613
 
 tss
 
 0.003203
 
 0.0020943
 
 Not queried
 
  1.1738777
 
       NA
 
           NA
  
 
  
 17
 
   7137923
 
   7138078
 
  ENSEMBL   UCSC 
 
  156
 
  5
 
 17064
 
 tss
 
 0.004765
 
 0.0032407
 
 Not queried
 
  1.1738717
 
       NA
 
           NA
  
 
  
 10
 
  30317625
 
  30317994
 
  ENSEMBL   UCSC 
 
  370
 
  5
 
 4548
 
 gene
 
 0.735259
 
 0.8650997
 
 Not queried
 
  1.1735566
 
       NA
 
           NA
  
 
  
 4
 
  56659770
 
  56660460
 
  ENSEMBL   UCSC 
 
  691
 
  8
 
 31061
 
 island
 
 0.177766
 
 0.6417913
 
 Not queried
 
  1.1733208
 
       NA
 
           NA
  
 
  
 17
 
  81047627
 
  81047941
 
  ENSEMBL   UCSC 
 
  315
 
  4
 
 16976
 
 gene;island
 
 0.814492
 
 0.8835368
 
 Not queried
 
  1.1729546
 
       NA
 
           NA
  
 
  
 12
 
 101603174
 
 101603581
 
  ENSEMBL   UCSC 
 
  408
 
  4
 
 9266
 
 gene;island
 
 0.182730
 
 0.0939679
 
 Not queried
 
  1.1725375
 
       NA
 
           NA
  
 
  
 10
 
 102419209
 
 102419617
 
  ENSEMBL   UCSC 
 
  409
 
  6
 
 6029
 
 island
 
 0.084740
 
 0.1800302
 
 Not queried
 
  1.1724654
 
       NA
 
           NA
  
 
  
 16
 
  46655096
 
  46655473
 
  ENSEMBL   UCSC 
 
  378
 
  6
 
 15894
 
 island
 
 0.001737
 
 0.0008721
 
 Not queried
 
  1.1724511
 
       NA
 
           NA
  
 
  
 4
 
 175138997
 
 175139240
 
  ENSEMBL   UCSC 
 
  244
 
  4
 
 31434
 
 island
 
 0.007186
 
 0.0111593
 
 Not queried
 
  1.1720717
 
       NA
 
           NA
  
 
  
 2
 
 119603383
 
 119603969
 
  ENSEMBL   UCSC 
 
  587
 
  5
 
 22841
 
 gene;island
 
 0.018805
 
 0.0452476
 
 Not queried
 
  1.1719428
 
       NA
 
           NA
  
 
  
 1
 
  34098656
 
  34099118
 
  ENSEMBL   UCSC 
 
  463
 
  4
 
 406
 
 gene;island
 
 0.018672
 
 0.1506118
 
 Not queried
 
  1.1717672
 
       NA
 
           NA
  
 
  
 6
 
 170591749
 
 170592108
 
  ENSEMBL   UCSC 
 
  360
 
  6
 
 34581
 
 gene;island
 
 0.899486
 
 0.9528200
 
 Not queried
 
  1.1717353
 
       NA
 
           NA
  
 
  
 4
 
 155702172
 
 155702610
 
  ENSEMBL   UCSC 
 
  439
 
  9
 
 31388
 
 island
 
 0.568223
 
 0.4757184
 
 Not queried
 
  1.1716289
 
       NA
 
           NA
  
 
  
 17
 
  74497614
 
  74497851
 
  ENSEMBL   UCSC 
 
  238
 
  4
 
 17680
 
 tss
 
 0.012651
 
 0.0090909
 
 Not queried
 
  1.1714986
 
       NA
 
           NA
  
 
  
 21
 
  42218932
 
  42219299
 
  ENSEMBL   UCSC 
 
  368
 
  8
 
 26686
 
 island
 
 0.242497
 
 0.4127262
 
 Not queried
 
  1.1714325
 
       NA
 
           NA
  
 
  
 12
 
 103358619
 
 103358881
 
  ENSEMBL   UCSC 
 
  263
 
  6
 
 10729
 
 island
 
 0.188932
 
 0.0416586
 
 Not queried
 
  1.1709594
 
       NA
 
           NA
  
 
  
 5
 
    892523
 
    892775
 
  ENSEMBL   UCSC 
 
  253
 
  8
 
 31514
 
 gene;tss;island
 
 0.002054
 
 0.0027236
 
 Not queried
 
  1.1708619
 
       NA
 
           NA
  
 
  
 6
 
  76059450
 
  76059773
 
  ENSEMBL   UCSC 
 
  324
 
  6
 
 34396
 
 gene
 
 0.066972
 
 0.0155550
 
 Not queried
 
  1.1704887
 
       NA
 
           NA
  
 
  
 16
 
  29757344
 
  29757461
 
  ENSEMBL   UCSC 
 
  118
 
  4
 
 15167
 
 tss
 
 0.021504
 
 0.0102827
 
 Not queried
 
  1.1704587
 
       NA
 
           NA
  
 
  
 17
 
  37844398
 
  37844579
 
  ENSEMBL   UCSC 
 
  182
 
  5
 
 16552
 
 gene
 
 0.008782
 
 0.0019360
 
 Not queried
 
  1.1700300
 
       NA
 
           NA
  
 
  
 7
 
 157402263
 
 157402673
 
  ENSEMBL   UCSC 
 
  411
 
  5
 
 37331
 
 gene;island
 
 0.365795
 
 0.5301683
 
 Not queried
 
  1.1699153
 
       NA
 
           NA
  
 
  
 6
 
  29364418
 
  29365287
 
  ENSEMBL   UCSC 
 
  870
 
 11
 
 33654
 
 gene
 
 0.573787
 
 0.7096100
 
 Not queried
 
  1.1698101
 
       NA
 
           NA
  
 
  
 22
 
  31090071
 
  31090352
 
  ENSEMBL   UCSC 
 
  282
 
  4
 
 27069
 
 tss;island
 
 0.040628
 
 0.0229588
 
 Not queried
 
  1.1696374
 
       NA
 
           NA
  
 
  
 2
 
 241974858
 
 241975140
 
  ENSEMBL   UCSC 
 
  283
 
  4
 
 23127
 
 gene;island
 
 0.947860
 
 0.9683082
 
 Not queried
 
  1.1694863
 
       NA
 
           NA
  
 
  
 19
 
  58458572
 
  58458979
 
  ENSEMBL   UCSC 
 
  408
 
  4
 
 20255
 
 gene
 
 0.060290
 
 0.0198716
 
 Not queried
 
  1.1694527
 
       NA
 
           NA
  
 
  
 16
 
  56691551
 
  56692077
 
  ENSEMBL   UCSC 
 
  527
 
 10
 
 15953
 
 island
 
 0.006245
 
 0.0252415
 
 Not queried
 
  1.1693824
 
       NA
 
           NA
  
 
  
 1
 
 203055422
 
 203055662
 
  ENSEMBL   UCSC 
 
  241
 
  4
 
 2189
 
 tss
 
 0.178068
 
 0.4156675
 
 Not queried
 
  1.1689133
 
       NA
 
           NA
  
 
  
 14
 
  37051693
 
  37051949
 
  ENSEMBL   UCSC 
 
  257
 
  7
 
 12787
 
 island
 
 0.007974
 
 0.0189459
 
 Not queried
 
  1.1688627
 
       NA
 
           NA
  
 
  
 17
 
  37322330
 
  37322730
 
  ENSEMBL   UCSC 
 
  401
 
  4
 
 18267
 
 island
 
 0.458204
 
 0.4804693
 
 Not queried
 
  1.1687627
 
       NA
 
           NA
  
 
  
 17
 
  79859809
 
  79859999
 
  ENSEMBL   UCSC 
 
  191
 
  7
 
 17754
 
 tss;island
 
 0.069169
 
 0.2314180
 
 Not queried
 
  1.1687259
 
       NA
 
           NA
  
 
  
 5
 
 132948185
 
 132948515
 
  ENSEMBL   UCSC 
 
  331
 
  6
 
 33108
 
 island
 
 0.017982
 
 0.0071919
 
 Not queried
 
  1.1685727
 
       NA
 
           NA
  
 
  
 4
 
  95678703
 
  95678939
 
  ENSEMBL   UCSC 
 
  237
 
  5
 
 30504
 
 tss;island
 
 0.023536
 
 0.1135221
 
 Not queried
 
  1.1683259
 
       NA
 
           NA
  
 
  
 17
 
  72580901
 
  72581081
 
  ENSEMBL   UCSC 
 
  181
 
  5
 
 16759
 
 gene
 
 0.502938
 
 0.6993584
 
 Not queried
 
  1.1681177
 
       NA
 
           NA
  
 
  
 21
 
  39047670
 
  39047966
 
  ENSEMBL   UCSC 
 
  297
 
  5
 
 26424
 
 gene;island
 
 0.057061
 
 0.0187751
 
 Not queried
 
  1.1679715
 
       NA
 
           NA
  
 
  
 17
 
  79818544
 
  79818808
 
  ENSEMBL   UCSC 
 
  265
 
  5
 
 17750
 
 tss
 
 0.002875
 
 0.0049493
 
 Not queried
 
  1.1678703
 
       NA
 
           NA
  
 
  
 19
 
  44575997
 
  44576295
 
  ENSEMBL   UCSC 
 
  299
 
 10
 
 20841
 
 tss;island
 
 0.005938
 
 0.0108015
 
 Not queried
 
  1.1677649
 
       NA
 
           NA
  
 
  
 17
 
  79935740
 
  79936123
 
  ENSEMBL   UCSC 
 
  384
 
  4
 
 16933
 
 gene;island
 
 0.004118
 
 0.0074787
 
 Not queried
 
  1.1675748
 
       NA
 
           NA
  
 
  
 14
 
 105045877
 
 105045992
 
  ENSEMBL   UCSC 
 
  116
 
  4
 
 12659
 
 tss
 
 0.269053
 
 0.4003291
 
 Not queried
 
  1.1675683
 
       NA
 
           NA
  
 
  
 1
 
  35258435
 
  35258933
 
  ENSEMBL   UCSC 
 
  499
 
  4
 
 3025
 
 island
 
 0.040863
 
 0.1589417
 
 Not queried
 
  1.1673999
 
       NA
 
           NA
  
 
  
 6
 
  31088188
 
  31088434
 
  ENSEMBL   UCSC 
 
  247
 
  5
 
 33850
 
 gene
 
 0.440157
 
 0.5371105
 
 Not queried
 
  1.1673417
 
       NA
 
           NA
  
 
  
 13
 
 112721712
 
 112721950
 
  ENSEMBL   UCSC 
 
  239
 
  7
 
 11910
 
 island
 
 0.170758
 
 0.2557774
 
 Not queried
 
  1.1672023
 
       NA
 
           NA
  
 
  
 7
 
   2742274
 
   2742484
 
  ENSEMBL   UCSC 
 
  211
 
  5
 
 36814
 
 gene;island
 
 0.946593
 
 0.9121753
 
 Not queried
 
  1.1670758
 
       NA
 
           NA
  
 
  
 4
 
   1046821
 
   1047097
 
  ENSEMBL   UCSC 
 
  277
 
  4
 
 30764
 
 island
 
 0.583772
 
 0.7573655
 
 Not queried
 
  1.1666315
 
       NA
 
           NA
  
 
  
 11
 
   2907670
 
   2908471
 
  ENSEMBL   UCSC 
 
  802
 
 14
 
 7081
 
 tss;island
 
 0.385869
 
 0.6877515
 
 Not queried
 
  1.1665197
 
       NA
 
           NA
  
 
  
 13
 
  51417686
 
  51418221
 
  ENSEMBL   UCSC 
 
  536
 
 10
 
 11745
 
 island
 
 0.663577
 
 0.4816651
 
 Not queried
 
  1.1662835
 
       NA
 
           NA
  
 
  
 19
 
  52674949
 
  52675470
 
  ENSEMBL   UCSC 
 
  522
 
 10
 
 21008
 
 tss
 
 0.048278
 
 0.0419922
 
 Not queried
 
  1.1660769
 
       NA
 
           NA
  
 
  
 5
 
  79286648
 
  79287008
 
  ENSEMBL   UCSC 
 
  361
 
  4
 
 31661
 
 gene
 
 0.080135
 
 0.1139190
 
 Not queried
 
  1.1657153
 
       NA
 
           NA
  
 
  
 15
 
  66790240
 
  66790410
 
  ENSEMBL   UCSC 
 
  171
 
  4
 
 13749
 
 tss
 
 0.014310
 
 0.0046703
 
 Not queried
 
  1.1655913
 
       NA
 
           NA
  
 
  
 19
 
  51774377
 
  51774666
 
  ENSEMBL   UCSC 
 
  290
 
  5
 
 22339
 
 island
 
 0.660676
 
 0.7501103
 
 Not queried
 
  1.1653437
 
       NA
 
           NA
  
 
  
 12
 
  54357408
 
  54357771
 
  ENSEMBL   UCSC 
 
  364
 
  5
 
 9155
 
 gene;island
 
 0.014375
 
 0.1007004
 
 Not queried
 
  1.1651978
 
       NA
 
           NA
  
 
  
 19
 
  16528764
 
  16529096
 
  ENSEMBL   UCSC 
 
  333
 
  4
 
 19911
 
 gene;island
 
 0.318347
 
 0.5172990
 
 Not queried
 
  1.1651817
 
       NA
 
           NA
  
 
  
 17
 
   7311620
 
   7312081
 
  ENSEMBL   UCSC 
 
  462
 
  6
 
 16394
 
 gene;island
 
 0.736582
 
 0.7217630
 
 Not queried
 
  1.1650184
 
       NA
 
           NA
  
 
  
 19
 
  54290737
 
  54291194
 
  ENSEMBL   UCSC 
 
  458
 
 11
 
 21039
 
 tss
 
 0.837774
 
 0.8961279
 
 Not queried
 
  1.1649261
 
       NA
 
           NA
  
 
  
 12
 
  12715438
 
  12715811
 
  ENSEMBL   UCSC 
 
  374
 
  5
 
 9571
 
 tss;island
 
 0.006351
 
 0.0075559
 
 Not queried
 
  1.1645124
 
       NA
 
           NA
  
 
  
 1
 
 212457915
 
 212458162
 
  ENSEMBL   UCSC 
 
  248
 
  4
 
 2243
 
 tss;island
 
 0.004630
 
 0.0073321
 
 Not queried
 
  1.1643133
 
       NA
 
           NA
  
 
  
 17
 
  78079608
 
  78079795
 
  ENSEMBL   UCSC 
 
  188
 
  5
 
 16856
 
 gene;island
 
 0.957585
 
 0.9632542
 
 Not queried
 
  1.1643105
 
       NA
 
           NA
  
 
  
 11
 
  89463427
 
  89463689
 
  ENSEMBL   UCSC 
 
  263
 
  4
 
 8722
 
 island
 
 0.552384
 
 0.6810384
 
 Not queried
 
  1.1641396
 
       NA
 
           NA
  
 
  
 4
 
   3443608
 
   3443694
 
  ENSEMBL   UCSC 
 
   87
 
  5
 
 30261
 
 tss
 
 0.557675
 
 0.7052619
 
 Not queried
 
  1.1640142
 
       NA
 
           NA
  
 
  
 3
 
 157261004
 
 157261106
 
  ENSEMBL   UCSC 
 
  103
 
  6
 
 28681
 
 tss
 
 0.068960
 
 0.0219566
 
 Not queried
 
  1.1637654
 
       NA
 
           NA
  
 
  
 19
 
  50936038
 
  50936172
 
  ENSEMBL   UCSC 
 
  135
 
  5
 
 22310
 
 island
 
 0.021022
 
 0.0130772
 
 Not queried
 
  1.1634910
 
       NA
 
           NA
  
 
  
 16
 
  15736658
 
  15736927
 
  ENSEMBL   UCSC 
 
  270
 
  4
 
 14676
 
 gene;tss
 
 0.004807
 
 0.0147337
 
 Not queried
 
  1.1631829
 
       NA
 
           NA
  
 
  
 7
 
 157593864
 
 157594102
 
  ENSEMBL   UCSC 
 
  239
 
  4
 
 37345
 
 gene;island
 
 0.817685
 
 0.9103640
 
 Not queried
 
  1.1629329
 
       NA
 
           NA
  
 
  
 4
 
 122302226
 
 122302421
 
  ENSEMBL   UCSC 
 
  196
 
  7
 
 30570
 
 tss;island
 
 0.645488
 
 0.1370770
 
 Not queried
 
  1.1625878
 
       NA
 
           NA
  
 
  
 2
 
 232651086
 
 232651151
 
  ENSEMBL   UCSC 
 
   66
 
  6
 
 23945
 
 tss
 
 0.002390
 
 0.0011971
 
 Not queried
 
  1.1624827
 
       NA
 
           NA
  
 
  
 3
 
  38388172
 
  38388808
 
  ENSEMBL   UCSC 
 
  637
 
  6
 
 28990
 
 island
 
 0.035526
 
 0.0281353
 
 Not queried
 
  1.1624347
 
       NA
 
           NA
  
 
  
 18
 
  21852118
 
  21852436
 
  ENSEMBL   UCSC 
 
  319
 
  6
 
 19078
 
 gene;island
 
 0.020066
 
 0.0330348
 
 Not queried
 
  1.1622100
 
       NA
 
           NA
  
 
  
 19
 
   7913785
 
   7913967
 
  ENSEMBL   UCSC 
 
  183
 
  4
 
 19787
 
 gene;island
 
 0.872389
 
 0.9236488
 
 Not queried
 
  1.1618876
 
       NA
 
           NA
  
 
  
 10
 
 131665838
 
 131666314
 
  ENSEMBL   UCSC 
 
  477
 
  6
 
 4831
 
 gene;island
 
 0.524639
 
 0.7096955
 
 Not queried
 
  1.1617485
 
       NA
 
           NA
  
 
  
 10
 
  88718164
 
  88718393
 
  ENSEMBL   UCSC 
 
  230
 
  4
 
 5201
 
 tss
 
 0.624685
 
 0.5483636
 
 Not queried
 
  1.1616941
 
       NA
 
           NA
  
 
  
 19
 
  18794107
 
  18794263
 
  ENSEMBL   UCSC 
 
  157
 
  4
 
 20627
 
 tss;island
 
 0.006676
 
 0.0047762
 
 Not queried
 
  1.1611967
 
       NA
 
           NA
  
 
  
 2
 
  91847976
 
  91848380
 
  ENSEMBL   UCSC 
 
  405
 
  9
 
 23505
 
 tss
 
 0.493479
 
 0.2662804
 
 Not queried
 
  1.1611111
 
       NA
 
           NA
  
 
  
 7
 
   1272255
 
   1272710
 
  ENSEMBL   UCSC 
 
  456
 
  9
 
 38091
 
 island
 
 0.106363
 
 0.1623739
 
 Not queried
 
  1.1605094
 
       NA
 
           NA
  
 
  
 7
 
 143059218
 
 143059300
 
  ENSEMBL   UCSC 
 
   83
 
  6
 
 37254
 
 gene;tss;island
 
 0.006380
 
 0.0097460
 
 Not queried
 
  1.1601771
 
       NA
 
           NA
  
 
  
 1
 
 206680459
 
 206681158
 
  ENSEMBL   UCSC 
 
  700
 
  8
 
 4140
 
 island
 
 0.004742
 
 0.0083171
 
 Not queried
 
  1.1601649
 
       NA
 
           NA
  
 
  
 3
 
 159756648
 
 159757071
 
  ENSEMBL   UCSC 
 
  424
 
  6
 
 29626
 
 island
 
 0.053092
 
 0.0408796
 
 Not queried
 
  1.1600526
 
       NA
 
           NA
  
 
  
 6
 
  41528198
 
  41528785
 
  ENSEMBL   UCSC 
 
  588
 
  8
 
 34329
 
 gene;island
 
 0.006521
 
 0.0215309
 
 Not queried
 
  1.1600150
 
       NA
 
           NA
  
 
  
 10
 
  38739819
 
  38740029
 
  ENSEMBL   UCSC 
 
  211
 
  4
 
 4562
 
 gene;island
 
 0.706640
 
 0.8432949
 
 Not queried
 
  1.1598952
 
       NA
 
           NA
  
 
  
 4
 
 148538143
 
 148538527
 
  ENSEMBL   UCSC 
 
  385
 
  8
 
 30627
 
 tss
 
 0.017657
 
 0.0027695
 
 Not queried
 
  1.1597139
 
       NA
 
           NA
  
 
  
 19
 
  49990748
 
  49990808
 
  ENSEMBL   UCSC 
 
   61
 
  5
 
 20948
 
 tss
 
 0.002820
 
 0.0058844
 
 Not queried
 
  1.1596487
 
       NA
 
           NA
  
 
  
 1
 
  45082704
 
  45083278
 
  ENSEMBL   UCSC 
 
  575
 
  7
 
 491
 
 gene;island
 
 0.898719
 
 0.9545406
 
 Not queried
 
  1.1594532
 
       NA
 
           NA
  
 
  
 6
 
 139012860
 
 139013146
 
  ENSEMBL   UCSC 
 
  287
 
  4
 
 36515
 
 island
 
 0.012078
 
 0.0053946
 
 Not queried
 
  1.1592775
 
       NA
 
           NA
  
 
  
 17
 
  76976091
 
  76976357
 
  ENSEMBL   UCSC 
 
  267
 
  5
 
 17709
 
 tss
 
 0.474068
 
 0.3683871
 
 Not queried
 
  1.1591150
 
       NA
 
           NA
  
 
  
 3
 
  85008587
 
  85008991
 
  ENSEMBL   UCSC 
 
  405
 
  5
 
 27865
 
 gene;island
 
 0.045485
 
 0.0886299
 
 Not queried
 
  1.1585419
 
       NA
 
           NA
  
 
  
 1
 
  40367713
 
  40367965
 
  ENSEMBL   UCSC 
 
  253
 
  7
 
 1530
 
 tss
 
 0.002744
 
 0.0058749
 
 Not queried
 
  1.1581116
 
       NA
 
           NA
  
 
  
 2
 
  74880862
 
  74881292
 
  ENSEMBL   UCSC 
 
  431
 
  5
 
 23466
 
 tss
 
 0.013077
 
 0.0040220
 
 Not queried
 
  1.1580882
 
       NA
 
           NA
  
 
  
 1
 
  41832369
 
  41832649
 
  ENSEMBL   UCSC 
 
  281
 
  4
 
 3138
 
 island
 
 0.016632
 
 0.0339879
 
 Not queried
 
  1.1577578
 
       NA
 
           NA
  
 
  
 11
 
  78780700
 
  78780983
 
  ENSEMBL   UCSC 
 
  284
 
  4
 
 6848
 
 gene;island
 
 0.835222
 
 0.9208953
 
 Not queried
 
  1.1576916
 
       NA
 
           NA
  
 
  
 19
 
  50643420
 
  50643731
 
  ENSEMBL   UCSC 
 
  312
 
  5
 
 20972
 
 tss
 
 0.628278
 
 0.7903736
 
 Not queried
 
  1.1575747
 
       NA
 
           NA
  
 
  
 1
 
   6313762
 
   6314113
 
  ENSEMBL   UCSC 
 
  352
 
  4
 
 166
 
 gene;island
 
 0.678588
 
 0.8249900
 
 Not queried
 
  1.1570763
 
       NA
 
           NA
  
 
  
 16
 
    284384
 
    284647
 
  ENSEMBL   UCSC 
 
  264
 
  4
 
 14963
 
 tss;island
 
 0.006860
 
 0.0029568
 
 Not queried
 
  1.1563050
 
       NA
 
           NA
  
 
  
 10
 
   3373441
 
   3373770
 
  ENSEMBL   UCSC 
 
  330
 
  5
 
 5510
 
 island
 
 0.662261
 
 0.8588482
 
 Not queried
 
  1.1561678
 
       NA
 
           NA
  
 
  
 6
 
 106773695
 
 106774172
 
  ENSEMBL   UCSC 
 
  478
 
  9
 
 35149
 
 tss
 
 0.007817
 
 0.0105283
 
 Not queried
 
  1.1559035
 
       NA
 
           NA
  
 
  
 2
 
  68546899
 
  68547349
 
  ENSEMBL   UCSC 
 
  451
 
  7
 
 24363
 
 island
 
 0.098065
 
 0.0461077
 
 Not queried
 
  1.1558522
 
       NA
 
           NA
  
 
  
 2
 
 105461096
 
 105461392
 
  ENSEMBL   UCSC 
 
  297
 
  4
 
 24577
 
 island
 
 0.017405
 
 0.0421731
 
 Not queried
 
  1.1555710
 
       NA
 
           NA
  
 
  
 6
 
  42927940
 
  42928546
 
  ENSEMBL   UCSC 
 
  607
 
 25
 
 36145
 
 island
 
 0.057714
 
 0.2044288
 
 Not queried
 
  1.1552486
 
       NA
 
           NA
  
 
  
 11
 
    926062
 
    926250
 
  ENSEMBL   UCSC 
 
  189
 
  4
 
 6397
 
 gene;island
 
 0.003758
 
 0.0026747
 
 Not queried
 
  1.1549062
 
       NA
 
           NA
  
 
  
 15
 
  74466704
 
  74467158
 
  ENSEMBL   UCSC 
 
  455
 
  6
 
 13418
 
 gene;island
 
 0.552047
 
 0.7323835
 
 Not queried
 
  1.1547262
 
       NA
 
           NA
  
 
  
 22
 
  42353589
 
  42353933
 
  ENSEMBL   UCSC 
 
  345
 
  4
 
 26885
 
 gene;island
 
 0.165232
 
 0.2577765
 
 Not queried
 
  1.1546936
 
       NA
 
           NA
  
 
  
 1
 
   8377724
 
   8377868
 
  ENSEMBL   UCSC 
 
  145
 
  4
 
 2635
 
 island
 
 0.741725
 
 0.9378776
 
 Not queried
 
  1.1546329
 
       NA
 
           NA
  
 
  
 19
 
  54466297
 
  54466724
 
  ENSEMBL   UCSC 
 
  428
 
  5
 
 20212
 
 gene
 
 0.029248
 
 0.1263314
 
 Not queried
 
  1.1546251
 
       NA
 
           NA
  
 
  
 2
 
 128433058
 
 128433507
 
  ENSEMBL   UCSC 
 
  450
 
  6
 
 22863
 
 gene;tss;island
 
 0.009574
 
 0.0151798
 
 Not queried
 
  1.1545257
 
       NA
 
           NA
  
 
  
 12
 
 132824225
 
 132824612
 
  ENSEMBL   UCSC 
 
  388
 
  4
 
 9437
 
 gene;island
 
 0.703156
 
 0.8773272
 
 Not queried
 
  1.1543695
 
       NA
 
           NA
  
 
  
 10
 
 134996789
 
 134997074
 
  ENSEMBL   UCSC 
 
  286
 
  5
 
 4890
 
 gene;island
 
 0.601179
 
 0.7954174
 
 Not queried
 
  1.1541262
 
       NA
 
           NA
  
 
  
 16
 
   2265388
 
   2265528
 
  ENSEMBL   UCSC 
 
  141
 
  5
 
 15023
 
 tss;island
 
 0.000955
 
 0.0028837
 
 Not queried
 
  1.1540245
 
       NA
 
           NA
  
 
  
 2
 
  66659348
 
  66659590
 
  ENSEMBL   UCSC 
 
  243
 
  4
 
 24355
 
 island
 
 0.033110
 
 0.1188309
 
 Not queried
 
  1.1535900
 
       NA
 
           NA
  
 
  
 2
 
  75937889
 
  75938438
 
  ENSEMBL   UCSC 
 
  550
 
 12
 
 24459
 
 island
 
 0.028042
 
 0.0129924
 
 Not queried
 
  1.1533265
 
       NA
 
           NA
  
 
  
 12
 
   6442873
 
   6443045
 
  ENSEMBL   UCSC 
 
  173
 
  4
 
 9026
 
 gene
 
 0.958384
 
 0.9785070
 
 Not queried
 
  1.1531722
 
       NA
 
           NA
  
 
  
 1
 
 205537752
 
 205537877
 
  ENSEMBL   UCSC 
 
  126
 
  4
 
 2206
 
 tss;island
 
 0.254930
 
 0.0791230
 
 Not queried
 
  1.1529279
 
       NA
 
           NA
  
 
  
 6
 
  29429909
 
  29430506
 
  ENSEMBL   UCSC 
 
  598
 
  6
 
 33658
 
 gene
 
 0.012797
 
 0.1165382
 
 Not queried
 
  1.1528689
 
       NA
 
           NA
  
 
  
 2
 
 119605761
 
 119606746
 
  ENSEMBL   UCSC 
 
  986
 
 10
 
 23590
 
 tss;island
 
 0.060266
 
 0.0885233
 
 Not queried
 
  1.1528319
 
       NA
 
           NA
  
 
  
 1
 
 209979487
 
 209979779
 
  ENSEMBL   UCSC 
 
  293
 
  8
 
 2233
 
 tss
 
 0.031436
 
 0.0378760
 
 Not queried
 
  1.1528303
 
       NA
 
           NA
  
 
  
 2
 
  27546052
 
  27546399
 
  ENSEMBL   UCSC 
 
  348
 
  5
 
 23264
 
 tss
 
 0.001840
 
 0.0012725
 
 Not queried
 
  1.1524713
 
       NA
 
           NA
  
 
  
 11
 
 124311131
 
 124311423
 
  ENSEMBL   UCSC 
 
  293
 
  5
 
 7782
 
 tss
 
 0.313559
 
 0.4482490
 
 Not queried
 
  1.1524032
 
       NA
 
           NA
  
 
  
 3
 
 113160071
 
 113160821
 
  ENSEMBL   UCSC 
 
  751
 
 10
 
 29364
 
 island
 
 0.513179
 
 0.3606504
 
 Not queried
 
  1.1522800
 
       NA
 
           NA
  
 
  
 16
 
  71392615
 
  71392838
 
  ENSEMBL   UCSC 
 
  224
 
  4
 
 16075
 
 island
 
 0.042355
 
 0.0033034
 
 Not queried
 
  1.1522019
 
       NA
 
           NA
  
 
  
 5
 
   1110019
 
   1110315
 
  ENSEMBL   UCSC 
 
  297
 
  4
 
 31525
 
 gene;island
 
 0.960727
 
 0.8639428
 
 Not queried
 
  1.1521729
 
       NA
 
           NA
  
 
  
 13
 
  26586254
 
  26586713
 
  ENSEMBL   UCSC 
 
  460
 
  6
 
 11110
 
 gene;island
 
 0.189022
 
 0.1639610
 
 Not queried
 
  1.1521456
 
       NA
 
           NA
  
 
  
 1
 
  48459813
 
  48460182
 
  ENSEMBL   UCSC 
 
  370
 
  5
 
 3245
 
 island
 
 0.192230
 
 0.6081266
 
 Not queried
 
  1.1520572
 
       NA
 
           NA
  
 
  
 19
 
   1510494
 
   1510692
 
  ENSEMBL   UCSC 
 
  199
 
  4
 
 19657
 
 gene;island
 
 0.253052
 
 0.3983901
 
 Not queried
 
  1.1517567
 
       NA
 
           NA
  
 
  
 4
 
 184644017
 
 184644413
 
  ENSEMBL   UCSC 
 
  397
 
  4
 
 31455
 
 island
 
 0.023460
 
 0.0572813
 
 Not queried
 
  1.1515507
 
       NA
 
           NA
  
 
  
 11
 
 115530150
 
 115530590
 
  ENSEMBL   UCSC 
 
  441
 
  6
 
 8829
 
 island
 
 0.024066
 
 0.0480567
 
 Not queried
 
  1.1514585
 
       NA
 
           NA
  
 
  
 10
 
 131812411
 
 131812878
 
  ENSEMBL   UCSC 
 
  468
 
  6
 
 6269
 
 island
 
 0.660341
 
 0.8216370
 
 Not queried
 
  1.1512367
 
       NA
 
           NA
  
 
  
 7
 
  92462706
 
  92462981
 
  ENSEMBL   UCSC 
 
  276
 
  4
 
 37087
 
 gene;island
 
 0.010434
 
 0.0047247
 
 Not queried
 
  1.1509479
 
       NA
 
           NA
  
 
  
 10
 
 134791323
 
 134791660
 
  ENSEMBL   UCSC 
 
  338
 
  5
 
 6323
 
 island
 
 0.646151
 
 0.8780765
 
 Not queried
 
  1.1509469
 
       NA
 
           NA
  
 
  
 6
 
  32130375
 
  32130758
 
  ENSEMBL   UCSC 
 
  384
 
  7
 
 34063
 
 gene
 
 0.849740
 
 0.9300673
 
 Not queried
 
  1.1506692
 
       NA
 
           NA
  
 
  
 10
 
  77163316
 
  77163401
 
  ENSEMBL   UCSC 
 
   86
 
  6
 
 4643
 
 gene;tss;island
 
 0.005733
 
 0.0136663
 
 Not queried
 
  1.1504960
 
       NA
 
           NA
  
 
  
 18
 
    904963
 
    905611
 
  ENSEMBL   UCSC 
 
  649
 
 10
 
 19033
 
 gene
 
 0.035745
 
 0.2540165
 
 Not queried
 
  1.1501860
 
       NA
 
           NA
  
 
  
 12
 
 124773870
 
 124774078
 
  ENSEMBL   UCSC 
 
  209
 
  4
 
 9387
 
 gene
 
 0.679547
 
 0.7999891
 
 Not queried
 
  1.1500862
 
       NA
 
           NA
  
 
  
 7
 
  30029231
 
  30029442
 
  ENSEMBL   UCSC 
 
  212
 
  4
 
 36951
 
 gene;island
 
 0.008631
 
 0.0096856
 
 Not queried
 
  1.1498132
 
       NA
 
           NA
  
 
  
 16
 
  31117067
 
  31117318
 
  ENSEMBL   UCSC 
 
  252
 
  4
 
 15868
 
 island
 
 0.021787
 
 0.0235594
 
 Not queried
 
  1.1498020
 
       NA
 
           NA
  
 
  
 19
 
  40324746
 
  40325128
 
  ENSEMBL   UCSC 
 
  383
 
 11
 
 22006
 
 island
 
 0.021112
 
 0.0070674
 
 Not queried
 
  1.1497388
 
       NA
 
           NA
  
 
  
 13
 
  36728869
 
  36729247
 
  ENSEMBL   UCSC 
 
  379
 
  6
 
 11666
 
 island
 
 0.045347
 
 0.0978974
 
 Not queried
 
  1.1497215
 
       NA
 
           NA
  
 
  
 11
 
  69258203
 
  69258477
 
  ENSEMBL   UCSC 
 
  275
 
  4
 
 8587
 
 island
 
 0.032930
 
 0.0479172
 
 Not queried
 
  1.1497190
 
       NA
 
           NA
  
 
  
 19
 
  19729535
 
  19729732
 
  ENSEMBL   UCSC 
 
  198
 
  7
 
 20646
 
 tss
 
 0.001704
 
 0.0060674
 
 Not queried
 
  1.1490621
 
       NA
 
           NA
  
 
  
 12
 
    670520
 
    671027
 
  ENSEMBL   UCSC 
 
  508
 
  6
 
 8995
 
 gene
 
 0.733592
 
 0.7327627
 
 Not queried
 
  1.1490590
 
       NA
 
           NA
  
 
  
 1
 
   6188946
 
   6189304
 
  ENSEMBL   UCSC 
 
  359
 
  5
 
 161
 
 gene;island
 
 0.899441
 
 0.9188090
 
 Not queried
 
  1.1489972
 
       NA
 
           NA
  
 
  
 17
 
  74524852
 
  74525125
 
  ENSEMBL   UCSC 
 
  274
 
  4
 
 16792
 
 gene
 
 0.012011
 
 0.0140565
 
 Not queried
 
  1.1488342
 
       NA
 
           NA
  
 
  
 20
 
  43029468
 
  43029997
 
  ENSEMBL   UCSC 
 
  530
 
  8
 
 25424
 
 gene
 
 0.703462
 
 0.7605950
 
 Not queried
 
  1.1487700
 
       NA
 
           NA
  
 
  
 1
 
 180991969
 
 180992117
 
  ENSEMBL   UCSC 
 
  149
 
  5
 
 4013
 
 island
 
 0.001454
 
 0.0012105
 
 Not queried
 
  1.1485182
 
       NA
 
           NA
  
 
  
 19
 
  23456575
 
  23456790
 
  ENSEMBL   UCSC 
 
  216
 
  4
 
 21846
 
 island
 
 0.011878
 
 0.0181224
 
 Not queried
 
  1.1483735
 
       NA
 
           NA
  
 
  
 19
 
  40005557
 
  40005727
 
  ENSEMBL   UCSC 
 
  171
 
  7
 
 20775
 
 tss
 
 0.028342
 
 0.0379975
 
 Not queried
 
  1.1482449
 
       NA
 
           NA
  
 
  
 12
 
  54321302
 
  54321653
 
  ENSEMBL   UCSC 
 
  352
 
  4
 
 10475
 
 island
 
 0.359699
 
 0.0354814
 
 Not queried
 
  1.1477813
 
       NA
 
           NA
  
 
  
 19
 
  53496664
 
  53497048
 
  ENSEMBL   UCSC 
 
  385
 
  8
 
 22367
 
 island
 
 0.008974
 
 0.0194297
 
 Not queried
 
  1.1475155
 
       NA
 
           NA
  
 
  
 18
 
  76782568
 
  76782895
 
  ENSEMBL   UCSC 
 
  328
 
  5
 
 19594
 
 island
 
 0.913078
 
 0.9714952
 
 Not queried
 
  1.1473003
 
       NA
 
           NA
  
 
  
 1
 
 200639085
 
 200639502
 
  ENSEMBL   UCSC 
 
  418
 
  6
 
 4067
 
 island
 
 0.006670
 
 0.0009764
 
 Not queried
 
  1.1471201
 
       NA
 
           NA
  
 
  
 11
 
  92930739
 
  92930990
 
  ENSEMBL   UCSC 
 
  252
 
  4
 
 6858
 
 gene;island
 
 0.003995
 
 0.0031956
 
 Not queried
 
  1.1470744
 
       NA
 
           NA
  
 
  
 10
 
  25465171
 
  25465503
 
  ENSEMBL   UCSC 
 
  333
 
  8
 
 4534
 
 gene;island
 
 0.047656
 
 0.0908209
 
 Not queried
 
  1.1470190
 
       NA
 
           NA
  
 
  
 4
 
 119809845
 
 119809975
 
  ENSEMBL   UCSC 
 
  131
 
  4
 
 30564
 
 tss
 
 0.017815
 
 0.0084842
 
 Not queried
 
  1.1468988
 
       NA
 
           NA
  
 
  
 12
 
  72057381
 
  72057690
 
  ENSEMBL   UCSC 
 
  310
 
  7
 
 9216
 
 gene
 
 0.012050
 
 0.0012024
 
 Not queried
 
  1.1467835
 
       NA
 
           NA
  
 
  
 10
 
  15761192
 
  15761657
 
  ENSEMBL   UCSC 
 
  466
 
  4
 
 4514
 
 gene
 
 0.125080
 
 0.1461318
 
 Not queried
 
  1.1466582
 
       NA
 
           NA
  
 
  
 5
 
 128300967
 
 128301488
 
  ENSEMBL   UCSC 
 
  522
 
 10
 
 33072
 
 island
 
 0.032749
 
 0.1119540
 
 Not queried
 
  1.1466218
 
       NA
 
           NA
  
 
  
 1
 
  87797008
 
  87797390
 
  ENSEMBL   UCSC 
 
  383
 
  6
 
 615
 
 gene;island
 
 0.000964
 
 0.0038535
 
 Not queried
 
  1.1465410
 
       NA
 
           NA
  
 
  
 2
 
 211090247
 
 211090487
 
  ENSEMBL   UCSC 
 
  241
 
  6
 
 23855
 
 tss
 
 0.171096
 
 0.0638654
 
 Not queried
 
  1.1464359
 
       NA
 
           NA
  
 
  
 10
 
    421981
 
    422393
 
  ENSEMBL   UCSC 
 
  413
 
  5
 
 4436
 
 gene;island
 
 0.799707
 
 0.8972259
 
 Not queried
 
  1.1463710
 
       NA
 
           NA
  
 
  
 6
 
  32526263
 
  32526414
 
  ENSEMBL   UCSC 
 
  152
 
  5
 
 34118
 
 gene
 
 0.789486
 
 0.9316599
 
 Not queried
 
  1.1460164
 
       NA
 
           NA
  
 
  
 1
 
  79472282
 
  79472452
 
  ENSEMBL   UCSC 
 
  171
 
  5
 
 596
 
 gene
 
 0.403307
 
 0.5986351
 
 Not queried
 
  1.1456369
 
       NA
 
           NA
  
 
  
 1
 
   3147891
 
   3148005
 
  ENSEMBL   UCSC 
 
  115
 
  4
 
 110
 
 gene;island
 
 0.624446
 
 0.7926947
 
 Not queried
 
  1.1456111
 
       NA
 
           NA
  
 
  
 15
 
  75748402
 
  75748568
 
  ENSEMBL   UCSC 
 
  167
 
  4
 
 13813
 
 tss;island
 
 0.006164
 
 0.0152505
 
 Not queried
 
  1.1455192
 
       NA
 
           NA
  
 
  
 17
 
  72350354
 
  72350710
 
  ENSEMBL   UCSC 
 
  357
 
  4
 
 16752
 
 gene;island
 
 0.025738
 
 0.0744339
 
 Not queried
 
  1.1455181
 
       NA
 
           NA
  
 
  
 4
 
   6114498
 
   6114699
 
  ENSEMBL   UCSC 
 
  202
 
  4
 
 29926
 
 gene;island
 
 0.915035
 
 0.8032769
 
 Not queried
 
  1.1454375
 
       NA
 
           NA
  
 
  
 11
 
  64794610
 
  64794867
 
  ENSEMBL   UCSC 
 
  258
 
  7
 
 7410
 
 tss
 
 0.033323
 
 0.0153714
 
 Not queried
 
  1.1453953
 
       NA
 
           NA
  
 
  
 11
 
 109292789
 
 109293216
 
  ENSEMBL   UCSC 
 
  428
 
  7
 
 8787
 
 island
 
 0.044922
 
 0.0365143
 
 Not queried
 
  1.1446831
 
       NA
 
           NA
  
 
  
 10
 
   1401818
 
   1402293
 
  ENSEMBL   UCSC 
 
  476
 
  5
 
 4460
 
 gene;island
 
 0.526383
 
 0.8091003
 
 Not queried
 
  1.1442727
 
       NA
 
           NA
  
 
  
 19
 
  10077007
 
  10077324
 
  ENSEMBL   UCSC 
 
  318
 
  4
 
 19805
 
 gene;island
 
 0.079125
 
 0.1431286
 
 Not queried
 
  1.1441192
 
       NA
 
           NA
  
 
  
 3
 
 197517855
 
 197518017
 
  ENSEMBL   UCSC 
 
  163
 
  8
 
 28826
 
 tss
 
 0.012564
 
 0.0116557
 
 Not queried
 
  1.1440528
 
       NA
 
           NA
  
 
  
 11
 
 134152000
 
 134152202
 
  ENSEMBL   UCSC 
 
  203
 
  4
 
 6995
 
 gene
 
 0.551376
 
 0.6904732
 
 Not queried
 
  1.1438646
 
       NA
 
           NA
  
 
  
 12
 
 113549832
 
 113550113
 
  ENSEMBL   UCSC 
 
  282
 
  4
 
 9319
 
 gene;island
 
 0.603334
 
 0.8303747
 
 Not queried
 
  1.1438527
 
       NA
 
           NA
  
 
  
 12
 
  69632957
 
  69633273
 
  ENSEMBL   UCSC 
 
  317
 
  6
 
 9859
 
 tss
 
 0.016687
 
 0.0100028
 
 Not queried
 
  1.1435920
 
       NA
 
           NA
  
 
  
 5
 
  72921439
 
  72921899
 
  ENSEMBL   UCSC 
 
  461
 
  8
 
 32123
 
 tss;island
 
 0.007066
 
 0.0153609
 
 Not queried
 
  1.1435618
 
       NA
 
           NA
  
 
  
 5
 
  74063125
 
  74063363
 
  ENSEMBL   UCSC 
 
  239
 
  5
 
 31647
 
 gene
 
 0.012286
 
 0.0078393
 
 Not queried
 
  1.1433521
 
       NA
 
           NA
  
 
  
 6
 
  32078398
 
  32078624
 
  ENSEMBL   UCSC 
 
  227
 
  8
 
 34862
 
 tss
 
 0.494910
 
 0.6045061
 
 Not queried
 
  1.1429037
 
       NA
 
           NA
  
 
  
 3
 
 121902442
 
 121902510
 
  ENSEMBL   UCSC 
 
   69
 
  4
 
 28534
 
 tss
 
 0.347167
 
 0.3894808
 
 Not queried
 
  1.1428280
 
       NA
 
           NA
  
 
  
 17
 
  26732836
 
  26733068
 
  ENSEMBL   UCSC 
 
  233
 
  4
 
 16497
 
 gene;island
 
 0.034994
 
 0.0597107
 
 Not queried
 
  1.1427205
 
       NA
 
           NA
  
 
  
 9
 
 111696404
 
 111697324
 
  ENSEMBL   UCSC 
 
  921
 
  7
 
 40779
 
 gene;tss;island
 
 0.011267
 
 0.0066775
 
 Not queried
 
  1.1426463
 
       NA
 
           NA
  
 
  
 17
 
  66098595
 
  66098659
 
  ENSEMBL   UCSC 
 
   65
 
  4
 
 16739
 
 gene;island
 
 0.067827
 
 0.0182548
 
 Not queried
 
  1.1425449
 
       NA
 
           NA
  
 
  
 12
 
   6166874
 
   6167147
 
  ENSEMBL   UCSC 
 
  274
 
  4
 
 9022
 
 gene;island
 
 0.515688
 
 0.6742833
 
 Not queried
 
  1.1423555
 
       NA
 
           NA
  
 
  
 1
 
   3056542
 
   3056951
 
  ENSEMBL   UCSC 
 
  410
 
  7
 
 102
 
 gene;island
 
 0.293418
 
 0.5736655
 
 Not queried
 
  1.1423117
 
       NA
 
           NA
  
 
  
 15
 
  75932673
 
  75933022
 
  ENSEMBL   UCSC 
 
  350
 
  9
 
 13816
 
 tss
 
 0.006172
 
 0.0036845
 
 Not queried
 
  1.1422758
 
       NA
 
           NA
  
 
  
 19
 
  57149436
 
  57149813
 
  ENSEMBL   UCSC 
 
  378
 
  5
 
 22451
 
 island
 
 0.024449
 
 0.1737318
 
 Not queried
 
  1.1421717
 
       NA
 
           NA
  
 
  
 2
 
  27665507
 
  27665711
 
  ENSEMBL   UCSC 
 
  205
 
  4
 
 22630
 
 gene;island
 
 0.937486
 
 0.9828393
 
 Not queried
 
  1.1420888
 
       NA
 
           NA
  
 
  
 15
 
  64443650
 
  64443896
 
  ENSEMBL   UCSC 
 
  247
 
  8
 
 13727
 
 tss
 
 0.012290
 
 0.0117160
 
 Not queried
 
  1.1420786
 
       NA
 
           NA
  
 
  
 12
 
  50898249
 
  50898763
 
  ENSEMBL   UCSC 
 
  515
 
  9
 
 9706
 
 tss
 
 0.010592
 
 0.0049370
 
 Not queried
 
  1.1419030
 
       NA
 
           NA
  
 
  
 19
 
  15217635
 
  15218329
 
  ENSEMBL   UCSC 
 
  695
 
 10
 
 21693
 
 island
 
 0.017812
 
 0.0326554
 
 Not queried
 
  1.1418741
 
       NA
 
           NA
  
 
  
 12
 
 120703729
 
 120704006
 
  ENSEMBL   UCSC 
 
  278
 
  4
 
 10056
 
 tss;island
 
 0.102474
 
 0.0633134
 
 Not queried
 
  1.1412954
 
       NA
 
           NA
  
 
  
 8
 
  22078982
 
  22079330
 
  ENSEMBL   UCSC 
 
  349
 
  6
 
 39229
 
 gene;island
 
 0.583793
 
 0.7727131
 
 Not queried
 
  1.1411191
 
       NA
 
           NA
  
 
  
 19
 
    840737
 
    840950
 
  ENSEMBL   UCSC 
 
  214
 
  4
 
 20281
 
 tss
 
 0.063843
 
 0.2774131
 
 Not queried
 
  1.1406177
 
       NA
 
           NA
  
 
  
 19
 
    840737
 
    841082
 
  ENSEMBL   UCSC 
 
  346
 
  6
 
 21178
 
 island
 
 0.097038
 
 0.2939200
 
 Not queried
 
  1.1406177
 
       NA
 
           NA
  
 
  
 12
 
 123756720
 
 123757070
 
  ENSEMBL   UCSC 
 
  351
 
  7
 
 10097
 
 tss;island
 
 0.013612
 
 0.5080134
 
 Not queried
 
  1.1405790
 
       NA
 
           NA
  
 
  
 10
 
 102469807
 
 102470340
 
  ENSEMBL   UCSC 
 
  534
 
  5
 
 6031
 
 island
 
 0.038465
 
 0.0823210
 
 Not queried
 
  1.1404249
 
       NA
 
           NA
  
 
  
 14
 
  70826455
 
  70826572
 
  ENSEMBL   UCSC 
 
  118
 
  5
 
 12486
 
 tss
 
 0.003088
 
 0.0013640
 
 Not queried
 
  1.1402142
 
       NA
 
           NA
  
 
  
 1
 
  15479095
 
  15479474
 
  ENSEMBL   UCSC 
 
  380
 
  4
 
 243
 
 gene
 
 0.020282
 
 0.0436615
 
 Not queried
 
  1.1397587
 
       NA
 
           NA
  
 
  
 12
 
  48396630
 
  48397225
 
  ENSEMBL   UCSC 
 
  596
 
  5
 
 9107
 
 gene;island
 
 0.011834
 
 0.0329372
 
 Not queried
 
  1.1396500
 
       NA
 
           NA
  
 
  
 10
 
  35484768
 
  35484972
 
  ENSEMBL   UCSC 
 
  205
 
  4
 
 4558
 
 gene
 
 0.005158
 
 0.0019295
 
 Not queried
 
  1.1396008
 
       NA
 
           NA
  
 
  
 8
 
  63160127
 
  63160853
 
  ENSEMBL   UCSC 
 
  727
 
 11
 
 39729
 
 tss;island
 
 0.032194
 
 0.0075978
 
 Not queried
 
  1.1395824
 
       NA
 
           NA
  
 
  
 7
 
 157709849
 
 157710215
 
  ENSEMBL   UCSC 
 
  367
 
  4
 
 37355
 
 gene
 
 0.531318
 
 0.6912488
 
 Not queried
 
  1.1395767
 
       NA
 
           NA
  
 
  
 1
 
 214725246
 
 214725918
 
  ENSEMBL   UCSC 
 
  673
 
 12
 
 2251
 
 tss;island
 
 0.078868
 
 0.0147654
 
 Not queried
 
  1.1393651
 
       NA
 
           NA
  
 
  
 4
 
  84406202
 
  84406624
 
  ENSEMBL   UCSC 
 
  423
 
  7
 
 31155
 
 island
 
 0.008873
 
 0.0089152
 
 Not queried
 
  1.1393028
 
       NA
 
           NA
  
 
  
 2
 
 154729622
 
 154730326
 
  ENSEMBL   UCSC 
 
  705
 
  6
 
 22900
 
 gene;island
 
 0.014773
 
 0.0199255
 
 Not queried
 
  1.1391878
 
       NA
 
           NA
  
 
  
 11
 
  69931206
 
  69931514
 
  ENSEMBL   UCSC 
 
  309
 
  8
 
 6785
 
 gene;tss;island
 
 0.365471
 
 0.4757532
 
 Not queried
 
  1.1390483
 
       NA
 
           NA
  
 
  
 11
 
   6342214
 
   6342510
 
  ENSEMBL   UCSC 
 
  297
 
  4
 
 7107
 
 tss;island
 
 0.010257
 
 0.1112029
 
 Not queried
 
  1.1390377
 
       NA
 
           NA
  
 
  
 19
 
  54668230
 
  54668604
 
  ENSEMBL   UCSC 
 
  375
 
  5
 
 20218
 
 gene;island
 
 0.029469
 
 0.0662797
 
 Not queried
 
  1.1390168
 
       NA
 
           NA
  
 
  
 12
 
  14132940
 
  14133426
 
  ENSEMBL   UCSC 
 
  487
 
  7
 
 10262
 
 island
 
 0.047098
 
 0.0217310
 
 Not queried
 
  1.1389849
 
       NA
 
           NA
  
 
  
 12
 
 129279936
 
 129280449
 
  ENSEMBL   UCSC 
 
  514
 
  6
 
 9409
 
 gene;island
 
 0.564944
 
 0.7485679
 
 Not queried
 
  1.1387830
 
       NA
 
           NA
  
 
  
 12
 
 133656595
 
 133656953
 
  ENSEMBL   UCSC 
 
  359
 
  7
 
 10138
 
 tss;island
 
 0.013697
 
 0.0071992
 
 Not queried
 
  1.1387396
 
       NA
 
           NA
  
 
  
 7
 
  38468944
 
  38469273
 
  ENSEMBL   UCSC 
 
  330
 
  5
 
 36971
 
 gene
 
 0.319718
 
 0.5084900
 
 Not queried
 
  1.1385022
 
       NA
 
           NA
  
 
  
 17
 
  38278395
 
  38278610
 
  ENSEMBL   UCSC 
 
  216
 
  4
 
 17324
 
 tss;island
 
 0.025981
 
 0.0092505
 
 Not queried
 
  1.1383231
 
       NA
 
           NA
  
 
  
 2
 
   1234387
 
   1234756
 
  ENSEMBL   UCSC 
 
  370
 
  4
 
 22527
 
 gene
 
 0.530356
 
 0.7411132
 
 Not queried
 
  1.1382578
 
       NA
 
           NA
  
 
  
 12
 
 111800905
 
 111801411
 
  ENSEMBL   UCSC 
 
  507
 
  6
 
 9307
 
 gene;island
 
 0.860470
 
 0.9566764
 
 Not queried
 
  1.1382178
 
       NA
 
           NA
  
 
  
 10
 
  97889373
 
  97889705
 
  ENSEMBL   UCSC 
 
  333
 
  6
 
 5972
 
 island
 
 0.075721
 
 0.4838893
 
 Not queried
 
  1.1381802
 
       NA
 
           NA
  
 
  
 1
 
 240776162
 
 240776316
 
  ENSEMBL   UCSC 
 
  155
 
  4
 
 2348
 
 tss
 
 0.893013
 
 0.9562236
 
 Not queried
 
  1.1380597
 
       NA
 
           NA
  
 
  
 6
 
  25992047
 
  25992401
 
  ENSEMBL   UCSC 
 
  355
 
  4
 
 35503
 
 island
 
 0.019970
 
 0.0256338
 
 Not queried
 
  1.1375133
 
       NA
 
           NA
  
 
  
 6
 
  29323191
 
  29323330
 
  ENSEMBL   UCSC 
 
  140
 
  4
 
 33652
 
 gene
 
 0.506960
 
 0.6438606
 
 Not queried
 
  1.1374823
 
       NA
 
           NA
  
 
  
 3
 
 172167445
 
 172167644
 
  ENSEMBL   UCSC 
 
  200
 
  4
 
 28721
 
 tss
 
 0.388519
 
 0.2756334
 
 Not queried
 
  1.1373935
 
       NA
 
           NA
  
 
  
 7
 
   8481994
 
   8482325
 
  ENSEMBL   UCSC 
 
  332
 
  5
 
 36892
 
 gene;island
 
 0.281285
 
 0.1187266
 
 Not queried
 
  1.1372951
 
       NA
 
           NA
  
 
  
 14
 
  55657967
 
  55658290
 
  ENSEMBL   UCSC 
 
  324
 
  4
 
 12087
 
 gene
 
 0.012888
 
 0.0099701
 
 Not queried
 
  1.1372252
 
       NA
 
           NA
  
 
  
 17
 
  37729644
 
  37730450
 
  ENSEMBL   UCSC 
 
  807
 
  7
 
 18276
 
 island
 
 0.053832
 
 0.0747961
 
 Not queried
 
  1.1367770
 
       NA
 
           NA
  
 
  
 6
 
 160679391
 
 160679811
 
  ENSEMBL   UCSC 
 
  421
 
  4
 
 34534
 
 gene
 
 0.681191
 
 0.7576042
 
 Not queried
 
  1.1367340
 
       NA
 
           NA
  
 
  
 10
 
  70480731
 
  70481056
 
  ENSEMBL   UCSC 
 
  326
 
  8
 
 5796
 
 island
 
 0.005413
 
 0.0024473
 
 Not queried
 
  1.1364639
 
       NA
 
           NA
  
 
  
 4
 
 124426923
 
 124427390
 
  ENSEMBL   UCSC 
 
  468
 
  5
 
 31294
 
 island
 
 0.044696
 
 0.0209026
 
 Not queried
 
  1.1363982
 
       NA
 
           NA
  
 
  
 12
 
   9227173
 
   9227320
 
  ENSEMBL   UCSC 
 
  148
 
  5
 
 9051
 
 gene
 
 0.220790
 
 0.3884541
 
 Not queried
 
  1.1360905
 
       NA
 
           NA
  
 
  
 11
 
 133402497
 
 133402589
 
  ENSEMBL   UCSC 
 
   93
 
  4
 
 7825
 
 tss
 
 0.077677
 
 0.2163786
 
 Not queried
 
  1.1357271
 
       NA
 
           NA
  
 
  
 6
 
  10416337
 
  10416531
 
  ENSEMBL   UCSC 
 
  195
 
  4
 
 33562
 
 gene;tss;island
 
 0.357274
 
 0.2138238
 
 Not queried
 
  1.1355130
 
       NA
 
           NA
  
 
  
 10
 
 119806460
 
 119806516
 
  ENSEMBL   UCSC 
 
   57
 
  4
 
 4788
 
 gene;tss;island
 
 0.006799
 
 0.0052021
 
 Not queried
 
  1.1353690
 
       NA
 
           NA
  
 
  
 22
 
  17850431
 
  17850690
 
  ENSEMBL   UCSC 
 
  260
 
  4
 
 27273
 
 island
 
 0.032630
 
 0.0111434
 
 Not queried
 
  1.1353088
 
       NA
 
           NA
  
 
  
 5
 
 130506206
 
 130506616
 
  ENSEMBL   UCSC 
 
  411
 
  5
 
 32262
 
 tss
 
 0.007612
 
 0.0034884
 
 Not queried
 
  1.1351941
 
       NA
 
           NA
  
 
  
 1
 
 236686564
 
 236686804
 
  ENSEMBL   UCSC 
 
  241
 
  5
 
 1098
 
 gene;tss;island
 
 0.975242
 
 0.5964428
 
 Not queried
 
  1.1351134
 
       NA
 
           NA
  
 
  
 1
 
 101360652
 
 101360775
 
  ENSEMBL   UCSC 
 
  124
 
  4
 
 1780
 
 tss;island
 
 0.003605
 
 0.0039770
 
 Not queried
 
  1.1348689
 
       NA
 
           NA
  
 
  
 6
 
 150067738
 
 150068017
 
  ENSEMBL   UCSC 
 
  280
 
  4
 
 35287
 
 tss
 
 0.059015
 
 0.0443126
 
 Not queried
 
  1.1348662
 
       NA
 
           NA
  
 
  
 7
 
  55224743
 
  55225183
 
  ENSEMBL   UCSC 
 
  441
 
  7
 
 37018
 
 gene
 
 0.567439
 
 0.8769746
 
 Not queried
 
  1.1344351
 
       NA
 
           NA
  
 
  
 3
 
  46619131
 
  46619364
 
  ENSEMBL   UCSC 
 
  234
 
  4
 
 27751
 
 gene;island
 
 0.647073
 
 0.7263347
 
 Not queried
 
  1.1343953
 
       NA
 
           NA
  
 
  
 6
 
  32064491
 
  32065211
 
  ENSEMBL   UCSC 
 
  721
 
 29
 
 34047
 
 gene;island
 
 0.526350
 
 0.2243901
 
 Not queried
 
  1.1342716
 
       NA
 
           NA
  
 
  
 16
 
  84178939
 
  84179331
 
  ENSEMBL   UCSC 
 
  393
 
  6
 
 14864
 
 gene
 
 0.014180
 
 0.0114108
 
 Not queried
 
  1.1340601
 
       NA
 
           NA
  
 
  
 19
 
  55962948
 
  55963317
 
  ENSEMBL   UCSC 
 
  370
 
  4
 
 22425
 
 island
 
 0.031489
 
 0.0179368
 
 Not queried
 
  1.1335608
 
       NA
 
           NA
  
 
  
 11
 
  34937958
 
  34938507
 
  ENSEMBL   UCSC 
 
  550
 
  9
 
 7244
 
 tss
 
 0.008861
 
 0.0065298
 
 Not queried
 
  1.1331752
 
       NA
 
           NA
  
 
  
 17
 
  61819722
 
  61820015
 
  ENSEMBL   UCSC 
 
  294
 
  4
 
 17570
 
 tss;island
 
 0.005577
 
 0.0217849
 
 Not queried
 
  1.1327245
 
       NA
 
           NA
  
 
  
 11
 
 123986026
 
 123986094
 
  ENSEMBL   UCSC 
 
   69
 
  5
 
 7781
 
 tss
 
 0.008140
 
 0.0037668
 
 Not queried
 
  1.1326687
 
       NA
 
           NA
  
 
  
 5
 
 140044452
 
 140044696
 
  ENSEMBL   UCSC 
 
  245
 
  4
 
 31790
 
 gene
 
 0.011004
 
 0.0166987
 
 Not queried
 
  1.1326601
 
       NA
 
           NA
  
 
  
 10
 
    685962
 
    686354
 
  ENSEMBL   UCSC 
 
  393
 
  4
 
 4448
 
 gene;island
 
 0.621265
 
 0.7909476
 
 Not queried
 
  1.1325092
 
       NA
 
           NA
  
 
  
 1
 
  41445556
 
  41445888
 
  ENSEMBL   UCSC 
 
  333
 
  4
 
 459
 
 gene;island
 
 0.009991
 
 0.0045673
 
 Not queried
 
  1.1324592
 
       NA
 
           NA
  
 
  
 2
 
 128848455
 
 128848689
 
  ENSEMBL   UCSC 
 
  235
 
  5
 
 23622
 
 tss
 
 0.005923
 
 0.0080825
 
 Not queried
 
  1.1324326
 
       NA
 
           NA
  
 
  
 12
 
 124155451
 
 124155620
 
  ENSEMBL   UCSC 
 
  170
 
  5
 
 10105
 
 tss
 
 0.006895
 
 0.0187573
 
 Not queried
 
  1.1315939
 
       NA
 
           NA
  
 
  
 1
 
  15573457
 
  15573655
 
  ENSEMBL   UCSC 
 
  199
 
  7
 
 1269
 
 tss;island
 
 0.105578
 
 0.5635343
 
 Not queried
 
  1.1315599
 
       NA
 
           NA
  
 
  
 4
 
   2470698
 
   2470786
 
  ENSEMBL   UCSC 
 
   89
 
  4
 
 30250
 
 tss
 
 0.010679
 
 0.0108486
 
 Not queried
 
  1.1315038
 
       NA
 
           NA
  
 
  
 4
 
   2470698
 
   2471025
 
  ENSEMBL   UCSC 
 
  328
 
  6
 
 30817
 
 island
 
 0.008180
 
 0.0079997
 
 Not queried
 
  1.1315038
 
       NA
 
           NA
  
 
  
 12
 
  56652163
 
  56652449
 
  ENSEMBL   UCSC 
 
  287
 
  7
 
 9791
 
 tss
 
 0.006501
 
 0.0037337
 
 Not queried
 
  1.1314994
 
       NA
 
           NA
  
 
  
 7
 
 158380884
 
 158381220
 
  ENSEMBL   UCSC 
 
  337
 
  4
 
 38021
 
 tss;island
 
 0.161231
 
 0.3373719
 
 Not queried
 
  1.1314611
 
       NA
 
           NA
  
 
  
 1
 
 245727127
 
 245727658
 
  ENSEMBL   UCSC 
 
  532
 
  6
 
 1117
 
 gene
 
 0.470603
 
 0.6020890
 
 Not queried
 
  1.1313520
 
       NA
 
           NA
  
 
  
 6
 
 151814892
 
 151814994
 
  ENSEMBL   UCSC 
 
  103
 
  5
 
 35301
 
 tss;island
 
 0.258816
 
 0.4420310
 
 Not queried
 
  1.1307842
 
       NA
 
           NA
  
 
  
 7
 
  18067282
 
  18067463
 
  ENSEMBL   UCSC 
 
  182
 
  4
 
 36907
 
 gene
 
 0.912808
 
 0.9439123
 
 Not queried
 
  1.1307649
 
       NA
 
           NA
  
 
  
 3
 
  15140507
 
  15140889
 
  ENSEMBL   UCSC 
 
  383
 
  6
 
 28916
 
 island
 
 0.003570
 
 0.0019405
 
 Not queried
 
  1.1306538
 
       NA
 
           NA
  
 
  
 2
 
  20850523
 
  20850809
 
  ENSEMBL   UCSC 
 
  287
 
  4
 
 22590
 
 gene
 
 0.007316
 
 0.0061019
 
 Not queried
 
  1.1305471
 
       NA
 
           NA
  
 
  
 1
 
 115259226
 
 115259668
 
  ENSEMBL   UCSC 
 
  443
 
  4
 
 701
 
 gene
 
 0.005154
 
 0.0104569
 
 Not queried
 
  1.1301108
 
       NA
 
           NA
  
 
  
 18
 
  30352975
 
  30353283
 
  ENSEMBL   UCSC 
 
  309
 
  7
 
 19226
 
 tss
 
 0.002910
 
 0.0037621
 
 Not queried
 
  1.1300831
 
       NA
 
           NA
  
 
  
 6
 
  31382027
 
  31382250
 
  ENSEMBL   UCSC 
 
  224
 
  6
 
 33875
 
 gene
 
 0.937109
 
 0.8023724
 
 Not queried
 
  1.1300797
 
       NA
 
           NA
  
 
  
 2
 
  37458357
 
  37458730
 
  ENSEMBL   UCSC 
 
  374
 
  8
 
 22647
 
 gene
 
 0.010017
 
 0.0059642
 
 Not queried
 
  1.1299885
 
       NA
 
           NA
  
 
  
 20
 
  32899771
 
  32899839
 
  ENSEMBL   UCSC 
 
   69
 
  5
 
 25690
 
 tss
 
 0.014152
 
 0.0303424
 
 Not queried
 
  1.1298645
 
       NA
 
           NA
  
 
  
 10
 
 103052161
 
 103052605
 
  ENSEMBL   UCSC 
 
  445
 
  5
 
 6066
 
 island
 
 0.086213
 
 0.0269699
 
 Not queried
 
  1.1297952
 
       NA
 
           NA
  
 
  
 5
 
  23507450
 
  23507656
 
  ENSEMBL   UCSC 
 
  207
 
  9
 
 32001
 
 tss
 
 0.779110
 
 0.8220652
 
 Not queried
 
  1.1295954
 
       NA
 
           NA
  
 
  
 10
 
  42970618
 
  42970923
 
  ENSEMBL   UCSC 
 
  306
 
  6
 
 5040
 
 tss
 
 0.365013
 
 0.5916556
 
 Not queried
 
  1.1295383
 
       NA
 
           NA
  
 
  
 2
 
  51254021
 
  51254170
 
  ENSEMBL   UCSC 
 
  150
 
  4
 
 22677
 
 gene;island
 
 0.249588
 
 0.5185316
 
 Not queried
 
  1.1295221
 
       NA
 
           NA
  
 
  
 11
 
   1858467
 
   1858737
 
  ENSEMBL   UCSC 
 
  271
 
  4
 
 6433
 
 gene
 
 0.474032
 
 0.5566557
 
 Not queried
 
  1.1291297
 
       NA
 
           NA
  
 
  
 22
 
  26137738
 
  26137997
 
  ENSEMBL   UCSC 
 
  260
 
  5
 
 27032
 
 tss
 
 0.469348
 
 0.8794094
 
 Not queried
 
  1.1290686
 
       NA
 
           NA
  
 
  
 12
 
  64062221
 
  64062724
 
  ENSEMBL   UCSC 
 
  504
 
  9
 
 10589
 
 island
 
 0.028653
 
 0.0930488
 
 Not queried
 
  1.1288848
 
       NA
 
           NA
  
 
  
 1
 
 161500441
 
 161500751
 
  ENSEMBL   UCSC 
 
  311
 
  4
 
 3910
 
 island
 
 0.016712
 
 0.0112711
 
 Not queried
 
  1.1288689
 
       NA
 
           NA
  
 
  
 11
 
   1037640
 
   1037923
 
  ENSEMBL   UCSC 
 
  284
 
  5
 
 7037
 
 tss;island
 
 0.446575
 
 0.6959410
 
 Not queried
 
  1.1284128
 
       NA
 
           NA
  
 
  
 12
 
  89746101
 
  89746771
 
  ENSEMBL   UCSC 
 
  671
 
 10
 
 10664
 
 island
 
 0.025371
 
 0.0102661
 
 Not queried
 
  1.1283979
 
       NA
 
           NA
  
 
  
 6
 
  29578124
 
  29578496
 
  ENSEMBL   UCSC 
 
  373
 
  6
 
 33669
 
 gene
 
 0.445418
 
 0.7288831
 
 Not queried
 
  1.1282823
 
       NA
 
           NA
  
 
  
 6
 
 168122607
 
 168122963
 
  ENSEMBL   UCSC 
 
  357
 
  6
 
 36647
 
 island
 
 0.527061
 
 0.9236063
 
 Not queried
 
  1.1282762
 
       NA
 
           NA
  
 
  
 8
 
  38831148
 
  38831598
 
  ENSEMBL   UCSC 
 
  451
 
  4
 
 39660
 
 tss
 
 0.599899
 
 0.8644420
 
 Not queried
 
  1.1282434
 
       NA
 
           NA
  
 
  
 14
 
  74318575
 
  74318774
 
  ENSEMBL   UCSC 
 
  200
 
  4
 
 12135
 
 gene
 
 0.012651
 
 0.0078902
 
 Not queried
 
  1.1282091
 
       NA
 
           NA
  
 
  
 11
 
 113184936
 
 113185349
 
  ENSEMBL   UCSC 
 
  414
 
 10
 
 8814
 
 island
 
 0.014096
 
 0.0517508
 
 Not queried
 
  1.1280027
 
       NA
 
           NA
  
 
  
 17
 
  10600976
 
  10601181
 
  ENSEMBL   UCSC 
 
  206
 
  6
 
 16444
 
 gene
 
 0.003649
 
 0.0023594
 
 Not queried
 
  1.1279726
 
       NA
 
           NA
  
 
  
 10
 
 114610508
 
 114610635
 
  ENSEMBL   UCSC 
 
  128
 
  4
 
 5367
 
 tss
 
 0.492879
 
 0.3125264
 
 Not queried
 
  1.1279488
 
       NA
 
           NA
  
 
  
 4
 
  95128661
 
  95129403
 
  ENSEMBL   UCSC 
 
  743
 
 15
 
 31187
 
 island
 
 0.004487
 
 0.0025955
 
 Not queried
 
  1.1277313
 
       NA
 
           NA
  
 
  
 5
 
  89705635
 
  89706015
 
  ENSEMBL   UCSC 
 
  381
 
  8
 
 32171
 
 tss
 
 0.005488
 
 0.0026207
 
 Not queried
 
  1.1276064
 
       NA
 
           NA
  
 
  
 10
 
 100175079
 
 100175236
 
  ENSEMBL   UCSC 
 
  158
 
  4
 
 5277
 
 tss
 
 0.351990
 
 0.3601417
 
 Not queried
 
  1.1271399
 
       NA
 
           NA
  
 
  
 16
 
  14013678
 
  14013937
 
  ENSEMBL   UCSC 
 
  260
 
  6
 
 15099
 
 tss
 
 0.010377
 
 0.0078355
 
 Not queried
 
  1.1264385
 
       NA
 
           NA
  
 
  
 8
 
 104153592
 
 104153829
 
  ENSEMBL   UCSC 
 
  238
 
  8
 
 39389
 
 gene;tss;island
 
 0.015662
 
 0.0357078
 
 Not queried
 
  1.1261600
 
       NA
 
           NA
  
 
  
 21
 
  33957846
 
  33958060
 
  ENSEMBL   UCSC 
 
  215
 
  4
 
 26511
 
 tss
 
 0.597923
 
 0.7679062
 
 Not queried
 
  1.1259061
 
       NA
 
           NA
  
 
  
 7
 
 107302255
 
 107302510
 
  ENSEMBL   UCSC 
 
  256
 
  7
 
 37183
 
 gene;tss;island
 
 0.032325
 
 0.0092689
 
 Not queried
 
  1.1255705
 
       NA
 
           NA
  
 
  
 12
 
  95942761
 
  95943114
 
  ENSEMBL   UCSC 
 
  354
 
  5
 
 9253
 
 gene;tss;island
 
 0.615159
 
 0.0739186
 
 Not queried
 
  1.1254008
 
       NA
 
           NA
  
 
  
 7
 
    960115
 
    960528
 
  ENSEMBL   UCSC 
 
  414
 
  5
 
 36727
 
 gene;island
 
 0.973654
 
 0.6341755
 
 Not queried
 
  1.1253912
 
       NA
 
           NA
  
 
  
 13
 
 114103477
 
 114103796
 
  ENSEMBL   UCSC 
 
  320
 
  6
 
 11550
 
 tss
 
 0.832332
 
 0.8984878
 
 Not queried
 
  1.1253722
 
       NA
 
           NA
  
 
  
 5
 
 140248564
 
 140249080
 
  ENSEMBL   UCSC 
 
  517
 
  5
 
 31801
 
 gene;island
 
 0.167738
 
 0.2080288
 
 Not queried
 
  1.1252773
 
       NA
 
           NA
  
 
  
 12
 
 133019854
 
 133020030
 
  ENSEMBL   UCSC 
 
  177
 
  4
 
 11057
 
 island
 
 0.785219
 
 0.9359964
 
 Not queried
 
  1.1251546
 
       NA
 
           NA
  
 
  
 12
 
  49504691
 
  49505053
 
  ENSEMBL   UCSC 
 
  363
 
  6
 
 9682
 
 tss
 
 0.028180
 
 0.0038339
 
 Not queried
 
  1.1251032
 
       NA
 
           NA
  
 
  
 12
 
 111284527
 
 111284806
 
  ENSEMBL   UCSC 
 
  280
 
  8
 
 10011
 
 tss
 
 0.011298
 
 0.0348091
 
 Not queried
 
  1.1247206
 
       NA
 
           NA
  
 
  
 17
 
  46620300
 
  46620460
 
  ENSEMBL   UCSC 
 
  161
 
  5
 
 16650
 
 gene;island
 
 0.022961
 
 0.0496391
 
 Not queried
 
  1.1247177
 
       NA
 
           NA
  
 
  
 15
 
  22833149
 
  22833335
 
  ENSEMBL   UCSC 
 
  187
 
  7
 
 13511
 
 tss
 
 0.006888
 
 0.0071167
 
 Not queried
 
  1.1244076
 
       NA
 
           NA
  
 
  
 3
 
 182511312
 
 182511586
 
  ENSEMBL   UCSC 
 
  275
 
  4
 
 28046
 
 gene;island
 
 0.008600
 
 0.0065234
 
 Not queried
 
  1.1240445
 
       NA
 
           NA
  
 
  
 5
 
  43515194
 
  43515527
 
  ENSEMBL   UCSC 
 
  334
 
  5
 
 32048
 
 tss
 
 0.005518
 
 0.0025241
 
 Not queried
 
  1.1234465
 
       NA
 
           NA
  
 
  
 6
 
  30302509
 
  30303126
 
  ENSEMBL   UCSC 
 
  618
 
  5
 
 33763
 
 gene
 
 0.798559
 
 0.9691057
 
 Not queried
 
  1.1231775
 
       NA
 
           NA
  
 
  
 2
 
 171673110
 
 171673272
 
  ENSEMBL   UCSC 
 
  163
 
  4
 
 24850
 
 island
 
 0.019709
 
 0.0093899
 
 Not queried
 
  1.1230961
 
       NA
 
           NA
  
 
  
 14
 
  29247148
 
  29247705
 
  ENSEMBL   UCSC 
 
  558
 
  6
 
 12054
 
 gene;island
 
 0.085960
 
 0.0564320
 
 Not queried
 
  1.1229909
 
       NA
 
           NA
  
 
  
 6
 
  17393396
 
  17393647
 
  ENSEMBL   UCSC 
 
  252
 
  7
 
 34655
 
 tss
 
 0.067680
 
 0.0311775
 
 Not queried
 
  1.1229414
 
       NA
 
           NA
  
 
  
 11
 
   2018088
 
   2018247
 
  ENSEMBL   UCSC 
 
  160
 
  7
 
 7053
 
 tss
 
 0.821483
 
 0.8880823
 
 Not queried
 
  1.1227004
 
       NA
 
           NA
  
 
  
 1
 
  15943511
 
  15943714
 
  ENSEMBL   UCSC 
 
  204
 
  4
 
 1274
 
 tss;island
 
 0.005750
 
 0.0044286
 
 Not queried
 
  1.1226979
 
       NA
 
           NA
  
 
  
 16
 
  10275767
 
  10276119
 
  ENSEMBL   UCSC 
 
  353
 
  5
 
 14658
 
 gene;island
 
 0.021717
 
 0.0147969
 
 Not queried
 
  1.1225365
 
       NA
 
           NA
  
 
  
 7
 
  45953027
 
  45953207
 
  ENSEMBL   UCSC 
 
  181
 
  4
 
 37001
 
 gene
 
 0.777208
 
 0.8401773
 
 Not queried
 
  1.1224715
 
       NA
 
           NA
  
 
  
 4
 
  17783205
 
  17783502
 
  ENSEMBL   UCSC 
 
  298
 
  8
 
 30318
 
 tss
 
 0.138257
 
 0.0494837
 
 Not queried
 
  1.1224437
 
       NA
 
           NA
  
 
  
 6
 
  31380198
 
  31380600
 
  ENSEMBL   UCSC 
 
  403
 
  8
 
 33874
 
 gene
 
 0.924827
 
 0.7617431
 
 Not queried
 
  1.1217208
 
       NA
 
           NA
  
 
  
 12
 
 128899735
 
 128899917
 
  ENSEMBL   UCSC 
 
  183
 
  4
 
 9407
 
 gene;island
 
 0.380120
 
 0.5676359
 
 Not queried
 
  1.1216168
 
       NA
 
           NA
  
 
  
 1
 
  38061605
 
  38061737
 
  ENSEMBL   UCSC 
 
  133
 
  5
 
 1503
 
 tss
 
 0.003206
 
 0.0050963
 
 Not queried
 
  1.1215278
 
       NA
 
           NA
  
 
  
 8
 
  48873190
 
  48873533
 
  ENSEMBL   UCSC 
 
  344
 
  4
 
 39690
 
 tss;island
 
 0.009025
 
 0.0063585
 
 Not queried
 
  1.1214376
 
       NA
 
           NA
  
 
  
 18
 
  77336130
 
  77336267
 
  ENSEMBL   UCSC 
 
  138
 
  4
 
 19604
 
 island
 
 0.833620
 
 0.9550653
 
 Not queried
 
  1.1214128
 
       NA
 
           NA
  
 
  
 8
 
  55370192
 
  55370579
 
  ENSEMBL   UCSC 
 
  388
 
 10
 
 40302
 
 island
 
 0.172905
 
 0.0466648
 
 Not queried
 
  1.1212584
 
       NA
 
           NA
  
 
  
 1
 
  14219312
 
  14219825
 
  ENSEMBL   UCSC 
 
  514
 
  4
 
 2711
 
 island
 
 0.110670
 
 0.0544035
 
 Not queried
 
  1.1211092
 
       NA
 
           NA
  
 
  
 16
 
  28943019
 
  28943232
 
  ENSEMBL   UCSC 
 
  214
 
  4
 
 15161
 
 tss
 
 0.013080
 
 0.0044616
 
 Not queried
 
  1.1209344
 
       NA
 
           NA
  
 
  
 13
 
 113676726
 
 113677325
 
  ENSEMBL   UCSC 
 
  600
 
  6
 
 11269
 
 gene;island
 
 0.430081
 
 0.6721365
 
 Not queried
 
  1.1208788
 
       NA
 
           NA
  
 
  
 5
 
 158893318
 
 158893488
 
  ENSEMBL   UCSC 
 
  171
 
  4
 
 32463
 
 tss
 
 0.843326
 
 0.9560133
 
 Not queried
 
  1.1208656
 
       NA
 
           NA
  
 
  
 1
 
 150669681
 
 150669977
 
  ENSEMBL   UCSC 
 
  297
 
  4
 
 1912
 
 tss
 
 0.004305
 
 0.0053452
 
 Not queried
 
  1.1207638
 
       NA
 
           NA
  
 
  
 15
 
  72104228
 
  72104417
 
  ENSEMBL   UCSC 
 
  190
 
  4
 
 13404
 
 gene;island
 
 0.655225
 
 0.8341195
 
 Not queried
 
  1.1206178
 
       NA
 
           NA
  
 
  
 12
 
 105478345
 
 105478590
 
  ENSEMBL   UCSC 
 
  246
 
  7
 
 9962
 
 tss
 
 0.015336
 
 0.0139658
 
 Not queried
 
  1.1205207
 
       NA
 
           NA
  
 
  
 2
 
  74607507
 
  74607697
 
  ENSEMBL   UCSC 
 
  191
 
  4
 
 23451
 
 tss
 
 0.343320
 
 0.4942852
 
 Not queried
 
  1.1205040
 
       NA
 
           NA
  
 
  
 1
 
   3435887
 
   3436219
 
  ENSEMBL   UCSC 
 
  333
 
  5
 
 128
 
 gene;island
 
 0.439330
 
 0.6300902
 
 Not queried
 
  1.1204216
 
       NA
 
           NA
  
 
  
 6
 
  32013699
 
  32015389
 
  ENSEMBL   UCSC 
 
 1691
 
 28
 
 34013
 
 gene
 
 0.399582
 
 0.5533941
 
 Not queried
 
  1.1199103
 
       NA
 
           NA
  
 
  
 6
 
  10521142
 
  10521558
 
  ENSEMBL   UCSC 
 
  417
 
  4
 
 34627
 
 tss
 
 0.007374
 
 0.1942313
 
 Not queried
 
  1.1198858
 
       NA
 
           NA
  
 
  
 5
 
   3962310
 
   3962477
 
  ENSEMBL   UCSC 
 
  168
 
  4
 
 32671
 
 island
 
 0.769238
 
 0.9165823
 
 Not queried
 
  1.1197626
 
       NA
 
           NA
  
 
  
 6
 
 168402227
 
 168402503
 
  ENSEMBL   UCSC 
 
  277
 
  4
 
 36654
 
 island
 
 0.717933
 
 0.8601243
 
 Not queried
 
  1.1196398
 
       NA
 
           NA
  
 
  
 13
 
  97646639
 
  97646811
 
  ENSEMBL   UCSC 
 
  173
 
  7
 
 11506
 
 tss;island
 
 0.016657
 
 0.0647471
 
 Not queried
 
  1.1195288
 
       NA
 
           NA
  
 
  
 3
 
  49314595
 
  49314920
 
  ENSEMBL   UCSC 
 
  326
 
  8
 
 27779
 
 gene
 
 0.020157
 
 0.0356778
 
 Not queried
 
  1.1191969
 
       NA
 
           NA
  
 
  
 19
 
   3285973
 
   3286244
 
  ENSEMBL   UCSC 
 
  272
 
  4
 
 19713
 
 gene;island
 
 0.202168
 
 0.1036599
 
 Not queried
 
  1.1189607
 
       NA
 
           NA
  
 
  
 3
 
 101232109
 
 101232368
 
  ENSEMBL   UCSC 
 
  260
 
  7
 
 28472
 
 tss
 
 0.001113
 
 0.0007284
 
 Not queried
 
  1.1187182
 
       NA
 
           NA
  
 
  
 10
 
 116581112
 
 116581358
 
  ENSEMBL   UCSC 
 
  247
 
  6
 
 5378
 
 tss;island
 
 0.004583
 
 0.0032522
 
 Not queried
 
  1.1186660
 
       NA
 
           NA
  
 
  
 11
 
  67250046
 
  67250809
 
  ENSEMBL   UCSC 
 
  764
 
 12
 
 8553
 
 island
 
 0.007648
 
 0.0102439
 
 Not queried
 
  1.1186375
 
       NA
 
           NA
  
 
  
 19
 
   1826707
 
   1826957
 
  ENSEMBL   UCSC 
 
  251
 
  4
 
 19665
 
 gene;island
 
 0.774961
 
 0.8738404
 
 Not queried
 
  1.1185126
 
       NA
 
           NA
  
 
  
 10
 
 135270766
 
 135271052
 
  ENSEMBL   UCSC 
 
  287
 
  5
 
 4905
 
 gene;island
 
 0.673753
 
 0.8059662
 
 Not queried
 
  1.1184420
 
       NA
 
           NA
  
 
  
 18
 
  48086162
 
  48086416
 
  ENSEMBL   UCSC 
 
  255
 
  5
 
 19276
 
 tss
 
 0.019200
 
 0.0323475
 
 Not queried
 
  1.1183187
 
       NA
 
           NA
  
 
  
 12
 
 105379893
 
 105380171
 
  ENSEMBL   UCSC 
 
  279
 
  5
 
 10741
 
 island
 
 0.004803
 
 0.0026418
 
 Not queried
 
  1.1179374
 
       NA
 
           NA
  
 
  
 2
 
  10443632
 
  10443818
 
  ENSEMBL   UCSC 
 
  187
 
  6
 
 22565
 
 gene;tss;island
 
 0.005725
 
 0.0056247
 
 Not queried
 
  1.1178000
 
       NA
 
           NA
  
 
  
 5
 
 140857474
 
 140858107
 
  ENSEMBL   UCSC 
 
  634
 
  8
 
 31838
 
 gene;island
 
 0.590195
 
 0.7404903
 
 Not queried
 
  1.1177773
 
       NA
 
           NA
  
 
  
 8
 
  10273666
 
  10273890
 
  ENSEMBL   UCSC 
 
  225
 
  4
 
 39196
 
 gene
 
 0.535029
 
 0.8188713
 
 Not queried
 
  1.1173459
 
       NA
 
           NA
  
 
  
 19
 
   1307393
 
   1307668
 
  ENSEMBL   UCSC 
 
  276
 
  4
 
 21213
 
 island
 
 0.943751
 
 0.9669276
 
 Not queried
 
  1.1172937
 
       NA
 
           NA
  
 
  
 10
 
  69609229
 
  69609574
 
  ENSEMBL   UCSC 
 
  346
 
  4
 
 5784
 
 island
 
 0.004361
 
 0.0020661
 
 Not queried
 
  1.1172641
 
       NA
 
           NA
  
 
  
 2
 
 172778961
 
 172779282
 
  ENSEMBL   UCSC 
 
  322
 
  4
 
 22920
 
 gene
 
 0.003556
 
 0.0032217
 
 Not queried
 
  1.1170878
 
       NA
 
           NA
  
 
  
 19
 
  51219975
 
  51220537
 
  ENSEMBL   UCSC 
 
  563
 
 10
 
 22320
 
 island
 
 0.342200
 
 0.5206889
 
 Not queried
 
  1.1166984
 
       NA
 
           NA
  
 
  
 1
 
 151103869
 
 151104367
 
  ENSEMBL   UCSC 
 
  499
 
  4
 
 3739
 
 island
 
 0.071169
 
 0.4899134
 
 Not queried
 
  1.1163248
 
       NA
 
           NA
  
 
  
 13
 
  29352633
 
  29353104
 
  ENSEMBL   UCSC 
 
  472
 
  4
 
 11643
 
 island
 
 0.487086
 
 0.7107318
 
 Not queried
 
  1.1161913
 
       NA
 
           NA
  
 
  
 11
 
    910094
 
    910475
 
  ENSEMBL   UCSC 
 
  382
 
  4
 
 6395
 
 gene;island
 
 0.005041
 
 0.0030716
 
 Not queried
 
  1.1161376
 
       NA
 
           NA
  
 
  
 17
 
  72352688
 
  72352968
 
  ENSEMBL   UCSC 
 
  281
 
  4
 
 16753
 
 gene;island
 
 0.328223
 
 0.6047932
 
 Not queried
 
  1.1156384
 
       NA
 
           NA
  
 
  
 18
 
  56435611
 
  56435982
 
  ENSEMBL   UCSC 
 
  372
 
  4
 
 19540
 
 island
 
 0.008208
 
 0.0072342
 
 Not queried
 
  1.1155398
 
       NA
 
           NA
  
 
  
 4
 
  83720039
 
  83720240
 
  ENSEMBL   UCSC 
 
  202
 
  6
 
 30468
 
 tss
 
 0.023638
 
 0.0545514
 
 Not queried
 
  1.1154768
 
       NA
 
           NA
  
 
  
 6
 
  16238434
 
  16238691
 
  ENSEMBL   UCSC 
 
  258
 
  6
 
 34651
 
 tss;island
 
 0.040668
 
 0.0224470
 
 Not queried
 
  1.1153879
 
       NA
 
           NA
  
 
  
 12
 
  54090254
 
  54090519
 
  ENSEMBL   UCSC 
 
  266
 
  4
 
 10472
 
 island
 
 0.144314
 
 0.0363193
 
 Not queried
 
  1.1151969
 
       NA
 
           NA
  
 
  
 6
 
  32044104
 
  32044496
 
  ENSEMBL   UCSC 
 
  393
 
  8
 
 34033
 
 gene;island
 
 0.184524
 
 0.2737191
 
 Not queried
 
  1.1146895
 
       NA
 
           NA
  
 
  
 10
 
 118890977
 
 118891355
 
  ENSEMBL   UCSC 
 
  379
 
  4
 
 4777
 
 gene;island
 
 0.021741
 
 0.0552886
 
 Not queried
 
  1.1146398
 
       NA
 
           NA
  
 
  
 16
 
   3184694
 
   3185100
 
  ENSEMBL   UCSC 
 
  407
 
  7
 
 15052
 
 tss
 
 0.007840
 
 0.0020694
 
 Not queried
 
  1.1145435
 
       NA
 
           NA
  
 
  
 13
 
  41768782
 
  41768978
 
  ENSEMBL   UCSC 
 
  197
 
  5
 
 11410
 
 tss
 
 0.004572
 
 0.0021257
 
 Not queried
 
  1.1145313
 
       NA
 
           NA
  
 
  
 11
 
 110300429
 
 110300584
 
  ENSEMBL   UCSC 
 
  156
 
  4
 
 7678
 
 tss
 
 0.018367
 
 0.0189596
 
 Not queried
 
  1.1144318
 
       NA
 
           NA
  
 
  
 15
 
  63569245
 
  63569719
 
  ENSEMBL   UCSC 
 
  475
 
 10
 
 13721
 
 tss
 
 0.003256
 
 0.0100898
 
 Not queried
 
  1.1141928
 
       NA
 
           NA
  
 
  
 7
 
  73275265
 
  73275436
 
  ENSEMBL   UCSC 
 
  172
 
  6
 
 37644
 
 tss
 
 0.319047
 
 0.4691692
 
 Not queried
 
  1.1138344
 
       NA
 
           NA
  
 
  
 4
 
 189580331
 
 189580798
 
  ENSEMBL   UCSC 
 
  468
 
  5
 
 31492
 
 island
 
 0.884992
 
 0.9160054
 
 Not queried
 
  1.1137125
 
       NA
 
           NA
  
 
  
 3
 
 194406190
 
 194406572
 
  ENSEMBL   UCSC 
 
  383
 
  9
 
 28802
 
 tss;island
 
 0.003615
 
 0.0021057
 
 Not queried
 
  1.1135202
 
       NA
 
           NA
  
 
  
 8
 
   1893793
 
   1893977
 
  ENSEMBL   UCSC 
 
  185
 
  4
 
 39183
 
 gene;island
 
 0.475236
 
 0.5971746
 
 Not queried
 
  1.1134205
 
       NA
 
           NA
  
 
  
 12
 
 132628967
 
 132629051
 
  ENSEMBL   UCSC 
 
   85
 
  6
 
 10128
 
 tss;island
 
 0.003448
 
 0.0057967
 
 Not queried
 
  1.1132939
 
       NA
 
           NA
  
 
  
 3
 
 164913828
 
 164914437
 
  ENSEMBL   UCSC 
 
  610
 
  5
 
 28019
 
 gene
 
 0.055129
 
 0.1074027
 
 Not queried
 
  1.1132912
 
       NA
 
           NA
  
 
  
 12
 
 130821453
 
 130821607
 
  ENSEMBL   UCSC 
 
  155
 
  4
 
 10119
 
 tss;island
 
 0.688270
 
 0.8605512
 
 Not queried
 
  1.1128436
 
       NA
 
           NA
  
 
  
 8
 
  57360586
 
  57360738
 
  ENSEMBL   UCSC 
 
  153
 
  5
 
 39716
 
 tss;island
 
 0.108293
 
 0.1030747
 
 Not queried
 
  1.1127677
 
       NA
 
           NA
  
 
  
 22
 
  30234293
 
  30234508
 
  ENSEMBL   UCSC 
 
  216
 
  4
 
 27054
 
 tss
 
 0.008185
 
 0.0034449
 
 Not queried
 
  1.1127614
 
       NA
 
           NA
  
 
  
 22
 
  30234213
 
  30234508
 
  ENSEMBL   UCSC 
 
  296
 
  6
 
 27403
 
 island
 
 0.005905
 
 0.0040328
 
 Not queried
 
  1.1127614
 
       NA
 
           NA
  
 
  
 6
 
 132272545
 
 132272790
 
  ENSEMBL   UCSC 
 
  246
 
  4
 
 35225
 
 tss
 
 0.048607
 
 0.0285330
 
 Not queried
 
  1.1126718
 
       NA
 
           NA
  
 
  
 6
 
 132272342
 
 132272790
 
  ENSEMBL   UCSC 
 
  449
 
  6
 
 36481
 
 island
 
 0.022866
 
 0.0315452
 
 Not queried
 
  1.1126718
 
       NA
 
           NA
  
 
  
 8
 
  77589908
 
  77590513
 
  ENSEMBL   UCSC 
 
  606
 
  5
 
 39341
 
 gene;island
 
 0.021331
 
 0.0438785
 
 Not queried
 
  1.1125144
 
       NA
 
           NA
  
 
  
 13
 
 112212075
 
 112212266
 
  ENSEMBL   UCSC 
 
  192
 
  4
 
 11899
 
 island
 
 0.135800
 
 0.2049139
 
 Not queried
 
  1.1119823
 
       NA
 
           NA
  
 
  
 20
 
  32891238
 
  32891428
 
  ENSEMBL   UCSC 
 
  191
 
  7
 
 25689
 
 tss
 
 0.023800
 
 0.0105164
 
 Not queried
 
  1.1113515
 
       NA
 
           NA
  
 
  
 20
 
  17296030
 
  17296597
 
  ENSEMBL   UCSC 
 
  568
 
  5
 
 25364
 
 gene;island
 
 0.075207
 
 0.1252352
 
 Not queried
 
  1.1112305
 
       NA
 
           NA
  
 
  
 11
 
  17717574
 
  17717818
 
  ENSEMBL   UCSC 
 
  245
 
  4
 
 8102
 
 island
 
 0.046292
 
 0.1154431
 
 Not queried
 
  1.1105698
 
       NA
 
           NA
  
 
  
 6
 
  82462430
 
  82462746
 
  ENSEMBL   UCSC 
 
  317
 
  8
 
 35086
 
 tss
 
 0.024373
 
 0.0096740
 
 Not queried
 
  1.1104963
 
       NA
 
           NA
  
 
  
 19
 
  58399865
 
  58400574
 
  ENSEMBL   UCSC 
 
  710
 
  9
 
 22485
 
 island
 
 0.065433
 
 0.0300527
 
 Not queried
 
  1.1104868
 
       NA
 
           NA
  
 
  
 3
 
  64009142
 
  64009226
 
  ENSEMBL   UCSC 
 
   85
 
  5
 
 28432
 
 tss
 
 0.003093
 
 0.0030331
 
 Not queried
 
  1.1104233
 
       NA
 
           NA
  
 
  
 5
 
  38846475
 
  38846743
 
  ENSEMBL   UCSC 
 
  269
 
  4
 
 31597
 
 gene;island
 
 0.086724
 
 0.1910503
 
 Not queried
 
  1.1100883
 
       NA
 
           NA
  
 
  
 22
 
  37215611
 
  37215656
 
  ENSEMBL   UCSC 
 
   46
 
  4
 
 27112
 
 tss;island
 
 0.271239
 
 0.4494402
 
 Not queried
 
  1.1100667
 
       NA
 
           NA
  
 
  
 12
 
  12419921
 
  12420135
 
  ENSEMBL   UCSC 
 
  215
 
  4
 
 9568
 
 tss
 
 0.010352
 
 0.0039044
 
 Not queried
 
  1.1100151
 
       NA
 
           NA
  
 
  
 5
 
 140501321
 
 140501540
 
  ENSEMBL   UCSC 
 
  220
 
  7
 
 32362
 
 tss
 
 0.311734
 
 0.5082381
 
 Not queried
 
  1.1099910
 
       NA
 
           NA
  
 
  
 7
 
 117512803
 
 117513101
 
  ENSEMBL   UCSC 
 
  299
 
  4
 
 37201
 
 gene;island
 
 0.053459
 
 0.0229947
 
 Not queried
 
  1.1097701
 
       NA
 
           NA
  
 
  
 12
 
 111664582
 
 111664785
 
  ENSEMBL   UCSC 
 
  204
 
  6
 
 9306
 
 gene
 
 0.669426
 
 0.7486700
 
 Not queried
 
  1.1096473
 
       NA
 
           NA
  
 
  
 16
 
   4802469
 
   4802990
 
  ENSEMBL   UCSC 
 
  522
 
  5
 
 14645
 
 gene;island
 
 0.614797
 
 0.7750049
 
 Not queried
 
  1.1096418
 
       NA
 
           NA
  
 
  
 16
 
  70680320
 
  70680406
 
  ENSEMBL   UCSC 
 
   87
 
  4
 
 15342
 
 tss
 
 0.590289
 
 0.7526405
 
 Not queried
 
  1.1095188
 
       NA
 
           NA
  
 
  
 1
 
   2850512
 
   2850951
 
  ENSEMBL   UCSC 
 
  440
 
  5
 
 2522
 
 island
 
 0.557126
 
 0.7581658
 
 Not queried
 
  1.1092845
 
       NA
 
           NA
  
 
  
 8
 
  49647579
 
  49648363
 
  ENSEMBL   UCSC 
 
  785
 
 11
 
 40284
 
 island
 
 0.129347
 
 0.0642053
 
 Not queried
 
  1.1091826
 
       NA
 
           NA
  
 
  
 8
 
 145654007
 
 145654090
 
  ENSEMBL   UCSC 
 
   84
 
  5
 
 39983
 
 tss
 
 0.004088
 
 0.0065626
 
 Not queried
 
  1.1089199
 
       NA
 
           NA
  
 
  
 16
 
  46918956
 
  46919194
 
  ENSEMBL   UCSC 
 
  239
 
  5
 
 14742
 
 gene;island
 
 0.384055
 
 0.2603782
 
 Not queried
 
  1.1088971
 
       NA
 
           NA
  
 
  
 19
 
   1026491
 
   1026757
 
  ENSEMBL   UCSC 
 
  267
 
  4
 
 19639
 
 gene;island
 
 0.022818
 
 0.0287962
 
 Not queried
 
  1.1088534
 
       NA
 
           NA
  
 
  
 12
 
 133532874
 
 133533354
 
  ENSEMBL   UCSC 
 
  481
 
  7
 
 10135
 
 tss;island
 
 0.032455
 
 0.0183233
 
 Not queried
 
  1.1088267
 
       NA
 
           NA
  
 
  
 11
 
  33279084
 
  33279136
 
  ENSEMBL   UCSC 
 
   53
 
  4
 
 7233
 
 tss
 
 0.002462
 
 0.0027024
 
 Not queried
 
  1.1087432
 
       NA
 
           NA
  
 
  
 5
 
 114631921
 
 114632301
 
  ENSEMBL   UCSC 
 
  381
 
  7
 
 31720
 
 gene;island
 
 0.030564
 
 0.0142805
 
 Not queried
 
  1.1087297
 
       NA
 
           NA
  
 
  
 17
 
  76921500
 
  76921558
 
  ENSEMBL   UCSC 
 
   59
 
  5
 
 17707
 
 tss;island
 
 0.026242
 
 0.0130009
 
 Not queried
 
  1.1084113
 
       NA
 
           NA
  
 
  
 7
 
  72299837
 
  72300134
 
  ENSEMBL   UCSC 
 
  298
 
  6
 
 37629
 
 tss;island
 
 0.002340
 
 0.0030811
 
 Not queried
 
  1.1083435
 
       NA
 
           NA
  
 
  
 13
 
  41635251
 
  41635664
 
  ENSEMBL   UCSC 
 
  414
 
  6
 
 11408
 
 tss
 
 0.008547
 
 0.0114507
 
 Not queried
 
  1.1083380
 
       NA
 
           NA
  
 
  
 20
 
   2489835
 
   2490109
 
  ENSEMBL   UCSC 
 
  275
 
  8
 
 25545
 
 tss;island
 
 0.004049
 
 0.0062168
 
 Not queried
 
  1.1078322
 
       NA
 
           NA
  
 
  
 20
 
  23402196
 
  23402404
 
  ENSEMBL   UCSC 
 
  209
 
  8
 
 25639
 
 tss
 
 0.003265
 
 0.0029001
 
 Not queried
 
  1.1078055
 
       NA
 
           NA
  
 
  
 20
 
  23402051
 
  23402404
 
  ENSEMBL   UCSC 
 
  354
 
 10
 
 26051
 
 island
 
 0.003265
 
 0.0029001
 
 Not queried
 
  1.1078055
 
       NA
 
           NA
  
 
  
 1
 
  47697429
 
  47697663
 
  ENSEMBL   UCSC 
 
  235
 
  4
 
 3232
 
 island
 
 0.009995
 
 0.0063382
 
 Not queried
 
  1.1077112
 
       NA
 
           NA
  
 
  
 7
 
  35494345
 
  35494808
 
  ENSEMBL   UCSC 
 
  464
 
  4
 
 38409
 
 island
 
 0.377384
 
 0.2226203
 
 Not queried
 
  1.1074735
 
       NA
 
           NA
  
 
  
 17
 
  76836740
 
  76836835
 
  ENSEMBL   UCSC 
 
   96
 
  4
 
 16828
 
 gene
 
 0.010688
 
 0.0125661
 
 Not queried
 
  1.1074002
 
       NA
 
           NA
  
 
  
 11
 
 133804526
 
 133805057
 
  ENSEMBL   UCSC 
 
  532
 
  6
 
 6987
 
 gene
 
 0.586016
 
 0.7681111
 
 Not queried
 
  1.1072469
 
       NA
 
           NA
  
 
  
 11
 
 133804526
 
 133804886
 
  ENSEMBL   UCSC 
 
  361
 
  4
 
 8971
 
 island
 
 0.635953
 
 0.7681111
 
 Not queried
 
  1.1072469
 
       NA
 
           NA
  
 
  
 5
 
 150399781
 
 150400672
 
  ENSEMBL   UCSC 
 
  892
 
  9
 
 33315
 
 island
 
 0.089399
 
 0.4864948
 
 Not queried
 
  1.1072206
 
       NA
 
           NA
  
 
  
 11
 
 117069780
 
 117069966
 
  ENSEMBL   UCSC 
 
  187
 
  7
 
 7717
 
 tss
 
 0.949261
 
 0.8873127
 
 Not queried
 
  1.1070005
 
       NA
 
           NA
  
 
  
 12
 
  91573135
 
  91573372
 
  ENSEMBL   UCSC 
 
  238
 
  4
 
 9242
 
 gene;tss
 
 0.244034
 
 0.5441072
 
 Not queried
 
  1.1068453
 
       NA
 
           NA
  
 
  
 1
 
 110036808
 
 110037126
 
  ENSEMBL   UCSC 
 
  319
 
  5
 
 668
 
 gene;island
 
 0.008739
 
 0.0063734
 
 Not queried
 
  1.1067967
 
       NA
 
           NA
  
 
  
 12
 
 107168153
 
 107168633
 
  ENSEMBL   UCSC 
 
  481
 
  5
 
 10755
 
 island
 
 0.003277
 
 0.0074327
 
 Not queried
 
  1.1064461
 
       NA
 
           NA
  
 
  
 17
 
   6926003
 
   6926414
 
  ENSEMBL   UCSC 
 
  412
 
  5
 
 17916
 
 island
 
 0.053166
 
 0.1162292
 
 Not queried
 
  1.1063383
 
       NA
 
           NA
  
 
  
 17
 
  77903985
 
  77904396
 
  ENSEMBL   UCSC 
 
  412
 
  5
 
 18847
 
 island
 
 0.111264
 
 0.0357263
 
 Not queried
 
  1.1061797
 
       NA
 
           NA
  
 
  
 6
 
  31833747
 
  31834178
 
  ENSEMBL   UCSC 
 
  432
 
 10
 
 33977
 
 gene;island
 
 0.545567
 
 0.8088666
 
 Not queried
 
  1.1060295
 
       NA
 
           NA
  
 
  
 6
 
  31634602
 
  31634890
 
  ENSEMBL   UCSC 
 
  289
 
  6
 
 33934
 
 gene;island
 
 0.622242
 
 0.7975138
 
 Not queried
 
  1.1059092
 
       NA
 
           NA
  
 
  
 2
 
 223536069
 
 223536327
 
  ENSEMBL   UCSC 
 
  259
 
  6
 
 23920
 
 tss
 
 0.045344
 
 0.0937102
 
 Not queried
 
  1.1058921
 
       NA
 
           NA
  
 
  
 2
 
 223536069
 
 223536609
 
  ENSEMBL   UCSC 
 
  541
 
  8
 
 25155
 
 island
 
 0.045344
 
 0.0937102
 
 Not queried
 
  1.1058921
 
       NA
 
           NA
  
 
  
 17
 
  15902501
 
  15902694
 
  ENSEMBL   UCSC 
 
  194
 
  6
 
 17153
 
 tss
 
 0.006116
 
 0.0045253
 
 Not queried
 
  1.1058718
 
       NA
 
           NA
  
 
  
 6
 
  30000566
 
  30000797
 
  ENSEMBL   UCSC 
 
  232
 
  4
 
 33711
 
 gene
 
 0.961569
 
 0.9822023
 
 Not queried
 
  1.1054847
 
       NA
 
           NA
  
 
  
 18
 
  44526430
 
  44527026
 
  ENSEMBL   UCSC 
 
  597
 
  8
 
 19493
 
 island
 
 0.005827
 
 0.0061578
 
 Not queried
 
  1.1052687
 
       NA
 
           NA
  
 
  
 1
 
 107684059
 
 107684425
 
  ENSEMBL   UCSC 
 
  367
 
  4
 
 659
 
 gene;island
 
 0.004878
 
 0.0087822
 
 Not queried
 
  1.1048395
 
       NA
 
           NA
  
 
  
 19
 
   4007529
 
   4007604
 
  ENSEMBL   UCSC 
 
   76
 
  4
 
 20358
 
 tss;island
 
 0.001500
 
 0.0025276
 
 Not queried
 
  1.1045186
 
       NA
 
           NA
  
 
  
 17
 
   8213195
 
   8213522
 
  ENSEMBL   UCSC 
 
  328
 
  4
 
 17117
 
 tss
 
 0.494840
 
 0.5613993
 
 Not queried
 
  1.1044499
 
       NA
 
           NA
  
 
  
 21
 
  38070524
 
  38071066
 
  ENSEMBL   UCSC 
 
  543
 
 11
 
 26538
 
 tss;island
 
 0.039971
 
 0.0405470
 
 Not queried
 
  1.1041085
 
       NA
 
           NA
  
 
  
 19
 
  15311886
 
  15312233
 
  ENSEMBL   UCSC 
 
  348
 
  5
 
 20556
 
 tss;island
 
 0.701270
 
 0.0394334
 
 Not queried
 
  1.1039975
 
       NA
 
           NA
  
 
  
 2
 
  39893078
 
  39893520
 
  ENSEMBL   UCSC 
 
  443
 
  6
 
 22654
 
 gene;island
 
 0.021605
 
 0.0269329
 
 Not queried
 
  1.1036707
 
       NA
 
           NA
  
 
  
 18
 
  43608309
 
  43608548
 
  ENSEMBL   UCSC 
 
  240
 
  4
 
 19098
 
 gene;island
 
 0.058912
 
 0.2504611
 
 Not queried
 
  1.1036539
 
       NA
 
           NA
  
 
  
 1
 
  86042661
 
  86042732
 
  ENSEMBL   UCSC 
 
   72
 
  4
 
 607
 
 gene;island
 
 0.007561
 
 0.0039968
 
 Not queried
 
  1.1035160
 
       NA
 
           NA
  
 
  
 12
 
 112204716
 
 112204829
 
  ENSEMBL   UCSC 
 
  114
 
  4
 
 9312
 
 gene;island
 
 0.068305
 
 0.0312618
 
 Not queried
 
  1.1034999
 
       NA
 
           NA
  
 
  
 1
 
 154245185
 
 154245414
 
  ENSEMBL   UCSC 
 
  230
 
  5
 
 776
 
 gene;island
 
 0.012825
 
 0.0169256
 
 Not queried
 
  1.1033804
 
       NA
 
           NA
  
 
  
 16
 
  29674903
 
  29675214
 
  ENSEMBL   UCSC 
 
  312
 
  5
 
 14710
 
 gene;island
 
 0.008879
 
 0.0106220
 
 Not queried
 
  1.1026647
 
       NA
 
           NA
  
 
  
 3
 
 195489708
 
 195490309
 
  ENSEMBL   UCSC 
 
  602
 
  8
 
 28094
 
 gene;island
 
 0.603140
 
 0.7397986
 
 Not queried
 
  1.1026350
 
       NA
 
           NA
  
 
  
 17
 
   4689640
 
   4689893
 
  ENSEMBL   UCSC 
 
  254
 
  6
 
 17878
 
 island
 
 0.032435
 
 0.0155342
 
 Not queried
 
  1.1024564
 
       NA
 
           NA
  
 
  
 3
 
 196756621
 
 196756875
 
  ENSEMBL   UCSC 
 
  255
 
  4
 
 29815
 
 island
 
 0.002766
 
 0.0055286
 
 Not queried
 
  1.1022993
 
       NA
 
           NA
  
 
  
 3
 
 160116810
 
 160117091
 
  ENSEMBL   UCSC 
 
  282
 
  4
 
 28014
 
 gene;tss;island
 
 0.001835
 
 0.0026844
 
 Not queried
 
  1.1016663
 
       NA
 
           NA
  
 
  
 5
 
 176074273
 
 176074358
 
  ENSEMBL   UCSC 
 
   86
 
  4
 
 32517
 
 tss
 
 0.005127
 
 0.0028019
 
 Not queried
 
  1.1016439
 
       NA
 
           NA
  
 
  
 19
 
  36035831
 
  36036881
 
  ENSEMBL   UCSC 
 
 1051
 
 10
 
 21906
 
 island
 
 0.023615
 
 0.0105487
 
 Not queried
 
  1.1015042
 
       NA
 
           NA
  
 
  
 22
 
  38506712
 
  38506781
 
  ENSEMBL   UCSC 
 
   70
 
  4
 
 27131
 
 tss
 
 0.931120
 
 0.9658381
 
 Not queried
 
  1.1014401
 
       NA
 
           NA
  
 
  
 8
 
   1899127
 
   1899538
 
  ENSEMBL   UCSC 
 
  412
 
  5
 
 39184
 
 gene
 
 0.624880
 
 0.8935111
 
 Not queried
 
  1.1013992
 
       NA
 
           NA
  
 
  
 17
 
  53315783
 
  53316308
 
  ENSEMBL   UCSC 
 
  526
 
  5
 
 18574
 
 island
 
 0.029175
 
 0.0009770
 
 Not queried
 
  1.1013932
 
       NA
 
           NA
  
 
  
 12
 
 124864528
 
 124864681
 
  ENSEMBL   UCSC 
 
  154
 
  4
 
 9392
 
 gene
 
 0.379857
 
 0.5473844
 
 Not queried
 
  1.1009149
 
       NA
 
           NA
  
 
  
 17
 
   7121848
 
   7122261
 
  ENSEMBL   UCSC 
 
  414
 
  4
 
 16385
 
 gene;tss;island
 
 0.623850
 
 0.7988524
 
 Not queried
 
  1.1006224
 
       NA
 
           NA
  
 
  
 7
 
 102553293
 
 102553457
 
  ENSEMBL   UCSC 
 
  165
 
  5
 
 37169
 
 gene
 
 0.712222
 
 0.8815944
 
 Not queried
 
  1.1005682
 
       NA
 
           NA
  
 
  
 14
 
  78227565
 
  78227771
 
  ENSEMBL   UCSC 
 
  207
 
  8
 
 12158
 
 gene;tss;island
 
 0.002275
 
 0.0009962
 
 Not queried
 
  1.1003531
 
       NA
 
           NA
  
 
  
 2
 
 239049389
 
 239049701
 
  ENSEMBL   UCSC 
 
  313
 
  5
 
 23095
 
 gene;island
 
 0.836218
 
 0.8799602
 
 Not queried
 
  1.1002881
 
       NA
 
           NA
  
 
  
 11
 
 108338268
 
 108338461
 
  ENSEMBL   UCSC 
 
  194
 
  7
 
 7671
 
 tss
 
 0.006474
 
 0.0038763
 
 Not queried
 
  1.1001799
 
       NA
 
           NA
  
 
  
 3
 
 149374761
 
 149375293
 
  ENSEMBL   UCSC 
 
  533
 
  7
 
 27992
 
 gene;island
 
 0.024324
 
 0.1122739
 
 Not queried
 
  1.1001161
 
       NA
 
           NA
  
 
  
 19
 
  11449933
 
  11450261
 
  ENSEMBL   UCSC 
 
  329
 
  4
 
 19834
 
 gene;island
 
 0.006400
 
 0.0039633
 
 Not queried
 
  1.0997155
 
       NA
 
           NA
  
 
  
 2
 
 223916502
 
 223916820
 
  ENSEMBL   UCSC 
 
  319
 
  5
 
 23921
 
 tss
 
 0.677855
 
 0.8846883
 
 Not queried
 
  1.0995592
 
       NA
 
           NA
  
 
  
 2
 
 223916502
 
 223916952
 
  ENSEMBL   UCSC 
 
  451
 
  7
 
 25157
 
 island
 
 0.677855
 
 0.8077871
 
 Not queried
 
  1.0995592
 
       NA
 
           NA
  
 
  
 1
 
  84543951
 
  84544284
 
  ENSEMBL   UCSC 
 
  334
 
  4
 
 599
 
 gene;island
 
 0.006919
 
 0.0081997
 
 Not queried
 
  1.0994700
 
       NA
 
           NA
  
 
  
 2
 
 131674361
 
 131674687
 
  ENSEMBL   UCSC 
 
  327
 
  4
 
 22868
 
 gene
 
 0.564036
 
 0.7933147
 
 Not queried
 
  1.0993279
 
       NA
 
           NA
  
 
  
 1
 
  23495375
 
  23495577
 
  ENSEMBL   UCSC 
 
  203
 
  8
 
 1335
 
 tss
 
 0.013080
 
 0.0093788
 
 Not queried
 
  1.0992326
 
       NA
 
           NA
  
 
  
 3
 
  33318720
 
  33319219
 
  ENSEMBL   UCSC 
 
  500
 
  7
 
 28973
 
 island
 
 0.013541
 
 0.0170595
 
 Not queried
 
  1.0991530
 
       NA
 
           NA
  
 
  
 4
 
  15005434
 
  15005593
 
  ENSEMBL   UCSC 
 
  160
 
  7
 
 30936
 
 island
 
 0.002967
 
 0.0015605
 
 Not queried
 
  1.0989606
 
       NA
 
           NA
  
 
  
 5
 
 150326174
 
 150326642
 
  ENSEMBL   UCSC 
 
  469
 
  8
 
 32436
 
 tss
 
 0.252559
 
 0.4709870
 
 Not queried
 
  1.0986190
 
       NA
 
           NA
  
 
  
 19
 
  42894425
 
  42894693
 
  ENSEMBL   UCSC 
 
  269
 
  4
 
 22079
 
 island
 
 0.342041
 
 0.4592973
 
 Not queried
 
  1.0983219
 
       NA
 
           NA
  
 
  
 2
 
 241640149
 
 241640444
 
  ENSEMBL   UCSC 
 
  296
 
  4
 
 25278
 
 island
 
 0.795147
 
 0.8835272
 
 Not queried
 
  1.0982096
 
       NA
 
           NA
  
 
  
 2
 
  27632449
 
  27632926
 
  ENSEMBL   UCSC 
 
  478
 
  6
 
 23268
 
 tss
 
 0.005683
 
 0.0105868
 
 Not queried
 
  1.0979740
 
       NA
 
           NA
  
 
  
 8
 
  82644373
 
  82645116
 
  ENSEMBL   UCSC 
 
  744
 
 10
 
 40422
 
 island
 
 0.027229
 
 0.0120800
 
 Not queried
 
  1.0974916
 
       NA
 
           NA
  
 
  
 19
 
  48018510
 
  48018781
 
  ENSEMBL   UCSC 
 
  272
 
  9
 
 20907
 
 tss
 
 0.001251
 
 0.0027871
 
 Not queried
 
  1.0974176
 
       NA
 
           NA
  
 
  
 5
 
 141071381
 
 141071893
 
  ENSEMBL   UCSC 
 
  513
 
  6
 
 33260
 
 island
 
 0.007276
 
 0.0087051
 
 Not queried
 
  1.0973353
 
       NA
 
           NA
  
 
  
 11
 
   1471410
 
   1471699
 
  ENSEMBL   UCSC 
 
  290
 
  6
 
 6420
 
 gene;island
 
 0.627792
 
 0.7668112
 
 Not queried
 
  1.0971910
 
       NA
 
           NA
  
 
  
 7
 
   2078650
 
   2079008
 
  ENSEMBL   UCSC 
 
  359
 
  5
 
 36787
 
 gene
 
 0.059643
 
 0.0124568
 
 Not queried
 
  1.0969251
 
       NA
 
           NA
  
 
  
 8
 
  73163524
 
  73164024
 
  ENSEMBL   UCSC 
 
  501
 
  5
 
 40386
 
 island
 
 0.017241
 
 0.0518320
 
 Not queried
 
  1.0967604
 
       NA
 
           NA
  
 
  
 17
 
  26925852
 
  26926050
 
  ENSEMBL   UCSC 
 
  199
 
  4
 
 16498
 
 gene
 
 0.011363
 
 0.0096341
 
 Not queried
 
  1.0965444
 
       NA
 
           NA
  
 
  
 13
 
  50707427
 
  50708043
 
  ENSEMBL   UCSC 
 
  617
 
  5
 
 11744
 
 island
 
 0.610739
 
 0.9482117
 
 Not queried
 
  1.0965350
 
       NA
 
           NA
  
 
  
 6
 
  31895464
 
  31895598
 
  ENSEMBL   UCSC 
 
  135
 
  4
 
 33994
 
 gene
 
 0.271638
 
 0.3706504
 
 Not queried
 
  1.0963692
 
       NA
 
           NA
  
 
  
 1
 
   2776972
 
   2777244
 
  ENSEMBL   UCSC 
 
  273
 
  4
 
 2519
 
 island
 
 0.303466
 
 0.4172026
 
 Not queried
 
  1.0961682
 
       NA
 
           NA
  
 
  
 12
 
 108985685
 
 108986019
 
  ENSEMBL   UCSC 
 
  335
 
  4
 
 9286
 
 gene
 
 0.832538
 
 0.9249442
 
 Not queried
 
  1.0958376
 
       NA
 
           NA
  
 
  
 14
 
  57283942
 
  57284319
 
  ENSEMBL   UCSC 
 
  378
 
  4
 
 12093
 
 gene;island
 
 0.032610
 
 0.0514906
 
 Not queried
 
  1.0958199
 
       NA
 
           NA
  
 
  
 15
 
  42500107
 
  42500463
 
  ENSEMBL   UCSC 
 
  357
 
  5
 
 13309
 
 gene
 
 0.039594
 
 0.0253223
 
 Not queried
 
  1.0957788
 
       NA
 
           NA
  
 
  
 1
 
 159046773
 
 159047163
 
  ENSEMBL   UCSC 
 
  391
 
  5
 
 2028
 
 tss
 
 0.002187
 
 0.0004088
 
 Not queried
 
  1.0955743
 
       NA
 
           NA
  
 
  
 10
 
 103603563
 
 103604019
 
  ENSEMBL   UCSC 
 
  457
 
  7
 
 6074
 
 island
 
 0.035976
 
 0.0281197
 
 Not queried
 
  1.0954438
 
       NA
 
           NA
  
 
  
 13
 
 114214248
 
 114214740
 
  ENSEMBL   UCSC 
 
  493
 
  5
 
 11971
 
 island
 
 0.829179
 
 0.9497738
 
 Not queried
 
  1.0953707
 
       NA
 
           NA
  
 
  
 13
 
 114103452
 
 114103796
 
  ENSEMBL   UCSC 
 
  345
 
  7
 
 11286
 
 gene
 
 0.867038
 
 0.8988706
 
 Not queried
 
  1.0953328
 
       NA
 
           NA
  
 
  
 1
 
 154377807
 
 154378025
 
  ENSEMBL   UCSC 
 
  219
 
  6
 
 777
 
 gene
 
 0.004784
 
 0.0137430
 
 Not queried
 
  1.0952409
 
       NA
 
           NA
  
 
  
 17
 
  79047681
 
  79047872
 
  ENSEMBL   UCSC 
 
  192
 
  4
 
 16892
 
 gene;island
 
 0.677880
 
 0.8001186
 
 Not queried
 
  1.0952159
 
       NA
 
           NA
  
 
  
 15
 
  65341703
 
  65341995
 
  ENSEMBL   UCSC 
 
  293
 
  5
 
 13378
 
 gene
 
 0.002834
 
 0.0078829
 
 Not queried
 
  1.0950239
 
       NA
 
           NA
  
 
  
 19
 
  46417551
 
  46417734
 
  ENSEMBL   UCSC 
 
  184
 
  4
 
 20107
 
 gene;island
 
 0.593976
 
 0.7924557
 
 Not queried
 
  1.0947478
 
       NA
 
           NA
  
 
  
 2
 
 183731169
 
 183732062
 
  ENSEMBL   UCSC 
 
  894
 
 10
 
 24941
 
 island
 
 0.047991
 
 0.0273263
 
 Not queried
 
  1.0946311
 
       NA
 
           NA
  
 
  
 20
 
  48552575
 
  48552853
 
  ENSEMBL   UCSC 
 
  279
 
  7
 
 25813
 
 tss;island
 
 0.016544
 
 0.0156926
 
 Not queried
 
  1.0941108
 
       NA
 
           NA
  
 
  
 1
 
  12677936
 
  12678355
 
  ENSEMBL   UCSC 
 
  420
 
  7
 
 1259
 
 tss;island
 
 0.005582
 
 0.0030335
 
 Not queried
 
  1.0940552
 
       NA
 
           NA
  
 
  
 15
 
  76195876
 
  76196799
 
  ENSEMBL   UCSC 
 
  924
 
  9
 
 14337
 
 island
 
 0.006169
 
 0.0157116
 
 Not queried
 
  1.0939099
 
       NA
 
           NA
  
 
  
 2
 
 208989209
 
 208989579
 
  ENSEMBL   UCSC 
 
  371
 
  7
 
 25047
 
 island
 
 0.458259
 
 0.0975279
 
 Not queried
 
  1.0938558
 
       NA
 
           NA
  
 
  
 8
 
  23261731
 
  23262159
 
  ENSEMBL   UCSC 
 
  429
 
  6
 
 39600
 
 tss
 
 0.049600
 
 0.1658243
 
 Not queried
 
  1.0935516
 
       NA
 
           NA
  
 
  
 6
 
 111408745
 
 111408840
 
  ENSEMBL   UCSC 
 
   96
 
  6
 
 36418
 
 island
 
 0.030514
 
 0.0216327
 
 Not queried
 
  1.0935161
 
       NA
 
           NA
  
 
  
 5
 
  92914036
 
  92914319
 
  ENSEMBL   UCSC 
 
  284
 
  5
 
 31689
 
 gene;island
 
 0.083754
 
 0.0571700
 
 Not queried
 
  1.0935051
 
       NA
 
           NA
  
 
  
 13
 
 113783975
 
 113784438
 
  ENSEMBL   UCSC 
 
  464
 
  4
 
 11276
 
 gene;island
 
 0.682445
 
 0.6871485
 
 Not queried
 
  1.0933172
 
       NA
 
           NA
  
 
  
 7
 
 157919649
 
 157920174
 
  ENSEMBL   UCSC 
 
  526
 
  4
 
 37359
 
 gene;island
 
 0.461429
 
 0.6369851
 
 Not queried
 
  1.0931175
 
       NA
 
           NA
  
 
  
 20
 
  44538605
 
  44538675
 
  ENSEMBL   UCSC 
 
   71
 
  4
 
 25437
 
 gene;island
 
 0.898998
 
 0.7897317
 
 Not queried
 
  1.0927381
 
       NA
 
           NA
  
 
  
 17
 
  48350468
 
  48350615
 
  ENSEMBL   UCSC 
 
  148
 
  4
 
 16684
 
 gene;tss;island
 
 0.045860
 
 0.0233689
 
 Not queried
 
  1.0927317
 
       NA
 
           NA
  
 
  
 12
 
 110156245
 
 110156459
 
  ENSEMBL   UCSC 
 
  215
 
  5
 
 9295
 
 gene;island
 
 0.430420
 
 0.1859958
 
 Not queried
 
  1.0922806
 
       NA
 
           NA
  
 
  
 17
 
  74467662
 
  74468141
 
  ENSEMBL   UCSC 
 
  480
 
  4
 
 16789
 
 gene;island
 
 0.880291
 
 0.9411227
 
 Not queried
 
  1.0922463
 
       NA
 
           NA
  
 
  
 3
 
 128151940
 
 128152268
 
  ENSEMBL   UCSC 
 
  329
 
  4
 
 29453
 
 island
 
 0.176838
 
 0.0991374
 
 Not queried
 
  1.0920481
 
       NA
 
           NA
  
 
  
 21
 
  38077258
 
  38077673
 
  ENSEMBL   UCSC 
 
  416
 
  6
 
 26419
 
 gene;island
 
 0.060038
 
 0.0314114
 
 Not queried
 
  1.0916789
 
       NA
 
           NA
  
 
  
 10
 
  25465219
 
  25465503
 
  ENSEMBL   UCSC 
 
  285
 
  7
 
 4992
 
 tss
 
 0.054268
 
 0.0807519
 
 Not queried
 
  1.0916634
 
       NA
 
           NA
  
 
  
 15
 
  90319420
 
  90319680
 
  ENSEMBL   UCSC 
 
  261
 
  5
 
 14442
 
 island
 
 0.048741
 
 0.0874889
 
 Not queried
 
  1.0912259
 
       NA
 
           NA
  
 
  
 1
 
   7765245
 
   7765564
 
  ENSEMBL   UCSC 
 
  320
 
  4
 
 188
 
 gene;island
 
 0.010054
 
 0.0130439
 
 Not queried
 
  1.0911859
 
       NA
 
           NA
  
 
  
 1
 
   3184434
 
   3184704
 
  ENSEMBL   UCSC 
 
  271
 
  4
 
 115
 
 gene;island
 
 0.657910
 
 0.8040562
 
 Not queried
 
  1.0911797
 
       NA
 
           NA
  
 
  
 17
 
  19140625
 
  19140672
 
  ENSEMBL   UCSC 
 
   48
 
  6
 
 17185
 
 tss;island
 
 0.003200
 
 0.0063465
 
 Not queried
 
  1.0910825
 
       NA
 
           NA
  
 
  
 10
 
  63213104
 
  63213510
 
  ENSEMBL   UCSC 
 
  407
 
  7
 
 5768
 
 island
 
 0.070881
 
 0.2450456
 
 Not queried
 
  1.0908335
 
       NA
 
           NA
  
 
  
 15
 
  71184279
 
  71184618
 
  ENSEMBL   UCSC 
 
  340
 
  5
 
 13401
 
 gene
 
 0.027733
 
 0.0163823
 
 Not queried
 
  1.0907894
 
       NA
 
           NA
  
 
  
 13
 
  37004536
 
  37005582
 
  ENSEMBL   UCSC 
 
 1047
 
 29
 
 11396
 
 tss;island
 
 0.017602
 
 0.0322881
 
 Not queried
 
  1.0906386
 
       NA
 
           NA
  
 
  
 8
 
  65492528
 
  65492792
 
  ENSEMBL   UCSC 
 
  265
 
  8
 
 39736
 
 tss
 
 0.004297
 
 0.0012435
 
 Not queried
 
  1.0906340
 
       NA
 
           NA
  
 
  
 11
 
  13983705
 
  13984067
 
  ENSEMBL   UCSC 
 
  363
 
 10
 
 8072
 
 island
 
 0.750115
 
 0.8757462
 
 Not queried
 
  1.0905869
 
       NA
 
           NA
  
 
  
 19
 
  44905782
 
  44905980
 
  ENSEMBL   UCSC 
 
  199
 
  7
 
 20848
 
 tss
 
 0.063600
 
 0.0361140
 
 Not queried
 
  1.0905191
 
       NA
 
           NA
  
 
  
 11
 
  65657704
 
  65657895
 
  ENSEMBL   UCSC 
 
  192
 
  8
 
 8489
 
 island
 
 0.024531
 
 0.0161849
 
 Not queried
 
  1.0903629
 
       NA
 
           NA
  
 
  
 18
 
  51750950
 
  51751092
 
  ENSEMBL   UCSC 
 
  143
 
  4
 
 19118
 
 gene
 
 0.004350
 
 0.0035837
 
 Not queried
 
  1.0900543
 
       NA
 
           NA
  
 
  
 1
 
 156211409
 
 156211896
 
  ENSEMBL   UCSC 
 
  488
 
 10
 
 2011
 
 tss
 
 0.184674
 
 0.4677842
 
 Not queried
 
  1.0899926
 
       NA
 
           NA
  
 
  
 7
 
  27138712
 
  27138974
 
  ENSEMBL   UCSC 
 
  263
 
  4
 
 38337
 
 island
 
 0.984814
 
 0.9772826
 
 Not queried
 
  1.0895757
 
       NA
 
           NA
  
 
  
 11
 
   1330593
 
   1330840
 
  ENSEMBL   UCSC 
 
  248
 
  6
 
 7040
 
 tss;island
 
 0.007301
 
 0.0069738
 
 Not queried
 
  1.0889520
 
       NA
 
           NA
  
 
  
 7
 
   2595131
 
   2595589
 
  ENSEMBL   UCSC 
 
  459
 
  9
 
 38163
 
 island
 
 0.005055
 
 0.0084863
 
 Not queried
 
  1.0887352
 
       NA
 
           NA
  
 
  
 12
 
  22777790
 
  22778020
 
  ENSEMBL   UCSC 
 
  231
 
  8
 
 9604
 
 tss
 
 0.014820
 
 0.0095447
 
 Not queried
 
  1.0886828
 
       NA
 
           NA
  
 
  
 5
 
 156886816
 
 156887005
 
  ENSEMBL   UCSC 
 
  190
 
  6
 
 32456
 
 tss
 
 0.023751
 
 0.0396721
 
 Not queried
 
  1.0885881
 
       NA
 
           NA
  
 
  
 7
 
 156792850
 
 156793362
 
  ENSEMBL   UCSC 
 
  513
 
  4
 
 39089
 
 island
 
 0.007874
 
 0.0056419
 
 Not queried
 
  1.0877418
 
       NA
 
           NA
  
 
  
 19
 
  55173482
 
  55173527
 
  ENSEMBL   UCSC 
 
   46
 
  4
 
 21064
 
 tss
 
 0.105558
 
 0.1813079
 
 Not queried
 
  1.0875874
 
       NA
 
           NA
  
 
  
 13
 
 100497453
 
 100497789
 
  ENSEMBL   UCSC 
 
  337
 
  4
 
 11206
 
 gene
 
 0.793751
 
 0.8950569
 
 Not queried
 
  1.0875537
 
       NA
 
           NA
  
 
  
 12
 
 121416315
 
 121416796
 
  ENSEMBL   UCSC 
 
  482
 
  9
 
 10904
 
 island
 
 0.603944
 
 0.8300423
 
 Not queried
 
  1.0875319
 
       NA
 
           NA
  
 
  
 19
 
  50145283
 
  50145354
 
  ENSEMBL   UCSC 
 
   72
 
  4
 
 20956
 
 tss;island
 
 0.005404
 
 0.0084891
 
 Not queried
 
  1.0874736
 
       NA
 
           NA
  
 
  
 10
 
 126107471
 
 126107861
 
  ENSEMBL   UCSC 
 
  391
 
 11
 
 6227
 
 island
 
 0.027944
 
 0.0275288
 
 Not queried
 
  1.0871760
 
       NA
 
           NA
  
 
  
 16
 
   3333472
 
   3333539
 
  ENSEMBL   UCSC 
 
   68
 
  4
 
 15645
 
 island
 
 0.003041
 
 0.0037092
 
 Not queried
 
  1.0870364
 
       NA
 
           NA
  
 
  
 6
 
  32185818
 
  32186244
 
  ENSEMBL   UCSC 
 
  427
 
  9
 
 34097
 
 gene
 
 0.386838
 
 0.4972215
 
 Not queried
 
  1.0869136
 
       NA
 
           NA
  
 
  
 3
 
  58419588
 
  58419937
 
  ENSEMBL   UCSC 
 
  350
 
  7
 
 28417
 
 tss
 
 0.007823
 
 0.0042427
 
 Not queried
 
  1.0866878
 
       NA
 
           NA
  
 
  
 12
 
  67072951
 
  67073062
 
  ENSEMBL   UCSC 
 
  112
 
  4
 
 9850
 
 tss
 
 0.292726
 
 0.5904812
 
 Not queried
 
  1.0866671
 
       NA
 
           NA
  
 
  
 19
 
  10691765
 
  10692029
 
  ENSEMBL   UCSC 
 
  265
 
  6
 
 19816
 
 gene;island
 
 0.024000
 
 0.0060257
 
 Not queried
 
  1.0866442
 
       NA
 
           NA
  
 
  
 7
 
 158886098
 
 158886532
 
  ENSEMBL   UCSC 
 
  435
 
  6
 
 37384
 
 gene;island
 
 0.646955
 
 0.8048257
 
 Not queried
 
  1.0865590
 
       NA
 
           NA
  
 
  
 8
 
  70983348
 
  70983760
 
  ENSEMBL   UCSC 
 
  413
 
  7
 
 40374
 
 island
 
 0.129940
 
 0.1093391
 
 Not queried
 
  1.0862322
 
       NA
 
           NA
  
 
  
 13
 
  77460607
 
  77460723
 
  ENSEMBL   UCSC 
 
  117
 
  6
 
 11481
 
 tss;island
 
 0.005284
 
 0.0042747
 
 Not queried
 
  1.0860764
 
       NA
 
           NA
  
 
  
 21
 
  43491213
 
  43491424
 
  ENSEMBL   UCSC 
 
  212
 
  5
 
 26563
 
 tss
 
 0.279321
 
 0.3585680
 
 Not queried
 
  1.0859752
 
       NA
 
           NA
  
 
  
 8
 
 143609379
 
 143609637
 
  ENSEMBL   UCSC 
 
  259
 
  4
 
 39465
 
 gene;island
 
 0.460969
 
 0.6315951
 
 Not queried
 
  1.0859116
 
       NA
 
           NA
  
 
  
 8
 
 104426639
 
 104427370
 
  ENSEMBL   UCSC 
 
  732
 
  9
 
 39391
 
 gene;island
 
 0.013216
 
 0.0068837
 
 Not queried
 
  1.0857027
 
       NA
 
           NA
  
 
  
 1
 
 216774208
 
 216774677
 
  ENSEMBL   UCSC 
 
  470
 
  6
 
 1010
 
 gene;island
 
 0.007767
 
 0.0156079
 
 Not queried
 
  1.0856877
 
       NA
 
           NA
  
 
  
 12
 
 118810744
 
 118811074
 
  ENSEMBL   UCSC 
 
  331
 
 10
 
 10869
 
 island
 
 0.008224
 
 0.0051222
 
 Not queried
 
  1.0856836
 
       NA
 
           NA
  
 
  
 7
 
   4275634
 
   4275962
 
  ENSEMBL   UCSC 
 
  329
 
  4
 
 36837
 
 gene
 
 0.305580
 
 0.4766892
 
 Not queried
 
  1.0855920
 
       NA
 
           NA
  
 
  
 1
 
 107682674
 
 107682963
 
  ENSEMBL   UCSC 
 
  290
 
  4
 
 657
 
 gene
 
 0.029558
 
 0.0735091
 
 Not queried
 
  1.0853562
 
       NA
 
           NA
  
 
  
 17
 
  79374181
 
  79374741
 
  ENSEMBL   UCSC 
 
  561
 
  6
 
 16906
 
 gene;island
 
 0.563507
 
 0.3922278
 
 Not queried
 
  1.0853230
 
       NA
 
           NA
  
 
  
 1
 
  10714469
 
  10714701
 
  ENSEMBL   UCSC 
 
  233
 
  4
 
 213
 
 gene;island
 
 0.889092
 
 0.7008472
 
 Not queried
 
  1.0848651
 
       NA
 
           NA
  
 
  
 7
 
 106505688
 
 106505914
 
  ENSEMBL   UCSC 
 
  227
 
  4
 
 37822
 
 tss
 
 0.011242
 
 0.0043103
 
 Not queried
 
  1.0847942
 
       NA
 
           NA
  
 
  
 6
 
 169242769
 
 169243075
 
  ENSEMBL   UCSC 
 
  307
 
  4
 
 36668
 
 island
 
 0.709544
 
 0.8549114
 
 Not queried
 
  1.0846489
 
       NA
 
           NA
  
 
  
 5
 
   1282984
 
   1283350
 
  ENSEMBL   UCSC 
 
  367
 
  4
 
 31533
 
 gene;island
 
 0.795045
 
 0.6918076
 
 Not queried
 
  1.0845136
 
       NA
 
           NA
  
 
  
 21
 
  45811363
 
  45811549
 
  ENSEMBL   UCSC 
 
  187
 
  4
 
 26456
 
 gene;island
 
 0.969189
 
 0.9422270
 
 Not queried
 
  1.0845126
 
       NA
 
           NA
  
 
  
 16
 
  15595854
 
  15596082
 
  ENSEMBL   UCSC 
 
  229
 
  6
 
 15107
 
 tss
 
 0.513677
 
 0.5918291
 
 Not queried
 
  1.0844371
 
       NA
 
           NA
  
 
  
 5
 
 169532686
 
 169533120
 
  ENSEMBL   UCSC 
 
  435
 
  8
 
 33376
 
 island
 
 0.444368
 
 0.7363530
 
 Not queried
 
  1.0843655
 
       NA
 
           NA
  
 
  
 6
 
   8436218
 
   8436466
 
  ENSEMBL   UCSC 
 
  249
 
  4
 
 34621
 
 tss;island
 
 0.861906
 
 0.9820643
 
 Not queried
 
  1.0843335
 
       NA
 
           NA
  
 
  
 20
 
  62290277
 
  62290499
 
  ENSEMBL   UCSC 
 
  223
 
  4
 
 25505
 
 gene;island
 
 0.027861
 
 0.0221258
 
 Not queried
 
  1.0840024
 
       NA
 
           NA
  
 
  
 2
 
 176971820
 
 176972113
 
  ENSEMBL   UCSC 
 
  294
 
  8
 
 24896
 
 island
 
 0.118719
 
 0.2931865
 
 Not queried
 
  1.0840023
 
       NA
 
           NA
  
 
  
 4
 
  84456674
 
  84457116
 
  ENSEMBL   UCSC 
 
  443
 
  4
 
 30477
 
 tss;island
 
 0.003683
 
 0.0017865
 
 Not queried
 
  1.0839388
 
       NA
 
           NA
  
 
  
 7
 
 128049508
 
 128049980
 
  ENSEMBL   UCSC 
 
  473
 
  7
 
 37213
 
 gene
 
 0.005465
 
 0.0051380
 
 Not queried
 
  1.0838658
 
       NA
 
           NA
  
 
  
 10
 
  47083283
 
  47083632
 
  ENSEMBL   UCSC 
 
  350
 
  9
 
 5719
 
 island
 
 0.087603
 
 0.0226622
 
 Not queried
 
  1.0838346
 
       NA
 
           NA
  
 
  
 15
 
  25436114
 
  25436246
 
  ENSEMBL   UCSC 
 
  133
 
  4
 
 13532
 
 tss
 
 0.502850
 
 0.5584663
 
 Not queried
 
  1.0835469
 
       NA
 
           NA
  
 
  
 7
 
   4153707
 
   4153919
 
  ENSEMBL   UCSC 
 
  213
 
  5
 
 36827
 
 gene;island
 
 0.391625
 
 0.4006930
 
 Not queried
 
  1.0832767
 
       NA
 
           NA
  
 
  
 16
 
  58073817
 
  58074046
 
  ENSEMBL   UCSC 
 
  230
 
  6
 
 14770
 
 gene;island
 
 0.854320
 
 0.8975031
 
 Not queried
 
  1.0832581
 
       NA
 
           NA
  
 
  
 17
 
  44061117
 
  44061467
 
  ENSEMBL   UCSC 
 
  351
 
  4
 
 16643
 
 gene;island
 
 0.563715
 
 0.7895279
 
 Not queried
 
  1.0831363
 
       NA
 
           NA
  
 
  
 20
 
  61041274
 
  61041765
 
  ENSEMBL   UCSC 
 
  492
 
  5
 
 25486
 
 gene;island
 
 0.453040
 
 0.6058179
 
 Not queried
 
  1.0829346
 
       NA
 
           NA
  
 
  
 21
 
  46706579
 
  46707012
 
  ENSEMBL   UCSC 
 
  434
 
  4
 
 26465
 
 gene;tss;island
 
 0.862606
 
 0.8398641
 
 Not queried
 
  1.0828877
 
       NA
 
           NA
  
 
  
 4
 
   2935884
 
   2936284
 
  ENSEMBL   UCSC 
 
  401
 
  8
 
 30254
 
 tss
 
 0.004710
 
 0.0046700
 
 Not queried
 
  1.0827326
 
       NA
 
           NA
  
 
  
 11
 
  20620680
 
  20620920
 
  ENSEMBL   UCSC 
 
  241
 
  6
 
 7199
 
 tss
 
 0.360111
 
 0.2315085
 
 Not queried
 
  1.0826068
 
       NA
 
           NA
  
 
  
 12
 
  45269519
 
  45269790
 
  ENSEMBL   UCSC 
 
  272
 
  5
 
 9651
 
 tss
 
 0.005253
 
 0.0165035
 
 Not queried
 
  1.0825822
 
       NA
 
           NA
  
 
  
 11
 
  67206263
 
  67206434
 
  ENSEMBL   UCSC 
 
  172
 
  4
 
 6756
 
 gene;tss;island
 
 0.917099
 
 0.9635734
 
 Not queried
 
  1.0816616
 
       NA
 
           NA
  
 
  
 14
 
  61116782
 
  61117162
 
  ENSEMBL   UCSC 
 
  381
 
  5
 
 12440
 
 tss;island
 
 0.040548
 
 0.0191597
 
 Not queried
 
  1.0816596
 
       NA
 
           NA
  
 
  
 19
 
  51293367
 
  51293654
 
  ENSEMBL   UCSC 
 
  288
 
  5
 
 20985
 
 tss
 
 0.884532
 
 0.8065595
 
 Not queried
 
  1.0814287
 
       NA
 
           NA
  
 
  
 8
 
 121457799
 
 121457935
 
  ENSEMBL   UCSC 
 
  137
 
  4
 
 39412
 
 gene
 
 0.006151
 
 0.0095617
 
 Not queried
 
  1.0813696
 
       NA
 
           NA
  
 
  
 17
 
  79604207
 
  79604385
 
  ENSEMBL   UCSC 
 
  179
 
  7
 
 17742
 
 tss
 
 0.011860
 
 0.0045987
 
 Not queried
 
  1.0812500
 
       NA
 
           NA
  
 
  
 7
 
 107220968
 
 107221141
 
  ENSEMBL   UCSC 
 
  174
 
  4
 
 37828
 
 tss
 
 0.007089
 
 0.0057095
 
 Not queried
 
  1.0811898
 
       NA
 
           NA
  
 
  
 8
 
 129061048
 
 129061382
 
  ENSEMBL   UCSC 
 
  335
 
  4
 
 39431
 
 gene;tss
 
 0.025199
 
 0.0485773
 
 Not queried
 
  1.0811843
 
       NA
 
           NA
  
 
  
 22
 
  46450059
 
  46450114
 
  ENSEMBL   UCSC 
 
   56
 
  4
 
 27227
 
 tss
 
 0.068238
 
 0.0145566
 
 Not queried
 
  1.0810117
 
       NA
 
           NA
  
 
  
 6
 
 167051518
 
 167051781
 
  ENSEMBL   UCSC 
 
  264
 
  5
 
 34552
 
 gene
 
 0.698744
 
 0.9031495
 
 Not queried
 
  1.0809829
 
       NA
 
           NA
  
 
  
 10
 
 117884825
 
 117885028
 
  ENSEMBL   UCSC 
 
  204
 
  4
 
 4771
 
 gene;island
 
 0.357548
 
 0.5314099
 
 Not queried
 
  1.0807238
 
       NA
 
           NA
  
 
  
 3
 
  44063315
 
  44063832
 
  ENSEMBL   UCSC 
 
  518
 
  6
 
 29031
 
 island
 
 0.015143
 
 0.0064353
 
 Not queried
 
  1.0804765
 
       NA
 
           NA
  
 
  
 3
 
  35680791
 
  35680899
 
  ENSEMBL   UCSC 
 
  109
 
  4
 
 28977
 
 island
 
 0.071782
 
 0.2257004
 
 Not queried
 
  1.0803958
 
       NA
 
           NA
  
 
  
 11
 
   1948933
 
   1949113
 
  ENSEMBL   UCSC 
 
  181
 
  5
 
 6439
 
 gene;island
 
 0.583336
 
 0.7626859
 
 Not queried
 
  1.0800579
 
       NA
 
           NA
  
 
  
 2
 
  85843042
 
  85843380
 
  ENSEMBL   UCSC 
 
  339
 
 10
 
 24486
 
 island
 
 0.006974
 
 0.0044216
 
 Not queried
 
  1.0798755
 
       NA
 
           NA
  
 
  
 12
 
   6798753
 
   6799136
 
  ENSEMBL   UCSC 
 
  384
 
  9
 
 9522
 
 tss;island
 
 0.005706
 
 0.0023988
 
 Not queried
 
  1.0796891
 
       NA
 
           NA
  
 
  
 15
 
  73735098
 
  73735694
 
  ENSEMBL   UCSC 
 
  597
 
 12
 
 14289
 
 island
 
 0.483241
 
 0.6062311
 
 Not queried
 
  1.0796164
 
       NA
 
           NA
  
 
  
 19
 
  50979561
 
  50979637
 
  ENSEMBL   UCSC 
 
   77
 
  4
 
 20979
 
 tss
 
 0.002211
 
 0.0059004
 
 Not queried
 
  1.0794545
 
       NA
 
           NA
  
 
  
 2
 
 175199259
 
 175199803
 
  ENSEMBL   UCSC 
 
  545
 
 11
 
 23723
 
 tss
 
 0.138118
 
 0.0396942
 
 Not queried
 
  1.0793334
 
       NA
 
           NA
  
 
  
 17
 
  72363447
 
  72363634
 
  ENSEMBL   UCSC 
 
  188
 
  4
 
 17625
 
 tss
 
 0.271771
 
 0.4302692
 
 Not queried
 
  1.0791271
 
       NA
 
           NA
  
 
  
 1
 
 111746873
 
 111747324
 
  ENSEMBL   UCSC 
 
  452
 
  8
 
 3589
 
 island
 
 0.009420
 
 0.0164767
 
 Not queried
 
  1.0791005
 
       NA
 
           NA
  
 
  
 11
 
  44116911
 
  44117243
 
  ENSEMBL   UCSC 
 
  333
 
  8
 
 8222
 
 island
 
 0.018077
 
 0.0181051
 
 Not queried
 
  1.0788449
 
       NA
 
           NA
  
 
  
 2
 
 177021702
 
 177022285
 
  ENSEMBL   UCSC 
 
  584
 
  7
 
 24910
 
 island
 
 0.053139
 
 0.0576918
 
 Not queried
 
  1.0787196
 
       NA
 
           NA
  
 
  
 4
 
 126237325
 
 126237597
 
  ENSEMBL   UCSC 
 
  273
 
  7
 
 31296
 
 island
 
 0.013744
 
 0.0044063
 
 Not queried
 
  1.0786882
 
       NA
 
           NA
  
 
  
 12
 
  95867190
 
  95867808
 
  ENSEMBL   UCSC 
 
  619
 
 10
 
 9924
 
 tss
 
 0.005730
 
 0.0025949
 
 Not queried
 
  1.0785479
 
       NA
 
           NA
  
 
  
 5
 
  79286648
 
  79287188
 
  ENSEMBL   UCSC 
 
  541
 
  6
 
 32917
 
 island
 
 0.036253
 
 0.0539816
 
 Not queried
 
  1.0783847
 
       NA
 
           NA
  
 
  
 1
 
 167682516
 
 167683013
 
  ENSEMBL   UCSC 
 
  498
 
  6
 
 3948
 
 island
 
 0.024556
 
 0.2199876
 
 Not queried
 
  1.0781608
 
       NA
 
           NA
  
 
  
 1
 
 225996810
 
 225997038
 
  ENSEMBL   UCSC 
 
  229
 
  4
 
 2281
 
 tss;island
 
 0.975464
 
 0.9868637
 
 Not queried
 
  1.0780020
 
       NA
 
           NA
  
 
  
 4
 
   5890289
 
   5890430
 
  ENSEMBL   UCSC 
 
  142
 
  7
 
 29922
 
 gene;island
 
 0.024339
 
 0.0099470
 
 Not queried
 
  1.0779490
 
       NA
 
           NA
  
 
  
 21
 
  15352608
 
  15352983
 
  ENSEMBL   UCSC 
 
  376
 
  7
 
 26606
 
 island
 
 0.169320
 
 0.0596223
 
 Not queried
 
  1.0775671
 
       NA
 
           NA
  
 
  
 11
 
  31530778
 
  31531135
 
  ENSEMBL   UCSC 
 
  358
 
  4
 
 6554
 
 gene
 
 0.009916
 
 0.0094106
 
 Not queried
 
  1.0775384
 
       NA
 
           NA
  
 
  
 17
 
  76850052
 
  76850277
 
  ENSEMBL   UCSC 
 
  226
 
  5
 
 16829
 
 gene;island
 
 0.899055
 
 0.9641638
 
 Not queried
 
  1.0774125
 
       NA
 
           NA
  
 
  
 6
 
  27832728
 
  27833095
 
  ENSEMBL   UCSC 
 
  368
 
  8
 
 34725
 
 tss
 
 0.003277
 
 0.0011437
 
 Not queried
 
  1.0771844
 
       NA
 
           NA
  
 
  
 17
 
  16472670
 
  16472866
 
  ENSEMBL   UCSC 
 
  197
 
  7
 
 17160
 
 tss;island
 
 0.019989
 
 0.0127398
 
 Not queried
 
  1.0769114
 
       NA
 
           NA
  
 
  
 10
 
    372859
 
    373158
 
  ENSEMBL   UCSC 
 
  300
 
  4
 
 4433
 
 gene;island
 
 0.751967
 
 0.9077642
 
 Not queried
 
  1.0767935
 
       NA
 
           NA
  
 
  
 3
 
  49314539
 
  49314920
 
  ENSEMBL   UCSC 
 
  382
 
  9
 
 28325
 
 tss
 
 0.016589
 
 0.0355561
 
 Not queried
 
  1.0767276
 
       NA
 
           NA
  
 
  
 4
 
   1537622
 
   1537799
 
  ENSEMBL   UCSC 
 
  178
 
  4
 
 30790
 
 island
 
 0.559393
 
 0.6496419
 
 Not queried
 
  1.0762651
 
       NA
 
           NA
  
 
  
 4
 
   3769468
 
   3769810
 
  ENSEMBL   UCSC 
 
  343
 
  4
 
 29913
 
 gene;island
 
 0.557849
 
 0.2101158
 
 Not queried
 
  1.0762308
 
       NA
 
           NA
  
 
  
 8
 
 124253374
 
 124253829
 
  ENSEMBL   UCSC 
 
  456
 
  7
 
 40574
 
 island
 
 0.023816
 
 0.0087862
 
 Not queried
 
  1.0761888
 
       NA
 
           NA
  
 
  
 18
 
  18822579
 
  18823249
 
  ENSEMBL   UCSC 
 
  671
 
  7
 
 19066
 
 gene;island
 
 0.412540
 
 0.0347487
 
 Not queried
 
  1.0759513
 
       NA
 
           NA
  
 
  
 1
 
 151254500
 
 151254817
 
  ENSEMBL   UCSC 
 
  318
 
  5
 
 3742
 
 island
 
 0.006284
 
 0.0036852
 
 Not queried
 
  1.0759156
 
       NA
 
           NA
  
 
  
 1
 
  11042161
 
  11042602
 
  ENSEMBL   UCSC 
 
  442
 
  5
 
 1240
 
 tss
 
 0.924168
 
 0.8221565
 
 Not queried
 
  1.0758341
 
       NA
 
           NA
  
 
  
 2
 
  27072271
 
  27072564
 
  ENSEMBL   UCSC 
 
  294
 
  4
 
 22617
 
 gene;island
 
 0.038977
 
 0.0755807
 
 Not queried
 
  1.0757873
 
       NA
 
           NA
  
 
  
 6
 
 154360344
 
 154360894
 
  ENSEMBL   UCSC 
 
  551
 
  7
 
 34519
 
 gene;island
 
 0.406371
 
 0.2453934
 
 Not queried
 
  1.0757604
 
       NA
 
           NA
  
 
  
 12
 
  52281482
 
  52281676
 
  ENSEMBL   UCSC 
 
  195
 
  4
 
 9720
 
 tss
 
 0.906205
 
 0.9244402
 
 Not queried
 
  1.0756331
 
       NA
 
           NA
  
 
  
 7
 
 128049508
 
 128050304
 
  ENSEMBL   UCSC 
 
  797
 
 10
 
 38894
 
 island
 
 0.006169
 
 0.0048118
 
 Not queried
 
  1.0752107
 
       NA
 
           NA
  
 
  
 19
 
  58629828
 
  58630612
 
  ENSEMBL   UCSC 
 
  785
 
  9
 
 21134
 
 tss
 
 0.431216
 
 0.6136834
 
 Not queried
 
  1.0747331
 
       NA
 
           NA
  
 
  
 1
 
  33351568
 
  33351946
 
  ENSEMBL   UCSC 
 
  379
 
  6
 
 1465
 
 tss;island
 
 0.035837
 
 0.0572584
 
 Not queried
 
  1.0747100
 
       NA
 
           NA
  
 
  
 20
 
  31261784
 
  31261994
 
  ENSEMBL   UCSC 
 
  211
 
  4
 
 25675
 
 tss
 
 0.558572
 
 0.4095386
 
 Not queried
 
  1.0745156
 
       NA
 
           NA
  
 
  
 16
 
  19896246
 
  19896644
 
  ENSEMBL   UCSC 
 
  399
 
  4
 
 15120
 
 tss
 
 0.044186
 
 0.0620502
 
 Not queried
 
  1.0744633
 
       NA
 
           NA
  
 
  
 22
 
  31477006
 
  31477247
 
  ENSEMBL   UCSC 
 
  242
 
  8
 
 27073
 
 tss
 
 0.009267
 
 0.0051988
 
 Not queried
 
  1.0742012
 
       NA
 
           NA
  
 
  
 6
 
  31023592
 
  31023770
 
  ENSEMBL   UCSC 
 
  179
 
  4
 
 33848
 
 gene
 
 0.516032
 
 0.7156712
 
 Not queried
 
  1.0741426
 
       NA
 
           NA
  
 
  
 6
 
 166755991
 
 166756218
 
  ENSEMBL   UCSC 
 
  228
 
  5
 
 35336
 
 tss
 
 0.003239
 
 0.0080811
 
 Not queried
 
  1.0740657
 
       NA
 
           NA
  
 
  
 19
 
  52452317
 
  52452528
 
  ENSEMBL   UCSC 
 
  212
 
  4
 
 22345
 
 island
 
 0.063406
 
 0.0772708
 
 Not queried
 
  1.0739578
 
       NA
 
           NA
  
 
  
 9
 
 138966848
 
 138967347
 
  ENSEMBL   UCSC 
 
  500
 
  5
 
 40793
 
 gene;island
 
 0.951712
 
 0.7719029
 
 Not queried
 
  1.0739095
 
       NA
 
           NA
  
 
  
 6
 
  32976805
 
  32976897
 
  ENSEMBL   UCSC 
 
   93
 
  4
 
 34178
 
 gene;island
 
 0.005126
 
 0.0125711
 
 Not queried
 
  1.0736961
 
       NA
 
           NA
  
 
  
 6
 
 110501610
 
 110501899
 
  ENSEMBL   UCSC 
 
  290
 
  5
 
 35168
 
 tss;island
 
 0.002048
 
 0.0034278
 
 Not queried
 
  1.0736782
 
       NA
 
           NA
  
 
  
 19
 
  38747174
 
  38747378
 
  ENSEMBL   UCSC 
 
  205
 
  8
 
 20747
 
 tss
 
 0.039937
 
 0.0509090
 
 Not queried
 
  1.0733859
 
       NA
 
           NA
  
 
  
 12
 
  79198115
 
  79198467
 
  ENSEMBL   UCSC 
 
  353
 
  4
 
 10645
 
 island
 
 0.651627
 
 0.6931284
 
 Not queried
 
  1.0732537
 
       NA
 
           NA
  
 
  
 1
 
  32083737
 
  32083990
 
  ENSEMBL   UCSC 
 
  254
 
  4
 
 382
 
 gene;island
 
 0.043664
 
 0.0214524
 
 Not queried
 
  1.0731132
 
       NA
 
           NA
  
 
  
 6
 
 161694613
 
 161695026
 
  ENSEMBL   UCSC 
 
  414
 
  5
 
 34538
 
 gene
 
 0.017542
 
 0.0095092
 
 Not queried
 
  1.0728936
 
       NA
 
           NA
  
 
  
 11
 
   2444462
 
   2445442
 
  ENSEMBL   UCSC 
 
  981
 
 17
 
 7072
 
 tss;island
 
 0.297342
 
 0.4730066
 
 Not queried
 
  1.0726612
 
       NA
 
           NA
  
 
  
 13
 
  99405102
 
  99405269
 
  ENSEMBL   UCSC 
 
  168
 
  5
 
 11510
 
 tss;island
 
 0.297047
 
 0.6523218
 
 Not queried
 
  1.0725532
 
       NA
 
           NA
  
 
  
 8
 
    893319
 
    893659
 
  ENSEMBL   UCSC 
 
  341
 
  4
 
 40008
 
 island
 
 0.499733
 
 0.7307785
 
 Not queried
 
  1.0723423
 
       NA
 
           NA
  
 
  
 17
 
  47074576
 
  47074713
 
  ENSEMBL   UCSC 
 
  138
 
  7
 
 17480
 
 tss
 
 0.009461
 
 0.0074015
 
 Not queried
 
  1.0723161
 
       NA
 
           NA
  
 
  
 4
 
 153897117
 
 153897705
 
  ENSEMBL   UCSC 
 
  589
 
  6
 
 30151
 
 gene;island
 
 0.506632
 
 0.7437645
 
 Not queried
 
  1.0720671
 
       NA
 
           NA
  
 
  
 2
 
 164592477
 
 164592571
 
  ENSEMBL   UCSC 
 
   95
 
  4
 
 24828
 
 island
 
 0.568648
 
 0.3231923
 
 Not queried
 
  1.0719454
 
       NA
 
           NA
  
 
  
 2
 
 145147129
 
 145147558
 
  ENSEMBL   UCSC 
 
  430
 
  5
 
 22889
 
 gene;island
 
 0.935127
 
 0.9673685
 
 Not queried
 
  1.0717438
 
       NA
 
           NA
  
 
  
 1
 
 211750839
 
 211751127
 
  ENSEMBL   UCSC 
 
  289
 
  4
 
 995
 
 gene;island
 
 0.005131
 
 0.0035314
 
 Not queried
 
  1.0716640
 
       NA
 
           NA
  
 
  
 19
 
  20150159
 
  20150505
 
  ENSEMBL   UCSC 
 
  347
 
  7
 
 20655
 
 tss;island
 
 0.026078
 
 0.0194521
 
 Not queried
 
  1.0715725
 
       NA
 
           NA
  
 
  
 2
 
 190445551
 
 190446181
 
  ENSEMBL   UCSC 
 
  631
 
  6
 
 23779
 
 tss
 
 0.017661
 
 0.0350854
 
 Not queried
 
  1.0712623
 
       NA
 
           NA
  
 
  
 2
 
  44314198
 
  44314662
 
  ENSEMBL   UCSC 
 
  465
 
  5
 
 24254
 
 island
 
 0.005896
 
 0.0322278
 
 Not queried
 
  1.0711555
 
       NA
 
           NA
  
 
  
 1
 
 107682504
 
 107682963
 
  ENSEMBL   UCSC 
 
  460
 
  6
 
 3537
 
 island
 
 0.034514
 
 0.2629468
 
 Not queried
 
  1.0709265
 
       NA
 
           NA
  
 
  
 17
 
  77984086
 
  77984481
 
  ENSEMBL   UCSC 
 
  396
 
  5
 
 16850
 
 gene;island
 
 0.598532
 
 0.7433926
 
 Not queried
 
  1.0708122
 
       NA
 
           NA
  
 
  
 17
 
  46800444
 
  46800680
 
  ENSEMBL   UCSC 
 
  237
 
  4
 
 17470
 
 tss;island
 
 0.071073
 
 0.1447415
 
 Not queried
 
  1.0707548
 
       NA
 
           NA
  
 
  
 7
 
   1809087
 
   1809455
 
  ENSEMBL   UCSC 
 
  369
 
  6
 
 38125
 
 island
 
 0.931139
 
 0.9682262
 
 Not queried
 
  1.0707272
 
       NA
 
           NA
  
 
  
 22
 
  41347240
 
  41347381
 
  ENSEMBL   UCSC 
 
  142
 
  4
 
 27166
 
 tss
 
 0.007803
 
 0.0110295
 
 Not queried
 
  1.0706209
 
       NA
 
           NA
  
 
  
 5
 
  80529067
 
  80529135
 
  ENSEMBL   UCSC 
 
   69
 
  4
 
 32153
 
 tss
 
 0.051040
 
 0.1545022
 
 Not queried
 
  1.0699670
 
       NA
 
           NA
  
 
  
 19
 
  36347276
 
  36347698
 
  ENSEMBL   UCSC 
 
  423
 
  6
 
 20716
 
 tss;island
 
 0.018048
 
 0.0098990
 
 Not queried
 
  1.0697398
 
       NA
 
           NA
  
 
  
 21
 
  19191835
 
  19192138
 
  ENSEMBL   UCSC 
 
  304
 
  6
 
 26493
 
 tss;island
 
 0.006489
 
 0.0050228
 
 Not queried
 
  1.0697280
 
       NA
 
           NA
  
 
  
 7
 
  24796981
 
  24797486
 
  ENSEMBL   UCSC 
 
  506
 
  5
 
 36922
 
 gene
 
 0.006453
 
 0.0230265
 
 Not queried
 
  1.0697010
 
       NA
 
           NA
  
 
  
 16
 
  49311698
 
  49312033
 
  ENSEMBL   UCSC 
 
  336
 
  6
 
 15906
 
 island
 
 0.011272
 
 0.0433411
 
 Not queried
 
  1.0694648
 
       NA
 
           NA
  
 
  
 7
 
 100493365
 
 100493849
 
  ENSEMBL   UCSC 
 
  485
 
  7
 
 38785
 
 island
 
 0.005432
 
 0.0241032
 
 Not queried
 
  1.0694222
 
       NA
 
           NA
  
 
  
 11
 
  32421514
 
  32421845
 
  ENSEMBL   UCSC 
 
  332
 
  6
 
 6564
 
 gene
 
 0.738164
 
 0.8734407
 
 Not queried
 
  1.0693092
 
       NA
 
           NA
  
 
  
 10
 
 118956639
 
 118956998
 
  ENSEMBL   UCSC 
 
  360
 
  7
 
 5390
 
 tss
 
 0.409693
 
 0.4210742
 
 Not queried
 
  1.0692952
 
       NA
 
           NA
  
 
  
 12
 
   4923404
 
   4923529
 
  ENSEMBL   UCSC 
 
  126
 
  4
 
 9018
 
 gene;island
 
 0.542919
 
 0.7123701
 
 Not queried
 
  1.0690216
 
       NA
 
           NA
  
 
  
 8
 
  76319672
 
  76320150
 
  ENSEMBL   UCSC 
 
  479
 
  5
 
 40401
 
 island
 
 0.006867
 
 0.0014970
 
 Not queried
 
  1.0686931
 
       NA
 
           NA
  
 
  
 11
 
  63887426
 
  63887634
 
  ENSEMBL   UCSC 
 
  209
 
  4
 
 6665
 
 gene;island
 
 0.747437
 
 0.9300840
 
 Not queried
 
  1.0686489
 
       NA
 
           NA
  
 
  
 11
 
  33758121
 
  33758529
 
  ENSEMBL   UCSC 
 
  409
 
  7
 
 7235
 
 tss
 
 0.024719
 
 0.1677021
 
 Not queried
 
  1.0686362
 
       NA
 
           NA
  
 
  
 7
 
 134855396
 
 134855774
 
  ENSEMBL   UCSC 
 
  379
 
 11
 
 38938
 
 island
 
 0.001720
 
 0.0067688
 
 Not queried
 
  1.0685171
 
       NA
 
           NA
  
 
  
 8
 
  22014425
 
  22014724
 
  ENSEMBL   UCSC 
 
  300
 
  8
 
 39582
 
 tss;island
 
 0.047336
 
 0.0973352
 
 Not queried
 
  1.0683283
 
       NA
 
           NA
  
 
  
 11
 
   2597528
 
   2597838
 
  ENSEMBL   UCSC 
 
  311
 
  4
 
 6465
 
 gene;island
 
 0.600154
 
 0.6804612
 
 Not queried
 
  1.0683237
 
       NA
 
           NA
  
 
  
 17
 
  73775612
 
  73776120
 
  ENSEMBL   UCSC 
 
  509
 
 12
 
 18762
 
 island
 
 0.003674
 
 0.0025611
 
 Not queried
 
  1.0682695
 
       NA
 
           NA
  
 
  
 10
 
  12648032
 
  12648526
 
  ENSEMBL   UCSC 
 
  495
 
  4
 
 4500
 
 gene
 
 0.024530
 
 0.0467398
 
 Not queried
 
  1.0682131
 
       NA
 
           NA
  
 
  
 12
 
  56727304
 
  56727806
 
  ENSEMBL   UCSC 
 
  503
 
  9
 
 9179
 
 gene
 
 0.008425
 
 0.0044654
 
 Not queried
 
  1.0682078
 
       NA
 
           NA
  
 
  
 7
 
 132261287
 
 132261418
 
  ENSEMBL   UCSC 
 
  132
 
  4
 
 37230
 
 gene;island
 
 0.076011
 
 0.1128305
 
 Not queried
 
  1.0681816
 
       NA
 
           NA
  
 
  
 19
 
   2578938
 
   2579285
 
  ENSEMBL   UCSC 
 
  348
 
  4
 
 19694
 
 gene;island
 
 0.101878
 
 0.2448458
 
 Not queried
 
  1.0681489
 
       NA
 
           NA
  
 
  
 4
 
  17616339
 
  17616649
 
  ENSEMBL   UCSC 
 
  311
 
  4
 
 29962
 
 gene
 
 0.003253
 
 0.0058060
 
 Not queried
 
  1.0678370
 
       NA
 
           NA
  
 
  
 16
 
    717419
 
    717707
 
  ENSEMBL   UCSC 
 
  289
 
  4
 
 14536
 
 gene;tss;island
 
 0.900759
 
 0.7157511
 
 Not queried
 
  1.0678330
 
       NA
 
           NA
  
 
  
 1
 
 171640102
 
 171640387
 
  ENSEMBL   UCSC 
 
  286
 
  4
 
 3963
 
 island
 
 0.773093
 
 0.7228579
 
 Not queried
 
  1.0678069
 
       NA
 
           NA
  
 
  
 5
 
 131347439
 
 131347773
 
  ENSEMBL   UCSC 
 
  335
 
  5
 
 32266
 
 tss
 
 0.038400
 
 0.0213025
 
 Not queried
 
  1.0674398
 
       NA
 
           NA
  
 
  
 12
 
 111807000
 
 111807189
 
  ENSEMBL   UCSC 
 
  190
 
  8
 
 10014
 
 tss;island
 
 0.005616
 
 0.0013834
 
 Not queried
 
  1.0673375
 
       NA
 
           NA
  
 
  
 7
 
  27195918
 
  27196555
 
  ENSEMBL   UCSC 
 
  638
 
 11
 
 38353
 
 island
 
 0.034783
 
 0.0218363
 
 Not queried
 
  1.0669458
 
       NA
 
           NA
  
 
  
 17
 
  48858534
 
  48858873
 
  ENSEMBL   UCSC 
 
  340
 
  5
 
 18561
 
 island
 
 0.547916
 
 0.5708047
 
 Not queried
 
  1.0668539
 
       NA
 
           NA
  
 
  
 11
 
  75046147
 
  75046354
 
  ENSEMBL   UCSC 
 
  208
 
  4
 
 6832
 
 gene
 
 0.828748
 
 0.9023848
 
 Not queried
 
  1.0666316
 
       NA
 
           NA
  
 
  
 5
 
 140734543
 
 140735027
 
  ENSEMBL   UCSC 
 
  485
 
  6
 
 31820
 
 gene;island
 
 0.267442
 
 0.4082000
 
 Not queried
 
  1.0663825
 
       NA
 
           NA
  
 
  
 2
 
  42795925
 
  42796178
 
  ENSEMBL   UCSC 
 
  254
 
  4
 
 22656
 
 gene;island
 
 0.005435
 
 0.0124372
 
 Not queried
 
  1.0661318
 
       NA
 
           NA
  
 
  
 10
 
  54073047
 
  54073642
 
  ENSEMBL   UCSC 
 
  596
 
  8
 
 5086
 
 tss;island
 
 0.022251
 
 0.0129658
 
 Not queried
 
  1.0655490
 
       NA
 
           NA
  
 
  
 2
 
  47797415
 
  47797590
 
  ENSEMBL   UCSC 
 
  176
 
  4
 
 24283
 
 island
 
 0.034701
 
 0.0962156
 
 Not queried
 
  1.0651526
 
       NA
 
           NA
  
 
  
 22
 
  31364274
 
  31364356
 
  ENSEMBL   UCSC 
 
   83
 
  5
 
 27071
 
 tss;island
 
 0.977442
 
 0.9881267
 
 Not queried
 
  1.0648387
 
       NA
 
           NA
  
 
  
 10
 
    574291
 
    574732
 
  ENSEMBL   UCSC 
 
  442
 
  6
 
 4442
 
 gene;island
 
 0.509729
 
 0.6656880
 
 Not queried
 
  1.0647819
 
       NA
 
           NA
  
 
  
 5
 
 172661803
 
 172662463
 
  ENSEMBL   UCSC 
 
  661
 
  8
 
 33407
 
 island
 
 0.019488
 
 0.0057586
 
 Not queried
 
  1.0643884
 
       NA
 
           NA
  
 
  
 5
 
 139944046
 
 139944509
 
  ENSEMBL   UCSC 
 
  464
 
 10
 
 32343
 
 tss;island
 
 0.007406
 
 0.0051958
 
 Not queried
 
  1.0641773
 
       NA
 
           NA
  
 
  
 19
 
  18721531
 
  18721840
 
  ENSEMBL   UCSC 
 
  310
 
  4
 
 21788
 
 island
 
 0.044313
 
 0.0293326
 
 Not queried
 
  1.0641633
 
       NA
 
           NA
  
 
  
 16
 
  30934656
 
  30934727
 
  ENSEMBL   UCSC 
 
   72
 
  5
 
 15198
 
 tss;island
 
 0.004070
 
 0.0025882
 
 Not queried
 
  1.0638854
 
       NA
 
           NA
  
 
  
 6
 
  25652531
 
  25652815
 
  ENSEMBL   UCSC 
 
  285
 
  4
 
 33595
 
 gene
 
 0.116551
 
 0.0687270
 
 Not queried
 
  1.0638781
 
       NA
 
           NA
  
 
  
 3
 
 126062402
 
 126062822
 
  ENSEMBL   UCSC 
 
  421
 
  4
 
 27929
 
 gene;island
 
 0.602234
 
 0.8386548
 
 Not queried
 
  1.0638460
 
       NA
 
           NA
  
 
  
 18
 
  24130267
 
  24130633
 
  ENSEMBL   UCSC 
 
  367
 
  4
 
 19081
 
 gene;tss;island
 
 0.065709
 
 0.0633284
 
 Not queried
 
  1.0635780
 
       NA
 
           NA
  
 
  
 1
 
  11986612
 
  11986842
 
  ENSEMBL   UCSC 
 
  231
 
  5
 
 1253
 
 tss;island
 
 0.024281
 
 0.0103731
 
 Not queried
 
  1.0635211
 
       NA
 
           NA
  
 
  
 5
 
  73937299
 
  73937330
 
  ENSEMBL   UCSC 
 
   32
 
  4
 
 32124
 
 tss
 
 0.001163
 
 0.0034540
 
 Not queried
 
  1.0633186
 
       NA
 
           NA
  
 
  
 7
 
  11871837
 
  11872073
 
  ENSEMBL   UCSC 
 
  237
 
  7
 
 37442
 
 tss
 
 0.011108
 
 0.1816952
 
 Not queried
 
  1.0630005
 
       NA
 
           NA
  
 
  
 5
 
 127871927
 
 127872329
 
  ENSEMBL   UCSC 
 
  403
 
  5
 
 31738
 
 gene;island
 
 0.700876
 
 0.8165602
 
 Not queried
 
  1.0629419
 
       NA
 
           NA
  
 
  
 10
 
 132912784
 
 132913255
 
  ENSEMBL   UCSC 
 
  472
 
  5
 
 4842
 
 gene
 
 0.438235
 
 0.6043911
 
 Not queried
 
  1.0628966
 
       NA
 
           NA
  
 
  
 19
 
  40596853
 
  40597063
 
  ENSEMBL   UCSC 
 
  211
 
  5
 
 20781
 
 tss
 
 0.006136
 
 0.0021042
 
 Not queried
 
  1.0628384
 
       NA
 
           NA
  
 
  
 13
 
 102104573
 
 102104848
 
  ENSEMBL   UCSC 
 
  276
 
  4
 
 11520
 
 tss
 
 0.744575
 
 0.7988021
 
 Not queried
 
  1.0626838
 
       NA
 
           NA
  
 
  
 6
 
  32022898
 
  32023650
 
  ENSEMBL   UCSC 
 
  753
 
 13
 
 34020
 
 gene
 
 0.514553
 
 0.6880687
 
 Not queried
 
  1.0621898
 
       NA
 
           NA
  
 
  
 11
 
  69462660
 
  69463323
 
  ENSEMBL   UCSC 
 
  664
 
  6
 
 6781
 
 gene;island
 
 0.455245
 
 0.5179664
 
 Not queried
 
  1.0618437
 
       NA
 
           NA
  
 
  
 20
 
  33680648
 
  33681223
 
  ENSEMBL   UCSC 
 
  576
 
 11
 
 25700
 
 tss
 
 0.020412
 
 0.0098161
 
 Not queried
 
  1.0613990
 
       NA
 
           NA
  
 
  
 20
 
  33680135
 
  33681223
 
  ENSEMBL   UCSC 
 
 1089
 
 15
 
 26118
 
 island
 
 0.020412
 
 0.0098161
 
 Not queried
 
  1.0613990
 
       NA
 
           NA
  
 
  
 4
 
   7436693
 
   7437156
 
  ENSEMBL   UCSC 
 
  464
 
  6
 
 29937
 
 gene
 
 0.062389
 
 0.2156669
 
 Not queried
 
  1.0613000
 
       NA
 
           NA
  
 
  
 11
 
   1029029
 
   1029336
 
  ENSEMBL   UCSC 
 
  308
 
  5
 
 6400
 
 gene;island
 
 0.421758
 
 0.7394216
 
 Not queried
 
  1.0612943
 
       NA
 
           NA
  
 
  
 5
 
  41510325
 
  41510851
 
  ENSEMBL   UCSC 
 
  527
 
  7
 
 32754
 
 island
 
 0.060435
 
 0.0712006
 
 Not queried
 
  1.0607877
 
       NA
 
           NA
  
 
  
 2
 
  87035502
 
  87035758
 
  ENSEMBL   UCSC 
 
  257
 
  8
 
 24497
 
 island
 
 0.013455
 
 0.0041174
 
 Not queried
 
  1.0605215
 
       NA
 
           NA
  
 
  
 6
 
 166419479
 
 166419943
 
  ENSEMBL   UCSC 
 
  465
 
  6
 
 36625
 
 island
 
 0.018760
 
 0.0213337
 
 Not queried
 
  1.0602591
 
       NA
 
           NA
  
 
  
 14
 
 100111183
 
 100111392
 
  ENSEMBL   UCSC 
 
  210
 
  8
 
 12599
 
 tss;island
 
 0.054780
 
 0.1516434
 
 Not queried
 
  1.0601193
 
       NA
 
           NA
  
 
  
 8
 
  56987020
 
  56987302
 
  ENSEMBL   UCSC 
 
  283
 
  8
 
 39712
 
 tss;island
 
 0.004140
 
 0.0027771
 
 Not queried
 
  1.0601045
 
       NA
 
           NA
  
 
  
 2
 
 207629829
 
 207630025
 
  ENSEMBL   UCSC 
 
  197
 
  6
 
 22996
 
 gene
 
 0.007813
 
 0.0036352
 
 Not queried
 
  1.0600575
 
       NA
 
           NA
  
 
  
 11
 
  20620680
 
  20621109
 
  ENSEMBL   UCSC 
 
  430
 
  7
 
 8130
 
 island
 
 0.495904
 
 0.2260961
 
 Not queried
 
  1.0599035
 
       NA
 
           NA
  
 
  
 10
 
  64578739
 
  64579140
 
  ENSEMBL   UCSC 
 
  402
 
  5
 
 5777
 
 island
 
 0.065058
 
 0.0159935
 
 Not queried
 
  1.0597289
 
       NA
 
           NA
  
 
  
 2
 
  75426726
 
  75427699
 
  ENSEMBL   UCSC 
 
  974
 
 12
 
 23468
 
 tss;island
 
 0.061838
 
 0.0128254
 
 Not queried
 
  1.0596431
 
       NA
 
           NA
  
 
  
 7
 
 137028165
 
 137028535
 
  ENSEMBL   UCSC 
 
  371
 
  6
 
 37237
 
 gene
 
 0.007471
 
 0.0062747
 
 Not queried
 
  1.0595151
 
       NA
 
           NA
  
 
  
 7
 
 157697057
 
 157697628
 
  ENSEMBL   UCSC 
 
  572
 
  5
 
 37353
 
 gene;island
 
 0.251501
 
 0.4118255
 
 Not queried
 
  1.0594124
 
       NA
 
           NA
  
 
  
 10
 
   8095121
 
   8097354
 
  ENSEMBL   UCSC 
 
 2234
 
 48
 
 5540
 
 island
 
 0.008151
 
 0.0205710
 
 Not queried
 
  1.0592766
 
       NA
 
           NA
  
 
  
 4
 
  41880280
 
  41880747
 
  ENSEMBL   UCSC 
 
  468
 
  6
 
 31008
 
 island
 
 0.490480
 
 0.4576961
 
 Not queried
 
  1.0591481
 
       NA
 
           NA
  
 
  
 2
 
 132152167
 
 132152543
 
  ENSEMBL   UCSC 
 
  377
 
  4
 
 24730
 
 island
 
 0.127198
 
 0.3432577
 
 Not queried
 
  1.0591158
 
       NA
 
           NA
  
 
  
 11
 
  10589810
 
  10590282
 
  ENSEMBL   UCSC 
 
  473
 
  4
 
 6508
 
 gene
 
 0.308461
 
 0.4413559
 
 Not queried
 
  1.0591064
 
       NA
 
           NA
  
 
  
 12
 
 123636480
 
 123636717
 
  ENSEMBL   UCSC 
 
  238
 
  4
 
 10944
 
 island
 
 0.925120
 
 0.9821350
 
 Not queried
 
  1.0590670
 
       NA
 
           NA
  
 
  
 19
 
  48947560
 
  48947631
 
  ENSEMBL   UCSC 
 
   72
 
  4
 
 20124
 
 gene;tss;island
 
 0.360153
 
 0.2031045
 
 Not queried
 
  1.0588062
 
       NA
 
           NA
  
 
  
 12
 
   3601880
 
   3602291
 
  ENSEMBL   UCSC 
 
  412
 
  6
 
 9011
 
 gene;island
 
 0.012816
 
 0.0075382
 
 Not queried
 
  1.0586944
 
       NA
 
           NA
  
 
  
 10
 
 102090297
 
 102090589
 
  ENSEMBL   UCSC 
 
  293
 
  5
 
 5291
 
 tss
 
 0.645646
 
 0.5036541
 
 Not queried
 
  1.0586206
 
       NA
 
           NA
  
 
  
 16
 
  85074868
 
  85075249
 
  ENSEMBL   UCSC 
 
  382
 
  5
 
 14874
 
 gene
 
 0.695936
 
 0.7618649
 
 Not queried
 
  1.0585387
 
       NA
 
           NA
  
 
  
 6
 
   2765499
 
   2765594
 
  ENSEMBL   UCSC 
 
   96
 
  5
 
 34591
 
 tss;island
 
 0.010402
 
 0.0058417
 
 Not queried
 
  1.0584237
 
       NA
 
           NA
  
 
  
 7
 
  94538165
 
  94538408
 
  ENSEMBL   UCSC 
 
  244
 
  7
 
 37095
 
 gene;tss;island
 
 0.060268
 
 0.0784102
 
 Not queried
 
  1.0584093
 
       NA
 
           NA
  
 
  
 13
 
  78493012
 
  78494462
 
  ENSEMBL   UCSC 
 
 1451
 
 42
 
 11487
 
 tss
 
 0.047576
 
 0.1211797
 
 Not queried
 
  1.0582448
 
       NA
 
           NA
  
 
  
 12
 
 113574066
 
 113574368
 
  ENSEMBL   UCSC 
 
  303
 
  6
 
 10025
 
 tss
 
 0.130251
 
 0.0301927
 
 Not queried
 
  1.0577536
 
       NA
 
           NA
  
 
  
 8
 
 124657935
 
 124658143
 
  ENSEMBL   UCSC 
 
  209
 
  4
 
 39420
 
 gene
 
 0.544648
 
 0.7143464
 
 Not queried
 
  1.0577322
 
       NA
 
           NA
  
 
  
 1
 
  78511140
 
  78512129
 
  ENSEMBL   UCSC 
 
  990
 
 12
 
 3402
 
 island
 
 0.036383
 
 0.2754704
 
 Not queried
 
  1.0571789
 
       NA
 
           NA
  
 
  
 15
 
  50646099
 
  50646617
 
  ENSEMBL   UCSC 
 
  519
 
  6
 
 13344
 
 gene;island
 
 0.013392
 
 0.0145648
 
 Not queried
 
  1.0569116
 
       NA
 
           NA
  
 
  
 15
 
  50646099
 
  50646350
 
  ENSEMBL   UCSC 
 
  252
 
  4
 
 13666
 
 tss
 
 0.013392
 
 0.0203719
 
 Not queried
 
  1.0569116
 
       NA
 
           NA
  
 
  
 1
 
 146543992
 
 146544267
 
  ENSEMBL   UCSC 
 
  276
 
  4
 
 3690
 
 island
 
 0.701440
 
 0.6132986
 
 Not queried
 
  1.0566803
 
       NA
 
           NA
  
 
  
 6
 
  27342376
 
  27342738
 
  ENSEMBL   UCSC 
 
  363
 
  5
 
 33619
 
 gene;island
 
 0.007304
 
 0.0109302
 
 Not queried
 
  1.0566568
 
       NA
 
           NA
  
 
  
 7
 
 100086099
 
 100086445
 
  ENSEMBL   UCSC 
 
  347
 
  4
 
 37141
 
 gene;island
 
 0.673939
 
 0.8601004
 
 Not queried
 
  1.0566393
 
       NA
 
           NA
  
 
  
 15
 
  29499226
 
  29499526
 
  ENSEMBL   UCSC 
 
  301
 
  5
 
 13270
 
 gene
 
 0.572772
 
 0.8946704
 
 Not queried
 
  1.0566383
 
       NA
 
           NA
  
 
  
 6
 
  30851417
 
  30852092
 
  ENSEMBL   UCSC 
 
  676
 
 12
 
 34791
 
 tss;island
 
 0.021310
 
 0.0722154
 
 Not queried
 
  1.0563578
 
       NA
 
           NA
  
 
  
 12
 
  59314172
 
  59314530
 
  ENSEMBL   UCSC 
 
  359
 
  7
 
 9831
 
 tss;island
 
 0.023803
 
 0.0485282
 
 Not queried
 
  1.0560782
 
       NA
 
           NA
  
 
  
 8
 
  41507621
 
  41507937
 
  ENSEMBL   UCSC 
 
  317
 
  5
 
 40249
 
 island
 
 0.960885
 
 0.9816489
 
 Not queried
 
  1.0560628
 
       NA
 
           NA
  
 
  
 7
 
  27197455
 
  27197713
 
  ENSEMBL   UCSC 
 
  259
 
  4
 
 37495
 
 tss
 
 0.004514
 
 0.0060926
 
 Not queried
 
  1.0559133
 
       NA
 
           NA
  
 
  
 11
 
   2170870
 
   2171184
 
  ENSEMBL   UCSC 
 
  315
 
  5
 
 7059
 
 tss
 
 0.004030
 
 0.0115081
 
 Not queried
 
  1.0558131
 
       NA
 
           NA
  
 
  
 5
 
   1087095
 
   1087341
 
  ENSEMBL   UCSC 
 
  247
 
  4
 
 31521
 
 gene;island
 
 0.973349
 
 0.9351250
 
 Not queried
 
  1.0555729
 
       NA
 
           NA
  
 
  
 12
 
 132859441
 
 132859951
 
  ENSEMBL   UCSC 
 
  511
 
  5
 
 9442
 
 gene;island
 
 0.176113
 
 0.4735193
 
 Not queried
 
  1.0555386
 
       NA
 
           NA
  
 
  
 5
 
 150461039
 
 150461211
 
  ENSEMBL   UCSC 
 
  173
 
  6
 
 32437
 
 tss
 
 0.004021
 
 0.0022271
 
 Not queried
 
  1.0555356
 
       NA
 
           NA
  
 
  
 3
 
  45883249
 
  45883859
 
  ENSEMBL   UCSC 
 
  611
 
 12
 
 29052
 
 island
 
 0.009966
 
 0.0151978
 
 Not queried
 
  1.0554259
 
       NA
 
           NA
  
 
  
 1
 
 244080781
 
 244081189
 
  ENSEMBL   UCSC 
 
  409
 
  6
 
 4390
 
 island
 
 0.028675
 
 0.0318999
 
 Not queried
 
  1.0554018
 
       NA
 
           NA
  
 
  
 19
 
  14142047
 
  14142353
 
  ENSEMBL   UCSC 
 
  307
 
  4
 
 21667
 
 island
 
 0.746062
 
 0.5665270
 
 Not queried
 
  1.0553618
 
       NA
 
           NA
  
 
  
 1
 
  63785867
 
  63786306
 
  ENSEMBL   UCSC 
 
  440
 
  5
 
 3341
 
 island
 
 0.228608
 
 0.3004279
 
 Not queried
 
  1.0553411
 
       NA
 
           NA
  
 
  
 4
 
  77227489
 
  77227879
 
  ENSEMBL   UCSC 
 
  391
 
  8
 
 31116
 
 island
 
 0.017469
 
 0.0068118
 
 Not queried
 
  1.0552699
 
       NA
 
           NA
  
 
  
 14
 
  61119122
 
  61119419
 
  ENSEMBL   UCSC 
 
  298
 
  4
 
 12909
 
 island
 
 0.035382
 
 0.0288809
 
 Not queried
 
  1.0552219
 
       NA
 
           NA
  
 
  
 1
 
  52082575
 
  52082719
 
  ENSEMBL   UCSC 
 
  145
 
  6
 
 1624
 
 tss
 
 0.027610
 
 0.0153945
 
 Not queried
 
  1.0547792
 
       NA
 
           NA
  
 
  
 6
 
 168417574
 
 168417743
 
  ENSEMBL   UCSC 
 
  170
 
  4
 
 35345
 
 tss
 
 0.953049
 
 0.9761688
 
 Not queried
 
  1.0546965
 
       NA
 
           NA
  
 
  
 5
 
 155754009
 
 155754265
 
  ENSEMBL   UCSC 
 
  257
 
  4
 
 31871
 
 gene
 
 0.466480
 
 0.7899453
 
 Not queried
 
  1.0546755
 
       NA
 
           NA
  
 
  
 16
 
  22825621
 
  22825843
 
  ENSEMBL   UCSC 
 
  223
 
  4
 
 15134
 
 tss;island
 
 0.168723
 
 0.1874783
 
 Not queried
 
  1.0546454
 
       NA
 
           NA
  
 
  
 2
 
 209271661
 
 209272057
 
  ENSEMBL   UCSC 
 
  397
 
  5
 
 23000
 
 gene
 
 0.048409
 
 0.0981072
 
 Not queried
 
  1.0545299
 
       NA
 
           NA
  
 
  
 16
 
  14013678
 
  14014103
 
  ENSEMBL   UCSC 
 
  426
 
  7
 
 15718
 
 island
 
 0.009101
 
 0.0063702
 
 Not queried
 
  1.0544241
 
       NA
 
           NA
  
 
  
 2
 
  24299374
 
  24299646
 
  ENSEMBL   UCSC 
 
  273
 
  6
 
 23223
 
 tss
 
 0.011817
 
 0.0199703
 
 Not queried
 
  1.0543684
 
       NA
 
           NA
  
 
  
 1
 
 182354699
 
 182354972
 
  ENSEMBL   UCSC 
 
  274
 
  4
 
 910
 
 gene
 
 0.880961
 
 0.8421114
 
 Not queried
 
  1.0543074
 
       NA
 
           NA
  
 
  
 19
 
   9473565
 
   9473691
 
  ENSEMBL   UCSC 
 
  127
 
  6
 
 20423
 
 tss
 
 0.046169
 
 0.0542582
 
 Not queried
 
  1.0541580
 
       NA
 
           NA
  
 
  
 10
 
   1444456
 
   1444808
 
  ENSEMBL   UCSC 
 
  353
 
  6
 
 4463
 
 gene
 
 0.770517
 
 0.8461598
 
 Not queried
 
  1.0540963
 
       NA
 
           NA
  
 
  
 5
 
   2755820
 
   2756113
 
  ENSEMBL   UCSC 
 
  294
 
  4
 
 32659
 
 island
 
 0.018078
 
 0.0234366
 
 Not queried
 
  1.0539731
 
       NA
 
           NA
  
 
  
 3
 
  69101575
 
  69101684
 
  ENSEMBL   UCSC 
 
  110
 
  5
 
 28439
 
 tss
 
 0.006248
 
 0.0031418
 
 Not queried
 
  1.0539408
 
       NA
 
           NA
  
 
  
 7
 
     82270
 
     82623
 
  ENSEMBL   UCSC 
 
  354
 
  5
 
 38027
 
 island
 
 0.196567
 
 0.6279334
 
 Not queried
 
  1.0538400
 
       NA
 
           NA
  
 
  
 19
 
   9846267
 
   9846701
 
  ENSEMBL   UCSC 
 
  435
 
  4
 
 21504
 
 island
 
 0.754580
 
 0.8732121
 
 Not queried
 
  1.0536519
 
       NA
 
           NA
  
 
  
 4
 
  81951951
 
  81952099
 
  ENSEMBL   UCSC 
 
  149
 
  6
 
 30463
 
 tss;island
 
 0.007935
 
 0.0057390
 
 Not queried
 
  1.0536178
 
       NA
 
           NA
  
 
  
 1
 
 240160972
 
 240161447
 
  ENSEMBL   UCSC 
 
  476
 
  5
 
 4372
 
 island
 
 0.058060
 
 0.0963773
 
 Not queried
 
  1.0534904
 
       NA
 
           NA
  
 
  
 3
 
  13860477
 
  13860812
 
  ENSEMBL   UCSC 
 
  336
 
  4
 
 27684
 
 gene;island
 
 0.443324
 
 0.6533608
 
 Not queried
 
  1.0528293
 
       NA
 
           NA
  
 
  
 1
 
 161129255
 
 161129745
 
  ENSEMBL   UCSC 
 
  491
 
  5
 
 851
 
 gene
 
 0.004621
 
 0.0037402
 
 Not queried
 
  1.0525701
 
       NA
 
           NA
  
 
  
 16
 
  53407421
 
  53407808
 
  ENSEMBL   UCSC 
 
  388
 
  7
 
 15924
 
 island
 
 0.862369
 
 0.8852917
 
 Not queried
 
  1.0523291
 
       NA
 
           NA
  
 
  
 1
 
  50513451
 
  50513661
 
  ENSEMBL   UCSC 
 
  211
 
  4
 
 1614
 
 tss
 
 0.211652
 
 0.0925981
 
 Not queried
 
  1.0519359
 
       NA
 
           NA
  
 
  
 1
 
 214161251
 
 214161371
 
  ENSEMBL   UCSC 
 
  121
 
  4
 
 2250
 
 tss;island
 
 0.042853
 
 0.0666798
 
 Not queried
 
  1.0516526
 
       NA
 
           NA
  
 
  
 5
 
    493262
 
    493893
 
  ENSEMBL   UCSC 
 
  632
 
  6
 
 31507
 
 gene;island
 
 0.860100
 
 0.8087815
 
 Not queried
 
  1.0513911
 
       NA
 
           NA
  
 
  
 3
 
  57262354
 
  57262569
 
  ENSEMBL   UCSC 
 
  216
 
  4
 
 27842
 
 gene;island
 
 0.005858
 
 0.0015772
 
 Not queried
 
  1.0512002
 
       NA
 
           NA
  
 
  
 1
 
   2410885
 
   2411643
 
  ENSEMBL   UCSC 
 
  759
 
  9
 
 85
 
 gene;island
 
 0.482044
 
 0.6256868
 
 Not queried
 
  1.0509605
 
       NA
 
           NA
  
 
  
 19
 
  46650855
 
  46651227
 
  ENSEMBL   UCSC 
 
  373
 
  5
 
 20890
 
 tss
 
 0.494897
 
 0.6646657
 
 Not queried
 
  1.0508007
 
       NA
 
           NA
  
 
  
 19
 
   6495655
 
   6495957
 
  ENSEMBL   UCSC 
 
  303
 
  7
 
 20392
 
 tss
 
 0.671762
 
 0.8811883
 
 Not queried
 
  1.0507472
 
       NA
 
           NA
  
 
  
 11
 
  66725820
 
  66725961
 
  ENSEMBL   UCSC 
 
  142
 
  6
 
 8530
 
 island
 
 0.049304
 
 0.0192893
 
 Not queried
 
  1.0506234
 
       NA
 
           NA
  
 
  
 11
 
   9112989
 
   9113458
 
  ENSEMBL   UCSC 
 
  470
 
 11
 
 8040
 
 island
 
 0.015942
 
 0.0213143
 
 Not queried
 
  1.0505519
 
       NA
 
           NA
  
 
  
 5
 
 114515626
 
 114515936
 
  ENSEMBL   UCSC 
 
  311
 
  4
 
 31718
 
 gene;island
 
 0.011624
 
 0.0409161
 
 Not queried
 
  1.0502170
 
       NA
 
           NA
  
 
  
 11
 
  18034626
 
  18034920
 
  ENSEMBL   UCSC 
 
  295
 
  7
 
 8105
 
 island
 
 0.001560
 
 0.0069572
 
 Not queried
 
  1.0500225
 
       NA
 
           NA
  
 
  
 7
 
  93519855
 
  93520566
 
  ENSEMBL   UCSC 
 
  712
 
 19
 
 38669
 
 island
 
 0.031686
 
 0.0150092
 
 Not queried
 
  1.0500176
 
       NA
 
           NA
  
 
  
 3
 
  45883621
 
  45883859
 
  ENSEMBL   UCSC 
 
  239
 
  9
 
 28272
 
 tss
 
 0.007583
 
 0.0111924
 
 Not queried
 
  1.0496627
 
       NA
 
           NA
  
 
  
 12
 
 113587240
 
 113587581
 
  ENSEMBL   UCSC 
 
  342
 
  5
 
 10026
 
 tss
 
 0.043763
 
 0.2677995
 
 Not queried
 
  1.0495937
 
       NA
 
           NA
  
 
  
 4
 
 153859106
 
 153859560
 
  ENSEMBL   UCSC 
 
  455
 
  5
 
 31368
 
 island
 
 0.041853
 
 0.0202211
 
 Not queried
 
  1.0494453
 
       NA
 
           NA
  
 
  
 4
 
 175750109
 
 175751001
 
  ENSEMBL   UCSC 
 
  893
 
 13
 
 31438
 
 island
 
 0.043041
 
 0.0126823
 
 Not queried
 
  1.0493501
 
       NA
 
           NA
  
 
  
 21
 
  45526957
 
  45527029
 
  ENSEMBL   UCSC 
 
   73
 
  4
 
 26580
 
 tss;island
 
 0.015204
 
 0.0084091
 
 Not queried
 
  1.0492979
 
       NA
 
           NA
  
 
  
 12
 
   7023195
 
   7023352
 
  ENSEMBL   UCSC 
 
  158
 
  4
 
 9039
 
 gene
 
 0.625826
 
 0.4494442
 
 Not queried
 
  1.0490845
 
       NA
 
           NA
  
 
  
 6
 
  39693228
 
  39693479
 
  ENSEMBL   UCSC 
 
  252
 
  6
 
 34957
 
 tss
 
 0.021133
 
 0.0140875
 
 Not queried
 
  1.0485172
 
       NA
 
           NA
  
 
  
 7
 
 108095719
 
 108096041
 
  ENSEMBL   UCSC 
 
  323
 
  5
 
 37185
 
 gene;island
 
 0.021358
 
 0.0112364
 
 Not queried
 
  1.0484463
 
       NA
 
           NA
  
 
  
 1
 
  25869837
 
  25870007
 
  ENSEMBL   UCSC 
 
  171
 
  6
 
 1371
 
 tss
 
 0.028077
 
 0.0737287
 
 Not queried
 
  1.0482020
 
       NA
 
           NA
  
 
  
 17
 
   7227499
 
   7227737
 
  ENSEMBL   UCSC 
 
  239
 
  4
 
 16391
 
 gene;island
 
 0.033204
 
 0.1732931
 
 Not queried
 
  1.0480524
 
       NA
 
           NA
  
 
  
 20
 
  60540388
 
  60540747
 
  ENSEMBL   UCSC 
 
  360
 
  4
 
 26309
 
 island
 
 0.352952
 
 0.2596413
 
 Not queried
 
  1.0480345
 
       NA
 
           NA
  
 
  
 17
 
  58212864
 
  58213165
 
  ENSEMBL   UCSC 
 
  302
 
  5
 
 18617
 
 island
 
 0.009066
 
 0.0231773
 
 Not queried
 
  1.0479718
 
       NA
 
           NA
  
 
  
 2
 
 136743411
 
 136743698
 
  ENSEMBL   UCSC 
 
  288
 
  5
 
 23648
 
 tss;island
 
 0.009344
 
 0.0156917
 
 Not queried
 
  1.0479169
 
       NA
 
           NA
  
 
  
 17
 
  77020041
 
  77020267
 
  ENSEMBL   UCSC 
 
  227
 
  5
 
 18838
 
 island
 
 0.010205
 
 0.0564768
 
 Not queried
 
  1.0477840
 
       NA
 
           NA
  
 
  
 2
 
 219773416
 
 219774183
 
  ENSEMBL   UCSC 
 
  768
 
  8
 
 25095
 
 island
 
 0.025620
 
 0.0553061
 
 Not queried
 
  1.0476808
 
       NA
 
           NA
  
 
  
 19
 
  49520359
 
  49520532
 
  ENSEMBL   UCSC 
 
  174
 
  4
 
 20936
 
 tss;island
 
 0.695179
 
 0.9091723
 
 Not queried
 
  1.0476423
 
       NA
 
           NA
  
 
  
 16
 
  82045140
 
  82045222
 
  ENSEMBL   UCSC 
 
   83
 
  4
 
 15383
 
 tss
 
 0.039297
 
 0.0179120
 
 Not queried
 
  1.0475265
 
       NA
 
           NA
  
 
  
 13
 
  27131484
 
  27131780
 
  ENSEMBL   UCSC 
 
  297
 
  9
 
 11355
 
 tss;island
 
 0.057398
 
 0.1144014
 
 Not queried
 
  1.0473196
 
       NA
 
           NA
  
 
  
 1
 
  53905709
 
  53905934
 
  ENSEMBL   UCSC 
 
  226
 
  5
 
 1638
 
 tss
 
 0.832493
 
 0.8335846
 
 Not queried
 
  1.0472355
 
       NA
 
           NA
  
 
  
 11
 
 117103610
 
 117103880
 
  ENSEMBL   UCSC 
 
  271
 
  4
 
 6917
 
 gene;tss;island
 
 0.003867
 
 0.0058137
 
 Not queried
 
  1.0470876
 
       NA
 
           NA
  
 
  
 1
 
 120395857
 
 120396167
 
  ENSEMBL   UCSC 
 
  311
 
  4
 
 3665
 
 island
 
 0.704941
 
 0.8320119
 
 Not queried
 
  1.0470272
 
       NA
 
           NA
  
 
  
 8
 
 146012669
 
 146013229
 
  ENSEMBL   UCSC 
 
  561
 
  8
 
 40748
 
 island
 
 0.004127
 
 0.0084721
 
 Not queried
 
  1.0469686
 
       NA
 
           NA
  
 
  
 2
 
    289147
 
    289731
 
  ENSEMBL   UCSC 
 
  585
 
  6
 
 23154
 
 tss;island
 
 0.004730
 
 0.0115000
 
 Not queried
 
  1.0468701
 
       NA
 
           NA
  
 
  
 4
 
  93224895
 
  93225466
 
  ENSEMBL   UCSC 
 
  572
 
  7
 
 30499
 
 tss
 
 0.005427
 
 0.0162459
 
 Not queried
 
  1.0467163
 
       NA
 
           NA
  
 
  
 11
 
  76777529
 
  76777881
 
  ENSEMBL   UCSC 
 
  353
 
  7
 
 7581
 
 tss
 
 0.013661
 
 0.0092293
 
 Not queried
 
  1.0465273
 
       NA
 
           NA
  
 
  
 3
 
 185655949
 
 185656289
 
  ENSEMBL   UCSC 
 
  341
 
  4
 
 28769
 
 tss
 
 0.168280
 
 0.2355235
 
 Not queried
 
  1.0464395
 
       NA
 
           NA
  
 
  
 8
 
  75262469
 
  75262759
 
  ENSEMBL   UCSC 
 
  291
 
  7
 
 40399
 
 island
 
 0.015262
 
 0.0133157
 
 Not queried
 
  1.0463011
 
       NA
 
           NA
  
 
  
 10
 
  93392870
 
  93393509
 
  ENSEMBL   UCSC 
 
  640
 
 10
 
 5223
 
 tss
 
 0.075204
 
 0.0173851
 
 Not queried
 
  1.0461017
 
       NA
 
           NA
  
 
  
 10
 
  93392644
 
  93393509
 
  ENSEMBL   UCSC 
 
  866
 
 14
 
 5932
 
 island
 
 0.065127
 
 0.0173851
 
 Not queried
 
  1.0461017
 
       NA
 
           NA
  
 
  
 3
 
 126237289
 
 126237462
 
  ENSEMBL   UCSC 
 
  174
 
  4
 
 28560
 
 tss
 
 0.482086
 
 0.7029277
 
 Not queried
 
  1.0460986
 
       NA
 
           NA
  
 
  
 11
 
  64597090
 
  64597513
 
  ENSEMBL   UCSC 
 
  424
 
  4
 
 6697
 
 gene;island
 
 0.789525
 
 0.8631160
 
 Not queried
 
  1.0460961
 
       NA
 
           NA
  
 
  
 17
 
  77020041
 
  77020243
 
  ENSEMBL   UCSC 
 
  203
 
  4
 
 17711
 
 tss
 
 0.009419
 
 0.0362971
 
 Not queried
 
  1.0460488
 
       NA
 
           NA
  
 
  
 16
 
  31382930
 
  31383136
 
  ENSEMBL   UCSC 
 
  207
 
  4
 
 14736
 
 gene;island
 
 0.456906
 
 0.6114098
 
 Not queried
 
  1.0458277
 
       NA
 
           NA
  
 
  
 10
 
  83636072
 
  83636325
 
  ENSEMBL   UCSC 
 
  254
 
  4
 
 4656
 
 gene;tss;island
 
 0.030198
 
 0.0149703
 
 Not queried
 
  1.0456995
 
       NA
 
           NA
  
 
  
 1
 
   2014244
 
   2014628
 
  ENSEMBL   UCSC 
 
  385
 
  4
 
 58
 
 gene
 
 0.558397
 
 0.7413131
 
 Not queried
 
  1.0456712
 
       NA
 
           NA
  
 
  
 11
 
   1219189
 
   1219644
 
  ENSEMBL   UCSC 
 
  456
 
  5
 
 7905
 
 island
 
 0.300197
 
 0.6802573
 
 Not queried
 
  1.0456709
 
       NA
 
           NA
  
 
  
 10
 
 134853084
 
 134853344
 
  ENSEMBL   UCSC 
 
  261
 
  4
 
 6328
 
 island
 
 0.388112
 
 0.5343201
 
 Not queried
 
  1.0456137
 
       NA
 
           NA
  
 
  
 12
 
  69632957
 
  69633319
 
  ENSEMBL   UCSC 
 
  363
 
  7
 
 10613
 
 island
 
 0.015611
 
 0.0061497
 
 Not queried
 
  1.0450660
 
       NA
 
           NA
  
 
  
 19
 
  58220295
 
  58220516
 
  ENSEMBL   UCSC 
 
  222
 
  4
 
 20249
 
 gene
 
 0.016360
 
 0.0498090
 
 Not queried
 
  1.0450602
 
       NA
 
           NA
  
 
  
 17
 
  42992917
 
  42993050
 
  ENSEMBL   UCSC 
 
  134
 
  4
 
 17419
 
 tss
 
 0.511628
 
 0.6645147
 
 Not queried
 
  1.0448250
 
       NA
 
           NA
  
 
  
 6
 
 163149167
 
 163149453
 
  ENSEMBL   UCSC 
 
  287
 
  4
 
 34540
 
 gene;tss;island
 
 0.301933
 
 0.3903995
 
 Not queried
 
  1.0447889
 
       NA
 
           NA
  
 
  
 7
 
  29603121
 
  29603541
 
  ENSEMBL   UCSC 
 
  421
 
  8
 
 38376
 
 island
 
 0.033704
 
 0.0174034
 
 Not queried
 
  1.0446816
 
       NA
 
           NA
  
 
  
 16
 
  67450370
 
  67450810
 
  ENSEMBL   UCSC 
 
  441
 
  6
 
 15302
 
 tss;island
 
 0.044758
 
 0.0175897
 
 Not queried
 
  1.0446524
 
       NA
 
           NA
  
 
  
 6
 
  30433749
 
  30434552
 
  ENSEMBL   UCSC 
 
  804
 
 21
 
 35683
 
 island
 
 0.038209
 
 0.0178406
 
 Not queried
 
  1.0446138
 
       NA
 
           NA
  
 
  
 2
 
 216973806
 
 216973988
 
  ENSEMBL   UCSC 
 
  183
 
  5
 
 23866
 
 tss
 
 0.003151
 
 0.0052075
 
 Not queried
 
  1.0446058
 
       NA
 
           NA
  
 
  
 16
 
    734504
 
    734844
 
  ENSEMBL   UCSC 
 
  341
 
  7
 
 14976
 
 tss
 
 0.039826
 
 0.0345776
 
 Not queried
 
  1.0445610
 
       NA
 
           NA
  
 
  
 3
 
  27410970
 
  27411065
 
  ENSEMBL   UCSC 
 
   96
 
  4
 
 28195
 
 tss
 
 0.004956
 
 0.0020084
 
 Not queried
 
  1.0444683
 
       NA
 
           NA
  
 
  
 12
 
  96336486
 
  96336545
 
  ENSEMBL   UCSC 
 
   60
 
  4
 
 9928
 
 tss;island
 
 0.020543
 
 0.0089350
 
 Not queried
 
  1.0442408
 
       NA
 
           NA
  
 
  
 1
 
   4471975
 
   4472087
 
  ENSEMBL   UCSC 
 
  113
 
  4
 
 1194
 
 tss
 
 0.812017
 
 0.8737671
 
 Not queried
 
  1.0442344
 
       NA
 
           NA
  
 
  
 18
 
  23713407
 
  23713741
 
  ENSEMBL   UCSC 
 
  335
 
  6
 
 19215
 
 tss
 
 0.736218
 
 0.5843219
 
 Not queried
 
  1.0439689
 
       NA
 
           NA
  
 
  
 2
 
  18765848
 
  18766294
 
  ENSEMBL   UCSC 
 
  447
 
  5
 
 22585
 
 gene;island
 
 0.744546
 
 0.9233584
 
 Not queried
 
  1.0439046
 
       NA
 
           NA
  
 
  
 12
 
 124155451
 
 124155880
 
  ENSEMBL   UCSC 
 
  430
 
  7
 
 10956
 
 island
 
 0.007931
 
 0.0187573
 
 Not queried
 
  1.0438115
 
       NA
 
           NA
  
 
  
 2
 
 216301341
 
 216301766
 
  ENSEMBL   UCSC 
 
  426
 
  5
 
 23863
 
 tss;island
 
 0.007860
 
 0.0133533
 
 Not queried
 
  1.0430317
 
       NA
 
           NA
  
 
  
 19
 
  56915403
 
  56915879
 
  ENSEMBL   UCSC 
 
  477
 
  9
 
 20237
 
 gene;island
 
 0.037873
 
 0.0472288
 
 Not queried
 
  1.0426578
 
       NA
 
           NA
  
 
  
 17
 
    780838
 
    781075
 
  ENSEMBL   UCSC 
 
  238
 
  5
 
 16299
 
 gene
 
 0.826673
 
 0.9069854
 
 Not queried
 
  1.0425326
 
       NA
 
           NA
  
 
  
 19
 
  53794492
 
  53794975
 
  ENSEMBL   UCSC 
 
  484
 
  6
 
 22372
 
 island
 
 0.569220
 
 0.8310426
 
 Not queried
 
  1.0421824
 
       NA
 
           NA
  
 
  
 2
 
 135809940
 
 135810355
 
  ENSEMBL   UCSC 
 
  416
 
  4
 
 22881
 
 gene
 
 0.001773
 
 0.0082579
 
 Not queried
 
  1.0421674
 
       NA
 
           NA
  
 
  
 14
 
  51562486
 
  51562646
 
  ENSEMBL   UCSC 
 
  161
 
  5
 
 12391
 
 tss
 
 0.040874
 
 0.0268856
 
 Not queried
 
  1.0421662
 
       NA
 
           NA
  
 
  
 5
 
  14665478
 
  14665592
 
  ENSEMBL   UCSC 
 
  115
 
  4
 
 31565
 
 gene;island
 
 0.010276
 
 0.0108092
 
 Not queried
 
  1.0420758
 
       NA
 
           NA
  
 
  
 20
 
   9494838
 
   9495596
 
  ENSEMBL   UCSC 
 
  759
 
 10
 
 25996
 
 island
 
 0.029419
 
 0.0159709
 
 Not queried
 
  1.0419672
 
       NA
 
           NA
  
 
  
 19
 
   4759746
 
   4760016
 
  ENSEMBL   UCSC 
 
  271
 
  4
 
 21387
 
 island
 
 0.009363
 
 0.0340818
 
 Not queried
 
  1.0416751
 
       NA
 
           NA
  
 
  
 2
 
  33953195
 
  33953437
 
  ENSEMBL   UCSC 
 
  243
 
  4
 
 24216
 
 island
 
 0.438666
 
 0.5875405
 
 Not queried
 
  1.0415797
 
       NA
 
           NA
  
 
  
 5
 
 176237221
 
 176237381
 
  ENSEMBL   UCSC 
 
  161
 
  4
 
 32518
 
 tss;island
 
 0.008247
 
 0.0061704
 
 Not queried
 
  1.0415685
 
       NA
 
           NA
  
 
  
 8
 
  30892276
 
  30892490
 
  ENSEMBL   UCSC 
 
  215
 
  4
 
 39273
 
 gene;tss
 
 0.367441
 
 0.1317384
 
 Not queried
 
  1.0412303
 
       NA
 
           NA
  
 
  
 14
 
 104387812
 
 104388185
 
  ENSEMBL   UCSC 
 
  374
 
  5
 
 13198
 
 island
 
 0.006761
 
 0.0060914
 
 Not queried
 
  1.0411582
 
       NA
 
           NA
  
 
  
 6
 
 100911526
 
 100911746
 
  ENSEMBL   UCSC 
 
  221
 
  8
 
 36365
 
 island
 
 0.140058
 
 0.2579408
 
 Not queried
 
  1.0410886
 
       NA
 
           NA
  
 
  
 4
 
  17615782
 
  17616265
 
  ENSEMBL   UCSC 
 
  484
 
 10
 
 30317
 
 tss
 
 0.007209
 
 0.0029615
 
 Not queried
 
  1.0409057
 
       NA
 
           NA
  
 
  
 19
 
  56028425
 
  56028883
 
  ENSEMBL   UCSC 
 
  459
 
  5
 
 20232
 
 gene;island
 
 0.028353
 
 0.0140190
 
 Not queried
 
  1.0407876
 
       NA
 
           NA
  
 
  
 4
 
  77135005
 
  77135506
 
  ENSEMBL   UCSC 
 
  502
 
  8
 
 31114
 
 island
 
 0.010751
 
 0.0041040
 
 Not queried
 
  1.0406463
 
       NA
 
           NA
  
 
  
 13
 
 113652069
 
 113652368
 
  ENSEMBL   UCSC 
 
  300
 
  5
 
 11266
 
 gene;island
 
 0.092397
 
 0.3284932
 
 Not queried
 
  1.0403860
 
       NA
 
           NA
  
 
  
 22
 
  27068716
 
  27068761
 
  ENSEMBL   UCSC 
 
   46
 
  4
 
 26827
 
 gene
 
 0.000562
 
 0.0006537
 
 Not queried
 
  1.0403077
 
       NA
 
           NA
  
 
  
 20
 
   4982196
 
   4982389
 
  ENSEMBL   UCSC 
 
  194
 
  5
 
 25351
 
 gene;tss
 
 0.009997
 
 0.0097994
 
 Not queried
 
  1.0401474
 
       NA
 
           NA
  
 
  
 22
 
  25003400
 
  25003552
 
  ENSEMBL   UCSC 
 
  153
 
  4
 
 27025
 
 tss
 
 0.197547
 
 0.4692643
 
 Not queried
 
  1.0398723
 
       NA
 
           NA
  
 
  
 15
 
  63569245
 
  63569834
 
  ENSEMBL   UCSC 
 
  590
 
 11
 
 14205
 
 island
 
 0.003992
 
 0.0144330
 
 Not queried
 
  1.0398569
 
       NA
 
           NA
  
 
  
 17
 
   9808600
 
   9808824
 
  ENSEMBL   UCSC 
 
  225
 
  4
 
 18019
 
 island
 
 0.569620
 
 0.6795053
 
 Not queried
 
  1.0397472
 
       NA
 
           NA
  
 
  
 4
 
  20701575
 
  20702377
 
  ENSEMBL   UCSC 
 
  803
 
 12
 
 30954
 
 island
 
 0.008178
 
 0.0044318
 
 Not queried
 
  1.0397435
 
       NA
 
           NA
  
 
  
 2
 
 132056834
 
 132057166
 
  ENSEMBL   UCSC 
 
  333
 
  4
 
 22870
 
 gene;island
 
 0.465067
 
 0.5799207
 
 Not queried
 
  1.0395411
 
       NA
 
           NA
  
 
  
 12
 
 132822250
 
 132822693
 
  ENSEMBL   UCSC 
 
  444
 
  4
 
 9436
 
 gene;island
 
 0.800629
 
 0.8671024
 
 Not queried
 
  1.0394407
 
       NA
 
           NA
  
 
  
 11
 
   2596300
 
   2596606
 
  ENSEMBL   UCSC 
 
  307
 
  4
 
 6464
 
 gene;island
 
 0.307998
 
 0.4845214
 
 Not queried
 
  1.0393084
 
       NA
 
           NA
  
 
  
 1
 
  66999588
 
  66999813
 
  ENSEMBL   UCSC 
 
  226
 
  4
 
 1679
 
 tss
 
 0.032051
 
 0.0806536
 
 Not queried
 
  1.0392048
 
       NA
 
           NA
  
 
  
 17
 
  17740344
 
  17740468
 
  ENSEMBL   UCSC 
 
  125
 
  5
 
 17169
 
 tss
 
 0.007751
 
 0.0042053
 
 Not queried
 
  1.0391437
 
       NA
 
           NA
  
 
  
 10
 
  28032282
 
  28032855
 
  ENSEMBL   UCSC 
 
  574
 
  7
 
 4543
 
 gene;island
 
 0.053247
 
 0.1048574
 
 Not queried
 
  1.0390301
 
       NA
 
           NA
  
 
  
 17
 
  37764220
 
  37764293
 
  ENSEMBL   UCSC 
 
   74
 
  4
 
 17310
 
 tss
 
 0.017003
 
 0.0058775
 
 Not queried
 
  1.0387666
 
       NA
 
           NA
  
 
  
 17
 
  74878301
 
  74878420
 
  ENSEMBL   UCSC 
 
  120
 
  5
 
 16799
 
 gene;island
 
 0.608690
 
 0.7509544
 
 Not queried
 
  1.0386981
 
       NA
 
           NA
  
 
  
 6
 
  32629786
 
  32629955
 
  ENSEMBL   UCSC 
 
  170
 
  5
 
 34124
 
 gene;island
 
 0.791197
 
 0.4808590
 
 Not queried
 
  1.0385469
 
       NA
 
           NA
  
 
  
 15
 
  45315202
 
  45315438
 
  ENSEMBL   UCSC 
 
  237
 
  8
 
 14103
 
 island
 
 0.004885
 
 0.0030882
 
 Not queried
 
  1.0385309
 
       NA
 
           NA
  
 
  
 1
 
  16302232
 
  16302590
 
  ENSEMBL   UCSC 
 
  359
 
  6
 
 257
 
 gene
 
 0.006177
 
 0.0090617
 
 Not queried
 
  1.0383510
 
       NA
 
           NA
  
 
  
 6
 
  32116207
 
  32116317
 
  ENSEMBL   UCSC 
 
  111
 
  4
 
 34056
 
 gene
 
 0.026855
 
 0.0500828
 
 Not queried
 
  1.0382481
 
       NA
 
           NA
  
 
  
 11
 
 110300429
 
 110300632
 
  ENSEMBL   UCSC 
 
  204
 
  5
 
 8792
 
 island
 
 0.017667
 
 0.0085039
 
 Not queried
 
  1.0381999
 
       NA
 
           NA
  
 
  
 11
 
   2016513
 
   2017084
 
  ENSEMBL   UCSC 
 
  572
 
  7
 
 6442
 
 gene;island
 
 0.637183
 
 0.7514472
 
 Not queried
 
  1.0380969
 
       NA
 
           NA
  
 
  
 4
 
   7802192
 
   7802545
 
  ENSEMBL   UCSC 
 
  354
 
  5
 
 29942
 
 gene;island
 
 0.397986
 
 0.4832701
 
 Not queried
 
  1.0377813
 
       NA
 
           NA
  
 
  
 6
 
  73972704
 
  73972852
 
  ENSEMBL   UCSC 
 
  149
 
  4
 
 34386
 
 gene
 
 0.015162
 
 0.0076405
 
 Not queried
 
  1.0376610
 
       NA
 
           NA
  
 
  
 17
 
  74928552
 
  74928863
 
  ENSEMBL   UCSC 
 
  312
 
  6
 
 16800
 
 gene;island
 
 0.682491
 
 0.7853678
 
 Not queried
 
  1.0376510
 
       NA
 
           NA
  
 
  
 7
 
  42533001
 
  42533414
 
  ENSEMBL   UCSC 
 
  414
 
  5
 
 38437
 
 island
 
 0.011236
 
 0.0613213
 
 Not queried
 
  1.0375820
 
       NA
 
           NA
  
 
  
 16
 
  56701994
 
  56702158
 
  ENSEMBL   UCSC 
 
  165
 
  5
 
 15251
 
 tss
 
 0.030113
 
 0.0636765
 
 Not queried
 
  1.0373913
 
       NA
 
           NA
  
 
  
 16
 
  56701868
 
  56702158
 
  ENSEMBL   UCSC 
 
  291
 
  7
 
 15954
 
 island
 
 0.047519
 
 0.1048972
 
 Not queried
 
  1.0373913
 
       NA
 
           NA
  
 
  
 2
 
  63285365
 
  63286049
 
  ENSEMBL   UCSC 
 
  685
 
  8
 
 24342
 
 island
 
 0.026107
 
 0.0889852
 
 Not queried
 
  1.0373792
 
       NA
 
           NA
  
 
  
 12
 
  93963179
 
  93963603
 
  ENSEMBL   UCSC 
 
  425
 
 12
 
 10675
 
 island
 
 0.003485
 
 0.0014721
 
 Not queried
 
  1.0372558
 
       NA
 
           NA
  
 
  
 8
 
 144798535
 
 144799022
 
  ENSEMBL   UCSC 
 
  488
 
  6
 
 39489
 
 gene
 
 0.369278
 
 0.5258117
 
 Not queried
 
  1.0370989
 
       NA
 
           NA
  
 
  
 12
 
 130953464
 
 130953814
 
  ENSEMBL   UCSC 
 
  351
 
  8
 
 9416
 
 gene;island
 
 0.941247
 
 0.9607835
 
 Not queried
 
  1.0370199
 
       NA
 
           NA
  
 
  
 14
 
 101491377
 
 101492406
 
  ENSEMBL   UCSC 
 
 1030
 
 11
 
 12621
 
 tss
 
 0.419723
 
 0.6361791
 
 Not queried
 
  1.0368848
 
       NA
 
           NA
  
 
  
 2
 
 133428422
 
 133429299
 
  ENSEMBL   UCSC 
 
  878
 
 10
 
 24746
 
 island
 
 0.169101
 
 0.4647029
 
 Not queried
 
  1.0365839
 
       NA
 
           NA
  
 
  
 1
 
   2084246
 
   2084595
 
  ENSEMBL   UCSC 
 
  350
 
  5
 
 65
 
 gene;island
 
 0.745191
 
 0.8607335
 
 Not queried
 
  1.0365385
 
       NA
 
           NA
  
 
  
 11
 
  60524073
 
  60524323
 
  ENSEMBL   UCSC 
 
  251
 
  4
 
 7321
 
 tss
 
 0.672314
 
 0.7278614
 
 Not queried
 
  1.0363128
 
       NA
 
           NA
  
 
  
 16
 
  30645674
 
  30646006
 
  ENSEMBL   UCSC 
 
  333
 
  4
 
 15841
 
 island
 
 0.018877
 
 0.0134149
 
 Not queried
 
  1.0362803
 
       NA
 
           NA
  
 
  
 4
 
  39368001
 
  39368212
 
  ENSEMBL   UCSC 
 
  212
 
  6
 
 30349
 
 tss
 
 0.020817
 
 0.0077092
 
 Not queried
 
  1.0361565
 
       NA
 
           NA
  
 
  
 12
 
  93835939
 
  93836653
 
  ENSEMBL   UCSC 
 
  715
 
 11
 
 10673
 
 island
 
 0.003913
 
 0.0034309
 
 Not queried
 
  1.0358159
 
       NA
 
           NA
  
 
  
 8
 
  57358130
 
  57358713
 
  ENSEMBL   UCSC 
 
  584
 
  9
 
 39314
 
 gene;island
 
 0.071842
 
 0.1032060
 
 Not queried
 
  1.0356926
 
       NA
 
           NA
  
 
  
 3
 
   9404422
 
   9404837
 
  ENSEMBL   UCSC 
 
  416
 
  9
 
 28855
 
 island
 
 0.010478
 
 0.0042301
 
 Not queried
 
  1.0356550
 
       NA
 
           NA
  
 
  
 20
 
  53091600
 
  53092474
 
  ENSEMBL   UCSC 
 
  875
 
  8
 
 26267
 
 island
 
 0.077554
 
 0.0226287
 
 Not queried
 
  1.0355023
 
       NA
 
           NA
  
 
  
 21
 
  27543229
 
  27543721
 
  ENSEMBL   UCSC 
 
  493
 
  5
 
 26499
 
 tss
 
 0.011813
 
 0.0165007
 
 Not queried
 
  1.0354373
 
       NA
 
           NA
  
 
  
 21
 
  27543045
 
  27543721
 
  ENSEMBL   UCSC 
 
  677
 
  7
 
 26620
 
 island
 
 0.010019
 
 0.0109953
 
 Not queried
 
  1.0354373
 
       NA
 
           NA
  
 
  
 9
 
 131965232
 
 131965635
 
  ENSEMBL   UCSC 
 
  404
 
  4
 
 40957
 
 island
 
 0.063822
 
 0.0257515
 
 Not queried
 
  1.0354003
 
       NA
 
           NA
  
 
  
 1
 
 102462806
 
 102462877
 
  ENSEMBL   UCSC 
 
   72
 
  4
 
 1784
 
 tss
 
 0.794124
 
 0.8955987
 
 Not queried
 
  1.0352684
 
       NA
 
           NA
  
 
  
 12
 
   2921865
 
   2922039
 
  ENSEMBL   UCSC 
 
  175
 
  4
 
 9008
 
 gene
 
 0.000880
 
 0.0001285
 
 Not queried
 
  1.0351519
 
       NA
 
           NA
  
 
  
 14
 
  91283255
 
  91283606
 
  ENSEMBL   UCSC 
 
  352
 
  4
 
 12556
 
 tss;island
 
 0.147478
 
 0.3465369
 
 Not queried
 
  1.0350145
 
       NA
 
           NA
  
 
  
 3
 
 138067350
 
 138067623
 
  ENSEMBL   UCSC 
 
  274
 
  6
 
 27963
 
 gene;island
 
 0.011188
 
 0.0034535
 
 Not queried
 
  1.0349472
 
       NA
 
           NA
  
 
  
 2
 
 236866987
 
 236867278
 
  ENSEMBL   UCSC 
 
  292
 
  4
 
 23086
 
 gene;island
 
 0.216065
 
 0.4733520
 
 Not queried
 
  1.0345685
 
       NA
 
           NA
  
 
  
 7
 
 138602901
 
 138603662
 
  ENSEMBL   UCSC 
 
  762
 
  7
 
 37242
 
 gene
 
 0.901210
 
 0.9712788
 
 Not queried
 
  1.0344961
 
       NA
 
           NA
  
 
  
 13
 
 100547728
 
 100548078
 
  ENSEMBL   UCSC 
 
  351
 
  5
 
 11208
 
 gene;island
 
 0.125554
 
 0.4196517
 
 Not queried
 
  1.0343912
 
       NA
 
           NA
  
 
  
 17
 
  43922122
 
  43922423
 
  ENSEMBL   UCSC 
 
  302
 
  6
 
 16638
 
 gene
 
 0.373682
 
 0.6110195
 
 Not queried
 
  1.0343814
 
       NA
 
           NA
  
 
  
 2
 
  31456964
 
  31457061
 
  ENSEMBL   UCSC 
 
   98
 
  5
 
 22638
 
 gene
 
 0.004335
 
 0.0088480
 
 Not queried
 
  1.0341915
 
       NA
 
           NA
  
 
  
 12
 
 130935796
 
 130936355
 
  ENSEMBL   UCSC 
 
  560
 
  7
 
 9415
 
 gene;island
 
 0.705435
 
 0.8351939
 
 Not queried
 
  1.0341617
 
       NA
 
           NA
  
 
  
 11
 
   2554198
 
   2554583
 
  ENSEMBL   UCSC 
 
  386
 
  6
 
 6462
 
 gene;island
 
 0.012752
 
 0.0346527
 
 Not queried
 
  1.0341446
 
       NA
 
           NA
  
 
  
 13
 
  31191332
 
  31191824
 
  ENSEMBL   UCSC 
 
  493
 
  8
 
 11375
 
 tss;island
 
 0.002048
 
 0.0021711
 
 Not queried
 
  1.0341338
 
       NA
 
           NA
  
 
  
 4
 
 100009950
 
 100010258
 
  ENSEMBL   UCSC 
 
  309
 
  9
 
 30511
 
 tss
 
 0.004146
 
 0.0046579
 
 Not queried
 
  1.0339100
 
       NA
 
           NA
  
 
  
 15
 
  89943283
 
  89943836
 
  ENSEMBL   UCSC 
 
  554
 
  5
 
 14432
 
 island
 
 0.012820
 
 0.0297084
 
 Not queried
 
  1.0338297
 
       NA
 
           NA
  
 
  
 8
 
  97173335
 
  97173722
 
  ENSEMBL   UCSC 
 
  388
 
  4
 
 39824
 
 tss;island
 
 0.063166
 
 0.0174788
 
 Not queried
 
  1.0337131
 
       NA
 
           NA
  
 
  
 21
 
  38071673
 
  38071911
 
  ENSEMBL   UCSC 
 
  239
 
  4
 
 26539
 
 tss
 
 0.169995
 
 0.0872778
 
 Not queried
 
  1.0334648
 
       NA
 
           NA
  
 
  
 1
 
  55445834
 
  55446708
 
  ENSEMBL   UCSC 
 
  875
 
 10
 
 3304
 
 island
 
 0.067800
 
 0.3275040
 
 Not queried
 
  1.0333762
 
       NA
 
           NA
  
 
  
 11
 
  87908631
 
  87908938
 
  ENSEMBL   UCSC 
 
  308
 
  8
 
 7612
 
 tss
 
 0.016257
 
 0.0458416
 
 Not queried
 
  1.0333048
 
       NA
 
           NA
  
 
  
 22
 
  26908119
 
  26908376
 
  ENSEMBL   UCSC 
 
  258
 
  4
 
 26825
 
 gene
 
 0.004866
 
 0.0019175
 
 Not queried
 
  1.0332750
 
       NA
 
           NA
  
 
  
 7
 
 102984880
 
 102985587
 
  ENSEMBL   UCSC 
 
  708
 
  8
 
 38810
 
 island
 
 0.044550
 
 0.0232806
 
 Not queried
 
  1.0331288
 
       NA
 
           NA
  
 
  
 14
 
  24809297
 
  24809759
 
  ENSEMBL   UCSC 
 
  463
 
  4
 
 12329
 
 tss
 
 0.010189
 
 0.0066784
 
 Not queried
 
  1.0331079
 
       NA
 
           NA
  
 
  
 11
 
  33279899
 
  33280348
 
  ENSEMBL   UCSC 
 
  450
 
  4
 
 6575
 
 gene;island
 
 0.019087
 
 0.0131217
 
 Not queried
 
  1.0329772
 
       NA
 
           NA
  
 
  
 19
 
  58446312
 
  58446988
 
  ENSEMBL   UCSC 
 
  677
 
 12
 
 22487
 
 island
 
 0.069505
 
 0.0305862
 
 Not queried
 
  1.0328868
 
       NA
 
           NA
  
 
  
 19
 
  37407257
 
  37407486
 
  ENSEMBL   UCSC 
 
  230
 
  6
 
 20006
 
 gene
 
 0.015248
 
 0.0081197
 
 Not queried
 
  1.0327220
 
       NA
 
           NA
  
 
  
 3
 
  53290118
 
  53290343
 
  ENSEMBL   UCSC 
 
  226
 
  8
 
 28398
 
 tss
 
 0.064286
 
 0.1448679
 
 Not queried
 
  1.0319707
 
       NA
 
           NA
  
 
  
 8
 
  15397729
 
  15398333
 
  ENSEMBL   UCSC 
 
  605
 
  7
 
 39213
 
 gene
 
 0.041148
 
 0.0206345
 
 Not queried
 
  1.0316424
 
       NA
 
           NA
  
 
  
 17
 
  48649219
 
  48649370
 
  ENSEMBL   UCSC 
 
  152
 
  4
 
 16690
 
 gene;island
 
 0.377347
 
 0.6071781
 
 Not queried
 
  1.0315599
 
       NA
 
           NA
  
 
  
 13
 
  19919156
 
  19919540
 
  ENSEMBL   UCSC 
 
  385
 
  6
 
 11323
 
 tss;island
 
 0.365406
 
 0.2744577
 
 Not queried
 
  1.0314668
 
       NA
 
           NA
  
 
  
 5
 
 137774431
 
 137774650
 
  ENSEMBL   UCSC 
 
  220
 
  7
 
 32321
 
 tss
 
 0.013392
 
 0.0800318
 
 Not queried
 
  1.0314280
 
       NA
 
           NA
  
 
  
 18
 
  19752292
 
  19752414
 
  ENSEMBL   UCSC 
 
  123
 
  4
 
 19068
 
 gene;island
 
 0.575706
 
 0.8599757
 
 Not queried
 
  1.0310994
 
       NA
 
           NA
  
 
  
 11
 
    695461
 
    695585
 
  ENSEMBL   UCSC 
 
  125
 
  5
 
 6387
 
 gene;tss;island
 
 0.002120
 
 0.0081225
 
 Not queried
 
  1.0309335
 
       NA
 
           NA
  
 
  
 3
 
  12882711
 
  12883055
 
  ENSEMBL   UCSC 
 
  345
 
  4
 
 27676
 
 gene
 
 0.011159
 
 0.0065332
 
 Not queried
 
  1.0308821
 
       NA
 
           NA
  
 
  
 2
 
  97405364
 
  97405809
 
  ENSEMBL   UCSC 
 
  446
 
  5
 
 22785
 
 gene
 
 0.009468
 
 0.0015376
 
 Not queried
 
  1.0308687
 
       NA
 
           NA
  
 
  
 11
 
  34535357
 
  34535579
 
  ENSEMBL   UCSC 
 
  223
 
  7
 
 7242
 
 tss
 
 0.906623
 
 0.9586930
 
 Not queried
 
  1.0306225
 
       NA
 
           NA
  
 
  
 6
 
  32013974
 
  32015389
 
  ENSEMBL   UCSC 
 
 1416
 
 25
 
 34861
 
 tss
 
 0.387564
 
 0.5429513
 
 Not queried
 
  1.0305844
 
       NA
 
           NA
  
 
  
 2
 
 240650018
 
 240650458
 
  ENSEMBL   UCSC 
 
  441
 
  5
 
 25262
 
 island
 
 0.473498
 
 0.6474975
 
 Not queried
 
  1.0303939
 
       NA
 
           NA
  
 
  
 10
 
 123356205
 
 123356336
 
  ENSEMBL   UCSC 
 
  132
 
  4
 
 5405
 
 tss
 
 0.144536
 
 0.0528508
 
 Not queried
 
  1.0302990
 
       NA
 
           NA
  
 
  
 6
 
  30074078
 
  30074219
 
  ENSEMBL   UCSC 
 
  142
 
  5
 
 33721
 
 gene;island
 
 0.422022
 
 0.5985632
 
 Not queried
 
  1.0302372
 
       NA
 
           NA
  
 
  
 7
 
  94953956
 
  94954202
 
  ENSEMBL   UCSC 
 
  247
 
  4
 
 37725
 
 tss
 
 0.393778
 
 0.7053131
 
 Not queried
 
  1.0300109
 
       NA
 
           NA
  
 
  
 19
 
   4867785
 
   4868079
 
  ENSEMBL   UCSC 
 
  295
 
  8
 
 20374
 
 tss
 
 0.005821
 
 0.0063154
 
 Not queried
 
  1.0298658
 
       NA
 
           NA
  
 
  
 7
 
  48129797
 
  48129992
 
  ENSEMBL   UCSC 
 
  196
 
  5
 
 37006
 
 gene;island
 
 0.963358
 
 0.9787744
 
 Not queried
 
  1.0298634
 
       NA
 
           NA
  
 
  
 10
 
   1209315
 
   1209463
 
  ENSEMBL   UCSC 
 
  149
 
  5
 
 4456
 
 gene;island
 
 0.756376
 
 0.9130558
 
 Not queried
 
  1.0297762
 
       NA
 
           NA
  
 
  
 4
 
 148401661
 
 148401958
 
  ENSEMBL   UCSC 
 
  298
 
  5
 
 30626
 
 tss
 
 0.369369
 
 0.5444474
 
 Not queried
 
  1.0295981
 
       NA
 
           NA
  
 
  
 6
 
   5995766
 
   5996657
 
  ENSEMBL   UCSC 
 
  892
 
  8
 
 35403
 
 island
 
 0.095631
 
 0.0304443
 
 Not queried
 
  1.0295900
 
       NA
 
           NA
  
 
  
 11
 
  61159602
 
  61159837
 
  ENSEMBL   UCSC 
 
  236
 
  7
 
 7334
 
 tss;island
 
 0.461724
 
 0.0626719
 
 Not queried
 
  1.0294876
 
       NA
 
           NA
  
 
  
 6
 
 134274008
 
 134274298
 
  ENSEMBL   UCSC 
 
  291
 
  5
 
 35233
 
 tss
 
 0.013512
 
 0.0075066
 
 Not queried
 
  1.0292539
 
       NA
 
           NA
  
 
  
 1
 
 230468464
 
 230468773
 
  ENSEMBL   UCSC 
 
  310
 
  5
 
 1071
 
 gene;island
 
 0.331729
 
 0.3606278
 
 Not queried
 
  1.0290759
 
       NA
 
           NA
  
 
  
 10
 
  11047271
 
  11047586
 
  ENSEMBL   UCSC 
 
  316
 
  6
 
 4491
 
 gene
 
 0.818857
 
 0.9147146
 
 Not queried
 
  1.0289129
 
       NA
 
           NA
  
 
  
 6
 
 168615184
 
 168615500
 
  ENSEMBL   UCSC 
 
  317
 
  6
 
 36661
 
 island
 
 0.584421
 
 0.7458609
 
 Not queried
 
  1.0287465
 
       NA
 
           NA
  
 
  
 12
 
 104351068
 
 104351300
 
  ENSEMBL   UCSC 
 
  233
 
  4
 
 9954
 
 tss
 
 0.014083
 
 0.0092749
 
 Not queried
 
  1.0285505
 
       NA
 
           NA
  
 
  
 15
 
  96876248
 
  96876486
 
  ENSEMBL   UCSC 
 
  239
 
  5
 
 13494
 
 gene;tss;island
 
 0.052866
 
 0.1672415
 
 Not queried
 
  1.0284493
 
       NA
 
           NA
  
 
  
 5
 
  16179873
 
  16180419
 
  ENSEMBL   UCSC 
 
  547
 
 13
 
 32702
 
 island
 
 0.027381
 
 0.0427912
 
 Not queried
 
  1.0284425
 
       NA
 
           NA
  
 
  
 19
 
  57988958
 
  57989282
 
  ENSEMBL   UCSC 
 
  325
 
  8
 
 21116
 
 tss
 
 0.051842
 
 0.0205126
 
 Not queried
 
  1.0283627
 
       NA
 
           NA
  
 
  
 3
 
 119187426
 
 119187686
 
  ENSEMBL   UCSC 
 
  261
 
  4
 
 28521
 
 tss;island
 
 0.013310
 
 0.2222705
 
 Not queried
 
  1.0282802
 
       NA
 
           NA
  
 
  
 10
 
  94452236
 
  94452554
 
  ENSEMBL   UCSC 
 
  319
 
  4
 
 4677
 
 gene;island
 
 0.040087
 
 0.0718834
 
 Not queried
 
  1.0280202
 
       NA
 
           NA
  
 
  
 5
 
 140719008
 
 140719302
 
  ENSEMBL   UCSC 
 
  295
 
  4
 
 31817
 
 gene;island
 
 0.855259
 
 0.5918107
 
 Not queried
 
  1.0276804
 
       NA
 
           NA
  
 
  
 11
 
  62414183
 
  62414275
 
  ENSEMBL   UCSC 
 
   93
 
  5
 
 7351
 
 tss
 
 0.007901
 
 0.0076707
 
 Not queried
 
  1.0276512
 
       NA
 
           NA
  
 
  
 17
 
   7083015
 
   7083519
 
  ENSEMBL   UCSC 
 
  505
 
  5
 
 17061
 
 tss
 
 0.399284
 
 0.5607135
 
 Not queried
 
  1.0276239
 
       NA
 
           NA
  
 
  
 4
 
 164253207
 
 164254230
 
  ENSEMBL   UCSC 
 
 1024
 
 11
 
 31400
 
 island
 
 0.026977
 
 0.0174842
 
 Not queried
 
  1.0275775
 
       NA
 
           NA
  
 
  
 11
 
  63912583
 
  63912769
 
  ENSEMBL   UCSC 
 
  187
 
  4
 
 6666
 
 gene
 
 0.960124
 
 0.9823120
 
 Not queried
 
  1.0275596
 
       NA
 
           NA
  
 
  
 6
 
  97285662
 
  97285948
 
  ENSEMBL   UCSC 
 
  287
 
  5
 
 35128
 
 tss;island
 
 0.005924
 
 0.0047635
 
 Not queried
 
  1.0275433
 
       NA
 
           NA
  
 
  
 17
 
  39844656
 
  39845092
 
  ENSEMBL   UCSC 
 
  437
 
  8
 
 17342
 
 tss
 
 0.004250
 
 0.0046711
 
 Not queried
 
  1.0273145
 
       NA
 
           NA
  
 
  
 4
 
  77135162
 
  77135506
 
  ENSEMBL   UCSC 
 
  345
 
  7
 
 30446
 
 tss
 
 0.012458
 
 0.0048541
 
 Not queried
 
  1.0270425
 
       NA
 
           NA
  
 
  
 19
 
  19729252
 
  19729732
 
  ENSEMBL   UCSC 
 
  481
 
 10
 
 21823
 
 island
 
 0.011081
 
 0.0146437
 
 Not queried
 
  1.0270261
 
       NA
 
           NA
  
 
  
 3
 
  58477487
 
  58477622
 
  ENSEMBL   UCSC 
 
  136
 
  4
 
 28418
 
 tss;island
 
 0.002202
 
 0.0052703
 
 Not queried
 
  1.0267974
 
       NA
 
           NA
  
 
  
 13
 
 103053409
 
 103053761
 
  ENSEMBL   UCSC 
 
  353
 
  4
 
 11216
 
 gene;island
 
 0.018383
 
 0.0368695
 
 Not queried
 
  1.0267911
 
       NA
 
           NA
  
 
  
 11
 
    915163
 
    915440
 
  ENSEMBL   UCSC 
 
  278
 
  6
 
 7035
 
 tss;island
 
 0.290659
 
 0.5834491
 
 Not queried
 
  1.0266688
 
       NA
 
           NA
  
 
  
 17
 
  30814514
 
  30814814
 
  ENSEMBL   UCSC 
 
  301
 
  5
 
 16513
 
 gene;island
 
 0.015052
 
 0.0093225
 
 Not queried
 
  1.0266444
 
       NA
 
           NA
  
 
  
 8
 
 104310606
 
 104310886
 
  ENSEMBL   UCSC 
 
  281
 
  6
 
 39857
 
 tss
 
 0.009668
 
 0.0124249
 
 Not queried
 
  1.0266186
 
       NA
 
           NA
  
 
  
 2
 
  54557836
 
  54557974
 
  ENSEMBL   UCSC 
 
  139
 
  6
 
 23355
 
 tss
 
 0.011034
 
 0.0113040
 
 Not queried
 
  1.0262971
 
       NA
 
           NA
  
 
  
 2
 
 107459661
 
 107459860
 
  ENSEMBL   UCSC 
 
  200
 
  4
 
 22816
 
 gene;island
 
 0.610084
 
 0.7934849
 
 Not queried
 
  1.0262588
 
       NA
 
           NA
  
 
  
 4
 
  74847646
 
  74848016
 
  ENSEMBL   UCSC 
 
  371
 
  8
 
 31099
 
 island
 
 0.531131
 
 0.6641830
 
 Not queried
 
  1.0262368
 
       NA
 
           NA
  
 
  
 2
 
  71134524
 
  71134936
 
  ENSEMBL   UCSC 
 
  413
 
  6
 
 22726
 
 gene;island
 
 0.014772
 
 0.0558499
 
 Not queried
 
  1.0261632
 
       NA
 
           NA
  
 
  
 14
 
  75642944
 
  75643727
 
  ENSEMBL   UCSC 
 
  784
 
 11
 
 13014
 
 island
 
 0.003338
 
 0.0019049
 
 Not queried
 
  1.0259247
 
       NA
 
           NA
  
 
  
 6
 
  26021768
 
  26021843
 
  ENSEMBL   UCSC 
 
   76
 
  4
 
 34679
 
 tss
 
 0.002424
 
 0.0044362
 
 Not queried
 
  1.0257078
 
       NA
 
           NA
  
 
  
 12
 
 133007675
 
 133008035
 
  ENSEMBL   UCSC 
 
  361
 
  5
 
 11053
 
 island
 
 0.548517
 
 0.5141251
 
 Not queried
 
  1.0256858
 
       NA
 
           NA
  
 
  
 20
 
  61446962
 
  61448325
 
  ENSEMBL   UCSC 
 
 1364
 
 42
 
 25882
 
 tss;island
 
 0.175629
 
 0.0948077
 
 Not queried
 
  1.0253035
 
       NA
 
           NA
  
 
  
 12
 
  13197196
 
  13197827
 
  ENSEMBL   UCSC 
 
  632
 
  5
 
 10260
 
 island
 
 0.004391
 
 0.0023376
 
 Not queried
 
  1.0252329
 
       NA
 
           NA
  
 
  
 2
 
 171568791
 
 171569381
 
  ENSEMBL   UCSC 
 
  591
 
  7
 
 24845
 
 island
 
 0.387345
 
 0.0824426
 
 Not queried
 
  1.0250270
 
       NA
 
           NA
  
 
  
 12
 
  48500072
 
  48500318
 
  ENSEMBL   UCSC 
 
  247
 
  5
 
 9110
 
 gene;tss;island
 
 0.000294
 
 0.0004263
 
 Not queried
 
  1.0249587
 
       NA
 
           NA
  
 
  
 4
 
  96760945
 
  96761231
 
  ENSEMBL   UCSC 
 
  287
 
  5
 
 30507
 
 tss
 
 0.707345
 
 0.7522904
 
 Not queried
 
  1.0246273
 
       NA
 
           NA
  
 
  
 7
 
    766100
 
    766323
 
  ENSEMBL   UCSC 
 
  224
 
  6
 
 37389
 
 tss
 
 0.001806
 
 0.0046271
 
 Not queried
 
  1.0245433
 
       NA
 
           NA
  
 
  
 15
 
  35087506
 
  35087773
 
  ENSEMBL   UCSC 
 
  268
 
  5
 
 13281
 
 gene
 
 0.028640
 
 0.0124962
 
 Not queried
 
  1.0244047
 
       NA
 
           NA
  
 
  
 7
 
 102312200
 
 102312331
 
  ENSEMBL   UCSC 
 
  132
 
  5
 
 37805
 
 tss;island
 
 0.009019
 
 0.0046325
 
 Not queried
 
  1.0243683
 
       NA
 
           NA
  
 
  
 3
 
  42726640
 
  42727160
 
  ENSEMBL   UCSC 
 
  521
 
  5
 
 29019
 
 island
 
 0.211344
 
 0.5941698
 
 Not queried
 
  1.0243304
 
       NA
 
           NA
  
 
  
 10
 
 102731747
 
 102732323
 
  ENSEMBL   UCSC 
 
  577
 
  8
 
 6039
 
 island
 
 0.803566
 
 0.8881745
 
 Not queried
 
  1.0242400
 
       NA
 
           NA
  
 
  
 7
 
 157368881
 
 157369158
 
  ENSEMBL   UCSC 
 
  278
 
  6
 
 37329
 
 gene;island
 
 0.621846
 
 0.6434239
 
 Not queried
 
  1.0240378
 
       NA
 
           NA
  
 
  
 13
 
  50706583
 
  50707065
 
  ENSEMBL   UCSC 
 
  483
 
  6
 
 11743
 
 island
 
 0.312005
 
 0.5089573
 
 Not queried
 
  1.0238958
 
       NA
 
           NA
  
 
  
 19
 
  40006192
 
  40006625
 
  ENSEMBL   UCSC 
 
  434
 
  4
 
 20029
 
 gene;island
 
 0.025751
 
 0.0842738
 
 Not queried
 
  1.0238337
 
       NA
 
           NA
  
 
  
 2
 
 202097062
 
 202097173
 
  ENSEMBL   UCSC 
 
  112
 
  4
 
 23816
 
 tss
 
 0.002260
 
 0.0022295
 
 Not queried
 
  1.0238234
 
       NA
 
           NA
  
 
  
 11
 
 130298172
 
 130298880
 
  ENSEMBL   UCSC 
 
  709
 
 11
 
 8961
 
 island
 
 0.021564
 
 0.0673235
 
 Not queried
 
  1.0236429
 
       NA
 
           NA
  
 
  
 15
 
  25299157
 
  25299260
 
  ENSEMBL   UCSC 
 
  104
 
  4
 
 13520
 
 tss
 
 0.649814
 
 0.7905171
 
 Not queried
 
  1.0234604
 
       NA
 
           NA
  
 
  
 4
 
  84256184
 
  84256229
 
  ENSEMBL   UCSC 
 
   46
 
  4
 
 30044
 
 gene
 
 0.022644
 
 0.0097316
 
 Not queried
 
  1.0232717
 
       NA
 
           NA
  
 
  
 5
 
  43602595
 
  43602919
 
  ENSEMBL   UCSC 
 
  325
 
  5
 
 32050
 
 tss;island
 
 0.004385
 
 0.0026446
 
 Not queried
 
  1.0230338
 
       NA
 
           NA
  
 
  
 1
 
  31191544
 
  31191746
 
  ENSEMBL   UCSC 
 
  203
 
  5
 
 378
 
 gene;island
 
 0.010750
 
 0.0165441
 
 Not queried
 
  1.0229513
 
       NA
 
           NA
  
 
  
 8
 
 116681193
 
 116681258
 
  ENSEMBL   UCSC 
 
   66
 
  4
 
 40547
 
 island
 
 0.002448
 
 0.0011353
 
 Not queried
 
  1.0223301
 
       NA
 
           NA
  
 
  
 4
 
 113432595
 
 113432911
 
  ENSEMBL   UCSC 
 
  317
 
  4
 
 31255
 
 island
 
 0.186635
 
 0.5261952
 
 Not queried
 
  1.0221935
 
       NA
 
           NA
  
 
  
 12
 
  99139301
 
  99139867
 
  ENSEMBL   UCSC 
 
  567
 
  6
 
 9261
 
 gene;island
 
 0.078370
 
 0.0247228
 
 Not queried
 
  1.0221859
 
       NA
 
           NA
  
 
  
 3
 
  48885314
 
  48885665
 
  ENSEMBL   UCSC 
 
  352
 
  9
 
 28308
 
 tss
 
 0.006993
 
 0.0189278
 
 Not queried
 
  1.0221423
 
       NA
 
           NA
  
 
  
 18
 
  61089798
 
  61090076
 
  ENSEMBL   UCSC 
 
  279
 
  6
 
 19311
 
 tss
 
 0.005619
 
 0.0082134
 
 Not queried
 
  1.0217035
 
       NA
 
           NA
  
 
  
 8
 
  11324048
 
  11324448
 
  ENSEMBL   UCSC 
 
  401
 
  5
 
 40080
 
 island
 
 0.004733
 
 0.0149102
 
 Not queried
 
  1.0216771
 
       NA
 
           NA
  
 
  
 5
 
 150460850
 
 150461211
 
  ENSEMBL   UCSC 
 
  362
 
  8
 
 33316
 
 island
 
 0.004021
 
 0.0022271
 
 Not queried
 
  1.0215915
 
       NA
 
           NA
  
 
  
 16
 
  84346722
 
  84346930
 
  ENSEMBL   UCSC 
 
  209
 
  4
 
 14868
 
 gene;island
 
 0.527326
 
 0.2452753
 
 Not queried
 
  1.0215709
 
       NA
 
           NA
  
 
  
 10
 
  71333037
 
  71333216
 
  ENSEMBL   UCSC 
 
  180
 
  6
 
 5806
 
 island
 
 0.039073
 
 0.0776122
 
 Not queried
 
  1.0214914
 
       NA
 
           NA
  
 
  
 11
 
  93583672
 
  93583973
 
  ENSEMBL   UCSC 
 
  302
 
  6
 
 7626
 
 tss
 
 0.408429
 
 0.4049737
 
 Not queried
 
  1.0213529
 
       NA
 
           NA
  
 
  
 4
 
   3873884
 
   3874143
 
  ENSEMBL   UCSC 
 
  260
 
  4
 
 30856
 
 island
 
 0.189129
 
 0.0230401
 
 Not queried
 
  1.0213414
 
       NA
 
           NA
  
 
  
 7
 
  22894795
 
  22894898
 
  ENSEMBL   UCSC 
 
  104
 
  4
 
 37467
 
 tss;island
 
 0.001784
 
 0.0012829
 
 Not queried
 
  1.0210080
 
       NA
 
           NA
  
 
  
 12
 
 120835663
 
 120835856
 
  ENSEMBL   UCSC 
 
  194
 
  4
 
 10889
 
 island
 
 0.623899
 
 0.6247605
 
 Not queried
 
  1.0209613
 
       NA
 
           NA
  
 
  
 4
 
  36245396
 
  36245632
 
  ENSEMBL   UCSC 
 
  237
 
  5
 
 29974
 
 gene;island
 
 0.012011
 
 0.0035740
 
 Not queried
 
  1.0209008
 
       NA
 
           NA
  
 
  
 4
 
 169240001
 
 169240081
 
  ENSEMBL   UCSC 
 
   81
 
  4
 
 30671
 
 tss
 
 0.007573
 
 0.0033625
 
 Not queried
 
  1.0207851
 
       NA
 
           NA
  
 
  
 7
 
    569642
 
    570041
 
  ENSEMBL   UCSC 
 
  400
 
  5
 
 38036
 
 island
 
 0.871776
 
 0.9543338
 
 Not queried
 
  1.0206785
 
       NA
 
           NA
  
 
  
 6
 
  27228101
 
  27228330
 
  ENSEMBL   UCSC 
 
  230
 
  4
 
 35551
 
 island
 
 0.123097
 
 0.3394468
 
 Not queried
 
  1.0206594
 
       NA
 
           NA
  
 
  
 4
 
  15376072
 
  15376170
 
  ENSEMBL   UCSC 
 
   99
 
  6
 
 30308
 
 tss
 
 0.798142
 
 0.9574458
 
 Not queried
 
  1.0206009
 
       NA
 
           NA
  
 
  
 6
 
 135516912
 
 135517046
 
  ENSEMBL   UCSC 
 
  135
 
  4
 
 34485
 
 gene
 
 0.521772
 
 0.5198523
 
 Not queried
 
  1.0205788
 
       NA
 
           NA
  
 
  
 19
 
  13885098
 
  13885429
 
  ENSEMBL   UCSC 
 
  332
 
  5
 
 21655
 
 island
 
 0.004469
 
 0.0081625
 
 Not queried
 
  1.0205594
 
       NA
 
           NA
  
 
  
 2
 
 223162666
 
 223163326
 
  ENSEMBL   UCSC 
 
  661
 
  9
 
 23047
 
 gene;island
 
 0.119445
 
 0.0451042
 
 Not queried
 
  1.0204829
 
       NA
 
           NA
  
 
  
 17
 
   2240014
 
   2240362
 
  ENSEMBL   UCSC 
 
  349
 
  4
 
 16330
 
 gene;tss;island
 
 0.006432
 
 0.0035466
 
 Not queried
 
  1.0202782
 
       NA
 
           NA
  
 
  
 4
 
   5712581
 
   5713100
 
  ENSEMBL   UCSC 
 
  520
 
  9
 
 30880
 
 island
 
 0.008908
 
 0.0054910
 
 Not queried
 
  1.0199366
 
       NA
 
           NA
  
 
  
 7
 
  75443075
 
  75443246
 
  ENSEMBL   UCSC 
 
  172
 
  4
 
 37657
 
 tss
 
 0.012259
 
 0.0277693
 
 Not queried
 
  1.0198052
 
       NA
 
           NA
  
 
  
 2
 
 175190821
 
 175191069
 
  ENSEMBL   UCSC 
 
  249
 
  4
 
 24871
 
 island
 
 0.004801
 
 0.0027620
 
 Not queried
 
  1.0197323
 
       NA
 
           NA
  
 
  
 9
 
 101017931
 
 101018113
 
  ENSEMBL   UCSC 
 
  183
 
  4
 
 40921
 
 island
 
 0.006814
 
 0.0035194
 
 Not queried
 
  1.0196844
 
       NA
 
           NA
  
 
  
 6
 
 170863483
 
 170864036
 
  ENSEMBL   UCSC 
 
  554
 
  6
 
 34584
 
 gene
 
 0.006360
 
 0.0034116
 
 Not queried
 
  1.0196739
 
       NA
 
           NA
  
 
  
 2
 
 233320486
 
 233320854
 
  ENSEMBL   UCSC 
 
  369
 
  5
 
 25194
 
 island
 
 0.059740
 
 0.2015724
 
 Not queried
 
  1.0196071
 
       NA
 
           NA
  
 
  
 5
 
 168728076
 
 168728270
 
  ENSEMBL   UCSC 
 
  195
 
  6
 
 33374
 
 island
 
 0.039199
 
 0.0110004
 
 Not queried
 
  1.0194657
 
       NA
 
           NA
  
 
  
 5
 
   1243714
 
   1243949
 
  ENSEMBL   UCSC 
 
  236
 
  5
 
 31528
 
 gene;island
 
 0.358876
 
 0.5315199
 
 Not queried
 
  1.0194449
 
       NA
 
           NA
  
 
  
 3
 
  51428372
 
  51428651
 
  ENSEMBL   UCSC 
 
  280
 
  7
 
 28363
 
 tss;island
 
 0.013345
 
 0.0103422
 
 Not queried
 
  1.0194422
 
       NA
 
           NA
  
 
  
 17
 
   1657448
 
   1657676
 
  ENSEMBL   UCSC 
 
  229
 
  4
 
 16322
 
 gene;island
 
 0.889769
 
 0.7408944
 
 Not queried
 
  1.0194334
 
       NA
 
           NA
  
 
  
 17
 
  21279561
 
  21279874
 
  ENSEMBL   UCSC 
 
  314
 
  6
 
 18118
 
 island
 
 0.012063
 
 0.0089075
 
 Not queried
 
  1.0191918
 
       NA
 
           NA
  
 
  
 3
 
  49208514
 
  49209008
 
  ENSEMBL   UCSC 
 
  495
 
  8
 
 28323
 
 tss
 
 0.029997
 
 0.0154461
 
 Not queried
 
  1.0191595
 
       NA
 
           NA
  
 
  
 7
 
  27191914
 
  27192656
 
  ENSEMBL   UCSC 
 
  743
 
  8
 
 38351
 
 island
 
 0.033801
 
 0.0377981
 
 Not queried
 
  1.0191389
 
       NA
 
           NA
  
 
  
 5
 
   2564514
 
   2564751
 
  ENSEMBL   UCSC 
 
  238
 
  5
 
 32653
 
 island
 
 0.908120
 
 0.9614018
 
 Not queried
 
  1.0189332
 
       NA
 
           NA
  
 
  
 22
 
  19137874
 
  19138267
 
  ENSEMBL   UCSC 
 
  394
 
  5
 
 26955
 
 tss;island
 
 0.099226
 
 0.0357022
 
 Not queried
 
  1.0188691
 
       NA
 
           NA
  
 
  
 6
 
  41909532
 
  41909736
 
  ENSEMBL   UCSC 
 
  205
 
  5
 
 34335
 
 gene;island
 
 0.016475
 
 0.0073116
 
 Not queried
 
  1.0187018
 
       NA
 
           NA
  
 
  
 6
 
 169013150
 
 169013766
 
  ENSEMBL   UCSC 
 
  617
 
  7
 
 34563
 
 gene
 
 0.509453
 
 0.6782554
 
 Not queried
 
  1.0186959
 
       NA
 
           NA
  
 
  
 12
 
  57915555
 
  57915636
 
  ENSEMBL   UCSC 
 
   82
 
  4
 
 9818
 
 tss;island
 
 0.858020
 
 0.7299755
 
 Not queried
 
  1.0185825
 
       NA
 
           NA
  
 
  
 4
 
 151937026
 
 151937577
 
  ENSEMBL   UCSC 
 
  552
 
  6
 
 30632
 
 tss;island
 
 0.008922
 
 0.0165552
 
 Not queried
 
  1.0185750
 
       NA
 
           NA
  
 
  
 2
 
 203103432
 
 203103763
 
  ENSEMBL   UCSC 
 
  332
 
  5
 
 23824
 
 tss;island
 
 0.018017
 
 0.0095884
 
 Not queried
 
  1.0185541
 
       NA
 
           NA
  
 
  
 10
 
 102295463
 
 102295624
 
  ENSEMBL   UCSC 
 
  162
 
  6
 
 5295
 
 tss
 
 0.006342
 
 0.0041382
 
 Not queried
 
  1.0183132
 
       NA
 
           NA
  
 
  
 4
 
  95128817
 
  95129403
 
  ENSEMBL   UCSC 
 
  587
 
 10
 
 30062
 
 gene
 
 0.004339
 
 0.0035553
 
 Not queried
 
  1.0182815
 
       NA
 
           NA
  
 
  
 10
 
  45455245
 
  45455500
 
  ENSEMBL   UCSC 
 
  256
 
  4
 
 4573
 
 gene
 
 0.016371
 
 0.0158905
 
 Not queried
 
  1.0174093
 
       NA
 
           NA
  
 
  
 11
 
 132107419
 
 132107699
 
  ENSEMBL   UCSC 
 
  281
 
  6
 
 6979
 
 gene
 
 0.267598
 
 0.6004821
 
 Not queried
 
  1.0173378
 
       NA
 
           NA
  
 
  
 10
 
  98802990
 
  98803335
 
  ENSEMBL   UCSC 
 
  346
 
  4
 
 4691
 
 gene;island
 
 0.524394
 
 0.6565650
 
 Not queried
 
  1.0173323
 
       NA
 
           NA
  
 
  
 14
 
  70826282
 
  70826572
 
  ENSEMBL   UCSC 
 
  291
 
  8
 
 12977
 
 island
 
 0.003350
 
 0.0025382
 
 Not queried
 
  1.0170115
 
       NA
 
           NA
  
 
  
 8
 
  27850215
 
  27850423
 
  ENSEMBL   UCSC 
 
  209
 
  6
 
 39619
 
 tss
 
 0.033090
 
 0.0342829
 
 Not queried
 
  1.0170013
 
       NA
 
           NA
  
 
  
 12
 
 110010952
 
 110011337
 
  ENSEMBL   UCSC 
 
  386
 
  8
 
 9293
 
 gene
 
 0.004345
 
 0.0039798
 
 Not queried
 
  1.0166624
 
       NA
 
           NA
  
 
  
 16
 
  75241082
 
  75241305
 
  ENSEMBL   UCSC 
 
  224
 
  4
 
 15359
 
 tss
 
 0.757534
 
 0.8089858
 
 Not queried
 
  1.0164324
 
       NA
 
           NA
  
 
  
 6
 
  30686747
 
  30686922
 
  ENSEMBL   UCSC 
 
  176
 
  4
 
 34785
 
 tss;island
 
 0.523894
 
 0.5019767
 
 Not queried
 
  1.0163006
 
       NA
 
           NA
  
 
  
 18
 
  10471657
 
  10471866
 
  ENSEMBL   UCSC 
 
  210
 
  4
 
 19054
 
 gene;island
 
 0.506818
 
 0.6717115
 
 Not queried
 
  1.0161976
 
       NA
 
           NA
  
 
  
 4
 
  47915995
 
  47916193
 
  ENSEMBL   UCSC 
 
  199
 
  4
 
 29997
 
 gene;island
 
 0.009517
 
 0.0101824
 
 Not queried
 
  1.0161112
 
       NA
 
           NA
  
 
  
 10
 
   1416546
 
   1416920
 
  ENSEMBL   UCSC 
 
  375
 
  6
 
 4462
 
 gene;island
 
 0.806749
 
 0.9039186
 
 Not queried
 
  1.0157792
 
       NA
 
           NA
  
 
  
 22
 
  22307371
 
  22307684
 
  ENSEMBL   UCSC 
 
  314
 
  6
 
 27002
 
 tss
 
 0.066019
 
 0.1668411
 
 Not queried
 
  1.0153734
 
       NA
 
           NA
  
 
  
 6
 
  29585579
 
  29585658
 
  ENSEMBL   UCSC 
 
   80
 
  4
 
 33673
 
 gene
 
 0.875961
 
 0.9171710
 
 Not queried
 
  1.0150771
 
       NA
 
           NA
  
 
  
 3
 
  52931604
 
  52931946
 
  ENSEMBL   UCSC 
 
  343
 
  6
 
 28395
 
 tss
 
 0.005815
 
 0.0037516
 
 Not queried
 
  1.0149379
 
       NA
 
           NA
  
 
  
 22
 
  45403493
 
  45403893
 
  ENSEMBL   UCSC 
 
  401
 
  5
 
 26897
 
 gene;island
 
 0.005212
 
 0.0059171
 
 Not queried
 
  1.0148383
 
       NA
 
           NA
  
 
  
 10
 
 131909018
 
 131909379
 
  ENSEMBL   UCSC 
 
  362
 
  4
 
 6271
 
 island
 
 0.006254
 
 0.0114428
 
 Not queried
 
  1.0147863
 
       NA
 
           NA
  
 
  
 11
 
 111383515
 
 111383668
 
  ENSEMBL   UCSC 
 
  154
 
  6
 
 7685
 
 tss;island
 
 0.084857
 
 0.0410961
 
 Not queried
 
  1.0147581
 
       NA
 
           NA
  
 
  
 14
 
  74318162
 
  74318774
 
  ENSEMBL   UCSC 
 
  613
 
 11
 
 12993
 
 island
 
 0.015025
 
 0.0099514
 
 Not queried
 
  1.0146716
 
       NA
 
           NA
  
 
  
 19
 
  49866779
 
  49867114
 
  ENSEMBL   UCSC 
 
  336
 
 10
 
 20943
 
 tss;island
 
 0.007414
 
 0.0028833
 
 Not queried
 
  1.0146623
 
       NA
 
           NA
  
 
  
 19
 
  54392916
 
  54393394
 
  ENSEMBL   UCSC 
 
  479
 
  5
 
 20210
 
 gene;island
 
 0.362771
 
 0.0354463
 
 Not queried
 
  1.0145504
 
       NA
 
           NA
  
 
  
 2
 
  86564436
 
  86564659
 
  ENSEMBL   UCSC 
 
  224
 
  4
 
 22769
 
 gene;island
 
 0.045983
 
 0.0213358
 
 Not queried
 
  1.0145284
 
       NA
 
           NA
  
 
  
 17
 
  79869748
 
  79870056
 
  ENSEMBL   UCSC 
 
  309
 
  6
 
 17756
 
 tss;island
 
 0.731471
 
 0.5847125
 
 Not queried
 
  1.0137089
 
       NA
 
           NA
  
 
  
 19
 
  34263905
 
  34264111
 
  ENSEMBL   UCSC 
 
  207
 
  4
 
 19975
 
 gene;island
 
 0.136841
 
 0.3318825
 
 Not queried
 
  1.0136805
 
       NA
 
           NA
  
 
  
 17
 
  41984994
 
  41985408
 
  ENSEMBL   UCSC 
 
  415
 
  6
 
 18380
 
 island
 
 0.030833
 
 0.0117249
 
 Not queried
 
  1.0136682
 
       NA
 
           NA
  
 
  
 6
 
  31838402
 
  31839025
 
  ENSEMBL   UCSC 
 
  624
 
 15
 
 33978
 
 gene
 
 0.612353
 
 0.6157709
 
 Not queried
 
  1.0136024
 
       NA
 
           NA
  
 
  
 19
 
  58514497
 
  58514839
 
  ENSEMBL   UCSC 
 
  343
 
  4
 
 22489
 
 island
 
 0.415095
 
 0.4053012
 
 Not queried
 
  1.0135519
 
       NA
 
           NA
  
 
  
 19
 
  58951434
 
  58951885
 
  ENSEMBL   UCSC 
 
  452
 
  9
 
 22509
 
 island
 
 0.003071
 
 0.0052227
 
 Not queried
 
  1.0135049
 
       NA
 
           NA
  
 
  
 22
 
  21984385
 
  21984483
 
  ENSEMBL   UCSC 
 
   99
 
  6
 
 26995
 
 tss;island
 
 0.001487
 
 0.0020668
 
 Not queried
 
  1.0134681
 
       NA
 
           NA
  
 
  
 13
 
  41363245
 
  41363591
 
  ENSEMBL   UCSC 
 
  347
 
  4
 
 11687
 
 island
 
 0.003448
 
 0.0016675
 
 Not queried
 
  1.0133819
 
       NA
 
           NA
  
 
  
 6
 
  29590966
 
  29591155
 
  ENSEMBL   UCSC 
 
  190
 
  5
 
 33676
 
 gene
 
 0.486321
 
 0.6798394
 
 Not queried
 
  1.0133181
 
       NA
 
           NA
  
 
  
 17
 
  14213447
 
  14213798
 
  ENSEMBL   UCSC 
 
  352
 
  4
 
 16452
 
 gene;island
 
 0.647802
 
 0.7600655
 
 Not queried
 
  1.0132952
 
       NA
 
           NA
  
 
  
 19
 
  50831762
 
  50832037
 
  ENSEMBL   UCSC 
 
  276
 
  5
 
 20173
 
 gene;island
 
 0.022730
 
 0.0266781
 
 Not queried
 
  1.0131047
 
       NA
 
           NA
  
 
  
 21
 
  39493025
 
  39493629
 
  ENSEMBL   UCSC 
 
  605
 
 11
 
 26545
 
 tss
 
 0.762238
 
 0.8428859
 
 Not queried
 
  1.0129455
 
       NA
 
           NA
  
 
  
 4
 
 102268622
 
 102268957
 
  ENSEMBL   UCSC 
 
  336
 
  6
 
 31205
 
 island
 
 0.007539
 
 0.0079195
 
 Not queried
 
  1.0129059
 
       NA
 
           NA
  
 
  
 4
 
   8006893
 
   8007156
 
  ENSEMBL   UCSC 
 
  264
 
  4
 
 29944
 
 gene
 
 0.529288
 
 0.7267852
 
 Not queried
 
  1.0128750
 
       NA
 
           NA
  
 
  
 1
 
 231155449
 
 231155983
 
  ENSEMBL   UCSC 
 
  535
 
 12
 
 1075
 
 gene
 
 0.626114
 
 0.8144372
 
 Not queried
 
  1.0123824
 
       NA
 
           NA
  
 
  
 19
 
  51670251
 
  51670552
 
  ENSEMBL   UCSC 
 
  302
 
  5
 
 20994
 
 tss
 
 0.011753
 
 0.0176872
 
 Not queried
 
  1.0121051
 
       NA
 
           NA
  
 
  
 11
 
  23752001
 
  23752469
 
  ENSEMBL   UCSC 
 
  469
 
  5
 
 8138
 
 island
 
 0.524273
 
 0.6649188
 
 Not queried
 
  1.0118788
 
       NA
 
           NA
  
 
  
 20
 
  46130755
 
  46131144
 
  ENSEMBL   UCSC 
 
  390
 
  5
 
 25441
 
 gene
 
 0.002130
 
 0.0055322
 
 Not queried
 
  1.0115966
 
       NA
 
           NA
  
 
  
 8
 
   8820844
 
   8821258
 
  ENSEMBL   UCSC 
 
  415
 
  5
 
 40059
 
 island
 
 0.072582
 
 0.0156237
 
 Not queried
 
  1.0114701
 
       NA
 
           NA
  
 
  
 14
 
  37117706
 
  37117947
 
  ENSEMBL   UCSC 
 
  242
 
  4
 
 12789
 
 island
 
 0.034969
 
 0.1658640
 
 Not queried
 
  1.0113241
 
       NA
 
           NA
  
 
  
 2
 
 189156874
 
 189157361
 
  ENSEMBL   UCSC 
 
  488
 
  9
 
 23777
 
 tss
 
 0.029218
 
 0.0783745
 
 Not queried
 
  1.0112160
 
       NA
 
           NA
  
 
  
 2
 
 189156874
 
 189157566
 
  ENSEMBL   UCSC 
 
  693
 
 11
 
 24953
 
 island
 
 0.029218
 
 0.0783745
 
 Not queried
 
  1.0112160
 
       NA
 
           NA
  
 
  
 6
 
  31629900
 
  31630923
 
  ENSEMBL   UCSC 
 
 1024
 
 10
 
 33930
 
 gene;island
 
 0.709325
 
 0.8636215
 
 Not queried
 
  1.0111283
 
       NA
 
           NA
  
 
  
 1
 
 108508067
 
 108508207
 
  ENSEMBL   UCSC 
 
  141
 
  4
 
 1788
 
 tss;island
 
 0.008866
 
 0.0150469
 
 Not queried
 
  1.0108832
 
       NA
 
           NA
  
 
  
 4
 
 140221967
 
 140222272
 
  ENSEMBL   UCSC 
 
  306
 
  4
 
 30599
 
 tss;island
 
 0.053567
 
 0.0097223
 
 Not queried
 
  1.0107610
 
       NA
 
           NA
  
 
  
 19
 
  23653837
 
  23654274
 
  ENSEMBL   UCSC 
 
  438
 
  5
 
 21847
 
 island
 
 0.259646
 
 0.1674625
 
 Not queried
 
  1.0107588
 
       NA
 
           NA
  
 
  
 3
 
 138679347
 
 138679578
 
  ENSEMBL   UCSC 
 
  232
 
  4
 
 29531
 
 island
 
 0.026069
 
 0.0196050
 
 Not queried
 
  1.0106493
 
       NA
 
           NA
  
 
  
 12
 
  52679967
 
  52680245
 
  ENSEMBL   UCSC 
 
  279
 
  4
 
 9136
 
 gene
 
 0.716270
 
 0.8488224
 
 Not queried
 
  1.0105197
 
       NA
 
           NA
  
 
  
 17
 
  16120399
 
  16120890
 
  ENSEMBL   UCSC 
 
  492
 
  9
 
 18048
 
 island
 
 0.007238
 
 0.0060126
 
 Not queried
 
  1.0104913
 
       NA
 
           NA
  
 
  
 7
 
 105221826
 
 105222180
 
  ENSEMBL   UCSC 
 
  355
 
  8
 
 38821
 
 island
 
 0.041400
 
 0.0212603
 
 Not queried
 
  1.0104578
 
       NA
 
           NA
  
 
  
 12
 
  21654745
 
  21654897
 
  ENSEMBL   UCSC 
 
  153
 
  4
 
 9078
 
 gene
 
 0.026922
 
 0.0094816
 
 Not queried
 
  1.0102012
 
       NA
 
           NA
  
 
  
 1
 
 207626214
 
 207626535
 
  ENSEMBL   UCSC 
 
  322
 
  4
 
 2221
 
 tss;island
 
 0.961845
 
 0.9792584
 
 Not queried
 
  1.0101634
 
       NA
 
           NA
  
 
  
 7
 
  29603121
 
  29603407
 
  ENSEMBL   UCSC 
 
  287
 
  7
 
 37512
 
 tss
 
 0.037251
 
 0.0182182
 
 Not queried
 
  1.0100793
 
       NA
 
           NA
  
 
  
 13
 
  45694523
 
  45694609
 
  ENSEMBL   UCSC 
 
   87
 
  7
 
 11419
 
 tss
 
 0.007206
 
 0.0042113
 
 Not queried
 
  1.0099867
 
       NA
 
           NA
  
 
  
 11
 
 128813442
 
 128813583
 
  ENSEMBL   UCSC 
 
  142
 
  4
 
 7810
 
 tss
 
 0.811644
 
 0.9098381
 
 Not queried
 
  1.0099437
 
       NA
 
           NA
  
 
  
 12
 
  16758692
 
  16759029
 
  ENSEMBL   UCSC 
 
  338
 
  6
 
 9072
 
 gene
 
 0.008503
 
 0.0096678
 
 Not queried
 
  1.0097138
 
       NA
 
           NA
  
 
  
 6
 
  53412803
 
  53413189
 
  ENSEMBL   UCSC 
 
  387
 
  5
 
 36215
 
 island
 
 0.002840
 
 0.0055523
 
 Not queried
 
  1.0095814
 
       NA
 
           NA
  
 
  
 4
 
 141294522
 
 141294594
 
  ENSEMBL   UCSC 
 
   73
 
  5
 
 30605
 
 tss
 
 0.006834
 
 0.0086977
 
 Not queried
 
  1.0095189
 
       NA
 
           NA
  
 
  
 6
 
  79576851
 
  79577177
 
  ENSEMBL   UCSC 
 
  327
 
  8
 
 35079
 
 tss
 
 0.003526
 
 0.0019361
 
 Not queried
 
  1.0093815
 
       NA
 
           NA
  
 
  
 15
 
  74422275
 
  74423077
 
  ENSEMBL   UCSC 
 
  803
 
 14
 
 13417
 
 gene;tss;island
 
 0.032765
 
 0.0213289
 
 Not queried
 
  1.0092166
 
       NA
 
           NA
  
 
  
 17
 
  17726965
 
  17727281
 
  ENSEMBL   UCSC 
 
  317
 
  4
 
 16469
 
 gene;island
 
 0.008063
 
 0.0037441
 
 Not queried
 
  1.0091673
 
       NA
 
           NA
  
 
  
 17
 
  48911141
 
  48911513
 
  ENSEMBL   UCSC 
 
  373
 
  6
 
 17507
 
 tss
 
 0.698136
 
 0.9153104
 
 Not queried
 
  1.0087874
 
       NA
 
           NA
  
 
  
 6
 
  33172817
 
  33173581
 
  ENSEMBL   UCSC 
 
  765
 
 17
 
 34228
 
 gene;island
 
 0.032622
 
 0.0959937
 
 Not queried
 
  1.0084280
 
       NA
 
           NA
  
 
  
 7
 
   5183992
 
   5184295
 
  ENSEMBL   UCSC 
 
  304
 
  4
 
 38212
 
 island
 
 0.307573
 
 0.3830005
 
 Not queried
 
  1.0081230
 
       NA
 
           NA
  
 
  
 11
 
  73587557
 
  73587864
 
  ENSEMBL   UCSC 
 
  308
 
  4
 
 6823
 
 gene
 
 0.003421
 
 0.0031455
 
 Not queried
 
  1.0077022
 
       NA
 
           NA
  
 
  
 12
 
 133102136
 
 133102529
 
  ENSEMBL   UCSC 
 
  394
 
  4
 
 9450
 
 gene;island
 
 0.794714
 
 0.6983857
 
 Not queried
 
  1.0074810
 
       NA
 
           NA
  
 
  
 1
 
 109288968
 
 109289113
 
  ENSEMBL   UCSC 
 
  146
 
  6
 
 1792
 
 tss
 
 0.007946
 
 0.0104612
 
 Not queried
 
  1.0071549
 
       NA
 
           NA
  
 
  
 16
 
  89070647
 
  89070903
 
  ENSEMBL   UCSC 
 
  257
 
  4
 
 16235
 
 island
 
 0.020085
 
 0.0576876
 
 Not queried
 
  1.0071297
 
       NA
 
           NA
  
 
  
 1
 
  45278971
 
  45279349
 
  ENSEMBL   UCSC 
 
  379
 
  4
 
 498
 
 gene;island
 
 0.722244
 
 0.5021772
 
 Not queried
 
  1.0070878
 
       NA
 
           NA
  
 
  
 17
 
  37821447
 
  37821661
 
  ENSEMBL   UCSC 
 
  215
 
  5
 
 18283
 
 island
 
 0.847585
 
 0.8142854
 
 Not queried
 
  1.0070009
 
       NA
 
           NA
  
 
  
 12
 
  52760975
 
  52761416
 
  ENSEMBL   UCSC 
 
  442
 
  6
 
 10442
 
 island
 
 0.367938
 
 0.5301581
 
 Not queried
 
  1.0063142
 
       NA
 
           NA
  
 
  
 7
 
 151553471
 
 151553868
 
  ENSEMBL   UCSC 
 
  398
 
  6
 
 37297
 
 gene
 
 0.551406
 
 0.6345641
 
 Not queried
 
  1.0062850
 
       NA
 
           NA
  
 
  
 19
 
  52531703
 
  52531956
 
  ENSEMBL   UCSC 
 
  254
 
  6
 
 21004
 
 tss
 
 0.017824
 
 0.0109356
 
 Not queried
 
  1.0061925
 
       NA
 
           NA
  
 
  
 9
 
  99540324
 
  99540614
 
  ENSEMBL   UCSC 
 
  291
 
  4
 
 40918
 
 island
 
 0.001824
 
 0.0004112
 
 Not queried
 
  1.0060573
 
       NA
 
           NA
  
 
  
 8
 
  32406613
 
  32406928
 
  ENSEMBL   UCSC 
 
  316
 
  4
 
 39274
 
 gene;island
 
 0.028661
 
 0.0091195
 
 Not queried
 
  1.0060553
 
       NA
 
           NA
  
 
  
 17
 
  73780688
 
  73781011
 
  ENSEMBL   UCSC 
 
  324
 
  4
 
 16779
 
 gene
 
 0.011771
 
 0.0108887
 
 Not queried
 
  1.0060550
 
       NA
 
           NA
  
 
  
 16
 
   9058197
 
   9058365
 
  ENSEMBL   UCSC 
 
  169
 
  4
 
 15084
 
 tss;island
 
 0.033248
 
 0.0196765
 
 Not queried
 
  1.0058834
 
       NA
 
           NA
  
 
  
 3
 
  99904510
 
  99904650
 
  ENSEMBL   UCSC 
 
  141
 
  4
 
 28465
 
 tss
 
 0.963869
 
 0.9781559
 
 Not queried
 
  1.0057277
 
       NA
 
           NA
  
 
  
 17
 
  26574231
 
  26574374
 
  ENSEMBL   UCSC 
 
  144
 
  4
 
 17206
 
 tss
 
 0.266903
 
 0.4112464
 
 Not queried
 
  1.0057047
 
       NA
 
           NA
  
 
  
 2
 
 233390712
 
 233390969
 
  ENSEMBL   UCSC 
 
  258
 
  5
 
 25198
 
 island
 
 0.032800
 
 0.3944005
 
 Not queried
 
  1.0054642
 
       NA
 
           NA
  
 
  
 1
 
  86046512
 
  86046961
 
  ENSEMBL   UCSC 
 
  450
 
  5
 
 608
 
 gene
 
 0.003980
 
 0.0076799
 
 Not queried
 
  1.0053692
 
       NA
 
           NA
  
 
  
 13
 
  37574747
 
  37575268
 
  ENSEMBL   UCSC 
 
  522
 
  6
 
 11137
 
 gene
 
 0.008240
 
 0.0065798
 
 Not queried
 
  1.0053083
 
       NA
 
           NA
  
 
  
 8
 
   1495025
 
   1495332
 
  ENSEMBL   UCSC 
 
  308
 
  5
 
 39166
 
 gene;island
 
 0.468167
 
 0.6150584
 
 Not queried
 
  1.0052194
 
       NA
 
           NA
  
 
  
 7
 
 130794910
 
 130795031
 
  ENSEMBL   UCSC 
 
  122
 
  4
 
 37228
 
 gene
 
 0.024724
 
 0.0122595
 
 Not queried
 
  1.0052018
 
       NA
 
           NA
  
 
  
 17
 
  16487631
 
  16487821
 
  ENSEMBL   UCSC 
 
  191
 
  4
 
 18056
 
 island
 
 0.025175
 
 0.0224613
 
 Not queried
 
  1.0051436
 
       NA
 
           NA
  
 
  
 3
 
 156878512
 
 156878622
 
  ENSEMBL   UCSC 
 
  111
 
  4
 
 28678
 
 tss;island
 
 0.000344
 
 0.0002246
 
 Not queried
 
  1.0050866
 
       NA
 
           NA
  
 
  
 1
 
   3615390
 
   3615762
 
  ENSEMBL   UCSC 
 
  373
 
  5
 
 139
 
 gene
 
 0.986955
 
 0.9602797
 
 Not queried
 
  1.0048022
 
       NA
 
           NA
  
 
  
 2
 
  45168798
 
  45168924
 
  ENSEMBL   UCSC 
 
  127
 
  4
 
 23333
 
 tss;island
 
 0.003315
 
 0.0240120
 
 Not queried
 
  1.0047017
 
       NA
 
           NA
  
 
  
 10
 
   2815004
 
   2815547
 
  ENSEMBL   UCSC 
 
  544
 
  6
 
 5502
 
 island
 
 0.542105
 
 0.5886597
 
 Not queried
 
  1.0045122
 
       NA
 
           NA
  
 
  
 19
 
  48774392
 
  48774652
 
  ENSEMBL   UCSC 
 
  261
 
  6
 
 20911
 
 tss;island
 
 0.010339
 
 0.0067118
 
 Not queried
 
  1.0044548
 
       NA
 
           NA
  
 
  
 17
 
  15602714
 
  15603030
 
  ENSEMBL   UCSC 
 
  317
 
  4
 
 18042
 
 island
 
 0.013260
 
 0.0106860
 
 Not queried
 
  1.0043710
 
       NA
 
           NA
  
 
  
 10
 
 133953871
 
 133954211
 
  ENSEMBL   UCSC 
 
  341
 
  4
 
 4854
 
 gene;island
 
 0.563854
 
 0.7642105
 
 Not queried
 
  1.0043510
 
       NA
 
           NA
  
 
  
 12
 
  57824134
 
  57824322
 
  ENSEMBL   UCSC 
 
  189
 
  4
 
 10558
 
 island
 
 0.006870
 
 0.0050065
 
 Not queried
 
  1.0043417
 
       NA
 
           NA
  
 
  
 10
 
 100996579
 
 100997023
 
  ENSEMBL   UCSC 
 
  445
 
  4
 
 5279
 
 tss;island
 
 0.194356
 
 0.3295675
 
 Not queried
 
  1.0041602
 
       NA
 
           NA
  
 
  
 2
 
  71787431
 
  71787827
 
  ENSEMBL   UCSC 
 
  397
 
  6
 
 22732
 
 gene;island
 
 0.515618
 
 0.7716144
 
 Not queried
 
  1.0040679
 
       NA
 
           NA
  
 
  
 14
 
  24911599
 
  24912113
 
  ENSEMBL   UCSC 
 
  515
 
  9
 
 12752
 
 island
 
 0.003456
 
 0.0040365
 
 Not queried
 
  1.0039620
 
       NA
 
           NA
  
 
  
 1
 
 207627372
 
 207627601
 
  ENSEMBL   UCSC 
 
  230
 
  5
 
 2222
 
 tss;island
 
 0.005428
 
 0.0105217
 
 Not queried
 
  1.0036134
 
       NA
 
           NA
  
 
  
 19
 
  49944817
 
  49944964
 
  ENSEMBL   UCSC 
 
  148
 
  5
 
 20945
 
 tss
 
 0.040087
 
 0.0132356
 
 Not queried
 
  1.0032834
 
       NA
 
           NA
  
 
  
 6
 
 170863287
 
 170864036
 
  ENSEMBL   UCSC 
 
  750
 
 13
 
 36698
 
 island
 
 0.007362
 
 0.0031486
 
 Not queried
 
  1.0031635
 
       NA
 
           NA
  
 
  
 10
 
  49482723
 
  49482871
 
  ENSEMBL   UCSC 
 
  149
 
  4
 
 5066
 
 tss
 
 0.921224
 
 0.9243953
 
 Not queried
 
  1.0031051
 
       NA
 
           NA
  
 
  
 17
 
  46894486
 
  46894684
 
  ENSEMBL   UCSC 
 
  199
 
  6
 
 17474
 
 tss
 
 0.022133
 
 0.0072328
 
 Not queried
 
  1.0028890
 
       NA
 
           NA
  
 
  
 17
 
  46894191
 
  46894684
 
  ENSEMBL   UCSC 
 
  494
 
 10
 
 18509
 
 island
 
 0.022133
 
 0.0072328
 
 Not queried
 
  1.0028890
 
       NA
 
           NA
  
 
  
 14
 
  70345898
 
  70346082
 
  ENSEMBL   UCSC 
 
  185
 
  5
 
 12484
 
 tss
 
 0.022978
 
 0.0231375
 
 Not queried
 
  1.0028513
 
       NA
 
           NA
  
 
  
 14
 
 105190158
 
 105190590
 
  ENSEMBL   UCSC 
 
  433
 
  6
 
 13216
 
 island
 
 0.011063
 
 0.0050842
 
 Not queried
 
  1.0027032
 
       NA
 
           NA
  
 
  
 10
 
 134918671
 
 134918974
 
  ENSEMBL   UCSC 
 
  304
 
  5
 
 4885
 
 gene;island
 
 0.590258
 
 0.7056141
 
 Not queried
 
  1.0026767
 
       NA
 
           NA
  
 
  
 12
 
  54785003
 
  54785320
 
  ENSEMBL   UCSC 
 
  318
 
  7
 
 10504
 
 island
 
 0.018733
 
 0.0078836
 
 Not queried
 
  1.0026132
 
       NA
 
           NA
  
 
  
 7
 
  43153023
 
  43153392
 
  ENSEMBL   UCSC 
 
  370
 
  5
 
 36980
 
 gene;island
 
 0.045822
 
 0.1811076
 
 Not queried
 
  1.0025210
 
       NA
 
           NA
  
 
  
 1
 
  13910138
 
  13910224
 
  ENSEMBL   UCSC 
 
   87
 
  6
 
 1261
 
 tss;island
 
 0.025641
 
 0.0199409
 
 Not queried
 
  1.0025165
 
       NA
 
           NA
  
 
  
 6
 
  30900149
 
  30900299
 
  ENSEMBL   UCSC 
 
  151
 
  4
 
 34794
 
 tss
 
 0.458163
 
 0.6177013
 
 Not queried
 
  1.0023426
 
       NA
 
           NA
  
 
  
 12
 
   8123380
 
   8123627
 
  ENSEMBL   UCSC 
 
  248
 
  4
 
 10230
 
 island
 
 0.001263
 
 0.0040109
 
 Not queried
 
  1.0023353
 
       NA
 
           NA
  
 
  
 19
 
  18709415
 
  18709521
 
  ENSEMBL   UCSC 
 
  107
 
  5
 
 19938
 
 gene;island
 
 0.452653
 
 0.6159854
 
 Not queried
 
  1.0021614
 
       NA
 
           NA
  
 
  
 3
 
 138170552
 
 138170939
 
  ENSEMBL   UCSC 
 
  388
 
  4
 
 27964
 
 gene;island
 
 0.639405
 
 0.7629571
 
 Not queried
 
  1.0020619
 
       NA
 
           NA
  
 
  
 7
 
  73498045
 
  73498099
 
  ENSEMBL   UCSC 
 
   55
 
  4
 
 37645
 
 tss;island
 
 0.001431
 
 0.0014145
 
 Not queried
 
  1.0018312
 
       NA
 
           NA
  
 
  
 7
 
   1081061
 
   1081709
 
  ENSEMBL   UCSC 
 
  649
 
  6
 
 36739
 
 gene;island
 
 0.919591
 
 0.9370822
 
 Not queried
 
  1.0015259
 
       NA
 
           NA
  
 
  
 16
 
  51147461
 
  51147902
 
  ENSEMBL   UCSC 
 
  442
 
  6
 
 15916
 
 island
 
 0.044256
 
 0.0221940
 
 Not queried
 
  1.0014252
 
       NA
 
           NA
  
 
  
 4
 
  25097558
 
  25098059
 
  ENSEMBL   UCSC 
 
  502
 
  5
 
 30959
 
 island
 
 0.815926
 
 0.8979995
 
 Not queried
 
  1.0012133
 
       NA
 
           NA
  
 
  
 4
 
    981485
 
    981630
 
  ENSEMBL   UCSC 
 
  146
 
  4
 
 29853
 
 gene;island
 
 0.491520
 
 0.7354499
 
 Not queried
 
  1.0010556
 
       NA
 
           NA
  
 
  
 1
 
 221055401
 
 221055964
 
  ENSEMBL   UCSC 
 
  564
 
  6
 
 1021
 
 gene;island
 
 0.948752
 
 0.8731022
 
 Not queried
 
  1.0008733
 
       NA
 
           NA
  
 
  
 19
 
   7197021
 
   7197397
 
  ENSEMBL   UCSC 
 
  377
 
  4
 
 19773
 
 gene;island
 
 0.039145
 
 0.0318998
 
 Not queried
 
  1.0008316
 
       NA
 
           NA
  
 
  
 16
 
  75285526
 
  75285726
 
  ENSEMBL   UCSC 
 
  201
 
  4
 
 15360
 
 tss
 
 0.011113
 
 0.0088980
 
 Not queried
 
  1.0005199
 
       NA
 
           NA
  
 
  
 19
 
  15217635
 
  15218169
 
  ENSEMBL   UCSC 
 
  535
 
  9
 
 20554
 
 tss
 
 0.019020
 
 0.0327424
 
 Not queried
 
  1.0003414
 
       NA
 
           NA
  
 
  
 1
 
 114447334
 
 114447798
 
  ENSEMBL   UCSC 
 
  465
 
  8
 
 1846
 
 tss;island
 
 0.005900
 
 0.0051890
 
 Not queried
 
  1.0003139
 
       NA
 
           NA
  
 
  
 13
 
  88323266
 
  88323607
 
  ENSEMBL   UCSC 
 
  342
 
  5
 
 11807
 
 island
 
 0.003209
 
 0.0060881
 
 Not queried
 
  1.0000408
 
       NA
 
           NA
  
 
  
 17
 
  46806445
 
  46806935
 
  ENSEMBL   UCSC 
 
  491
 
  6
 
 17472
 
 tss;island
 
 0.015803
 
 0.0518035
 
 Not queried
 
  1.0000115
 
       NA
 
           NA
  
 
  
 2
 
 121279886
 
 121280106
 
  ENSEMBL   UCSC 
 
  221
 
  4
 
 24667
 
 island
 
 0.216085
 
 0.3980404
 
 Not queried
 
  0.9998798
 
       NA
 
           NA
  
 
  
 20
 
  33680135
 
  33680493
 
  ENSEMBL   UCSC 
 
  359
 
  4
 
 25404
 
 gene
 
 0.012666
 
 0.0104342
 
 Not queried
 
  0.9996575
 
       NA
 
           NA
  
 
  
 1
 
 151431750
 
 151431931
 
  ENSEMBL   UCSC 
 
  182
 
  4
 
 750
 
 gene
 
 0.007197
 
 0.0104114
 
 Not queried
 
  0.9996525
 
       NA
 
           NA
  
 
  
 11
 
 116708299
 
 116708517
 
  ENSEMBL   UCSC 
 
  219
 
  4
 
 8839
 
 island
 
 0.677306
 
 0.8101774
 
 Not queried
 
  0.9996351
 
       NA
 
           NA
  
 
  
 12
 
  52300911
 
  52301163
 
  ENSEMBL   UCSC 
 
  253
 
  4
 
 9721
 
 tss
 
 0.050251
 
 0.0853813
 
 Not queried
 
  0.9996166
 
       NA
 
           NA
  
 
  
 17
 
  27224801
 
  27225046
 
  ENSEMBL   UCSC 
 
  246
 
  7
 
 17227
 
 tss
 
 0.002737
 
 0.0293337
 
 Not queried
 
  0.9994761
 
       NA
 
           NA
  
 
  
 11
 
  86662968
 
  86663418
 
  ENSEMBL   UCSC 
 
  451
 
  6
 
 6855
 
 gene;island
 
 0.545549
 
 0.7849030
 
 Not queried
 
  0.9991475
 
       NA
 
           NA
  
 
  
 4
 
 157997360
 
 157997880
 
  ENSEMBL   UCSC 
 
  521
 
  7
 
 30161
 
 gene
 
 0.015858
 
 0.0442329
 
 Not queried
 
  0.9991340
 
       NA
 
           NA
  
 
  
 16
 
  30845651
 
  30846150
 
  ENSEMBL   UCSC 
 
  500
 
  5
 
 15850
 
 island
 
 0.946431
 
 0.9582826
 
 Not queried
 
  0.9991210
 
       NA
 
           NA
  
 
  
 6
 
  33394324
 
  33394537
 
  ENSEMBL   UCSC 
 
  214
 
  4
 
 34286
 
 gene;island
 
 0.022117
 
 0.0140892
 
 Not queried
 
  0.9990524
 
       NA
 
           NA
  
 
  
 6
 
  31373932
 
  31374206
 
  ENSEMBL   UCSC 
 
  275
 
  4
 
 33872
 
 gene;island
 
 0.833600
 
 0.7876740
 
 Not queried
 
  0.9988673
 
       NA
 
           NA
  
 
  
 15
 
  25301783
 
  25301950
 
  ENSEMBL   UCSC 
 
  168
 
  4
 
 13521
 
 tss
 
 0.904460
 
 0.9593950
 
 Not queried
 
  0.9987273
 
       NA
 
           NA
  
 
  
 16
 
    838502
 
    838782
 
  ENSEMBL   UCSC 
 
  281
 
  4
 
 14984
 
 tss;island
 
 0.010974
 
 0.0096606
 
 Not queried
 
  0.9986123
 
       NA
 
           NA
  
 
  
 4
 
   7069733
 
   7070051
 
  ENSEMBL   UCSC 
 
  319
 
  8
 
 30905
 
 island
 
 0.012311
 
 0.0035354
 
 Not queried
 
  0.9984324
 
       NA
 
           NA
  
 
  
 16
 
  56622951
 
  56623215
 
  ENSEMBL   UCSC 
 
  265
 
  6
 
 15246
 
 tss
 
 0.197078
 
 0.3176004
 
 Not queried
 
  0.9978223
 
       NA
 
           NA
  
 
  
 1
 
  86046322
 
  86046961
 
  ENSEMBL   UCSC 
 
  640
 
  8
 
 3426
 
 island
 
 0.019198
 
 0.0363309
 
 Not queried
 
  0.9975084
 
       NA
 
           NA
  
 
  
 7
 
 158103293
 
 158103582
 
  ENSEMBL   UCSC 
 
  290
 
  4
 
 37365
 
 gene;island
 
 0.719318
 
 0.8350856
 
 Not queried
 
  0.9973188
 
       NA
 
           NA
  
 
  
 8
 
 145501817
 
 145502297
 
  ENSEMBL   UCSC 
 
  481
 
  5
 
 39513
 
 gene;island
 
 0.892935
 
 0.9224319
 
 Not queried
 
  0.9969997
 
       NA
 
           NA
  
 
  
 10
 
  21784250
 
  21784432
 
  ENSEMBL   UCSC 
 
  183
 
  4
 
 4526
 
 gene;island
 
 0.011363
 
 0.0055777
 
 Not queried
 
  0.9969330
 
       NA
 
           NA
  
 
  
 3
 
 129830295
 
 129830607
 
  ENSEMBL   UCSC 
 
  313
 
  9
 
 28589
 
 tss;island
 
 0.010103
 
 0.0055912
 
 Not queried
 
  0.9967985
 
       NA
 
           NA
  
 
  
 19
 
   3971119
 
   3971272
 
  ENSEMBL   UCSC 
 
  154
 
  4
 
 20355
 
 tss
 
 0.005008
 
 0.0084583
 
 Not queried
 
  0.9965610
 
       NA
 
           NA
  
 
  
 10
 
   7661622
 
   7661796
 
  ENSEMBL   UCSC 
 
  175
 
  4
 
 4937
 
 tss
 
 0.762601
 
 0.9170196
 
 Not queried
 
  0.9964047
 
       NA
 
           NA
  
 
  
 22
 
  38071455
 
  38071677
 
  ENSEMBL   UCSC 
 
  223
 
  7
 
 27468
 
 island
 
 0.020489
 
 0.0956214
 
 Not queried
 
  0.9962487
 
       NA
 
           NA
  
 
  
 1
 
  47697948
 
  47698126
 
  ENSEMBL   UCSC 
 
  179
 
  4
 
 3233
 
 island
 
 0.024725
 
 0.0629202
 
 Not queried
 
  0.9962022
 
       NA
 
           NA
  
 
  
 17
 
  33446966
 
  33447236
 
  ENSEMBL   UCSC 
 
  271
 
  9
 
 17265
 
 tss;island
 
 0.007659
 
 0.0032901
 
 Not queried
 
  0.9959945
 
       NA
 
           NA
  
 
  
 7
 
  28220498
 
  28220645
 
  ENSEMBL   UCSC 
 
  148
 
  8
 
 37503
 
 tss;island
 
 0.014274
 
 0.0274130
 
 Not queried
 
  0.9958228
 
       NA
 
           NA
  
 
  
 17
 
  21416258
 
  21416410
 
  ENSEMBL   UCSC 
 
  153
 
  4
 
 18124
 
 island
 
 0.133646
 
 0.1105454
 
 Not queried
 
  0.9957699
 
       NA
 
           NA
  
 
  
 5
 
   1948875
 
   1949277
 
  ENSEMBL   UCSC 
 
  403
 
  5
 
 32647
 
 island
 
 0.236228
 
 0.3837124
 
 Not queried
 
  0.9956979
 
       NA
 
           NA
  
 
  
 6
 
  32049053
 
  32049953
 
  ENSEMBL   UCSC 
 
  901
 
 19
 
 34036
 
 gene;island
 
 0.551533
 
 0.6170950
 
 Not queried
 
  0.9954811
 
       NA
 
           NA
  
 
  
 17
 
  57408935
 
  57409010
 
  ENSEMBL   UCSC 
 
   76
 
  5
 
 17545
 
 tss
 
 0.000248
 
 0.0003444
 
 Not queried
 
  0.9953184
 
       NA
 
           NA
  
 
  
 20
 
  55100881
 
  55101089
 
  ENSEMBL   UCSC 
 
  209
 
  5
 
 25464
 
 gene
 
 0.639906
 
 0.7763990
 
 Not queried
 
  0.9951771
 
       NA
 
           NA
  
 
  
 2
 
  27294705
 
  27294909
 
  ENSEMBL   UCSC 
 
  205
 
  6
 
 23251
 
 tss;island
 
 0.004650
 
 0.0066715
 
 Not queried
 
  0.9949662
 
       NA
 
           NA
  
 
  
 17
 
  66287480
 
  66287507
 
  ENSEMBL   UCSC 
 
   28
 
  5
 
 17607
 
 tss
 
 0.013077
 
 0.0174082
 
 Not queried
 
  0.9948838
 
       NA
 
           NA
  
 
  
 8
 
  12990213
 
  12991196
 
  ENSEMBL   UCSC 
 
  984
 
 12
 
 39212
 
 gene;island
 
 0.045572
 
 0.0143689
 
 Not queried
 
  0.9948427
 
       NA
 
           NA
  
 
  
 17
 
  74075092
 
  74075336
 
  ENSEMBL   UCSC 
 
  245
 
  5
 
 18772
 
 island
 
 0.627117
 
 0.9304420
 
 Not queried
 
  0.9948032
 
       NA
 
           NA
  
 
  
 4
 
  48484997
 
  48485328
 
  ENSEMBL   UCSC 
 
  332
 
  8
 
 30382
 
 tss;island
 
 0.167694
 
 0.0547163
 
 Not queried
 
  0.9947927
 
       NA
 
           NA
  
 
  
 4
 
  77172617
 
  77173197
 
  ENSEMBL   UCSC 
 
  581
 
 10
 
 31115
 
 island
 
 0.020743
 
 0.0198863
 
 Not queried
 
  0.9946825
 
       NA
 
           NA
  
 
  
 16
 
  30441151
 
  30442009
 
  ENSEMBL   UCSC 
 
  859
 
  9
 
 15829
 
 island
 
 0.016502
 
 0.0240450
 
 Not queried
 
  0.9945873
 
       NA
 
           NA
  
 
  
 7
 
 117513825
 
 117514017
 
  ENSEMBL   UCSC 
 
  193
 
  5
 
 37857
 
 tss;island
 
 0.195619
 
 0.4996782
 
 Not queried
 
  0.9944767
 
       NA
 
           NA
  
 
  
 3
 
  10400395
 
  10400548
 
  ENSEMBL   UCSC 
 
  154
 
  5
 
 27661
 
 gene;island
 
 0.752625
 
 0.8386911
 
 Not queried
 
  0.9942847
 
       NA
 
           NA
  
 
  
 1
 
  47134188
 
  47134527
 
  ENSEMBL   UCSC 
 
  340
 
  8
 
 1604
 
 tss;island
 
 0.051882
 
 0.1810954
 
 Not queried
 
  0.9941793
 
       NA
 
           NA
  
 
  
 10
 
  43572065
 
  43572467
 
  ENSEMBL   UCSC 
 
  403
 
  7
 
 5044
 
 tss
 
 0.013442
 
 0.0121310
 
 Not queried
 
  0.9940312
 
       NA
 
           NA
  
 
  
 11
 
  22214333
 
  22214683
 
  ENSEMBL   UCSC 
 
  351
 
  5
 
 7201
 
 tss
 
 0.014396
 
 0.0159918
 
 Not queried
 
  0.9939308
 
       NA
 
           NA
  
 
  
 14
 
  76127164
 
  76127409
 
  ENSEMBL   UCSC 
 
  246
 
  6
 
 12146
 
 gene;tss;island
 
 0.011241
 
 0.0059224
 
 Not queried
 
  0.9939061
 
       NA
 
           NA
  
 
  
 8
 
    496327
 
    496739
 
  ENSEMBL   UCSC 
 
  413
 
  5
 
 39533
 
 tss;island
 
 0.577191
 
 0.7936643
 
 Not queried
 
  0.9938330
 
       NA
 
           NA
  
 
  
 8
 
  21640100
 
  21640304
 
  ENSEMBL   UCSC 
 
  205
 
  4
 
 39223
 
 gene;island
 
 0.648001
 
 0.8178055
 
 Not queried
 
  0.9937234
 
       NA
 
           NA
  
 
  
 13
 
  41344913
 
  41345332
 
  ENSEMBL   UCSC 
 
  420
 
  7
 
 11144
 
 gene
 
 0.005869
 
 0.0041529
 
 Not queried
 
  0.9935238
 
       NA
 
           NA
  
 
  
 19
 
  36138893
 
  36139062
 
  ENSEMBL   UCSC 
 
  170
 
  6
 
 20711
 
 tss
 
 0.019982
 
 0.0084625
 
 Not queried
 
  0.9934881
 
       NA
 
           NA
  
 
  
 3
 
  62355319
 
  62355525
 
  ENSEMBL   UCSC 
 
  207
 
  4
 
 29246
 
 island
 
 0.471026
 
 0.3848298
 
 Not queried
 
  0.9934535
 
       NA
 
           NA
  
 
  
 1
 
 150521958
 
 150522367
 
  ENSEMBL   UCSC 
 
  410
 
  4
 
 740
 
 gene
 
 0.691901
 
 0.4336728
 
 Not queried
 
  0.9934414
 
       NA
 
           NA
  
 
  
 21
 
  38353114
 
  38353318
 
  ENSEMBL   UCSC 
 
  205
 
  4
 
 26420
 
 gene;island
 
 0.014122
 
 0.0195321
 
 Not queried
 
  0.9933340
 
       NA
 
           NA
  
 
  
 5
 
  76115672
 
  76116169
 
  ENSEMBL   UCSC 
 
  498
 
  6
 
 31652
 
 gene;island
 
 0.027277
 
 0.0785753
 
 Not queried
 
  0.9932579
 
       NA
 
           NA
  
 
  
 3
 
  53880717
 
  53880872
 
  ENSEMBL   UCSC 
 
  156
 
  4
 
 27832
 
 gene;tss;island
 
 0.117267
 
 0.0337157
 
 Not queried
 
  0.9930525
 
       NA
 
           NA
  
 
  
 22
 
  48027641
 
  48027921
 
  ENSEMBL   UCSC 
 
  281
 
  4
 
 27599
 
 island
 
 0.596967
 
 0.6885438
 
 Not queried
 
  0.9927255
 
       NA
 
           NA
  
 
  
 3
 
 141205540
 
 141205767
 
  ENSEMBL   UCSC 
 
  228
 
  5
 
 28638
 
 tss;island
 
 0.005829
 
 0.0171633
 
 Not queried
 
  0.9925933
 
       NA
 
           NA
  
 
  
 19
 
  18063041
 
  18063313
 
  ENSEMBL   UCSC 
 
  273
 
  4
 
 19925
 
 gene;island
 
 0.004608
 
 0.0049408
 
 Not queried
 
  0.9921332
 
       NA
 
           NA
  
 
  
 2
 
 203036093
 
 203036235
 
  ENSEMBL   UCSC 
 
  143
 
  4
 
 25017
 
 island
 
 0.130550
 
 0.2901945
 
 Not queried
 
  0.9919869
 
       NA
 
           NA
  
 
  
 10
 
  72575514
 
  72575605
 
  ENSEMBL   UCSC 
 
   92
 
  6
 
 5139
 
 tss;island
 
 0.010501
 
 0.0126464
 
 Not queried
 
  0.9919575
 
       NA
 
           NA
  
 
  
 6
 
  32015618
 
  32015773
 
  ENSEMBL   UCSC 
 
  156
 
  8
 
 34014
 
 gene
 
 0.473387
 
 0.7124053
 
 Not queried
 
  0.9915153
 
       NA
 
           NA
  
 
  
 9
 
  36572645
 
  36572901
 
  ENSEMBL   UCSC 
 
  257
 
  4
 
 40813
 
 tss
 
 0.006635
 
 0.0053726
 
 Not queried
 
  0.9913536
 
       NA
 
           NA
  
 
  
 18
 
  28622474
 
  28623159
 
  ENSEMBL   UCSC 
 
  686
 
 13
 
 19446
 
 island
 
 0.372071
 
 0.1480906
 
 Not queried
 
  0.9912890
 
       NA
 
           NA
  
 
  
 17
 
  37123669
 
  37123949
 
  ENSEMBL   UCSC 
 
  281
 
  8
 
 17302
 
 tss
 
 0.234852
 
 0.5032804
 
 Not queried
 
  0.9912817
 
       NA
 
           NA
  
 
  
 11
 
  36310999
 
  36311375
 
  ENSEMBL   UCSC 
 
  377
 
  9
 
 7248
 
 tss
 
 0.007399
 
 0.0170953
 
 Not queried
 
  0.9911549
 
       NA
 
           NA
  
 
  
 1
 
  44440160
 
  44440401
 
  ENSEMBL   UCSC 
 
  242
 
  4
 
 1568
 
 tss;island
 
 0.011703
 
 0.0078785
 
 Not queried
 
  0.9910682
 
       NA
 
           NA
  
 
  
 15
 
  25427394
 
  25427509
 
  ENSEMBL   UCSC 
 
  116
 
  4
 
 13528
 
 tss
 
 0.344115
 
 0.4493721
 
 Not queried
 
  0.9909857
 
       NA
 
           NA
  
 
  
 2
 
  33359198
 
  33359688
 
  ENSEMBL   UCSC 
 
  491
 
  6
 
 22641
 
 gene
 
 0.575387
 
 0.7016279
 
 Not queried
 
  0.9909608
 
       NA
 
           NA
  
 
  
 2
 
 201171255
 
 201171404
 
  ENSEMBL   UCSC 
 
  150
 
  6
 
 22979
 
 gene;island
 
 0.008577
 
 0.0038462
 
 Not queried
 
  0.9906537
 
       NA
 
           NA
  
 
  
 6
 
  34164073
 
  34164564
 
  ENSEMBL   UCSC 
 
  492
 
  6
 
 36052
 
 island
 
 0.011324
 
 0.0104619
 
 Not queried
 
  0.9901445
 
       NA
 
           NA
  
 
  
 11
 
 116451437
 
 116451788
 
  ENSEMBL   UCSC 
 
  352
 
  4
 
 8833
 
 island
 
 0.019022
 
 0.0867069
 
 Not queried
 
  0.9900875
 
       NA
 
           NA
  
 
  
 7
 
  30635712
 
  30635889
 
  ENSEMBL   UCSC 
 
  178
 
  4
 
 36956
 
 gene;island
 
 0.008186
 
 0.0043778
 
 Not queried
 
  0.9900340
 
       NA
 
           NA
  
 
  
 11
 
  31391061
 
  31391307
 
  ENSEMBL   UCSC 
 
  247
 
  8
 
 6552
 
 gene
 
 0.001706
 
 0.0084523
 
 Not queried
 
  0.9900126
 
       NA
 
           NA
  
 
  
 6
 
 169018751
 
 169018877
 
  ENSEMBL   UCSC 
 
  127
 
  4
 
 34564
 
 gene
 
 0.386025
 
 0.5158293
 
 Not queried
 
  0.9899084
 
       NA
 
           NA
  
 
  
 19
 
  16178091
 
  16178253
 
  ENSEMBL   UCSC 
 
  163
 
  6
 
 20566
 
 tss
 
 0.905113
 
 0.9686551
 
 Not queried
 
  0.9899039
 
       NA
 
           NA
  
 
  
 11
 
  16759900
 
  16760091
 
  ENSEMBL   UCSC 
 
  192
 
  9
 
 7170
 
 tss
 
 0.006959
 
 0.0048248
 
 Not queried
 
  0.9898777
 
       NA
 
           NA
  
 
  
 4
 
  54232041
 
  54232638
 
  ENSEMBL   UCSC 
 
  598
 
  7
 
 31047
 
 island
 
 0.005477
 
 0.0097900
 
 Not queried
 
  0.9898631
 
       NA
 
           NA
  
 
  
 12
 
  51610927
 
  51611356
 
  ENSEMBL   UCSC 
 
  430
 
  4
 
 9131
 
 gene;island
 
 0.022453
 
 0.0195363
 
 Not queried
 
  0.9898585
 
       NA
 
           NA
  
 
  
 14
 
 100258973
 
 100259352
 
  ENSEMBL   UCSC 
 
  380
 
  6
 
 12601
 
 tss;island
 
 0.020249
 
 0.0202612
 
 Not queried
 
  0.9897614
 
       NA
 
           NA
  
 
  
 6
 
 146919854
 
 146920129
 
  ENSEMBL   UCSC 
 
  276
 
  8
 
 35277
 
 tss
 
 0.100758
 
 0.0217623
 
 Not queried
 
  0.9896872
 
       NA
 
           NA
  
 
  
 5
 
 173315549
 
 173315978
 
  ENSEMBL   UCSC 
 
  430
 
  5
 
 31906
 
 gene;island
 
 0.006136
 
 0.0080355
 
 Not queried
 
  0.9895740
 
       NA
 
           NA
  
 
  
 3
 
 180707572
 
 180707907
 
  ENSEMBL   UCSC 
 
  336
 
  4
 
 28738
 
 tss
 
 0.004008
 
 0.0046236
 
 Not queried
 
  0.9894664
 
       NA
 
           NA
  
 
  
 15
 
  27111388
 
  27112430
 
  ENSEMBL   UCSC 
 
 1043
 
 24
 
 13965
 
 island
 
 0.028740
 
 0.0765622
 
 Not queried
 
  0.9893061
 
       NA
 
           NA
  
 
  
 12
 
 113415883
 
 113416268
 
  ENSEMBL   UCSC 
 
  386
 
  5
 
 10024
 
 tss
 
 0.010335
 
 0.0121618
 
 Not queried
 
  0.9892519
 
       NA
 
           NA
  
 
  
 13
 
 114840185
 
 114840743
 
  ENSEMBL   UCSC 
 
  559
 
  5
 
 11312
 
 gene;island
 
 0.969006
 
 0.9710980
 
 Not queried
 
  0.9890118
 
       NA
 
           NA
  
 
  
 10
 
    711897
 
    712592
 
  ENSEMBL   UCSC 
 
  696
 
  8
 
 4450
 
 gene;island
 
 0.655962
 
 0.8020992
 
 Not queried
 
  0.9889989
 
       NA
 
           NA
  
 
  
 4
 
 175138377
 
 175138785
 
  ENSEMBL   UCSC 
 
  409
 
  4
 
 31433
 
 island
 
 0.030315
 
 0.1470058
 
 Not queried
 
  0.9887727
 
       NA
 
           NA
  
 
  
 7
 
    194719
 
    194861
 
  ENSEMBL   UCSC 
 
  143
 
  4
 
 36702
 
 gene;island
 
 0.402002
 
 0.5543888
 
 Not queried
 
  0.9885655
 
       NA
 
           NA
  
 
  
 1
 
   3230250
 
   3230647
 
  ENSEMBL   UCSC 
 
  398
 
  5
 
 119
 
 gene
 
 0.198501
 
 0.3717292
 
 Not queried
 
  0.9883845
 
       NA
 
           NA
  
 
  
 7
 
 157956042
 
 157956161
 
  ENSEMBL   UCSC 
 
  120
 
  4
 
 37360
 
 gene;island
 
 0.610325
 
 0.7668638
 
 Not queried
 
  0.9875764
 
       NA
 
           NA
  
 
  
 11
 
  70563580
 
  70563878
 
  ENSEMBL   UCSC 
 
  299
 
  5
 
 6795
 
 gene;island
 
 0.387768
 
 0.3927034
 
 Not queried
 
  0.9874863
 
       NA
 
           NA
  
 
  
 14
 
 105190158
 
 105190481
 
  ENSEMBL   UCSC 
 
  324
 
  5
 
 12661
 
 tss
 
 0.010956
 
 0.0046313
 
 Not queried
 
  0.9874210
 
       NA
 
           NA
  
 
  
 1
 
   1334791
 
   1334814
 
  ENSEMBL   UCSC 
 
   24
 
  4
 
 1149
 
 tss;island
 
 0.004679
 
 0.0049610
 
 Not queried
 
  0.9873276
 
       NA
 
           NA
  
 
  
 17
 
  36956264
 
  36956474
 
  ENSEMBL   UCSC 
 
  211
 
  4
 
 17297
 
 tss
 
 0.009129
 
 0.0146532
 
 Not queried
 
  0.9872752
 
       NA
 
           NA
  
 
  
 4
 
 148401661
 
 148402128
 
  ENSEMBL   UCSC 
 
  468
 
  6
 
 31354
 
 island
 
 0.272660
 
 0.5020908
 
 Not queried
 
  0.9872576
 
       NA
 
           NA
  
 
  
 9
 
  90439478
 
  90440066
 
  ENSEMBL   UCSC 
 
  589
 
  5
 
 40904
 
 island
 
 0.863091
 
 0.8324319
 
 Not queried
 
  0.9872554
 
       NA
 
           NA
  
 
  
 7
 
   4264980
 
   4265548
 
  ENSEMBL   UCSC 
 
  569
 
  6
 
 36836
 
 gene
 
 0.493116
 
 0.6220469
 
 Not queried
 
  0.9871953
 
       NA
 
           NA
  
 
  
 8
 
  29210580
 
  29210800
 
  ENSEMBL   UCSC 
 
  221
 
  4
 
 40202
 
 island
 
 0.057936
 
 0.2813327
 
 Not queried
 
  0.9871778
 
       NA
 
           NA
  
 
  
 7
 
 145813413
 
 145813494
 
  ENSEMBL   UCSC 
 
   82
 
  7
 
 38981
 
 island
 
 0.032266
 
 0.1224886
 
 Not queried
 
  0.9871641
 
       NA
 
           NA
  
 
  
 2
 
 201450323
 
 201450690
 
  ENSEMBL   UCSC 
 
  368
 
  9
 
 23810
 
 tss
 
 0.026589
 
 0.0185010
 
 Not queried
 
  0.9871266
 
       NA
 
           NA
  
 
  
 7
 
   1451136
 
   1451332
 
  ENSEMBL   UCSC 
 
  197
 
  4
 
 38103
 
 island
 
 0.852179
 
 0.9441097
 
 Not queried
 
  0.9871140
 
       NA
 
           NA
  
 
  
 4
 
 187984412
 
 187984718
 
  ENSEMBL   UCSC 
 
  307
 
  4
 
 31488
 
 island
 
 0.173499
 
 0.3405858
 
 Not queried
 
  0.9868304
 
       NA
 
           NA
  
 
  
 12
 
 105478329
 
 105478590
 
  ENSEMBL   UCSC 
 
  262
 
  8
 
 10742
 
 island
 
 0.016002
 
 0.0152400
 
 Not queried
 
  0.9868143
 
       NA
 
           NA
  
 
  
 16
 
  28550525
 
  28550637
 
  ENSEMBL   UCSC 
 
  113
 
  4
 
 15155
 
 tss
 
 0.560713
 
 0.7546283
 
 Not queried
 
  0.9866234
 
       NA
 
           NA
  
 
  
 1
 
 241682884
 
 241683161
 
  ENSEMBL   UCSC 
 
  278
 
  5
 
 4378
 
 island
 
 0.005567
 
 0.0028942
 
 Not queried
 
  0.9865670
 
       NA
 
           NA
  
 
  
 16
 
   2097210
 
   2097837
 
  ENSEMBL   UCSC 
 
  628
 
  6
 
 14595
 
 gene
 
 0.005845
 
 0.0097957
 
 Not queried
 
  0.9862687
 
       NA
 
           NA
  
 
  
 22
 
  18632404
 
  18633123
 
  ENSEMBL   UCSC 
 
  720
 
 10
 
 27285
 
 island
 
 0.010718
 
 0.1066376
 
 Not queried
 
  0.9862079
 
       NA
 
           NA
  
 
  
 15
 
  88799523
 
  88799999
 
  ENSEMBL   UCSC 
 
  477
 
  6
 
 14416
 
 island
 
 0.008587
 
 0.0183118
 
 Not queried
 
  0.9861869
 
       NA
 
           NA
  
 
  
 4
 
 164264796
 
 164265012
 
  ENSEMBL   UCSC 
 
  217
 
  7
 
 30661
 
 tss;island
 
 0.023835
 
 0.0275770
 
 Not queried
 
  0.9861458
 
       NA
 
           NA
  
 
  
 1
 
 198828157
 
 198828249
 
  ENSEMBL   UCSC 
 
   93
 
  4
 
 2159
 
 tss
 
 0.969098
 
 0.9733893
 
 Not queried
 
  0.9861308
 
       NA
 
           NA
  
 
  
 16
 
  14397576
 
  14397892
 
  ENSEMBL   UCSC 
 
  317
 
  6
 
 15721
 
 island
 
 0.021487
 
 0.0070686
 
 Not queried
 
  0.9860576
 
       NA
 
           NA
  
 
  
 12
 
  69080752
 
  69081084
 
  ENSEMBL   UCSC 
 
  333
 
  4
 
 9209
 
 gene
 
 0.006539
 
 0.0025509
 
 Not queried
 
  0.9859427
 
       NA
 
           NA
  
 
  
 10
 
 103329853
 
 103330164
 
  ENSEMBL   UCSC 
 
  312
 
  4
 
 6069
 
 island
 
 0.027838
 
 0.0242841
 
 Not queried
 
  0.9858659
 
       NA
 
           NA
  
 
  
 1
 
 161493932
 
 161494110
 
  ENSEMBL   UCSC 
 
  179
 
  4
 
 3909
 
 island
 
 0.107533
 
 0.1116441
 
 Not queried
 
  0.9858267
 
       NA
 
           NA
  
 
  
 19
 
  53970585
 
  53970942
 
  ENSEMBL   UCSC 
 
  358
 
  7
 
 21029
 
 tss
 
 0.009833
 
 0.0138466
 
 Not queried
 
  0.9857484
 
       NA
 
           NA
  
 
  
 12
 
 117593259
 
 117593728
 
  ENSEMBL   UCSC 
 
  470
 
  7
 
 9336
 
 gene;island
 
 0.968123
 
 0.9540069
 
 Not queried
 
  0.9854096
 
       NA
 
           NA
  
 
  
 7
 
  96653617
 
  96654198
 
  ENSEMBL   UCSC 
 
  582
 
  9
 
 38696
 
 island
 
 0.022917
 
 0.1011383
 
 Not queried
 
  0.9853756
 
       NA
 
           NA
  
 
  
 2
 
 209271164
 
 209272057
 
  ENSEMBL   UCSC 
 
  894
 
 10
 
 25050
 
 island
 
 0.045160
 
 0.1085673
 
 Not queried
 
  0.9853438
 
       NA
 
           NA
  
 
  
 19
 
  10226304
 
  10226701
 
  ENSEMBL   UCSC 
 
  398
 
  5
 
 19810
 
 gene;island
 
 0.939633
 
 0.9632810
 
 Not queried
 
  0.9851059
 
       NA
 
           NA
  
 
  
 6
 
  30951084
 
  30951394
 
  ENSEMBL   UCSC 
 
  311
 
  4
 
 34796
 
 tss
 
 0.508472
 
 0.6960324
 
 Not queried
 
  0.9849941
 
       NA
 
           NA
  
 
  
 10
 
  44101496
 
  44101845
 
  ENSEMBL   UCSC 
 
  350
 
  9
 
 5053
 
 tss
 
 0.111373
 
 0.4486258
 
 Not queried
 
  0.9848932
 
       NA
 
           NA
  
 
  
 16
 
    966362
 
    966659
 
  ENSEMBL   UCSC 
 
  298
 
  4
 
 14553
 
 gene;island
 
 0.729730
 
 0.8505042
 
 Not queried
 
  0.9847571
 
       NA
 
           NA
  
 
  
 1
 
 161171211
 
 161171902
 
  ENSEMBL   UCSC 
 
  692
 
  8
 
 2053
 
 tss
 
 0.191572
 
 0.4078206
 
 Not queried
 
  0.9845739
 
       NA
 
           NA
  
 
  
 11
 
  17566011
 
  17566163
 
  ENSEMBL   UCSC 
 
  153
 
  6
 
 7177
 
 tss
 
 0.178097
 
 0.2876768
 
 Not queried
 
  0.9843360
 
       NA
 
           NA
  
 
  
 17
 
  39728362
 
  39728628
 
  ENSEMBL   UCSC 
 
  267
 
  5
 
 17340
 
 tss
 
 0.253062
 
 0.3098210
 
 Not queried
 
  0.9843102
 
       NA
 
           NA
  
 
  
 6
 
  31431002
 
  31431100
 
  ENSEMBL   UCSC 
 
   99
 
  4
 
 33876
 
 gene
 
 0.010308
 
 0.0188753
 
 Not queried
 
  0.9842582
 
       NA
 
           NA
  
 
  
 6
 
 101847318
 
 101847706
 
  ENSEMBL   UCSC 
 
  389
 
  6
 
 34433
 
 gene;island
 
 0.049432
 
 0.1172447
 
 Not queried
 
  0.9841752
 
       NA
 
           NA
  
 
  
 5
 
 122372722
 
 122372942
 
  ENSEMBL   UCSC 
 
  221
 
  5
 
 32247
 
 tss;island
 
 0.014436
 
 0.0103920
 
 Not queried
 
  0.9837708
 
       NA
 
           NA
  
 
  
 4
 
 170678860
 
 170679294
 
  ENSEMBL   UCSC 
 
  435
 
 11
 
 31417
 
 island
 
 0.006125
 
 0.0051142
 
 Not queried
 
  0.9835690
 
       NA
 
           NA
  
 
  
 12
 
  80328999
 
  80329299
 
  ENSEMBL   UCSC 
 
  301
 
  5
 
 9893
 
 tss
 
 0.009736
 
 0.0163046
 
 Not queried
 
  0.9835643
 
       NA
 
           NA
  
 
  
 13
 
  95364333
 
  95364675
 
  ENSEMBL   UCSC 
 
  343
 
  7
 
 11822
 
 island
 
 0.113699
 
 0.1679841
 
 Not queried
 
  0.9834799
 
       NA
 
           NA
  
 
  
 2
 
 133173433
 
 133173718
 
  ENSEMBL   UCSC 
 
  286
 
  4
 
 23638
 
 tss;island
 
 0.185343
 
 0.1562429
 
 Not queried
 
  0.9833720
 
       NA
 
           NA
  
 
  
 15
 
  22833149
 
  22833400
 
  ENSEMBL   UCSC 
 
  252
 
  8
 
 13939
 
 island
 
 0.009284
 
 0.0090982
 
 Not queried
 
  0.9833597
 
       NA
 
           NA
  
 
  
 2
 
  54557836
 
  54558089
 
  ENSEMBL   UCSC 
 
  254
 
  7
 
 24300
 
 island
 
 0.013116
 
 0.0123398
 
 Not queried
 
  0.9831800
 
       NA
 
           NA
  
 
  
 3
 
 133614782
 
 133615241
 
  ENSEMBL   UCSC 
 
  460
 
  9
 
 28604
 
 tss;island
 
 0.028072
 
 0.0227564
 
 Not queried
 
  0.9828263
 
       NA
 
           NA
  
 
  
 12
 
  91572142
 
  91572276
 
  ENSEMBL   UCSC 
 
  135
 
  4
 
 9241
 
 gene
 
 0.188277
 
 0.3618167
 
 Not queried
 
  0.9826442
 
       NA
 
           NA
  
 
  
 11
 
  64360724
 
  64361020
 
  ENSEMBL   UCSC 
 
  297
 
  6
 
 6690
 
 gene
 
 0.632424
 
 0.7081752
 
 Not queried
 
  0.9826033
 
       NA
 
           NA
  
 
  
 1
 
 161171211
 
 161172049
 
  ENSEMBL   UCSC 
 
  839
 
  9
 
 852
 
 gene;island
 
 0.090434
 
 0.3159671
 
 Not queried
 
  0.9824347
 
       NA
 
           NA
  
 
  
 16
 
    734426
 
    734844
 
  ENSEMBL   UCSC 
 
  419
 
  8
 
 15474
 
 island
 
 0.022375
 
 0.0204640
 
 Not queried
 
  0.9822707
 
       NA
 
           NA
  
 
  
 17
 
  79128885
 
  79129078
 
  ENSEMBL   UCSC 
 
  194
 
  5
 
 16898
 
 gene;island
 
 0.788782
 
 0.8803083
 
 Not queried
 
  0.9821968
 
       NA
 
           NA
  
 
  
 3
 
 170303540
 
 170303830
 
  ENSEMBL   UCSC 
 
  291
 
  4
 
 28030
 
 gene
 
 0.087538
 
 0.2347561
 
 Not queried
 
  0.9821107
 
       NA
 
           NA
  
 
  
 6
 
  33041218
 
  33041343
 
  ENSEMBL   UCSC 
 
  126
 
  5
 
 34184
 
 gene
 
 0.003380
 
 0.0094685
 
 Not queried
 
  0.9816855
 
       NA
 
           NA
  
 
  
 10
 
 127464165
 
 127464762
 
  ENSEMBL   UCSC 
 
  598
 
  9
 
 6238
 
 island
 
 0.522724
 
 0.7653521
 
 Not queried
 
  0.9814879
 
       NA
 
           NA
  
 
  
 11
 
 125325970
 
 125326173
 
  ENSEMBL   UCSC 
 
  204
 
  4
 
 6958
 
 gene
 
 0.384870
 
 0.2686341
 
 Not queried
 
  0.9814579
 
       NA
 
           NA
  
 
  
 11
 
   8704225
 
   8704501
 
  ENSEMBL   UCSC 
 
  277
 
  4
 
 6497
 
 gene;island
 
 0.009454
 
 0.0028597
 
 Not queried
 
  0.9812508
 
       NA
 
           NA
  
 
  
 14
 
 103592395
 
 103592734
 
  ENSEMBL   UCSC 
 
  340
 
  4
 
 13182
 
 island
 
 0.792153
 
 0.5409254
 
 Not queried
 
  0.9812159
 
       NA
 
           NA
  
 
  
 11
 
  10673844
 
  10674182
 
  ENSEMBL   UCSC 
 
  339
 
  4
 
 6509
 
 gene
 
 0.882121
 
 0.7990847
 
 Not queried
 
  0.9812113
 
       NA
 
           NA
  
 
  
 4
 
  76555547
 
  76556042
 
  ENSEMBL   UCSC 
 
  496
 
 11
 
 31107
 
 island
 
 0.024022
 
 0.0304886
 
 Not queried
 
  0.9805748
 
       NA
 
           NA
  
 
  
 1
 
 214153294
 
 214153645
 
  ENSEMBL   UCSC 
 
  352
 
  5
 
 4194
 
 island
 
 0.039676
 
 0.0775437
 
 Not queried
 
  0.9804860
 
       NA
 
           NA
  
 
  
 19
 
   3730505
 
   3730768
 
  ENSEMBL   UCSC 
 
  264
 
  4
 
 19722
 
 gene;island
 
 0.649756
 
 0.9191472
 
 Not queried
 
  0.9803910
 
       NA
 
           NA
  
 
  
 6
 
  31765567
 
  31765634
 
  ENSEMBL   UCSC 
 
   68
 
  5
 
 33960
 
 gene;island
 
 0.057223
 
 0.0324736
 
 Not queried
 
  0.9803549
 
       NA
 
           NA
  
 
  
 10
 
 129845375
 
 129845766
 
  ENSEMBL   UCSC 
 
  392
 
  6
 
 5438
 
 tss
 
 0.035862
 
 0.0484241
 
 Not queried
 
  0.9800868
 
       NA
 
           NA
  
 
  
 5
 
 140235435
 
 140236081
 
  ENSEMBL   UCSC 
 
  647
 
  9
 
 31800
 
 gene;island
 
 0.190045
 
 0.2838954
 
 Not queried
 
  0.9797996
 
       NA
 
           NA
  
 
  
 6
 
  71666682
 
  71666858
 
  ENSEMBL   UCSC 
 
  177
 
  5
 
 36242
 
 island
 
 0.026048
 
 0.0270785
 
 Not queried
 
  0.9794186
 
       NA
 
           NA
  
 
  
 1
 
  32110477
 
  32110960
 
  ENSEMBL   UCSC 
 
  484
 
  9
 
 1449
 
 tss
 
 0.001831
 
 0.0070734
 
 Not queried
 
  0.9792992
 
       NA
 
           NA
  
 
  
 18
 
  21719263
 
  21719568
 
  ENSEMBL   UCSC 
 
  306
 
  7
 
 19077
 
 gene;island
 
 0.015331
 
 0.0431270
 
 Not queried
 
  0.9791013
 
       NA
 
           NA
  
 
  
 22
 
  35937058
 
  35937319
 
  ENSEMBL   UCSC 
 
  262
 
  6
 
 27100
 
 tss
 
 0.009853
 
 0.0204073
 
 Not queried
 
  0.9790963
 
       NA
 
           NA
  
 
  
 22
 
  35937058
 
  35937531
 
  ENSEMBL   UCSC 
 
  474
 
  8
 
 27448
 
 island
 
 0.010101
 
 0.0233732
 
 Not queried
 
  0.9790963
 
       NA
 
           NA
  
 
  
 17
 
  21454528
 
  21455116
 
  ENSEMBL   UCSC 
 
  589
 
  6
 
 18125
 
 island
 
 0.065631
 
 0.0880868
 
 Not queried
 
  0.9790454
 
       NA
 
           NA
  
 
  
 1
 
  11795746
 
  11796297
 
  ENSEMBL   UCSC 
 
  552
 
 13
 
 2687
 
 island
 
 0.016924
 
 0.0178249
 
 Not queried
 
  0.9787761
 
       NA
 
           NA
  
 
  
 7
 
  12443478
 
  12444290
 
  ENSEMBL   UCSC 
 
  813
 
 11
 
 38266
 
 island
 
 0.026076
 
 0.0177188
 
 Not queried
 
  0.9785652
 
       NA
 
           NA
  
 
  
 2
 
 175197240
 
 175198039
 
  ENSEMBL   UCSC 
 
  800
 
  6
 
 24873
 
 island
 
 0.125405
 
 0.2750564
 
 Not queried
 
  0.9783467
 
       NA
 
           NA
  
 
  
 11
 
 108092087
 
 108092453
 
  ENSEMBL   UCSC 
 
  367
 
 10
 
 6885
 
 gene;tss;island
 
 0.003159
 
 0.0030488
 
 Not queried
 
  0.9782293
 
       NA
 
           NA
  
 
  
 5
 
 140207460
 
 140208201
 
  ENSEMBL   UCSC 
 
  742
 
  9
 
 31797
 
 gene;island
 
 0.561976
 
 0.6215940
 
 Not queried
 
  0.9781316
 
       NA
 
           NA
  
 
  
 16
 
  10837597
 
  10837690
 
  ENSEMBL   UCSC 
 
   94
 
  6
 
 15088
 
 tss
 
 0.005415
 
 0.0039144
 
 Not queried
 
  0.9779166
 
       NA
 
           NA
  
 
  
 16
 
  15737176
 
  15737314
 
  ENSEMBL   UCSC 
 
  139
 
  4
 
 14677
 
 gene;tss
 
 0.001462
 
 0.0031162
 
 Not queried
 
  0.9779153
 
       NA
 
           NA
  
 
  
 2
 
   1417109
 
   1417431
 
  ENSEMBL   UCSC 
 
  323
 
  5
 
 23157
 
 tss
 
 0.073074
 
 0.2187296
 
 Not queried
 
  0.9779126
 
       NA
 
           NA
  
 
  
 1
 
 236925843
 
 236925965
 
  ENSEMBL   UCSC 
 
  123
 
  4
 
 1101
 
 gene
 
 0.949895
 
 0.9704109
 
 Not queried
 
  0.9778747
 
       NA
 
           NA
  
 
  
 10
 
  54074040
 
  54074250
 
  ENSEMBL   UCSC 
 
  211
 
  4
 
 4593
 
 gene
 
 0.104158
 
 0.0568662
 
 Not queried
 
  0.9778410
 
       NA
 
           NA
  
 
  
 2
 
 111878294
 
 111878481
 
  ENSEMBL   UCSC 
 
  188
 
  6
 
 23572
 
 tss;island
 
 0.013408
 
 0.0198525
 
 Not queried
 
  0.9777250
 
       NA
 
           NA
  
 
  
 20
 
  21106483
 
  21106763
 
  ENSEMBL   UCSC 
 
  281
 
  6
 
 26038
 
 island
 
 0.006542
 
 0.0054273
 
 Not queried
 
  0.9776542
 
       NA
 
           NA
  
 
  
 19
 
  16771653
 
  16771949
 
  ENSEMBL   UCSC 
 
  297
 
  4
 
 20574
 
 tss;island
 
 0.009362
 
 0.0052541
 
 Not queried
 
  0.9775780
 
       NA
 
           NA
  
 
  
 7
 
  44836797
 
  44837140
 
  ENSEMBL   UCSC 
 
  344
 
  5
 
 36995
 
 gene;island
 
 0.007272
 
 0.0035074
 
 Not queried
 
  0.9774682
 
       NA
 
           NA
  
 
  
 2
 
  54483431
 
  54483817
 
  ENSEMBL   UCSC 
 
  387
 
  5
 
 23354
 
 tss
 
 0.895682
 
 0.9630693
 
 Not queried
 
  0.9773623
 
       NA
 
           NA
  
 
  
 8
 
 143915776
 
 143916377
 
  ENSEMBL   UCSC 
 
  602
 
 10
 
 40646
 
 island
 
 0.588400
 
 0.7897805
 
 Not queried
 
  0.9772365
 
       NA
 
           NA
  
 
  
 19
 
   7069182
 
   7069348
 
  ENSEMBL   UCSC 
 
  167
 
  4
 
 20395
 
 tss
 
 0.054178
 
 0.0277009
 
 Not queried
 
  0.9771542
 
       NA
 
           NA
  
 
  
 2
 
 162930175
 
 162930454
 
  ENSEMBL   UCSC 
 
  280
 
  4
 
 22910
 
 gene;island
 
 0.012434
 
 0.0122274
 
 Not queried
 
  0.9771377
 
       NA
 
           NA
  
 
  
 8
 
  21899862
 
  21900337
 
  ENSEMBL   UCSC 
 
  476
 
  6
 
 39579
 
 tss
 
 0.057272
 
 0.0257577
 
 Not queried
 
  0.9768867
 
       NA
 
           NA
  
 
  
 19
 
   3178742
 
   3178955
 
  ENSEMBL   UCSC 
 
  214
 
  4
 
 21316
 
 island
 
 0.008722
 
 0.0051538
 
 Not queried
 
  0.9768810
 
       NA
 
           NA
  
 
  
 14
 
  88792913
 
  88793311
 
  ENSEMBL   UCSC 
 
  399
 
  8
 
 13057
 
 island
 
 0.078324
 
 0.0850560
 
 Not queried
 
  0.9767221
 
       NA
 
           NA
  
 
  
 16
 
  68676451
 
  68676806
 
  ENSEMBL   UCSC 
 
  356
 
  5
 
 16049
 
 island
 
 0.237249
 
 0.0701122
 
 Not queried
 
  0.9765201
 
       NA
 
           NA
  
 
  
 20
 
  56286391
 
  56286732
 
  ENSEMBL   UCSC 
 
  342
 
  7
 
 25846
 
 tss;island
 
 0.422694
 
 0.5453922
 
 Not queried
 
  0.9764389
 
       NA
 
           NA
  
 
  
 15
 
  45815087
 
  45815405
 
  ENSEMBL   UCSC 
 
  319
 
  7
 
 13334
 
 gene;tss;island
 
 0.047446
 
 0.0184721
 
 Not queried
 
  0.9763687
 
       NA
 
           NA
  
 
  
 19
 
  13262683
 
  13263065
 
  ENSEMBL   UCSC 
 
  383
 
  4
 
 19879
 
 gene;island
 
 0.010640
 
 0.0040544
 
 Not queried
 
  0.9763372
 
       NA
 
           NA
  
 
  
 1
 
 201951076
 
 201951606
 
  ENSEMBL   UCSC 
 
  531
 
  6
 
 2172
 
 tss;island
 
 0.229003
 
 0.5154780
 
 Not queried
 
  0.9762833
 
       NA
 
           NA
  
 
  
 11
 
  43701985
 
  43702128
 
  ENSEMBL   UCSC 
 
  144
 
  5
 
 7255
 
 tss
 
 0.002574
 
 0.0015659
 
 Not queried
 
  0.9760614
 
       NA
 
           NA
  
 
  
 4
 
  42400738
 
  42401242
 
  ENSEMBL   UCSC 
 
  505
 
  6
 
 29994
 
 gene;island
 
 0.011471
 
 0.0495895
 
 Not queried
 
  0.9758362
 
       NA
 
           NA
  
 
  
 19
 
  44952417
 
  44952808
 
  ENSEMBL   UCSC 
 
  392
 
 10
 
 22109
 
 island
 
 0.029613
 
 0.1263621
 
 Not queried
 
  0.9756454
 
       NA
 
           NA
  
 
  
 6
 
  32036179
 
  32038230
 
  ENSEMBL   UCSC 
 
 2052
 
 47
 
 34029
 
 gene
 
 0.444290
 
 0.5267575
 
 Not queried
 
  0.9755394
 
       NA
 
           NA
  
 
  
 19
 
  11564442
 
  11564539
 
  ENSEMBL   UCSC 
 
   98
 
  4
 
 19842
 
 gene;island
 
 0.928503
 
 0.9199424
 
 Not queried
 
  0.9755370
 
       NA
 
           NA
  
 
  
 1
 
  68698376
 
  68698718
 
  ENSEMBL   UCSC 
 
  343
 
  7
 
 1688
 
 tss;island
 
 0.015337
 
 0.0078342
 
 Not queried
 
  0.9751331
 
       NA
 
           NA
  
 
  
 2
 
 128185947
 
 128186258
 
  ENSEMBL   UCSC 
 
  312
 
  4
 
 22858
 
 gene;island
 
 0.855159
 
 0.9252200
 
 Not queried
 
  0.9750895
 
       NA
 
           NA
  
 
  
 6
 
  29759718
 
  29760164
 
  ENSEMBL   UCSC 
 
  447
 
 14
 
 33693
 
 gene;island
 
 0.070711
 
 0.1445902
 
 Not queried
 
  0.9748154
 
       NA
 
           NA
  
 
  
 13
 
 113759771
 
 113760286
 
  ENSEMBL   UCSC 
 
  516
 
  6
 
 11956
 
 island
 
 0.559282
 
 0.7772820
 
 Not queried
 
  0.9747884
 
       NA
 
           NA
  
 
  
 14
 
 100203942
 
 100204258
 
  ENSEMBL   UCSC 
 
  317
 
  6
 
 13130
 
 island
 
 0.063305
 
 0.0301071
 
 Not queried
 
  0.9747197
 
       NA
 
           NA
  
 
  
 5
 
 179499304
 
 179499488
 
  ENSEMBL   UCSC 
 
  185
 
  4
 
 32558
 
 tss
 
 0.103946
 
 0.1001023
 
 Not queried
 
  0.9744718
 
       NA
 
           NA
  
 
  
 14
 
  96967798
 
  96968050
 
  ENSEMBL   UCSC 
 
  253
 
  4
 
 12595
 
 tss;island
 
 0.015092
 
 0.0036812
 
 Not queried
 
  0.9743908
 
       NA
 
           NA
  
 
  
 1
 
 165513253
 
 165513361
 
  ENSEMBL   UCSC 
 
  109
 
  5
 
 864
 
 gene;tss
 
 0.467444
 
 0.0318525
 
 Not queried
 
  0.9741579
 
       NA
 
           NA
  
 
  
 5
 
  87980882
 
  87981449
 
  ENSEMBL   UCSC 
 
  568
 
  7
 
 32168
 
 tss;island
 
 0.516548
 
 0.2273698
 
 Not queried
 
  0.9739507
 
       NA
 
           NA
  
 
  
 15
 
  71184279
 
  71184982
 
  ENSEMBL   UCSC 
 
  704
 
 11
 
 13769
 
 tss;island
 
 0.017123
 
 0.0111903
 
 Not queried
 
  0.9738627
 
       NA
 
           NA
  
 
  
 19
 
  50379799
 
  50380022
 
  ENSEMBL   UCSC 
 
  224
 
  4
 
 20167
 
 gene;tss;island
 
 0.004559
 
 0.0023932
 
 Not queried
 
  0.9735999
 
       NA
 
           NA
  
 
  
 14
 
  74318162
 
  74318606
 
  ENSEMBL   UCSC 
 
  445
 
 10
 
 12501
 
 tss
 
 0.012651
 
 0.0108586
 
 Not queried
 
  0.9733321
 
       NA
 
           NA
  
 
  
 6
 
  86158972
 
  86159426
 
  ENSEMBL   UCSC 
 
  455
 
  7
 
 36300
 
 island
 
 0.031727
 
 0.0415572
 
 Not queried
 
  0.9732670
 
       NA
 
           NA
  
 
  
 6
 
 170863287
 
 170863845
 
  ENSEMBL   UCSC 
 
  559
 
 12
 
 35358
 
 tss
 
 0.007176
 
 0.0031477
 
 Not queried
 
  0.9731895
 
       NA
 
           NA
  
 
  
 1
 
 149149314
 
 149149465
 
  ENSEMBL   UCSC 
 
  152
 
  4
 
 3705
 
 island
 
 0.355941
 
 0.2287780
 
 Not queried
 
  0.9730671
 
       NA
 
           NA
  
 
  
 4
 
    967432
 
    967541
 
  ENSEMBL   UCSC 
 
  110
 
  6
 
 30232
 
 tss
 
 0.004219
 
 0.0066674
 
 Not queried
 
  0.9730568
 
       NA
 
           NA
  
 
  
 4
 
    967324
 
    967541
 
  ENSEMBL   UCSC 
 
  218
 
  8
 
 30750
 
 island
 
 0.004219
 
 0.0066674
 
 Not queried
 
  0.9730568
 
       NA
 
           NA
  
 
  
 7
 
  27291346
 
  27291695
 
  ENSEMBL   UCSC 
 
  350
 
  4
 
 38367
 
 island
 
 0.032982
 
 0.0633655
 
 Not queried
 
  0.9729045
 
       NA
 
           NA
  
 
  
 2
 
 173600362
 
 173600676
 
  ENSEMBL   UCSC 
 
  315
 
  4
 
 22926
 
 gene;island
 
 0.033393
 
 0.0325852
 
 Not queried
 
  0.9728991
 
       NA
 
           NA
  
 
  
 3
 
 190040223
 
 190040375
 
  ENSEMBL   UCSC 
 
  153
 
  4
 
 28784
 
 tss
 
 0.029898
 
 0.0153470
 
 Not queried
 
  0.9727830
 
       NA
 
           NA
  
 
  
 15
 
  63570099
 
  63570580
 
  ENSEMBL   UCSC 
 
  482
 
  5
 
 13374
 
 gene;island
 
 0.008616
 
 0.0040667
 
 Not queried
 
  0.9727550
 
       NA
 
           NA
  
 
  
 1
 
 110880366
 
 110880555
 
  ENSEMBL   UCSC 
 
  190
 
  4
 
 3578
 
 island
 
 0.005462
 
 0.0023090
 
 Not queried
 
  0.9725870
 
       NA
 
           NA
  
 
  
 12
 
    322514
 
    322983
 
  ENSEMBL   UCSC 
 
  470
 
 10
 
 8991
 
 gene;tss
 
 0.184794
 
 0.3330706
 
 Not queried
 
  0.9724901
 
       NA
 
           NA
  
 
  
 1
 
    990293
 
    990557
 
  ENSEMBL   UCSC 
 
  265
 
  4
 
 11
 
 gene;island
 
 0.925842
 
 0.9632498
 
 Not queried
 
  0.9724573
 
       NA
 
           NA
  
 
  
 6
 
 149067627
 
 149067875
 
  ENSEMBL   UCSC 
 
  249
 
  4
 
 35281
 
 tss;island
 
 0.046816
 
 0.0617426
 
 Not queried
 
  0.9723564
 
       NA
 
           NA
  
 
  
 5
 
 135170000
 
 135170281
 
  ENSEMBL   UCSC 
 
  282
 
  7
 
 32307
 
 tss
 
 0.036145
 
 0.1303543
 
 Not queried
 
  0.9721243
 
       NA
 
           NA
  
 
  
 11
 
  27744363
 
  27744816
 
  ENSEMBL   UCSC 
 
  454
 
  6
 
 7216
 
 tss;island
 
 0.035533
 
 0.0306481
 
 Not queried
 
  0.9718314
 
       NA
 
           NA
  
 
  
 14
 
  55595666
 
  55595958
 
  ENSEMBL   UCSC 
 
  293
 
  5
 
 12864
 
 island
 
 0.007705
 
 0.0031965
 
 Not queried
 
  0.9717455
 
       NA
 
           NA
  
 
  
 17
 
  36507744
 
  36507992
 
  ENSEMBL   UCSC 
 
  249
 
  4
 
 17292
 
 tss
 
 0.009300
 
 0.0077290
 
 Not queried
 
  0.9717364
 
       NA
 
           NA
  
 
  
 14
 
  55226964
 
  55227154
 
  ENSEMBL   UCSC 
 
  191
 
  4
 
 12084
 
 gene;island
 
 0.806162
 
 0.8942195
 
 Not queried
 
  0.9716694
 
       NA
 
           NA
  
 
  
 7
 
 151722461
 
 151722628
 
  ENSEMBL   UCSC 
 
  168
 
  7
 
 38004
 
 tss
 
 0.017764
 
 0.0662909
 
 Not queried
 
  0.9714744
 
       NA
 
           NA
  
 
  
 20
 
    524484
 
    524791
 
  ENSEMBL   UCSC 
 
  308
 
  8
 
 25527
 
 tss
 
 0.001683
 
 0.0008986
 
 Not queried
 
  0.9709955
 
       NA
 
           NA
  
 
  
 7
 
 129845338
 
 129845597
 
  ENSEMBL   UCSC 
 
  260
 
 10
 
 37897
 
 tss
 
 0.009494
 
 0.0065276
 
 Not queried
 
  0.9708068
 
       NA
 
           NA
  
 
  
 7
 
 129845202
 
 129845597
 
  ENSEMBL   UCSC 
 
  396
 
 12
 
 38917
 
 island
 
 0.007341
 
 0.0038756
 
 Not queried
 
  0.9708068
 
       NA
 
           NA
  
 
  
 2
 
  45838438
 
  45838581
 
  ENSEMBL   UCSC 
 
  144
 
  4
 
 23336
 
 tss
 
 0.002804
 
 0.0010462
 
 Not queried
 
  0.9707433
 
       NA
 
           NA
  
 
  
 19
 
  58011309
 
  58011582
 
  ENSEMBL   UCSC 
 
  274
 
  4
 
 20247
 
 gene
 
 0.005848
 
 0.0039356
 
 Not queried
 
  0.9707297
 
       NA
 
           NA
  
 
  
 6
 
  31504181
 
  31504388
 
  ENSEMBL   UCSC 
 
  208
 
  4
 
 33891
 
 gene
 
 0.963746
 
 0.9206972
 
 Not queried
 
  0.9706927
 
       NA
 
           NA
  
 
  
 2
 
   3683030
 
   3683283
 
  ENSEMBL   UCSC 
 
  254
 
  4
 
 22554
 
 gene;island
 
 0.341639
 
 0.5025627
 
 Not queried
 
  0.9706513
 
       NA
 
           NA
  
 
  
 14
 
  32546448
 
  32546633
 
  ENSEMBL   UCSC 
 
  186
 
  4
 
 12344
 
 tss;island
 
 0.012620
 
 0.0030473
 
 Not queried
 
  0.9703272
 
       NA
 
           NA
  
 
  
 12
 
   3281520
 
   3281841
 
  ENSEMBL   UCSC 
 
  322
 
  4
 
 9010
 
 gene
 
 0.767637
 
 0.8662033
 
 Not queried
 
  0.9701632
 
       NA
 
           NA
  
 
  
 4
 
  47839876
 
  47840162
 
  ENSEMBL   UCSC 
 
  287
 
  6
 
 31025
 
 island
 
 0.024951
 
 0.0078332
 
 Not queried
 
  0.9700335
 
       NA
 
           NA
  
 
  
 11
 
  69061244
 
  69061473
 
  ENSEMBL   UCSC 
 
  230
 
  4
 
 7518
 
 tss
 
 0.609774
 
 0.4189669
 
 Not queried
 
  0.9700067
 
       NA
 
           NA
  
 
  
 11
 
 126225941
 
 126226154
 
  ENSEMBL   UCSC 
 
  214
 
  7
 
 8934
 
 island
 
 0.035474
 
 0.0188484
 
 Not queried
 
  0.9698100
 
       NA
 
           NA
  
 
  
 6
 
  50813551
 
  50814089
 
  ENSEMBL   UCSC 
 
  539
 
  5
 
 34368
 
 gene;island
 
 0.070129
 
 0.0260648
 
 Not queried
 
  0.9697553
 
       NA
 
           NA
  
 
  
 10
 
 131354782
 
 131355302
 
  ENSEMBL   UCSC 
 
  521
 
  9
 
 4826
 
 gene
 
 0.701386
 
 0.8990631
 
 Not queried
 
  0.9695740
 
       NA
 
           NA
  
 
  
 17
 
  72357992
 
  72358370
 
  ENSEMBL   UCSC 
 
  379
 
  5
 
 17624
 
 tss;island
 
 0.384227
 
 0.4308034
 
 Not queried
 
  0.9695061
 
       NA
 
           NA
  
 
  
 15
 
  66796922
 
  66797164
 
  ENSEMBL   UCSC 
 
  243
 
  4
 
 13390
 
 gene
 
 0.006591
 
 0.0029670
 
 Not queried
 
  0.9694219
 
       NA
 
           NA
  
 
  
 16
 
  83841324
 
  83841917
 
  ENSEMBL   UCSC 
 
  594
 
  8
 
 16131
 
 island
 
 0.005847
 
 0.0079598
 
 Not queried
 
  0.9691812
 
       NA
 
           NA
  
 
  
 2
 
   3383078
 
   3383413
 
  ENSEMBL   UCSC 
 
  336
 
  5
 
 23160
 
 tss
 
 0.014676
 
 0.0059351
 
 Not queried
 
  0.9691525
 
       NA
 
           NA
  
 
  
 8
 
  74005537
 
  74005813
 
  ENSEMBL   UCSC 
 
  277
 
  8
 
 39767
 
 tss;island
 
 0.035433
 
 0.0181433
 
 Not queried
 
  0.9690757
 
       NA
 
           NA
  
 
  
 2
 
  71680559
 
  71680721
 
  ENSEMBL   UCSC 
 
  163
 
  4
 
 23430
 
 tss
 
 0.069571
 
 0.0496999
 
 Not queried
 
  0.9686394
 
       NA
 
           NA
  
 
  
 6
 
  31111076
 
  31111270
 
  ENSEMBL   UCSC 
 
  195
 
  4
 
 33855
 
 gene
 
 0.910377
 
 0.7583920
 
 Not queried
 
  0.9685953
 
       NA
 
           NA
  
 
  
 6
 
  99872786
 
  99872940
 
  ENSEMBL   UCSC 
 
  155
 
  7
 
 34424
 
 gene;island
 
 0.002889
 
 0.0082269
 
 Not queried
 
  0.9685306
 
       NA
 
           NA
  
 
  
 11
 
  65383415
 
  65383706
 
  ENSEMBL   UCSC 
 
  292
 
  4
 
 7436
 
 tss
 
 0.004714
 
 0.0045326
 
 Not queried
 
  0.9685136
 
       NA
 
           NA
  
 
  
 4
 
  41875470
 
  41875875
 
  ENSEMBL   UCSC 
 
  406
 
  4
 
 31007
 
 island
 
 0.186160
 
 0.2681277
 
 Not queried
 
  0.9685013
 
       NA
 
           NA
  
 
  
 7
 
  96746798
 
  96747063
 
  ENSEMBL   UCSC 
 
  266
 
  4
 
 37108
 
 gene;island
 
 0.007133
 
 0.0035109
 
 Not queried
 
  0.9684636
 
       NA
 
           NA
  
 
  
 1
 
  22109708
 
  22109947
 
  ENSEMBL   UCSC 
 
  240
 
  6
 
 1327
 
 tss
 
 0.004171
 
 0.0028152
 
 Not queried
 
  0.9682759
 
       NA
 
           NA
  
 
  
 2
 
 175351869
 
 175351956
 
  ENSEMBL   UCSC 
 
   88
 
  6
 
 23725
 
 tss
 
 0.009860
 
 0.0155172
 
 Not queried
 
  0.9682354
 
       NA
 
           NA
  
 
  
 5
 
 180071709
 
 180071930
 
  ENSEMBL   UCSC 
 
  222
 
  5
 
 31945
 
 gene;island
 
 0.579927
 
 0.6309702
 
 Not queried
 
  0.9682140
 
       NA
 
           NA
  
 
  
 17
 
  32907636
 
  32907997
 
  ENSEMBL   UCSC 
 
  362
 
  7
 
 18202
 
 island
 
 0.094418
 
 0.0361769
 
 Not queried
 
  0.9679705
 
       NA
 
           NA
  
 
  
 6
 
  37673335
 
  37673863
 
  ENSEMBL   UCSC 
 
  529
 
  6
 
 36101
 
 island
 
 0.039240
 
 0.0473378
 
 Not queried
 
  0.9678693
 
       NA
 
           NA
  
 
  
 10
 
 104628846
 
 104629179
 
  ENSEMBL   UCSC 
 
  334
 
  8
 
 5332
 
 tss;island
 
 0.009150
 
 0.0062759
 
 Not queried
 
  0.9674998
 
       NA
 
           NA
  
 
  
 14
 
  73392648
 
  73392937
 
  ENSEMBL   UCSC 
 
  290
 
  7
 
 12492
 
 tss;island
 
 0.006981
 
 0.0060702
 
 Not queried
 
  0.9674337
 
       NA
 
           NA
  
 
  
 13
 
  19692423
 
  19692848
 
  ENSEMBL   UCSC 
 
  426
 
  7
 
 11321
 
 tss
 
 0.234983
 
 0.8656393
 
 Not queried
 
  0.9672301
 
       NA
 
           NA
  
 
  
 11
 
  65743851
 
  65744159
 
  ENSEMBL   UCSC 
 
  309
 
  5
 
 6719
 
 gene;island
 
 0.953892
 
 0.9684481
 
 Not queried
 
  0.9671096
 
       NA
 
           NA
  
 
  
 19
 
  51522454
 
  51522921
 
  ENSEMBL   UCSC 
 
  468
 
  7
 
 20188
 
 gene;island
 
 0.012368
 
 0.0235297
 
 Not queried
 
  0.9669960
 
       NA
 
           NA
  
 
  
 1
 
  25566331
 
  25566753
 
  ENSEMBL   UCSC 
 
  423
 
  5
 
 2868
 
 island
 
 0.001682
 
 0.0016439
 
 Not queried
 
  0.9667813
 
       NA
 
           NA
  
 
  
 7
 
 155166205
 
 155166619
 
  ENSEMBL   UCSC 
 
  415
 
  5
 
 39063
 
 island
 
 0.074542
 
 0.0185632
 
 Not queried
 
  0.9667631
 
       NA
 
           NA
  
 
  
 2
 
 176987161
 
 176987605
 
  ENSEMBL   UCSC 
 
  445
 
  8
 
 24902
 
 island
 
 0.315902
 
 0.2432492
 
 Not queried
 
  0.9667628
 
       NA
 
           NA
  
 
  
 17
 
  75398290
 
  75398583
 
  ENSEMBL   UCSC 
 
  294
 
  4
 
 16807
 
 gene;island
 
 0.966422
 
 0.9817033
 
 Not queried
 
  0.9667515
 
       NA
 
           NA
  
 
  
 4
 
  21699336
 
  21699534
 
  ENSEMBL   UCSC 
 
  199
 
  6
 
 30324
 
 tss
 
 0.160141
 
 0.4445257
 
 Not queried
 
  0.9666252
 
       NA
 
           NA
  
 
  
 17
 
  41176744
 
  41177097
 
  ENSEMBL   UCSC 
 
  354
 
  4
 
 17382
 
 tss;island
 
 0.037735
 
 0.0150069
 
 Not queried
 
  0.9665379
 
       NA
 
           NA
  
 
  
 6
 
  27832728
 
  27833555
 
  ENSEMBL   UCSC 
 
  828
 
 13
 
 35570
 
 island
 
 0.004143
 
 0.0013791
 
 Not queried
 
  0.9663185
 
       NA
 
           NA
  
 
  
 2
 
 236715747
 
 236716372
 
  ENSEMBL   UCSC 
 
  626
 
  6
 
 23081
 
 gene
 
 0.307513
 
 0.4859051
 
 Not queried
 
  0.9661237
 
       NA
 
           NA
  
 
  
 10
 
  43866966
 
  43867071
 
  ENSEMBL   UCSC 
 
  106
 
  5
 
 5046
 
 tss
 
 0.312655
 
 0.4380869
 
 Not queried
 
  0.9661066
 
       NA
 
           NA
  
 
  
 4
 
   3748154
 
   3748347
 
  ENSEMBL   UCSC 
 
  194
 
  4
 
 30853
 
 island
 
 0.664183
 
 0.7396869
 
 Not queried
 
  0.9660501
 
       NA
 
           NA
  
 
  
 6
 
 123317124
 
 123318023
 
  ENSEMBL   UCSC 
 
  900
 
 11
 
 36454
 
 island
 
 0.018938
 
 0.0379922
 
 Not queried
 
  0.9659067
 
       NA
 
           NA
  
 
  
 15
 
  89438611
 
  89438964
 
  ENSEMBL   UCSC 
 
  354
 
  7
 
 14423
 
 island
 
 0.012090
 
 0.0145678
 
 Not queried
 
  0.9658232
 
       NA
 
           NA
  
 
  
 16
 
  29196472
 
  29196699
 
  ENSEMBL   UCSC 
 
  228
 
  4
 
 15800
 
 island
 
 0.751152
 
 0.9301257
 
 Not queried
 
  0.9657765
 
       NA
 
           NA
  
 
  
 2
 
    306606
 
    307058
 
  ENSEMBL   UCSC 
 
  453
 
  4
 
 24001
 
 island
 
 0.724568
 
 0.8224941
 
 Not queried
 
  0.9655993
 
       NA
 
           NA
  
 
  
 15
 
  93447445
 
  93447854
 
  ENSEMBL   UCSC 
 
  410
 
  6
 
 13487
 
 gene;island
 
 0.005967
 
 0.0037753
 
 Not queried
 
  0.9653690
 
       NA
 
           NA
  
 
  
 11
 
   1673436
 
   1674211
 
  ENSEMBL   UCSC 
 
  776
 
 10
 
 6427
 
 gene;island
 
 0.096587
 
 0.1778690
 
 Not queried
 
  0.9652084
 
       NA
 
           NA
  
 
  
 19
 
   3606757
 
   3607269
 
  ENSEMBL   UCSC 
 
  513
 
  8
 
 21337
 
 island
 
 0.079348
 
 0.1529170
 
 Not queried
 
  0.9651042
 
       NA
 
           NA
  
 
  
 11
 
   1862020
 
   1862477
 
  ENSEMBL   UCSC 
 
  458
 
  4
 
 6434
 
 gene
 
 0.971982
 
 0.9579074
 
 Not queried
 
  0.9649518
 
       NA
 
           NA
  
 
  
 7
 
 132937434
 
 132937811
 
  ENSEMBL   UCSC 
 
  378
 
  7
 
 37908
 
 tss
 
 0.005205
 
 0.0058616
 
 Not queried
 
  0.9648241
 
       NA
 
           NA
  
 
  
 1
 
 116694665
 
 116695026
 
  ENSEMBL   UCSC 
 
  362
 
  5
 
 3636
 
 island
 
 0.014993
 
 0.0074852
 
 Not queried
 
  0.9643487
 
       NA
 
           NA
  
 
  
 3
 
 151987182
 
 151987324
 
  ENSEMBL   UCSC 
 
  143
 
  4
 
 27997
 
 gene;island
 
 0.020502
 
 0.0173583
 
 Not queried
 
  0.9642312
 
       NA
 
           NA
  
 
  
 13
 
  41837767
 
  41838093
 
  ENSEMBL   UCSC 
 
  327
 
  7
 
 11411
 
 tss
 
 0.005397
 
 0.0029921
 
 Not queried
 
  0.9641424
 
       NA
 
           NA
  
 
  
 16
 
  81254010
 
  81254209
 
  ENSEMBL   UCSC 
 
  200
 
  4
 
 15380
 
 tss
 
 0.624766
 
 0.7253716
 
 Not queried
 
  0.9640409
 
       NA
 
           NA
  
 
  
 11
 
   2011216
 
   2011346
 
  ENSEMBL   UCSC 
 
  131
 
  6
 
 7052
 
 tss;island
 
 0.435098
 
 0.5484224
 
 Not queried
 
  0.9640356
 
       NA
 
           NA
  
 
  
 4
 
 122744060
 
 122744383
 
  ENSEMBL   UCSC 
 
  324
 
  4
 
 30116
 
 gene;island
 
 0.004154
 
 0.0014670
 
 Not queried
 
  0.9639647
 
       NA
 
           NA
  
 
  
 4
 
 187645059
 
 187645448
 
  ENSEMBL   UCSC 
 
  390
 
  6
 
 30708
 
 tss;island
 
 0.101429
 
 0.0638633
 
 Not queried
 
  0.9639513
 
       NA
 
           NA
  
 
  
 5
 
 169532686
 
 169532775
 
  ENSEMBL   UCSC 
 
   90
 
  6
 
 32482
 
 tss
 
 0.642955
 
 0.8035895
 
 Not queried
 
  0.9638935
 
       NA
 
           NA
  
 
  
 11
 
    313120
 
    313624
 
  ENSEMBL   UCSC 
 
  505
 
  6
 
 7008
 
 tss;island
 
 0.006079
 
 0.0256253
 
 Not queried
 
  0.9637648
 
       NA
 
           NA
  
 
  
 3
 
 186544871
 
 186545102
 
  ENSEMBL   UCSC 
 
  232
 
  4
 
 29755
 
 island
 
 0.025328
 
 0.0131137
 
 Not queried
 
  0.9637260
 
       NA
 
           NA
  
 
  
 21
 
  46850449
 
  46850840
 
  ENSEMBL   UCSC 
 
  392
 
  5
 
 26467
 
 gene;island
 
 0.410170
 
 0.5755142
 
 Not queried
 
  0.9635055
 
       NA
 
           NA
  
 
  
 5
 
 176873332
 
 176873808
 
  ENSEMBL   UCSC 
 
  477
 
  7
 
 33453
 
 island
 
 0.004277
 
 0.0182081
 
 Not queried
 
  0.9634315
 
       NA
 
           NA
  
 
  
 4
 
  72897565
 
  72897868
 
  ENSEMBL   UCSC 
 
  304
 
  5
 
 30019
 
 gene;island
 
 0.040805
 
 0.1361643
 
 Not queried
 
  0.9634077
 
       NA
 
           NA
  
 
  
 8
 
  87081978
 
  87082205
 
  ENSEMBL   UCSC 
 
  228
 
  7
 
 39797
 
 tss;island
 
 0.633912
 
 0.3244784
 
 Not queried
 
  0.9631512
 
       NA
 
           NA
  
 
  
 13
 
  95364908
 
  95364993
 
  ENSEMBL   UCSC 
 
   86
 
  5
 
 11501
 
 tss;island
 
 0.174192
 
 0.1152288
 
 Not queried
 
  0.9630606
 
       NA
 
           NA
  
 
  
 17
 
  35305905
 
  35306063
 
  ENSEMBL   UCSC 
 
  159
 
  6
 
 17285
 
 tss;island
 
 0.013985
 
 0.0198303
 
 Not queried
 
  0.9630132
 
       NA
 
           NA
  
 
  
 21
 
  47851470
 
  47851941
 
  ENSEMBL   UCSC 
 
  472
 
  5
 
 26478
 
 gene;island
 
 0.957611
 
 0.9672347
 
 Not queried
 
  0.9628821
 
       NA
 
           NA
  
 
  
 6
 
  44225443
 
  44225648
 
  ENSEMBL   UCSC 
 
  206
 
  5
 
 35010
 
 tss;island
 
 0.001643
 
 0.0021429
 
 Not queried
 
  0.9626323
 
       NA
 
           NA
  
 
  
 12
 
  56545428
 
  56545810
 
  ENSEMBL   UCSC 
 
  383
 
  4
 
 9786
 
 tss;island
 
 0.020057
 
 0.0345558
 
 Not queried
 
  0.9623639
 
       NA
 
           NA
  
 
  
 19
 
  46996347
 
  46996868
 
  ENSEMBL   UCSC 
 
  522
 
  4
 
 20110
 
 gene;island
 
 0.054383
 
 0.0408915
 
 Not queried
 
  0.9623546
 
       NA
 
           NA
  
 
  
 6
 
 147829799
 
 147830055
 
  ENSEMBL   UCSC 
 
  257
 
  7
 
 35279
 
 tss
 
 0.012621
 
 0.0082845
 
 Not queried
 
  0.9623278
 
       NA
 
           NA
  
 
  
 11
 
   1593555
 
   1593797
 
  ENSEMBL   UCSC 
 
  243
 
  4
 
 6424
 
 gene;tss;island
 
 0.020537
 
 0.0222316
 
 Not queried
 
  0.9622560
 
       NA
 
           NA
  
 
  
 22
 
  25201891
 
  25202086
 
  ENSEMBL   UCSC 
 
  196
 
  7
 
 27027
 
 tss
 
 0.013286
 
 0.0404890
 
 Not queried
 
  0.9621768
 
       NA
 
           NA
  
 
  
 6
 
  32158153
 
  32158513
 
  ENSEMBL   UCSC 
 
  361
 
  9
 
 34870
 
 tss
 
 0.014773
 
 0.0082695
 
 Not queried
 
  0.9621119
 
       NA
 
           NA
  
 
  
 14
 
  65209844
 
  65210088
 
  ENSEMBL   UCSC 
 
  245
 
  4
 
 12113
 
 gene;island
 
 0.475361
 
 0.5993996
 
 Not queried
 
  0.9620404
 
       NA
 
           NA
  
 
  
 8
 
  15397637
 
  15398333
 
  ENSEMBL   UCSC 
 
  697
 
  8
 
 40099
 
 island
 
 0.066408
 
 0.0451186
 
 Not queried
 
  0.9617753
 
       NA
 
           NA
  
 
  
 4
 
 158141449
 
 158141570
 
  ENSEMBL   UCSC 
 
  122
 
  5
 
 30654
 
 tss;island
 
 0.147611
 
 0.2375075
 
 Not queried
 
  0.9615568
 
       NA
 
           NA
  
 
  
 5
 
    600817
 
    601208
 
  ENSEMBL   UCSC 
 
  392
 
  5
 
 32590
 
 island
 
 0.914508
 
 0.9490096
 
 Not queried
 
  0.9615520
 
       NA
 
           NA
  
 
  
 7
 
   5535302
 
   5535934
 
  ENSEMBL   UCSC 
 
  633
 
  9
 
 36881
 
 gene;island
 
 0.927631
 
 0.9658348
 
 Not queried
 
  0.9614195
 
       NA
 
           NA
  
 
  
 10
 
  97453958
 
  97454076
 
  ENSEMBL   UCSC 
 
  119
 
  6
 
 5248
 
 tss
 
 0.013452
 
 0.0161486
 
 Not queried
 
  0.9612579
 
       NA
 
           NA
  
 
  
 17
 
  48637104
 
  48638190
 
  ENSEMBL   UCSC 
 
 1087
 
 24
 
 17503
 
 tss;island
 
 0.019169
 
 0.0091154
 
 Not queried
 
  0.9605839
 
       NA
 
           NA
  
 
  
 7
 
  39989474
 
  39990042
 
  ENSEMBL   UCSC 
 
  569
 
  7
 
 38429
 
 island
 
 0.004878
 
 0.0075104
 
 Not queried
 
  0.9603853
 
       NA
 
           NA
  
 
  
 19
 
   8509784
 
   8509833
 
  ENSEMBL   UCSC 
 
   50
 
  4
 
 21486
 
 island
 
 0.004611
 
 0.0090602
 
 Not queried
 
  0.9602957
 
       NA
 
           NA
  
 
  
 20
 
  23066999
 
  23067159
 
  ENSEMBL   UCSC 
 
  161
 
  6
 
 25637
 
 tss
 
 0.092531
 
 0.0227112
 
 Not queried
 
  0.9602218
 
       NA
 
           NA
  
 
  
 14
 
 103986227
 
 103986467
 
  ENSEMBL   UCSC 
 
  241
 
  4
 
 12236
 
 gene;island
 
 0.933925
 
 0.8199114
 
 Not queried
 
  0.9601424
 
       NA
 
           NA
  
 
  
 1
 
   1710158
 
   1710265
 
  ENSEMBL   UCSC 
 
  108
 
  5
 
 1161
 
 tss;island
 
 0.007623
 
 0.0042139
 
 Not queried
 
  0.9597127
 
       NA
 
           NA
  
 
  
 6
 
  31093846
 
  31094185
 
  ENSEMBL   UCSC 
 
  340
 
  4
 
 33851
 
 gene
 
 0.087253
 
 0.2335992
 
 Not queried
 
  0.9596790
 
       NA
 
           NA
  
 
  
 6
 
 170494301
 
 170494475
 
  ENSEMBL   UCSC 
 
  175
 
  4
 
 36681
 
 island
 
 0.531653
 
 0.7159911
 
 Not queried
 
  0.9596022
 
       NA
 
           NA
  
 
  
 4
 
 165304443
 
 165304540
 
  ENSEMBL   UCSC 
 
   98
 
  6
 
 30663
 
 tss
 
 0.184156
 
 0.0629486
 
 Not queried
 
  0.9595088
 
       NA
 
           NA
  
 
  
 4
 
 165304259
 
 165304540
 
  ENSEMBL   UCSC 
 
  282
 
  8
 
 31404
 
 island
 
 0.154149
 
 0.0412831
 
 Not queried
 
  0.9595088
 
       NA
 
           NA
  
 
  
 12
 
 132851783
 
 132852053
 
  ENSEMBL   UCSC 
 
  271
 
  4
 
 9440
 
 gene;island
 
 0.319594
 
 0.4153881
 
 Not queried
 
  0.9594814
 
       NA
 
           NA
  
 
  
 10
 
 134790769
 
 134791043
 
  ENSEMBL   UCSC 
 
  275
 
  4
 
 6322
 
 island
 
 0.482315
 
 0.6113362
 
 Not queried
 
  0.9593949
 
       NA
 
           NA
  
 
  
 13
 
  78492568
 
  78494462
 
  ENSEMBL   UCSC 
 
 1895
 
 47
 
 11183
 
 gene;island
 
 0.076660
 
 0.1180853
 
 Not queried
 
  0.9592347
 
       NA
 
           NA
  
 
  
 2
 
   1895643
 
   1895961
 
  ENSEMBL   UCSC 
 
  319
 
  5
 
 22540
 
 gene;island
 
 0.746703
 
 0.9286939
 
 Not queried
 
  0.9590900
 
       NA
 
           NA
  
 
  
 19
 
  46800054
 
  46800602
 
  ENSEMBL   UCSC 
 
  549
 
  8
 
 20891
 
 tss;island
 
 0.016113
 
 0.0329897
 
 Not queried
 
  0.9589151
 
       NA
 
           NA
  
 
  
 11
 
  93394605
 
  93394809
 
  ENSEMBL   UCSC 
 
  205
 
  8
 
 7621
 
 tss
 
 0.002736
 
 0.0044982
 
 Not queried
 
  0.9588304
 
       NA
 
           NA
  
 
  
 2
 
   9459293
 
   9459596
 
  ENSEMBL   UCSC 
 
  304
 
  6
 
 22561
 
 gene;island
 
 0.519843
 
 0.6891677
 
 Not queried
 
  0.9587443
 
       NA
 
           NA
  
 
  
 1
 
  50489240
 
  50489596
 
  ENSEMBL   UCSC 
 
  357
 
  7
 
 522
 
 gene
 
 0.050754
 
 0.1292805
 
 Not queried
 
  0.9586667
 
       NA
 
           NA
  
 
  
 3
 
  50275394
 
  50275694
 
  ENSEMBL   UCSC 
 
  301
 
  5
 
 27796
 
 gene;island
 
 0.011183
 
 0.0055683
 
 Not queried
 
  0.9582082
 
       NA
 
           NA
  
 
  
 1
 
 156211409
 
 156212005
 
  ENSEMBL   UCSC 
 
  597
 
 11
 
 3848
 
 island
 
 0.192006
 
 0.4721466
 
 Not queried
 
  0.9576977
 
       NA
 
           NA
  
 
  
 10
 
 115614308
 
 115614712
 
  ENSEMBL   UCSC 
 
  405
 
  8
 
 5372
 
 tss;island
 
 0.005334
 
 0.0042414
 
 Not queried
 
  0.9574840
 
       NA
 
           NA
  
 
  
 11
 
  89956573
 
  89956777
 
  ENSEMBL   UCSC 
 
  205
 
  6
 
 7616
 
 tss
 
 0.003770
 
 0.0033663
 
 Not queried
 
  0.9574635
 
       NA
 
           NA
  
 
  
 19
 
  37096010
 
  37096588
 
  ENSEMBL   UCSC 
 
  579
 
  7
 
 20004
 
 gene;tss;island
 
 0.033880
 
 0.0163442
 
 Not queried
 
  0.9573642
 
       NA
 
           NA
  
 
  
 18
 
  28622811
 
  28623159
 
  ENSEMBL   UCSC 
 
  349
 
 10
 
 19220
 
 tss
 
 0.372805
 
 0.1263657
 
 Not queried
 
  0.9572205
 
       NA
 
           NA
  
 
  
 12
 
 113495386
 
 113495839
 
  ENSEMBL   UCSC 
 
  454
 
  5
 
 10824
 
 island
 
 0.057334
 
 0.0380671
 
 Not queried
 
  0.9571631
 
       NA
 
           NA
  
 
  
 14
 
 104387923
 
 104388185
 
  ENSEMBL   UCSC 
 
  263
 
  4
 
 12655
 
 tss
 
 0.006049
 
 0.0066071
 
 Not queried
 
  0.9571581
 
       NA
 
           NA
  
 
  
 22
 
  44576869
 
  44577265
 
  ENSEMBL   UCSC 
 
  397
 
  9
 
 26891
 
 gene
 
 0.008021
 
 0.0267509
 
 Not queried
 
  0.9570089
 
       NA
 
           NA
  
 
  
 11
 
 118977782
 
 118978076
 
  ENSEMBL   UCSC 
 
  295
 
  6
 
 7750
 
 tss
 
 0.006215
 
 0.0033348
 
 Not queried
 
  0.9569923
 
       NA
 
           NA
  
 
  
 6
 
  33130918
 
  33132727
 
  ENSEMBL   UCSC 
 
 1810
 
 30
 
 34193
 
 gene;island
 
 0.473426
 
 0.7059361
 
 Not queried
 
  0.9569488
 
       NA
 
           NA
  
 
  
 17
 
  55333738
 
  55333926
 
  ENSEMBL   UCSC 
 
  189
 
  4
 
 17526
 
 tss;island
 
 0.005092
 
 0.0038257
 
 Not queried
 
  0.9567130
 
       NA
 
           NA
  
 
  
 19
 
    474994
 
    475344
 
  ENSEMBL   UCSC 
 
  351
 
  7
 
 20272
 
 tss
 
 0.592932
 
 0.5490772
 
 Not queried
 
  0.9564037
 
       NA
 
           NA
  
 
  
 2
 
 128051348
 
 128051925
 
  ENSEMBL   UCSC 
 
  578
 
  9
 
 24687
 
 island
 
 0.011517
 
 0.0071784
 
 Not queried
 
  0.9562762
 
       NA
 
           NA
  
 
  
 3
 
 110790533
 
 110790767
 
  ENSEMBL   UCSC 
 
  235
 
  5
 
 28495
 
 tss;island
 
 0.003353
 
 0.0021873
 
 Not queried
 
  0.9561881
 
       NA
 
           NA
  
 
  
 7
 
  23510005
 
  23510123
 
  ENSEMBL   UCSC 
 
  119
 
  5
 
 37472
 
 tss;island
 
 0.018045
 
 0.0504408
 
 Not queried
 
  0.9560964
 
       NA
 
           NA
  
 
  
 3
 
  48885189
 
  48885665
 
  ENSEMBL   UCSC 
 
  477
 
 10
 
 29098
 
 island
 
 0.007613
 
 0.0158248
 
 Not queried
 
  0.9559079
 
       NA
 
           NA
  
 
  
 1
 
  32714023
 
  32714308
 
  ENSEMBL   UCSC 
 
  286
 
  5
 
 392
 
 gene;island
 
 0.014066
 
 0.1156577
 
 Not queried
 
  0.9556100
 
       NA
 
           NA
  
 
  
 16
 
  58767723
 
  58768168
 
  ENSEMBL   UCSC 
 
  446
 
  5
 
 14776
 
 gene
 
 0.008762
 
 0.0045113
 
 Not queried
 
  0.9555449
 
       NA
 
           NA
  
 
  
 12
 
  57118908
 
  57119264
 
  ENSEMBL   UCSC 
 
  357
 
  6
 
 9182
 
 gene
 
 0.007787
 
 0.0060520
 
 Not queried
 
  0.9552019
 
       NA
 
           NA
  
 
  
 10
 
  18940291
 
  18940404
 
  ENSEMBL   UCSC 
 
  114
 
  4
 
 4524
 
 gene;island
 
 0.003177
 
 0.0034581
 
 Not queried
 
  0.9550700
 
       NA
 
           NA
  
 
  
 1
 
  35222918
 
  35223247
 
  ENSEMBL   UCSC 
 
  330
 
  5
 
 411
 
 gene
 
 0.323320
 
 0.6728016
 
 Not queried
 
  0.9550571
 
       NA
 
           NA
  
 
  
 3
 
 118753705
 
 118753871
 
  ENSEMBL   UCSC 
 
  167
 
  5
 
 28515
 
 tss
 
 0.006020
 
 0.0057482
 
 Not queried
 
  0.9549629
 
       NA
 
           NA
  
 
  
 4
 
    699224
 
    699435
 
  ENSEMBL   UCSC 
 
  212
 
  6
 
 30230
 
 tss;island
 
 0.020204
 
 0.0237221
 
 Not queried
 
  0.9549081
 
       NA
 
           NA
  
 
  
 16
 
  89146961
 
  89147306
 
  ENSEMBL   UCSC 
 
  346
 
  4
 
 16237
 
 island
 
 0.429465
 
 0.6158274
 
 Not queried
 
  0.9548084
 
       NA
 
           NA
  
 
  
 12
 
  57943724
 
  57944059
 
  ENSEMBL   UCSC 
 
  336
 
  4
 
 10564
 
 island
 
 0.015370
 
 0.0872202
 
 Not queried
 
  0.9547942
 
       NA
 
           NA
  
 
  
 16
 
  31884589
 
  31884761
 
  ENSEMBL   UCSC 
 
  173
 
  4
 
 15217
 
 tss;island
 
 0.876659
 
 0.9616071
 
 Not queried
 
  0.9547248
 
       NA
 
           NA
  
 
  
 2
 
 198569719
 
 198570073
 
  ENSEMBL   UCSC 
 
  355
 
  7
 
 23801
 
 tss
 
 0.010374
 
 0.0028394
 
 Not queried
 
  0.9547156
 
       NA
 
           NA
  
 
  
 22
 
  38200894
 
  38201142
 
  ENSEMBL   UCSC 
 
  249
 
  4
 
 27472
 
 island
 
 0.013033
 
 0.0065347
 
 Not queried
 
  0.9546258
 
       NA
 
           NA
  
 
  
 19
 
   2096672
 
   2097096
 
  ENSEMBL   UCSC 
 
  425
 
  9
 
 20318
 
 tss;island
 
 0.004336
 
 0.0017894
 
 Not queried
 
  0.9543408
 
       NA
 
           NA
  
 
  
 11
 
 118901407
 
 118901610
 
  ENSEMBL   UCSC 
 
  204
 
  4
 
 6934
 
 gene
 
 0.003342
 
 0.0059795
 
 Not queried
 
  0.9542931
 
       NA
 
           NA
  
 
  
 11
 
  64993076
 
  64993335
 
  ENSEMBL   UCSC 
 
  260
 
  4
 
 6704
 
 gene;island
 
 0.014748
 
 0.0322030
 
 Not queried
 
  0.9542356
 
       NA
 
           NA
  
 
  
 17
 
    171006
 
    171434
 
  ENSEMBL   UCSC 
 
  429
 
  5
 
 16290
 
 gene;island
 
 0.467092
 
 0.6591785
 
 Not queried
 
  0.9540872
 
       NA
 
           NA
  
 
  
 14
 
  69726197
 
  69727092
 
  ENSEMBL   UCSC 
 
  896
 
 11
 
 12965
 
 island
 
 0.126836
 
 0.0440992
 
 Not queried
 
  0.9539661
 
       NA
 
           NA
  
 
  
 11
 
  69924046
 
  69924390
 
  ENSEMBL   UCSC 
 
  345
 
 10
 
 7524
 
 tss;island
 
 0.069226
 
 0.1496463
 
 Not queried
 
  0.9537683
 
       NA
 
           NA
  
 
  
 5
 
 138677027
 
 138677638
 
  ENSEMBL   UCSC 
 
  612
 
  7
 
 32330
 
 tss;island
 
 0.007403
 
 0.0092586
 
 Not queried
 
  0.9537176
 
       NA
 
           NA
  
 
  
 5
 
 110408740
 
 110408997
 
  ENSEMBL   UCSC 
 
  258
 
  7
 
 32206
 
 tss
 
 0.010663
 
 0.0254239
 
 Not queried
 
  0.9536800
 
       NA
 
           NA
  
 
  
 19
 
  40023151
 
  40023459
 
  ENSEMBL   UCSC 
 
  309
 
  4
 
 20030
 
 gene
 
 0.006424
 
 0.0107447
 
 Not queried
 
  0.9534192
 
       NA
 
           NA
  
 
  
 1
 
  36771714
 
  36771844
 
  ENSEMBL   UCSC 
 
  131
 
  4
 
 1494
 
 tss;island
 
 0.580664
 
 0.7394200
 
 Not queried
 
  0.9534186
 
       NA
 
           NA
  
 
  
 3
 
  11762011
 
  11762372
 
  ENSEMBL   UCSC 
 
  362
 
  5
 
 28887
 
 island
 
 0.020371
 
 0.0273088
 
 Not queried
 
  0.9534038
 
       NA
 
           NA
  
 
  
 16
 
  22824877
 
  22825282
 
  ENSEMBL   UCSC 
 
  406
 
  5
 
 15133
 
 tss;island
 
 0.063175
 
 0.2432249
 
 Not queried
 
  0.9533253
 
       NA
 
           NA
  
 
  
 20
 
   3827226
 
   3827437
 
  ENSEMBL   UCSC 
 
  212
 
  9
 
 25571
 
 tss
 
 0.004128
 
 0.0047252
 
 Not queried
 
  0.9529899
 
       NA
 
           NA
  
 
  
 17
 
  11900692
 
  11901031
 
  ENSEMBL   UCSC 
 
  340
 
  4
 
 17138
 
 tss
 
 0.012971
 
 0.0107083
 
 Not queried
 
  0.9529459
 
       NA
 
           NA
  
 
  
 17
 
  11900443
 
  11901031
 
  ENSEMBL   UCSC 
 
  589
 
  6
 
 18024
 
 island
 
 0.005441
 
 0.0053290
 
 Not queried
 
  0.9529459
 
       NA
 
           NA
  
 
  
 15
 
  27111388
 
  27112197
 
  ENSEMBL   UCSC 
 
  810
 
 21
 
 13542
 
 tss
 
 0.029628
 
 0.0903986
 
 Not queried
 
  0.9529233
 
       NA
 
           NA
  
 
  
 10
 
   5855475
 
   5855675
 
  ENSEMBL   UCSC 
 
  201
 
  4
 
 5523
 
 island
 
 0.011908
 
 0.0100277
 
 Not queried
 
  0.9527184
 
       NA
 
           NA
  
 
  
 20
 
  62680986
 
  62681428
 
  ENSEMBL   UCSC 
 
  443
 
  9
 
 25909
 
 tss
 
 0.011560
 
 0.0071476
 
 Not queried
 
  0.9526116
 
       NA
 
           NA
  
 
  
 18
 
  53257019
 
  53257340
 
  ENSEMBL   UCSC 
 
  322
 
  5
 
 19286
 
 tss;island
 
 0.008710
 
 0.0095935
 
 Not queried
 
  0.9525892
 
       NA
 
           NA
  
 
  
 17
 
   1394477
 
   1394841
 
  ENSEMBL   UCSC 
 
  365
 
  4
 
 16314
 
 gene;island
 
 0.011056
 
 0.0107952
 
 Not queried
 
  0.9525032
 
       NA
 
           NA
  
 
  
 11
 
 114309932
 
 114310121
 
  ENSEMBL   UCSC 
 
  190
 
  8
 
 8825
 
 island
 
 0.007813
 
 0.0050774
 
 Not queried
 
  0.9523402
 
       NA
 
           NA
  
 
  
 7
 
  92463218
 
  92463390
 
  ENSEMBL   UCSC 
 
  173
 
  7
 
 37088
 
 gene;island
 
 0.005469
 
 0.0086647
 
 Not queried
 
  0.9523340
 
       NA
 
           NA
  
 
  
 1
 
  40203334
 
  40203715
 
  ENSEMBL   UCSC 
 
  382
 
  4
 
 1527
 
 tss;island
 
 0.902936
 
 0.9333612
 
 Not queried
 
  0.9520590
 
       NA
 
           NA
  
 
  
 6
 
  34024220
 
  34024479
 
  ENSEMBL   UCSC 
 
  260
 
  4
 
 34301
 
 gene;island
 
 0.685662
 
 0.5745958
 
 Not queried
 
  0.9520330
 
       NA
 
           NA
  
 
  
 6
 
  32187918
 
  32188404
 
  ENSEMBL   UCSC 
 
  487
 
  5
 
 34098
 
 gene
 
 0.073084
 
 0.1464799
 
 Not queried
 
  0.9519036
 
       NA
 
           NA
  
 
  
 4
 
  26492153
 
  26492488
 
  ENSEMBL   UCSC 
 
  336
 
  6
 
 30337
 
 tss
 
 0.452576
 
 0.5791041
 
 Not queried
 
  0.9518223
 
       NA
 
           NA
  
 
  
 1
 
 235530811
 
 235530995
 
  ENSEMBL   UCSC 
 
  185
 
  4
 
 1089
 
 gene
 
 0.003218
 
 0.0071476
 
 Not queried
 
  0.9517482
 
       NA
 
           NA
  
 
  
 12
 
 108155154
 
 108155279
 
  ENSEMBL   UCSC 
 
  126
 
  4
 
 9978
 
 tss;island
 
 0.010511
 
 0.0049938
 
 Not queried
 
  0.9517045
 
       NA
 
           NA
  
 
  
 7
 
 157690609
 
 157690769
 
  ENSEMBL   UCSC 
 
  161
 
  4
 
 37350
 
 gene;island
 
 0.701538
 
 0.8445501
 
 Not queried
 
  0.9516021
 
       NA
 
           NA
  
 
  
 6
 
 167535764
 
 167536184
 
  ENSEMBL   UCSC 
 
  421
 
  7
 
 34556
 
 gene;tss
 
 0.002815
 
 0.0143080
 
 Not queried
 
  0.9514428
 
       NA
 
           NA
  
 
  
 3
 
 197517855
 
 197518344
 
  ENSEMBL   UCSC 
 
  490
 
 10
 
 29822
 
 island
 
 0.007204
 
 0.0032237
 
 Not queried
 
  0.9510538
 
       NA
 
           NA
  
 
  
 7
 
 127881269
 
 127881440
 
  ENSEMBL   UCSC 
 
  172
 
  5
 
 38892
 
 island
 
 0.738956
 
 0.8531145
 
 Not queried
 
  0.9508596
 
       NA
 
           NA
  
 
  
 12
 
 117627133
 
 117627651
 
  ENSEMBL   UCSC 
 
  519
 
  7
 
 9337
 
 gene;island
 
 0.024794
 
 0.2280986
 
 Not queried
 
  0.9507746
 
       NA
 
           NA
  
 
  
 6
 
  46097392
 
  46097784
 
  ENSEMBL   UCSC 
 
  393
 
  9
 
 36184
 
 island
 
 0.006093
 
 0.0027746
 
 Not queried
 
  0.9505998
 
       NA
 
           NA
  
 
  
 15
 
  93609305
 
  93609551
 
  ENSEMBL   UCSC 
 
  247
 
  4
 
 13488
 
 gene;island
 
 0.379641
 
 0.7014560
 
 Not queried
 
  0.9505865
 
       NA
 
           NA
  
 
  
 16
 
  27280150
 
  27280434
 
  ENSEMBL   UCSC 
 
  285
 
  8
 
 15150
 
 tss
 
 0.001887
 
 0.0036258
 
 Not queried
 
  0.9503644
 
       NA
 
           NA
  
 
  
 1
 
  47133596
 
  47133953
 
  ENSEMBL   UCSC 
 
  358
 
  4
 
 513
 
 gene;island
 
 0.007317
 
 0.0062118
 
 Not queried
 
  0.9500799
 
       NA
 
           NA
  
 
  
 19
 
  37701476
 
  37701738
 
  ENSEMBL   UCSC 
 
  263
 
  7
 
 20735
 
 tss
 
 0.006842
 
 0.0079089
 
 Not queried
 
  0.9500745
 
       NA
 
           NA
  
 
  
 19
 
  51071294
 
  51071328
 
  ENSEMBL   UCSC 
 
   35
 
  5
 
 22315
 
 island
 
 0.010598
 
 0.0248541
 
 Not queried
 
  0.9500177
 
       NA
 
           NA
  
 
  
 17
 
  45177378
 
  45177628
 
  ENSEMBL   UCSC 
 
  251
 
  5
 
 18451
 
 island
 
 0.006148
 
 0.0079630
 
 Not queried
 
  0.9497785
 
       NA
 
           NA
  
 
  
 4
 
 111397134
 
 111398146
 
  ENSEMBL   UCSC 
 
 1013
 
 13
 
 31244
 
 island
 
 0.459156
 
 0.7825848
 
 Not queried
 
  0.9497416
 
       NA
 
           NA
  
 
  
 20
 
  42983920
 
  42984394
 
  ENSEMBL   UCSC 
 
  475
 
  9
 
 25758
 
 tss
 
 0.429305
 
 0.7587622
 
 Not queried
 
  0.9496478
 
       NA
 
           NA
  
 
  
 4
 
 134069236
 
 134069670
 
  ENSEMBL   UCSC 
 
  435
 
  6
 
 30593
 
 tss;island
 
 0.077434
 
 0.0576298
 
 Not queried
 
  0.9495541
 
       NA
 
           NA
  
 
  
 6
 
  29768219
 
  29768275
 
  ENSEMBL   UCSC 
 
   57
 
  7
 
 35631
 
 island
 
 0.655738
 
 0.8549971
 
 Not queried
 
  0.9495014
 
       NA
 
           NA
  
 
  
 3
 
 190040181
 
 190040375
 
  ENSEMBL   UCSC 
 
  195
 
  5
 
 29768
 
 island
 
 0.037370
 
 0.0191556
 
 Not queried
 
  0.9494346
 
       NA
 
           NA
  
 
  
 2
 
  70780723
 
  70781101
 
  ENSEMBL   UCSC 
 
  379
 
  5
 
 22724
 
 gene
 
 0.016616
 
 0.0102549
 
 Not queried
 
  0.9493683
 
       NA
 
           NA
  
 
  
 10
 
 124768526
 
 124768896
 
  ENSEMBL   UCSC 
 
  371
 
  5
 
 4801
 
 gene;tss;island
 
 0.003981
 
 0.0035014
 
 Not queried
 
  0.9492681
 
       NA
 
           NA
  
 
  
 15
 
  99057732
 
  99058098
 
  ENSEMBL   UCSC 
 
  367
 
  4
 
 13922
 
 tss
 
 0.306344
 
 0.6064264
 
 Not queried
 
  0.9491993
 
       NA
 
           NA
  
 
  
 17
 
     85064
 
     85492
 
  ENSEMBL   UCSC 
 
  429
 
  5
 
 16284
 
 gene;island
 
 0.599021
 
 0.8180296
 
 Not queried
 
  0.9490769
 
       NA
 
           NA
  
 
  
 11
 
 123525662
 
 123525714
 
  ENSEMBL   UCSC 
 
   53
 
  4
 
 7779
 
 tss;island
 
 0.083696
 
 0.1396061
 
 Not queried
 
  0.9490313
 
       NA
 
           NA
  
 
  
 11
 
   2889602
 
   2890725
 
  ENSEMBL   UCSC 
 
 1124
 
 35
 
 7976
 
 island
 
 0.223825
 
 0.0585214
 
 Not queried
 
  0.9489709
 
       NA
 
           NA
  
 
  
 10
 
 130012874
 
 130013154
 
  ENSEMBL   UCSC 
 
  281
 
  6
 
 6254
 
 island
 
 0.421113
 
 0.5905936
 
 Not queried
 
  0.9488400
 
       NA
 
           NA
  
 
  
 7
 
   2695197
 
   2695468
 
  ENSEMBL   UCSC 
 
  272
 
  5
 
 36809
 
 gene;island
 
 0.964997
 
 0.9067375
 
 Not queried
 
  0.9487730
 
       NA
 
           NA
  
 
  
 17
 
  73726093
 
  73726430
 
  ENSEMBL   UCSC 
 
  338
 
  6
 
 16777
 
 gene;island
 
 0.648867
 
 0.8140998
 
 Not queried
 
  0.9487201
 
       NA
 
           NA
  
 
  
 19
 
  14542609
 
  14543048
 
  ENSEMBL   UCSC 
 
  440
 
  4
 
 21680
 
 island
 
 0.009305
 
 0.2467952
 
 Not queried
 
  0.9486820
 
       NA
 
           NA
  
 
  
 19
 
  47334484
 
  47334821
 
  ENSEMBL   UCSC 
 
  338
 
  4
 
 20900
 
 tss
 
 0.722298
 
 0.9542599
 
 Not queried
 
  0.9485147
 
       NA
 
           NA
  
 
  
 7
 
 102789948
 
 102790168
 
  ENSEMBL   UCSC 
 
  221
 
  4
 
 37808
 
 tss;island
 
 0.054028
 
 0.0421651
 
 Not queried
 
  0.9485130
 
       NA
 
           NA
  
 
  
 5
 
  14872207
 
  14872612
 
  ENSEMBL   UCSC 
 
  406
 
  4
 
 31993
 
 tss;island
 
 0.007967
 
 0.0045467
 
 Not queried
 
  0.9484443
 
       NA
 
           NA
  
 
  
 17
 
   1880884
 
   1881333
 
  ENSEMBL   UCSC 
 
  450
 
  6
 
 16325
 
 gene;island
 
 0.176968
 
 0.1292824
 
 Not queried
 
  0.9484064
 
       NA
 
           NA
  
 
  
 22
 
  19554366
 
  19554706
 
  ENSEMBL   UCSC 
 
  341
 
  5
 
 26962
 
 tss
 
 0.792023
 
 0.7598878
 
 Not queried
 
  0.9483189
 
       NA
 
           NA
  
 
  
 6
 
  10383526
 
  10383804
 
  ENSEMBL   UCSC 
 
  279
 
  4
 
 35422
 
 island
 
 0.025002
 
 0.0584343
 
 Not queried
 
  0.9483129
 
       NA
 
           NA
  
 
  
 17
 
   4981403
 
   4981610
 
  ENSEMBL   UCSC 
 
  208
 
  5
 
 17040
 
 tss;island
 
 0.003879
 
 0.0064807
 
 Not queried
 
  0.9483078
 
       NA
 
           NA
  
 
  
 8
 
  71520850
 
  71520989
 
  ENSEMBL   UCSC 
 
  140
 
  4
 
 39760
 
 tss;island
 
 0.511461
 
 0.2337952
 
 Not queried
 
  0.9479515
 
       NA
 
           NA
  
 
  
 5
 
 119799822
 
 119799864
 
  ENSEMBL   UCSC 
 
   43
 
  4
 
 32240
 
 tss;island
 
 0.001582
 
 0.0065457
 
 Not queried
 
  0.9477929
 
       NA
 
           NA
  
 
  
 18
 
    904851
 
    905611
 
  ENSEMBL   UCSC 
 
  761
 
 15
 
 19351
 
 island
 
 0.022084
 
 0.0849719
 
 Not queried
 
  0.9476689
 
       NA
 
           NA
  
 
  
 17
 
  77030221
 
  77030546
 
  ENSEMBL   UCSC 
 
  326
 
  5
 
 16834
 
 gene
 
 0.453879
 
 0.5599452
 
 Not queried
 
  0.9475775
 
       NA
 
           NA
  
 
  
 7
 
   2423231
 
   2423467
 
  ENSEMBL   UCSC 
 
  237
 
  4
 
 38156
 
 island
 
 0.950237
 
 0.9725626
 
 Not queried
 
  0.9475762
 
       NA
 
           NA
  
 
  
 7
 
  19157634
 
  19158747
 
  ENSEMBL   UCSC 
 
 1114
 
 22
 
 37460
 
 tss;island
 
 0.017334
 
 0.0570081
 
 Not queried
 
  0.9474178
 
       NA
 
           NA
  
 
  
 14
 
 106321751
 
 106321936
 
  ENSEMBL   UCSC 
 
  186
 
  4
 
 13246
 
 island
 
 0.236927
 
 0.4254188
 
 Not queried
 
  0.9472258
 
       NA
 
           NA
  
 
  
 17
 
  63557887
 
  63558518
 
  ENSEMBL   UCSC 
 
  632
 
  6
 
 17593
 
 tss
 
 0.065509
 
 0.0347711
 
 Not queried
 
  0.9470336
 
       NA
 
           NA
  
 
  
 17
 
  63557609
 
  63558518
 
  ENSEMBL   UCSC 
 
  910
 
  8
 
 18674
 
 island
 
 0.036739
 
 0.0328286
 
 Not queried
 
  0.9470336
 
       NA
 
           NA
  
 
  
 2
 
 225266733
 
 225266880
 
  ENSEMBL   UCSC 
 
  148
 
  6
 
 23925
 
 tss
 
 0.005908
 
 0.0077675
 
 Not queried
 
  0.9469873
 
       NA
 
           NA
  
 
  
 2
 
 161350329
 
 161350625
 
  ENSEMBL   UCSC 
 
  297
 
  6
 
 23685
 
 tss
 
 0.012531
 
 0.0236527
 
 Not queried
 
  0.9469207
 
       NA
 
           NA
  
 
  
 2
 
 161350154
 
 161350625
 
  ENSEMBL   UCSC 
 
  472
 
  8
 
 24818
 
 island
 
 0.012531
 
 0.0203187
 
 Not queried
 
  0.9469207
 
       NA
 
           NA
  
 
  
 21
 
  41238940
 
  41239323
 
  ENSEMBL   UCSC 
 
  384
 
  4
 
 26553
 
 tss
 
 0.018640
 
 0.0144861
 
 Not queried
 
  0.9468890
 
       NA
 
           NA
  
 
  
 2
 
 197664308
 
 197664530
 
  ENSEMBL   UCSC 
 
  223
 
  4
 
 24978
 
 island
 
 0.002242
 
 0.0019736
 
 Not queried
 
  0.9467475
 
       NA
 
           NA
  
 
  
 19
 
  12476027
 
  12476363
 
  ENSEMBL   UCSC 
 
  337
 
  4
 
 19854
 
 gene
 
 0.047515
 
 0.0163407
 
 Not queried
 
  0.9467060
 
       NA
 
           NA
  
 
  
 19
 
  12476027
 
  12476577
 
  ENSEMBL   UCSC 
 
  551
 
  8
 
 21600
 
 island
 
 0.012837
 
 0.0082219
 
 Not queried
 
  0.9467060
 
       NA
 
           NA
  
 
  
 12
 
  57619178
 
  57619401
 
  ENSEMBL   UCSC 
 
  224
 
  5
 
 9189
 
 gene;island
 
 0.036803
 
 0.0405855
 
 Not queried
 
  0.9467043
 
       NA
 
           NA
  
 
  
 12
 
  56651912
 
  56652449
 
  ENSEMBL   UCSC 
 
  538
 
 10
 
 10530
 
 island
 
 0.005654
 
 0.0032867
 
 Not queried
 
  0.9465634
 
       NA
 
           NA
  
 
  
 6
 
  74072242
 
  74072376
 
  ENSEMBL   UCSC 
 
  135
 
  6
 
 35067
 
 tss
 
 0.791111
 
 0.8853230
 
 Not queried
 
  0.9463966
 
       NA
 
           NA
  
 
  
 3
 
 147125712
 
 147127143
 
  ENSEMBL   UCSC 
 
 1432
 
 27
 
 28652
 
 tss
 
 0.369073
 
 0.6112269
 
 Not queried
 
  0.9463199
 
       NA
 
           NA
  
 
  
 2
 
 228336753
 
 228336928
 
  ENSEMBL   UCSC 
 
  176
 
  4
 
 25167
 
 island
 
 0.010112
 
 0.0065459
 
 Not queried
 
  0.9463150
 
       NA
 
           NA
  
 
  
 1
 
  45187551
 
  45187946
 
  ENSEMBL   UCSC 
 
  396
 
  4
 
 494
 
 gene;island
 
 0.083925
 
 0.0473853
 
 Not queried
 
  0.9462435
 
       NA
 
           NA
  
 
  
 2
 
 150444370
 
 150444834
 
  ENSEMBL   UCSC 
 
  465
 
  6
 
 23657
 
 tss
 
 0.006916
 
 0.0625583
 
 Not queried
 
  0.9461778
 
       NA
 
           NA
  
 
  
 1
 
  40367661
 
  40367965
 
  ENSEMBL   UCSC 
 
  305
 
  8
 
 3110
 
 island
 
 0.003959
 
 0.0052209
 
 Not queried
 
  0.9461251
 
       NA
 
           NA
  
 
  
 4
 
   4543301
 
   4543764
 
  ENSEMBL   UCSC 
 
  464
 
  5
 
 29917
 
 gene
 
 0.006212
 
 0.0039159
 
 Not queried
 
  0.9460950
 
       NA
 
           NA
  
 
  
 16
 
   9185438
 
   9185479
 
  ENSEMBL   UCSC 
 
   42
 
  4
 
 15085
 
 tss
 
 0.005018
 
 0.0084961
 
 Not queried
 
  0.9459569
 
       NA
 
           NA
  
 
  
 15
 
  75932502
 
  75933022
 
  ENSEMBL   UCSC 
 
  521
 
 12
 
 14336
 
 island
 
 0.005596
 
 0.0034357
 
 Not queried
 
  0.9455687
 
       NA
 
           NA
  
 
  
 13
 
  21635665
 
  21635991
 
  ENSEMBL   UCSC 
 
  327
 
  9
 
 11583
 
 island
 
 0.011921
 
 0.0078641
 
 Not queried
 
  0.9455379
 
       NA
 
           NA
  
 
  
 19
 
  31847893
 
  31848310
 
  ENSEMBL   UCSC 
 
  418
 
  6
 
 21863
 
 island
 
 0.032221
 
 0.0297506
 
 Not queried
 
  0.9451905
 
       NA
 
           NA
  
 
  
 17
 
  79818530
 
  79818808
 
  ENSEMBL   UCSC 
 
  279
 
  6
 
 18945
 
 island
 
 0.003063
 
 0.0054234
 
 Not queried
 
  0.9451164
 
       NA
 
           NA
  
 
  
 17
 
  76100911
 
  76101391
 
  ENSEMBL   UCSC 
 
  481
 
  5
 
 16813
 
 gene;island
 
 0.964832
 
 0.9752683
 
 Not queried
 
  0.9447965
 
       NA
 
           NA
  
 
  
 10
 
 102590168
 
 102590401
 
  ENSEMBL   UCSC 
 
  234
 
  4
 
 6036
 
 island
 
 0.129145
 
 0.0606875
 
 Not queried
 
  0.9445843
 
       NA
 
           NA
  
 
  
 7
 
  96654425
 
  96654572
 
  ENSEMBL   UCSC 
 
  148
 
  4
 
 37736
 
 tss;island
 
 0.043575
 
 0.0574199
 
 Not queried
 
  0.9445768
 
       NA
 
           NA
  
 
  
 18
 
  77138860
 
  77139085
 
  ENSEMBL   UCSC 
 
  226
 
  4
 
 19597
 
 island
 
 0.131497
 
 0.2062004
 
 Not queried
 
  0.9444242
 
       NA
 
           NA
  
 
  
 8
 
  74888080
 
  74888271
 
  ENSEMBL   UCSC 
 
  192
 
  4
 
 39772
 
 tss;island
 
 0.009727
 
 0.0033398
 
 Not queried
 
  0.9443918
 
       NA
 
           NA
  
 
  
 20
 
  62209427
 
  62209642
 
  ENSEMBL   UCSC 
 
  216
 
  4
 
 26361
 
 island
 
 0.780258
 
 0.8881664
 
 Not queried
 
  0.9443326
 
       NA
 
           NA
  
 
  
 7
 
 157561010
 
 157561161
 
  ENSEMBL   UCSC 
 
  152
 
  4
 
 37342
 
 gene;island
 
 0.803402
 
 0.8619012
 
 Not queried
 
  0.9440147
 
       NA
 
           NA
  
 
  
 6
 
  91006592
 
  91006647
 
  ENSEMBL   UCSC 
 
   56
 
  6
 
 35123
 
 tss;island
 
 0.000631
 
 0.0039734
 
 Not queried
 
  0.9438718
 
       NA
 
           NA
  
 
  
 1
 
  62119764
 
  62120090
 
  ENSEMBL   UCSC 
 
  327
 
  5
 
 3333
 
 island
 
 0.915617
 
 0.9308259
 
 Not queried
 
  0.9438058
 
       NA
 
           NA
  
 
  
 16
 
  89626662
 
  89626761
 
  ENSEMBL   UCSC 
 
  100
 
  4
 
 15426
 
 tss;island
 
 0.002420
 
 0.0052063
 
 Not queried
 
  0.9437176
 
       NA
 
           NA
  
 
  
 1
 
 247694271
 
 247694557
 
  ENSEMBL   UCSC 
 
  287
 
  5
 
 1127
 
 gene;tss;island
 
 0.950308
 
 0.8513915
 
 Not queried
 
  0.9436027
 
       NA
 
           NA
  
 
  
 17
 
   8534048
 
   8534187
 
  ENSEMBL   UCSC 
 
  140
 
  6
 
 17120
 
 tss
 
 0.009355
 
 0.0041131
 
 Not queried
 
  0.9434404
 
       NA
 
           NA
  
 
  
 1
 
  32671304
 
  32671645
 
  ENSEMBL   UCSC 
 
  342
 
  5
 
 389
 
 gene
 
 0.035026
 
 0.0240891
 
 Not queried
 
  0.9434341
 
       NA
 
           NA
  
 
  
 11
 
  47608047
 
  47608487
 
  ENSEMBL   UCSC 
 
  441
 
  4
 
 8270
 
 island
 
 0.703473
 
 0.8975050
 
 Not queried
 
  0.9429846
 
       NA
 
           NA
  
 
  
 1
 
   1606206
 
   1606557
 
  ENSEMBL   UCSC 
 
  352
 
  4
 
 45
 
 gene;island
 
 0.942708
 
 0.9745467
 
 Not queried
 
  0.9429822
 
       NA
 
           NA
  
 
  
 3
 
  21792857
 
  21792991
 
  ENSEMBL   UCSC 
 
  135
 
  4
 
 28182
 
 tss
 
 0.010247
 
 0.0093229
 
 Not queried
 
  0.9429045
 
       NA
 
           NA
  
 
  
 5
 
 172175604
 
 172175855
 
  ENSEMBL   UCSC 
 
  252
 
  4
 
 33395
 
 island
 
 0.656209
 
 0.7606219
 
 Not queried
 
  0.9427988
 
       NA
 
           NA
  
 
  
 7
 
 121943990
 
 121944500
 
  ENSEMBL   UCSC 
 
  511
 
  5
 
 37205
 
 gene
 
 0.342367
 
 0.1978374
 
 Not queried
 
  0.9426319
 
       NA
 
           NA
  
 
  
 2
 
 239112427
 
 239112731
 
  ENSEMBL   UCSC 
 
  305
 
  5
 
 23971
 
 tss
 
 0.015670
 
 0.0283169
 
 Not queried
 
  0.9425653
 
       NA
 
           NA
  
 
  
 15
 
  89764999
 
  89765204
 
  ENSEMBL   UCSC 
 
  206
 
  4
 
 13884
 
 tss
 
 0.461341
 
 0.6290989
 
 Not queried
 
  0.9424954
 
       NA
 
           NA
  
 
  
 12
 
 120105372
 
 120105711
 
  ENSEMBL   UCSC 
 
  340
 
  5
 
 10051
 
 tss
 
 0.010555
 
 0.0040852
 
 Not queried
 
  0.9424286
 
       NA
 
           NA
  
 
  
 5
 
 113697861
 
 113698506
 
  ENSEMBL   UCSC 
 
  646
 
  8
 
 33017
 
 island
 
 0.122149
 
 0.0744738
 
 Not queried
 
  0.9423314
 
       NA
 
           NA
  
 
  
 1
 
  23521272
 
  23521417
 
  ENSEMBL   UCSC 
 
  146
 
  4
 
 1336
 
 tss
 
 0.528198
 
 0.6427617
 
 Not queried
 
  0.9421431
 
       NA
 
           NA
  
 
  
 17
 
  17206295
 
  17206590
 
  ENSEMBL   UCSC 
 
  296
 
  6
 
 17165
 
 tss;island
 
 0.015415
 
 0.0087085
 
 Not queried
 
  0.9415350
 
       NA
 
           NA
  
 
  
 1
 
  35324702
 
  35324786
 
  ENSEMBL   UCSC 
 
   85
 
  4
 
 1479
 
 tss
 
 0.009672
 
 0.0045668
 
 Not queried
 
  0.9414802
 
       NA
 
           NA
  
 
  
 15
 
  75249787
 
  75249914
 
  ENSEMBL   UCSC 
 
  128
 
  6
 
 13806
 
 tss;island
 
 0.009602
 
 0.0275118
 
 Not queried
 
  0.9414272
 
       NA
 
           NA
  
 
  
 12
 
  30848820
 
  30849114
 
  ENSEMBL   UCSC 
 
  295
 
  4
 
 9624
 
 tss
 
 0.004025
 
 0.0055739
 
 Not queried
 
  0.9412501
 
       NA
 
           NA
  
 
  
 4
 
   1342161
 
   1342459
 
  ENSEMBL   UCSC 
 
  299
 
  4
 
 29870
 
 gene;island
 
 0.929057
 
 0.9586635
 
 Not queried
 
  0.9410608
 
       NA
 
           NA
  
 
  
 8
 
  38585881
 
  38586183
 
  ENSEMBL   UCSC 
 
  303
 
  4
 
 39285
 
 gene
 
 0.127809
 
 0.2152031
 
 Not queried
 
  0.9410178
 
       NA
 
           NA
  
 
  
 6
 
  30882994
 
  30883203
 
  ENSEMBL   UCSC 
 
  210
 
  5
 
 33838
 
 gene;island
 
 0.651072
 
 0.9553265
 
 Not queried
 
  0.9407023
 
       NA
 
           NA
  
 
  
 4
 
 141264393
 
 141264602
 
  ENSEMBL   UCSC 
 
  210
 
  4
 
 30604
 
 tss
 
 0.003843
 
 0.0272402
 
 Not queried
 
  0.9406374
 
       NA
 
           NA
  
 
  
 16
 
   2961485
 
   2961939
 
  ENSEMBL   UCSC 
 
  455
 
  5
 
 15040
 
 tss;island
 
 0.005360
 
 0.0044695
 
 Not queried
 
  0.9406210
 
       NA
 
           NA
  
 
  
 12
 
 122906318
 
 122906821
 
  ENSEMBL   UCSC 
 
  504
 
  5
 
 9373
 
 gene;island
 
 0.001868
 
 0.0041428
 
 Not queried
 
  0.9404290
 
       NA
 
           NA
  
 
  
 11
 
  93063516
 
  93064234
 
  ENSEMBL   UCSC 
 
  719
 
  9
 
 8727
 
 island
 
 0.013158
 
 0.0579540
 
 Not queried
 
  0.9404073
 
       NA
 
           NA
  
 
  
 19
 
  52511502
 
  52511900
 
  ENSEMBL   UCSC 
 
  399
 
 10
 
 21003
 
 tss
 
 0.016667
 
 0.0116743
 
 Not queried
 
  0.9403535
 
       NA
 
           NA
  
 
  
 2
 
 190526173
 
 190526349
 
  ENSEMBL   UCSC 
 
  177
 
  4
 
 22957
 
 gene
 
 0.012989
 
 0.0067541
 
 Not queried
 
  0.9400988
 
       NA
 
           NA
  
 
  
 2
 
  85843042
 
  85843265
 
  ENSEMBL   UCSC 
 
  224
 
  9
 
 23487
 
 tss
 
 0.007833
 
 0.0044290
 
 Not queried
 
  0.9400767
 
       NA
 
           NA
  
 
  
 7
 
 148823215
 
 148823355
 
  ENSEMBL   UCSC 
 
  141
 
  4
 
 37261
 
 gene;tss;island
 
 0.004197
 
 0.0056950
 
 Not queried
 
  0.9400123
 
       NA
 
           NA
  
 
  
 17
 
  78078605
 
  78079086
 
  ENSEMBL   UCSC 
 
  482
 
  5
 
 16855
 
 gene;island
 
 0.814270
 
 0.9650583
 
 Not queried
 
  0.9397727
 
       NA
 
           NA
  
 
  
 16
 
   1030383
 
   1030619
 
  ENSEMBL   UCSC 
 
  237
 
  8
 
 14986
 
 tss;island
 
 0.068111
 
 0.0275989
 
 Not queried
 
  0.9396355
 
       NA
 
           NA
  
 
  
 6
 
  11232665
 
  11233125
 
  ENSEMBL   UCSC 
 
  461
 
  7
 
 33570
 
 gene
 
 0.002550
 
 0.0033913
 
 Not queried
 
  0.9394677
 
       NA
 
           NA
  
 
  
 4
 
 147867081
 
 147867360
 
  ENSEMBL   UCSC 
 
  280
 
  6
 
 30625
 
 tss;island
 
 0.020241
 
 0.0170234
 
 Not queried
 
  0.9392250
 
       NA
 
           NA
  
 
  
 13
 
 100740910
 
 100741308
 
  ENSEMBL   UCSC 
 
  399
 
 10
 
 11516
 
 tss
 
 0.004265
 
 0.0072074
 
 Not queried
 
  0.9391916
 
       NA
 
           NA
  
 
  
 1
 
 221068730
 
 221068935
 
  ENSEMBL   UCSC 
 
  206
 
  4
 
 4226
 
 island
 
 0.942361
 
 0.8679662
 
 Not queried
 
  0.9388457
 
       NA
 
           NA
  
 
  
 5
 
  20305576
 
  20305935
 
  ENSEMBL   UCSC 
 
  360
 
  4
 
 32710
 
 island
 
 0.741922
 
 0.8223429
 
 Not queried
 
  0.9388139
 
       NA
 
           NA
  
 
  
 13
 
  50656354
 
  50656747
 
  ENSEMBL   UCSC 
 
  394
 
  6
 
 11168
 
 gene;island
 
 0.003038
 
 0.0011425
 
 Not queried
 
  0.9387921
 
       NA
 
           NA
  
 
  
 1
 
  41131453
 
  41131751
 
  ENSEMBL   UCSC 
 
  299
 
  5
 
 1540
 
 tss
 
 0.001427
 
 0.0008723
 
 Not queried
 
  0.9387878
 
       NA
 
           NA
  
 
  
 1
 
  41131309
 
  41131751
 
  ENSEMBL   UCSC 
 
  443
 
  7
 
 3128
 
 island
 
 0.008361
 
 0.0029775
 
 Not queried
 
  0.9387878
 
       NA
 
           NA
  
 
  
 17
 
  19281512
 
  19281708
 
  ENSEMBL   UCSC 
 
  197
 
  6
 
 17189
 
 tss
 
 0.004985
 
 0.0040196
 
 Not queried
 
  0.9386155
 
       NA
 
           NA
  
 
  
 8
 
  33370637
 
  33370693
 
  ENSEMBL   UCSC 
 
   57
 
  5
 
 39277
 
 gene
 
 0.013739
 
 0.0076023
 
 Not queried
 
  0.9383901
 
       NA
 
           NA
  
 
  
 5
 
   1225074
 
   1225719
 
  ENSEMBL   UCSC 
 
  646
 
 10
 
 32616
 
 island
 
 0.341188
 
 0.4149192
 
 Not queried
 
  0.9382981
 
       NA
 
           NA
  
 
  
 19
 
  13113455
 
  13113668
 
  ENSEMBL   UCSC 
 
  214
 
  4
 
 19874
 
 gene;island
 
 0.222693
 
 0.3858171
 
 Not queried
 
  0.9381916
 
       NA
 
           NA
  
 
  
 4
 
 185939339
 
 185940036
 
  ENSEMBL   UCSC 
 
  698
 
 11
 
 30699
 
 tss
 
 0.023855
 
 0.0166529
 
 Not queried
 
  0.9381309
 
       NA
 
           NA
  
 
  
 2
 
 130956098
 
 130956271
 
  ENSEMBL   UCSC 
 
  174
 
  7
 
 23626
 
 tss
 
 0.894290
 
 0.9525061
 
 Not queried
 
  0.9379006
 
       NA
 
           NA
  
 
  
 18
 
  56296243
 
  56296607
 
  ENSEMBL   UCSC 
 
  365
 
  6
 
 19293
 
 tss
 
 0.968613
 
 0.9522583
 
 Not queried
 
  0.9376996
 
       NA
 
           NA
  
 
  
 15
 
  90039805
 
  90039913
 
  ENSEMBL   UCSC 
 
  109
 
  6
 
 13888
 
 tss
 
 0.033766
 
 0.0398061
 
 Not queried
 
  0.9375048
 
       NA
 
           NA
  
 
  
 15
 
  90039409
 
  90039913
 
  ENSEMBL   UCSC 
 
  505
 
 10
 
 14437
 
 island
 
 0.052679
 
 0.0384266
 
 Not queried
 
  0.9375048
 
       NA
 
           NA
  
 
  
 1
 
 165204745
 
 165205506
 
  ENSEMBL   UCSC 
 
  762
 
 12
 
 863
 
 gene;island
 
 0.031846
 
 0.0847610
 
 Not queried
 
  0.9373102
 
       NA
 
           NA
  
 
  
 4
 
   4859935
 
   4860190
 
  ENSEMBL   UCSC 
 
  256
 
 10
 
 30269
 
 tss
 
 0.331868
 
 0.5127038
 
 Not queried
 
  0.9370726
 
       NA
 
           NA
  
 
  
 5
 
 153852554
 
 153853283
 
  ENSEMBL   UCSC 
 
  730
 
  8
 
 33328
 
 island
 
 0.037247
 
 0.1990756
 
 Not queried
 
  0.9369810
 
       NA
 
           NA
  
 
  
 2
 
 113992762
 
 113993313
 
  ENSEMBL   UCSC 
 
  552
 
  8
 
 22834
 
 gene;tss;island
 
 0.636191
 
 0.8764930
 
 Not queried
 
  0.9368335
 
       NA
 
           NA
  
 
  
 10
 
 133850441
 
 133850760
 
  ENSEMBL   UCSC 
 
  320
 
  5
 
 6284
 
 island
 
 0.049710
 
 0.0196890
 
 Not queried
 
  0.9364207
 
       NA
 
           NA
  
 
  
 1
 
  43533796
 
  43534324
 
  ENSEMBL   UCSC 
 
  529
 
  6
 
 3157
 
 island
 
 0.137685
 
 0.0562431
 
 Not queried
 
  0.9363094
 
       NA
 
           NA
  
 
  
 20
 
  21000809
 
  21001266
 
  ENSEMBL   UCSC 
 
  458
 
  4
 
 26037
 
 island
 
 0.259475
 
 0.3627684
 
 Not queried
 
  0.9361836
 
       NA
 
           NA
  
 
  
 15
 
  73735098
 
  73735476
 
  ENSEMBL   UCSC 
 
  379
 
  8
 
 13782
 
 tss
 
 0.388562
 
 0.5962621
 
 Not queried
 
  0.9359012
 
       NA
 
           NA
  
 
  
 17
 
  61498814
 
  61499248
 
  ENSEMBL   UCSC 
 
  435
 
  5
 
 16724
 
 gene
 
 0.175068
 
 0.4576848
 
 Not queried
 
  0.9358642
 
       NA
 
           NA
  
 
  
 12
 
  12871752
 
  12871924
 
  ENSEMBL   UCSC 
 
  173
 
  4
 
 9059
 
 gene;island
 
 0.006958
 
 0.0035676
 
 Not queried
 
  0.9358303
 
       NA
 
           NA
  
 
  
 9
 
 140473545
 
 140473875
 
  ENSEMBL   UCSC 
 
  331
 
  5
 
 40859
 
 tss;island
 
 0.962902
 
 0.9246575
 
 Not queried
 
  0.9357517
 
       NA
 
           NA
  
 
  
 17
 
  73521354
 
  73521755
 
  ENSEMBL   UCSC 
 
  402
 
  4
 
 17655
 
 tss;island
 
 0.003251
 
 0.0092815
 
 Not queried
 
  0.9357189
 
       NA
 
           NA
  
 
  
 20
 
   3827226
 
   3827665
 
  ENSEMBL   UCSC 
 
  440
 
 11
 
 25980
 
 island
 
 0.004275
 
 0.0047252
 
 Not queried
 
  0.9356549
 
       NA
 
           NA
  
 
  
 10
 
 129535669
 
 129535968
 
  ENSEMBL   UCSC 
 
  300
 
  5
 
 4820
 
 gene
 
 0.566559
 
 0.2958775
 
 Not queried
 
  0.9356460
 
       NA
 
           NA
  
 
  
 7
 
 145812842
 
 145813066
 
  ENSEMBL   UCSC 
 
  225
 
  5
 
 37958
 
 tss;island
 
 0.045069
 
 0.0536272
 
 Not queried
 
  0.9356444
 
       NA
 
           NA
  
 
  
 2
 
 242805838
 
 242806119
 
  ENSEMBL   UCSC 
 
  282
 
  5
 
 25311
 
 island
 
 0.002837
 
 0.0022673
 
 Not queried
 
  0.9355989
 
       NA
 
           NA
  
 
  
 6
 
  32410690
 
  32411185
 
  ENSEMBL   UCSC 
 
  496
 
  6
 
 34114
 
 gene
 
 0.819201
 
 0.7734984
 
 Not queried
 
  0.9354830
 
       NA
 
           NA
  
 
  
 7
 
 120497998
 
 120498369
 
  ENSEMBL   UCSC 
 
  372
 
  5
 
 38863
 
 island
 
 0.003149
 
 0.0059536
 
 Not queried
 
  0.9354341
 
       NA
 
           NA
  
 
  
 1
 
 149223316
 
 149223634
 
  ENSEMBL   UCSC 
 
  319
 
  4
 
 3706
 
 island
 
 0.210727
 
 0.0613510
 
 Not queried
 
  0.9354274
 
       NA
 
           NA
  
 
  
 17
 
  27717641
 
  27717927
 
  ENSEMBL   UCSC 
 
  287
 
  8
 
 17233
 
 tss
 
 0.002308
 
 0.0027240
 
 Not queried
 
  0.9354094
 
       NA
 
           NA
  
 
  
 7
 
 134250141
 
 134250223
 
  ENSEMBL   UCSC 
 
   83
 
  4
 
 37912
 
 tss
 
 0.368517
 
 0.5936125
 
 Not queried
 
  0.9352389
 
       NA
 
           NA
  
 
  
 19
 
  36359211
 
  36359327
 
  ENSEMBL   UCSC 
 
  117
 
  6
 
 20717
 
 tss
 
 0.030560
 
 0.1758232
 
 Not queried
 
  0.9350597
 
       NA
 
           NA
  
 
  
 8
 
   9791214
 
   9791557
 
  ENSEMBL   UCSC 
 
  344
 
  4
 
 40067
 
 island
 
 0.071589
 
 0.1010642
 
 Not queried
 
  0.9347987
 
       NA
 
           NA
  
 
  
 6
 
  28543508
 
  28543693
 
  ENSEMBL   UCSC 
 
  186
 
  5
 
 33637
 
 gene
 
 0.948749
 
 0.9812253
 
 Not queried
 
  0.9347931
 
       NA
 
           NA
  
 
  
 12
 
  57118908
 
  57119373
 
  ENSEMBL   UCSC 
 
  466
 
 10
 
 10542
 
 island
 
 0.005056
 
 0.0051793
 
 Not queried
 
  0.9347813
 
       NA
 
           NA
  
 
  
 20
 
  55101001
 
  55101089
 
  ENSEMBL   UCSC 
 
   89
 
  4
 
 25837
 
 tss
 
 0.679009
 
 0.8056235
 
 Not queried
 
  0.9347461
 
       NA
 
           NA
  
 
  
 2
 
 206790633
 
 206790898
 
  ENSEMBL   UCSC 
 
  266
 
  4
 
 25029
 
 island
 
 0.007540
 
 0.0079338
 
 Not queried
 
  0.9347299
 
       NA
 
           NA
  
 
  
 14
 
  89290504
 
  89290958
 
  ENSEMBL   UCSC 
 
  455
 
  8
 
 12548
 
 tss
 
 0.007972
 
 0.0041660
 
 Not queried
 
  0.9343849
 
       NA
 
           NA
  
 
  
 14
 
  89290504
 
  89291237
 
  ENSEMBL   UCSC 
 
  734
 
 10
 
 13063
 
 island
 
 0.007972
 
 0.0041660
 
 Not queried
 
  0.9343849
 
       NA
 
           NA
  
 
  
 2
 
 201450323
 
 201450743
 
  ENSEMBL   UCSC 
 
  421
 
 11
 
 25003
 
 island
 
 0.026589
 
 0.0185010
 
 Not queried
 
  0.9343108
 
       NA
 
           NA
  
 
  
 4
 
  18024261
 
  18024445
 
  ENSEMBL   UCSC 
 
  185
 
  4
 
 30321
 
 tss;island
 
 0.010405
 
 0.0184841
 
 Not queried
 
  0.9342784
 
       NA
 
           NA
  
 
  
 10
 
 134095646
 
 134096014
 
  ENSEMBL   UCSC 
 
  369
 
  5
 
 4860
 
 gene;island
 
 0.403684
 
 0.4621787
 
 Not queried
 
  0.9342426
 
       NA
 
           NA
  
 
  
 5
 
  31855088
 
  31855477
 
  ENSEMBL   UCSC 
 
  390
 
  4
 
 31577
 
 gene;island
 
 0.022283
 
 0.0462042
 
 Not queried
 
  0.9340432
 
       NA
 
           NA
  
 
  
 16
 
  88832476
 
  88832700
 
  ENSEMBL   UCSC 
 
  225
 
  6
 
 14918
 
 gene
 
 0.906365
 
 0.9741496
 
 Not queried
 
  0.9339029
 
       NA
 
           NA
  
 
  
 2
 
  26624450
 
  26624767
 
  ENSEMBL   UCSC 
 
  318
 
  7
 
 23243
 
 tss
 
 0.384577
 
 0.5355788
 
 Not queried
 
  0.9338165
 
       NA
 
           NA
  
 
  
 19
 
   5832033
 
   5832343
 
  ENSEMBL   UCSC 
 
  311
 
  4
 
 19756
 
 gene;island
 
 0.778988
 
 0.8013614
 
 Not queried
 
  0.9337957
 
       NA
 
           NA
  
 
  
 6
 
 166868856
 
 166869078
 
  ENSEMBL   UCSC 
 
  223
 
  4
 
 34544
 
 gene;island
 
 0.785472
 
 0.8692397
 
 Not queried
 
  0.9334840
 
       NA
 
           NA
  
 
  
 7
 
  45808481
 
  45808808
 
  ENSEMBL   UCSC 
 
  328
 
  8
 
 38476
 
 island
 
 0.025076
 
 0.0144599
 
 Not queried
 
  0.9334206
 
       NA
 
           NA
  
 
  
 17
 
    183931
 
    184131
 
  ENSEMBL   UCSC 
 
  201
 
  4
 
 16291
 
 gene;island
 
 0.046793
 
 0.1096657
 
 Not queried
 
  0.9334139
 
       NA
 
           NA
  
 
  
 6
 
  41007450
 
  41007692
 
  ENSEMBL   UCSC 
 
  243
 
  4
 
 34962
 
 tss
 
 0.703119
 
 0.8451153
 
 Not queried
 
  0.9334133
 
       NA
 
           NA
  
 
  
 2
 
  81694296
 
  81694608
 
  ENSEMBL   UCSC 
 
  313
 
  5
 
 24466
 
 island
 
 0.715549
 
 0.9020526
 
 Not queried
 
  0.9332969
 
       NA
 
           NA
  
 
  
 22
 
  42915845
 
  42915908
 
  ENSEMBL   UCSC 
 
   64
 
  5
 
 27192
 
 tss
 
 0.010501
 
 0.0059686
 
 Not queried
 
  0.9332930
 
       NA
 
           NA
  
 
  
 7
 
 156111254
 
 156111445
 
  ENSEMBL   UCSC 
 
  192
 
  4
 
 39083
 
 island
 
 0.480031
 
 0.6299952
 
 Not queried
 
  0.9331969
 
       NA
 
           NA
  
 
  
 4
 
   8594324
 
   8594549
 
  ENSEMBL   UCSC 
 
  226
 
  4
 
 30924
 
 island
 
 0.019968
 
 0.0735261
 
 Not queried
 
  0.9330496
 
       NA
 
           NA
  
 
  
 18
 
    904851
 
    905252
 
  ENSEMBL   UCSC 
 
  402
 
 12
 
 19156
 
 tss
 
 0.018166
 
 0.0408514
 
 Not queried
 
  0.9329987
 
       NA
 
           NA
  
 
  
 16
 
  30366237
 
  30366449
 
  ENSEMBL   UCSC 
 
  213
 
  4
 
 14719
 
 gene;island
 
 0.004744
 
 0.0035700
 
 Not queried
 
  0.9327869
 
       NA
 
           NA
  
 
  
 10
 
 104613767
 
 104613934
 
  ENSEMBL   UCSC 
 
  168
 
  6
 
 5331
 
 tss
 
 0.958606
 
 0.9170252
 
 Not queried
 
  0.9327557
 
       NA
 
           NA
  
 
  
 2
 
 232395269
 
 232395463
 
  ENSEMBL   UCSC 
 
  195
 
  6
 
 23942
 
 tss;island
 
 0.009557
 
 0.0036085
 
 Not queried
 
  0.9326631
 
       NA
 
           NA
  
 
  
 7
 
   4242769
 
   4243019
 
  ENSEMBL   UCSC 
 
  251
 
  4
 
 36833
 
 gene
 
 0.226112
 
 0.3590422
 
 Not queried
 
  0.9325905
 
       NA
 
           NA
  
 
  
 11
 
  66103823
 
  66104174
 
  ENSEMBL   UCSC 
 
  352
 
  7
 
 8509
 
 island
 
 0.039747
 
 0.0727364
 
 Not queried
 
  0.9325871
 
       NA
 
           NA
  
 
  
 14
 
 101489985
 
 101490253
 
  ENSEMBL   UCSC 
 
  269
 
 10
 
 12620
 
 tss
 
 0.499122
 
 0.7367073
 
 Not queried
 
  0.9321729
 
       NA
 
           NA
  
 
  
 15
 
  25958878
 
  25959239
 
  ENSEMBL   UCSC 
 
  362
 
  5
 
 13258
 
 gene;island
 
 0.490063
 
 0.8101977
 
 Not queried
 
  0.9320326
 
       NA
 
           NA
  
 
  
 18
 
  54318615
 
  54318940
 
  ENSEMBL   UCSC 
 
  326
 
  4
 
 19119
 
 gene
 
 0.028359
 
 0.0144795
 
 Not queried
 
  0.9320244
 
       NA
 
           NA
  
 
  
 11
 
  14280742
 
  14281053
 
  ENSEMBL   UCSC 
 
  312
 
  4
 
 6512
 
 gene;island
 
 0.710086
 
 0.5634453
 
 Not queried
 
  0.9319170
 
       NA
 
           NA
  
 
  
 22
 
  49637633
 
  49637888
 
  ENSEMBL   UCSC 
 
  256
 
  4
 
 27603
 
 island
 
 0.710478
 
 0.8716936
 
 Not queried
 
  0.9318434
 
       NA
 
           NA
  
 
  
 1
 
 111217691
 
 111217785
 
  ENSEMBL   UCSC 
 
   95
 
  4
 
 1818
 
 tss
 
 0.031704
 
 0.0824617
 
 Not queried
 
  0.9318341
 
       NA
 
           NA
  
 
  
 1
 
 235292131
 
 235292449
 
  ENSEMBL   UCSC 
 
  319
 
  8
 
 2331
 
 tss;island
 
 0.006036
 
 0.0050064
 
 Not queried
 
  0.9317305
 
       NA
 
           NA
  
 
  
 7
 
  55000889
 
  55001207
 
  ENSEMBL   UCSC 
 
  319
 
  4
 
 38502
 
 island
 
 0.092219
 
 0.0370156
 
 Not queried
 
  0.9316969
 
       NA
 
           NA
  
 
  
 17
 
  48711902
 
  48712212
 
  ENSEMBL   UCSC 
 
  311
 
  8
 
 17504
 
 tss
 
 0.052152
 
 0.0295925
 
 Not queried
 
  0.9316035
 
       NA
 
           NA
  
 
  
 17
 
  48711902
 
  48712370
 
  ENSEMBL   UCSC 
 
  469
 
 10
 
 18558
 
 island
 
 0.064621
 
 0.0325051
 
 Not queried
 
  0.9316035
 
       NA
 
           NA
  
 
  
 6
 
  70576168
 
  70576388
 
  ENSEMBL   UCSC 
 
  221
 
  4
 
 35056
 
 tss
 
 0.001775
 
 0.0009825
 
 Not queried
 
  0.9315744
 
       NA
 
           NA
  
 
  
 7
 
 107220968
 
 107221327
 
  ENSEMBL   UCSC 
 
  360
 
  5
 
 37181
 
 gene;island
 
 0.007780
 
 0.0061568
 
 Not queried
 
  0.9311338
 
       NA
 
           NA
  
 
  
 20
 
  30639705
 
  30640041
 
  ENSEMBL   UCSC 
 
  337
 
  8
 
 25671
 
 tss
 
 0.015515
 
 0.0156579
 
 Not queried
 
  0.9310765
 
       NA
 
           NA
  
 
  
 7
 
  20818657
 
  20818928
 
  ENSEMBL   UCSC 
 
  272
 
  6
 
 38292
 
 island
 
 0.157241
 
 0.4893628
 
 Not queried
 
  0.9308522
 
       NA
 
           NA
  
 
  
 17
 
  21279561
 
  21279696
 
  ENSEMBL   UCSC 
 
  136
 
  5
 
 17203
 
 tss
 
 0.012349
 
 0.0126397
 
 Not queried
 
  0.9306059
 
       NA
 
           NA
  
 
  
 8
 
  37797680
 
  37797828
 
  ENSEMBL   UCSC 
 
  149
 
  5
 
 39649
 
 tss
 
 0.570032
 
 0.5839165
 
 Not queried
 
  0.9306006
 
       NA
 
           NA
  
 
  
 1
 
  29101849
 
  29102153
 
  ENSEMBL   UCSC 
 
  305
 
  5
 
 2952
 
 island
 
 0.101289
 
 0.0424621
 
 Not queried
 
  0.9305929
 
       NA
 
           NA
  
 
  
 12
 
  38710408
 
  38710504
 
  ENSEMBL   UCSC 
 
   97
 
  6
 
 9635
 
 tss
 
 0.004187
 
 0.0034116
 
 Not queried
 
  0.9302963
 
       NA
 
           NA
  
 
  
 2
 
 177014467
 
 177015125
 
  ENSEMBL   UCSC 
 
  659
 
 13
 
 23740
 
 tss;island
 
 0.281456
 
 0.1667073
 
 Not queried
 
  0.9302958
 
       NA
 
           NA
  
 
  
 2
 
  10091661
 
  10091897
 
  ENSEMBL   UCSC 
 
  237
 
  6
 
 24059
 
 island
 
 0.019183
 
 0.0106179
 
 Not queried
 
  0.9302273
 
       NA
 
           NA
  
 
  
 8
 
  63998640
 
  63998778
 
  ENSEMBL   UCSC 
 
  139
 
  6
 
 39731
 
 tss
 
 0.021832
 
 0.0141715
 
 Not queried
 
  0.9302156
 
       NA
 
           NA
  
 
  
 6
 
  28553927
 
  28555079
 
  ENSEMBL   UCSC 
 
 1153
 
 20
 
 33639
 
 gene
 
 0.060742
 
 0.0243027
 
 Not queried
 
  0.9300581
 
       NA
 
           NA
  
 
  
 1
 
 115828679
 
 115829610
 
  ENSEMBL   UCSC 
 
  932
 
  8
 
 703
 
 gene
 
 0.565885
 
 0.6585975
 
 Not queried
 
  0.9300236
 
       NA
 
           NA
  
 
  
 19
 
  58629719
 
  58630612
 
  ENSEMBL   UCSC 
 
  894
 
 10
 
 22494
 
 island
 
 0.523279
 
 0.6124219
 
 Not queried
 
  0.9299674
 
       NA
 
           NA
  
 
  
 1
 
 235267474
 
 235267828
 
  ENSEMBL   UCSC 
 
  355
 
  6
 
 4342
 
 island
 
 0.026485
 
 0.0053131
 
 Not queried
 
  0.9297562
 
       NA
 
           NA
  
 
  
 19
 
   6768010
 
   6768251
 
  ENSEMBL   UCSC 
 
  242
 
  4
 
 20394
 
 tss;island
 
 0.014124
 
 0.0267563
 
 Not queried
 
  0.9296853
 
       NA
 
           NA
  
 
  
 16
 
   3096477
 
   3096662
 
  ENSEMBL   UCSC 
 
  186
 
  5
 
 15049
 
 tss
 
 0.006928
 
 0.0433309
 
 Not queried
 
  0.9294694
 
       NA
 
           NA
  
 
  
 1
 
   6537569
 
   6537840
 
  ENSEMBL   UCSC 
 
  272
 
  4
 
 175
 
 gene
 
 0.576605
 
 0.6110025
 
 Not queried
 
  0.9292638
 
       NA
 
           NA
  
 
  
 7
 
  27205159
 
  27205658
 
  ENSEMBL   UCSC 
 
  500
 
 10
 
 37496
 
 tss
 
 0.021304
 
 0.0290725
 
 Not queried
 
  0.9292468
 
       NA
 
           NA
  
 
  
 15
 
  29213626
 
  29213748
 
  ENSEMBL   UCSC 
 
  123
 
  4
 
 13545
 
 tss
 
 0.071279
 
 0.3417538
 
 Not queried
 
  0.9291240
 
       NA
 
           NA
  
 
  
 17
 
  39216457
 
  39216687
 
  ENSEMBL   UCSC 
 
  231
 
  4
 
 17337
 
 tss
 
 0.492811
 
 0.6039583
 
 Not queried
 
  0.9290925
 
       NA
 
           NA
  
 
  
 3
 
   8810980
 
   8811758
 
  ENSEMBL   UCSC 
 
  779
 
  8
 
 28851
 
 island
 
 0.034812
 
 0.0238798
 
 Not queried
 
  0.9290837
 
       NA
 
           NA
  
 
  
 5
 
 150325954
 
 150326642
 
  ENSEMBL   UCSC 
 
  689
 
 11
 
 33314
 
 island
 
 0.205757
 
 0.4351022
 
 Not queried
 
  0.9287753
 
       NA
 
           NA
  
 
  
 3
 
 157155088
 
 157155476
 
  ENSEMBL   UCSC 
 
  389
 
  5
 
 28007
 
 gene;island
 
 0.035631
 
 0.0143521
 
 Not queried
 
  0.9286649
 
       NA
 
           NA
  
 
  
 1
 
  31158026
 
  31158299
 
  ENSEMBL   UCSC 
 
  274
 
  5
 
 2964
 
 island
 
 0.018982
 
 0.0425308
 
 Not queried
 
  0.9281548
 
       NA
 
           NA
  
 
  
 3
 
 131221830
 
 131222106
 
  ENSEMBL   UCSC 
 
  277
 
  7
 
 28595
 
 tss
 
 0.002793
 
 0.0048047
 
 Not queried
 
  0.9281230
 
       NA
 
           NA
  
 
  
 2
 
  37384227
 
  37384845
 
  ENSEMBL   UCSC 
 
  619
 
 11
 
 23304
 
 tss
 
 0.009589
 
 0.0057604
 
 Not queried
 
  0.9278592
 
       NA
 
           NA
  
 
  
 3
 
  96495651
 
  96496135
 
  ENSEMBL   UCSC 
 
  485
 
  5
 
 29309
 
 island
 
 0.820875
 
 0.8527539
 
 Not queried
 
  0.9277107
 
       NA
 
           NA
  
 
  
 11
 
  59328249
 
  59328586
 
  ENSEMBL   UCSC 
 
  338
 
  4
 
 8306
 
 island
 
 0.005407
 
 0.0033430
 
 Not queried
 
  0.9276070
 
       NA
 
           NA
  
 
  
 4
 
   1366463
 
   1366826
 
  ENSEMBL   UCSC 
 
  364
 
  4
 
 29872
 
 gene;island
 
 0.962763
 
 0.9075956
 
 Not queried
 
  0.9275305
 
       NA
 
           NA
  
 
  
 1
 
 209825672
 
 209825747
 
  ENSEMBL   UCSC 
 
   76
 
  4
 
 985
 
 gene
 
 0.902941
 
 0.9500592
 
 Not queried
 
  0.9274524
 
       NA
 
           NA
  
 
  
 7
 
  77669697
 
  77670191
 
  ENSEMBL   UCSC 
 
  495
 
  5
 
 37064
 
 gene;island
 
 0.346414
 
 0.2179632
 
 Not queried
 
  0.9273262
 
       NA
 
           NA
  
 
  
 19
 
  36138893
 
  36139233
 
  ENSEMBL   UCSC 
 
  341
 
  7
 
 21911
 
 island
 
 0.013005
 
 0.0078831
 
 Not queried
 
  0.9271592
 
       NA
 
           NA
  
 
  
 4
 
 159593022
 
 159593173
 
  ENSEMBL   UCSC 
 
  152
 
  5
 
 30164
 
 gene
 
 0.001236
 
 0.0003735
 
 Not queried
 
  0.9270968
 
       NA
 
           NA
  
 
  
 17
 
  42462958
 
  42463277
 
  ENSEMBL   UCSC 
 
  320
 
  4
 
 16618
 
 gene;island
 
 0.008957
 
 0.0172931
 
 Not queried
 
  0.9269275
 
       NA
 
           NA
  
 
  
 2
 
  26624450
 
  26625047
 
  ENSEMBL   UCSC 
 
  598
 
 11
 
 24152
 
 island
 
 0.334761
 
 0.4398002
 
 Not queried
 
  0.9267946
 
       NA
 
           NA
  
 
  
 5
 
 161494711
 
 161494810
 
  ENSEMBL   UCSC 
 
  100
 
  4
 
 31881
 
 gene
 
 0.003558
 
 0.0022297
 
 Not queried
 
  0.9267922
 
       NA
 
           NA
  
 
  
 1
 
  38099874
 
  38100467
 
  ENSEMBL   UCSC 
 
  594
 
  6
 
 435
 
 gene;island
 
 0.208506
 
 0.2925047
 
 Not queried
 
  0.9266384
 
       NA
 
           NA
  
 
  
 17
 
  50236344
 
  50236765
 
  ENSEMBL   UCSC 
 
  422
 
  4
 
 16697
 
 gene;tss;island
 
 0.079209
 
 0.0269001
 
 Not queried
 
  0.9263546
 
       NA
 
           NA
  
 
  
 3
 
 138739807
 
 138740022
 
  ENSEMBL   UCSC 
 
  216
 
  6
 
 28628
 
 tss
 
 0.731458
 
 0.5390967
 
 Not queried
 
  0.9263515
 
       NA
 
           NA
  
 
  
 8
 
 144099482
 
 144099719
 
  ENSEMBL   UCSC 
 
  238
 
  4
 
 39471
 
 gene
 
 0.058055
 
 0.0343135
 
 Not queried
 
  0.9263404
 
       NA
 
           NA
  
 
  
 17
 
  35716004
 
  35716418
 
  ENSEMBL   UCSC 
 
  415
 
  6
 
 16530
 
 gene;island
 
 0.005301
 
 0.0040913
 
 Not queried
 
  0.9261595
 
       NA
 
           NA
  
 
  
 12
 
 108955791
 
 108956318
 
  ENSEMBL   UCSC 
 
  528
 
  8
 
 9983
 
 tss;island
 
 0.002819
 
 0.0035751
 
 Not queried
 
  0.9260841
 
       NA
 
           NA
  
 
  
 2
 
  11051728
 
  11051991
 
  ENSEMBL   UCSC 
 
  264
 
  5
 
 23186
 
 tss
 
 0.032610
 
 0.0187825
 
 Not queried
 
  0.9252714
 
       NA
 
           NA
  
 
  
 2
 
  11051728
 
  11052102
 
  ENSEMBL   UCSC 
 
  375
 
  7
 
 24074
 
 island
 
 0.032610
 
 0.0180739
 
 Not queried
 
  0.9252714
 
       NA
 
           NA
  
 
  
 3
 
 182971430
 
 182971663
 
  ENSEMBL   UCSC 
 
  234
 
  4
 
 28050
 
 gene;island
 
 0.015473
 
 0.0114872
 
 Not queried
 
  0.9250167
 
       NA
 
           NA
  
 
  
 10
 
 126331180
 
 126331425
 
  ENSEMBL   UCSC 
 
  246
 
  4
 
 4808
 
 gene
 
 0.953472
 
 0.9576144
 
 Not queried
 
  0.9249893
 
       NA
 
           NA
  
 
  
 17
 
  19648846
 
  19649293
 
  ENSEMBL   UCSC 
 
  448
 
 10
 
 17194
 
 tss
 
 0.844694
 
 0.9009678
 
 Not queried
 
  0.9248317
 
       NA
 
           NA
  
 
  
 11
 
 125772925
 
 125773242
 
  ENSEMBL   UCSC 
 
  318
 
  6
 
 7794
 
 tss;island
 
 0.006815
 
 0.0031257
 
 Not queried
 
  0.9248264
 
       NA
 
           NA
  
 
  
 14
 
  70316898
 
  70317239
 
  ENSEMBL   UCSC 
 
  342
 
  5
 
 12973
 
 island
 
 0.735827
 
 0.9195268
 
 Not queried
 
  0.9245449
 
       NA
 
           NA
  
 
  
 16
 
   1199836
 
   1200097
 
  ENSEMBL   UCSC 
 
  262
 
  4
 
 15519
 
 island
 
 0.848844
 
 0.9119686
 
 Not queried
 
  0.9245295
 
       NA
 
           NA
  
 
  
 12
 
 121533475
 
 121534028
 
  ENSEMBL   UCSC 
 
  554
 
  5
 
 10906
 
 island
 
 0.012770
 
 0.0260888
 
 Not queried
 
  0.9243780
 
       NA
 
           NA
  
 
  
 3
 
 147125712
 
 147127193
 
  ENSEMBL   UCSC 
 
 1482
 
 28
 
 29571
 
 island
 
 0.416655
 
 0.6182111
 
 Not queried
 
  0.9243539
 
       NA
 
           NA
  
 
  
 8
 
  95274566
 
  95274933
 
  ENSEMBL   UCSC 
 
  368
 
  6
 
 39814
 
 tss
 
 0.098068
 
 0.0311675
 
 Not queried
 
  0.9242940
 
       NA
 
           NA
  
 
  
 12
 
 113229582
 
 113230065
 
  ENSEMBL   UCSC 
 
  484
 
  8
 
 9317
 
 gene
 
 0.031030
 
 0.0425914
 
 Not queried
 
  0.9238149
 
       NA
 
           NA
  
 
  
 7
 
  27260102
 
  27260585
 
  ENSEMBL   UCSC 
 
  484
 
  6
 
 38363
 
 island
 
 0.079016
 
 0.2017796
 
 Not queried
 
  0.9237188
 
       NA
 
           NA
  
 
  
 1
 
  70035080
 
  70035479
 
  ENSEMBL   UCSC 
 
  400
 
  4
 
 3376
 
 island
 
 0.006321
 
 0.0142714
 
 Not queried
 
  0.9237036
 
       NA
 
           NA
  
 
  
 5
 
 175792454
 
 175792510
 
  ENSEMBL   UCSC 
 
   57
 
  5
 
 33425
 
 island
 
 0.006295
 
 0.0053897
 
 Not queried
 
  0.9236113
 
       NA
 
           NA
  
 
  
 7
 
   2271673
 
   2271814
 
  ENSEMBL   UCSC 
 
  142
 
  4
 
 36795
 
 gene;island
 
 0.011926
 
 0.0120872
 
 Not queried
 
  0.9231670
 
       NA
 
           NA
  
 
  
 11
 
  71159218
 
  71159392
 
  ENSEMBL   UCSC 
 
  175
 
  4
 
 6802
 
 gene;island
 
 0.001660
 
 0.0009899
 
 Not queried
 
  0.9231496
 
       NA
 
           NA
  
 
  
 16
 
  89574433
 
  89574810
 
  ENSEMBL   UCSC 
 
  378
 
  7
 
 16258
 
 island
 
 0.007799
 
 0.0047629
 
 Not queried
 
  0.9230786
 
       NA
 
           NA
  
 
  
 20
 
  48184639
 
  48184832
 
  ENSEMBL   UCSC 
 
  194
 
  4
 
 26235
 
 island
 
 0.008484
 
 0.0223541
 
 Not queried
 
  0.9230216
 
       NA
 
           NA
  
 
  
 1
 
 245134348
 
 245134786
 
  ENSEMBL   UCSC 
 
  439
 
  5
 
 1116
 
 gene;island
 
 0.017761
 
 0.0079864
 
 Not queried
 
  0.9222458
 
       NA
 
           NA
  
 
  
 19
 
   6712321
 
   6712593
 
  ENSEMBL   UCSC 
 
  273
 
  7
 
 19767
 
 gene;island
 
 0.763113
 
 0.5508817
 
 Not queried
 
  0.9221143
 
       NA
 
           NA
  
 
  
 10
 
  54073878
 
  54074250
 
  ENSEMBL   UCSC 
 
  373
 
  5
 
 5751
 
 island
 
 0.117608
 
 0.0672037
 
 Not queried
 
  0.9220485
 
       NA
 
           NA
  
 
  
 5
 
  24644988
 
  24645147
 
  ENSEMBL   UCSC 
 
  160
 
  5
 
 32002
 
 tss
 
 0.015676
 
 0.0921047
 
 Not queried
 
  0.9218810
 
       NA
 
           NA
  
 
  
 13
 
  79168044
 
  79168370
 
  ENSEMBL   UCSC 
 
  327
 
  5
 
 11791
 
 island
 
 0.118385
 
 0.0390637
 
 Not queried
 
  0.9218695
 
       NA
 
           NA
  
 
  
 1
 
  17380506
 
  17380646
 
  ENSEMBL   UCSC 
 
  141
 
  5
 
 265
 
 gene
 
 0.005740
 
 0.0032822
 
 Not queried
 
  0.9218296
 
       NA
 
           NA
  
 
  
 12
 
  48152204
 
  48152585
 
  ENSEMBL   UCSC 
 
  382
 
  6
 
 9105
 
 gene;tss;island
 
 0.010325
 
 0.0239287
 
 Not queried
 
  0.9217752
 
       NA
 
           NA
  
 
  
 8
 
  41505411
 
  41505696
 
  ENSEMBL   UCSC 
 
  286
 
  4
 
 39672
 
 tss;island
 
 0.286077
 
 0.5332872
 
 Not queried
 
  0.9216903
 
       NA
 
           NA
  
 
  
 7
 
 121950198
 
 121950506
 
  ENSEMBL   UCSC 
 
  309
 
  4
 
 38871
 
 island
 
 0.035182
 
 0.0756868
 
 Not queried
 
  0.9216420
 
       NA
 
           NA
  
 
  
 16
 
   2848117
 
   2848409
 
  ENSEMBL   UCSC 
 
  293
 
  5
 
 15036
 
 tss
 
 0.408061
 
 0.6134922
 
 Not queried
 
  0.9215971
 
       NA
 
           NA
  
 
  
 1
 
   3772978
 
   3773316
 
  ENSEMBL   UCSC 
 
  339
 
  4
 
 148
 
 gene;tss;island
 
 0.002098
 
 0.0033808
 
 Not queried
 
  0.9215328
 
       NA
 
           NA
  
 
  
 3
 
 125803249
 
 125803341
 
  ENSEMBL   UCSC 
 
   93
 
  4
 
 27928
 
 gene;tss;island
 
 0.016393
 
 0.0090212
 
 Not queried
 
  0.9213048
 
       NA
 
           NA
  
 
  
 17
 
  46691820
 
  46692248
 
  ENSEMBL   UCSC 
 
  429
 
  5
 
 16660
 
 gene
 
 0.029319
 
 0.0614566
 
 Not queried
 
  0.9213037
 
       NA
 
           NA
  
 
  
 19
 
  11450511
 
  11450739
 
  ENSEMBL   UCSC 
 
  229
 
  5
 
 20467
 
 tss;island
 
 0.020497
 
 0.0170191
 
 Not queried
 
  0.9211514
 
       NA
 
           NA
  
 
  
 11
 
   2017362
 
   2018247
 
  ENSEMBL   UCSC 
 
  886
 
 13
 
 6443
 
 gene;island
 
 0.654877
 
 0.8509327
 
 Not queried
 
  0.9208933
 
       NA
 
           NA
  
 
  
 20
 
  24929607
 
  24929822
 
  ENSEMBL   UCSC 
 
  216
 
  5
 
 25643
 
 tss
 
 0.025085
 
 0.0538533
 
 Not queried
 
  0.9208695
 
       NA
 
           NA
  
 
  
 1
 
 202896457
 
 202896601
 
  ENSEMBL   UCSC 
 
  145
 
  4
 
 2185
 
 tss
 
 0.012452
 
 0.0167564
 
 Not queried
 
  0.9207417
 
       NA
 
           NA
  
 
  
 6
 
 168078772
 
 168079212
 
  ENSEMBL   UCSC 
 
  441
 
  5
 
 36644
 
 island
 
 0.026145
 
 0.0138958
 
 Not queried
 
  0.9206895
 
       NA
 
           NA
  
 
  
 10
 
  69523794
 
  69524272
 
  ENSEMBL   UCSC 
 
  479
 
  7
 
 5783
 
 island
 
 0.005147
 
 0.0037791
 
 Not queried
 
  0.9205165
 
       NA
 
           NA
  
 
  
 2
 
 139660149
 
 139660341
 
  ENSEMBL   UCSC 
 
  193
 
  4
 
 24765
 
 island
 
 0.742130
 
 0.8578042
 
 Not queried
 
  0.9201673
 
       NA
 
           NA
  
 
  
 16
 
  27279496
 
  27279887
 
  ENSEMBL   UCSC 
 
  392
 
  4
 
 14701
 
 gene;island
 
 0.209802
 
 0.3774441
 
 Not queried
 
  0.9201081
 
       NA
 
           NA
  
 
  
 11
 
  17498236
 
  17498619
 
  ENSEMBL   UCSC 
 
  384
 
  9
 
 8100
 
 island
 
 0.057078
 
 0.1484435
 
 Not queried
 
  0.9199253
 
       NA
 
           NA
  
 
  
 2
 
 217236783
 
 217237043
 
  ENSEMBL   UCSC 
 
  261
 
 10
 
 23867
 
 tss
 
 0.034426
 
 0.0175050
 
 Not queried
 
  0.9198363
 
       NA
 
           NA
  
 
  
 2
 
  23784893
 
  23785338
 
  ENSEMBL   UCSC 
 
  446
 
  5
 
 22593
 
 gene;island
 
 0.937912
 
 0.9124807
 
 Not queried
 
  0.9197852
 
       NA
 
           NA
  
 
  
 7
 
 101005832
 
 101006089
 
  ENSEMBL   UCSC 
 
  258
 
  9
 
 37802
 
 tss;island
 
 0.060347
 
 0.0118093
 
 Not queried
 
  0.9193367
 
       NA
 
           NA
  
 
  
 7
 
  96643454
 
  96643821
 
  ENSEMBL   UCSC 
 
  368
 
  5
 
 37734
 
 tss;island
 
 0.011473
 
 0.0109254
 
 Not queried
 
  0.9192926
 
       NA
 
           NA
  
 
  
 8
 
  27950625
 
  27950972
 
  ENSEMBL   UCSC 
 
  348
 
  5
 
 39260
 
 gene
 
 0.006339
 
 0.0015574
 
 Not queried
 
  0.9192776
 
       NA
 
           NA
  
 
  
 6
 
  10881782
 
  10882336
 
  ENSEMBL   UCSC 
 
  555
 
 11
 
 35439
 
 island
 
 0.042872
 
 0.0573023
 
 Not queried
 
  0.9191866
 
       NA
 
           NA
  
 
  
 2
 
 102314465
 
 102314757
 
  ENSEMBL   UCSC 
 
  293
 
  5
 
 24572
 
 island
 
 0.010724
 
 0.0204032
 
 Not queried
 
  0.9190728
 
       NA
 
           NA
  
 
  
 17
 
  79604097
 
  79604385
 
  ENSEMBL   UCSC 
 
  289
 
  8
 
 18932
 
 island
 
 0.009115
 
 0.0061801
 
 Not queried
 
  0.9189101
 
       NA
 
           NA
  
 
  
 14
 
  85996101
 
  85996499
 
  ENSEMBL   UCSC 
 
  399
 
 10
 
 13053
 
 island
 
 0.086680
 
 0.1681176
 
 Not queried
 
  0.9188515
 
       NA
 
           NA
  
 
  
 10
 
 133955398
 
 133955570
 
  ENSEMBL   UCSC 
 
  173
 
  5
 
 4855
 
 gene;island
 
 0.724052
 
 0.8317925
 
 Not queried
 
  0.9187919
 
       NA
 
           NA
  
 
  
 17
 
  15164050
 
  15164292
 
  ENSEMBL   UCSC 
 
  243
 
  8
 
 16453
 
 gene;island
 
 0.009641
 
 0.0132233
 
 Not queried
 
  0.9187779
 
       NA
 
           NA
  
 
  
 4
 
 144480206
 
 144480561
 
  ENSEMBL   UCSC 
 
  356
 
 11
 
 30614
 
 tss
 
 0.008762
 
 0.0066957
 
 Not queried
 
  0.9186747
 
       NA
 
           NA
  
 
  
 3
 
 142681401
 
 142681704
 
  ENSEMBL   UCSC 
 
  304
 
  4
 
 27977
 
 gene;island
 
 0.867726
 
 0.9311370
 
 Not queried
 
  0.9185268
 
       NA
 
           NA
  
 
  
 2
 
    628881
 
    629121
 
  ENSEMBL   UCSC 
 
  241
 
  5
 
 24009
 
 island
 
 0.468495
 
 0.6248791
 
 Not queried
 
  0.9184301
 
       NA
 
           NA
  
 
  
 15
 
  96868857
 
  96869220
 
  ENSEMBL   UCSC 
 
  364
 
  8
 
 14485
 
 island
 
 0.017759
 
 0.0493876
 
 Not queried
 
  0.9183384
 
       NA
 
           NA
  
 
  
 3
 
 137483328
 
 137483555
 
  ENSEMBL   UCSC 
 
  228
 
  6
 
 28616
 
 tss
 
 0.307184
 
 0.1025495
 
 Not queried
 
  0.9181133
 
       NA
 
           NA
  
 
  
 1
 
  39339062
 
  39339683
 
  ENSEMBL   UCSC 
 
  622
 
  9
 
 1518
 
 tss
 
 0.009010
 
 0.0074571
 
 Not queried
 
  0.9180490
 
       NA
 
           NA
  
 
  
 19
 
  43774820
 
  43774982
 
  ENSEMBL   UCSC 
 
  163
 
  4
 
 20827
 
 tss
 
 0.300056
 
 0.5265930
 
 Not queried
 
  0.9180134
 
       NA
 
           NA
  
 
  
 19
 
  50173392
 
  50173685
 
  ENSEMBL   UCSC 
 
  294
 
  4
 
 20160
 
 gene;island
 
 0.916824
 
 0.9551837
 
 Not queried
 
  0.9179288
 
       NA
 
           NA
  
 
  
 16
 
  56228442
 
  56228901
 
  ENSEMBL   UCSC 
 
  460
 
  9
 
 15243
 
 tss
 
 0.005095
 
 0.0067503
 
 Not queried
 
  0.9178802
 
       NA
 
           NA
  
 
  
 17
 
  77922738
 
  77922972
 
  ENSEMBL   UCSC 
 
  235
 
  4
 
 16845
 
 gene;island
 
 0.391420
 
 0.6374820
 
 Not queried
 
  0.9177149
 
       NA
 
           NA
  
 
  
 10
 
  95462161
 
  95462325
 
  ENSEMBL   UCSC 
 
  165
 
  5
 
 4683
 
 gene
 
 0.005578
 
 0.0048370
 
 Not queried
 
  0.9176162
 
       NA
 
           NA
  
 
  
 7
 
 128494060
 
 128494245
 
  ENSEMBL   UCSC 
 
  186
 
  4
 
 37217
 
 gene;island
 
 0.578374
 
 0.6586348
 
 Not queried
 
  0.9175980
 
       NA
 
           NA
  
 
  
 16
 
  58718767
 
  58718971
 
  ENSEMBL   UCSC 
 
  205
 
  4
 
 15276
 
 tss
 
 0.513418
 
 0.2793739
 
 Not queried
 
  0.9174503
 
       NA
 
           NA
  
 
  
 6
 
  56954677
 
  56954784
 
  ENSEMBL   UCSC 
 
  108
 
  4
 
 35048
 
 tss
 
 0.001713
 
 0.0060999
 
 Not queried
 
  0.9174387
 
       NA
 
           NA
  
 
  
 18
 
  48086162
 
  48086680
 
  ENSEMBL   UCSC 
 
  519
 
  7
 
 19514
 
 island
 
 0.019200
 
 0.0323475
 
 Not queried
 
  0.9174124
 
       NA
 
           NA
  
 
  
 5
 
  50674642
 
  50675192
 
  ENSEMBL   UCSC 
 
  551
 
  6
 
 32787
 
 island
 
 0.040800
 
 0.0186093
 
 Not queried
 
  0.9172122
 
       NA
 
           NA
  
 
  
 7
 
 130352754
 
 130353136
 
  ENSEMBL   UCSC 
 
  383
 
  5
 
 37226
 
 gene;island
 
 0.011526
 
 0.0090633
 
 Not queried
 
  0.9171605
 
       NA
 
           NA
  
 
  
 7
 
 131012106
 
 131012552
 
  ENSEMBL   UCSC 
 
  447
 
 11
 
 37906
 
 tss
 
 0.006209
 
 0.0030389
 
 Not queried
 
  0.9170431
 
       NA
 
           NA
  
 
  
 10
 
  95326033
 
  95326409
 
  ENSEMBL   UCSC 
 
  377
 
  9
 
 5238
 
 tss;island
 
 0.036337
 
 0.1059780
 
 Not queried
 
  0.9169452
 
       NA
 
           NA
  
 
  
 1
 
  10707917
 
  10708091
 
  ENSEMBL   UCSC 
 
  175
 
  5
 
 212
 
 gene;island
 
 0.856343
 
 0.7230253
 
 Not queried
 
  0.9166100
 
       NA
 
           NA
  
 
  
 14
 
 101350645
 
 101351026
 
  ENSEMBL   UCSC 
 
  382
 
  7
 
 12214
 
 gene
 
 0.540525
 
 0.7063927
 
 Not queried
 
  0.9165887
 
       NA
 
           NA
  
 
  
 2
 
 233470847
 
 233471248
 
  ENSEMBL   UCSC 
 
  402
 
  5
 
 23067
 
 gene
 
 0.014286
 
 0.0129891
 
 Not queried
 
  0.9164848
 
       NA
 
           NA
  
 
  
 15
 
  37180325
 
  37181087
 
  ENSEMBL   UCSC 
 
  763
 
  6
 
 14010
 
 island
 
 0.468485
 
 0.1749250
 
 Not queried
 
  0.9163758
 
       NA
 
           NA
  
 
  
 10
 
 102976327
 
 102977342
 
  ENSEMBL   UCSC 
 
 1016
 
 11
 
 6056
 
 island
 
 0.058203
 
 0.1333103
 
 Not queried
 
  0.9161890
 
       NA
 
           NA
  
 
  
 2
 
 209271164
 
 209271505
 
  ENSEMBL   UCSC 
 
  342
 
  5
 
 23850
 
 tss
 
 0.041911
 
 0.1444433
 
 Not queried
 
  0.9161576
 
       NA
 
           NA
  
 
  
 1
 
  40839445
 
  40839764
 
  ENSEMBL   UCSC 
 
  320
 
  4
 
 3121
 
 island
 
 0.018265
 
 0.0153994
 
 Not queried
 
  0.9160074
 
       NA
 
           NA
  
 
  
 1
 
 153747551
 
 153747728
 
  ENSEMBL   UCSC 
 
  178
 
  6
 
 1953
 
 tss
 
 0.510193
 
 0.5846900
 
 Not queried
 
  0.9159742
 
       NA
 
           NA
  
 
  
 1
 
  32645027
 
  32645337
 
  ENSEMBL   UCSC 
 
  311
 
  8
 
 1453
 
 tss
 
 0.008233
 
 0.0088261
 
 Not queried
 
  0.9157756
 
       NA
 
           NA
  
 
  
 8
 
  28748202
 
  28748679
 
  ENSEMBL   UCSC 
 
  478
 
  5
 
 39266
 
 gene;tss;island
 
 0.011793
 
 0.0122185
 
 Not queried
 
  0.9157271
 
       NA
 
           NA
  
 
  
 5
 
   3607033
 
   3607134
 
  ENSEMBL   UCSC 
 
  102
 
  4
 
 32669
 
 island
 
 0.257149
 
 0.3665464
 
 Not queried
 
  0.9155689
 
       NA
 
           NA
  
 
  
 1
 
   1177703
 
   1178245
 
  ENSEMBL   UCSC 
 
  543
 
  6
 
 2417
 
 island
 
 0.268461
 
 0.5354221
 
 Not queried
 
  0.9155321
 
       NA
 
           NA
  
 
  
 16
 
  90148388
 
  90148962
 
  ENSEMBL   UCSC 
 
  575
 
  5
 
 16283
 
 island
 
 0.016179
 
 0.0121073
 
 Not queried
 
  0.9154188
 
       NA
 
           NA
  
 
  
 6
 
 105628000
 
 105628165
 
  ENSEMBL   UCSC 
 
  166
 
  9
 
 35146
 
 tss;island
 
 0.015923
 
 0.0091517
 
 Not queried
 
  0.9153258
 
       NA
 
           NA
  
 
  
 6
 
 130031504
 
 130031784
 
  ENSEMBL   UCSC 
 
  281
 
  6
 
 35219
 
 tss
 
 0.014458
 
 0.0049175
 
 Not queried
 
  0.9152714
 
       NA
 
           NA
  
 
  
 20
 
    524243
 
    524791
 
  ENSEMBL   UCSC 
 
  549
 
 10
 
 25924
 
 island
 
 0.003362
 
 0.0015772
 
 Not queried
 
  0.9152346
 
       NA
 
           NA
  
 
  
 1
 
 228466481
 
 228466971
 
  ENSEMBL   UCSC 
 
  491
 
  5
 
 1053
 
 gene;island
 
 0.575060
 
 0.7602548
 
 Not queried
 
  0.9152229
 
       NA
 
           NA
  
 
  
 19
 
  42437245
 
  42437425
 
  ENSEMBL   UCSC 
 
  181
 
  4
 
 22060
 
 island
 
 0.954265
 
 0.9739966
 
 Not queried
 
  0.9152062
 
       NA
 
           NA
  
 
  
 4
 
   4859772
 
   4860190
 
  ENSEMBL   UCSC 
 
  419
 
 11
 
 30866
 
 island
 
 0.376444
 
 0.5016869
 
 Not queried
 
  0.9149733
 
       NA
 
           NA
  
 
  
 6
 
 169261176
 
 169261475
 
  ENSEMBL   UCSC 
 
  300
 
  5
 
 36669
 
 island
 
 0.679407
 
 0.7609590
 
 Not queried
 
  0.9148432
 
       NA
 
           NA
  
 
  
 6
 
  69344406
 
  69344702
 
  ENSEMBL   UCSC 
 
  297
 
  5
 
 35055
 
 tss;island
 
 0.016951
 
 0.0385747
 
 Not queried
 
  0.9143497
 
       NA
 
           NA
  
 
  
 19
 
  10381577
 
  10382048
 
  ENSEMBL   UCSC 
 
  472
 
  4
 
 19811
 
 gene
 
 0.003315
 
 0.0026314
 
 Not queried
 
  0.9143387
 
       NA
 
           NA
  
 
  
 7
 
   5085252
 
   5085485
 
  ENSEMBL   UCSC 
 
  234
 
  7
 
 37419
 
 tss
 
 0.010812
 
 0.0044912
 
 Not queried
 
  0.9143071
 
       NA
 
           NA
  
 
  
 7
 
  27187372
 
  27187691
 
  ENSEMBL   UCSC 
 
  320
 
  8
 
 37493
 
 tss
 
 0.011146
 
 0.0471205
 
 Not queried
 
  0.9141340
 
       NA
 
           NA
  
 
  
 5
 
  79551870
 
  79552177
 
  ENSEMBL   UCSC 
 
  308
 
  6
 
 32149
 
 tss
 
 0.005668
 
 0.0042770
 
 Not queried
 
  0.9140454
 
       NA
 
           NA
  
 
  
 6
 
  26172091
 
  26172397
 
  ENSEMBL   UCSC 
 
  307
 
  4
 
 35520
 
 island
 
 0.005530
 
 0.0024297
 
 Not queried
 
  0.9139500
 
       NA
 
           NA
  
 
  
 10
 
    575899
 
    576100
 
  ENSEMBL   UCSC 
 
  202
 
  5
 
 4443
 
 gene;island
 
 0.810681
 
 0.9129934
 
 Not queried
 
  0.9137226
 
       NA
 
           NA
  
 
  
 1
 
 116311561
 
 116311749
 
  ENSEMBL   UCSC 
 
  189
 
  4
 
 1860
 
 tss
 
 0.571472
 
 0.6444288
 
 Not queried
 
  0.9136787
 
       NA
 
           NA
  
 
  
 17
 
  48183258
 
  48183477
 
  ENSEMBL   UCSC 
 
  220
 
  5
 
 16680
 
 gene;island
 
 0.516414
 
 0.8831125
 
 Not queried
 
  0.9134565
 
       NA
 
           NA
  
 
  
 15
 
  76005189
 
  76005446
 
  ENSEMBL   UCSC 
 
  258
 
  4
 
 13817
 
 tss
 
 0.031277
 
 0.0680124
 
 Not queried
 
  0.9132336
 
       NA
 
           NA
  
 
  
 14
 
  73925161
 
  73925225
 
  ENSEMBL   UCSC 
 
   65
 
  4
 
 12130
 
 gene
 
 0.005561
 
 0.0029343
 
 Not queried
 
  0.9131697
 
       NA
 
           NA
  
 
  
 14
 
  73925161
 
  73925622
 
  ENSEMBL   UCSC 
 
  462
 
  8
 
 12987
 
 island
 
 0.005911
 
 0.0029343
 
 Not queried
 
  0.9131697
 
       NA
 
           NA
  
 
  
 3
 
  55522611
 
  55522835
 
  ENSEMBL   UCSC 
 
  225
 
  5
 
 29216
 
 island
 
 0.005149
 
 0.0037370
 
 Not queried
 
  0.9131582
 
       NA
 
           NA
  
 
  
 2
 
  23913322
 
  23913568
 
  ENSEMBL   UCSC 
 
  247
 
  4
 
 22596
 
 gene;island
 
 0.948619
 
 0.8098020
 
 Not queried
 
  0.9131077
 
       NA
 
           NA
  
 
  
 10
 
 121484846
 
 121485184
 
  ENSEMBL   UCSC 
 
  339
 
  6
 
 5402
 
 tss;island
 
 0.005684
 
 0.0160183
 
 Not queried
 
  0.9127070
 
       NA
 
           NA
  
 
  
 1
 
 115631930
 
 115632727
 
  ENSEMBL   UCSC 
 
  798
 
 10
 
 3628
 
 island
 
 0.016451
 
 0.0164477
 
 Not queried
 
  0.9124301
 
       NA
 
           NA
  
 
  
 1
 
 150241102
 
 150241941
 
  ENSEMBL   UCSC 
 
  840
 
 12
 
 3718
 
 island
 
 0.004654
 
 0.0051036
 
 Not queried
 
  0.9123424
 
       NA
 
           NA
  
 
  
 10
 
  38299242
 
  38299574
 
  ENSEMBL   UCSC 
 
  333
 
  9
 
 5035
 
 tss
 
 0.007906
 
 0.0038527
 
 Not queried
 
  0.9119732
 
       NA
 
           NA
  
 
  
 10
 
  31609200
 
  31609531
 
  ENSEMBL   UCSC 
 
  332
 
  4
 
 4553
 
 gene;tss;island
 
 0.006110
 
 0.0079256
 
 Not queried
 
  0.9119022
 
       NA
 
           NA
  
 
  
 10
 
  38299242
 
  38299653
 
  ENSEMBL   UCSC 
 
  412
 
 10
 
 5672
 
 island
 
 0.008085
 
 0.0049414
 
 Not queried
 
  0.9117281
 
       NA
 
           NA
  
 
  
 15
 
  65903322
 
  65903461
 
  ENSEMBL   UCSC 
 
  140
 
  4
 
 13383
 
 gene;island
 
 0.019408
 
 0.0101830
 
 Not queried
 
  0.9116723
 
       NA
 
           NA
  
 
  
 19
 
  12075882
 
  12076348
 
  ENSEMBL   UCSC 
 
  467
 
  6
 
 19851
 
 gene
 
 0.023077
 
 0.0137070
 
 Not queried
 
  0.9115469
 
       NA
 
           NA
  
 
  
 5
 
    191127
 
    191600
 
  ENSEMBL   UCSC 
 
  474
 
  8
 
 31954
 
 tss
 
 0.102524
 
 0.0578805
 
 Not queried
 
  0.9107523
 
       NA
 
           NA
  
 
  
 5
 
  80529067
 
  80529340
 
  ENSEMBL   UCSC 
 
  274
 
  6
 
 31666
 
 gene
 
 0.077310
 
 0.1369996
 
 Not queried
 
  0.9107079
 
       NA
 
           NA
  
 
  
 11
 
  94276895
 
  94276960
 
  ENSEMBL   UCSC 
 
   66
 
  4
 
 7631
 
 tss
 
 0.031263
 
 0.0125828
 
 Not queried
 
  0.9102660
 
       NA
 
           NA
  
 
  
 19
 
  46974886
 
  46975180
 
  ENSEMBL   UCSC 
 
  295
 
  7
 
 20892
 
 tss;island
 
 0.005477
 
 0.0163072
 
 Not queried
 
  0.9101839
 
       NA
 
           NA
  
 
  
 19
 
  52872916
 
  52873125
 
  ENSEMBL   UCSC 
 
  210
 
  6
 
 21012
 
 tss
 
 0.060569
 
 0.0335084
 
 Not queried
 
  0.9100609
 
       NA
 
           NA
  
 
  
 19
 
  52872916
 
  52873624
 
  ENSEMBL   UCSC 
 
  709
 
 10
 
 22356
 
 island
 
 0.060569
 
 0.0358982
 
 Not queried
 
  0.9100609
 
       NA
 
           NA
  
 
  
 7
 
  53254947
 
  53255225
 
  ENSEMBL   UCSC 
 
  279
 
  5
 
 38497
 
 island
 
 0.446280
 
 0.6021499
 
 Not queried
 
  0.9096649
 
       NA
 
           NA
  
 
  
 12
 
  40499811
 
  40500139
 
  ENSEMBL   UCSC 
 
  329
 
  4
 
 9639
 
 tss
 
 0.079302
 
 0.0282253
 
 Not queried
 
  0.9094587
 
       NA
 
           NA
  
 
  
 1
 
 153919171
 
 153919388
 
  ENSEMBL   UCSC 
 
  218
 
  6
 
 1955
 
 tss;island
 
 0.008685
 
 0.0040483
 
 Not queried
 
  0.9093113
 
       NA
 
           NA
  
 
  
 4
 
  39979757
 
  39979791
 
  ENSEMBL   UCSC 
 
   35
 
  4
 
 30355
 
 tss;island
 
 0.007198
 
 0.0123249
 
 Not queried
 
  0.9092622
 
       NA
 
           NA
  
 
  
 18
 
  11851233
 
  11851603
 
  ENSEMBL   UCSC 
 
  371
 
 10
 
 19057
 
 gene;island
 
 0.004819
 
 0.0028300
 
 Not queried
 
  0.9088532
 
       NA
 
           NA
  
 
  
 4
 
  95128661
 
  95129012
 
  ENSEMBL   UCSC 
 
  352
 
 11
 
 30502
 
 tss
 
 0.004487
 
 0.0023130
 
 Not queried
 
  0.9088316
 
       NA
 
           NA
  
 
  
 1
 
  26699448
 
  26699617
 
  ENSEMBL   UCSC 
 
  170
 
  4
 
 1390
 
 tss
 
 0.592443
 
 0.7136859
 
 Not queried
 
  0.9086651
 
       NA
 
           NA
  
 
  
 22
 
  17489112
 
  17489367
 
  ENSEMBL   UCSC 
 
  256
 
  7
 
 26938
 
 tss;island
 
 0.881615
 
 0.9161025
 
 Not queried
 
  0.9085259
 
       NA
 
           NA
  
 
  
 15
 
  25841831
 
  25842111
 
  ENSEMBL   UCSC 
 
  281
 
  4
 
 13956
 
 island
 
 0.753033
 
 0.8714615
 
 Not queried
 
  0.9085144
 
       NA
 
           NA
  
 
  
 11
 
  64335575
 
  64335895
 
  ENSEMBL   UCSC 
 
  321
 
  4
 
 6689
 
 gene;island
 
 0.370570
 
 0.5114811
 
 Not queried
 
  0.9085028
 
       NA
 
           NA
  
 
  
 4
 
   1404128
 
   1404607
 
  ENSEMBL   UCSC 
 
  480
 
  4
 
 30785
 
 island
 
 0.285914
 
 0.1353299
 
 Not queried
 
  0.9082561
 
       NA
 
           NA
  
 
  
 6
 
  32120584
 
  32121611
 
  ENSEMBL   UCSC 
 
 1028
 
 35
 
 34866
 
 tss
 
 0.508610
 
 0.7990585
 
 Not queried
 
  0.9081856
 
       NA
 
           NA
  
 
  
 11
 
  85375937
 
  85376233
 
  ENSEMBL   UCSC 
 
  297
 
  5
 
 8706
 
 island
 
 0.010558
 
 0.0058223
 
 Not queried
 
  0.9080529
 
       NA
 
           NA
  
 
  
 6
 
  33147499
 
  33147659
 
  ENSEMBL   UCSC 
 
  161
 
  4
 
 34208
 
 gene
 
 0.488287
 
 0.4617846
 
 Not queried
 
  0.9080449
 
       NA
 
           NA
  
 
  
 8
 
  72274546
 
  72274615
 
  ENSEMBL   UCSC 
 
   70
 
  4
 
 39762
 
 tss
 
 0.098871
 
 0.0715512
 
 Not queried
 
  0.9080306
 
       NA
 
           NA
  
 
  
 1
 
 153514264
 
 153514482
 
  ENSEMBL   UCSC 
 
  219
 
  4
 
 1943
 
 tss
 
 0.625948
 
 0.7809379
 
 Not queried
 
  0.9077942
 
       NA
 
           NA
  
 
  
 1
 
 117602490
 
 117602840
 
  ENSEMBL   UCSC 
 
  351
 
  4
 
 1867
 
 tss;island
 
 0.006469
 
 0.0083210
 
 Not queried
 
  0.9077202
 
       NA
 
           NA
  
 
  
 7
 
   4210949
 
   4211755
 
  ENSEMBL   UCSC 
 
  807
 
  8
 
 36831
 
 gene
 
 0.618362
 
 0.7720407
 
 Not queried
 
  0.9076534
 
       NA
 
           NA
  
 
  
 7
 
 107205042
 
 107205392
 
  ENSEMBL   UCSC 
 
  351
 
  5
 
 37180
 
 gene;tss;island
 
 0.979600
 
 0.9602961
 
 Not queried
 
  0.9075069
 
       NA
 
           NA
  
 
  
 6
 
  55039232
 
  55039382
 
  ENSEMBL   UCSC 
 
  151
 
  4
 
 34374
 
 gene;island
 
 0.037179
 
 0.0275721
 
 Not queried
 
  0.9074872
 
       NA
 
           NA
  
 
  
 6
 
  24494462
 
  24494793
 
  ENSEMBL   UCSC 
 
  332
 
  5
 
 34669
 
 tss;island
 
 0.156890
 
 0.0230212
 
 Not queried
 
  0.9073979
 
       NA
 
           NA
  
 
  
 5
 
 110847639
 
 110848191
 
  ENSEMBL   UCSC 
 
  553
 
  6
 
 33002
 
 island
 
 0.008953
 
 0.0088561
 
 Not queried
 
  0.9073559
 
       NA
 
           NA
  
 
  
 3
 
  53289970
 
  53290343
 
  ENSEMBL   UCSC 
 
  374
 
 10
 
 29201
 
 island
 
 0.014592
 
 0.0281475
 
 Not queried
 
  0.9072034
 
       NA
 
           NA
  
 
  
 22
 
  29949725
 
  29949986
 
  ENSEMBL   UCSC 
 
  262
 
  6
 
 27050
 
 tss
 
 0.003746
 
 0.0074025
 
 Not queried
 
  0.9071267
 
       NA
 
           NA
  
 
  
 5
 
 176057740
 
 176057996
 
  ENSEMBL   UCSC 
 
  257
 
  5
 
 31911
 
 gene;tss;island
 
 0.029924
 
 0.5064149
 
 Not queried
 
  0.9069234
 
       NA
 
           NA
  
 
  
 18
 
  56886915
 
  56887351
 
  ENSEMBL   UCSC 
 
  437
 
  9
 
 19298
 
 tss
 
 0.008662
 
 0.0494190
 
 Not queried
 
  0.9068587
 
       NA
 
           NA
  
 
  
 6
 
 152011103
 
 152011415
 
  ENSEMBL   UCSC 
 
  313
 
  5
 
 35302
 
 tss
 
 0.980345
 
 0.7014363
 
 Not queried
 
  0.9068327
 
       NA
 
           NA
  
 
  
 6
 
  29855890
 
  29856938
 
  ENSEMBL   UCSC 
 
 1049
 
 29
 
 33700
 
 gene;island
 
 0.030913
 
 0.0426651
 
 Not queried
 
  0.9068105
 
       NA
 
           NA
  
 
  
 14
 
 101531403
 
 101532352
 
  ENSEMBL   UCSC 
 
  950
 
 20
 
 12634
 
 tss;island
 
 0.492840
 
 0.6064194
 
 Not queried
 
  0.9067415
 
       NA
 
           NA
  
 
  
 8
 
  38239693
 
  38239917
 
  ENSEMBL   UCSC 
 
  225
 
  4
 
 40232
 
 island
 
 0.008436
 
 0.0040982
 
 Not queried
 
  0.9066325
 
       NA
 
           NA
  
 
  
 11
 
    304037
 
    304351
 
  ENSEMBL   UCSC 
 
  315
 
  4
 
 7839
 
 island
 
 0.909853
 
 0.9628372
 
 Not queried
 
  0.9064333
 
       NA
 
           NA
  
 
  
 20
 
  50701266
 
  50701701
 
  ENSEMBL   UCSC 
 
  436
 
  5
 
 25455
 
 gene;island
 
 0.963667
 
 0.9796655
 
 Not queried
 
  0.9064178
 
       NA
 
           NA
  
 
  
 16
 
  51184786
 
  51185147
 
  ENSEMBL   UCSC 
 
  362
 
  9
 
 14749
 
 gene
 
 0.051501
 
 0.1251034
 
 Not queried
 
  0.9063749
 
       NA
 
           NA
  
 
  
 8
 
  95565739
 
  95565953
 
  ENSEMBL   UCSC 
 
  215
 
  4
 
 39816
 
 tss
 
 0.006664
 
 0.0032005
 
 Not queried
 
  0.9063409
 
       NA
 
           NA
  
 
  
 1
 
  65775467
 
  65775772
 
  ENSEMBL   UCSC 
 
  306
 
  5
 
 570
 
 gene;island
 
 0.039416
 
 0.1528138
 
 Not queried
 
  0.9063332
 
       NA
 
           NA
  
 
  
 20
 
  43936981
 
  43937467
 
  ENSEMBL   UCSC 
 
  487
 
  6
 
 25771
 
 tss
 
 0.578012
 
 0.5257156
 
 Not queried
 
  0.9060911
 
       NA
 
           NA
  
 
  
 5
 
 122434178
 
 122434544
 
  ENSEMBL   UCSC 
 
  367
 
  5
 
 31731
 
 gene;island
 
 0.440052
 
 0.3391786
 
 Not queried
 
  0.9060371
 
       NA
 
           NA
  
 
  
 5
 
 121648313
 
 121648479
 
  ENSEMBL   UCSC 
 
  167
 
  4
 
 31727
 
 gene;island
 
 0.064597
 
 0.0505134
 
 Not queried
 
  0.9059249
 
       NA
 
           NA
  
 
  
 7
 
  73153139
 
  73153318
 
  ENSEMBL   UCSC 
 
  180
 
  4
 
 38567
 
 island
 
 0.015564
 
 0.0130048
 
 Not queried
 
  0.9055355
 
       NA
 
           NA
  
 
  
 19
 
  55866246
 
  55866412
 
  ENSEMBL   UCSC 
 
  167
 
  7
 
 21076
 
 tss
 
 0.057668
 
 0.0171873
 
 Not queried
 
  0.9054126
 
       NA
 
           NA
  
 
  
 8
 
   1764878
 
   1765387
 
  ENSEMBL   UCSC 
 
  510
 
 10
 
 39535
 
 tss
 
 0.112353
 
 0.0453190
 
 Not queried
 
  0.9053785
 
       NA
 
           NA
  
 
  
 8
 
   1764878
 
   1765477
 
  ENSEMBL   UCSC 
 
  600
 
 12
 
 40038
 
 island
 
 0.075073
 
 0.0453190
 
 Not queried
 
  0.9053785
 
       NA
 
           NA
  
 
  
 13
 
  52769662
 
  52769951
 
  ENSEMBL   UCSC 
 
  290
 
  4
 
 11460
 
 tss;island
 
 0.042292
 
 0.0193829
 
 Not queried
 
  0.9052018
 
       NA
 
           NA
  
 
  
 19
 
  16222203
 
  16222446
 
  ENSEMBL   UCSC 
 
  244
 
  4
 
 20567
 
 tss
 
 0.007628
 
 0.0061707
 
 Not queried
 
  0.9048936
 
       NA
 
           NA
  
 
  
 3
 
 107601860
 
 107602025
 
  ENSEMBL   UCSC 
 
  166
 
  4
 
 28486
 
 tss
 
 0.823079
 
 0.8816572
 
 Not queried
 
  0.9048634
 
       NA
 
           NA
  
 
  
 3
 
  49203799
 
  49203975
 
  ENSEMBL   UCSC 
 
  177
 
  5
 
 28322
 
 tss
 
 0.595551
 
 0.1388057
 
 Not queried
 
  0.9047967
 
       NA
 
           NA
  
 
  
 3
 
  49203769
 
  49203975
 
  ENSEMBL   UCSC 
 
  207
 
  7
 
 29110
 
 island
 
 0.513148
 
 0.0492885
 
 Not queried
 
  0.9047967
 
       NA
 
           NA
  
 
  
 7
 
   5821099
 
   5821596
 
  ENSEMBL   UCSC 
 
  498
 
 10
 
 38235
 
 island
 
 0.003617
 
 0.0058881
 
 Not queried
 
  0.9046896
 
       NA
 
           NA
  
 
  
 5
 
  66124395
 
  66124801
 
  ENSEMBL   UCSC 
 
  407
 
  7
 
 31636
 
 gene
 
 0.009060
 
 0.0124195
 
 Not queried
 
  0.9046432
 
       NA
 
           NA
  
 
  
 13
 
 111719677
 
 111720031
 
  ENSEMBL   UCSC 
 
  355
 
  5
 
 11895
 
 island
 
 0.046775
 
 0.0256141
 
 Not queried
 
  0.9046423
 
       NA
 
           NA
  
 
  
 5
 
 140723331
 
 140723899
 
  ENSEMBL   UCSC 
 
  569
 
  9
 
 31818
 
 gene;island
 
 0.560224
 
 0.0486858
 
 Not queried
 
  0.9045429
 
       NA
 
           NA
  
 
  
 13
 
  93879303
 
  93879769
 
  ENSEMBL   UCSC 
 
  467
 
  5
 
 11190
 
 gene;island
 
 0.198830
 
 0.0788234
 
 Not queried
 
  0.9045200
 
       NA
 
           NA
  
 
  
 17
 
   5026540
 
   5026840
 
  ENSEMBL   UCSC 
 
  301
 
  7
 
 17041
 
 tss
 
 0.338931
 
 0.5096575
 
 Not queried
 
  0.9043651
 
       NA
 
           NA
  
 
  
 15
 
  93199018
 
  93199180
 
  ENSEMBL   UCSC 
 
  163
 
  6
 
 14473
 
 island
 
 0.018537
 
 0.0242788
 
 Not queried
 
  0.9043015
 
       NA
 
           NA
  
 
  
 19
 
  54584636
 
  54585167
 
  ENSEMBL   UCSC 
 
  532
 
  4
 
 21048
 
 tss
 
 0.511843
 
 0.6425700
 
 Not queried
 
  0.9041830
 
       NA
 
           NA
  
 
  
 2
 
 131513608
 
 131513927
 
  ENSEMBL   UCSC 
 
  320
 
  6
 
 22867
 
 gene;island
 
 0.075809
 
 0.0456568
 
 Not queried
 
  0.9040938
 
       NA
 
           NA
  
 
  
 4
 
  83932010
 
  83932227
 
  ENSEMBL   UCSC 
 
  218
 
  7
 
 30471
 
 tss
 
 0.004924
 
 0.0041934
 
 Not queried
 
  0.9040763
 
       NA
 
           NA
  
 
  
 2
 
 128051348
 
 128051708
 
  ENSEMBL   UCSC 
 
  361
 
  4
 
 22856
 
 gene
 
 0.063706
 
 0.0646989
 
 Not queried
 
  0.9040006
 
       NA
 
           NA
  
 
  
 1
 
   9324688
 
   9325015
 
  ENSEMBL   UCSC 
 
  328
 
  5
 
 201
 
 gene;island
 
 0.907135
 
 0.8854739
 
 Not queried
 
  0.9038925
 
       NA
 
           NA
  
 
  
 1
 
 161049443
 
 161049677
 
  ENSEMBL   UCSC 
 
  235
 
  5
 
 847
 
 gene;island
 
 0.890066
 
 0.9640605
 
 Not queried
 
  0.9038163
 
       NA
 
           NA
  
 
  
 15
 
  23810163
 
  23810378
 
  ENSEMBL   UCSC 
 
  216
 
  5
 
 13515
 
 tss
 
 0.378624
 
 0.5324445
 
 Not queried
 
  0.9036813
 
       NA
 
           NA
  
 
  
 17
 
   6616644
 
   6617192
 
  ENSEMBL   UCSC 
 
  549
 
  8
 
 17909
 
 island
 
 0.025957
 
 0.0262049
 
 Not queried
 
  0.9035782
 
       NA
 
           NA
  
 
  
 19
 
   1438017
 
   1438795
 
  ENSEMBL   UCSC 
 
  779
 
 14
 
 21223
 
 island
 
 0.017648
 
 0.0434194
 
 Not queried
 
  0.9035343
 
       NA
 
           NA
  
 
  
 8
 
  38964784
 
  38965026
 
  ENSEMBL   UCSC 
 
  243
 
  7
 
 39662
 
 tss
 
 0.116187
 
 0.2997064
 
 Not queried
 
  0.9028949
 
       NA
 
           NA
  
 
  
 15
 
  83680418
 
  83680570
 
  ENSEMBL   UCSC 
 
  153
 
  6
 
 13863
 
 tss
 
 0.005021
 
 0.0024517
 
 Not queried
 
  0.9027868
 
       NA
 
           NA
  
 
  
 12
 
  95467350
 
  95467612
 
  ENSEMBL   UCSC 
 
  263
 
  6
 
 10684
 
 island
 
 0.003181
 
 0.0028690
 
 Not queried
 
  0.9027069
 
       NA
 
           NA
  
 
  
 19
 
  49458215
 
  49458596
 
  ENSEMBL   UCSC 
 
  382
 
  5
 
 20140
 
 gene;island
 
 0.009552
 
 0.0056616
 
 Not queried
 
  0.9026360
 
       NA
 
           NA
  
 
  
 7
 
 143042585
 
 143042798
 
  ENSEMBL   UCSC 
 
  214
 
  4
 
 37253
 
 gene;island
 
 0.152055
 
 0.0572358
 
 Not queried
 
  0.9025940
 
       NA
 
           NA
  
 
  
 18
 
  43913565
 
  43913755
 
  ENSEMBL   UCSC 
 
  191
 
  5
 
 19256
 
 tss;island
 
 0.027462
 
 0.0285321
 
 Not queried
 
  0.9025679
 
       NA
 
           NA
  
 
  
 19
 
  37019165
 
  37019614
 
  ENSEMBL   UCSC 
 
  450
 
  9
 
 21941
 
 island
 
 0.005920
 
 0.0056532
 
 Not queried
 
  0.9024662
 
       NA
 
           NA
  
 
  
 21
 
  47401109
 
  47401492
 
  ENSEMBL   UCSC 
 
  384
 
  5
 
 26598
 
 tss;island
 
 0.066028
 
 0.0519468
 
 Not queried
 
  0.9019330
 
       NA
 
           NA
  
 
  
 7
 
 151129122
 
 151129313
 
  ENSEMBL   UCSC 
 
  192
 
  5
 
 37293
 
 gene;island
 
 0.845265
 
 0.9277601
 
 Not queried
 
  0.9018695
 
       NA
 
           NA
  
 
  
 2
 
 223161771
 
 223162128
 
  ENSEMBL   UCSC 
 
  358
 
  4
 
 23046
 
 gene;tss;island
 
 0.067890
 
 0.1885033
 
 Not queried
 
  0.9018281
 
       NA
 
           NA
  
 
  
 2
 
  65454430
 
  65454860
 
  ENSEMBL   UCSC 
 
  431
 
 10
 
 24352
 
 island
 
 0.006786
 
 0.0052119
 
 Not queried
 
  0.9017553
 
       NA
 
           NA
  
 
  
 22
 
  50919817
 
  50919961
 
  ENSEMBL   UCSC 
 
  145
 
  5
 
 27255
 
 tss
 
 0.016808
 
 0.0109550
 
 Not queried
 
  0.9017118
 
       NA
 
           NA
  
 
  
 4
 
  89444989
 
  89445274
 
  ENSEMBL   UCSC 
 
  286
 
  8
 
 30493
 
 tss
 
 0.038290
 
 0.0103279
 
 Not queried
 
  0.9016442
 
       NA
 
           NA
  
 
  
 11
 
 129234117
 
 129234377
 
  ENSEMBL   UCSC 
 
  261
 
  6
 
 8946
 
 island
 
 0.006462
 
 0.0029147
 
 Not queried
 
  0.9014943
 
       NA
 
           NA
  
 
  
 6
 
 117002099
 
 117002400
 
  ENSEMBL   UCSC 
 
  302
 
  9
 
 36432
 
 island
 
 0.005851
 
 0.0039330
 
 Not queried
 
  0.9014143
 
       NA
 
           NA
  
 
  
 5
 
 140807225
 
 140807787
 
  ENSEMBL   UCSC 
 
  563
 
  5
 
 31835
 
 gene;island
 
 0.830561
 
 0.9571297
 
 Not queried
 
  0.9014140
 
       NA
 
           NA
  
 
  
 3
 
 137483328
 
 137483696
 
  ENSEMBL   UCSC 
 
  369
 
  8
 
 29512
 
 island
 
 0.171856
 
 0.1880322
 
 Not queried
 
  0.9013982
 
       NA
 
           NA
  
 
  
 1
 
 197880384
 
 197880923
 
  ENSEMBL   UCSC 
 
  540
 
  6
 
 2156
 
 tss;island
 
 0.030001
 
 0.0151130
 
 Not queried
 
  0.9013086
 
       NA
 
           NA
  
 
  
 12
 
  16506202
 
  16506341
 
  ENSEMBL   UCSC 
 
  140
 
  4
 
 9070
 
 gene;tss
 
 0.658796
 
 0.8396585
 
 Not queried
 
  0.9012894
 
       NA
 
           NA
  
 
  
 12
 
  49782966
 
  49783222
 
  ENSEMBL   UCSC 
 
  257
 
  5
 
 9119
 
 gene;island
 
 0.040873
 
 0.1405362
 
 Not queried
 
  0.9012748
 
       NA
 
           NA
  
 
  
 6
 
   4018820
 
   4019159
 
  ENSEMBL   UCSC 
 
  340
 
  5
 
 35390
 
 island
 
 0.002080
 
 0.0039175
 
 Not queried
 
  0.9012386
 
       NA
 
           NA
  
 
  
 19
 
  13397419
 
  13397750
 
  ENSEMBL   UCSC 
 
  332
 
  4
 
 19881
 
 gene;island
 
 0.717527
 
 0.9084817
 
 Not queried
 
  0.9011475
 
       NA
 
           NA
  
 
  
 2
 
 240868184
 
 240868631
 
  ENSEMBL   UCSC 
 
  448
 
  5
 
 25264
 
 island
 
 0.874702
 
 0.9447519
 
 Not queried
 
  0.9010598
 
       NA
 
           NA
  
 
  
 10
 
 102731747
 
 102732224
 
  ENSEMBL   UCSC 
 
  478
 
  7
 
 5299
 
 tss
 
 0.895405
 
 0.9412456
 
 Not queried
 
  0.9009736
 
       NA
 
           NA
  
 
  
 11
 
  62369394
 
  62369870
 
  ENSEMBL   UCSC 
 
  477
 
  7
 
 7348
 
 tss;island
 
 0.027477
 
 0.0125906
 
 Not queried
 
  0.9006566
 
       NA
 
           NA
  
 
  
 13
 
  28366427
 
  28366759
 
  ENSEMBL   UCSC 
 
  333
 
  8
 
 11364
 
 tss
 
 0.043046
 
 0.0593458
 
 Not queried
 
  0.9005197
 
       NA
 
           NA
  
 
  
 1
 
  28832468
 
  28832638
 
  ENSEMBL   UCSC 
 
  171
 
  4
 
 365
 
 gene
 
 0.013547
 
 0.0073504
 
 Not queried
 
  0.9004215
 
       NA
 
           NA
  
 
  
 3
 
 186490656
 
 186490915
 
  ENSEMBL   UCSC 
 
  260
 
  5
 
 29752
 
 island
 
 0.044494
 
 0.0231423
 
 Not queried
 
  0.9001937
 
       NA
 
           NA
  
 
  
 17
 
  59480674
 
  59481280
 
  ENSEMBL   UCSC 
 
  607
 
  5
 
 16719
 
 gene;island
 
 0.008477
 
 0.0135071
 
 Not queried
 
  0.9000314
 
       NA
 
           NA
  
 
  
 11
 
  65366953
 
  65367309
 
  ENSEMBL   UCSC 
 
  357
 
  4
 
 6712
 
 gene;island
 
 0.953181
 
 0.9723026
 
 Not queried
 
  0.8999378
 
       NA
 
           NA
  
 
  
 17
 
  45569959
 
  45570168
 
  ENSEMBL   UCSC 
 
  210
 
  4
 
 18455
 
 island
 
 0.011888
 
 0.0029323
 
 Not queried
 
  0.8996903
 
       NA
 
           NA
  
 
  
 1
 
 107683532
 
 107683775
 
  ENSEMBL   UCSC 
 
  244
 
  5
 
 658
 
 gene;island
 
 0.004971
 
 0.0059074
 
 Not queried
 
  0.8996661
 
       NA
 
           NA
  
 
  
 20
 
  13201193
 
  13201844
 
  ENSEMBL   UCSC 
 
  652
 
 17
 
 25602
 
 tss;island
 
 0.009918
 
 0.0180631
 
 Not queried
 
  0.8995395
 
       NA
 
           NA
  
 
  
 1
 
   6265802
 
   6266099
 
  ENSEMBL   UCSC 
 
  298
 
  6
 
 1201
 
 tss;island
 
 0.010960
 
 0.0238873
 
 Not queried
 
  0.8993816
 
       NA
 
           NA
  
 
  
 11
 
  57364841
 
  57365088
 
  ENSEMBL   UCSC 
 
  248
 
  6
 
 7304
 
 tss
 
 0.803912
 
 0.9039228
 
 Not queried
 
  0.8992815
 
       NA
 
           NA
  
 
  
 6
 
 108436768
 
 108437116
 
  ENSEMBL   UCSC 
 
  349
 
  5
 
 36391
 
 island
 
 0.006063
 
 0.0138464
 
 Not queried
 
  0.8991322
 
       NA
 
           NA
  
 
  
 10
 
 121356513
 
 121356865
 
  ENSEMBL   UCSC 
 
  353
 
  9
 
 6194
 
 island
 
 0.016521
 
 0.0112801
 
 Not queried
 
  0.8990303
 
       NA
 
           NA
  
 
  
 1
 
  94313343
 
  94313745
 
  ENSEMBL   UCSC 
 
  403
 
  6
 
 3496
 
 island
 
 0.006493
 
 0.0041314
 
 Not queried
 
  0.8990256
 
       NA
 
           NA
  
 
  
 16
 
  66599843
 
  66600105
 
  ENSEMBL   UCSC 
 
  263
 
  4
 
 14780
 
 gene
 
 0.980012
 
 0.9655857
 
 Not queried
 
  0.8988327
 
       NA
 
           NA
  
 
  
 12
 
 132982231
 
 132982483
 
  ENSEMBL   UCSC 
 
  253
 
  4
 
 11050
 
 island
 
 0.254315
 
 0.2275790
 
 Not queried
 
  0.8986437
 
       NA
 
           NA
  
 
  
 3
 
 196594273
 
 196594695
 
  ENSEMBL   UCSC 
 
  423
 
  6
 
 28818
 
 tss
 
 0.090763
 
 0.0498033
 
 Not queried
 
  0.8985346
 
       NA
 
           NA
  
 
  
 10
 
  75118622
 
  75118678
 
  ENSEMBL   UCSC 
 
   57
 
  6
 
 5158
 
 tss
 
 0.026482
 
 0.0175212
 
 Not queried
 
  0.8983135
 
       NA
 
           NA
  
 
  
 11
 
 126870233
 
 126870702
 
  ENSEMBL   UCSC 
 
  470
 
  5
 
 6964
 
 gene
 
 0.036456
 
 0.0197246
 
 Not queried
 
  0.8982771
 
       NA
 
           NA
  
 
  
 1
 
   2537547
 
   2537838
 
  ENSEMBL   UCSC 
 
  292
 
  6
 
 92
 
 gene;island
 
 0.584812
 
 0.7113264
 
 Not queried
 
  0.8982340
 
       NA
 
           NA
  
 
  
 22
 
  46646366
 
  46646564
 
  ENSEMBL   UCSC 
 
  199
 
  6
 
 27231
 
 tss;island
 
 0.002535
 
 0.0048565
 
 Not queried
 
  0.8979637
 
       NA
 
           NA
  
 
  
 8
 
 124084365
 
 124084822
 
  ENSEMBL   UCSC 
 
  458
 
  6
 
 39896
 
 tss;island
 
 0.044220
 
 0.0236008
 
 Not queried
 
  0.8977744
 
       NA
 
           NA
  
 
  
 11
 
 111750159
 
 111750412
 
  ENSEMBL   UCSC 
 
  254
 
  4
 
 7692
 
 tss
 
 0.022842
 
 0.0123965
 
 Not queried
 
  0.8977684
 
       NA
 
           NA
  
 
  
 6
 
  31782675
 
  31783545
 
  ENSEMBL   UCSC 
 
  871
 
 29
 
 34839
 
 tss;island
 
 0.007455
 
 0.0125140
 
 Not queried
 
  0.8977111
 
       NA
 
           NA
  
 
  
 13
 
 100637113
 
 100637258
 
  ENSEMBL   UCSC 
 
  146
 
  4
 
 11210
 
 gene;island
 
 0.146111
 
 0.2940947
 
 Not queried
 
  0.8976969
 
       NA
 
           NA
  
 
  
 11
 
  89224684
 
  89224799
 
  ENSEMBL   UCSC 
 
  116
 
  6
 
 7614
 
 tss
 
 0.085617
 
 0.0311605
 
 Not queried
 
  0.8976480
 
       NA
 
           NA
  
 
  
 1
 
  46598380
 
  46598507
 
  ENSEMBL   UCSC 
 
  128
 
  5
 
 1592
 
 tss
 
 0.005374
 
 0.0076263
 
 Not queried
 
  0.8974490
 
       NA
 
           NA
  
 
  
 19
 
  53970585
 
  53971035
 
  ENSEMBL   UCSC 
 
  451
 
  8
 
 22379
 
 island
 
 0.022789
 
 0.0227042
 
 Not queried
 
  0.8973564
 
       NA
 
           NA
  
 
  
 16
 
  70323367
 
  70323915
 
  ENSEMBL   UCSC 
 
  549
 
 10
 
 16067
 
 island
 
 0.001389
 
 0.0014992
 
 Not queried
 
  0.8971534
 
       NA
 
           NA
  
 
  
 22
 
  46067363
 
  46067586
 
  ENSEMBL   UCSC 
 
  224
 
  4
 
 27224
 
 tss;island
 
 0.007584
 
 0.0104826
 
 Not queried
 
  0.8967839
 
       NA
 
           NA
  
 
  
 6
 
  29944403
 
  29945155
 
  ENSEMBL   UCSC 
 
  753
 
 30
 
 33704
 
 gene;island
 
 0.032557
 
 0.0170297
 
 Not queried
 
  0.8966267
 
       NA
 
           NA
  
 
  
 10
 
 121652237
 
 121652380
 
  ENSEMBL   UCSC 
 
  144
 
  4
 
 4791
 
 gene
 
 0.004260
 
 0.0070231
 
 Not queried
 
  0.8966106
 
       NA
 
           NA
  
 
  
 18
 
  47013750
 
  47014260
 
  ENSEMBL   UCSC 
 
  511
 
  6
 
 19269
 
 tss;island
 
 0.017316
 
 0.0624733
 
 Not queried
 
  0.8965686
 
       NA
 
           NA
  
 
  
 20
 
   1974738
 
   1974924
 
  ENSEMBL   UCSC 
 
  187
 
  4
 
 25540
 
 tss
 
 0.018363
 
 0.0214383
 
 Not queried
 
  0.8965659
 
       NA
 
           NA
  
 
  
 17
 
  26574231
 
  26574551
 
  ENSEMBL   UCSC 
 
  321
 
  5
 
 18136
 
 island
 
 0.327785
 
 0.4626984
 
 Not queried
 
  0.8965312
 
       NA
 
           NA
  
 
  
 11
 
  13689411
 
  13689686
 
  ENSEMBL   UCSC 
 
  276
 
  5
 
 7158
 
 tss;island
 
 0.007738
 
 0.0073128
 
 Not queried
 
  0.8962020
 
       NA
 
           NA
  
 
  
 22
 
  51021491
 
  51021532
 
  ENSEMBL   UCSC 
 
   42
 
  4
 
 26934
 
 gene
 
 0.010122
 
 0.0141128
 
 Not queried
 
  0.8961602
 
       NA
 
           NA
  
 
  
 5
 
 141017675
 
 141017903
 
  ENSEMBL   UCSC 
 
  229
 
  4
 
 31840
 
 gene;tss;island
 
 0.006947
 
 0.0284793
 
 Not queried
 
  0.8961174
 
       NA
 
           NA
  
 
  
 6
 
  32017024
 
  32017261
 
  ENSEMBL   UCSC 
 
  238
 
  8
 
 34016
 
 gene
 
 0.589420
 
 0.7693977
 
 Not queried
 
  0.8961134
 
       NA
 
           NA
  
 
  
 2
 
 172947846
 
 172948176
 
  ENSEMBL   UCSC 
 
  331
 
  4
 
 24859
 
 island
 
 0.177659
 
 0.3848749
 
 Not queried
 
  0.8960620
 
       NA
 
           NA
  
 
  
 15
 
  65477998
 
  65478125
 
  ENSEMBL   UCSC 
 
  128
 
  4
 
 13741
 
 tss;island
 
 0.002346
 
 0.0025467
 
 Not queried
 
  0.8959150
 
       NA
 
           NA
  
 
  
 1
 
  55445834
 
  55446387
 
  ENSEMBL   UCSC 
 
  554
 
  7
 
 1650
 
 tss
 
 0.249828
 
 0.4143129
 
 Not queried
 
  0.8956961
 
       NA
 
           NA
  
 
  
 2
 
  86422958
 
  86423274
 
  ENSEMBL   UCSC 
 
  317
 
  8
 
 23492
 
 tss
 
 0.002918
 
 0.0031206
 
 Not queried
 
  0.8956053
 
       NA
 
           NA
  
 
  
 5
 
   2757023
 
   2757244
 
  ENSEMBL   UCSC 
 
  222
 
  4
 
 32660
 
 island
 
 0.015567
 
 0.0414969
 
 Not queried
 
  0.8955021
 
       NA
 
           NA
  
 
  
 1
 
   3123338
 
   3123854
 
  ENSEMBL   UCSC 
 
  517
 
  4
 
 108
 
 gene
 
 0.463193
 
 0.5436921
 
 Not queried
 
  0.8954907
 
       NA
 
           NA
  
 
  
 8
 
  75896975
 
  75897310
 
  ENSEMBL   UCSC 
 
  336
 
  4
 
 39339
 
 gene
 
 0.030919
 
 0.0231228
 
 Not queried
 
  0.8953023
 
       NA
 
           NA
  
 
  
 20
 
  48099248
 
  48099479
 
  ENSEMBL   UCSC 
 
  232
 
  7
 
 25808
 
 tss
 
 0.021887
 
 0.0413700
 
 Not queried
 
  0.8952096
 
       NA
 
           NA
  
 
  
 17
 
  41150487
 
  41150871
 
  ENSEMBL   UCSC 
 
  385
 
  4
 
 16593
 
 gene
 
 0.001592
 
 0.0043468
 
 Not queried
 
  0.8951990
 
       NA
 
           NA
  
 
  
 15
 
  85144305
 
  85144807
 
  ENSEMBL   UCSC 
 
  503
 
  4
 
 13460
 
 gene
 
 0.013688
 
 0.0314588
 
 Not queried
 
  0.8951071
 
       NA
 
           NA
  
 
  
 1
 
 161736000
 
 161736072
 
  ENSEMBL   UCSC 
 
   73
 
  5
 
 2055
 
 tss
 
 0.003701
 
 0.0003614
 
 Not queried
 
  0.8949671
 
       NA
 
           NA
  
 
  
 13
 
 115047880
 
 115048081
 
  ENSEMBL   UCSC 
 
  202
 
  4
 
 11319
 
 gene;island
 
 0.040178
 
 0.0313204
 
 Not queried
 
  0.8947663
 
       NA
 
           NA
  
 
  
 17
 
  28902652
 
  28903005
 
  ENSEMBL   UCSC 
 
  354
 
  4
 
 17242
 
 tss;island
 
 0.491120
 
 0.6328143
 
 Not queried
 
  0.8946870
 
       NA
 
           NA
  
 
  
 7
 
   2556962
 
   2557466
 
  ENSEMBL   UCSC 
 
  505
 
  8
 
 36802
 
 gene;tss;island
 
 0.524931
 
 0.7603485
 
 Not queried
 
  0.8944913
 
       NA
 
           NA
  
 
  
 2
 
 133174035
 
 133174136
 
  ENSEMBL   UCSC 
 
  102
 
  5
 
 23639
 
 tss
 
 0.015003
 
 0.0461545
 
 Not queried
 
  0.8944846
 
       NA
 
           NA
  
 
  
 8
 
   1132712
 
   1133105
 
  ENSEMBL   UCSC 
 
  394
 
  5
 
 40017
 
 island
 
 0.394639
 
 0.6199136
 
 Not queried
 
  0.8944178
 
       NA
 
           NA
  
 
  
 14
 
  77965123
 
  77965391
 
  ENSEMBL   UCSC 
 
  269
 
  4
 
 13037
 
 island
 
 0.003437
 
 0.0074978
 
 Not queried
 
  0.8942450
 
       NA
 
           NA
  
 
  
 12
 
  31882198
 
  31882596
 
  ENSEMBL   UCSC 
 
  399
 
  7
 
 9629
 
 tss
 
 0.026231
 
 0.0150781
 
 Not queried
 
  0.8942261
 
       NA
 
           NA
  
 
  
 3
 
 181428046
 
 181428697
 
  ENSEMBL   UCSC 
 
  652
 
 15
 
 28041
 
 gene;island
 
 0.146162
 
 0.0701565
 
 Not queried
 
  0.8941561
 
       NA
 
           NA
  
 
  
 17
 
  34058433
 
  34058623
 
  ENSEMBL   UCSC 
 
  191
 
  5
 
 17274
 
 tss;island
 
 0.027519
 
 0.0500221
 
 Not queried
 
  0.8940530
 
       NA
 
           NA
  
 
  
 11
 
 119294283
 
 119294441
 
  ENSEMBL   UCSC 
 
  159
 
  5
 
 7757
 
 tss;island
 
 0.445126
 
 0.4697241
 
 Not queried
 
  0.8939090
 
       NA
 
           NA
  
 
  
 19
 
  12476508
 
  12476577
 
  ENSEMBL   UCSC 
 
   70
 
  4
 
 20494
 
 tss
 
 0.003533
 
 0.0016709
 
 Not queried
 
  0.8937369
 
       NA
 
           NA
  
 
  
 5
 
 159846223
 
 159846285
 
  ENSEMBL   UCSC 
 
   63
 
  4
 
 32469
 
 tss
 
 0.004667
 
 0.0040023
 
 Not queried
 
  0.8932936
 
       NA
 
           NA
  
 
  
 14
 
 102027514
 
 102027797
 
  ENSEMBL   UCSC 
 
  284
 
  6
 
 12636
 
 tss;island
 
 0.568145
 
 0.2648492
 
 Not queried
 
  0.8932311
 
       NA
 
           NA
  
 
  
 10
 
 102729046
 
 102729112
 
  ENSEMBL   UCSC 
 
   67
 
  4
 
 6038
 
 island
 
 0.010107
 
 0.0084365
 
 Not queried
 
  0.8931333
 
       NA
 
           NA
  
 
  
 6
 
 130686592
 
 130686865
 
  ENSEMBL   UCSC 
 
  274
 
  5
 
 36477
 
 island
 
 0.007385
 
 0.0135572
 
 Not queried
 
  0.8930959
 
       NA
 
           NA
  
 
  
 17
 
  44896748
 
  44897024
 
  ENSEMBL   UCSC 
 
  277
 
  4
 
 17436
 
 tss;island
 
 0.082495
 
 0.0463567
 
 Not queried
 
  0.8930577
 
       NA
 
           NA
  
 
  
 6
 
  30131001
 
  30132226
 
  ENSEMBL   UCSC 
 
 1226
 
 24
 
 33736
 
 gene
 
 0.411006
 
 0.6491494
 
 Not queried
 
  0.8928956
 
       NA
 
           NA
  
 
  
 1
 
   3713094
 
   3713580
 
  ENSEMBL   UCSC 
 
  487
 
  7
 
 1190
 
 tss
 
 0.024893
 
 0.0129518
 
 Not queried
 
  0.8928609
 
       NA
 
           NA
  
 
  
 5
 
 140018644
 
 140018944
 
  ENSEMBL   UCSC 
 
  301
 
  9
 
 32344
 
 tss
 
 0.003996
 
 0.0091635
 
 Not queried
 
  0.8928494
 
       NA
 
           NA
  
 
  
 7
 
  64023509
 
  64023824
 
  ENSEMBL   UCSC 
 
  316
 
  9
 
 37615
 
 tss
 
 0.004173
 
 0.0036931
 
 Not queried
 
  0.8927216
 
       NA
 
           NA
  
 
  
 6
 
  32782617
 
  32783046
 
  ENSEMBL   UCSC 
 
  430
 
  5
 
 34134
 
 gene
 
 0.890920
 
 0.8897947
 
 Not queried
 
  0.8927049
 
       NA
 
           NA
  
 
  
 4
 
  79102707
 
  79103290
 
  ENSEMBL   UCSC 
 
  584
 
  7
 
 30031
 
 gene;island
 
 0.604018
 
 0.7734100
 
 Not queried
 
  0.8924752
 
       NA
 
           NA
  
 
  
 11
 
  70023032
 
  70023240
 
  ENSEMBL   UCSC 
 
  209
 
  4
 
 6788
 
 gene;island
 
 0.908669
 
 0.9480349
 
 Not queried
 
  0.8921949
 
       NA
 
           NA
  
 
  
 7
 
  82792239
 
  82792411
 
  ENSEMBL   UCSC 
 
  173
 
  7
 
 37678
 
 tss
 
 0.001648
 
 0.0098479
 
 Not queried
 
  0.8920903
 
       NA
 
           NA
  
 
  
 19
 
  53193912
 
  53194124
 
  ENSEMBL   UCSC 
 
  213
 
  4
 
 21017
 
 tss;island
 
 0.060879
 
 0.0442101
 
 Not queried
 
  0.8920528
 
       NA
 
           NA
  
 
  
 5
 
   1084425
 
   1084670
 
  ENSEMBL   UCSC 
 
  246
 
  4
 
 31520
 
 gene;island
 
 0.937323
 
 0.8081872
 
 Not queried
 
  0.8918346
 
       NA
 
           NA
  
 
  
 10
 
  94833042
 
  94833214
 
  ENSEMBL   UCSC 
 
  173
 
  6
 
 5234
 
 tss;island
 
 0.265967
 
 0.0932218
 
 Not queried
 
  0.8915404
 
       NA
 
           NA
  
 
  
 10
 
 116528187
 
 116528363
 
  ENSEMBL   UCSC 
 
  177
 
  4
 
 6155
 
 island
 
 0.071269
 
 0.2891419
 
 Not queried
 
  0.8914097
 
       NA
 
           NA
  
 
  
 5
 
 115152413
 
 115152494
 
  ENSEMBL   UCSC 
 
   82
 
  6
 
 32230
 
 tss
 
 0.018012
 
 0.0089812
 
 Not queried
 
  0.8912457
 
       NA
 
           NA
  
 
  
 12
 
  52216212
 
  52216298
 
  ENSEMBL   UCSC 
 
   87
 
  4
 
 9719
 
 tss;island
 
 0.689632
 
 0.8549925
 
 Not queried
 
  0.8909234
 
       NA
 
           NA
  
 
  
 17
 
  81052622
 
  81052999
 
  ENSEMBL   UCSC 
 
  378
 
  8
 
 19030
 
 island
 
 0.908935
 
 0.9437950
 
 Not queried
 
  0.8908588
 
       NA
 
           NA
  
 
  
 13
 
 114281582
 
 114281749
 
  ENSEMBL   UCSC 
 
  168
 
  4
 
 11296
 
 gene;island
 
 0.977576
 
 0.9597914
 
 Not queried
 
  0.8907437
 
       NA
 
           NA
  
 
  
 19
 
  50936038
 
  50936093
 
  ENSEMBL   UCSC 
 
   56
 
  4
 
 20978
 
 tss
 
 0.018569
 
 0.0127494
 
 Not queried
 
  0.8907419
 
       NA
 
           NA
  
 
  
 17
 
  45056626
 
  45056976
 
  ENSEMBL   UCSC 
 
  351
 
  8
 
 17438
 
 tss;island
 
 0.018848
 
 0.0293469
 
 Not queried
 
  0.8907244
 
       NA
 
           NA
  
 
  
 6
 
 100050480
 
 100051116
 
  ENSEMBL   UCSC 
 
  637
 
  8
 
 36356
 
 island
 
 0.030282
 
 0.0084180
 
 Not queried
 
  0.8906735
 
       NA
 
           NA
  
 
  
 14
 
 100658902
 
 100659150
 
  ENSEMBL   UCSC 
 
  249
 
  4
 
 13135
 
 island
 
 0.056701
 
 0.0253074
 
 Not queried
 
  0.8906041
 
       NA
 
           NA
  
 
  
 20
 
  25604700
 
  25604805
 
  ENSEMBL   UCSC 
 
  106
 
  7
 
 25653
 
 tss
 
 0.033554
 
 0.0115450
 
 Not queried
 
  0.8905841
 
       NA
 
           NA
  
 
  
 6
 
 108582561
 
 108582638
 
  ENSEMBL   UCSC 
 
   78
 
  4
 
 35159
 
 tss;island
 
 0.017938
 
 0.0135948
 
 Not queried
 
  0.8905768
 
       NA
 
           NA
  
 
  
 1
 
 235813839
 
 235814462
 
  ENSEMBL   UCSC 
 
  624
 
 11
 
 2336
 
 tss;island
 
 0.529153
 
 0.1574631
 
 Not queried
 
  0.8905441
 
       NA
 
           NA
  
 
  
 14
 
  61447816
 
  61447923
 
  ENSEMBL   UCSC 
 
  108
 
  5
 
 12442
 
 tss;island
 
 0.005110
 
 0.0037536
 
 Not queried
 
  0.8902796
 
       NA
 
           NA
  
 
  
 2
 
 242987510
 
 242987840
 
  ENSEMBL   UCSC 
 
  331
 
  4
 
 25320
 
 island
 
 0.807738
 
 0.9029789
 
 Not queried
 
  0.8902578
 
       NA
 
           NA
  
 
  
 17
 
  42402347
 
  42402401
 
  ENSEMBL   UCSC 
 
   55
 
  4
 
 17407
 
 tss
 
 0.003090
 
 0.0035205
 
 Not queried
 
  0.8901953
 
       NA
 
           NA
  
 
  
 17
 
  40835849
 
  40836125
 
  ENSEMBL   UCSC 
 
  277
 
  4
 
 16586
 
 gene;island
 
 0.543600
 
 0.4588700
 
 Not queried
 
  0.8901683
 
       NA
 
           NA
  
 
  
 11
 
  11862470
 
  11862879
 
  ENSEMBL   UCSC 
 
  410
 
  5
 
 7152
 
 tss;island
 
 0.269557
 
 0.5744911
 
 Not queried
 
  0.8901608
 
       NA
 
           NA
  
 
  
 10
 
  13628544
 
  13628859
 
  ENSEMBL   UCSC 
 
  316
 
  7
 
 4957
 
 tss
 
 0.005163
 
 0.0052975
 
 Not queried
 
  0.8896772
 
       NA
 
           NA
  
 
  
 7
 
  23338735
 
  23338897
 
  ENSEMBL   UCSC 
 
  163
 
  8
 
 37471
 
 tss
 
 0.004659
 
 0.0063309
 
 Not queried
 
  0.8895570
 
       NA
 
           NA
  
 
  
 3
 
 138739722
 
 138740022
 
  ENSEMBL   UCSC 
 
  301
 
  7
 
 29533
 
 island
 
 0.749223
 
 0.5750955
 
 Not queried
 
  0.8894914
 
       NA
 
           NA
  
 
  
 6
 
  79576851
 
  79577260
 
  ENSEMBL   UCSC 
 
  410
 
 11
 
 36271
 
 island
 
 0.006153
 
 0.0039173
 
 Not queried
 
  0.8893455
 
       NA
 
           NA
  
 
  
 19
 
  21511996
 
  21512202
 
  ENSEMBL   UCSC 
 
  207
 
  6
 
 19958
 
 gene
 
 0.021890
 
 0.0099781
 
 Not queried
 
  0.8893315
 
       NA
 
           NA
  
 
  
 16
 
  31227884
 
  31228555
 
  ENSEMBL   UCSC 
 
  672
 
  9
 
 14735
 
 gene;island
 
 0.047316
 
 0.0316802
 
 Not queried
 
  0.8887433
 
       NA
 
           NA
  
 
  
 13
 
 114876061
 
 114876521
 
  ENSEMBL   UCSC 
 
  461
 
  4
 
 11315
 
 gene;island
 
 0.461388
 
 0.6178941
 
 Not queried
 
  0.8887394
 
       NA
 
           NA
  
 
  
 1
 
  16070629
 
  16070820
 
  ENSEMBL   UCSC 
 
  192
 
  4
 
 250
 
 gene;island
 
 0.656999
 
 0.7989242
 
 Not queried
 
  0.8885407
 
       NA
 
           NA
  
 
  
 15
 
 100890907
 
 100890996
 
  ENSEMBL   UCSC 
 
   90
 
  4
 
 13928
 
 tss;island
 
 0.908946
 
 0.9434785
 
 Not queried
 
  0.8884110
 
       NA
 
           NA
  
 
  
 3
 
  57742858
 
  57743171
 
  ENSEMBL   UCSC 
 
  314
 
  6
 
 28412
 
 tss;island
 
 0.306477
 
 0.0654934
 
 Not queried
 
  0.8882968
 
       NA
 
           NA
  
 
  
 8
 
 102092795
 
 102092965
 
  ENSEMBL   UCSC 
 
  171
 
  4
 
 40502
 
 island
 
 0.028707
 
 0.1594901
 
 Not queried
 
  0.8882484
 
       NA
 
           NA
  
 
  
 7
 
  27142100
 
  27142275
 
  ENSEMBL   UCSC 
 
  176
 
  4
 
 36928
 
 gene
 
 0.498394
 
 0.1301768
 
 Not queried
 
  0.8881354
 
       NA
 
           NA
  
 
  
 17
 
  67410623
 
  67410806
 
  ENSEMBL   UCSC 
 
  184
 
  7
 
 17610
 
 tss
 
 0.023613
 
 0.0243139
 
 Not queried
 
  0.8881281
 
       NA
 
           NA
  
 
  
 17
 
  81042782
 
  81043176
 
  ENSEMBL   UCSC 
 
  395
 
  4
 
 16975
 
 gene;island
 
 0.669203
 
 0.7571180
 
 Not queried
 
  0.8878835
 
       NA
 
           NA
  
 
  
 11
 
  67407037
 
  67407224
 
  ENSEMBL   UCSC 
 
  188
 
  5
 
 7499
 
 tss
 
 0.547790
 
 0.9096205
 
 Not queried
 
  0.8877654
 
       NA
 
           NA
  
 
  
 18
 
   4455337
 
   4455862
 
  ENSEMBL   UCSC 
 
  526
 
  5
 
 19368
 
 island
 
 0.034616
 
 0.0165749
 
 Not queried
 
  0.8873014
 
       NA
 
           NA
  
 
  
 19
 
  49939549
 
  49940062
 
  ENSEMBL   UCSC 
 
  514
 
  6
 
 20150
 
 gene;island
 
 0.101312
 
 0.3762981
 
 Not queried
 
  0.8872598
 
       NA
 
           NA
  
 
  
 8
 
  11324666
 
  11324817
 
  ENSEMBL   UCSC 
 
  152
 
  4
 
 39551
 
 tss;island
 
 0.008899
 
 0.0047641
 
 Not queried
 
  0.8867930
 
       NA
 
           NA
  
 
  
 19
 
  55549722
 
  55549842
 
  ENSEMBL   UCSC 
 
  121
 
  6
 
 21068
 
 tss
 
 0.918014
 
 0.8138319
 
 Not queried
 
  0.8867505
 
       NA
 
           NA
  
 
  
 20
 
  34129839
 
  34130163
 
  ENSEMBL   UCSC 
 
  325
 
  5
 
 25406
 
 gene
 
 0.008504
 
 0.0044378
 
 Not queried
 
  0.8866748
 
       NA
 
           NA
  
 
  
 8
 
  41386345
 
  41386864
 
  ENSEMBL   UCSC 
 
  520
 
  8
 
 40244
 
 island
 
 0.010787
 
 0.0148015
 
 Not queried
 
  0.8866272
 
       NA
 
           NA
  
 
  
 7
 
  24323559
 
  24323939
 
  ENSEMBL   UCSC 
 
  381
 
  8
 
 38317
 
 island
 
 0.020027
 
 0.0065238
 
 Not queried
 
  0.8865411
 
       NA
 
           NA
  
 
  
 2
 
 192015279
 
 192015918
 
  ENSEMBL   UCSC 
 
  640
 
 10
 
 22962
 
 gene
 
 0.013196
 
 0.0231908
 
 Not queried
 
  0.8864845
 
       NA
 
           NA
  
 
  
 2
 
 196521297
 
 196521792
 
  ENSEMBL   UCSC 
 
  496
 
 11
 
 23791
 
 tss
 
 0.006379
 
 0.0080636
 
 Not queried
 
  0.8864310
 
       NA
 
           NA
  
 
  
 2
 
 196521297
 
 196521989
 
  ENSEMBL   UCSC 
 
  693
 
 13
 
 24971
 
 island
 
 0.006379
 
 0.0080636
 
 Not queried
 
  0.8864310
 
       NA
 
           NA
  
 
  
 6
 
  31371423
 
  31371967
 
  ENSEMBL   UCSC 
 
  545
 
 22
 
 33871
 
 gene
 
 0.009330
 
 0.0040032
 
 Not queried
 
  0.8862849
 
       NA
 
           NA
  
 
  
 11
 
 133938676
 
 133938810
 
  ENSEMBL   UCSC 
 
  135
 
  4
 
 7827
 
 tss
 
 0.025519
 
 0.0138365
 
 Not queried
 
  0.8862518
 
       NA
 
           NA
  
 
  
 12
 
 114843188
 
 114843955
 
  ENSEMBL   UCSC 
 
  768
 
 10
 
 9326
 
 gene;island
 
 0.013861
 
 0.0252096
 
 Not queried
 
  0.8862496
 
       NA
 
           NA
  
 
  
 19
 
   4670319
 
   4670669
 
  ENSEMBL   UCSC 
 
  351
 
 10
 
 21385
 
 island
 
 0.013264
 
 0.0068509
 
 Not queried
 
  0.8861907
 
       NA
 
           NA
  
 
  
 10
 
 133949683
 
 133950036
 
  ENSEMBL   UCSC 
 
  354
 
  4
 
 4852
 
 gene;island
 
 0.319239
 
 0.4318314
 
 Not queried
 
  0.8860408
 
       NA
 
           NA
  
 
  
 19
 
  15618961
 
  15619329
 
  ENSEMBL   UCSC 
 
  369
 
  4
 
 20565
 
 tss
 
 0.265929
 
 0.3195005
 
 Not queried
 
  0.8859866
 
       NA
 
           NA
  
 
  
 5
 
  39721549
 
  39721760
 
  ENSEMBL   UCSC 
 
  212
 
  4
 
 32749
 
 island
 
 0.864232
 
 0.9193701
 
 Not queried
 
  0.8859687
 
       NA
 
           NA
  
 
  
 5
 
 174151043
 
 174151779
 
  ENSEMBL   UCSC 
 
  737
 
  9
 
 33416
 
 island
 
 0.051493
 
 0.1429931
 
 Not queried
 
  0.8859552
 
       NA
 
           NA
  
 
  
 16
 
   1670054
 
   1670473
 
  ENSEMBL   UCSC 
 
  420
 
  4
 
 14579
 
 gene
 
 0.567917
 
 0.6695452
 
 Not queried
 
  0.8858789
 
       NA
 
           NA
  
 
  
 18
 
  33647344
 
  33647664
 
  ENSEMBL   UCSC 
 
  321
 
  6
 
 19468
 
 island
 
 0.000856
 
 0.0006385
 
 Not queried
 
  0.8857530
 
       NA
 
           NA
  
 
  
 3
 
  49208514
 
  49209160
 
  ENSEMBL   UCSC 
 
  647
 
  9
 
 29111
 
 island
 
 0.032884
 
 0.0182622
 
 Not queried
 
  0.8855958
 
       NA
 
           NA
  
 
  
 16
 
  25026707
 
  25026956
 
  ENSEMBL   UCSC 
 
  250
 
  7
 
 15146
 
 tss
 
 0.005897
 
 0.0040689
 
 Not queried
 
  0.8855305
 
       NA
 
           NA
  
 
  
 8
 
  65492528
 
  65492936
 
  ENSEMBL   UCSC 
 
  409
 
 10
 
 40347
 
 island
 
 0.002472
 
 0.0011713
 
 Not queried
 
  0.8855181
 
       NA
 
           NA
  
 
  
 17
 
  74944775
 
  74944970
 
  ENSEMBL   UCSC 
 
  196
 
  4
 
 16801
 
 gene;island
 
 0.448417
 
 0.5764485
 
 Not queried
 
  0.8853450
 
       NA
 
           NA
  
 
  
 11
 
  86748885
 
  86749237
 
  ENSEMBL   UCSC 
 
  353
 
  4
 
 6856
 
 gene
 
 0.002971
 
 0.0019886
 
 Not queried
 
  0.8853246
 
       NA
 
           NA
  
 
  
 7
 
  25219840
 
  25220162
 
  ENSEMBL   UCSC 
 
  323
 
  9
 
 37480
 
 tss
 
 0.009692
 
 0.0058848
 
 Not queried
 
  0.8853083
 
       NA
 
           NA
  
 
  
 2
 
  10091661
 
  10091721
 
  ENSEMBL   UCSC 
 
   61
 
  5
 
 23175
 
 tss
 
 0.020448
 
 0.0115562
 
 Not queried
 
  0.8852537
 
       NA
 
           NA
  
 
  
 16
 
  68344995
 
  68345214
 
  ENSEMBL   UCSC 
 
  220
 
  7
 
 14814
 
 gene
 
 0.003632
 
 0.0020022
 
 Not queried
 
  0.8849947
 
       NA
 
           NA
  
 
  
 6
 
  32032689
 
  32033006
 
  ENSEMBL   UCSC 
 
  318
 
 10
 
 34028
 
 gene
 
 0.569787
 
 0.7480489
 
 Not queried
 
  0.8849837
 
       NA
 
           NA
  
 
  
 6
 
  32546614
 
  32546743
 
  ENSEMBL   UCSC 
 
  130
 
  4
 
 34119
 
 gene
 
 0.945791
 
 0.8222970
 
 Not queried
 
  0.8849060
 
       NA
 
           NA
  
 
  
 4
 
  89205684
 
  89206201
 
  ENSEMBL   UCSC 
 
  518
 
 10
 
 31172
 
 island
 
 0.003358
 
 0.0060069
 
 Not queried
 
  0.8848150
 
       NA
 
           NA
  
 
  
 6
 
  30951917
 
  30952175
 
  ENSEMBL   UCSC 
 
  259
 
  4
 
 33844
 
 gene
 
 0.504781
 
 0.5300187
 
 Not queried
 
  0.8847987
 
       NA
 
           NA
  
 
  
 8
 
  72469430
 
  72469560
 
  ENSEMBL   UCSC 
 
  131
 
  4
 
 40380
 
 island
 
 0.072666
 
 0.1288114
 
 Not queried
 
  0.8846299
 
       NA
 
           NA
  
 
  
 5
 
 134240760
 
 134240791
 
  ENSEMBL   UCSC 
 
   32
 
  6
 
 32300
 
 tss;island
 
 0.002716
 
 0.0043670
 
 Not queried
 
  0.8845814
 
       NA
 
           NA
  
 
  
 16
 
  19535085
 
  19535170
 
  ENSEMBL   UCSC 
 
   86
 
  5
 
 15117
 
 tss
 
 0.007599
 
 0.0088351
 
 Not queried
 
  0.8843880
 
       NA
 
           NA
  
 
  
 16
 
  71495698
 
  71496082
 
  ENSEMBL   UCSC 
 
  385
 
  4
 
 14831
 
 gene
 
 0.002078
 
 0.0038445
 
 Not queried
 
  0.8842861
 
       NA
 
           NA
  
 
  
 8
 
  22526663
 
  22526889
 
  ENSEMBL   UCSC 
 
  227
 
  5
 
 39593
 
 tss
 
 0.023972
 
 0.0293253
 
 Not queried
 
  0.8842304
 
       NA
 
           NA
  
 
  
 10
 
  81107507
 
  81108060
 
  ENSEMBL   UCSC 
 
  554
 
  5
 
 4650
 
 gene;island
 
 0.000779
 
 0.0026541
 
 Not queried
 
  0.8842042
 
       NA
 
           NA
  
 
  
 20
 
  62738197
 
  62738358
 
  ENSEMBL   UCSC 
 
  162
 
  5
 
 25914
 
 tss
 
 0.784803
 
 0.8509438
 
 Not queried
 
  0.8838725
 
       NA
 
           NA
  
 
  
 19
 
  16466298
 
  16466823
 
  ENSEMBL   UCSC 
 
  526
 
  6
 
 21718
 
 island
 
 0.739188
 
 0.9533334
 
 Not queried
 
  0.8838082
 
       NA
 
           NA
  
 
  
 8
 
   1796429
 
   1796676
 
  ENSEMBL   UCSC 
 
  248
 
  4
 
 39179
 
 gene;island
 
 0.677180
 
 0.7551860
 
 Not queried
 
  0.8836044
 
       NA
 
           NA
  
 
  
 17
 
  21495693
 
  21495791
 
  ENSEMBL   UCSC 
 
   99
 
  4
 
 18126
 
 island
 
 0.641991
 
 0.7232833
 
 Not queried
 
  0.8835504
 
       NA
 
           NA
  
 
  
 8
 
   1579039
 
   1579240
 
  ENSEMBL   UCSC 
 
  202
 
  4
 
 39169
 
 gene;island
 
 0.608028
 
 0.6865092
 
 Not queried
 
  0.8835166
 
       NA
 
           NA
  
 
  
 14
 
  95027382
 
  95027692
 
  ENSEMBL   UCSC 
 
  311
 
  4
 
 12584
 
 tss
 
 0.190839
 
 0.4374135
 
 Not queried
 
  0.8833815
 
       NA
 
           NA
  
 
  
 15
 
  78632572
 
  78632861
 
  ENSEMBL   UCSC 
 
  290
 
  8
 
 14352
 
 island
 
 0.025878
 
 0.0268640
 
 Not queried
 
  0.8833347
 
       NA
 
           NA
  
 
  
 19
 
  57078727
 
  57078794
 
  ENSEMBL   UCSC 
 
   68
 
  5
 
 21101
 
 tss;island
 
 0.031385
 
 0.0555143
 
 Not queried
 
  0.8831315
 
       NA
 
           NA
  
 
  
 19
 
  42811017
 
  42811173
 
  ENSEMBL   UCSC 
 
  157
 
  4
 
 20069
 
 gene;island
 
 0.946166
 
 0.9621861
 
 Not queried
 
  0.8830249
 
       NA
 
           NA
  
 
  
 11
 
  65685702
 
  65686288
 
  ENSEMBL   UCSC 
 
  587
 
  6
 
 6718
 
 gene;tss;island
 
 0.018406
 
 0.0173131
 
 Not queried
 
  0.8828107
 
       NA
 
           NA
  
 
  
 4
 
   8271196
 
   8271414
 
  ENSEMBL   UCSC 
 
  219
 
  6
 
 30296
 
 tss
 
 0.007333
 
 0.0353477
 
 Not queried
 
  0.8827140
 
       NA
 
           NA
  
 
  
 21
 
  44588552
 
  44589128
 
  ENSEMBL   UCSC 
 
  577
 
  9
 
 26574
 
 tss
 
 0.463204
 
 0.6981401
 
 Not queried
 
  0.8826679
 
       NA
 
           NA
  
 
  
 19
 
  10697690
 
  10698098
 
  ENSEMBL   UCSC 
 
  409
 
  7
 
 21540
 
 island
 
 0.306097
 
 0.1426065
 
 Not queried
 
  0.8825304
 
       NA
 
           NA
  
 
  
 19
 
  53073242
 
  53073420
 
  ENSEMBL   UCSC 
 
  179
 
  7
 
 21016
 
 tss
 
 0.074273
 
 0.0392972
 
 Not queried
 
  0.8823948
 
       NA
 
           NA
  
 
  
 19
 
  53073242
 
  53074012
 
  ENSEMBL   UCSC 
 
  771
 
 11
 
 22360
 
 island
 
 0.069645
 
 0.0258776
 
 Not queried
 
  0.8823948
 
       NA
 
           NA
  
 
  
 3
 
  12837740
 
  12838266
 
  ENSEMBL   UCSC 
 
  527
 
  8
 
 28894
 
 island
 
 0.021569
 
 0.0340665
 
 Not queried
 
  0.8822741
 
       NA
 
           NA
  
 
  
 10
 
  46222474
 
  46222657
 
  ENSEMBL   UCSC 
 
  184
 
  4
 
 5716
 
 island
 
 0.005419
 
 0.0092772
 
 Not queried
 
  0.8822145
 
       NA
 
           NA
  
 
  
 10
 
  49893336
 
  49893463
 
  ENSEMBL   UCSC 
 
  128
 
  6
 
 5069
 
 tss
 
 0.008593
 
 0.0041173
 
 Not queried
 
  0.8821030
 
       NA
 
           NA
  
 
  
 10
 
  96305598
 
  96305691
 
  ENSEMBL   UCSC 
 
   94
 
  4
 
 4686
 
 gene
 
 0.004369
 
 0.0032268
 
 Not queried
 
  0.8819610
 
       NA
 
           NA
  
 
  
 10
 
 118030848
 
 118031256
 
  ENSEMBL   UCSC 
 
  409
 
  4
 
 4772
 
 gene;island
 
 0.044796
 
 0.1262715
 
 Not queried
 
  0.8819441
 
       NA
 
           NA
  
 
  
 15
 
  57668039
 
  57668261
 
  ENSEMBL   UCSC 
 
  223
 
  4
 
 13694
 
 tss;island
 
 0.949949
 
 0.8012857
 
 Not queried
 
  0.8816446
 
       NA
 
           NA
  
 
  
 11
 
  18610297
 
  18610557
 
  ENSEMBL   UCSC 
 
  261
 
  7
 
 7187
 
 tss
 
 0.003506
 
 0.0035264
 
 Not queried
 
  0.8816005
 
       NA
 
           NA
  
 
  
 10
 
 105036611
 
 105036863
 
  ENSEMBL   UCSC 
 
  253
 
  7
 
 5334
 
 tss;island
 
 0.009372
 
 0.0233629
 
 Not queried
 
  0.8815850
 
       NA
 
           NA
  
 
  
 1
 
   3028813
 
   3029070
 
  ENSEMBL   UCSC 
 
  258
 
  6
 
 100
 
 gene;island
 
 0.493984
 
 0.6705939
 
 Not queried
 
  0.8815013
 
       NA
 
           NA
  
 
  
 13
 
  24734712
 
  24734795
 
  ENSEMBL   UCSC 
 
   84
 
  4
 
 11345
 
 tss
 
 0.010533
 
 0.0042381
 
 Not queried
 
  0.8814715
 
       NA
 
           NA
  
 
  
 3
 
  69129230
 
  69129751
 
  ENSEMBL   UCSC 
 
  522
 
  6
 
 29271
 
 island
 
 0.006420
 
 0.0082437
 
 Not queried
 
  0.8814434
 
       NA
 
           NA
  
 
  
 12
 
  56101736
 
  56102252
 
  ENSEMBL   UCSC 
 
  517
 
  6
 
 9767
 
 tss
 
 0.012277
 
 0.0133819
 
 Not queried
 
  0.8813964
 
       NA
 
           NA
  
 
  
 5
 
 156886816
 
 156887129
 
  ENSEMBL   UCSC 
 
  314
 
  7
 
 33342
 
 island
 
 0.020553
 
 0.0395153
 
 Not queried
 
  0.8812398
 
       NA
 
           NA
  
 
  
 4
 
 154170410
 
 154170822
 
  ENSEMBL   UCSC 
 
  413
 
  6
 
 30153
 
 gene;island
 
 0.009331
 
 0.0046370
 
 Not queried
 
  0.8810659
 
       NA
 
           NA
  
 
  
 2
 
 119916431
 
 119916686
 
  ENSEMBL   UCSC 
 
  256
 
  7
 
 24651
 
 island
 
 0.022648
 
 0.0725458
 
 Not queried
 
  0.8808738
 
       NA
 
           NA
  
 
  
 12
 
  42984093
 
  42984466
 
  ENSEMBL   UCSC 
 
  374
 
  6
 
 9646
 
 tss;island
 
 0.031195
 
 0.0140047
 
 Not queried
 
  0.8806650
 
       NA
 
           NA
  
 
  
 15
 
  96960214
 
  96960725
 
  ENSEMBL   UCSC 
 
  512
 
  5
 
 14491
 
 island
 
 0.036720
 
 0.0828895
 
 Not queried
 
  0.8805409
 
       NA
 
           NA
  
 
  
 1
 
 117284260
 
 117284802
 
  ENSEMBL   UCSC 
 
  543
 
  7
 
 3645
 
 island
 
 0.967740
 
 0.8857126
 
 Not queried
 
  0.8803264
 
       NA
 
           NA
  
 
  
 1
 
   9883887
 
   9884042
 
  ENSEMBL   UCSC 
 
  156
 
  6
 
 208
 
 gene;island
 
 0.004127
 
 0.0045490
 
 Not queried
 
  0.8801648
 
       NA
 
           NA
  
 
  
 11
 
   1330593
 
   1330807
 
  ENSEMBL   UCSC 
 
  215
 
  5
 
 6416
 
 gene
 
 0.008625
 
 0.0102989
 
 Not queried
 
  0.8800949
 
       NA
 
           NA
  
 
  
 16
 
  89989024
 
  89989483
 
  ENSEMBL   UCSC 
 
  460
 
  4
 
 15434
 
 tss;island
 
 0.043616
 
 0.0126342
 
 Not queried
 
  0.8798730
 
       NA
 
           NA
  
 
  
 5
 
 140800398
 
 140800495
 
  ENSEMBL   UCSC 
 
   98
 
  4
 
 32382
 
 tss
 
 0.064040
 
 0.0303278
 
 Not queried
 
  0.8798392
 
       NA
 
           NA
  
 
  
 6
 
 152128258
 
 152128805
 
  ENSEMBL   UCSC 
 
  548
 
 18
 
 35305
 
 tss
 
 0.058177
 
 0.0316724
 
 Not queried
 
  0.8797638
 
       NA
 
           NA
  
 
  
 2
 
 105857809
 
 105858167
 
  ENSEMBL   UCSC 
 
  359
 
  8
 
 23552
 
 tss
 
 0.664034
 
 0.8282229
 
 Not queried
 
  0.8797357
 
       NA
 
           NA
  
 
  
 20
 
  43743835
 
  43744110
 
  ENSEMBL   UCSC 
 
  276
 
  5
 
 25769
 
 tss
 
 0.436926
 
 0.5586415
 
 Not queried
 
  0.8796030
 
       NA
 
           NA
  
 
  
 13
 
  52378287
 
  52378744
 
  ENSEMBL   UCSC 
 
  458
 
 10
 
 11453
 
 tss
 
 0.007766
 
 0.0049417
 
 Not queried
 
  0.8795864
 
       NA
 
           NA
  
 
  
 11
 
  61560157
 
  61560534
 
  ENSEMBL   UCSC 
 
  378
 
  7
 
 6634
 
 gene
 
 0.004740
 
 0.0042748
 
 Not queried
 
  0.8794967
 
       NA
 
           NA
  
 
  
 19
 
  58125438
 
  58126053
 
  ENSEMBL   UCSC 
 
  616
 
 11
 
 22477
 
 island
 
 0.019025
 
 0.0070522
 
 Not queried
 
  0.8794940
 
       NA
 
           NA
  
 
  
 2
 
 242702553
 
 242702932
 
  ENSEMBL   UCSC 
 
  380
 
  4
 
 23144
 
 gene
 
 0.975155
 
 0.9556300
 
 Not queried
 
  0.8794618
 
       NA
 
           NA
  
 
  
 11
 
  14993866
 
  14995322
 
  ENSEMBL   UCSC 
 
 1457
 
 34
 
 7167
 
 tss
 
 0.035606
 
 0.0645686
 
 Not queried
 
  0.8794557
 
       NA
 
           NA
  
 
  
 11
 
  14993378
 
  14995322
 
  ENSEMBL   UCSC 
 
 1945
 
 40
 
 8081
 
 island
 
 0.040851
 
 0.1190447
 
 Not queried
 
  0.8794557
 
       NA
 
           NA
  
 
  
 4
 
 109093158
 
 109093243
 
  ENSEMBL   UCSC 
 
   86
 
  4
 
 30539
 
 tss
 
 0.005959
 
 0.0051849
 
 Not queried
 
  0.8790971
 
       NA
 
           NA
  
 
  
 2
 
  45236630
 
  45236743
 
  ENSEMBL   UCSC 
 
  114
 
  7
 
 23334
 
 tss;island
 
 0.053810
 
 0.0659474
 
 Not queried
 
  0.8790819
 
       NA
 
           NA
  
 
  
 10
 
 134121413
 
 134121712
 
  ENSEMBL   UCSC 
 
  300
 
  7
 
 6299
 
 island
 
 0.006320
 
 0.0032902
 
 Not queried
 
  0.8789005
 
       NA
 
           NA
  
 
  
 5
 
  74349644
 
  74350214
 
  ENSEMBL   UCSC 
 
  571
 
  5
 
 32882
 
 island
 
 0.602395
 
 0.5993312
 
 Not queried
 
  0.8788088
 
       NA
 
           NA
  
 
  
 11
 
  65640137
 
  65640516
 
  ENSEMBL   UCSC 
 
  380
 
  6
 
 8485
 
 island
 
 0.004660
 
 0.0097185
 
 Not queried
 
  0.8787381
 
       NA
 
           NA
  
 
  
 2
 
 220428116
 
 220428308
 
  ENSEMBL   UCSC 
 
  193
 
  4
 
 23041
 
 gene;island
 
 0.477901
 
 0.6299461
 
 Not queried
 
  0.8786803
 
       NA
 
           NA
  
 
  
 21
 
  35287829
 
  35288096
 
  ENSEMBL   UCSC 
 
  268
 
  4
 
 26409
 
 gene
 
 0.017682
 
 0.0098631
 
 Not queried
 
  0.8785227
 
       NA
 
           NA
  
 
  
 17
 
  19648718
 
  19649293
 
  ENSEMBL   UCSC 
 
  576
 
 11
 
 16484
 
 gene;island
 
 0.849147
 
 0.8900564
 
 Not queried
 
  0.8784293
 
       NA
 
           NA
  
 
  
 1
 
 184005360
 
 184005990
 
  ENSEMBL   UCSC 
 
  631
 
  5
 
 920
 
 gene;island
 
 0.209111
 
 0.0238868
 
 Not queried
 
  0.8782674
 
       NA
 
           NA
  
 
  
 2
 
  56150925
 
  56151179
 
  ENSEMBL   UCSC 
 
  255
 
  6
 
 23366
 
 tss;island
 
 0.025087
 
 0.0600752
 
 Not queried
 
  0.8782081
 
       NA
 
           NA
  
 
  
 4
 
 124317779
 
 124317983
 
  ENSEMBL   UCSC 
 
  205
 
  8
 
 31291
 
 island
 
 0.002595
 
 0.0033730
 
 Not queried
 
  0.8781696
 
       NA
 
           NA
  
 
  
 5
 
  35617687
 
  35618055
 
  ENSEMBL   UCSC 
 
  369
 
 10
 
 32728
 
 island
 
 0.103585
 
 0.0200145
 
 Not queried
 
  0.8780699
 
       NA
 
           NA
  
 
  
 7
 
   5534412
 
   5534721
 
  ENSEMBL   UCSC 
 
  310
 
  4
 
 36880
 
 gene;island
 
 0.010733
 
 0.0070805
 
 Not queried
 
  0.8779566
 
       NA
 
           NA
  
 
  
 2
 
 191878686
 
 191878941
 
  ENSEMBL   UCSC 
 
  256
 
  4
 
 22961
 
 gene
 
 0.005578
 
 0.0066050
 
 Not queried
 
  0.8778449
 
       NA
 
           NA
  
 
  
 1
 
   2428864
 
   2429005
 
  ENSEMBL   UCSC 
 
  142
 
  4
 
 87
 
 gene
 
 0.087604
 
 0.2949155
 
 Not queried
 
  0.8778247
 
       NA
 
           NA
  
 
  
 12
 
  96350519
 
  96350795
 
  ENSEMBL   UCSC 
 
  277
 
  5
 
 9255
 
 gene;island
 
 0.779948
 
 0.8794330
 
 Not queried
 
  0.8776455
 
       NA
 
           NA
  
 
  
 13
 
  28491326
 
  28491975
 
  ENSEMBL   UCSC 
 
  650
 
  7
 
 11632
 
 island
 
 0.008467
 
 0.0283545
 
 Not queried
 
  0.8776293
 
       NA
 
           NA
  
 
  
 7
 
  97800895
 
  97801051
 
  ENSEMBL   UCSC 
 
  157
 
  4
 
 37110
 
 gene;island
 
 0.945295
 
 0.9568039
 
 Not queried
 
  0.8775057
 
       NA
 
           NA
  
 
  
 3
 
  49314370
 
  49314920
 
  ENSEMBL   UCSC 
 
  551
 
 11
 
 29113
 
 island
 
 0.016589
 
 0.0355561
 
 Not queried
 
  0.8772096
 
       NA
 
           NA
  
 
  
 6
 
  58287758
 
  58287938
 
  ENSEMBL   UCSC 
 
  181
 
  5
 
 35051
 
 tss
 
 0.013179
 
 0.0018655
 
 Not queried
 
  0.8771872
 
       NA
 
           NA
  
 
  
 10
 
 134842688
 
 134842984
 
  ENSEMBL   UCSC 
 
  297
 
  4
 
 6327
 
 island
 
 0.043684
 
 0.2759025
 
 Not queried
 
  0.8771134
 
       NA
 
           NA
  
 
  
 16
 
   3142911
 
   3143250
 
  ENSEMBL   UCSC 
 
  340
 
  6
 
 15051
 
 tss
 
 0.754527
 
 0.7512143
 
 Not queried
 
  0.8770908
 
       NA
 
           NA
  
 
  
 15
 
  31175309
 
  31175522
 
  ENSEMBL   UCSC 
 
  214
 
  4
 
 13979
 
 island
 
 0.680576
 
 0.6487732
 
 Not queried
 
  0.8769079
 
       NA
 
           NA
  
 
  
 19
 
  58978139
 
  58978454
 
  ENSEMBL   UCSC 
 
  316
 
  5
 
 21145
 
 tss
 
 0.014881
 
 0.0168039
 
 Not queried
 
  0.8766438
 
       NA
 
           NA
  
 
  
 6
 
 166711138
 
 166711488
 
  ENSEMBL   UCSC 
 
  351
 
  5
 
 36629
 
 island
 
 0.609649
 
 0.9553660
 
 Not queried
 
  0.8766344
 
       NA
 
           NA
  
 
  
 1
 
 192778168
 
 192778396
 
  ENSEMBL   UCSC 
 
  229
 
  4
 
 927
 
 gene
 
 0.010862
 
 0.0057818
 
 Not queried
 
  0.8764026
 
       NA
 
           NA
  
 
  
 10
 
  64028521
 
  64028750
 
  ENSEMBL   UCSC 
 
  230
 
  5
 
 5100
 
 tss
 
 0.020448
 
 0.0095184
 
 Not queried
 
  0.8762444
 
       NA
 
           NA
  
 
  
 1
 
 116380316
 
 116380575
 
  ENSEMBL   UCSC 
 
  260
 
  4
 
 704
 
 gene;island
 
 0.376306
 
 0.1858909
 
 Not queried
 
  0.8759573
 
       NA
 
           NA
  
 
  
 6
 
 121758653
 
 121758980
 
  ENSEMBL   UCSC 
 
  328
 
  4
 
 34460
 
 gene
 
 0.044374
 
 0.1073810
 
 Not queried
 
  0.8759044
 
       NA
 
           NA
  
 
  
 21
 
  44588552
 
  44589331
 
  ENSEMBL   UCSC 
 
  780
 
 11
 
 26716
 
 island
 
 0.639760
 
 0.7002151
 
 Not queried
 
  0.8758799
 
       NA
 
           NA
  
 
  
 8
 
   9760855
 
   9761171
 
  ENSEMBL   UCSC 
 
  317
 
  7
 
 39545
 
 tss;island
 
 0.034610
 
 0.0184341
 
 Not queried
 
  0.8758193
 
       NA
 
           NA
  
 
  
 11
 
  14380628
 
  14380999
 
  ENSEMBL   UCSC 
 
  372
 
  8
 
 7161
 
 tss;island
 
 0.012587
 
 0.0105271
 
 Not queried
 
  0.8758144
 
       NA
 
           NA
  
 
  
 18
 
  59992157
 
  59992501
 
  ENSEMBL   UCSC 
 
  345
 
  7
 
 19303
 
 tss;island
 
 0.031012
 
 0.0131979
 
 Not queried
 
  0.8755004
 
       NA
 
           NA
  
 
  
 17
 
  65040527
 
  65040616
 
  ENSEMBL   UCSC 
 
   90
 
  6
 
 17596
 
 tss
 
 0.735099
 
 0.7525036
 
 Not queried
 
  0.8754142
 
       NA
 
           NA
  
 
  
 19
 
  54177144
 
  54177261
 
  ENSEMBL   UCSC 
 
  118
 
  4
 
 21034
 
 tss
 
 0.412553
 
 0.7440089
 
 Not queried
 
  0.8753262
 
       NA
 
           NA
  
 
  
 18
 
  53255401
 
  53255894
 
  ENSEMBL   UCSC 
 
  494
 
  4
 
 19527
 
 island
 
 0.019374
 
 0.0059150
 
 Not queried
 
  0.8752913
 
       NA
 
           NA
  
 
  
 7
 
  27219224
 
  27219704
 
  ENSEMBL   UCSC 
 
  481
 
  4
 
 36940
 
 gene;island
 
 0.352335
 
 0.3386677
 
 Not queried
 
  0.8751443
 
       NA
 
           NA
  
 
  
 5
 
  71615866
 
  71616077
 
  ENSEMBL   UCSC 
 
  212
 
  4
 
 31641
 
 gene
 
 0.005256
 
 0.0035442
 
 Not queried
 
  0.8750270
 
       NA
 
           NA
  
 
  
 1
 
  85219864
 
  85220242
 
  ENSEMBL   UCSC 
 
  379
 
  5
 
 3414
 
 island
 
 0.947809
 
 0.9404339
 
 Not queried
 
  0.8749457
 
       NA
 
           NA
  
 
  
 10
 
 126107537
 
 126107861
 
  ENSEMBL   UCSC 
 
  325
 
  9
 
 5421
 
 tss
 
 0.027944
 
 0.0275288
 
 Not queried
 
  0.8749454
 
       NA
 
           NA
  
 
  
 3
 
 197676755
 
 197676828
 
  ENSEMBL   UCSC 
 
   74
 
  4
 
 28109
 
 gene;tss
 
 0.003305
 
 0.0041333
 
 Not queried
 
  0.8747479
 
       NA
 
           NA
  
 
  
 4
 
 101111617
 
 101111927
 
  ENSEMBL   UCSC 
 
  311
 
  5
 
 30516
 
 tss
 
 0.014221
 
 0.0175632
 
 Not queried
 
  0.8743004
 
       NA
 
           NA
  
 
  
 2
 
 107502023
 
 107502354
 
  ENSEMBL   UCSC 
 
  332
 
  4
 
 22817
 
 gene;island
 
 0.030081
 
 0.0319796
 
 Not queried
 
  0.8742368
 
       NA
 
           NA
  
 
  
 16
 
  66599843
 
  66600311
 
  ENSEMBL   UCSC 
 
  469
 
  9
 
 15281
 
 tss
 
 0.980831
 
 0.9624426
 
 Not queried
 
  0.8742005
 
       NA
 
           NA
  
 
  
 16
 
  66599843
 
  66600700
 
  ENSEMBL   UCSC 
 
  858
 
 13
 
 15989
 
 island
 
 0.980831
 
 0.9569809
 
 Not queried
 
  0.8742005
 
       NA
 
           NA
  
 
  
 13
 
 112984478
 
 112984976
 
  ENSEMBL   UCSC 
 
  499
 
  5
 
 11919
 
 island
 
 0.802610
 
 0.8741951
 
 Not queried
 
  0.8741057
 
       NA
 
           NA
  
 
  
 6
 
  26457928
 
  26458489
 
  ENSEMBL   UCSC 
 
  562
 
  9
 
 35536
 
 island
 
 0.009622
 
 0.0042587
 
 Not queried
 
  0.8740479
 
       NA
 
           NA
  
 
  
 2
 
   3691145
 
   3691651
 
  ENSEMBL   UCSC 
 
  507
 
  6
 
 22556
 
 gene;island
 
 0.726203
 
 0.7897848
 
 Not queried
 
  0.8738755
 
       NA
 
           NA
  
 
  
 20
 
  43104357
 
  43104476
 
  ENSEMBL   UCSC 
 
  120
 
  5
 
 25760
 
 tss
 
 0.021620
 
 0.0124986
 
 Not queried
 
  0.8738670
 
       NA
 
           NA
  
 
  
 20
 
  43104357
 
  43104574
 
  ENSEMBL   UCSC 
 
  218
 
  7
 
 26182
 
 island
 
 0.021620
 
 0.0124986
 
 Not queried
 
  0.8738670
 
       NA
 
           NA
  
 
  
 12
 
  69139955
 
  69140079
 
  ENSEMBL   UCSC 
 
  125
 
  4
 
 9210
 
 gene
 
 0.003281
 
 0.0051106
 
 Not queried
 
  0.8737706
 
       NA
 
           NA
  
 
  
 6
 
 170861803
 
 170862245
 
  ENSEMBL   UCSC 
 
  443
 
  7
 
 34583
 
 gene;island
 
 0.004528
 
 0.0049066
 
 Not queried
 
  0.8734598
 
       NA
 
           NA
  
 
  
 11
 
  32449163
 
  32450244
 
  ENSEMBL   UCSC 
 
 1082
 
  8
 
 6565
 
 gene;island
 
 0.160537
 
 0.4793520
 
 Not queried
 
  0.8733981
 
       NA
 
           NA
  
 
  
 14
 
  38091400
 
  38092175
 
  ENSEMBL   UCSC 
 
  776
 
  7
 
 12800
 
 island
 
 0.166719
 
 0.1379946
 
 Not queried
 
  0.8733440
 
       NA
 
           NA
  
 
  
 14
 
  38065127
 
  38065582
 
  ENSEMBL   UCSC 
 
  456
 
  6
 
 12363
 
 tss;island
 
 0.043346
 
 0.0254647
 
 Not queried
 
  0.8732787
 
       NA
 
           NA
  
 
  
 18
 
   3247527
 
   3247815
 
  ENSEMBL   UCSC 
 
  289
 
  4
 
 19038
 
 gene
 
 0.005871
 
 0.0114354
 
 Not queried
 
  0.8731442
 
       NA
 
           NA
  
 
  
 7
 
   1589295
 
   1589856
 
  ENSEMBL   UCSC 
 
  562
 
  6
 
 36761
 
 gene;island
 
 0.660857
 
 0.8042307
 
 Not queried
 
  0.8731302
 
       NA
 
           NA
  
 
  
 1
 
 156549118
 
 156549431
 
  ENSEMBL   UCSC 
 
  314
 
  4
 
 2017
 
 tss
 
 0.567247
 
 0.7940856
 
 Not queried
 
  0.8731228
 
       NA
 
           NA
  
 
  
 4
 
 119274013
 
 119274341
 
  ENSEMBL   UCSC 
 
  329
 
  4
 
 30561
 
 tss
 
 0.043672
 
 0.0117101
 
 Not queried
 
  0.8730205
 
       NA
 
           NA
  
 
  
 4
 
 119273827
 
 119274341
 
  ENSEMBL   UCSC 
 
  515
 
  6
 
 31266
 
 island
 
 0.043672
 
 0.0117101
 
 Not queried
 
  0.8730205
 
       NA
 
           NA
  
 
  
 5
 
 176764116
 
 176764419
 
  ENSEMBL   UCSC 
 
  304
 
  4
 
 31918
 
 gene;island
 
 0.981910
 
 0.9664925
 
 Not queried
 
  0.8728752
 
       NA
 
           NA
  
 
  
 12
 
  75728104
 
  75728458
 
  ENSEMBL   UCSC 
 
  355
 
  5
 
 9880
 
 tss
 
 0.002615
 
 0.0019845
 
 Not queried
 
  0.8728532
 
       NA
 
           NA
  
 
  
 1
 
 209957601
 
 209957750
 
  ENSEMBL   UCSC 
 
  150
 
  5
 
 987
 
 gene;island
 
 0.019569
 
 0.0138867
 
 Not queried
 
  0.8725854
 
       NA
 
           NA
  
 
  
 17
 
   8028735
 
   8028948
 
  ENSEMBL   UCSC 
 
  214
 
  4
 
 17988
 
 island
 
 0.017611
 
 0.0121689
 
 Not queried
 
  0.8722553
 
       NA
 
           NA
  
 
  
 13
 
  41885025
 
  41885311
 
  ENSEMBL   UCSC 
 
  287
 
  9
 
 11412
 
 tss;island
 
 0.004963
 
 0.0083866
 
 Not queried
 
  0.8721253
 
       NA
 
           NA
  
 
  
 6
 
  72130539
 
  72130799
 
  ENSEMBL   UCSC 
 
  261
 
  9
 
 35061
 
 tss
 
 0.092617
 
 0.3066436
 
 Not queried
 
  0.8720725
 
       NA
 
           NA
  
 
  
 2
 
  85109273
 
  85109418
 
  ENSEMBL   UCSC 
 
  146
 
  4
 
 23477
 
 tss;island
 
 0.003674
 
 0.0052065
 
 Not queried
 
  0.8716623
 
       NA
 
           NA
  
 
  
 6
 
 158937125
 
 158937485
 
  ENSEMBL   UCSC 
 
  361
 
  4
 
 36596
 
 island
 
 0.337737
 
 0.5423035
 
 Not queried
 
  0.8715932
 
       NA
 
           NA
  
 
  
 13
 
  51825774
 
  51826086
 
  ENSEMBL   UCSC 
 
  313
 
  4
 
 11170
 
 gene;island
 
 0.865157
 
 0.9209166
 
 Not queried
 
  0.8713647
 
       NA
 
           NA
  
 
  
 7
 
 158854447
 
 158854856
 
  ENSEMBL   UCSC 
 
  410
 
  4
 
 37383
 
 gene;island
 
 0.791465
 
 0.8739161
 
 Not queried
 
  0.8713225
 
       NA
 
           NA
  
 
  
 2
 
  61293073
 
  61293359
 
  ENSEMBL   UCSC 
 
  287
 
  9
 
 23376
 
 tss
 
 0.006956
 
 0.0047457
 
 Not queried
 
  0.8710299
 
       NA
 
           NA
  
 
  
 17
 
  33288564
 
  33288597
 
  ENSEMBL   UCSC 
 
   34
 
  4
 
 16518
 
 gene
 
 0.006609
 
 0.0039774
 
 Not queried
 
  0.8709535
 
       NA
 
           NA
  
 
  
 1
 
 173887009
 
 173887360
 
  ENSEMBL   UCSC 
 
  352
 
  5
 
 2101
 
 tss
 
 0.886724
 
 0.9335308
 
 Not queried
 
  0.8708142
 
       NA
 
           NA
  
 
  
 3
 
 164914996
 
 164915196
 
  ENSEMBL   UCSC 
 
  201
 
  5
 
 28700
 
 tss
 
 0.149016
 
 0.1959707
 
 Not queried
 
  0.8706959
 
       NA
 
           NA
  
 
  
 12
 
  99039011
 
  99039295
 
  ENSEMBL   UCSC 
 
  285
 
  6
 
 9937
 
 tss;island
 
 0.016297
 
 0.0095768
 
 Not queried
 
  0.8706389
 
       NA
 
           NA
  
 
  
 17
 
  38599271
 
  38599508
 
  ENSEMBL   UCSC 
 
  238
 
  4
 
 17334
 
 tss
 
 0.143497
 
 0.2358243
 
 Not queried
 
  0.8705623
 
       NA
 
           NA
  
 
  
 17
 
  38599271
 
  38599763
 
  ENSEMBL   UCSC 
 
  493
 
  6
 
 18306
 
 island
 
 0.068036
 
 0.0917072
 
 Not queried
 
  0.8705623
 
       NA
 
           NA
  
 
  
 19
 
   5455176
 
   5455638
 
  ENSEMBL   UCSC 
 
  463
 
  8
 
 21404
 
 island
 
 0.700510
 
 0.8963585
 
 Not queried
 
  0.8704737
 
       NA
 
           NA
  
 
  
 7
 
   2353682
 
   2354066
 
  ENSEMBL   UCSC 
 
  385
 
  4
 
 36800
 
 gene
 
 0.005656
 
 0.0024341
 
 Not queried
 
  0.8702943
 
       NA
 
           NA
  
 
  
 19
 
  54219381
 
  54219607
 
  ENSEMBL   UCSC 
 
  227
 
  4
 
 21037
 
 tss
 
 0.262581
 
 0.4264948
 
 Not queried
 
  0.8702873
 
       NA
 
           NA
  
 
  
 7
 
  97841334
 
  97841591
 
  ENSEMBL   UCSC 
 
  258
 
  6
 
 38709
 
 island
 
 0.933261
 
 0.7323108
 
 Not queried
 
  0.8702515
 
       NA
 
           NA
  
 
  
 13
 
  36429936
 
  36430134
 
  ENSEMBL   UCSC 
 
  199
 
  4
 
 11128
 
 gene
 
 0.012496
 
 0.0251419
 
 Not queried
 
  0.8700570
 
       NA
 
           NA
  
 
  
 5
 
 140709983
 
 140710237
 
  ENSEMBL   UCSC 
 
  255
 
  4
 
 32372
 
 tss;island
 
 0.393858
 
 0.1922205
 
 Not queried
 
  0.8699967
 
       NA
 
           NA
  
 
  
 3
 
  10436249
 
  10436869
 
  ENSEMBL   UCSC 
 
  621
 
  8
 
 28150
 
 tss
 
 0.323746
 
 0.4652901
 
 Not queried
 
  0.8698389
 
       NA
 
           NA
  
 
  
 14
 
  52734156
 
  52734397
 
  ENSEMBL   UCSC 
 
  242
 
  6
 
 12396
 
 tss
 
 0.193480
 
 0.0788572
 
 Not queried
 
  0.8696992
 
       NA
 
           NA
  
 
  
 14
 
  52734156
 
  52734529
 
  ENSEMBL   UCSC 
 
  374
 
  8
 
 12842
 
 island
 
 0.061304
 
 0.0563260
 
 Not queried
 
  0.8696992
 
       NA
 
           NA
  
 
  
 2
 
 162272107
 
 162272687
 
  ENSEMBL   UCSC 
 
  581
 
  7
 
 24821
 
 island
 
 0.049375
 
 0.0396133
 
 Not queried
 
  0.8695060
 
       NA
 
           NA
  
 
  
 7
 
 145813413
 
 145813439
 
  ENSEMBL   UCSC 
 
   27
 
  6
 
 37959
 
 tss
 
 0.062696
 
 0.1270770
 
 Not queried
 
  0.8694341
 
       NA
 
           NA
  
 
  
 19
 
  50921232
 
  50921745
 
  ENSEMBL   UCSC 
 
  514
 
  4
 
 20977
 
 tss
 
 0.010241
 
 0.0128002
 
 Not queried
 
  0.8694228
 
       NA
 
           NA
  
 
  
 8
 
   1136127
 
   1136538
 
  ENSEMBL   UCSC 
 
  412
 
  6
 
 40018
 
 island
 
 0.508558
 
 0.6655345
 
 Not queried
 
  0.8694055
 
       NA
 
           NA
  
 
  
 2
 
  47499521
 
  47499812
 
  ENSEMBL   UCSC 
 
  292
 
  4
 
 24281
 
 island
 
 0.012852
 
 0.0395115
 
 Not queried
 
  0.8693266
 
       NA
 
           NA
  
 
  
 6
 
  32076079
 
  32076592
 
  ENSEMBL   UCSC 
 
  514
 
  9
 
 34049
 
 gene
 
 0.010599
 
 0.0248904
 
 Not queried
 
  0.8693153
 
       NA
 
           NA
  
 
  
 16
 
  84270422
 
  84270722
 
  ENSEMBL   UCSC 
 
  301
 
  4
 
 14867
 
 gene;island
 
 0.845840
 
 0.8753413
 
 Not queried
 
  0.8692761
 
       NA
 
           NA
  
 
  
 6
 
 126660872
 
 126661251
 
  ENSEMBL   UCSC 
 
  380
 
  6
 
 35214
 
 tss
 
 0.019359
 
 0.0122725
 
 Not queried
 
  0.8692476
 
       NA
 
           NA
  
 
  
 5
 
 140344290
 
 140344745
 
  ENSEMBL   UCSC 
 
  456
 
  4
 
 31805
 
 gene;tss;island
 
 0.166449
 
 0.2662882
 
 Not queried
 
  0.8691718
 
       NA
 
           NA
  
 
  
 11
 
   3876513
 
   3876822
 
  ENSEMBL   UCSC 
 
  310
 
  8
 
 7101
 
 tss;island
 
 0.010765
 
 0.0059592
 
 Not queried
 
  0.8691312
 
       NA
 
           NA
  
 
  
 6
 
 127840328
 
 127840755
 
  ENSEMBL   UCSC 
 
  428
 
 10
 
 36473
 
 island
 
 0.096492
 
 0.1104944
 
 Not queried
 
  0.8689651
 
       NA
 
           NA
  
 
  
 12
 
   1026102
 
   1026392
 
  ENSEMBL   UCSC 
 
  291
 
  4
 
 8997
 
 gene
 
 0.979540
 
 0.9828933
 
 Not queried
 
  0.8689237
 
       NA
 
           NA
  
 
  
 8
 
  19674705
 
  19674847
 
  ENSEMBL   UCSC 
 
  143
 
  5
 
 39573
 
 tss;island
 
 0.007733
 
 0.0050372
 
 Not queried
 
  0.8687852
 
       NA
 
           NA
  
 
  
 1
 
 119535500
 
 119536064
 
  ENSEMBL   UCSC 
 
  565
 
  7
 
 3658
 
 island
 
 0.095433
 
 0.0375415
 
 Not queried
 
  0.8685834
 
       NA
 
           NA
  
 
  
 12
 
 131568441
 
 131568764
 
  ENSEMBL   UCSC 
 
  324
 
  5
 
 9424
 
 gene
 
 0.926638
 
 0.8812579
 
 Not queried
 
  0.8682896
 
       NA
 
           NA
  
 
  
 22
 
  44576869
 
  44577240
 
  ENSEMBL   UCSC 
 
  372
 
  8
 
 27211
 
 tss
 
 0.008741
 
 0.0266198
 
 Not queried
 
  0.8682284
 
       NA
 
           NA
  
 
  
 7
 
    766100
 
    766384
 
  ENSEMBL   UCSC 
 
  285
 
  7
 
 36717
 
 gene;island
 
 0.002368
 
 0.0051423
 
 Not queried
 
  0.8681950
 
       NA
 
           NA
  
 
  
 8
 
   2003810
 
   2004050
 
  ENSEMBL   UCSC 
 
  241
 
  5
 
 39187
 
 gene;island
 
 0.791522
 
 0.7131013
 
 Not queried
 
  0.8679957
 
       NA
 
           NA
  
 
  
 7
 
 150715634
 
 150716072
 
  ENSEMBL   UCSC 
 
  439
 
  4
 
 37282
 
 gene;island
 
 0.104292
 
 0.0663996
 
 Not queried
 
  0.8674068
 
       NA
 
           NA
  
 
  
 1
 
 115124302
 
 115124491
 
  ENSEMBL   UCSC 
 
  190
 
  7
 
 1851
 
 tss
 
 0.002790
 
 0.0050569
 
 Not queried
 
  0.8673951
 
       NA
 
           NA
  
 
  
 5
 
 133968566
 
 133968668
 
  ENSEMBL   UCSC 
 
  103
 
  6
 
 32295
 
 tss
 
 0.006744
 
 0.0052828
 
 Not queried
 
  0.8673481
 
       NA
 
           NA
  
 
  
 12
 
  32112763
 
  32113101
 
  ENSEMBL   UCSC 
 
  339
 
  6
 
 9093
 
 gene;island
 
 0.002244
 
 0.0026500
 
 Not queried
 
  0.8671637
 
       NA
 
           NA
  
 
  
 6
 
  29454557
 
  29455532
 
  ENSEMBL   UCSC 
 
  976
 
 15
 
 33659
 
 gene
 
 0.035479
 
 0.2126449
 
 Not queried
 
  0.8671442
 
       NA
 
           NA
  
 
  
 11
 
 125036088
 
 125036420
 
  ENSEMBL   UCSC 
 
  333
 
  4
 
 6957
 
 gene;island
 
 0.023603
 
 0.0462626
 
 Not queried
 
  0.8670040
 
       NA
 
           NA
  
 
  
 15
 
  83951663
 
  83952019
 
  ENSEMBL   UCSC 
 
  357
 
  4
 
 13458
 
 gene;island
 
 0.238964
 
 0.2984687
 
 Not queried
 
  0.8669319
 
       NA
 
           NA
  
 
  
 15
 
  74467432
 
  74467891
 
  ENSEMBL   UCSC 
 
  460
 
  4
 
 13419
 
 gene;island
 
 0.835161
 
 0.9183371
 
 Not queried
 
  0.8666303
 
       NA
 
           NA
  
 
  
 5
 
  32709071
 
  32709396
 
  ENSEMBL   UCSC 
 
  326
 
  4
 
 32719
 
 island
 
 0.024889
 
 0.0370133
 
 Not queried
 
  0.8664789
 
       NA
 
           NA
  
 
  
 12
 
  45269318
 
  45269790
 
  ENSEMBL   UCSC 
 
  473
 
  7
 
 9100
 
 gene;island
 
 0.006032
 
 0.0165035
 
 Not queried
 
  0.8662684
 
       NA
 
           NA
  
 
  
 2
 
 131862302
 
 131862633
 
  ENSEMBL   UCSC 
 
  332
 
  6
 
 24728
 
 island
 
 0.002319
 
 0.0016198
 
 Not queried
 
  0.8660489
 
       NA
 
           NA
  
 
  
 16
 
    898803
 
    899108
 
  ENSEMBL   UCSC 
 
  306
 
  4
 
 15494
 
 island
 
 0.474606
 
 0.6486933
 
 Not queried
 
  0.8659260
 
       NA
 
           NA
  
 
  
 4
 
 155662217
 
 155662515
 
  ENSEMBL   UCSC 
 
  299
 
  4
 
 31385
 
 island
 
 0.276232
 
 0.1341068
 
 Not queried
 
  0.8658821
 
       NA
 
           NA
  
 
  
 12
 
 122277214
 
 122277522
 
  ENSEMBL   UCSC 
 
  309
 
  4
 
 10923
 
 island
 
 0.027250
 
 0.0186910
 
 Not queried
 
  0.8655164
 
       NA
 
           NA
  
 
  
 8
 
    786658
 
    787298
 
  ENSEMBL   UCSC 
 
  641
 
  6
 
 40006
 
 island
 
 0.650817
 
 0.7661431
 
 Not queried
 
  0.8653428
 
       NA
 
           NA
  
 
  
 16
 
   1869043
 
   1869400
 
  ENSEMBL   UCSC 
 
  358
 
  5
 
 14586
 
 gene;island
 
 0.879156
 
 0.9526942
 
 Not queried
 
  0.8650663
 
       NA
 
           NA
  
 
  
 20
 
  32319621
 
  32319743
 
  ENSEMBL   UCSC 
 
  123
 
  5
 
 25686
 
 tss;island
 
 0.003143
 
 0.0073229
 
 Not queried
 
  0.8649904
 
       NA
 
           NA
  
 
  
 19
 
  45754802
 
  45755260
 
  ENSEMBL   UCSC 
 
  459
 
  5
 
 22127
 
 island
 
 0.019249
 
 0.0098021
 
 Not queried
 
  0.8646073
 
       NA
 
           NA
  
 
  
 19
 
  40719835
 
  40720114
 
  ENSEMBL   UCSC 
 
  280
 
  4
 
 20035
 
 gene;island
 
 0.973370
 
 0.9520600
 
 Not queried
 
  0.8642305
 
       NA
 
           NA
  
 
  
 2
 
 220118655
 
 220118770
 
  ENSEMBL   UCSC 
 
  116
 
  5
 
 23900
 
 tss
 
 0.005596
 
 0.0116915
 
 Not queried
 
  0.8641960
 
       NA
 
           NA
  
 
  
 14
 
  75230239
 
  75230530
 
  ENSEMBL   UCSC 
 
  292
 
  4
 
 12142
 
 gene;island
 
 0.006652
 
 0.0027507
 
 Not queried
 
  0.8641427
 
       NA
 
           NA
  
 
  
 10
 
  82363213
 
  82363434
 
  ENSEMBL   UCSC 
 
  222
 
  5
 
 4655
 
 gene
 
 0.946379
 
 0.9630066
 
 Not queried
 
  0.8641392
 
       NA
 
           NA
  
 
  
 17
 
  47074576
 
  47074883
 
  ENSEMBL   UCSC 
 
  308
 
  8
 
 18516
 
 island
 
 0.008434
 
 0.0068240
 
 Not queried
 
  0.8639921
 
       NA
 
           NA
  
 
  
 12
 
  49760222
 
  49760606
 
  ENSEMBL   UCSC 
 
  385
 
  4
 
 9688
 
 tss;island
 
 0.007644
 
 0.0056657
 
 Not queried
 
  0.8638392
 
       NA
 
           NA
  
 
  
 20
 
  61427684
 
  61427693
 
  ENSEMBL   UCSC 
 
   10
 
  4
 
 25880
 
 tss;island
 
 0.022619
 
 0.0040593
 
 Not queried
 
  0.8638085
 
       NA
 
           NA
  
 
  
 12
 
  56862350
 
  56862729
 
  ENSEMBL   UCSC 
 
  380
 
  5
 
 9180
 
 gene
 
 0.005647
 
 0.0037632
 
 Not queried
 
  0.8638011
 
       NA
 
           NA
  
 
  
 2
 
  27485967
 
  27486133
 
  ENSEMBL   UCSC 
 
  167
 
  5
 
 23260
 
 tss
 
 0.005876
 
 0.0050137
 
 Not queried
 
  0.8637931
 
       NA
 
           NA
  
 
  
 1
 
  32645027
 
  32645422
 
  ENSEMBL   UCSC 
 
  396
 
 10
 
 2984
 
 island
 
 0.008233
 
 0.0088261
 
 Not queried
 
  0.8637822
 
       NA
 
           NA
  
 
  
 15
 
  40861791
 
  40862171
 
  ENSEMBL   UCSC 
 
  381
 
  4
 
 13295
 
 gene;island
 
 0.014769
 
 0.0093012
 
 Not queried
 
  0.8636782
 
       NA
 
           NA
  
 
  
 3
 
 123813191
 
 123813504
 
  ENSEMBL   UCSC 
 
  314
 
  8
 
 28548
 
 tss
 
 0.290696
 
 0.5686189
 
 Not queried
 
  0.8636322
 
       NA
 
           NA
  
 
  
 19
 
  40732076
 
  40732316
 
  ENSEMBL   UCSC 
 
  241
 
  4
 
 20036
 
 gene;island
 
 0.009206
 
 0.0182454
 
 Not queried
 
  0.8635467
 
       NA
 
           NA
  
 
  
 1
 
  17281819
 
  17282055
 
  ENSEMBL   UCSC 
 
  237
 
  5
 
 263
 
 gene;island
 
 0.927173
 
 0.9585875
 
 Not queried
 
  0.8634779
 
       NA
 
           NA
  
 
  
 11
 
  71524500
 
  71524971
 
  ENSEMBL   UCSC 
 
  472
 
  5
 
 8623
 
 island
 
 0.068942
 
 0.2617968
 
 Not queried
 
  0.8633964
 
       NA
 
           NA
  
 
  
 14
 
  60557893
 
  60558163
 
  ENSEMBL   UCSC 
 
  271
 
  4
 
 12434
 
 tss;island
 
 0.030860
 
 0.0100927
 
 Not queried
 
  0.8630728
 
       NA
 
           NA
  
 
  
 3
 
 185827275
 
 185827485
 
  ENSEMBL   UCSC 
 
  211
 
  4
 
 28771
 
 tss;island
 
 0.007622
 
 0.0154274
 
 Not queried
 
  0.8629204
 
       NA
 
           NA
  
 
  
 10
 
 135029097
 
 135029461
 
  ENSEMBL   UCSC 
 
  365
 
  4
 
 4892
 
 gene;island
 
 0.626363
 
 0.7589658
 
 Not queried
 
  0.8627677
 
       NA
 
           NA
  
 
  
 15
 
  49255686
 
  49256042
 
  ENSEMBL   UCSC 
 
  357
 
  4
 
 13660
 
 tss
 
 0.027520
 
 0.0213544
 
 Not queried
 
  0.8626353
 
       NA
 
           NA
  
 
  
 4
 
  89080013
 
  89080423
 
  ENSEMBL   UCSC 
 
  411
 
  8
 
 30490
 
 tss;island
 
 0.043057
 
 0.0260103
 
 Not queried
 
  0.8625892
 
       NA
 
           NA
  
 
  
 14
 
  24610792
 
  24611268
 
  ENSEMBL   UCSC 
 
  477
 
 10
 
 12730
 
 island
 
 0.005792
 
 0.0116074
 
 Not queried
 
  0.8623316
 
       NA
 
           NA
  
 
  
 19
 
  51308339
 
  51308546
 
  ENSEMBL   UCSC 
 
  208
 
  4
 
 20987
 
 tss;island
 
 0.008549
 
 0.0445577
 
 Not queried
 
  0.8623200
 
       NA
 
           NA
  
 
  
 18
 
  72265164
 
  72265361
 
  ENSEMBL   UCSC 
 
  198
 
  8
 
 19325
 
 tss
 
 0.003083
 
 0.0022144
 
 Not queried
 
  0.8621769
 
       NA
 
           NA
  
 
  
 18
 
  72264895
 
  72265361
 
  ENSEMBL   UCSC 
 
  467
 
 12
 
 19572
 
 island
 
 0.003944
 
 0.0032812
 
 Not queried
 
  0.8621769
 
       NA
 
           NA
  
 
  
 20
 
   3218393
 
   3218579
 
  ENSEMBL   UCSC 
 
  187
 
  7
 
 25563
 
 tss
 
 0.807125
 
 0.8256133
 
 Not queried
 
  0.8621053
 
       NA
 
           NA
  
 
  
 20
 
   3218145
 
   3218579
 
  ENSEMBL   UCSC 
 
  435
 
  9
 
 25968
 
 island
 
 0.807125
 
 0.8899121
 
 Not queried
 
  0.8621053
 
       NA
 
           NA
  
 
  
 12
 
  48592270
 
  48592818
 
  ENSEMBL   UCSC 
 
  549
 
  5
 
 10367
 
 island
 
 0.063681
 
 0.0365132
 
 Not queried
 
  0.8617113
 
       NA
 
           NA
  
 
  
 12
 
  49932779
 
  49932860
 
  ENSEMBL   UCSC 
 
   82
 
  4
 
 9689
 
 tss
 
 0.020006
 
 0.0118586
 
 Not queried
 
  0.8616294
 
       NA
 
           NA
  
 
  
 8
 
    914335
 
    914572
 
  ENSEMBL   UCSC 
 
  238
 
  5
 
 40009
 
 island
 
 0.829200
 
 0.9108916
 
 Not queried
 
  0.8614728
 
       NA
 
           NA
  
 
  
 3
 
  79815639
 
  79815848
 
  ENSEMBL   UCSC 
 
  210
 
  4
 
 27863
 
 gene;island
 
 0.414399
 
 0.3544777
 
 Not queried
 
  0.8614478
 
       NA
 
           NA
  
 
  
 4
 
 188534448
 
 188534912
 
  ENSEMBL   UCSC 
 
  465
 
  6
 
 31490
 
 island
 
 0.485171
 
 0.7512047
 
 Not queried
 
  0.8613498
 
       NA
 
           NA
  
 
  
 3
 
  39230030
 
  39230320
 
  ENSEMBL   UCSC 
 
  291
 
  4
 
 27724
 
 gene
 
 0.493705
 
 0.6644237
 
 Not queried
 
  0.8611894
 
       NA
 
           NA
  
 
  
 16
 
  90030558
 
  90030838
 
  ENSEMBL   UCSC 
 
  281
 
  4
 
 14954
 
 gene;island
 
 0.629977
 
 0.7707535
 
 Not queried
 
  0.8611322
 
       NA
 
           NA
  
 
  
 16
 
  69344779
 
  69345210
 
  ENSEMBL   UCSC 
 
  432
 
  6
 
 15329
 
 tss
 
 0.010027
 
 0.0084324
 
 Not queried
 
  0.8610993
 
       NA
 
           NA
  
 
  
 6
 
  25732093
 
  25732239
 
  ENSEMBL   UCSC 
 
  147
 
  4
 
 35501
 
 island
 
 0.825695
 
 0.8924427
 
 Not queried
 
  0.8610279
 
       NA
 
           NA
  
 
  
 17
 
  42385923
 
  42386419
 
  ENSEMBL   UCSC 
 
  497
 
  8
 
 18397
 
 island
 
 0.028320
 
 0.0231722
 
 Not queried
 
  0.8605097
 
       NA
 
           NA
  
 
  
 1
 
   1098900
 
   1099166
 
  ENSEMBL   UCSC 
 
  267
 
  4
 
 2405
 
 island
 
 0.781672
 
 0.8616184
 
 Not queried
 
  0.8604606
 
       NA
 
           NA
  
 
  
 21
 
  34143261
 
  34143691
 
  ENSEMBL   UCSC 
 
  431
 
  5
 
 26401
 
 gene;tss;island
 
 0.019335
 
 0.0107780
 
 Not queried
 
  0.8601130
 
       NA
 
           NA
  
 
  
 12
 
  54673731
 
  54674114
 
  ENSEMBL   UCSC 
 
  384
 
  8
 
 9759
 
 tss;island
 
 0.006663
 
 0.0034842
 
 Not queried
 
  0.8598769
 
       NA
 
           NA
  
 
  
 3
 
 158288680
 
 158289160
 
  ENSEMBL   UCSC 
 
  481
 
 12
 
 29620
 
 island
 
 0.018206
 
 0.0336040
 
 Not queried
 
  0.8598453
 
       NA
 
           NA
  
 
  
 17
 
  75523939
 
  75524350
 
  ENSEMBL   UCSC 
 
  412
 
  5
 
 18810
 
 island
 
 0.045935
 
 0.0298878
 
 Not queried
 
  0.8597687
 
       NA
 
           NA
  
 
  
 8
 
  49835988
 
  49836230
 
  ENSEMBL   UCSC 
 
  243
 
  4
 
 40287
 
 island
 
 0.744395
 
 0.9026615
 
 Not queried
 
  0.8596302
 
       NA
 
           NA
  
 
  
 6
 
 146864752
 
 146864885
 
  ENSEMBL   UCSC 
 
  134
 
  4
 
 36544
 
 island
 
 0.074129
 
 0.1251341
 
 Not queried
 
  0.8596218
 
       NA
 
           NA
  
 
  
 7
 
 138601935
 
 138602271
 
  ENSEMBL   UCSC 
 
  337
 
  4
 
 37241
 
 gene
 
 0.460931
 
 0.6918820
 
 Not queried
 
  0.8595456
 
       NA
 
           NA
  
 
  
 9
 
  36258825
 
  36258971
 
  ENSEMBL   UCSC 
 
  147
 
  4
 
 40765
 
 gene;tss;island
 
 0.041414
 
 0.0995952
 
 Not queried
 
  0.8594928
 
       NA
 
           NA
  
 
  
 1
 
   1114912
 
   1115070
 
  ENSEMBL   UCSC 
 
  159
 
  4
 
 1143
 
 tss
 
 0.040274
 
 0.0347534
 
 Not queried
 
  0.8593792
 
       NA
 
           NA
  
 
  
 8
 
   1049167
 
   1049477
 
  ENSEMBL   UCSC 
 
  311
 
  5
 
 40010
 
 island
 
 0.390024
 
 0.4765589
 
 Not queried
 
  0.8593287
 
       NA
 
           NA
  
 
  
 1
 
 205782099
 
 205782872
 
  ENSEMBL   UCSC 
 
  774
 
  9
 
 4137
 
 island
 
 0.002810
 
 0.0033158
 
 Not queried
 
  0.8593110
 
       NA
 
           NA
  
 
  
 2
 
 105857809
 
 105858511
 
  ENSEMBL   UCSC 
 
  703
 
 11
 
 24584
 
 island
 
 0.621145
 
 0.7723647
 
 Not queried
 
  0.8590791
 
       NA
 
           NA
  
 
  
 3
 
 181428242
 
 181428697
 
  ENSEMBL   UCSC 
 
  456
 
 14
 
 28739
 
 tss
 
 0.106443
 
 0.0825130
 
 Not queried
 
  0.8588274
 
       NA
 
           NA
  
 
  
 4
 
  41992067
 
  41992621
 
  ENSEMBL   UCSC 
 
  555
 
 11
 
 31012
 
 island
 
 0.004592
 
 0.0047193
 
 Not queried
 
  0.8586977
 
       NA
 
           NA
  
 
  
 7
 
  27190431
 
  27190849
 
  ENSEMBL   UCSC 
 
  419
 
  5
 
 38350
 
 island
 
 0.028359
 
 0.0346228
 
 Not queried
 
  0.8586025
 
       NA
 
           NA
  
 
  
 1
 
 109288968
 
 109289295
 
  ENSEMBL   UCSC 
 
  328
 
  7
 
 3545
 
 island
 
 0.008977
 
 0.0129233
 
 Not queried
 
  0.8584855
 
       NA
 
           NA
  
 
  
 7
 
 142636371
 
 142636518
 
  ENSEMBL   UCSC 
 
  148
 
  4
 
 37949
 
 tss
 
 0.653577
 
 0.5967300
 
 Not queried
 
  0.8583174
 
       NA
 
           NA
  
 
  
 16
 
  80574553
 
  80574757
 
  ENSEMBL   UCSC 
 
  205
 
  4
 
 15374
 
 tss
 
 0.025353
 
 0.0179552
 
 Not queried
 
  0.8581658
 
       NA
 
           NA
  
 
  
 16
 
  80574553
 
  80575018
 
  ENSEMBL   UCSC 
 
  466
 
  6
 
 16117
 
 island
 
 0.022823
 
 0.0133027
 
 Not queried
 
  0.8581658
 
       NA
 
           NA
  
 
  
 12
 
   6809887
 
   6809939
 
  ENSEMBL   UCSC 
 
   53
 
  4
 
 9523
 
 tss
 
 0.003192
 
 0.0067414
 
 Not queried
 
  0.8580152
 
       NA
 
           NA
  
 
  
 7
 
  98030477
 
  98030795
 
  ENSEMBL   UCSC 
 
  319
 
  4
 
 37746
 
 tss
 
 0.041802
 
 0.0235159
 
 Not queried
 
  0.8579936
 
       NA
 
           NA
  
 
  
 4
 
   1581921
 
   1582248
 
  ENSEMBL   UCSC 
 
  328
 
  4
 
 30793
 
 island
 
 0.016506
 
 0.0128954
 
 Not queried
 
  0.8579765
 
       NA
 
           NA
  
 
  
 5
 
  92931623
 
  92932120
 
  ENSEMBL   UCSC 
 
  498
 
  5
 
 32962
 
 island
 
 0.197772
 
 0.2582243
 
 Not queried
 
  0.8578286
 
       NA
 
           NA
  
 
  
 6
 
 160147693
 
 160148041
 
  ENSEMBL   UCSC 
 
  349
 
  5
 
 35320
 
 tss;island
 
 0.004200
 
 0.0032676
 
 Not queried
 
  0.8574730
 
       NA
 
           NA
  
 
  
 1
 
   2334785
 
   2334974
 
  ENSEMBL   UCSC 
 
  190
 
  4
 
 82
 
 gene;island
 
 0.985811
 
 0.9732390
 
 Not queried
 
  0.8573783
 
       NA
 
           NA
  
 
  
 1
 
  27709575
 
  27709791
 
  ENSEMBL   UCSC 
 
  217
 
  6
 
 354
 
 gene
 
 0.350373
 
 0.2945209
 
 Not queried
 
  0.8573601
 
       NA
 
           NA
  
 
  
 8
 
 143596671
 
 143596947
 
  ENSEMBL   UCSC 
 
  277
 
  4
 
 39464
 
 gene
 
 0.493900
 
 0.6364332
 
 Not queried
 
  0.8570029
 
       NA
 
           NA
  
 
  
 11
 
  31391061
 
  31391649
 
  ENSEMBL   UCSC 
 
  589
 
 15
 
 7221
 
 tss;island
 
 0.001733
 
 0.0054770
 
 Not queried
 
  0.8569896
 
       NA
 
           NA
  
 
  
 7
 
 157216599
 
 157216893
 
  ENSEMBL   UCSC 
 
  295
 
  4
 
 39102
 
 island
 
 0.643470
 
 0.7881776
 
 Not queried
 
  0.8569586
 
       NA
 
           NA
  
 
  
 4
 
 175444096
 
 175444268
 
  ENSEMBL   UCSC 
 
  173
 
  4
 
 30685
 
 tss;island
 
 0.024985
 
 0.0288477
 
 Not queried
 
  0.8569374
 
       NA
 
           NA
  
 
  
 21
 
  30670919
 
  30671158
 
  ENSEMBL   UCSC 
 
  240
 
  7
 
 26503
 
 tss;island
 
 0.008577
 
 0.0051962
 
 Not queried
 
  0.8569278
 
       NA
 
           NA
  
 
  
 15
 
  40697419
 
  40698044
 
  ENSEMBL   UCSC 
 
  626
 
  7
 
 14037
 
 island
 
 0.018393
 
 0.0040233
 
 Not queried
 
  0.8569080
 
       NA
 
           NA
  
 
  
 1
 
   6614341
 
   6614587
 
  ENSEMBL   UCSC 
 
  247
 
  4
 
 178
 
 gene
 
 0.010467
 
 0.0064892
 
 Not queried
 
  0.8568074
 
       NA
 
           NA
  
 
  
 1
 
 203462261
 
 203462521
 
  ENSEMBL   UCSC 
 
  261
 
  4
 
 2193
 
 tss
 
 0.290023
 
 0.3793434
 
 Not queried
 
  0.8567801
 
       NA
 
           NA
  
 
  
 6
 
 150285031
 
 150285139
 
  ENSEMBL   UCSC 
 
  109
 
  4
 
 35292
 
 tss
 
 0.044154
 
 0.0323376
 
 Not queried
 
  0.8566832
 
       NA
 
           NA
  
 
  
 19
 
  52643011
 
  52643172
 
  ENSEMBL   UCSC 
 
  162
 
  4
 
 20191
 
 gene
 
 0.013625
 
 0.0071018
 
 Not queried
 
  0.8566751
 
       NA
 
           NA
  
 
  
 19
 
  52643011
 
  52643487
 
  ENSEMBL   UCSC 
 
  477
 
 10
 
 22350
 
 island
 
 0.014505
 
 0.0083428
 
 Not queried
 
  0.8566751
 
       NA
 
           NA
  
 
  
 20
 
  39657308
 
  39657451
 
  ENSEMBL   UCSC 
 
  144
 
  5
 
 25743
 
 tss
 
 0.008943
 
 0.0095796
 
 Not queried
 
  0.8566587
 
       NA
 
           NA
  
 
  
 11
 
  58345523
 
  58345717
 
  ENSEMBL   UCSC 
 
  195
 
  7
 
 7311
 
 tss;island
 
 0.003087
 
 0.0064719
 
 Not queried
 
  0.8562311
 
       NA
 
           NA
  
 
  
 17
 
  73874443
 
  73874637
 
  ENSEMBL   UCSC 
 
  195
 
  5
 
 16780
 
 gene
 
 0.099132
 
 0.0522686
 
 Not queried
 
  0.8558729
 
       NA
 
           NA
  
 
  
 11
 
    208399
 
    208661
 
  ENSEMBL   UCSC 
 
  263
 
  7
 
 7004
 
 tss;island
 
 0.012164
 
 0.0101472
 
 Not queried
 
  0.8558337
 
       NA
 
           NA
  
 
  
 18
 
  22006316
 
  22006514
 
  ENSEMBL   UCSC 
 
  199
 
  8
 
 19213
 
 tss
 
 0.145571
 
 0.0579438
 
 Not queried
 
  0.8558307
 
       NA
 
           NA
  
 
  
 6
 
  46702683
 
  46703422
 
  ENSEMBL   UCSC 
 
  740
 
 12
 
 34360
 
 gene
 
 0.007416
 
 0.0095278
 
 Not queried
 
  0.8557029
 
       NA
 
           NA
  
 
  
 1
 
   3319264
 
   3319509
 
  ENSEMBL   UCSC 
 
  246
 
  4
 
 123
 
 gene;island
 
 0.465635
 
 0.5452171
 
 Not queried
 
  0.8556606
 
       NA
 
           NA
  
 
  
 6
 
  32020377
 
  32020750
 
  ENSEMBL   UCSC 
 
  374
 
  9
 
 34018
 
 gene
 
 0.516693
 
 0.6713848
 
 Not queried
 
  0.8556076
 
       NA
 
           NA
  
 
  
 17
 
  43198124
 
  43198610
 
  ENSEMBL   UCSC 
 
  487
 
  4
 
 16627
 
 gene;island
 
 0.726327
 
 0.6858654
 
 Not queried
 
  0.8555103
 
       NA
 
           NA
  
 
  
 16
 
   1080388
 
   1080808
 
  ENSEMBL   UCSC 
 
  421
 
  4
 
 15509
 
 island
 
 0.634964
 
 0.7049756
 
 Not queried
 
  0.8554766
 
       NA
 
           NA
  
 
  
 12
 
  57595353
 
  57595605
 
  ENSEMBL   UCSC 
 
  253
 
  6
 
 9188
 
 gene;island
 
 0.490721
 
 0.5042232
 
 Not queried
 
  0.8554174
 
       NA
 
           NA
  
 
  
 2
 
   1732172
 
   1732454
 
  ENSEMBL   UCSC 
 
  283
 
  4
 
 22535
 
 gene
 
 0.512201
 
 0.6397792
 
 Not queried
 
  0.8551979
 
       NA
 
           NA
  
 
  
 12
 
 132990805
 
 132991277
 
  ENSEMBL   UCSC 
 
  473
 
  5
 
 11051
 
 island
 
 0.470894
 
 0.6866371
 
 Not queried
 
  0.8551255
 
       NA
 
           NA
  
 
  
 11
 
   1855286
 
   1855665
 
  ENSEMBL   UCSC 
 
  380
 
  7
 
 7047
 
 tss
 
 0.203559
 
 0.2304344
 
 Not queried
 
  0.8548456
 
       NA
 
           NA
  
 
  
 5
 
 112770203
 
 112770721
 
  ENSEMBL   UCSC 
 
  519
 
  6
 
 31716
 
 gene
 
 0.468733
 
 0.6504802
 
 Not queried
 
  0.8548351
 
       NA
 
           NA
  
 
  
 3
 
  10436197
 
  10436869
 
  ENSEMBL   UCSC 
 
  673
 
  9
 
 27662
 
 gene
 
 0.336990
 
 0.4873564
 
 Not queried
 
  0.8547854
 
       NA
 
           NA
  
 
  
 2
 
  96068451
 
  96068839
 
  ENSEMBL   UCSC 
 
  389
 
  5
 
 22777
 
 gene
 
 0.033885
 
 0.0221476
 
 Not queried
 
  0.8546605
 
       NA
 
           NA
  
 
  
 17
 
  55962522
 
  55962841
 
  ENSEMBL   UCSC 
 
  320
 
  4
 
 16705
 
 gene;island
 
 0.691006
 
 0.8401245
 
 Not queried
 
  0.8546426
 
       NA
 
           NA
  
 
  
 12
 
 116713947
 
 116714304
 
  ENSEMBL   UCSC 
 
  358
 
  6
 
 9328
 
 gene
 
 0.008816
 
 0.0072175
 
 Not queried
 
  0.8546096
 
       NA
 
           NA
  
 
  
 11
 
  67777618
 
  67777952
 
  ENSEMBL   UCSC 
 
  335
 
  8
 
 6766
 
 gene
 
 0.049335
 
 0.1191028
 
 Not queried
 
  0.8545332
 
       NA
 
           NA
  
 
  
 11
 
  67777618
 
  67777770
 
  ENSEMBL   UCSC 
 
  153
 
  6
 
 7503
 
 tss
 
 0.041424
 
 0.1065642
 
 Not queried
 
  0.8545332
 
       NA
 
           NA
  
 
  
 10
 
 134884177
 
 134884645
 
  ENSEMBL   UCSC 
 
  469
 
  7
 
 6330
 
 island
 
 0.306419
 
 0.5069123
 
 Not queried
 
  0.8545296
 
       NA
 
           NA
  
 
  
 6
 
  28105036
 
  28105417
 
  ENSEMBL   UCSC 
 
  382
 
  4
 
 35578
 
 island
 
 0.014704
 
 0.0143214
 
 Not queried
 
  0.8544365
 
       NA
 
           NA
  
 
  
 10
 
 135171559
 
 135171746
 
  ENSEMBL   UCSC 
 
  188
 
  7
 
 5463
 
 tss
 
 0.051276
 
 0.0175036
 
 Not queried
 
  0.8543801
 
       NA
 
           NA
  
 
  
 2
 
 242127690
 
 242127852
 
  ENSEMBL   UCSC 
 
  163
 
  4
 
 23986
 
 tss
 
 0.021801
 
 0.0394122
 
 Not queried
 
  0.8542834
 
       NA
 
           NA
  
 
  
 14
 
  37124018
 
  37124434
 
  ENSEMBL   UCSC 
 
  417
 
  5
 
 12790
 
 island
 
 0.280164
 
 0.2554480
 
 Not queried
 
  0.8542277
 
       NA
 
           NA
  
 
  
 11
 
  86511210
 
  86511545
 
  ENSEMBL   UCSC 
 
  336
 
  4
 
 8713
 
 island
 
 0.089316
 
 0.0552700
 
 Not queried
 
  0.8539017
 
       NA
 
           NA
  
 
  
 17
 
  79913303
 
  79913431
 
  ENSEMBL   UCSC 
 
  129
 
  4
 
 16932
 
 gene;island
 
 0.855824
 
 0.8795721
 
 Not queried
 
  0.8536032
 
       NA
 
           NA
  
 
  
 20
 
  43935222
 
  43935471
 
  ENSEMBL   UCSC 
 
  250
 
  8
 
 25770
 
 tss
 
 0.106557
 
 0.2529223
 
 Not queried
 
  0.8535974
 
       NA
 
           NA
  
 
  
 6
 
  12012544
 
  12012580
 
  ENSEMBL   UCSC 
 
   37
 
  6
 
 34639
 
 tss;island
 
 0.019554
 
 0.0104163
 
 Not queried
 
  0.8535078
 
       NA
 
           NA
  
 
  
 15
 
  75660973
 
  75661354
 
  ENSEMBL   UCSC 
 
  382
 
  9
 
 13811
 
 tss
 
 0.010906
 
 0.0052712
 
 Not queried
 
  0.8532667
 
       NA
 
           NA
  
 
  
 3
 
  10547373
 
  10547584
 
  ENSEMBL   UCSC 
 
  212
 
  4
 
 28151
 
 tss
 
 0.012467
 
 0.0231693
 
 Not queried
 
  0.8532462
 
       NA
 
           NA
  
 
  
 14
 
  26674046
 
  26674563
 
  ENSEMBL   UCSC 
 
  518
 
  7
 
 12753
 
 island
 
 0.030552
 
 0.0268111
 
 Not queried
 
  0.8530974
 
       NA
 
           NA
  
 
  
 1
 
  35259746
 
  35260256
 
  ENSEMBL   UCSC 
 
  511
 
  5
 
 414
 
 gene;island
 
 0.439537
 
 0.5942413
 
 Not queried
 
  0.8530728
 
       NA
 
           NA
  
 
  
 11
 
  95522834
 
  95523144
 
  ENSEMBL   UCSC 
 
  311
 
  7
 
 7636
 
 tss;island
 
 0.004417
 
 0.0017461
 
 Not queried
 
  0.8527331
 
       NA
 
           NA
  
 
  
 1
 
  55464530
 
  55464927
 
  ENSEMBL   UCSC 
 
  398
 
  5
 
 3305
 
 island
 
 0.241617
 
 0.3652134
 
 Not queried
 
  0.8526720
 
       NA
 
           NA
  
 
  
 20
 
  61924240
 
  61924423
 
  ENSEMBL   UCSC 
 
  184
 
  4
 
 25895
 
 tss;island
 
 0.019386
 
 0.0242019
 
 Not queried
 
  0.8525169
 
       NA
 
           NA
  
 
  
 8
 
   1771973
 
   1772393
 
  ENSEMBL   UCSC 
 
  421
 
  6
 
 40039
 
 island
 
 0.078760
 
 0.0301839
 
 Not queried
 
  0.8525163
 
       NA
 
           NA
  
 
  
 18
 
  12407563
 
  12407812
 
  ENSEMBL   UCSC 
 
  250
 
  6
 
 19188
 
 tss;island
 
 0.014343
 
 0.0074030
 
 Not queried
 
  0.8523972
 
       NA
 
           NA
  
 
  
 3
 
 142607939
 
 142608228
 
  ENSEMBL   UCSC 
 
  290
 
  8
 
 28643
 
 tss
 
 0.023013
 
 0.0136613
 
 Not queried
 
  0.8523440
 
       NA
 
           NA
  
 
  
 3
 
 142607715
 
 142608228
 
  ENSEMBL   UCSC 
 
  514
 
 10
 
 29554
 
 island
 
 0.023013
 
 0.0113543
 
 Not queried
 
  0.8523440
 
       NA
 
           NA
  
 
  
 2
 
 173940203
 
 173940561
 
  ENSEMBL   UCSC 
 
  359
 
  6
 
 23721
 
 tss
 
 0.042189
 
 0.0390606
 
 Not queried
 
  0.8523279
 
       NA
 
           NA
  
 
  
 1
 
 220863263
 
 220863572
 
  ENSEMBL   UCSC 
 
  310
 
  7
 
 2261
 
 tss
 
 0.061016
 
 0.0444887
 
 Not queried
 
  0.8521190
 
       NA
 
           NA
  
 
  
 1
 
 220863263
 
 220863796
 
  ENSEMBL   UCSC 
 
  534
 
  9
 
 4217
 
 island
 
 0.061016
 
 0.0444887
 
 Not queried
 
  0.8521190
 
       NA
 
           NA
  
 
  
 15
 
  37391424
 
  37391721
 
  ENSEMBL   UCSC 
 
  298
 
  4
 
 13284
 
 gene;tss;island
 
 0.010831
 
 0.0197106
 
 Not queried
 
  0.8519748
 
       NA
 
           NA
  
 
  
 8
 
 101224915
 
 101225361
 
  ENSEMBL   UCSC 
 
  447
 
  6
 
 39378
 
 gene;island
 
 0.006199
 
 0.0061297
 
 Not queried
 
  0.8519061
 
       NA
 
           NA
  
 
  
 11
 
 118272104
 
 118272451
 
  ENSEMBL   UCSC 
 
  348
 
  4
 
 6929
 
 gene
 
 0.004816
 
 0.0039164
 
 Not queried
 
  0.8518833
 
       NA
 
           NA
  
 
  
 20
 
  62492074
 
  62492380
 
  ENSEMBL   UCSC 
 
  307
 
  4
 
 25903
 
 tss;island
 
 0.518575
 
 0.8753691
 
 Not queried
 
  0.8516392
 
       NA
 
           NA
  
 
  
 15
 
  65068326
 
  65068594
 
  ENSEMBL   UCSC 
 
  269
 
  4
 
 13732
 
 tss;island
 
 0.107653
 
 0.0381360
 
 Not queried
 
  0.8516301
 
       NA
 
           NA
  
 
  
 12
 
  52300911
 
  52301307
 
  ENSEMBL   UCSC 
 
  397
 
  5
 
 10432
 
 island
 
 0.092781
 
 0.1546742
 
 Not queried
 
  0.8513915
 
       NA
 
           NA
  
 
  
 14
 
  24610802
 
  24611268
 
  ENSEMBL   UCSC 
 
  467
 
  9
 
 12314
 
 tss
 
 0.005885
 
 0.0130838
 
 Not queried
 
  0.8512359
 
       NA
 
           NA
  
 
  
 17
 
  18128578
 
  18128806
 
  ENSEMBL   UCSC 
 
  229
 
  5
 
 17174
 
 tss;island
 
 0.007947
 
 0.0062832
 
 Not queried
 
  0.8509436
 
       NA
 
           NA
  
 
  
 11
 
  61196912
 
  61197075
 
  ENSEMBL   UCSC 
 
  164
 
  4
 
 6628
 
 gene;tss;island
 
 0.004027
 
 0.0041697
 
 Not queried
 
  0.8508738
 
       NA
 
           NA
  
 
  
 6
 
  30568959
 
  30569211
 
  ENSEMBL   UCSC 
 
  253
 
  5
 
 33782
 
 gene
 
 0.964949
 
 0.8908057
 
 Not queried
 
  0.8507894
 
       NA
 
           NA
  
 
  
 16
 
  27280098
 
  27280434
 
  ENSEMBL   UCSC 
 
  337
 
  9
 
 15782
 
 island
 
 0.002706
 
 0.0036482
 
 Not queried
 
  0.8507604
 
       NA
 
           NA
  
 
  
 18
 
   3411743
 
   3411996
 
  ENSEMBL   UCSC 
 
  254
 
  4
 
 19162
 
 tss
 
 0.341177
 
 0.5183783
 
 Not queried
 
  0.8505870
 
       NA
 
           NA
  
 
  
 5
 
   2866767
 
   2866877
 
  ENSEMBL   UCSC 
 
  111
 
  4
 
 32661
 
 island
 
 0.149919
 
 0.2254677
 
 Not queried
 
  0.8504314
 
       NA
 
           NA
  
 
  
 19
 
   1467921
 
   1467979
 
  ENSEMBL   UCSC 
 
   59
 
  4
 
 19656
 
 gene;island
 
 0.039249
 
 0.0265399
 
 Not queried
 
  0.8502511
 
       NA
 
           NA
  
 
  
 1
 
  94703079
 
  94703176
 
  ENSEMBL   UCSC 
 
   98
 
  5
 
 641
 
 gene
 
 0.005214
 
 0.0017881
 
 Not queried
 
  0.8502492
 
       NA
 
           NA
  
 
  
 8
 
   1955069
 
   1955323
 
  ENSEMBL   UCSC 
 
  255
 
  4
 
 40045
 
 island
 
 0.522716
 
 0.6164598
 
 Not queried
 
  0.8501730
 
       NA
 
           NA
  
 
  
 1
 
  68962124
 
  68962318
 
  ENSEMBL   UCSC 
 
  195
 
  4
 
 576
 
 gene;island
 
 0.004884
 
 0.0044661
 
 Not queried
 
  0.8500854
 
       NA
 
           NA
  
 
  
 8
 
  11561283
 
  11561728
 
  ENSEMBL   UCSC 
 
  446
 
 11
 
 40087
 
 island
 
 0.110159
 
 0.0298513
 
 Not queried
 
  0.8500791
 
       NA
 
           NA
  
 
  
 16
 
   2848117
 
   2848577
 
  ENSEMBL   UCSC 
 
  461
 
  6
 
 15619
 
 island
 
 0.444411
 
 0.6200247
 
 Not queried
 
  0.8500082
 
       NA
 
           NA
  
 
  
 6
 
  39290342
 
  39290877
 
  ENSEMBL   UCSC 
 
  536
 
  7
 
 34956
 
 tss
 
 0.025521
 
 0.3703254
 
 Not queried
 
  0.8499067
 
       NA
 
           NA
  
 
  
 3
 
 145878963
 
 145879710
 
  ENSEMBL   UCSC 
 
  748
 
 10
 
 29562
 
 island
 
 0.045539
 
 0.0347434
 
 Not queried
 
  0.8497612
 
       NA
 
           NA
  
 
  
 14
 
  20881581
 
  20881764
 
  ENSEMBL   UCSC 
 
  184
 
  5
 
 12269
 
 tss
 
 0.003086
 
 0.0051376
 
 Not queried
 
  0.8497533
 
       NA
 
           NA
  
 
  
 4
 
 111397332
 
 111398146
 
  ENSEMBL   UCSC 
 
  815
 
  8
 
 30093
 
 gene
 
 0.489790
 
 0.7311919
 
 Not queried
 
  0.8497517
 
       NA
 
           NA
  
 
  
 3
 
  46448753
 
  46449100
 
  ENSEMBL   UCSC 
 
  348
 
  5
 
 27748
 
 gene
 
 0.014106
 
 0.0075820
 
 Not queried
 
  0.8496393
 
       NA
 
           NA
  
 
  
 1
 
  16679092
 
  16679500
 
  ENSEMBL   UCSC 
 
  409
 
  8
 
 1282
 
 tss;island
 
 0.010217
 
 0.0088531
 
 Not queried
 
  0.8488554
 
       NA
 
           NA
  
 
  
 6
 
 168449117
 
 168449422
 
  ENSEMBL   UCSC 
 
  306
 
  4
 
 36656
 
 island
 
 0.565275
 
 0.6633084
 
 Not queried
 
  0.8488132
 
       NA
 
           NA
  
 
  
 20
 
    305874
 
    306049
 
  ENSEMBL   UCSC 
 
  176
 
  5
 
 25524
 
 tss;island
 
 0.004979
 
 0.0081204
 
 Not queried
 
  0.8487643
 
       NA
 
           NA
  
 
  
 3
 
  55523815
 
  55524048
 
  ENSEMBL   UCSC 
 
  234
 
  4
 
 29217
 
 island
 
 0.042618
 
 0.0436434
 
 Not queried
 
  0.8486952
 
       NA
 
           NA
  
 
  
 18
 
  44526430
 
  44526756
 
  ENSEMBL   UCSC 
 
  327
 
  6
 
 19260
 
 tss
 
 0.003621
 
 0.0090084
 
 Not queried
 
  0.8485692
 
       NA
 
           NA
  
 
  
 17
 
  54230419
 
  54230793
 
  ENSEMBL   UCSC 
 
  375
 
  5
 
 17520
 
 tss
 
 0.385473
 
 0.3647029
 
 Not queried
 
  0.8484981
 
       NA
 
           NA
  
 
  
 8
 
  37963284
 
  37963526
 
  ENSEMBL   UCSC 
 
  243
 
  6
 
 39281
 
 gene;island
 
 0.024825
 
 0.0163042
 
 Not queried
 
  0.8484936
 
       NA
 
           NA
  
 
  
 2
 
 239112214
 
 239112731
 
  ENSEMBL   UCSC 
 
  518
 
  8
 
 25240
 
 island
 
 0.011451
 
 0.0107138
 
 Not queried
 
  0.8483567
 
       NA
 
           NA
  
 
  
 19
 
  15580310
 
  15580445
 
  ENSEMBL   UCSC 
 
  136
 
  4
 
 19908
 
 gene;island
 
 0.018411
 
 0.0414153
 
 Not queried
 
  0.8481225
 
       NA
 
           NA
  
 
  
 20
 
  62680970
 
  62681428
 
  ENSEMBL   UCSC 
 
  459
 
 11
 
 26380
 
 island
 
 0.011560
 
 0.0078407
 
 Not queried
 
  0.8480478
 
       NA
 
           NA
  
 
  
 20
 
  31755609
 
  31755943
 
  ENSEMBL   UCSC 
 
  335
 
  4
 
 25679
 
 tss
 
 0.485096
 
 0.6689016
 
 Not queried
 
  0.8480388
 
       NA
 
           NA
  
 
  
 8
 
 144942912
 
 144943163
 
  ENSEMBL   UCSC 
 
  252
 
  4
 
 39494
 
 gene;island
 
 0.737004
 
 0.8114810
 
 Not queried
 
  0.8478065
 
       NA
 
           NA
  
 
  
 2
 
 130955979
 
 130956271
 
  ENSEMBL   UCSC 
 
  293
 
  8
 
 24716
 
 island
 
 0.924716
 
 0.9654046
 
 Not queried
 
  0.8477270
 
       NA
 
           NA
  
 
  
 2
 
 232825958
 
 232826398
 
  ENSEMBL   UCSC 
 
  441
 
  8
 
 25192
 
 island
 
 0.014378
 
 0.0096215
 
 Not queried
 
  0.8476933
 
       NA
 
           NA
  
 
  
 20
 
   1093493
 
   1093802
 
  ENSEMBL   UCSC 
 
  310
 
  6
 
 25532
 
 tss
 
 0.662198
 
 0.8299745
 
 Not queried
 
  0.8476921
 
       NA
 
           NA
  
 
  
 7
 
    856023
 
    856295
 
  ENSEMBL   UCSC 
 
  273
 
  4
 
 38052
 
 island
 
 0.013569
 
 0.0069407
 
 Not queried
 
  0.8474562
 
       NA
 
           NA
  
 
  
 17
 
  73851514
 
  73851984
 
  ENSEMBL   UCSC 
 
  471
 
  7
 
 17664
 
 tss
 
 0.007383
 
 0.0041729
 
 Not queried
 
  0.8471898
 
       NA
 
           NA
  
 
  
 17
 
  73851399
 
  73851984
 
  ENSEMBL   UCSC 
 
  586
 
  9
 
 18764
 
 island
 
 0.007383
 
 0.0041729
 
 Not queried
 
  0.8471898
 
       NA
 
           NA
  
 
  
 7
 
  43288635
 
  43288889
 
  ENSEMBL   UCSC 
 
  255
 
  4
 
 36981
 
 gene;island
 
 0.517921
 
 0.7451259
 
 Not queried
 
  0.8470821
 
       NA
 
           NA
  
 
  
 11
 
  66627194
 
  66627706
 
  ENSEMBL   UCSC 
 
  513
 
  5
 
 6739
 
 gene;island
 
 0.872145
 
 0.9471286
 
 Not queried
 
  0.8470378
 
       NA
 
           NA
  
 
  
 6
 
  74072242
 
  74072447
 
  ENSEMBL   UCSC 
 
  206
 
  7
 
 36251
 
 island
 
 0.865196
 
 0.8463835
 
 Not queried
 
  0.8466767
 
       NA
 
           NA
  
 
  
 17
 
  38521067
 
  38521482
 
  ENSEMBL   UCSC 
 
  416
 
  4
 
 17332
 
 tss;island
 
 0.968229
 
 0.9316239
 
 Not queried
 
  0.8464840
 
       NA
 
           NA
  
 
  
 14
 
  24836084
 
  24836219
 
  ENSEMBL   UCSC 
 
  136
 
  5
 
 12330
 
 tss;island
 
 0.003654
 
 0.0047658
 
 Not queried
 
  0.8463859
 
       NA
 
           NA
  
 
  
 8
 
  30890108
 
  30890632
 
  ENSEMBL   UCSC 
 
  525
 
 11
 
 39271
 
 gene;tss
 
 0.010062
 
 0.0117447
 
 Not queried
 
  0.8462716
 
       NA
 
           NA
  
 
  
 19
 
  35630106
 
  35630474
 
  ENSEMBL   UCSC 
 
  369
 
  7
 
 19980
 
 gene;island
 
 0.456753
 
 0.5435990
 
 Not queried
 
  0.8462413
 
       NA
 
           NA
  
 
  
 10
 
  64133674
 
  64133896
 
  ENSEMBL   UCSC 
 
  223
 
  8
 
 5101
 
 tss;island
 
 0.020918
 
 0.0155448
 
 Not queried
 
  0.8460839
 
       NA
 
           NA
  
 
  
 5
 
  31532447
 
  31532653
 
  ENSEMBL   UCSC 
 
  207
 
  4
 
 31576
 
 gene
 
 0.003357
 
 0.0040901
 
 Not queried
 
  0.8460002
 
       NA
 
           NA
  
 
  
 7
 
   2946264
 
   2946518
 
  ENSEMBL   UCSC 
 
  255
 
  4
 
 36819
 
 gene;island
 
 0.975147
 
 0.9489279
 
 Not queried
 
  0.8459335
 
       NA
 
           NA
  
 
  
 10
 
  51572113
 
  51572323
 
  ENSEMBL   UCSC 
 
  211
 
  6
 
 5079
 
 tss
 
 0.020125
 
 0.0073040
 
 Not queried
 
  0.8457652
 
       NA
 
           NA
  
 
  
 3
 
  43147320
 
  43148002
 
  ENSEMBL   UCSC 
 
  683
 
 11
 
 29027
 
 island
 
 0.056241
 
 0.0276299
 
 Not queried
 
  0.8457460
 
       NA
 
           NA
  
 
  
 20
 
  62716332
 
  62716606
 
  ENSEMBL   UCSC 
 
  275
 
  4
 
 25520
 
 gene;tss;island
 
 0.415290
 
 0.3814251
 
 Not queried
 
  0.8456310
 
       NA
 
           NA
  
 
  
 17
 
  78172177
 
  78172613
 
  ENSEMBL   UCSC 
 
  437
 
  5
 
 16860
 
 gene;island
 
 0.792472
 
 0.8927160
 
 Not queried
 
  0.8455582
 
       NA
 
           NA
  
 
  
 6
 
  48036212
 
  48036676
 
  ENSEMBL   UCSC 
 
  465
 
 10
 
 36192
 
 island
 
 0.165552
 
 0.3143714
 
 Not queried
 
  0.8455139
 
       NA
 
           NA
  
 
  
 2
 
  33359198
 
  33359550
 
  ENSEMBL   UCSC 
 
  353
 
  5
 
 23298
 
 tss
 
 0.563254
 
 0.5014673
 
 Not queried
 
  0.8454800
 
       NA
 
           NA
  
 
  
 4
 
  41361623
 
  41362397
 
  ENSEMBL   UCSC 
 
  775
 
  9
 
 30363
 
 tss;island
 
 0.486902
 
 0.5014565
 
 Not queried
 
  0.8453096
 
       NA
 
           NA
  
 
  
 7
 
  42276814
 
  42277071
 
  ENSEMBL   UCSC 
 
  258
 
 11
 
 37550
 
 tss;island
 
 0.010225
 
 0.0379736
 
 Not queried
 
  0.8452256
 
       NA
 
           NA
  
 
  
 11
 
 128737300
 
 128737467
 
  ENSEMBL   UCSC 
 
  168
 
  5
 
 7805
 
 tss
 
 0.900447
 
 0.9633736
 
 Not queried
 
  0.8451755
 
       NA
 
           NA
  
 
  
 5
 
 140624869
 
 140625389
 
  ENSEMBL   UCSC 
 
  521
 
 10
 
 33228
 
 island
 
 0.020502
 
 0.0631581
 
 Not queried
 
  0.8451591
 
       NA
 
           NA
  
 
  
 5
 
 146258159
 
 146258785
 
  ENSEMBL   UCSC 
 
  627
 
 14
 
 32408
 
 tss
 
 0.007477
 
 0.0117799
 
 Not queried
 
  0.8451458
 
       NA
 
           NA
  
 
  
 1
 
 205649791
 
 205649956
 
  ENSEMBL   UCSC 
 
  166
 
  8
 
 2207
 
 tss;island
 
 0.010420
 
 0.0089616
 
 Not queried
 
  0.8450423
 
       NA
 
           NA
  
 
  
 11
 
 102188233
 
 102188539
 
  ENSEMBL   UCSC 
 
  307
 
  4
 
 6877
 
 gene
 
 0.001864
 
 0.0045823
 
 Not queried
 
  0.8446857
 
       NA
 
           NA
  
 
  
 11
 
  94276895
 
  94277190
 
  ENSEMBL   UCSC 
 
  296
 
  6
 
 8740
 
 island
 
 0.023715
 
 0.0123626
 
 Not queried
 
  0.8446777
 
       NA
 
           NA
  
 
  
 4
 
 109541393
 
 109541595
 
  ENSEMBL   UCSC 
 
  203
 
  8
 
 30087
 
 gene
 
 0.005622
 
 0.0049359
 
 Not queried
 
  0.8445129
 
       NA
 
           NA
  
 
  
 10
 
 135049991
 
 135050355
 
  ENSEMBL   UCSC 
 
  365
 
 13
 
 5455
 
 tss;island
 
 0.026184
 
 0.0397887
 
 Not queried
 
  0.8444051
 
       NA
 
           NA
  
 
  
 3
 
 142839837
 
 142840240
 
  ENSEMBL   UCSC 
 
  404
 
  5
 
 27980
 
 gene;island
 
 0.053216
 
 0.0524260
 
 Not queried
 
  0.8441870
 
       NA
 
           NA
  
 
  
 11
 
   2013417
 
   2013668
 
  ENSEMBL   UCSC 
 
  252
 
  4
 
 7943
 
 island
 
 0.367916
 
 0.6382638
 
 Not queried
 
  0.8440777
 
       NA
 
           NA
  
 
  
 13
 
  22245333
 
  22245730
 
  ENSEMBL   UCSC 
 
  398
 
  5
 
 11100
 
 gene
 
 0.012312
 
 0.0078586
 
 Not queried
 
  0.8439765
 
       NA
 
           NA
  
 
  
 11
 
 132813235
 
 132813563
 
  ENSEMBL   UCSC 
 
  329
 
  4
 
 6981
 
 gene;tss;island
 
 0.010597
 
 0.0224309
 
 Not queried
 
  0.8438143
 
       NA
 
           NA
  
 
  
 6
 
  33136859
 
  33137336
 
  ENSEMBL   UCSC 
 
  478
 
  7
 
 34199
 
 gene
 
 0.816826
 
 0.7390761
 
 Not queried
 
  0.8437459
 
       NA
 
           NA
  
 
  
 3
 
 133464463
 
 133464971
 
  ENSEMBL   UCSC 
 
  509
 
  6
 
 28602
 
 tss;island
 
 0.260000
 
 0.2338678
 
 Not queried
 
  0.8437269
 
       NA
 
           NA
  
 
  
 22
 
  51016501
 
  51017166
 
  ENSEMBL   UCSC 
 
  666
 
 14
 
 26932
 
 gene;island
 
 0.532361
 
 0.6494467
 
 Not queried
 
  0.8437240
 
       NA
 
           NA
  
 
  
 17
 
  16341984
 
  16342235
 
  ENSEMBL   UCSC 
 
  252
 
  8
 
 17158
 
 tss;island
 
 0.007230
 
 0.0043014
 
 Not queried
 
  0.8436323
 
       NA
 
           NA
  
 
  
 12
 
  68041257
 
  68041428
 
  ENSEMBL   UCSC 
 
  172
 
  4
 
 9852
 
 tss;island
 
 0.926196
 
 0.9571452
 
 Not queried
 
  0.8435516
 
       NA
 
           NA
  
 
  
 10
 
  65733092
 
  65733575
 
  ENSEMBL   UCSC 
 
  484
 
  4
 
 5780
 
 island
 
 0.639728
 
 0.7246531
 
 Not queried
 
  0.8435266
 
       NA
 
           NA
  
 
  
 4
 
 169239550
 
 169239900
 
  ENSEMBL   UCSC 
 
  351
 
  6
 
 30174
 
 gene
 
 0.019819
 
 0.0104923
 
 Not queried
 
  0.8434827
 
       NA
 
           NA
  
 
  
 4
 
 169239550
 
 169240081
 
  ENSEMBL   UCSC 
 
  532
 
 10
 
 31412
 
 island
 
 0.013380
 
 0.0069399
 
 Not queried
 
  0.8434827
 
       NA
 
           NA
  
 
  
 8
 
   1132050
 
   1132189
 
  ENSEMBL   UCSC 
 
  140
 
  4
 
 40016
 
 island
 
 0.290560
 
 0.4321559
 
 Not queried
 
  0.8433709
 
       NA
 
           NA
  
 
  
 16
 
   1967781
 
   1968232
 
  ENSEMBL   UCSC 
 
  452
 
  4
 
 15569
 
 island
 
 0.017822
 
 0.0434435
 
 Not queried
 
  0.8433425
 
       NA
 
           NA
  
 
  
 10
 
 135278717
 
 135279147
 
  ENSEMBL   UCSC 
 
  431
 
  5
 
 4906
 
 gene;island
 
 0.468700
 
 0.6974667
 
 Not queried
 
  0.8432991
 
       NA
 
           NA
  
 
  
 1
 
  32208487
 
  32208703
 
  ENSEMBL   UCSC 
 
  217
 
  4
 
 383
 
 gene;island
 
 0.783715
 
 0.9041189
 
 Not queried
 
  0.8431796
 
       NA
 
           NA
  
 
  
 2
 
 131675994
 
 131676293
 
  ENSEMBL   UCSC 
 
  300
 
  4
 
 22869
 
 gene;island
 
 0.625604
 
 0.7005710
 
 Not queried
 
  0.8430601
 
       NA
 
           NA
  
 
  
 12
 
   6933322
 
   6933689
 
  ENSEMBL   UCSC 
 
  368
 
  4
 
 9034
 
 gene;island
 
 0.301114
 
 0.3716231
 
 Not queried
 
  0.8429674
 
       NA
 
           NA
  
 
  
 8
 
  93107604
 
  93107744
 
  ENSEMBL   UCSC 
 
  141
 
  5
 
 39809
 
 tss
 
 0.022408
 
 0.0427567
 
 Not queried
 
  0.8428675
 
       NA
 
           NA
  
 
  
 16
 
  50875140
 
  50875496
 
  ENSEMBL   UCSC 
 
  357
 
  4
 
 15915
 
 island
 
 0.027602
 
 0.0382028
 
 Not queried
 
  0.8428170
 
       NA
 
           NA
  
 
  
 1
 
  19229049
 
  19229145
 
  ENSEMBL   UCSC 
 
   97
 
  7
 
 278
 
 gene
 
 0.009094
 
 0.0056964
 
 Not queried
 
  0.8425922
 
       NA
 
           NA
  
 
  
 10
 
  64577216
 
  64577607
 
  ENSEMBL   UCSC 
 
  392
 
  4
 
 4603
 
 gene;tss;island
 
 0.033054
 
 0.0133321
 
 Not queried
 
  0.8425768
 
       NA
 
           NA
  
 
  
 14
 
  37116121
 
  37116408
 
  ENSEMBL   UCSC 
 
  288
 
  4
 
 12788
 
 island
 
 0.060930
 
 0.0813099
 
 Not queried
 
  0.8425626
 
       NA
 
           NA
  
 
  
 5
 
 148033708
 
 148034319
 
  ENSEMBL   UCSC 
 
  612
 
  7
 
 33291
 
 island
 
 0.117061
 
 0.0478460
 
 Not queried
 
  0.8425496
 
       NA
 
           NA
  
 
  
 8
 
 124553450
 
 124553689
 
  ENSEMBL   UCSC 
 
  240
 
  7
 
 39901
 
 tss;island
 
 0.014690
 
 0.0095708
 
 Not queried
 
  0.8421888
 
       NA
 
           NA
  
 
  
 3
 
  32023056
 
  32023475
 
  ENSEMBL   UCSC 
 
  420
 
 10
 
 28203
 
 tss;island
 
 0.027674
 
 0.0270661
 
 Not queried
 
  0.8421473
 
       NA
 
           NA
  
 
  
 14
 
  68086581
 
  68086880
 
  ENSEMBL   UCSC 
 
  300
 
  4
 
 12119
 
 gene
 
 0.013453
 
 0.0070910
 
 Not queried
 
  0.8418530
 
       NA
 
           NA
  
 
  
 20
 
   6103629
 
   6104274
 
  ENSEMBL   UCSC 
 
  646
 
  7
 
 25992
 
 island
 
 0.490079
 
 0.4879242
 
 Not queried
 
  0.8418420
 
       NA
 
           NA
  
 
  
 3
 
  50341009
 
  50341293
 
  ENSEMBL   UCSC 
 
  285
 
  4
 
 27801
 
 gene;tss
 
 0.718978
 
 0.8689817
 
 Not queried
 
  0.8416924
 
       NA
 
           NA
  
 
  
 10
 
 102756795
 
 102757026
 
  ENSEMBL   UCSC 
 
  232
 
  8
 
 6042
 
 island
 
 0.011815
 
 0.0066665
 
 Not queried
 
  0.8414669
 
       NA
 
           NA
  
 
  
 6
 
  30712331
 
  30713442
 
  ENSEMBL   UCSC 
 
 1112
 
 20
 
 34789
 
 tss
 
 0.146508
 
 0.3203142
 
 Not queried
 
  0.8414513
 
       NA
 
           NA
  
 
  
 15
 
  91642909
 
  91643135
 
  ENSEMBL   UCSC 
 
  227
 
  9
 
 13910
 
 tss;island
 
 0.007440
 
 0.0109152
 
 Not queried
 
  0.8412433
 
       NA
 
           NA
  
 
  
 1
 
  64669336
 
  64669472
 
  ENSEMBL   UCSC 
 
  137
 
  6
 
 1672
 
 tss
 
 0.915332
 
 0.9126239
 
 Not queried
 
  0.8412124
 
       NA
 
           NA
  
 
  
 3
 
 158288680
 
 158288944
 
  ENSEMBL   UCSC 
 
  265
 
  9
 
 28684
 
 tss
 
 0.012981
 
 0.0195717
 
 Not queried
 
  0.8410432
 
       NA
 
           NA
  
 
  
 11
 
 111783267
 
 111783563
 
  ENSEMBL   UCSC 
 
  297
 
  7
 
 7693
 
 tss
 
 0.157159
 
 0.3663603
 
 Not queried
 
  0.8410221
 
       NA
 
           NA
  
 
  
 4
 
 184930883
 
 184931143
 
  ENSEMBL   UCSC 
 
  261
 
  8
 
 30199
 
 gene
 
 0.539054
 
 0.8381915
 
 Not queried
 
  0.8408414
 
       NA
 
           NA
  
 
  
 1
 
 212587676
 
 212588117
 
  ENSEMBL   UCSC 
 
  442
 
  5
 
 998
 
 gene;island
 
 0.019150
 
 0.0145194
 
 Not queried
 
  0.8408296
 
       NA
 
           NA
  
 
  
 17
 
  19247837
 
  19248009
 
  ENSEMBL   UCSC 
 
  173
 
  8
 
 16479
 
 gene;island
 
 0.889625
 
 0.9621948
 
 Not queried
 
  0.8406880
 
       NA
 
           NA
  
 
  
 7
 
   1607146
 
   1607868
 
  ENSEMBL   UCSC 
 
  723
 
  7
 
 36762
 
 gene;island
 
 0.969222
 
 0.9704425
 
 Not queried
 
  0.8404902
 
       NA
 
           NA
  
 
  
 18
 
  29598296
 
  29598437
 
  ENSEMBL   UCSC 
 
  142
 
  4
 
 19222
 
 tss;island
 
 0.005406
 
 0.0065497
 
 Not queried
 
  0.8403194
 
       NA
 
           NA
  
 
  
 5
 
  75013131
 
  75013238
 
  ENSEMBL   UCSC 
 
  108
 
  4
 
 31649
 
 gene
 
 0.011643
 
 0.0169286
 
 Not queried
 
  0.8402781
 
       NA
 
           NA
  
 
  
 14
 
  78226708
 
  78227346
 
  ENSEMBL   UCSC 
 
  639
 
  6
 
 12157
 
 gene;island
 
 0.001882
 
 0.0029766
 
 Not queried
 
  0.8401598
 
       NA
 
           NA
  
 
  
 20
 
  37230326
 
  37230484
 
  ENSEMBL   UCSC 
 
  159
 
  5
 
 25737
 
 tss
 
 0.059512
 
 0.0650061
 
 Not queried
 
  0.8401000
 
       NA
 
           NA
  
 
  
 12
 
  69863677
 
  69864077
 
  ENSEMBL   UCSC 
 
  401
 
  7
 
 9861
 
 tss
 
 0.003638
 
 0.0085604
 
 Not queried
 
  0.8400829
 
       NA
 
           NA
  
 
  
 12
 
  69863677
 
  69864352
 
  ENSEMBL   UCSC 
 
  676
 
  9
 
 10615
 
 island
 
 0.006572
 
 0.0085604
 
 Not queried
 
  0.8400829
 
       NA
 
           NA
  
 
  
 7
 
  99716977
 
  99717359
 
  ENSEMBL   UCSC 
 
  383
 
  8
 
 37772
 
 tss
 
 0.006135
 
 0.0057472
 
 Not queried
 
  0.8398870
 
       NA
 
           NA
  
 
  
 10
 
 118031864
 
 118032081
 
  ENSEMBL   UCSC 
 
  218
 
  5
 
 4773
 
 gene;tss;island
 
 0.005158
 
 0.0058638
 
 Not queried
 
  0.8398754
 
       NA
 
           NA
  
 
  
 7
 
  32802477
 
  32802765
 
  ENSEMBL   UCSC 
 
  289
 
  4
 
 38396
 
 island
 
 0.021404
 
 0.1244636
 
 Not queried
 
  0.8397778
 
       NA
 
           NA
  
 
  
 19
 
   7964481
 
   7964703
 
  ENSEMBL   UCSC 
 
  223
 
  4
 
 19788
 
 gene;island
 
 0.861314
 
 0.9086638
 
 Not queried
 
  0.8397133
 
       NA
 
           NA
  
 
  
 3
 
  16924563
 
  16925048
 
  ENSEMBL   UCSC 
 
  486
 
  4
 
 28926
 
 island
 
 0.968371
 
 0.9808501
 
 Not queried
 
  0.8395463
 
       NA
 
           NA
  
 
  
 17
 
  45266785
 
  45267114
 
  ENSEMBL   UCSC 
 
  330
 
  8
 
 17439
 
 tss
 
 0.008053
 
 0.0055014
 
 Not queried
 
  0.8395376
 
       NA
 
           NA
  
 
  
 11
 
  10562070
 
  10562473
 
  ENSEMBL   UCSC 
 
  404
 
  4
 
 6507
 
 gene;island
 
 0.007987
 
 0.0046166
 
 Not queried
 
  0.8393429
 
       NA
 
           NA
  
 
  
 5
 
  72596646
 
  72597316
 
  ENSEMBL   UCSC 
 
  671
 
  9
 
 32870
 
 island
 
 0.027491
 
 0.1292084
 
 Not queried
 
  0.8393314
 
       NA
 
           NA
  
 
  
 7
 
   1062652
 
   1062965
 
  ENSEMBL   UCSC 
 
  314
 
  9
 
 36736
 
 gene;island
 
 0.126058
 
 0.2640272
 
 Not queried
 
  0.8389911
 
       NA
 
           NA
  
 
  
 1
 
   3007701
 
   3008033
 
  ENSEMBL   UCSC 
 
  333
 
  4
 
 98
 
 gene
 
 0.681652
 
 0.8030497
 
 Not queried
 
  0.8388633
 
       NA
 
           NA
  
 
  
 16
 
   4525918
 
   4526354
 
  ENSEMBL   UCSC 
 
  437
 
  7
 
 15070
 
 tss
 
 0.031222
 
 0.0313398
 
 Not queried
 
  0.8385742
 
       NA
 
           NA
  
 
  
 1
 
  22109557
 
  22109947
 
  ENSEMBL   UCSC 
 
  391
 
  9
 
 2813
 
 island
 
 0.004844
 
 0.0028252
 
 Not queried
 
  0.8385322
 
       NA
 
           NA
  
 
  
 19
 
   1401118
 
   1401492
 
  ENSEMBL   UCSC 
 
  375
 
  5
 
 19654
 
 gene
 
 0.003431
 
 0.0016778
 
 Not queried
 
  0.8384907
 
       NA
 
           NA
  
 
  
 3
 
 156544070
 
 156544233
 
  ENSEMBL   UCSC 
 
  164
 
  5
 
 29610
 
 island
 
 0.003399
 
 0.0021373
 
 Not queried
 
  0.8384085
 
       NA
 
           NA
  
 
  
 17
 
  40805728
 
  40805956
 
  ENSEMBL   UCSC 
 
  229
 
  5
 
 18346
 
 island
 
 0.128749
 
 0.2212432
 
 Not queried
 
  0.8382442
 
       NA
 
           NA
  
 
  
 8
 
 107782513
 
 107782734
 
  ENSEMBL   UCSC 
 
  222
 
  5
 
 39864
 
 tss
 
 0.702660
 
 0.7757420
 
 Not queried
 
  0.8382351
 
       NA
 
           NA
  
 
  
 16
 
  10912331
 
  10912718
 
  ENSEMBL   UCSC 
 
  388
 
  6
 
 15696
 
 island
 
 0.869579
 
 0.9654931
 
 Not queried
 
  0.8381364
 
       NA
 
           NA
  
 
  
 19
 
  12991918
 
  12992305
 
  ENSEMBL   UCSC 
 
  388
 
  6
 
 19872
 
 gene
 
 0.019616
 
 0.0076764
 
 Not queried
 
  0.8381031
 
       NA
 
           NA
  
 
  
 8
 
  82434437
 
  82434638
 
  ENSEMBL   UCSC 
 
  202
 
  5
 
 40418
 
 island
 
 0.670016
 
 0.9072435
 
 Not queried
 
  0.8379972
 
       NA
 
           NA
  
 
  
 19
 
  44952665
 
  44952808
 
  ENSEMBL   UCSC 
 
  144
 
  6
 
 20849
 
 tss
 
 0.029613
 
 0.2283003
 
 Not queried
 
  0.8379285
 
       NA
 
           NA
  
 
  
 8
 
  49173116
 
  49173425
 
  ENSEMBL   UCSC 
 
  310
 
  4
 
 40279
 
 island
 
 0.900498
 
 0.9537576
 
 Not queried
 
  0.8378139
 
       NA
 
           NA
  
 
  
 1
 
   6259688
 
   6259905
 
  ENSEMBL   UCSC 
 
  218
 
  4
 
 1198
 
 tss
 
 0.002774
 
 0.0023223
 
 Not queried
 
  0.8377959
 
       NA
 
           NA
  
 
  
 3
 
  67704890
 
  67705285
 
  ENSEMBL   UCSC 
 
  396
 
  7
 
 29267
 
 island
 
 0.013683
 
 0.0060879
 
 Not queried
 
  0.8374988
 
       NA
 
           NA
  
 
  
 14
 
  60975811
 
  60975912
 
  ENSEMBL   UCSC 
 
  102
 
  5
 
 12438
 
 tss
 
 0.039988
 
 0.0319032
 
 Not queried
 
  0.8374951
 
       NA
 
           NA
  
 
  
 7
 
  19157296
 
  19157420
 
  ENSEMBL   UCSC 
 
  125
 
  4
 
 37459
 
 tss
 
 0.011905
 
 0.0150650
 
 Not queried
 
  0.8373987
 
       NA
 
           NA
  
 
  
 8
 
  33330723
 
  33330940
 
  ENSEMBL   UCSC 
 
  218
 
  9
 
 39638
 
 tss
 
 0.011997
 
 0.0079581
 
 Not queried
 
  0.8373579
 
       NA
 
           NA
  
 
  
 2
 
  11272783
 
  11273075
 
  ENSEMBL   UCSC 
 
  293
 
  4
 
 23187
 
 tss
 
 0.008038
 
 0.0112558
 
 Not queried
 
  0.8372112
 
       NA
 
           NA
  
 
  
 1
 
 210856989
 
 210857380
 
  ENSEMBL   UCSC 
 
  392
 
  5
 
 992
 
 gene;island
 
 0.924559
 
 0.8954532
 
 Not queried
 
  0.8371938
 
       NA
 
           NA
  
 
  
 6
 
  33153052
 
  33153499
 
  ENSEMBL   UCSC 
 
  448
 
  7
 
 34211
 
 gene
 
 0.904284
 
 0.9475636
 
 Not queried
 
  0.8370739
 
       NA
 
           NA
  
 
  
 15
 
  71408603
 
  71408914
 
  ENSEMBL   UCSC 
 
  312
 
  4
 
 13771
 
 tss;island
 
 0.244095
 
 0.1376268
 
 Not queried
 
  0.8368610
 
       NA
 
           NA
  
 
  
 1
 
  92494898
 
  92495083
 
  ENSEMBL   UCSC 
 
  186
 
  4
 
 1746
 
 tss;island
 
 0.700198
 
 0.8096929
 
 Not queried
 
  0.8366477
 
       NA
 
           NA
  
 
  
 17
 
  79109729
 
  79110023
 
  ENSEMBL   UCSC 
 
  295
 
  5
 
 16897
 
 gene;island
 
 0.753109
 
 0.9167201
 
 Not queried
 
  0.8365685
 
       NA
 
           NA
  
 
  
 13
 
 100153203
 
 100153694
 
  ENSEMBL   UCSC 
 
  492
 
  9
 
 11513
 
 tss
 
 0.002499
 
 0.0009658
 
 Not queried
 
  0.8365135
 
       NA
 
           NA
  
 
  
 10
 
 119000927
 
 119001590
 
  ENSEMBL   UCSC 
 
  664
 
  8
 
 4778
 
 gene;island
 
 0.020737
 
 0.0146329
 
 Not queried
 
  0.8364184
 
       NA
 
           NA
  
 
  
 15
 
  89921672
 
  89922071
 
  ENSEMBL   UCSC 
 
  400
 
  4
 
 13468
 
 gene;island
 
 0.112923
 
 0.2072322
 
 Not queried
 
  0.8363951
 
       NA
 
           NA
  
 
  
 1
 
 192777870
 
 192778160
 
  ENSEMBL   UCSC 
 
  291
 
  5
 
 2148
 
 tss
 
 0.003957
 
 0.0032450
 
 Not queried
 
  0.8363695
 
       NA
 
           NA
  
 
  
 1
 
 192777870
 
 192778396
 
  ENSEMBL   UCSC 
 
  527
 
  9
 
 4048
 
 island
 
 0.007165
 
 0.0040375
 
 Not queried
 
  0.8363695
 
       NA
 
           NA
  
 
  
 4
 
  24981801
 
  24982173
 
  ENSEMBL   UCSC 
 
  373
 
  6
 
 30329
 
 tss
 
 0.006690
 
 0.0197453
 
 Not queried
 
  0.8363181
 
       NA
 
           NA
  
 
  
 17
 
  19247888
 
  19248009
 
  ENSEMBL   UCSC 
 
  122
 
  7
 
 17186
 
 tss
 
 0.950356
 
 0.9636524
 
 Not queried
 
  0.8363093
 
       NA
 
           NA
  
 
  
 5
 
 132148706
 
 132149015
 
  ENSEMBL   UCSC 
 
  310
 
  7
 
 32278
 
 tss
 
 0.009145
 
 0.0180322
 
 Not queried
 
  0.8361993
 
       NA
 
           NA
  
 
  
 12
 
 130645423
 
 130645645
 
  ENSEMBL   UCSC 
 
  223
 
  4
 
 10999
 
 island
 
 0.158271
 
 0.2871118
 
 Not queried
 
  0.8361715
 
       NA
 
           NA
  
 
  
 5
 
  95297792
 
  95298307
 
  ENSEMBL   UCSC 
 
  516
 
  7
 
 32188
 
 tss
 
 0.010616
 
 0.0080351
 
 Not queried
 
  0.8361636
 
       NA
 
           NA
  
 
  
 2
 
 220432815
 
 220432956
 
  ENSEMBL   UCSC 
 
  142
 
  4
 
 23042
 
 gene;island
 
 0.614354
 
 0.7455699
 
 Not queried
 
  0.8361203
 
       NA
 
           NA
  
 
  
 20
 
  62737818
 
  62738358
 
  ENSEMBL   UCSC 
 
  541
 
  8
 
 26386
 
 island
 
 0.443214
 
 0.5526150
 
 Not queried
 
  0.8359401
 
       NA
 
           NA
  
 
  
 8
 
 142190049
 
 142190410
 
  ENSEMBL   UCSC 
 
  362
 
  4
 
 39449
 
 gene
 
 0.980315
 
 0.9351404
 
 Not queried
 
  0.8359121
 
       NA
 
           NA
  
 
  
 8
 
   1327367
 
   1327621
 
  ENSEMBL   UCSC 
 
  255
 
  6
 
 40022
 
 island
 
 0.549995
 
 0.7665024
 
 Not queried
 
  0.8358466
 
       NA
 
           NA
  
 
  
 1
 
  45987615
 
  45987898
 
  ENSEMBL   UCSC 
 
  284
 
  8
 
 1586
 
 tss
 
 0.025316
 
 0.0056379
 
 Not queried
 
  0.8358412
 
       NA
 
           NA
  
 
  
 17
 
   7690159
 
   7690529
 
  ENSEMBL   UCSC 
 
  371
 
  5
 
 16413
 
 gene;island
 
 0.899471
 
 0.9302368
 
 Not queried
 
  0.8357504
 
       NA
 
           NA
  
 
  
 19
 
   3880535
 
   3880670
 
  ENSEMBL   UCSC 
 
  136
 
  4
 
 21353
 
 island
 
 0.081979
 
 0.1383827
 
 Not queried
 
  0.8356588
 
       NA
 
           NA
  
 
  
 22
 
  19746984
 
  19747140
 
  ENSEMBL   UCSC 
 
  157
 
  4
 
 26789
 
 gene;island
 
 0.159575
 
 0.2246386
 
 Not queried
 
  0.8356132
 
       NA
 
           NA
  
 
  
 4
 
 174451141
 
 174451564
 
  ENSEMBL   UCSC 
 
  424
 
 11
 
 30682
 
 tss;island
 
 0.009240
 
 0.0089939
 
 Not queried
 
  0.8352714
 
       NA
 
           NA
  
 
  
 2
 
 242930039
 
 242930747
 
  ENSEMBL   UCSC 
 
  709
 
  7
 
 25319
 
 island
 
 0.929746
 
 0.9589034
 
 Not queried
 
  0.8349171
 
       NA
 
           NA
  
 
  
 18
 
  78004045
 
  78004474
 
  ENSEMBL   UCSC 
 
  430
 
  4
 
 19147
 
 gene;island
 
 0.364277
 
 0.2006961
 
 Not queried
 
  0.8345373
 
       NA
 
           NA
  
 
  
 10
 
   6130787
 
   6130867
 
  ENSEMBL   UCSC 
 
   81
 
  5
 
 4931
 
 tss;island
 
 0.002637
 
 0.0034824
 
 Not queried
 
  0.8344950
 
       NA
 
           NA
  
 
  
 7
 
  99102313
 
  99103043
 
  ENSEMBL   UCSC 
 
  731
 
 10
 
 37128
 
 gene
 
 0.009615
 
 0.0068120
 
 Not queried
 
  0.8344284
 
       NA
 
           NA
  
 
  
 4
 
   3464653
 
   3465004
 
  ENSEMBL   UCSC 
 
  352
 
  9
 
 30262
 
 tss
 
 0.028703
 
 0.3177184
 
 Not queried
 
  0.8343338
 
       NA
 
           NA
  
 
  
 10
 
 118429452
 
 118429952
 
  ENSEMBL   UCSC 
 
  501
 
  8
 
 6165
 
 island
 
 0.176650
 
 0.4757850
 
 Not queried
 
  0.8342661
 
       NA
 
           NA
  
 
  
 14
 
  95693784
 
  95693981
 
  ENSEMBL   UCSC 
 
  198
 
  4
 
 12197
 
 gene
 
 0.962255
 
 0.9237200
 
 Not queried
 
  0.8336563
 
       NA
 
           NA
  
 
  
 4
 
   2011006
 
   2011099
 
  ENSEMBL   UCSC 
 
   94
 
  4
 
 30246
 
 tss
 
 0.008355
 
 0.0056324
 
 Not queried
 
  0.8336475
 
       NA
 
           NA
  
 
  
 4
 
 184938291
 
 184938470
 
  ENSEMBL   UCSC 
 
  180
 
  4
 
 30200
 
 gene
 
 0.813045
 
 0.8607506
 
 Not queried
 
  0.8335114
 
       NA
 
           NA
  
 
  
 11
 
  34937341
 
  34938507
 
  ENSEMBL   UCSC 
 
 1167
 
 18
 
 6582
 
 gene;island
 
 0.011411
 
 0.0066229
 
 Not queried
 
  0.8333240
 
       NA
 
           NA
  
 
  
 6
 
  10881782
 
  10882050
 
  ENSEMBL   UCSC 
 
  269
 
  6
 
 33567
 
 gene
 
 0.108540
 
 0.1678050
 
 Not queried
 
  0.8332450
 
       NA
 
           NA
  
 
  
 15
 
  60285478
 
  60285821
 
  ENSEMBL   UCSC 
 
  344
 
  5
 
 14186
 
 island
 
 0.946832
 
 0.9416946
 
 Not queried
 
  0.8330364
 
       NA
 
           NA
  
 
  
 2
 
  65454430
 
  65454775
 
  ENSEMBL   UCSC 
 
  346
 
  9
 
 23396
 
 tss
 
 0.006538
 
 0.0048690
 
 Not queried
 
  0.8330359
 
       NA
 
           NA
  
 
  
 4
 
 124317779
 
 124317901
 
  ENSEMBL   UCSC 
 
  123
 
  5
 
 30583
 
 tss
 
 0.001608
 
 0.0012854
 
 Not queried
 
  0.8330130
 
       NA
 
           NA
  
 
  
 6
 
  28956226
 
  28956989
 
  ENSEMBL   UCSC 
 
  764
 
 22
 
 35607
 
 island
 
 0.042133
 
 0.0268035
 
 Not queried
 
  0.8328274
 
       NA
 
           NA
  
 
  
 11
 
  10772408
 
  10772781
 
  ENSEMBL   UCSC 
 
  374
 
  9
 
 7148
 
 tss
 
 0.001692
 
 0.0010634
 
 Not queried
 
  0.8327573
 
       NA
 
           NA
  
 
  
 11
 
  10772408
 
  10773025
 
  ENSEMBL   UCSC 
 
  618
 
 11
 
 8060
 
 island
 
 0.004679
 
 0.0010634
 
 Not queried
 
  0.8327573
 
       NA
 
           NA
  
 
  
 5
 
 140624869
 
 140625130
 
  ENSEMBL   UCSC 
 
  262
 
  7
 
 32369
 
 tss
 
 0.023738
 
 0.0880291
 
 Not queried
 
  0.8325990
 
       NA
 
           NA
  
 
  
 6
 
  32732585
 
  32732806
 
  ENSEMBL   UCSC 
 
  222
 
  4
 
 34873
 
 tss;island
 
 0.485945
 
 0.6659938
 
 Not queried
 
  0.8325053
 
       NA
 
           NA
  
 
  
 6
 
  32119874
 
  32120324
 
  ENSEMBL   UCSC 
 
  451
 
  7
 
 34865
 
 tss
 
 0.021175
 
 0.0120799
 
 Not queried
 
  0.8324717
 
       NA
 
           NA
  
 
  
 2
 
  70314274
 
  70314576
 
  ENSEMBL   UCSC 
 
  303
 
  6
 
 23413
 
 tss;island
 
 0.010109
 
 0.0126674
 
 Not queried
 
  0.8324198
 
       NA
 
           NA
  
 
  
 22
 
  25595352
 
  25595814
 
  ENSEMBL   UCSC 
 
  463
 
  7
 
 27028
 
 tss
 
 0.455807
 
 0.5604131
 
 Not queried
 
  0.8324091
 
       NA
 
           NA
  
 
  
 20
 
  46414866
 
  46414999
 
  ENSEMBL   UCSC 
 
  134
 
  5
 
 25801
 
 tss
 
 0.005715
 
 0.0192987
 
 Not queried
 
  0.8322514
 
       NA
 
           NA
  
 
  
 21
 
  28339262
 
  28339487
 
  ENSEMBL   UCSC 
 
  226
 
  5
 
 26622
 
 island
 
 0.055808
 
 0.0282689
 
 Not queried
 
  0.8321624
 
       NA
 
           NA
  
 
  
 7
 
  79764176
 
  79764387
 
  ENSEMBL   UCSC 
 
  212
 
  4
 
 37067
 
 gene;island
 
 0.058367
 
 0.0259281
 
 Not queried
 
  0.8321261
 
       NA
 
           NA
  
 
  
 12
 
 122018177
 
 122019117
 
  ENSEMBL   UCSC 
 
  941
 
 15
 
 10915
 
 island
 
 0.014259
 
 0.0219106
 
 Not queried
 
  0.8321201
 
       NA
 
           NA
  
 
  
 10
 
  11504503
 
  11505056
 
  ENSEMBL   UCSC 
 
  554
 
  6
 
 4495
 
 gene;island
 
 0.945304
 
 0.9684222
 
 Not queried
 
  0.8320928
 
       NA
 
           NA
  
 
  
 7
 
 111846693
 
 111846915
 
  ENSEMBL   UCSC 
 
  223
 
  5
 
 37189
 
 gene
 
 0.003930
 
 0.0034870
 
 Not queried
 
  0.8320253
 
       NA
 
           NA
  
 
  
 17
 
   7826155
 
   7826413
 
  ENSEMBL   UCSC 
 
  259
 
  5
 
 16421
 
 gene;island
 
 0.109374
 
 0.0646771
 
 Not queried
 
  0.8319526
 
       NA
 
           NA
  
 
  
 2
 
 234652363
 
 234652681
 
  ENSEMBL   UCSC 
 
  319
 
  5
 
 23077
 
 gene;island
 
 0.788768
 
 0.8603310
 
 Not queried
 
  0.8316800
 
       NA
 
           NA
  
 
  
 1
 
 233749295
 
 233749672
 
  ENSEMBL   UCSC 
 
  378
 
  8
 
 2327
 
 tss;island
 
 0.047522
 
 0.0262424
 
 Not queried
 
  0.8313156
 
       NA
 
           NA
  
 
  
 15
 
  75748063
 
  75748191
 
  ENSEMBL   UCSC 
 
  129
 
  5
 
 14331
 
 island
 
 0.010482
 
 0.0018485
 
 Not queried
 
  0.8312125
 
       NA
 
           NA
  
 
  
 6
 
 106439790
 
 106440215
 
  ENSEMBL   UCSC 
 
  426
 
  4
 
 36379
 
 island
 
 0.062650
 
 0.0193445
 
 Not queried
 
  0.8310870
 
       NA
 
           NA
  
 
  
 19
 
   1812282
 
   1812854
 
  ENSEMBL   UCSC 
 
  573
 
  6
 
 20312
 
 tss;island
 
 0.008513
 
 0.0060758
 
 Not queried
 
  0.8310692
 
       NA
 
           NA
  
 
  
 11
 
 133907114
 
 133907497
 
  ENSEMBL   UCSC 
 
  384
 
  4
 
 6988
 
 gene;island
 
 0.009187
 
 0.0144353
 
 Not queried
 
  0.8308609
 
       NA
 
           NA
  
 
  
 22
 
  35777001
 
  35777245
 
  ENSEMBL   UCSC 
 
  245
 
  4
 
 27446
 
 island
 
 0.006600
 
 0.0040436
 
 Not queried
 
  0.8307223
 
       NA
 
           NA
  
 
  
 4
 
 146100881
 
 146101151
 
  ENSEMBL   UCSC 
 
  271
 
  4
 
 30618
 
 tss
 
 0.000877
 
 0.0009125
 
 Not queried
 
  0.8306488
 
       NA
 
           NA
  
 
  
 16
 
  31214307
 
  31214607
 
  ENSEMBL   UCSC 
 
  301
 
  8
 
 15209
 
 tss
 
 0.012164
 
 0.0257854
 
 Not queried
 
  0.8302543
 
       NA
 
           NA
  
 
  
 16
 
  31214126
 
  31214607
 
  ENSEMBL   UCSC 
 
  482
 
 10
 
 15873
 
 island
 
 0.013560
 
 0.0270637
 
 Not queried
 
  0.8302543
 
       NA
 
           NA
  
 
  
 8
 
  12989237
 
  12989686
 
  ENSEMBL   UCSC 
 
  450
 
  4
 
 39211
 
 gene;island
 
 0.222685
 
 0.2518703
 
 Not queried
 
  0.8302109
 
       NA
 
           NA
  
 
  
 17
 
  48546258
 
  48546620
 
  ENSEMBL   UCSC 
 
  363
 
  8
 
 17500
 
 tss
 
 0.053806
 
 0.1073512
 
 Not queried
 
  0.8301206
 
       NA
 
           NA
  
 
  
 12
 
  56511647
 
  56511953
 
  ENSEMBL   UCSC 
 
  307
 
  8
 
 9784
 
 tss
 
 0.006758
 
 0.0049940
 
 Not queried
 
  0.8300099
 
       NA
 
           NA
  
 
  
 19
 
  58125438
 
  58125678
 
  ENSEMBL   UCSC 
 
  241
 
  8
 
 21121
 
 tss
 
 0.008243
 
 0.0041843
 
 Not queried
 
  0.8298669
 
       NA
 
           NA
  
 
  
 2
 
 119607097
 
 119607636
 
  ENSEMBL   UCSC 
 
  540
 
  7
 
 24649
 
 island
 
 0.042500
 
 0.2685019
 
 Not queried
 
  0.8296177
 
       NA
 
           NA
  
 
  
 3
 
 122102727
 
 122103261
 
  ENSEMBL   UCSC 
 
  535
 
  6
 
 28536
 
 tss;island
 
 0.002299
 
 0.0026598
 
 Not queried
 
  0.8296067
 
       NA
 
           NA
  
 
  
 19
 
   7741602
 
   7741955
 
  ENSEMBL   UCSC 
 
  354
 
  5
 
 21462
 
 island
 
 0.010551
 
 0.0056305
 
 Not queried
 
  0.8295825
 
       NA
 
           NA
  
 
  
 12
 
 133065724
 
 133066069
 
  ENSEMBL   UCSC 
 
  346
 
 16
 
 10130
 
 tss;island
 
 0.006413
 
 0.0117325
 
 Not queried
 
  0.8295601
 
       NA
 
           NA
  
 
  
 11
 
   8190448
 
   8190837
 
  ENSEMBL   UCSC 
 
  390
 
  9
 
 8030
 
 island
 
 0.074790
 
 0.0749069
 
 Not queried
 
  0.8294369
 
       NA
 
           NA
  
 
  
 17
 
  42143602
 
  42143874
 
  ENSEMBL   UCSC 
 
  273
 
  4
 
 16604
 
 gene;island
 
 0.021103
 
 0.0146240
 
 Not queried
 
  0.8292663
 
       NA
 
           NA
  
 
  
 16
 
  89574433
 
  89574710
 
  ENSEMBL   UCSC 
 
  278
 
  6
 
 15425
 
 tss
 
 0.005062
 
 0.0040506
 
 Not queried
 
  0.8291876
 
       NA
 
           NA
  
 
  
 17
 
  39913753
 
  39913952
 
  ENSEMBL   UCSC 
 
  200
 
  4
 
 16571
 
 gene;island
 
 0.720286
 
 0.8309586
 
 Not queried
 
  0.8290986
 
       NA
 
           NA
  
 
  
 1
 
  91870362
 
  91870450
 
  ENSEMBL   UCSC 
 
   89
 
  4
 
 3472
 
 island
 
 0.010105
 
 0.0094396
 
 Not queried
 
  0.8289924
 
       NA
 
           NA
  
 
  
 10
 
 129845375
 
 129846082
 
  ENSEMBL   UCSC 
 
  708
 
  8
 
 4821
 
 gene
 
 0.035370
 
 0.0484241
 
 Not queried
 
  0.8289168
 
       NA
 
           NA
  
 
  
 5
 
 177697400
 
 177697669
 
  ENSEMBL   UCSC 
 
  270
 
  4
 
 31926
 
 gene
 
 0.542535
 
 0.6783276
 
 Not queried
 
  0.8288914
 
       NA
 
           NA
  
 
  
 7
 
 152131894
 
 152132218
 
  ENSEMBL   UCSC 
 
  325
 
  4
 
 37300
 
 gene
 
 0.012461
 
 0.0272205
 
 Not queried
 
  0.8288774
 
       NA
 
           NA
  
 
  
 22
 
  45705512
 
  45705707
 
  ENSEMBL   UCSC 
 
  196
 
  6
 
 27222
 
 tss
 
 0.003108
 
 0.0022674
 
 Not queried
 
  0.8288525
 
       NA
 
           NA
  
 
  
 1
 
  91300215
 
  91300559
 
  ENSEMBL   UCSC 
 
  345
 
  7
 
 3466
 
 island
 
 0.029869
 
 0.2158189
 
 Not queried
 
  0.8287650
 
       NA
 
           NA
  
 
  
 1
 
  35226707
 
  35227272
 
  ENSEMBL   UCSC 
 
  566
 
  6
 
 412
 
 gene;island
 
 0.411750
 
 0.5920182
 
 Not queried
 
  0.8286899
 
       NA
 
           NA
  
 
  
 7
 
  27239556
 
  27239763
 
  ENSEMBL   UCSC 
 
  208
 
  4
 
 38362
 
 island
 
 0.043959
 
 0.0535334
 
 Not queried
 
  0.8286307
 
       NA
 
           NA
  
 
  
 7
 
   5462583
 
   5462771
 
  ENSEMBL   UCSC 
 
  189
 
  4
 
 36877
 
 gene;island
 
 0.009076
 
 0.0070625
 
 Not queried
 
  0.8284679
 
       NA
 
           NA
  
 
  
 3
 
 160823231
 
 160823505
 
  ENSEMBL   UCSC 
 
  275
 
  7
 
 28697
 
 tss;island
 
 0.200745
 
 0.1675404
 
 Not queried
 
  0.8284500
 
       NA
 
           NA
  
 
  
 1
 
 156405249
 
 156405495
 
  ENSEMBL   UCSC 
 
  247
 
  4
 
 3856
 
 island
 
 0.038743
 
 0.0143658
 
 Not queried
 
  0.8284421
 
       NA
 
           NA
  
 
  
 17
 
    883084
 
    883294
 
  ENSEMBL   UCSC 
 
  211
 
  4
 
 16983
 
 tss;island
 
 0.017559
 
 0.0246684
 
 Not queried
 
  0.8283736
 
       NA
 
           NA
  
 
  
 7
 
   1315546
 
   1315866
 
  ENSEMBL   UCSC 
 
  321
 
  4
 
 38096
 
 island
 
 0.783170
 
 0.8438328
 
 Not queried
 
  0.8283538
 
       NA
 
           NA
  
 
  
 22
 
  19419839
 
  19420336
 
  ENSEMBL   UCSC 
 
  498
 
  8
 
 26957
 
 tss;island
 
 0.004308
 
 0.0027036
 
 Not queried
 
  0.8283097
 
       NA
 
           NA
  
 
  
 16
 
  71659830
 
  71660055
 
  ENSEMBL   UCSC 
 
  226
 
  5
 
 15348
 
 tss
 
 0.013257
 
 0.0236694
 
 Not queried
 
  0.8281780
 
       NA
 
           NA
  
 
  
 16
 
  71659830
 
  71660113
 
  ENSEMBL   UCSC 
 
  284
 
  7
 
 16077
 
 island
 
 0.014298
 
 0.0236694
 
 Not queried
 
  0.8281780
 
       NA
 
           NA
  
 
  
 19
 
  18061155
 
  18061472
 
  ENSEMBL   UCSC 
 
  318
 
  4
 
 20606
 
 tss;island
 
 0.012267
 
 0.0075670
 
 Not queried
 
  0.8281108
 
       NA
 
           NA
  
 
  
 5
 
 178801332
 
 178801812
 
  ENSEMBL   UCSC 
 
  481
 
  4
 
 33484
 
 island
 
 0.149856
 
 0.2391349
 
 Not queried
 
  0.8280961
 
       NA
 
           NA
  
 
  
 1
 
 202780290
 
 202780680
 
  ENSEMBL   UCSC 
 
  391
 
  4
 
 4092
 
 island
 
 0.038316
 
 0.0182121
 
 Not queried
 
  0.8278445
 
       NA
 
           NA
  
 
  
 1
 
  24645885
 
  24646392
 
  ENSEMBL   UCSC 
 
  508
 
  9
 
 326
 
 gene
 
 0.018852
 
 0.0648931
 
 Not queried
 
  0.8277118
 
       NA
 
           NA
  
 
  
 17
 
   7518273
 
   7518515
 
  ENSEMBL   UCSC 
 
  243
 
  5
 
 17092
 
 tss
 
 0.007469
 
 0.0117082
 
 Not queried
 
  0.8277013
 
       NA
 
           NA
  
 
  
 19
 
  56165125
 
  56165316
 
  ENSEMBL   UCSC 
 
  192
 
  4
 
 21088
 
 tss
 
 0.011743
 
 0.0076608
 
 Not queried
 
  0.8274346
 
       NA
 
           NA
  
 
  
 20
 
  25062754
 
  25063052
 
  ENSEMBL   UCSC 
 
  299
 
  8
 
 26057
 
 island
 
 0.041382
 
 0.1041237
 
 Not queried
 
  0.8272630
 
       NA
 
           NA
  
 
  
 1
 
 156862113
 
 156862299
 
  ENSEMBL   UCSC 
 
  187
 
  4
 
 2025
 
 tss;island
 
 0.006113
 
 0.0064529
 
 Not queried
 
  0.8272537
 
       NA
 
           NA
  
 
  
 8
 
 102217569
 
 102217924
 
  ENSEMBL   UCSC 
 
  356
 
  7
 
 39383
 
 gene
 
 0.023216
 
 0.0326316
 
 Not queried
 
  0.8266233
 
       NA
 
           NA
  
 
  
 12
 
 115120789
 
 115121489
 
  ENSEMBL   UCSC 
 
  701
 
  8
 
 9327
 
 gene;island
 
 0.058676
 
 0.0560806
 
 Not queried
 
  0.8266160
 
       NA
 
           NA
  
 
  
 5
 
  39425138
 
  39425549
 
  ENSEMBL   UCSC 
 
  412
 
  8
 
 32748
 
 island
 
 0.049614
 
 0.0315732
 
 Not queried
 
  0.8264432
 
       NA
 
           NA
  
 
  
 1
 
 247275326
 
 247275542
 
  ENSEMBL   UCSC 
 
  217
 
  5
 
 1124
 
 gene
 
 0.058122
 
 0.0638767
 
 Not queried
 
  0.8262711
 
       NA
 
           NA
  
 
  
 13
 
  52158382
 
  52158459
 
  ENSEMBL   UCSC 
 
   78
 
  5
 
 11452
 
 tss;island
 
 0.004475
 
 0.0025357
 
 Not queried
 
  0.8261745
 
       NA
 
           NA
  
 
  
 2
 
  27994656
 
  27994816
 
  ENSEMBL   UCSC 
 
  161
 
  4
 
 22634
 
 gene
 
 0.005913
 
 0.0054844
 
 Not queried
 
  0.8260873
 
       NA
 
           NA
  
 
  
 4
 
   4866454
 
   4867164
 
  ENSEMBL   UCSC 
 
  711
 
  6
 
 30872
 
 island
 
 0.117995
 
 0.1176576
 
 Not queried
 
  0.8260652
 
       NA
 
           NA
  
 
  
 3
 
   8543014
 
   8543508
 
  ENSEMBL   UCSC 
 
  495
 
  8
 
 28120
 
 tss
 
 0.013936
 
 0.0086856
 
 Not queried
 
  0.8260583
 
       NA
 
           NA
  
 
  
 3
 
   8543014
 
   8543732
 
  ENSEMBL   UCSC 
 
  719
 
 10
 
 28849
 
 island
 
 0.020489
 
 0.0104369
 
 Not queried
 
  0.8260583
 
       NA
 
           NA
  
 
  
 11
 
 134582289
 
 134582508
 
  ENSEMBL   UCSC 
 
  220
 
  4
 
 8989
 
 island
 
 0.525576
 
 0.5510485
 
 Not queried
 
  0.8260186
 
       NA
 
           NA
  
 
  
 19
 
  58838420
 
  58838697
 
  ENSEMBL   UCSC 
 
  278
 
  4
 
 20260
 
 gene
 
 0.003011
 
 0.0027722
 
 Not queried
 
  0.8259610
 
       NA
 
           NA
  
 
  
 4
 
   4861618
 
   4862240
 
  ENSEMBL   UCSC 
 
  623
 
  6
 
 29918
 
 gene;island
 
 0.016002
 
 0.0094697
 
 Not queried
 
  0.8259164
 
       NA
 
           NA
  
 
  
 17
 
   1945076
 
   1945160
 
  ENSEMBL   UCSC 
 
   85
 
  6
 
 16326
 
 gene;tss;island
 
 0.013424
 
 0.0093340
 
 Not queried
 
  0.8258166
 
       NA
 
           NA
  
 
  
 3
 
 128206790
 
 128206967
 
  ENSEMBL   UCSC 
 
  178
 
  5
 
 28569
 
 tss
 
 0.020444
 
 0.0195939
 
 Not queried
 
  0.8257558
 
       NA
 
           NA
  
 
  
 16
 
  70720079
 
  70720440
 
  ENSEMBL   UCSC 
 
  362
 
  6
 
 15343
 
 tss;island
 
 0.117378
 
 0.3209400
 
 Not queried
 
  0.8256672
 
       NA
 
           NA
  
 
  
 7
 
  56031952
 
  56032122
 
  ENSEMBL   UCSC 
 
  171
 
  6
 
 37606
 
 tss;island
 
 0.027918
 
 0.0145930
 
 Not queried
 
  0.8256128
 
       NA
 
           NA
  
 
  
 19
 
  40005557
 
  40005836
 
  ENSEMBL   UCSC 
 
  280
 
 10
 
 22001
 
 island
 
 0.026756
 
 0.0280053
 
 Not queried
 
  0.8255035
 
       NA
 
           NA
  
 
  
 3
 
  42306467
 
  42306974
 
  ENSEMBL   UCSC 
 
  508
 
  7
 
 28242
 
 tss
 
 0.117843
 
 0.1092857
 
 Not queried
 
  0.8252955
 
       NA
 
           NA
  
 
  
 1
 
 149859456
 
 149859690
 
  ENSEMBL   UCSC 
 
  235
 
  5
 
 1899
 
 tss;island
 
 0.000734
 
 0.0014734
 
 Not queried
 
  0.8250153
 
       NA
 
           NA
  
 
  
 8
 
  21644953
 
  21645524
 
  ENSEMBL   UCSC 
 
  572
 
  6
 
 39224
 
 gene;island
 
 0.012482
 
 0.0197647
 
 Not queried
 
  0.8248382
 
       NA
 
           NA
  
 
  
 18
 
   5196738
 
   5197071
 
  ENSEMBL   UCSC 
 
  334
 
  4
 
 19046
 
 gene;island
 
 0.145687
 
 0.0518704
 
 Not queried
 
  0.8245476
 
       NA
 
           NA
  
 
  
 4
 
 185939339
 
 185940173
 
  ENSEMBL   UCSC 
 
  835
 
 12
 
 31467
 
 island
 
 0.024101
 
 0.0169633
 
 Not queried
 
  0.8244883
 
       NA
 
           NA
  
 
  
 2
 
  74880862
 
  74881474
 
  ENSEMBL   UCSC 
 
  613
 
  7
 
 24450
 
 island
 
 0.018986
 
 0.0096618
 
 Not queried
 
  0.8242964
 
       NA
 
           NA
  
 
  
 19
 
  57831544
 
  57831849
 
  ENSEMBL   UCSC 
 
  306
 
 11
 
 21110
 
 tss
 
 0.008242
 
 0.0094386
 
 Not queried
 
  0.8240527
 
       NA
 
           NA
  
 
  
 8
 
  28560467
 
  28560723
 
  ENSEMBL   UCSC 
 
  257
 
  5
 
 39262
 
 gene;island
 
 0.941271
 
 0.8540018
 
 Not queried
 
  0.8238901
 
       NA
 
           NA
  
 
  
 14
 
  38725650
 
  38725750
 
  ENSEMBL   UCSC 
 
  101
 
  4
 
 12364
 
 tss
 
 0.234826
 
 0.4235697
 
 Not queried
 
  0.8237222
 
       NA
 
           NA
  
 
  
 20
 
  33759605
 
  33759753
 
  ENSEMBL   UCSC 
 
  149
 
  4
 
 25702
 
 tss
 
 0.038913
 
 0.1814282
 
 Not queried
 
  0.8236911
 
       NA
 
           NA
  
 
  
 14
 
  74111075
 
  74111498
 
  ENSEMBL   UCSC 
 
  424
 
  6
 
 12498
 
 tss
 
 0.014635
 
 0.0100611
 
 Not queried
 
  0.8236857
 
       NA
 
           NA
  
 
  
 12
 
 114846313
 
 114846503
 
  ENSEMBL   UCSC 
 
  191
 
  7
 
 10033
 
 tss
 
 0.047300
 
 0.0183605
 
 Not queried
 
  0.8235878
 
       NA
 
           NA
  
 
  
 15
 
  40697714
 
  40698044
 
  ENSEMBL   UCSC 
 
  331
 
  4
 
 13292
 
 gene
 
 0.007907
 
 0.0034282
 
 Not queried
 
  0.8232928
 
       NA
 
           NA
  
 
  
 4
 
    828098
 
    828281
 
  ENSEMBL   UCSC 
 
  184
 
  4
 
 30738
 
 island
 
 0.843388
 
 0.8738382
 
 Not queried
 
  0.8230913
 
       NA
 
           NA
  
 
  
 3
 
  85007732
 
  85008156
 
  ENSEMBL   UCSC 
 
  425
 
  8
 
 29297
 
 island
 
 0.012160
 
 0.0292687
 
 Not queried
 
  0.8230763
 
       NA
 
           NA
  
 
  
 5
 
 140207460
 
 140207636
 
  ENSEMBL   UCSC 
 
  177
 
  5
 
 32352
 
 tss
 
 0.742487
 
 0.6679894
 
 Not queried
 
  0.8230514
 
       NA
 
           NA
  
 
  
 1
 
 248100183
 
 248100427
 
  ENSEMBL   UCSC 
 
  245
 
  6
 
 2372
 
 tss
 
 0.044265
 
 0.2448408
 
 Not queried
 
  0.8228742
 
       NA
 
           NA
  
 
  
 2
 
  38830581
 
  38830741
 
  ENSEMBL   UCSC 
 
  161
 
  4
 
 23311
 
 tss;island
 
 0.006145
 
 0.0070676
 
 Not queried
 
  0.8228014
 
       NA
 
           NA
  
 
  
 17
 
  56832804
 
  56833201
 
  ENSEMBL   UCSC 
 
  398
 
 11
 
 17540
 
 tss
 
 0.013136
 
 0.0392230
 
 Not queried
 
  0.8226902
 
       NA
 
           NA
  
 
  
 6
 
 116575311
 
 116575339
 
  ENSEMBL   UCSC 
 
   29
 
  5
 
 35188
 
 tss
 
 0.002464
 
 0.0025077
 
 Not queried
 
  0.8226560
 
       NA
 
           NA
  
 
  
 6
 
 170456806
 
 170457111
 
  ENSEMBL   UCSC 
 
  306
 
  5
 
 36679
 
 island
 
 0.694269
 
 0.8007014
 
 Not queried
 
  0.8225646
 
       NA
 
           NA
  
 
  
 14
 
 101489415
 
 101489781
 
  ENSEMBL   UCSC 
 
  367
 
  7
 
 12619
 
 tss
 
 0.346930
 
 0.5089585
 
 Not queried
 
  0.8224233
 
       NA
 
           NA
  
 
  
 2
 
  97173852
 
  97174391
 
  ENSEMBL   UCSC 
 
  540
 
 10
 
 23517
 
 tss
 
 0.019482
 
 0.0255726
 
 Not queried
 
  0.8222651
 
       NA
 
           NA
  
 
  
 15
 
 100882213
 
 100882647
 
  ENSEMBL   UCSC 
 
  435
 
  8
 
 13926
 
 tss
 
 0.092373
 
 0.0280294
 
 Not queried
 
  0.8222355
 
       NA
 
           NA
  
 
  
 5
 
  54528989
 
  54529452
 
  ENSEMBL   UCSC 
 
  464
 
  5
 
 31616
 
 gene
 
 0.049447
 
 0.0143242
 
 Not queried
 
  0.8220226
 
       NA
 
           NA
  
 
  
 16
 
  89225495
 
  89225922
 
  ENSEMBL   UCSC 
 
  428
 
  6
 
 16244
 
 island
 
 0.726172
 
 0.9148802
 
 Not queried
 
  0.8219184
 
       NA
 
           NA
  
 
  
 7
 
 100034139
 
 100034454
 
  ENSEMBL   UCSC 
 
  316
 
  6
 
 37782
 
 tss
 
 0.014039
 
 0.0064669
 
 Not queried
 
  0.8217791
 
       NA
 
           NA
  
 
  
 4
 
  17513197
 
  17513991
 
  ENSEMBL   UCSC 
 
  795
 
  9
 
 30944
 
 island
 
 0.018965
 
 0.0100799
 
 Not queried
 
  0.8217714
 
       NA
 
           NA
  
 
  
 7
 
 100463416
 
 100463759
 
  ENSEMBL   UCSC 
 
  344
 
  4
 
 37148
 
 gene;island
 
 0.582457
 
 0.6692927
 
 Not queried
 
  0.8217130
 
       NA
 
           NA
  
 
  
 12
 
  58068506
 
  58068806
 
  ENSEMBL   UCSC 
 
  301
 
  6
 
 10571
 
 island
 
 0.698948
 
 0.7834649
 
 Not queried
 
  0.8216099
 
       NA
 
           NA
  
 
  
 14
 
 104583632
 
 104584279
 
  ENSEMBL   UCSC 
 
  648
 
  6
 
 13202
 
 island
 
 0.302824
 
 0.1848829
 
 Not queried
 
  0.8214565
 
       NA
 
           NA
  
 
  
 3
 
 141496874
 
 141496998
 
  ENSEMBL   UCSC 
 
  125
 
  5
 
 28639
 
 tss
 
 0.693815
 
 0.5236773
 
 Not queried
 
  0.8213946
 
       NA
 
           NA
  
 
  
 1
 
   6639570
 
   6640149
 
  ENSEMBL   UCSC 
 
  580
 
  9
 
 2613
 
 island
 
 0.008238
 
 0.0100845
 
 Not queried
 
  0.8213540
 
       NA
 
           NA
  
 
  
 1
 
 180472145
 
 180472354
 
  ENSEMBL   UCSC 
 
  210
 
  5
 
 2125
 
 tss;island
 
 0.005429
 
 0.0021422
 
 Not queried
 
  0.8211609
 
       NA
 
           NA
  
 
  
 16
 
  14403004
 
  14403138
 
  ENSEMBL   UCSC 
 
  135
 
  4
 
 15102
 
 tss
 
 0.012320
 
 0.0081305
 
 Not queried
 
  0.8210507
 
       NA
 
           NA
  
 
  
 17
 
  76967629
 
  76967825
 
  ENSEMBL   UCSC 
 
  197
 
  4
 
 16830
 
 gene
 
 0.254831
 
 0.3607273
 
 Not queried
 
  0.8210002
 
       NA
 
           NA
  
 
  
 2
 
  85555411
 
  85555614
 
  ENSEMBL   UCSC 
 
  204
 
  8
 
 23479
 
 tss
 
 0.020587
 
 0.0115597
 
 Not queried
 
  0.8209894
 
       NA
 
           NA
  
 
  
 20
 
  61885236
 
  61885291
 
  ENSEMBL   UCSC 
 
   56
 
  6
 
 25892
 
 tss
 
 0.004077
 
 0.0078901
 
 Not queried
 
  0.8208113
 
       NA
 
           NA
  
 
  
 8
 
  29940777
 
  29940921
 
  ENSEMBL   UCSC 
 
  145
 
  5
 
 39629
 
 tss
 
 0.006133
 
 0.0069199
 
 Not queried
 
  0.8205800
 
       NA
 
           NA
  
 
  
 10
 
  99532138
 
  99532410
 
  ENSEMBL   UCSC 
 
  273
 
  6
 
 5273
 
 tss;island
 
 0.551178
 
 0.7229669
 
 Not queried
 
  0.8204694
 
       NA
 
           NA
  
 
  
 6
 
  32137968
 
  32138406
 
  ENSEMBL   UCSC 
 
  439
 
  9
 
 34067
 
 gene;island
 
 0.771201
 
 0.9039872
 
 Not queried
 
  0.8203162
 
       NA
 
           NA
  
 
  
 19
 
  23941711
 
  23942126
 
  ENSEMBL   UCSC 
 
  416
 
  8
 
 20665
 
 tss
 
 0.161592
 
 0.5893007
 
 Not queried
 
  0.8200389
 
       NA
 
           NA
  
 
  
 21
 
  42879998
 
  42880107
 
  ENSEMBL   UCSC 
 
  110
 
  4
 
 26558
 
 tss;island
 
 0.012045
 
 0.0145942
 
 Not queried
 
  0.8199659
 
       NA
 
           NA
  
 
  
 2
 
 127865197
 
 127865431
 
  ENSEMBL   UCSC 
 
  235
 
  5
 
 23609
 
 tss;island
 
 0.010501
 
 0.0452623
 
 Not queried
 
  0.8199526
 
       NA
 
           NA
  
 
  
 4
 
  87927655
 
  87928110
 
  ENSEMBL   UCSC 
 
  456
 
  9
 
 30485
 
 tss
 
 0.008042
 
 0.0048795
 
 Not queried
 
  0.8198663
 
       NA
 
           NA
  
 
  
 17
 
  29719023
 
  29719431
 
  ENSEMBL   UCSC 
 
  409
 
  5
 
 16510
 
 gene;island
 
 0.059722
 
 0.0335014
 
 Not queried
 
  0.8198416
 
       NA
 
           NA
  
 
  
 5
 
 178651074
 
 178651519
 
  ENSEMBL   UCSC 
 
  446
 
  4
 
 31930
 
 gene;island
 
 0.760455
 
 0.8483730
 
 Not queried
 
  0.8198181
 
       NA
 
           NA
  
 
  
 8
 
   1899943
 
   1900524
 
  ENSEMBL   UCSC 
 
  582
 
  6
 
 39185
 
 gene
 
 0.467896
 
 0.6061385
 
 Not queried
 
  0.8197799
 
       NA
 
           NA
  
 
  
 1
 
 245753302
 
 245753635
 
  ENSEMBL   UCSC 
 
  334
 
  4
 
 1118
 
 gene;island
 
 0.402798
 
 0.6538689
 
 Not queried
 
  0.8195260
 
       NA
 
           NA
  
 
  
 11
 
  76757006
 
  76757278
 
  ENSEMBL   UCSC 
 
  273
 
  5
 
 8677
 
 island
 
 0.065738
 
 0.0332735
 
 Not queried
 
  0.8194696
 
       NA
 
           NA
  
 
  
 17
 
  72869061
 
  72869237
 
  ENSEMBL   UCSC 
 
  177
 
  9
 
 18727
 
 island
 
 0.004888
 
 0.0046811
 
 Not queried
 
  0.8194427
 
       NA
 
           NA
  
 
  
 10
 
  57389676
 
  57390047
 
  ENSEMBL   UCSC 
 
  372
 
  4
 
 5754
 
 island
 
 0.023008
 
 0.0093364
 
 Not queried
 
  0.8193060
 
       NA
 
           NA
  
 
  
 1
 
 234614614
 
 234614834
 
  ENSEMBL   UCSC 
 
  221
 
  4
 
 1086
 
 gene
 
 0.005405
 
 0.0023107
 
 Not queried
 
  0.8192595
 
       NA
 
           NA
  
 
  
 4
 
  37455280
 
  37455499
 
  ENSEMBL   UCSC 
 
  220
 
  7
 
 30341
 
 tss;island
 
 0.059755
 
 0.0384162
 
 Not queried
 
  0.8192188
 
       NA
 
           NA
  
 
  
 5
 
 109026877
 
 109027037
 
  ENSEMBL   UCSC 
 
  161
 
  4
 
 31706
 
 gene;island
 
 0.004905
 
 0.0050325
 
 Not queried
 
  0.8192136
 
       NA
 
           NA
  
 
  
 13
 
  23489817
 
  23490053
 
  ENSEMBL   UCSC 
 
  237
 
  4
 
 11595
 
 island
 
 0.246076
 
 0.3426546
 
 Not queried
 
  0.8191602
 
       NA
 
           NA
  
 
  
 6
 
 134638742
 
 134639020
 
  ENSEMBL   UCSC 
 
  279
 
  5
 
 34483
 
 gene
 
 0.100761
 
 0.0494505
 
 Not queried
 
  0.8190916
 
       NA
 
           NA
  
 
  
 4
 
  68567018
 
  68567174
 
  ENSEMBL   UCSC 
 
  157
 
  7
 
 30014
 
 gene
 
 0.004012
 
 0.0027055
 
 Not queried
 
  0.8189227
 
       NA
 
           NA
  
 
  
 12
 
  16034932
 
  16035282
 
  ENSEMBL   UCSC 
 
  351
 
  8
 
 9593
 
 tss
 
 0.005483
 
 0.0022122
 
 Not queried
 
  0.8186167
 
       NA
 
           NA
  
 
  
 17
 
   7475727
 
   7476052
 
  ENSEMBL   UCSC 
 
  326
 
  8
 
 17088
 
 tss
 
 0.022985
 
 0.0144081
 
 Not queried
 
  0.8185859
 
       NA
 
           NA
  
 
  
 1
 
 201438445
 
 201438757
 
  ENSEMBL   UCSC 
 
  313
 
  5
 
 2167
 
 tss
 
 0.025522
 
 0.0152781
 
 Not queried
 
  0.8185497
 
       NA
 
           NA
  
 
  
 16
 
  34777682
 
  34778087
 
  ENSEMBL   UCSC 
 
  406
 
  4
 
 15893
 
 island
 
 0.460753
 
 0.6680334
 
 Not queried
 
  0.8184782
 
       NA
 
           NA
  
 
  
 7
 
  72349822
 
  72350033
 
  ENSEMBL   UCSC 
 
  212
 
  6
 
 38549
 
 island
 
 0.011867
 
 0.0072592
 
 Not queried
 
  0.8184476
 
       NA
 
           NA
  
 
  
 2
 
  99952550
 
  99952800
 
  ENSEMBL   UCSC 
 
  251
 
  4
 
 22809
 
 gene
 
 0.002604
 
 0.0056828
 
 Not queried
 
  0.8177705
 
       NA
 
           NA
  
 
  
 13
 
  24844846
 
  24844938
 
  ENSEMBL   UCSC 
 
   93
 
  6
 
 11106
 
 gene
 
 0.005308
 
 0.0029387
 
 Not queried
 
  0.8175699
 
       NA
 
           NA
  
 
  
 17
 
  77901030
 
  77901317
 
  ENSEMBL   UCSC 
 
  288
 
  4
 
 18846
 
 island
 
 0.423040
 
 0.7201635
 
 Not queried
 
  0.8175160
 
       NA
 
           NA
  
 
  
 6
 
  70992907
 
  70993107
 
  ENSEMBL   UCSC 
 
  201
 
  4
 
 34383
 
 gene;island
 
 0.135094
 
 0.1997451
 
 Not queried
 
  0.8173624
 
       NA
 
           NA
  
 
  
 18
 
    908130
 
    908505
 
  ENSEMBL   UCSC 
 
  376
 
  4
 
 19034
 
 gene;island
 
 0.054463
 
 0.0592989
 
 Not queried
 
  0.8173522
 
       NA
 
           NA
  
 
  
 12
 
  69979077
 
  69979187
 
  ENSEMBL   UCSC 
 
  111
 
  4
 
 9862
 
 tss
 
 0.011015
 
 0.0071439
 
 Not queried
 
  0.8172679
 
       NA
 
           NA
  
 
  
 10
 
 134360912
 
 134361203
 
  ENSEMBL   UCSC 
 
  292
 
  5
 
 4864
 
 gene;island
 
 0.877432
 
 0.5846848
 
 Not queried
 
  0.8171608
 
       NA
 
           NA
  
 
  
 13
 
  19755954
 
  19756318
 
  ENSEMBL   UCSC 
 
  365
 
  8
 
 11322
 
 tss
 
 0.428559
 
 0.5961450
 
 Not queried
 
  0.8170831
 
       NA
 
           NA
  
 
  
 19
 
   8809057
 
   8809360
 
  ENSEMBL   UCSC 
 
  304
 
  4
 
 21491
 
 island
 
 0.909163
 
 0.9318044
 
 Not queried
 
  0.8170617
 
       NA
 
           NA
  
 
  
 5
 
 122759586
 
 122759911
 
  ENSEMBL   UCSC 
 
  326
 
  4
 
 32249
 
 tss;island
 
 0.007963
 
 0.0043682
 
 Not queried
 
  0.8170617
 
       NA
 
           NA
  
 
  
 2
 
 136633306
 
 136633712
 
  ENSEMBL   UCSC 
 
  407
 
  4
 
 22884
 
 gene;island
 
 0.008055
 
 0.0099524
 
 Not queried
 
  0.8170165
 
       NA
 
           NA
  
 
  
 6
 
  32525813
 
  32526027
 
  ENSEMBL   UCSC 
 
  215
 
  5
 
 34117
 
 gene
 
 0.602107
 
 0.7478835
 
 Not queried
 
  0.8169130
 
       NA
 
           NA
  
 
  
 21
 
  36041334
 
  36041699
 
  ENSEMBL   UCSC 
 
  366
 
  8
 
 26662
 
 island
 
 0.063565
 
 0.0465508
 
 Not queried
 
  0.8168684
 
       NA
 
           NA
  
 
  
 10
 
 103578255
 
 103578627
 
  ENSEMBL   UCSC 
 
  373
 
  7
 
 5310
 
 tss;island
 
 0.003510
 
 0.0013194
 
 Not queried
 
  0.8168385
 
       NA
 
           NA
  
 
  
 5
 
 178487123
 
 178487576
 
  ENSEMBL   UCSC 
 
  454
 
 10
 
 32548
 
 tss
 
 0.045879
 
 0.0716797
 
 Not queried
 
  0.8167692
 
       NA
 
           NA
  
 
  
 14
 
  38064456
 
  38064532
 
  ENSEMBL   UCSC 
 
   77
 
  5
 
 12796
 
 island
 
 0.195116
 
 0.1209598
 
 Not queried
 
  0.8167070
 
       NA
 
           NA
  
 
  
 2
 
  73340007
 
  73340489
 
  ENSEMBL   UCSC 
 
  483
 
  5
 
 24410
 
 island
 
 0.006523
 
 0.0034673
 
 Not queried
 
  0.8166661
 
       NA
 
           NA
  
 
  
 1
 
  38470540
 
  38470665
 
  ENSEMBL   UCSC 
 
  126
 
  4
 
 441
 
 gene;island
 
 0.019735
 
 0.0112116
 
 Not queried
 
  0.8166127
 
       NA
 
           NA
  
 
  
 13
 
  23471764
 
  23471968
 
  ENSEMBL   UCSC 
 
  205
 
  4
 
 11594
 
 island
 
 0.727027
 
 0.8490610
 
 Not queried
 
  0.8165508
 
       NA
 
           NA
  
 
  
 12
 
 124086193
 
 124086670
 
  ENSEMBL   UCSC 
 
  478
 
 11
 
 10103
 
 tss
 
 0.010524
 
 0.0031193
 
 Not queried
 
  0.8164954
 
       NA
 
           NA
  
 
  
 18
 
  74960996
 
  74961494
 
  ENSEMBL   UCSC 
 
  499
 
 14
 
 19330
 
 tss;island
 
 0.029787
 
 0.0402332
 
 Not queried
 
  0.8164733
 
       NA
 
           NA
  
 
  
 15
 
  64443650
 
  64444055
 
  ENSEMBL   UCSC 
 
  406
 
 10
 
 14212
 
 island
 
 0.012911
 
 0.0143366
 
 Not queried
 
  0.8162102
 
       NA
 
           NA
  
 
  
 10
 
  90967113
 
  90967233
 
  ENSEMBL   UCSC 
 
  121
 
  6
 
 5211
 
 tss
 
 0.014478
 
 0.0089048
 
 Not queried
 
  0.8160435
 
       NA
 
           NA
  
 
  
 12
 
  16034932
 
  16035312
 
  ENSEMBL   UCSC 
 
  381
 
  9
 
 10271
 
 island
 
 0.005796
 
 0.0030756
 
 Not queried
 
  0.8159450
 
       NA
 
           NA
  
 
  
 12
 
  56727551
 
  56727976
 
  ENSEMBL   UCSC 
 
  426
 
 12
 
 9796
 
 tss
 
 0.012904
 
 0.0044139
 
 Not queried
 
  0.8156156
 
       NA
 
           NA
  
 
  
 21
 
  40752046
 
  40752891
 
  ENSEMBL   UCSC 
 
  846
 
 10
 
 26683
 
 island
 
 0.023387
 
 0.0120380
 
 Not queried
 
  0.8154746
 
       NA
 
           NA
  
 
  
 20
 
   2276435
 
   2276571
 
  ENSEMBL   UCSC 
 
  137
 
  4
 
 25542
 
 tss
 
 0.393946
 
 0.5302501
 
 Not queried
 
  0.8153454
 
       NA
 
           NA
  
 
  
 2
 
  25016078
 
  25016199
 
  ENSEMBL   UCSC 
 
  122
 
  5
 
 22599
 
 gene
 
 0.004842
 
 0.0090193
 
 Not queried
 
  0.8151507
 
       NA
 
           NA
  
 
  
 7
 
   5821294
 
   5821596
 
  ENSEMBL   UCSC 
 
  303
 
  8
 
 37426
 
 tss
 
 0.007554
 
 0.0071696
 
 Not queried
 
  0.8151051
 
       NA
 
           NA
  
 
  
 8
 
  41504078
 
  41504331
 
  ENSEMBL   UCSC 
 
  254
 
  4
 
 39288
 
 gene;island
 
 0.017362
 
 0.0101309
 
 Not queried
 
  0.8150572
 
       NA
 
           NA
  
 
  
 19
 
  18942465
 
  18942818
 
  ENSEMBL   UCSC 
 
  354
 
  6
 
 21795
 
 island
 
 0.004055
 
 0.0043846
 
 Not queried
 
  0.8148855
 
       NA
 
           NA
  
 
  
 10
 
 134672412
 
 134672917
 
  ENSEMBL   UCSC 
 
  506
 
  8
 
 6314
 
 island
 
 0.869545
 
 0.9292957
 
 Not queried
 
  0.8148638
 
       NA
 
           NA
  
 
  
 7
 
  73894573
 
  73895278
 
  ENSEMBL   UCSC 
 
  706
 
  7
 
 37044
 
 gene;island
 
 0.544547
 
 0.6593751
 
 Not queried
 
  0.8146667
 
       NA
 
           NA
  
 
  
 12
 
 110433832
 
 110434092
 
  ENSEMBL   UCSC 
 
  261
 
  5
 
 9298
 
 gene
 
 0.002580
 
 0.0038905
 
 Not queried
 
  0.8146230
 
       NA
 
           NA
  
 
  
 11
 
  31849061
 
  31849262
 
  ENSEMBL   UCSC 
 
  202
 
  4
 
 8171
 
 island
 
 0.038237
 
 0.0196800
 
 Not queried
 
  0.8146074
 
       NA
 
           NA
  
 
  
 19
 
  37019255
 
  37019614
 
  ENSEMBL   UCSC 
 
  360
 
  8
 
 20726
 
 tss
 
 0.005807
 
 0.0036647
 
 Not queried
 
  0.8145709
 
       NA
 
           NA
  
 
  
 6
 
  30124647
 
  30124942
 
  ENSEMBL   UCSC 
 
  296
 
  7
 
 33732
 
 gene
 
 0.423857
 
 0.7586741
 
 Not queried
 
  0.8144607
 
       NA
 
           NA
  
 
  
 5
 
  82373317
 
  82373672
 
  ENSEMBL   UCSC 
 
  356
 
  7
 
 31669
 
 gene
 
 0.009383
 
 0.0064324
 
 Not queried
 
  0.8144039
 
       NA
 
           NA
  
 
  
 17
 
  25676201
 
  25676574
 
  ENSEMBL   UCSC 
 
  374
 
  4
 
 18134
 
 island
 
 0.085056
 
 0.0421528
 
 Not queried
 
  0.8141034
 
       NA
 
           NA
  
 
  
 17
 
  74449187
 
  74449810
 
  ENSEMBL   UCSC 
 
  624
 
  7
 
 17678
 
 tss;island
 
 0.015225
 
 0.0224467
 
 Not queried
 
  0.8140106
 
       NA
 
           NA
  
 
  
 16
 
   2762569
 
   2762819
 
  ENSEMBL   UCSC 
 
  251
 
  4
 
 14609
 
 gene;island
 
 0.942317
 
 0.9018832
 
 Not queried
 
  0.8136684
 
       NA
 
           NA
  
 
  
 16
 
   1429719
 
   1430367
 
  ENSEMBL   UCSC 
 
  649
 
  8
 
 14993
 
 tss;island
 
 0.306996
 
 0.4213456
 
 Not queried
 
  0.8136414
 
       NA
 
           NA
  
 
  
 14
 
  76819107
 
  76819459
 
  ENSEMBL   UCSC 
 
  353
 
  5
 
 13023
 
 island
 
 0.019972
 
 0.0607356
 
 Not queried
 
  0.8134694
 
       NA
 
           NA
  
 
  
 12
 
 102270723
 
 102270990
 
  ENSEMBL   UCSC 
 
  268
 
  4
 
 9948
 
 tss
 
 0.027646
 
 0.0301253
 
 Not queried
 
  0.8134502
 
       NA
 
           NA
  
 
  
 5
 
 145758586
 
 145758881
 
  ENSEMBL   UCSC 
 
  296
 
  5
 
 33284
 
 island
 
 0.106613
 
 0.5702754
 
 Not queried
 
  0.8134336
 
       NA
 
           NA
  
 
  
 17
 
  73150870
 
  73151149
 
  ENSEMBL   UCSC 
 
  280
 
  5
 
 17645
 
 tss
 
 0.009984
 
 0.0161259
 
 Not queried
 
  0.8132085
 
       NA
 
           NA
  
 
  
 4
 
  89299948
 
  89300321
 
  ENSEMBL   UCSC 
 
  374
 
  4
 
 30054
 
 gene
 
 0.006412
 
 0.0176751
 
 Not queried
 
  0.8131645
 
       NA
 
           NA
  
 
  
 14
 
  47121033
 
  47121158
 
  ENSEMBL   UCSC 
 
  126
 
  5
 
 12375
 
 tss
 
 0.789657
 
 0.9008924
 
 Not queried
 
  0.8130270
 
       NA
 
           NA
  
 
  
 14
 
  47120795
 
  47121158
 
  ENSEMBL   UCSC 
 
  364
 
  9
 
 12817
 
 island
 
 0.789657
 
 0.9008924
 
 Not queried
 
  0.8130270
 
       NA
 
           NA
  
 
  
 4
 
  20255061
 
  20255343
 
  ENSEMBL   UCSC 
 
  283
 
  5
 
 30953
 
 island
 
 0.106136
 
 0.1279462
 
 Not queried
 
  0.8129585
 
       NA
 
           NA
  
 
  
 5
 
 135170000
 
 135170456
 
  ENSEMBL   UCSC 
 
  457
 
  8
 
 33140
 
 island
 
 0.051462
 
 0.0806967
 
 Not queried
 
  0.8129400
 
       NA
 
           NA
  
 
  
 19
 
  48972665
 
  48973001
 
  ENSEMBL   UCSC 
 
  337
 
  4
 
 20127
 
 gene;island
 
 0.007407
 
 0.0072464
 
 Not queried
 
  0.8127151
 
       NA
 
           NA
  
 
  
 19
 
  22816896
 
  22817496
 
  ENSEMBL   UCSC 
 
  601
 
 10
 
 21843
 
 island
 
 0.258572
 
 0.0824663
 
 Not queried
 
  0.8126166
 
       NA
 
           NA
  
 
  
 15
 
  71056115
 
  71056145
 
  ENSEMBL   UCSC 
 
   31
 
  4
 
 13767
 
 tss;island
 
 0.025498
 
 0.0203511
 
 Not queried
 
  0.8124147
 
       NA
 
           NA
  
 
  
 19
 
  15236457
 
  15236813
 
  ENSEMBL   UCSC 
 
  357
 
  7
 
 21695
 
 island
 
 0.007440
 
 0.0017669
 
 Not queried
 
  0.8124100
 
       NA
 
           NA
  
 
  
 7
 
 144533232
 
 144533471
 
  ENSEMBL   UCSC 
 
  240
 
  7
 
 37957
 
 tss
 
 0.015224
 
 0.0087782
 
 Not queried
 
  0.8123229
 
       NA
 
           NA
  
 
  
 6
 
 169902378
 
 169902667
 
  ENSEMBL   UCSC 
 
  290
 
  4
 
 34568
 
 gene
 
 0.613649
 
 0.7979792
 
 Not queried
 
  0.8123221
 
       NA
 
           NA
  
 
  
 10
 
  11783794
 
  11784253
 
  ENSEMBL   UCSC 
 
  460
 
  8
 
 4945
 
 tss
 
 0.040242
 
 0.0370493
 
 Not queried
 
  0.8122820
 
       NA
 
           NA
  
 
  
 3
 
  21447391
 
  21447667
 
  ENSEMBL   UCSC 
 
  277
 
  4
 
 27699
 
 gene
 
 0.718299
 
 0.7361055
 
 Not queried
 
  0.8122365
 
       NA
 
           NA
  
 
  
 5
 
 132200337
 
 132200666
 
  ENSEMBL   UCSC 
 
  330
 
  5
 
 33102
 
 island
 
 0.987492
 
 0.9803759
 
 Not queried
 
  0.8122194
 
       NA
 
           NA
  
 
  
 1
 
 182993172
 
 182993471
 
  ENSEMBL   UCSC 
 
  300
 
  4
 
 913
 
 gene;island
 
 0.010643
 
 0.0071977
 
 Not queried
 
  0.8118005
 
       NA
 
           NA
  
 
  
 7
 
 121512781
 
 121513065
 
  ENSEMBL   UCSC 
 
  285
 
  4
 
 37863
 
 tss;island
 
 0.020964
 
 0.0121706
 
 Not queried
 
  0.8117952
 
       NA
 
           NA
  
 
  
 9
 
  96009796
 
  96010087
 
  ENSEMBL   UCSC 
 
  292
 
  4
 
 40774
 
 gene;island
 
 0.411173
 
 0.6222535
 
 Not queried
 
  0.8117918
 
       NA
 
           NA
  
 
  
 2
 
 172945058
 
 172945383
 
  ENSEMBL   UCSC 
 
  326
 
  5
 
 22921
 
 gene;island
 
 0.033375
 
 0.0274390
 
 Not queried
 
  0.8115953
 
       NA
 
           NA
  
 
  
 4
 
   6247571
 
   6247830
 
  ENSEMBL   UCSC 
 
  260
 
  4
 
 30886
 
 island
 
 0.368350
 
 0.4848532
 
 Not queried
 
  0.8115787
 
       NA
 
           NA
  
 
  
 6
 
  31941361
 
  31941629
 
  ENSEMBL   UCSC 
 
  269
 
  4
 
 34009
 
 gene;island
 
 0.934296
 
 0.9066431
 
 Not queried
 
  0.8115730
 
       NA
 
           NA
  
 
  
 7
 
 155089390
 
 155089803
 
  ENSEMBL   UCSC 
 
  414
 
  6
 
 39060
 
 island
 
 0.004833
 
 0.0034719
 
 Not queried
 
  0.8113886
 
       NA
 
           NA
  
 
  
 7
 
   5085252
 
   5085745
 
  ENSEMBL   UCSC 
 
  494
 
  9
 
 38210
 
 island
 
 0.006345
 
 0.0044912
 
 Not queried
 
  0.8113360
 
       NA
 
           NA
  
 
  
 4
 
   1554380
 
   1554677
 
  ENSEMBL   UCSC 
 
  298
 
  5
 
 30791
 
 island
 
 0.944088
 
 0.9693408
 
 Not queried
 
  0.8113271
 
       NA
 
           NA
  
 
  
 19
 
   4891192
 
   4891471
 
  ENSEMBL   UCSC 
 
  280
 
  5
 
 19741
 
 gene;island
 
 0.660785
 
 0.6707029
 
 Not queried
 
  0.8110026
 
       NA
 
           NA
  
 
  
 11
 
 112831501
 
 112832249
 
  ENSEMBL   UCSC 
 
  749
 
 12
 
 8813
 
 island
 
 0.089872
 
 0.1294634
 
 Not queried
 
  0.8109426
 
       NA
 
           NA
  
 
  
 9
 
  71395063
 
  71395486
 
  ENSEMBL   UCSC 
 
  424
 
  4
 
 40767
 
 gene;island
 
 0.029561
 
 0.0190815
 
 Not queried
 
  0.8109004
 
       NA
 
           NA
  
 
  
 17
 
  54671073
 
  54671575
 
  ENSEMBL   UCSC 
 
  503
 
  5
 
 16702
 
 gene
 
 0.074164
 
 0.0193488
 
 Not queried
 
  0.8108882
 
       NA
 
           NA
  
 
  
 2
 
 120980838
 
 120981005
 
  ENSEMBL   UCSC 
 
  168
 
  4
 
 24662
 
 island
 
 0.004961
 
 0.0052428
 
 Not queried
 
  0.8108880
 
       NA
 
           NA
  
 
  
 20
 
  30326920
 
  30327157
 
  ENSEMBL   UCSC 
 
  238
 
  4
 
 25381
 
 gene
 
 0.000404
 
 0.0022741
 
 Not queried
 
  0.8108488
 
       NA
 
           NA
  
 
  
 4
 
 135865398
 
 135865584
 
  ENSEMBL   UCSC 
 
  187
 
  4
 
 31308
 
 island
 
 0.807457
 
 0.9275422
 
 Not queried
 
  0.8107128
 
       NA
 
           NA
  
 
  
 17
 
    291017
 
    291251
 
  ENSEMBL   UCSC 
 
  235
 
  4
 
 16293
 
 gene;island
 
 0.810733
 
 0.9248133
 
 Not queried
 
  0.8106131
 
       NA
 
           NA
  
 
  
 14
 
  67708177
 
  67708475
 
  ENSEMBL   UCSC 
 
  299
 
  4
 
 12117
 
 gene;island
 
 0.003610
 
 0.0020957
 
 Not queried
 
  0.8105295
 
       NA
 
           NA
  
 
  
 19
 
  37406932
 
  37407486
 
  ENSEMBL   UCSC 
 
  555
 
 14
 
 20732
 
 tss;island
 
 0.017947
 
 0.0101615
 
 Not queried
 
  0.8104610
 
       NA
 
           NA
  
 
  
 12
 
  49110781
 
  49110861
 
  ENSEMBL   UCSC 
 
   81
 
  6
 
 9672
 
 tss
 
 0.006264
 
 0.0037485
 
 Not queried
 
  0.8104304
 
       NA
 
           NA
  
 
  
 6
 
  47277552
 
  47278002
 
  ENSEMBL   UCSC 
 
  451
 
  7
 
 36190
 
 island
 
 0.010544
 
 0.0151130
 
 Not queried
 
  0.8102868
 
       NA
 
           NA
  
 
  
 21
 
  35831954
 
  35832364
 
  ENSEMBL   UCSC 
 
  411
 
  9
 
 26529
 
 tss
 
 0.564433
 
 0.6014103
 
 Not queried
 
  0.8098828
 
       NA
 
           NA
  
 
  
 12
 
  21654197
 
  21654897
 
  ENSEMBL   UCSC 
 
  701
 
 12
 
 9599
 
 tss;island
 
 0.005931
 
 0.0063609
 
 Not queried
 
  0.8092984
 
       NA
 
           NA
  
 
  
 22
 
  38479181
 
  38479332
 
  ENSEMBL   UCSC 
 
  152
 
  6
 
 27130
 
 tss
 
 0.820811
 
 0.9094991
 
 Not queried
 
  0.8092357
 
       NA
 
           NA
  
 
  
 1
 
 249131916
 
 249132477
 
  ENSEMBL   UCSC 
 
  562
 
  9
 
 2375
 
 tss;island
 
 0.005577
 
 0.0019084
 
 Not queried
 
  0.8091929
 
       NA
 
           NA
  
 
  
 15
 
  52311035
 
  52311380
 
  ENSEMBL   UCSC 
 
  346
 
  4
 
 13676
 
 tss
 
 0.012476
 
 0.0435118
 
 Not queried
 
  0.8091845
 
       NA
 
           NA
  
 
  
 5
 
 178487123
 
 178488100
 
  ENSEMBL   UCSC 
 
  978
 
 14
 
 33479
 
 island
 
 0.058513
 
 0.0988135
 
 Not queried
 
  0.8089879
 
       NA
 
           NA
  
 
  
 2
 
  80531483
 
  80531898
 
  ENSEMBL   UCSC 
 
  416
 
 10
 
 22756
 
 gene;island
 
 0.018894
 
 0.0333001
 
 Not queried
 
  0.8089637
 
       NA
 
           NA
  
 
  
 10
 
  71333152
 
  71333216
 
  ENSEMBL   UCSC 
 
   65
 
  5
 
 5126
 
 tss
 
 0.049001
 
 0.1183571
 
 Not queried
 
  0.8088253
 
       NA
 
           NA
  
 
  
 12
 
  56615532
 
  56615727
 
  ENSEMBL   UCSC 
 
  196
 
  5
 
 9175
 
 gene
 
 0.004555
 
 0.0024467
 
 Not queried
 
  0.8087519
 
       NA
 
           NA
  
 
  
 19
 
  48219924
 
  48220334
 
  ENSEMBL   UCSC 
 
  411
 
  4
 
 20119
 
 gene;island
 
 0.797460
 
 0.8736350
 
 Not queried
 
  0.8086363
 
       NA
 
           NA
  
 
  
 17
 
  26646373
 
  26646651
 
  ENSEMBL   UCSC 
 
  279
 
  4
 
 16493
 
 gene;island
 
 0.009653
 
 0.0068714
 
 Not queried
 
  0.8084920
 
       NA
 
           NA
  
 
  
 17
 
  37355961
 
  37356047
 
  ENSEMBL   UCSC 
 
   87
 
  4
 
 17305
 
 tss;island
 
 0.016591
 
 0.0366799
 
 Not queried
 
  0.8083093
 
       NA
 
           NA
  
 
  
 12
 
  66583029
 
  66583431
 
  ENSEMBL   UCSC 
 
  403
 
  5
 
 9207
 
 gene;island
 
 0.017740
 
 0.0058112
 
 Not queried
 
  0.8079614
 
       NA
 
           NA
  
 
  
 7
 
  37960731
 
  37960974
 
  ENSEMBL   UCSC 
 
  244
 
  4
 
 36970
 
 gene;island
 
 0.018770
 
 0.0166960
 
 Not queried
 
  0.8078588
 
       NA
 
           NA
  
 
  
 4
 
  54959419
 
  54960259
 
  ENSEMBL   UCSC 
 
  841
 
  8
 
 31050
 
 island
 
 0.050057
 
 0.0923279
 
 Not queried
 
  0.8078293
 
       NA
 
           NA
  
 
  
 14
 
  23815798
 
  23816109
 
  ENSEMBL   UCSC 
 
  312
 
  5
 
 12028
 
 gene;island
 
 0.373064
 
 0.5101534
 
 Not queried
 
  0.8075786
 
       NA
 
           NA
  
 
  
 17
 
    259755
 
    260058
 
  ENSEMBL   UCSC 
 
  304
 
  9
 
 16979
 
 tss;island
 
 0.007745
 
 0.0049928
 
 Not queried
 
  0.8073501
 
       NA
 
           NA
  
 
  
 4
 
   6271485
 
   6271557
 
  ENSEMBL   UCSC 
 
   73
 
  4
 
 30279
 
 tss
 
 0.005260
 
 0.0093391
 
 Not queried
 
  0.8073142
 
       NA
 
           NA
  
 
  
 13
 
 100643858
 
 100644657
 
  ENSEMBL   UCSC 
 
  800
 
  8
 
 11850
 
 island
 
 0.065661
 
 0.0428508
 
 Not queried
 
  0.8070425
 
       NA
 
           NA
  
 
  
 1
 
 160312929
 
 160313258
 
  ENSEMBL   UCSC 
 
  330
 
  9
 
 843
 
 gene;island
 
 0.006132
 
 0.0042118
 
 Not queried
 
  0.8070041
 
       NA
 
           NA
  
 
  
 19
 
  38810162
 
  38810437
 
  ENSEMBL   UCSC 
 
  276
 
  8
 
 20751
 
 tss
 
 0.011742
 
 0.0558745
 
 Not queried
 
  0.8068994
 
       NA
 
           NA
  
 
  
 16
 
  88355788
 
  88356046
 
  ENSEMBL   UCSC 
 
  259
 
  4
 
 16198
 
 island
 
 0.608799
 
 0.6978611
 
 Not queried
 
  0.8068990
 
       NA
 
           NA
  
 
  
 19
 
    855899
 
    856145
 
  ENSEMBL   UCSC 
 
  247
 
  4
 
 19634
 
 gene;island
 
 0.294475
 
 0.4311224
 
 Not queried
 
  0.8064550
 
       NA
 
           NA
  
 
  
 15
 
  31372742
 
  31373178
 
  ENSEMBL   UCSC 
 
  437
 
  4
 
 13272
 
 gene
 
 0.498581
 
 0.3806181
 
 Not queried
 
  0.8063841
 
       NA
 
           NA
  
 
  
 6
 
  25726884
 
  25727291
 
  ENSEMBL   UCSC 
 
  408
 
  9
 
 34675
 
 tss;island
 
 0.841309
 
 0.9517934
 
 Not queried
 
  0.8063018
 
       NA
 
           NA
  
 
  
 11
 
   2156282
 
   2156699
 
  ENSEMBL   UCSC 
 
  418
 
  6
 
 6446
 
 gene;tss;island
 
 0.619607
 
 0.7299187
 
 Not queried
 
  0.8061619
 
       NA
 
           NA
  
 
  
 5
 
   5139334
 
   5140029
 
  ENSEMBL   UCSC 
 
  696
 
 16
 
 31979
 
 tss;island
 
 0.169617
 
 0.0603692
 
 Not queried
 
  0.8061442
 
       NA
 
           NA
  
 
  
 17
 
  77924180
 
  77924371
 
  ENSEMBL   UCSC 
 
  192
 
  4
 
 16846
 
 gene;island
 
 0.856735
 
 0.9278157
 
 Not queried
 
  0.8060812
 
       NA
 
           NA
  
 
  
 2
 
  24272451
 
  24272547
 
  ENSEMBL   UCSC 
 
   97
 
  6
 
 23222
 
 tss
 
 0.004224
 
 0.0065724
 
 Not queried
 
  0.8059955
 
       NA
 
           NA
  
 
  
 8
 
 145691582
 
 145691776
 
  ENSEMBL   UCSC 
 
  195
 
  4
 
 39985
 
 tss;island
 
 0.002468
 
 0.0019523
 
 Not queried
 
  0.8056138
 
       NA
 
           NA
  
 
  
 4
 
    619162
 
    619392
 
  ENSEMBL   UCSC 
 
  231
 
  6
 
 30723
 
 island
 
 0.411456
 
 0.4527222
 
 Not queried
 
  0.8055606
 
       NA
 
           NA
  
 
  
 6
 
  74233269
 
  74233640
 
  ENSEMBL   UCSC 
 
  372
 
  4
 
 36260
 
 island
 
 0.015608
 
 0.0114840
 
 Not queried
 
  0.8055582
 
       NA
 
           NA
  
 
  
 22
 
  19435790
 
  19436004
 
  ENSEMBL   UCSC 
 
  215
 
  4
 
 26958
 
 tss;island
 
 0.070728
 
 0.0661282
 
 Not queried
 
  0.8054253
 
       NA
 
           NA
  
 
  
 6
 
  29576818
 
  29577348
 
  ENSEMBL   UCSC 
 
  531
 
  6
 
 33668
 
 gene
 
 0.354503
 
 0.5000424
 
 Not queried
 
  0.8052781
 
       NA
 
           NA
  
 
  
 17
 
   7905636
 
   7906098
 
  ENSEMBL   UCSC 
 
  463
 
  6
 
 17981
 
 island
 
 0.011435
 
 0.0250470
 
 Not queried
 
  0.8052235
 
       NA
 
           NA
  
 
  
 6
 
  31146124
 
  31146583
 
  ENSEMBL   UCSC 
 
  460
 
  5
 
 34802
 
 tss;island
 
 0.020200
 
 0.0169224
 
 Not queried
 
  0.8051350
 
       NA
 
           NA
  
 
  
 17
 
  79895003
 
  79895100
 
  ENSEMBL   UCSC 
 
   98
 
  6
 
 17760
 
 tss;island
 
 0.006241
 
 0.0041230
 
 Not queried
 
  0.8050840
 
       NA
 
           NA
  
 
  
 6
 
  37617864
 
  37618123
 
  ENSEMBL   UCSC 
 
  260
 
  4
 
 34324
 
 gene;island
 
 0.559036
 
 0.5314043
 
 Not queried
 
  0.8050818
 
       NA
 
           NA
  
 
  
 19
 
  11689488
 
  11689726
 
  ENSEMBL   UCSC 
 
  239
 
  6
 
 19846
 
 gene
 
 0.042932
 
 0.0975136
 
 Not queried
 
  0.8048349
 
       NA
 
           NA
  
 
  
 19
 
  11689488
 
  11689997
 
  ENSEMBL   UCSC 
 
  510
 
 12
 
 20477
 
 tss;island
 
 0.034197
 
 0.0686495
 
 Not queried
 
  0.8048349
 
       NA
 
           NA
  
 
  
 20
 
  25604462
 
  25604805
 
  ENSEMBL   UCSC 
 
  344
 
  9
 
 26065
 
 island
 
 0.033554
 
 0.0247718
 
 Not queried
 
  0.8047284
 
       NA
 
           NA
  
 
  
 5
 
 110408740
 
 110409196
 
  ENSEMBL   UCSC 
 
  457
 
  8
 
 31708
 
 gene;island
 
 0.013267
 
 0.0258805
 
 Not queried
 
  0.8042259
 
       NA
 
           NA
  
 
  
 2
 
 216946201
 
 216946473
 
  ENSEMBL   UCSC 
 
  273
 
  6
 
 23008
 
 gene
 
 0.008372
 
 0.0054575
 
 Not queried
 
  0.8041918
 
       NA
 
           NA
  
 
  
 16
 
    875257
 
    875626
 
  ENSEMBL   UCSC 
 
  370
 
  4
 
 15491
 
 island
 
 0.571295
 
 0.6883384
 
 Not queried
 
  0.8040708
 
       NA
 
           NA
  
 
  
 5
 
  72595112
 
  72595958
 
  ENSEMBL   UCSC 
 
  847
 
  9
 
 32869
 
 island
 
 0.023866
 
 0.0295217
 
 Not queried
 
  0.8040359
 
       NA
 
           NA
  
 
  
 5
 
 112824497
 
 112824920
 
  ENSEMBL   UCSC 
 
  424
 
  7
 
 33013
 
 island
 
 0.454902
 
 0.5215176
 
 Not queried
 
  0.8038251
 
       NA
 
           NA
  
 
  
 15
 
  75017914
 
  75018266
 
  ENSEMBL   UCSC 
 
  353
 
  8
 
 13798
 
 tss;island
 
 0.014936
 
 0.0125502
 
 Not queried
 
  0.8038119
 
       NA
 
           NA
  
 
  
 21
 
  44486205
 
  44486452
 
  ENSEMBL   UCSC 
 
  248
 
  4
 
 26442
 
 gene;island
 
 0.931952
 
 0.9652177
 
 Not queried
 
  0.8037245
 
       NA
 
           NA
  
 
  
 16
 
   3062597
 
   3062975
 
  ENSEMBL   UCSC 
 
  379
 
  4
 
 14619
 
 gene
 
 0.670804
 
 0.4818352
 
 Not queried
 
  0.8036782
 
       NA
 
           NA
  
 
  
 3
 
 126737201
 
 126737358
 
  ENSEMBL   UCSC 
 
  158
 
  4
 
 27930
 
 gene;island
 
 0.897756
 
 0.9334439
 
 Not queried
 
  0.8036604
 
       NA
 
           NA
  
 
  
 10
 
  92617429
 
  92618141
 
  ENSEMBL   UCSC 
 
  713
 
  7
 
 5928
 
 island
 
 0.040193
 
 0.0341917
 
 Not queried
 
  0.8036470
 
       NA
 
           NA
  
 
  
 19
 
   3985464
 
   3985688
 
  ENSEMBL   UCSC 
 
  225
 
  8
 
 20357
 
 tss
 
 0.007661
 
 0.0039377
 
 Not queried
 
  0.8035466
 
       NA
 
           NA
  
 
  
 19
 
   3985414
 
   3985688
 
  ENSEMBL   UCSC 
 
  275
 
 10
 
 21360
 
 island
 
 0.009634
 
 0.0059625
 
 Not queried
 
  0.8035466
 
       NA
 
           NA
  
 
  
 16
 
  30798540
 
  30798776
 
  ENSEMBL   UCSC 
 
  237
 
  5
 
 15195
 
 tss
 
 0.024726
 
 0.0421340
 
 Not queried
 
  0.8035015
 
       NA
 
           NA
  
 
  
 16
 
  20996560
 
  20996704
 
  ENSEMBL   UCSC 
 
  145
 
  4
 
 14686
 
 gene
 
 0.803967
 
 0.8939388
 
 Not queried
 
  0.8032187
 
       NA
 
           NA
  
 
  
 8
 
 145848921
 
 145849607
 
  ENSEMBL   UCSC 
 
  687
 
  8
 
 40744
 
 island
 
 0.650195
 
 0.6754419
 
 Not queried
 
  0.8031521
 
       NA
 
           NA
  
 
  
 18
 
  56886915
 
  56887410
 
  ENSEMBL   UCSC 
 
  496
 
 11
 
 19543
 
 island
 
 0.010311
 
 0.0632919
 
 Not queried
 
  0.8031399
 
       NA
 
           NA
  
 
  
 11
 
  60681193
 
  60681305
 
  ENSEMBL   UCSC 
 
  113
 
  6
 
 7325
 
 tss;island
 
 0.001416
 
 0.0007753
 
 Not queried
 
  0.8027789
 
       NA
 
           NA
  
 
  
 12
 
 123450766
 
 123451323
 
  ENSEMBL   UCSC 
 
  558
 
 11
 
 9376
 
 gene;island
 
 0.038239
 
 0.0120820
 
 Not queried
 
  0.8027391
 
       NA
 
           NA
  
 
  
 12
 
 123451056
 
 123451323
 
  ENSEMBL   UCSC 
 
  268
 
  7
 
 10092
 
 tss
 
 0.055234
 
 0.0216790
 
 Not queried
 
  0.8027391
 
       NA
 
           NA
  
 
  
 2
 
 119916486
 
 119916686
 
  ENSEMBL   UCSC 
 
  201
 
  6
 
 23591
 
 tss
 
 0.024017
 
 0.0637827
 
 Not queried
 
  0.8027199
 
       NA
 
           NA
  
 
  
 19
 
   9434309
 
   9434924
 
  ENSEMBL   UCSC 
 
  616
 
 10
 
 20422
 
 tss
 
 0.031564
 
 0.0225732
 
 Not queried
 
  0.8026937
 
       NA
 
           NA
  
 
  
 4
 
  56212072
 
  56212371
 
  ENSEMBL   UCSC 
 
  300
 
  7
 
 30403
 
 tss;island
 
 0.006309
 
 0.0035556
 
 Not queried
 
  0.8026141
 
       NA
 
           NA
  
 
  
 17
 
  45973408
 
  45973469
 
  ENSEMBL   UCSC 
 
   62
 
  5
 
 17447
 
 tss
 
 0.004145
 
 0.0084350
 
 Not queried
 
  0.8026087
 
       NA
 
           NA
  
 
  
 21
 
  43431083
 
  43431529
 
  ENSEMBL   UCSC 
 
  447
 
  4
 
 26562
 
 tss;island
 
 0.029460
 
 0.0202300
 
 Not queried
 
  0.8023423
 
       NA
 
           NA
  
 
  
 5
 
 172044853
 
 172045075
 
  ENSEMBL   UCSC 
 
  223
 
  4
 
 33392
 
 island
 
 0.476001
 
 0.6140239
 
 Not queried
 
  0.8021686
 
       NA
 
           NA
  
 
  
 5
 
   1218507
 
   1218653
 
  ENSEMBL   UCSC 
 
  147
 
  4
 
 31527
 
 gene;island
 
 0.820094
 
 0.9129066
 
 Not queried
 
  0.8020976
 
       NA
 
           NA
  
 
  
 10
 
 131479182
 
 131479437
 
  ENSEMBL   UCSC 
 
  256
 
  4
 
 4829
 
 gene
 
 0.728615
 
 0.8068679
 
 Not queried
 
  0.8020226
 
       NA
 
           NA
  
 
  
 1
 
  99469577
 
  99469819
 
  ENSEMBL   UCSC 
 
  243
 
  4
 
 647
 
 gene;island
 
 0.052894
 
 0.2437935
 
 Not queried
 
  0.8020223
 
       NA
 
           NA
  
 
  
 6
 
  33244976
 
  33246488
 
  ENSEMBL   UCSC 
 
 1513
 
 45
 
 34246
 
 gene
 
 0.083871
 
 0.1796398
 
 Not queried
 
  0.8018860
 
       NA
 
           NA
  
 
  
 4
 
 176987009
 
 176987577
 
  ENSEMBL   UCSC 
 
  569
 
  7
 
 30186
 
 gene
 
 0.011095
 
 0.0045679
 
 Not queried
 
  0.8018333
 
       NA
 
           NA
  
 
  
 17
 
  43922122
 
  43922244
 
  ENSEMBL   UCSC 
 
  123
 
  4
 
 17432
 
 tss
 
 0.461279
 
 0.5560319
 
 Not queried
 
  0.8016451
 
       NA
 
           NA
  
 
  
 3
 
  49727025
 
  49727474
 
  ENSEMBL   UCSC 
 
  450
 
  5
 
 27787
 
 gene
 
 0.017253
 
 0.0120970
 
 Not queried
 
  0.8016041
 
       NA
 
           NA
  
 
  
 8
 
  66546482
 
  66546718
 
  ENSEMBL   UCSC 
 
  237
 
  6
 
 39738
 
 tss
 
 0.002978
 
 0.0009740
 
 Not queried
 
  0.8015663
 
       NA
 
           NA
  
 
  
 5
 
   1212434
 
   1212641
 
  ENSEMBL   UCSC 
 
  208
 
  5
 
 31526
 
 gene;island
 
 0.867343
 
 0.9350850
 
 Not queried
 
  0.8013842
 
       NA
 
           NA
  
 
  
 9
 
  99801371
 
  99801723
 
  ENSEMBL   UCSC 
 
  353
 
  4
 
 40919
 
 island
 
 0.049967
 
 0.0282993
 
 Not queried
 
  0.8013812
 
       NA
 
           NA
  
 
  
 2
 
 180725637
 
 180726035
 
  ENSEMBL   UCSC 
 
  399
 
  6
 
 23758
 
 tss
 
 0.028717
 
 0.0226956
 
 Not queried
 
  0.8013053
 
       NA
 
           NA
  
 
  
 17
 
  42219409
 
  42219681
 
  ENSEMBL   UCSC 
 
  273
 
  4
 
 16606
 
 gene
 
 0.005783
 
 0.0092778
 
 Not queried
 
  0.8012992
 
       NA
 
           NA
  
 
  
 1
 
  67217673
 
  67218135
 
  ENSEMBL   UCSC 
 
  463
 
 10
 
 1680
 
 tss
 
 0.056612
 
 0.0417128
 
 Not queried
 
  0.8012957
 
       NA
 
           NA
  
 
  
 17
 
  27717641
 
  27718019
 
  ENSEMBL   UCSC 
 
  379
 
  9
 
 18168
 
 island
 
 0.002447
 
 0.0031016
 
 Not queried
 
  0.8010893
 
       NA
 
           NA
  
 
  
 3
 
 169490563
 
 169490789
 
  ENSEMBL   UCSC 
 
  227
 
  5
 
 28708
 
 tss
 
 0.008209
 
 0.0049373
 
 Not queried
 
  0.8010597
 
       NA
 
           NA
  
 
  
 3
 
 169490563
 
 169491284
 
  ENSEMBL   UCSC 
 
  722
 
  9
 
 29649
 
 island
 
 0.008246
 
 0.0071103
 
 Not queried
 
  0.8010597
 
       NA
 
           NA
  
 
  
 1
 
  27816678
 
  27816827
 
  ENSEMBL   UCSC 
 
  150
 
  5
 
 1413
 
 tss
 
 0.006749
 
 0.0059940
 
 Not queried
 
  0.8009782
 
       NA
 
           NA
  
 
  
 4
 
  83206108
 
  83206604
 
  ENSEMBL   UCSC 
 
  497
 
  5
 
 31141
 
 island
 
 0.007270
 
 0.0090055
 
 Not queried
 
  0.8007995
 
       NA
 
           NA
  
 
  
 7
 
  75115567
 
  75115859
 
  ENSEMBL   UCSC 
 
  293
 
  9
 
 38584
 
 island
 
 0.004066
 
 0.0035793
 
 Not queried
 
  0.8007859
 
       NA
 
           NA
  
 
  
 2
 
 237072673
 
 237072998
 
  ENSEMBL   UCSC 
 
  326
 
  4
 
 25223
 
 island
 
 0.069781
 
 0.0566262
 
 Not queried
 
  0.8006912
 
       NA
 
           NA
  
 
  
 3
 
 194354240
 
 194354433
 
  ENSEMBL   UCSC 
 
  194
 
  5
 
 28800
 
 tss;island
 
 0.003309
 
 0.0097382
 
 Not queried
 
  0.8002626
 
       NA
 
           NA
  
 
  
 19
 
  57742394
 
  57742444
 
  ENSEMBL   UCSC 
 
   51
 
  4
 
 20245
 
 gene
 
 0.971767
 
 0.9493066
 
 Not queried
 
  0.8001900
 
       NA
 
           NA
  
 
  
 1
 
 106623429
 
 106623967
 
  ENSEMBL   UCSC 
 
  539
 
  6
 
 3535
 
 island
 
 0.506624
 
 0.3770759
 
 Not queried
 
  0.8001741
 
       NA
 
           NA
  
 
  
 1
 
  26632948
 
  26633632
 
  ENSEMBL   UCSC 
 
  685
 
 14
 
 339
 
 gene;island
 
 0.003977
 
 0.0069572
 
 Not queried
 
  0.7999287
 
       NA
 
           NA
  
 
  
 6
 
  89855751
 
  89855994
 
  ENSEMBL   UCSC 
 
  244
 
  5
 
 36320
 
 island
 
 0.015089
 
 0.0120175
 
 Not queried
 
  0.7998324
 
       NA
 
           NA
  
 
  
 19
 
  42463610
 
  42463824
 
  ENSEMBL   UCSC 
 
  215
 
  5
 
 20817
 
 tss
 
 0.009359
 
 0.0055088
 
 Not queried
 
  0.7997970
 
       NA
 
           NA
  
 
  
 19
 
  42463363
 
  42463824
 
  ENSEMBL   UCSC 
 
  462
 
  7
 
 22062
 
 island
 
 0.009359
 
 0.0048272
 
 Not queried
 
  0.7997970
 
       NA
 
           NA
  
 
  
 21
 
  15755931
 
  15756122
 
  ENSEMBL   UCSC 
 
  192
 
  5
 
 26489
 
 tss;island
 
 0.001643
 
 0.0005242
 
 Not queried
 
  0.7997869
 
       NA
 
           NA
  
 
  
 6
 
  29425885
 
  29426132
 
  ENSEMBL   UCSC 
 
  248
 
  5
 
 34747
 
 tss
 
 0.931577
 
 0.9367680
 
 Not queried
 
  0.7995654
 
       NA
 
           NA
  
 
  
 7
 
  26438234
 
  26438411
 
  ENSEMBL   UCSC 
 
  178
 
  5
 
 38332
 
 island
 
 0.003307
 
 0.0017450
 
 Not queried
 
  0.7994916
 
       NA
 
           NA
  
 
  
 2
 
 128458240
 
 128458556
 
  ENSEMBL   UCSC 
 
  317
 
  6
 
 23616
 
 tss
 
 0.001628
 
 0.0033495
 
 Not queried
 
  0.7993179
 
       NA
 
           NA
  
 
  
 4
 
  20701575
 
  20701990
 
  ENSEMBL   UCSC 
 
  416
 
  8
 
 30322
 
 tss
 
 0.010030
 
 0.0050990
 
 Not queried
 
  0.7992724
 
       NA
 
           NA
  
 
  
 14
 
  23340566
 
  23340939
 
  ENSEMBL   UCSC 
 
  374
 
  5
 
 12289
 
 tss
 
 0.012719
 
 0.0031523
 
 Not queried
 
  0.7992567
 
       NA
 
           NA
  
 
  
 16
 
  88569083
 
  88569490
 
  ENSEMBL   UCSC 
 
  408
 
  5
 
 14906
 
 gene;island
 
 0.927114
 
 0.9259401
 
 Not queried
 
  0.7991702
 
       NA
 
           NA
  
 
  
 4
 
  84406319
 
  84406624
 
  ENSEMBL   UCSC 
 
  306
 
  6
 
 30476
 
 tss
 
 0.008188
 
 0.0071121
 
 Not queried
 
  0.7991598
 
       NA
 
           NA
  
 
  
 16
 
   8961914
 
   8962224
 
  ENSEMBL   UCSC 
 
  311
 
  4
 
 14654
 
 gene;island
 
 0.008714
 
 0.0068458
 
 Not queried
 
  0.7989423
 
       NA
 
           NA
  
 
  
 16
 
  64984626
 
  64984977
 
  ENSEMBL   UCSC 
 
  352
 
  5
 
 14777
 
 gene;island
 
 0.676359
 
 0.7058640
 
 Not queried
 
  0.7988163
 
       NA
 
           NA
  
 
  
 1
 
 119526783
 
 119527156
 
  ENSEMBL   UCSC 
 
  374
 
  5
 
 715
 
 gene;island
 
 0.083045
 
 0.0776109
 
 Not queried
 
  0.7987629
 
       NA
 
           NA
  
 
  
 17
 
  47653018
 
  47653309
 
  ENSEMBL   UCSC 
 
  292
 
  5
 
 18528
 
 island
 
 0.030357
 
 0.0133445
 
 Not queried
 
  0.7986719
 
       NA
 
           NA
  
 
  
 19
 
  47290532
 
  47290716
 
  ENSEMBL   UCSC 
 
  185
 
  5
 
 20115
 
 gene;island
 
 0.017394
 
 0.0056088
 
 Not queried
 
  0.7986001
 
       NA
 
           NA
  
 
  
 6
 
  48036584
 
  48036676
 
  ENSEMBL   UCSC 
 
   93
 
  7
 
 35025
 
 tss
 
 0.172881
 
 0.3465083
 
 Not queried
 
  0.7985246
 
       NA
 
           NA
  
 
  
 5
 
 127874463
 
 127874587
 
  ENSEMBL   UCSC 
 
  125
 
  4
 
 32259
 
 tss;island
 
 0.019171
 
 0.0213775
 
 Not queried
 
  0.7985011
 
       NA
 
           NA
  
 
  
 2
 
 219187654
 
 219188197
 
  ENSEMBL   UCSC 
 
  544
 
  4
 
 23015
 
 gene;island
 
 0.009109
 
 0.0149107
 
 Not queried
 
  0.7984782
 
       NA
 
           NA
  
 
  
 5
 
  94620929
 
  94621055
 
  ENSEMBL   UCSC 
 
  127
 
  4
 
 32182
 
 tss;island
 
 0.050540
 
 0.0294700
 
 Not queried
 
  0.7981813
 
       NA
 
           NA
  
 
  
 1
 
  25869837
 
  25870291
 
  ENSEMBL   UCSC 
 
  455
 
  9
 
 2873
 
 island
 
 0.008847
 
 0.0681090
 
 Not queried
 
  0.7978998
 
       NA
 
           NA
  
 
  
 1
 
  87379771
 
  87379926
 
  ENSEMBL   UCSC 
 
  156
 
  4
 
 610
 
 gene;tss;island
 
 0.004706
 
 0.0047688
 
 Not queried
 
  0.7978086
 
       NA
 
           NA
  
 
  
 11
 
  34195914
 
  34196316
 
  ENSEMBL   UCSC 
 
  403
 
  7
 
 6580
 
 gene;island
 
 0.009785
 
 0.0059186
 
 Not queried
 
  0.7978007
 
       NA
 
           NA
  
 
  
 20
 
  25062771
 
  25063052
 
  ENSEMBL   UCSC 
 
  282
 
  7
 
 25645
 
 tss
 
 0.046220
 
 0.1336864
 
 Not queried
 
  0.7977807
 
       NA
 
           NA
  
 
  
 6
 
  10390811
 
  10391412
 
  ENSEMBL   UCSC 
 
  602
 
  6
 
 35424
 
 island
 
 0.095787
 
 0.2912850
 
 Not queried
 
  0.7977048
 
       NA
 
           NA
  
 
  
 17
 
  58218643
 
  58218965
 
  ENSEMBL   UCSC 
 
  323
 
  4
 
 18619
 
 island
 
 0.063538
 
 0.1142034
 
 Not queried
 
  0.7976189
 
       NA
 
           NA
  
 
  
 17
 
  55938748
 
  55939089
 
  ENSEMBL   UCSC 
 
  342
 
  4
 
 18587
 
 island
 
 0.011852
 
 0.0220033
 
 Not queried
 
  0.7975320
 
       NA
 
           NA
  
 
  
 22
 
  24199851
 
  24200019
 
  ENSEMBL   UCSC 
 
  169
 
  5
 
 27013
 
 tss
 
 0.008570
 
 0.0070366
 
 Not queried
 
  0.7972883
 
       NA
 
           NA
  
 
  
 2
 
  38829956
 
  38830276
 
  ENSEMBL   UCSC 
 
  321
 
  4
 
 24231
 
 island
 
 0.011614
 
 0.0083953
 
 Not queried
 
  0.7971877
 
       NA
 
           NA
  
 
  
 16
 
  78133117
 
  78133395
 
  ENSEMBL   UCSC 
 
  279
 
  4
 
 15372
 
 tss;island
 
 0.004526
 
 0.0026714
 
 Not queried
 
  0.7971465
 
       NA
 
           NA
  
 
  
 11
 
 134122771
 
 134122914
 
  ENSEMBL   UCSC 
 
  144
 
  4
 
 6992
 
 gene;tss;island
 
 0.012028
 
 0.0090841
 
 Not queried
 
  0.7969358
 
       NA
 
           NA
  
 
  
 7
 
  22539703
 
  22539822
 
  ENSEMBL   UCSC 
 
  120
 
  4
 
 36912
 
 gene
 
 0.026320
 
 0.0442699
 
 Not queried
 
  0.7969228
 
       NA
 
           NA
  
 
  
 12
 
 110841169
 
 110841516
 
  ENSEMBL   UCSC 
 
  348
 
  4
 
 9300
 
 gene
 
 0.008977
 
 0.0069333
 
 Not queried
 
  0.7969187
 
       NA
 
           NA
  
 
  
 19
 
   8408048
 
   8408509
 
  ENSEMBL   UCSC 
 
  462
 
 11
 
 21481
 
 island
 
 0.046766
 
 0.0187760
 
 Not queried
 
  0.7969111
 
       NA
 
           NA
  
 
  
 22
 
  31608022
 
  31608245
 
  ENSEMBL   UCSC 
 
  224
 
  4
 
 27077
 
 tss
 
 0.010530
 
 0.0159235
 
 Not queried
 
  0.7967465
 
       NA
 
           NA
  
 
  
 1
 
  35545014
 
  35545352
 
  ENSEMBL   UCSC 
 
  339
 
  7
 
 419
 
 gene
 
 0.002451
 
 0.0057732
 
 Not queried
 
  0.7967231
 
       NA
 
           NA
  
 
  
 1
 
  91172710
 
  91173030
 
  ENSEMBL   UCSC 
 
  321
 
  5
 
 3455
 
 island
 
 0.009830
 
 0.0143626
 
 Not queried
 
  0.7966999
 
       NA
 
           NA
  
 
  
 19
 
  58570419
 
  58570491
 
  ENSEMBL   UCSC 
 
   73
 
  6
 
 21132
 
 tss;island
 
 0.125436
 
 0.0542803
 
 Not queried
 
  0.7966354
 
       NA
 
           NA
  
 
  
 11
 
   8190659
 
   8190837
 
  ENSEMBL   UCSC 
 
  179
 
  6
 
 7124
 
 tss
 
 0.136707
 
 0.0800583
 
 Not queried
 
  0.7965038
 
       NA
 
           NA
  
 
  
 1
 
 114697352
 
 114697707
 
  ENSEMBL   UCSC 
 
  356
 
  4
 
 1849
 
 tss;island
 
 0.194068
 
 0.3727288
 
 Not queried
 
  0.7964507
 
       NA
 
           NA
  
 
  
 3
 
 184104362
 
 184104688
 
  ENSEMBL   UCSC 
 
  327
 
  5
 
 28063
 
 gene;island
 
 0.572150
 
 0.6988137
 
 Not queried
 
  0.7963833
 
       NA
 
           NA
  
 
  
 6
 
 119256092
 
 119256466
 
  ENSEMBL   UCSC 
 
  375
 
  5
 
 36445
 
 island
 
 0.009610
 
 0.0056302
 
 Not queried
 
  0.7963238
 
       NA
 
           NA
  
 
  
 2
 
 121735681
 
 121736138
 
  ENSEMBL   UCSC 
 
  458
 
  6
 
 22850
 
 gene
 
 0.437676
 
 0.5795022
 
 Not queried
 
  0.7960253
 
       NA
 
           NA
  
 
  
 21
 
  44312851
 
  44313047
 
  ENSEMBL   UCSC 
 
  197
 
  4
 
 26571
 
 tss;island
 
 0.041332
 
 0.0231055
 
 Not queried
 
  0.7957728
 
       NA
 
           NA
  
 
  
 12
 
 113573143
 
 113574368
 
  ENSEMBL   UCSC 
 
 1226
 
 17
 
 10826
 
 island
 
 0.023354
 
 0.0157115
 
 Not queried
 
  0.7957380
 
       NA
 
           NA
  
 
  
 15
 
  75660780
 
  75661354
 
  ENSEMBL   UCSC 
 
  575
 
 10
 
 14329
 
 island
 
 0.010732
 
 0.0061454
 
 Not queried
 
  0.7952586
 
       NA
 
           NA
  
 
  
 3
 
 125239063
 
 125239413
 
  ENSEMBL   UCSC 
 
  351
 
  8
 
 28552
 
 tss
 
 0.005162
 
 0.0068935
 
 Not queried
 
  0.7950446
 
       NA
 
           NA
  
 
  
 7
 
   4030274
 
   4030736
 
  ENSEMBL   UCSC 
 
  463
 
  5
 
 36824
 
 gene
 
 0.369629
 
 0.5462377
 
 Not queried
 
  0.7950042
 
       NA
 
           NA
  
 
  
 16
 
  69965465
 
  69965788
 
  ENSEMBL   UCSC 
 
  324
 
  4
 
 14824
 
 gene
 
 0.949453
 
 0.9579313
 
 Not queried
 
  0.7949777
 
       NA
 
           NA
  
 
  
 20
 
  61493177
 
  61493341
 
  ENSEMBL   UCSC 
 
  165
 
  9
 
 25883
 
 tss;island
 
 0.004304
 
 0.0026000
 
 Not queried
 
  0.7948856
 
       NA
 
           NA
  
 
  
 16
 
  82660206
 
  82660873
 
  ENSEMBL   UCSC 
 
  668
 
 14
 
 16129
 
 island
 
 0.135537
 
 0.0528622
 
 Not queried
 
  0.7947247
 
       NA
 
           NA
  
 
  
 11
 
    976950
 
    977280
 
  ENSEMBL   UCSC 
 
  331
 
  4
 
 6398
 
 gene;island
 
 0.961137
 
 0.9397444
 
 Not queried
 
  0.7946857
 
       NA
 
           NA
  
 
  
 13
 
 112172244
 
 112172440
 
  ENSEMBL   UCSC 
 
  197
 
  4
 
 11897
 
 island
 
 0.710060
 
 0.8258578
 
 Not queried
 
  0.7945677
 
       NA
 
           NA
  
 
  
 19
 
  13915583
 
  13915877
 
  ENSEMBL   UCSC 
 
  295
 
  6
 
 19883
 
 gene;island
 
 0.947776
 
 0.9666487
 
 Not queried
 
  0.7944850
 
       NA
 
           NA
  
 
  
 14
 
  74254023
 
  74254319
 
  ENSEMBL   UCSC 
 
  297
 
  6
 
 12500
 
 tss
 
 0.013138
 
 0.0104285
 
 Not queried
 
  0.7943953
 
       NA
 
           NA
  
 
  
 14
 
  58666496
 
  58666796
 
  ENSEMBL   UCSC 
 
  301
 
  4
 
 12421
 
 tss
 
 0.002457
 
 0.0024502
 
 Not queried
 
  0.7943836
 
       NA
 
           NA
  
 
  
 4
 
 119757509
 
 119757755
 
  ENSEMBL   UCSC 
 
  247
 
  7
 
 30563
 
 tss;island
 
 0.004536
 
 0.0008924
 
 Not queried
 
  0.7943703
 
       NA
 
           NA
  
 
  
 5
 
 159739701
 
 159739851
 
  ENSEMBL   UCSC 
 
  151
 
  4
 
 32466
 
 tss;island
 
 0.052585
 
 0.0322173
 
 Not queried
 
  0.7943190
 
       NA
 
           NA
  
 
  
 2
 
  45182153
 
  45182417
 
  ENSEMBL   UCSC 
 
  265
 
  4
 
 24266
 
 island
 
 0.062537
 
 0.1773065
 
 Not queried
 
  0.7942897
 
       NA
 
           NA
  
 
  
 12
 
  53661747
 
  53662079
 
  ENSEMBL   UCSC 
 
  333
 
  5
 
 9736
 
 tss;island
 
 0.005814
 
 0.0080269
 
 Not queried
 
  0.7942554
 
       NA
 
           NA
  
 
  
 13
 
 112721712
 
 112721789
 
  ENSEMBL   UCSC 
 
   78
 
  6
 
 11543
 
 tss
 
 0.156087
 
 0.2487668
 
 Not queried
 
  0.7942041
 
       NA
 
           NA
  
 
  
 17
 
  60744606
 
  60744836
 
  ENSEMBL   UCSC 
 
  231
 
  4
 
 16723
 
 gene;island
 
 0.782551
 
 0.8414065
 
 Not queried
 
  0.7939046
 
       NA
 
           NA
  
 
  
 3
 
 170136159
 
 170136539
 
  ENSEMBL   UCSC 
 
  381
 
  9
 
 28714
 
 tss;island
 
 0.056563
 
 0.1496984
 
 Not queried
 
  0.7937134
 
       NA
 
           NA
  
 
  
 8
 
 118532625
 
 118532951
 
  ENSEMBL   UCSC 
 
  327
 
  8
 
 39883
 
 tss
 
 0.007668
 
 0.0042283
 
 Not queried
 
  0.7936063
 
       NA
 
           NA
  
 
  
 17
 
  38574180
 
  38574599
 
  ENSEMBL   UCSC 
 
  420
 
 11
 
 17333
 
 tss
 
 0.006161
 
 0.0061887
 
 Not queried
 
  0.7935325
 
       NA
 
           NA
  
 
  
 20
 
  10015646
 
  10015688
 
  ENSEMBL   UCSC 
 
   43
 
  4
 
 25594
 
 tss
 
 0.009559
 
 0.0181616
 
 Not queried
 
  0.7934446
 
       NA
 
           NA
  
 
  
 2
 
 204399782
 
 204400358
 
  ENSEMBL   UCSC 
 
  577
 
  8
 
 25026
 
 island
 
 0.023630
 
 0.0155801
 
 Not queried
 
  0.7934424
 
       NA
 
           NA
  
 
  
 6
 
   4282075
 
   4282346
 
  ENSEMBL   UCSC 
 
  272
 
  4
 
 35394
 
 island
 
 0.117647
 
 0.0395333
 
 Not queried
 
  0.7933785
 
       NA
 
           NA
  
 
  
 11
 
  66084469
 
  66084841
 
  ENSEMBL   UCSC 
 
  373
 
  5
 
 8506
 
 island
 
 0.043779
 
 0.0458718
 
 Not queried
 
  0.7932152
 
       NA
 
           NA
  
 
  
 11
 
  22851383
 
  22851591
 
  ENSEMBL   UCSC 
 
  209
 
  7
 
 7204
 
 tss
 
 0.008043
 
 0.0040558
 
 Not queried
 
  0.7931492
 
       NA
 
           NA
  
 
  
 17
 
  72322388
 
  72322791
 
  ENSEMBL   UCSC 
 
  404
 
  4
 
 16751
 
 gene;island
 
 0.178001
 
 0.0121434
 
 Not queried
 
  0.7930555
 
       NA
 
           NA
  
 
  
 16
 
  11038165
 
  11038308
 
  ENSEMBL   UCSC 
 
  144
 
  4
 
 15090
 
 tss
 
 0.003585
 
 0.0024580
 
 Not queried
 
  0.7929759
 
       NA
 
           NA
  
 
  
 8
 
  81490273
 
  81490635
 
  ENSEMBL   UCSC 
 
  363
 
  4
 
 40415
 
 island
 
 0.034301
 
 0.0166152
 
 Not queried
 
  0.7929225
 
       NA
 
           NA
  
 
  
 16
 
   5007694
 
   5008289
 
  ENSEMBL   UCSC 
 
  596
 
  8
 
 15078
 
 tss
 
 0.030665
 
 0.0097132
 
 Not queried
 
  0.7928789
 
       NA
 
           NA
  
 
  
 5
 
  93447204
 
  93447700
 
  ENSEMBL   UCSC 
 
  497
 
 10
 
 32965
 
 island
 
 0.005871
 
 0.0231872
 
 Not queried
 
  0.7927886
 
       NA
 
           NA
  
 
  
 22
 
  39898040
 
  39898247
 
  ENSEMBL   UCSC 
 
  208
 
  6
 
 27157
 
 tss
 
 0.067557
 
 0.0631312
 
 Not queried
 
  0.7926653
 
       NA
 
           NA
  
 
  
 2
 
  86422857
 
  86423274
 
  ENSEMBL   UCSC 
 
  418
 
  9
 
 24491
 
 island
 
 0.003270
 
 0.0031414
 
 Not queried
 
  0.7926149
 
       NA
 
           NA
  
 
  
 6
 
   3227556
 
   3227981
 
  ENSEMBL   UCSC 
 
  426
 
  5
 
 35385
 
 island
 
 0.012645
 
 0.0072817
 
 Not queried
 
  0.7925354
 
       NA
 
           NA
  
 
  
 2
 
 240062051
 
 240062353
 
  ENSEMBL   UCSC 
 
  303
 
  4
 
 23107
 
 gene;island
 
 0.941410
 
 0.9021695
 
 Not queried
 
  0.7924229
 
       NA
 
           NA
  
 
  
 12
 
  49319263
 
  49319767
 
  ENSEMBL   UCSC 
 
  505
 
 10
 
 9677
 
 tss;island
 
 0.008694
 
 0.0071837
 
 Not queried
 
  0.7922248
 
       NA
 
           NA
  
 
  
 4
 
   7042894
 
   7043264
 
  ENSEMBL   UCSC 
 
  371
 
  4
 
 29932
 
 gene;island
 
 0.958062
 
 0.9592773
 
 Not queried
 
  0.7922217
 
       NA
 
           NA
  
 
  
 6
 
 111136363
 
 111136640
 
  ENSEMBL   UCSC 
 
  278
 
  6
 
 36414
 
 island
 
 0.006768
 
 0.0056003
 
 Not queried
 
  0.7918275
 
       NA
 
           NA
  
 
  
 12
 
 124069168
 
 124069758
 
  ENSEMBL   UCSC 
 
  591
 
  5
 
 9384
 
 gene
 
 0.006527
 
 0.0034409
 
 Not queried
 
  0.7918259
 
       NA
 
           NA
  
 
  
 3
 
  72704324
 
  72704701
 
  ENSEMBL   UCSC 
 
  378
 
  5
 
 29281
 
 island
 
 0.602440
 
 0.4193707
 
 Not queried
 
  0.7917474
 
       NA
 
           NA
  
 
  
 19
 
  43918868
 
  43919028
 
  ENSEMBL   UCSC 
 
  161
 
  6
 
 20828
 
 tss
 
 0.540466
 
 0.5922197
 
 Not queried
 
  0.7913733
 
       NA
 
           NA
  
 
  
 12
 
 129338783
 
 129338939
 
  ENSEMBL   UCSC 
 
  157
 
  4
 
 9411
 
 gene;island
 
 0.116066
 
 0.0508921
 
 Not queried
 
  0.7913507
 
       NA
 
           NA
  
 
  
 4
 
  15779642
 
  15780011
 
  ENSEMBL   UCSC 
 
  370
 
  5
 
 30940
 
 island
 
 0.015484
 
 0.0117716
 
 Not queried
 
  0.7912395
 
       NA
 
           NA
  
 
  
 1
 
 175162044
 
 175162553
 
  ENSEMBL   UCSC 
 
  510
 
 13
 
 2105
 
 tss;island
 
 0.018434
 
 0.0112808
 
 Not queried
 
  0.7910791
 
       NA
 
           NA
  
 
  
 5
 
  63257554
 
  63257941
 
  ENSEMBL   UCSC 
 
  388
 
  9
 
 32087
 
 tss
 
 0.044553
 
 0.0557717
 
 Not queried
 
  0.7910164
 
       NA
 
           NA
  
 
  
 5
 
  76114373
 
  76114822
 
  ENSEMBL   UCSC 
 
  450
 
  8
 
 32137
 
 tss
 
 0.030239
 
 0.0124813
 
 Not queried
 
  0.7908319
 
       NA
 
           NA
  
 
  
 5
 
 137673565
 
 137673902
 
  ENSEMBL   UCSC 
 
  338
 
  7
 
 31768
 
 gene;island
 
 0.005457
 
 0.0045158
 
 Not queried
 
  0.7905121
 
       NA
 
           NA
  
 
  
 5
 
 137673565
 
 137673699
 
  ENSEMBL   UCSC 
 
  135
 
  5
 
 32319
 
 tss
 
 0.007013
 
 0.0053627
 
 Not queried
 
  0.7905121
 
       NA
 
           NA
  
 
  
 11
 
 130786525
 
 130786720
 
  ENSEMBL   UCSC 
 
  196
 
  6
 
 7821
 
 tss
 
 0.007472
 
 0.0113272
 
 Not queried
 
  0.7903888
 
       NA
 
           NA
  
 
  
 2
 
 198651303
 
 198651576
 
  ENSEMBL   UCSC 
 
  274
 
  4
 
 23803
 
 tss;island
 
 0.072027
 
 0.0525817
 
 Not queried
 
  0.7903693
 
       NA
 
           NA
  
 
  
 4
 
    779480
 
    779880
 
  ENSEMBL   UCSC 
 
  401
 
  4
 
 29837
 
 gene;island
 
 0.015880
 
 0.0157220
 
 Not queried
 
  0.7903458
 
       NA
 
           NA
  
 
  
 1
 
  87380673
 
  87380955
 
  ENSEMBL   UCSC 
 
  283
 
  4
 
 611
 
 gene;tss;island
 
 0.001997
 
 0.0003599
 
 Not queried
 
  0.7902128
 
       NA
 
           NA
  
 
  
 20
 
  42086101
 
  42086476
 
  ENSEMBL   UCSC 
 
  376
 
  9
 
 25749
 
 tss
 
 0.005781
 
 0.0046663
 
 Not queried
 
  0.7901105
 
       NA
 
           NA
  
 
  
 6
 
  35109121
 
  35109722
 
  ENSEMBL   UCSC 
 
  602
 
 11
 
 36069
 
 island
 
 0.867619
 
 0.9640472
 
 Not queried
 
  0.7895871
 
       NA
 
           NA
  
 
  
 1
 
 228890801
 
 228891036
 
  ENSEMBL   UCSC 
 
  236
 
  5
 
 4306
 
 island
 
 0.969095
 
 0.9506997
 
 Not queried
 
  0.7895499
 
       NA
 
           NA
  
 
  
 1
 
 228504281
 
 228504761
 
  ENSEMBL   UCSC 
 
  481
 
  5
 
 1055
 
 gene;island
 
 0.449298
 
 0.5729448
 
 Not queried
 
  0.7893831
 
       NA
 
           NA
  
 
  
 19
 
  45311781
 
  45312060
 
  ENSEMBL   UCSC 
 
  280
 
  4
 
 20853
 
 tss;island
 
 0.015455
 
 0.0136725
 
 Not queried
 
  0.7893825
 
       NA
 
           NA
  
 
  
 3
 
  71180032
 
  71180071
 
  ENSEMBL   UCSC 
 
   40
 
  4
 
 27858
 
 gene
 
 0.027088
 
 0.0223566
 
 Not queried
 
  0.7893696
 
       NA
 
           NA
  
 
  
 12
 
   8025517
 
   8025646
 
  ENSEMBL   UCSC 
 
  130
 
  6
 
 9551
 
 tss
 
 0.211588
 
 0.2974107
 
 Not queried
 
  0.7891925
 
       NA
 
           NA
  
 
  
 4
 
  40631571
 
  40632020
 
  ENSEMBL   UCSC 
 
  450
 
  6
 
 30995
 
 island
 
 0.001694
 
 0.0030615
 
 Not queried
 
  0.7890802
 
       NA
 
           NA
  
 
  
 8
 
 145981074
 
 145981224
 
  ENSEMBL   UCSC 
 
  151
 
  8
 
 39991
 
 tss;island
 
 0.005715
 
 0.0088899
 
 Not queried
 
  0.7890468
 
       NA
 
           NA
  
 
  
 19
 
  10811855
 
  10812018
 
  ENSEMBL   UCSC 
 
  164
 
  4
 
 20457
 
 tss
 
 0.007302
 
 0.0249962
 
 Not queried
 
  0.7888567
 
       NA
 
           NA
  
 
  
 7
 
  21583226
 
  21583623
 
  ENSEMBL   UCSC 
 
  398
 
  4
 
 36911
 
 gene;island
 
 0.006968
 
 0.0176728
 
 Not queried
 
  0.7888496
 
       NA
 
           NA
  
 
  
 6
 
  74171350
 
  74171446
 
  ENSEMBL   UCSC 
 
   97
 
  4
 
 35071
 
 tss
 
 0.010759
 
 0.0069263
 
 Not queried
 
  0.7888345
 
       NA
 
           NA
  
 
  
 6
 
   3258929
 
   3259048
 
  ENSEMBL   UCSC 
 
  120
 
  4
 
 34598
 
 tss
 
 0.026626
 
 0.0111291
 
 Not queried
 
  0.7886768
 
       NA
 
           NA
  
 
  
 4
 
 164087672
 
 164088186
 
  ENSEMBL   UCSC 
 
  515
 
  9
 
 31399
 
 island
 
 0.006066
 
 0.0020611
 
 Not queried
 
  0.7886280
 
       NA
 
           NA
  
 
  
 16
 
  88453579
 
  88453939
 
  ENSEMBL   UCSC 
 
  361
 
  5
 
 16199
 
 island
 
 0.522165
 
 0.4528337
 
 Not queried
 
  0.7886062
 
       NA
 
           NA
  
 
  
 2
 
 103353026
 
 103353231
 
  ENSEMBL   UCSC 
 
  206
 
  4
 
 22812
 
 gene;island
 
 0.005431
 
 0.0031199
 
 Not queried
 
  0.7885697
 
       NA
 
           NA
  
 
  
 8
 
  23082233
 
  23082961
 
  ENSEMBL   UCSC 
 
  729
 
 12
 
 40148
 
 island
 
 0.015205
 
 0.0282715
 
 Not queried
 
  0.7883741
 
       NA
 
           NA
  
 
  
 17
 
  78039301
 
  78039528
 
  ENSEMBL   UCSC 
 
  228
 
  4
 
 16854
 
 gene;island
 
 0.758084
 
 0.8553823
 
 Not queried
 
  0.7882408
 
       NA
 
           NA
  
 
  
 8
 
  38757532
 
  38757859
 
  ENSEMBL   UCSC 
 
  328
 
  5
 
 39659
 
 tss;island
 
 0.013252
 
 0.0153803
 
 Not queried
 
  0.7880318
 
       NA
 
           NA
  
 
  
 17
 
   7287652
 
   7288001
 
  ENSEMBL   UCSC 
 
  350
 
  4
 
 16393
 
 gene;island
 
 0.349651
 
 0.3915688
 
 Not queried
 
  0.7878821
 
       NA
 
           NA
  
 
  
 10
 
  93392644
 
  93392839
 
  ENSEMBL   UCSC 
 
  196
 
  4
 
 4673
 
 gene
 
 0.044543
 
 0.0362784
 
 Not queried
 
  0.7878647
 
       NA
 
           NA
  
 
  
 3
 
  52279902
 
  52280329
 
  ENSEMBL   UCSC 
 
  428
 
  6
 
 29181
 
 island
 
 0.016700
 
 0.0280447
 
 Not queried
 
  0.7878395
 
       NA
 
           NA
  
 
  
 11
 
 118442837
 
 118443028
 
  ENSEMBL   UCSC 
 
  192
 
  6
 
 7736
 
 tss;island
 
 0.012459
 
 0.0108260
 
 Not queried
 
  0.7877003
 
       NA
 
           NA
  
 
  
 16
 
   3184694
 
   3185250
 
  ENSEMBL   UCSC 
 
  557
 
  8
 
 15637
 
 island
 
 0.008221
 
 0.0035871
 
 Not queried
 
  0.7874298
 
       NA
 
           NA
  
 
  
 16
 
  67144054
 
  67144419
 
  ENSEMBL   UCSC 
 
  366
 
  4
 
 14787
 
 gene;island
 
 0.014775
 
 0.0100667
 
 Not queried
 
  0.7874024
 
       NA
 
           NA
  
 
  
 12
 
  95467511
 
  95467612
 
  ENSEMBL   UCSC 
 
  102
 
  5
 
 9921
 
 tss
 
 0.003823
 
 0.0045044
 
 Not queried
 
  0.7872536
 
       NA
 
           NA
  
 
  
 6
 
  30128219
 
  30128686
 
  ENSEMBL   UCSC 
 
  468
 
 12
 
 33735
 
 gene
 
 0.591672
 
 0.7272294
 
 Not queried
 
  0.7872514
 
       NA
 
           NA
  
 
  
 19
 
  52674854
 
  52675470
 
  ENSEMBL   UCSC 
 
  617
 
 11
 
 22351
 
 island
 
 0.041640
 
 0.0299340
 
 Not queried
 
  0.7872324
 
       NA
 
           NA
  
 
  
 1
 
  91487516
 
  91487603
 
  ENSEMBL   UCSC 
 
   88
 
  4
 
 627
 
 gene;tss;island
 
 0.003478
 
 0.0044855
 
 Not queried
 
  0.7869061
 
       NA
 
           NA
  
 
  
 5
 
 131746062
 
 131746416
 
  ENSEMBL   UCSC 
 
  355
 
  5
 
 32271
 
 tss;island
 
 0.011750
 
 0.0043196
 
 Not queried
 
  0.7868333
 
       NA
 
           NA
  
 
  
 7
 
  86273058
 
  86273180
 
  ENSEMBL   UCSC 
 
  123
 
  5
 
 37680
 
 tss
 
 0.004892
 
 0.0022007
 
 Not queried
 
  0.7867498
 
       NA
 
           NA
  
 
  
 1
 
  57888343
 
  57888796
 
  ENSEMBL   UCSC 
 
  454
 
  5
 
 554
 
 gene;island
 
 0.011700
 
 0.0919939
 
 Not queried
 
  0.7866271
 
       NA
 
           NA
  
 
  
 16
 
   3074294
 
   3074537
 
  ENSEMBL   UCSC 
 
  244
 
  8
 
 15047
 
 tss
 
 0.006119
 
 0.0032681
 
 Not queried
 
  0.7865267
 
       NA
 
           NA
  
 
  
 20
 
   3761916
 
   3762167
 
  ENSEMBL   UCSC 
 
  252
 
  5
 
 25975
 
 island
 
 0.025281
 
 0.0105553
 
 Not queried
 
  0.7863373
 
       NA
 
           NA
  
 
  
 2
 
 178483847
 
 178484058
 
  ENSEMBL   UCSC 
 
  212
 
  4
 
 23750
 
 tss;island
 
 0.016598
 
 0.0201140
 
 Not queried
 
  0.7860526
 
       NA
 
           NA
  
 
  
 5
 
 169010289
 
 169010600
 
  ENSEMBL   UCSC 
 
  312
 
  5
 
 32481
 
 tss
 
 0.003419
 
 0.0016583
 
 Not queried
 
  0.7860515
 
       NA
 
           NA
  
 
  
 2
 
  45870771
 
  45871073
 
  ENSEMBL   UCSC 
 
  303
 
  4
 
 24272
 
 island
 
 0.048420
 
 0.0347489
 
 Not queried
 
  0.7860327
 
       NA
 
           NA
  
 
  
 16
 
    334445
 
    334889
 
  ENSEMBL   UCSC 
 
  445
 
  4
 
 14526
 
 gene;island
 
 0.968261
 
 0.9781039
 
 Not queried
 
  0.7859776
 
       NA
 
           NA
  
 
  
 19
 
  50879393
 
  50879698
 
  ENSEMBL   UCSC 
 
  306
 
  9
 
 22306
 
 island
 
 0.006682
 
 0.0063409
 
 Not queried
 
  0.7858143
 
       NA
 
           NA
  
 
  
 7
 
 158217561
 
 158217861
 
  ENSEMBL   UCSC 
 
  301
 
  4
 
 37369
 
 gene;island
 
 0.739340
 
 0.8918774
 
 Not queried
 
  0.7857625
 
       NA
 
           NA
  
 
  
 6
 
  21666391
 
  21666666
 
  ENSEMBL   UCSC 
 
  276
 
  5
 
 34664
 
 tss
 
 0.018301
 
 0.0125046
 
 Not queried
 
  0.7857319
 
       NA
 
           NA
  
 
  
 16
 
    331050
 
    331454
 
  ENSEMBL   UCSC 
 
  405
 
  4
 
 14524
 
 gene;island
 
 0.220266
 
 0.4376459
 
 Not queried
 
  0.7857172
 
       NA
 
           NA
  
 
  
 17
 
  59940709
 
  59941132
 
  ENSEMBL   UCSC 
 
  424
 
  8
 
 18633
 
 island
 
 0.005750
 
 0.0036723
 
 Not queried
 
  0.7856354
 
       NA
 
           NA
  
 
  
 7
 
 148036314
 
 148036740
 
  ENSEMBL   UCSC 
 
  427
 
  7
 
 37258
 
 gene;island
 
 0.021864
 
 0.0376847
 
 Not queried
 
  0.7855990
 
       NA
 
           NA
  
 
  
 12
 
 133613766
 
 133613924
 
  ENSEMBL   UCSC 
 
  159
 
  6
 
 10137
 
 tss;island
 
 0.015056
 
 0.0087545
 
 Not queried
 
  0.7853188
 
       NA
 
           NA
  
 
  
 1
 
 179198950
 
 179199199
 
  ENSEMBL   UCSC 
 
  250
 
  4
 
 2116
 
 tss;island
 
 0.012587
 
 0.0189416
 
 Not queried
 
  0.7851581
 
       NA
 
           NA
  
 
  
 1
 
  53905612
 
  53905934
 
  ENSEMBL   UCSC 
 
  323
 
  6
 
 3284
 
 island
 
 0.766340
 
 0.8020034
 
 Not queried
 
  0.7850308
 
       NA
 
           NA
  
 
  
 17
 
  76227227
 
  76227681
 
  ENSEMBL   UCSC 
 
  455
 
  7
 
 18820
 
 island
 
 0.626155
 
 0.5125330
 
 Not queried
 
  0.7849897
 
       NA
 
           NA
  
 
  
 10
 
 118084109
 
 118084587
 
  ENSEMBL   UCSC 
 
  479
 
  6
 
 4775
 
 gene
 
 0.218492
 
 0.3141107
 
 Not queried
 
  0.7848128
 
       NA
 
           NA
  
 
  
 3
 
 131245815
 
 131246097
 
  ENSEMBL   UCSC 
 
  283
 
  4
 
 29482
 
 island
 
 0.296175
 
 0.3789652
 
 Not queried
 
  0.7848082
 
       NA
 
           NA
  
 
  
 6
 
  29855401
 
  29855636
 
  ENSEMBL   UCSC 
 
  236
 
  9
 
 33699
 
 gene
 
 0.006520
 
 0.0191780
 
 Not queried
 
  0.7847574
 
       NA
 
           NA
  
 
  
 6
 
  30431563
 
  30431860
 
  ENSEMBL   UCSC 
 
  298
 
 15
 
 35682
 
 island
 
 0.031338
 
 0.0219965
 
 Not queried
 
  0.7847108
 
       NA
 
           NA
  
 
  
 14
 
  51411049
 
  51411647
 
  ENSEMBL   UCSC 
 
  599
 
 10
 
 12835
 
 island
 
 0.009719
 
 0.0056860
 
 Not queried
 
  0.7846456
 
       NA
 
           NA
  
 
  
 7
 
 107643925
 
 107644024
 
  ENSEMBL   UCSC 
 
  100
 
  6
 
 37833
 
 tss;island
 
 0.005425
 
 0.0071371
 
 Not queried
 
  0.7841843
 
       NA
 
           NA
  
 
  
 22
 
  38071455
 
  38071607
 
  ENSEMBL   UCSC 
 
  153
 
  5
 
 27121
 
 tss
 
 0.011675
 
 0.0489412
 
 Not queried
 
  0.7841628
 
       NA
 
           NA
  
 
  
 19
 
  46142574
 
  46142930
 
  ENSEMBL   UCSC 
 
  357
 
  9
 
 20877
 
 tss;island
 
 0.044144
 
 0.0430939
 
 Not queried
 
  0.7841274
 
       NA
 
           NA
  
 
  
 17
 
   7461260
 
   7461556
 
  ENSEMBL   UCSC 
 
  297
 
  4
 
 17087
 
 tss
 
 0.031266
 
 0.0517942
 
 Not queried
 
  0.7839853
 
       NA
 
           NA
  
 
  
 2
 
 112895559
 
 112895809
 
  ENSEMBL   UCSC 
 
  251
 
  6
 
 23576
 
 tss;island
 
 0.100724
 
 0.0774497
 
 Not queried
 
  0.7839209
 
       NA
 
           NA
  
 
  
 6
 
 168389700
 
 168390347
 
  ENSEMBL   UCSC 
 
  648
 
  6
 
 36652
 
 island
 
 0.649411
 
 0.8585309
 
 Not queried
 
  0.7838065
 
       NA
 
           NA
  
 
  
 1
 
  43751270
 
  43751563
 
  ENSEMBL   UCSC 
 
  294
 
  8
 
 1558
 
 tss
 
 0.726672
 
 0.8567381
 
 Not queried
 
  0.7835740
 
       NA
 
           NA
  
 
  
 6
 
  32130562
 
  32130758
 
  ENSEMBL   UCSC 
 
  197
 
  5
 
 35905
 
 island
 
 0.871596
 
 0.9436504
 
 Not queried
 
  0.7832598
 
       NA
 
           NA
  
 
  
 1
 
  29138919
 
  29139278
 
  ENSEMBL   UCSC 
 
  360
 
  5
 
 371
 
 gene;island
 
 0.008265
 
 0.0099215
 
 Not queried
 
  0.7830292
 
       NA
 
           NA
  
 
  
 4
 
  76862254
 
  76862425
 
  ENSEMBL   UCSC 
 
  172
 
  7
 
 30442
 
 tss;island
 
 0.034318
 
 0.0108879
 
 Not queried
 
  0.7829736
 
       NA
 
           NA
  
 
  
 4
 
  99064459
 
  99064904
 
  ENSEMBL   UCSC 
 
  446
 
  7
 
 30508
 
 tss
 
 0.005074
 
 0.0011034
 
 Not queried
 
  0.7829443
 
       NA
 
           NA
  
 
  
 6
 
  31364895
 
  31364999
 
  ENSEMBL   UCSC 
 
  105
 
  4
 
 35769
 
 island
 
 0.011622
 
 0.0053531
 
 Not queried
 
  0.7829249
 
       NA
 
           NA
  
 
  
 1
 
 101704441
 
 101704656
 
  ENSEMBL   UCSC 
 
  216
 
  4
 
 655
 
 gene;island
 
 0.012658
 
 0.0251826
 
 Not queried
 
  0.7827249
 
       NA
 
           NA
  
 
  
 6
 
 147829799
 
 147830172
 
  ENSEMBL   UCSC 
 
  374
 
  8
 
 36549
 
 island
 
 0.010565
 
 0.0081618
 
 Not queried
 
  0.7826995
 
       NA
 
           NA
  
 
  
 14
 
  94254586
 
  94254816
 
  ENSEMBL   UCSC 
 
  231
 
  4
 
 13100
 
 island
 
 0.010741
 
 0.0319304
 
 Not queried
 
  0.7825694
 
       NA
 
           NA
  
 
  
 3
 
 123987591
 
 123988013
 
  ENSEMBL   UCSC 
 
  423
 
  6
 
 27921
 
 gene;island
 
 0.511489
 
 0.6521260
 
 Not queried
 
  0.7821639
 
       NA
 
           NA
  
 
  
 5
 
 135364328
 
 135364580
 
  ENSEMBL   UCSC 
 
  253
 
  8
 
 32309
 
 tss;island
 
 0.076032
 
 0.0433606
 
 Not queried
 
  0.7821071
 
       NA
 
           NA
  
 
  
 8
 
  74205400
 
  74205666
 
  ENSEMBL   UCSC 
 
  267
 
  4
 
 39336
 
 gene;island
 
 0.005847
 
 0.0041387
 
 Not queried
 
  0.7820973
 
       NA
 
           NA
  
 
  
 7
 
 155674426
 
 155674940
 
  ENSEMBL   UCSC 
 
  515
 
  5
 
 39079
 
 island
 
 0.276097
 
 0.3632406
 
 Not queried
 
  0.7819548
 
       NA
 
           NA
  
 
  
 17
 
   6899085
 
   6899888
 
  ENSEMBL   UCSC 
 
  804
 
 14
 
 17913
 
 island
 
 0.924730
 
 0.5803616
 
 Not queried
 
  0.7816944
 
       NA
 
           NA
  
 
  
 6
 
 117086931
 
 117087238
 
  ENSEMBL   UCSC 
 
  308
 
  7
 
 35193
 
 tss
 
 0.090182
 
 0.0940780
 
 Not queried
 
  0.7816674
 
       NA
 
           NA
  
 
  
 6
 
  30709428
 
  30709924
 
  ENSEMBL   UCSC 
 
  497
 
  5
 
 33822
 
 gene;island
 
 0.011920
 
 0.3233738
 
 Not queried
 
  0.7814779
 
       NA
 
           NA
  
 
  
 11
 
  65547778
 
  65548028
 
  ENSEMBL   UCSC 
 
  251
 
  7
 
 8478
 
 island
 
 0.009615
 
 0.0100224
 
 Not queried
 
  0.7811730
 
       NA
 
           NA
  
 
  
 8
 
  99129132
 
  99129319
 
  ENSEMBL   UCSC 
 
  188
 
  4
 
 39372
 
 gene
 
 0.015488
 
 0.0104659
 
 Not queried
 
  0.7811015
 
       NA
 
           NA
  
 
  
 12
 
 103352235
 
 103352454
 
  ENSEMBL   UCSC 
 
  220
 
  5
 
 9270
 
 gene;island
 
 0.006917
 
 0.0041122
 
 Not queried
 
  0.7809140
 
       NA
 
           NA
  
 
  
 10
 
 133644553
 
 133644760
 
  ENSEMBL   UCSC 
 
  208
 
  4
 
 6279
 
 island
 
 0.156808
 
 0.2457849
 
 Not queried
 
  0.7807322
 
       NA
 
           NA
  
 
  
 19
 
   4851963
 
   4852205
 
  ENSEMBL   UCSC 
 
  243
 
  4
 
 19740
 
 gene;island
 
 0.964729
 
 0.9803417
 
 Not queried
 
  0.7806822
 
       NA
 
           NA
  
 
  
 1
 
 224180288
 
 224180516
 
  ENSEMBL   UCSC 
 
  229
 
  4
 
 4239
 
 island
 
 0.200023
 
 0.1119563
 
 Not queried
 
  0.7805250
 
       NA
 
           NA
  
 
  
 8
 
 121137537
 
 121137879
 
  ENSEMBL   UCSC 
 
  343
 
  4
 
 39410
 
 gene;island
 
 0.121950
 
 0.1112361
 
 Not queried
 
  0.7804907
 
       NA
 
           NA
  
 
  
 3
 
  25706262
 
  25706456
 
  ENSEMBL   UCSC 
 
  195
 
  4
 
 28192
 
 tss;island
 
 0.005728
 
 0.0075288
 
 Not queried
 
  0.7804236
 
       NA
 
           NA
  
 
  
 4
 
  93224895
 
  93225565
 
  ENSEMBL   UCSC 
 
  671
 
  8
 
 31183
 
 island
 
 0.010765
 
 0.0152383
 
 Not queried
 
  0.7803787
 
       NA
 
           NA
  
 
  
 8
 
  67974213
 
  67974639
 
  ENSEMBL   UCSC 
 
  427
 
 10
 
 40364
 
 island
 
 0.004053
 
 0.0036506
 
 Not queried
 
  0.7803588
 
       NA
 
           NA
  
 
  
 6
 
  46097392
 
  46097695
 
  ENSEMBL   UCSC 
 
  304
 
  8
 
 35018
 
 tss
 
 0.004898
 
 0.0026088
 
 Not queried
 
  0.7803461
 
       NA
 
           NA
  
 
  
 1
 
 113933133
 
 113933413
 
  ENSEMBL   UCSC 
 
  281
 
  5
 
 1843
 
 tss;island
 
 0.040043
 
 0.0189574
 
 Not queried
 
  0.7802488
 
       NA
 
           NA
  
 
  
 3
 
 169378675
 
 169379010
 
  ENSEMBL   UCSC 
 
  336
 
  5
 
 28022
 
 gene;island
 
 0.053421
 
 0.0231418
 
 Not queried
 
  0.7802374
 
       NA
 
           NA
  
 
  
 5
 
  70751216
 
  70751435
 
  ENSEMBL   UCSC 
 
  220
 
  7
 
 32113
 
 tss;island
 
 0.003341
 
 0.0020483
 
 Not queried
 
  0.7802067
 
       NA
 
           NA
  
 
  
 19
 
   4670455
 
   4670669
 
  ENSEMBL   UCSC 
 
  215
 
  7
 
 20369
 
 tss
 
 0.015470
 
 0.0077591
 
 Not queried
 
  0.7800041
 
       NA
 
           NA
  
 
  
 10
 
 134599783
 
 134600998
 
  ENSEMBL   UCSC 
 
 1216
 
 30
 
 5450
 
 tss;island
 
 0.058468
 
 0.1047510
 
 Not queried
 
  0.7799739
 
       NA
 
           NA
  
 
  
 6
 
  32126315
 
  32126616
 
  ENSEMBL   UCSC 
 
  302
 
  4
 
 34062
 
 gene
 
 0.933089
 
 0.9493286
 
 Not queried
 
  0.7794900
 
       NA
 
           NA
  
 
  
 11
 
 125439061
 
 125439275
 
  ENSEMBL   UCSC 
 
  215
 
  6
 
 7792
 
 tss
 
 0.017692
 
 0.0105025
 
 Not queried
 
  0.7794009
 
       NA
 
           NA
  
 
  
 20
 
  43936663
 
  43937467
 
  ENSEMBL   UCSC 
 
  805
 
  8
 
 25428
 
 gene;island
 
 0.558198
 
 0.4732615
 
 Not queried
 
  0.7793777
 
       NA
 
           NA
  
 
  
 11
 
  10476494
 
  10476662
 
  ENSEMBL   UCSC 
 
  169
 
  5
 
 6506
 
 gene;tss;island
 
 0.042896
 
 0.0284730
 
 Not queried
 
  0.7792805
 
       NA
 
           NA
  
 
  
 11
 
 119234950
 
 119235213
 
  ENSEMBL   UCSC 
 
  264
 
  5
 
 7755
 
 tss
 
 0.040688
 
 0.0175276
 
 Not queried
 
  0.7792698
 
       NA
 
           NA
  
 
  
 19
 
  36422477
 
  36422730
 
  ENSEMBL   UCSC 
 
  254
 
  5
 
 21926
 
 island
 
 0.006666
 
 0.0048178
 
 Not queried
 
  0.7790724
 
       NA
 
           NA
  
 
  
 11
 
  65194933
 
  65195039
 
  ENSEMBL   UCSC 
 
  107
 
  4
 
 8456
 
 island
 
 0.005814
 
 0.0065430
 
 Not queried
 
  0.7789364
 
       NA
 
           NA
  
 
  
 8
 
  10470600
 
  10470999
 
  ENSEMBL   UCSC 
 
  400
 
  4
 
 39198
 
 gene;island
 
 0.819073
 
 0.8837396
 
 Not queried
 
  0.7787257
 
       NA
 
           NA
  
 
  
 5
 
 150400001
 
 150400672
 
  ENSEMBL   UCSC 
 
  672
 
  6
 
 31866
 
 gene
 
 0.080530
 
 0.2354433
 
 Not queried
 
  0.7786860
 
       NA
 
           NA
  
 
  
 17
 
  18684650
 
  18685008
 
  ENSEMBL   UCSC 
 
  359
 
  4
 
 16475
 
 gene
 
 0.124867
 
 0.1389428
 
 Not queried
 
  0.7786443
 
       NA
 
           NA
  
 
  
 1
 
  36915704
 
  36916744
 
  ENSEMBL   UCSC 
 
 1041
 
 17
 
 3060
 
 island
 
 0.012859
 
 0.0112793
 
 Not queried
 
  0.7785843
 
       NA
 
           NA
  
 
  
 2
 
  80531500
 
  80531898
 
  ENSEMBL   UCSC 
 
  399
 
  9
 
 23473
 
 tss
 
 0.022970
 
 0.0459062
 
 Not queried
 
  0.7782432
 
       NA
 
           NA
  
 
  
 4
 
 119606135
 
 119606544
 
  ENSEMBL   UCSC 
 
  410
 
  7
 
 30562
 
 tss
 
 0.015424
 
 0.0033869
 
 Not queried
 
  0.7782368
 
       NA
 
           NA
  
 
  
 3
 
 125239027
 
 125239413
 
  ENSEMBL   UCSC 
 
  387
 
 10
 
 29422
 
 island
 
 0.006997
 
 0.0089116
 
 Not queried
 
  0.7781697
 
       NA
 
           NA
  
 
  
 5
 
  16508920
 
  16509123
 
  ENSEMBL   UCSC 
 
  204
 
  4
 
 31567
 
 gene
 
 0.582001
 
 0.7269247
 
 Not queried
 
  0.7781052
 
       NA
 
           NA
  
 
  
 19
 
  53073577
 
  53074012
 
  ENSEMBL   UCSC 
 
  436
 
  4
 
 20197
 
 gene
 
 0.039376
 
 0.0215759
 
 Not queried
 
  0.7780128
 
       NA
 
           NA
  
 
  
 8
 
 144373269
 
 144373569
 
  ENSEMBL   UCSC 
 
  301
 
  7
 
 40652
 
 island
 
 0.022723
 
 0.0073534
 
 Not queried
 
  0.7778453
 
       NA
 
           NA
  
 
  
 7
 
  28338584
 
  28338790
 
  ENSEMBL   UCSC 
 
  207
 
  5
 
 37505
 
 tss
 
 0.803253
 
 0.6231677
 
 Not queried
 
  0.7774858
 
       NA
 
           NA
  
 
  
 5
 
  43514915
 
  43515527
 
  ENSEMBL   UCSC 
 
  613
 
  9
 
 32776
 
 island
 
 0.003379
 
 0.0025241
 
 Not queried
 
  0.7773169
 
       NA
 
           NA
  
 
  
 1
 
 156675322
 
 156675562
 
  ENSEMBL   UCSC 
 
  241
 
  7
 
 3863
 
 island
 
 0.024393
 
 0.0118280
 
 Not queried
 
  0.7770292
 
       NA
 
           NA
  
 
  
 12
 
 133335468
 
 133335850
 
  ENSEMBL   UCSC 
 
  383
 
  5
 
 9458
 
 gene;island
 
 0.914517
 
 0.9517853
 
 Not queried
 
  0.7769722
 
       NA
 
           NA
  
 
  
 1
 
 113051685
 
 113052167
 
  ENSEMBL   UCSC 
 
  483
 
  6
 
 691
 
 gene;island
 
 0.082217
 
 0.1128683
 
 Not queried
 
  0.7769541
 
       NA
 
           NA
  
 
  
 21
 
  45148694
 
  45149141
 
  ENSEMBL   UCSC 
 
  448
 
  5
 
 26447
 
 gene;island
 
 0.009504
 
 0.0636256
 
 Not queried
 
  0.7769180
 
       NA
 
           NA
  
 
  
 11
 
  64108241
 
  64108550
 
  ENSEMBL   UCSC 
 
  310
 
  4
 
 6682
 
 gene;island
 
 0.223816
 
 0.0209475
 
 Not queried
 
  0.7768674
 
       NA
 
           NA
  
 
  
 1
 
  29508658
 
  29508888
 
  ENSEMBL   UCSC 
 
  231
 
  6
 
 1440
 
 tss
 
 0.017945
 
 0.0121858
 
 Not queried
 
  0.7768616
 
       NA
 
           NA
  
 
  
 1
 
 156675388
 
 156675562
 
  ENSEMBL   UCSC 
 
  175
 
  6
 
 2020
 
 tss
 
 0.022136
 
 0.0129541
 
 Not queried
 
  0.7768244
 
       NA
 
           NA
  
 
  
 4
 
  77227489
 
  77227631
 
  ENSEMBL   UCSC 
 
  143
 
  5
 
 30448
 
 tss
 
 0.010221
 
 0.0067703
 
 Not queried
 
  0.7767517
 
       NA
 
           NA
  
 
  
 15
 
  67458370
 
  67458665
 
  ENSEMBL   UCSC 
 
  296
 
  4
 
 13393
 
 gene
 
 0.902014
 
 0.9568297
 
 Not queried
 
  0.7766034
 
       NA
 
           NA
  
 
  
 7
 
  65447069
 
  65447249
 
  ENSEMBL   UCSC 
 
  181
 
  5
 
 37028
 
 gene
 
 0.011395
 
 0.0115533
 
 Not queried
 
  0.7765442
 
       NA
 
           NA
  
 
  
 1
 
 246930936
 
 246931160
 
  ENSEMBL   UCSC 
 
  225
 
  4
 
 1123
 
 gene
 
 0.989585
 
 0.9846142
 
 Not queried
 
  0.7763849
 
       NA
 
           NA
  
 
  
 2
 
 233470647
 
 233471248
 
  ENSEMBL   UCSC 
 
  602
 
 10
 
 25200
 
 island
 
 0.008922
 
 0.0124513
 
 Not queried
 
  0.7762344
 
       NA
 
           NA
  
 
  
 19
 
  36909326
 
  36909606
 
  ENSEMBL   UCSC 
 
  281
 
  4
 
 21939
 
 island
 
 0.005868
 
 0.0089077
 
 Not queried
 
  0.7760799
 
       NA
 
           NA
  
 
  
 12
 
  58087331
 
  58087721
 
  ENSEMBL   UCSC 
 
  391
 
  8
 
 9824
 
 tss
 
 0.041291
 
 0.0196169
 
 Not queried
 
  0.7759626
 
       NA
 
           NA
  
 
  
 17
 
  36908169
 
  36908971
 
  ENSEMBL   UCSC 
 
  803
 
 11
 
 17296
 
 tss
 
 0.005318
 
 0.0029422
 
 Not queried
 
  0.7758711
 
       NA
 
           NA
  
 
  
 3
 
   5021225
 
   5021469
 
  ENSEMBL   UCSC 
 
  245
 
  4
 
 27643
 
 gene;island
 
 0.003576
 
 0.0025669
 
 Not queried
 
  0.7758193
 
       NA
 
           NA
  
 
  
 1
 
 154475068
 
 154475269
 
  ENSEMBL   UCSC 
 
  202
 
  4
 
 779
 
 gene;tss;island
 
 0.058414
 
 0.0815131
 
 Not queried
 
  0.7757320
 
       NA
 
           NA
  
 
  
 7
 
 158277675
 
 158278040
 
  ENSEMBL   UCSC 
 
  366
 
  5
 
 37375
 
 gene;island
 
 0.756962
 
 0.8558855
 
 Not queried
 
  0.7757318
 
       NA
 
           NA
  
 
  
 12
 
  57634549
 
  57634855
 
  ENSEMBL   UCSC 
 
  307
 
  7
 
 9814
 
 tss
 
 0.024692
 
 0.0150335
 
 Not queried
 
  0.7756490
 
       NA
 
           NA
  
 
  
 16
 
  81526954
 
  81527504
 
  ENSEMBL   UCSC 
 
  551
 
  7
 
 14854
 
 gene
 
 0.949193
 
 0.9736100
 
 Not queried
 
  0.7754415
 
       NA
 
           NA
  
 
  
 14
 
  54421381
 
  54421578
 
  ENSEMBL   UCSC 
 
  198
 
  7
 
 12083
 
 gene;tss;island
 
 0.064136
 
 0.0272535
 
 Not queried
 
  0.7754159
 
       NA
 
           NA
  
 
  
 11
 
   1036677
 
   1036938
 
  ENSEMBL   UCSC 
 
  262
 
  8
 
 7897
 
 island
 
 0.164691
 
 0.2579884
 
 Not queried
 
  0.7753191
 
       NA
 
           NA
  
 
  
 2
 
  45233390
 
  45233773
 
  ENSEMBL   UCSC 
 
  384
 
  4
 
 22665
 
 gene;island
 
 0.032602
 
 0.0637853
 
 Not queried
 
  0.7752514
 
       NA
 
           NA
  
 
  
 11
 
   1096283
 
   1096455
 
  ENSEMBL   UCSC 
 
  173
 
  4
 
 6404
 
 gene;island
 
 0.741156
 
 0.8884902
 
 Not queried
 
  0.7752466
 
       NA
 
           NA
  
 
  
 1
 
   1655803
 
   1655942
 
  ENSEMBL   UCSC 
 
  140
 
  4
 
 1159
 
 tss
 
 0.019163
 
 0.0161994
 
 Not queried
 
  0.7751902
 
       NA
 
           NA
  
 
  
 6
 
  29973221
 
  29973669
 
  ENSEMBL   UCSC 
 
  449
 
  5
 
 33705
 
 gene;tss;island
 
 0.960960
 
 0.9668567
 
 Not queried
 
  0.7751497
 
       NA
 
           NA
  
 
  
 19
 
  51416010
 
  51416213
 
  ENSEMBL   UCSC 
 
  204
 
  4
 
 22329
 
 island
 
 0.008371
 
 0.0117170
 
 Not queried
 
  0.7751116
 
       NA
 
           NA
  
 
  
 3
 
  43020791
 
  43020952
 
  ENSEMBL   UCSC 
 
  162
 
  4
 
 27737
 
 gene
 
 0.037409
 
 0.0231519
 
 Not queried
 
  0.7749812
 
       NA
 
           NA
  
 
  
 4
 
  87797502
 
  87797676
 
  ENSEMBL   UCSC 
 
  175
 
  4
 
 30051
 
 gene
 
 0.806543
 
 0.8536630
 
 Not queried
 
  0.7748037
 
       NA
 
           NA
  
 
  
 17
 
  36105117
 
  36105517
 
  ENSEMBL   UCSC 
 
  401
 
  6
 
 17291
 
 tss
 
 0.006476
 
 0.0053551
 
 Not queried
 
  0.7747213
 
       NA
 
           NA
  
 
  
 17
 
  78444348
 
  78444761
 
  ENSEMBL   UCSC 
 
  414
 
  5
 
 16869
 
 gene;island
 
 0.590373
 
 0.5993660
 
 Not queried
 
  0.7746275
 
       NA
 
           NA
  
 
  
 5
 
 148931071
 
 148931367
 
  ENSEMBL   UCSC 
 
  297
 
  8
 
 32419
 
 tss;island
 
 0.001888
 
 0.0011442
 
 Not queried
 
  0.7746092
 
       NA
 
           NA
  
 
  
 6
 
 118241243
 
 118241659
 
  ENSEMBL   UCSC 
 
  417
 
  5
 
 34456
 
 gene;island
 
 0.036353
 
 0.0199988
 
 Not queried
 
  0.7746023
 
       NA
 
           NA
  
 
  
 11
 
   1483731
 
   1483973
 
  ENSEMBL   UCSC 
 
  243
 
  4
 
 7926
 
 island
 
 0.892741
 
 0.9490178
 
 Not queried
 
  0.7742636
 
       NA
 
           NA
  
 
  
 11
 
  15962841
 
  15963167
 
  ENSEMBL   UCSC 
 
  327
 
  5
 
 8086
 
 island
 
 0.265400
 
 0.5523095
 
 Not queried
 
  0.7741063
 
       NA
 
           NA
  
 
  
 16
 
  30441409
 
  30442009
 
  ENSEMBL   UCSC 
 
  601
 
  6
 
 15184
 
 tss
 
 0.018901
 
 0.0643724
 
 Not queried
 
  0.7739916
 
       NA
 
           NA
  
 
  
 21
 
  11098970
 
  11099431
 
  ENSEMBL   UCSC 
 
  462
 
  4
 
 26484
 
 tss
 
 0.545301
 
 0.5816551
 
 Not queried
 
  0.7738347
 
       NA
 
           NA
  
 
  
 17
 
  46755422
 
  46755786
 
  ENSEMBL   UCSC 
 
  365
 
  4
 
 18496
 
 island
 
 0.161542
 
 0.2115493
 
 Not queried
 
  0.7737545
 
       NA
 
           NA
  
 
  
 3
 
 138763670
 
 138764193
 
  ENSEMBL   UCSC 
 
  524
 
  8
 
 29534
 
 island
 
 0.857064
 
 0.6249850
 
 Not queried
 
  0.7737449
 
       NA
 
           NA
  
 
  
 8
 
  30240920
 
  30241661
 
  ENSEMBL   UCSC 
 
  742
 
  8
 
 39631
 
 tss;island
 
 0.038326
 
 0.0236699
 
 Not queried
 
  0.7737385
 
       NA
 
           NA
  
 
  
 7
 
   1199903
 
   1200143
 
  ENSEMBL   UCSC 
 
  241
 
  5
 
 37397
 
 tss
 
 0.009576
 
 0.0052346
 
 Not queried
 
  0.7732926
 
       NA
 
           NA
  
 
  
 7
 
   1199841
 
   1200143
 
  ENSEMBL   UCSC 
 
  303
 
  7
 
 38087
 
 island
 
 0.009673
 
 0.0052346
 
 Not queried
 
  0.7732926
 
       NA
 
           NA
  
 
  
 17
 
  35294472
 
  35294740
 
  ENSEMBL   UCSC 
 
  269
 
  7
 
 18237
 
 island
 
 0.005877
 
 0.0054550
 
 Not queried
 
  0.7730984
 
       NA
 
           NA
  
 
  
 15
 
  93199037
 
  93199180
 
  ENSEMBL   UCSC 
 
  144
 
  5
 
 13912
 
 tss
 
 0.027215
 
 0.0189241
 
 Not queried
 
  0.7730771
 
       NA
 
           NA
  
 
  
 12
 
  89746328
 
  89746771
 
  ENSEMBL   UCSC 
 
  444
 
  8
 
 9906
 
 tss
 
 0.033292
 
 0.0102958
 
 Not queried
 
  0.7730519
 
       NA
 
           NA
  
 
  
 19
 
  54415702
 
  54416165
 
  ENSEMBL   UCSC 
 
  464
 
  7
 
 22390
 
 island
 
 0.255461
 
 0.3533334
 
 Not queried
 
  0.7729374
 
       NA
 
           NA
  
 
  
 19
 
   9929784
 
   9929951
 
  ENSEMBL   UCSC 
 
  168
 
  7
 
 20431
 
 tss
 
 0.003523
 
 0.0088971
 
 Not queried
 
  0.7727526
 
       NA
 
           NA
  
 
  
 1
 
  45769308
 
  45769727
 
  ENSEMBL   UCSC 
 
  420
 
  7
 
 3197
 
 island
 
 0.013596
 
 0.0060935
 
 Not queried
 
  0.7725865
 
       NA
 
           NA
  
 
  
 7
 
  12726089
 
  12726571
 
  ENSEMBL   UCSC 
 
  483
 
  8
 
 37446
 
 tss;island
 
 0.010917
 
 0.0103233
 
 Not queried
 
  0.7725252
 
       NA
 
           NA
  
 
  
 1
 
  70385100
 
  70385450
 
  ENSEMBL   UCSC 
 
  351
 
  5
 
 577
 
 gene;island
 
 0.602345
 
 0.8553599
 
 Not queried
 
  0.7724542
 
       NA
 
           NA
  
 
  
 2
 
  97202260
 
  97202475
 
  ENSEMBL   UCSC 
 
  216
 
  5
 
 24528
 
 island
 
 0.003039
 
 0.0064634
 
 Not queried
 
  0.7722344
 
       NA
 
           NA
  
 
  
 13
 
 113299697
 
 113300120
 
  ENSEMBL   UCSC 
 
  424
 
  5
 
 11928
 
 island
 
 0.852707
 
 0.8924995
 
 Not queried
 
  0.7722246
 
       NA
 
           NA
  
 
  
 10
 
 131412507
 
 131412764
 
  ENSEMBL   UCSC 
 
  258
 
  4
 
 4828
 
 gene
 
 0.272750
 
 0.3875850
 
 Not queried
 
  0.7721731
 
       NA
 
           NA
  
 
  
 6
 
 106773518
 
 106774172
 
  ENSEMBL   UCSC 
 
  655
 
 10
 
 36382
 
 island
 
 0.014553
 
 0.0155722
 
 Not queried
 
  0.7720464
 
       NA
 
           NA
  
 
  
 1
 
  86174060
 
  86174240
 
  ENSEMBL   UCSC 
 
  181
 
  7
 
 3428
 
 island
 
 0.002166
 
 0.0040501
 
 Not queried
 
  0.7716650
 
       NA
 
           NA
  
 
  
 17
 
  72306008
 
  72306215
 
  ENSEMBL   UCSC 
 
  208
 
  4
 
 16750
 
 gene
 
 0.282400
 
 0.4598481
 
 Not queried
 
  0.7716314
 
       NA
 
           NA
  
 
  
 11
 
 124632245
 
 124632552
 
  ENSEMBL   UCSC 
 
  308
 
  4
 
 7785
 
 tss
 
 0.018774
 
 0.0107231
 
 Not queried
 
  0.7716119
 
       NA
 
           NA
  
 
  
 5
 
 162932455
 
 162932808
 
  ENSEMBL   UCSC 
 
  354
 
  6
 
 31883
 
 gene;island
 
 0.007665
 
 0.0042523
 
 Not queried
 
  0.7715901
 
       NA
 
           NA
  
 
  
 5
 
  65222276
 
  65222365
 
  ENSEMBL   UCSC 
 
   90
 
  4
 
 32098
 
 tss
 
 0.022277
 
 0.0185208
 
 Not queried
 
  0.7715221
 
       NA
 
           NA
  
 
  
 3
 
 182817190
 
 182817338
 
  ENSEMBL   UCSC 
 
  149
 
  4
 
 28048
 
 gene
 
 0.001903
 
 0.0038325
 
 Not queried
 
  0.7714374
 
       NA
 
           NA
  
 
  
 3
 
   5024761
 
   5024963
 
  ENSEMBL   UCSC 
 
  203
 
  5
 
 27644
 
 gene;island
 
 0.967687
 
 0.9581178
 
 Not queried
 
  0.7712339
 
       NA
 
           NA
  
 
  
 1
 
 213189048
 
 213189260
 
  ENSEMBL   UCSC 
 
  213
 
  6
 
 4191
 
 island
 
 0.008324
 
 0.0052619
 
 Not queried
 
  0.7711888
 
       NA
 
           NA
  
 
  
 2
 
 148602278
 
 148602504
 
  ENSEMBL   UCSC 
 
  227
 
  4
 
 23654
 
 tss
 
 0.039490
 
 0.0121360
 
 Not queried
 
  0.7710434
 
       NA
 
           NA
  
 
  
 16
 
  67260661
 
  67260896
 
  ENSEMBL   UCSC 
 
  236
 
  6
 
 14794
 
 gene
 
 0.011564
 
 0.0081500
 
 Not queried
 
  0.7709319
 
       NA
 
           NA
  
 
  
 1
 
   6546574
 
   6546839
 
  ENSEMBL   UCSC 
 
  266
 
  5
 
 176
 
 gene;tss;island
 
 0.016449
 
 0.0069844
 
 Not queried
 
  0.7709167
 
       NA
 
           NA
  
 
  
 17
 
  80189962
 
  80190154
 
  ENSEMBL   UCSC 
 
  193
 
  7
 
 16940
 
 gene;island
 
 0.087955
 
 0.2718838
 
 Not queried
 
  0.7708869
 
       NA
 
           NA
  
 
  
 15
 
  67546975
 
  67547294
 
  ENSEMBL   UCSC 
 
  320
 
  8
 
 13394
 
 gene;tss;island
 
 0.003263
 
 0.0022377
 
 Not queried
 
  0.7708866
 
       NA
 
           NA
  
 
  
 22
 
  38479099
 
  38479332
 
  ENSEMBL   UCSC 
 
  234
 
  7
 
 27480
 
 island
 
 0.844358
 
 0.9234306
 
 Not queried
 
  0.7706798
 
       NA
 
           NA
  
 
  
 1
 
    870791
 
    871057
 
  ENSEMBL   UCSC 
 
  267
 
  5
 
 2
 
 gene;island
 
 0.219924
 
 0.3247678
 
 Not queried
 
  0.7706238
 
       NA
 
           NA
  
 
  
 14
 
  51411267
 
  51411647
 
  ENSEMBL   UCSC 
 
  381
 
  7
 
 12390
 
 tss
 
 0.005786
 
 0.0039844
 
 Not queried
 
  0.7703587
 
       NA
 
           NA
  
 
  
 2
 
 241562424
 
 241562898
 
  ENSEMBL   UCSC 
 
  475
 
  6
 
 25276
 
 island
 
 0.293960
 
 0.3680331
 
 Not queried
 
  0.7702961
 
       NA
 
           NA
  
 
  
 13
 
 111358803
 
 111358924
 
  ENSEMBL   UCSC 
 
  122
 
  4
 
 11536
 
 tss;island
 
 0.064115
 
 0.0562157
 
 Not queried
 
  0.7701946
 
       NA
 
           NA
  
 
  
 17
 
   7297619
 
   7298077
 
  ENSEMBL   UCSC 
 
  459
 
  6
 
 17943
 
 island
 
 0.007757
 
 0.0048436
 
 Not queried
 
  0.7701325
 
       NA
 
           NA
  
 
  
 6
 
 170919283
 
 170919448
 
  ENSEMBL   UCSC 
 
  166
 
  4
 
 36700
 
 island
 
 0.816929
 
 0.8842865
 
 Not queried
 
  0.7701230
 
       NA
 
           NA
  
 
  
 1
 
 119542159
 
 119542629
 
  ENSEMBL   UCSC 
 
  471
 
  6
 
 3659
 
 island
 
 0.079276
 
 0.2175627
 
 Not queried
 
  0.7699978
 
       NA
 
           NA
  
 
  
 1
 
 147379964
 
 147380445
 
  ENSEMBL   UCSC 
 
  482
 
  6
 
 732
 
 gene;island
 
 0.649207
 
 0.7717908
 
 Not queried
 
  0.7697464
 
       NA
 
           NA
  
 
  
 1
 
  39339004
 
  39339683
 
  ENSEMBL   UCSC 
 
  680
 
 11
 
 3095
 
 island
 
 0.009010
 
 0.0074571
 
 Not queried
 
  0.7695604
 
       NA
 
           NA
  
 
  
 6
 
  10694545
 
  10694962
 
  ENSEMBL   UCSC 
 
  418
 
  9
 
 33564
 
 gene
 
 0.005761
 
 0.0050283
 
 Not queried
 
  0.7694491
 
       NA
 
           NA
  
 
  
 6
 
  10694545
 
  10695389
 
  ENSEMBL   UCSC 
 
  845
 
 19
 
 34628
 
 tss;island
 
 0.012966
 
 0.0050283
 
 Not queried
 
  0.7694491
 
       NA
 
           NA
  
 
  
 3
 
  40518669
 
  40518798
 
  ENSEMBL   UCSC 
 
  130
 
  4
 
 27728
 
 gene
 
 0.003773
 
 0.0049872
 
 Not queried
 
  0.7693196
 
       NA
 
           NA
  
 
  
 1
 
  21031009
 
  21031312
 
  ENSEMBL   UCSC 
 
  304
 
  4
 
 293
 
 gene;island
 
 0.852696
 
 0.8891556
 
 Not queried
 
  0.7693078
 
       NA
 
           NA
  
 
  
 7
 
  43622300
 
  43622659
 
  ENSEMBL   UCSC 
 
  360
 
  7
 
 37556
 
 tss;island
 
 0.006953
 
 0.0032723
 
 Not queried
 
  0.7692857
 
       NA
 
           NA
  
 
  
 1
 
   1395945
 
   1396296
 
  ENSEMBL   UCSC 
 
  352
 
  7
 
 37
 
 gene;island
 
 0.127990
 
 0.1326916
 
 Not queried
 
  0.7692433
 
       NA
 
           NA
  
 
  
 15
 
  45003702
 
  45003832
 
  ENSEMBL   UCSC 
 
  131
 
  4
 
 13324
 
 gene
 
 0.001887
 
 0.0019840
 
 Not queried
 
  0.7692253
 
       NA
 
           NA
  
 
  
 10
 
 131323903
 
 131324189
 
  ENSEMBL   UCSC 
 
  287
 
  4
 
 4824
 
 gene
 
 0.737725
 
 0.8304403
 
 Not queried
 
  0.7691712
 
       NA
 
           NA
  
 
  
 20
 
   2644865
 
   2645106
 
  ENSEMBL   UCSC 
 
  242
 
  9
 
 25550
 
 tss
 
 0.008997
 
 0.0046931
 
 Not queried
 
  0.7688839
 
       NA
 
           NA
  
 
  
 18
 
   5543801
 
   5544237
 
  ENSEMBL   UCSC 
 
  437
 
 11
 
 19375
 
 island
 
 0.026784
 
 0.0484025
 
 Not queried
 
  0.7687593
 
       NA
 
           NA
  
 
  
 14
 
  23291749
 
  23292277
 
  ENSEMBL   UCSC 
 
  529
 
  5
 
 12701
 
 island
 
 0.009321
 
 0.0047246
 
 Not queried
 
  0.7687537
 
       NA
 
           NA
  
 
  
 1
 
   1976394
 
   1976627
 
  ENSEMBL   UCSC 
 
  234
 
  4
 
 2470
 
 island
 
 0.010815
 
 0.0067084
 
 Not queried
 
  0.7687529
 
       NA
 
           NA
  
 
  
 11
 
 122073862
 
 122074089
 
  ENSEMBL   UCSC 
 
  228
 
  6
 
 7772
 
 tss
 
 0.049910
 
 0.0541682
 
 Not queried
 
  0.7682178
 
       NA
 
           NA
  
 
  
 20
 
  48960165
 
  48960348
 
  ENSEMBL   UCSC 
 
  184
 
  4
 
 26247
 
 island
 
 0.970277
 
 0.9439194
 
 Not queried
 
  0.7681579
 
       NA
 
           NA
  
 
  
 22
 
  21271315
 
  21271664
 
  ENSEMBL   UCSC 
 
  350
 
  6
 
 26984
 
 tss
 
 0.003504
 
 0.0045781
 
 Not queried
 
  0.7681300
 
       NA
 
           NA
  
 
  
 4
 
    647194
 
    647480
 
  ENSEMBL   UCSC 
 
  287
 
  4
 
 29830
 
 gene;island
 
 0.420003
 
 0.4835236
 
 Not queried
 
  0.7677074
 
       NA
 
           NA
  
 
  
 2
 
 136633963
 
 136634366
 
  ENSEMBL   UCSC 
 
  404
 
  9
 
 24755
 
 island
 
 0.002096
 
 0.0024364
 
 Not queried
 
  0.7676682
 
       NA
 
           NA
  
 
  
 4
 
 190906046
 
 190906123
 
  ENSEMBL   UCSC 
 
   78
 
  5
 
 30712
 
 tss
 
 0.868848
 
 0.7257947
 
 Not queried
 
  0.7676261
 
       NA
 
           NA
  
 
  
 4
 
 190905801
 
 190906123
 
  ENSEMBL   UCSC 
 
  323
 
  7
 
 31495
 
 island
 
 0.698126
 
 0.6121670
 
 Not queried
 
  0.7676261
 
       NA
 
           NA
  
 
  
 2
 
 211341126
 
 211341483
 
  ENSEMBL   UCSC 
 
  358
 
  7
 
 23004
 
 gene
 
 0.005799
 
 0.0010818
 
 Not queried
 
  0.7675309
 
       NA
 
           NA
  
 
  
 2
 
 211341126
 
 211341572
 
  ENSEMBL   UCSC 
 
  447
 
  9
 
 23856
 
 tss;island
 
 0.005799
 
 0.0019846
 
 Not queried
 
  0.7675309
 
       NA
 
           NA
  
 
  
 14
 
  61190762
 
  61191253
 
  ENSEMBL   UCSC 
 
  492
 
  7
 
 12912
 
 island
 
 0.010995
 
 0.0254698
 
 Not queried
 
  0.7673653
 
       NA
 
           NA
  
 
  
 11
 
  94226767
 
  94227038
 
  ENSEMBL   UCSC 
 
  272
 
  5
 
 6865
 
 gene
 
 0.004758
 
 0.0042870
 
 Not queried
 
  0.7672676
 
       NA
 
           NA
  
 
  
 19
 
  19144494
 
  19144964
 
  ENSEMBL   UCSC 
 
  471
 
  5
 
 19945
 
 gene
 
 0.002953
 
 0.0042458
 
 Not queried
 
  0.7671354
 
       NA
 
           NA
  
 
  
 4
 
  85419494
 
  85419557
 
  ENSEMBL   UCSC 
 
   64
 
  6
 
 30479
 
 tss
 
 0.074752
 
 0.0393571
 
 Not queried
 
  0.7669344
 
       NA
 
           NA
  
 
  
 4
 
  85419151
 
  85419557
 
  ENSEMBL   UCSC 
 
  407
 
  8
 
 31161
 
 island
 
 0.055471
 
 0.0393571
 
 Not queried
 
  0.7669344
 
       NA
 
           NA
  
 
  
 5
 
  92938149
 
  92938402
 
  ENSEMBL   UCSC 
 
  254
 
  4
 
 32963
 
 island
 
 0.228101
 
 0.2807504
 
 Not queried
 
  0.7667949
 
       NA
 
           NA
  
 
  
 19
 
  56739573
 
  56739868
 
  ENSEMBL   UCSC 
 
  296
 
  7
 
 22441
 
 island
 
 0.854546
 
 0.9425537
 
 Not queried
 
  0.7666576
 
       NA
 
           NA
  
 
  
 6
 
  29978314
 
  29978637
 
  ENSEMBL   UCSC 
 
  324
 
  5
 
 33708
 
 gene;island
 
 0.863653
 
 0.9746080
 
 Not queried
 
  0.7665076
 
       NA
 
           NA
  
 
  
 2
 
    264120
 
    264204
 
  ENSEMBL   UCSC 
 
   85
 
  6
 
 23152
 
 tss;island
 
 0.005485
 
 0.0054633
 
 Not queried
 
  0.7664093
 
       NA
 
           NA
  
 
  
 7
 
 116594421
 
 116594693
 
  ENSEMBL   UCSC 
 
  273
 
  6
 
 37199
 
 gene;tss;island
 
 0.002962
 
 0.0041342
 
 Not queried
 
  0.7662540
 
       NA
 
           NA
  
 
  
 4
 
 109092628
 
 109092918
 
  ENSEMBL   UCSC 
 
  291
 
  4
 
 30085
 
 gene;tss;island
 
 0.017347
 
 0.0128359
 
 Not queried
 
  0.7662278
 
       NA
 
           NA
  
 
  
 19
 
  56904442
 
  56905152
 
  ENSEMBL   UCSC 
 
  711
 
 12
 
 22444
 
 island
 
 0.046607
 
 0.0170819
 
 Not queried
 
  0.7659971
 
       NA
 
           NA
  
 
  
 8
 
 107227878
 
 107228203
 
  ENSEMBL   UCSC 
 
  326
 
  4
 
 40526
 
 island
 
 0.487975
 
 0.6219600
 
 Not queried
 
  0.7659936
 
       NA
 
           NA
  
 
  
 13
 
  21047387
 
  21047671
 
  ENSEMBL   UCSC 
 
  285
 
  5
 
 11098
 
 gene
 
 0.951256
 
 0.9604267
 
 Not queried
 
  0.7659048
 
       NA
 
           NA
  
 
  
 12
 
  14996508
 
  14996776
 
  ENSEMBL   UCSC 
 
  269
 
  8
 
 9588
 
 tss
 
 0.857597
 
 0.9141523
 
 Not queried
 
  0.7655633
 
       NA
 
           NA
  
 
  
 2
 
    503065
 
    503383
 
  ENSEMBL   UCSC 
 
  319
 
  4
 
 24005
 
 island
 
 0.850788
 
 0.9114576
 
 Not queried
 
  0.7653777
 
       NA
 
           NA
  
 
  
 12
 
  57881856
 
  57882021
 
  ENSEMBL   UCSC 
 
  166
 
  4
 
 9193
 
 gene
 
 0.009457
 
 0.0104360
 
 Not queried
 
  0.7653720
 
       NA
 
           NA
  
 
  
 11
 
  68781976
 
  68782211
 
  ENSEMBL   UCSC 
 
  236
 
  5
 
 7516
 
 tss;island
 
 0.624994
 
 0.8239237
 
 Not queried
 
  0.7653683
 
       NA
 
           NA
  
 
  
 11
 
   2151676
 
   2151725
 
  ENSEMBL   UCSC 
 
   50
 
  4
 
 6444
 
 gene;island
 
 0.651198
 
 0.4465064
 
 Not queried
 
  0.7653636
 
       NA
 
           NA
  
 
  
 17
 
  56609534
 
  56609687
 
  ENSEMBL   UCSC 
 
  154
 
  5
 
 17538
 
 tss;island
 
 0.061621
 
 0.0219552
 
 Not queried
 
  0.7653457
 
       NA
 
           NA
  
 
  
 6
 
  41620951
 
  41621322
 
  ENSEMBL   UCSC 
 
  372
 
  6
 
 34331
 
 gene;island
 
 0.479485
 
 0.5666744
 
 Not queried
 
  0.7652833
 
       NA
 
           NA
  
 
  
 1
 
  64239391
 
  64239633
 
  ENSEMBL   UCSC 
 
  243
 
  8
 
 1671
 
 tss;island
 
 0.015354
 
 0.0064974
 
 Not queried
 
  0.7652623
 
       NA
 
           NA
  
 
  
 13
 
  30424821
 
  30424932
 
  ENSEMBL   UCSC 
 
  112
 
  4
 
 11372
 
 tss;island
 
 0.002612
 
 0.0010799
 
 Not queried
 
  0.7651658
 
       NA
 
           NA
  
 
  
 6
 
 122931154
 
 122931308
 
  ENSEMBL   UCSC 
 
  155
 
  8
 
 35206
 
 tss
 
 0.068126
 
 0.0367088
 
 Not queried
 
  0.7651286
 
       NA
 
           NA
  
 
  
 6
 
  10422139
 
  10422636
 
  ENSEMBL   UCSC 
 
  498
 
  5
 
 35434
 
 island
 
 0.098562
 
 0.1687575
 
 Not queried
 
  0.7649043
 
       NA
 
           NA
  
 
  
 14
 
  74253857
 
  74254319
 
  ENSEMBL   UCSC 
 
  463
 
  7
 
 12992
 
 island
 
 0.012655
 
 0.0102668
 
 Not queried
 
  0.7647961
 
       NA
 
           NA
  
 
  
 11
 
  32112091
 
  32112810
 
  ENSEMBL   UCSC 
 
  720
 
  8
 
 8173
 
 island
 
 0.045158
 
 0.2462001
 
 Not queried
 
  0.7647241
 
       NA
 
           NA
  
 
  
 3
 
  73673893
 
  73674702
 
  ENSEMBL   UCSC 
 
  810
 
 15
 
 29286
 
 island
 
 0.506583
 
 0.2122390
 
 Not queried
 
  0.7646430
 
       NA
 
           NA
  
 
  
 18
 
  11751343
 
  11751566
 
  ENSEMBL   UCSC 
 
  224
 
  6
 
 19055
 
 gene;island
 
 0.408740
 
 0.4271986
 
 Not queried
 
  0.7646163
 
       NA
 
           NA
  
 
  
 1
 
  24105080
 
  24105319
 
  ENSEMBL   UCSC 
 
  240
 
  4
 
 319
 
 gene;island
 
 0.007075
 
 0.0053764
 
 Not queried
 
  0.7645205
 
       NA
 
           NA
  
 
  
 5
 
   3188066
 
   3188374
 
  ENSEMBL   UCSC 
 
  309
 
  5
 
 32662
 
 island
 
 0.774394
 
 0.8009536
 
 Not queried
 
  0.7645086
 
       NA
 
           NA
  
 
  
 10
 
 105215053
 
 105215423
 
  ENSEMBL   UCSC 
 
  371
 
  4
 
 4742
 
 gene;island
 
 0.892620
 
 0.9334917
 
 Not queried
 
  0.7644205
 
       NA
 
           NA
  
 
  
 4
 
 187985253
 
 187985472
 
  ENSEMBL   UCSC 
 
  220
 
  4
 
 31489
 
 island
 
 0.741355
 
 0.9037942
 
 Not queried
 
  0.7642548
 
       NA
 
           NA
  
 
  
 6
 
  71377345
 
  71377551
 
  ENSEMBL   UCSC 
 
  207
 
  8
 
 36240
 
 island
 
 0.003123
 
 0.0038188
 
 Not queried
 
  0.7642131
 
       NA
 
           NA
  
 
  
 6
 
   8102731
 
   8103092
 
  ENSEMBL   UCSC 
 
  362
 
 11
 
 35419
 
 island
 
 0.017507
 
 0.0057721
 
 Not queried
 
  0.7641837
 
       NA
 
           NA
  
 
  
 10
 
  79470767
 
  79471309
 
  ENSEMBL   UCSC 
 
  543
 
  5
 
 5873
 
 island
 
 0.020750
 
 0.0699397
 
 Not queried
 
  0.7641747
 
       NA
 
           NA
  
 
  
 7
 
  93550986
 
  93551014
 
  ENSEMBL   UCSC 
 
   29
 
  4
 
 37716
 
 tss
 
 0.071886
 
 0.0476522
 
 Not queried
 
  0.7641570
 
       NA
 
           NA
  
 
  
 16
 
  86542659
 
  86543092
 
  ENSEMBL   UCSC 
 
  434
 
 10
 
 15400
 
 tss;island
 
 0.010046
 
 0.0169282
 
 Not queried
 
  0.7639071
 
       NA
 
           NA
  
 
  
 7
 
  32109831
 
  32110239
 
  ENSEMBL   UCSC 
 
  409
 
  4
 
 36958
 
 gene;island
 
 0.223098
 
 0.2589667
 
 Not queried
 
  0.7638950
 
       NA
 
           NA
  
 
  
 3
 
  82857026
 
  82857285
 
  ENSEMBL   UCSC 
 
  260
 
  4
 
 29296
 
 island
 
 0.859166
 
 0.6953616
 
 Not queried
 
  0.7638477
 
       NA
 
           NA
  
 
  
 2
 
 131485095
 
 131485486
 
  ENSEMBL   UCSC 
 
  392
 
  6
 
 24721
 
 island
 
 0.008632
 
 0.0054001
 
 Not queried
 
  0.7637794
 
       NA
 
           NA
  
 
  
 10
 
 128193219
 
 128193497
 
  ENSEMBL   UCSC 
 
  279
 
  5
 
 4817
 
 gene;island
 
 0.367401
 
 0.4343530
 
 Not queried
 
  0.7636336
 
       NA
 
           NA
  
 
  
 7
 
   1119827
 
   1120247
 
  ENSEMBL   UCSC 
 
  421
 
  4
 
 36746
 
 gene;island
 
 0.955983
 
 0.9783996
 
 Not queried
 
  0.7635978
 
       NA
 
           NA
  
 
  
 12
 
  50222071
 
  50222202
 
  ENSEMBL   UCSC 
 
  132
 
  4
 
 9125
 
 gene
 
 0.016228
 
 0.0170419
 
 Not queried
 
  0.7635424
 
       NA
 
           NA
  
 
  
 19
 
   8643540
 
   8643798
 
  ENSEMBL   UCSC 
 
  259
 
  4
 
 20421
 
 tss
 
 0.043595
 
 0.0272928
 
 Not queried
 
  0.7634669
 
       NA
 
           NA
  
 
  
 10
 
 127586397
 
 127586670
 
  ENSEMBL   UCSC 
 
  274
 
  6
 
 4813
 
 gene;island
 
 0.841992
 
 0.8996610
 
 Not queried
 
  0.7633622
 
       NA
 
           NA
  
 
  
 1
 
  26633206
 
  26633632
 
  ENSEMBL   UCSC 
 
  427
 
 10
 
 1389
 
 tss
 
 0.005300
 
 0.0086084
 
 Not queried
 
  0.7629607
 
       NA
 
           NA
  
 
  
 12
 
  50898249
 
  50899018
 
  ENSEMBL   UCSC 
 
  770
 
 11
 
 10416
 
 island
 
 0.010592
 
 0.0049370
 
 Not queried
 
  0.7627962
 
       NA
 
           NA
  
 
  
 4
 
 175443243
 
 175443867
 
  ENSEMBL   UCSC 
 
  625
 
  6
 
 30184
 
 gene;island
 
 0.016503
 
 0.0088266
 
 Not queried
 
  0.7627731
 
       NA
 
           NA
  
 
  
 10
 
  38265481
 
  38265937
 
  ENSEMBL   UCSC 
 
  457
 
  8
 
 5034
 
 tss;island
 
 0.006385
 
 0.0025305
 
 Not queried
 
  0.7626609
 
       NA
 
           NA
  
 
  
 16
 
  85518792
 
  85519308
 
  ENSEMBL   UCSC 
 
  517
 
  8
 
 16157
 
 island
 
 0.674101
 
 0.7290825
 
 Not queried
 
  0.7626138
 
       NA
 
           NA
  
 
  
 7
 
  19184950
 
  19185407
 
  ENSEMBL   UCSC 
 
  458
 
  7
 
 38287
 
 island
 
 0.048476
 
 0.0377959
 
 Not queried
 
  0.7624931
 
       NA
 
           NA
  
 
  
 16
 
  34407715
 
  34408014
 
  ENSEMBL   UCSC 
 
  300
 
  5
 
 15892
 
 island
 
 0.568283
 
 0.6018673
 
 Not queried
 
  0.7623308
 
       NA
 
           NA
  
 
  
 18
 
  61143926
 
  61143993
 
  ENSEMBL   UCSC 
 
   68
 
  5
 
 19312
 
 tss
 
 0.760609
 
 0.9164253
 
 Not queried
 
  0.7621469
 
       NA
 
           NA
  
 
  
 14
 
  38677095
 
  38677295
 
  ENSEMBL   UCSC 
 
  201
 
  4
 
 12801
 
 island
 
 0.011532
 
 0.0063348
 
 Not queried
 
  0.7620629
 
       NA
 
           NA
  
 
  
 1
 
 156897330
 
 156897930
 
  ENSEMBL   UCSC 
 
  601
 
  6
 
 834
 
 gene;island
 
 0.016578
 
 0.0259862
 
 Not queried
 
  0.7620508
 
       NA
 
           NA
  
 
  
 7
 
 102105124
 
 102105256
 
  ENSEMBL   UCSC 
 
  133
 
  5
 
 37168
 
 gene
 
 0.002636
 
 0.0034733
 
 Not queried
 
  0.7619760
 
       NA
 
           NA
  
 
  
 15
 
  58357874
 
  58357989
 
  ENSEMBL   UCSC 
 
  116
 
  7
 
 14175
 
 island
 
 0.011426
 
 0.0081261
 
 Not queried
 
  0.7617735
 
       NA
 
           NA
  
 
  
 2
 
  46612724
 
  46613024
 
  ENSEMBL   UCSC 
 
  301
 
  4
 
 22666
 
 gene
 
 0.394420
 
 0.5458777
 
 Not queried
 
  0.7617227
 
       NA
 
           NA
  
 
  
 19
 
  18313289
 
  18313561
 
  ENSEMBL   UCSC 
 
  273
 
  4
 
 19930
 
 gene;island
 
 0.975302
 
 0.9755833
 
 Not queried
 
  0.7616519
 
       NA
 
           NA
  
 
  
 13
 
  24462720
 
  24463018
 
  ENSEMBL   UCSC 
 
  299
 
  8
 
 11104
 
 gene;tss;island
 
 0.005647
 
 0.0032520
 
 Not queried
 
  0.7616249
 
       NA
 
           NA
  
 
  
 18
 
  23670518
 
  23670778
 
  ENSEMBL   UCSC 
 
  261
 
  4
 
 19438
 
 island
 
 0.005564
 
 0.0035429
 
 Not queried
 
  0.7614838
 
       NA
 
           NA
  
 
  
 19
 
  30155866
 
  30156658
 
  ENSEMBL   UCSC 
 
  793
 
  7
 
 21854
 
 island
 
 0.007271
 
 0.0028475
 
 Not queried
 
  0.7612048
 
       NA
 
           NA
  
 
  
 4
 
  24914750
 
  24914970
 
  ENSEMBL   UCSC 
 
  221
 
  5
 
 29968
 
 gene;tss
 
 0.068275
 
 0.3304126
 
 Not queried
 
  0.7612008
 
       NA
 
           NA
  
 
  
 1
 
  47799146
 
  47799348
 
  ENSEMBL   UCSC 
 
  203
 
  4
 
 1609
 
 tss;island
 
 0.005214
 
 0.0041820
 
 Not queried
 
  0.7610036
 
       NA
 
           NA
  
 
  
 13
 
 111145564
 
 111145770
 
  ENSEMBL   UCSC 
 
  207
 
  6
 
 11232
 
 gene
 
 0.871925
 
 0.9332073
 
 Not queried
 
  0.7610024
 
       NA
 
           NA
  
 
  
 12
 
 121163144
 
 121163497
 
  ENSEMBL   UCSC 
 
  354
 
  7
 
 10067
 
 tss
 
 0.071787
 
 0.0500962
 
 Not queried
 
  0.7609624
 
       NA
 
           NA
  
 
  
 1
 
 205473048
 
 205473662
 
  ENSEMBL   UCSC 
 
  615
 
  7
 
 2205
 
 tss
 
 0.358575
 
 0.3678225
 
 Not queried
 
  0.7608139
 
       NA
 
           NA
  
 
  
 4
 
 186456837
 
 186457012
 
  ENSEMBL   UCSC 
 
  176
 
  5
 
 30702
 
 tss
 
 0.064443
 
 0.0964686
 
 Not queried
 
  0.7606402
 
       NA
 
           NA
  
 
  
 4
 
 186456397
 
 186457012
 
  ENSEMBL   UCSC 
 
  616
 
  9
 
 31477
 
 island
 
 0.025272
 
 0.0376483
 
 Not queried
 
  0.7606402
 
       NA
 
           NA
  
 
  
 15
 
  32321637
 
  32321944
 
  ENSEMBL   UCSC 
 
  308
 
  4
 
 13551
 
 tss;island
 
 0.024025
 
 0.0141206
 
 Not queried
 
  0.7605752
 
       NA
 
           NA
  
 
  
 16
 
  67198029
 
  67198367
 
  ENSEMBL   UCSC 
 
  339
 
  4
 
 14791
 
 gene;island
 
 0.013484
 
 0.0235792
 
 Not queried
 
  0.7605387
 
       NA
 
           NA
  
 
  
 10
 
  92617735
 
  92618141
 
  ENSEMBL   UCSC 
 
  407
 
  5
 
 5218
 
 tss
 
 0.040193
 
 0.0341917
 
 Not queried
 
  0.7605239
 
       NA
 
           NA
  
 
  
 2
 
 219255879
 
 219256187
 
  ENSEMBL   UCSC 
 
  309
 
  4
 
 23016
 
 gene;island
 
 0.869344
 
 0.8913143
 
 Not queried
 
  0.7604598
 
       NA
 
           NA
  
 
  
 3
 
  52279902
 
  52280139
 
  ENSEMBL   UCSC 
 
  238
 
  5
 
 28376
 
 tss
 
 0.016385
 
 0.0275172
 
 Not queried
 
  0.7604259
 
       NA
 
           NA
  
 
  
 12
 
 122018375
 
 122019117
 
  ENSEMBL   UCSC 
 
  743
 
 14
 
 10074
 
 tss
 
 0.017278
 
 0.0240052
 
 Not queried
 
  0.7602786
 
       NA
 
           NA
  
 
  
 3
 
 132756721
 
 132757973
 
  ENSEMBL   UCSC 
 
 1253
 
 16
 
 29487
 
 island
 
 0.051732
 
 0.1173839
 
 Not queried
 
  0.7602769
 
       NA
 
           NA
  
 
  
 11
 
  87908558
 
  87908938
 
  ENSEMBL   UCSC 
 
  381
 
  9
 
 8719
 
 island
 
 0.019012
 
 0.0199201
 
 Not queried
 
  0.7602727
 
       NA
 
           NA
  
 
  
 12
 
  85430123
 
  85430336
 
  ENSEMBL   UCSC 
 
  214
 
  7
 
 9233
 
 gene
 
 0.001392
 
 0.0016846
 
 Not queried
 
  0.7601795
 
       NA
 
           NA
  
 
  
 14
 
 104636633
 
 104637213
 
  ENSEMBL   UCSC 
 
  581
 
  5
 
 12246
 
 gene;island
 
 0.728397
 
 0.8848128
 
 Not queried
 
  0.7601434
 
       NA
 
           NA
  
 
  
 12
 
  93963179
 
  93963555
 
  ENSEMBL   UCSC 
 
  377
 
 10
 
 9916
 
 tss
 
 0.002570
 
 0.0010806
 
 Not queried
 
  0.7600657
 
       NA
 
           NA
  
 
  
 10
 
  72163475
 
  72164137
 
  ENSEMBL   UCSC 
 
  663
 
  7
 
 5817
 
 island
 
 0.011915
 
 0.0030014
 
 Not queried
 
  0.7600012
 
       NA
 
           NA
  
 
  
 1
 
   8021250
 
   8021611
 
  ENSEMBL   UCSC 
 
  362
 
  6
 
 1219
 
 tss
 
 0.003584
 
 0.0040628
 
 Not queried
 
  0.7595295
 
       NA
 
           NA
  
 
  
 1
 
   8021250
 
   8021962
 
  ENSEMBL   UCSC 
 
  713
 
 10
 
 2631
 
 island
 
 0.003584
 
 0.0044997
 
 Not queried
 
  0.7595295
 
       NA
 
           NA
  
 
  
 1
 
  12655992
 
  12656315
 
  ENSEMBL   UCSC 
 
  324
 
  4
 
 234
 
 gene;island
 
 0.542024
 
 0.5510806
 
 Not queried
 
  0.7595158
 
       NA
 
           NA
  
 
  
 14
 
  50698328
 
  50698997
 
  ENSEMBL   UCSC 
 
  670
 
  6
 
 12385
 
 tss;island
 
 0.003413
 
 0.0020714
 
 Not queried
 
  0.7595051
 
       NA
 
           NA
  
 
  
 2
 
 232825958
 
 232826275
 
  ENSEMBL   UCSC 
 
  318
 
  7
 
 23948
 
 tss
 
 0.015954
 
 0.0096758
 
 Not queried
 
  0.7592920
 
       NA
 
           NA
  
 
  
 1
 
 215256254
 
 215256451
 
  ENSEMBL   UCSC 
 
  198
 
  4
 
 2253
 
 tss
 
 0.018465
 
 0.0077462
 
 Not queried
 
  0.7592407
 
       NA
 
           NA
  
 
  
 10
 
  85997177
 
  85997517
 
  ENSEMBL   UCSC 
 
  341
 
  4
 
 4657
 
 gene;island
 
 0.367096
 
 0.4434682
 
 Not queried
 
  0.7590404
 
       NA
 
           NA
  
 
  
 16
 
  82660596
 
  82660873
 
  ENSEMBL   UCSC 
 
  278
 
  5
 
 14858
 
 gene
 
 0.087916
 
 0.0205536
 
 Not queried
 
  0.7586190
 
       NA
 
           NA
  
 
  
 11
 
 109292894
 
 109293216
 
  ENSEMBL   UCSC 
 
  323
 
  4
 
 6892
 
 gene
 
 0.042006
 
 0.0285463
 
 Not queried
 
  0.7586073
 
       NA
 
           NA
  
 
  
 3
 
   4534791
 
   4535021
 
  ENSEMBL   UCSC 
 
  231
 
  7
 
 28117
 
 tss
 
 0.005179
 
 0.0049029
 
 Not queried
 
  0.7586003
 
       NA
 
           NA
  
 
  
 13
 
  34391699
 
  34392163
 
  ENSEMBL   UCSC 
 
  465
 
  8
 
 11388
 
 tss
 
 0.007660
 
 0.0099793
 
 Not queried
 
  0.7585129
 
       NA
 
           NA
  
 
  
 2
 
  17935443
 
  17935919
 
  ENSEMBL   UCSC 
 
  477
 
  5
 
 22584
 
 gene;tss;island
 
 0.006489
 
 0.0043989
 
 Not queried
 
  0.7583657
 
       NA
 
           NA
  
 
  
 19
 
  45663415
 
  45663527
 
  ENSEMBL   UCSC 
 
  113
 
  4
 
 20862
 
 tss
 
 0.440846
 
 0.5446321
 
 Not queried
 
  0.7583284
 
       NA
 
           NA
  
 
  
 5
 
   5422589
 
   5422799
 
  ENSEMBL   UCSC 
 
  211
 
  5
 
 31980
 
 tss
 
 0.005542
 
 0.0051602
 
 Not queried
 
  0.7582658
 
       NA
 
           NA
  
 
  
 12
 
  54812824
 
  54813155
 
  ENSEMBL   UCSC 
 
  332
 
  5
 
 10505
 
 island
 
 0.038086
 
 0.0262081
 
 Not queried
 
  0.7580409
 
       NA
 
           NA
  
 
  
 1
 
 155827015
 
 155827082
 
  ENSEMBL   UCSC 
 
   68
 
  4
 
 2004
 
 tss
 
 0.004119
 
 0.0067220
 
 Not queried
 
  0.7580048
 
       NA
 
           NA
  
 
  
 14
 
  55907007
 
  55907501
 
  ENSEMBL   UCSC 
 
  495
 
 10
 
 12869
 
 island
 
 0.490440
 
 0.5541476
 
 Not queried
 
  0.7579977
 
       NA
 
           NA
  
 
  
 11
 
  46389164
 
  46389363
 
  ENSEMBL   UCSC 
 
  200
 
  4
 
 6600
 
 gene;island
 
 0.101805
 
 0.0954670
 
 Not queried
 
  0.7577482
 
       NA
 
           NA
  
 
  
 15
 
  68098945
 
  68099144
 
  ENSEMBL   UCSC 
 
  200
 
  4
 
 13395
 
 gene
 
 0.943600
 
 0.9233083
 
 Not queried
 
  0.7576886
 
       NA
 
           NA
  
 
  
 8
 
 127570482
 
 127570908
 
  ENSEMBL   UCSC 
 
  427
 
  6
 
 39911
 
 tss
 
 0.024109
 
 0.0466549
 
 Not queried
 
  0.7573030
 
       NA
 
           NA
  
 
  
 22
 
  46692687
 
  46692881
 
  ENSEMBL   UCSC 
 
  195
 
  4
 
 26911
 
 gene
 
 0.004287
 
 0.0036761
 
 Not queried
 
  0.7572685
 
       NA
 
           NA
  
 
  
 14
 
  35008500
 
  35008877
 
  ENSEMBL   UCSC 
 
  378
 
  5
 
 12060
 
 gene;island
 
 0.007947
 
 0.0063634
 
 Not queried
 
  0.7571380
 
       NA
 
           NA
  
 
  
 7
 
 157294324
 
 157294502
 
  ENSEMBL   UCSC 
 
  179
 
  4
 
 39105
 
 island
 
 0.570575
 
 0.5912039
 
 Not queried
 
  0.7570135
 
       NA
 
           NA
  
 
  
 10
 
 102671993
 
 102672311
 
  ENSEMBL   UCSC 
 
  319
 
  7
 
 5298
 
 tss
 
 0.007050
 
 0.0099628
 
 Not queried
 
  0.7569075
 
       NA
 
           NA
  
 
  
 4
 
  25657365
 
  25657628
 
  ENSEMBL   UCSC 
 
  264
 
  7
 
 30963
 
 island
 
 0.017190
 
 0.0309795
 
 Not queried
 
  0.7567743
 
       NA
 
           NA
  
 
  
 19
 
  46174395
 
  46174565
 
  ENSEMBL   UCSC 
 
  171
 
  4
 
 20104
 
 gene;island
 
 0.164998
 
 0.4366564
 
 Not queried
 
  0.7567484
 
       NA
 
           NA
  
 
  
 10
 
  69835125
 
  69835323
 
  ENSEMBL   UCSC 
 
  199
 
  8
 
 5108
 
 tss
 
 0.012094
 
 0.0153865
 
 Not queried
 
  0.7563552
 
       NA
 
           NA
  
 
  
 19
 
  38183253
 
  38183456
 
  ENSEMBL   UCSC 
 
  204
 
  6
 
 20743
 
 tss
 
 0.070205
 
 0.0458952
 
 Not queried
 
  0.7563530
 
       NA
 
           NA
  
 
  
 1
 
   3059517
 
   3059820
 
  ENSEMBL   UCSC 
 
  304
 
  4
 
 104
 
 gene;island
 
 0.553775
 
 0.7712490
 
 Not queried
 
  0.7562142
 
       NA
 
           NA
  
 
  
 8
 
 144631768
 
 144632005
 
  ENSEMBL   UCSC 
 
  238
 
  5
 
 40667
 
 island
 
 0.033453
 
 0.0720385
 
 Not queried
 
  0.7562141
 
       NA
 
           NA
  
 
  
 3
 
 148415443
 
 148415650
 
  ENSEMBL   UCSC 
 
  208
 
  4
 
 28653
 
 tss
 
 0.036403
 
 0.0368573
 
 Not queried
 
  0.7561743
 
       NA
 
           NA
  
 
  
 11
 
  62623202
 
  62623623
 
  ENSEMBL   UCSC 
 
  422
 
 12
 
 7372
 
 tss;island
 
 0.008124
 
 0.0056892
 
 Not queried
 
  0.7560278
 
       NA
 
           NA
  
 
  
 5
 
 137610255
 
 137610645
 
  ENSEMBL   UCSC 
 
  391
 
  7
 
 32317
 
 tss
 
 0.003475
 
 0.0206462
 
 Not queried
 
  0.7559951
 
       NA
 
           NA
  
 
  
 14
 
  24808987
 
  24809226
 
  ENSEMBL   UCSC 
 
  240
 
  4
 
 12048
 
 gene
 
 0.013628
 
 0.0078020
 
 Not queried
 
  0.7558315
 
       NA
 
           NA
  
 
  
 16
 
  27414272
 
  27414536
 
  ENSEMBL   UCSC 
 
  265
 
  4
 
 14702
 
 gene
 
 0.003621
 
 0.0023088
 
 Not queried
 
  0.7557499
 
       NA
 
           NA
  
 
  
 6
 
  26204333
 
  26204823
 
  ENSEMBL   UCSC 
 
  491
 
  9
 
 34695
 
 tss
 
 0.005616
 
 0.0031550
 
 Not queried
 
  0.7556460
 
       NA
 
           NA
  
 
  
 10
 
  45914276
 
  45914949
 
  ENSEMBL   UCSC 
 
  674
 
  7
 
 4576
 
 gene;island
 
 0.008692
 
 0.0131877
 
 Not queried
 
  0.7555013
 
       NA
 
           NA
  
 
  
 15
 
  66795595
 
  66795844
 
  ENSEMBL   UCSC 
 
  250
 
  4
 
 13389
 
 gene;tss
 
 0.511546
 
 0.6215258
 
 Not queried
 
  0.7553943
 
       NA
 
           NA
  
 
  
 9
 
   1042605
 
   1042970
 
  ENSEMBL   UCSC 
 
  366
 
  4
 
 40861
 
 island
 
 0.120493
 
 0.1251831
 
 Not queried
 
  0.7552862
 
       NA
 
           NA
  
 
  
 17
 
  16342463
 
  16343134
 
  ENSEMBL   UCSC 
 
  672
 
 13
 
 16459
 
 gene;tss;island
 
 0.002335
 
 0.0022927
 
 Not queried
 
  0.7551709
 
       NA
 
           NA
  
 
  
 1
 
  40420503
 
  40420692
 
  ENSEMBL   UCSC 
 
  190
 
  9
 
 1531
 
 tss
 
 0.008522
 
 0.0110301
 
 Not queried
 
  0.7550499
 
       NA
 
           NA
  
 
  
 7
 
  35293753
 
  35293892
 
  ENSEMBL   UCSC 
 
  140
 
  5
 
 37535
 
 tss;island
 
 0.184840
 
 0.1691924
 
 Not queried
 
  0.7550207
 
       NA
 
           NA
  
 
  
 17
 
  42385942
 
  42386419
 
  ENSEMBL   UCSC 
 
  478
 
  7
 
 16613
 
 gene
 
 0.038469
 
 0.0368462
 
 Not queried
 
  0.7549445
 
       NA
 
           NA
  
 
  
 14
 
  51562381
 
  51562646
 
  ENSEMBL   UCSC 
 
  266
 
  6
 
 12837
 
 island
 
 0.064495
 
 0.0401871
 
 Not queried
 
  0.7546495
 
       NA
 
           NA
  
 
  
 17
 
  80889047
 
  80889350
 
  ENSEMBL   UCSC 
 
  304
 
  4
 
 16966
 
 gene;island
 
 0.972075
 
 0.9825547
 
 Not queried
 
  0.7546302
 
       NA
 
           NA
  
 
  
 10
 
   4868328
 
   4868398
 
  ENSEMBL   UCSC 
 
   71
 
  6
 
 4920
 
 tss
 
 0.122395
 
 0.1681400
 
 Not queried
 
  0.7545249
 
       NA
 
           NA
  
 
  
 10
 
   4868328
 
   4868690
 
  ENSEMBL   UCSC 
 
  363
 
  8
 
 5516
 
 island
 
 0.090161
 
 0.1421307
 
 Not queried
 
  0.7545249
 
       NA
 
           NA
  
 
  
 8
 
 102380960
 
 102381107
 
  ENSEMBL   UCSC 
 
  148
 
  4
 
 39849
 
 tss
 
 0.558252
 
 0.6631046
 
 Not queried
 
  0.7544275
 
       NA
 
           NA
  
 
  
 1
 
 151254500
 
 151254649
 
  ENSEMBL   UCSC 
 
  150
 
  4
 
 1926
 
 tss
 
 0.007376
 
 0.0045673
 
 Not queried
 
  0.7542642
 
       NA
 
           NA
  
 
  
 19
 
   2944943
 
   2945201
 
  ENSEMBL   UCSC 
 
  259
 
  8
 
 21309
 
 island
 
 0.009660
 
 0.0078737
 
 Not queried
 
  0.7541952
 
       NA
 
           NA
  
 
  
 5
 
  10441490
 
  10441903
 
  ENSEMBL   UCSC 
 
  414
 
  8
 
 31989
 
 tss;island
 
 0.003750
 
 0.0042846
 
 Not queried
 
  0.7541552
 
       NA
 
           NA
  
 
  
 10
 
 128152075
 
 128152247
 
  ENSEMBL   UCSC 
 
  173
 
  4
 
 4816
 
 gene
 
 0.011089
 
 0.0190970
 
 Not queried
 
  0.7540495
 
       NA
 
           NA
  
 
  
 10
 
  94459183
 
  94459665
 
  ENSEMBL   UCSC 
 
  483
 
  6
 
 5947
 
 island
 
 0.047910
 
 0.0221429
 
 Not queried
 
  0.7538354
 
       NA
 
           NA
  
 
  
 1
 
   1845940
 
   1846154
 
  ENSEMBL   UCSC 
 
  215
 
  5
 
 1163
 
 tss
 
 0.420290
 
 0.6991701
 
 Not queried
 
  0.7538263
 
       NA
 
           NA
  
 
  
 1
 
 113261488
 
 113261894
 
  ENSEMBL   UCSC 
 
  407
 
  6
 
 3609
 
 island
 
 0.690414
 
 0.3910336
 
 Not queried
 
  0.7538257
 
       NA
 
           NA
  
 
  
 12
 
  54674346
 
  54674547
 
  ENSEMBL   UCSC 
 
  202
 
  7
 
 9760
 
 tss;island
 
 0.005208
 
 0.0031060
 
 Not queried
 
  0.7538247
 
       NA
 
           NA
  
 
  
 22
 
  46805673
 
  46805918
 
  ENSEMBL   UCSC 
 
  246
 
  4
 
 26914
 
 gene;island
 
 0.741219
 
 0.7871245
 
 Not queried
 
  0.7537908
 
       NA
 
           NA
  
 
  
 12
 
   4382878
 
   4383281
 
  ENSEMBL   UCSC 
 
  404
 
  6
 
 10171
 
 island
 
 0.006111
 
 0.0046031
 
 Not queried
 
  0.7537782
 
       NA
 
           NA
  
 
  
 19
 
  50303041
 
  50303389
 
  ENSEMBL   UCSC 
 
  349
 
  5
 
 20164
 
 gene;island
 
 0.972288
 
 0.9701407
 
 Not queried
 
  0.7535863
 
       NA
 
           NA
  
 
  
 11
 
  93583509
 
  93583973
 
  ENSEMBL   UCSC 
 
  465
 
  8
 
 8733
 
 island
 
 0.295190
 
 0.1591946
 
 Not queried
 
  0.7533902
 
       NA
 
           NA
  
 
  
 19
 
  11517079
 
  11517436
 
  ENSEMBL   UCSC 
 
  358
 
  5
 
 19838
 
 gene;island
 
 0.123969
 
 0.0552587
 
 Not queried
 
  0.7532840
 
       NA
 
           NA
  
 
  
 13
 
 101327124
 
 101327190
 
  ENSEMBL   UCSC 
 
   67
 
  6
 
 11518
 
 tss;island
 
 0.005837
 
 0.0046621
 
 Not queried
 
  0.7531747
 
       NA
 
           NA
  
 
  
 1
 
   8384440
 
   8384718
 
  ENSEMBL   UCSC 
 
  279
 
  6
 
 193
 
 gene;island
 
 0.613909
 
 0.7592771
 
 Not queried
 
  0.7531710
 
       NA
 
           NA
  
 
  
 17
 
   6947032
 
   6947464
 
  ENSEMBL   UCSC 
 
  433
 
 10
 
 17918
 
 island
 
 0.035029
 
 0.0779925
 
 Not queried
 
  0.7530990
 
       NA
 
           NA
  
 
  
 5
 
 172386517
 
 172387101
 
  ENSEMBL   UCSC 
 
  585
 
  8
 
 31899
 
 gene
 
 0.037630
 
 0.0271388
 
 Not queried
 
  0.7530408
 
       NA
 
           NA
  
 
  
 2
 
 242138408
 
 242138778
 
  ENSEMBL   UCSC 
 
  371
 
  4
 
 23130
 
 gene;island
 
 0.579793
 
 0.6241118
 
 Not queried
 
  0.7530224
 
       NA
 
           NA
  
 
  
 10
 
  42970618
 
  42971251
 
  ENSEMBL   UCSC 
 
  634
 
 10
 
 5681
 
 island
 
 0.234043
 
 0.2926644
 
 Not queried
 
  0.7527845
 
       NA
 
           NA
  
 
  
 2
 
  42068380
 
  42068648
 
  ENSEMBL   UCSC 
 
  269
 
  5
 
 24238
 
 island
 
 0.034413
 
 0.0810259
 
 Not queried
 
  0.7527562
 
       NA
 
           NA
  
 
  
 5
 
 178368071
 
 178368415
 
  ENSEMBL   UCSC 
 
  345
 
  8
 
 33476
 
 island
 
 0.405887
 
 0.1949446
 
 Not queried
 
  0.7525751
 
       NA
 
           NA
  
 
  
 4
 
 148538143
 
 148538954
 
  ENSEMBL   UCSC 
 
  812
 
 11
 
 31356
 
 island
 
 0.015169
 
 0.0037165
 
 Not queried
 
  0.7525549
 
       NA
 
           NA
  
 
  
 1
 
  27226578
 
  27227109
 
  ENSEMBL   UCSC 
 
  532
 
  8
 
 2905
 
 island
 
 0.009619
 
 0.0056804
 
 Not queried
 
  0.7525433
 
       NA
 
           NA
  
 
  
 11
 
  33037397
 
  33037626
 
  ENSEMBL   UCSC 
 
  230
 
  5
 
 7230
 
 tss
 
 0.005943
 
 0.0071926
 
 Not queried
 
  0.7523911
 
       NA
 
           NA
  
 
  
 8
 
 142368026
 
 142368332
 
  ENSEMBL   UCSC 
 
  307
 
  6
 
 39452
 
 gene;island
 
 0.622053
 
 0.7231499
 
 Not queried
 
  0.7523593
 
       NA
 
           NA
  
 
  
 2
 
 124782586
 
 124782831
 
  ENSEMBL   UCSC 
 
  246
 
  4
 
 23607
 
 tss
 
 0.108114
 
 0.2067204
 
 Not queried
 
  0.7522612
 
       NA
 
           NA
  
 
  
 17
 
  19483367
 
  19483721
 
  ENSEMBL   UCSC 
 
  355
 
  5
 
 18100
 
 island
 
 0.096656
 
 0.0605173
 
 Not queried
 
  0.7522609
 
       NA
 
           NA
  
 
  
 15
 
  32907173
 
  32907450
 
  ENSEMBL   UCSC 
 
  278
 
  4
 
 13552
 
 tss;island
 
 0.002039
 
 0.0022588
 
 Not queried
 
  0.7522526
 
       NA
 
           NA
  
 
  
 7
 
  25219543
 
  25220162
 
  ENSEMBL   UCSC 
 
  620
 
 12
 
 38322
 
 island
 
 0.009351
 
 0.0054219
 
 Not queried
 
  0.7521997
 
       NA
 
           NA
  
 
  
 4
 
  38869436
 
  38869779
 
  ENSEMBL   UCSC 
 
  344
 
  8
 
 29979
 
 gene
 
 0.038241
 
 0.0528442
 
 Not queried
 
  0.7521842
 
       NA
 
           NA
  
 
  
 1
 
  75139470
 
  75139746
 
  ENSEMBL   UCSC 
 
  277
 
  6
 
 1696
 
 tss
 
 0.032803
 
 0.0481109
 
 Not queried
 
  0.7521252
 
       NA
 
           NA
  
 
  
 11
 
   6255951
 
   6256091
 
  ENSEMBL   UCSC 
 
  141
 
  6
 
 7105
 
 tss
 
 0.009797
 
 0.0130025
 
 Not queried
 
  0.7515950
 
       NA
 
           NA
  
 
  
 3
 
 128840619
 
 128840836
 
  ENSEMBL   UCSC 
 
  218
 
 10
 
 28577
 
 tss;island
 
 0.038458
 
 0.0561304
 
 Not queried
 
  0.7515808
 
       NA
 
           NA
  
 
  
 8
 
   1615950
 
   1616925
 
  ENSEMBL   UCSC 
 
  976
 
 10
 
 39171
 
 gene;island
 
 0.470599
 
 0.5456651
 
 Not queried
 
  0.7514513
 
       NA
 
           NA
  
 
  
 7
 
  65447322
 
  65447622
 
  ENSEMBL   UCSC 
 
  301
 
 10
 
 37620
 
 tss
 
 0.120109
 
 0.0695454
 
 Not queried
 
  0.7509328
 
       NA
 
           NA
  
 
  
 4
 
   1004110
 
   1005405
 
  ENSEMBL   UCSC 
 
 1296
 
 28
 
 30235
 
 tss;island
 
 0.014997
 
 0.0264361
 
 Not queried
 
  0.7508455
 
       NA
 
           NA
  
 
  
 7
 
 158264663
 
 158264935
 
  ENSEMBL   UCSC 
 
  273
 
  5
 
 37374
 
 gene
 
 0.485849
 
 0.5165020
 
 Not queried
 
  0.7506541
 
       NA
 
           NA
  
 
  
 3
 
 183145870
 
 183146204
 
  ENSEMBL   UCSC 
 
  335
 
  4
 
 28745
 
 tss
 
 0.017878
 
 0.0279298
 
 Not queried
 
  0.7505535
 
       NA
 
           NA
  
 
  
 5
 
  50678451
 
  50678928
 
  ENSEMBL   UCSC 
 
  478
 
  9
 
 32057
 
 tss;island
 
 0.242914
 
 0.1163051
 
 Not queried
 
  0.7504787
 
       NA
 
           NA
  
 
  
 7
 
  35292896
 
  35293309
 
  ENSEMBL   UCSC 
 
  414
 
  6
 
 36963
 
 gene;island
 
 0.229825
 
 0.1035690
 
 Not queried
 
  0.7504577
 
       NA
 
           NA
  
 
  
 17
 
  80818992
 
  80819418
 
  ENSEMBL   UCSC 
 
  427
 
  7
 
 16961
 
 gene;island
 
 0.958795
 
 0.6136646
 
 Not queried
 
  0.7504470
 
       NA
 
           NA
  
 
  
 3
 
 118864587
 
 118864895
 
  ENSEMBL   UCSC 
 
  309
 
  7
 
 27905
 
 gene
 
 0.869612
 
 0.9231814
 
 Not queried
 
  0.7502799
 
       NA
 
           NA
  
 
  
 17
 
  74965015
 
  74965245
 
  ENSEMBL   UCSC 
 
  231
 
  4
 
 18799
 
 island
 
 0.011928
 
 0.0160775
 
 Not queried
 
  0.7502025
 
       NA
 
           NA
  
 
  
 8
 
  27449716
 
  27450062
 
  ENSEMBL   UCSC 
 
  347
 
  6
 
 40182
 
 island
 
 0.012723
 
 0.0096490
 
 Not queried
 
  0.7500643
 
       NA
 
           NA
  
 
  
 11
 
  74303294
 
  74303539
 
  ENSEMBL   UCSC 
 
  246
 
  6
 
 7560
 
 tss
 
 0.013659
 
 0.0088797
 
 Not queried
 
  0.7499870
 
       NA
 
           NA
  
 
  
 4
 
  77723057
 
  77723415
 
  ENSEMBL   UCSC 
 
  359
 
  4
 
 31119
 
 island
 
 0.577366
 
 0.8439987
 
 Not queried
 
  0.7498445
 
       NA
 
           NA
  
 
  
 22
 
  51016604
 
  51017166
 
  ENSEMBL   UCSC 
 
  563
 
 13
 
 27260
 
 tss
 
 0.533286
 
 0.6307301
 
 Not queried
 
  0.7498271
 
       NA
 
           NA
  
 
  
 1
 
  15850456
 
  15850746
 
  ENSEMBL   UCSC 
 
  291
 
  5
 
 246
 
 gene
 
 0.022741
 
 0.0053684
 
 Not queried
 
  0.7498003
 
       NA
 
           NA
  
 
  
 3
 
  49726775
 
  49727474
 
  ENSEMBL   UCSC 
 
  700
 
 12
 
 28332
 
 tss;island
 
 0.014864
 
 0.0113079
 
 Not queried
 
  0.7496979
 
       NA
 
           NA
  
 
  
 11
 
  27494352
 
  27494432
 
  ENSEMBL   UCSC 
 
   81
 
  5
 
 7208
 
 tss
 
 0.016570
 
 0.0078428
 
 Not queried
 
  0.7496509
 
       NA
 
           NA
  
 
  
 11
 
  27494133
 
  27494432
 
  ENSEMBL   UCSC 
 
  300
 
  7
 
 8142
 
 island
 
 0.015586
 
 0.0071166
 
 Not queried
 
  0.7496509
 
       NA
 
           NA
  
 
  
 19
 
  16013630
 
  16013895
 
  ENSEMBL   UCSC 
 
  266
 
  4
 
 21711
 
 island
 
 0.745314
 
 0.6275403
 
 Not queried
 
  0.7496265
 
       NA
 
           NA
  
 
  
 11
 
  71163428
 
  71164189
 
  ENSEMBL   UCSC 
 
  762
 
 11
 
 7531
 
 tss
 
 0.004684
 
 0.0035599
 
 Not queried
 
  0.7495914
 
       NA
 
           NA
  
 
  
 13
 
  84455525
 
  84455742
 
  ENSEMBL   UCSC 
 
  218
 
  4
 
 11185
 
 gene;island
 
 0.008811
 
 0.0434471
 
 Not queried
 
  0.7493790
 
       NA
 
           NA
  
 
  
 19
 
  17982599
 
  17982759
 
  ENSEMBL   UCSC 
 
  161
 
  7
 
 20604
 
 tss
 
 0.086237
 
 0.0529983
 
 Not queried
 
  0.7493457
 
       NA
 
           NA
  
 
  
 12
 
  54366760
 
  54367008
 
  ENSEMBL   UCSC 
 
  249
 
  6
 
 10484
 
 island
 
 0.113495
 
 0.2121656
 
 Not queried
 
  0.7492113
 
       NA
 
           NA
  
 
  
 11
 
  33757858
 
  33758529
 
  ENSEMBL   UCSC 
 
  672
 
 11
 
 8194
 
 island
 
 0.006799
 
 0.0133273
 
 Not queried
 
  0.7491813
 
       NA
 
           NA
  
 
  
 19
 
   1241377
 
   1241808
 
  ENSEMBL   UCSC 
 
  432
 
  6
 
 21205
 
 island
 
 0.006002
 
 0.0055048
 
 Not queried
 
  0.7490225
 
       NA
 
           NA
  
 
  
 11
 
   6704667
 
   6704982
 
  ENSEMBL   UCSC 
 
  316
 
 10
 
 7117
 
 tss
 
 0.003455
 
 0.0020631
 
 Not queried
 
  0.7485870
 
       NA
 
           NA
  
 
  
 7
 
  87257356
 
  87257786
 
  ENSEMBL   UCSC 
 
  431
 
  7
 
 37073
 
 gene;island
 
 0.003860
 
 0.0026845
 
 Not queried
 
  0.7485249
 
       NA
 
           NA
  
 
  
 7
 
  87257356
 
  87257673
 
  ENSEMBL   UCSC 
 
  318
 
  5
 
 37687
 
 tss
 
 0.008220
 
 0.0065348
 
 Not queried
 
  0.7485249
 
       NA
 
           NA
  
 
  
 5
 
 173738161
 
 173738479
 
  ENSEMBL   UCSC 
 
  319
 
  4
 
 33415
 
 island
 
 0.364863
 
 0.3604879
 
 Not queried
 
  0.7484397
 
       NA
 
           NA
  
 
  
 7
 
  30068068
 
  30068347
 
  ENSEMBL   UCSC 
 
  280
 
  4
 
 38382
 
 island
 
 0.048182
 
 0.0307615
 
 Not queried
 
  0.7481814
 
       NA
 
           NA
  
 
  
 7
 
 133811808
 
 133812072
 
  ENSEMBL   UCSC 
 
  265
 
  8
 
 37909
 
 tss
 
 0.021255
 
 0.0112163
 
 Not queried
 
  0.7481560
 
       NA
 
           NA
  
 
  
 20
 
  62184207
 
  62184397
 
  ENSEMBL   UCSC 
 
  191
 
  4
 
 26357
 
 island
 
 0.008571
 
 0.0048629
 
 Not queried
 
  0.7481320
 
       NA
 
           NA
  
 
  
 1
 
   1990780
 
   1991051
 
  ENSEMBL   UCSC 
 
  272
 
  4
 
 53
 
 gene;island
 
 0.706951
 
 0.8077503
 
 Not queried
 
  0.7481001
 
       NA
 
           NA
  
 
  
 17
 
   6947288
 
   6947464
 
  ENSEMBL   UCSC 
 
  177
 
  8
 
 17060
 
 tss
 
 0.031159
 
 0.0435293
 
 Not queried
 
  0.7480943
 
       NA
 
           NA
  
 
  
 11
 
  71639806
 
  71640085
 
  ENSEMBL   UCSC 
 
  280
 
  4
 
 6805
 
 gene
 
 0.001222
 
 0.0015890
 
 Not queried
 
  0.7478272
 
       NA
 
           NA
  
 
  
 17
 
  77952109
 
  77952488
 
  ENSEMBL   UCSC 
 
  380
 
  6
 
 16847
 
 gene;island
 
 0.841656
 
 0.8945971
 
 Not queried
 
  0.7477918
 
       NA
 
           NA
  
 
  
 14
 
 103989208
 
 103989789
 
  ENSEMBL   UCSC 
 
  582
 
  9
 
 12649
 
 tss;island
 
 0.122982
 
 0.0447849
 
 Not queried
 
  0.7477565
 
       NA
 
           NA
  
 
  
 10
 
 102504022
 
 102504297
 
  ENSEMBL   UCSC 
 
  276
 
  5
 
 5296
 
 tss;island
 
 0.009798
 
 0.0162324
 
 Not queried
 
  0.7477517
 
       NA
 
           NA
  
 
  
 4
 
   8271196
 
   8271507
 
  ENSEMBL   UCSC 
 
  312
 
  7
 
 30917
 
 island
 
 0.008469
 
 0.0298801
 
 Not queried
 
  0.7477068
 
       NA
 
           NA
  
 
  
 3
 
 138657291
 
 138657774
 
  ENSEMBL   UCSC 
 
  484
 
  5
 
 29528
 
 island
 
 0.022721
 
 0.0499420
 
 Not queried
 
  0.7476729
 
       NA
 
           NA
  
 
  
 7
 
  16684321
 
  16684906
 
  ENSEMBL   UCSC 
 
  586
 
  6
 
 36904
 
 gene;tss;island
 
 0.010110
 
 0.0036671
 
 Not queried
 
  0.7476343
 
       NA
 
           NA
  
 
  
 8
 
 116660311
 
 116660697
 
  ENSEMBL   UCSC 
 
  387
 
  4
 
 39403
 
 gene;island
 
 0.009104
 
 0.0252021
 
 Not queried
 
  0.7475772
 
       NA
 
           NA
  
 
  
 17
 
  46669455
 
  46669781
 
  ENSEMBL   UCSC 
 
  327
 
  8
 
 16654
 
 gene;island
 
 0.158073
 
 0.4603370
 
 Not queried
 
  0.7475223
 
       NA
 
           NA
  
 
  
 3
 
 120170383
 
 120170646
 
  ENSEMBL   UCSC 
 
  264
 
  4
 
 28528
 
 tss;island
 
 0.645084
 
 0.9114221
 
 Not queried
 
  0.7474596
 
       NA
 
           NA
  
 
  
 4
 
  93226245
 
  93226625
 
  ENSEMBL   UCSC 
 
  381
 
  5
 
 30061
 
 gene;island
 
 0.011275
 
 0.0153003
 
 Not queried
 
  0.7473250
 
       NA
 
           NA
  
 
  
 15
 
  86313834
 
  86313973
 
  ENSEMBL   UCSC 
 
  140
 
  7
 
 13463
 
 gene;tss;island
 
 0.804176
 
 0.8364019
 
 Not queried
 
  0.7472966
 
       NA
 
           NA
  
 
  
 10
 
  25464008
 
  25464513
 
  ENSEMBL   UCSC 
 
  506
 
 12
 
 4533
 
 gene;island
 
 0.047561
 
 0.0632971
 
 Not queried
 
  0.7472408
 
       NA
 
           NA
  
 
  
 10
 
  32734661
 
  32734958
 
  ENSEMBL   UCSC 
 
  298
 
  7
 
 5022
 
 tss
 
 0.004412
 
 0.0082120
 
 Not queried
 
  0.7472359
 
       NA
 
           NA
  
 
  
 6
 
  82462253
 
  82462746
 
  ENSEMBL   UCSC 
 
  494
 
  9
 
 36281
 
 island
 
 0.024195
 
 0.0100162
 
 Not queried
 
  0.7470164
 
       NA
 
           NA
  
 
  
 8
 
  70746191
 
  70746368
 
  ENSEMBL   UCSC 
 
  178
 
  4
 
 39332
 
 gene;tss;island
 
 0.005720
 
 0.0022048
 
 Not queried
 
  0.7469917
 
       NA
 
           NA
  
 
  
 2
 
  62132430
 
  62132778
 
  ENSEMBL   UCSC 
 
  349
 
  7
 
 23381
 
 tss
 
 0.002642
 
 0.0036944
 
 Not queried
 
  0.7469731
 
       NA
 
           NA
  
 
  
 22
 
  18063857
 
  18064224
 
  ENSEMBL   UCSC 
 
  368
 
  5
 
 26780
 
 gene;island
 
 0.754823
 
 0.6945181
 
 Not queried
 
  0.7468902
 
       NA
 
           NA
  
 
  
 11
 
 118938260
 
 118938475
 
  ENSEMBL   UCSC 
 
  216
 
  7
 
 7747
 
 tss
 
 0.003583
 
 0.0031777
 
 Not queried
 
  0.7468602
 
       NA
 
           NA
  
 
  
 20
 
  10199210
 
  10199473
 
  ENSEMBL   UCSC 
 
  264
 
  7
 
 25595
 
 tss
 
 0.009221
 
 0.0062998
 
 Not queried
 
  0.7467758
 
       NA
 
           NA
  
 
  
 1
 
  36851558
 
  36851860
 
  ENSEMBL   UCSC 
 
  303
 
  7
 
 1495
 
 tss;island
 
 0.028309
 
 0.0143874
 
 Not queried
 
  0.7465804
 
       NA
 
           NA
  
 
  
 3
 
 101231982
 
 101232368
 
  ENSEMBL   UCSC 
 
  387
 
  9
 
 29330
 
 island
 
 0.001552
 
 0.0011022
 
 Not queried
 
  0.7464462
 
       NA
 
           NA
  
 
  
 5
 
 122110867
 
 122111354
 
  ENSEMBL   UCSC 
 
  488
 
  5
 
 31728
 
 gene
 
 0.017539
 
 0.0178388
 
 Not queried
 
  0.7464106
 
       NA
 
           NA
  
 
  
 17
 
  17942241
 
  17942403
 
  ENSEMBL   UCSC 
 
  163
 
  4
 
 16471
 
 gene
 
 0.004991
 
 0.0060252
 
 Not queried
 
  0.7462934
 
       NA
 
           NA
  
 
  
 1
 
 166039444
 
 166039918
 
  ENSEMBL   UCSC 
 
  475
 
  5
 
 867
 
 gene
 
 0.300916
 
 0.4486035
 
 Not queried
 
  0.7460838
 
       NA
 
           NA
  
 
  
 5
 
  42424356
 
  42424604
 
  ENSEMBL   UCSC 
 
  249
 
  5
 
 31601
 
 gene;island
 
 0.017810
 
 0.0374984
 
 Not queried
 
  0.7460050
 
       NA
 
           NA
  
 
  
 14
 
  31028682
 
  31029063
 
  ENSEMBL   UCSC 
 
  382
 
  4
 
 12056
 
 gene;island
 
 0.003189
 
 0.0006748
 
 Not queried
 
  0.7459886
 
       NA
 
           NA
  
 
  
 1
 
  87380128
 
  87380200
 
  ENSEMBL   UCSC 
 
   73
 
  5
 
 1730
 
 tss;island
 
 0.009287
 
 0.0072964
 
 Not queried
 
  0.7459829
 
       NA
 
           NA
  
 
  
 12
 
  48152844
 
  48153133
 
  ENSEMBL   UCSC 
 
  290
 
  8
 
 9662
 
 tss;island
 
 0.008465
 
 0.0027080
 
 Not queried
 
  0.7458945
 
       NA
 
           NA
  
 
  
 3
 
  75707805
 
  75708197
 
  ENSEMBL   UCSC 
 
  393
 
  4
 
 29289
 
 island
 
 0.232109
 
 0.3303945
 
 Not queried
 
  0.7458662
 
       NA
 
           NA
  
 
  
 2
 
 233286639
 
 233286870
 
  ENSEMBL   UCSC 
 
  232
 
  4
 
 25193
 
 island
 
 0.798348
 
 0.8745140
 
 Not queried
 
  0.7458516
 
       NA
 
           NA
  
 
  
 19
 
  57182844
 
  57183268
 
  ENSEMBL   UCSC 
 
  425
 
  4
 
 20238
 
 gene;island
 
 0.108300
 
 0.2269871
 
 Not queried
 
  0.7456448
 
       NA
 
           NA
  
 
  
 6
 
  32165877
 
  32165917
 
  ENSEMBL   UCSC 
 
   41
 
  4
 
 34084
 
 gene;island
 
 0.876843
 
 0.9668324
 
 Not queried
 
  0.7454681
 
       NA
 
           NA
  
 
  
 3
 
 157217556
 
 157217842
 
  ENSEMBL   UCSC 
 
  287
 
  6
 
 28679
 
 tss
 
 0.056078
 
 0.0523394
 
 Not queried
 
  0.7454627
 
       NA
 
           NA
  
 
  
 1
 
   3276413
 
   3276786
 
  ENSEMBL   UCSC 
 
  374
 
  6
 
 121
 
 gene;island
 
 0.432537
 
 0.5655306
 
 Not queried
 
  0.7454528
 
       NA
 
           NA
  
 
  
 11
 
  16627534
 
  16628010
 
  ENSEMBL   UCSC 
 
  477
 
  8
 
 8088
 
 island
 
 0.030159
 
 0.0083426
 
 Not queried
 
  0.7453479
 
       NA
 
           NA
  
 
  
 6
 
  33390907
 
  33391341
 
  ENSEMBL   UCSC 
 
  435
 
  9
 
 34284
 
 gene;island
 
 0.944280
 
 0.9536728
 
 Not queried
 
  0.7452411
 
       NA
 
           NA
  
 
  
 14
 
  29235894
 
  29236221
 
  ENSEMBL   UCSC 
 
  328
 
 11
 
 12336
 
 tss
 
 0.215411
 
 0.0817498
 
 Not queried
 
  0.7452135
 
       NA
 
           NA
  
 
  
 12
 
  57630107
 
  57630456
 
  ENSEMBL   UCSC 
 
  350
 
  4
 
 9191
 
 gene;island
 
 0.124161
 
 0.3173800
 
 Not queried
 
  0.7451714
 
       NA
 
           NA
  
 
  
 12
 
  80328930
 
  80329299
 
  ENSEMBL   UCSC 
 
  370
 
  6
 
 10647
 
 island
 
 0.007737
 
 0.0132047
 
 Not queried
 
  0.7449045
 
       NA
 
           NA
  
 
  
 11
 
   8892616
 
   8892893
 
  ENSEMBL   UCSC 
 
  278
 
  4
 
 6501
 
 gene;island
 
 0.064102
 
 0.0431952
 
 Not queried
 
  0.7447542
 
       NA
 
           NA
  
 
  
 3
 
  27764473
 
  27764816
 
  ENSEMBL   UCSC 
 
  344
 
  4
 
 28196
 
 tss;island
 
 0.024441
 
 0.0110500
 
 Not queried
 
  0.7446510
 
       NA
 
           NA
  
 
  
 14
 
  23503928
 
  23504423
 
  ENSEMBL   UCSC 
 
  496
 
  6
 
 12022
 
 gene
 
 0.002354
 
 0.0039756
 
 Not queried
 
  0.7446185
 
       NA
 
           NA
  
 
  
 17
 
  22016686
 
  22017365
 
  ENSEMBL   UCSC 
 
  680
 
  6
 
 18129
 
 island
 
 0.383969
 
 0.4670236
 
 Not queried
 
  0.7445267
 
       NA
 
           NA
  
 
  
 17
 
  46651822
 
  46651952
 
  ENSEMBL   UCSC 
 
  131
 
  5
 
 17458
 
 tss
 
 0.942081
 
 0.9646201
 
 Not queried
 
  0.7445170
 
       NA
 
           NA
  
 
  
 17
 
   6347533
 
   6348200
 
  ENSEMBL   UCSC 
 
  668
 
 11
 
 17904
 
 island
 
 0.044632
 
 0.0125362
 
 Not queried
 
  0.7444075
 
       NA
 
           NA
  
 
  
 11
 
  17410863
 
  17411270
 
  ENSEMBL   UCSC 
 
  408
 
  5
 
 7175
 
 tss;island
 
 0.047758
 
 0.0121313
 
 Not queried
 
  0.7442521
 
       NA
 
           NA
  
 
  
 13
 
  44947923
 
  44948152
 
  ENSEMBL   UCSC 
 
  230
 
  4
 
 11703
 
 island
 
 0.065555
 
 0.0464180
 
 Not queried
 
  0.7442157
 
       NA
 
           NA
  
 
  
 19
 
   6737385
 
   6737494
 
  ENSEMBL   UCSC 
 
  110
 
  5
 
 19769
 
 gene
 
 0.025333
 
 0.0081768
 
 Not queried
 
  0.7442070
 
       NA
 
           NA
  
 
  
 19
 
   6737385
 
   6738068
 
  ENSEMBL   UCSC 
 
  684
 
 15
 
 20393
 
 tss;island
 
 0.011862
 
 0.0057730
 
 Not queried
 
  0.7442070
 
       NA
 
           NA
  
 
  
 15
 
  40544855
 
  40545511
 
  ENSEMBL   UCSC 
 
  657
 
 11
 
 13290
 
 gene;island
 
 0.007348
 
 0.0037613
 
 Not queried
 
  0.7441879
 
       NA
 
           NA
  
 
  
 8
 
 117887184
 
 117887318
 
  ENSEMBL   UCSC 
 
  135
 
  8
 
 39881
 
 tss
 
 0.002661
 
 0.0031322
 
 Not queried
 
  0.7441594
 
       NA
 
           NA
  
 
  
 17
 
  73569211
 
  73569524
 
  ENSEMBL   UCSC 
 
  314
 
  4
 
 16771
 
 gene;island
 
 0.911271
 
 0.8855520
 
 Not queried
 
  0.7440594
 
       NA
 
           NA
  
 
  
 12
 
  90102477
 
  90102578
 
  ENSEMBL   UCSC 
 
  102
 
  4
 
 9910
 
 tss;island
 
 0.004399
 
 0.0085768
 
 Not queried
 
  0.7438466
 
       NA
 
           NA
  
 
  
 3
 
  44753865
 
  44754587
 
  ENSEMBL   UCSC 
 
  723
 
 12
 
 29040
 
 island
 
 0.274387
 
 0.4575167
 
 Not queried
 
  0.7437178
 
       NA
 
           NA
  
 
  
 2
 
  95522774
 
  95522902
 
  ENSEMBL   UCSC 
 
  129
 
  4
 
 24509
 
 island
 
 0.575916
 
 0.4037180
 
 Not queried
 
  0.7437093
 
       NA
 
           NA
  
 
  
 11
 
   7041194
 
   7041987
 
  ENSEMBL   UCSC 
 
  794
 
 18
 
 7119
 
 tss;island
 
 0.079744
 
 0.0481278
 
 Not queried
 
  0.7436451
 
       NA
 
           NA
  
 
  
 19
 
  38270199
 
  38270616
 
  ENSEMBL   UCSC 
 
  418
 
  8
 
 21962
 
 island
 
 0.035761
 
 0.0125410
 
 Not queried
 
  0.7436416
 
       NA
 
           NA
  
 
  
 19
 
   7680053
 
   7680443
 
  ENSEMBL   UCSC 
 
  391
 
  6
 
 19781
 
 gene;island
 
 0.945315
 
 0.9653895
 
 Not queried
 
  0.7435248
 
       NA
 
           NA
  
 
  
 1
 
  51434293
 
  51434666
 
  ENSEMBL   UCSC 
 
  374
 
  6
 
 1619
 
 tss;island
 
 0.002419
 
 0.0089434
 
 Not queried
 
  0.7435017
 
       NA
 
           NA
  
 
  
 2
 
 237077920
 
 237078733
 
  ENSEMBL   UCSC 
 
  814
 
  9
 
 25225
 
 island
 
 0.022131
 
 0.1023621
 
 Not queried
 
  0.7433040
 
       NA
 
           NA
  
 
  
 2
 
 135476601
 
 135477056
 
  ENSEMBL   UCSC 
 
  456
 
  8
 
 23642
 
 tss;island
 
 0.027827
 
 0.0579822
 
 Not queried
 
  0.7432617
 
       NA
 
           NA
  
 
  
 8
 
 109094032
 
 109094346
 
  ENSEMBL   UCSC 
 
  315
 
  4
 
 39394
 
 gene;island
 
 0.013541
 
 0.0196069
 
 Not queried
 
  0.7431841
 
       NA
 
           NA
  
 
  
 16
 
   4664854
 
   4665307
 
  ENSEMBL   UCSC 
 
  454
 
  6
 
 15672
 
 island
 
 0.281719
 
 0.0725612
 
 Not queried
 
  0.7431558
 
       NA
 
           NA
  
 
  
 12
 
  95867190
 
  95867833
 
  ENSEMBL   UCSC 
 
  644
 
 12
 
 10687
 
 island
 
 0.005730
 
 0.0025949
 
 Not queried
 
  0.7430812
 
       NA
 
           NA
  
 
  
 11
 
  66139712
 
  66139869
 
  ENSEMBL   UCSC 
 
  158
 
  4
 
 7467
 
 tss;island
 
 0.013247
 
 0.0213489
 
 Not queried
 
  0.7429167
 
       NA
 
           NA
  
 
  
 19
 
   5622997
 
   5623257
 
  ENSEMBL   UCSC 
 
  261
 
 11
 
 20378
 
 tss;island
 
 0.003594
 
 0.0036035
 
 Not queried
 
  0.7425491
 
       NA
 
           NA
  
 
  
 6
 
 167104601
 
 167104926
 
  ENSEMBL   UCSC 
 
  326
 
  5
 
 34553
 
 gene
 
 0.360039
 
 0.5463367
 
 Not queried
 
  0.7423656
 
       NA
 
           NA
  
 
  
 18
 
   5237527
 
   5237922
 
  ENSEMBL   UCSC 
 
  396
 
  5
 
 19047
 
 gene;tss;island
 
 0.009011
 
 0.0039969
 
 Not queried
 
  0.7421387
 
       NA
 
           NA
  
 
  
 2
 
 219827660
 
 219827930
 
  ENSEMBL   UCSC 
 
  271
 
  4
 
 25097
 
 island
 
 0.165187
 
 0.0422481
 
 Not queried
 
  0.7420735
 
       NA
 
           NA
  
 
  
 8
 
  49647907
 
  49648363
 
  ENSEMBL   UCSC 
 
  457
 
  5
 
 39692
 
 tss
 
 0.072911
 
 0.0834849
 
 Not queried
 
  0.7420036
 
       NA
 
           NA
  
 
  
 2
 
  71126275
 
  71127576
 
  ENSEMBL   UCSC 
 
 1302
 
 26
 
 23422
 
 tss;island
 
 0.031536
 
 0.0550652
 
 Not queried
 
  0.7418182
 
       NA
 
           NA
  
 
  
 14
 
  23623480
 
  23623935
 
  ENSEMBL   UCSC 
 
  456
 
  6
 
 12027
 
 gene
 
 0.346784
 
 0.4929260
 
 Not queried
 
  0.7411921
 
       NA
 
           NA
  
 
  
 6
 
 123317124
 
 123317569
 
  ENSEMBL   UCSC 
 
  446
 
  8
 
 35207
 
 tss
 
 0.017645
 
 0.0320910
 
 Not queried
 
  0.7411725
 
       NA
 
           NA
  
 
  
 1
 
 146522365
 
 146522554
 
  ENSEMBL   UCSC 
 
  190
 
  4
 
 3689
 
 island
 
 0.821708
 
 0.8295886
 
 Not queried
 
  0.7410940
 
       NA
 
           NA
  
 
  
 13
 
  37006063
 
  37006340
 
  ENSEMBL   UCSC 
 
  278
 
  7
 
 11132
 
 gene;tss;island
 
 0.019879
 
 0.0437893
 
 Not queried
 
  0.7410537
 
       NA
 
           NA
  
 
  
 19
 
  54415702
 
  54416068
 
  ENSEMBL   UCSC 
 
  367
 
  6
 
 21045
 
 tss
 
 0.139181
 
 0.2462084
 
 Not queried
 
  0.7410313
 
       NA
 
           NA
  
 
  
 2
 
  27371537
 
  27371891
 
  ENSEMBL   UCSC 
 
  355
 
  8
 
 23256
 
 tss;island
 
 0.515772
 
 0.6664359
 
 Not queried
 
  0.7409712
 
       NA
 
           NA
  
 
  
 2
 
 242448543
 
 242448802
 
  ENSEMBL   UCSC 
 
  260
 
  4
 
 23990
 
 tss;island
 
 0.383162
 
 0.0864982
 
 Not queried
 
  0.7409691
 
       NA
 
           NA
  
 
  
 19
 
   4474970
 
   4475274
 
  ENSEMBL   UCSC 
 
  305
 
  7
 
 19733
 
 gene;island
 
 0.772282
 
 0.4590135
 
 Not queried
 
  0.7409219
 
       NA
 
           NA
  
 
  
 1
 
 201508965
 
 201509415
 
  ENSEMBL   UCSC 
 
  451
 
  5
 
 4078
 
 island
 
 0.018265
 
 0.0120667
 
 Not queried
 
  0.7409127
 
       NA
 
           NA
  
 
  
 1
 
    983260
 
    983611
 
  ENSEMBL   UCSC 
 
  352
 
  4
 
 10
 
 gene;island
 
 0.914938
 
 0.9664791
 
 Not queried
 
  0.7408998
 
       NA
 
           NA
  
 
  
 17
 
  74734106
 
  74734280
 
  ENSEMBL   UCSC 
 
  175
 
  4
 
 16797
 
 gene;tss;island
 
 0.008858
 
 0.0142027
 
 Not queried
 
  0.7408739
 
       NA
 
           NA
  
 
  
 17
 
   9862873
 
   9863081
 
  ENSEMBL   UCSC 
 
  209
 
  7
 
 17132
 
 tss
 
 0.041265
 
 0.0747379
 
 Not queried
 
  0.7407371
 
       NA
 
           NA
  
 
  
 8
 
  77618625
 
  77618893
 
  ENSEMBL   UCSC 
 
  269
 
  4
 
 39345
 
 gene
 
 0.671586
 
 0.6744875
 
 Not queried
 
  0.7406398
 
       NA
 
           NA
  
 
  
 11
 
 120080945
 
 120081259
 
  ENSEMBL   UCSC 
 
  315
 
  4
 
 7759
 
 tss;island
 
 0.008251
 
 0.0130940
 
 Not queried
 
  0.7404731
 
       NA
 
           NA
  
 
  
 14
 
  23398313
 
  23398847
 
  ENSEMBL   UCSC 
 
  535
 
  8
 
 12707
 
 island
 
 0.004146
 
 0.0041833
 
 Not queried
 
  0.7402551
 
       NA
 
           NA
  
 
  
 2
 
 223289173
 
 223289306
 
  ENSEMBL   UCSC 
 
  134
 
  4
 
 23918
 
 tss;island
 
 0.007208
 
 0.0155522
 
 Not queried
 
  0.7402444
 
       NA
 
           NA
  
 
  
 4
 
   2303638
 
   2303860
 
  ENSEMBL   UCSC 
 
  223
 
  5
 
 29887
 
 gene;island
 
 0.453600
 
 0.5809630
 
 Not queried
 
  0.7401525
 
       NA
 
           NA
  
 
  
 15
 
  89954560
 
  89955171
 
  ENSEMBL   UCSC 
 
  612
 
  6
 
 14434
 
 island
 
 0.021886
 
 0.0612983
 
 Not queried
 
  0.7401011
 
       NA
 
           NA
  
 
  
 20
 
  42544648
 
  42544794
 
  ENSEMBL   UCSC 
 
  147
 
  8
 
 25422
 
 gene;island
 
 0.020733
 
 0.0140195
 
 Not queried
 
  0.7400348
 
       NA
 
           NA
  
 
  
 20
 
  42544648
 
  42544728
 
  ENSEMBL   UCSC 
 
   81
 
  6
 
 25753
 
 tss
 
 0.034116
 
 0.0219089
 
 Not queried
 
  0.7400348
 
       NA
 
           NA
  
 
  
 4
 
 187065417
 
 187065955
 
  ENSEMBL   UCSC 
 
  539
 
  9
 
 30704
 
 tss;island
 
 0.014276
 
 0.0049608
 
 Not queried
 
  0.7398652
 
       NA
 
           NA
  
 
  
 2
 
 241185137
 
 241185296
 
  ENSEMBL   UCSC 
 
  160
 
  4
 
 25269
 
 island
 
 0.687967
 
 0.8728079
 
 Not queried
 
  0.7397008
 
       NA
 
           NA
  
 
  
 18
 
  22006316
 
  22006735
 
  ENSEMBL   UCSC 
 
  420
 
 11
 
 19437
 
 island
 
 0.042841
 
 0.0413679
 
 Not queried
 
  0.7395630
 
       NA
 
           NA
  
 
  
 9
 
  94186882
 
  94187181
 
  ENSEMBL   UCSC 
 
  300
 
  4
 
 40820
 
 tss;island
 
 0.011899
 
 0.0069906
 
 Not queried
 
  0.7394963
 
       NA
 
           NA
  
 
  
 5
 
 121414067
 
 121414425
 
  ENSEMBL   UCSC 
 
  359
 
 11
 
 32243
 
 tss
 
 0.025884
 
 0.0436495
 
 Not queried
 
  0.7394394
 
       NA
 
           NA
  
 
  
 5
 
 121414025
 
 121414425
 
  ENSEMBL   UCSC 
 
  401
 
 13
 
 33044
 
 island
 
 0.025884
 
 0.0436495
 
 Not queried
 
  0.7394394
 
       NA
 
           NA
  
 
  
 14
 
  38064490
 
  38064532
 
  ENSEMBL   UCSC 
 
   43
 
  4
 
 12362
 
 tss
 
 0.177271
 
 0.0974959
 
 Not queried
 
  0.7393415
 
       NA
 
           NA
  
 
  
 4
 
  96470237
 
  96470349
 
  ENSEMBL   UCSC 
 
  113
 
  5
 
 30505
 
 tss
 
 0.035692
 
 0.0820044
 
 Not queried
 
  0.7392249
 
       NA
 
           NA
  
 
  
 4
 
  96470053
 
  96470349
 
  ENSEMBL   UCSC 
 
  297
 
  7
 
 31191
 
 island
 
 0.022139
 
 0.0815731
 
 Not queried
 
  0.7392249
 
       NA
 
           NA
  
 
  
 17
 
  36105064
 
  36105517
 
  ENSEMBL   UCSC 
 
  454
 
  7
 
 18250
 
 island
 
 0.007417
 
 0.0060835
 
 Not queried
 
  0.7391967
 
       NA
 
           NA
  
 
  
 8
 
  26481666
 
  26481822
 
  ENSEMBL   UCSC 
 
  157
 
  4
 
 39252
 
 gene
 
 0.957087
 
 0.8453099
 
 Not queried
 
  0.7391710
 
       NA
 
           NA
  
 
  
 3
 
  38080635
 
  38080925
 
  ENSEMBL   UCSC 
 
  291
 
  5
 
 28986
 
 island
 
 0.005885
 
 0.0041283
 
 Not queried
 
  0.7391425
 
       NA
 
           NA
  
 
  
 17
 
  27038886
 
  27039132
 
  ENSEMBL   UCSC 
 
  247
 
  5
 
 17217
 
 tss
 
 0.023049
 
 0.0216314
 
 Not queried
 
  0.7391303
 
       NA
 
           NA
  
 
  
 17
 
  27038470
 
  27039132
 
  ENSEMBL   UCSC 
 
  663
 
  9
 
 18150
 
 island
 
 0.023740
 
 0.0301022
 
 Not queried
 
  0.7391303
 
       NA
 
           NA
  
 
  
 5
 
    476497
 
    476842
 
  ENSEMBL   UCSC 
 
  346
 
  4
 
 31505
 
 gene;island
 
 0.888113
 
 0.9547376
 
 Not queried
 
  0.7390883
 
       NA
 
           NA
  
 
  
 1
 
 120612147
 
 120612404
 
  ENSEMBL   UCSC 
 
  258
 
  4
 
 3666
 
 island
 
 0.008405
 
 0.0053268
 
 Not queried
 
  0.7390842
 
       NA
 
           NA
  
 
  
 10
 
  77165618
 
  77165905
 
  ENSEMBL   UCSC 
 
  288
 
  4
 
 4645
 
 gene;island
 
 0.976762
 
 0.9593439
 
 Not queried
 
  0.7389076
 
       NA
 
           NA
  
 
  
 3
 
  56502612
 
  56502814
 
  ENSEMBL   UCSC 
 
  203
 
  5
 
 28404
 
 tss;island
 
 0.048614
 
 0.0640954
 
 Not queried
 
  0.7388678
 
       NA
 
           NA
  
 
  
 8
 
 126104329
 
 126104540
 
  ENSEMBL   UCSC 
 
  212
 
  4
 
 39425
 
 gene;tss;island
 
 0.009511
 
 0.0055097
 
 Not queried
 
  0.7388268
 
       NA
 
           NA
  
 
  
 1
 
 112531263
 
 112532085
 
  ENSEMBL   UCSC 
 
  823
 
 11
 
 3599
 
 island
 
 0.015198
 
 0.0588278
 
 Not queried
 
  0.7387647
 
       NA
 
           NA
  
 
  
 12
 
  56727304
 
  56727976
 
  ENSEMBL   UCSC 
 
  673
 
 15
 
 10535
 
 island
 
 0.009870
 
 0.0044654
 
 Not queried
 
  0.7386960
 
       NA
 
           NA
  
 
  
 1
 
   1108820
 
   1109244
 
  ENSEMBL   UCSC 
 
  425
 
  6
 
 1142
 
 tss
 
 0.077583
 
 0.1714590
 
 Not queried
 
  0.7386714
 
       NA
 
           NA
  
 
  
 6
 
  33771799
 
  33772017
 
  ENSEMBL   UCSC 
 
  219
 
  4
 
 34914
 
 tss
 
 0.046068
 
 0.1058650
 
 Not queried
 
  0.7381990
 
       NA
 
           NA
  
 
  
 2
 
 241721922
 
 241722281
 
  ENSEMBL   UCSC 
 
  360
 
  4
 
 23125
 
 gene;island
 
 0.726899
 
 0.8357642
 
 Not queried
 
  0.7381787
 
       NA
 
           NA
  
 
  
 16
 
  56484937
 
  56485254
 
  ENSEMBL   UCSC 
 
  318
 
  5
 
 14759
 
 gene
 
 0.004968
 
 0.0073771
 
 Not queried
 
  0.7380705
 
       NA
 
           NA
  
 
  
 6
 
  32046663
 
  32047410
 
  ENSEMBL   UCSC 
 
  748
 
 10
 
 34034
 
 gene;island
 
 0.371463
 
 0.5882172
 
 Not queried
 
  0.7380029
 
       NA
 
           NA
  
 
  
 16
 
  56672387
 
  56672722
 
  ENSEMBL   UCSC 
 
  336
 
  6
 
 15951
 
 island
 
 0.116348
 
 0.2588869
 
 Not queried
 
  0.7379282
 
       NA
 
           NA
  
 
  
 3
 
 101292685
 
 101293020
 
  ENSEMBL   UCSC 
 
  336
 
  8
 
 28474
 
 tss
 
 0.006821
 
 0.0029200
 
 Not queried
 
  0.7378311
 
       NA
 
           NA
  
 
  
 7
 
 150754040
 
 150754283
 
  ENSEMBL   UCSC 
 
  244
 
  4
 
 37284
 
 gene;island
 
 0.696038
 
 0.8451357
 
 Not queried
 
  0.7378043
 
       NA
 
           NA
  
 
  
 12
 
   2986427
 
   2987138
 
  ENSEMBL   UCSC 
 
  712
 
  6
 
 9009
 
 gene
 
 0.012248
 
 0.0161112
 
 Not queried
 
  0.7377663
 
       NA
 
           NA
  
 
  
 4
 
   6695203
 
   6695475
 
  ENSEMBL   UCSC 
 
  273
 
  6
 
 30283
 
 tss
 
 0.543721
 
 0.7396535
 
 Not queried
 
  0.7375125
 
       NA
 
           NA
  
 
  
 5
 
 134074090
 
 134074186
 
  ENSEMBL   UCSC 
 
   97
 
  4
 
 32297
 
 tss;island
 
 0.007790
 
 0.0050931
 
 Not queried
 
  0.7375024
 
       NA
 
           NA
  
 
  
 1
 
  24306639
 
  24306777
 
  ENSEMBL   UCSC 
 
  139
 
  4
 
 324
 
 gene
 
 0.004299
 
 0.0025482
 
 Not queried
 
  0.7373317
 
       NA
 
           NA
  
 
  
 5
 
 172660796
 
 172661132
 
  ENSEMBL   UCSC 
 
  337
 
  4
 
 31903
 
 gene;island
 
 0.170279
 
 0.0641839
 
 Not queried
 
  0.7372950
 
       NA
 
           NA
  
 
  
 19
 
  54224374
 
  54224709
 
  ENSEMBL   UCSC 
 
  336
 
  4
 
 21038
 
 tss
 
 0.441018
 
 0.5565883
 
 Not queried
 
  0.7371572
 
       NA
 
           NA
  
 
  
 8
 
 145813981
 
 145814390
 
  ENSEMBL   UCSC 
 
  410
 
  6
 
 39528
 
 gene;island
 
 0.486728
 
 0.6745944
 
 Not queried
 
  0.7371251
 
       NA
 
           NA
  
 
  
 19
 
  38182773
 
  38183210
 
  ENSEMBL   UCSC 
 
  438
 
  5
 
 20009
 
 gene
 
 0.021382
 
 0.0181963
 
 Not queried
 
  0.7368694
 
       NA
 
           NA
  
 
  
 19
 
  38182773
 
  38183456
 
  ENSEMBL   UCSC 
 
  684
 
 11
 
 21960
 
 island
 
 0.060106
 
 0.0419486
 
 Not queried
 
  0.7368694
 
       NA
 
           NA
  
 
  
 11
 
  43702277
 
  43702737
 
  ENSEMBL   UCSC 
 
  461
 
  7
 
 6587
 
 gene
 
 0.011252
 
 0.0153828
 
 Not queried
 
  0.7368071
 
       NA
 
           NA
  
 
  
 7
 
  23144799
 
  23145311
 
  ENSEMBL   UCSC 
 
  513
 
 10
 
 37469
 
 tss;island
 
 0.004388
 
 0.0057246
 
 Not queried
 
  0.7367432
 
       NA
 
           NA
  
 
  
 9
 
 108006611
 
 108007124
 
  ENSEMBL   UCSC 
 
  514
 
  4
 
 40925
 
 island
 
 0.006775
 
 0.0116994
 
 Not queried
 
  0.7364703
 
       NA
 
           NA
  
 
  
 1
 
  78353198
 
  78353422
 
  ENSEMBL   UCSC 
 
  225
 
  4
 
 1708
 
 tss;island
 
 0.988126
 
 0.9836009
 
 Not queried
 
  0.7364661
 
       NA
 
           NA
  
 
  
 11
 
 118938260
 
 118938596
 
  ENSEMBL   UCSC 
 
  337
 
  8
 
 8869
 
 island
 
 0.003161
 
 0.0031528
 
 Not queried
 
  0.7363924
 
       NA
 
           NA
  
 
  
 4
 
 165304842
 
 165305334
 
  ENSEMBL   UCSC 
 
  493
 
  7
 
 30664
 
 tss;island
 
 0.042121
 
 0.1158525
 
 Not queried
 
  0.7363392
 
       NA
 
           NA
  
 
  
 19
 
  34311123
 
  34311562
 
  ENSEMBL   UCSC 
 
  440
 
  5
 
 21883
 
 island
 
 0.047756
 
 0.0336996
 
 Not queried
 
  0.7362998
 
       NA
 
           NA
  
 
  
 19
 
  57828900
 
  57829175
 
  ENSEMBL   UCSC 
 
  276
 
  4
 
 22464
 
 island
 
 0.361057
 
 0.5327609
 
 Not queried
 
  0.7361865
 
       NA
 
           NA
  
 
  
 6
 
 111303046
 
 111303269
 
  ENSEMBL   UCSC 
 
  224
 
  8
 
 35174
 
 tss
 
 0.021469
 
 0.0134097
 
 Not queried
 
  0.7358250
 
       NA
 
           NA
  
 
  
 2
 
  74981765
 
  74981960
 
  ENSEMBL   UCSC 
 
  196
 
  4
 
 24452
 
 island
 
 0.648743
 
 0.7677519
 
 Not queried
 
  0.7357944
 
       NA
 
           NA
  
 
  
 5
 
  65018493
 
  65018638
 
  ENSEMBL   UCSC 
 
  146
 
  4
 
 31633
 
 gene;tss;island
 
 0.009966
 
 0.0073101
 
 Not queried
 
  0.7357855
 
       NA
 
           NA
  
 
  
 19
 
  52956622
 
  52956832
 
  ENSEMBL   UCSC 
 
  211
 
  8
 
 22358
 
 island
 
 0.538059
 
 0.2416576
 
 Not queried
 
  0.7356828
 
       NA
 
           NA
  
 
  
 6
 
  32190281
 
  32190556
 
  ENSEMBL   UCSC 
 
  276
 
  6
 
 34100
 
 gene
 
 0.885620
 
 0.8196158
 
 Not queried
 
  0.7355811
 
       NA
 
           NA
  
 
  
 16
 
    474271
 
    474528
 
  ENSEMBL   UCSC 
 
  258
 
  4
 
 14967
 
 tss;island
 
 0.636578
 
 0.8296326
 
 Not queried
 
  0.7355071
 
       NA
 
           NA
  
 
  
 4
 
 109541739
 
 109542144
 
  ENSEMBL   UCSC 
 
  406
 
  6
 
 30088
 
 gene
 
 0.003334
 
 0.0027341
 
 Not queried
 
  0.7353714
 
       NA
 
           NA
  
 
  
 2
 
 231921295
 
 231921595
 
  ENSEMBL   UCSC 
 
  301
 
  6
 
 23937
 
 tss
 
 0.005682
 
 0.0051725
 
 Not queried
 
  0.7353292
 
       NA
 
           NA
  
 
  
 5
 
  61699692
 
  61700164
 
  ENSEMBL   UCSC 
 
  473
 
  9
 
 32825
 
 island
 
 0.013568
 
 0.0094214
 
 Not queried
 
  0.7352489
 
       NA
 
           NA
  
 
  
 6
 
  41715166
 
  41715322
 
  ENSEMBL   UCSC 
 
  157
 
  4
 
 34971
 
 tss
 
 0.617075
 
 0.7736938
 
 Not queried
 
  0.7351206
 
       NA
 
           NA
  
 
  
 6
 
  32407289
 
  32407570
 
  ENSEMBL   UCSC 
 
  282
 
  4
 
 34872
 
 tss
 
 0.012832
 
 0.0166668
 
 Not queried
 
  0.7349405
 
       NA
 
           NA
  
 
  
 17
 
  34957477
 
  34957969
 
  ENSEMBL   UCSC 
 
  493
 
  8
 
 17282
 
 tss;island
 
 0.017265
 
 0.0113529
 
 Not queried
 
  0.7348415
 
       NA
 
           NA
  
 
  
 12
 
 125348525
 
 125348591
 
  ENSEMBL   UCSC 
 
   67
 
  4
 
 10109
 
 tss
 
 0.055381
 
 0.0344385
 
 Not queried
 
  0.7348350
 
       NA
 
           NA
  
 
  
 19
 
   5824065
 
   5824300
 
  ENSEMBL   UCSC 
 
  236
 
  4
 
 19755
 
 gene;island
 
 0.849226
 
 0.8951959
 
 Not queried
 
  0.7348031
 
       NA
 
           NA
  
 
  
 7
 
   8483249
 
   8483710
 
  ENSEMBL   UCSC 
 
  462
 
  4
 
 36893
 
 gene;island
 
 0.086241
 
 0.0647279
 
 Not queried
 
  0.7346857
 
       NA
 
           NA
  
 
  
 1
 
 201196031
 
 201196197
 
  ENSEMBL   UCSC 
 
  167
 
  4
 
 938
 
 gene;island
 
 0.591708
 
 0.7796146
 
 Not queried
 
  0.7345926
 
       NA
 
           NA
  
 
  
 1
 
   1102276
 
   1102446
 
  ENSEMBL   UCSC 
 
  171
 
  5
 
 1139
 
 tss;island
 
 0.479137
 
 0.6292191
 
 Not queried
 
  0.7344137
 
       NA
 
           NA
  
 
  
 2
 
 203776787
 
 203776997
 
  ENSEMBL   UCSC 
 
  211
 
  6
 
 22994
 
 gene;tss;island
 
 0.005160
 
 0.0031078
 
 Not queried
 
  0.7342623
 
       NA
 
           NA
  
 
  
 10
 
 120355201
 
 120355427
 
  ENSEMBL   UCSC 
 
  227
 
  7
 
 5396
 
 tss
 
 0.073236
 
 0.1983834
 
 Not queried
 
  0.7341873
 
       NA
 
           NA
  
 
  
 5
 
 140767197
 
 140767412
 
  ENSEMBL   UCSC 
 
  216
 
  7
 
 31827
 
 gene;tss;island
 
 0.107734
 
 0.1230391
 
 Not queried
 
  0.7340279
 
       NA
 
           NA
  
 
  
 4
 
  42399699
 
  42399851
 
  ENSEMBL   UCSC 
 
  153
 
  6
 
 30367
 
 tss
 
 0.014703
 
 0.0048866
 
 Not queried
 
  0.7339554
 
       NA
 
           NA
  
 
  
 18
 
  74091393
 
  74091787
 
  ENSEMBL   UCSC 
 
  395
 
  5
 
 19130
 
 gene;island
 
 0.895458
 
 0.9721687
 
 Not queried
 
  0.7338926
 
       NA
 
           NA
  
 
  
 6
 
  72298242
 
  72298704
 
  ENSEMBL   UCSC 
 
  463
 
  5
 
 36244
 
 island
 
 0.680790
 
 0.7244444
 
 Not queried
 
  0.7337464
 
       NA
 
           NA
  
 
  
 6
 
  47277680
 
  47278002
 
  ENSEMBL   UCSC 
 
  323
 
  6
 
 35023
 
 tss
 
 0.008276
 
 0.0122576
 
 Not queried
 
  0.7336318
 
       NA
 
           NA
  
 
  
 11
 
 123396039
 
 123396505
 
  ENSEMBL   UCSC 
 
  467
 
  6
 
 7778
 
 tss
 
 0.787631
 
 0.8988010
 
 Not queried
 
  0.7334422
 
       NA
 
           NA
  
 
  
 20
 
   5930982
 
   5931158
 
  ENSEMBL   UCSC 
 
  177
 
  4
 
 25353
 
 gene
 
 0.005456
 
 0.0038578
 
 Not queried
 
  0.7332489
 
       NA
 
           NA
  
 
  
 19
 
  19971578
 
  19972043
 
  ENSEMBL   UCSC 
 
  466
 
  4
 
 21829
 
 island
 
 0.161126
 
 0.0974485
 
 Not queried
 
  0.7331200
 
       NA
 
           NA
  
 
  
 14
 
  21769105
 
  21769379
 
  ENSEMBL   UCSC 
 
  275
 
  5
 
 12012
 
 gene;island
 
 0.027493
 
 0.0049160
 
 Not queried
 
  0.7330607
 
       NA
 
           NA
  
 
  
 19
 
  52074293
 
  52074501
 
  ENSEMBL   UCSC 
 
  209
 
  8
 
 20998
 
 tss;island
 
 0.004560
 
 0.0034803
 
 Not queried
 
  0.7330072
 
       NA
 
           NA
  
 
  
 1
 
   6052487
 
   6052500
 
  ENSEMBL   UCSC 
 
   14
 
  4
 
 159
 
 gene
 
 0.003832
 
 0.0023152
 
 Not queried
 
  0.7328773
 
       NA
 
           NA
  
 
  
 6
 
 170582184
 
 170582543
 
  ENSEMBL   UCSC 
 
  360
 
  4
 
 36686
 
 island
 
 0.708107
 
 0.8228962
 
 Not queried
 
  0.7328703
 
       NA
 
           NA
  
 
  
 2
 
 217276744
 
 217277354
 
  ENSEMBL   UCSC 
 
  611
 
 11
 
 23868
 
 tss;island
 
 0.007883
 
 0.0079601
 
 Not queried
 
  0.7326558
 
       NA
 
           NA
  
 
  
 11
 
    811545
 
    811791
 
  ENSEMBL   UCSC 
 
  247
 
  5
 
 6392
 
 gene;island
 
 0.666340
 
 0.9364514
 
 Not queried
 
  0.7324847
 
       NA
 
           NA
  
 
  
 19
 
  46999055
 
  46999444
 
  ENSEMBL   UCSC 
 
  390
 
 10
 
 22175
 
 island
 
 0.200410
 
 0.0474928
 
 Not queried
 
  0.7324756
 
       NA
 
           NA
  
 
  
 7
 
 157536681
 
 157537120
 
  ENSEMBL   UCSC 
 
  440
 
  5
 
 37341
 
 gene
 
 0.655107
 
 0.8058740
 
 Not queried
 
  0.7324706
 
       NA
 
           NA
  
 
  
 6
 
  83073619
 
  83073964
 
  ENSEMBL   UCSC 
 
  346
 
  8
 
 34401
 
 gene;island
 
 0.016819
 
 0.0254325
 
 Not queried
 
  0.7322561
 
       NA
 
           NA
  
 
  
 5
 
  43042108
 
  43042233
 
  ENSEMBL   UCSC 
 
  126
 
  4
 
 32044
 
 tss
 
 0.009366
 
 0.0060018
 
 Not queried
 
  0.7320962
 
       NA
 
           NA
  
 
  
 22
 
  21986960
 
  21987048
 
  ENSEMBL   UCSC 
 
   89
 
  6
 
 26996
 
 tss
 
 0.301746
 
 0.1722766
 
 Not queried
 
  0.7319953
 
       NA
 
           NA
  
 
  
 1
 
  40041376
 
  40041788
 
  ENSEMBL   UCSC 
 
  413
 
  4
 
 448
 
 gene;island
 
 0.005508
 
 0.0111955
 
 Not queried
 
  0.7316374
 
       NA
 
           NA
  
 
  
 12
 
 114404181
 
 114404317
 
  ENSEMBL   UCSC 
 
  137
 
  5
 
 10031
 
 tss
 
 0.014366
 
 0.0085499
 
 Not queried
 
  0.7315704
 
       NA
 
           NA
  
 
  
 6
 
  46655306
 
  46655543
 
  ENSEMBL   UCSC 
 
  238
 
  7
 
 35021
 
 tss;island
 
 0.671769
 
 0.6010817
 
 Not queried
 
  0.7315224
 
       NA
 
           NA
  
 
  
 1
 
  40627050
 
  40627188
 
  ENSEMBL   UCSC 
 
  139
 
  5
 
 452
 
 gene
 
 0.006899
 
 0.0114401
 
 Not queried
 
  0.7314989
 
       NA
 
           NA
  
 
  
 12
 
  52281810
 
  52282079
 
  ENSEMBL   UCSC 
 
  270
 
  4
 
 9133
 
 gene
 
 0.749736
 
 0.8278319
 
 Not queried
 
  0.7313851
 
       NA
 
           NA
  
 
  
 1
 
  32041507
 
  32042037
 
  ENSEMBL   UCSC 
 
  531
 
  7
 
 1448
 
 tss
 
 0.410211
 
 0.2525068
 
 Not queried
 
  0.7312177
 
       NA
 
           NA
  
 
  
 1
 
  18967220
 
  18967570
 
  ENSEMBL   UCSC 
 
  351
 
  4
 
 274
 
 gene;island
 
 0.099185
 
 0.1476397
 
 Not queried
 
  0.7312005
 
       NA
 
           NA
  
 
  
 5
 
  34043333
 
  34043509
 
  ENSEMBL   UCSC 
 
  177
 
  4
 
 32011
 
 tss
 
 0.867132
 
 0.8072622
 
 Not queried
 
  0.7311265
 
       NA
 
           NA
  
 
  
 7
 
   1443496
 
   1443857
 
  ENSEMBL   UCSC 
 
  362
 
  5
 
 38102
 
 island
 
 0.064429
 
 0.0213950
 
 Not queried
 
  0.7310082
 
       NA
 
           NA
  
 
  
 8
 
 100905962
 
 100906373
 
  ENSEMBL   UCSC 
 
  412
 
  8
 
 39838
 
 tss
 
 0.013549
 
 0.0268047
 
 Not queried
 
  0.7309277
 
       NA
 
           NA
  
 
  
 18
 
  48346350
 
  48346843
 
  ENSEMBL   UCSC 
 
  494
 
  7
 
 19114
 
 gene
 
 0.018011
 
 0.0060746
 
 Not queried
 
  0.7308614
 
       NA
 
           NA
  
 
  
 16
 
   1608794
 
   1608969
 
  ENSEMBL   UCSC 
 
  176
 
  4
 
 14578
 
 gene;island
 
 0.857801
 
 0.9068643
 
 Not queried
 
  0.7308119
 
       NA
 
           NA
  
 
  
 12
 
  58240787
 
  58241215
 
  ENSEMBL   UCSC 
 
  429
 
  8
 
 9830
 
 tss;island
 
 0.003281
 
 0.0055960
 
 Not queried
 
  0.7307381
 
       NA
 
           NA
  
 
  
 17
 
  61819335
 
  61819520
 
  ENSEMBL   UCSC 
 
  186
 
  4
 
 17569
 
 tss
 
 0.015378
 
 0.0064397
 
 Not queried
 
  0.7306976
 
       NA
 
           NA
  
 
  
 11
 
 125439061
 
 125439882
 
  ENSEMBL   UCSC 
 
  822
 
 10
 
 8925
 
 island
 
 0.008471
 
 0.0099477
 
 Not queried
 
  0.7305854
 
       NA
 
           NA
  
 
  
 5
 
 167913298
 
 167913534
 
  ENSEMBL   UCSC 
 
  237
 
  5
 
 33371
 
 island
 
 0.004818
 
 0.0090620
 
 Not queried
 
  0.7304112
 
       NA
 
           NA
  
 
  
 2
 
  85555240
 
  85555614
 
  ENSEMBL   UCSC 
 
  375
 
  9
 
 24475
 
 island
 
 0.016051
 
 0.0106351
 
 Not queried
 
  0.7303345
 
       NA
 
           NA
  
 
  
 3
 
 186284968
 
 186285398
 
  ENSEMBL   UCSC 
 
  431
 
  8
 
 28073
 
 gene;island
 
 0.009871
 
 0.0070783
 
 Not queried
 
  0.7302902
 
       NA
 
           NA
  
 
  
 9
 
  34620091
 
  34620572
 
  ENSEMBL   UCSC 
 
  482
 
  5
 
 40879
 
 island
 
 0.008314
 
 0.0078265
 
 Not queried
 
  0.7302541
 
       NA
 
           NA
  
 
  
 11
 
 133800685
 
 133800913
 
  ENSEMBL   UCSC 
 
  229
 
  4
 
 6986
 
 gene;island
 
 0.524539
 
 0.6646680
 
 Not queried
 
  0.7301174
 
       NA
 
           NA
  
 
  
 6
 
 166139905
 
 166140706
 
  ENSEMBL   UCSC 
 
  802
 
  8
 
 36621
 
 island
 
 0.649254
 
 0.6620319
 
 Not queried
 
  0.7299781
 
       NA
 
           NA
  
 
  
 6
 
   1394986
 
   1395278
 
  ENSEMBL   UCSC 
 
  293
 
  4
 
 33532
 
 gene;island
 
 0.416053
 
 0.3838278
 
 Not queried
 
  0.7298823
 
       NA
 
           NA
  
 
  
 19
 
  13227474
 
  13227748
 
  ENSEMBL   UCSC 
 
  275
 
  6
 
 20524
 
 tss
 
 0.011879
 
 0.0045740
 
 Not queried
 
  0.7298705
 
       NA
 
           NA
  
 
  
 12
 
 125478005
 
 125478251
 
  ENSEMBL   UCSC 
 
  247
 
  6
 
 10981
 
 island
 
 0.005808
 
 0.0057626
 
 Not queried
 
  0.7298698
 
       NA
 
           NA
  
 
  
 19
 
  19843923
 
  19844368
 
  ENSEMBL   UCSC 
 
  446
 
  9
 
 20651
 
 tss
 
 0.040744
 
 0.0198826
 
 Not queried
 
  0.7298679
 
       NA
 
           NA
  
 
  
 10
 
  28287388
 
  28287942
 
  ENSEMBL   UCSC 
 
  555
 
  5
 
 4544
 
 gene
 
 0.012072
 
 0.0123308
 
 Not queried
 
  0.7297871
 
       NA
 
           NA
  
 
  
 18
 
  77203371
 
  77203667
 
  ENSEMBL   UCSC 
 
  297
 
  4
 
 19139
 
 gene;island
 
 0.745590
 
 0.8363448
 
 Not queried
 
  0.7297836
 
       NA
 
           NA
  
 
  
 13
 
 113489734
 
 113490012
 
  ENSEMBL   UCSC 
 
  279
 
  4
 
 11258
 
 gene;island
 
 0.953410
 
 0.9603523
 
 Not queried
 
  0.7297614
 
       NA
 
           NA
  
 
  
 1
 
 170630397
 
 170630734
 
  ENSEMBL   UCSC 
 
  338
 
  5
 
 3961
 
 island
 
 0.036849
 
 0.0532323
 
 Not queried
 
  0.7297312
 
       NA
 
           NA
  
 
  
 3
 
  23958490
 
  23959054
 
  ENSEMBL   UCSC 
 
  565
 
 10
 
 27702
 
 gene;tss;island
 
 0.009094
 
 0.0065942
 
 Not queried
 
  0.7297270
 
       NA
 
           NA
  
 
  
 15
 
  65578830
 
  65579557
 
  ENSEMBL   UCSC 
 
  728
 
 10
 
 14230
 
 island
 
 0.005140
 
 0.0057850
 
 Not queried
 
  0.7296736
 
       NA
 
           NA
  
 
  
 1
 
 247614654
 
 247614943
 
  ENSEMBL   UCSC 
 
  290
 
  5
 
 1126
 
 gene;island
 
 0.523784
 
 0.6830392
 
 Not queried
 
  0.7294897
 
       NA
 
           NA
  
 
  
 4
 
   1504989
 
   1505559
 
  ENSEMBL   UCSC 
 
  571
 
  6
 
 30788
 
 island
 
 0.632209
 
 0.7486146
 
 Not queried
 
  0.7294652
 
       NA
 
           NA
  
 
  
 17
 
  63096911
 
  63097195
 
  ENSEMBL   UCSC 
 
  285
 
  4
 
 18670
 
 island
 
 0.012829
 
 0.0200757
 
 Not queried
 
  0.7294446
 
       NA
 
           NA
  
 
  
 6
 
   3258929
 
   3259246
 
  ENSEMBL   UCSC 
 
  318
 
  6
 
 35386
 
 island
 
 0.014503
 
 0.0058093
 
 Not queried
 
  0.7294420
 
       NA
 
           NA
  
 
  
 3
 
 131221642
 
 131222106
 
  ENSEMBL   UCSC 
 
  465
 
  9
 
 29481
 
 island
 
 0.002793
 
 0.0048047
 
 Not queried
 
  0.7292947
 
       NA
 
           NA
  
 
  
 19
 
  51225848
 
  51226557
 
  ENSEMBL   UCSC 
 
  710
 
  6
 
 20984
 
 tss;island
 
 0.283017
 
 0.3378566
 
 Not queried
 
  0.7292746
 
       NA
 
           NA
  
 
  
 3
 
 187463496
 
 187463617
 
  ENSEMBL   UCSC 
 
  122
 
  5
 
 28779
 
 tss
 
 0.003134
 
 0.0080367
 
 Not queried
 
  0.7292405
 
       NA
 
           NA
  
 
  
 3
 
 187463294
 
 187463617
 
  ENSEMBL   UCSC 
 
  324
 
  7
 
 29763
 
 island
 
 0.003134
 
 0.0080367
 
 Not queried
 
  0.7292405
 
       NA
 
           NA
  
 
  
 4
 
  66535145
 
  66535575
 
  ENSEMBL   UCSC 
 
  431
 
  7
 
 30013
 
 gene
 
 0.023882
 
 0.0586846
 
 Not queried
 
  0.7292308
 
       NA
 
           NA
  
 
  
 4
 
  66535145
 
  66535732
 
  ENSEMBL   UCSC 
 
  588
 
  9
 
 31078
 
 island
 
 0.023882
 
 0.0444677
 
 Not queried
 
  0.7292308
 
       NA
 
           NA
  
 
  
 1
 
 153930510
 
 153930915
 
  ENSEMBL   UCSC 
 
  406
 
  5
 
 769
 
 gene;island
 
 0.006062
 
 0.0066988
 
 Not queried
 
  0.7292112
 
       NA
 
           NA
  
 
  
 6
 
  83073619
 
  83073954
 
  ENSEMBL   UCSC 
 
  336
 
  7
 
 35090
 
 tss
 
 0.016441
 
 0.0237686
 
 Not queried
 
  0.7287910
 
       NA
 
           NA
  
 
  
 11
 
   1483099
 
   1483390
 
  ENSEMBL   UCSC 
 
  292
 
  4
 
 7925
 
 island
 
 0.928963
 
 0.9513359
 
 Not queried
 
  0.7287735
 
       NA
 
           NA
  
 
  
 16
 
    737214
 
    737490
 
  ENSEMBL   UCSC 
 
  277
 
  4
 
 14538
 
 gene;island
 
 0.909610
 
 0.8124560
 
 Not queried
 
  0.7287068
 
       NA
 
           NA
  
 
  
 16
 
  30103218
 
  30103624
 
  ENSEMBL   UCSC 
 
  407
 
  5
 
 15179
 
 tss
 
 0.007869
 
 0.0043903
 
 Not queried
 
  0.7286646
 
       NA
 
           NA
  
 
  
 1
 
  91192451
 
  91192803
 
  ENSEMBL   UCSC 
 
  353
 
  5
 
 3464
 
 island
 
 0.100851
 
 0.1170582
 
 Not queried
 
  0.7285479
 
       NA
 
           NA
  
 
  
 4
 
 103940876
 
 103941300
 
  ENSEMBL   UCSC 
 
  425
 
  9
 
 30524
 
 tss
 
 0.009043
 
 0.0083466
 
 Not queried
 
  0.7284337
 
       NA
 
           NA
  
 
  
 4
 
 103940711
 
 103941300
 
  ENSEMBL   UCSC 
 
  590
 
 11
 
 31213
 
 island
 
 0.009043
 
 0.0083466
 
 Not queried
 
  0.7284337
 
       NA
 
           NA
  
 
  
 13
 
  27744825
 
  27745019
 
  ENSEMBL   UCSC 
 
  195
 
  5
 
 11114
 
 gene
 
 0.002930
 
 0.0020313
 
 Not queried
 
  0.7283884
 
       NA
 
           NA
  
 
  
 1
 
   6663269
 
   6663664
 
  ENSEMBL   UCSC 
 
  396
 
  5
 
 1212
 
 tss;island
 
 0.020557
 
 0.0135014
 
 Not queried
 
  0.7282879
 
       NA
 
           NA
  
 
  
 2
 
 160919147
 
 160919532
 
  ENSEMBL   UCSC 
 
  386
 
 10
 
 23684
 
 tss
 
 0.038806
 
 0.0426062
 
 Not queried
 
  0.7281954
 
       NA
 
           NA
  
 
  
 8
 
  12869510
 
  12869722
 
  ENSEMBL   UCSC 
 
  213
 
  5
 
 39210
 
 gene;tss
 
 0.597227
 
 0.8826774
 
 Not queried
 
  0.7281778
 
       NA
 
           NA
  
 
  
 3
 
  99536008
 
  99536406
 
  ENSEMBL   UCSC 
 
  399
 
  7
 
 27874
 
 gene;tss;island
 
 0.008511
 
 0.0108591
 
 Not queried
 
  0.7281353
 
       NA
 
           NA
  
 
  
 16
 
  67062687
 
  67062970
 
  ENSEMBL   UCSC 
 
  284
 
  7
 
 15290
 
 tss;island
 
 0.010583
 
 0.0077365
 
 Not queried
 
  0.7281293
 
       NA
 
           NA
  
 
  
 12
 
 106460568
 
 106461007
 
  ENSEMBL   UCSC 
 
  440
 
  5
 
 9276
 
 gene;island
 
 0.415199
 
 0.5404273
 
 Not queried
 
  0.7281192
 
       NA
 
           NA
  
 
  
 19
 
   9473565
 
   9473880
 
  ENSEMBL   UCSC 
 
  316
 
 10
 
 21497
 
 island
 
 0.137625
 
 0.0789921
 
 Not queried
 
  0.7280293
 
       NA
 
           NA
  
 
  
 8
 
 143545214
 
 143545574
 
  ENSEMBL   UCSC 
 
  361
 
  5
 
 40635
 
 island
 
 0.023071
 
 0.1290146
 
 Not queried
 
  0.7279929
 
       NA
 
           NA
  
 
  
 11
 
  76813828
 
  76814245
 
  ENSEMBL   UCSC 
 
  418
 
  4
 
 6838
 
 gene;island
 
 0.524170
 
 0.5970384
 
 Not queried
 
  0.7278180
 
       NA
 
           NA
  
 
  
 13
 
  96743492
 
  96743895
 
  ENSEMBL   UCSC 
 
  404
 
  5
 
 11195
 
 gene;island
 
 0.029402
 
 0.0091570
 
 Not queried
 
  0.7276759
 
       NA
 
           NA
  
 
  
 15
 
  25318197
 
  25318384
 
  ENSEMBL   UCSC 
 
  188
 
  4
 
 13523
 
 tss
 
 0.723124
 
 0.8305457
 
 Not queried
 
  0.7275662
 
       NA
 
           NA
  
 
  
 19
 
   4173254
 
   4173482
 
  ENSEMBL   UCSC 
 
  229
 
  4
 
 21366
 
 island
 
 0.057883
 
 0.0980698
 
 Not queried
 
  0.7274618
 
       NA
 
           NA
  
 
  
 6
 
   7390083
 
   7390340
 
  ENSEMBL   UCSC 
 
  258
 
  6
 
 33552
 
 gene
 
 0.004833
 
 0.0077397
 
 Not queried
 
  0.7271432
 
       NA
 
           NA
  
 
  
 12
 
 125002007
 
 125002474
 
  ENSEMBL   UCSC 
 
  468
 
  4
 
 9400
 
 gene;island
 
 0.935062
 
 0.9795890
 
 Not queried
 
  0.7268951
 
       NA
 
           NA
  
 
  
 7
 
  47344579
 
  47344750
 
  ENSEMBL   UCSC 
 
  172
 
  4
 
 37002
 
 gene
 
 0.780891
 
 0.6945707
 
 Not queried
 
  0.7268488
 
       NA
 
           NA
  
 
  
 2
 
 131513608
 
 131513735
 
  ENSEMBL   UCSC 
 
  128
 
  5
 
 23630
 
 tss
 
 0.057067
 
 0.0348295
 
 Not queried
 
  0.7267936
 
       NA
 
           NA
  
 
  
 7
 
 155255122
 
 155255369
 
  ENSEMBL   UCSC 
 
  248
 
  4
 
 37309
 
 gene;island
 
 0.167102
 
 0.4027258
 
 Not queried
 
  0.7267275
 
       NA
 
           NA
  
 
  
 8
 
  20112813
 
  20113188
 
  ENSEMBL   UCSC 
 
  376
 
  7
 
 39576
 
 tss
 
 0.366544
 
 0.4193738
 
 Not queried
 
  0.7265712
 
       NA
 
           NA
  
 
  
 17
 
  75471193
 
  75471491
 
  ENSEMBL   UCSC 
 
  299
 
  6
 
 16811
 
 gene
 
 0.019403
 
 0.0271808
 
 Not queried
 
  0.7265614
 
       NA
 
           NA
  
 
  
 1
 
  46216399
 
  46216737
 
  ENSEMBL   UCSC 
 
  339
 
  6
 
 3208
 
 island
 
 0.003692
 
 0.0032049
 
 Not queried
 
  0.7265181
 
       NA
 
           NA
  
 
  
 6
 
  39197256
 
  39197537
 
  ENSEMBL   UCSC 
 
  282
 
  7
 
 34954
 
 tss
 
 0.014764
 
 0.0248000
 
 Not queried
 
  0.7264354
 
       NA
 
           NA
  
 
  
 11
 
  32454718
 
  32455735
 
  ENSEMBL   UCSC 
 
 1018
 
 12
 
 6567
 
 gene;island
 
 0.168769
 
 0.1936260
 
 Not queried
 
  0.7262221
 
       NA
 
           NA
  
 
  
 4
 
  15964913
 
  15965102
 
  ENSEMBL   UCSC 
 
  190
 
  5
 
 30312
 
 tss
 
 0.010400
 
 0.0186690
 
 Not queried
 
  0.7261650
 
       NA
 
           NA
  
 
  
 4
 
 186741788
 
 186742139
 
  ENSEMBL   UCSC 
 
  352
 
  5
 
 30213
 
 gene
 
 0.815850
 
 0.7419231
 
 Not queried
 
  0.7255921
 
       NA
 
           NA
  
 
  
 11
 
   1940856
 
   1941145
 
  ENSEMBL   UCSC 
 
  290
 
  4
 
 6437
 
 gene
 
 0.524044
 
 0.5207466
 
 Not queried
 
  0.7252719
 
       NA
 
           NA
  
 
  
 10
 
 132873657
 
 132873903
 
  ENSEMBL   UCSC 
 
  247
 
  5
 
 6275
 
 island
 
 0.536774
 
 0.7813287
 
 Not queried
 
  0.7252552
 
       NA
 
           NA
  
 
  
 1
 
  10725218
 
  10725332
 
  ENSEMBL   UCSC 
 
  115
 
  4
 
 215
 
 gene;island
 
 0.960273
 
 0.9151400
 
 Not queried
 
  0.7251948
 
       NA
 
           NA
  
 
  
 6
 
  31781814
 
  31782158
 
  ENSEMBL   UCSC 
 
  345
 
  4
 
 33966
 
 gene;tss;island
 
 0.387290
 
 0.5636511
 
 Not queried
 
  0.7250422
 
       NA
 
           NA
  
 
  
 4
 
  56458449
 
  56458622
 
  ENSEMBL   UCSC 
 
  174
 
  4
 
 30404
 
 tss
 
 0.737929
 
 0.7823050
 
 Not queried
 
  0.7249202
 
       NA
 
           NA
  
 
  
 11
 
  57434378
 
  57434710
 
  ENSEMBL   UCSC 
 
  333
 
  5
 
 7307
 
 tss;island
 
 0.019492
 
 0.0088624
 
 Not queried
 
  0.7248782
 
       NA
 
           NA
  
 
  
 12
 
  62586552
 
  62586892
 
  ENSEMBL   UCSC 
 
  341
 
  6
 
 9832
 
 tss
 
 0.002671
 
 0.0055859
 
 Not queried
 
  0.7248361
 
       NA
 
           NA
  
 
  
 5
 
 118603742
 
 118603938
 
  ENSEMBL   UCSC 
 
  197
 
  5
 
 32236
 
 tss;island
 
 0.008580
 
 0.0073402
 
 Not queried
 
  0.7247956
 
       NA
 
           NA
  
 
  
 3
 
  97483466
 
  97483591
 
  ENSEMBL   UCSC 
 
  126
 
  6
 
 28458
 
 tss
 
 0.017657
 
 0.0054289
 
 Not queried
 
  0.7247941
 
       NA
 
           NA
  
 
  
 2
 
 171572714
 
 171572902
 
  ENSEMBL   UCSC 
 
  189
 
  4
 
 22918
 
 gene;island
 
 0.016084
 
 0.0165145
 
 Not queried
 
  0.7245217
 
       NA
 
           NA
  
 
  
 13
 
 111972854
 
 111972997
 
  ENSEMBL   UCSC 
 
  144
 
  6
 
 11542
 
 tss
 
 0.842859
 
 0.8894311
 
 Not queried
 
  0.7244766
 
       NA
 
           NA
  
 
  
 3
 
  58223060
 
  58223165
 
  ENSEMBL   UCSC 
 
  106
 
  4
 
 28414
 
 tss;island
 
 0.019699
 
 0.0124207
 
 Not queried
 
  0.7243713
 
       NA
 
           NA
  
 
  
 17
 
  47210027
 
  47210368
 
  ENSEMBL   UCSC 
 
  342
 
  7
 
 16672
 
 gene;island
 
 0.021862
 
 0.0521197
 
 Not queried
 
  0.7243351
 
       NA
 
           NA
  
 
  
 7
 
    211714
 
    212112
 
  ENSEMBL   UCSC 
 
  399
 
  4
 
 36704
 
 gene
 
 0.452233
 
 0.7077922
 
 Not queried
 
  0.7240275
 
       NA
 
           NA
  
 
  
 6
 
  33133329
 
  33133752
 
  ENSEMBL   UCSC 
 
  424
 
  9
 
 34195
 
 gene
 
 0.614485
 
 0.6931282
 
 Not queried
 
  0.7239844
 
       NA
 
           NA
  
 
  
 14
 
 102785760
 
 102785987
 
  ENSEMBL   UCSC 
 
  228
 
  4
 
 12643
 
 tss;island
 
 0.011509
 
 0.0098154
 
 Not queried
 
  0.7239204
 
       NA
 
           NA
  
 
  
 2
 
 187713554
 
 187714279
 
  ENSEMBL   UCSC 
 
  726
 
 12
 
 24951
 
 island
 
 0.056392
 
 0.0195457
 
 Not queried
 
  0.7237995
 
       NA
 
           NA
  
 
  
 5
 
   1879525
 
   1880021
 
  ENSEMBL   UCSC 
 
  497
 
  6
 
 31549
 
 gene;island
 
 0.257208
 
 0.4060299
 
 Not queried
 
  0.7237126
 
       NA
 
           NA
  
 
  
 8
 
  82598140
 
  82598664
 
  ENSEMBL   UCSC 
 
  525
 
  6
 
 40420
 
 island
 
 0.010608
 
 0.0106074
 
 Not queried
 
  0.7236957
 
       NA
 
           NA
  
 
  
 1
 
  63249197
 
  63249213
 
  ENSEMBL   UCSC 
 
   17
 
  4
 
 1666
 
 tss
 
 0.009110
 
 0.0142954
 
 Not queried
 
  0.7234899
 
       NA
 
           NA
  
 
  
 2
 
  85839322
 
  85839722
 
  ENSEMBL   UCSC 
 
  401
 
  5
 
 23486
 
 tss
 
 0.071474
 
 0.0596243
 
 Not queried
 
  0.7234743
 
       NA
 
           NA
  
 
  
 10
 
  30026015
 
  30026143
 
  ENSEMBL   UCSC 
 
  129
 
  5
 
 5010
 
 tss;island
 
 0.066038
 
 0.0869022
 
 Not queried
 
  0.7234362
 
       NA
 
           NA
  
 
  
 13
 
  30002334
 
  30002657
 
  ENSEMBL   UCSC 
 
  324
 
  6
 
 11119
 
 gene;tss
 
 0.019620
 
 0.0258888
 
 Not queried
 
  0.7234011
 
       NA
 
           NA
  
 
  
 3
 
 196594273
 
 196594898
 
  ENSEMBL   UCSC 
 
  626
 
  8
 
 29812
 
 island
 
 0.064962
 
 0.0498033
 
 Not queried
 
  0.7233810
 
       NA
 
           NA
  
 
  
 13
 
 113660246
 
 113660495
 
  ENSEMBL   UCSC 
 
  250
 
  4
 
 11267
 
 gene;island
 
 0.762329
 
 0.9252784
 
 Not queried
 
  0.7231734
 
       NA
 
           NA
  
 
  
 2
 
 128381653
 
 128381877
 
  ENSEMBL   UCSC 
 
  225
 
  5
 
 22861
 
 gene;island
 
 0.625657
 
 0.5829545
 
 Not queried
 
  0.7229770
 
       NA
 
           NA
  
 
  
 3
 
 190580589
 
 190580644
 
  ENSEMBL   UCSC 
 
   56
 
  4
 
 28787
 
 tss
 
 0.035032
 
 0.0215841
 
 Not queried
 
  0.7229413
 
       NA
 
           NA
  
 
  
 6
 
  28784788
 
  28785059
 
  ENSEMBL   UCSC 
 
  272
 
  6
 
 35596
 
 island
 
 0.125560
 
 0.0222622
 
 Not queried
 
  0.7227208
 
       NA
 
           NA
  
 
  
 16
 
  31022234
 
  31022521
 
  ENSEMBL   UCSC 
 
  288
 
  4
 
 15202
 
 tss
 
 0.068032
 
 0.0438885
 
 Not queried
 
  0.7226442
 
       NA
 
           NA
  
 
  
 21
 
  46221839
 
  46221955
 
  ENSEMBL   UCSC 
 
  117
 
  4
 
 26588
 
 tss
 
 0.020754
 
 0.0145853
 
 Not queried
 
  0.7225957
 
       NA
 
           NA
  
 
  
 11
 
   1229901
 
   1230144
 
  ENSEMBL   UCSC 
 
  244
 
  4
 
 7907
 
 island
 
 0.540350
 
 0.6398882
 
 Not queried
 
  0.7224282
 
       NA
 
           NA
  
 
  
 3
 
 158519410
 
 158519925
 
  ENSEMBL   UCSC 
 
  516
 
  9
 
 29623
 
 island
 
 0.012704
 
 0.0081250
 
 Not queried
 
  0.7224198
 
       NA
 
           NA
  
 
  
 5
 
   1112207
 
   1112355
 
  ENSEMBL   UCSC 
 
  149
 
  6
 
 31966
 
 tss
 
 0.009033
 
 0.0137853
 
 Not queried
 
  0.7223369
 
       NA
 
           NA
  
 
  
 14
 
 103655242
 
 103655773
 
  ENSEMBL   UCSC 
 
  532
 
  5
 
 13183
 
 island
 
 0.055377
 
 0.0676636
 
 Not queried
 
  0.7222741
 
       NA
 
           NA
  
 
  
 22
 
  22007095
 
  22007286
 
  ENSEMBL   UCSC 
 
  192
 
  4
 
 26998
 
 tss;island
 
 0.088548
 
 0.1239176
 
 Not queried
 
  0.7220583
 
       NA
 
           NA
  
 
  
 3
 
  52813652
 
  52813920
 
  ENSEMBL   UCSC 
 
  269
 
  6
 
 27826
 
 gene;tss
 
 0.022343
 
 0.0475713
 
 Not queried
 
  0.7218191
 
       NA
 
           NA
  
 
  
 12
 
 100594586
 
 100594894
 
  ENSEMBL   UCSC 
 
  309
 
  5
 
 9264
 
 gene
 
 0.003929
 
 0.0055576
 
 Not queried
 
  0.7218180
 
       NA
 
           NA
  
 
  
 6
 
  39281421
 
  39281885
 
  ENSEMBL   UCSC 
 
  465
 
  5
 
 34325
 
 gene;island
 
 0.018292
 
 0.0079157
 
 Not queried
 
  0.7217354
 
       NA
 
           NA
  
 
  
 7
 
 140773422
 
 140774161
 
  ENSEMBL   UCSC 
 
  740
 
  6
 
 38967
 
 island
 
 0.034221
 
 0.0256041
 
 Not queried
 
  0.7217243
 
       NA
 
           NA
  
 
  
 19
 
  49994097
 
  49994372
 
  ENSEMBL   UCSC 
 
  276
 
  4
 
 20155
 
 gene;tss;island
 
 0.973527
 
 0.9773517
 
 Not queried
 
  0.7217095
 
       NA
 
           NA
  
 
  
 3
 
  69133862
 
  69134087
 
  ENSEMBL   UCSC 
 
  226
 
  4
 
 28440
 
 tss
 
 0.000403
 
 0.0002190
 
 Not queried
 
  0.7216579
 
       NA
 
           NA
  
 
  
 11
 
  47429649
 
  47429944
 
  ENSEMBL   UCSC 
 
  296
 
  5
 
 7287
 
 tss;island
 
 0.006444
 
 0.0086764
 
 Not queried
 
  0.7216506
 
       NA
 
           NA
  
 
  
 19
 
  35264148
 
  35264428
 
  ENSEMBL   UCSC 
 
  281
 
  9
 
 20696
 
 tss
 
 0.003556
 
 0.0022010
 
 Not queried
 
  0.7216179
 
       NA
 
           NA
  
 
  
 17
 
  78965417
 
  78965457
 
  ENSEMBL   UCSC 
 
   41
 
  4
 
 17729
 
 tss;island
 
 0.007861
 
 0.0072973
 
 Not queried
 
  0.7216022
 
       NA
 
           NA
  
 
  
 19
 
  37406932
 
  37407152
 
  ENSEMBL   UCSC 
 
  221
 
  6
 
 20005
 
 gene
 
 0.022364
 
 0.0144748
 
 Not queried
 
  0.7214894
 
       NA
 
           NA
  
 
  
 19
 
  18699118
 
  18699444
 
  ENSEMBL   UCSC 
 
  327
 
  9
 
 20623
 
 tss
 
 0.245247
 
 0.0180703
 
 Not queried
 
  0.7212960
 
       NA
 
           NA
  
 
  
 1
 
 155826844
 
 155827082
 
  ENSEMBL   UCSC 
 
  239
 
  6
 
 3834
 
 island
 
 0.004119
 
 0.0066361
 
 Not queried
 
  0.7212867
 
       NA
 
           NA
  
 
  
 2
 
 239008929
 
 239009502
 
  ENSEMBL   UCSC 
 
  574
 
  9
 
 25237
 
 island
 
 0.941435
 
 0.9641966
 
 Not queried
 
  0.7212233
 
       NA
 
           NA
  
 
  
 13
 
 114927579
 
 114928233
 
  ENSEMBL   UCSC 
 
  655
 
  8
 
 11999
 
 island
 
 0.480270
 
 0.3713920
 
 Not queried
 
  0.7211208
 
       NA
 
           NA
  
 
  
 1
 
 111681818
 
 111682281
 
  ENSEMBL   UCSC 
 
  464
 
  9
 
 680
 
 gene;tss;island
 
 0.003610
 
 0.0016488
 
 Not queried
 
  0.7210056
 
       NA
 
           NA
  
 
  
 5
 
  37835506
 
  37836392
 
  ENSEMBL   UCSC 
 
  887
 
 11
 
 31591
 
 gene;tss;island
 
 0.058403
 
 0.1214262
 
 Not queried
 
  0.7209927
 
       NA
 
           NA
  
 
  
 19
 
  12912941
 
  12913155
 
  ENSEMBL   UCSC 
 
  215
 
  4
 
 20512
 
 tss;island
 
 0.018485
 
 0.0446844
 
 Not queried
 
  0.7208109
 
       NA
 
           NA
  
 
  
 3
 
 190231933
 
 190232443
 
  ENSEMBL   UCSC 
 
  511
 
  6
 
 28082
 
 gene
 
 0.005538
 
 0.0373632
 
 Not queried
 
  0.7207581
 
       NA
 
           NA
  
 
  
 2
 
 228028601
 
 228028776
 
  ENSEMBL   UCSC 
 
  176
 
  4
 
 23057
 
 gene;tss;island
 
 0.003622
 
 0.0079035
 
 Not queried
 
  0.7207445
 
       NA
 
           NA
  
 
  
 19
 
  57106501
 
  57106852
 
  ENSEMBL   UCSC 
 
  352
 
  4
 
 22450
 
 island
 
 0.018767
 
 0.0254083
 
 Not queried
 
  0.7207432
 
       NA
 
           NA
  
 
  
 19
 
   1074727
 
   1075104
 
  ENSEMBL   UCSC 
 
  378
 
  4
 
 19641
 
 gene;island
 
 0.014166
 
 0.0224418
 
 Not queried
 
  0.7206314
 
       NA
 
           NA
  
 
  
 7
 
  75831194
 
  75831678
 
  ENSEMBL   UCSC 
 
  485
 
  8
 
 38595
 
 island
 
 0.003411
 
 0.0039661
 
 Not queried
 
  0.7205862
 
       NA
 
           NA
  
 
  
 22
 
  19929066
 
  19929557
 
  ENSEMBL   UCSC 
 
  492
 
  9
 
 26791
 
 gene;island
 
 0.005719
 
 0.0074148
 
 Not queried
 
  0.7205832
 
       NA
 
           NA
  
 
  
 22
 
  19929066
 
  19929254
 
  ENSEMBL   UCSC 
 
  189
 
  5
 
 26967
 
 tss
 
 0.005238
 
 0.0037139
 
 Not queried
 
  0.7205832
 
       NA
 
           NA
  
 
  
 13
 
 110521956
 
 110522297
 
  ENSEMBL   UCSC 
 
  342
 
  5
 
 11874
 
 island
 
 0.510617
 
 0.3897177
 
 Not queried
 
  0.7205782
 
       NA
 
           NA
  
 
  
 19
 
   3762581
 
   3762740
 
  ENSEMBL   UCSC 
 
  160
 
  6
 
 20352
 
 tss;island
 
 0.001391
 
 0.0018681
 
 Not queried
 
  0.7205713
 
       NA
 
           NA
  
 
  
 12
 
  46766537
 
  46766730
 
  ENSEMBL   UCSC 
 
  194
 
  5
 
 10349
 
 island
 
 0.010535
 
 0.0064548
 
 Not queried
 
  0.7203779
 
       NA
 
           NA
  
 
  
 14
 
  47120795
 
  47121002
 
  ENSEMBL   UCSC 
 
  208
 
  4
 
 12075
 
 gene
 
 0.775946
 
 0.8280941
 
 Not queried
 
  0.7203543
 
       NA
 
           NA
  
 
  
 17
 
  46019006
 
  46019184
 
  ENSEMBL   UCSC 
 
  179
 
  5
 
 16648
 
 gene
 
 0.019198
 
 0.0122891
 
 Not queried
 
  0.7202684
 
       NA
 
           NA
  
 
  
 2
 
 106015518
 
 106015869
 
  ENSEMBL   UCSC 
 
  352
 
  9
 
 22815
 
 gene;island
 
 0.082412
 
 0.0496176
 
 Not queried
 
  0.7201674
 
       NA
 
           NA
  
 
  
 11
 
  68227804
 
  68228228
 
  ENSEMBL   UCSC 
 
  425
 
 10
 
 8574
 
 island
 
 0.006581
 
 0.0077523
 
 Not queried
 
  0.7201392
 
       NA
 
           NA
  
 
  
 14
 
  56584485
 
  56584987
 
  ENSEMBL   UCSC 
 
  503
 
  5
 
 12416
 
 tss
 
 0.004173
 
 0.0021753
 
 Not queried
 
  0.7199185
 
       NA
 
           NA
  
 
  
 12
 
  42877429
 
  42877658
 
  ENSEMBL   UCSC 
 
  230
 
  5
 
 9644
 
 tss
 
 0.003145
 
 0.0056417
 
 Not queried
 
  0.7197747
 
       NA
 
           NA
  
 
  
 10
 
  97453638
 
  97454076
 
  ENSEMBL   UCSC 
 
  439
 
  9
 
 5967
 
 island
 
 0.012212
 
 0.0100779
 
 Not queried
 
  0.7197056
 
       NA
 
           NA
  
 
  
 4
 
 100009749
 
 100010258
 
  ENSEMBL   UCSC 
 
  510
 
 11
 
 31197
 
 island
 
 0.004349
 
 0.0058049
 
 Not queried
 
  0.7196647
 
       NA
 
           NA
  
 
  
 8
 
  19095053
 
  19095448
 
  ENSEMBL   UCSC 
 
  396
 
  5
 
 40110
 
 island
 
 0.796131
 
 0.8311553
 
 Not queried
 
  0.7196545
 
       NA
 
           NA
  
 
  
 1
 
  16939983
 
  16940181
 
  ENSEMBL   UCSC 
 
  199
 
  5
 
 1284
 
 tss
 
 0.017479
 
 0.0097181
 
 Not queried
 
  0.7195841
 
       NA
 
           NA
  
 
  
 4
 
 122854330
 
 122854532
 
  ENSEMBL   UCSC 
 
  203
 
  4
 
 30577
 
 tss
 
 0.842298
 
 0.6281650
 
 Not queried
 
  0.7194955
 
       NA
 
           NA
  
 
  
 2
 
  55458963
 
  55459287
 
  ENSEMBL   UCSC 
 
  325
 
  5
 
 22687
 
 gene;tss
 
 0.001128
 
 0.0026196
 
 Not queried
 
  0.7191219
 
       NA
 
           NA
  
 
  
 8
 
  75262469
 
  75262582
 
  ENSEMBL   UCSC 
 
  114
 
  5
 
 39774
 
 tss
 
 0.015262
 
 0.0133157
 
 Not queried
 
  0.7191092
 
       NA
 
           NA
  
 
  
 11
 
   6440690
 
   6440968
 
  ENSEMBL   UCSC 
 
  279
 
 10
 
 7110
 
 tss;island
 
 0.020493
 
 0.0068699
 
 Not queried
 
  0.7190278
 
       NA
 
           NA
  
 
  
 4
 
  52917271
 
  52917660
 
  ENSEMBL   UCSC 
 
  390
 
 10
 
 31040
 
 island
 
 0.009712
 
 0.0093883
 
 Not queried
 
  0.7188421
 
       NA
 
           NA
  
 
  
 15
 
  48623422
 
  48623668
 
  ENSEMBL   UCSC 
 
  247
 
  5
 
 13656
 
 tss;island
 
 0.007760
 
 0.0071613
 
 Not queried
 
  0.7187328
 
       NA
 
           NA
  
 
  
 16
 
   1728187
 
   1728344
 
  ENSEMBL   UCSC 
 
  158
 
  6
 
 15558
 
 island
 
 0.003363
 
 0.0013379
 
 Not queried
 
  0.7184878
 
       NA
 
           NA
  
 
  
 7
 
  30810852
 
  30810882
 
  ENSEMBL   UCSC 
 
   31
 
  5
 
 37521
 
 tss;island
 
 0.017212
 
 0.0161596
 
 Not queried
 
  0.7184661
 
       NA
 
           NA
  
 
  
 1
 
  86174125
 
  86174240
 
  ENSEMBL   UCSC 
 
  116
 
  6
 
 1723
 
 tss
 
 0.003590
 
 0.0062676
 
 Not queried
 
  0.7184582
 
       NA
 
           NA
  
 
  
 8
 
 144635260
 
 144635547
 
  ENSEMBL   UCSC 
 
  288
 
  9
 
 39950
 
 tss
 
 0.012388
 
 0.0063659
 
 Not queried
 
  0.7183314
 
       NA
 
           NA
  
 
  
 8
 
  67974213
 
  67974458
 
  ENSEMBL   UCSC 
 
  246
 
  8
 
 39327
 
 gene
 
 0.008547
 
 0.0070862
 
 Not queried
 
  0.7182543
 
       NA
 
           NA
  
 
  
 1
 
 117452304
 
 117452580
 
  ENSEMBL   UCSC 
 
  277
 
  7
 
 1866
 
 tss;island
 
 0.090571
 
 0.0665848
 
 Not queried
 
  0.7181975
 
       NA
 
           NA
  
 
  
 6
 
  30876137
 
  30876159
 
  ENSEMBL   UCSC 
 
   23
 
  5
 
 33831
 
 gene;island
 
 0.012003
 
 0.0051537
 
 Not queried
 
  0.7181940
 
       NA
 
           NA
  
 
  
 19
 
  19249194
 
  19249481
 
  ENSEMBL   UCSC 
 
  288
 
  7
 
 21804
 
 island
 
 0.003607
 
 0.0086567
 
 Not queried
 
  0.7179981
 
       NA
 
           NA
  
 
  
 10
 
  71561678
 
  71562095
 
  ENSEMBL   UCSC 
 
  418
 
  6
 
 4615
 
 gene
 
 0.013079
 
 0.0120547
 
 Not queried
 
  0.7179445
 
       NA
 
           NA
  
 
  
 16
 
  81348309
 
  81348534
 
  ENSEMBL   UCSC 
 
  226
 
  4
 
 15381
 
 tss;island
 
 0.002689
 
 0.0019618
 
 Not queried
 
  0.7179310
 
       NA
 
           NA
  
 
  
 16
 
  84328104
 
  84328391
 
  ENSEMBL   UCSC 
 
  288
 
  6
 
 15392
 
 tss;island
 
 0.007680
 
 0.0147528
 
 Not queried
 
  0.7179155
 
       NA
 
           NA
  
 
  
 4
 
   6711606
 
   6711995
 
  ENSEMBL   UCSC 
 
  390
 
  7
 
 30284
 
 tss
 
 0.001447
 
 0.0034438
 
 Not queried
 
  0.7177991
 
       NA
 
           NA
  
 
  
 19
 
  49991271
 
  49991517
 
  ENSEMBL   UCSC 
 
  247
 
  4
 
 20153
 
 gene;island
 
 0.014195
 
 0.0082741
 
 Not queried
 
  0.7175896
 
       NA
 
           NA
  
 
  
 16
 
  11367114
 
  11367509
 
  ENSEMBL   UCSC 
 
  396
 
  5
 
 15704
 
 island
 
 0.478158
 
 0.7298226
 
 Not queried
 
  0.7174678
 
       NA
 
           NA
  
 
  
 4
 
  57371368
 
  57371927
 
  ENSEMBL   UCSC 
 
  560
 
  7
 
 31068
 
 island
 
 0.065170
 
 0.5108568
 
 Not queried
 
  0.7173829
 
       NA
 
           NA
  
 
  
 22
 
  49297824
 
  49297925
 
  ENSEMBL   UCSC 
 
  102
 
  4
 
 27601
 
 island
 
 0.522149
 
 0.6777718
 
 Not queried
 
  0.7173308
 
       NA
 
           NA
  
 
  
 10
 
  42862876
 
  42863173
 
  ENSEMBL   UCSC 
 
  298
 
  4
 
 4563
 
 gene;island
 
 0.199676
 
 0.0835374
 
 Not queried
 
  0.7172676
 
       NA
 
           NA
  
 
  
 7
 
  73133982
 
  73134111
 
  ENSEMBL   UCSC 
 
  130
 
  6
 
 38566
 
 island
 
 0.013885
 
 0.0109746
 
 Not queried
 
  0.7172646
 
       NA
 
           NA
  
 
  
 6
 
  29635110
 
  29635692
 
  ENSEMBL   UCSC 
 
  583
 
  8
 
 33686
 
 gene
 
 0.368653
 
 0.3765099
 
 Not queried
 
  0.7169955
 
       NA
 
           NA
  
 
  
 11
 
  62622834
 
  62622991
 
  ENSEMBL   UCSC 
 
  158
 
  7
 
 6650
 
 gene;tss;island
 
 0.010262
 
 0.0047359
 
 Not queried
 
  0.7169388
 
       NA
 
           NA
  
 
  
 1
 
  52520593
 
  52520979
 
  ENSEMBL   UCSC 
 
  387
 
  5
 
 530
 
 gene;tss;island
 
 0.003750
 
 0.0059181
 
 Not queried
 
  0.7168057
 
       NA
 
           NA
  
 
  
 11
 
  18433500
 
  18433745
 
  ENSEMBL   UCSC 
 
  246
 
  6
 
 7184
 
 tss
 
 0.789768
 
 0.9468892
 
 Not queried
 
  0.7165552
 
       NA
 
           NA
  
 
  
 19
 
  55866305
 
  55866412
 
  ENSEMBL   UCSC 
 
  108
 
  5
 
 20228
 
 gene
 
 0.018871
 
 0.0131490
 
 Not queried
 
  0.7165028
 
       NA
 
           NA
  
 
  
 4
 
 156298166
 
 156298427
 
  ENSEMBL   UCSC 
 
  262
 
  7
 
 30650
 
 tss
 
 0.026860
 
 0.0282138
 
 Not queried
 
  0.7162731
 
       NA
 
           NA
  
 
  
 10
 
  14050305
 
  14050521
 
  ENSEMBL   UCSC 
 
  217
 
  4
 
 4507
 
 gene
 
 0.025525
 
 0.0088548
 
 Not queried
 
  0.7161881
 
       NA
 
           NA
  
 
  
 12
 
 131417943
 
 131418404
 
  ENSEMBL   UCSC 
 
  462
 
  4
 
 11011
 
 island
 
 0.287582
 
 0.4429713
 
 Not queried
 
  0.7159641
 
       NA
 
           NA
  
 
  
 8
 
 144365685
 
 144366016
 
  ENSEMBL   UCSC 
 
  332
 
  4
 
 40650
 
 island
 
 0.800860
 
 0.8929303
 
 Not queried
 
  0.7159573
 
       NA
 
           NA
  
 
  
 5
 
  77805886
 
  77806312
 
  ENSEMBL   UCSC 
 
  427
 
  4
 
 31657
 
 gene;island
 
 0.056681
 
 0.0319230
 
 Not queried
 
  0.7159311
 
       NA
 
           NA
  
 
  
 4
 
   1029410
 
   1029654
 
  ENSEMBL   UCSC 
 
  245
 
  4
 
 30761
 
 island
 
 0.397616
 
 0.5599451
 
 Not queried
 
  0.7158737
 
       NA
 
           NA
  
 
  
 19
 
  51071307
 
  51071328
 
  ENSEMBL   UCSC 
 
   22
 
  4
 
 20981
 
 tss
 
 0.029219
 
 0.0433976
 
 Not queried
 
  0.7158715
 
       NA
 
           NA
  
 
  
 19
 
  13044570
 
  13045039
 
  ENSEMBL   UCSC 
 
  470
 
  9
 
 20520
 
 tss
 
 0.008863
 
 0.0072012
 
 Not queried
 
  0.7158638
 
       NA
 
           NA
  
 
  
 11
 
  46940434
 
  46940613
 
  ENSEMBL   UCSC 
 
  180
 
  4
 
 7282
 
 tss;island
 
 0.289926
 
 0.2885041
 
 Not queried
 
  0.7158206
 
       NA
 
           NA
  
 
  
 10
 
  82168043
 
  82168153
 
  ENSEMBL   UCSC 
 
  111
 
  5
 
 5189
 
 tss
 
 0.014585
 
 0.0077289
 
 Not queried
 
  0.7156348
 
       NA
 
           NA
  
 
  
 1
 
  91190005
 
  91190620
 
  ENSEMBL   UCSC 
 
  616
 
  7
 
 3461
 
 island
 
 0.307494
 
 0.2116304
 
 Not queried
 
  0.7156146
 
       NA
 
           NA
  
 
  
 3
 
  49761600
 
  49761936
 
  ENSEMBL   UCSC 
 
  337
 
  5
 
 28334
 
 tss;island
 
 0.004039
 
 0.0073511
 
 Not queried
 
  0.7155904
 
       NA
 
           NA
  
 
  
 12
 
  10766016
 
  10766153
 
  ENSEMBL   UCSC 
 
  138
 
  4
 
 9053
 
 gene
 
 0.024703
 
 0.0185311
 
 Not queried
 
  0.7153718
 
       NA
 
           NA
  
 
  
 11
 
  13983705
 
  13983893
 
  ENSEMBL   UCSC 
 
  189
 
  7
 
 7160
 
 tss
 
 0.908224
 
 0.9252943
 
 Not queried
 
  0.7152438
 
       NA
 
           NA
  
 
  
 20
 
  45338072
 
  45338122
 
  ENSEMBL   UCSC 
 
   51
 
  4
 
 25797
 
 tss;island
 
 0.035607
 
 0.0429669
 
 Not queried
 
  0.7149879
 
       NA
 
           NA
  
 
  
 12
 
  57985077
 
  57985301
 
  ENSEMBL   UCSC 
 
  225
 
  4
 
 9194
 
 gene
 
 0.012805
 
 0.0072918
 
 Not queried
 
  0.7149588
 
       NA
 
           NA
  
 
  
 2
 
 241512254
 
 241512735
 
  ENSEMBL   UCSC 
 
  482
 
  6
 
 23123
 
 gene;island
 
 0.957851
 
 0.9684568
 
 Not queried
 
  0.7148911
 
       NA
 
           NA
  
 
  
 20
 
  61147572
 
  61147652
 
  ENSEMBL   UCSC 
 
   81
 
  5
 
 25873
 
 tss
 
 0.056373
 
 0.0261660
 
 Not queried
 
  0.7148549
 
       NA
 
           NA
  
 
  
 14
 
  56045827
 
  56046098
 
  ENSEMBL   UCSC 
 
  272
 
  4
 
 12089
 
 gene;tss;island
 
 0.580790
 
 0.7089953
 
 Not queried
 
  0.7148521
 
       NA
 
           NA
  
 
  
 9
 
  72873345
 
  72873728
 
  ENSEMBL   UCSC 
 
  384
 
  4
 
 40815
 
 tss;island
 
 0.002949
 
 0.0040542
 
 Not queried
 
  0.7147462
 
       NA
 
           NA
  
 
  
 4
 
  74904498
 
  74904790
 
  ENSEMBL   UCSC 
 
  293
 
  8
 
 30433
 
 tss
 
 0.009279
 
 0.0182265
 
 Not queried
 
  0.7146951
 
       NA
 
           NA
  
 
  
 2
 
 118771821
 
 118771984
 
  ENSEMBL   UCSC 
 
  164
 
  6
 
 23588
 
 tss
 
 0.002414
 
 0.0052586
 
 Not queried
 
  0.7146934
 
       NA
 
           NA
  
 
  
 3
 
   8810980
 
   8811279
 
  ENSEMBL   UCSC 
 
  300
 
  4
 
 27647
 
 gene
 
 0.023934
 
 0.0183859
 
 Not queried
 
  0.7146311
 
       NA
 
           NA
  
 
  
 8
 
  38965122
 
  38965492
 
  ENSEMBL   UCSC 
 
  371
 
  4
 
 39286
 
 gene
 
 0.271561
 
 0.1070218
 
 Not queried
 
  0.7145937
 
       NA
 
           NA
  
 
  
 3
 
 184870891
 
 184871070
 
  ENSEMBL   UCSC 
 
  180
 
  6
 
 28765
 
 tss
 
 0.006048
 
 0.0111767
 
 Not queried
 
  0.7145614
 
       NA
 
           NA
  
 
  
 16
 
  50099438
 
  50099896
 
  ENSEMBL   UCSC 
 
  459
 
  5
 
 15911
 
 island
 
 0.032708
 
 0.0200200
 
 Not queried
 
  0.7145129
 
       NA
 
           NA
  
 
  
 1
 
  78148262
 
  78148604
 
  ENSEMBL   UCSC 
 
  343
 
  8
 
 3397
 
 island
 
 0.002340
 
 0.0049975
 
 Not queried
 
  0.7144590
 
       NA
 
           NA
  
 
  
 8
 
  67874178
 
  67874366
 
  ENSEMBL   UCSC 
 
  189
 
  4
 
 40362
 
 island
 
 0.016114
 
 0.0088767
 
 Not queried
 
  0.7144285
 
       NA
 
           NA
  
 
  
 13
 
  31506685
 
  31506773
 
  ENSEMBL   UCSC 
 
   89
 
  5
 
 11377
 
 tss
 
 0.386641
 
 0.4517308
 
 Not queried
 
  0.7144081
 
       NA
 
           NA
  
 
  
 4
 
   7056300
 
   7056774
 
  ENSEMBL   UCSC 
 
  475
 
  4
 
 29934
 
 gene;island
 
 0.981275
 
 0.9844169
 
 Not queried
 
  0.7142103
 
       NA
 
           NA
  
 
  
 11
 
  49914985
 
  49915371
 
  ENSEMBL   UCSC 
 
  387
 
  4
 
 8277
 
 island
 
 0.763073
 
 0.8405708
 
 Not queried
 
  0.7140713
 
       NA
 
           NA
  
 
  
 17
 
   4613992
 
   4614422
 
  ENSEMBL   UCSC 
 
  431
 
  5
 
 16356
 
 gene;island
 
 0.003080
 
 0.0051877
 
 Not queried
 
  0.7138793
 
       NA
 
           NA
  
 
  
 5
 
   3590820
 
   3591171
 
  ENSEMBL   UCSC 
 
  352
 
  4
 
 32665
 
 island
 
 0.014613
 
 0.0239177
 
 Not queried
 
  0.7138014
 
       NA
 
           NA
  
 
  
 10
 
  63422511
 
  63422841
 
  ENSEMBL   UCSC 
 
  331
 
  8
 
 5769
 
 island
 
 0.343303
 
 0.0487429
 
 Not queried
 
  0.7136376
 
       NA
 
           NA
  
 
  
 11
 
 131239234
 
 131239703
 
  ENSEMBL   UCSC 
 
  470
 
  6
 
 7822
 
 tss
 
 0.380681
 
 0.6350072
 
 Not queried
 
  0.7136300
 
       NA
 
           NA
  
 
  
 11
 
  61891354
 
  61891497
 
  ENSEMBL   UCSC 
 
  144
 
  4
 
 8338
 
 island
 
 0.009397
 
 0.0129443
 
 Not queried
 
  0.7136003
 
       NA
 
           NA
  
 
  
 22
 
  41843111
 
  41843233
 
  ENSEMBL   UCSC 
 
  123
 
  6
 
 27172
 
 tss;island
 
 0.005419
 
 0.0032766
 
 Not queried
 
  0.7135709
 
       NA
 
           NA
  
 
  
 6
 
 127587654
 
 127588115
 
  ENSEMBL   UCSC 
 
  462
 
  8
 
 36469
 
 island
 
 0.006332
 
 0.0079615
 
 Not queried
 
  0.7135428
 
       NA
 
           NA
  
 
  
 10
 
  98945775
 
  98946238
 
  ENSEMBL   UCSC 
 
  464
 
 10
 
 5257
 
 tss
 
 0.004676
 
 0.0046225
 
 Not queried
 
  0.7134827
 
       NA
 
           NA
  
 
  
 1
 
 179560700
 
 179560983
 
  ENSEMBL   UCSC 
 
  284
 
  5
 
 2119
 
 tss
 
 0.061194
 
 0.1128228
 
 Not queried
 
  0.7134063
 
       NA
 
           NA
  
 
  
 1
 
 110765666
 
 110765955
 
  ENSEMBL   UCSC 
 
  290
 
  4
 
 676
 
 gene;island
 
 0.911397
 
 0.8022494
 
 Not queried
 
  0.7133922
 
       NA
 
           NA
  
 
  
 7
 
  39605536
 
  39605996
 
  ENSEMBL   UCSC 
 
  461
 
  8
 
 37545
 
 tss
 
 0.001822
 
 0.0049995
 
 Not queried
 
  0.7133547
 
       NA
 
           NA
  
 
  
 17
 
  19617084
 
  19617395
 
  ENSEMBL   UCSC 
 
  312
 
  4
 
 16482
 
 gene;island
 
 0.727691
 
 0.8272875
 
 Not queried
 
  0.7132875
 
       NA
 
           NA
  
 
  
 18
 
  21242319
 
  21242479
 
  ENSEMBL   UCSC 
 
  161
 
  4
 
 19074
 
 gene;island
 
 0.015149
 
 0.0072789
 
 Not queried
 
  0.7132008
 
       NA
 
           NA
  
 
  
 10
 
  13688030
 
  13688165
 
  ENSEMBL   UCSC 
 
  136
 
  5
 
 4502
 
 gene
 
 0.964595
 
 0.9656958
 
 Not queried
 
  0.7131866
 
       NA
 
           NA
  
 
  
 11
 
  72145126
 
  72145533
 
  ENSEMBL   UCSC 
 
  408
 
  4
 
 6812
 
 gene
 
 0.004213
 
 0.0057305
 
 Not queried
 
  0.7130389
 
       NA
 
           NA
  
 
  
 11
 
   2920052
 
   2920819
 
  ENSEMBL   UCSC 
 
  768
 
 16
 
 6469
 
 gene;tss;island
 
 0.355367
 
 0.4710645
 
 Not queried
 
  0.7126555
 
       NA
 
           NA
  
 
  
 17
 
  71161157
 
  71161502
 
  ENSEMBL   UCSC 
 
  346
 
  7
 
 18699
 
 island
 
 0.008071
 
 0.0226985
 
 Not queried
 
  0.7126332
 
       NA
 
           NA
  
 
  
 4
 
  81256889
 
  81257269
 
  ENSEMBL   UCSC 
 
  381
 
  5
 
 30036
 
 gene
 
 0.015567
 
 0.0122863
 
 Not queried
 
  0.7124929
 
       NA
 
           NA
  
 
  
 7
 
   1641705
 
   1641922
 
  ENSEMBL   UCSC 
 
  218
 
  4
 
 38120
 
 island
 
 0.477381
 
 0.5994157
 
 Not queried
 
  0.7124592
 
       NA
 
           NA
  
 
  
 18
 
  47340211
 
  47340501
 
  ENSEMBL   UCSC 
 
  291
 
  5
 
 19111
 
 gene;tss;island
 
 0.002957
 
 0.0052041
 
 Not queried
 
  0.7123204
 
       NA
 
           NA
  
 
  
 6
 
  88411006
 
  88411075
 
  ENSEMBL   UCSC 
 
   70
 
  4
 
 34412
 
 gene;tss;island
 
 0.015081
 
 0.0094959
 
 Not queried
 
  0.7119895
 
       NA
 
           NA
  
 
  
 9
 
 140115806
 
 140116137
 
  ENSEMBL   UCSC 
 
  332
 
  4
 
 40858
 
 tss;island
 
 0.481563
 
 0.5718950
 
 Not queried
 
  0.7119751
 
       NA
 
           NA
  
 
  
 11
 
  17740887
 
  17741070
 
  ENSEMBL   UCSC 
 
  184
 
  4
 
 7178
 
 tss
 
 0.211100
 
 0.1622905
 
 Not queried
 
  0.7118531
 
       NA
 
           NA
  
 
  
 6
 
  32169650
 
  32169934
 
  ENSEMBL   UCSC 
 
  285
 
  7
 
 34087
 
 gene
 
 0.249448
 
 0.4779301
 
 Not queried
 
  0.7117936
 
       NA
 
           NA
  
 
  
 14
 
 100772763
 
 100773152
 
  ENSEMBL   UCSC 
 
  390
 
  6
 
 12603
 
 tss;island
 
 0.004726
 
 0.0040376
 
 Not queried
 
  0.7117553
 
       NA
 
           NA
  
 
  
 17
 
  72983622
 
  72983826
 
  ENSEMBL   UCSC 
 
  205
 
  5
 
 18734
 
 island
 
 0.008387
 
 0.0051721
 
 Not queried
 
  0.7117058
 
       NA
 
           NA
  
 
  
 17
 
  43238612
 
  43238884
 
  ENSEMBL   UCSC 
 
  273
 
  4
 
 16631
 
 gene;island
 
 0.005191
 
 0.0073743
 
 Not queried
 
  0.7117009
 
       NA
 
           NA
  
 
  
 7
 
  57247871
 
  57248018
 
  ENSEMBL   UCSC 
 
  148
 
  7
 
 37612
 
 tss
 
 0.509723
 
 0.6164082
 
 Not queried
 
  0.7115999
 
       NA
 
           NA
  
 
  
 11
 
  63606115
 
  63606512
 
  ENSEMBL   UCSC 
 
  398
 
  7
 
 8374
 
 island
 
 0.003329
 
 0.0055153
 
 Not queried
 
  0.7114556
 
       NA
 
           NA
  
 
  
 8
 
 144635260
 
 144635610
 
  ENSEMBL   UCSC 
 
  351
 
 10
 
 40668
 
 island
 
 0.012545
 
 0.0072889
 
 Not queried
 
  0.7114458
 
       NA
 
           NA
  
 
  
 19
 
  19303269
 
  19303735
 
  ENSEMBL   UCSC 
 
  467
 
  7
 
 19948
 
 gene;island
 
 0.013584
 
 0.0072928
 
 Not queried
 
  0.7110983
 
       NA
 
           NA
  
 
  
 6
 
  27841633
 
  27841865
 
  ENSEMBL   UCSC 
 
  233
 
  4
 
 34729
 
 tss;island
 
 0.008692
 
 0.0176088
 
 Not queried
 
  0.7110625
 
       NA
 
           NA
  
 
  
 6
 
  31927895
 
  31928257
 
  ENSEMBL   UCSC 
 
  363
 
  5
 
 34855
 
 tss
 
 0.930367
 
 0.9372185
 
 Not queried
 
  0.7105966
 
       NA
 
           NA
  
 
  
 10
 
 112679006
 
 112679113
 
  ENSEMBL   UCSC 
 
  108
 
  4
 
 4759
 
 gene
 
 0.009936
 
 0.0041999
 
 Not queried
 
  0.7105204
 
       NA
 
           NA
  
 
  
 1
 
   1713944
 
   1714271
 
  ENSEMBL   UCSC 
 
  328
 
  5
 
 2462
 
 island
 
 0.064097
 
 0.1129995
 
 Not queried
 
  0.7104813
 
       NA
 
           NA
  
 
  
 17
 
   8113904
 
   8114032
 
  ENSEMBL   UCSC 
 
  129
 
  5
 
 17113
 
 tss
 
 0.004713
 
 0.0047025
 
 Not queried
 
  0.7102466
 
       NA
 
           NA
  
 
  
 19
 
   3971119
 
   3971417
 
  ENSEMBL   UCSC 
 
  299
 
  5
 
 21357
 
 island
 
 0.005339
 
 0.0116069
 
 Not queried
 
  0.7101447
 
       NA
 
           NA
  
 
  
 4
 
  73434487
 
  73434856
 
  ENSEMBL   UCSC 
 
  370
 
  5
 
 31092
 
 island
 
 0.009387
 
 0.0097487
 
 Not queried
 
  0.7101318
 
       NA
 
           NA
  
 
  
 10
 
  17685563
 
  17686119
 
  ENSEMBL   UCSC 
 
  557
 
  9
 
 4971
 
 tss
 
 0.005132
 
 0.0022555
 
 Not queried
 
  0.7101161
 
       NA
 
           NA
  
 
  
 4
 
  83295282
 
  83295361
 
  ENSEMBL   UCSC 
 
   80
 
  4
 
 30465
 
 tss;island
 
 0.007369
 
 0.0107606
 
 Not queried
 
  0.7100861
 
       NA
 
           NA
  
 
  
 11
 
 120008025
 
 120008607
 
  ENSEMBL   UCSC 
 
  583
 
  6
 
 6943
 
 gene;island
 
 0.575673
 
 0.6303969
 
 Not queried
 
  0.7100716
 
       NA
 
           NA
  
 
  
 2
 
  10567526
 
  10567843
 
  ENSEMBL   UCSC 
 
  318
 
  4
 
 24068
 
 island
 
 0.975934
 
 0.9684351
 
 Not queried
 
  0.7100387
 
       NA
 
           NA
  
 
  
 21
 
  34638868
 
  34639113
 
  ENSEMBL   UCSC 
 
  246
 
  4
 
 26404
 
 gene;island
 
 0.004344
 
 0.0050727
 
 Not queried
 
  0.7099018
 
       NA
 
           NA
  
 
  
 10
 
 126849609
 
 126849780
 
  ENSEMBL   UCSC 
 
  172
 
  6
 
 5427
 
 tss;island
 
 0.053985
 
 0.0322844
 
 Not queried
 
  0.7098331
 
       NA
 
           NA
  
 
  
 19
 
   2611105
 
   2611456
 
  ENSEMBL   UCSC 
 
  352
 
  4
 
 19697
 
 gene;island
 
 0.008567
 
 0.0073482
 
 Not queried
 
  0.7097646
 
       NA
 
           NA
  
 
  
 10
 
 115860606
 
 115860959
 
  ENSEMBL   UCSC 
 
  354
 
  4
 
 6148
 
 island
 
 0.331280
 
 0.0860213
 
 Not queried
 
  0.7096500
 
       NA
 
           NA
  
 
  
 2
 
  24150123
 
  24150353
 
  ENSEMBL   UCSC 
 
  231
 
  4
 
 23218
 
 tss;island
 
 0.025118
 
 0.0244945
 
 Not queried
 
  0.7095803
 
       NA
 
           NA
  
 
  
 8
 
 124780287
 
 124780424
 
  ENSEMBL   UCSC 
 
  138
 
  4
 
 39902
 
 tss;island
 
 0.007785
 
 0.0042823
 
 Not queried
 
  0.7094449
 
       NA
 
           NA
  
 
  
 17
 
  54670875
 
  54671575
 
  ENSEMBL   UCSC 
 
  701
 
  6
 
 18577
 
 island
 
 0.058811
 
 0.0275258
 
 Not queried
 
  0.7094154
 
       NA
 
           NA
  
 
  
 22
 
  17517062
 
  17517418
 
  ENSEMBL   UCSC 
 
  357
 
  5
 
 26939
 
 tss;island
 
 0.293044
 
 0.3547097
 
 Not queried
 
  0.7093724
 
       NA
 
           NA
  
 
  
 4
 
 185942529
 
 185942800
 
  ENSEMBL   UCSC 
 
  272
 
  4
 
 31470
 
 island
 
 0.018037
 
 0.0728130
 
 Not queried
 
  0.7092959
 
       NA
 
           NA
  
 
  
 2
 
  99952550
 
  99953048
 
  ENSEMBL   UCSC 
 
  499
 
 10
 
 23542
 
 tss;island
 
 0.003307
 
 0.0056828
 
 Not queried
 
  0.7092057
 
       NA
 
           NA
  
 
  
 2
 
 236720683
 
 236720980
 
  ENSEMBL   UCSC 
 
  298
 
  5
 
 23082
 
 gene
 
 0.645646
 
 0.8400877
 
 Not queried
 
  0.7091827
 
       NA
 
           NA
  
 
  
 3
 
  44596178
 
  44596684
 
  ENSEMBL   UCSC 
 
  507
 
  8
 
 28256
 
 tss
 
 0.909329
 
 0.3893681
 
 Not queried
 
  0.7089515
 
       NA
 
           NA
  
 
  
 4
 
 104641033
 
 104641319
 
  ENSEMBL   UCSC 
 
  287
 
  6
 
 30527
 
 tss;island
 
 0.102196
 
 0.0554685
 
 Not queried
 
  0.7088385
 
       NA
 
           NA
  
 
  
 11
 
  22851321
 
  22851591
 
  ENSEMBL   UCSC 
 
  271
 
  8
 
 8137
 
 island
 
 0.010441
 
 0.0046349
 
 Not queried
 
  0.7088148
 
       NA
 
           NA
  
 
  
 1
 
  35324574
 
  35324786
 
  ENSEMBL   UCSC 
 
  213
 
  5
 
 416
 
 gene;island
 
 0.010780
 
 0.0053339
 
 Not queried
 
  0.7086153
 
       NA
 
           NA
  
 
  
 6
 
  30910583
 
  30910646
 
  ENSEMBL   UCSC 
 
   64
 
  4
 
 33842
 
 gene
 
 0.104281
 
 0.0620509
 
 Not queried
 
  0.7086076
 
       NA
 
           NA
  
 
  
 11
 
  33680220
 
  33680427
 
  ENSEMBL   UCSC 
 
  208
 
  4
 
 6576
 
 gene;island
 
 0.953963
 
 0.8992734
 
 Not queried
 
  0.7084434
 
       NA
 
           NA
  
 
  
 18
 
  72201716
 
  72201785
 
  ENSEMBL   UCSC 
 
   70
 
  4
 
 19127
 
 gene
 
 0.805745
 
 0.8588695
 
 Not queried
 
  0.7084108
 
       NA
 
           NA
  
 
  
 16
 
  85465111
 
  85465283
 
  ENSEMBL   UCSC 
 
  173
 
  4
 
 16155
 
 island
 
 0.656001
 
 0.8097321
 
 Not queried
 
  0.7083477
 
       NA
 
           NA
  
 
  
 16
 
  48643813
 
  48644020
 
  ENSEMBL   UCSC 
 
  208
 
  4
 
 14744
 
 gene
 
 0.003939
 
 0.0048114
 
 Not queried
 
  0.7082764
 
       NA
 
           NA
  
 
  
 17
 
  40950545
 
  40950901
 
  ENSEMBL   UCSC 
 
  357
 
  7
 
 17375
 
 tss;island
 
 0.005979
 
 0.0036664
 
 Not queried
 
  0.7082120
 
       NA
 
           NA
  
 
  
 6
 
   5003999
 
   5004202
 
  ENSEMBL   UCSC 
 
  204
 
  4
 
 33543
 
 gene
 
 0.014848
 
 0.0141358
 
 Not queried
 
  0.7081629
 
       NA
 
           NA
  
 
  
 17
 
  39680217
 
  39680662
 
  ENSEMBL   UCSC 
 
  446
 
  4
 
 16570
 
 gene;island
 
 0.490090
 
 0.6655473
 
 Not queried
 
  0.7080951
 
       NA
 
           NA
  
 
  
 12
 
  31743857
 
  31744436
 
  ENSEMBL   UCSC 
 
  580
 
  6
 
 10312
 
 island
 
 0.012732
 
 0.0178849
 
 Not queried
 
  0.7080869
 
       NA
 
           NA
  
 
  
 3
 
 112280241
 
 112281021
 
  ENSEMBL   UCSC 
 
  781
 
  7
 
 28502
 
 tss;island
 
 0.012062
 
 0.0092075
 
 Not queried
 
  0.7080631
 
       NA
 
           NA
  
 
  
 17
 
  40439433
 
  40439628
 
  ENSEMBL   UCSC 
 
  196
 
  4
 
 18335
 
 island
 
 0.004109
 
 0.0041790
 
 Not queried
 
  0.7080432
 
       NA
 
           NA
  
 
  
 7
 
 142494148
 
 142495098
 
  ENSEMBL   UCSC 
 
  951
 
 15
 
 38971
 
 island
 
 0.009519
 
 0.0038213
 
 Not queried
 
  0.7079704
 
       NA
 
           NA
  
 
  
 16
 
  30569726
 
  30569978
 
  ENSEMBL   UCSC 
 
  253
 
  8
 
 15187
 
 tss;island
 
 0.008763
 
 0.0114403
 
 Not queried
 
  0.7078567
 
       NA
 
           NA
  
 
  
 5
 
 131563492
 
 131564050
 
  ENSEMBL   UCSC 
 
  559
 
 10
 
 32267
 
 tss;island
 
 0.009716
 
 0.0133165
 
 Not queried
 
  0.7077152
 
       NA
 
           NA
  
 
  
 1
 
  16173721
 
  16173906
 
  ENSEMBL   UCSC 
 
  186
 
  5
 
 253
 
 gene;tss;island
 
 0.005793
 
 0.0050124
 
 Not queried
 
  0.7074850
 
       NA
 
           NA
  
 
  
 10
 
 135090098
 
 135090565
 
  ENSEMBL   UCSC 
 
  468
 
  5
 
 6350
 
 island
 
 0.012612
 
 0.0099945
 
 Not queried
 
  0.7073590
 
       NA
 
           NA
  
 
  
 11
 
   9113152
 
   9113458
 
  ENSEMBL   UCSC 
 
  307
 
  9
 
 7132
 
 tss
 
 0.015942
 
 0.0208446
 
 Not queried
 
  0.7073521
 
       NA
 
           NA
  
 
  
 19
 
  59066570
 
  59066700
 
  ENSEMBL   UCSC 
 
  131
 
  7
 
 21150
 
 tss;island
 
 0.005450
 
 0.0029965
 
 Not queried
 
  0.7071305
 
       NA
 
           NA
  
 
  
 4
 
   1027885
 
   1028067
 
  ENSEMBL   UCSC 
 
  183
 
  4
 
 30760
 
 island
 
 0.775318
 
 0.7715508
 
 Not queried
 
  0.7070444
 
       NA
 
           NA
  
 
  
 19
 
  12948848
 
  12949060
 
  ENSEMBL   UCSC 
 
  213
 
  4
 
 20516
 
 tss;island
 
 0.253787
 
 0.2740877
 
 Not queried
 
  0.7069348
 
       NA
 
           NA
  
 
  
 2
 
 153191524
 
 153191822
 
  ENSEMBL   UCSC 
 
  299
 
  7
 
 24789
 
 island
 
 0.012112
 
 0.0145772
 
 Not queried
 
  0.7068462
 
       NA
 
           NA
  
 
  
 6
 
 129478865
 
 129479024
 
  ENSEMBL   UCSC 
 
  160
 
  4
 
 34472
 
 gene
 
 0.551180
 
 0.6307014
 
 Not queried
 
  0.7066528
 
       NA
 
           NA
  
 
  
 2
 
 223160102
 
 223160569
 
  ENSEMBL   UCSC 
 
  468
 
  5
 
 23045
 
 gene;island
 
 0.092677
 
 0.0788150
 
 Not queried
 
  0.7066156
 
       NA
 
           NA
  
 
  
 7
 
 155249398
 
 155249941
 
  ENSEMBL   UCSC 
 
  544
 
  6
 
 38010
 
 tss;island
 
 0.040530
 
 0.0134680
 
 Not queried
 
  0.7064797
 
       NA
 
           NA
  
 
  
 10
 
 131761150
 
 131761386
 
  ENSEMBL   UCSC 
 
  237
 
  6
 
 4838
 
 gene;island
 
 0.369461
 
 0.3233036
 
 Not queried
 
  0.7062096
 
       NA
 
           NA
  
 
  
 12
 
 108523395
 
 108523597
 
  ENSEMBL   UCSC 
 
  203
 
  6
 
 10767
 
 island
 
 0.027459
 
 0.0180347
 
 Not queried
 
  0.7061055
 
       NA
 
           NA
  
 
  
 5
 
  71404443
 
  71404897
 
  ENSEMBL   UCSC 
 
  455
 
  4
 
 31640
 
 gene;island
 
 0.006075
 
 0.0039818
 
 Not queried
 
  0.7059791
 
       NA
 
           NA
  
 
  
 3
 
  52570476
 
  52570591
 
  ENSEMBL   UCSC 
 
  116
 
  4
 
 28385
 
 tss;island
 
 0.011402
 
 0.0082204
 
 Not queried
 
  0.7059663
 
       NA
 
           NA
  
 
  
 6
 
  41010111
 
  41010375
 
  ENSEMBL   UCSC 
 
  265
 
  5
 
 34963
 
 tss
 
 0.813418
 
 0.8702903
 
 Not queried
 
  0.7059466
 
       NA
 
           NA
  
 
  
 11
 
   2162341
 
   2162930
 
  ENSEMBL   UCSC 
 
  590
 
 17
 
 7058
 
 tss
 
 0.050414
 
 0.0786182
 
 Not queried
 
  0.7059204
 
       NA
 
           NA
  
 
  
 10
 
 104005240
 
 104005256
 
  ENSEMBL   UCSC 
 
   17
 
  4
 
 5320
 
 tss
 
 0.005589
 
 0.0061409
 
 Not queried
 
  0.7054816
 
       NA
 
           NA
  
 
  
 14
 
  37051863
 
  37051949
 
  ENSEMBL   UCSC 
 
   87
 
  4
 
 12359
 
 tss
 
 0.005868
 
 0.0141951
 
 Not queried
 
  0.7053524
 
       NA
 
           NA
  
 
  
 20
 
  30407202
 
  30407388
 
  ENSEMBL   UCSC 
 
  187
 
  4
 
 25382
 
 gene
 
 0.571136
 
 0.6272372
 
 Not queried
 
  0.7052676
 
       NA
 
           NA
  
 
  
 6
 
 117002099
 
 117002360
 
  ENSEMBL   UCSC 
 
  262
 
  8
 
 35192
 
 tss
 
 0.006383
 
 0.0040689
 
 Not queried
 
  0.7049243
 
       NA
 
           NA
  
 
  
 11
 
  60414615
 
  60415111
 
  ENSEMBL   UCSC 
 
  497
 
  5
 
 6621
 
 gene;island
 
 0.865137
 
 0.7988965
 
 Not queried
 
  0.7045981
 
       NA
 
           NA
  
 
  
 12
 
 122356316
 
 122356852
 
  ENSEMBL   UCSC 
 
  537
 
  6
 
 10925
 
 island
 
 0.048011
 
 0.0382542
 
 Not queried
 
  0.7045476
 
       NA
 
           NA
  
 
  
 10
 
  51784543
 
  51784888
 
  ENSEMBL   UCSC 
 
  346
 
  4
 
 5742
 
 island
 
 0.563951
 
 0.4705253
 
 Not queried
 
  0.7045365
 
       NA
 
           NA
  
 
  
 5
 
 146257862
 
 146258785
 
  ENSEMBL   UCSC 
 
  924
 
 16
 
 31852
 
 gene;island
 
 0.010009
 
 0.0117799
 
 Not queried
 
  0.7044810
 
       NA
 
           NA
  
 
  
 5
 
   3326343
 
   3326731
 
  ENSEMBL   UCSC 
 
  389
 
  5
 
 32663
 
 island
 
 0.806874
 
 0.8795317
 
 Not queried
 
  0.7044636
 
       NA
 
           NA
  
 
  
 17
 
  35969361
 
  35969432
 
  ENSEMBL   UCSC 
 
   72
 
  4
 
 16535
 
 gene
 
 0.009530
 
 0.0056572
 
 Not queried
 
  0.7044345
 
       NA
 
           NA
  
 
  
 19
 
  12901966
 
  12902295
 
  ENSEMBL   UCSC 
 
  330
 
  4
 
 20511
 
 tss
 
 0.009014
 
 0.0026983
 
 Not queried
 
  0.7043140
 
       NA
 
           NA
  
 
  
 6
 
  30923241
 
  30924004
 
  ENSEMBL   UCSC 
 
  764
 
 17
 
 35746
 
 island
 
 0.011447
 
 0.0149927
 
 Not queried
 
  0.7042672
 
       NA
 
           NA
  
 
  
 8
 
 144679319
 
 144679350
 
  ENSEMBL   UCSC 
 
   32
 
  4
 
 39485
 
 gene;tss;island
 
 0.010913
 
 0.0064858
 
 Not queried
 
  0.7042045
 
       NA
 
           NA
  
 
  
 5
 
  54603267
 
  54603461
 
  ENSEMBL   UCSC 
 
  195
 
  5
 
 31617
 
 gene
 
 0.005033
 
 0.0020156
 
 Not queried
 
  0.7041647
 
       NA
 
           NA
  
 
  
 1
 
  41157553
 
  41157966
 
  ENSEMBL   UCSC 
 
  414
 
  5
 
 458
 
 gene;island
 
 0.001667
 
 0.0035391
 
 Not queried
 
  0.7041446
 
       NA
 
           NA
  
 
  
 16
 
  89159988
 
  89160058
 
  ENSEMBL   UCSC 
 
   71
 
  5
 
 15419
 
 tss;island
 
 0.019413
 
 0.0193360
 
 Not queried
 
  0.7039834
 
       NA
 
           NA
  
 
  
 10
 
 124767941
 
 124768276
 
  ENSEMBL   UCSC 
 
  336
 
  8
 
 4800
 
 gene;tss;island
 
 0.032715
 
 0.0498111
 
 Not queried
 
  0.7039073
 
       NA
 
           NA
  
 
  
 1
 
 112162349
 
 112162410
 
  ENSEMBL   UCSC 
 
   62
 
  6
 
 3595
 
 island
 
 0.004634
 
 0.0047571
 
 Not queried
 
  0.7038499
 
       NA
 
           NA
  
 
  
 3
 
 150421865
 
 150422143
 
  ENSEMBL   UCSC 
 
  279
 
  5
 
 28662
 
 tss
 
 0.013005
 
 0.0039582
 
 Not queried
 
  0.7038032
 
       NA
 
           NA
  
 
  
 6
 
  31021134
 
  31021422
 
  ENSEMBL   UCSC 
 
  289
 
  4
 
 34797
 
 tss
 
 0.022129
 
 0.0748026
 
 Not queried
 
  0.7037012
 
       NA
 
           NA
  
 
  
 17
 
   1627652
 
   1627862
 
  ENSEMBL   UCSC 
 
  211
 
  7
 
 16320
 
 gene;tss;island
 
 0.040918
 
 0.0355544
 
 Not queried
 
  0.7036237
 
       NA
 
           NA
  
 
  
 9
 
  34457129
 
  34457500
 
  ENSEMBL   UCSC 
 
  372
 
  4
 
 40761
 
 gene;island
 
 0.856154
 
 0.9226175
 
 Not queried
 
  0.7035436
 
       NA
 
           NA
  
 
  
 7
 
 142984834
 
 142985245
 
  ENSEMBL   UCSC 
 
  412
 
  4
 
 37951
 
 tss
 
 0.508930
 
 0.3075561
 
 Not queried
 
  0.7034538
 
       NA
 
           NA
  
 
  
 12
 
 108523395
 
 108523472
 
  ENSEMBL   UCSC 
 
   78
 
  5
 
 9979
 
 tss
 
 0.028556
 
 0.0181369
 
 Not queried
 
  0.7034236
 
       NA
 
           NA
  
 
  
 11
 
  47586666
 
  47586994
 
  ENSEMBL   UCSC 
 
  329
 
  4
 
 8267
 
 island
 
 0.018502
 
 0.0141507
 
 Not queried
 
  0.7034164
 
       NA
 
           NA
  
 
  
 12
 
 133757907
 
 133758301
 
  ENSEMBL   UCSC 
 
  395
 
  8
 
 11092
 
 island
 
 0.022301
 
 0.0226701
 
 Not queried
 
  0.7033380
 
       NA
 
           NA
  
 
  
 11
 
  10472001
 
  10472161
 
  ENSEMBL   UCSC 
 
  161
 
  7
 
 7144
 
 tss;island
 
 0.021982
 
 0.0174262
 
 Not queried
 
  0.7033014
 
       NA
 
           NA
  
 
  
 1
 
 217804338
 
 217804877
 
  ENSEMBL   UCSC 
 
  540
 
  9
 
 2256
 
 tss;island
 
 0.007833
 
 0.0075572
 
 Not queried
 
  0.7031147
 
       NA
 
           NA
  
 
  
 2
 
 219646316
 
 219646831
 
  ENSEMBL   UCSC 
 
  516
 
  5
 
 25094
 
 island
 
 0.036659
 
 0.0298723
 
 Not queried
 
  0.7030644
 
       NA
 
           NA
  
 
  
 19
 
  20012036
 
  20012489
 
  ENSEMBL   UCSC 
 
  454
 
  4
 
 19957
 
 gene;island
 
 0.091227
 
 0.0371923
 
 Not queried
 
  0.7029647
 
       NA
 
           NA
  
 
  
 17
 
  72355995
 
  72356254
 
  ENSEMBL   UCSC 
 
  260
 
  4
 
 16754
 
 gene;island
 
 0.601361
 
 0.4852556
 
 Not queried
 
  0.7028898
 
       NA
 
           NA
  
 
  
 16
 
  50613289
 
  50613700
 
  ENSEMBL   UCSC 
 
  412
 
  4
 
 14746
 
 gene;island
 
 0.913246
 
 0.9274643
 
 Not queried
 
  0.7028502
 
       NA
 
           NA
  
 
  
 7
 
  98870608
 
  98870835
 
  ENSEMBL   UCSC 
 
  228
 
  4
 
 37750
 
 tss
 
 0.694981
 
 0.8079309
 
 Not queried
 
  0.7027982
 
       NA
 
           NA
  
 
  
 7
 
  23338735
 
  23339395
 
  ENSEMBL   UCSC 
 
  661
 
 11
 
 38309
 
 island
 
 0.004639
 
 0.0050520
 
 Not queried
 
  0.7027737
 
       NA
 
           NA
  
 
  
 10
 
 119134981
 
 119135296
 
  ENSEMBL   UCSC 
 
  316
 
  5
 
 5391
 
 tss
 
 0.003744
 
 0.0062681
 
 Not queried
 
  0.7027440
 
       NA
 
           NA
  
 
  
 17
 
  74379779
 
  74379922
 
  ENSEMBL   UCSC 
 
  144
 
  4
 
 17676
 
 tss;island
 
 0.022424
 
 0.0163918
 
 Not queried
 
  0.7026960
 
       NA
 
           NA
  
 
  
 8
 
 142189535
 
 142189806
 
  ENSEMBL   UCSC 
 
  272
 
  6
 
 39448
 
 gene
 
 0.982607
 
 0.9657251
 
 Not queried
 
  0.7026687
 
       NA
 
           NA
  
 
  
 15
 
  96868857
 
  96869132
 
  ENSEMBL   UCSC 
 
  276
 
  7
 
 13917
 
 tss
 
 0.015359
 
 0.0207097
 
 Not queried
 
  0.7024666
 
       NA
 
           NA
  
 
  
 19
 
  35531639
 
  35532097
 
  ENSEMBL   UCSC 
 
  459
 
  5
 
 19979
 
 gene;island
 
 0.025966
 
 0.0799062
 
 Not queried
 
  0.7024637
 
       NA
 
           NA
  
 
  
 10
 
  50747167
 
  50747328
 
  ENSEMBL   UCSC 
 
  162
 
  4
 
 5072
 
 tss
 
 0.003077
 
 0.0033724
 
 Not queried
 
  0.7022799
 
       NA
 
           NA
  
 
  
 10
 
  70883261
 
  70883726
 
  ENSEMBL   UCSC 
 
  466
 
  7
 
 5121
 
 tss;island
 
 0.005066
 
 0.0048622
 
 Not queried
 
  0.7022650
 
       NA
 
           NA
  
 
  
 11
 
    278394
 
    278538
 
  ENSEMBL   UCSC 
 
  145
 
  6
 
 7006
 
 tss;island
 
 0.771450
 
 0.8869313
 
 Not queried
 
  0.7022348
 
       NA
 
           NA
  
 
  
 19
 
   2389655
 
   2389770
 
  ENSEMBL   UCSC 
 
  116
 
  5
 
 20325
 
 tss
 
 0.742503
 
 0.8222747
 
 Not queried
 
  0.7020391
 
       NA
 
           NA
  
 
  
 3
 
  99594893
 
  99595254
 
  ENSEMBL   UCSC 
 
  362
 
  6
 
 27876
 
 gene;island
 
 0.005296
 
 0.0033799
 
 Not queried
 
  0.7020318
 
       NA
 
           NA
  
 
  
 10
 
  73157611
 
  73158072
 
  ENSEMBL   UCSC 
 
  462
 
  5
 
 4621
 
 gene;island
 
 0.009645
 
 0.0241179
 
 Not queried
 
  0.7019329
 
       NA
 
           NA
  
 
  
 7
 
 116139477
 
 116139849
 
  ENSEMBL   UCSC 
 
  373
 
  4
 
 37193
 
 gene
 
 0.021880
 
 0.0201987
 
 Not queried
 
  0.7018200
 
       NA
 
           NA
  
 
  
 14
 
  97685060
 
  97686001
 
  ENSEMBL   UCSC 
 
  942
 
 10
 
 13123
 
 island
 
 0.035204
 
 0.0230127
 
 Not queried
 
  0.7018171
 
       NA
 
           NA
  
 
  
 2
 
  24162256
 
  24162667
 
  ENSEMBL   UCSC 
 
  412
 
  5
 
 23219
 
 tss;island
 
 0.006465
 
 0.0740626
 
 Not queried
 
  0.7017511
 
       NA
 
           NA
  
 
  
 17
 
  73727435
 
  73728061
 
  ENSEMBL   UCSC 
 
  627
 
  6
 
 16778
 
 gene;island
 
 0.899438
 
 0.9383611
 
 Not queried
 
  0.7017367
 
       NA
 
           NA
  
 
  
 13
 
  37572869
 
  37573100
 
  ENSEMBL   UCSC 
 
  232
 
  4
 
 11136
 
 gene;island
 
 0.009490
 
 0.0053084
 
 Not queried
 
  0.7017366
 
       NA
 
           NA
  
 
  
 17
 
  21743878
 
  21744126
 
  ENSEMBL   UCSC 
 
  249
 
  4
 
 18128
 
 island
 
 0.704792
 
 0.6031689
 
 Not queried
 
  0.7016408
 
       NA
 
           NA
  
 
  
 5
 
 140220686
 
 140221106
 
  ENSEMBL   UCSC 
 
  421
 
  6
 
 31799
 
 gene;island
 
 0.761635
 
 0.5932461
 
 Not queried
 
  0.7014467
 
       NA
 
           NA
  
 
  
 5
 
  79551865
 
  79552177
 
  ENSEMBL   UCSC 
 
  313
 
  7
 
 32919
 
 island
 
 0.005026
 
 0.0059957
 
 Not queried
 
  0.7013866
 
       NA
 
           NA
  
 
  
 3
 
 159942832
 
 159943466
 
  ENSEMBL   UCSC 
 
  635
 
  9
 
 29627
 
 island
 
 0.009010
 
 0.0092630
 
 Not queried
 
  0.7013093
 
       NA
 
           NA
  
 
  
 2
 
 176994142
 
 176994764
 
  ENSEMBL   UCSC 
 
  623
 
  9
 
 24905
 
 island
 
 0.162402
 
 0.2091567
 
 Not queried
 
  0.7012579
 
       NA
 
           NA
  
 
  
 20
 
  61150472
 
  61150637
 
  ENSEMBL   UCSC 
 
  166
 
  4
 
 25489
 
 gene;tss;island
 
 0.462407
 
 0.5803736
 
 Not queried
 
  0.7012188
 
       NA
 
           NA
  
 
  
 8
 
 120651190
 
 120651398
 
  ENSEMBL   UCSC 
 
  209
 
  4
 
 39886
 
 tss
 
 0.019335
 
 0.0097127
 
 Not queried
 
  0.7011868
 
       NA
 
           NA
  
 
  
 22
 
  31688300
 
  31688645
 
  ENSEMBL   UCSC 
 
  346
 
  6
 
 27427
 
 island
 
 0.007750
 
 0.0144023
 
 Not queried
 
  0.7011365
 
       NA
 
           NA
  
 
  
 4
 
 140477613
 
 140477727
 
  ENSEMBL   UCSC 
 
  115
 
  5
 
 30602
 
 tss
 
 0.033541
 
 0.0298191
 
 Not queried
 
  0.7010867
 
       NA
 
           NA
  
 
  
 7
 
  33168625
 
  33169033
 
  ENSEMBL   UCSC 
 
  409
 
  6
 
 37532
 
 tss
 
 0.016289
 
 0.0232302
 
 Not queried
 
  0.7010424
 
       NA
 
           NA
  
 
  
 7
 
  55433949
 
  55434429
 
  ENSEMBL   UCSC 
 
  481
 
  4
 
 37019
 
 gene;island
 
 0.009365
 
 0.0102472
 
 Not queried
 
  0.7009880
 
       NA
 
           NA
  
 
  
 12
 
 104359248
 
 104359478
 
  ENSEMBL   UCSC 
 
  231
 
  5
 
 9955
 
 tss
 
 0.004656
 
 0.0017843
 
 Not queried
 
  0.7009450
 
       NA
 
           NA
  
 
  
 2
 
 112811586
 
 112811924
 
  ENSEMBL   UCSC 
 
  339
 
  4
 
 23574
 
 tss;island
 
 0.002458
 
 0.0051661
 
 Not queried
 
  0.7009122
 
       NA
 
           NA
  
 
  
 10
 
 106399877
 
 106400824
 
  ENSEMBL   UCSC 
 
  948
 
 10
 
 5350
 
 tss
 
 0.027659
 
 0.0415997
 
 Not queried
 
  0.7008776
 
       NA
 
           NA
  
 
  
 2
 
  15731646
 
  15731827
 
  ENSEMBL   UCSC 
 
  182
 
  4
 
 24091
 
 island
 
 0.056356
 
 0.0254737
 
 Not queried
 
  0.7008759
 
       NA
 
           NA
  
 
  
 4
 
 164253767
 
 164254230
 
  ENSEMBL   UCSC 
 
  464
 
  7
 
 30660
 
 tss
 
 0.010671
 
 0.0059651
 
 Not queried
 
  0.7007311
 
       NA
 
           NA
  
 
  
 10
 
  94050516
 
  94050907
 
  ENSEMBL   UCSC 
 
  392
 
  6
 
 5226
 
 tss;island
 
 0.011080
 
 0.0074751
 
 Not queried
 
  0.7006874
 
       NA
 
           NA
  
 
  
 3
 
  50378407
 
  50378664
 
  ENSEMBL   UCSC 
 
  258
 
  9
 
 28353
 
 tss
 
 0.006647
 
 0.0066882
 
 Not queried
 
  0.7005767
 
       NA
 
           NA
  
 
  
 3
 
 127872092
 
 127872288
 
  ENSEMBL   UCSC 
 
  197
 
  5
 
 28568
 
 tss
 
 0.004142
 
 0.0084074
 
 Not queried
 
  0.7004362
 
       NA
 
           NA
  
 
  
 11
 
  70557445
 
  70557881
 
  ENSEMBL   UCSC 
 
  437
 
  6
 
 6794
 
 gene
 
 0.554843
 
 0.6620599
 
 Not queried
 
  0.7004036
 
       NA
 
           NA
  
 
  
 4
 
  54243685
 
  54243829
 
  ENSEMBL   UCSC 
 
  145
 
  5
 
 31048
 
 island
 
 0.019210
 
 0.0120603
 
 Not queried
 
  0.7002817
 
       NA
 
           NA
  
 
  
 10
 
  11206772
 
  11206892
 
  ENSEMBL   UCSC 
 
  121
 
  7
 
 4944
 
 tss
 
 0.011624
 
 0.0187782
 
 Not queried
 
  0.7000668
 
       NA
 
           NA
  
 
  
 6
 
   1604013
 
   1604212
 
  ENSEMBL   UCSC 
 
  200
 
  5
 
 35372
 
 island
 
 0.937403
 
 0.8633414
 
 Not queried
 
  0.6999013
 
       NA
 
           NA
  
 
  
 2
 
 230786584
 
 230786830
 
  ENSEMBL   UCSC 
 
  247
 
  5
 
 23934
 
 tss;island
 
 0.003490
 
 0.0053153
 
 Not queried
 
  0.6998739
 
       NA
 
           NA
  
 
  
 5
 
  36241908
 
  36242220
 
  ENSEMBL   UCSC 
 
  313
 
  5
 
 31585
 
 gene
 
 0.021429
 
 0.0134327
 
 Not queried
 
  0.6998703
 
       NA
 
           NA
  
 
  
 6
 
  33128825
 
  33130034
 
  ENSEMBL   UCSC 
 
 1210
 
 27
 
 35961
 
 island
 
 0.038210
 
 0.5118188
 
 Not queried
 
  0.6997635
 
       NA
 
           NA
  
 
  
 11
 
   6502690
 
   6502990
 
  ENSEMBL   UCSC 
 
  301
 
  6
 
 6491
 
 gene
 
 0.001477
 
 0.0025710
 
 Not queried
 
  0.6995951
 
       NA
 
           NA
  
 
  
 15
 
  99409194
 
  99409506
 
  ENSEMBL   UCSC 
 
  313
 
  4
 
 13497
 
 gene
 
 0.457253
 
 0.5678565
 
 Not queried
 
  0.6995519
 
       NA
 
           NA
  
 
  
 5
 
 148206173
 
 148206412
 
  ENSEMBL   UCSC 
 
  240
 
  4
 
 31854
 
 gene
 
 0.075253
 
 0.0609273
 
 Not queried
 
  0.6995162
 
       NA
 
           NA
  
 
  
 2
 
 175351471
 
 175351956
 
  ENSEMBL   UCSC 
 
  486
 
  9
 
 24877
 
 island
 
 0.008859
 
 0.0117658
 
 Not queried
 
  0.6994525
 
       NA
 
           NA
  
 
  
 19
 
  19843456
 
  19844368
 
  ENSEMBL   UCSC 
 
  913
 
 14
 
 21828
 
 island
 
 0.031823
 
 0.0152764
 
 Not queried
 
  0.6993489
 
       NA
 
           NA
  
 
  
 10
 
 129948776
 
 129949058
 
  ENSEMBL   UCSC 
 
  283
 
  5
 
 6252
 
 island
 
 0.015630
 
 0.0566165
 
 Not queried
 
  0.6993483
 
       NA
 
           NA
  
 
  
 18
 
  76160108
 
  76160443
 
  ENSEMBL   UCSC 
 
  336
 
  5
 
 19590
 
 island
 
 0.390289
 
 0.5382758
 
 Not queried
 
  0.6993477
 
       NA
 
           NA
  
 
  
 4
 
  40337262
 
  40337443
 
  ENSEMBL   UCSC 
 
  182
 
  4
 
 30357
 
 tss
 
 0.687319
 
 0.7778469
 
 Not queried
 
  0.6992855
 
       NA
 
           NA
  
 
  
 3
 
 149768455
 
 149768762
 
  ENSEMBL   UCSC 
 
  308
 
  5
 
 29586
 
 island
 
 0.926181
 
 0.9529815
 
 Not queried
 
  0.6992441
 
       NA
 
           NA
  
 
  
 2
 
  87035609
 
  87035758
 
  ENSEMBL   UCSC 
 
  150
 
  7
 
 23496
 
 tss
 
 0.018842
 
 0.0045364
 
 Not queried
 
  0.6991917
 
       NA
 
           NA
  
 
  
 7
 
    814391
 
    814759
 
  ENSEMBL   UCSC 
 
  369
 
  5
 
 36722
 
 gene;island
 
 0.924835
 
 0.9052544
 
 Not queried
 
  0.6991254
 
       NA
 
           NA
  
 
  
 16
 
  58767723
 
  58768565
 
  ENSEMBL   UCSC 
 
  843
 
 12
 
 15983
 
 island
 
 0.013208
 
 0.0093976
 
 Not queried
 
  0.6990894
 
       NA
 
           NA
  
 
  
 1
 
  54304230
 
  54304392
 
  ENSEMBL   UCSC 
 
  163
 
  4
 
 1639
 
 tss
 
 0.002536
 
 0.0032051
 
 Not queried
 
  0.6990652
 
       NA
 
           NA
  
 
  
 19
 
  51017123
 
  51017331
 
  ENSEMBL   UCSC 
 
  209
 
  4
 
 20178
 
 gene;island
 
 0.766600
 
 0.6283333
 
 Not queried
 
  0.6990420
 
       NA
 
           NA
  
 
  
 22
 
  50311792
 
  50312035
 
  ENSEMBL   UCSC 
 
  244
 
  4
 
 26921
 
 gene
 
 0.019545
 
 0.0178605
 
 Not queried
 
  0.6988727
 
       NA
 
           NA
  
 
  
 2
 
 162930666
 
 162931175
 
  ENSEMBL   UCSC 
 
  510
 
  5
 
 24825
 
 island
 
 0.011287
 
 0.0112054
 
 Not queried
 
  0.6987390
 
       NA
 
           NA
  
 
  
 1
 
  71546754
 
  71547110
 
  ENSEMBL   UCSC 
 
  357
 
  6
 
 1694
 
 tss
 
 0.002808
 
 0.0025017
 
 Not queried
 
  0.6986855
 
       NA
 
           NA
  
 
  
 1
 
  71546313
 
  71547110
 
  ENSEMBL   UCSC 
 
  798
 
 10
 
 3383
 
 island
 
 0.003875
 
 0.0056714
 
 Not queried
 
  0.6986855
 
       NA
 
           NA
  
 
  
 10
 
  70939636
 
  70939989
 
  ENSEMBL   UCSC 
 
  354
 
  8
 
 5122
 
 tss
 
 0.003200
 
 0.0022558
 
 Not queried
 
  0.6985995
 
       NA
 
           NA
  
 
  
 1
 
 201438018
 
 201438757
 
  ENSEMBL   UCSC 
 
  740
 
  8
 
 4076
 
 island
 
 0.031949
 
 0.0157424
 
 Not queried
 
  0.6983713
 
       NA
 
           NA
  
 
  
 10
 
  44880542
 
  44880819
 
  ENSEMBL   UCSC 
 
  278
 
  7
 
 5055
 
 tss;island
 
 0.006246
 
 0.0092065
 
 Not queried
 
  0.6982765
 
       NA
 
           NA
  
 
  
 18
 
  49865711
 
  49866548
 
  ENSEMBL   UCSC 
 
  838
 
 18
 
 19518
 
 island
 
 0.062318
 
 0.1118652
 
 Not queried
 
  0.6982573
 
       NA
 
           NA
  
 
  
 1
 
   6051551
 
   6051640
 
  ENSEMBL   UCSC 
 
   90
 
  4
 
 158
 
 gene;island
 
 0.017237
 
 0.0220487
 
 Not queried
 
  0.6980321
 
       NA
 
           NA
  
 
  
 6
 
  36355488
 
  36355825
 
  ENSEMBL   UCSC 
 
  338
 
  6
 
 34936
 
 tss
 
 0.014986
 
 0.0128810
 
 Not queried
 
  0.6980108
 
       NA
 
           NA
  
 
  
 19
 
  41935153
 
  41935467
 
  ENSEMBL   UCSC 
 
  315
 
  4
 
 20811
 
 tss
 
 0.846755
 
 0.7956008
 
 Not queried
 
  0.6979582
 
       NA
 
           NA
  
 
  
 1
 
 200004529
 
 200004757
 
  ENSEMBL   UCSC 
 
  229
 
  5
 
 933
 
 gene;island
 
 0.104025
 
 0.0566218
 
 Not queried
 
  0.6979371
 
       NA
 
           NA
  
 
  
 1
 
   1406606
 
   1407066
 
  ENSEMBL   UCSC 
 
  461
 
  6
 
 1153
 
 tss
 
 0.010374
 
 0.0049554
 
 Not queried
 
  0.6979083
 
       NA
 
           NA
  
 
  
 1
 
   1406606
 
   1407183
 
  ENSEMBL   UCSC 
 
  578
 
  8
 
 2440
 
 island
 
 0.005664
 
 0.0045320
 
 Not queried
 
  0.6979083
 
       NA
 
           NA
  
 
  
 1
 
 204966000
 
 204966512
 
  ENSEMBL   UCSC 
 
  513
 
  7
 
 962
 
 gene
 
 0.536989
 
 0.5738409
 
 Not queried
 
  0.6979047
 
       NA
 
           NA
  
 
  
 5
 
   1882885
 
   1883011
 
  ENSEMBL   UCSC 
 
  127
 
  6
 
 31974
 
 tss
 
 0.048553
 
 0.1452221
 
 Not queried
 
  0.6978590
 
       NA
 
           NA
  
 
  
 4
 
  52917271
 
  52917567
 
  ENSEMBL   UCSC 
 
  297
 
  9
 
 30390
 
 tss
 
 0.008775
 
 0.0078834
 
 Not queried
 
  0.6978194
 
       NA
 
           NA
  
 
  
 2
 
  68546899
 
  68547141
 
  ENSEMBL   UCSC 
 
  243
 
  4
 
 22713
 
 gene
 
 0.077558
 
 0.0991395
 
 Not queried
 
  0.6977281
 
       NA
 
           NA
  
 
  
 2
 
  96971423
 
  96971862
 
  ENSEMBL   UCSC 
 
  440
 
  6
 
 23514
 
 tss;island
 
 0.010711
 
 0.0103920
 
 Not queried
 
  0.6976228
 
       NA
 
           NA
  
 
  
 1
 
 165600170
 
 165600573
 
  ENSEMBL   UCSC 
 
  404
 
  5
 
 3931
 
 island
 
 0.012117
 
 0.0091517
 
 Not queried
 
  0.6976175
 
       NA
 
           NA
  
 
  
 6
 
  39197233
 
  39197537
 
  ENSEMBL   UCSC 
 
  305
 
  8
 
 36107
 
 island
 
 0.011947
 
 0.0223960
 
 Not queried
 
  0.6975756
 
       NA
 
           NA
  
 
  
 13
 
  20437874
 
  20437969
 
  ENSEMBL   UCSC 
 
   96
 
  4
 
 11325
 
 tss
 
 0.008515
 
 0.0079171
 
 Not queried
 
  0.6972741
 
       NA
 
           NA
  
 
  
 1
 
 111217406
 
 111217785
 
  ENSEMBL   UCSC 
 
  380
 
  7
 
 3583
 
 island
 
 0.023822
 
 0.0274124
 
 Not queried
 
  0.6972732
 
       NA
 
           NA
  
 
  
 1
 
  26185992
 
  26186256
 
  ENSEMBL   UCSC 
 
  265
 
  6
 
 1378
 
 tss;island
 
 0.009378
 
 0.0048082
 
 Not queried
 
  0.6972172
 
       NA
 
           NA
  
 
  
 19
 
  16435203
 
  16435660
 
  ENSEMBL   UCSC 
 
  458
 
  5
 
 21717
 
 island
 
 0.039065
 
 0.0269510
 
 Not queried
 
  0.6971803
 
       NA
 
           NA
  
 
  
 8
 
  54507118
 
  54507532
 
  ENSEMBL   UCSC 
 
  415
 
  5
 
 40297
 
 island
 
 0.927678
 
 0.9671345
 
 Not queried
 
  0.6971419
 
       NA
 
           NA
  
 
  
 4
 
  68566961
 
  68567174
 
  ENSEMBL   UCSC 
 
  214
 
  9
 
 30419
 
 tss;island
 
 0.004421
 
 0.0031625
 
 Not queried
 
  0.6971148
 
       NA
 
           NA
  
 
  
 8
 
 102504447
 
 102504666
 
  ENSEMBL   UCSC 
 
  220
 
  7
 
 39850
 
 tss
 
 0.158170
 
 0.1186085
 
 Not queried
 
  0.6971073
 
       NA
 
           NA
  
 
  
 8
 
 102504447
 
 102504859
 
  ENSEMBL   UCSC 
 
  413
 
  9
 
 40506
 
 island
 
 0.205996
 
 0.1186085
 
 Not queried
 
  0.6971073
 
       NA
 
           NA
  
 
  
 6
 
  47444699
 
  47445311
 
  ENSEMBL   UCSC 
 
  613
 
  5
 
 35024
 
 tss;island
 
 0.008082
 
 0.0098309
 
 Not queried
 
  0.6969256
 
       NA
 
           NA
  
 
  
 1
 
 101361235
 
 101361426
 
  ENSEMBL   UCSC 
 
  192
 
  4
 
 1781
 
 tss;island
 
 0.004850
 
 0.0031614
 
 Not queried
 
  0.6968996
 
       NA
 
           NA
  
 
  
 18
 
  75402282
 
  75402530
 
  ENSEMBL   UCSC 
 
  249
 
  4
 
 19588
 
 island
 
 0.461156
 
 0.6055868
 
 Not queried
 
  0.6968865
 
       NA
 
           NA
  
 
  
 6
 
  33094069
 
  33094339
 
  ENSEMBL   UCSC 
 
  271
 
  4
 
 34191
 
 gene
 
 0.400983
 
 0.2874554
 
 Not queried
 
  0.6966949
 
       NA
 
           NA
  
 
  
 8
 
 120845108
 
 120845432
 
  ENSEMBL   UCSC 
 
  325
 
  5
 
 39887
 
 tss
 
 0.006936
 
 0.0109852
 
 Not queried
 
  0.6966636
 
       NA
 
           NA
  
 
  
 1
 
  40105664
 
  40105764
 
  ENSEMBL   UCSC 
 
  101
 
  5
 
 1524
 
 tss;island
 
 0.418371
 
 0.5382024
 
 Not queried
 
  0.6965284
 
       NA
 
           NA
  
 
  
 2
 
 103235258
 
 103235720
 
  ENSEMBL   UCSC 
 
  463
 
  5
 
 23549
 
 tss;island
 
 0.020988
 
 0.0326245
 
 Not queried
 
  0.6964963
 
       NA
 
           NA
  
 
  
 6
 
  27782909
 
  27783226
 
  ENSEMBL   UCSC 
 
  318
 
  4
 
 33620
 
 gene
 
 0.051453
 
 0.0321744
 
 Not queried
 
  0.6962138
 
       NA
 
           NA
  
 
  
 17
 
   1619840
 
   1620044
 
  ENSEMBL   UCSC 
 
  205
 
  4
 
 16319
 
 gene
 
 0.003417
 
 0.0019933
 
 Not queried
 
  0.6961042
 
       NA
 
           NA
  
 
  
 11
 
  85338771
 
  85338971
 
  ENSEMBL   UCSC 
 
  201
 
  4
 
 7602
 
 tss;island
 
 0.005165
 
 0.0091493
 
 Not queried
 
  0.6960063
 
       NA
 
           NA
  
 
  
 3
 
 184290594
 
 184290794
 
  ENSEMBL   UCSC 
 
  201
 
  4
 
 28065
 
 gene;island
 
 0.780445
 
 0.8679708
 
 Not queried
 
  0.6960046
 
       NA
 
           NA
  
 
  
 13
 
 111268673
 
 111268714
 
  ENSEMBL   UCSC 
 
   42
 
  4
 
 11233
 
 gene;island
 
 0.036637
 
 0.0196637
 
 Not queried
 
  0.6959930
 
       NA
 
           NA
  
 
  
 8
 
  49292486
 
  49293005
 
  ENSEMBL   UCSC 
 
  520
 
  5
 
 40281
 
 island
 
 0.057752
 
 0.0261414
 
 Not queried
 
  0.6959695
 
       NA
 
           NA
  
 
  
 5
 
 178322737
 
 178322924
 
  ENSEMBL   UCSC 
 
  188
 
  6
 
 33475
 
 island
 
 0.068961
 
 0.0800451
 
 Not queried
 
  0.6958517
 
       NA
 
           NA
  
 
  
 21
 
  34914246
 
  34914662
 
  ENSEMBL   UCSC 
 
  417
 
  7
 
 26406
 
 gene;tss;island
 
 0.004094
 
 0.0055381
 
 Not queried
 
  0.6958482
 
       NA
 
           NA
  
 
  
 6
 
 146349312
 
 146349638
 
  ENSEMBL   UCSC 
 
  327
 
  4
 
 34499
 
 gene;island
 
 0.085810
 
 0.0363471
 
 Not queried
 
  0.6957962
 
       NA
 
           NA
  
 
  
 2
 
 237086398
 
 237086782
 
  ENSEMBL   UCSC 
 
  385
 
  4
 
 25227
 
 island
 
 0.062779
 
 0.0836583
 
 Not queried
 
  0.6957403
 
       NA
 
           NA
  
 
  
 4
 
 155547985
 
 155548273
 
  ENSEMBL   UCSC 
 
  289
 
  5
 
 31383
 
 island
 
 0.033788
 
 0.0211673
 
 Not queried
 
  0.6957320
 
       NA
 
           NA
  
 
  
 12
 
 101604027
 
 101604258
 
  ENSEMBL   UCSC 
 
  232
 
  4
 
 9943
 
 tss
 
 0.128156
 
 0.1450505
 
 Not queried
 
  0.6956513
 
       NA
 
           NA
  
 
  
 17
 
  16394618
 
  16394899
 
  ENSEMBL   UCSC 
 
  282
 
  4
 
 16460
 
 gene;island
 
 0.040823
 
 0.0597242
 
 Not queried
 
  0.6955543
 
       NA
 
           NA
  
 
  
 13
 
  36045201
 
  36045352
 
  ENSEMBL   UCSC 
 
  152
 
  4
 
 11126
 
 gene;island
 
 0.131281
 
 0.1199975
 
 Not queried
 
  0.6955356
 
       NA
 
           NA
  
 
  
 6
 
 146863357
 
 146863878
 
  ENSEMBL   UCSC 
 
  522
 
  5
 
 35275
 
 tss;island
 
 0.863642
 
 0.9399087
 
 Not queried
 
  0.6955282
 
       NA
 
           NA
  
 
  
 8
 
  72987836
 
  72988022
 
  ENSEMBL   UCSC 
 
  187
 
  7
 
 39765
 
 tss
 
 0.037634
 
 0.0139472
 
 Not queried
 
  0.6954981
 
       NA
 
           NA
  
 
  
 8
 
 144699502
 
 144699837
 
  ENSEMBL   UCSC 
 
  336
 
  4
 
 40677
 
 island
 
 0.008638
 
 0.0067415
 
 Not queried
 
  0.6954426
 
       NA
 
           NA
  
 
  
 15
 
  83680325
 
  83680570
 
  ENSEMBL   UCSC 
 
  246
 
  7
 
 14391
 
 island
 
 0.004775
 
 0.0018635
 
 Not queried
 
  0.6954406
 
       NA
 
           NA
  
 
  
 5
 
  90679194
 
  90679543
 
  ENSEMBL   UCSC 
 
  350
 
  8
 
 32175
 
 tss
 
 0.014489
 
 0.0078652
 
 Not queried
 
  0.6953348
 
       NA
 
           NA
  
 
  
 17
 
  56736571
 
  56736918
 
  ENSEMBL   UCSC 
 
  348
 
  5
 
 16710
 
 gene;island
 
 0.002983
 
 0.0060024
 
 Not queried
 
  0.6953180
 
       NA
 
           NA
  
 
  
 18
 
  76829138
 
  76829335
 
  ENSEMBL   UCSC 
 
  198
 
  4
 
 19333
 
 tss;island
 
 0.005514
 
 0.0051508
 
 Not queried
 
  0.6952848
 
       NA
 
           NA
  
 
  
 22
 
  29949575
 
  29949986
 
  ENSEMBL   UCSC 
 
  412
 
  7
 
 27396
 
 island
 
 0.004372
 
 0.0070971
 
 Not queried
 
  0.6949853
 
       NA
 
           NA
  
 
  
 16
 
  69760564
 
  69760966
 
  ENSEMBL   UCSC 
 
  403
 
  6
 
 15334
 
 tss
 
 0.019973
 
 0.0106390
 
 Not queried
 
  0.6948760
 
       NA
 
           NA
  
 
  
 10
 
  60935910
 
  60937048
 
  ENSEMBL   UCSC 
 
 1139
 
 15
 
 5092
 
 tss;island
 
 0.091614
 
 0.0566858
 
 Not queried
 
  0.6946181
 
       NA
 
           NA
  
 
  
 14
 
 101521409
 
 101521809
 
  ENSEMBL   UCSC 
 
  401
 
 10
 
 12629
 
 tss
 
 0.458640
 
 0.5979800
 
 Not queried
 
  0.6945458
 
       NA
 
           NA
  
 
  
 11
 
   8832111
 
   8832283
 
  ENSEMBL   UCSC 
 
  173
 
  4
 
 6499
 
 gene
 
 0.075481
 
 0.0471726
 
 Not queried
 
  0.6943499
 
       NA
 
           NA
  
 
  
 2
 
  18741691
 
  18742235
 
  ENSEMBL   UCSC 
 
  545
 
  8
 
 24098
 
 island
 
 0.014952
 
 0.0122178
 
 Not queried
 
  0.6942323
 
       NA
 
           NA
  
 
  
 14
 
  68086242
 
  68086576
 
  ENSEMBL   UCSC 
 
  335
 
 10
 
 12474
 
 tss
 
 0.006479
 
 0.0074347
 
 Not queried
 
  0.6941948
 
       NA
 
           NA
  
 
  
 19
 
    836606
 
    836898
 
  ENSEMBL   UCSC 
 
  293
 
  4
 
 21177
 
 island
 
 0.079498
 
 0.0997857
 
 Not queried
 
  0.6940611
 
       NA
 
           NA
  
 
  
 14
 
  88788343
 
  88788672
 
  ENSEMBL   UCSC 
 
  330
 
  6
 
 12165
 
 gene;island
 
 0.250971
 
 0.4665747
 
 Not queried
 
  0.6940229
 
       NA
 
           NA
  
 
  
 12
 
   7066563
 
   7066938
 
  ENSEMBL   UCSC 
 
  376
 
  5
 
 9041
 
 gene
 
 0.486986
 
 0.6055653
 
 Not queried
 
  0.6937711
 
       NA
 
           NA
  
 
  
 19
 
  14247481
 
  14247627
 
  ENSEMBL   UCSC 
 
  147
 
  6
 
 20541
 
 tss
 
 0.005785
 
 0.0085265
 
 Not queried
 
  0.6937636
 
       NA
 
           NA
  
 
  
 12
 
 124086193
 
 124087057
 
  ENSEMBL   UCSC 
 
  865
 
 14
 
 10954
 
 island
 
 0.010199
 
 0.0031897
 
 Not queried
 
  0.6937214
 
       NA
 
           NA
  
 
  
 8
 
 144601734
 
 144601883
 
  ENSEMBL   UCSC 
 
  150
 
  5
 
 39480
 
 gene;island
 
 0.968739
 
 0.9581639
 
 Not queried
 
  0.6934208
 
       NA
 
           NA
  
 
  
 19
 
  50879393
 
  50879636
 
  ENSEMBL   UCSC 
 
  244
 
  8
 
 20975
 
 tss
 
 0.007428
 
 0.0065381
 
 Not queried
 
  0.6933078
 
       NA
 
           NA
  
 
  
 5
 
   2112109
 
   2112561
 
  ENSEMBL   UCSC 
 
  453
 
  7
 
 32652
 
 island
 
 0.779258
 
 0.8950698
 
 Not queried
 
  0.6933044
 
       NA
 
           NA
  
 
  
 19
 
  36024217
 
  36024439
 
  ENSEMBL   UCSC 
 
  223
 
  8
 
 21905
 
 island
 
 0.881528
 
 0.9533342
 
 Not queried
 
  0.6932054
 
       NA
 
           NA
  
 
  
 2
 
  69239676
 
  69240197
 
  ENSEMBL   UCSC 
 
  522
 
  8
 
 23403
 
 tss;island
 
 0.005412
 
 0.0104878
 
 Not queried
 
  0.6932040
 
       NA
 
           NA
  
 
  
 2
 
  75788212
 
  75788552
 
  ENSEMBL   UCSC 
 
  341
 
  8
 
 23469
 
 tss
 
 0.011967
 
 0.0152384
 
 Not queried
 
  0.6931492
 
       NA
 
           NA
  
 
  
 3
 
  49893745
 
  49893964
 
  ENSEMBL   UCSC 
 
  220
 
  4
 
 27791
 
 gene
 
 0.009086
 
 0.0167991
 
 Not queried
 
  0.6931266
 
       NA
 
           NA
  
 
  
 16
 
   1755945
 
   1756201
 
  ENSEMBL   UCSC 
 
  257
 
  4
 
 15002
 
 tss
 
 0.005917
 
 0.0115921
 
 Not queried
 
  0.6930887
 
       NA
 
           NA
  
 
  
 16
 
   1014818
 
   1015103
 
  ENSEMBL   UCSC 
 
  286
 
  4
 
 14556
 
 gene;island
 
 0.962739
 
 0.9673081
 
 Not queried
 
  0.6930484
 
       NA
 
           NA
  
 
  
 5
 
 162931471
 
 162931829
 
  ENSEMBL   UCSC 
 
  359
 
  4
 
 31882
 
 gene;tss;island
 
 0.005519
 
 0.0127981
 
 Not queried
 
  0.6929865
 
       NA
 
           NA
  
 
  
 14
 
  50863043
 
  50863307
 
  ENSEMBL   UCSC 
 
  265
 
  4
 
 12387
 
 tss;island
 
 0.019875
 
 0.0045397
 
 Not queried
 
  0.6929684
 
       NA
 
           NA
  
 
  
 20
 
   9495326
 
   9495596
 
  ENSEMBL   UCSC 
 
  271
 
  5
 
 25355
 
 gene
 
 0.049300
 
 0.0217143
 
 Not queried
 
  0.6927236
 
       NA
 
           NA
  
 
  
 21
 
  42219139
 
  42219299
 
  ENSEMBL   UCSC 
 
  161
 
  6
 
 26554
 
 tss
 
 0.316098
 
 0.5721169
 
 Not queried
 
  0.6926658
 
       NA
 
           NA
  
 
  
 13
 
  25745768
 
  25746644
 
  ENSEMBL   UCSC 
 
  877
 
 13
 
 11610
 
 island
 
 0.103872
 
 0.1671568
 
 Not queried
 
  0.6924903
 
       NA
 
           NA
  
 
  
 16
 
   1451278
 
   1451656
 
  ENSEMBL   UCSC 
 
  379
 
  4
 
 14570
 
 gene;island
 
 0.917612
 
 0.9247032
 
 Not queried
 
  0.6923811
 
       NA
 
           NA
  
 
  
 11
 
  66049756
 
  66050123
 
  ENSEMBL   UCSC 
 
  368
 
  4
 
 6728
 
 gene;island
 
 0.024997
 
 0.0465265
 
 Not queried
 
  0.6923281
 
       NA
 
           NA
  
 
  
 4
 
 186456397
 
 186456682
 
  ENSEMBL   UCSC 
 
  286
 
  4
 
 30211
 
 gene
 
 0.014857
 
 0.0230704
 
 Not queried
 
  0.6922835
 
       NA
 
           NA
  
 
  
 21
 
  35831871
 
  35832364
 
  ENSEMBL   UCSC 
 
  494
 
 10
 
 26410
 
 gene;island
 
 0.585832
 
 0.5831396
 
 Not queried
 
  0.6922529
 
       NA
 
           NA
  
 
  
 18
 
    500817
 
    501234
 
  ENSEMBL   UCSC 
 
  418
 
  5
 
 19150
 
 tss;island
 
 0.014719
 
 0.0421849
 
 Not queried
 
  0.6922288
 
       NA
 
           NA
  
 
  
 12
 
 113229245
 
 113229534
 
  ENSEMBL   UCSC 
 
  290
 
  7
 
 10023
 
 tss
 
 0.065542
 
 0.1094713
 
 Not queried
 
  0.6921891
 
       NA
 
           NA
  
 
  
 19
 
  53935018
 
  53935276
 
  ENSEMBL   UCSC 
 
  259
 
  8
 
 22377
 
 island
 
 0.085861
 
 0.0650860
 
 Not queried
 
  0.6921312
 
       NA
 
           NA
  
 
  
 11
 
  66631259
 
  66631536
 
  ENSEMBL   UCSC 
 
  278
 
  4
 
 6740
 
 gene;island
 
 0.611051
 
 0.7847159
 
 Not queried
 
  0.6919634
 
       NA
 
           NA
  
 
  
 14
 
 101350645
 
 101351197
 
  ENSEMBL   UCSC 
 
  553
 
  8
 
 12614
 
 tss;island
 
 0.532879
 
 0.6482077
 
 Not queried
 
  0.6916023
 
       NA
 
           NA
  
 
  
 4
 
    979022
 
    979389
 
  ENSEMBL   UCSC 
 
  368
 
  6
 
 29851
 
 gene;island
 
 0.530604
 
 0.4798317
 
 Not queried
 
  0.6914537
 
       NA
 
           NA
  
 
  
 8
 
 103251433
 
 103251664
 
  ENSEMBL   UCSC 
 
  232
 
  5
 
 39851
 
 tss;island
 
 0.015259
 
 0.0039591
 
 Not queried
 
  0.6914354
 
       NA
 
           NA
  
 
  
 16
 
   3073707
 
   3073960
 
  ENSEMBL   UCSC 
 
  254
 
  5
 
 14620
 
 gene;tss;island
 
 0.009140
 
 0.0059921
 
 Not queried
 
  0.6912416
 
       NA
 
           NA
  
 
  
 4
 
   3685309
 
   3685875
 
  ENSEMBL   UCSC 
 
  567
 
  6
 
 30851
 
 island
 
 0.311661
 
 0.4440630
 
 Not queried
 
  0.6912337
 
       NA
 
           NA
  
 
  
 11
 
 129912344
 
 129912579
 
  ENSEMBL   UCSC 
 
  236
 
  4
 
 8953
 
 island
 
 0.777499
 
 0.6909339
 
 Not queried
 
  0.6911712
 
       NA
 
           NA
  
 
  
 17
 
  42148188
 
  42148592
 
  ENSEMBL   UCSC 
 
  405
 
  4
 
 16605
 
 gene
 
 0.008426
 
 0.0076829
 
 Not queried
 
  0.6911644
 
       NA
 
           NA
  
 
  
 8
 
 145641882
 
 145642243
 
  ENSEMBL   UCSC 
 
  362
 
  7
 
 39521
 
 gene
 
 0.666238
 
 0.7437737
 
 Not queried
 
  0.6910353
 
       NA
 
           NA
  
 
  
 8
 
 145641882
 
 145642456
 
  ENSEMBL   UCSC 
 
  575
 
 11
 
 40728
 
 island
 
 0.666238
 
 0.7437737
 
 Not queried
 
  0.6910353
 
       NA
 
           NA
  
 
  
 10
 
  22624663
 
  22625015
 
  ENSEMBL   UCSC 
 
  353
 
  4
 
 5601
 
 island
 
 0.190798
 
 0.0842176
 
 Not queried
 
  0.6910118
 
       NA
 
           NA
  
 
  
 4
 
 144480206
 
 144480740
 
  ENSEMBL   UCSC 
 
  535
 
 12
 
 31337
 
 island
 
 0.008035
 
 0.0056096
 
 Not queried
 
  0.6908369
 
       NA
 
           NA
  
 
  
 6
 
  32178017
 
  32178298
 
  ENSEMBL   UCSC 
 
  282
 
  5
 
 34090
 
 gene
 
 0.756976
 
 0.8704411
 
 Not queried
 
  0.6906495
 
       NA
 
           NA
  
 
  
 1
 
 244998656
 
 244998909
 
  ENSEMBL   UCSC 
 
  254
 
  6
 
 1114
 
 gene
 
 0.010348
 
 0.0155110
 
 Not queried
 
  0.6905457
 
       NA
 
           NA
  
 
  
 4
 
 156297854
 
 156298427
 
  ENSEMBL   UCSC 
 
  574
 
 12
 
 31390
 
 island
 
 0.021970
 
 0.0223492
 
 Not queried
 
  0.6904047
 
       NA
 
           NA
  
 
  
 10
 
  73497514
 
  73497772
 
  ENSEMBL   UCSC 
 
  259
 
  4
 
 4623
 
 gene
 
 0.403382
 
 0.6052017
 
 Not queried
 
  0.6901178
 
       NA
 
           NA
  
 
  
 7
 
  27213893
 
  27214383
 
  ENSEMBL   UCSC 
 
  491
 
 10
 
 36939
 
 gene;island
 
 0.059909
 
 0.0928701
 
 Not queried
 
  0.6899458
 
       NA
 
           NA
  
 
  
 7
 
  27213966
 
  27214383
 
  ENSEMBL   UCSC 
 
  418
 
  8
 
 37498
 
 tss
 
 0.053364
 
 0.0928701
 
 Not queried
 
  0.6899458
 
       NA
 
           NA
  
 
  
 20
 
  54978525
 
  54978854
 
  ENSEMBL   UCSC 
 
  330
 
  4
 
 25463
 
 gene;island
 
 0.982002
 
 0.9882852
 
 Not queried
 
  0.6897937
 
       NA
 
           NA
  
 
  
 7
 
   4998415
 
   4998714
 
  ENSEMBL   UCSC 
 
  300
 
  4
 
 36865
 
 gene;island
 
 0.076426
 
 0.1280716
 
 Not queried
 
  0.6897851
 
       NA
 
           NA
  
 
  
 1
 
  26146449
 
  26146759
 
  ENSEMBL   UCSC 
 
  311
 
  5
 
 333
 
 gene;tss;island
 
 0.006520
 
 0.0051939
 
 Not queried
 
  0.6896823
 
       NA
 
           NA
  
 
  
 19
 
  48837272
 
  48837598
 
  ENSEMBL   UCSC 
 
  327
 
  4
 
 20122
 
 gene;island
 
 0.017495
 
 0.0116945
 
 Not queried
 
  0.6896011
 
       NA
 
           NA
  
 
  
 1
 
  28573736
 
  28573940
 
  ENSEMBL   UCSC 
 
  205
 
  4
 
 2940
 
 island
 
 0.009732
 
 0.0037621
 
 Not queried
 
  0.6894990
 
       NA
 
           NA
  
 
  
 1
 
 156084271
 
 156084750
 
  ENSEMBL   UCSC 
 
  480
 
  8
 
 3844
 
 island
 
 0.004828
 
 0.0039002
 
 Not queried
 
  0.6894586
 
       NA
 
           NA
  
 
  
 19
 
   6502101
 
   6502304
 
  ENSEMBL   UCSC 
 
  204
 
  4
 
 19765
 
 gene
 
 0.018438
 
 0.0254083
 
 Not queried
 
  0.6893537
 
       NA
 
           NA
  
 
  
 6
 
 132931714
 
 132931926
 
  ENSEMBL   UCSC 
 
  213
 
  4
 
 35228
 
 tss
 
 0.765926
 
 0.6184845
 
 Not queried
 
  0.6891530
 
       NA
 
           NA
  
 
  
 19
 
  52772664
 
  52773035
 
  ENSEMBL   UCSC 
 
  372
 
  6
 
 22353
 
 island
 
 0.009410
 
 0.0063198
 
 Not queried
 
  0.6889797
 
       NA
 
           NA
  
 
  
 17
 
  42083193
 
  42083306
 
  ENSEMBL   UCSC 
 
  114
 
  4
 
 16601
 
 gene;tss;island
 
 0.047558
 
 0.0318743
 
 Not queried
 
  0.6888764
 
       NA
 
           NA
  
 
  
 5
 
 140700449
 
 140700676
 
  ENSEMBL   UCSC 
 
  228
 
  9
 
 32371
 
 tss
 
 0.011388
 
 0.0039008
 
 Not queried
 
  0.6887492
 
       NA
 
           NA
  
 
  
 14
 
  70655686
 
  70656116
 
  ENSEMBL   UCSC 
 
  431
 
  9
 
 12976
 
 island
 
 0.010306
 
 0.0159063
 
 Not queried
 
  0.6887251
 
       NA
 
           NA
  
 
  
 11
 
  57102965
 
  57103334
 
  ENSEMBL   UCSC 
 
  370
 
  4
 
 6611
 
 gene
 
 0.002202
 
 0.0009863
 
 Not queried
 
  0.6886990
 
       NA
 
           NA
  
 
  
 10
 
  99789825
 
  99790205
 
  ENSEMBL   UCSC 
 
  381
 
  4
 
 4703
 
 gene;island
 
 0.069880
 
 0.1125158
 
 Not queried
 
  0.6886504
 
       NA
 
           NA
  
 
  
 1
 
  23003462
 
  23003692
 
  ENSEMBL   UCSC 
 
  231
 
  4
 
 2826
 
 island
 
 0.804248
 
 0.8684646
 
 Not queried
 
  0.6886470
 
       NA
 
           NA
  
 
  
 13
 
 111340159
 
 111340409
 
  ENSEMBL   UCSC 
 
  251
 
  5
 
 11238
 
 gene
 
 0.960539
 
 0.9560129
 
 Not queried
 
  0.6885635
 
       NA
 
           NA
  
 
  
 9
 
 131486520
 
 131486918
 
  ENSEMBL   UCSC 
 
  399
 
  6
 
 40843
 
 tss
 
 0.007692
 
 0.0118234
 
 Not queried
 
  0.6884892
 
       NA
 
           NA
  
 
  
 2
 
  33952423
 
  33952673
 
  ENSEMBL   UCSC 
 
  251
 
  4
 
 22644
 
 gene;island
 
 0.344671
 
 0.5627239
 
 Not queried
 
  0.6882120
 
       NA
 
           NA
  
 
  
 22
 
  46685472
 
  46685728
 
  ENSEMBL   UCSC 
 
  257
 
  4
 
 26909
 
 gene;island
 
 0.951727
 
 0.9306349
 
 Not queried
 
  0.6881868
 
       NA
 
           NA
  
 
  
 15
 
  92937992
 
  92938295
 
  ENSEMBL   UCSC 
 
  304
 
  4
 
 13486
 
 gene;island
 
 0.016446
 
 0.0267141
 
 Not queried
 
  0.6880765
 
       NA
 
           NA
  
 
  
 2
 
 179343408
 
 179343499
 
  ENSEMBL   UCSC 
 
   92
 
  4
 
 23756
 
 tss
 
 0.003805
 
 0.0101387
 
 Not queried
 
  0.6879416
 
       NA
 
           NA
  
 
  
 19
 
  44259148
 
  44259196
 
  ENSEMBL   UCSC 
 
   49
 
  6
 
 20834
 
 tss
 
 0.003803
 
 0.0051832
 
 Not queried
 
  0.6876808
 
       NA
 
           NA
  
 
  
 19
 
  44259116
 
  44259196
 
  ENSEMBL   UCSC 
 
   81
 
  8
 
 22094
 
 island
 
 0.002861
 
 0.0046504
 
 Not queried
 
  0.6876808
 
       NA
 
           NA
  
 
  
 5
 
 131562848
 
 131563099
 
  ENSEMBL   UCSC 
 
  252
 
  4
 
 31745
 
 gene;island
 
 0.061609
 
 0.4866036
 
 Not queried
 
  0.6876766
 
       NA
 
           NA
  
 
  
 6
 
  31379066
 
  31379358
 
  ENSEMBL   UCSC 
 
  293
 
  4
 
 33873
 
 gene
 
 0.953976
 
 0.8237567
 
 Not queried
 
  0.6876230
 
       NA
 
           NA
  
 
  
 18
 
  32556626
 
  32557027
 
  ENSEMBL   UCSC 
 
  402
 
  7
 
 19231
 
 tss;island
 
 0.028626
 
 0.0153035
 
 Not queried
 
  0.6875299
 
       NA
 
           NA
  
 
  
 7
 
 158622328
 
 158622612
 
  ENSEMBL   UCSC 
 
  285
 
  6
 
 38023
 
 tss;island
 
 0.004954
 
 0.0033530
 
 Not queried
 
  0.6874843
 
       NA
 
           NA
  
 
  
 17
 
  62340505
 
  62340798
 
  ENSEMBL   UCSC 
 
  294
 
  6
 
 18658
 
 island
 
 0.008613
 
 0.0121521
 
 Not queried
 
  0.6873447
 
       NA
 
           NA
  
 
  
 2
 
  70351227
 
  70351524
 
  ENSEMBL   UCSC 
 
  298
 
  4
 
 22721
 
 gene;island
 
 0.012685
 
 0.0154116
 
 Not queried
 
  0.6872445
 
       NA
 
           NA
  
 
  
 7
 
 155250210
 
 155250299
 
  ENSEMBL   UCSC 
 
   90
 
  4
 
 38011
 
 tss;island
 
 0.122057
 
 0.1025827
 
 Not queried
 
  0.6872374
 
       NA
 
           NA
  
 
  
 15
 
  78894282
 
  78894483
 
  ENSEMBL   UCSC 
 
  202
 
  4
 
 13439
 
 gene;island
 
 0.968870
 
 0.9806354
 
 Not queried
 
  0.6872001
 
       NA
 
           NA
  
 
  
 6
 
  25882328
 
  25882590
 
  ENSEMBL   UCSC 
 
  263
 
  5
 
 35502
 
 island
 
 0.521347
 
 0.6125387
 
 Not queried
 
  0.6870290
 
       NA
 
           NA
  
 
  
 22
 
  38082108
 
  38082349
 
  ENSEMBL   UCSC 
 
  242
 
  7
 
 27470
 
 island
 
 0.001661
 
 0.0039822
 
 Not queried
 
  0.6869476
 
       NA
 
           NA
  
 
  
 5
 
 125930870
 
 125931362
 
  ENSEMBL   UCSC 
 
  493
 
  8
 
 33060
 
 island
 
 0.120354
 
 0.0722304
 
 Not queried
 
  0.6867893
 
       NA
 
           NA
  
 
  
 8
 
  33369905
 
  33370172
 
  ENSEMBL   UCSC 
 
  268
 
  5
 
 39276
 
 gene;island
 
 0.021176
 
 0.0203465
 
 Not queried
 
  0.6867768
 
       NA
 
           NA
  
 
  
 8
 
  42948412
 
  42948552
 
  ENSEMBL   UCSC 
 
  141
 
  5
 
 39685
 
 tss
 
 0.010684
 
 0.0079644
 
 Not queried
 
  0.6866812
 
       NA
 
           NA
  
 
  
 19
 
  18548845
 
  18549040
 
  ENSEMBL   UCSC 
 
  196
 
  4
 
 19935
 
 gene
 
 0.026085
 
 0.0460803
 
 Not queried
 
  0.6866655
 
       NA
 
           NA
  
 
  
 7
 
 142984834
 
 142985443
 
  ENSEMBL   UCSC 
 
  610
 
  5
 
 38972
 
 island
 
 0.034577
 
 0.0235986
 
 Not queried
 
  0.6862932
 
       NA
 
           NA
  
 
  
 6
 
 100583956
 
 100584395
 
  ENSEMBL   UCSC 
 
  440
 
  6
 
 36362
 
 island
 
 0.733553
 
 0.7885945
 
 Not queried
 
  0.6862568
 
       NA
 
           NA
  
 
  
 1
 
 233086372
 
 233086622
 
  ENSEMBL   UCSC 
 
  251
 
  4
 
 1082
 
 gene
 
 0.008955
 
 0.0049734
 
 Not queried
 
  0.6862501
 
       NA
 
           NA
  
 
  
 3
 
  64253534
 
  64253818
 
  ENSEMBL   UCSC 
 
  285
 
  6
 
 29260
 
 island
 
 0.307462
 
 0.3434033
 
 Not queried
 
  0.6861702
 
       NA
 
           NA
  
 
  
 22
 
  32555255
 
  32555310
 
  ENSEMBL   UCSC 
 
   56
 
  4
 
 27087
 
 tss
 
 0.521585
 
 0.6317295
 
 Not queried
 
  0.6861595
 
       NA
 
           NA
  
 
  
 17
 
  79283062
 
  79283390
 
  ENSEMBL   UCSC 
 
  329
 
  8
 
 17736
 
 tss
 
 0.031735
 
 0.0212006
 
 Not queried
 
  0.6861357
 
       NA
 
           NA
  
 
  
 17
 
  79282893
 
  79283390
 
  ENSEMBL   UCSC 
 
  498
 
 10
 
 18913
 
 island
 
 0.032854
 
 0.0258906
 
 Not queried
 
  0.6861357
 
       NA
 
           NA
  
 
  
 19
 
  39805850
 
  39806102
 
  ENSEMBL   UCSC 
 
  253
 
  4
 
 21991
 
 island
 
 0.713703
 
 0.8077989
 
 Not queried
 
  0.6860752
 
       NA
 
           NA
  
 
  
 3
 
  43731886
 
  43732329
 
  ENSEMBL   UCSC 
 
  444
 
 13
 
 28253
 
 tss;island
 
 0.016774
 
 0.0096410
 
 Not queried
 
  0.6859728
 
       NA
 
           NA
  
 
  
 5
 
 141705351
 
 141705539
 
  ENSEMBL   UCSC 
 
  189
 
  5
 
 32397
 
 tss;island
 
 0.008536
 
 0.0076025
 
 Not queried
 
  0.6858903
 
       NA
 
           NA
  
 
  
 1
 
  67896128
 
  67896194
 
  ENSEMBL   UCSC 
 
   67
 
  5
 
 1684
 
 tss;island
 
 0.020114
 
 0.0126408
 
 Not queried
 
  0.6856456
 
       NA
 
           NA
  
 
  
 4
 
   1166755
 
   1166990
 
  ENSEMBL   UCSC 
 
  236
 
  8
 
 29858
 
 gene;tss;island
 
 0.463272
 
 0.6005116
 
 Not queried
 
  0.6855477
 
       NA
 
           NA
  
 
  
 6
 
   2971950
 
   2972195
 
  ENSEMBL   UCSC 
 
  246
 
  7
 
 35380
 
 island
 
 0.030156
 
 0.0218573
 
 Not queried
 
  0.6853712
 
       NA
 
           NA
  
 
  
 15
 
  26874098
 
  26874515
 
  ENSEMBL   UCSC 
 
  418
 
  4
 
 13261
 
 gene;island
 
 0.790647
 
 0.8702923
 
 Not queried
 
  0.6853557
 
       NA
 
           NA
  
 
  
 1
 
 156570825
 
 156571221
 
  ENSEMBL   UCSC 
 
  397
 
  4
 
 825
 
 gene
 
 0.011709
 
 0.0101391
 
 Not queried
 
  0.6852768
 
       NA
 
           NA
  
 
  
 6
 
  16266038
 
  16266723
 
  ENSEMBL   UCSC 
 
  686
 
  6
 
 33585
 
 gene
 
 0.603529
 
 0.7971482
 
 Not queried
 
  0.6851927
 
       NA
 
           NA
  
 
  
 7
 
  19748663
 
  19748896
 
  ENSEMBL   UCSC 
 
  234
 
  7
 
 37462
 
 tss
 
 0.006632
 
 0.0114709
 
 Not queried
 
  0.6850874
 
       NA
 
           NA
  
 
  
 6
 
  26033394
 
  26034145
 
  ENSEMBL   UCSC 
 
  752
 
 12
 
 34682
 
 tss;island
 
 0.008986
 
 0.0041069
 
 Not queried
 
  0.6848976
 
       NA
 
           NA
  
 
  
 1
 
  51425958
 
  51426199
 
  ENSEMBL   UCSC 
 
  242
 
  7
 
 1617
 
 tss
 
 0.001456
 
 0.0048635
 
 Not queried
 
  0.6848481
 
       NA
 
           NA
  
 
  
 4
 
 175750507
 
 175751001
 
  ENSEMBL   UCSC 
 
  495
 
 10
 
 30686
 
 tss
 
 0.033650
 
 0.0125962
 
 Not queried
 
  0.6848271
 
       NA
 
           NA
  
 
  
 15
 
 100273145
 
 100273611
 
  ENSEMBL   UCSC 
 
  467
 
  4
 
 13503
 
 gene
 
 0.012743
 
 0.0120530
 
 Not queried
 
  0.6847339
 
       NA
 
           NA
  
 
  
 10
 
  97321188
 
  97321482
 
  ENSEMBL   UCSC 
 
  295
 
  7
 
 5246
 
 tss
 
 0.003707
 
 0.0057998
 
 Not queried
 
  0.6846385
 
       NA
 
           NA
  
 
  
 10
 
  97321104
 
  97321482
 
  ENSEMBL   UCSC 
 
  379
 
  9
 
 5965
 
 island
 
 0.003707
 
 0.0057998
 
 Not queried
 
  0.6846385
 
       NA
 
           NA
  
 
  
 5
 
  39074485
 
  39074787
 
  ENSEMBL   UCSC 
 
  303
 
  6
 
 32746
 
 island
 
 0.009061
 
 0.0068620
 
 Not queried
 
  0.6846044
 
       NA
 
           NA
  
 
  
 7
 
 100472263
 
 100472765
 
  ENSEMBL   UCSC 
 
  503
 
  7
 
 38781
 
 island
 
 0.003772
 
 0.0034493
 
 Not queried
 
  0.6844355
 
       NA
 
           NA
  
 
  
 3
 
 157827682
 
 157828290
 
  ENSEMBL   UCSC 
 
  609
 
  8
 
 29619
 
 island
 
 0.010344
 
 0.0061086
 
 Not queried
 
  0.6844088
 
       NA
 
           NA
  
 
  
 11
 
   2891065
 
   2891118
 
  ENSEMBL   UCSC 
 
   54
 
  5
 
 7079
 
 tss;island
 
 0.235368
 
 0.3307123
 
 Not queried
 
  0.6843709
 
       NA
 
           NA
  
 
  
 1
 
  29213106
 
  29213367
 
  ENSEMBL   UCSC 
 
  262
 
  4
 
 1437
 
 tss;island
 
 0.701128
 
 0.3658432
 
 Not queried
 
  0.6843396
 
       NA
 
           NA
  
 
  
 7
 
 150076338
 
 150076646
 
  ENSEMBL   UCSC 
 
  309
 
  5
 
 39010
 
 island
 
 0.003073
 
 0.0049994
 
 Not queried
 
  0.6843069
 
       NA
 
           NA
  
 
  
 19
 
  12886455
 
  12886624
 
  ENSEMBL   UCSC 
 
  170
 
  4
 
 20510
 
 tss
 
 0.006950
 
 0.0042331
 
 Not queried
 
  0.6842094
 
       NA
 
           NA
  
 
  
 3
 
 184279336
 
 184279728
 
  ENSEMBL   UCSC 
 
  393
 
 10
 
 29730
 
 island
 
 0.028841
 
 0.0100937
 
 Not queried
 
  0.6841491
 
       NA
 
           NA
  
 
  
 4
 
  17516413
 
  17516752
 
  ENSEMBL   UCSC 
 
  340
 
  6
 
 30315
 
 tss
 
 0.820246
 
 0.8857820
 
 Not queried
 
  0.6840844
 
       NA
 
           NA
  
 
  
 22
 
  19938378
 
  19938620
 
  ENSEMBL   UCSC 
 
  243
 
  4
 
 26792
 
 gene;tss
 
 0.967230
 
 0.9405477
 
 Not queried
 
  0.6840772
 
       NA
 
           NA
  
 
  
 19
 
  18335359
 
  18335429
 
  ENSEMBL   UCSC 
 
   71
 
  4
 
 20614
 
 tss
 
 0.003759
 
 0.0015526
 
 Not queried
 
  0.6839725
 
       NA
 
           NA
  
 
  
 15
 
  83317281
 
  83317632
 
  ENSEMBL   UCSC 
 
  352
 
  5
 
 13858
 
 tss;island
 
 0.530087
 
 0.6081660
 
 Not queried
 
  0.6838293
 
       NA
 
           NA
  
 
  
 10
 
  90967052
 
  90967233
 
  ENSEMBL   UCSC 
 
  182
 
  7
 
 5920
 
 island
 
 0.014550
 
 0.0092437
 
 Not queried
 
  0.6838190
 
       NA
 
           NA
  
 
  
 3
 
  50654228
 
  50654499
 
  ENSEMBL   UCSC 
 
  272
 
  6
 
 28361
 
 tss;island
 
 0.013243
 
 0.0071055
 
 Not queried
 
  0.6837874
 
       NA
 
           NA
  
 
  
 2
 
 107503547
 
 107503834
 
  ENSEMBL   UCSC 
 
  288
 
  6
 
 23560
 
 tss;island
 
 0.024539
 
 0.0381838
 
 Not queried
 
  0.6836720
 
       NA
 
           NA
  
 
  
 1
 
 155948126
 
 155948318
 
  ENSEMBL   UCSC 
 
  193
 
  4
 
 811
 
 gene;tss;island
 
 0.022063
 
 0.0148870
 
 Not queried
 
  0.6836625
 
       NA
 
           NA
  
 
  
 1
 
 212781767
 
 212782207
 
  ENSEMBL   UCSC 
 
  441
 
  8
 
 999
 
 gene;island
 
 0.022459
 
 0.0112777
 
 Not queried
 
  0.6836481
 
       NA
 
           NA
  
 
  
 1
 
 212781767
 
 212781985
 
  ENSEMBL   UCSC 
 
  219
 
  6
 
 2245
 
 tss
 
 0.022459
 
 0.0112777
 
 Not queried
 
  0.6836481
 
       NA
 
           NA
  
 
  
 15
 
  79043428
 
  79043637
 
  ENSEMBL   UCSC 
 
  210
 
  4
 
 14360
 
 island
 
 0.137858
 
 0.0875188
 
 Not queried
 
  0.6834738
 
       NA
 
           NA
  
 
  
 19
 
   2269397
 
   2269619
 
  ENSEMBL   UCSC 
 
  223
 
  5
 
 21275
 
 island
 
 0.004894
 
 0.0047896
 
 Not queried
 
  0.6833671
 
       NA
 
           NA
  
 
  
 2
 
  98354060
 
  98354292
 
  ENSEMBL   UCSC 
 
  233
 
  4
 
 22797
 
 gene;island
 
 0.796876
 
 0.8301455
 
 Not queried
 
  0.6833441
 
       NA
 
           NA
  
 
  
 17
 
  46969953
 
  46970032
 
  ENSEMBL   UCSC 
 
   80
 
  4
 
 17476
 
 tss
 
 0.006347
 
 0.0046614
 
 Not queried
 
  0.6833393
 
       NA
 
           NA
  
 
  
 1
 
  28844397
 
  28844869
 
  ENSEMBL   UCSC 
 
  473
 
  7
 
 366
 
 gene;island
 
 0.011161
 
 0.0052909
 
 Not queried
 
  0.6833204
 
       NA
 
           NA
  
 
  
 12
 
  93967225
 
  93967711
 
  ENSEMBL   UCSC 
 
  487
 
  4
 
 9245
 
 gene;island
 
 0.035767
 
 0.0237404
 
 Not queried
 
  0.6832299
 
       NA
 
           NA
  
 
  
 4
 
   6643098
 
   6643382
 
  ENSEMBL   UCSC 
 
  285
 
  4
 
 29928
 
 gene;island
 
 0.018128
 
 0.0149088
 
 Not queried
 
  0.6831584
 
       NA
 
           NA
  
 
  
 20
 
  48530309
 
  48530654
 
  ENSEMBL   UCSC 
 
  346
 
  4
 
 25449
 
 gene;tss;island
 
 0.955569
 
 0.9719340
 
 Not queried
 
  0.6829144
 
       NA
 
           NA
  
 
  
 10
 
 102497298
 
 102497666
 
  ENSEMBL   UCSC 
 
  369
 
  5
 
 6032
 
 island
 
 0.009593
 
 0.0153935
 
 Not queried
 
  0.6827101
 
       NA
 
           NA
  
 
  
 6
 
  43197245
 
  43197675
 
  ENSEMBL   UCSC 
 
  431
 
  8
 
 34995
 
 tss
 
 0.021870
 
 0.0153298
 
 Not queried
 
  0.6825915
 
       NA
 
           NA
  
 
  
 4
 
 109093158
 
 109093430
 
  ENSEMBL   UCSC 
 
  273
 
  5
 
 30086
 
 gene;island
 
 0.007165
 
 0.0070095
 
 Not queried
 
  0.6825792
 
       NA
 
           NA
  
 
  
 11
 
   3400491
 
   3400651
 
  ENSEMBL   UCSC 
 
  161
 
  7
 
 7095
 
 tss
 
 0.019938
 
 0.0228072
 
 Not queried
 
  0.6825382
 
       NA
 
           NA
  
 
  
 20
 
   6748710
 
   6748730
 
  ENSEMBL   UCSC 
 
   21
 
  4
 
 25590
 
 tss;island
 
 0.051053
 
 0.0353228
 
 Not queried
 
  0.6823210
 
       NA
 
           NA
  
 
  
 13
 
 111090669
 
 111090898
 
  ENSEMBL   UCSC 
 
  230
 
  4
 
 11230
 
 gene;island
 
 0.514322
 
 0.5270705
 
 Not queried
 
  0.6822489
 
       NA
 
           NA
  
 
  
 6
 
  32136890
 
  32137294
 
  ENSEMBL   UCSC 
 
  405
 
  8
 
 34066
 
 gene;island
 
 0.901683
 
 0.9372174
 
 Not queried
 
  0.6821028
 
       NA
 
           NA
  
 
  
 19
 
  14629212
 
  14629247
 
  ENSEMBL   UCSC 
 
   36
 
  6
 
 20546
 
 tss
 
 0.004372
 
 0.0081793
 
 Not queried
 
  0.6820711
 
       NA
 
           NA
  
 
  
 5
 
 127872767
 
 127873711
 
  ENSEMBL   UCSC 
 
  945
 
  7
 
 31739
 
 gene;island
 
 0.250436
 
 0.0700661
 
 Not queried
 
  0.6820562
 
       NA
 
           NA
  
 
  
 7
 
  99036699
 
  99036841
 
  ENSEMBL   UCSC 
 
  143
 
  4
 
 37127
 
 gene
 
 0.017654
 
 0.0123048
 
 Not queried
 
  0.6818914
 
       NA
 
           NA
  
 
  
 1
 
 224301701
 
 224302585
 
  ENSEMBL   UCSC 
 
  885
 
 11
 
 4240
 
 island
 
 0.007917
 
 0.0060853
 
 Not queried
 
  0.6818679
 
       NA
 
           NA
  
 
  
 1
 
 214170114
 
 214170376
 
  ENSEMBL   UCSC 
 
  263
 
  5
 
 1005
 
 gene
 
 0.889534
 
 0.9262858
 
 Not queried
 
  0.6816903
 
       NA
 
           NA
  
 
  
 14
 
  77278949
 
  77279508
 
  ENSEMBL   UCSC 
 
  560
 
  7
 
 13026
 
 island
 
 0.015921
 
 0.0241243
 
 Not queried
 
  0.6814979
 
       NA
 
           NA
  
 
  
 22
 
  28035064
 
  28035309
 
  ENSEMBL   UCSC 
 
  246
 
  4
 
 27381
 
 island
 
 0.414485
 
 0.6794738
 
 Not queried
 
  0.6814945
 
       NA
 
           NA
  
 
  
 6
 
 109416235
 
 109416245
 
  ENSEMBL   UCSC 
 
   11
 
  4
 
 35162
 
 tss;island
 
 0.003116
 
 0.0052570
 
 Not queried
 
  0.6814869
 
       NA
 
           NA
  
 
  
 16
 
  72821353
 
  72821665
 
  ENSEMBL   UCSC 
 
  313
 
  4
 
 14836
 
 gene;island
 
 0.007021
 
 0.0082895
 
 Not queried
 
  0.6814233
 
       NA
 
           NA
  
 
  
 6
 
  29571363
 
  29571483
 
  ENSEMBL   UCSC 
 
  121
 
  7
 
 33664
 
 gene
 
 0.704798
 
 0.8199455
 
 Not queried
 
  0.6814216
 
       NA
 
           NA
  
 
  
 1
 
 230272306
 
 230272656
 
  ENSEMBL   UCSC 
 
  351
 
  5
 
 1069
 
 gene
 
 0.974009
 
 0.9814865
 
 Not queried
 
  0.6814039
 
       NA
 
           NA
  
 
  
 19
 
  44506804
 
  44507157
 
  ENSEMBL   UCSC 
 
  354
 
 11
 
 22099
 
 island
 
 0.003059
 
 0.0017266
 
 Not queried
 
  0.6812227
 
       NA
 
           NA
  
 
  
 17
 
  62493555
 
  62493832
 
  ENSEMBL   UCSC 
 
  278
 
  4
 
 17583
 
 tss;island
 
 0.035599
 
 0.0168847
 
 Not queried
 
  0.6811817
 
       NA
 
           NA
  
 
  
 6
 
 152128258
 
 152129036
 
  ENSEMBL   UCSC 
 
  779
 
 20
 
 34514
 
 gene;island
 
 0.051507
 
 0.0294917
 
 Not queried
 
  0.6811402
 
       NA
 
           NA
  
 
  
 19
 
  35981371
 
  35981677
 
  ENSEMBL   UCSC 
 
  307
 
  5
 
 20704
 
 tss
 
 0.320786
 
 0.4233140
 
 Not queried
 
  0.6810403
 
       NA
 
           NA
  
 
  
 5
 
 176810904
 
 176811305
 
  ENSEMBL   UCSC 
 
  402
 
  6
 
 32526
 
 tss
 
 0.385030
 
 0.6900939
 
 Not queried
 
  0.6810266
 
       NA
 
           NA
  
 
  
 20
 
  48099146
 
  48099479
 
  ENSEMBL   UCSC 
 
  334
 
  8
 
 26234
 
 island
 
 0.022553
 
 0.0429848
 
 Not queried
 
  0.6809343
 
       NA
 
           NA
  
 
  
 5
 
 137610127
 
 137610645
 
  ENSEMBL   UCSC 
 
  519
 
  9
 
 33154
 
 island
 
 0.003897
 
 0.0067516
 
 Not queried
 
  0.6807699
 
       NA
 
           NA
  
 
  
 6
 
  28550343
 
  28550437
 
  ENSEMBL   UCSC 
 
   95
 
  5
 
 33638
 
 gene
 
 0.973364
 
 0.9710237
 
 Not queried
 
  0.6806932
 
       NA
 
           NA
  
 
  
 2
 
 219920195
 
 219920517
 
  ENSEMBL   UCSC 
 
  323
 
  4
 
 23024
 
 gene;island
 
 0.648048
 
 0.7539272
 
 Not queried
 
  0.6805645
 
       NA
 
           NA
  
 
  
 11
 
   3818695
 
   3818872
 
  ENSEMBL   UCSC 
 
  178
 
  6
 
 6481
 
 gene
 
 0.002216
 
 0.0067266
 
 Not queried
 
  0.6805032
 
       NA
 
           NA
  
 
  
 4
 
  87515467
 
  87515700
 
  ENSEMBL   UCSC 
 
  234
 
  4
 
 30050
 
 gene
 
 0.019568
 
 0.0248611
 
 Not queried
 
  0.6804794
 
       NA
 
           NA
  
 
  
 7
 
  99774935
 
  99775207
 
  ENSEMBL   UCSC 
 
  273
 
  7
 
 37775
 
 tss;island
 
 0.013263
 
 0.0132607
 
 Not queried
 
  0.6804550
 
       NA
 
           NA
  
 
  
 19
 
  13044304
 
  13045039
 
  ENSEMBL   UCSC 
 
  736
 
 12
 
 21636
 
 island
 
 0.011603
 
 0.0148949
 
 Not queried
 
  0.6803831
 
       NA
 
           NA
  
 
  
 12
 
 131323926
 
 131324319
 
  ENSEMBL   UCSC 
 
  394
 
  6
 
 10120
 
 tss;island
 
 0.030666
 
 0.0363498
 
 Not queried
 
  0.6803555
 
       NA
 
           NA
  
 
  
 16
 
  30406122
 
  30406533
 
  ENSEMBL   UCSC 
 
  412
 
  5
 
 15183
 
 tss;island
 
 0.040275
 
 0.0414070
 
 Not queried
 
  0.6802907
 
       NA
 
           NA
  
 
  
 15
 
  78370102
 
  78370247
 
  ENSEMBL   UCSC 
 
  146
 
  6
 
 13827
 
 tss;island
 
 0.007242
 
 0.0046911
 
 Not queried
 
  0.6802146
 
       NA
 
           NA
  
 
  
 7
 
 134832770
 
 134833299
 
  ENSEMBL   UCSC 
 
  530
 
  7
 
 37235
 
 gene
 
 0.004812
 
 0.0024336
 
 Not queried
 
  0.6800881
 
       NA
 
           NA
  
 
  
 5
 
 115152326
 
 115152494
 
  ENSEMBL   UCSC 
 
  169
 
  8
 
 33027
 
 island
 
 0.026111
 
 0.0144582
 
 Not queried
 
  0.6800225
 
       NA
 
           NA
  
 
  
 7
 
  70597458
 
  70597921
 
  ENSEMBL   UCSC 
 
  464
 
  7
 
 38546
 
 island
 
 0.207966
 
 0.1645144
 
 Not queried
 
  0.6799571
 
       NA
 
           NA
  
 
  
 7
 
 155165295
 
 155165891
 
  ENSEMBL   UCSC 
 
  597
 
  5
 
 39062
 
 island
 
 0.051508
 
 0.0716578
 
 Not queried
 
  0.6798926
 
       NA
 
           NA
  
 
  
 3
 
  49711033
 
  49711660
 
  ENSEMBL   UCSC 
 
  628
 
 13
 
 29123
 
 island
 
 0.003915
 
 0.0075366
 
 Not queried
 
  0.6798653
 
       NA
 
           NA
  
 
  
 7
 
  97600926
 
  97601190
 
  ENSEMBL   UCSC 
 
  265
 
  4
 
 37109
 
 gene;island
 
 0.022710
 
 0.0124567
 
 Not queried
 
  0.6798306
 
       NA
 
           NA
  
 
  
 16
 
  25122757
 
  25122981
 
  ENSEMBL   UCSC 
 
  225
 
  4
 
 15147
 
 tss;island
 
 0.002566
 
 0.0015300
 
 Not queried
 
  0.6797890
 
       NA
 
           NA
  
 
  
 17
 
  36507744
 
  36508031
 
  ENSEMBL   UCSC 
 
  288
 
  5
 
 18251
 
 island
 
 0.009235
 
 0.0039239
 
 Not queried
 
  0.6797743
 
       NA
 
           NA
  
 
  
 18
 
  49865711
 
  49866531
 
  ENSEMBL   UCSC 
 
  821
 
 17
 
 19281
 
 tss
 
 0.063138
 
 0.1105057
 
 Not queried
 
  0.6796888
 
       NA
 
           NA
  
 
  
 12
 
  74931289
 
  74931535
 
  ENSEMBL   UCSC 
 
  247
 
  6
 
 9878
 
 tss
 
 0.009989
 
 0.0041726
 
 Not queried
 
  0.6793725
 
       NA
 
           NA
  
 
  
 1
 
 114471711
 
 114471974
 
  ENSEMBL   UCSC 
 
  264
 
  9
 
 1847
 
 tss
 
 0.004376
 
 0.0042446
 
 Not queried
 
  0.6793282
 
       NA
 
           NA
  
 
  
 1
 
 114471711
 
 114472148
 
  ENSEMBL   UCSC 
 
  438
 
 11
 
 3619
 
 island
 
 0.007183
 
 0.0062807
 
 Not queried
 
  0.6793282
 
       NA
 
           NA
  
 
  
 10
 
 102973432
 
 102973678
 
  ENSEMBL   UCSC 
 
  247
 
  5
 
 6055
 
 island
 
 0.006996
 
 0.0081114
 
 Not queried
 
  0.6793192
 
       NA
 
           NA
  
 
  
 8
 
   1789169
 
   1789670
 
  ENSEMBL   UCSC 
 
  502
 
  6
 
 39177
 
 gene
 
 0.788738
 
 0.7358299
 
 Not queried
 
  0.6790910
 
       NA
 
           NA
  
 
  
 22
 
  20861680
 
  20861768
 
  ENSEMBL   UCSC 
 
   89
 
  4
 
 26981
 
 tss
 
 0.006672
 
 0.0108023
 
 Not queried
 
  0.6789707
 
       NA
 
           NA
  
 
  
 10
 
  98273714
 
  98273924
 
  ENSEMBL   UCSC 
 
  211
 
  5
 
 5254
 
 tss
 
 0.043287
 
 0.0173227
 
 Not queried
 
  0.6788813
 
       NA
 
           NA
  
 
  
 11
 
  85566190
 
  85566560
 
  ENSEMBL   UCSC 
 
  371
 
  4
 
 6853
 
 gene;island
 
 0.037634
 
 0.2641201
 
 Not queried
 
  0.6787660
 
       NA
 
           NA
  
 
  
 3
 
 101568173
 
 101568246
 
  ENSEMBL   UCSC 
 
   74
 
  6
 
 27884
 
 gene;tss;island
 
 0.021144
 
 0.0119740
 
 Not queried
 
  0.6787623
 
       NA
 
           NA
  
 
  
 10
 
  60936383
 
  60937048
 
  ENSEMBL   UCSC 
 
  666
 
  9
 
 4595
 
 gene
 
 0.117422
 
 0.0692570
 
 Not queried
 
  0.6787610
 
       NA
 
           NA
  
 
  
 8
 
  70404514
 
  70404928
 
  ENSEMBL   UCSC 
 
  415
 
  7
 
 39329
 
 gene;tss
 
 0.015919
 
 0.0769311
 
 Not queried
 
  0.6787549
 
       NA
 
           NA
  
 
  
 17
 
  79885823
 
  79886116
 
  ENSEMBL   UCSC 
 
  294
 
  4
 
 16930
 
 gene
 
 0.007555
 
 0.0043401
 
 Not queried
 
  0.6786790
 
       NA
 
           NA
  
 
  
 19
 
  36631058
 
  36631318
 
  ENSEMBL   UCSC 
 
  261
 
  4
 
 20000
 
 gene
 
 0.001267
 
 0.0030261
 
 Not queried
 
  0.6786123
 
       NA
 
           NA
  
 
  
 19
 
  16308750
 
  16309072
 
  ENSEMBL   UCSC 
 
  323
 
  4
 
 19910
 
 gene
 
 0.010303
 
 0.0074776
 
 Not queried
 
  0.6785967
 
       NA
 
           NA
  
 
  
 4
 
   8588716
 
   8588929
 
  ENSEMBL   UCSC 
 
  214
 
  5
 
 29955
 
 gene;island
 
 0.451708
 
 0.6438547
 
 Not queried
 
  0.6784185
 
       NA
 
           NA
  
 
  
 17
 
  80923424
 
  80923777
 
  ENSEMBL   UCSC 
 
  354
 
  5
 
 16971
 
 gene;island
 
 0.917484
 
 0.8739923
 
 Not queried
 
  0.6783879
 
       NA
 
           NA
  
 
  
 8
 
  11536778
 
  11537112
 
  ENSEMBL   UCSC 
 
  335
 
  4
 
 40083
 
 island
 
 0.399834
 
 0.3085275
 
 Not queried
 
  0.6781153
 
       NA
 
           NA
  
 
  
 12
 
  54942782
 
  54943102
 
  ENSEMBL   UCSC 
 
  321
 
  5
 
 9765
 
 tss
 
 0.032109
 
 0.0953376
 
 Not queried
 
  0.6780480
 
       NA
 
           NA
  
 
  
 16
 
  48277879
 
  48278340
 
  ENSEMBL   UCSC 
 
  462
 
 11
 
 15902
 
 island
 
 0.007848
 
 0.0036990
 
 Not queried
 
  0.6780218
 
       NA
 
           NA
  
 
  
 11
 
  94964273
 
  94964516
 
  ENSEMBL   UCSC 
 
  244
 
  6
 
 7634
 
 tss
 
 0.004826
 
 0.0053884
 
 Not queried
 
  0.6779847
 
       NA
 
           NA
  
 
  
 11
 
  60623504
 
  60623918
 
  ENSEMBL   UCSC 
 
  415
 
  6
 
 7323
 
 tss
 
 0.250828
 
 0.3407647
 
 Not queried
 
  0.6779551
 
       NA
 
           NA
  
 
  
 2
 
 219866437
 
 219866628
 
  ENSEMBL   UCSC 
 
  192
 
  5
 
 23888
 
 tss
 
 0.065881
 
 0.1013516
 
 Not queried
 
  0.6779434
 
       NA
 
           NA
  
 
  
 4
 
 105416281
 
 105416480
 
  ENSEMBL   UCSC 
 
  200
 
  4
 
 31219
 
 island
 
 0.015621
 
 0.0094860
 
 Not queried
 
  0.6777692
 
       NA
 
           NA
  
 
  
 19
 
  46272454
 
  46272660
 
  ENSEMBL   UCSC 
 
  207
 
  5
 
 22156
 
 island
 
 0.006388
 
 0.0050054
 
 Not queried
 
  0.6777240
 
       NA
 
           NA
  
 
  
 17
 
  42992567
 
  42993050
 
  ENSEMBL   UCSC 
 
  484
 
  8
 
 18415
 
 island
 
 0.533112
 
 0.6901579
 
 Not queried
 
  0.6774025
 
       NA
 
           NA
  
 
  
 20
 
  61147572
 
  61147679
 
  ENSEMBL   UCSC 
 
  108
 
  6
 
 25487
 
 gene;island
 
 0.043418
 
 0.0187207
 
 Not queried
 
  0.6773041
 
       NA
 
           NA
  
 
  
 6
 
  31539973
 
  31540051
 
  ENSEMBL   UCSC 
 
   79
 
  7
 
 34818
 
 tss
 
 0.003117
 
 0.0051929
 
 Not queried
 
  0.6772371
 
       NA
 
           NA
  
 
  
 6
 
 134209935
 
 134210307
 
  ENSEMBL   UCSC 
 
  373
 
  9
 
 36489
 
 island
 
 0.062407
 
 0.2636364
 
 Not queried
 
  0.6772352
 
       NA
 
           NA
  
 
  
 14
 
  27067638
 
  27068135
 
  ENSEMBL   UCSC 
 
  498
 
  5
 
 12334
 
 tss;island
 
 0.039757
 
 0.0322807
 
 Not queried
 
  0.6772310
 
       NA
 
           NA
  
 
  
 4
 
 147561669
 
 147562073
 
  ENSEMBL   UCSC 
 
  405
 
  4
 
 30149
 
 gene;island
 
 0.066501
 
 0.1466102
 
 Not queried
 
  0.6772299
 
       NA
 
           NA
  
 
  
 1
 
  48058877
 
  48059354
 
  ENSEMBL   UCSC 
 
  478
 
  5
 
 3243
 
 island
 
 0.018041
 
 0.0283346
 
 Not queried
 
  0.6770562
 
       NA
 
           NA
  
 
  
 5
 
 148960831
 
 148961011
 
  ENSEMBL   UCSC 
 
  181
 
  7
 
 32420
 
 tss;island
 
 0.040465
 
 0.0224069
 
 Not queried
 
  0.6770178
 
       NA
 
           NA
  
 
  
 7
 
  57247619
 
  57248018
 
  ENSEMBL   UCSC 
 
  400
 
 10
 
 38517
 
 island
 
 0.519139
 
 0.6031032
 
 Not queried
 
  0.6769910
 
       NA
 
           NA
  
 
  
 10
 
 120840446
 
 120840913
 
  ENSEMBL   UCSC 
 
  468
 
  9
 
 5398
 
 tss;island
 
 0.007604
 
 0.0087830
 
 Not queried
 
  0.6768305
 
       NA
 
           NA
  
 
  
 1
 
  90309205
 
  90309605
 
  ENSEMBL   UCSC 
 
  401
 
  5
 
 622
 
 gene;island
 
 0.003985
 
 0.0072687
 
 Not queried
 
  0.6767900
 
       NA
 
           NA
  
 
  
 14
 
  23298687
 
  23299469
 
  ENSEMBL   UCSC 
 
  783
 
 13
 
 12702
 
 island
 
 0.005853
 
 0.0104240
 
 Not queried
 
  0.6764987
 
       NA
 
           NA
  
 
  
 19
 
  19303443
 
  19303735
 
  ENSEMBL   UCSC 
 
  293
 
  5
 
 20637
 
 tss
 
 0.013480
 
 0.0056255
 
 Not queried
 
  0.6762202
 
       NA
 
           NA
  
 
  
 1
 
 203097234
 
 203097628
 
  ENSEMBL   UCSC 
 
  395
 
  5
 
 950
 
 gene;island
 
 0.029887
 
 0.0129273
 
 Not queried
 
  0.6761585
 
       NA
 
           NA
  
 
  
 8
 
 145018010
 
 145018300
 
  ENSEMBL   UCSC 
 
  291
 
  5
 
 39499
 
 gene;tss;island
 
 0.026907
 
 0.0416285
 
 Not queried
 
  0.6761001
 
       NA
 
           NA
  
 
  
 4
 
  15005434
 
  15005517
 
  ENSEMBL   UCSC 
 
   84
 
  6
 
 30307
 
 tss
 
 0.003375
 
 0.0027940
 
 Not queried
 
  0.6760883
 
       NA
 
           NA
  
 
  
 3
 
  52931527
 
  52931946
 
  ENSEMBL   UCSC 
 
  420
 
  8
 
 29195
 
 island
 
 0.005815
 
 0.0037516
 
 Not queried
 
  0.6760215
 
       NA
 
           NA
  
 
  
 3
 
 111805225
 
 111805408
 
  ENSEMBL   UCSC 
 
  184
 
  6
 
 27894
 
 gene
 
 0.023141
 
 0.0170926
 
 Not queried
 
  0.6759720
 
       NA
 
           NA
  
 
  
 12
 
  89919186
 
  89919388
 
  ENSEMBL   UCSC 
 
  203
 
  4
 
 9239
 
 gene;tss;island
 
 0.022080
 
 0.0117427
 
 Not queried
 
  0.6759074
 
       NA
 
           NA
  
 
  
 4
 
    980604
 
    980676
 
  ENSEMBL   UCSC 
 
   73
 
  5
 
 29852
 
 gene;tss;island
 
 0.014946
 
 0.0130244
 
 Not queried
 
  0.6758113
 
       NA
 
           NA
  
 
  
 5
 
 170814123
 
 170814656
 
  ENSEMBL   UCSC 
 
  534
 
 10
 
 32487
 
 tss
 
 0.011713
 
 0.0059231
 
 Not queried
 
  0.6755653
 
       NA
 
           NA
  
 
  
 14
 
  96505296
 
  96505636
 
  ENSEMBL   UCSC 
 
  341
 
  9
 
 12591
 
 tss
 
 0.031490
 
 0.1038530
 
 Not queried
 
  0.6754571
 
       NA
 
           NA
  
 
  
 2
 
 192542487
 
 192542626
 
  ENSEMBL   UCSC 
 
  140
 
  7
 
 23790
 
 tss;island
 
 0.007070
 
 0.0039758
 
 Not queried
 
  0.6753097
 
       NA
 
           NA
  
 
  
 17
 
  40610755
 
  40610858
 
  ENSEMBL   UCSC 
 
  104
 
  6
 
 17363
 
 tss
 
 0.006895
 
 0.0022647
 
 Not queried
 
  0.6752714
 
       NA
 
           NA
  
 
  
 17
 
  64188108
 
  64188166
 
  ENSEMBL   UCSC 
 
   59
 
  5
 
 17594
 
 tss;island
 
 0.011957
 
 0.0042578
 
 Not queried
 
  0.6752496
 
       NA
 
           NA
  
 
  
 8
 
  97505593
 
  97505868
 
  ENSEMBL   UCSC 
 
  276
 
  7
 
 39827
 
 tss;island
 
 0.033613
 
 0.0415419
 
 Not queried
 
  0.6752186
 
       NA
 
           NA
  
 
  
 11
 
 118977782
 
 118978198
 
  ENSEMBL   UCSC 
 
  417
 
  9
 
 8874
 
 island
 
 0.005462
 
 0.0045368
 
 Not queried
 
  0.6751042
 
       NA
 
           NA
  
 
  
 21
 
  40817540
 
  40818036
 
  ENSEMBL   UCSC 
 
  497
 
  7
 
 26685
 
 island
 
 0.002422
 
 0.0073050
 
 Not queried
 
  0.6750846
 
       NA
 
           NA
  
 
  
 11
 
  18548210
 
  18548408
 
  ENSEMBL   UCSC 
 
  199
 
  4
 
 6531
 
 gene
 
 0.008720
 
 0.0073694
 
 Not queried
 
  0.6749468
 
       NA
 
           NA
  
 
  
 3
 
  49893745
 
  49894441
 
  ENSEMBL   UCSC 
 
  697
 
 13
 
 29131
 
 island
 
 0.015811
 
 0.0224445
 
 Not queried
 
  0.6749433
 
       NA
 
           NA
  
 
  
 16
 
  66878426
 
  66879016
 
  ENSEMBL   UCSC 
 
  591
 
 11
 
 14783
 
 gene;island
 
 0.017990
 
 0.0115227
 
 Not queried
 
  0.6748649
 
       NA
 
           NA
  
 
  
 16
 
  66878426
 
  66878810
 
  ENSEMBL   UCSC 
 
  385
 
  9
 
 15286
 
 tss
 
 0.015670
 
 0.0115227
 
 Not queried
 
  0.6748649
 
       NA
 
           NA
  
 
  
 12
 
  52760975
 
  52761221
 
  ENSEMBL   UCSC 
 
  247
 
  4
 
 9138
 
 gene
 
 0.648300
 
 0.7623168
 
 Not queried
 
  0.6746968
 
       NA
 
           NA
  
 
  
 19
 
  38886554
 
  38886789
 
  ENSEMBL   UCSC 
 
  236
 
  4
 
 20016
 
 gene;island
 
 0.028397
 
 0.0351655
 
 Not queried
 
  0.6746239
 
       NA
 
           NA
  
 
  
 1
 
  15930094
 
  15930386
 
  ENSEMBL   UCSC 
 
  293
 
  4
 
 2722
 
 island
 
 0.028844
 
 0.0166657
 
 Not queried
 
  0.6745988
 
       NA
 
           NA
  
 
  
 19
 
   5997013
 
   5997434
 
  ENSEMBL   UCSC 
 
  422
 
  5
 
 19759
 
 gene;island
 
 0.962713
 
 0.9801280
 
 Not queried
 
  0.6745333
 
       NA
 
           NA
  
 
  
 2
 
  27958142
 
  27958897
 
  ENSEMBL   UCSC 
 
  756
 
  8
 
 24191
 
 island
 
 0.039739
 
 0.0865990
 
 Not queried
 
  0.6744322
 
       NA
 
           NA
  
 
  
 8
 
 140716495
 
 140716766
 
  ENSEMBL   UCSC 
 
  272
 
  6
 
 39924
 
 tss;island
 
 0.028487
 
 0.0280580
 
 Not queried
 
  0.6741237
 
       NA
 
           NA
  
 
  
 16
 
  12897684
 
  12897821
 
  ENSEMBL   UCSC 
 
  138
 
  4
 
 15715
 
 island
 
 0.002165
 
 0.0025663
 
 Not queried
 
  0.6739710
 
       NA
 
           NA
  
 
  
 2
 
 233561679
 
 233561917
 
  ENSEMBL   UCSC 
 
  239
 
  6
 
 23954
 
 tss
 
 0.039480
 
 0.0131362
 
 Not queried
 
  0.6738946
 
       NA
 
           NA
  
 
  
 7
 
 140372725
 
 140373033
 
  ENSEMBL   UCSC 
 
  309
 
  6
 
 38962
 
 island
 
 0.011337
 
 0.0092823
 
 Not queried
 
  0.6737118
 
       NA
 
           NA
  
 
  
 7
 
 155436277
 
 155437171
 
  ENSEMBL   UCSC 
 
  895
 
 12
 
 38013
 
 tss
 
 0.020325
 
 0.0221174
 
 Not queried
 
  0.6736856
 
       NA
 
           NA
  
 
  
 5
 
 133702574
 
 133702757
 
  ENSEMBL   UCSC 
 
  184
 
  4
 
 31751
 
 gene
 
 0.009835
 
 0.0102683
 
 Not queried
 
  0.6736545
 
       NA
 
           NA
  
 
  
 17
 
  27224533
 
  27225046
 
  ENSEMBL   UCSC 
 
  514
 
  9
 
 16503
 
 gene;island
 
 0.002547
 
 0.0243828
 
 Not queried
 
  0.6736461
 
       NA
 
           NA
  
 
  
 10
 
  70480731
 
  70480922
 
  ENSEMBL   UCSC 
 
  192
 
  6
 
 5115
 
 tss
 
 0.004903
 
 0.0023743
 
 Not queried
 
  0.6734710
 
       NA
 
           NA
  
 
  
 18
 
   8659509
 
   8659964
 
  ENSEMBL   UCSC 
 
  456
 
  5
 
 19382
 
 island
 
 0.570772
 
 0.6562108
 
 Not queried
 
  0.6733790
 
       NA
 
           NA
  
 
  
 13
 
 114321214
 
 114321595
 
  ENSEMBL   UCSC 
 
  382
 
  6
 
 11555
 
 tss
 
 0.532662
 
 0.6429112
 
 Not queried
 
  0.6733433
 
       NA
 
           NA
  
 
  
 8
 
   1642380
 
   1642625
 
  ENSEMBL   UCSC 
 
  246
 
  4
 
 39172
 
 gene;island
 
 0.597638
 
 0.8452637
 
 Not queried
 
  0.6730835
 
       NA
 
           NA
  
 
  
 3
 
  12837740
 
  12838136
 
  ENSEMBL   UCSC 
 
  397
 
  7
 
 28161
 
 tss
 
 0.022662
 
 0.0353471
 
 Not queried
 
  0.6729767
 
       NA
 
           NA
  
 
  
 2
 
  47169024
 
  47169299
 
  ENSEMBL   UCSC 
 
  276
 
  6
 
 23341
 
 tss
 
 0.004925
 
 0.0104744
 
 Not queried
 
  0.6728031
 
       NA
 
           NA
  
 
  
 5
 
 177557700
 
 177558283
 
  ENSEMBL   UCSC 
 
  584
 
  7
 
 33463
 
 island
 
 0.004844
 
 0.0118448
 
 Not queried
 
  0.6727647
 
       NA
 
           NA
  
 
  
 13
 
 112610681
 
 112611028
 
  ENSEMBL   UCSC 
 
  348
 
  4
 
 11904
 
 island
 
 0.728634
 
 0.7297460
 
 Not queried
 
  0.6726835
 
       NA
 
           NA
  
 
  
 11
 
  36531914
 
  36532056
 
  ENSEMBL   UCSC 
 
  143
 
  4
 
 7249
 
 tss
 
 0.008038
 
 0.0040557
 
 Not queried
 
  0.6725961
 
       NA
 
           NA
  
 
  
 2
 
  19562750
 
  19563449
 
  ENSEMBL   UCSC 
 
  700
 
 10
 
 24104
 
 island
 
 0.019179
 
 0.0240152
 
 Not queried
 
  0.6725022
 
       NA
 
           NA
  
 
  
 19
 
   1168733
 
   1169138
 
  ENSEMBL   UCSC 
 
  406
 
  4
 
 19648
 
 gene;island
 
 0.918798
 
 0.9433384
 
 Not queried
 
  0.6724330
 
       NA
 
           NA
  
 
  
 10
 
  61666543
 
  61667009
 
  ENSEMBL   UCSC 
 
  467
 
  4
 
 5765
 
 island
 
 0.020254
 
 0.0158401
 
 Not queried
 
  0.6724156
 
       NA
 
           NA
  
 
  
 5
 
  37379267
 
  37379396
 
  ENSEMBL   UCSC 
 
  130
 
  6
 
 32024
 
 tss
 
 0.008826
 
 0.0056831
 
 Not queried
 
  0.6722913
 
       NA
 
           NA
  
 
  
 12
 
 132293329
 
 132293702
 
  ENSEMBL   UCSC 
 
  374
 
  5
 
 11015
 
 island
 
 0.906516
 
 0.9392199
 
 Not queried
 
  0.6722504
 
       NA
 
           NA
  
 
  
 1
 
  36788387
 
  36788627
 
  ENSEMBL   UCSC 
 
  241
 
  4
 
 427
 
 gene;island
 
 0.027837
 
 0.1492874
 
 Not queried
 
  0.6722371
 
       NA
 
           NA
  
 
  
 5
 
 114961835
 
 114962299
 
  ENSEMBL   UCSC 
 
  465
 
  8
 
 33026
 
 island
 
 0.002525
 
 0.0011905
 
 Not queried
 
  0.6722354
 
       NA
 
           NA
  
 
  
 5
 
 101834365
 
 101834675
 
  ENSEMBL   UCSC 
 
  311
 
  4
 
 31703
 
 gene
 
 0.816992
 
 0.8765172
 
 Not queried
 
  0.6722246
 
       NA
 
           NA
  
 
  
 6
 
 139694265
 
 139694674
 
  ENSEMBL   UCSC 
 
  410
 
  4
 
 34491
 
 gene;island
 
 0.006065
 
 0.0060987
 
 Not queried
 
  0.6721546
 
       NA
 
           NA
  
 
  
 8
 
  13424080
 
  13424269
 
  ENSEMBL   UCSC 
 
  190
 
  5
 
 39561
 
 tss
 
 0.023562
 
 0.0184452
 
 Not queried
 
  0.6720985
 
       NA
 
           NA
  
 
  
 1
 
   6526049
 
   6526583
 
  ENSEMBL   UCSC 
 
  535
 
 12
 
 174
 
 gene;island
 
 0.052645
 
 0.0505809
 
 Not queried
 
  0.6720105
 
       NA
 
           NA
  
 
  
 6
 
  52441723
 
  52441892
 
  ENSEMBL   UCSC 
 
  170
 
  4
 
 36204
 
 island
 
 0.010429
 
 0.0128692
 
 Not queried
 
  0.6720073
 
       NA
 
           NA
  
 
  
 12
 
 112123558
 
 112123710
 
  ENSEMBL   UCSC 
 
  153
 
  6
 
 9310
 
 gene
 
 0.046031
 
 0.0072994
 
 Not queried
 
  0.6719035
 
       NA
 
           NA
  
 
  
 10
 
   5060291
 
   5060566
 
  ENSEMBL   UCSC 
 
  276
 
  4
 
 4921
 
 tss
 
 0.010910
 
 0.1704294
 
 Not queried
 
  0.6719033
 
       NA
 
           NA
  
 
  
 6
 
  35309867
 
  35310274
 
  ENSEMBL   UCSC 
 
  408
 
  7
 
 34929
 
 tss;island
 
 0.022433
 
 0.0146977
 
 Not queried
 
  0.6718559
 
       NA
 
           NA
  
 
  
 17
 
  72869156
 
  72869237
 
  ENSEMBL   UCSC 
 
   82
 
  6
 
 17633
 
 tss
 
 0.005479
 
 0.0041425
 
 Not queried
 
  0.6715505
 
       NA
 
           NA
  
 
  
 19
 
  41630511
 
  41630815
 
  ENSEMBL   UCSC 
 
  305
 
  5
 
 20053
 
 gene;island
 
 0.966044
 
 0.9780535
 
 Not queried
 
  0.6714633
 
       NA
 
           NA
  
 
  
 7
 
  75115627
 
  75115859
 
  ENSEMBL   UCSC 
 
  233
 
  8
 
 37653
 
 tss
 
 0.003987
 
 0.0026866
 
 Not queried
 
  0.6714407
 
       NA
 
           NA
  
 
  
 8
 
   6664028
 
   6664281
 
  ENSEMBL   UCSC 
 
  254
 
  4
 
 40053
 
 island
 
 0.592503
 
 0.7030556
 
 Not queried
 
  0.6714299
 
       NA
 
           NA
  
 
  
 20
 
  30225325
 
  30226046
 
  ENSEMBL   UCSC 
 
  722
 
  9
 
 26077
 
 island
 
 0.623873
 
 0.5381193
 
 Not queried
 
  0.6714144
 
       NA
 
           NA
  
 
  
 17
 
  77708928
 
  77709291
 
  ENSEMBL   UCSC 
 
  364
 
  4
 
 16842
 
 gene;island
 
 0.914826
 
 0.9195354
 
 Not queried
 
  0.6713195
 
       NA
 
           NA
  
 
  
 3
 
 138153127
 
 138153439
 
  ENSEMBL   UCSC 
 
  313
 
  9
 
 29521
 
 island
 
 0.060995
 
 0.0231789
 
 Not queried
 
  0.6712920
 
       NA
 
           NA
  
 
  
 1
 
  44456953
 
  44457185
 
  ENSEMBL   UCSC 
 
  233
 
  5
 
 1569
 
 tss
 
 0.003908
 
 0.0014267
 
 Not queried
 
  0.6708527
 
       NA
 
           NA
  
 
  
 15
 
  80445127
 
  80445334
 
  ENSEMBL   UCSC 
 
  208
 
  6
 
 13849
 
 tss
 
 0.025535
 
 0.0224944
 
 Not queried
 
  0.6707693
 
       NA
 
           NA
  
 
  
 14
 
  20903410
 
  20903611
 
  ENSEMBL   UCSC 
 
  202
 
  4
 
 12005
 
 gene;island
 
 0.975906
 
 0.9720863
 
 Not queried
 
  0.6707121
 
       NA
 
           NA
  
 
  
 17
 
  25308830
 
  25309467
 
  ENSEMBL   UCSC 
 
  638
 
  7
 
 18132
 
 island
 
 0.453178
 
 0.7043065
 
 Not queried
 
  0.6706512
 
       NA
 
           NA
  
 
  
 15
 
  82555336
 
  82555552
 
  ENSEMBL   UCSC 
 
  217
 
  4
 
 13452
 
 gene;tss;island
 
 0.139789
 
 0.0193621
 
 Not queried
 
  0.6704947
 
       NA
 
           NA
  
 
  
 1
 
 200639189
 
 200639502
 
  ENSEMBL   UCSC 
 
  314
 
  5
 
 2161
 
 tss
 
 0.008601
 
 0.0008894
 
 Not queried
 
  0.6704823
 
       NA
 
           NA
  
 
  
 14
 
 101499893
 
 101500080
 
  ENSEMBL   UCSC 
 
  188
 
  4
 
 12624
 
 tss
 
 0.354430
 
 0.5248206
 
 Not queried
 
  0.6704308
 
       NA
 
           NA
  
 
  
 14
 
 102695264
 
 102695875
 
  ENSEMBL   UCSC 
 
  612
 
  7
 
 12230
 
 gene;island
 
 0.938499
 
 0.9609249
 
 Not queried
 
  0.6702831
 
       NA
 
           NA
  
 
  
 20
 
  47013687
 
  47013919
 
  ENSEMBL   UCSC 
 
  233
 
  4
 
 26226
 
 island
 
 0.836398
 
 0.7659804
 
 Not queried
 
  0.6700994
 
       NA
 
           NA
  
 
  
 7
 
  73184526
 
  73184671
 
  ENSEMBL   UCSC 
 
  146
 
  7
 
 38568
 
 island
 
 0.009415
 
 0.0224112
 
 Not queried
 
  0.6700679
 
       NA
 
           NA
  
 
  
 3
 
  43122118
 
  43122470
 
  ENSEMBL   UCSC 
 
  353
 
  5
 
 27738
 
 gene;island
 
 0.518770
 
 0.6523195
 
 Not queried
 
  0.6699708
 
       NA
 
           NA
  
 
  
 12
 
  42877401
 
  42877658
 
  ENSEMBL   UCSC 
 
  258
 
  6
 
 9097
 
 gene;island
 
 0.009868
 
 0.0078477
 
 Not queried
 
  0.6699581
 
       NA
 
           NA
  
 
  
 18
 
    580188
 
    580334
 
  ENSEMBL   UCSC 
 
  147
 
  6
 
 19151
 
 tss
 
 0.650784
 
 0.8102431
 
 Not queried
 
  0.6698451
 
       NA
 
           NA
  
 
  
 5
 
 115910592
 
 115910732
 
  ENSEMBL   UCSC 
 
  141
 
  4
 
 32233
 
 tss
 
 0.011256
 
 0.0078357
 
 Not queried
 
  0.6698015
 
       NA
 
           NA
  
 
  
 3
 
 122512442
 
 122513065
 
  ENSEMBL   UCSC 
 
  624
 
 10
 
 28541
 
 tss;island
 
 0.009951
 
 0.0062771
 
 Not queried
 
  0.6697198
 
       NA
 
           NA
  
 
  
 22
 
  45405621
 
  45405919
 
  ENSEMBL   UCSC 
 
  299
 
  9
 
 27216
 
 tss;island
 
 0.015444
 
 0.0151190
 
 Not queried
 
  0.6697015
 
       NA
 
           NA
  
 
  
 18
 
  43684692
 
  43685106
 
  ENSEMBL   UCSC 
 
  415
 
  6
 
 19102
 
 gene;tss;island
 
 0.006691
 
 0.0117809
 
 Not queried
 
  0.6695211
 
       NA
 
           NA
  
 
  
 19
 
  15560076
 
  15560314
 
  ENSEMBL   UCSC 
 
  239
 
  8
 
 19907
 
 gene;tss;island
 
 0.017936
 
 0.0144820
 
 Not queried
 
  0.6694852
 
       NA
 
           NA
  
 
  
 6
 
  72130209
 
  72130799
 
  ENSEMBL   UCSC 
 
  591
 
 12
 
 36243
 
 island
 
 0.080626
 
 0.1003853
 
 Not queried
 
  0.6694505
 
       NA
 
           NA
  
 
  
 3
 
 196065289
 
 196065688
 
  ENSEMBL   UCSC 
 
  400
 
 10
 
 28812
 
 tss
 
 0.836334
 
 0.8821195
 
 Not queried
 
  0.6693607
 
       NA
 
           NA
  
 
  
 16
 
   1401824
 
   1402058
 
  ENSEMBL   UCSC 
 
  235
 
  5
 
 14991
 
 tss;island
 
 0.003262
 
 0.0013832
 
 Not queried
 
  0.6692841
 
       NA
 
           NA
  
 
  
 10
 
  49864382
 
  49864621
 
  ENSEMBL   UCSC 
 
  240
 
  5
 
 5727
 
 island
 
 0.012451
 
 0.0079026
 
 Not queried
 
  0.6692296
 
       NA
 
           NA
  
 
  
 1
 
 205560943
 
 205561401
 
  ENSEMBL   UCSC 
 
  459
 
  9
 
 967
 
 gene;island
 
 0.006210
 
 0.0083734
 
 Not queried
 
  0.6690376
 
       NA
 
           NA
  
 
  
 6
 
 170101828
 
 170102133
 
  ENSEMBL   UCSC 
 
  306
 
  5
 
 34575
 
 gene
 
 0.003863
 
 0.0024434
 
 Not queried
 
  0.6689492
 
       NA
 
           NA
  
 
  
 19
 
   1021257
 
   1021368
 
  ENSEMBL   UCSC 
 
  112
 
  4
 
 20288
 
 tss;island
 
 0.004032
 
 0.0043102
 
 Not queried
 
  0.6689354
 
       NA
 
           NA
  
 
  
 1
 
 150980086
 
 150980411
 
  ENSEMBL   UCSC 
 
  326
 
  4
 
 744
 
 gene;tss;island
 
 0.933652
 
 0.9431956
 
 Not queried
 
  0.6688874
 
       NA
 
           NA
  
 
  
 6
 
  70576168
 
  70577198
 
  ENSEMBL   UCSC 
 
 1031
 
 13
 
 36235
 
 island
 
 0.003240
 
 0.0031178
 
 Not queried
 
  0.6687925
 
       NA
 
           NA
  
 
  
 16
 
  87984259
 
  87985026
 
  ENSEMBL   UCSC 
 
  768
 
  7
 
 15408
 
 tss
 
 0.016542
 
 0.0145869
 
 Not queried
 
  0.6687892
 
       NA
 
           NA
  
 
  
 4
 
  17516413
 
  17516819
 
  ENSEMBL   UCSC 
 
  407
 
  7
 
 30945
 
 island
 
 0.745848
 
 0.8372324
 
 Not queried
 
  0.6685255
 
       NA
 
           NA
  
 
  
 12
 
 113376135
 
 113376681
 
  ENSEMBL   UCSC 
 
  547
 
  5
 
 10823
 
 island
 
 0.011154
 
 0.0102900
 
 Not queried
 
  0.6683593
 
       NA
 
           NA
  
 
  
 10
 
 112632370
 
 112632681
 
  ENSEMBL   UCSC 
 
  312
 
  4
 
 4757
 
 gene;island
 
 0.006571
 
 0.0079592
 
 Not queried
 
  0.6682510
 
       NA
 
           NA
  
 
  
 3
 
 107809956
 
 107810145
 
  ENSEMBL   UCSC 
 
  190
 
  4
 
 28487
 
 tss;island
 
 0.007248
 
 0.0071954
 
 Not queried
 
  0.6680223
 
       NA
 
           NA
  
 
  
 15
 
  57998711
 
  57998894
 
  ENSEMBL   UCSC 
 
  184
 
  5
 
 13696
 
 tss
 
 0.001229
 
 0.0016833
 
 Not queried
 
  0.6679153
 
       NA
 
           NA
  
 
  
 6
 
   3140546
 
   3140901
 
  ENSEMBL   UCSC 
 
  356
 
  5
 
 33535
 
 gene;island
 
 0.950711
 
 0.9721168
 
 Not queried
 
  0.6677828
 
       NA
 
           NA
  
 
  
 10
 
 135342218
 
 135342620
 
  ENSEMBL   UCSC 
 
  403
 
  4
 
 4907
 
 gene;island
 
 0.289649
 
 0.3886441
 
 Not queried
 
  0.6675825
 
       NA
 
           NA
  
 
  
 6
 
  41339246
 
  41340058
 
  ENSEMBL   UCSC 
 
  813
 
  7
 
 36119
 
 island
 
 0.031433
 
 0.0206386
 
 Not queried
 
  0.6675085
 
       NA
 
           NA
  
 
  
 14
 
  97208114
 
  97208333
 
  ENSEMBL   UCSC 
 
  220
 
  4
 
 13120
 
 island
 
 0.303651
 
 0.4566487
 
 Not queried
 
  0.6674107
 
       NA
 
           NA
  
 
  
 1
 
   3775078
 
   3775334
 
  ENSEMBL   UCSC 
 
  257
 
  4
 
 149
 
 gene;island
 
 0.947424
 
 0.9690321
 
 Not queried
 
  0.6673807
 
       NA
 
           NA
  
 
  
 12
 
  22777790
 
  22778127
 
  ENSEMBL   UCSC 
 
  338
 
 10
 
 10286
 
 island
 
 0.014820
 
 0.0095447
 
 Not queried
 
  0.6671768
 
       NA
 
           NA
  
 
  
 2
 
 239008986
 
 239009502
 
  ENSEMBL   UCSC 
 
  517
 
  7
 
 23094
 
 gene
 
 0.974442
 
 0.9837210
 
 Not queried
 
  0.6670301
 
       NA
 
           NA
  
 
  
 10
 
 134994186
 
 134994455
 
  ENSEMBL   UCSC 
 
  270
 
  5
 
 4889
 
 gene;island
 
 0.857382
 
 0.9624789
 
 Not queried
 
  0.6670236
 
       NA
 
           NA
  
 
  
 1
 
 214776613
 
 214776904
 
  ENSEMBL   UCSC 
 
  292
 
  4
 
 1008
 
 gene
 
 0.006880
 
 0.0028119
 
 Not queried
 
  0.6669833
 
       NA
 
           NA
  
 
  
 14
 
  60632234
 
  60632532
 
  ENSEMBL   UCSC 
 
  299
 
  4
 
 12436
 
 tss
 
 0.008156
 
 0.0054932
 
 Not queried
 
  0.6669814
 
       NA
 
           NA
  
 
  
 20
 
  24449668
 
  24449804
 
  ENSEMBL   UCSC 
 
  137
 
  5
 
 25642
 
 tss
 
 0.049602
 
 0.0897371
 
 Not queried
 
  0.6669518
 
       NA
 
           NA
  
 
  
 19
 
   9271911
 
   9272145
 
  ENSEMBL   UCSC 
 
  235
 
  4
 
 19799
 
 gene;island
 
 0.963091
 
 0.9762054
 
 Not queried
 
  0.6669014
 
       NA
 
           NA
  
 
  
 7
 
  99214442
 
  99214548
 
  ENSEMBL   UCSC 
 
  107
 
  7
 
 37761
 
 tss
 
 0.009821
 
 0.0035078
 
 Not queried
 
  0.6668937
 
       NA
 
           NA
  
 
  
 13
 
  70682019
 
  70682324
 
  ENSEMBL   UCSC 
 
  306
 
  4
 
 11175
 
 gene;island
 
 0.126099
 
 0.2878999
 
 Not queried
 
  0.6668787
 
       NA
 
           NA
  
 
  
 1
 
   1342230
 
   1342795
 
  ENSEMBL   UCSC 
 
  566
 
  6
 
 2435
 
 island
 
 0.006047
 
 0.0048178
 
 Not queried
 
  0.6667705
 
       NA
 
           NA
  
 
  
 1
 
  20878760
 
  20878928
 
  ENSEMBL   UCSC 
 
  169
 
  4
 
 1315
 
 tss
 
 0.055666
 
 0.0343364
 
 Not queried
 
  0.6667657
 
       NA
 
           NA
  
 
  
 5
 
   1225074
 
   1225434
 
  ENSEMBL   UCSC 
 
  361
 
  8
 
 31968
 
 tss
 
 0.373226
 
 0.3788388
 
 Not queried
 
  0.6667363
 
       NA
 
           NA
  
 
  
 11
 
 128812825
 
 128813223
 
  ENSEMBL   UCSC 
 
  399
 
 10
 
 7809
 
 tss
 
 0.443386
 
 0.5555027
 
 Not queried
 
  0.6665618
 
       NA
 
           NA
  
 
  
 16
 
  77822083
 
  77822441
 
  ENSEMBL   UCSC 
 
  359
 
  8
 
 15371
 
 tss
 
 0.014019
 
 0.0249984
 
 Not queried
 
  0.6665221
 
       NA
 
           NA
  
 
  
 2
 
 206950754
 
 206951158
 
  ENSEMBL   UCSC 
 
  405
 
  6
 
 25030
 
 island
 
 0.005947
 
 0.0044456
 
 Not queried
 
  0.6663609
 
       NA
 
           NA
  
 
  
 5
 
 180650106
 
 180650516
 
  ENSEMBL   UCSC 
 
  411
 
  8
 
 33523
 
 island
 
 0.004982
 
 0.0099580
 
 Not queried
 
  0.6663127
 
       NA
 
           NA
  
 
  
 10
 
  64564161
 
  64564468
 
  ENSEMBL   UCSC 
 
  308
 
  7
 
 5102
 
 tss
 
 0.008799
 
 0.0065962
 
 Not queried
 
  0.6662337
 
       NA
 
           NA
  
 
  
 15
 
  62457456
 
  62457681
 
  ENSEMBL   UCSC 
 
  226
 
  4
 
 14199
 
 island
 
 0.120602
 
 0.0799306
 
 Not queried
 
  0.6661282
 
       NA
 
           NA
  
 
  
 12
 
  56694186
 
  56694667
 
  ENSEMBL   UCSC 
 
  482
 
  9
 
 9794
 
 tss
 
 0.032835
 
 0.0341482
 
 Not queried
 
  0.6661050
 
       NA
 
           NA
  
 
  
 14
 
  45603667
 
  45603864
 
  ENSEMBL   UCSC 
 
  198
 
  6
 
 12372
 
 tss;island
 
 0.002442
 
 0.0023692
 
 Not queried
 
  0.6661032
 
       NA
 
           NA
  
 
  
 17
 
  42015369
 
  42015961
 
  ENSEMBL   UCSC 
 
  593
 
  7
 
 18381
 
 island
 
 0.029159
 
 0.0266671
 
 Not queried
 
  0.6660044
 
       NA
 
           NA
  
 
  
 7
 
  43965451
 
  43965942
 
  ENSEMBL   UCSC 
 
  492
 
  8
 
 36986
 
 gene;tss;island
 
 0.025551
 
 0.0122838
 
 Not queried
 
  0.6659966
 
       NA
 
           NA
  
 
  
 3
 
 112709521
 
 112709793
 
  ENSEMBL   UCSC 
 
  273
 
  8
 
 28503
 
 tss;island
 
 0.017092
 
 0.0130670
 
 Not queried
 
  0.6659927
 
       NA
 
           NA
  
 
  
 2
 
 135809693
 
 135809808
 
  ENSEMBL   UCSC 
 
  116
 
  5
 
 23643
 
 tss
 
 0.005892
 
 0.0152354
 
 Not queried
 
  0.6658541
 
       NA
 
           NA
  
 
  
 10
 
  89419177
 
  89419384
 
  ENSEMBL   UCSC 
 
  208
 
  5
 
 5203
 
 tss;island
 
 0.010713
 
 0.0065007
 
 Not queried
 
  0.6658446
 
       NA
 
           NA
  
 
  
 8
 
  42995089
 
  42995564
 
  ENSEMBL   UCSC 
 
  476
 
  4
 
 39686
 
 tss
 
 0.013091
 
 0.0166069
 
 Not queried
 
  0.6657623
 
       NA
 
           NA
  
 
  
 10
 
 118609728
 
 118609965
 
  ENSEMBL   UCSC 
 
  238
 
  4
 
 6168
 
 island
 
 0.430157
 
 0.4135781
 
 Not queried
 
  0.6656796
 
       NA
 
           NA
  
 
  
 7
 
  29605624
 
  29606082
 
  ENSEMBL   UCSC 
 
  459
 
  4
 
 36950
 
 gene;island
 
 0.085116
 
 0.0569060
 
 Not queried
 
  0.6655898
 
       NA
 
           NA
  
 
  
 5
 
 176900792
 
 176900842
 
  ENSEMBL   UCSC 
 
   51
 
  5
 
 32529
 
 tss
 
 0.012999
 
 0.0138170
 
 Not queried
 
  0.6654202
 
       NA
 
           NA
  
 
  
 10
 
  81205417
 
  81205554
 
  ENSEMBL   UCSC 
 
  138
 
  5
 
 5183
 
 tss;island
 
 0.004768
 
 0.0111940
 
 Not queried
 
  0.6654159
 
       NA
 
           NA
  
 
  
 7
 
    612458
 
    612876
 
  ENSEMBL   UCSC 
 
  419
 
  4
 
 36712
 
 gene;island
 
 0.931007
 
 0.9459037
 
 Not queried
 
  0.6654044
 
       NA
 
           NA
  
 
  
 6
 
 170101828
 
 170102495
 
  ENSEMBL   UCSC 
 
  668
 
  8
 
 35351
 
 tss;island
 
 0.006761
 
 0.0032325
 
 Not queried
 
  0.6653612
 
       NA
 
           NA
  
 
  
 5
 
 138630302
 
 138630502
 
  ENSEMBL   UCSC 
 
  201
 
  4
 
 31773
 
 gene;island
 
 0.002379
 
 0.0032202
 
 Not queried
 
  0.6653305
 
       NA
 
           NA
  
 
  
 10
 
 135191257
 
 135192232
 
  ENSEMBL   UCSC 
 
  976
 
 29
 
 5464
 
 tss;island
 
 0.027639
 
 0.0648601
 
 Not queried
 
  0.6652811
 
       NA
 
           NA
  
 
  
 2
 
 172378774
 
 172379087
 
  ENSEMBL   UCSC 
 
  314
 
  4
 
 24854
 
 island
 
 0.009260
 
 0.0063805
 
 Not queried
 
  0.6652104
 
       NA
 
           NA
  
 
  
 7
 
  79083408
 
  79084196
 
  ENSEMBL   UCSC 
 
  789
 
 16
 
 37674
 
 tss;island
 
 0.037255
 
 0.0569209
 
 Not queried
 
  0.6651921
 
       NA
 
           NA
  
 
  
 4
 
  99064322
 
  99064904
 
  ENSEMBL   UCSC 
 
  583
 
  8
 
 31193
 
 island
 
 0.003043
 
 0.0009763
 
 Not queried
 
  0.6650807
 
       NA
 
           NA
  
 
  
 10
 
 134491205
 
 134491593
 
  ENSEMBL   UCSC 
 
  389
 
  5
 
 4872
 
 gene
 
 0.915337
 
 0.9634842
 
 Not queried
 
  0.6650379
 
       NA
 
           NA
  
 
  
 3
 
 138153127
 
 138153381
 
  ENSEMBL   UCSC 
 
  255
 
  8
 
 28623
 
 tss
 
 0.041851
 
 0.0306853
 
 Not queried
 
  0.6650126
 
       NA
 
           NA
  
 
  
 12
 
  65218069
 
  65218413
 
  ENSEMBL   UCSC 
 
  345
 
  4
 
 10597
 
 island
 
 0.009846
 
 0.0347200
 
 Not queried
 
  0.6649297
 
       NA
 
           NA
  
 
  
 16
 
   4525918
 
   4526445
 
  ENSEMBL   UCSC 
 
  528
 
  8
 
 14640
 
 gene;island
 
 0.027763
 
 0.0283035
 
 Not queried
 
  0.6648271
 
       NA
 
           NA
  
 
  
 6
 
  33095664
 
  33096501
 
  ENSEMBL   UCSC 
 
  838
 
  9
 
 34192
 
 gene
 
 0.502480
 
 0.4964642
 
 Not queried
 
  0.6647933
 
       NA
 
           NA
  
 
  
 1
 
 203598573
 
 203599089
 
  ENSEMBL   UCSC 
 
  517
 
  6
 
 953
 
 gene;island
 
 0.012090
 
 0.0077739
 
 Not queried
 
  0.6647803
 
       NA
 
           NA
  
 
  
 10
 
 130063978
 
 130064296
 
  ENSEMBL   UCSC 
 
  319
 
  4
 
 6255
 
 island
 
 0.162938
 
 0.2176809
 
 Not queried
 
  0.6645579
 
       NA
 
           NA
  
 
  
 19
 
   9731681
 
   9731901
 
  ENSEMBL   UCSC 
 
  221
 
  4
 
 19802
 
 gene
 
 0.009767
 
 0.0137445
 
 Not queried
 
  0.6643357
 
       NA
 
           NA
  
 
  
 4
 
 103748367
 
 103748674
 
  ENSEMBL   UCSC 
 
  308
 
  6
 
 30521
 
 tss
 
 0.002835
 
 0.0029594
 
 Not queried
 
  0.6642959
 
       NA
 
           NA
  
 
  
 11
 
  68611260
 
  68611596
 
  ENSEMBL   UCSC 
 
  337
 
  4
 
 8579
 
 island
 
 0.014890
 
 0.0259570
 
 Not queried
 
  0.6642787
 
       NA
 
           NA
  
 
  
 1
 
 229567914
 
 229568467
 
  ENSEMBL   UCSC 
 
  554
 
  5
 
 1064
 
 gene;island
 
 0.531568
 
 0.4314209
 
 Not queried
 
  0.6642301
 
       NA
 
           NA
  
 
  
 3
 
  52444617
 
  52444833
 
  ENSEMBL   UCSC 
 
  217
 
  6
 
 27818
 
 gene;tss;island
 
 0.004211
 
 0.0087061
 
 Not queried
 
  0.6641287
 
       NA
 
           NA
  
 
  
 1
 
   1550479
 
   1550886
 
  ENSEMBL   UCSC 
 
  408
 
  9
 
 1158
 
 tss;island
 
 0.003970
 
 0.0059840
 
 Not queried
 
  0.6640905
 
       NA
 
           NA
  
 
  
 1
 
    901685
 
    902025
 
  ENSEMBL   UCSC 
 
  341
 
  6
 
 2387
 
 island
 
 0.015453
 
 0.0142578
 
 Not queried
 
  0.6640618
 
       NA
 
           NA
  
 
  
 12
 
 106480546
 
 106480771
 
  ENSEMBL   UCSC 
 
  226
 
  4
 
 9277
 
 gene
 
 0.669273
 
 0.8257654
 
 Not queried
 
  0.6638891
 
       NA
 
           NA
  
 
  
 1
 
 165414491
 
 165414806
 
  ENSEMBL   UCSC 
 
  316
 
  7
 
 2065
 
 tss
 
 0.009723
 
 0.0061233
 
 Not queried
 
  0.6638295
 
       NA
 
           NA
  
 
  
 11
 
  64794610
 
  64795040
 
  ENSEMBL   UCSC 
 
  431
 
 10
 
 8433
 
 island
 
 0.031025
 
 0.0180923
 
 Not queried
 
  0.6638170
 
       NA
 
           NA
  
 
  
 21
 
  46875142
 
  46875433
 
  ENSEMBL   UCSC 
 
  292
 
  5
 
 26468
 
 gene;island
 
 0.439458
 
 0.5539328
 
 Not queried
 
  0.6637258
 
       NA
 
           NA
  
 
  
 5
 
  34656553
 
  34656566
 
  ENSEMBL   UCSC 
 
   14
 
  4
 
 32012
 
 tss
 
 0.011146
 
 0.0080340
 
 Not queried
 
  0.6636593
 
       NA
 
           NA
  
 
  
 2
 
 242878496
 
 242878811
 
  ENSEMBL   UCSC 
 
  316
 
  4
 
 25317
 
 island
 
 0.964877
 
 0.9772433
 
 Not queried
 
  0.6636461
 
       NA
 
           NA
  
 
  
 1
 
 156783633
 
 156784152
 
  ENSEMBL   UCSC 
 
  520
 
  6
 
 831
 
 gene;island
 
 0.073285
 
 0.0411191
 
 Not queried
 
  0.6636138
 
       NA
 
           NA
  
 
  
 11
 
  68437736
 
  68438018
 
  ENSEMBL   UCSC 
 
  283
 
  4
 
 8575
 
 island
 
 0.888247
 
 0.9274584
 
 Not queried
 
  0.6635811
 
       NA
 
           NA
  
 
  
 17
 
  13972896
 
  13973192
 
  ENSEMBL   UCSC 
 
  297
 
  4
 
 16448
 
 gene
 
 0.021341
 
 0.0176820
 
 Not queried
 
  0.6634775
 
       NA
 
           NA
  
 
  
 2
 
 136634095
 
 136634366
 
  ENSEMBL   UCSC 
 
  272
 
  8
 
 23647
 
 tss
 
 0.002525
 
 0.0024119
 
 Not queried
 
  0.6633912
 
       NA
 
           NA
  
 
  
 1
 
 235530554
 
 235530719
 
  ENSEMBL   UCSC 
 
  166
 
  5
 
 2334
 
 tss
 
 0.006432
 
 0.0036644
 
 Not queried
 
  0.6633219
 
       NA
 
           NA
  
 
  
 7
 
  87849494
 
  87850031
 
  ENSEMBL   UCSC 
 
  538
 
 12
 
 37078
 
 gene;tss;island
 
 0.006035
 
 0.0074592
 
 Not queried
 
  0.6632685
 
       NA
 
           NA
  
 
  
 14
 
  90798288
 
  90798568
 
  ENSEMBL   UCSC 
 
  281
 
  7
 
 12554
 
 tss
 
 0.010970
 
 0.0112733
 
 Not queried
 
  0.6629091
 
       NA
 
           NA
  
 
  
 7
 
 150019955
 
 150020269
 
  ENSEMBL   UCSC 
 
  315
 
  9
 
 37974
 
 tss
 
 0.024728
 
 0.0090574
 
 Not queried
 
  0.6628614
 
       NA
 
           NA
  
 
  
 8
 
 145111298
 
 145111516
 
  ENSEMBL   UCSC 
 
  219
 
  4
 
 39508
 
 gene;island
 
 0.502463
 
 0.6132785
 
 Not queried
 
  0.6628286
 
       NA
 
           NA
  
 
  
 11
 
 111473054
 
 111473380
 
  ENSEMBL   UCSC 
 
  327
 
  4
 
 8803
 
 island
 
 0.001495
 
 0.0022555
 
 Not queried
 
  0.6627792
 
       NA
 
           NA
  
 
  
 2
 
   2754829
 
   2755386
 
  ENSEMBL   UCSC 
 
  558
 
  5
 
 24024
 
 island
 
 0.942030
 
 0.9639117
 
 Not queried
 
  0.6627747
 
       NA
 
           NA
  
 
  
 2
 
  71454252
 
  71454542
 
  ENSEMBL   UCSC 
 
  291
 
  9
 
 23428
 
 tss;island
 
 0.006812
 
 0.0041193
 
 Not queried
 
  0.6627389
 
       NA
 
           NA
  
 
  
 21
 
  35287829
 
  35288546
 
  ENSEMBL   UCSC 
 
  718
 
  8
 
 26657
 
 island
 
 0.017682
 
 0.0098631
 
 Not queried
 
  0.6627282
 
       NA
 
           NA
  
 
  
 8
 
 143535437
 
 143535891
 
  ENSEMBL   UCSC 
 
  455
 
  5
 
 40634
 
 island
 
 0.865500
 
 0.9396807
 
 Not queried
 
  0.6625914
 
       NA
 
           NA
  
 
  
 11
 
  65337543
 
  65338038
 
  ENSEMBL   UCSC 
 
  496
 
  6
 
 8464
 
 island
 
 0.005529
 
 0.0062273
 
 Not queried
 
  0.6625174
 
       NA
 
           NA
  
 
  
 19
 
  36450121
 
  36450528
 
  ENSEMBL   UCSC 
 
  408
 
  4
 
 21929
 
 island
 
 0.082737
 
 0.0962648
 
 Not queried
 
  0.6624836
 
       NA
 
           NA
  
 
  
 13
 
  48878466
 
  48878736
 
  ENSEMBL   UCSC 
 
  271
 
  4
 
 11160
 
 gene;island
 
 0.002015
 
 0.0025515
 
 Not queried
 
  0.6624589
 
       NA
 
           NA
  
 
  
 11
 
   6261411
 
   6261798
 
  ENSEMBL   UCSC 
 
  388
 
  5
 
 6486
 
 gene;island
 
 0.576277
 
 0.6818891
 
 Not queried
 
  0.6624475
 
       NA
 
           NA
  
 
  
 11
 
  67815010
 
  67815326
 
  ENSEMBL   UCSC 
 
  317
 
  5
 
 6769
 
 gene
 
 0.975362
 
 0.9853780
 
 Not queried
 
  0.6624283
 
       NA
 
           NA
  
 
  
 6
 
 157745216
 
 157745382
 
  ENSEMBL   UCSC 
 
  167
 
  4
 
 36591
 
 island
 
 0.054231
 
 0.0660950
 
 Not queried
 
  0.6623810
 
       NA
 
           NA
  
 
  
 19
 
  15838756
 
  15839006
 
  ENSEMBL   UCSC 
 
  251
 
  4
 
 21710
 
 island
 
 0.309607
 
 0.4350710
 
 Not queried
 
  0.6623195
 
       NA
 
           NA
  
 
  
 11
 
 105480771
 
 105480979
 
  ENSEMBL   UCSC 
 
  209
 
  5
 
 7654
 
 tss;island
 
 0.037754
 
 0.0631742
 
 Not queried
 
  0.6622759
 
       NA
 
           NA
  
 
  
 17
 
  74723362
 
  74723576
 
  ENSEMBL   UCSC 
 
  215
 
  4
 
 16795
 
 gene;tss;island
 
 0.022609
 
 0.0191919
 
 Not queried
 
  0.6622274
 
       NA
 
           NA
  
 
  
 6
 
  26240307
 
  26240579
 
  ENSEMBL   UCSC 
 
  273
 
  5
 
 34699
 
 tss;island
 
 0.007043
 
 0.0054882
 
 Not queried
 
  0.6620456
 
       NA
 
           NA
  
 
  
 8
 
  42128498
 
  42128690
 
  ENSEMBL   UCSC 
 
  193
 
  5
 
 39677
 
 tss
 
 0.007713
 
 0.0045852
 
 Not queried
 
  0.6619642
 
       NA
 
           NA
  
 
  
 16
 
  88551191
 
  88551489
 
  ENSEMBL   UCSC 
 
  299
 
  5
 
 14904
 
 gene;island
 
 0.437878
 
 0.6805042
 
 Not queried
 
  0.6619045
 
       NA
 
           NA
  
 
  
 1
 
 209799191
 
 209799353
 
  ENSEMBL   UCSC 
 
  163
 
  4
 
 984
 
 gene;island
 
 0.934941
 
 0.9295825
 
 Not queried
 
  0.6618176
 
       NA
 
           NA
  
 
  
 19
 
  17581060
 
  17581292
 
  ENSEMBL   UCSC 
 
  233
 
  7
 
 20592
 
 tss
 
 0.001641
 
 0.0020022
 
 Not queried
 
  0.6617732
 
       NA
 
           NA
  
 
  
 8
 
  94767133
 
  94767372
 
  ENSEMBL   UCSC 
 
  240
 
  4
 
 39360
 
 gene
 
 0.001627
 
 0.0041944
 
 Not queried
 
  0.6617594
 
       NA
 
           NA
  
 
  
 8
 
  22408966
 
  22409131
 
  ENSEMBL   UCSC 
 
  166
 
  5
 
 39587
 
 tss
 
 0.020223
 
 0.0363364
 
 Not queried
 
  0.6615632
 
       NA
 
           NA
  
 
  
 14
 
  29235894
 
  29236535
 
  ENSEMBL   UCSC 
 
  642
 
 14
 
 12757
 
 island
 
 0.266749
 
 0.1451401
 
 Not queried
 
  0.6614652
 
       NA
 
           NA
  
 
  
 1
 
  74663583
 
  74664160
 
  ENSEMBL   UCSC 
 
  578
 
 11
 
 1695
 
 tss;island
 
 0.007878
 
 0.0034572
 
 Not queried
 
  0.6614463
 
       NA
 
           NA
  
 
  
 1
 
 155904276
 
 155904439
 
  ENSEMBL   UCSC 
 
  164
 
  6
 
 2006
 
 tss
 
 0.013435
 
 0.0075965
 
 Not queried
 
  0.6613376
 
       NA
 
           NA
  
 
  
 20
 
  60757714
 
  60758083
 
  ENSEMBL   UCSC 
 
  370
 
  9
 
 26314
 
 island
 
 0.006078
 
 0.0027161
 
 Not queried
 
  0.6612654
 
       NA
 
           NA
  
 
  
 8
 
  27472374
 
  27472513
 
  ENSEMBL   UCSC 
 
  140
 
  7
 
 39616
 
 tss
 
 0.029329
 
 0.0363785
 
 Not queried
 
  0.6612508
 
       NA
 
           NA
  
 
  
 7
 
   4347499
 
   4348107
 
  ENSEMBL   UCSC 
 
  609
 
  7
 
 38187
 
 island
 
 0.503530
 
 0.6069788
 
 Not queried
 
  0.6609685
 
       NA
 
           NA
  
 
  
 19
 
  38270207
 
  38270616
 
  ENSEMBL   UCSC 
 
  410
 
  7
 
 20745
 
 tss
 
 0.024466
 
 0.0130303
 
 Not queried
 
  0.6609577
 
       NA
 
           NA
  
 
  
 18
 
  32557882
 
  32558112
 
  ENSEMBL   UCSC 
 
  231
 
  4
 
 19087
 
 gene;tss;island
 
 0.011794
 
 0.0064931
 
 Not queried
 
  0.6608807
 
       NA
 
           NA
  
 
  
 19
 
  39903392
 
  39903678
 
  ENSEMBL   UCSC 
 
  287
 
  6
 
 20771
 
 tss;island
 
 0.009249
 
 0.0037160
 
 Not queried
 
  0.6608655
 
       NA
 
           NA
  
 
  
 5
 
 112630623
 
 112631052
 
  ENSEMBL   UCSC 
 
  430
 
  7
 
 31715
 
 gene;tss;island
 
 0.007161
 
 0.0084340
 
 Not queried
 
  0.6606790
 
       NA
 
           NA
  
 
  
 1
 
 119543057
 
 119543336
 
  ENSEMBL   UCSC 
 
  280
 
  4
 
 3660
 
 island
 
 0.162870
 
 0.0903226
 
 Not queried
 
  0.6605908
 
       NA
 
           NA
  
 
  
 4
 
 108853204
 
 108853489
 
  ENSEMBL   UCSC 
 
  286
 
  4
 
 30083
 
 gene;island
 
 0.030088
 
 0.0151256
 
 Not queried
 
  0.6604768
 
       NA
 
           NA
  
 
  
 2
 
  88991205
 
  88991605
 
  ENSEMBL   UCSC 
 
  401
 
  5
 
 22775
 
 gene
 
 0.007439
 
 0.0020377
 
 Not queried
 
  0.6604763
 
       NA
 
           NA
  
 
  
 2
 
 113522299
 
 113522573
 
  ENSEMBL   UCSC 
 
  275
 
  7
 
 23580
 
 tss
 
 0.008109
 
 0.0056890
 
 Not queried
 
  0.6603172
 
       NA
 
           NA
  
 
  
 7
 
 158045980
 
 158046358
 
  ENSEMBL   UCSC 
 
  379
 
  6
 
 37362
 
 gene
 
 0.436290
 
 0.4717352
 
 Not queried
 
  0.6602974
 
       NA
 
           NA
  
 
  
 12
 
  54132086
 
  54132568
 
  ENSEMBL   UCSC 
 
  483
 
  6
 
 10473
 
 island
 
 0.031999
 
 0.0295643
 
 Not queried
 
  0.6602825
 
       NA
 
           NA
  
 
  
 18
 
  11751343
 
  11751556
 
  ENSEMBL   UCSC 
 
  214
 
  5
 
 19183
 
 tss
 
 0.202762
 
 0.3827509
 
 Not queried
 
  0.6601535
 
       NA
 
           NA
  
 
  
 6
 
  30685461
 
  30685744
 
  ENSEMBL   UCSC 
 
  284
 
  7
 
 34784
 
 tss
 
 0.005529
 
 0.0030079
 
 Not queried
 
  0.6601053
 
       NA
 
           NA
  
 
  
 2
 
 153032612
 
 153032884
 
  ENSEMBL   UCSC 
 
  273
 
  9
 
 23664
 
 tss
 
 0.009508
 
 0.0085323
 
 Not queried
 
  0.6601010
 
       NA
 
           NA
  
 
  
 2
 
 153032369
 
 153032884
 
  ENSEMBL   UCSC 
 
  516
 
 11
 
 24788
 
 island
 
 0.008978
 
 0.0085323
 
 Not queried
 
  0.6601010
 
       NA
 
           NA
  
 
  
 16
 
   2334163
 
   2334346
 
  ENSEMBL   UCSC 
 
  184
 
  4
 
 14604
 
 gene;island
 
 0.630969
 
 0.6788792
 
 Not queried
 
  0.6599744
 
       NA
 
           NA
  
 
  
 2
 
  48982509
 
  48982832
 
  ENSEMBL   UCSC 
 
  324
 
  4
 
 22674
 
 gene
 
 0.372807
 
 0.1311634
 
 Not queried
 
  0.6599094
 
       NA
 
           NA
  
 
  
 2
 
 242794854
 
 242795082
 
  ENSEMBL   UCSC 
 
  229
 
  5
 
 23148
 
 gene;island
 
 0.919868
 
 0.9851105
 
 Not queried
 
  0.6598310
 
       NA
 
           NA
  
 
  
 11
 
 121028592
 
 121028969
 
  ENSEMBL   UCSC 
 
  378
 
  4
 
 6944
 
 gene;island
 
 0.941681
 
 0.9405133
 
 Not queried
 
  0.6597981
 
       NA
 
           NA
  
 
  
 16
 
   2653222
 
   2653626
 
  ENSEMBL   UCSC 
 
  405
 
  9
 
 15610
 
 island
 
 0.008414
 
 0.0076578
 
 Not queried
 
  0.6596822
 
       NA
 
           NA
  
 
  
 5
 
   1008828
 
   1009204
 
  ENSEMBL   UCSC 
 
  377
 
  5
 
 32600
 
 island
 
 0.008356
 
 0.0080830
 
 Not queried
 
  0.6596696
 
       NA
 
           NA
  
 
  
 5
 
 134093972
 
 134094454
 
  ENSEMBL   UCSC 
 
  483
 
 10
 
 32298
 
 tss
 
 0.008681
 
 0.0099370
 
 Not queried
 
  0.6594514
 
       NA
 
           NA
  
 
  
 5
 
 134093972
 
 134094672
 
  ENSEMBL   UCSC 
 
  701
 
 12
 
 33123
 
 island
 
 0.005746
 
 0.0062638
 
 Not queried
 
  0.6594514
 
       NA
 
           NA
  
 
  
 21
 
  45789122
 
  45789379
 
  ENSEMBL   UCSC 
 
  258
 
  4
 
 26455
 
 gene;island
 
 0.962000
 
 0.9167755
 
 Not queried
 
  0.6593584
 
       NA
 
           NA
  
 
  
 17
 
  79829378
 
  79829466
 
  ENSEMBL   UCSC 
 
   89
 
  4
 
 17751
 
 tss
 
 0.006019
 
 0.0109217
 
 Not queried
 
  0.6593203
 
       NA
 
           NA
  
 
  
 15
 
  56535304
 
  56536071
 
  ENSEMBL   UCSC 
 
  768
 
  6
 
 14163
 
 island
 
 0.016322
 
 0.0143125
 
 Not queried
 
  0.6592976
 
       NA
 
           NA
  
 
  
 1
 
 209825672
 
 209825943
 
  ENSEMBL   UCSC 
 
  272
 
  7
 
 2230
 
 tss
 
 0.937622
 
 0.9658398
 
 Not queried
 
  0.6592274
 
       NA
 
           NA
  
 
  
 11
 
 130786230
 
 130786720
 
  ENSEMBL   UCSC 
 
  491
 
  8
 
 8964
 
 island
 
 0.012309
 
 0.0146020
 
 Not queried
 
  0.6590842
 
       NA
 
           NA
  
 
  
 3
 
  25469860
 
  25469991
 
  ENSEMBL   UCSC 
 
  132
 
  6
 
 27705
 
 gene
 
 0.025904
 
 0.0366459
 
 Not queried
 
  0.6589984
 
       NA
 
           NA
  
 
  
 17
 
  66254884
 
  66255265
 
  ENSEMBL   UCSC 
 
  382
 
  6
 
 17606
 
 tss
 
 0.016745
 
 0.0174394
 
 Not queried
 
  0.6589322
 
       NA
 
           NA
  
 
  
 3
 
 140660335
 
 140660642
 
  ENSEMBL   UCSC 
 
  308
 
  8
 
 28635
 
 tss
 
 0.003717
 
 0.0054689
 
 Not queried
 
  0.6586427
 
       NA
 
           NA
  
 
  
 10
 
  43818243
 
  43818911
 
  ENSEMBL   UCSC 
 
  669
 
  8
 
 5693
 
 island
 
 0.043722
 
 0.1810563
 
 Not queried
 
  0.6584585
 
       NA
 
           NA
  
 
  
 12
 
 111021178
 
 111021296
 
  ENSEMBL   UCSC 
 
  119
 
  6
 
 10006
 
 tss;island
 
 0.006766
 
 0.0056348
 
 Not queried
 
  0.6584466
 
       NA
 
           NA
  
 
  
 2
 
 236443773
 
 236444284
 
  ENSEMBL   UCSC 
 
  512
 
  7
 
 23080
 
 gene;island
 
 0.699789
 
 0.8574116
 
 Not queried
 
  0.6583511
 
       NA
 
           NA
  
 
  
 5
 
 172744616
 
 172744941
 
  ENSEMBL   UCSC 
 
  326
 
  5
 
 31905
 
 gene
 
 0.803489
 
 0.9735435
 
 Not queried
 
  0.6583498
 
       NA
 
           NA
  
 
  
 11
 
  93822022
 
  93822294
 
  ENSEMBL   UCSC 
 
  273
 
  4
 
 6863
 
 gene;island
 
 0.374117
 
 0.6294844
 
 Not queried
 
  0.6583279
 
       NA
 
           NA
  
 
  
 14
 
  24563410
 
  24563578
 
  ENSEMBL   UCSC 
 
  169
 
  4
 
 12725
 
 island
 
 0.016449
 
 0.0155289
 
 Not queried
 
  0.6581025
 
       NA
 
           NA
  
 
  
 8
 
  38964784
 
  38965492
 
  ENSEMBL   UCSC 
 
  709
 
 11
 
 40239
 
 island
 
 0.116187
 
 0.2204894
 
 Not queried
 
  0.6580761
 
       NA
 
           NA
  
 
  
 5
 
  61601529
 
  61601647
 
  ENSEMBL   UCSC 
 
  119
 
  4
 
 32084
 
 tss;island
 
 0.013147
 
 0.0247121
 
 Not queried
 
  0.6580205
 
       NA
 
           NA
  
 
  
 11
 
  70858237
 
  70858695
 
  ENSEMBL   UCSC 
 
  459
 
  6
 
 6799
 
 gene
 
 0.527696
 
 0.5791283
 
 Not queried
 
  0.6579693
 
       NA
 
           NA
  
 
  
 6
 
  33378912
 
  33379330
 
  ENSEMBL   UCSC 
 
  419
 
 10
 
 34278
 
 gene;island
 
 0.007593
 
 0.0053493
 
 Not queried
 
  0.6579044
 
       NA
 
           NA
  
 
  
 2
 
 239930734
 
 239931011
 
  ENSEMBL   UCSC 
 
  278
 
  4
 
 25249
 
 island
 
 0.491051
 
 0.5710837
 
 Not queried
 
  0.6576611
 
       NA
 
           NA
  
 
  
 7
 
  21582745
 
  21582834
 
  ENSEMBL   UCSC 
 
   90
 
  4
 
 38297
 
 island
 
 0.014227
 
 0.0203088
 
 Not queried
 
  0.6576176
 
       NA
 
           NA
  
 
  
 10
 
  50747084
 
  50747328
 
  ENSEMBL   UCSC 
 
  245
 
  5
 
 5734
 
 island
 
 0.005341
 
 0.0038670
 
 Not queried
 
  0.6575909
 
       NA
 
           NA
  
 
  
 3
 
  69101222
 
  69101684
 
  ENSEMBL   UCSC 
 
  463
 
  8
 
 29270
 
 island
 
 0.004825
 
 0.0030780
 
 Not queried
 
  0.6575687
 
       NA
 
           NA
  
 
  
 11
 
 116658528
 
 116658887
 
  ENSEMBL   UCSC 
 
  360
 
  8
 
 8835
 
 island
 
 0.010190
 
 0.0105574
 
 Not queried
 
  0.6572815
 
       NA
 
           NA
  
 
  
 12
 
  14956526
 
  14956701
 
  ENSEMBL   UCSC 
 
  176
 
  4
 
 9067
 
 gene
 
 0.001174
 
 0.0013281
 
 Not queried
 
  0.6572637
 
       NA
 
           NA
  
 
  
 19
 
  12721341
 
  12721610
 
  ENSEMBL   UCSC 
 
  270
 
  8
 
 19857
 
 gene
 
 0.008150
 
 0.0129735
 
 Not queried
 
  0.6571960
 
       NA
 
           NA
  
 
  
 16
 
  75657287
 
  75657395
 
  ENSEMBL   UCSC 
 
  109
 
  6
 
 15365
 
 tss
 
 0.012596
 
 0.0075620
 
 Not queried
 
  0.6569186
 
       NA
 
           NA
  
 
  
 4
 
  11430552
 
  11430908
 
  ENSEMBL   UCSC 
 
  357
 
  7
 
 30302
 
 tss
 
 0.032265
 
 0.0385494
 
 Not queried
 
  0.6568944
 
       NA
 
           NA
  
 
  
 14
 
  71067695
 
  71067802
 
  ENSEMBL   UCSC 
 
  108
 
  4
 
 12487
 
 tss
 
 0.003718
 
 0.0025587
 
 Not queried
 
  0.6567742
 
       NA
 
           NA
  
 
  
 4
 
 156587884
 
 156588387
 
  ENSEMBL   UCSC 
 
  504
 
  8
 
 30159
 
 gene;tss;island
 
 0.010315
 
 0.0051441
 
 Not queried
 
  0.6567686
 
       NA
 
           NA
  
 
  
 3
 
 130465237
 
 130465895
 
  ENSEMBL   UCSC 
 
  659
 
  8
 
 29475
 
 island
 
 0.005854
 
 0.0034251
 
 Not queried
 
  0.6566866
 
       NA
 
           NA
  
 
  
 22
 
  30639611
 
  30639979
 
  ENSEMBL   UCSC 
 
  369
 
  4
 
 26835
 
 gene;island
 
 0.681291
 
 0.7262460
 
 Not queried
 
  0.6565358
 
       NA
 
           NA
  
 
  
 11
 
  17740887
 
  17741243
 
  ENSEMBL   UCSC 
 
  357
 
  5
 
 8103
 
 island
 
 0.323815
 
 0.1568352
 
 Not queried
 
  0.6564911
 
       NA
 
           NA
  
 
  
 15
 
  72409903
 
  72410226
 
  ENSEMBL   UCSC 
 
  324
 
  4
 
 13405
 
 gene;tss;island
 
 0.002463
 
 0.0034292
 
 Not queried
 
  0.6564094
 
       NA
 
           NA
  
 
  
 22
 
  24373134
 
  24373618
 
  ENSEMBL   UCSC 
 
  485
 
  4
 
 26811
 
 gene
 
 0.038196
 
 0.0197598
 
 Not queried
 
  0.6563775
 
       NA
 
           NA
  
 
  
 7
 
  84815088
 
  84815543
 
  ENSEMBL   UCSC 
 
  456
 
  5
 
 38628
 
 island
 
 0.006349
 
 0.0035532
 
 Not queried
 
  0.6563649
 
       NA
 
           NA
  
 
  
 17
 
  56832804
 
  56833425
 
  ENSEMBL   UCSC 
 
  622
 
 13
 
 18605
 
 island
 
 0.024650
 
 0.0336143
 
 Not queried
 
  0.6563288
 
       NA
 
           NA
  
 
  
 17
 
  41477271
 
  41477468
 
  ENSEMBL   UCSC 
 
  198
 
  4
 
 16597
 
 gene;island
 
 0.938344
 
 0.9705512
 
 Not queried
 
  0.6561433
 
       NA
 
           NA
  
 
  
 15
 
  55581976
 
  55582570
 
  ENSEMBL   UCSC 
 
  595
 
  7
 
 14156
 
 island
 
 0.020218
 
 0.0133314
 
 Not queried
 
  0.6561396
 
       NA
 
           NA
  
 
  
 22
 
  28073992
 
  28074146
 
  ENSEMBL   UCSC 
 
  155
 
  4
 
 27382
 
 island
 
 0.704134
 
 0.7282905
 
 Not queried
 
  0.6560562
 
       NA
 
           NA
  
 
  
 10
 
  27150052
 
  27150529
 
  ENSEMBL   UCSC 
 
  478
 
  8
 
 4996
 
 tss
 
 0.024441
 
 0.0085037
 
 Not queried
 
  0.6560262
 
       NA
 
           NA
  
 
  
 1
 
 227915846
 
 227916120
 
  ENSEMBL   UCSC 
 
  275
 
  6
 
 2294
 
 tss
 
 0.022119
 
 0.0338374
 
 Not queried
 
  0.6559313
 
       NA
 
           NA
  
 
  
 1
 
 166845374
 
 166845621
 
  ENSEMBL   UCSC 
 
  248
 
  4
 
 870
 
 gene
 
 0.012073
 
 0.0103879
 
 Not queried
 
  0.6557990
 
       NA
 
           NA
  
 
  
 6
 
  32370520
 
  32370879
 
  ENSEMBL   UCSC 
 
  360
 
  7
 
 34108
 
 gene
 
 0.895145
 
 0.9325975
 
 Not queried
 
  0.6554252
 
       NA
 
           NA
  
 
  
 13
 
  47012103
 
  47012543
 
  ENSEMBL   UCSC 
 
  441
 
  4
 
 11715
 
 island
 
 0.689434
 
 0.8713476
 
 Not queried
 
  0.6553992
 
       NA
 
           NA
  
 
  
 22
 
  36236649
 
  36236760
 
  ENSEMBL   UCSC 
 
  112
 
  4
 
 26854
 
 gene;tss
 
 0.016357
 
 0.0034089
 
 Not queried
 
  0.6553960
 
       NA
 
           NA
  
 
  
 14
 
  45722617
 
  45722745
 
  ENSEMBL   UCSC 
 
  129
 
  6
 
 12374
 
 tss
 
 0.016837
 
 0.0056484
 
 Not queried
 
  0.6553809
 
       NA
 
           NA
  
 
  
 1
 
 115632236
 
 115632727
 
  ENSEMBL   UCSC 
 
  492
 
  8
 
 1857
 
 tss
 
 0.011412
 
 0.0120617
 
 Not queried
 
  0.6553622
 
       NA
 
           NA
  
 
  
 1
 
 109968980
 
 109969188
 
  ENSEMBL   UCSC 
 
  209
 
  8
 
 3560
 
 island
 
 0.004647
 
 0.0068413
 
 Not queried
 
  0.6552190
 
       NA
 
           NA
  
 
  
 20
 
  18487771
 
  18488185
 
  ENSEMBL   UCSC 
 
  415
 
  7
 
 25618
 
 tss
 
 0.005296
 
 0.0056751
 
 Not queried
 
  0.6551859
 
       NA
 
           NA
  
 
  
 20
 
  18487771
 
  18488366
 
  ENSEMBL   UCSC 
 
  596
 
  9
 
 26028
 
 island
 
 0.005296
 
 0.0056751
 
 Not queried
 
  0.6551859
 
       NA
 
           NA
  
 
  
 2
 
 115920474
 
 115920825
 
  ENSEMBL   UCSC 
 
  352
 
  4
 
 22839
 
 gene;island
 
 0.021479
 
 0.0197006
 
 Not queried
 
  0.6551509
 
       NA
 
           NA
  
 
  
 19
 
  36103707
 
  36103878
 
  ENSEMBL   UCSC 
 
  172
 
  4
 
 19986
 
 gene
 
 0.009670
 
 0.0087018
 
 Not queried
 
  0.6549809
 
       NA
 
           NA
  
 
  
 15
 
  41061984
 
  41062273
 
  ENSEMBL   UCSC 
 
  290
 
  6
 
 13296
 
 gene
 
 0.015078
 
 0.0048681
 
 Not queried
 
  0.6549592
 
       NA
 
           NA
  
 
  
 8
 
 142192468
 
 142192718
 
  ENSEMBL   UCSC 
 
  251
 
  5
 
 39450
 
 gene
 
 0.019080
 
 0.0293310
 
 Not queried
 
  0.6549590
 
       NA
 
           NA
  
 
  
 1
 
 153935719
 
 153936275
 
  ENSEMBL   UCSC 
 
  557
 
  6
 
 770
 
 gene;island
 
 0.011195
 
 0.0095918
 
 Not queried
 
  0.6549582
 
       NA
 
           NA
  
 
  
 13
 
  28366427
 
  28366814
 
  ENSEMBL   UCSC 
 
  388
 
  9
 
 11630
 
 island
 
 0.047467
 
 0.0736952
 
 Not queried
 
  0.6547612
 
       NA
 
           NA
  
 
  
 19
 
  47290586
 
  47290716
 
  ENSEMBL   UCSC 
 
  131
 
  4
 
 20898
 
 tss
 
 0.020552
 
 0.0098586
 
 Not queried
 
  0.6547377
 
       NA
 
           NA
  
 
  
 14
 
  85998439
 
  85998669
 
  ENSEMBL   UCSC 
 
  231
 
  4
 
 12163
 
 gene;island
 
 0.122548
 
 0.1112467
 
 Not queried
 
  0.6546457
 
       NA
 
           NA
  
 
  
 19
 
   1675848
 
   1676136
 
  ENSEMBL   UCSC 
 
  289
 
  4
 
 21237
 
 island
 
 0.018992
 
 0.0492638
 
 Not queried
 
  0.6546258
 
       NA
 
           NA
  
 
  
 8
 
  79427993
 
  79428325
 
  ENSEMBL   UCSC 
 
  333
 
  4
 
 39778
 
 tss
 
 0.011042
 
 0.0080800
 
 Not queried
 
  0.6544742
 
       NA
 
           NA
  
 
  
 14
 
  33403060
 
  33403226
 
  ENSEMBL   UCSC 
 
  167
 
  4
 
 12771
 
 island
 
 0.010642
 
 0.0059436
 
 Not queried
 
  0.6543472
 
       NA
 
           NA
  
 
  
 2
 
 127414802
 
 127415063
 
  ENSEMBL   UCSC 
 
  262
 
  4
 
 22854
 
 gene;island
 
 0.009597
 
 0.0145045
 
 Not queried
 
  0.6537920
 
       NA
 
           NA
  
 
  
 3
 
 195270315
 
 195270352
 
  ENSEMBL   UCSC 
 
   38
 
  4
 
 28804
 
 tss;island
 
 0.002182
 
 0.0023217
 
 Not queried
 
  0.6537892
 
       NA
 
           NA
  
 
  
 16
 
  69760119
 
  69760966
 
  ENSEMBL   UCSC 
 
  848
 
 11
 
 16062
 
 island
 
 0.006271
 
 0.0065945
 
 Not queried
 
  0.6537814
 
       NA
 
           NA
  
 
  
 17
 
   1082526
 
   1083491
 
  ENSEMBL   UCSC 
 
  966
 
  9
 
 16310
 
 gene;island
 
 0.013857
 
 0.0144530
 
 Not queried
 
  0.6537780
 
       NA
 
           NA
  
 
  
 19
 
   3595702
 
   3595996
 
  ENSEMBL   UCSC 
 
  295
 
  4
 
 19716
 
 gene;island
 
 0.872485
 
 0.9471572
 
 Not queried
 
  0.6537775
 
       NA
 
           NA
  
 
  
 5
 
  34656553
 
  34656625
 
  ENSEMBL   UCSC 
 
   73
 
  5
 
 31580
 
 gene;island
 
 0.014799
 
 0.0089693
 
 Not queried
 
  0.6537228
 
       NA
 
           NA
  
 
  
 7
 
   1535471
 
   1535941
 
  ENSEMBL   UCSC 
 
  471
 
  4
 
 36758
 
 gene;island
 
 0.962014
 
 0.8598063
 
 Not queried
 
  0.6536943
 
       NA
 
           NA
  
 
  
 17
 
  26662113
 
  26662842
 
  ENSEMBL   UCSC 
 
  730
 
 11
 
 16494
 
 gene;tss;island
 
 0.008410
 
 0.0041877
 
 Not queried
 
  0.6536697
 
       NA
 
           NA
  
 
  
 17
 
  57642628
 
  57642873
 
  ENSEMBL   UCSC 
 
  246
 
  8
 
 17546
 
 tss
 
 0.970368
 
 0.6004761
 
 Not queried
 
  0.6535150
 
       NA
 
           NA
  
 
  
 17
 
  57642628
 
  57643004
 
  ENSEMBL   UCSC 
 
  377
 
 10
 
 18611
 
 island
 
 0.923066
 
 0.3401981
 
 Not queried
 
  0.6535150
 
       NA
 
           NA
  
 
  
 1
 
 179560700
 
 179561111
 
  ENSEMBL   UCSC 
 
  412
 
  6
 
 4000
 
 island
 
 0.055035
 
 0.1010252
 
 Not queried
 
  0.6535034
 
       NA
 
           NA
  
 
  
 6
 
  31497233
 
  31497756
 
  ENSEMBL   UCSC 
 
  524
 
  4
 
 33888
 
 gene
 
 0.945942
 
 0.9382883
 
 Not queried
 
  0.6534775
 
       NA
 
           NA
  
 
  
 5
 
  52405349
 
  52405518
 
  ENSEMBL   UCSC 
 
  170
 
  6
 
 31612
 
 gene
 
 0.001351
 
 0.0025905
 
 Not queried
 
  0.6534675
 
       NA
 
           NA
  
 
  
 14
 
 101192190
 
 101193038
 
  ENSEMBL   UCSC 
 
  849
 
 16
 
 12607
 
 tss;island
 
 0.630117
 
 0.7191833
 
 Not queried
 
  0.6534653
 
       NA
 
           NA
  
 
  
 4
 
  77819534
 
  77819792
 
  ENSEMBL   UCSC 
 
  259
 
  4
 
 30450
 
 tss;island
 
 0.010632
 
 0.0101913
 
 Not queried
 
  0.6534360
 
       NA
 
           NA
  
 
  
 17
 
  62915551
 
  62915920
 
  ENSEMBL   UCSC 
 
  370
 
  4
 
 17589
 
 tss;island
 
 0.009476
 
 0.0104906
 
 Not queried
 
  0.6534055
 
       NA
 
           NA
  
 
  
 10
 
  28035631
 
  28035894
 
  ENSEMBL   UCSC 
 
  264
 
  7
 
 5005
 
 tss;island
 
 0.040505
 
 0.0315356
 
 Not queried
 
  0.6532883
 
       NA
 
           NA
  
 
  
 4
 
 101111147
 
 101111927
 
  ENSEMBL   UCSC 
 
  781
 
  9
 
 31203
 
 island
 
 0.014221
 
 0.0174509
 
 Not queried
 
  0.6532616
 
       NA
 
           NA
  
 
  
 8
 
 109260899
 
 109261275
 
  ENSEMBL   UCSC 
 
  377
 
 10
 
 40531
 
 island
 
 0.008518
 
 0.0047747
 
 Not queried
 
  0.6531714
 
       NA
 
           NA
  
 
  
 3
 
 185826924
 
 185827054
 
  ENSEMBL   UCSC 
 
  131
 
  7
 
 28770
 
 tss;island
 
 0.020070
 
 0.0187283
 
 Not queried
 
  0.6529863
 
       NA
 
           NA
  
 
  
 7
 
 155436277
 
 155437209
 
  ENSEMBL   UCSC 
 
  933
 
 13
 
 39073
 
 island
 
 0.028379
 
 0.0295466
 
 Not queried
 
  0.6528057
 
       NA
 
           NA
  
 
  
 1
 
  16163479
 
  16164122
 
  ENSEMBL   UCSC 
 
  644
 
  7
 
 252
 
 gene;island
 
 0.012985
 
 0.0201291
 
 Not queried
 
  0.6527823
 
       NA
 
           NA
  
 
  
 3
 
  73674074
 
  73674702
 
  ENSEMBL   UCSC 
 
  629
 
 12
 
 28446
 
 tss
 
 0.512424
 
 0.2727458
 
 Not queried
 
  0.6527618
 
       NA
 
           NA
  
 
  
 2
 
 152118408
 
 152118835
 
  ENSEMBL   UCSC 
 
  428
 
  8
 
 23658
 
 tss;island
 
 0.009545
 
 0.0068130
 
 Not queried
 
  0.6525931
 
       NA
 
           NA
  
 
  
 17
 
  79108051
 
  79108344
 
  ENSEMBL   UCSC 
 
  294
 
  6
 
 16896
 
 gene;tss;island
 
 0.659694
 
 0.7841140
 
 Not queried
 
  0.6525251
 
       NA
 
           NA
  
 
  
 19
 
  45996372
 
  45996644
 
  ENSEMBL   UCSC 
 
  273
 
  6
 
 20099
 
 gene;island
 
 0.023351
 
 0.0384343
 
 Not queried
 
  0.6524852
 
       NA
 
           NA
  
 
  
 16
 
  25026527
 
  25026956
 
  ENSEMBL   UCSC 
 
  430
 
  9
 
 15774
 
 island
 
 0.006985
 
 0.0055999
 
 Not queried
 
  0.6524796
 
       NA
 
           NA
  
 
  
 11
 
  61129269
 
  61129435
 
  ENSEMBL   UCSC 
 
  167
 
  7
 
 7332
 
 tss
 
 0.018511
 
 0.0182018
 
 Not queried
 
  0.6524258
 
       NA
 
           NA
  
 
  
 3
 
 187461796
 
 187462173
 
  ENSEMBL   UCSC 
 
  378
 
  5
 
 28080
 
 gene;island
 
 0.025417
 
 0.0398918
 
 Not queried
 
  0.6524028
 
       NA
 
           NA
  
 
  
 3
 
  27754391
 
  27754675
 
  ENSEMBL   UCSC 
 
  285
 
  5
 
 28947
 
 island
 
 0.018613
 
 0.0117691
 
 Not queried
 
  0.6523766
 
       NA
 
           NA
  
 
  
 15
 
  92936678
 
  92937030
 
  ENSEMBL   UCSC 
 
  353
 
  4
 
 13911
 
 tss;island
 
 0.004895
 
 0.0108196
 
 Not queried
 
  0.6523475
 
       NA
 
           NA
  
 
  
 1
 
  92545888
 
  92546135
 
  ENSEMBL   UCSC 
 
  248
 
  4
 
 633
 
 gene
 
 0.004285
 
 0.0130549
 
 Not queried
 
  0.6522715
 
       NA
 
           NA
  
 
  
 7
 
  34118464
 
  34118935
 
  ENSEMBL   UCSC 
 
  472
 
  5
 
 36961
 
 gene;island
 
 0.322684
 
 0.4281159
 
 Not queried
 
  0.6522243
 
       NA
 
           NA
  
 
  
 22
 
  29168272
 
  29168647
 
  ENSEMBL   UCSC 
 
  376
 
  8
 
 27041
 
 tss
 
 0.009467
 
 0.0098527
 
 Not queried
 
  0.6521752
 
       NA
 
           NA
  
 
  
 1
 
 101361710
 
 101362002
 
  ENSEMBL   UCSC 
 
  293
 
  4
 
 653
 
 gene;island
 
 0.003125
 
 0.0010845
 
 Not queried
 
  0.6518683
 
       NA
 
           NA
  
 
  
 8
 
  67340962
 
  67341108
 
  ENSEMBL   UCSC 
 
  147
 
  5
 
 39741
 
 tss
 
 0.016396
 
 0.0108556
 
 Not queried
 
  0.6518349
 
       NA
 
           NA
  
 
  
 19
 
  53662353
 
  53662723
 
  ENSEMBL   UCSC 
 
  371
 
  8
 
 21024
 
 tss
 
 0.004303
 
 0.0023588
 
 Not queried
 
  0.6517720
 
       NA
 
           NA
  
 
  
 1
 
 150336450
 
 150337482
 
  ENSEMBL   UCSC 
 
 1033
 
 16
 
 3722
 
 island
 
 0.010426
 
 0.0046592
 
 Not queried
 
  0.6516968
 
       NA
 
           NA
  
 
  
 6
 
  30619047
 
  30619242
 
  ENSEMBL   UCSC 
 
  196
 
 11
 
 33794
 
 gene;island
 
 0.785124
 
 0.1950919
 
 Not queried
 
  0.6516408
 
       NA
 
           NA
  
 
  
 1
 
  43854839
 
  43855122
 
  ENSEMBL   UCSC 
 
  284
 
  4
 
 477
 
 gene;tss;island
 
 0.036415
 
 0.0273728
 
 Not queried
 
  0.6514743
 
       NA
 
           NA
  
 
  
 3
 
   9027157
 
   9027576
 
  ENSEMBL   UCSC 
 
  420
 
  4
 
 27649
 
 gene;island
 
 0.634191
 
 0.8322591
 
 Not queried
 
  0.6514592
 
       NA
 
           NA
  
 
  
 1
 
  35250229
 
  35250839
 
  ENSEMBL   UCSC 
 
  611
 
  7
 
 413
 
 gene;island
 
 0.472906
 
 0.7537707
 
 Not queried
 
  0.6514427
 
       NA
 
           NA
  
 
  
 15
 
 100273699
 
 100273828
 
  ENSEMBL   UCSC 
 
  130
 
  8
 
 13925
 
 tss
 
 0.006417
 
 0.0040624
 
 Not queried
 
  0.6513935
 
       NA
 
           NA
  
 
  
 15
 
 100273145
 
 100273828
 
  ENSEMBL   UCSC 
 
  684
 
 12
 
 14505
 
 island
 
 0.007563
 
 0.0042775
 
 Not queried
 
  0.6513935
 
       NA
 
           NA
  
 
  
 20
 
   1373873
 
   1374086
 
  ENSEMBL   UCSC 
 
  214
 
  4
 
 25538
 
 tss;island
 
 0.018526
 
 0.0136723
 
 Not queried
 
  0.6513890
 
       NA
 
           NA
  
 
  
 12
 
 105723904
 
 105724410
 
  ENSEMBL   UCSC 
 
  507
 
  6
 
 9965
 
 tss
 
 0.022834
 
 0.0069416
 
 Not queried
 
  0.6513511
 
       NA
 
           NA
  
 
  
 1
 
  40506702
 
  40507006
 
  ENSEMBL   UCSC 
 
  305
 
  5
 
 450
 
 gene;island
 
 0.007179
 
 0.0079749
 
 Not queried
 
  0.6513477
 
       NA
 
           NA
  
 
  
 1
 
 145095823
 
 145096403
 
  ENSEMBL   UCSC 
 
  581
 
  8
 
 1882
 
 tss
 
 0.011166
 
 0.0082711
 
 Not queried
 
  0.6513000
 
       NA
 
           NA
  
 
  
 2
 
 187558355
 
 187558598
 
  ENSEMBL   UCSC 
 
  244
 
  5
 
 23774
 
 tss
 
 0.372365
 
 0.3893892
 
 Not queried
 
  0.6512464
 
       NA
 
           NA
  
 
  
 17
 
  77997663
 
  77997997
 
  ENSEMBL   UCSC 
 
  335
 
  5
 
 16851
 
 gene;island
 
 0.964019
 
 0.9767615
 
 Not queried
 
  0.6511298
 
       NA
 
           NA
  
 
  
 11
 
 132934128
 
 132934554
 
  ENSEMBL   UCSC 
 
  427
 
  5
 
 6983
 
 gene;island
 
 0.015105
 
 0.0210110
 
 Not queried
 
  0.6510942
 
       NA
 
           NA
  
 
  
 14
 
  96000874
 
  96000930
 
  ENSEMBL   UCSC 
 
   57
 
  4
 
 12198
 
 gene;tss;island
 
 0.004322
 
 0.0043957
 
 Not queried
 
  0.6509953
 
       NA
 
           NA
  
 
  
 7
 
 158800869
 
 158800986
 
  ENSEMBL   UCSC 
 
  118
 
  4
 
 38025
 
 tss;island
 
 0.461173
 
 0.5686998
 
 Not queried
 
  0.6507877
 
       NA
 
           NA
  
 
  
 15
 
  88799707
 
  88799999
 
  ENSEMBL   UCSC 
 
  293
 
  4
 
 13877
 
 tss
 
 0.008587
 
 0.0131542
 
 Not queried
 
  0.6507711
 
       NA
 
           NA
  
 
  
 10
 
 109674854
 
 109675284
 
  ENSEMBL   UCSC 
 
  431
 
  4
 
 6121
 
 island
 
 0.244033
 
 0.0937906
 
 Not queried
 
  0.6503797
 
       NA
 
           NA
  
 
  
 1
 
  38273312
 
  38273384
 
  ENSEMBL   UCSC 
 
   73
 
  4
 
 437
 
 gene;tss;island
 
 0.005956
 
 0.0039126
 
 Not queried
 
  0.6503607
 
       NA
 
           NA
  
 
  
 20
 
  21685834
 
  21686293
 
  ENSEMBL   UCSC 
 
  460
 
 10
 
 25631
 
 tss
 
 0.072573
 
 0.0653084
 
 Not queried
 
  0.6502821
 
       NA
 
           NA
  
 
  
 11
 
  58869663
 
  58870075
 
  ENSEMBL   UCSC 
 
  413
 
  5
 
 8301
 
 island
 
 0.017267
 
 0.0135049
 
 Not queried
 
  0.6502691
 
       NA
 
           NA
  
 
  
 14
 
 105714833
 
 105715025
 
  ENSEMBL   UCSC 
 
  193
 
  4
 
 12259
 
 gene;tss;island
 
 0.023800
 
 0.0240892
 
 Not queried
 
  0.6501768
 
       NA
 
           NA
  
 
  
 5
 
 176170138
 
 176170795
 
  ENSEMBL   UCSC 
 
  658
 
  7
 
 33433
 
 island
 
 0.060224
 
 0.1081924
 
 Not queried
 
  0.6501037
 
       NA
 
           NA
  
 
  
 1
 
 219347021
 
 219347458
 
  ENSEMBL   UCSC 
 
  438
 
  7
 
 4210
 
 island
 
 0.008237
 
 0.0044630
 
 Not queried
 
  0.6501025
 
       NA
 
           NA
  
 
  
 11
 
   2322286
 
   2323459
 
  ENSEMBL   UCSC 
 
 1174
 
 23
 
 6454
 
 gene;tss
 
 0.018851
 
 0.0241379
 
 Not queried
 
  0.6500030
 
       NA
 
           NA
  
 
  
 1
 
  27998703
 
  27998988
 
  ENSEMBL   UCSC 
 
  286
 
  6
 
 2928
 
 island
 
 0.009172
 
 0.0040336
 
 Not queried
 
  0.6499189
 
       NA
 
           NA
  
 
  
 8
 
 143359879
 
 143360290
 
  ENSEMBL   UCSC 
 
  412
 
  4
 
 39456
 
 gene;island
 
 0.832368
 
 0.8927761
 
 Not queried
 
  0.6498694
 
       NA
 
           NA
  
 
  
 12
 
   4382980
 
   4383281
 
  ENSEMBL   UCSC 
 
  302
 
  5
 
 9012
 
 gene
 
 0.003209
 
 0.0038107
 
 Not queried
 
  0.6498591
 
       NA
 
           NA
  
 
  
 20
 
    982623
 
    982831
 
  ENSEMBL   UCSC 
 
  209
 
  4
 
 25326
 
 gene
 
 0.094140
 
 0.1162168
 
 Not queried
 
  0.6498427
 
       NA
 
           NA
  
 
  
 12
 
  40499623
 
  40500139
 
  ENSEMBL   UCSC 
 
  517
 
  5
 
 10326
 
 island
 
 0.144428
 
 0.0480119
 
 Not queried
 
  0.6496603
 
       NA
 
           NA
  
 
  
 16
 
  88163698
 
  88163879
 
  ENSEMBL   UCSC 
 
  182
 
  4
 
 16195
 
 island
 
 0.702897
 
 0.8906837
 
 Not queried
 
  0.6495202
 
       NA
 
           NA
  
 
  
 3
 
 127771317
 
 127771497
 
  ENSEMBL   UCSC 
 
  181
 
  4
 
 27934
 
 gene;island
 
 0.008829
 
 0.0113449
 
 Not queried
 
  0.6493669
 
       NA
 
           NA
  
 
  
 1
 
 236559257
 
 236559675
 
  ENSEMBL   UCSC 
 
  419
 
  7
 
 1097
 
 gene;island
 
 0.123859
 
 0.5640660
 
 Not queried
 
  0.6493301
 
       NA
 
           NA
  
 
  
 2
 
 237993901
 
 237994083
 
  ENSEMBL   UCSC 
 
  183
 
  4
 
 25229
 
 island
 
 0.005940
 
 0.0076271
 
 Not queried
 
  0.6492543
 
       NA
 
           NA
  
 
  
 3
 
 141944300
 
 141944620
 
  ENSEMBL   UCSC 
 
  321
 
  6
 
 29550
 
 island
 
 0.003153
 
 0.0057361
 
 Not queried
 
  0.6491909
 
       NA
 
           NA
  
 
  
 15
 
  68127656
 
  68128261
 
  ENSEMBL   UCSC 
 
  606
 
  5
 
 14253
 
 island
 
 0.013170
 
 0.0417459
 
 Not queried
 
  0.6491709
 
       NA
 
           NA
  
 
  
 7
 
  99214442
 
  99214727
 
  ENSEMBL   UCSC 
 
  286
 
 10
 
 38740
 
 island
 
 0.010220
 
 0.0068846
 
 Not queried
 
  0.6491071
 
       NA
 
           NA
  
 
  
 3
 
 141497111
 
 141497491
 
  ENSEMBL   UCSC 
 
  381
 
  6
 
 27975
 
 gene
 
 0.689947
 
 0.8404010
 
 Not queried
 
  0.6490498
 
       NA
 
           NA
  
 
  
 7
 
  99698799
 
  99699161
 
  ENSEMBL   UCSC 
 
  363
 
  5
 
 37136
 
 gene;tss;island
 
 0.011430
 
 0.0072105
 
 Not queried
 
  0.6489180
 
       NA
 
           NA
  
 
  
 1
 
  28559030
 
  28559529
 
  ENSEMBL   UCSC 
 
  500
 
  7
 
 364
 
 gene
 
 0.007493
 
 0.0046795
 
 Not queried
 
  0.6488961
 
       NA
 
           NA
  
 
  
 1
 
 178693891
 
 178694150
 
  ENSEMBL   UCSC 
 
  260
 
  8
 
 2113
 
 tss
 
 0.010923
 
 0.0311631
 
 Not queried
 
  0.6488395
 
       NA
 
           NA
  
 
  
 6
 
  32938185
 
  32938475
 
  ENSEMBL   UCSC 
 
  291
 
  6
 
 34169
 
 gene;tss;island
 
 0.008863
 
 0.0080394
 
 Not queried
 
  0.6487579
 
       NA
 
           NA
  
 
  
 3
 
  33138872
 
  33139098
 
  ENSEMBL   UCSC 
 
  227
 
  4
 
 28210
 
 tss;island
 
 0.012660
 
 0.0097158
 
 Not queried
 
  0.6487033
 
       NA
 
           NA
  
 
  
 22
 
  31555670
 
  31556135
 
  ENSEMBL   UCSC 
 
  466
 
 10
 
 27076
 
 tss
 
 0.014177
 
 0.0124536
 
 Not queried
 
  0.6486951
 
       NA
 
           NA
  
 
  
 17
 
   7461260
 
   7461775
 
  ENSEMBL   UCSC 
 
  516
 
  6
 
 16401
 
 gene;island
 
 0.039042
 
 0.0329783
 
 Not queried
 
  0.6485122
 
       NA
 
           NA
  
 
  
 14
 
  88792913
 
  88793252
 
  ENSEMBL   UCSC 
 
  340
 
  6
 
 12166
 
 gene
 
 0.026147
 
 0.0497010
 
 Not queried
 
  0.6484896
 
       NA
 
           NA
  
 
  
 11
 
  74660429
 
  74660536
 
  ENSEMBL   UCSC 
 
  108
 
  4
 
 6830
 
 gene
 
 0.009466
 
 0.0131261
 
 Not queried
 
  0.6484818
 
       NA
 
           NA
  
 
  
 17
 
  32909042
 
  32909375
 
  ENSEMBL   UCSC 
 
  334
 
  4
 
 16516
 
 gene;island
 
 0.020618
 
 0.1128765
 
 Not queried
 
  0.6484354
 
       NA
 
           NA
  
 
  
 16
 
    188651
 
    189100
 
  ENSEMBL   UCSC 
 
  450
 
  8
 
 14960
 
 tss;island
 
 0.002962
 
 0.0041089
 
 Not queried
 
  0.6483244
 
       NA
 
           NA
  
 
  
 5
 
  87975798
 
  87976294
 
  ENSEMBL   UCSC 
 
  497
 
  5
 
 31681
 
 gene;island
 
 0.033947
 
 0.0610971
 
 Not queried
 
  0.6482724
 
       NA
 
           NA
  
 
  
 17
 
  79829189
 
  79829466
 
  ENSEMBL   UCSC 
 
  278
 
  5
 
 18946
 
 island
 
 0.006459
 
 0.0084413
 
 Not queried
 
  0.6482427
 
       NA
 
           NA
  
 
  
 1
 
  28559030
 
  28559620
 
  ENSEMBL   UCSC 
 
  591
 
 10
 
 2938
 
 island
 
 0.021463
 
 0.0066296
 
 Not queried
 
  0.6479498
 
       NA
 
           NA
  
 
  
 8
 
  53478023
 
  53478102
 
  ENSEMBL   UCSC 
 
   80
 
  4
 
 39698
 
 tss
 
 0.074692
 
 0.1117539
 
 Not queried
 
  0.6479299
 
       NA
 
           NA
  
 
  
 17
 
  78822406
 
  78822923
 
  ENSEMBL   UCSC 
 
  518
 
  5
 
 16877
 
 gene
 
 0.960191
 
 0.9732451
 
 Not queried
 
  0.6479209
 
       NA
 
           NA
  
 
  
 4
 
  84031053
 
  84031513
 
  ENSEMBL   UCSC 
 
  461
 
  9
 
 30473
 
 tss
 
 0.014945
 
 0.0086620
 
 Not queried
 
  0.6478794
 
       NA
 
           NA
  
 
  
 13
 
 114814024
 
 114814401
 
  ENSEMBL   UCSC 
 
  378
 
  5
 
 11310
 
 gene;island
 
 0.905957
 
 0.6536994
 
 Not queried
 
  0.6477870
 
       NA
 
           NA
  
 
  
 20
 
  13279761
 
  13280201
 
  ENSEMBL   UCSC 
 
  441
 
  6
 
 25359
 
 gene;island
 
 0.649310
 
 0.7166023
 
 Not queried
 
  0.6474921
 
       NA
 
           NA
  
 
  
 12
 
  48399140
 
  48399493
 
  ENSEMBL   UCSC 
 
  354
 
  5
 
 9666
 
 tss;island
 
 0.261328
 
 0.2826380
 
 Not queried
 
  0.6473228
 
       NA
 
           NA
  
 
  
 22
 
  39378224
 
  39378382
 
  ENSEMBL   UCSC 
 
  159
 
  4
 
 27148
 
 tss
 
 0.015584
 
 0.0176073
 
 Not queried
 
  0.6471355
 
       NA
 
           NA
  
 
  
 6
 
  36355343
 
  36355825
 
  ENSEMBL   UCSC 
 
  483
 
  7
 
 36081
 
 island
 
 0.021791
 
 0.0129635
 
 Not queried
 
  0.6471103
 
       NA
 
           NA
  
 
  
 17
 
  36003216
 
  36003574
 
  ENSEMBL   UCSC 
 
  359
 
  7
 
 18247
 
 island
 
 0.002768
 
 0.0049066
 
 Not queried
 
  0.6470950
 
       NA
 
           NA
  
 
  
 1
 
  45205136
 
  45205438
 
  ENSEMBL   UCSC 
 
  303
 
  8
 
 1574
 
 tss
 
 0.002477
 
 0.0025239
 
 Not queried
 
  0.6470861
 
       NA
 
           NA
  
 
  
 7
 
 105752848
 
 105753147
 
  ENSEMBL   UCSC 
 
  300
 
  9
 
 37820
 
 tss;island
 
 0.004202
 
 0.0063393
 
 Not queried
 
  0.6470808
 
       NA
 
           NA
  
 
  
 8
 
  55370192
 
  55370434
 
  ENSEMBL   UCSC 
 
  243
 
  8
 
 39706
 
 tss
 
 0.172905
 
 0.0466648
 
 Not queried
 
  0.6470740
 
       NA
 
           NA
  
 
  
 1
 
  77332991
 
  77333159
 
  ENSEMBL   UCSC 
 
  169
 
  6
 
 1703
 
 tss
 
 0.003600
 
 0.0104960
 
 Not queried
 
  0.6469829
 
       NA
 
           NA
  
 
  
 19
 
  52490101
 
  52490339
 
  ENSEMBL   UCSC 
 
  239
 
  9
 
 21002
 
 tss
 
 0.011613
 
 0.0073143
 
 Not queried
 
  0.6469565
 
       NA
 
           NA
  
 
  
 1
 
 113741483
 
 113741744
 
  ENSEMBL   UCSC 
 
  262
 
  4
 
 3614
 
 island
 
 0.645899
 
 0.8266330
 
 Not queried
 
  0.6469391
 
       NA
 
           NA
  
 
  
 22
 
  46481603
 
  46482023
 
  ENSEMBL   UCSC 
 
  421
 
 10
 
 27581
 
 island
 
 0.760446
 
 0.6495982
 
 Not queried
 
  0.6469199
 
       NA
 
           NA
  
 
  
 1
 
  21044031
 
  21044327
 
  ENSEMBL   UCSC 
 
  297
 
  6
 
 2801
 
 island
 
 0.016783
 
 0.0125099
 
 Not queried
 
  0.6469184
 
       NA
 
           NA
  
 
  
 20
 
  31407338
 
  31407559
 
  ENSEMBL   UCSC 
 
  222
 
  6
 
 25677
 
 tss;island
 
 0.086916
 
 0.0742729
 
 Not queried
 
  0.6467337
 
       NA
 
           NA
  
 
  
 1
 
   2938646
 
   2939117
 
  ENSEMBL   UCSC 
 
  472
 
  5
 
 94
 
 gene;island
 
 0.314986
 
 0.3467807
 
 Not queried
 
  0.6467184
 
       NA
 
           NA
  
 
  
 10
 
    661009
 
    661730
 
  ENSEMBL   UCSC 
 
  722
 
  8
 
 4446
 
 gene
 
 0.528116
 
 0.5771849
 
 Not queried
 
  0.6465689
 
       NA
 
           NA
  
 
  
 1
 
  32670916
 
  32671645
 
  ENSEMBL   UCSC 
 
  730
 
 12
 
 2987
 
 island
 
 0.028917
 
 0.0226781
 
 Not queried
 
  0.6465391
 
       NA
 
           NA
  
 
  
 8
 
  49834354
 
  49834643
 
  ENSEMBL   UCSC 
 
  290
 
  4
 
 39693
 
 tss;island
 
 0.077399
 
 0.0891690
 
 Not queried
 
  0.6465352
 
       NA
 
           NA
  
 
  
 16
 
  69166316
 
  69166964
 
  ENSEMBL   UCSC 
 
  649
 
 10
 
 14818
 
 gene;tss;island
 
 0.007908
 
 0.0050772
 
 Not queried
 
  0.6465114
 
       NA
 
           NA
  
 
  
 19
 
   4909175
 
   4909600
 
  ENSEMBL   UCSC 
 
  426
 
 11
 
 20375
 
 tss;island
 
 0.007565
 
 0.0051530
 
 Not queried
 
  0.6464118
 
       NA
 
           NA
  
 
  
 5
 
  43557435
 
  43557751
 
  ENSEMBL   UCSC 
 
  317
 
  8
 
 32049
 
 tss;island
 
 0.014701
 
 0.0138502
 
 Not queried
 
  0.6462269
 
       NA
 
           NA
  
 
  
 3
 
 195539297
 
 195539463
 
  ENSEMBL   UCSC 
 
  167
 
  5
 
 28806
 
 tss
 
 0.576935
 
 0.6574689
 
 Not queried
 
  0.6462146
 
       NA
 
           NA
  
 
  
 10
 
  71087834
 
  71088038
 
  ENSEMBL   UCSC 
 
  205
 
  4
 
 4613
 
 gene
 
 0.059916
 
 0.0469206
 
 Not queried
 
  0.6461146
 
       NA
 
           NA
  
 
  
 19
 
   3676240
 
   3676582
 
  ENSEMBL   UCSC 
 
  343
 
  5
 
 19720
 
 gene;island
 
 0.982580
 
 0.9700690
 
 Not queried
 
  0.6458667
 
       NA
 
           NA
  
 
  
 12
 
  94533765
 
  94533951
 
  ENSEMBL   UCSC 
 
  187
 
  4
 
 10679
 
 island
 
 0.813410
 
 0.8536114
 
 Not queried
 
  0.6458455
 
       NA
 
           NA
  
 
  
 4
 
   3464653
 
   3465064
 
  ENSEMBL   UCSC 
 
  412
 
 10
 
 30839
 
 island
 
 0.025105
 
 0.2119496
 
 Not queried
 
  0.6458394
 
       NA
 
           NA
  
 
  
 7
 
  27154262
 
  27155548
 
  ENSEMBL   UCSC 
 
 1287
 
 18
 
 36931
 
 gene;island
 
 0.783138
 
 0.8097297
 
 Not queried
 
  0.6458164
 
       NA
 
           NA
  
 
  
 7
 
  27154262
 
  27155039
 
  ENSEMBL   UCSC 
 
  778
 
 14
 
 37490
 
 tss
 
 0.692641
 
 0.7030214
 
 Not queried
 
  0.6458164
 
       NA
 
           NA
  
 
  
 8
 
  41435301
 
  41435469
 
  ENSEMBL   UCSC 
 
  169
 
  4
 
 39670
 
 tss;island
 
 0.014909
 
 0.0182725
 
 Not queried
 
  0.6457391
 
       NA
 
           NA
  
 
  
 7
 
  27207996
 
  27208285
 
  ENSEMBL   UCSC 
 
  290
 
  4
 
 38357
 
 island
 
 0.134564
 
 0.0714582
 
 Not queried
 
  0.6457062
 
       NA
 
           NA
  
 
  
 18
 
  47018095
 
  47018265
 
  ENSEMBL   UCSC 
 
  171
 
  4
 
 19110
 
 gene;tss;island
 
 0.017157
 
 0.0109130
 
 Not queried
 
  0.6455908
 
       NA
 
           NA
  
 
  
 16
 
    873426
 
    873777
 
  ENSEMBL   UCSC 
 
  352
 
  4
 
 15490
 
 island
 
 0.703504
 
 0.8357182
 
 Not queried
 
  0.6455649
 
       NA
 
           NA
  
 
  
 7
 
 123197632
 
 123197866
 
  ENSEMBL   UCSC 
 
  235
 
  4
 
 37206
 
 gene
 
 0.013289
 
 0.0109539
 
 Not queried
 
  0.6455219
 
       NA
 
           NA
  
 
  
 17
 
  45973527
 
  45973779
 
  ENSEMBL   UCSC 
 
  253
 
  4
 
 16647
 
 gene
 
 0.004465
 
 0.0029729
 
 Not queried
 
  0.6453639
 
       NA
 
           NA
  
 
  
 16
 
   3096477
 
   3096711
 
  ENSEMBL   UCSC 
 
  235
 
  6
 
 15635
 
 island
 
 0.008549
 
 0.0280324
 
 Not queried
 
  0.6451140
 
       NA
 
           NA
  
 
  
 19
 
  30363016
 
  30363526
 
  ENSEMBL   UCSC 
 
  511
 
  4
 
 21858
 
 island
 
 0.310964
 
 0.4393501
 
 Not queried
 
  0.6450385
 
       NA
 
           NA
  
 
  
 6
 
  30710200
 
  30710447
 
  ENSEMBL   UCSC 
 
  248
 
  5
 
 33823
 
 gene
 
 0.009488
 
 0.0018079
 
 Not queried
 
  0.6450151
 
       NA
 
           NA
  
 
  
 1
 
  46668822
 
  46668882
 
  ENSEMBL   UCSC 
 
   61
 
  4
 
 509
 
 gene;tss;island
 
 0.019284
 
 0.0127164
 
 Not queried
 
  0.6448481
 
       NA
 
           NA
  
 
  
 16
 
   3074170
 
   3074537
 
  ENSEMBL   UCSC 
 
  368
 
 10
 
 14621
 
 gene;island
 
 0.004105
 
 0.0032681
 
 Not queried
 
  0.6447693
 
       NA
 
           NA
  
 
  
 5
 
   1077533
 
   1077845
 
  ENSEMBL   UCSC 
 
  313
 
  4
 
 31519
 
 gene;island
 
 0.924607
 
 0.7251234
 
 Not queried
 
  0.6447289
 
       NA
 
           NA
  
 
  
 1
 
  47799633
 
  47799834
 
  ENSEMBL   UCSC 
 
  202
 
  4
 
 517
 
 gene;island
 
 0.008744
 
 0.0077784
 
 Not queried
 
  0.6446943
 
       NA
 
           NA
  
 
  
 16
 
  67184918
 
  67185194
 
  ENSEMBL   UCSC 
 
  277
 
  6
 
 15292
 
 tss;island
 
 0.014623
 
 0.0852200
 
 Not queried
 
  0.6446720
 
       NA
 
           NA
  
 
  
 16
 
   2014998
 
   2015157
 
  ENSEMBL   UCSC 
 
  160
 
  4
 
 14590
 
 gene
 
 0.003424
 
 0.0043678
 
 Not queried
 
  0.6446671
 
       NA
 
           NA
  
 
  
 18
 
  28682229
 
  28682497
 
  ENSEMBL   UCSC 
 
  269
 
  4
 
 19447
 
 island
 
 0.268923
 
 0.0500379
 
 Not queried
 
  0.6446447
 
       NA
 
           NA
  
 
  
 2
 
 207139066
 
 207139471
 
  ENSEMBL   UCSC 
 
  406
 
  9
 
 23835
 
 tss;island
 
 0.009528
 
 0.0166871
 
 Not queried
 
  0.6446437
 
       NA
 
           NA
  
 
  
 5
 
  64330880
 
  64330959
 
  ENSEMBL   UCSC 
 
   80
 
  4
 
 32832
 
 island
 
 0.577033
 
 0.2839273
 
 Not queried
 
  0.6445114
 
       NA
 
           NA
  
 
  
 14
 
  69283484
 
  69283595
 
  ENSEMBL   UCSC 
 
  112
 
  4
 
 12961
 
 island
 
 0.003933
 
 0.0037570
 
 Not queried
 
  0.6444420
 
       NA
 
           NA
  
 
  
 5
 
  86708854
 
  86709126
 
  ENSEMBL   UCSC 
 
  273
 
  4
 
 32164
 
 tss
 
 0.014184
 
 0.0098120
 
 Not queried
 
  0.6444017
 
       NA
 
           NA
  
 
  
 5
 
  86708659
 
  86709126
 
  ENSEMBL   UCSC 
 
  468
 
  6
 
 32938
 
 island
 
 0.005905
 
 0.0039694
 
 Not queried
 
  0.6444017
 
       NA
 
           NA
  
 
  
 18
 
  72837386
 
  72837782
 
  ENSEMBL   UCSC 
 
  397
 
  6
 
 19574
 
 island
 
 0.730912
 
 0.5142173
 
 Not queried
 
  0.6443701
 
       NA
 
           NA
  
 
  
 15
 
  89787223
 
  89787724
 
  ENSEMBL   UCSC 
 
  502
 
  5
 
 13467
 
 gene
 
 0.008497
 
 0.0080110
 
 Not queried
 
  0.6443266
 
       NA
 
           NA
  
 
  
 10
 
 123922620
 
 123923099
 
  ENSEMBL   UCSC 
 
  480
 
  8
 
 5408
 
 tss
 
 0.026870
 
 0.0270899
 
 Not queried
 
  0.6441997
 
       NA
 
           NA
  
 
  
 7
 
 156832709
 
 156833196
 
  ENSEMBL   UCSC 
 
  488
 
  6
 
 39095
 
 island
 
 0.702758
 
 0.6986625
 
 Not queried
 
  0.6441819
 
       NA
 
           NA
  
 
  
 10
 
  73848817
 
  73849167
 
  ENSEMBL   UCSC 
 
  351
 
  8
 
 5146
 
 tss
 
 0.936953
 
 0.8459318
 
 Not queried
 
  0.6441801
 
       NA
 
           NA
  
 
  
 7
 
  87563717
 
  87563931
 
  ENSEMBL   UCSC 
 
  215
 
  5
 
 37075
 
 gene
 
 0.023910
 
 0.0285502
 
 Not queried
 
  0.6441431
 
       NA
 
           NA
  
 
  
 5
 
   3535836
 
   3536279
 
  ENSEMBL   UCSC 
 
  444
 
  5
 
 32664
 
 island
 
 0.432191
 
 0.6579681
 
 Not queried
 
  0.6441228
 
       NA
 
           NA
  
 
  
 1
 
   1097027
 
   1097270
 
  ENSEMBL   UCSC 
 
  244
 
  4
 
 2404
 
 island
 
 0.437304
 
 0.5780078
 
 Not queried
 
  0.6441157
 
       NA
 
           NA
  
 
  
 4
 
 154680808
 
 154681323
 
  ENSEMBL   UCSC 
 
  516
 
  6
 
 30155
 
 gene;island
 
 0.087120
 
 0.0407163
 
 Not queried
 
  0.6440858
 
       NA
 
           NA
  
 
  
 19
 
  12273496
 
  12273905
 
  ENSEMBL   UCSC 
 
  410
 
  8
 
 20491
 
 tss
 
 0.015947
 
 0.0122151
 
 Not queried
 
  0.6438110
 
       NA
 
           NA
  
 
  
 19
 
  12273496
 
  12274129
 
  ENSEMBL   UCSC 
 
  634
 
 12
 
 21596
 
 island
 
 0.013563
 
 0.0119418
 
 Not queried
 
  0.6438110
 
       NA
 
           NA
  
 
  
 2
 
 177502735
 
 177503063
 
  ENSEMBL   UCSC 
 
  329
 
  6
 
 24917
 
 island
 
 0.021577
 
 0.0166586
 
 Not queried
 
  0.6438008
 
       NA
 
           NA
  
 
  
 4
 
   3471816
 
   3472246
 
  ENSEMBL   UCSC 
 
  431
 
  4
 
 29906
 
 gene;island
 
 0.394674
 
 0.6157794
 
 Not queried
 
  0.6432329
 
       NA
 
           NA
  
 
  
 13
 
  29394754
 
  29394960
 
  ENSEMBL   UCSC 
 
  207
 
  4
 
 11644
 
 island
 
 0.023601
 
 0.0150209
 
 Not queried
 
  0.6429862
 
       NA
 
           NA
  
 
  
 3
 
   9993731
 
   9994061
 
  ENSEMBL   UCSC 
 
  331
 
  5
 
 27657
 
 gene
 
 0.005634
 
 0.0076116
 
 Not queried
 
  0.6429681
 
       NA
 
           NA
  
 
  
 15
 
  55700431
 
  55700549
 
  ENSEMBL   UCSC 
 
  119
 
  4
 
 13359
 
 gene
 
 0.012119
 
 0.0136815
 
 Not queried
 
  0.6427615
 
       NA
 
           NA
  
 
  
 7
 
 150102660
 
 150102791
 
  ENSEMBL   UCSC 
 
  132
 
  5
 
 37978
 
 tss;island
 
 0.005965
 
 0.0039737
 
 Not queried
 
  0.6426347
 
       NA
 
           NA
  
 
  
 19
 
  36024217
 
  36024282
 
  ENSEMBL   UCSC 
 
   66
 
  5
 
 20706
 
 tss
 
 0.979816
 
 0.9861311
 
 Not queried
 
  0.6425169
 
       NA
 
           NA
  
 
  
 4
 
  21950567
 
  21951046
 
  ENSEMBL   UCSC 
 
  480
 
  6
 
 30325
 
 tss;island
 
 0.282897
 
 0.2333186
 
 Not queried
 
  0.6424911
 
       NA
 
           NA
  
 
  
 2
 
  54483058
 
  54483817
 
  ENSEMBL   UCSC 
 
  760
 
  9
 
 22683
 
 gene;island
 
 0.860081
 
 0.8408497
 
 Not queried
 
  0.6424790
 
       NA
 
           NA
  
 
  
 22
 
  24115096
 
  24115568
 
  ENSEMBL   UCSC 
 
  473
 
  4
 
 26809
 
 gene
 
 0.149065
 
 0.0996633
 
 Not queried
 
  0.6423341
 
       NA
 
           NA
  
 
  
 5
 
 150138399
 
 150138895
 
  ENSEMBL   UCSC 
 
  497
 
  7
 
 32434
 
 tss;island
 
 0.061769
 
 0.0117894
 
 Not queried
 
  0.6423013
 
       NA
 
           NA
  
 
  
 1
 
 109791839
 
 109792146
 
  ENSEMBL   UCSC 
 
  308
 
  4
 
 1800
 
 tss;island
 
 0.024159
 
 0.0110987
 
 Not queried
 
  0.6421962
 
       NA
 
           NA
  
 
  
 16
 
  66586309
 
  66586415
 
  ENSEMBL   UCSC 
 
  107
 
  5
 
 15280
 
 tss
 
 0.013860
 
 0.0182970
 
 Not queried
 
  0.6420311
 
       NA
 
           NA
  
 
  
 16
 
  66586309
 
  66586745
 
  ENSEMBL   UCSC 
 
  437
 
  9
 
 15988
 
 island
 
 0.017855
 
 0.0182970
 
 Not queried
 
  0.6420311
 
       NA
 
           NA
  
 
  
 1
 
 107599041
 
 107599246
 
  ENSEMBL   UCSC 
 
  206
 
  4
 
 1787
 
 tss;island
 
 0.031722
 
 0.0272039
 
 Not queried
 
  0.6419401
 
       NA
 
           NA
  
 
  
 3
 
  45729776
 
  45730181
 
  ENSEMBL   UCSC 
 
  406
 
  6
 
 28269
 
 tss;island
 
 0.007677
 
 0.0083404
 
 Not queried
 
  0.6418851
 
       NA
 
           NA
  
 
  
 4
 
  44679953
 
  44680862
 
  ENSEMBL   UCSC 
 
  910
 
  9
 
 31017
 
 island
 
 0.005007
 
 0.0046948
 
 Not queried
 
  0.6417747
 
       NA
 
           NA
  
 
  
 12
 
  54332992
 
  54333323
 
  ENSEMBL   UCSC 
 
  332
 
  4
 
 9154
 
 gene;island
 
 0.148423
 
 0.1983706
 
 Not queried
 
  0.6417073
 
       NA
 
           NA
  
 
  
 17
 
  41466953
 
  41467212
 
  ENSEMBL   UCSC 
 
  260
 
  4
 
 17388
 
 tss
 
 0.008489
 
 0.0053227
 
 Not queried
 
  0.6416583
 
       NA
 
           NA
  
 
  
 17
 
  58042144
 
  58042493
 
  ENSEMBL   UCSC 
 
  350
 
 11
 
 17550
 
 tss
 
 0.007819
 
 0.0062949
 
 Not queried
 
  0.6416564
 
       NA
 
           NA
  
 
  
 19
 
  36359211
 
  36359556
 
  ENSEMBL   UCSC 
 
  346
 
  8
 
 21922
 
 island
 
 0.014597
 
 0.0522534
 
 Not queried
 
  0.6415447
 
       NA
 
           NA
  
 
  
 12
 
  44200243
 
  44200524
 
  ENSEMBL   UCSC 
 
  282
 
  8
 
 9649
 
 tss;island
 
 0.019777
 
 0.0118653
 
 Not queried
 
  0.6415202
 
       NA
 
           NA
  
 
  
 7
 
  43946371
 
  43946651
 
  ENSEMBL   UCSC 
 
  281
 
  5
 
 36985
 
 gene;tss;island
 
 0.021075
 
 0.0304587
 
 Not queried
 
  0.6413406
 
       NA
 
           NA
  
 
  
 15
 
  70390233
 
  70390440
 
  ENSEMBL   UCSC 
 
  208
 
  6
 
 14267
 
 island
 
 0.003963
 
 0.0050535
 
 Not queried
 
  0.6413070
 
       NA
 
           NA
  
 
  
 7
 
  16685473
 
  16685943
 
  ENSEMBL   UCSC 
 
  471
 
 10
 
 37454
 
 tss;island
 
 0.031869
 
 0.0152471
 
 Not queried
 
  0.6412739
 
       NA
 
           NA
  
 
  
 19
 
  54410030
 
  54410305
 
  ENSEMBL   UCSC 
 
  276
 
  4
 
 20211
 
 gene;island
 
 0.020359
 
 0.0271390
 
 Not queried
 
  0.6410834
 
       NA
 
           NA
  
 
  
 18
 
  76189809
 
  76190031
 
  ENSEMBL   UCSC 
 
  223
 
  4
 
 19591
 
 island
 
 0.830626
 
 0.8974259
 
 Not queried
 
  0.6409202
 
       NA
 
           NA
  
 
  
 11
 
 118230307
 
 118230422
 
  ENSEMBL   UCSC 
 
  116
 
  4
 
 6928
 
 gene
 
 0.002369
 
 0.0038270
 
 Not queried
 
  0.6407085
 
       NA
 
           NA
  
 
  
 7
 
 106301534
 
 106301767
 
  ENSEMBL   UCSC 
 
  234
 
  5
 
 38824
 
 island
 
 0.022233
 
 0.0153139
 
 Not queried
 
  0.6404535
 
       NA
 
           NA
  
 
  
 19
 
   8669959
 
   8670193
 
  ENSEMBL   UCSC 
 
  235
 
  4
 
 19797
 
 gene;island
 
 0.767061
 
 0.7456151
 
 Not queried
 
  0.6403837
 
       NA
 
           NA
  
 
  
 16
 
  30710278
 
  30710486
 
  ENSEMBL   UCSC 
 
  209
 
  4
 
 15844
 
 island
 
 0.006499
 
 0.0017934
 
 Not queried
 
  0.6401094
 
       NA
 
           NA
  
 
  
 13
 
  80055594
 
  80055826
 
  ENSEMBL   UCSC 
 
  233
 
  5
 
 11184
 
 gene;island
 
 0.012129
 
 0.0118140
 
 Not queried
 
  0.6400471
 
       NA
 
           NA
  
 
  
 2
 
 128458240
 
 128458845
 
  ENSEMBL   UCSC 
 
  606
 
  8
 
 24700
 
 island
 
 0.002647
 
 0.0033495
 
 Not queried
 
  0.6399965
 
       NA
 
           NA
  
 
  
 2
 
 175499113
 
 175499377
 
  ENSEMBL   UCSC 
 
  265
 
  4
 
 22930
 
 gene
 
 0.003741
 
 0.0035242
 
 Not queried
 
  0.6399959
 
       NA
 
           NA
  
 
  
 20
 
  44650068
 
  44650270
 
  ENSEMBL   UCSC 
 
  203
 
  6
 
 25786
 
 tss
 
 0.103449
 
 0.2975079
 
 Not queried
 
  0.6398187
 
       NA
 
           NA
  
 
  
 6
 
 137540615
 
 137540814
 
  ENSEMBL   UCSC 
 
  200
 
  6
 
 35248
 
 tss;island
 
 0.004953
 
 0.0038901
 
 Not queried
 
  0.6397645
 
       NA
 
           NA
  
 
  
 8
 
  22436813
 
  22437193
 
  ENSEMBL   UCSC 
 
  381
 
  4
 
 39235
 
 gene;tss;island
 
 0.065873
 
 0.0475768
 
 Not queried
 
  0.6397496
 
       NA
 
           NA
  
 
  
 16
 
  89202817
 
  89203153
 
  ENSEMBL   UCSC 
 
  337
 
  4
 
 14933
 
 gene
 
 0.977165
 
 0.9690971
 
 Not queried
 
  0.6397260
 
       NA
 
           NA
  
 
  
 7
 
  28450687
 
  28451011
 
  ENSEMBL   UCSC 
 
  325
 
  5
 
 36944
 
 gene;tss;island
 
 0.009105
 
 0.0170400
 
 Not queried
 
  0.6396630
 
       NA
 
           NA
  
 
  
 11
 
    192763
 
    193062
 
  ENSEMBL   UCSC 
 
  300
 
  7
 
 7002
 
 tss
 
 0.404220
 
 0.5348096
 
 Not queried
 
  0.6395694
 
       NA
 
           NA
  
 
  
 18
 
  49867117
 
  49867488
 
  ENSEMBL   UCSC 
 
  372
 
  5
 
 19115
 
 gene;island
 
 0.047844
 
 0.2252315
 
 Not queried
 
  0.6395071
 
       NA
 
           NA
  
 
  
 16
 
  88752925
 
  88753119
 
  ENSEMBL   UCSC 
 
  195
 
  4
 
 15413
 
 tss
 
 0.008984
 
 0.0098277
 
 Not queried
 
  0.6393038
 
       NA
 
           NA
  
 
  
 3
 
 154042025
 
 154042778
 
  ENSEMBL   UCSC 
 
  754
 
 14
 
 29601
 
 island
 
 0.008870
 
 0.0143016
 
 Not queried
 
  0.6392674
 
       NA
 
           NA
  
 
  
 15
 
  83378637
 
  83378895
 
  ENSEMBL   UCSC 
 
  259
 
  8
 
 13859
 
 tss
 
 0.011342
 
 0.0069120
 
 Not queried
 
  0.6391216
 
       NA
 
           NA
  
 
  
 5
 
   1438168
 
   1438531
 
  ENSEMBL   UCSC 
 
  364
 
  5
 
 31539
 
 gene
 
 0.664508
 
 0.7479813
 
 Not queried
 
  0.6391118
 
       NA
 
           NA
  
 
  
 12
 
 107078410
 
 107078680
 
  ENSEMBL   UCSC 
 
  271
 
  5
 
 9281
 
 gene
 
 0.546362
 
 0.6524011
 
 Not queried
 
  0.6390981
 
       NA
 
           NA
  
 
  
 8
 
  41655673
 
  41656079
 
  ENSEMBL   UCSC 
 
  407
 
  6
 
 39294
 
 gene;tss;island
 
 0.374464
 
 0.1784320
 
 Not queried
 
  0.6390652
 
       NA
 
           NA
  
 
  
 19
 
  59031341
 
  59031463
 
  ENSEMBL   UCSC 
 
  123
 
  4
 
 21148
 
 tss;island
 
 0.006044
 
 0.0040799
 
 Not queried
 
  0.6389911
 
       NA
 
           NA
  
 
  
 17
 
  57297518
 
  57297815
 
  ENSEMBL   UCSC 
 
  298
 
  8
 
 17544
 
 tss
 
 0.004608
 
 0.0065828
 
 Not queried
 
  0.6388930
 
       NA
 
           NA
  
 
  
 6
 
 112408804
 
 112409181
 
  ENSEMBL   UCSC 
 
  378
 
 10
 
 35182
 
 tss
 
 0.007543
 
 0.0058358
 
 Not queried
 
  0.6388036
 
       NA
 
           NA
  
 
  
 15
 
  60690667
 
  60690974
 
  ENSEMBL   UCSC 
 
  308
 
  4
 
 13710
 
 tss;island
 
 0.036685
 
 0.0257605
 
 Not queried
 
  0.6386976
 
       NA
 
           NA
  
 
  
 19
 
  54385215
 
  54385441
 
  ENSEMBL   UCSC 
 
  227
 
  7
 
 21044
 
 tss;island
 
 0.016097
 
 0.0323958
 
 Not queried
 
  0.6386164
 
       NA
 
           NA
  
 
  
 20
 
  21494547
 
  21494977
 
  ENSEMBL   UCSC 
 
  431
 
  9
 
 26041
 
 island
 
 0.080158
 
 0.1257298
 
 Not queried
 
  0.6386087
 
       NA
 
           NA
  
 
  
 1
 
  93811564
 
  93811877
 
  ENSEMBL   UCSC 
 
  314
 
  6
 
 639
 
 gene
 
 0.021047
 
 0.0107946
 
 Not queried
 
  0.6386086
 
       NA
 
           NA
  
 
  
 15
 
  45996340
 
  45996787
 
  ENSEMBL   UCSC 
 
  448
 
  5
 
 14117
 
 island
 
 0.072550
 
 0.0343390
 
 Not queried
 
  0.6384962
 
       NA
 
           NA
  
 
  
 10
 
  23983496
 
  23983686
 
  ENSEMBL   UCSC 
 
  191
 
  5
 
 5612
 
 island
 
 0.024631
 
 0.0689700
 
 Not queried
 
  0.6384439
 
       NA
 
           NA
  
 
  
 5
 
 131281008
 
 131281574
 
  ENSEMBL   UCSC 
 
  567
 
  6
 
 33082
 
 island
 
 0.675260
 
 0.7864496
 
 Not queried
 
  0.6384413
 
       NA
 
           NA
  
 
  
 3
 
   2141614
 
   2141937
 
  ENSEMBL   UCSC 
 
  324
 
  4
 
 28112
 
 tss;island
 
 0.041278
 
 0.1023191
 
 Not queried
 
  0.6384234
 
       NA
 
           NA
  
 
  
 2
 
  26569718
 
  26570144
 
  ENSEMBL   UCSC 
 
  427
 
  8
 
 23242
 
 tss
 
 0.004733
 
 0.0044373
 
 Not queried
 
  0.6383258
 
       NA
 
           NA
  
 
  
 10
 
 131263962
 
 131264846
 
  ENSEMBL   UCSC 
 
  885
 
 12
 
 5439
 
 tss;island
 
 0.574623
 
 0.6554235
 
 Not queried
 
  0.6382921
 
       NA
 
           NA
  
 
  
 10
 
  13933996
 
  13934438
 
  ENSEMBL   UCSC 
 
  443
 
  5
 
 4505
 
 gene;island
 
 0.046609
 
 0.0254715
 
 Not queried
 
  0.6382449
 
       NA
 
           NA
  
 
  
 5
 
  93447407
 
  93447700
 
  ENSEMBL   UCSC 
 
  294
 
  7
 
 32179
 
 tss
 
 0.019104
 
 0.1936801
 
 Not queried
 
  0.6382339
 
       NA
 
           NA
  
 
  
 2
 
  19553237
 
  19553546
 
  ENSEMBL   UCSC 
 
  310
 
  4
 
 22587
 
 gene;island
 
 0.487469
 
 0.3388999
 
 Not queried
 
  0.6381950
 
       NA
 
           NA
  
 
  
 7
 
   4049531
 
   4050114
 
  ENSEMBL   UCSC 
 
  584
 
  7
 
 36825
 
 gene
 
 0.362258
 
 0.4692346
 
 Not queried
 
  0.6381777
 
       NA
 
           NA
  
 
  
 7
 
 131012106
 
 131012765
 
  ENSEMBL   UCSC 
 
  660
 
 13
 
 37229
 
 gene;island
 
 0.006209
 
 0.0040607
 
 Not queried
 
  0.6380227
 
       NA
 
           NA
  
 
  
 8
 
 144099482
 
 144099991
 
  ENSEMBL   UCSC 
 
  510
 
  6
 
 39942
 
 tss;island
 
 0.023845
 
 0.0215630
 
 Not queried
 
  0.6379796
 
       NA
 
           NA
  
 
  
 2
 
 220363628
 
 220363862
 
  ENSEMBL   UCSC 
 
  235
 
  4
 
 23035
 
 gene
 
 0.005271
 
 0.0056542
 
 Not queried
 
  0.6379204
 
       NA
 
           NA
  
 
  
 18
 
  44676959
 
  44677236
 
  ENSEMBL   UCSC 
 
  278
 
  6
 
 19263
 
 tss
 
 0.003741
 
 0.0029194
 
 Not queried
 
  0.6378315
 
       NA
 
           NA
  
 
  
 19
 
  41770635
 
  41771095
 
  ENSEMBL   UCSC 
 
  461
 
  4
 
 20055
 
 gene;island
 
 0.015880
 
 0.0264515
 
 Not queried
 
  0.6377434
 
       NA
 
           NA
  
 
  
 9
 
  95858233
 
  95858671
 
  ENSEMBL   UCSC 
 
  439
 
  5
 
 40910
 
 island
 
 0.018949
 
 0.0106163
 
 Not queried
 
  0.6374778
 
       NA
 
           NA
  
 
  
 6
 
  28583971
 
  28584464
 
  ENSEMBL   UCSC 
 
  494
 
 18
 
 35591
 
 island
 
 0.351542
 
 0.1955764
 
 Not queried
 
  0.6374613
 
       NA
 
           NA
  
 
  
 11
 
    353567
 
    354013
 
  ENSEMBL   UCSC 
 
  447
 
  4
 
 7844
 
 island
 
 0.904583
 
 0.9473441
 
 Not queried
 
  0.6374517
 
       NA
 
           NA
  
 
  
 7
 
 148480080
 
 148480503
 
  ENSEMBL   UCSC 
 
  424
 
  4
 
 37259
 
 gene;island
 
 0.967825
 
 0.9726945
 
 Not queried
 
  0.6374118
 
       NA
 
           NA
  
 
  
 12
 
  53715001
 
  53715787
 
  ENSEMBL   UCSC 
 
  787
 
 11
 
 10462
 
 island
 
 0.015514
 
 0.0119186
 
 Not queried
 
  0.6374095
 
       NA
 
           NA
  
 
  
 22
 
  36019471
 
  36019632
 
  ENSEMBL   UCSC 
 
  162
 
  4
 
 27102
 
 tss
 
 0.003598
 
 0.0018321
 
 Not queried
 
  0.6373415
 
       NA
 
           NA
  
 
  
 6
 
  32156071
 
  32156527
 
  ENSEMBL   UCSC 
 
  457
 
  7
 
 34080
 
 gene
 
 0.928285
 
 0.8974906
 
 Not queried
 
  0.6372524
 
       NA
 
           NA
  
 
  
 2
 
 110371607
 
 110371903
 
  ENSEMBL   UCSC 
 
  297
 
  9
 
 23569
 
 tss;island
 
 0.015367
 
 0.0121256
 
 Not queried
 
  0.6370306
 
       NA
 
           NA
  
 
  
 2
 
 219263974
 
 219264282
 
  ENSEMBL   UCSC 
 
  309
 
  5
 
 23878
 
 tss;island
 
 0.003781
 
 0.0125815
 
 Not queried
 
  0.6370256
 
       NA
 
           NA
  
 
  
 19
 
  56739731
 
  56739868
 
  ENSEMBL   UCSC 
 
  138
 
  6
 
 21094
 
 tss
 
 0.896046
 
 0.9482139
 
 Not queried
 
  0.6368449
 
       NA
 
           NA
  
 
  
 6
 
 108880961
 
 108881022
 
  ENSEMBL   UCSC 
 
   62
 
  6
 
 35160
 
 tss;island
 
 0.006538
 
 0.0059504
 
 Not queried
 
  0.6365564
 
       NA
 
           NA
  
 
  
 3
 
  50383167
 
  50383485
 
  ENSEMBL   UCSC 
 
  319
 
  7
 
 28354
 
 tss
 
 0.013552
 
 0.0140811
 
 Not queried
 
  0.6365505
 
       NA
 
           NA
  
 
  
 4
 
   3486039
 
   3486380
 
  ENSEMBL   UCSC 
 
  342
 
  4
 
 29908
 
 gene;island
 
 0.008931
 
 0.0192884
 
 Not queried
 
  0.6361892
 
       NA
 
           NA
  
 
  
 2
 
 233470647
 
 233470724
 
  ENSEMBL   UCSC 
 
   78
 
  5
 
 23952
 
 tss
 
 0.006949
 
 0.0096988
 
 Not queried
 
  0.6359839
 
       NA
 
           NA
  
 
  
 1
 
   7122541
 
   7122970
 
  ENSEMBL   UCSC 
 
  430
 
  6
 
 186
 
 gene;island
 
 0.454715
 
 0.8328901
 
 Not queried
 
  0.6359839
 
       NA
 
           NA
  
 
  
 7
 
   5553266
 
   5553627
 
  ENSEMBL   UCSC 
 
  362
 
  5
 
 38230
 
 island
 
 0.007007
 
 0.0061892
 
 Not queried
 
  0.6358362
 
       NA
 
           NA
  
 
  
 2
 
 218621320
 
 218621580
 
  ENSEMBL   UCSC 
 
  261
 
  6
 
 23872
 
 tss
 
 0.137619
 
 0.0802848
 
 Not queried
 
  0.6358147
 
       NA
 
           NA
  
 
  
 6
 
  29579253
 
  29579492
 
  ENSEMBL   UCSC 
 
  240
 
  4
 
 33670
 
 gene
 
 0.892244
 
 0.9312266
 
 Not queried
 
  0.6357350
 
       NA
 
           NA
  
 
  
 19
 
  58661689
 
  58661962
 
  ENSEMBL   UCSC 
 
  274
 
  4
 
 20257
 
 gene;island
 
 0.007845
 
 0.0074652
 
 Not queried
 
  0.6356984
 
       NA
 
           NA
  
 
  
 16
 
  87251692
 
  87251839
 
  ENSEMBL   UCSC 
 
  148
 
  4
 
 16179
 
 island
 
 0.458662
 
 0.6103748
 
 Not queried
 
  0.6355604
 
       NA
 
           NA
  
 
  
 2
 
 120005223
 
 120005755
 
  ENSEMBL   UCSC 
 
  533
 
  5
 
 22843
 
 gene;island
 
 0.904790
 
 0.9243095
 
 Not queried
 
  0.6353173
 
       NA
 
           NA
  
 
  
 13
 
  47127193
 
  47127418
 
  ENSEMBL   UCSC 
 
  226
 
  5
 
 11717
 
 island
 
 0.011976
 
 0.0166288
 
 Not queried
 
  0.6353056
 
       NA
 
           NA
  
 
  
 1
 
  19181040
 
  19181459
 
  ENSEMBL   UCSC 
 
  420
 
  6
 
 276
 
 gene;island
 
 0.577410
 
 0.7116675
 
 Not queried
 
  0.6352828
 
       NA
 
           NA
  
 
  
 17
 
  19281512
 
  19282105
 
  ENSEMBL   UCSC 
 
  594
 
 10
 
 16480
 
 gene;island
 
 0.007038
 
 0.0070890
 
 Not queried
 
  0.6352725
 
       NA
 
           NA
  
 
  
 2
 
 198569719
 
 198570100
 
  ENSEMBL   UCSC 
 
  382
 
  8
 
 24983
 
 island
 
 0.015384
 
 0.0042625
 
 Not queried
 
  0.6351932
 
       NA
 
           NA
  
 
  
 19
 
   6481819
 
   6482169
 
  ENSEMBL   UCSC 
 
  351
 
  8
 
 20391
 
 tss
 
 0.007695
 
 0.0123527
 
 Not queried
 
  0.6349763
 
       NA
 
           NA
  
 
  
 1
 
 160388964
 
 160389374
 
  ENSEMBL   UCSC 
 
  411
 
  5
 
 844
 
 gene;island
 
 0.797492
 
 0.8238536
 
 Not queried
 
  0.6348713
 
       NA
 
           NA
  
 
  
 1
 
 228297092
 
 228297275
 
  ENSEMBL   UCSC 
 
  184
 
  5
 
 2298
 
 tss
 
 0.007763
 
 0.0054874
 
 Not queried
 
  0.6348559
 
       NA
 
           NA
  
 
  
 11
 
  46354562
 
  46355283
 
  ENSEMBL   UCSC 
 
  722
 
 10
 
 8246
 
 island
 
 0.012620
 
 0.0103413
 
 Not queried
 
  0.6348340
 
       NA
 
           NA
  
 
  
 10
 
 112258394
 
 112258680
 
  ENSEMBL   UCSC 
 
  287
 
  4
 
 4754
 
 gene;island
 
 0.010040
 
 0.0137572
 
 Not queried
 
  0.6347791
 
       NA
 
           NA
  
 
  
 17
 
  74463439
 
  74463767
 
  ENSEMBL   UCSC 
 
  329
 
  6
 
 16788
 
 gene
 
 0.634452
 
 0.7918875
 
 Not queried
 
  0.6347217
 
       NA
 
           NA
  
 
  
 6
 
  31847922
 
  31848099
 
  ENSEMBL   UCSC 
 
  178
 
  4
 
 33980
 
 gene;tss
 
 0.934996
 
 0.9568296
 
 Not queried
 
  0.6346439
 
       NA
 
           NA
  
 
  
 4
 
 147558089
 
 147558544
 
  ENSEMBL   UCSC 
 
  456
 
  4
 
 31351
 
 island
 
 0.147767
 
 0.2586073
 
 Not queried
 
  0.6344765
 
       NA
 
           NA
  
 
  
 22
 
  39898040
 
  39898620
 
  ENSEMBL   UCSC 
 
  581
 
 10
 
 27505
 
 island
 
 0.014254
 
 0.0115790
 
 Not queried
 
  0.6342602
 
       NA
 
           NA
  
 
  
 10
 
  26222654
 
  26223310
 
  ENSEMBL   UCSC 
 
  657
 
  6
 
 5616
 
 island
 
 0.140567
 
 0.0326928
 
 Not queried
 
  0.6342216
 
       NA
 
           NA
  
 
  
 2
 
 132348567
 
 132348901
 
  ENSEMBL   UCSC 
 
  335
 
  4
 
 24735
 
 island
 
 0.802452
 
 0.8836887
 
 Not queried
 
  0.6341541
 
       NA
 
           NA
  
 
  
 1
 
  92683403
 
  92683821
 
  ENSEMBL   UCSC 
 
  419
 
  7
 
 3483
 
 island
 
 0.831758
 
 0.9300409
 
 Not queried
 
  0.6340736
 
       NA
 
           NA
  
 
  
 14
 
  96001170
 
  96001369
 
  ENSEMBL   UCSC 
 
  200
 
  9
 
 12588
 
 tss;island
 
 0.005868
 
 0.0103567
 
 Not queried
 
  0.6340665
 
       NA
 
           NA
  
 
  
 7
 
 130021519
 
 130021807
 
  ENSEMBL   UCSC 
 
  289
 
  4
 
 37222
 
 gene;island
 
 0.791870
 
 0.8781580
 
 Not queried
 
  0.6339996
 
       NA
 
           NA
  
 
  
 7
 
 151038446
 
 151038742
 
  ENSEMBL   UCSC 
 
  297
 
  7
 
 38000
 
 tss;island
 
 0.039676
 
 0.0274894
 
 Not queried
 
  0.6339187
 
       NA
 
           NA
  
 
  
 6
 
  26271716
 
  26271827
 
  ENSEMBL   UCSC 
 
  112
 
  4
 
 34702
 
 tss
 
 0.002414
 
 0.0035794
 
 Not queried
 
  0.6338559
 
       NA
 
           NA
  
 
  
 2
 
 113522207
 
 113522573
 
  ENSEMBL   UCSC 
 
  367
 
  9
 
 24625
 
 island
 
 0.008737
 
 0.0134551
 
 Not queried
 
  0.6337171
 
       NA
 
           NA
  
 
  
 7
 
  43151725
 
  43152254
 
  ENSEMBL   UCSC 
 
  530
 
  7
 
 38440
 
 island
 
 0.041407
 
 0.4867341
 
 Not queried
 
  0.6336437
 
       NA
 
           NA
  
 
  
 16
 
  31228427
 
  31228555
 
  ENSEMBL   UCSC 
 
  129
 
  4
 
 15210
 
 tss
 
 0.049323
 
 0.0343231
 
 Not queried
 
  0.6336092
 
       NA
 
           NA
  
 
  
 6
 
  90660913
 
  90661373
 
  ENSEMBL   UCSC 
 
  461
 
  5
 
 34416
 
 gene
 
 0.758075
 
 0.8684103
 
 Not queried
 
  0.6335298
 
       NA
 
           NA
  
 
  
 6
 
  57036894
 
  57037243
 
  ENSEMBL   UCSC 
 
  350
 
  6
 
 36225
 
 island
 
 0.002818
 
 0.0040574
 
 Not queried
 
  0.6334532
 
       NA
 
           NA
  
 
  
 1
 
 113615856
 
 113616118
 
  ENSEMBL   UCSC 
 
  263
 
  4
 
 698
 
 gene
 
 0.006121
 
 0.0076303
 
 Not queried
 
  0.6334330
 
       NA
 
           NA
  
 
  
 15
 
  43594466
 
  43594820
 
  ENSEMBL   UCSC 
 
  355
 
  5
 
 13625
 
 tss
 
 0.501332
 
 0.5580492
 
 Not queried
 
  0.6333442
 
       NA
 
           NA
  
 
  
 6
 
  32632565
 
  32633163
 
  ENSEMBL   UCSC 
 
  599
 
 22
 
 34126
 
 gene;island
 
 0.023273
 
 0.1067367
 
 Not queried
 
  0.6332703
 
       NA
 
           NA
  
 
  
 6
 
  33244709
 
  33246488
 
  ENSEMBL   UCSC 
 
 1780
 
 52
 
 35996
 
 island
 
 0.042533
 
 0.0986044
 
 Not queried
 
  0.6330830
 
       NA
 
           NA
  
 
  
 7
 
  65970924
 
  65971372
 
  ENSEMBL   UCSC 
 
  449
 
  4
 
 38536
 
 island
 
 0.410414
 
 0.4936588
 
 Not queried
 
  0.6330806
 
       NA
 
           NA
  
 
  
 12
 
 123319216
 
 123319366
 
  ENSEMBL   UCSC 
 
  151
 
  4
 
 10089
 
 tss;island
 
 0.029782
 
 0.0393052
 
 Not queried
 
  0.6330369
 
       NA
 
           NA
  
 
  
 2
 
 208030666
 
 208030732
 
  ENSEMBL   UCSC 
 
   67
 
  4
 
 23840
 
 tss;island
 
 0.012013
 
 0.0165200
 
 Not queried
 
  0.6328227
 
       NA
 
           NA
  
 
  
 5
 
   2751822
 
   2751872
 
  ENSEMBL   UCSC 
 
   51
 
  4
 
 31975
 
 tss;island
 
 0.014520
 
 0.0185232
 
 Not queried
 
  0.6328178
 
       NA
 
           NA
  
 
  
 18
 
  47825004
 
  47825363
 
  ENSEMBL   UCSC 
 
  360
 
  5
 
 19510
 
 island
 
 0.535134
 
 0.3418036
 
 Not queried
 
  0.6327686
 
       NA
 
           NA
  
 
  
 17
 
  19647973
 
  19648414
 
  ENSEMBL   UCSC 
 
  442
 
  4
 
 16483
 
 gene;island
 
 0.028098
 
 0.0291904
 
 Not queried
 
  0.6327560
 
       NA
 
           NA
  
 
  
 16
 
  15982478
 
  15982637
 
  ENSEMBL   UCSC 
 
  160
 
  6
 
 15110
 
 tss
 
 0.001398
 
 0.0086365
 
 Not queried
 
  0.6327479
 
       NA
 
           NA
  
 
  
 4
 
  79472605
 
  79472939
 
  ENSEMBL   UCSC 
 
  335
 
  5
 
 31127
 
 island
 
 0.057199
 
 0.0434985
 
 Not queried
 
  0.6326960
 
       NA
 
           NA
  
 
  
 2
 
 177027291
 
 177027931
 
  ENSEMBL   UCSC 
 
  641
 
  7
 
 24911
 
 island
 
 0.232558
 
 0.0380675
 
 Not queried
 
  0.6326273
 
       NA
 
           NA
  
 
  
 2
 
 163175044
 
 163175403
 
  ENSEMBL   UCSC 
 
  360
 
  7
 
 23688
 
 tss
 
 0.007555
 
 0.0044890
 
 Not queried
 
  0.6326034
 
       NA
 
           NA
  
 
  
 5
 
  32585221
 
  32585549
 
  ENSEMBL   UCSC 
 
  329
 
  6
 
 32007
 
 tss
 
 0.004961
 
 0.0060106
 
 Not queried
 
  0.6325924
 
       NA
 
           NA
  
 
  
 6
 
  31240651
 
  31241000
 
  ENSEMBL   UCSC 
 
  350
 
  4
 
 34806
 
 tss;island
 
 0.017644
 
 0.0121192
 
 Not queried
 
  0.6325131
 
       NA
 
           NA
  
 
  
 11
 
  69590113
 
  69590580
 
  ENSEMBL   UCSC 
 
  468
 
  8
 
 8597
 
 island
 
 0.010962
 
 0.0137479
 
 Not queried
 
  0.6323454
 
       NA
 
           NA
  
 
  
 10
 
 134833312
 
 134833586
 
  ENSEMBL   UCSC 
 
  275
 
  6
 
 6325
 
 island
 
 0.656563
 
 0.7372144
 
 Not queried
 
  0.6323405
 
       NA
 
           NA
  
 
  
 1
 
   2104223
 
   2104886
 
  ENSEMBL   UCSC 
 
  664
 
  6
 
 67
 
 gene
 
 0.352900
 
 0.6092047
 
 Not queried
 
  0.6322653
 
       NA
 
           NA
  
 
  
 7
 
 102987991
 
 102988366
 
  ENSEMBL   UCSC 
 
  376
 
  5
 
 38811
 
 island
 
 0.008800
 
 0.0086480
 
 Not queried
 
  0.6322577
 
       NA
 
           NA
  
 
  
 9
 
 108210605
 
 108210846
 
  ENSEMBL   UCSC 
 
  242
 
  4
 
 40778
 
 gene;island
 
 0.029986
 
 0.0188672
 
 Not queried
 
  0.6322395
 
       NA
 
           NA
  
 
  
 19
 
  38826104
 
  38826367
 
  ENSEMBL   UCSC 
 
  264
 
  6
 
 20752
 
 tss
 
 0.020245
 
 0.0259575
 
 Not queried
 
  0.6322049
 
       NA
 
           NA
  
 
  
 19
 
  45970962
 
  45971240
 
  ENSEMBL   UCSC 
 
  279
 
  8
 
 20872
 
 tss
 
 0.015847
 
 0.0117551
 
 Not queried
 
  0.6320808
 
       NA
 
           NA
  
 
  
 17
 
  58755185
 
  58755368
 
  ENSEMBL   UCSC 
 
  184
 
  4
 
 16718
 
 gene
 
 0.011346
 
 0.0055396
 
 Not queried
 
  0.6320777
 
       NA
 
           NA
  
 
  
 4
 
  57371467
 
  57371927
 
  ENSEMBL   UCSC 
 
  461
 
  6
 
 30009
 
 gene
 
 0.074686
 
 0.3074990
 
 Not queried
 
  0.6320192
 
       NA
 
           NA
  
 
  
 10
 
 134698483
 
 134698937
 
  ENSEMBL   UCSC 
 
  455
 
  4
 
 6316
 
 island
 
 0.561221
 
 0.6819641
 
 Not queried
 
  0.6319080
 
       NA
 
           NA
  
 
  
 6
 
  33383065
 
  33383412
 
  ENSEMBL   UCSC 
 
  348
 
  4
 
 34280
 
 gene;island
 
 0.763020
 
 0.9449758
 
 Not queried
 
  0.6316662
 
       NA
 
           NA
  
 
  
 19
 
  19373566
 
  19373755
 
  ENSEMBL   UCSC 
 
  190
 
  5
 
 21809
 
 island
 
 0.570499
 
 0.4370932
 
 Not queried
 
  0.6315252
 
       NA
 
           NA
  
 
  
 10
 
  75910788
 
  75911181
 
  ENSEMBL   UCSC 
 
  394
 
  6
 
 5168
 
 tss;island
 
 0.003102
 
 0.0048921
 
 Not queried
 
  0.6315242
 
       NA
 
           NA
  
 
  
 12
 
 132863782
 
 132864302
 
  ENSEMBL   UCSC 
 
  521
 
  6
 
 9444
 
 gene;island
 
 0.656992
 
 0.8275891
 
 Not queried
 
  0.6315202
 
       NA
 
           NA
  
 
  
 11
 
  74303682
 
  74304091
 
  ENSEMBL   UCSC 
 
  410
 
  5
 
 6828
 
 gene
 
 0.003765
 
 0.0061716
 
 Not queried
 
  0.6314388
 
       NA
 
           NA
  
 
  
 11
 
  74303294
 
  74304091
 
  ENSEMBL   UCSC 
 
  798
 
 11
 
 8652
 
 island
 
 0.008361
 
 0.0061716
 
 Not queried
 
  0.6314388
 
       NA
 
           NA
  
 
  
 16
 
  19078849
 
  19079607
 
  ENSEMBL   UCSC 
 
  759
 
 10
 
 15733
 
 island
 
 0.005761
 
 0.0038468
 
 Not queried
 
  0.6313653
 
       NA
 
           NA
  
 
  
 2
 
  12857398
 
  12857811
 
  ENSEMBL   UCSC 
 
  414
 
  4
 
 22576
 
 gene;island
 
 0.006806
 
 0.0049847
 
 Not queried
 
  0.6313635
 
       NA
 
           NA
  
 
  
 17
 
  27038470
 
  27038861
 
  ENSEMBL   UCSC 
 
  392
 
  4
 
 16499
 
 gene
 
 0.023824
 
 0.0608138
 
 Not queried
 
  0.6313192
 
       NA
 
           NA
  
 
  
 4
 
  17513894
 
  17513991
 
  ENSEMBL   UCSC 
 
   98
 
  4
 
 30314
 
 tss
 
 0.017106
 
 0.0093902
 
 Not queried
 
  0.6313004
 
       NA
 
           NA
  
 
  
 7
 
 100895380
 
 100895581
 
  ENSEMBL   UCSC 
 
  202
 
  4
 
 38793
 
 island
 
 0.017174
 
 0.0129850
 
 Not queried
 
  0.6312987
 
       NA
 
           NA
  
 
  
 4
 
   1333422
 
   1333755
 
  ENSEMBL   UCSC 
 
  334
 
  4
 
 29869
 
 gene
 
 0.986731
 
 0.9765347
 
 Not queried
 
  0.6312840
 
       NA
 
           NA
  
 
  
 3
 
 195477778
 
 195477968
 
  ENSEMBL   UCSC 
 
  191
 
  4
 
 28093
 
 gene;island
 
 0.679139
 
 0.7995288
 
 Not queried
 
  0.6312754
 
       NA
 
           NA
  
 
  
 1
 
  37500195
 
  37500508
 
  ENSEMBL   UCSC 
 
  314
 
  5
 
 1499
 
 tss;island
 
 0.022572
 
 0.1857324
 
 Not queried
 
  0.6309750
 
       NA
 
           NA
  
 
  
 10
 
  75173098
 
  75174282
 
  ENSEMBL   UCSC 
 
 1185
 
 15
 
 5851
 
 island
 
 0.011315
 
 0.0051780
 
 Not queried
 
  0.6309680
 
       NA
 
           NA
  
 
  
 18
 
  74691097
 
  74691423
 
  ENSEMBL   UCSC 
 
  327
 
  4
 
 19131
 
 gene;island
 
 0.954302
 
 0.9744381
 
 Not queried
 
  0.6308258
 
       NA
 
           NA
  
 
  
 16
 
  55513415
 
  55513660
 
  ENSEMBL   UCSC 
 
  246
 
  4
 
 14752
 
 gene;island
 
 0.015398
 
 0.0337391
 
 Not queried
 
  0.6307553
 
       NA
 
           NA
  
 
  
 19
 
  58838208
 
  58838374
 
  ENSEMBL   UCSC 
 
  167
 
  5
 
 21138
 
 tss
 
 0.010591
 
 0.0065236
 
 Not queried
 
  0.6307147
 
       NA
 
           NA
  
 
  
 19
 
  58838208
 
  58838697
 
  ENSEMBL   UCSC 
 
  490
 
  9
 
 22503
 
 island
 
 0.006309
 
 0.0062432
 
 Not queried
 
  0.6307147
 
       NA
 
           NA
  
 
  
 3
 
 152879629
 
 152879841
 
  ENSEMBL   UCSC 
 
  213
 
  4
 
 28666
 
 tss;island
 
 0.012347
 
 0.0143834
 
 Not queried
 
  0.6306579
 
       NA
 
           NA
  
 
  
 9
 
 140268481
 
 140268774
 
  ENSEMBL   UCSC 
 
  294
 
  5
 
 40797
 
 gene;island
 
 0.835558
 
 0.8367623
 
 Not queried
 
  0.6306310
 
       NA
 
           NA
  
 
  
 19
 
  56165125
 
  56165495
 
  ENSEMBL   UCSC 
 
  371
 
  5
 
 22435
 
 island
 
 0.017103
 
 0.0084603
 
 Not queried
 
  0.6304981
 
       NA
 
           NA
  
 
  
 4
 
  90758120
 
  90758537
 
  ENSEMBL   UCSC 
 
  418
 
  7
 
 30058
 
 gene;island
 
 0.079959
 
 0.0228139
 
 Not queried
 
  0.6303419
 
       NA
 
           NA
  
 
  
 5
 
  72861037
 
  72861858
 
  ENSEMBL   UCSC 
 
  822
 
 15
 
 31644
 
 gene;tss;island
 
 0.003533
 
 0.0028665
 
 Not queried
 
  0.6302535
 
       NA
 
           NA
  
 
  
 11
 
  14666436
 
  14666839
 
  ENSEMBL   UCSC 
 
  404
 
  4
 
 6518
 
 gene;island
 
 0.002981
 
 0.0054778
 
 Not queried
 
  0.6300789
 
       NA
 
           NA
  
 
  
 3
 
  52479069
 
  52479108
 
  ENSEMBL   UCSC 
 
   40
 
  4
 
 28380
 
 tss
 
 0.107441
 
 0.0585305
 
 Not queried
 
  0.6299244
 
       NA
 
           NA
  
 
  
 11
 
 111807548
 
 111807800
 
  ENSEMBL   UCSC 
 
  253
 
  6
 
 7694
 
 tss
 
 0.026092
 
 0.0145759
 
 Not queried
 
  0.6298887
 
       NA
 
           NA
  
 
  
 11
 
 111807548
 
 111808394
 
  ENSEMBL   UCSC 
 
  847
 
 10
 
 8807
 
 island
 
 0.030316
 
 0.0173181
 
 Not queried
 
  0.6298887
 
       NA
 
           NA
  
 
  
 11
 
  64889510
 
  64889651
 
  ENSEMBL   UCSC 
 
  142
 
  4
 
 6699
 
 gene
 
 0.010619
 
 0.0153391
 
 Not queried
 
  0.6298603
 
       NA
 
           NA
  
 
  
 11
 
  13030309
 
  13030876
 
  ENSEMBL   UCSC 
 
  568
 
 10
 
 8068
 
 island
 
 0.029758
 
 0.0434139
 
 Not queried
 
  0.6298531
 
       NA
 
           NA
  
 
  
 16
 
  89033692
 
  89034292
 
  ENSEMBL   UCSC 
 
  601
 
  7
 
 14926
 
 gene;island
 
 0.165943
 
 0.2467274
 
 Not queried
 
  0.6298224
 
       NA
 
           NA
  
 
  
 11
 
  20184128
 
  20184779
 
  ENSEMBL   UCSC 
 
  652
 
  7
 
 8125
 
 island
 
 0.020443
 
 0.0496857
 
 Not queried
 
  0.6297550
 
       NA
 
           NA
  
 
  
 1
 
  61522716
 
  61523012
 
  ENSEMBL   UCSC 
 
  297
 
  4
 
 3329
 
 island
 
 0.035391
 
 0.0179678
 
 Not queried
 
  0.6297529
 
       NA
 
           NA
  
 
  
 19
 
  51017855
 
  51018014
 
  ENSEMBL   UCSC 
 
  160
 
  4
 
 20980
 
 tss;island
 
 0.465662
 
 0.6589053
 
 Not queried
 
  0.6296437
 
       NA
 
           NA
  
 
  
 15
 
  99548859
 
  99549047
 
  ENSEMBL   UCSC 
 
  189
 
  4
 
 13501
 
 gene
 
 0.400336
 
 0.4654158
 
 Not queried
 
  0.6296213
 
       NA
 
           NA
  
 
  
 7
 
 123388973
 
 123389366
 
  ENSEMBL   UCSC 
 
  394
 
  5
 
 38878
 
 island
 
 0.016555
 
 0.0160725
 
 Not queried
 
  0.6293966
 
       NA
 
           NA
  
 
  
 2
 
  26541960
 
  26542188
 
  ENSEMBL   UCSC 
 
  229
 
  4
 
 22610
 
 gene;tss
 
 0.403233
 
 0.6242079
 
 Not queried
 
  0.6292635
 
       NA
 
           NA
  
 
  
 6
 
  34206400
 
  34206683
 
  ENSEMBL   UCSC 
 
  284
 
  4
 
 34302
 
 gene;island
 
 0.005385
 
 0.0036885
 
 Not queried
 
  0.6292247
 
       NA
 
           NA
  
 
  
 16
 
    809910
 
    810292
 
  ENSEMBL   UCSC 
 
  383
 
  5
 
 14981
 
 tss
 
 0.399523
 
 0.4987169
 
 Not queried
 
  0.6291409
 
       NA
 
           NA
  
 
  
 3
 
  49711033
 
  49711383
 
  ENSEMBL   UCSC 
 
  351
 
 10
 
 28331
 
 tss
 
 0.008560
 
 0.0134363
 
 Not queried
 
  0.6291022
 
       NA
 
           NA
  
 
  
 5
 
 132361924
 
 132362247
 
  ENSEMBL   UCSC 
 
  324
 
  5
 
 33106
 
 island
 
 0.007997
 
 0.0034247
 
 Not queried
 
  0.6289580
 
       NA
 
           NA
  
 
  
 2
 
 176947764
 
 176948105
 
  ENSEMBL   UCSC 
 
  342
 
  4
 
 22934
 
 gene;island
 
 0.042772
 
 0.0571754
 
 Not queried
 
  0.6288953
 
       NA
 
           NA
  
 
  
 6
 
  27782909
 
  27783331
 
  ENSEMBL   UCSC 
 
  423
 
  5
 
 34721
 
 tss;island
 
 0.048977
 
 0.0290733
 
 Not queried
 
  0.6287650
 
       NA
 
           NA
  
 
  
 3
 
   3221439
 
   3221832
 
  ENSEMBL   UCSC 
 
  394
 
  9
 
 28114
 
 tss
 
 0.011503
 
 0.0175032
 
 Not queried
 
  0.6285526
 
       NA
 
           NA
  
 
  
 2
 
  26987182
 
  26987250
 
  ENSEMBL   UCSC 
 
   69
 
  5
 
 22616
 
 gene
 
 0.002380
 
 0.0046098
 
 Not queried
 
  0.6285137
 
       NA
 
           NA
  
 
  
 3
 
  50304049
 
  50304463
 
  ENSEMBL   UCSC 
 
  415
 
  8
 
 28344
 
 tss
 
 0.426802
 
 0.6452958
 
 Not queried
 
  0.6284959
 
       NA
 
           NA
  
 
  
 19
 
  28284952
 
  28285395
 
  ENSEMBL   UCSC 
 
  444
 
  6
 
 20667
 
 tss
 
 0.014884
 
 0.0114233
 
 Not queried
 
  0.6284778
 
       NA
 
           NA
  
 
  
 19
 
  28284698
 
  28285395
 
  ENSEMBL   UCSC 
 
  698
 
 10
 
 21850
 
 island
 
 0.008672
 
 0.0074251
 
 Not queried
 
  0.6284778
 
       NA
 
           NA
  
 
  
 1
 
 151763548
 
 151764147
 
  ENSEMBL   UCSC 
 
  600
 
  6
 
 1932
 
 tss;island
 
 0.009331
 
 0.0184293
 
 Not queried
 
  0.6284341
 
       NA
 
           NA
  
 
  
 12
 
 100661322
 
 100661778
 
  ENSEMBL   UCSC 
 
  457
 
  9
 
 9940
 
 tss;island
 
 0.014813
 
 0.0145139
 
 Not queried
 
  0.6284172
 
       NA
 
           NA
  
 
  
 12
 
  89919758
 
  89920041
 
  ENSEMBL   UCSC 
 
  284
 
  8
 
 9909
 
 tss;island
 
 0.001952
 
 0.0019981
 
 Not queried
 
  0.6282576
 
       NA
 
           NA
  
 
  
 1
 
 235667903
 
 235668099
 
  ENSEMBL   UCSC 
 
  197
 
  6
 
 2335
 
 tss;island
 
 0.014914
 
 0.0067463
 
 Not queried
 
  0.6282032
 
       NA
 
           NA
  
 
  
 5
 
 147443067
 
 147443347
 
  ENSEMBL   UCSC 
 
  281
 
  4
 
 32410
 
 tss
 
 0.002899
 
 0.0310707
 
 Not queried
 
  0.6281815
 
       NA
 
           NA
  
 
  
 1
 
  23696021
 
  23696209
 
  ENSEMBL   UCSC 
 
  189
 
  4
 
 312
 
 gene;tss;island
 
 0.008416
 
 0.0115407
 
 Not queried
 
  0.6281652
 
       NA
 
           NA
  
 
  
 1
 
  26487591
 
  26488129
 
  ENSEMBL   UCSC 
 
  539
 
  5
 
 337
 
 gene;island
 
 0.388756
 
 0.6947515
 
 Not queried
 
  0.6280193
 
       NA
 
           NA
  
 
  
 8
 
 101322518
 
 101322791
 
  ENSEMBL   UCSC 
 
  274
 
  4
 
 39842
 
 tss;island
 
 0.003985
 
 0.0036773
 
 Not queried
 
  0.6277202
 
       NA
 
           NA
  
 
  
 7
 
 157361396
 
 157361759
 
  ENSEMBL   UCSC 
 
  364
 
  6
 
 37327
 
 gene;island
 
 0.047775
 
 0.0678909
 
 Not queried
 
  0.6276280
 
       NA
 
           NA
  
 
  
 22
 
  31555670
 
  31556357
 
  ENSEMBL   UCSC 
 
  688
 
 15
 
 27425
 
 island
 
 0.012559
 
 0.0106621
 
 Not queried
 
  0.6276199
 
       NA
 
           NA
  
 
  
 13
 
 114875001
 
 114875412
 
  ENSEMBL   UCSC 
 
  412
 
  5
 
 11314
 
 gene;island
 
 0.959988
 
 0.9666585
 
 Not queried
 
  0.6275310
 
       NA
 
           NA
  
 
  
 6
 
 137817512
 
 137817846
 
  ENSEMBL   UCSC 
 
  335
 
  4
 
 36509
 
 island
 
 0.028327
 
 0.0274962
 
 Not queried
 
  0.6275086
 
       NA
 
           NA
  
 
  
 7
 
   6144127
 
   6144399
 
  ENSEMBL   UCSC 
 
  273
 
  7
 
 37431
 
 tss;island
 
 0.008893
 
 0.0082534
 
 Not queried
 
  0.6275022
 
       NA
 
           NA
  
 
  
 5
 
 114631987
 
 114632301
 
  ENSEMBL   UCSC 
 
  315
 
  6
 
 32225
 
 tss
 
 0.043015
 
 0.0202090
 
 Not queried
 
  0.6274352
 
       NA
 
           NA
  
 
  
 7
 
 123389159
 
 123389366
 
  ENSEMBL   UCSC 
 
  208
 
  4
 
 37870
 
 tss
 
 0.037541
 
 0.0258686
 
 Not queried
 
  0.6273810
 
       NA
 
           NA
  
 
  
 5
 
 130970657
 
 130970833
 
  ENSEMBL   UCSC 
 
  177
 
  5
 
 31742
 
 gene
 
 0.004674
 
 0.0044046
 
 Not queried
 
  0.6270150
 
       NA
 
           NA
  
 
  
 3
 
  10157025
 
  10157352
 
  ENSEMBL   UCSC 
 
  328
 
  8
 
 28872
 
 island
 
 0.004424
 
 0.0064078
 
 Not queried
 
  0.6268212
 
       NA
 
           NA
  
 
  
 11
 
 134257429
 
 134257744
 
  ENSEMBL   UCSC 
 
  316
 
  4
 
 7000
 
 gene;island
 
 0.700405
 
 0.8346172
 
 Not queried
 
  0.6268111
 
       NA
 
           NA
  
 
  
 14
 
  91976487
 
  91976900
 
  ENSEMBL   UCSC 
 
  414
 
  9
 
 13081
 
 island
 
 0.011777
 
 0.0083126
 
 Not queried
 
  0.6267903
 
       NA
 
           NA
  
 
  
 10
 
   5541423
 
   5541823
 
  ENSEMBL   UCSC 
 
  401
 
  5
 
 5519
 
 island
 
 0.285307
 
 0.5410366
 
 Not queried
 
  0.6267158
 
       NA
 
           NA
  
 
  
 20
 
   2384240
 
   2384464
 
  ENSEMBL   UCSC 
 
  225
 
  4
 
 25332
 
 gene;island
 
 0.825411
 
 0.8510169
 
 Not queried
 
  0.6266260
 
       NA
 
           NA
  
 
  
 7
 
   3341524
 
   3342048
 
  ENSEMBL   UCSC 
 
  525
 
  6
 
 36823
 
 gene;island
 
 0.086086
 
 0.0877329
 
 Not queried
 
  0.6265523
 
       NA
 
           NA
  
 
  
 17
 
  18585705
 
  18585893
 
  ENSEMBL   UCSC 
 
  189
 
  5
 
 17180
 
 tss;island
 
 0.009666
 
 0.0082092
 
 Not queried
 
  0.6264424
 
       NA
 
           NA
  
 
  
 19
 
  10047197
 
  10047420
 
  ENSEMBL   UCSC 
 
  224
 
  5
 
 20434
 
 tss
 
 0.015552
 
 0.0250293
 
 Not queried
 
  0.6263925
 
       NA
 
           NA
  
 
  
 12
 
 133286734
 
 133286970
 
  ENSEMBL   UCSC 
 
  237
 
  4
 
 10133
 
 tss;island
 
 0.012761
 
 0.0131835
 
 Not queried
 
  0.6262801
 
       NA
 
           NA
  
 
  
 4
 
  99917380
 
  99917608
 
  ENSEMBL   UCSC 
 
  229
 
  4
 
 30066
 
 gene;island
 
 0.005972
 
 0.0029154
 
 Not queried
 
  0.6262534
 
       NA
 
           NA
  
 
  
 19
 
  39340493
 
  39340813
 
  ENSEMBL   UCSC 
 
  321
 
  6
 
 20021
 
 gene;island
 
 0.012517
 
 0.0029119
 
 Not queried
 
  0.6262447
 
       NA
 
           NA
  
 
  
 17
 
  61615504
 
  61615913
 
  ENSEMBL   UCSC 
 
  410
 
  4
 
 16728
 
 gene;island
 
 0.071213
 
 0.0449955
 
 Not queried
 
  0.6262423
 
       NA
 
           NA
  
 
  
 17
 
  43568337
 
  43568545
 
  ENSEMBL   UCSC 
 
  209
 
  4
 
 17431
 
 tss;island
 
 0.014953
 
 0.0101055
 
 Not queried
 
  0.6262412
 
       NA
 
           NA
  
 
  
 12
 
  53688851
 
  53689090
 
  ENSEMBL   UCSC 
 
  240
 
  6
 
 9737
 
 tss;island
 
 0.014778
 
 0.0044623
 
 Not queried
 
  0.6262080
 
       NA
 
           NA
  
 
  
 16
 
  56228114
 
  56228901
 
  ENSEMBL   UCSC 
 
  788
 
 12
 
 14756
 
 gene;island
 
 0.005558
 
 0.0071841
 
 Not queried
 
  0.6261891
 
       NA
 
           NA
  
 
  
 3
 
 137484220
 
 137484586
 
  ENSEMBL   UCSC 
 
  367
 
  4
 
 29513
 
 island
 
 0.023805
 
 0.0508293
 
 Not queried
 
  0.6261245
 
       NA
 
           NA
  
 
  
 12
 
  54422127
 
  54422488
 
  ENSEMBL   UCSC 
 
  362
 
  5
 
 9159
 
 gene;island
 
 0.017966
 
 0.0562708
 
 Not queried
 
  0.6258589
 
       NA
 
           NA
  
 
  
 1
 
   3143682
 
   3144209
 
  ENSEMBL   UCSC 
 
  528
 
  5
 
 109
 
 gene
 
 0.290183
 
 0.3868215
 
 Not queried
 
  0.6257185
 
       NA
 
           NA
  
 
  
 12
 
  65672405
 
  65672531
 
  ENSEMBL   UCSC 
 
  127
 
  4
 
 10599
 
 island
 
 0.014879
 
 0.0115511
 
 Not queried
 
  0.6256515
 
       NA
 
           NA
  
 
  
 2
 
 183731563
 
 183732062
 
  ENSEMBL   UCSC 
 
  500
 
  7
 
 23766
 
 tss
 
 0.038179
 
 0.0234759
 
 Not queried
 
  0.6255933
 
       NA
 
           NA
  
 
  
 6
 
 125474720
 
 125475385
 
  ENSEMBL   UCSC 
 
  666
 
 14
 
 35209
 
 tss;island
 
 0.011325
 
 0.0131284
 
 Not queried
 
  0.6255867
 
       NA
 
           NA
  
 
  
 3
 
 157827682
 
 157827874
 
  ENSEMBL   UCSC 
 
  193
 
  5
 
 28683
 
 tss
 
 0.012565
 
 0.0075876
 
 Not queried
 
  0.6255250
 
       NA
 
           NA
  
 
  
 1
 
  57890317
 
  57890636
 
  ENSEMBL   UCSC 
 
  320
 
  4
 
 555
 
 gene;island
 
 0.015410
 
 0.0123473
 
 Not queried
 
  0.6254513
 
       NA
 
           NA
  
 
  
 7
 
 150945869
 
 150946199
 
  ENSEMBL   UCSC 
 
  331
 
  5
 
 37289
 
 gene;tss;island
 
 0.013048
 
 0.0140307
 
 Not queried
 
  0.6253657
 
       NA
 
           NA
  
 
  
 2
 
 223520845
 
 223521104
 
  ENSEMBL   UCSC 
 
  260
 
  8
 
 23919
 
 tss
 
 0.003832
 
 0.0050506
 
 Not queried
 
  0.6252530
 
       NA
 
           NA
  
 
  
 17
 
  72968968
 
  72969066
 
  ENSEMBL   UCSC 
 
   99
 
  5
 
 17638
 
 tss;island
 
 0.015283
 
 0.0091878
 
 Not queried
 
  0.6251775
 
       NA
 
           NA
  
 
  
 2
 
 105953705
 
 105953959
 
  ENSEMBL   UCSC 
 
  255
 
  5
 
 23554
 
 tss
 
 0.014276
 
 0.0135040
 
 Not queried
 
  0.6250612
 
       NA
 
           NA
  
 
  
 5
 
 153862183
 
 153862450
 
  ENSEMBL   UCSC 
 
  268
 
  4
 
 33332
 
 island
 
 0.032255
 
 0.0112591
 
 Not queried
 
  0.6249497
 
       NA
 
           NA
  
 
  
 3
 
   9745369
 
   9745610
 
  ENSEMBL   UCSC 
 
  242
 
  4
 
 28859
 
 island
 
 0.226730
 
 0.1421391
 
 Not queried
 
  0.6247285
 
       NA
 
           NA
  
 
  
 14
 
 101347044
 
 101347358
 
  ENSEMBL   UCSC 
 
  315
 
  6
 
 12210
 
 gene;tss;island
 
 0.398071
 
 0.3406283
 
 Not queried
 
  0.6246637
 
       NA
 
           NA
  
 
  
 6
 
  30035104
 
  30035241
 
  ENSEMBL   UCSC 
 
  138
 
  4
 
 33714
 
 gene;island
 
 0.019228
 
 0.0314384
 
 Not queried
 
  0.6246008
 
       NA
 
           NA
  
 
  
 1
 
 244624521
 
 244624883
 
  ENSEMBL   UCSC 
 
  363
 
  6
 
 4394
 
 island
 
 0.019861
 
 0.0201886
 
 Not queried
 
  0.6245015
 
       NA
 
           NA
  
 
  
 6
 
  31506568
 
  31506774
 
  ENSEMBL   UCSC 
 
  207
 
  5
 
 33892
 
 gene;island
 
 0.978507
 
 0.9674291
 
 Not queried
 
  0.6243312
 
       NA
 
           NA
  
 
  
 14
 
 102430893
 
 102431106
 
  ENSEMBL   UCSC 
 
  214
 
  4
 
 12219
 
 gene
 
 0.009564
 
 0.0090266
 
 Not queried
 
  0.6242771
 
       NA
 
           NA
  
 
  
 6
 
 110501627
 
 110501899
 
  ENSEMBL   UCSC 
 
  273
 
  4
 
 34444
 
 gene
 
 0.002594
 
 0.0035677
 
 Not queried
 
  0.6242492
 
       NA
 
           NA
  
 
  
 12
 
 121975121
 
 121975669
 
  ENSEMBL   UCSC 
 
  549
 
  5
 
 9369
 
 gene;island
 
 0.007393
 
 0.0072185
 
 Not queried
 
  0.6242318
 
       NA
 
           NA
  
 
  
 13
 
  81229232
 
  81229541
 
  ENSEMBL   UCSC 
 
  310
 
  4
 
 11804
 
 island
 
 0.477197
 
 0.6095296
 
 Not queried
 
  0.6242117
 
       NA
 
           NA
  
 
  
 16
 
  77822083
 
  77822500
 
  ENSEMBL   UCSC 
 
  418
 
  9
 
 16111
 
 island
 
 0.017566
 
 0.0261855
 
 Not queried
 
  0.6241708
 
       NA
 
           NA
  
 
  
 2
 
  95537044
 
  95537210
 
  ENSEMBL   UCSC 
 
  167
 
  5
 
 23506
 
 tss
 
 0.364760
 
 0.4160018
 
 Not queried
 
  0.6240725
 
       NA
 
           NA
  
 
  
 12
 
  14518655
 
  14518974
 
  ENSEMBL   UCSC 
 
  320
 
  4
 
 9064
 
 gene
 
 0.028979
 
 0.0251204
 
 Not queried
 
  0.6239844
 
       NA
 
           NA
  
 
  
 1
 
  27339413
 
  27339786
 
  ENSEMBL   UCSC 
 
  374
 
  7
 
 1404
 
 tss
 
 0.017929
 
 0.0106966
 
 Not queried
 
  0.6238502
 
       NA
 
           NA
  
 
  
 1
 
  27339287
 
  27339786
 
  ENSEMBL   UCSC 
 
  500
 
  9
 
 2909
 
 island
 
 0.017929
 
 0.0106966
 
 Not queried
 
  0.6238502
 
       NA
 
           NA
  
 
  
 7
 
 158789723
 
 158790115
 
  ENSEMBL   UCSC 
 
  393
 
  4
 
 39158
 
 island
 
 0.877275
 
 0.9251988
 
 Not queried
 
  0.6235792
 
       NA
 
           NA
  
 
  
 9
 
 135906154
 
 135906520
 
  ENSEMBL   UCSC 
 
  367
 
  4
 
 40790
 
 gene
 
 0.017272
 
 0.0092342
 
 Not queried
 
  0.6234752
 
       NA
 
           NA
  
 
  
 11
 
  33061388
 
  33061907
 
  ENSEMBL   UCSC 
 
  520
 
  5
 
 6573
 
 gene;island
 
 0.003842
 
 0.0045789
 
 Not queried
 
  0.6234551
 
       NA
 
           NA
  
 
  
 19
 
   2717293
 
   2717615
 
  ENSEMBL   UCSC 
 
  323
 
  4
 
 19700
 
 gene;island
 
 0.949941
 
 0.9698525
 
 Not queried
 
  0.6233812
 
       NA
 
           NA
  
 
  
 1
 
 175162044
 
 175162153
 
  ENSEMBL   UCSC 
 
  110
 
  7
 
 894
 
 gene
 
 0.010867
 
 0.0068296
 
 Not queried
 
  0.6233149
 
       NA
 
           NA
  
 
  
 2
 
  73114144
 
  73114610
 
  ENSEMBL   UCSC 
 
  467
 
 10
 
 24406
 
 island
 
 0.017774
 
 0.0094699
 
 Not queried
 
  0.6232754
 
       NA
 
           NA
  
 
  
 12
 
 104350833
 
 104351300
 
  ENSEMBL   UCSC 
 
  468
 
  6
 
 10733
 
 island
 
 0.014083
 
 0.0099720
 
 Not queried
 
  0.6228618
 
       NA
 
           NA
  
 
  
 19
 
  58238850
 
  58239222
 
  ENSEMBL   UCSC 
 
  373
 
  6
 
 22481
 
 island
 
 0.006284
 
 0.0057913
 
 Not queried
 
  0.6227451
 
       NA
 
           NA
  
 
  
 15
 
  71185186
 
  71185336
 
  ENSEMBL   UCSC 
 
  151
 
  4
 
 13402
 
 gene;tss;island
 
 0.005677
 
 0.0059711
 
 Not queried
 
  0.6226575
 
       NA
 
           NA
  
 
  
 12
 
 117628187
 
 117628589
 
  ENSEMBL   UCSC 
 
  403
 
  8
 
 10863
 
 island
 
 0.022039
 
 0.0105965
 
 Not queried
 
  0.6225503
 
       NA
 
           NA
  
 
  
 4
 
   2936583
 
   2936740
 
  ENSEMBL   UCSC 
 
  158
 
  7
 
 30255
 
 tss;island
 
 0.018002
 
 0.0120973
 
 Not queried
 
  0.6224868
 
       NA
 
           NA
  
 
  
 5
 
   6632973
 
   6633577
 
  ENSEMBL   UCSC 
 
  605
 
 13
 
 31982
 
 tss;island
 
 0.010327
 
 0.0119324
 
 Not queried
 
  0.6223364
 
       NA
 
           NA
  
 
  
 6
 
 169026928
 
 169027218
 
  ENSEMBL   UCSC 
 
  291
 
  5
 
 34565
 
 gene
 
 0.558120
 
 0.6942350
 
 Not queried
 
  0.6222558
 
       NA
 
           NA
  
 
  
 14
 
  61190829
 
  61191253
 
  ENSEMBL   UCSC 
 
  425
 
  6
 
 12441
 
 tss
 
 0.017661
 
 0.0331170
 
 Not queried
 
  0.6222460
 
       NA
 
           NA
  
 
  
 6
 
   4021316
 
   4021498
 
  ENSEMBL   UCSC 
 
  183
 
  7
 
 34603
 
 tss
 
 0.011368
 
 0.0020893
 
 Not queried
 
  0.6222274
 
       NA
 
           NA
  
 
  
 6
 
   4021316
 
   4021748
 
  ENSEMBL   UCSC 
 
  433
 
  9
 
 35391
 
 island
 
 0.011368
 
 0.0028285
 
 Not queried
 
  0.6222274
 
       NA
 
           NA
  
 
  
 17
 
  27055507
 
  27055725
 
  ENSEMBL   UCSC 
 
  219
 
  5
 
 17222
 
 tss;island
 
 0.001835
 
 0.0042386
 
 Not queried
 
  0.6222151
 
       NA
 
           NA
  
 
  
 4
 
 113207063
 
 113207418
 
  ENSEMBL   UCSC 
 
  356
 
  8
 
 30552
 
 tss
 
 0.012005
 
 0.0073361
 
 Not queried
 
  0.6221859
 
       NA
 
           NA
  
 
  
 8
 
  82644747
 
  82645116
 
  ENSEMBL   UCSC 
 
  370
 
  4
 
 39350
 
 gene
 
 0.034762
 
 0.0179124
 
 Not queried
 
  0.6221191
 
       NA
 
           NA
  
 
  
 12
 
  85430025
 
  85430336
 
  ENSEMBL   UCSC 
 
  312
 
 10
 
 9899
 
 tss
 
 0.002106
 
 0.0018019
 
 Not queried
 
  0.6221023
 
       NA
 
           NA
  
 
  
 7
 
  72722923
 
  72723152
 
  ENSEMBL   UCSC 
 
  230
 
  5
 
 37631
 
 tss
 
 0.005340
 
 0.0040668
 
 Not queried
 
  0.6220480
 
       NA
 
           NA
  
 
  
 19
 
   8408146
 
   8408509
 
  ENSEMBL   UCSC 
 
  364
 
  8
 
 20417
 
 tss
 
 0.077174
 
 0.0445142
 
 Not queried
 
  0.6219792
 
       NA
 
           NA
  
 
  
 14
 
  21566698
 
  21567646
 
  ENSEMBL   UCSC 
 
  949
 
  7
 
 12011
 
 gene;tss;island
 
 0.045472
 
 0.0295872
 
 Not queried
 
  0.6219396
 
       NA
 
           NA
  
 
  
 2
 
  75788038
 
  75788552
 
  ENSEMBL   UCSC 
 
  515
 
  9
 
 22752
 
 gene;island
 
 0.013941
 
 0.0153384
 
 Not queried
 
  0.6219312
 
       NA
 
           NA
  
 
  
 22
 
  39101840
 
  39102110
 
  ENSEMBL   UCSC 
 
  271
 
  4
 
 26872
 
 gene
 
 0.008757
 
 0.0027157
 
 Not queried
 
  0.6216622
 
       NA
 
           NA
  
 
  
 16
 
  10674326
 
  10674663
 
  ENSEMBL   UCSC 
 
  338
 
  6
 
 15694
 
 island
 
 0.005327
 
 0.0035773
 
 Not queried
 
  0.6216596
 
       NA
 
           NA
  
 
  
 13
 
  49822161
 
  49822535
 
  ENSEMBL   UCSC 
 
  375
 
  4
 
 11164
 
 gene
 
 0.019233
 
 0.0140057
 
 Not queried
 
  0.6216407
 
       NA
 
           NA
  
 
  
 13
 
  53423940
 
  53424127
 
  ENSEMBL   UCSC 
 
  188
 
  4
 
 11466
 
 tss;island
 
 0.065009
 
 0.1442576
 
 Not queried
 
  0.6215695
 
       NA
 
           NA
  
 
  
 19
 
   2650727
 
   2650863
 
  ENSEMBL   UCSC 
 
  137
 
  4
 
 19699
 
 gene;island
 
 0.922657
 
 0.9283863
 
 Not queried
 
  0.6215522
 
       NA
 
           NA
  
 
  
 17
 
  76719591
 
  76719640
 
  ENSEMBL   UCSC 
 
   50
 
  4
 
 16825
 
 gene
 
 0.955603
 
 0.9386487
 
 Not queried
 
  0.6214940
 
       NA
 
           NA
  
 
  
 17
 
   2951666
 
   2951736
 
  ENSEMBL   UCSC 
 
   71
 
  4
 
 17850
 
 island
 
 0.727836
 
 0.8720962
 
 Not queried
 
  0.6214200
 
       NA
 
           NA
  
 
  
 12
 
   6898682
 
   6898975
 
  ENSEMBL   UCSC 
 
  294
 
  4
 
 9033
 
 gene
 
 0.245177
 
 0.2865514
 
 Not queried
 
  0.6213601
 
       NA
 
           NA
  
 
  
 16
 
  12662374
 
  12662659
 
  ENSEMBL   UCSC 
 
  286
 
  4
 
 14671
 
 gene;island
 
 0.983792
 
 0.9715768
 
 Not queried
 
  0.6212164
 
       NA
 
           NA
  
 
  
 13
 
  21893289
 
  21893662
 
  ENSEMBL   UCSC 
 
  374
 
  4
 
 11588
 
 island
 
 0.953387
 
 0.9692894
 
 Not queried
 
  0.6211483
 
       NA
 
           NA
  
 
  
 6
 
  99842082
 
  99842368
 
  ENSEMBL   UCSC 
 
  287
 
  7
 
 35133
 
 tss
 
 0.223716
 
 0.5378215
 
 Not queried
 
  0.6211379
 
       NA
 
           NA
  
 
  
 6
 
  99841605
 
  99842368
 
  ENSEMBL   UCSC 
 
  764
 
 11
 
 36351
 
 island
 
 0.079275
 
 0.0566012
 
 Not queried
 
  0.6211379
 
       NA
 
           NA
  
 
  
 1
 
  54954752
 
  54955149
 
  ENSEMBL   UCSC 
 
  398
 
  4
 
 3297
 
 island
 
 0.029253
 
 0.0141212
 
 Not queried
 
  0.6211058
 
       NA
 
           NA
  
 
  
 15
 
  42565522
 
  42565903
 
  ENSEMBL   UCSC 
 
  382
 
  9
 
 13615
 
 tss;island
 
 0.006622
 
 0.0062827
 
 Not queried
 
  0.6210472
 
       NA
 
           NA
  
 
  
 17
 
  49337186
 
  49337787
 
  ENSEMBL   UCSC 
 
  602
 
  8
 
 17512
 
 tss;island
 
 0.009047
 
 0.0079267
 
 Not queried
 
  0.6209271
 
       NA
 
           NA
  
 
  
 2
 
  53994559
 
  53995114
 
  ENSEMBL   UCSC 
 
  556
 
  8
 
 22678
 
 gene;island
 
 0.008314
 
 0.0045811
 
 Not queried
 
  0.6207929
 
       NA
 
           NA
  
 
  
 2
 
  53994559
 
  53994856
 
  ENSEMBL   UCSC 
 
  298
 
  6
 
 23350
 
 tss
 
 0.006719
 
 0.0039839
 
 Not queried
 
  0.6207929
 
       NA
 
           NA
  
 
  
 8
 
 109260982
 
 109261275
 
  ENSEMBL   UCSC 
 
  294
 
  9
 
 39867
 
 tss
 
 0.005954
 
 0.0026518
 
 Not queried
 
  0.6206700
 
       NA
 
           NA
  
 
  
 1
 
 120254619
 
 120255104
 
  ENSEMBL   UCSC 
 
  486
 
  5
 
 718
 
 gene;island
 
 0.012180
 
 0.0069216
 
 Not queried
 
  0.6206567
 
       NA
 
           NA
  
 
  
 20
 
   3776212
 
   3776357
 
  ENSEMBL   UCSC 
 
  146
 
  7
 
 25568
 
 tss
 
 0.010117
 
 0.0376973
 
 Not queried
 
  0.6206232
 
       NA
 
           NA
  
 
  
 12
 
  76741864
 
  76742207
 
  ENSEMBL   UCSC 
 
  344
 
  5
 
 9224
 
 gene
 
 0.009867
 
 0.0100857
 
 Not queried
 
  0.6205838
 
       NA
 
           NA
  
 
  
 15
 
  48470606
 
  48470973
 
  ENSEMBL   UCSC 
 
  368
 
  6
 
 13654
 
 tss
 
 0.427480
 
 0.2186868
 
 Not queried
 
  0.6204418
 
       NA
 
           NA
  
 
  
 1
 
  93250584
 
  93251218
 
  ENSEMBL   UCSC 
 
  635
 
  9
 
 637
 
 gene;island
 
 0.024496
 
 0.0264723
 
 Not queried
 
  0.6204317
 
       NA
 
           NA
  
 
  
 1
 
 164528441
 
 164528685
 
  ENSEMBL   UCSC 
 
  245
 
  6
 
 2062
 
 tss
 
 0.077633
 
 0.0178522
 
 Not queried
 
  0.6202820
 
       NA
 
           NA
  
 
  
 19
 
  54982500
 
  54982896
 
  ENSEMBL   UCSC 
 
  397
 
  4
 
 20224
 
 gene;island
 
 0.015506
 
 0.0278822
 
 Not queried
 
  0.6201124
 
       NA
 
           NA
  
 
  
 10
 
  71871364
 
  71871633
 
  ENSEMBL   UCSC 
 
  270
 
  4
 
 4617
 
 gene
 
 0.981321
 
 0.9855493
 
 Not queried
 
  0.6200049
 
       NA
 
           NA
  
 
  
 19
 
  22235272
 
  22235551
 
  ENSEMBL   UCSC 
 
  280
 
  5
 
 19964
 
 gene
 
 0.097403
 
 0.0953971
 
 Not queried
 
  0.6199990
 
       NA
 
           NA
  
 
  
 8
 
 145910546
 
 145910754
 
  ENSEMBL   UCSC 
 
  209
 
  4
 
 40745
 
 island
 
 0.109627
 
 0.0684827
 
 Not queried
 
  0.6199411
 
       NA
 
           NA
  
 
  
 8
 
 100905835
 
 100906373
 
  ENSEMBL   UCSC 
 
  539
 
 10
 
 40491
 
 island
 
 0.013549
 
 0.0195265
 
 Not queried
 
  0.6199314
 
       NA
 
           NA
  
 
  
 5
 
 114632606
 
 114632793
 
  ENSEMBL   UCSC 
 
  188
 
  6
 
 32226
 
 tss;island
 
 0.032502
 
 0.0340082
 
 Not queried
 
  0.6198579
 
       NA
 
           NA
  
 
  
 2
 
  95742689
 
  95743038
 
  ENSEMBL   UCSC 
 
  350
 
  5
 
 24511
 
 island
 
 0.023621
 
 0.0216133
 
 Not queried
 
  0.6198527
 
       NA
 
           NA
  
 
  
 17
 
  38334161
 
  38334186
 
  ENSEMBL   UCSC 
 
   26
 
  5
 
 17326
 
 tss;island
 
 0.006856
 
 0.0098887
 
 Not queried
 
  0.6197713
 
       NA
 
           NA
  
 
  
 3
 
 186285208
 
 186285398
 
  ENSEMBL   UCSC 
 
  191
 
  6
 
 28773
 
 tss
 
 0.009871
 
 0.0070783
 
 Not queried
 
  0.6197119
 
       NA
 
           NA
  
 
  
 17
 
  79895357
 
  79895584
 
  ENSEMBL   UCSC 
 
  228
 
  5
 
 17761
 
 tss;island
 
 0.006414
 
 0.0040459
 
 Not queried
 
  0.6196241
 
       NA
 
           NA
  
 
  
 17
 
   4634429
 
   4634708
 
  ENSEMBL   UCSC 
 
  280
 
  8
 
 17030
 
 tss
 
 0.003007
 
 0.0014921
 
 Not queried
 
  0.6195524
 
       NA
 
           NA
  
 
  
 1
 
  26232706
 
  26233035
 
  ENSEMBL   UCSC 
 
  330
 
  7
 
 335
 
 gene;tss;island
 
 0.003101
 
 0.0046368
 
 Not queried
 
  0.6194725
 
       NA
 
           NA
  
 
  
 2
 
 114256392
 
 114256454
 
  ENSEMBL   UCSC 
 
   63
 
  4
 
 23583
 
 tss;island
 
 0.615331
 
 0.7863955
 
 Not queried
 
  0.6194584
 
       NA
 
           NA
  
 
  
 7
 
  23749852
 
  23750085
 
  ENSEMBL   UCSC 
 
  234
 
  7
 
 36921
 
 gene
 
 0.906915
 
 0.9462346
 
 Not queried
 
  0.6194257
 
       NA
 
           NA
  
 
  
 8
 
 119964440
 
 119964729
 
  ENSEMBL   UCSC 
 
  290
 
  6
 
 39884
 
 tss
 
 0.460300
 
 0.3914831
 
 Not queried
 
  0.6193897
 
       NA
 
           NA
  
 
  
 4
 
  42154912
 
  42154983
 
  ENSEMBL   UCSC 
 
   72
 
  6
 
 30366
 
 tss
 
 0.009918
 
 0.0120974
 
 Not queried
 
  0.6193336
 
       NA
 
           NA
  
 
  
 7
 
  39649224
 
  39649443
 
  ENSEMBL   UCSC 
 
  220
 
  5
 
 36974
 
 gene;island
 
 0.079663
 
 0.0381039
 
 Not queried
 
  0.6193147
 
       NA
 
           NA
  
 
  
 19
 
   7599072
 
   7599173
 
  ENSEMBL   UCSC 
 
  102
 
  5
 
 19778
 
 gene
 
 0.019749
 
 0.0111695
 
 Not queried
 
  0.6192402
 
       NA
 
           NA
  
 
  
 6
 
  42016671
 
  42016713
 
  ENSEMBL   UCSC 
 
   43
 
  4
 
 34977
 
 tss;island
 
 0.003805
 
 0.0042501
 
 Not queried
 
  0.6192337
 
       NA
 
           NA
  
 
  
 22
 
  19420050
 
  19420336
 
  ENSEMBL   UCSC 
 
  287
 
  5
 
 26786
 
 gene
 
 0.005943
 
 0.0027331
 
 Not queried
 
  0.6191822
 
       NA
 
           NA
  
 
  
 2
 
 218621258
 
 218621580
 
  ENSEMBL   UCSC 
 
  323
 
  7
 
 25077
 
 island
 
 0.202846
 
 0.1133383
 
 Not queried
 
  0.6190850
 
       NA
 
           NA
  
 
  
 20
 
  33146263
 
  33146543
 
  ENSEMBL   UCSC 
 
  281
 
  6
 
 25400
 
 gene;island
 
 0.012746
 
 0.0064174
 
 Not queried
 
  0.6190355
 
       NA
 
           NA
  
 
  
 5
 
 156569687
 
 156569898
 
  ENSEMBL   UCSC 
 
  212
 
  5
 
 31873
 
 gene
 
 0.001563
 
 0.0046419
 
 Not queried
 
  0.6190040
 
       NA
 
           NA
  
 
  
 5
 
 156569790
 
 156570058
 
  ENSEMBL   UCSC 
 
  269
 
  7
 
 32454
 
 tss
 
 0.002938
 
 0.0046419
 
 Not queried
 
  0.6190040
 
       NA
 
           NA
  
 
  
 7
 
  76143178
 
  76143302
 
  ENSEMBL   UCSC 
 
  125
 
  4
 
 37058
 
 gene;island
 
 0.956467
 
 0.9797946
 
 Not queried
 
  0.6189082
 
       NA
 
           NA
  
 
  
 6
 
 167763758
 
 167764146
 
  ENSEMBL   UCSC 
 
  389
 
  4
 
 36643
 
 island
 
 0.156540
 
 0.2547253
 
 Not queried
 
  0.6188878
 
       NA
 
           NA
  
 
  
 1
 
 108743085
 
 108743728
 
  ENSEMBL   UCSC 
 
  644
 
  8
 
 1789
 
 tss;island
 
 0.009493
 
 0.0096761
 
 Not queried
 
  0.6187664
 
       NA
 
           NA
  
 
  
 17
 
  42289671
 
  42290028
 
  ENSEMBL   UCSC 
 
  358
 
  5
 
 16610
 
 gene;island
 
 0.922054
 
 0.9633852
 
 Not queried
 
  0.6187168
 
       NA
 
           NA
  
 
  
 4
 
   7666052
 
   7666259
 
  ENSEMBL   UCSC 
 
  208
 
  5
 
 29941
 
 gene;island
 
 0.640740
 
 0.7269566
 
 Not queried
 
  0.6186791
 
       NA
 
           NA
  
 
  
 10
 
 101088533
 
 101089149
 
  ENSEMBL   UCSC 
 
  617
 
  7
 
 6007
 
 island
 
 0.111063
 
 0.1726463
 
 Not queried
 
  0.6186560
 
       NA
 
           NA
  
 
  
 6
 
  41755394
 
  41755749
 
  ENSEMBL   UCSC 
 
  356
 
  6
 
 34334
 
 gene;island
 
 0.004058
 
 0.0053204
 
 Not queried
 
  0.6185702
 
       NA
 
           NA
  
 
  
 19
 
  48017995
 
  48018781
 
  ENSEMBL   UCSC 
 
  787
 
 14
 
 22197
 
 island
 
 0.005850
 
 0.0076892
 
 Not queried
 
  0.6185451
 
       NA
 
           NA
  
 
  
 6
 
  32051857
 
  32052736
 
  ENSEMBL   UCSC 
 
  880
 
 16
 
 34038
 
 gene;island
 
 0.473153
 
 0.5939891
 
 Not queried
 
  0.6184731
 
       NA
 
           NA
  
 
  
 3
 
  52008487
 
  52009002
 
  ENSEMBL   UCSC 
 
  516
 
 11
 
 28369
 
 tss;island
 
 0.034325
 
 0.0275653
 
 Not queried
 
  0.6183609
 
       NA
 
           NA
  
 
  
 1
 
 204183337
 
 204183655
 
  ENSEMBL   UCSC 
 
  319
 
  7
 
 2197
 
 tss
 
 0.239018
 
 0.1002625
 
 Not queried
 
  0.6183292
 
       NA
 
           NA
  
 
  
 2
 
 208890222
 
 208890496
 
  ENSEMBL   UCSC 
 
  275
 
  7
 
 25046
 
 island
 
 0.003215
 
 0.0193712
 
 Not queried
 
  0.6183134
 
       NA
 
           NA
  
 
  
 8
 
 145641991
 
 145642456
 
  ENSEMBL   UCSC 
 
  466
 
 10
 
 39982
 
 tss
 
 0.719384
 
 0.7709559
 
 Not queried
 
  0.6181976
 
       NA
 
           NA
  
 
  
 4
 
 128801645
 
 128802226
 
  ENSEMBL   UCSC 
 
  582
 
 10
 
 31299
 
 island
 
 0.009763
 
 0.0073417
 
 Not queried
 
  0.6180089
 
       NA
 
           NA
  
 
  
 6
 
  89827912
 
  89828172
 
  ENSEMBL   UCSC 
 
  261
 
  4
 
 35117
 
 tss;island
 
 0.235488
 
 0.4914664
 
 Not queried
 
  0.6179922
 
       NA
 
           NA
  
 
  
 10
 
  64028392
 
  64028750
 
  ENSEMBL   UCSC 
 
  359
 
  7
 
 5771
 
 island
 
 0.014729
 
 0.0095184
 
 Not queried
 
  0.6179746
 
       NA
 
           NA
  
 
  
 4
 
 107237473
 
 107238125
 
  ENSEMBL   UCSC 
 
  653
 
 11
 
 30081
 
 gene;tss
 
 0.002876
 
 0.0019963
 
 Not queried
 
  0.6178991
 
       NA
 
           NA
  
 
  
 1
 
 100731266
 
 100731633
 
  ENSEMBL   UCSC 
 
  368
 
  7
 
 1778
 
 tss
 
 0.008654
 
 0.0043973
 
 Not queried
 
  0.6177680
 
       NA
 
           NA
  
 
  
 19
 
  47292038
 
  47292253
 
  ENSEMBL   UCSC 
 
  216
 
  4
 
 20899
 
 tss;island
 
 0.060804
 
 0.0389547
 
 Not queried
 
  0.6176991
 
       NA
 
           NA
  
 
  
 17
 
   7608697
 
   7608859
 
  ENSEMBL   UCSC 
 
  163
 
  4
 
 16412
 
 gene;island
 
 0.018947
 
 0.0131127
 
 Not queried
 
  0.6176334
 
       NA
 
           NA
  
 
  
 10
 
  44101496
 
  44102227
 
  ENSEMBL   UCSC 
 
  732
 
 12
 
 5701
 
 island
 
 0.084927
 
 0.2603950
 
 Not queried
 
  0.6175092
 
       NA
 
           NA
  
 
  
 3
 
 195717344
 
 195717574
 
  ENSEMBL   UCSC 
 
  231
 
  4
 
 28808
 
 tss;island
 
 0.013957
 
 0.0138043
 
 Not queried
 
  0.6174743
 
       NA
 
           NA
  
 
  
 10
 
  23481385
 
  23481786
 
  ENSEMBL   UCSC 
 
  402
 
  6
 
 5610
 
 island
 
 0.030863
 
 0.0327216
 
 Not queried
 
  0.6174709
 
       NA
 
           NA
  
 
  
 21
 
  22369802
 
  22370444
 
  ENSEMBL   UCSC 
 
  643
 
  6
 
 26495
 
 tss;island
 
 0.081094
 
 0.1308999
 
 Not queried
 
  0.6174346
 
       NA
 
           NA
  
 
  
 16
 
  88563934
 
  88564366
 
  ENSEMBL   UCSC 
 
  433
 
  4
 
 14905
 
 gene;island
 
 0.954027
 
 0.8992144
 
 Not queried
 
  0.6174051
 
       NA
 
           NA
  
 
  
 5
 
 159435961
 
 159436125
 
  ENSEMBL   UCSC 
 
  165
 
  8
 
 32465
 
 tss
 
 0.005724
 
 0.0024235
 
 Not queried
 
  0.6174009
 
       NA
 
           NA
  
 
  
 11
 
 132864104
 
 132864357
 
  ENSEMBL   UCSC 
 
  254
 
  4
 
 6982
 
 gene
 
 0.097418
 
 0.2039623
 
 Not queried
 
  0.6173375
 
       NA
 
           NA
  
 
  
 7
 
  26904342
 
  26904713
 
  ENSEMBL   UCSC 
 
  372
 
  9
 
 37485
 
 tss
 
 0.002573
 
 0.0037736
 
 Not queried
 
  0.6170451
 
       NA
 
           NA
  
 
  
 3
 
 118864587
 
 118865050
 
  ENSEMBL   UCSC 
 
  464
 
 13
 
 28516
 
 tss
 
 0.846226
 
 0.9062800
 
 Not queried
 
  0.6170134
 
       NA
 
           NA
  
 
  
 9
 
 135905981
 
 135906520
 
  ENSEMBL   UCSC 
 
  540
 
  5
 
 40965
 
 island
 
 0.018148
 
 0.0111616
 
 Not queried
 
  0.6169185
 
       NA
 
           NA
  
 
  
 7
 
   5013451
 
   5013641
 
  ENSEMBL   UCSC 
 
  191
 
  8
 
 38209
 
 island
 
 0.021324
 
 0.0107754
 
 Not queried
 
  0.6168094
 
       NA
 
           NA
  
 
  
 10
 
 126106425
 
 126106614
 
  ENSEMBL   UCSC 
 
  190
 
  4
 
 4805
 
 gene;island
 
 0.777960
 
 0.5627027
 
 Not queried
 
  0.6167869
 
       NA
 
           NA
  
 
  
 18
 
  30050326
 
  30050398
 
  ENSEMBL   UCSC 
 
   73
 
  4
 
 19084
 
 gene;island
 
 0.037289
 
 0.0203240
 
 Not queried
 
  0.6167234
 
       NA
 
           NA
  
 
  
 9
 
  90113813
 
  90114156
 
  ENSEMBL   UCSC 
 
  344
 
  4
 
 40772
 
 gene;island
 
 0.007506
 
 0.0107126
 
 Not queried
 
  0.6165963
 
       NA
 
           NA
  
 
  
 14
 
  45356607
 
  45357076
 
  ENSEMBL   UCSC 
 
  470
 
  5
 
 12810
 
 island
 
 0.855498
 
 0.9117262
 
 Not queried
 
  0.6165820
 
       NA
 
           NA
  
 
  
 1
 
  85514134
 
  85514340
 
  ENSEMBL   UCSC 
 
  207
 
  4
 
 3419
 
 island
 
 0.003334
 
 0.0025479
 
 Not queried
 
  0.6165438
 
       NA
 
           NA
  
 
  
 5
 
 130970657
 
 130971264
 
  ENSEMBL   UCSC 
 
  608
 
 14
 
 33080
 
 island
 
 0.006597
 
 0.0047231
 
 Not queried
 
  0.6164830
 
       NA
 
           NA
  
 
  
 4
 
 140216770
 
 140217098
 
  ENSEMBL   UCSC 
 
  329
 
  7
 
 31314
 
 island
 
 0.010299
 
 0.0085370
 
 Not queried
 
  0.6164194
 
       NA
 
           NA
  
 
  
 5
 
   6755026
 
   6755439
 
  ENSEMBL   UCSC 
 
  414
 
  6
 
 31554
 
 gene;island
 
 0.974267
 
 0.9861040
 
 Not queried
 
  0.6163953
 
       NA
 
           NA
  
 
  
 13
 
  77461056
 
  77461426
 
  ENSEMBL   UCSC 
 
  371
 
  7
 
 11482
 
 tss;island
 
 0.104651
 
 0.0569952
 
 Not queried
 
  0.6163874
 
       NA
 
           NA
  
 
  
 17
 
   6659164
 
   6659562
 
  ENSEMBL   UCSC 
 
  399
 
  7
 
 16377
 
 gene
 
 0.007377
 
 0.0043346
 
 Not queried
 
  0.6162290
 
       NA
 
           NA
  
 
  
 2
 
  45169447
 
  45169681
 
  ENSEMBL   UCSC 
 
  235
 
  4
 
 22664
 
 gene;island
 
 0.011267
 
 0.0063281
 
 Not queried
 
  0.6161667
 
       NA
 
           NA
  
 
  
 3
 
 107646471
 
 107647202
 
  ENSEMBL   UCSC 
 
  732
 
  7
 
 27887
 
 gene;island
 
 0.012594
 
 0.0192393
 
 Not queried
 
  0.6161569
 
       NA
 
           NA
  
 
  
 3
 
   9404422
 
   9404684
 
  ENSEMBL   UCSC 
 
  263
 
  7
 
 28125
 
 tss
 
 0.010977
 
 0.0079942
 
 Not queried
 
  0.6160465
 
       NA
 
           NA
  
 
  
 5
 
  86563744
 
  86564119
 
  ENSEMBL   UCSC 
 
  376
 
  6
 
 32162
 
 tss;island
 
 0.010458
 
 0.0078208
 
 Not queried
 
  0.6159063
 
       NA
 
           NA
  
 
  
 5
 
 134241104
 
 134241313
 
  ENSEMBL   UCSC 
 
  210
 
  6
 
 31755
 
 gene;island
 
 0.004611
 
 0.0009404
 
 Not queried
 
  0.6158903
 
       NA
 
           NA
  
 
  
 10
 
  52834498
 
  52835078
 
  ENSEMBL   UCSC 
 
  581
 
  5
 
 4591
 
 gene;island
 
 0.012629
 
 0.0279777
 
 Not queried
 
  0.6158253
 
       NA
 
           NA
  
 
  
 3
 
  49894005
 
  49894441
 
  ENSEMBL   UCSC 
 
  437
 
  9
 
 28337
 
 tss
 
 0.033438
 
 0.0285185
 
 Not queried
 
  0.6158063
 
       NA
 
           NA
  
 
  
 19
 
  17378237
 
  17378296
 
  ENSEMBL   UCSC 
 
   60
 
  5
 
 19915
 
 gene
 
 0.009384
 
 0.0049339
 
 Not queried
 
  0.6155054
 
       NA
 
           NA
  
 
  
 7
 
 150065937
 
 150066350
 
  ENSEMBL   UCSC 
 
  414
 
  4
 
 37273
 
 gene;island
 
 0.014979
 
 0.0114335
 
 Not queried
 
  0.6154930
 
       NA
 
           NA
  
 
  
 1
 
   6684860
 
   6685162
 
  ENSEMBL   UCSC 
 
  303
 
  7
 
 1214
 
 tss;island
 
 0.006145
 
 0.0044913
 
 Not queried
 
  0.6154893
 
       NA
 
           NA
  
 
  
 19
 
  18451112
 
  18451650
 
  ENSEMBL   UCSC 
 
  539
 
 12
 
 21777
 
 island
 
 0.011639
 
 0.0248912
 
 Not queried
 
  0.6154774
 
       NA
 
           NA
  
 
  
 11
 
  77348600
 
  77348850
 
  ENSEMBL   UCSC 
 
  251
 
  4
 
 6841
 
 gene
 
 0.008059
 
 0.0052279
 
 Not queried
 
  0.6154539
 
       NA
 
           NA
  
 
  
 15
 
  93974164
 
  93974459
 
  ENSEMBL   UCSC 
 
  296
 
  5
 
 14483
 
 island
 
 0.566123
 
 0.7983094
 
 Not queried
 
  0.6153828
 
       NA
 
           NA
  
 
  
 19
 
  12780323
 
  12780627
 
  ENSEMBL   UCSC 
 
  305
 
  7
 
 19860
 
 gene;tss;island
 
 0.004065
 
 0.0013010
 
 Not queried
 
  0.6152906
 
       NA
 
           NA
  
 
  
 2
 
 240046227
 
 240046735
 
  ENSEMBL   UCSC 
 
  509
 
  5
 
 23106
 
 gene;island
 
 0.979285
 
 0.9721721
 
 Not queried
 
  0.6150696
 
       NA
 
           NA
  
 
  
 4
 
  81307586
 
  81307951
 
  ENSEMBL   UCSC 
 
  366
 
  4
 
 30037
 
 gene;island
 
 0.813239
 
 0.8821158
 
 Not queried
 
  0.6150433
 
       NA
 
           NA
  
 
  
 12
 
 120755296
 
 120755827
 
  ENSEMBL   UCSC 
 
  532
 
  6
 
 10887
 
 island
 
 0.002118
 
 0.0045990
 
 Not queried
 
  0.6149719
 
       NA
 
           NA
  
 
  
 5
 
  89770685
 
  89770788
 
  ENSEMBL   UCSC 
 
  104
 
  5
 
 31686
 
 gene
 
 0.008599
 
 0.0054185
 
 Not queried
 
  0.6149185
 
       NA
 
           NA
  
 
  
 17
 
  80375975
 
  80376196
 
  ENSEMBL   UCSC 
 
  222
 
  4
 
 16947
 
 gene;tss;island
 
 0.012755
 
 0.0245156
 
 Not queried
 
  0.6145424
 
       NA
 
           NA
  
 
  
 19
 
  45805662
 
  45805935
 
  ENSEMBL   UCSC 
 
  274
 
  4
 
 20091
 
 gene;island
 
 0.942842
 
 0.9694512
 
 Not queried
 
  0.6144388
 
       NA
 
           NA
  
 
  
 18
 
  71958904
 
  71959795
 
  ENSEMBL   UCSC 
 
  892
 
 12
 
 19569
 
 island
 
 0.008630
 
 0.0173712
 
 Not queried
 
  0.6143348
 
       NA
 
           NA
  
 
  
 11
 
  63606115
 
  63606397
 
  ENSEMBL   UCSC 
 
  283
 
  6
 
 7378
 
 tss
 
 0.004766
 
 0.0044402
 
 Not queried
 
  0.6141973
 
       NA
 
           NA
  
 
  
 1
 
 205180527
 
 205180802
 
  ENSEMBL   UCSC 
 
  276
 
  6
 
 4121
 
 island
 
 0.004334
 
 0.0077203
 
 Not queried
 
  0.6141661
 
       NA
 
           NA
  
 
  
 4
 
 184930219
 
 184930579
 
  ENSEMBL   UCSC 
 
  361
 
  6
 
 30198
 
 gene
 
 0.770988
 
 0.9506149
 
 Not queried
 
  0.6141357
 
       NA
 
           NA
  
 
  
 4
 
  52904379
 
  52905025
 
  ENSEMBL   UCSC 
 
  647
 
 10
 
 31039
 
 island
 
 0.030728
 
 0.0223368
 
 Not queried
 
  0.6141168
 
       NA
 
           NA
  
 
  
 19
 
  56347722
 
  56347927
 
  ENSEMBL   UCSC 
 
  206
 
  5
 
 20234
 
 gene;tss
 
 0.826866
 
 0.8842277
 
 Not queried
 
  0.6140426
 
       NA
 
           NA
  
 
  
 7
 
  41742630
 
  41743001
 
  ENSEMBL   UCSC 
 
  372
 
  4
 
 36975
 
 gene;island
 
 0.943495
 
 0.9614301
 
 Not queried
 
  0.6139367
 
       NA
 
           NA
  
 
  
 3
 
 147111120
 
 147111308
 
  ENSEMBL   UCSC 
 
  189
 
  4
 
 27987
 
 gene;tss;island
 
 0.219925
 
 0.1561786
 
 Not queried
 
  0.6139208
 
       NA
 
           NA
  
 
  
 1
 
  55464653
 
  55464927
 
  ENSEMBL   UCSC 
 
  275
 
  4
 
 551
 
 gene
 
 0.405685
 
 0.4985763
 
 Not queried
 
  0.6139098
 
       NA
 
           NA
  
 
  
 7
 
 105172664
 
 105173132
 
  ENSEMBL   UCSC 
 
  469
 
  5
 
 37176
 
 gene
 
 0.009760
 
 0.0111344
 
 Not queried
 
  0.6138106
 
       NA
 
           NA
  
 
  
 1
 
  62660188
 
  62660861
 
  ENSEMBL   UCSC 
 
  674
 
  8
 
 3335
 
 island
 
 0.605352
 
 0.4577939
 
 Not queried
 
  0.6137173
 
       NA
 
           NA
  
 
  
 5
 
 140855463
 
 140855562
 
  ENSEMBL   UCSC 
 
  100
 
  6
 
 32384
 
 tss
 
 0.012521
 
 0.0033428
 
 Not queried
 
  0.6135931
 
       NA
 
           NA
  
 
  
 2
 
 170683823
 
 170683981
 
  ENSEMBL   UCSC 
 
  159
 
  5
 
 23707
 
 tss
 
 0.015954
 
 0.0139781
 
 Not queried
 
  0.6135101
 
       NA
 
           NA
  
 
  
 2
 
 170683823
 
 170684197
 
  ENSEMBL   UCSC 
 
  375
 
  7
 
 24844
 
 island
 
 0.015954
 
 0.0139781
 
 Not queried
 
  0.6135101
 
       NA
 
           NA
  
 
  
 1
 
 224804053
 
 224804149
 
  ENSEMBL   UCSC 
 
   97
 
  4
 
 2278
 
 tss
 
 0.027362
 
 0.0423463
 
 Not queried
 
  0.6135035
 
       NA
 
           NA
  
 
  
 11
 
  17410182
 
  17410554
 
  ENSEMBL   UCSC 
 
  373
 
  4
 
 6523
 
 gene;island
 
 0.054212
 
 0.0581467
 
 Not queried
 
  0.6134774
 
       NA
 
           NA
  
 
  
 18
 
  12947517
 
  12947957
 
  ENSEMBL   UCSC 
 
  441
 
  8
 
 19191
 
 tss
 
 0.006765
 
 0.0069531
 
 Not queried
 
  0.6134295
 
       NA
 
           NA
  
 
  
 18
 
  12947517
 
  12948029
 
  ENSEMBL   UCSC 
 
  513
 
 10
 
 19408
 
 island
 
 0.006765
 
 0.0069531
 
 Not queried
 
  0.6134295
 
       NA
 
           NA
  
 
  
 16
 
  56659336
 
  56659724
 
  ENSEMBL   UCSC 
 
  389
 
  7
 
 15949
 
 island
 
 0.011127
 
 0.0256707
 
 Not queried
 
  0.6132261
 
       NA
 
           NA
  
 
  
 16
 
  86543519
 
  86543567
 
  ENSEMBL   UCSC 
 
   49
 
  5
 
 15401
 
 tss;island
 
 0.002115
 
 0.0043731
 
 Not queried
 
  0.6132123
 
       NA
 
           NA
  
 
  
 19
 
  58919807
 
  58920002
 
  ENSEMBL   UCSC 
 
  196
 
  7
 
 21142
 
 tss
 
 0.017624
 
 0.0102396
 
 Not queried
 
  0.6132103
 
       NA
 
           NA
  
 
  
 16
 
    475440
 
    475643
 
  ENSEMBL   UCSC 
 
  204
 
  4
 
 14968
 
 tss;island
 
 0.006555
 
 0.0135157
 
 Not queried
 
  0.6130840
 
       NA
 
           NA
  
 
  
 10
 
  13708059
 
  13708265
 
  ENSEMBL   UCSC 
 
  207
 
  9
 
 4503
 
 gene
 
 0.977264
 
 0.9858075
 
 Not queried
 
  0.6130613
 
       NA
 
           NA
  
 
  
 12
 
 133384565
 
 133384913
 
  ENSEMBL   UCSC 
 
  349
 
  4
 
 9463
 
 gene
 
 0.965855
 
 0.9503996
 
 Not queried
 
  0.6130288
 
       NA
 
           NA
  
 
  
 16
 
  85845626
 
  85845936
 
  ENSEMBL   UCSC 
 
  311
 
  4
 
 16165
 
 island
 
 0.359178
 
 0.4588298
 
 Not queried
 
  0.6129202
 
       NA
 
           NA
  
 
  
 17
 
  43339040
 
  43339831
 
  ENSEMBL   UCSC 
 
  792
 
 12
 
 16634
 
 gene;tss;island
 
 0.016565
 
 0.0172059
 
 Not queried
 
  0.6128999
 
       NA
 
           NA
  
 
  
 17
 
  80408768
 
  80408928
 
  ENSEMBL   UCSC 
 
  161
 
  7
 
 17775
 
 tss;island
 
 0.003836
 
 0.0011285
 
 Not queried
 
  0.6128875
 
       NA
 
           NA
  
 
  
 17
 
  66287369
 
  66287507
 
  ENSEMBL   UCSC 
 
  139
 
  6
 
 16741
 
 gene;island
 
 0.013081
 
 0.0194058
 
 Not queried
 
  0.6127465
 
       NA
 
           NA
  
 
  
 19
 
  55987809
 
  55988071
 
  ENSEMBL   UCSC 
 
  263
 
  4
 
 20231
 
 gene
 
 0.004722
 
 0.0074293
 
 Not queried
 
  0.6126805
 
       NA
 
           NA
  
 
  
 2
 
 201729279
 
 201729414
 
  ENSEMBL   UCSC 
 
  136
 
  7
 
 22980
 
 gene
 
 0.003130
 
 0.0055196
 
 Not queried
 
  0.6124389
 
       NA
 
           NA
  
 
  
 3
 
 138654924
 
 138655338
 
  ENSEMBL   UCSC 
 
  415
 
  4
 
 29527
 
 island
 
 0.041204
 
 0.0330016
 
 Not queried
 
  0.6123452
 
       NA
 
           NA
  
 
  
 15
 
  59665080
 
  59665308
 
  ENSEMBL   UCSC 
 
  229
 
  7
 
 13705
 
 tss
 
 0.004165
 
 0.0075668
 
 Not queried
 
  0.6122984
 
       NA
 
           NA
  
 
  
 5
 
 176326365
 
 176326619
 
  ENSEMBL   UCSC 
 
  255
 
  4
 
 32519
 
 tss
 
 0.293513
 
 0.5565334
 
 Not queried
 
  0.6122804
 
       NA
 
           NA
  
 
  
 6
 
  32182679
 
  32182745
 
  ENSEMBL   UCSC 
 
   67
 
  4
 
 34094
 
 gene
 
 0.935633
 
 0.9787314
 
 Not queried
 
  0.6122491
 
       NA
 
           NA
  
 
  
 8
 
 145729106
 
 145729373
 
  ENSEMBL   UCSC 
 
  268
 
  6
 
 39987
 
 tss
 
 0.552912
 
 0.7693718
 
 Not queried
 
  0.6122220
 
       NA
 
           NA
  
 
  
 6
 
 151646540
 
 151647133
 
  ENSEMBL   UCSC 
 
  594
 
  9
 
 34509
 
 gene;island
 
 0.040787
 
 0.0202081
 
 Not queried
 
  0.6122174
 
       NA
 
           NA
  
 
  
 6
 
 151646540
 
 151646651
 
  ENSEMBL   UCSC 
 
  112
 
  5
 
 35299
 
 tss
 
 0.067122
 
 0.0531478
 
 Not queried
 
  0.6122174
 
       NA
 
           NA
  
 
  
 3
 
 140660335
 
 140660748
 
  ENSEMBL   UCSC 
 
  414
 
  9
 
 29541
 
 island
 
 0.003597
 
 0.0054156
 
 Not queried
 
  0.6121260
 
       NA
 
           NA
  
 
  
 5
 
  56468858
 
  56469322
 
  ENSEMBL   UCSC 
 
  465
 
  4
 
 32074
 
 tss;island
 
 0.009031
 
 0.0102331
 
 Not queried
 
  0.6121203
 
       NA
 
           NA
  
 
  
 2
 
  86564885
 
  86565390
 
  ENSEMBL   UCSC 
 
  506
 
  9
 
 23493
 
 tss;island
 
 0.031112
 
 0.0568296
 
 Not queried
 
  0.6118863
 
       NA
 
           NA
  
 
  
 19
 
  44079778
 
  44079985
 
  ENSEMBL   UCSC 
 
  208
 
  7
 
 20831
 
 tss
 
 0.003540
 
 0.0037038
 
 Not queried
 
  0.6117922
 
       NA
 
           NA
  
 
  
 19
 
  44079527
 
  44079985
 
  ENSEMBL   UCSC 
 
  459
 
  9
 
 22090
 
 island
 
 0.003540
 
 0.0037038
 
 Not queried
 
  0.6117922
 
       NA
 
           NA
  
 
  
 7
 
  72741781
 
  72742188
 
  ENSEMBL   UCSC 
 
  408
 
 11
 
 37632
 
 tss;island
 
 0.969702
 
 0.9840028
 
 Not queried
 
  0.6117483
 
       NA
 
           NA
  
 
  
 3
 
  52516316
 
  52516676
 
  ENSEMBL   UCSC 
 
  361
 
  6
 
 27820
 
 gene
 
 0.975454
 
 0.9737214
 
 Not queried
 
  0.6115679
 
       NA
 
           NA
  
 
  
 1
 
  29508578
 
  29508888
 
  ENSEMBL   UCSC 
 
  311
 
  8
 
 2960
 
 island
 
 0.017945
 
 0.0121858
 
 Not queried
 
  0.6114603
 
       NA
 
           NA
  
 
  
 19
 
  12075601
 
  12076348
 
  ENSEMBL   UCSC 
 
  748
 
 11
 
 21588
 
 island
 
 0.016748
 
 0.0098687
 
 Not queried
 
  0.6114313
 
       NA
 
           NA
  
 
  
 11
 
  62623202
 
  62623324
 
  ENSEMBL   UCSC 
 
  123
 
  4
 
 6651
 
 gene
 
 0.005704
 
 0.0067141
 
 Not queried
 
  0.6114236
 
       NA
 
           NA
  
 
  
 11
 
  15959856
 
  15960076
 
  ENSEMBL   UCSC 
 
  221
 
  4
 
 8085
 
 island
 
 0.759177
 
 0.8550281
 
 Not queried
 
  0.6113589
 
       NA
 
           NA
  
 
  
 21
 
  42688675
 
  42689051
 
  ENSEMBL   UCSC 
 
  377
 
  4
 
 26429
 
 gene
 
 0.056772
 
 0.0321278
 
 Not queried
 
  0.6112022
 
       NA
 
           NA
  
 
  
 14
 
  21979401
 
  21979675
 
  ENSEMBL   UCSC 
 
  275
 
  9
 
 12697
 
 island
 
 0.004238
 
 0.0027497
 
 Not queried
 
  0.6111970
 
       NA
 
           NA
  
 
  
 6
 
  32712930
 
  32713188
 
  ENSEMBL   UCSC 
 
  259
 
  5
 
 34130
 
 gene
 
 0.312372
 
 0.4095551
 
 Not queried
 
  0.6111681
 
       NA
 
           NA
  
 
  
 17
 
   5974448
 
   5974609
 
  ENSEMBL   UCSC 
 
  162
 
  4
 
 16370
 
 gene;island
 
 0.016978
 
 0.0430591
 
 Not queried
 
  0.6110116
 
       NA
 
           NA
  
 
  
 2
 
 118771338
 
 118771984
 
  ENSEMBL   UCSC 
 
  647
 
  9
 
 24639
 
 island
 
 0.002434
 
 0.0052629
 
 Not queried
 
  0.6109494
 
       NA
 
           NA
  
 
  
 7
 
   2801732
 
   2802073
 
  ENSEMBL   UCSC 
 
  342
 
  4
 
 36817
 
 gene;island
 
 0.967553
 
 0.9390299
 
 Not queried
 
  0.6109021
 
       NA
 
           NA
  
 
  
 2
 
  47168905
 
  47169299
 
  ENSEMBL   UCSC 
 
  395
 
  8
 
 22671
 
 gene;island
 
 0.005620
 
 0.0104744
 
 Not queried
 
  0.6108589
 
       NA
 
           NA
  
 
  
 3
 
  67705044
 
  67705285
 
  ENSEMBL   UCSC 
 
  242
 
  5
 
 28436
 
 tss
 
 0.017913
 
 0.0139355
 
 Not queried
 
  0.6108292
 
       NA
 
           NA
  
 
  
 12
 
 133562589
 
 133563027
 
  ENSEMBL   UCSC 
 
  439
 
 10
 
 10136
 
 tss
 
 0.006434
 
 0.0065054
 
 Not queried
 
  0.6106730
 
       NA
 
           NA
  
 
  
 19
 
  55770037
 
  55770147
 
  ENSEMBL   UCSC 
 
  111
 
  4
 
 22416
 
 island
 
 0.015313
 
 0.0160136
 
 Not queried
 
  0.6106463
 
       NA
 
           NA
  
 
  
 11
 
   2481206
 
   2481641
 
  ENSEMBL   UCSC 
 
  436
 
  7
 
 6459
 
 gene;tss
 
 0.361834
 
 0.4484017
 
 Not queried
 
  0.6106277
 
       NA
 
           NA
  
 
  
 13
 
  50702675
 
  50703026
 
  ENSEMBL   UCSC 
 
  352
 
  6
 
 11742
 
 island
 
 0.468478
 
 0.7483059
 
 Not queried
 
  0.6106243
 
       NA
 
           NA
  
 
  
 5
 
 101834365
 
 101834780
 
  ENSEMBL   UCSC 
 
  416
 
  5
 
 32987
 
 island
 
 0.807428
 
 0.8744461
 
 Not queried
 
  0.6106151
 
       NA
 
           NA
  
 
  
 3
 
  49840361
 
  49840712
 
  ENSEMBL   UCSC 
 
  352
 
  4
 
 29130
 
 island
 
 0.015155
 
 0.0101255
 
 Not queried
 
  0.6105350
 
       NA
 
           NA
  
 
  
 2
 
  75426249
 
  75426512
 
  ENSEMBL   UCSC 
 
  264
 
  5
 
 22751
 
 gene;island
 
 0.029973
 
 0.0176039
 
 Not queried
 
  0.6104830
 
       NA
 
           NA
  
 
  
 1
 
 243053673
 
 243054071
 
  ENSEMBL   UCSC 
 
  399
 
  4
 
 4383
 
 island
 
 0.196101
 
 0.0389927
 
 Not queried
 
  0.6103602
 
       NA
 
           NA
  
 
  
 19
 
   9649310
 
   9649542
 
  ENSEMBL   UCSC 
 
  233
 
  7
 
 20426
 
 tss
 
 0.029296
 
 0.0131178
 
 Not queried
 
  0.6103526
 
       NA
 
           NA
  
 
  
 19
 
   9649241
 
   9649542
 
  ENSEMBL   UCSC 
 
  302
 
  9
 
 21500
 
 island
 
 0.023336
 
 0.0114339
 
 Not queried
 
  0.6103526
 
       NA
 
           NA
  
 
  
 14
 
  32030176
 
  32030584
 
  ENSEMBL   UCSC 
 
  409
 
  8
 
 12343
 
 tss
 
 0.009019
 
 0.0103791
 
 Not queried
 
  0.6103407
 
       NA
 
           NA
  
 
  
 18
 
  72999596
 
  73000004
 
  ENSEMBL   UCSC 
 
  409
 
  5
 
 19129
 
 gene;island
 
 0.967516
 
 0.9509324
 
 Not queried
 
  0.6101052
 
       NA
 
           NA
  
 
  
 6
 
  26158157
 
  26158342
 
  ENSEMBL   UCSC 
 
  186
 
  4
 
 34690
 
 tss
 
 0.002711
 
 0.0011712
 
 Not queried
 
  0.6101027
 
       NA
 
           NA
  
 
  
 1
 
 228604037
 
 228604595
 
  ENSEMBL   UCSC 
 
  559
 
  6
 
 4296
 
 island
 
 0.043758
 
 0.1211692
 
 Not queried
 
  0.6101010
 
       NA
 
           NA
  
 
  
 6
 
   3227556
 
   3227949
 
  ENSEMBL   UCSC 
 
  394
 
  4
 
 33536
 
 gene
 
 0.009479
 
 0.0059972
 
 Not queried
 
  0.6100594
 
       NA
 
           NA
  
 
  
 20
 
  39766189
 
  39766506
 
  ENSEMBL   UCSC 
 
  318
 
  5
 
 25420
 
 gene;island
 
 0.012955
 
 0.0138188
 
 Not queried
 
  0.6100291
 
       NA
 
           NA
  
 
  
 10
 
 118380175
 
 118380396
 
  ENSEMBL   UCSC 
 
  222
 
  4
 
 5385
 
 tss
 
 0.755525
 
 0.9068049
 
 Not queried
 
  0.6100138
 
       NA
 
           NA
  
 
  
 2
 
  66810341
 
  66810606
 
  ENSEMBL   UCSC 
 
  266
 
  4
 
 24359
 
 island
 
 0.008766
 
 0.0262471
 
 Not queried
 
  0.6099220
 
       NA
 
           NA
  
 
  
 5
 
 136834155
 
 136834492
 
  ENSEMBL   UCSC 
 
  338
 
  6
 
 31762
 
 gene;island
 
 0.079409
 
 0.0462150
 
 Not queried
 
  0.6099092
 
       NA
 
           NA
  
 
  
 19
 
   3700613
 
   3700773
 
  ENSEMBL   UCSC 
 
  161
 
  4
 
 20350
 
 tss;island
 
 0.003717
 
 0.0069296
 
 Not queried
 
  0.6098550
 
       NA
 
           NA
  
 
  
 11
 
  68518800
 
  68518961
 
  ENSEMBL   UCSC 
 
  162
 
  4
 
 6777
 
 gene
 
 0.008552
 
 0.0065842
 
 Not queried
 
  0.6096444
 
       NA
 
           NA
  
 
  
 10
 
  46090406
 
  46090807
 
  ENSEMBL   UCSC 
 
  402
 
  7
 
 5060
 
 tss
 
 0.015126
 
 0.0044876
 
 Not queried
 
  0.6095612
 
       NA
 
           NA
  
 
  
 6
 
  30956214
 
  30956796
 
  ENSEMBL   UCSC 
 
  583
 
  8
 
 33847
 
 gene
 
 0.545782
 
 0.6616506
 
 Not queried
 
  0.6095414
 
       NA
 
           NA
  
 
  
 12
 
    534563
 
    534917
 
  ENSEMBL   UCSC 
 
  355
 
  4
 
 8994
 
 gene
 
 0.981841
 
 0.9865225
 
 Not queried
 
  0.6095258
 
       NA
 
           NA
  
 
  
 12
 
 133345756
 
 133346306
 
  ENSEMBL   UCSC 
 
  551
 
  7
 
 9459
 
 gene;island
 
 0.910818
 
 0.9543515
 
 Not queried
 
  0.6094916
 
       NA
 
           NA
  
 
  
 1
 
 228651708
 
 228652067
 
  ENSEMBL   UCSC 
 
  360
 
  4
 
 4301
 
 island
 
 0.008504
 
 0.0592162
 
 Not queried
 
  0.6094503
 
       NA
 
           NA
  
 
  
 1
 
  91191583
 
  91192162
 
  ENSEMBL   UCSC 
 
  580
 
  6
 
 3463
 
 island
 
 0.044750
 
 0.0313129
 
 Not queried
 
  0.6094352
 
       NA
 
           NA
  
 
  
 2
 
 162273011
 
 162273648
 
  ENSEMBL   UCSC 
 
  638
 
  7
 
 22909
 
 gene;island
 
 0.381570
 
 0.0264556
 
 Not queried
 
  0.6094265
 
       NA
 
           NA
  
 
  
 7
 
   4814952
 
   4815219
 
  ENSEMBL   UCSC 
 
  268
 
  6
 
 37416
 
 tss
 
 0.002469
 
 0.0028306
 
 Not queried
 
  0.6093328
 
       NA
 
           NA
  
 
  
 3
 
  50604613
 
  50605156
 
  ENSEMBL   UCSC 
 
  544
 
  6
 
 27812
 
 gene
 
 0.011045
 
 0.0107466
 
 Not queried
 
  0.6092702
 
       NA
 
           NA
  
 
  
 6
 
 125284203
 
 125284359
 
  ENSEMBL   UCSC 
 
  157
 
  6
 
 35208
 
 tss;island
 
 0.022970
 
 0.0198721
 
 Not queried
 
  0.6092686
 
       NA
 
           NA
  
 
  
 2
 
 219824293
 
 219824401
 
  ENSEMBL   UCSC 
 
  109
 
  6
 
 25096
 
 island
 
 0.003677
 
 0.0032210
 
 Not queried
 
  0.6092102
 
       NA
 
           NA
  
 
  
 22
 
  19554053
 
  19554706
 
  ENSEMBL   UCSC 
 
  654
 
  7
 
 27299
 
 island
 
 0.832118
 
 0.8571737
 
 Not queried
 
  0.6091153
 
       NA
 
           NA
  
 
  
 10
 
 135020656
 
 135021100
 
  ENSEMBL   UCSC 
 
  445
 
  5
 
 4891
 
 gene;island
 
 0.542153
 
 0.6435736
 
 Not queried
 
  0.6090240
 
       NA
 
           NA
  
 
  
 2
 
 121199631
 
 121199920
 
  ENSEMBL   UCSC 
 
  290
 
  4
 
 24666
 
 island
 
 0.036744
 
 0.0465821
 
 Not queried
 
  0.6090093
 
       NA
 
           NA
  
 
  
 12
 
 107714673
 
 107715146
 
  ENSEMBL   UCSC 
 
  474
 
  7
 
 9283
 
 gene;island
 
 0.003379
 
 0.0102706
 
 Not queried
 
  0.6090009
 
       NA
 
           NA
  
 
  
 1
 
   1142132
 
   1142405
 
  ENSEMBL   UCSC 
 
  274
 
  4
 
 1144
 
 tss
 
 0.015281
 
 0.0371162
 
 Not queried
 
  0.6089109
 
       NA
 
           NA
  
 
  
 6
 
  32160455
 
  32161004
 
  ENSEMBL   UCSC 
 
  550
 
  7
 
 34082
 
 gene;island
 
 0.031732
 
 0.1749972
 
 Not queried
 
  0.6088541
 
       NA
 
           NA
  
 
  
 15
 
  42782927
 
  42783487
 
  ENSEMBL   UCSC 
 
  561
 
  7
 
 14075
 
 island
 
 0.006192
 
 0.0073596
 
 Not queried
 
  0.6088532
 
       NA
 
           NA
  
 
  
 21
 
  30671675
 
  30671710
 
  ENSEMBL   UCSC 
 
   36
 
  5
 
 26504
 
 tss
 
 0.005845
 
 0.0069469
 
 Not queried
 
  0.6086134
 
       NA
 
           NA
  
 
  
 12
 
 127212034
 
 127212528
 
  ENSEMBL   UCSC 
 
  495
 
  4
 
 10985
 
 island
 
 0.067750
 
 0.2207196
 
 Not queried
 
  0.6085128
 
       NA
 
           NA
  
 
  
 5
 
 154026930
 
 154027256
 
  ENSEMBL   UCSC 
 
  327
 
  4
 
 33334
 
 island
 
 0.023997
 
 0.0204680
 
 Not queried
 
  0.6084848
 
       NA
 
           NA
  
 
  
 1
 
 211433972
 
 211434314
 
  ENSEMBL   UCSC 
 
  343
 
  4
 
 994
 
 gene;island
 
 0.005746
 
 0.0041288
 
 Not queried
 
  0.6084398
 
       NA
 
           NA
  
 
  
 17
 
  61613061
 
  61613356
 
  ENSEMBL   UCSC 
 
  296
 
  5
 
 16727
 
 gene;island
 
 0.891868
 
 0.9291303
 
 Not queried
 
  0.6084296
 
       NA
 
           NA
  
 
  
 4
 
   1595294
 
   1595726
 
  ENSEMBL   UCSC 
 
  433
 
  5
 
 30794
 
 island
 
 0.910782
 
 0.8925274
 
 Not queried
 
  0.6083417
 
       NA
 
           NA
  
 
  
 20
 
  33999839
 
  33999889
 
  ENSEMBL   UCSC 
 
   51
 
  6
 
 25705
 
 tss
 
 0.016862
 
 0.0110016
 
 Not queried
 
  0.6081799
 
       NA
 
           NA
  
 
  
 15
 
  44116616
 
  44116879
 
  ENSEMBL   UCSC 
 
  264
 
  4
 
 13321
 
 gene
 
 0.012440
 
 0.0136372
 
 Not queried
 
  0.6080859
 
       NA
 
           NA
  
 
  
 20
 
  32031702
 
  32031951
 
  ENSEMBL   UCSC 
 
  250
 
  7
 
 25683
 
 tss;island
 
 0.011167
 
 0.0143372
 
 Not queried
 
  0.6079273
 
       NA
 
           NA
  
 
  
 8
 
  21999541
 
  21999703
 
  ENSEMBL   UCSC 
 
  163
 
  6
 
 39581
 
 tss
 
 0.028059
 
 0.0199392
 
 Not queried
 
  0.6079234
 
       NA
 
           NA
  
 
  
 5
 
 150028986
 
 150029342
 
  ENSEMBL   UCSC 
 
  357
 
  4
 
 31862
 
 gene;island
 
 0.953480
 
 0.9299012
 
 Not queried
 
  0.6078198
 
       NA
 
           NA
  
 
  
 20
 
  33759605
 
  33760017
 
  ENSEMBL   UCSC 
 
  413
 
  7
 
 26120
 
 island
 
 0.023396
 
 0.0603575
 
 Not queried
 
  0.6078170
 
       NA
 
           NA
  
 
  
 19
 
  15490553
 
  15490918
 
  ENSEMBL   UCSC 
 
  366
 
 11
 
 21703
 
 island
 
 0.011274
 
 0.0068792
 
 Not queried
 
  0.6077504
 
       NA
 
           NA
  
 
  
 6
 
  32016070
 
  32016803
 
  ENSEMBL   UCSC 
 
  734
 
 23
 
 34015
 
 gene
 
 0.041810
 
 0.0829052
 
 Not queried
 
  0.6076889
 
       NA
 
           NA
  
 
  
 17
 
  48278778
 
  48278952
 
  ENSEMBL   UCSC 
 
  175
 
  4
 
 16683
 
 gene
 
 0.033157
 
 0.0564655
 
 Not queried
 
  0.6076823
 
       NA
 
           NA
  
 
  
 7
 
  25018440
 
  25018595
 
  ENSEMBL   UCSC 
 
  156
 
  4
 
 36923
 
 gene;island
 
 0.912968
 
 0.9763874
 
 Not queried
 
  0.6076751
 
       NA
 
           NA
  
 
  
 7
 
 155623690
 
 155624002
 
  ENSEMBL   UCSC 
 
  313
 
  4
 
 39078
 
 island
 
 0.279353
 
 0.4299347
 
 Not queried
 
  0.6075496
 
       NA
 
           NA
  
 
  
 11
 
  73881872
 
  73882018
 
  ENSEMBL   UCSC 
 
  147
 
  4
 
 6825
 
 gene
 
 0.014061
 
 0.0187499
 
 Not queried
 
  0.6075441
 
       NA
 
           NA
  
 
  
 6
 
 137243219
 
 137243386
 
  ENSEMBL   UCSC 
 
  168
 
  5
 
 35246
 
 tss
 
 0.011355
 
 0.0045680
 
 Not queried
 
  0.6074680
 
       NA
 
           NA
  
 
  
 2
 
  74730507
 
  74730948
 
  ENSEMBL   UCSC 
 
  442
 
  9
 
 23461
 
 tss
 
 0.017377
 
 0.0495592
 
 Not queried
 
  0.6074101
 
       NA
 
           NA
  
 
  
 11
 
  66104018
 
  66104174
 
  ENSEMBL   UCSC 
 
  157
 
  4
 
 7465
 
 tss
 
 0.040436
 
 0.0603309
 
 Not queried
 
  0.6073460
 
       NA
 
           NA
  
 
  
 20
 
  31446183
 
  31446682
 
  ENSEMBL   UCSC 
 
  500
 
  5
 
 25678
 
 tss
 
 0.969987
 
 0.9235383
 
 Not queried
 
  0.6073235
 
       NA
 
           NA
  
 
  
 3
 
  53164576
 
  53164697
 
  ENSEMBL   UCSC 
 
  122
 
  6
 
 28396
 
 tss
 
 0.004066
 
 0.0033091
 
 Not queried
 
  0.6071614
 
       NA
 
           NA
  
 
  
 3
 
 108836693
 
 108836912
 
  ENSEMBL   UCSC 
 
  220
 
  4
 
 27889
 
 gene
 
 0.715835
 
 0.8130120
 
 Not queried
 
  0.6071388
 
       NA
 
           NA
  
 
  
 7
 
  26903972
 
  26904713
 
  ENSEMBL   UCSC 
 
  742
 
 13
 
 38333
 
 island
 
 0.005969
 
 0.0046919
 
 Not queried
 
  0.6071118
 
       NA
 
           NA
  
 
  
 2
 
 119532041
 
 119532574
 
  ENSEMBL   UCSC 
 
  534
 
  7
 
 24643
 
 island
 
 0.153144
 
 0.0115901
 
 Not queried
 
  0.6070532
 
       NA
 
           NA
  
 
  
 1
 
  21877524
 
  21877781
 
  ENSEMBL   UCSC 
 
  258
 
  5
 
 298
 
 gene;tss
 
 0.861774
 
 0.9590146
 
 Not queried
 
  0.6070109
 
       NA
 
           NA
  
 
  
 11
 
    719612
 
    719828
 
  ENSEMBL   UCSC 
 
  217
 
  4
 
 6389
 
 gene;island
 
 0.763206
 
 0.8797863
 
 Not queried
 
  0.6067504
 
       NA
 
           NA
  
 
  
 17
 
  72856049
 
  72856181
 
  ENSEMBL   UCSC 
 
  133
 
  4
 
 17632
 
 tss
 
 0.017967
 
 0.0113259
 
 Not queried
 
  0.6065628
 
       NA
 
           NA
  
 
  
 19
 
   3029503
 
   3029785
 
  ENSEMBL   UCSC 
 
  283
 
  4
 
 19702
 
 gene;tss;island
 
 0.101703
 
 0.1103444
 
 Not queried
 
  0.6064889
 
       NA
 
           NA
  
 
  
 1
 
  33502309
 
  33502810
 
  ENSEMBL   UCSC 
 
  502
 
 11
 
 3006
 
 island
 
 0.004526
 
 0.0054144
 
 Not queried
 
  0.6061530
 
       NA
 
           NA
  
 
  
 11
 
  67383377
 
  67384040
 
  ENSEMBL   UCSC 
 
  664
 
  8
 
 8559
 
 island
 
 0.440620
 
 0.4496699
 
 Not queried
 
  0.6060681
 
       NA
 
           NA
  
 
  
 19
 
  10811855
 
  10812419
 
  ENSEMBL   UCSC 
 
  565
 
  8
 
 21544
 
 island
 
 0.007302
 
 0.0123396
 
 Not queried
 
  0.6060345
 
       NA
 
           NA
  
 
  
 5
 
 130971021
 
 130971264
 
  ENSEMBL   UCSC 
 
  244
 
  9
 
 32264
 
 tss
 
 0.008520
 
 0.0048296
 
 Not queried
 
  0.6059509
 
       NA
 
           NA
  
 
  
 10
 
 122216135
 
 122216378
 
  ENSEMBL   UCSC 
 
  244
 
  7
 
 5403
 
 tss;island
 
 0.042207
 
 0.0197696
 
 Not queried
 
  0.6059209
 
       NA
 
           NA
  
 
  
 1
 
 153600762
 
 153600972
 
  ENSEMBL   UCSC 
 
  211
 
  4
 
 763
 
 gene;tss
 
 0.691164
 
 0.8283789
 
 Not queried
 
  0.6057517
 
       NA
 
           NA
  
 
  
 3
 
  53925231
 
  53925379
 
  ENSEMBL   UCSC 
 
  149
 
  4
 
 27834
 
 gene;island
 
 0.012897
 
 0.0060217
 
 Not queried
 
  0.6057007
 
       NA
 
           NA
  
 
  
 6
 
  26216541
 
  26217212
 
  ENSEMBL   UCSC 
 
  672
 
  8
 
 34696
 
 tss;island
 
 0.004540
 
 0.0011004
 
 Not queried
 
  0.6055725
 
       NA
 
           NA
  
 
  
 7
 
  23509546
 
  23509770
 
  ENSEMBL   UCSC 
 
  225
 
  4
 
 36915
 
 gene;island
 
 0.072019
 
 0.0746267
 
 Not queried
 
  0.6055611
 
       NA
 
           NA
  
 
  
 7
 
   4783964
 
   4784485
 
  ENSEMBL   UCSC 
 
  522
 
  6
 
 36845
 
 gene;island
 
 0.118974
 
 0.0580759
 
 Not queried
 
  0.6055415
 
       NA
 
           NA
  
 
  
 16
 
  75285489
 
  75285726
 
  ENSEMBL   UCSC 
 
  238
 
  6
 
 14843
 
 gene;island
 
 0.011113
 
 0.0090745
 
 Not queried
 
  0.6055360
 
       NA
 
           NA
  
 
  
 17
 
  60885856
 
  60886079
 
  ENSEMBL   UCSC 
 
  224
 
  4
 
 17562
 
 tss
 
 0.825684
 
 0.5710724
 
 Not queried
 
  0.6054644
 
       NA
 
           NA
  
 
  
 4
 
  81105167
 
  81105375
 
  ENSEMBL   UCSC 
 
  209
 
  4
 
 30458
 
 tss;island
 
 0.031781
 
 0.0216836
 
 Not queried
 
  0.6054296
 
       NA
 
           NA
  
 
  
 8
 
 125985352
 
 125985512
 
  ENSEMBL   UCSC 
 
  161
 
  4
 
 39908
 
 tss
 
 0.014440
 
 0.0201945
 
 Not queried
 
  0.6053541
 
       NA
 
           NA
  
 
  
 2
 
 187454259
 
 187454747
 
  ENSEMBL   UCSC 
 
  489
 
  7
 
 23773
 
 tss
 
 0.015625
 
 0.0133220
 
 Not queried
 
  0.6053449
 
       NA
 
           NA
  
 
  
 6
 
 137114064
 
 137114243
 
  ENSEMBL   UCSC 
 
  180
 
  4
 
 35243
 
 tss;island
 
 0.004598
 
 0.0041933
 
 Not queried
 
  0.6052090
 
       NA
 
           NA
  
 
  
 16
 
  81812565
 
  81812738
 
  ENSEMBL   UCSC 
 
  174
 
  4
 
 15382
 
 tss;island
 
 0.008465
 
 0.0196322
 
 Not queried
 
  0.6051945
 
       NA
 
           NA
  
 
  
 11
 
  67276127
 
  67276453
 
  ENSEMBL   UCSC 
 
  327
 
  9
 
 7496
 
 tss
 
 0.006281
 
 0.0053643
 
 Not queried
 
  0.6051325
 
       NA
 
           NA
  
 
  
 20
 
  61201022
 
  61201271
 
  ENSEMBL   UCSC 
 
  250
 
  4
 
 26328
 
 island
 
 0.033935
 
 0.0221737
 
 Not queried
 
  0.6051158
 
       NA
 
           NA
  
 
  
 1
 
 228633275
 
 228633664
 
  ENSEMBL   UCSC 
 
  390
 
  4
 
 4298
 
 island
 
 0.043711
 
 0.0906645
 
 Not queried
 
  0.6050805
 
       NA
 
           NA
  
 
  
 8
 
  52812092
 
  52812311
 
  ENSEMBL   UCSC 
 
  220
 
  4
 
 39696
 
 tss;island
 
 0.002674
 
 0.0025464
 
 Not queried
 
  0.6050015
 
       NA
 
           NA
  
 
  
 15
 
  56535484
 
  56536071
 
  ENSEMBL   UCSC 
 
  588
 
  5
 
 13687
 
 tss
 
 0.012223
 
 0.0078794
 
 Not queried
 
  0.6048491
 
       NA
 
           NA
  
 
  
 4
 
 109541393
 
 109542144
 
  ENSEMBL   UCSC 
 
  752
 
 17
 
 30540
 
 tss
 
 0.003523
 
 0.0028369
 
 Not queried
 
  0.6048292
 
       NA
 
           NA
  
 
  
 22
 
  30821505
 
  30821706
 
  ENSEMBL   UCSC 
 
  202
 
  7
 
 27062
 
 tss
 
 0.004914
 
 0.0050278
 
 Not queried
 
  0.6047280
 
       NA
 
           NA
  
 
  
 13
 
  45694523
 
  45695040
 
  ENSEMBL   UCSC 
 
  518
 
 11
 
 11708
 
 island
 
 0.008429
 
 0.0060985
 
 Not queried
 
  0.6046646
 
       NA
 
           NA
  
 
  
 4
 
  40058917
 
  40059040
 
  ENSEMBL   UCSC 
 
  124
 
  7
 
 29986
 
 gene;tss;island
 
 0.013692
 
 0.0058762
 
 Not queried
 
  0.6044286
 
       NA
 
           NA
  
 
  
 12
 
 111843215
 
 111843585
 
  ENSEMBL   UCSC 
 
  371
 
  6
 
 10015
 
 tss;island
 
 0.005849
 
 0.0035329
 
 Not queried
 
  0.6044238
 
       NA
 
           NA
  
 
  
 11
 
  14521079
 
  14521425
 
  ENSEMBL   UCSC 
 
  347
 
  5
 
 6514
 
 gene
 
 0.010926
 
 0.0064723
 
 Not queried
 
  0.6042631
 
       NA
 
           NA
  
 
  
 7
 
 117067616
 
 117067978
 
  ENSEMBL   UCSC 
 
  363
 
  8
 
 37855
 
 tss;island
 
 0.967823
 
 0.9236098
 
 Not queried
 
  0.6042037
 
       NA
 
           NA
  
 
  
 2
 
  24583331
 
  24583671
 
  ENSEMBL   UCSC 
 
  341
 
  7
 
 24133
 
 island
 
 0.007980
 
 0.0062656
 
 Not queried
 
  0.6039913
 
       NA
 
           NA
  
 
  
 15
 
  91498139
 
  91498497
 
  ENSEMBL   UCSC 
 
  359
 
  4
 
 13481
 
 gene
 
 0.013650
 
 0.0082018
 
 Not queried
 
  0.6038501
 
       NA
 
           NA
  
 
  
 7
 
  63774078
 
  63774504
 
  ENSEMBL   UCSC 
 
  427
 
  5
 
 37023
 
 gene
 
 0.005604
 
 0.0120687
 
 Not queried
 
  0.6038235
 
       NA
 
           NA
  
 
  
 11
 
   2170831
 
   2171184
 
  ENSEMBL   UCSC 
 
  354
 
  6
 
 6449
 
 gene
 
 0.005962
 
 0.1176480
 
 Not queried
 
  0.6036865
 
       NA
 
           NA
  
 
  
 16
 
  84401756
 
  84402160
 
  ENSEMBL   UCSC 
 
  405
 
  6
 
 16142
 
 island
 
 0.037165
 
 0.0280479
 
 Not queried
 
  0.6036817
 
       NA
 
           NA
  
 
  
 21
 
  47404109
 
  47404268
 
  ENSEMBL   UCSC 
 
  160
 
  4
 
 26472
 
 gene;island
 
 0.852630
 
 0.8790958
 
 Not queried
 
  0.6036636
 
       NA
 
           NA
  
 
  
 7
 
 128696069
 
 128696229
 
  ENSEMBL   UCSC 
 
  161
 
  4
 
 37220
 
 gene;tss;island
 
 0.938516
 
 0.8804881
 
 Not queried
 
  0.6036346
 
       NA
 
           NA
  
 
  
 19
 
  43918868
 
  43919039
 
  ENSEMBL   UCSC 
 
  172
 
  7
 
 20073
 
 gene;island
 
 0.537812
 
 0.6006453
 
 Not queried
 
  0.6035331
 
       NA
 
           NA
  
 
  
 16
 
  14397576
 
  14397766
 
  ENSEMBL   UCSC 
 
  191
 
  5
 
 15101
 
 tss
 
 0.017100
 
 0.0075444
 
 Not queried
 
  0.6035301
 
       NA
 
           NA
  
 
  
 12
 
 133281175
 
 133281504
 
  ENSEMBL   UCSC 
 
  330
 
  4
 
 9454
 
 gene
 
 0.974514
 
 0.9704972
 
 Not queried
 
  0.6035272
 
       NA
 
           NA
  
 
  
 3
 
  49577229
 
  49577663
 
  ENSEMBL   UCSC 
 
  435
 
  4
 
 29121
 
 island
 
 0.009983
 
 0.0077564
 
 Not queried
 
  0.6034871
 
       NA
 
           NA
  
 
  
 11
 
    405846
 
    406019
 
  ENSEMBL   UCSC 
 
  174
 
  4
 
 6371
 
 gene;island
 
 0.928758
 
 0.9596856
 
 Not queried
 
  0.6034276
 
       NA
 
           NA
  
 
  
 4
 
   5052379
 
   5052795
 
  ENSEMBL   UCSC 
 
  417
 
  6
 
 30273
 
 tss;island
 
 0.015485
 
 0.0178829
 
 Not queried
 
  0.6034211
 
       NA
 
           NA
  
 
  
 11
 
 128712280
 
 128712697
 
  ENSEMBL   UCSC 
 
  418
 
  6
 
 6968
 
 gene
 
 0.924571
 
 0.8505896
 
 Not queried
 
  0.6033669
 
       NA
 
           NA
  
 
  
 19
 
  13906139
 
  13906241
 
  ENSEMBL   UCSC 
 
  103
 
  5
 
 20529
 
 tss;island
 
 0.004934
 
 0.0079212
 
 Not queried
 
  0.6033527
 
       NA
 
           NA
  
 
  
 12
 
  69979077
 
  69979219
 
  ENSEMBL   UCSC 
 
  143
 
  5
 
 10616
 
 island
 
 0.010900
 
 0.0056915
 
 Not queried
 
  0.6030521
 
       NA
 
           NA
  
 
  
 19
 
   3500813
 
   3501167
 
  ENSEMBL   UCSC 
 
  355
 
  5
 
 20344
 
 tss;island
 
 0.009568
 
 0.0066731
 
 Not queried
 
  0.6030327
 
       NA
 
           NA
  
 
  
 3
 
   2139977
 
   2140256
 
  ENSEMBL   UCSC 
 
  280
 
  4
 
 28832
 
 island
 
 0.032774
 
 0.0637140
 
 Not queried
 
  0.6030263
 
       NA
 
           NA
  
 
  
 20
 
  49411195
 
  49411594
 
  ENSEMBL   UCSC 
 
  400
 
  6
 
 26250
 
 island
 
 0.008395
 
 0.0050515
 
 Not queried
 
  0.6029245
 
       NA
 
           NA
  
 
  
 5
 
 141061863
 
 141061943
 
  ENSEMBL   UCSC 
 
   81
 
  6
 
 32390
 
 tss
 
 0.036680
 
 0.0710288
 
 Not queried
 
  0.6027512
 
       NA
 
           NA
  
 
  
 8
 
 145016475
 
 145016828
 
  ENSEMBL   UCSC 
 
  354
 
  4
 
 39498
 
 gene;island
 
 0.007011
 
 0.0048048
 
 Not queried
 
  0.6027253
 
       NA
 
           NA
  
 
  
 7
 
  25989524
 
  25989763
 
  ENSEMBL   UCSC 
 
  240
 
  4
 
 38327
 
 island
 
 0.001317
 
 0.0012938
 
 Not queried
 
  0.6026989
 
       NA
 
           NA
  
 
  
 15
 
  72612556
 
  72613029
 
  ENSEMBL   UCSC 
 
  474
 
  7
 
 13775
 
 tss;island
 
 0.030794
 
 0.0115804
 
 Not queried
 
  0.6026973
 
       NA
 
           NA
  
 
  
 17
 
  29158893
 
  29158999
 
  ENSEMBL   UCSC 
 
  107
 
  6
 
 17243
 
 tss
 
 0.003075
 
 0.0014982
 
 Not queried
 
  0.6026776
 
       NA
 
           NA
  
 
  
 12
 
  66563381
 
  66563719
 
  ENSEMBL   UCSC 
 
  339
 
  5
 
 9206
 
 gene
 
 0.023651
 
 0.0224506
 
 Not queried
 
  0.6026197
 
       NA
 
           NA
  
 
  
 4
 
 159644558
 
 159644880
 
  ENSEMBL   UCSC 
 
  323
 
  9
 
 30657
 
 tss
 
 0.004986
 
 0.0037168
 
 Not queried
 
  0.6026190
 
       NA
 
           NA
  
 
  
 4
 
 159644465
 
 159644880
 
  ENSEMBL   UCSC 
 
  416
 
 11
 
 31398
 
 island
 
 0.004986
 
 0.0037168
 
 Not queried
 
  0.6026190
 
       NA
 
           NA
  
 
  
 1
 
  20250186
 
  20251013
 
  ENSEMBL   UCSC 
 
  828
 
  7
 
 1311
 
 tss
 
 0.229532
 
 0.3527401
 
 Not queried
 
  0.6024778
 
       NA
 
           NA
  
 
  
 2
 
  45878652
 
  45878977
 
  ENSEMBL   UCSC 
 
  326
 
  6
 
 23337
 
 tss;island
 
 0.012822
 
 0.0105032
 
 Not queried
 
  0.6024677
 
       NA
 
           NA
  
 
  
 8
 
  74360923
 
  74361427
 
  ENSEMBL   UCSC 
 
  505
 
  6
 
 39337
 
 gene;island
 
 0.968512
 
 0.9556388
 
 Not queried
 
  0.6022310
 
       NA
 
           NA
  
 
  
 16
 
  56998544
 
  56998712
 
  ENSEMBL   UCSC 
 
  169
 
  4
 
 14764
 
 gene
 
 0.004203
 
 0.0019756
 
 Not queried
 
  0.6022156
 
       NA
 
           NA
  
 
  
 5
 
   5422589
 
   5422880
 
  ENSEMBL   UCSC 
 
  292
 
  6
 
 32675
 
 island
 
 0.007142
 
 0.0072133
 
 Not queried
 
  0.6021005
 
       NA
 
           NA
  
 
  
 4
 
 149364543
 
 149364881
 
  ENSEMBL   UCSC 
 
  339
 
  4
 
 30630
 
 tss;island
 
 0.007623
 
 0.0100369
 
 Not queried
 
  0.6020088
 
       NA
 
           NA
  
 
  
 11
 
 126455596
 
 126456119
 
  ENSEMBL   UCSC 
 
  524
 
  6
 
 6963
 
 gene
 
 0.107372
 
 0.1553954
 
 Not queried
 
  0.6018809
 
       NA
 
           NA
  
 
  
 12
 
   8025394
 
   8025646
 
  ENSEMBL   UCSC 
 
  253
 
  7
 
 10229
 
 island
 
 0.368103
 
 0.3523872
 
 Not queried
 
  0.6018806
 
       NA
 
           NA
  
 
  
 13
 
  79980660
 
  79980793
 
  ENSEMBL   UCSC 
 
  134
 
  4
 
 11490
 
 tss;island
 
 0.004918
 
 0.0052433
 
 Not queried
 
  0.6018167
 
       NA
 
           NA
  
 
  
 19
 
   4607057
 
   4607324
 
  ENSEMBL   UCSC 
 
  268
 
  5
 
 21382
 
 island
 
 0.076336
 
 0.0875611
 
 Not queried
 
  0.6017733
 
       NA
 
           NA
  
 
  
 7
 
 149518783
 
 149519165
 
  ENSEMBL   UCSC 
 
  383
 
  4
 
 37267
 
 gene
 
 0.370732
 
 0.5455130
 
 Not queried
 
  0.6016592
 
       NA
 
           NA
  
 
  
 14
 
  60715218
 
  60715614
 
  ENSEMBL   UCSC 
 
  397
 
  4
 
 12102
 
 gene;tss;island
 
 0.003523
 
 0.0071677
 
 Not queried
 
  0.6016320
 
       NA
 
           NA
  
 
  
 12
 
  26986758
 
  26987164
 
  ENSEMBL   UCSC 
 
  407
 
 11
 
 9615
 
 tss;island
 
 0.003324
 
 0.0219859
 
 Not queried
 
  0.6015623
 
       NA
 
           NA
  
 
  
 17
 
  37356273
 
  37356554
 
  ENSEMBL   UCSC 
 
  282
 
  4
 
 18271
 
 island
 
 0.001776
 
 0.0046903
 
 Not queried
 
  0.6015526
 
       NA
 
           NA
  
 
  
 7
 
 129250987
 
 129251419
 
  ENSEMBL   UCSC 
 
  433
 
  5
 
 37893
 
 tss;island
 
 0.002692
 
 0.0023483
 
 Not queried
 
  0.6010999
 
       NA
 
           NA
  
 
  
 20
 
  32856747
 
  32857227
 
  ENSEMBL   UCSC 
 
  481
 
  6
 
 26107
 
 island
 
 0.211789
 
 0.2866314
 
 Not queried
 
  0.6008559
 
       NA
 
           NA
  
 
  
 16
 
  68481813
 
  68482277
 
  ENSEMBL   UCSC 
 
  465
 
  5
 
 14816
 
 gene;island
 
 0.030975
 
 0.0424432
 
 Not queried
 
  0.6008178
 
       NA
 
           NA
  
 
  
 19
 
  19843456
 
  19843912
 
  ENSEMBL   UCSC 
 
  457
 
  5
 
 19956
 
 gene
 
 0.018235
 
 0.0078879
 
 Not queried
 
  0.6006972
 
       NA
 
           NA
  
 
  
 12
 
  54982590
 
  54982717
 
  ENSEMBL   UCSC 
 
  128
 
  4
 
 9766
 
 tss;island
 
 0.052846
 
 0.0768209
 
 Not queried
 
  0.6005576
 
       NA
 
           NA
  
 
  
 9
 
 137533141
 
 137533366
 
  ENSEMBL   UCSC 
 
  226
 
  4
 
 40854
 
 tss;island
 
 0.008132
 
 0.0084908
 
 Not queried
 
  0.6004626
 
       NA
 
           NA
  
 
  
 12
 
   7858592
 
   7858848
 
  ENSEMBL   UCSC 
 
  257
 
  6
 
 10228
 
 island
 
 0.887876
 
 0.8572751
 
 Not queried
 
  0.6004362
 
       NA
 
           NA
  
 
  
 1
 
   2303963
 
   2304375
 
  ENSEMBL   UCSC 
 
  413
 
  5
 
 78
 
 gene;island
 
 0.479709
 
 0.5448908
 
 Not queried
 
  0.6003736
 
       NA
 
           NA
  
 
  
 2
 
 202645850
 
 202646121
 
  ENSEMBL   UCSC 
 
  272
 
  8
 
 25014
 
 island
 
 0.003121
 
 0.0018882
 
 Not queried
 
  0.6000583
 
       NA
 
           NA
  
 
  
 12
 
 117628347
 
 117628589
 
  ENSEMBL   UCSC 
 
  243
 
  7
 
 10044
 
 tss
 
 0.027365
 
 0.0104021
 
 Not queried
 
  0.6000283
 
       NA
 
           NA
  
 
  
 12
 
 128752040
 
 128752356
 
  ENSEMBL   UCSC 
 
  317
 
  4
 
 9405
 
 gene;island
 
 0.177555
 
 0.1043574
 
 Not queried
 
  0.5999371
 
       NA
 
           NA
  
 
  
 1
 
   1238436
 
   1238797
 
  ENSEMBL   UCSC 
 
  362
 
  5
 
 27
 
 gene;island
 
 0.926494
 
 0.9314930
 
 Not queried
 
  0.5999225
 
       NA
 
           NA
  
 
  
 10
 
   7708934
 
   7709073
 
  ENSEMBL   UCSC 
 
  140
 
  4
 
 4938
 
 tss
 
 0.007452
 
 0.0131688
 
 Not queried
 
  0.5999034
 
       NA
 
           NA
  
 
  
 19
 
  42580150
 
  42580259
 
  ENSEMBL   UCSC 
 
  110
 
  7
 
 20818
 
 tss
 
 0.005945
 
 0.0050956
 
 Not queried
 
  0.5998921
 
       NA
 
           NA
  
 
  
 19
 
  42580150
 
  42580541
 
  ENSEMBL   UCSC 
 
  392
 
  9
 
 22064
 
 island
 
 0.005945
 
 0.0050956
 
 Not queried
 
  0.5998921
 
       NA
 
           NA
  
 
  
 1
 
  35658023
 
  35658293
 
  ENSEMBL   UCSC 
 
  271
 
  5
 
 420
 
 gene;island
 
 0.006399
 
 0.0093055
 
 Not queried
 
  0.5998219
 
       NA
 
           NA
  
 
  
 3
 
  14220276
 
  14220525
 
  ENSEMBL   UCSC 
 
  250
 
  8
 
 27689
 
 gene
 
 0.003962
 
 0.0052916
 
 Not queried
 
  0.5996986
 
       NA
 
           NA
  
 
  
 7
 
 135242312
 
 135242648
 
  ENSEMBL   UCSC 
 
  337
 
 10
 
 37919
 
 tss
 
 0.004418
 
 0.0038648
 
 Not queried
 
  0.5996505
 
       NA
 
           NA
  
 
  
 1
 
  76251458
 
  76252133
 
  ENSEMBL   UCSC 
 
  676
 
 11
 
 1700
 
 tss
 
 0.005815
 
 0.0032114
 
 Not queried
 
  0.5996119
 
       NA
 
           NA
  
 
  
 5
 
  44809616
 
  44809978
 
  ENSEMBL   UCSC 
 
  363
 
  4
 
 31606
 
 gene;island
 
 0.005727
 
 0.0030291
 
 Not queried
 
  0.5995256
 
       NA
 
           NA
  
 
  
 8
 
  65281397
 
  65281689
 
  ENSEMBL   UCSC 
 
  293
 
  5
 
 40339
 
 island
 
 0.041717
 
 0.0345078
 
 Not queried
 
  0.5994027
 
       NA
 
           NA
  
 
  
 6
 
  31543169
 
  31543300
 
  ENSEMBL   UCSC 
 
  132
 
  4
 
 34819
 
 tss
 
 0.015872
 
 0.0185050
 
 Not queried
 
  0.5993150
 
       NA
 
           NA
  
 
  
 17
 
  48042605
 
  48043063
 
  ENSEMBL   UCSC 
 
  459
 
  4
 
 18533
 
 island
 
 0.025044
 
 0.0326179
 
 Not queried
 
  0.5993001
 
       NA
 
           NA
  
 
  
 20
 
  32856747
 
  32857072
 
  ENSEMBL   UCSC 
 
  326
 
  4
 
 25397
 
 gene
 
 0.363924
 
 0.4302038
 
 Not queried
 
  0.5992571
 
       NA
 
           NA
  
 
  
 6
 
  30127152
 
  30127389
 
  ENSEMBL   UCSC 
 
  238
 
  4
 
 33734
 
 gene
 
 0.523434
 
 0.6473759
 
 Not queried
 
  0.5991304
 
       NA
 
           NA
  
 
  
 15
 
  75182410
 
  75182590
 
  ENSEMBL   UCSC 
 
  181
 
  4
 
 13428
 
 gene
 
 0.006109
 
 0.0047274
 
 Not queried
 
  0.5990700
 
       NA
 
           NA
  
 
  
 11
 
   2398587
 
   2398856
 
  ENSEMBL   UCSC 
 
  270
 
  4
 
 6456
 
 gene
 
 0.017325
 
 0.0117607
 
 Not queried
 
  0.5990101
 
       NA
 
           NA
  
 
  
 12
 
   2224410
 
   2224755
 
  ENSEMBL   UCSC 
 
  346
 
  4
 
 9002
 
 gene;island
 
 0.203278
 
 0.3408430
 
 Not queried
 
  0.5989744
 
       NA
 
           NA
  
 
  
 13
 
  74708078
 
  74708194
 
  ENSEMBL   UCSC 
 
  117
 
  6
 
 11477
 
 tss
 
 0.004139
 
 0.0102810
 
 Not queried
 
  0.5988854
 
       NA
 
           NA
  
 
  
 6
 
  10723258
 
  10723702
 
  ENSEMBL   UCSC 
 
  445
 
  7
 
 33566
 
 gene
 
 0.003690
 
 0.0066194
 
 Not queried
 
  0.5988534
 
       NA
 
           NA
  
 
  
 8
 
   1105035
 
   1105422
 
  ENSEMBL   UCSC 
 
  388
 
  4
 
 40013
 
 island
 
 0.649623
 
 0.8141786
 
 Not queried
 
  0.5986961
 
       NA
 
           NA
  
 
  
 11
 
 126225941
 
 126226068
 
  ENSEMBL   UCSC 
 
  128
 
  6
 
 7800
 
 tss
 
 0.027625
 
 0.0227081
 
 Not queried
 
  0.5986629
 
       NA
 
           NA
  
 
  
 19
 
  35263485
 
  35264428
 
  ENSEMBL   UCSC 
 
  944
 
 16
 
 21891
 
 island
 
 0.008641
 
 0.0035789
 
 Not queried
 
  0.5986393
 
       NA
 
           NA
  
 
  
 3
 
 170302890
 
 170303286
 
  ENSEMBL   UCSC 
 
  397
 
  5
 
 28029
 
 gene;island
 
 0.026578
 
 0.1470115
 
 Not queried
 
  0.5986374
 
       NA
 
           NA
  
 
  
 4
 
 148653443
 
 148653624
 
  ENSEMBL   UCSC 
 
  182
 
  4
 
 31358
 
 island
 
 0.014584
 
 0.0108348
 
 Not queried
 
  0.5984728
 
       NA
 
           NA
  
 
  
 1
 
  45965343
 
  45965625
 
  ENSEMBL   UCSC 
 
  283
 
  4
 
 503
 
 gene
 
 0.005902
 
 0.0074787
 
 Not queried
 
  0.5984597
 
       NA
 
           NA
  
 
  
 20
 
   2644649
 
   2645106
 
  ENSEMBL   UCSC 
 
  458
 
 12
 
 25951
 
 island
 
 0.009921
 
 0.0045711
 
 Not queried
 
  0.5983382
 
       NA
 
           NA
  
 
  
 5
 
 179563738
 
 179563896
 
  ENSEMBL   UCSC 
 
  159
 
  5
 
 31939
 
 gene;island
 
 0.835823
 
 0.9017781
 
 Not queried
 
  0.5983198
 
       NA
 
           NA
  
 
  
 3
 
  50378191
 
  50378664
 
  ENSEMBL   UCSC 
 
  474
 
 11
 
 29153
 
 island
 
 0.008978
 
 0.0092455
 
 Not queried
 
  0.5982512
 
       NA
 
           NA
  
 
  
 15
 
  85174433
 
  85174687
 
  ENSEMBL   UCSC 
 
  255
 
  6
 
 13869
 
 tss
 
 0.004077
 
 0.0068808
 
 Not queried
 
  0.5981121
 
       NA
 
           NA
  
 
  
 11
 
  67070738
 
  67071064
 
  ENSEMBL   UCSC 
 
  327
 
  8
 
 8536
 
 island
 
 0.872517
 
 0.9133123
 
 Not queried
 
  0.5977988
 
       NA
 
           NA
  
 
  
 4
 
    331338
 
    331517
 
  ENSEMBL   UCSC 
 
  180
 
  7
 
 30223
 
 tss
 
 0.016026
 
 0.0089673
 
 Not queried
 
  0.5977928
 
       NA
 
           NA
  
 
  
 4
 
    331338
 
    331685
 
  ENSEMBL   UCSC 
 
  348
 
  9
 
 30716
 
 island
 
 0.016026
 
 0.0089673
 
 Not queried
 
  0.5977928
 
       NA
 
           NA
  
 
  
 10
 
  44070149
 
  44070200
 
  ENSEMBL   UCSC 
 
   52
 
  4
 
 5052
 
 tss
 
 0.036403
 
 0.0188041
 
 Not queried
 
  0.5977387
 
       NA
 
           NA
  
 
  
 6
 
 138866865
 
 138867125
 
  ENSEMBL   UCSC 
 
  261
 
  7
 
 34489
 
 gene
 
 0.972746
 
 0.9255241
 
 Not queried
 
  0.5975844
 
       NA
 
           NA
  
 
  
 17
 
  40687822
 
  40687943
 
  ENSEMBL   UCSC 
 
  122
 
  7
 
 17364
 
 tss
 
 0.009787
 
 0.0101817
 
 Not queried
 
  0.5974349
 
       NA
 
           NA
  
 
  
 16
 
  67969622
 
  67969984
 
  ENSEMBL   UCSC 
 
  363
 
  4
 
 14807
 
 gene;island
 
 0.013997
 
 0.0084484
 
 Not queried
 
  0.5974158
 
       NA
 
           NA
  
 
  
 16
 
  23724821
 
  23724943
 
  ENSEMBL   UCSC 
 
  123
 
  5
 
 15143
 
 tss
 
 0.784792
 
 0.8451465
 
 Not queried
 
  0.5974104
 
       NA
 
           NA
  
 
  
 6
 
 143381505
 
 143382018
 
  ENSEMBL   UCSC 
 
  514
 
  8
 
 35264
 
 tss
 
 0.013775
 
 0.0101107
 
 Not queried
 
  0.5973991
 
       NA
 
           NA
  
 
  
 1
 
 182758699
 
 182759062
 
  ENSEMBL   UCSC 
 
  364
 
  6
 
 4025
 
 island
 
 0.013331
 
 0.0072695
 
 Not queried
 
  0.5973542
 
       NA
 
           NA
  
 
  
 1
 
   3044741
 
   3045166
 
  ENSEMBL   UCSC 
 
  426
 
  6
 
 101
 
 gene
 
 0.291826
 
 0.5314699
 
 Not queried
 
  0.5972862
 
       NA
 
           NA
  
 
  
 1
 
 116710943
 
 116711077
 
  ENSEMBL   UCSC 
 
  135
 
  4
 
 3638
 
 island
 
 0.006544
 
 0.0090592
 
 Not queried
 
  0.5972117
 
       NA
 
           NA
  
 
  
 6
 
  46620708
 
  46620935
 
  ENSEMBL   UCSC 
 
  228
 
  4
 
 34359
 
 gene
 
 0.024118
 
 0.0101391
 
 Not queried
 
  0.5971524
 
       NA
 
           NA
  
 
  
 5
 
 146889536
 
 146889701
 
  ENSEMBL   UCSC 
 
  166
 
  4
 
 33289
 
 island
 
 0.039514
 
 0.0824138
 
 Not queried
 
  0.5970712
 
       NA
 
           NA
  
 
  
 5
 
 150080478
 
 150080579
 
  ENSEMBL   UCSC 
 
  102
 
  4
 
 31863
 
 gene
 
 0.009125
 
 0.0060654
 
 Not queried
 
  0.5969876
 
       NA
 
           NA
  
 
  
 16
 
  19078982
 
  19079607
 
  ENSEMBL   UCSC 
 
  626
 
  7
 
 14678
 
 gene
 
 0.005041
 
 0.0046519
 
 Not queried
 
  0.5966270
 
       NA
 
           NA
  
 
  
 17
 
  79045526
 
  79045877
 
  ENSEMBL   UCSC 
 
  352
 
  5
 
 16891
 
 gene;island
 
 0.731257
 
 0.8146264
 
 Not queried
 
  0.5965657
 
       NA
 
           NA
  
 
  
 19
 
  52800111
 
  52800391
 
  ENSEMBL   UCSC 
 
  281
 
  6
 
 21011
 
 tss
 
 0.049438
 
 0.0240334
 
 Not queried
 
  0.5963349
 
       NA
 
           NA
  
 
  
 12
 
   4829115
 
   4829690
 
  ENSEMBL   UCSC 
 
  576
 
  7
 
 9501
 
 tss
 
 0.229556
 
 0.4888735
 
 Not queried
 
  0.5963123
 
       NA
 
           NA
  
 
  
 1
 
  44411915
 
  44412380
 
  ENSEMBL   UCSC 
 
  466
 
  8
 
 1566
 
 tss
 
 0.003676
 
 0.0033849
 
 Not queried
 
  0.5962558
 
       NA
 
           NA
  
 
  
 1
 
  63791922
 
  63792239
 
  ENSEMBL   UCSC 
 
  318
 
  6
 
 3343
 
 island
 
 0.577442
 
 0.5081177
 
 Not queried
 
  0.5961144
 
       NA
 
           NA
  
 
  
 19
 
  56458926
 
  56459137
 
  ENSEMBL   UCSC 
 
  212
 
  4
 
 21091
 
 tss
 
 0.350027
 
 0.4487173
 
 Not queried
 
  0.5960814
 
       NA
 
           NA
  
 
  
 11
 
 124587993
 
 124588484
 
  ENSEMBL   UCSC 
 
  492
 
  5
 
 8914
 
 island
 
 0.016981
 
 0.0251174
 
 Not queried
 
  0.5960794
 
       NA
 
           NA
  
 
  
 7
 
 128809100
 
 128809442
 
  ENSEMBL   UCSC 
 
  343
 
  5
 
 38906
 
 island
 
 0.017735
 
 0.0932316
 
 Not queried
 
  0.5960554
 
       NA
 
           NA
  
 
  
 16
 
  56898573
 
  56899113
 
  ENSEMBL   UCSC 
 
  541
 
  6
 
 15254
 
 tss
 
 0.532775
 
 0.7841031
 
 Not queried
 
  0.5960511
 
       NA
 
           NA
  
 
  
 12
 
  51984943
 
  51985016
 
  ENSEMBL   UCSC 
 
   74
 
  5
 
 9718
 
 tss
 
 0.012522
 
 0.0165215
 
 Not queried
 
  0.5960175
 
       NA
 
           NA
  
 
  
 12
 
  51984943
 
  51985263
 
  ENSEMBL   UCSC 
 
  321
 
  7
 
 10428
 
 island
 
 0.012522
 
 0.0165215
 
 Not queried
 
  0.5960175
 
       NA
 
           NA
  
 
  
 22
 
  19512903
 
  19513176
 
  ENSEMBL   UCSC 
 
  274
 
  8
 
 26961
 
 tss
 
 0.771729
 
 0.8462171
 
 Not queried
 
  0.5959557
 
       NA
 
           NA
  
 
  
 11
 
  64546100
 
  64546224
 
  ENSEMBL   UCSC 
 
  125
 
  5
 
 6695
 
 gene
 
 0.004780
 
 0.0065131
 
 Not queried
 
  0.5958650
 
       NA
 
           NA
  
 
  
 7
 
  15725086
 
  15725652
 
  ENSEMBL   UCSC 
 
  567
 
  5
 
 36902
 
 gene
 
 0.082918
 
 0.0416500
 
 Not queried
 
  0.5958399
 
       NA
 
           NA
  
 
  
 12
 
  50185413
 
  50185781
 
  ENSEMBL   UCSC 
 
  369
 
  4
 
 9123
 
 gene;island
 
 0.967468
 
 0.9482997
 
 Not queried
 
  0.5957767
 
       NA
 
           NA
  
 
  
 1
 
 151512592
 
 151512707
 
  ENSEMBL   UCSC 
 
  116
 
  4
 
 1928
 
 tss;island
 
 0.055027
 
 0.0225274
 
 Not queried
 
  0.5956776
 
       NA
 
           NA
  
 
  
 7
 
 148922922
 
 148923218
 
  ENSEMBL   UCSC 
 
  297
 
  5
 
 37264
 
 gene;island
 
 0.934900
 
 0.9497924
 
 Not queried
 
  0.5956320
 
       NA
 
           NA
  
 
  
 6
 
  24403062
 
  24403143
 
  ENSEMBL   UCSC 
 
   82
 
  6
 
 34668
 
 tss
 
 0.017786
 
 0.0107524
 
 Not queried
 
  0.5955415
 
       NA
 
           NA
  
 
  
 14
 
  94595985
 
  94596163
 
  ENSEMBL   UCSC 
 
  179
 
  6
 
 12582
 
 tss
 
 0.004478
 
 0.0020466
 
 Not queried
 
  0.5954904
 
       NA
 
           NA
  
 
  
 1
 
 184943911
 
 184944288
 
  ENSEMBL   UCSC 
 
  378
 
  4
 
 2139
 
 tss;island
 
 0.828808
 
 0.5454630
 
 Not queried
 
  0.5954308
 
       NA
 
           NA
  
 
  
 19
 
  42387947
 
  42388476
 
  ENSEMBL   UCSC 
 
  530
 
  9
 
 20062
 
 gene;island
 
 0.015129
 
 0.0094187
 
 Not queried
 
  0.5953981
 
       NA
 
           NA
  
 
  
 19
 
  42387947
 
  42388348
 
  ENSEMBL   UCSC 
 
  402
 
  7
 
 20816
 
 tss
 
 0.014279
 
 0.0094187
 
 Not queried
 
  0.5953981
 
       NA
 
           NA
  
 
  
 15
 
  40986702
 
  40987368
 
  ENSEMBL   UCSC 
 
  667
 
 11
 
 13592
 
 tss
 
 0.008098
 
 0.0084918
 
 Not queried
 
  0.5953976
 
       NA
 
           NA
  
 
  
 16
 
   4783755
 
   4784147
 
  ENSEMBL   UCSC 
 
  393
 
  7
 
 14644
 
 gene
 
 0.010420
 
 0.0043874
 
 Not queried
 
  0.5953967
 
       NA
 
           NA
  
 
  
 7
 
  77648378
 
  77648624
 
  ENSEMBL   UCSC 
 
  247
 
  4
 
 37063
 
 gene;island
 
 0.007943
 
 0.0078172
 
 Not queried
 
  0.5953661
 
       NA
 
           NA
  
 
  
 10
 
 104263680
 
 104264023
 
  ENSEMBL   UCSC 
 
  344
 
  9
 
 6092
 
 island
 
 0.018078
 
 0.0106010
 
 Not queried
 
  0.5953333
 
       NA
 
           NA
  
 
  
 19
 
  15550742
 
  15550995
 
  ENSEMBL   UCSC 
 
  254
 
  5
 
 19906
 
 gene;island
 
 0.847000
 
 0.8935798
 
 Not queried
 
  0.5952598
 
       NA
 
           NA
  
 
  
 11
 
  70244230
 
  70244470
 
  ENSEMBL   UCSC 
 
  241
 
  5
 
 7528
 
 tss;island
 
 0.013777
 
 0.0076123
 
 Not queried
 
  0.5951365
 
       NA
 
           NA
  
 
  
 19
 
  46580305
 
  46580807
 
  ENSEMBL   UCSC 
 
  503
 
  4
 
 22169
 
 island
 
 0.047353
 
 0.0253610
 
 Not queried
 
  0.5951192
 
       NA
 
           NA
  
 
  
 3
 
  57204106
 
  57204530
 
  ENSEMBL   UCSC 
 
  425
 
  7
 
 29223
 
 island
 
 0.002440
 
 0.0032105
 
 Not queried
 
  0.5951074
 
       NA
 
           NA
  
 
  
 22
 
  37915250
 
  37915449
 
  ENSEMBL   UCSC 
 
  200
 
  6
 
 27117
 
 tss
 
 0.007843
 
 0.0054908
 
 Not queried
 
  0.5948046
 
       NA
 
           NA
  
 
  
 4
 
 184826215
 
 184826479
 
  ENSEMBL   UCSC 
 
  265
 
  9
 
 30693
 
 tss;island
 
 0.017916
 
 0.0121119
 
 Not queried
 
  0.5946682
 
       NA
 
           NA
  
 
  
 1
 
 207627941
 
 207628182
 
  ENSEMBL   UCSC 
 
  242
 
  4
 
 977
 
 gene;island
 
 0.007969
 
 0.0042138
 
 Not queried
 
  0.5945497
 
       NA
 
           NA
  
 
  
 5
 
 150080478
 
 150080927
 
  ENSEMBL   UCSC 
 
  450
 
 13
 
 33310
 
 island
 
 0.005373
 
 0.0019324
 
 Not queried
 
  0.5945205
 
       NA
 
           NA
  
 
  
 7
 
   6048614
 
   6048715
 
  ENSEMBL   UCSC 
 
  102
 
  5
 
 36882
 
 gene
 
 0.002195
 
 0.0036349
 
 Not queried
 
  0.5944080
 
       NA
 
           NA
  
 
  
 19
 
  58739734
 
  58740004
 
  ENSEMBL   UCSC 
 
  271
 
  7
 
 21136
 
 tss;island
 
 0.017672
 
 0.0111180
 
 Not queried
 
  0.5943822
 
       NA
 
           NA
  
 
  
 6
 
  21666391
 
  21666939
 
  ENSEMBL   UCSC 
 
  549
 
  8
 
 35486
 
 island
 
 0.018307
 
 0.0218317
 
 Not queried
 
  0.5943746
 
       NA
 
           NA
  
 
  
 1
 
   3606550
 
   3606892
 
  ENSEMBL   UCSC 
 
  343
 
  4
 
 137
 
 gene;tss;island
 
 0.863160
 
 0.6471677
 
 Not queried
 
  0.5943117
 
       NA
 
           NA
  
 
  
 1
 
   6240267
 
   6240508
 
  ENSEMBL   UCSC 
 
  242
 
  5
 
 1197
 
 tss;island
 
 0.062787
 
 0.0286556
 
 Not queried
 
  0.5942617
 
       NA
 
           NA
  
 
  
 22
 
  39898407
 
  39898620
 
  ENSEMBL   UCSC 
 
  214
 
  4
 
 26879
 
 gene
 
 0.004673
 
 0.0032311
 
 Not queried
 
  0.5941448
 
       NA
 
           NA
  
 
  
 6
 
 117586538
 
 117586681
 
  ENSEMBL   UCSC 
 
  144
 
  5
 
 35195
 
 tss
 
 0.069492
 
 0.0471278
 
 Not queried
 
  0.5938549
 
       NA
 
           NA
  
 
  
 6
 
 117586538
 
 117586918
 
  ENSEMBL   UCSC 
 
  381
 
  7
 
 36436
 
 island
 
 0.069492
 
 0.0471278
 
 Not queried
 
  0.5938549
 
       NA
 
           NA
  
 
  
 11
 
  93394605
 
  93394910
 
  ENSEMBL   UCSC 
 
  306
 
 10
 
 8729
 
 island
 
 0.004121
 
 0.0060210
 
 Not queried
 
  0.5938287
 
       NA
 
           NA
  
 
  
 2
 
 191513055
 
 191513482
 
  ENSEMBL   UCSC 
 
  428
 
  6
 
 23786
 
 tss;island
 
 0.008346
 
 0.0068346
 
 Not queried
 
  0.5937880
 
       NA
 
           NA
  
 
  
 7
 
  89748633
 
  89748810
 
  ENSEMBL   UCSC 
 
  178
 
  7
 
 38644
 
 island
 
 0.060782
 
 0.0683004
 
 Not queried
 
  0.5937792
 
       NA
 
           NA
  
 
  
 4
 
 123653523
 
 123654127
 
  ENSEMBL   UCSC 
 
  605
 
 10
 
 30580
 
 tss;island
 
 0.012565
 
 0.0101678
 
 Not queried
 
  0.5937784
 
       NA
 
           NA
  
 
  
 7
 
 102105124
 
 102105344
 
  ENSEMBL   UCSC 
 
  221
 
  6
 
 37804
 
 tss;island
 
 0.001794
 
 0.0028762
 
 Not queried
 
  0.5936907
 
       NA
 
           NA
  
 
  
 17
 
  40540457
 
  40540860
 
  ENSEMBL   UCSC 
 
  404
 
 10
 
 17361
 
 tss;island
 
 0.006462
 
 0.0101576
 
 Not queried
 
  0.5936737
 
       NA
 
           NA
  
 
  
 4
 
  87927655
 
  87928590
 
  ENSEMBL   UCSC 
 
  936
 
 13
 
 30052
 
 gene;island
 
 0.009606
 
 0.0071936
 
 Not queried
 
  0.5936478
 
       NA
 
           NA
  
 
  
 2
 
  60780746
 
  60780809
 
  ENSEMBL   UCSC 
 
   64
 
  4
 
 23370
 
 tss;island
 
 0.010369
 
 0.0183435
 
 Not queried
 
  0.5936070
 
       NA
 
           NA
  
 
  
 7
 
  96651915
 
  96652481
 
  ENSEMBL   UCSC 
 
  567
 
 10
 
 37106
 
 gene;island
 
 0.040103
 
 0.0687451
 
 Not queried
 
  0.5936039
 
       NA
 
           NA
  
 
  
 19
 
  56826044
 
  56826442
 
  ENSEMBL   UCSC 
 
  399
 
  4
 
 22442
 
 island
 
 0.001003
 
 0.0007777
 
 Not queried
 
  0.5935441
 
       NA
 
           NA
  
 
  
 20
 
  21685834
 
  21686728
 
  ENSEMBL   UCSC 
 
  895
 
 14
 
 26042
 
 island
 
 0.050857
 
 0.0598476
 
 Not queried
 
  0.5935204
 
       NA
 
           NA
  
 
  
 11
 
 111944977
 
 111945650
 
  ENSEMBL   UCSC 
 
  674
 
  8
 
 6905
 
 gene
 
 0.012361
 
 0.0066891
 
 Not queried
 
  0.5932373
 
       NA
 
           NA
  
 
  
 19
 
  49522683
 
  49523046
 
  ENSEMBL   UCSC 
 
  364
 
  4
 
 22249
 
 island
 
 0.229367
 
 0.1749162
 
 Not queried
 
  0.5931940
 
       NA
 
           NA
  
 
  
 5
 
  75698309
 
  75698508
 
  ENSEMBL   UCSC 
 
  200
 
  4
 
 32133
 
 tss;island
 
 0.008835
 
 0.0045168
 
 Not queried
 
  0.5931601
 
       NA
 
           NA
  
 
  
 5
 
 140855463
 
 140855582
 
  ENSEMBL   UCSC 
 
  120
 
  7
 
 31837
 
 gene;island
 
 0.012747
 
 0.0035334
 
 Not queried
 
  0.5930791
 
       NA
 
           NA
  
 
  
 12
 
  62586267
 
  62586892
 
  ENSEMBL   UCSC 
 
  626
 
  8
 
 10581
 
 island
 
 0.002671
 
 0.0055859
 
 Not queried
 
  0.5929617
 
       NA
 
           NA
  
 
  
 6
 
  36237982
 
  36238230
 
  ENSEMBL   UCSC 
 
  249
 
  7
 
 34935
 
 tss
 
 0.347922
 
 0.3684872
 
 Not queried
 
  0.5927603
 
       NA
 
           NA
  
 
  
 2
 
 154335593
 
 154335766
 
  ENSEMBL   UCSC 
 
  174
 
  4
 
 23668
 
 tss;island
 
 0.013050
 
 0.0117837
 
 Not queried
 
  0.5927321
 
       NA
 
           NA
  
 
  
 1
 
  11724057
 
  11724943
 
  ENSEMBL   UCSC 
 
  887
 
 10
 
 2683
 
 island
 
 0.006919
 
 0.0047791
 
 Not queried
 
  0.5925876
 
       NA
 
           NA
  
 
  
 8
 
  33370637
 
  33370902
 
  ENSEMBL   UCSC 
 
  266
 
  7
 
 40213
 
 island
 
 0.011703
 
 0.0060847
 
 Not queried
 
  0.5924959
 
       NA
 
           NA
  
 
  
 3
 
  85007732
 
  85008105
 
  ENSEMBL   UCSC 
 
  374
 
  7
 
 28450
 
 tss
 
 0.013927
 
 0.0239768
 
 Not queried
 
  0.5924950
 
       NA
 
           NA
  
 
  
 6
 
  57086154
 
  57087066
 
  ENSEMBL   UCSC 
 
  913
 
  8
 
 34377
 
 gene
 
 0.015269
 
 0.0108227
 
 Not queried
 
  0.5924348
 
       NA
 
           NA
  
 
  
 6
 
  43197151
 
  43197675
 
  ENSEMBL   UCSC 
 
  525
 
  9
 
 36157
 
 island
 
 0.008721
 
 0.0055867
 
 Not queried
 
  0.5923091
 
       NA
 
           NA
  
 
  
 12
 
  42719544
 
  42720167
 
  ENSEMBL   UCSC 
 
  624
 
 13
 
 9095
 
 gene;tss;island
 
 0.013633
 
 0.0087724
 
 Not queried
 
  0.5923040
 
       NA
 
           NA
  
 
  
 6
 
  58287694
 
  58287938
 
  ENSEMBL   UCSC 
 
  245
 
  6
 
 36227
 
 island
 
 0.016989
 
 0.0045343
 
 Not queried
 
  0.5921524
 
       NA
 
           NA
  
 
  
 8
 
  56436393
 
  56436771
 
  ENSEMBL   UCSC 
 
  379
 
  5
 
 39310
 
 gene
 
 0.656957
 
 0.8593931
 
 Not queried
 
  0.5920183
 
       NA
 
           NA
  
 
  
 2
 
  48844728
 
  48844916
 
  ENSEMBL   UCSC 
 
  189
 
  5
 
 23347
 
 tss
 
 0.742466
 
 0.8813447
 
 Not queried
 
  0.5919325
 
       NA
 
           NA
  
 
  
 1
 
   5932351
 
   5932678
 
  ENSEMBL   UCSC 
 
  328
 
  5
 
 155
 
 gene
 
 0.971689
 
 0.9735766
 
 Not queried
 
  0.5918910
 
       NA
 
           NA
  
 
  
 4
 
  37245916
 
  37246280
 
  ENSEMBL   UCSC 
 
  365
 
  4
 
 30340
 
 tss;island
 
 0.024172
 
 0.0379048
 
 Not queried
 
  0.5918809
 
       NA
 
           NA
  
 
  
 6
 
  33374613
 
  33374667
 
  ENSEMBL   UCSC 
 
   55
 
  5
 
 34276
 
 gene;island
 
 0.982604
 
 0.9774180
 
 Not queried
 
  0.5917470
 
       NA
 
           NA
  
 
  
 7
 
  50132903
 
  50133212
 
  ENSEMBL   UCSC 
 
  310
 
  8
 
 37593
 
 tss
 
 0.836847
 
 0.9832667
 
 Not queried
 
  0.5917116
 
       NA
 
           NA
  
 
  
 2
 
 177053274
 
 177053297
 
  ENSEMBL   UCSC 
 
   24
 
  6
 
 23744
 
 tss
 
 0.021644
 
 0.0359195
 
 Not queried
 
  0.5915527
 
       NA
 
           NA
  
 
  
 5
 
 134734932
 
 134735260
 
  ENSEMBL   UCSC 
 
  329
 
  6
 
 32302
 
 tss
 
 0.005942
 
 0.0049231
 
 Not queried
 
  0.5914755
 
       NA
 
           NA
  
 
  
 16
 
  75148330
 
  75148743
 
  ENSEMBL   UCSC 
 
  414
 
  4
 
 14842
 
 gene;island
 
 0.863450
 
 0.8974298
 
 Not queried
 
  0.5914412
 
       NA
 
           NA
  
 
  
 1
 
  55230344
 
  55230770
 
  ENSEMBL   UCSC 
 
  427
 
  8
 
 1647
 
 tss
 
 0.006562
 
 0.0039681
 
 Not queried
 
  0.5913890
 
       NA
 
           NA
  
 
  
 3
 
  48487808
 
  48488219
 
  ENSEMBL   UCSC 
 
  412
 
  7
 
 29088
 
 island
 
 0.018601
 
 0.0085873
 
 Not queried
 
  0.5913737
 
       NA
 
           NA
  
 
  
 13
 
  31479935
 
  31480335
 
  ENSEMBL   UCSC 
 
  401
 
  7
 
 11651
 
 island
 
 0.214409
 
 0.4687548
 
 Not queried
 
  0.5913120
 
       NA
 
           NA
  
 
  
 13
 
 113301636
 
 113301782
 
  ENSEMBL   UCSC 
 
  147
 
  4
 
 11248
 
 gene;island
 
 0.798756
 
 0.7496589
 
 Not queried
 
  0.5912897
 
       NA
 
           NA
  
 
  
 3
 
  27762636
 
  27763266
 
  ENSEMBL   UCSC 
 
  631
 
  5
 
 27707
 
 gene;island
 
 0.021672
 
 0.0441496
 
 Not queried
 
  0.5912417
 
       NA
 
           NA
  
 
  
 7
 
 140714412
 
 140714747
 
  ENSEMBL   UCSC 
 
  336
 
  9
 
 37249
 
 gene
 
 0.002339
 
 0.0016012
 
 Not queried
 
  0.5911655
 
       NA
 
           NA
  
 
  
 10
 
 134404873
 
 134405566
 
  ENSEMBL   UCSC 
 
  694
 
  8
 
 4867
 
 gene
 
 0.952235
 
 0.9817629
 
 Not queried
 
  0.5911434
 
       NA
 
           NA
  
 
  
 17
 
   6939138
 
   6939304
 
  ENSEMBL   UCSC 
 
  167
 
  6
 
 17059
 
 tss;island
 
 0.002629
 
 0.0069575
 
 Not queried
 
  0.5911137
 
       NA
 
           NA
  
 
  
 3
 
   9920535
 
   9920698
 
  ENSEMBL   UCSC 
 
  164
 
  4
 
 27656
 
 gene
 
 0.546636
 
 0.7254324
 
 Not queried
 
  0.5909429
 
       NA
 
           NA
  
 
  
 10
 
   5566543
 
   5566908
 
  ENSEMBL   UCSC 
 
  366
 
  6
 
 4926
 
 tss;island
 
 0.501345
 
 0.5849092
 
 Not queried
 
  0.5909191
 
       NA
 
           NA
  
 
  
 12
 
 113592319
 
 113592619
 
  ENSEMBL   UCSC 
 
  301
 
  4
 
 9321
 
 gene;island
 
 0.023846
 
 0.0256390
 
 Not queried
 
  0.5908527
 
       NA
 
           NA
  
 
  
 13
 
  99630462
 
  99630583
 
  ENSEMBL   UCSC 
 
  122
 
  4
 
 11203
 
 gene;tss
 
 0.522353
 
 0.6574606
 
 Not queried
 
  0.5908345
 
       NA
 
           NA
  
 
  
 14
 
  37132291
 
  37132774
 
  ENSEMBL   UCSC 
 
  484
 
  5
 
 12067
 
 gene;island
 
 0.183359
 
 0.1103978
 
 Not queried
 
  0.5908098
 
       NA
 
           NA
  
 
  
 19
 
  48894882
 
  48895030
 
  ENSEMBL   UCSC 
 
  149
 
  7
 
 20913
 
 tss
 
 0.007502
 
 0.0047406
 
 Not queried
 
  0.5906170
 
       NA
 
           NA
  
 
  
 19
 
  48894694
 
  48895030
 
  ENSEMBL   UCSC 
 
  337
 
  9
 
 22212
 
 island
 
 0.004990
 
 0.0029872
 
 Not queried
 
  0.5906170
 
       NA
 
           NA
  
 
  
 1
 
 116915511
 
 116915824
 
  ENSEMBL   UCSC 
 
  314
 
  5
 
 1862
 
 tss;island
 
 0.006596
 
 0.0062533
 
 Not queried
 
  0.5905388
 
       NA
 
           NA
  
 
  
 1
 
   6845395
 
   6845484
 
  ENSEMBL   UCSC 
 
   90
 
  5
 
 182
 
 gene
 
 0.012285
 
 0.0073184
 
 Not queried
 
  0.5904176
 
       NA
 
           NA
  
 
  
 19
 
  40909474
 
  40909817
 
  ENSEMBL   UCSC 
 
  344
 
  4
 
 20041
 
 gene;island
 
 0.009153
 
 0.0059014
 
 Not queried
 
  0.5903854
 
       NA
 
           NA
  
 
  
 1
 
 201123745
 
 201124117
 
  ENSEMBL   UCSC 
 
  373
 
  7
 
 2164
 
 tss
 
 0.528567
 
 0.1891516
 
 Not queried
 
  0.5902941
 
       NA
 
           NA
  
 
  
 14
 
 105359836
 
 105360085
 
  ENSEMBL   UCSC 
 
  250
 
  4
 
 12254
 
 gene;island
 
 0.544121
 
 0.5562443
 
 Not queried
 
  0.5902656
 
       NA
 
           NA
  
 
  
 2
 
 219824293
 
 219824395
 
  ENSEMBL   UCSC 
 
  103
 
  5
 
 23886
 
 tss
 
 0.003316
 
 0.0025914
 
 Not queried
 
  0.5902652
 
       NA
 
           NA
  
 
  
 2
 
  99225218
 
  99225662
 
  ENSEMBL   UCSC 
 
  445
 
  6
 
 22804
 
 gene;tss;island
 
 0.011847
 
 0.0102408
 
 Not queried
 
  0.5902545
 
       NA
 
           NA
  
 
  
 1
 
  27114614
 
  27114831
 
  ENSEMBL   UCSC 
 
  218
 
  4
 
 344
 
 gene
 
 0.013356
 
 0.0086309
 
 Not queried
 
  0.5901841
 
       NA
 
           NA
  
 
  
 4
 
 157996959
 
 157997880
 
  ENSEMBL   UCSC 
 
  922
 
 15
 
 31394
 
 island
 
 0.026550
 
 0.0526419
 
 Not queried
 
  0.5901367
 
       NA
 
           NA
  
 
  
 5
 
  93953866
 
  93954303
 
  ENSEMBL   UCSC 
 
  438
 
  9
 
 31694
 
 gene
 
 0.007328
 
 0.0062952
 
 Not queried
 
  0.5900555
 
       NA
 
           NA
  
 
  
 11
 
  71320513
 
  71320766
 
  ENSEMBL   UCSC 
 
  254
 
  4
 
 8622
 
 island
 
 0.705754
 
 0.8161014
 
 Not queried
 
  0.5899709
 
       NA
 
           NA
  
 
  
 5
 
    693476
 
    693638
 
  ENSEMBL   UCSC 
 
  163
 
  4
 
 32593
 
 island
 
 0.007076
 
 0.0060164
 
 Not queried
 
  0.5899157
 
       NA
 
           NA
  
 
  
 3
 
 188664632
 
 188664993
 
  ENSEMBL   UCSC 
 
  362
 
  6
 
 29766
 
 island
 
 0.605832
 
 0.6958924
 
 Not queried
 
  0.5898943
 
       NA
 
           NA
  
 
  
 1
 
  89664260
 
  89664582
 
  ENSEMBL   UCSC 
 
  323
 
  6
 
 619
 
 gene
 
 0.005373
 
 0.0033275
 
 Not queried
 
  0.5898084
 
       NA
 
           NA
  
 
  
 18
 
  72264895
 
  72265022
 
  ENSEMBL   UCSC 
 
  128
 
  4
 
 19128
 
 gene
 
 0.006470
 
 0.0061236
 
 Not queried
 
  0.5895409
 
       NA
 
           NA
  
 
  
 1
 
  21616619
 
  21617100
 
  ENSEMBL   UCSC 
 
  482
 
  5
 
 295
 
 gene;island
 
 0.007255
 
 0.0080027
 
 Not queried
 
  0.5893437
 
       NA
 
           NA
  
 
  
 17
 
  48227965
 
  48228143
 
  ENSEMBL   UCSC 
 
  179
 
  4
 
 17493
 
 tss;island
 
 0.087570
 
 0.0457297
 
 Not queried
 
  0.5893405
 
       NA
 
           NA
  
 
  
 3
 
  37902229
 
  37902741
 
  ENSEMBL   UCSC 
 
  513
 
  5
 
 28223
 
 tss;island
 
 0.018410
 
 0.0100417
 
 Not queried
 
  0.5893267
 
       NA
 
           NA
  
 
  
 11
 
   2884118
 
   2884460
 
  ENSEMBL   UCSC 
 
  343
 
  5
 
 7975
 
 island
 
 0.064911
 
 0.0809398
 
 Not queried
 
  0.5892223
 
       NA
 
           NA
  
 
  
 2
 
  71680559
 
  71680761
 
  ENSEMBL   UCSC 
 
  203
 
  5
 
 24399
 
 island
 
 0.032397
 
 0.0473271
 
 Not queried
 
  0.5891112
 
       NA
 
           NA
  
 
  
 8
 
 144328715
 
 144329027
 
  ENSEMBL   UCSC 
 
  313
 
  7
 
 39945
 
 tss
 
 0.005587
 
 0.0028317
 
 Not queried
 
  0.5890312
 
       NA
 
           NA
  
 
  
 3
 
 194117018
 
 194117403
 
  ENSEMBL   UCSC 
 
  386
 
  4
 
 28090
 
 gene;island
 
 0.023749
 
 0.1012332
 
 Not queried
 
  0.5889974
 
       NA
 
           NA
  
 
  
 11
 
 101917847
 
 101918710
 
  ENSEMBL   UCSC 
 
  864
 
 11
 
 8758
 
 island
 
 0.026910
 
 0.0360159
 
 Not queried
 
  0.5889391
 
       NA
 
           NA
  
 
  
 1
 
  10002719
 
  10003752
 
  ENSEMBL   UCSC 
 
 1034
 
 12
 
 1232
 
 tss;island
 
 0.009890
 
 0.0067865
 
 Not queried
 
  0.5889324
 
       NA
 
           NA
  
 
  
 2
 
  68290165
 
  68290497
 
  ENSEMBL   UCSC 
 
  333
 
  5
 
 23399
 
 tss
 
 0.009456
 
 0.0071733
 
 Not queried
 
  0.5889270
 
       NA
 
           NA
  
 
  
 1
 
 154744644
 
 154744863
 
  ENSEMBL   UCSC 
 
  220
 
  4
 
 784
 
 gene;island
 
 0.925827
 
 0.9452373
 
 Not queried
 
  0.5889185
 
       NA
 
           NA
  
 
  
 6
 
  99291343
 
  99291677
 
  ENSEMBL   UCSC 
 
  335
 
  4
 
 36346
 
 island
 
 0.060108
 
 0.0214177
 
 Not queried
 
  0.5887573
 
       NA
 
           NA
  
 
  
 6
 
  29975675
 
  29976071
 
  ENSEMBL   UCSC 
 
  397
 
  4
 
 33707
 
 gene;island
 
 0.793424
 
 0.8357905
 
 Not queried
 
  0.5886392
 
       NA
 
           NA
  
 
  
 8
 
 144328715
 
 144329279
 
  ENSEMBL   UCSC 
 
  565
 
 10
 
 40649
 
 island
 
 0.004920
 
 0.0028182
 
 Not queried
 
  0.5884898
 
       NA
 
           NA
  
 
  
 17
 
  78156060
 
  78156552
 
  ENSEMBL   UCSC 
 
  493
 
  6
 
 16857
 
 gene;island
 
 0.626839
 
 0.8089057
 
 Not queried
 
  0.5884731
 
       NA
 
           NA
  
 
  
 2
 
  73114144
 
  73114426
 
  ENSEMBL   UCSC 
 
  283
 
  9
 
 23434
 
 tss
 
 0.018477
 
 0.0088817
 
 Not queried
 
  0.5883002
 
       NA
 
           NA
  
 
  
 12
 
 133419166
 
 133419329
 
  ENSEMBL   UCSC 
 
  164
 
  4
 
 9465
 
 gene;island
 
 0.910415
 
 0.9371298
 
 Not queried
 
  0.5881833
 
       NA
 
           NA
  
 
  
 1
 
  34642000
 
  34642899
 
  ENSEMBL   UCSC 
 
  900
 
 13
 
 410
 
 gene;island
 
 0.175221
 
 0.4094729
 
 Not queried
 
  0.5880935
 
       NA
 
           NA
  
 
  
 1
 
 231664136
 
 231664671
 
  ENSEMBL   UCSC 
 
  536
 
 11
 
 4328
 
 island
 
 0.010825
 
 0.0064931
 
 Not queried
 
  0.5880797
 
       NA
 
           NA
  
 
  
 1
 
 240255377
 
 240255691
 
  ENSEMBL   UCSC 
 
  315
 
  4
 
 1105
 
 gene;island
 
 0.069250
 
 0.0425182
 
 Not queried
 
  0.5880759
 
       NA
 
           NA
  
 
  
 2
 
 208989324
 
 208989579
 
  ENSEMBL   UCSC 
 
  256
 
  5
 
 23846
 
 tss
 
 0.166078
 
 0.0975279
 
 Not queried
 
  0.5880730
 
       NA
 
           NA
  
 
  
 2
 
  20425246
 
  20425395
 
  ENSEMBL   UCSC 
 
  150
 
  6
 
 23211
 
 tss;island
 
 0.013029
 
 0.0043152
 
 Not queried
 
  0.5879519
 
       NA
 
           NA
  
 
  
 7
 
 107220040
 
 107220372
 
  ENSEMBL   UCSC 
 
  333
 
  7
 
 37827
 
 tss;island
 
 0.006575
 
 0.0056866
 
 Not queried
 
  0.5879420
 
       NA
 
           NA
  
 
  
 5
 
 125800764
 
 125800916
 
  ENSEMBL   UCSC 
 
  153
 
  4
 
 31734
 
 gene
 
 0.609695
 
 0.5823787
 
 Not queried
 
  0.5879299
 
       NA
 
           NA
  
 
  
 19
 
  42873746
 
  42873953
 
  ENSEMBL   UCSC 
 
  208
 
  4
 
 20070
 
 gene;island
 
 0.927543
 
 0.9567277
 
 Not queried
 
  0.5877274
 
       NA
 
           NA
  
 
  
 1
 
 155179117
 
 155179254
 
  ENSEMBL   UCSC 
 
  138
 
  5
 
 795
 
 gene;island
 
 0.010139
 
 0.0065106
 
 Not queried
 
  0.5876766
 
       NA
 
           NA
  
 
  
 15
 
  39872772
 
  39873304
 
  ENSEMBL   UCSC 
 
  533
 
 12
 
 14023
 
 island
 
 0.023689
 
 0.0365062
 
 Not queried
 
  0.5876072
 
       NA
 
           NA
  
 
  
 6
 
 109169227
 
 109169499
 
  ENSEMBL   UCSC 
 
  273
 
  7
 
 35161
 
 tss
 
 0.006055
 
 0.0100690
 
 Not queried
 
  0.5875470
 
       NA
 
           NA
  
 
  
 19
 
   7827560
 
   7827894
 
  ENSEMBL   UCSC 
 
  335
 
  4
 
 20406
 
 tss
 
 0.905057
 
 0.9379512
 
 Not queried
 
  0.5875400
 
       NA
 
           NA
  
 
  
 7
 
 131630226
 
 131630566
 
  ENSEMBL   UCSC 
 
  341
 
  5
 
 38930
 
 island
 
 0.009327
 
 0.0097702
 
 Not queried
 
  0.5874679
 
       NA
 
           NA
  
 
  
 19
 
  11849777
 
  11850013
 
  ENSEMBL   UCSC 
 
  237
 
  7
 
 20480
 
 tss
 
 0.005878
 
 0.0035122
 
 Not queried
 
  0.5874568
 
       NA
 
           NA
  
 
  
 1
 
 110949893
 
 110950539
 
  ENSEMBL   UCSC 
 
  647
 
  6
 
 678
 
 gene
 
 0.009147
 
 0.0090691
 
 Not queried
 
  0.5873316
 
       NA
 
           NA
  
 
  
 3
 
 192635966
 
 192636189
 
  ENSEMBL   UCSC 
 
  224
 
  7
 
 28793
 
 tss;island
 
 0.003327
 
 0.0077252
 
 Not queried
 
  0.5872139
 
       NA
 
           NA
  
 
  
 2
 
  47142867
 
  47143656
 
  ENSEMBL   UCSC 
 
  790
 
 12
 
 22670
 
 gene;island
 
 0.003385
 
 0.0026212
 
 Not queried
 
  0.5871778
 
       NA
 
           NA
  
 
  
 12
 
  30975749
 
  30976016
 
  ENSEMBL   UCSC 
 
  268
 
  4
 
 10309
 
 island
 
 0.007982
 
 0.0141487
 
 Not queried
 
  0.5871294
 
       NA
 
           NA
  
 
  
 20
 
  21494679
 
  21494977
 
  ENSEMBL   UCSC 
 
  299
 
  8
 
 25630
 
 tss
 
 0.067537
 
 0.1014314
 
 Not queried
 
  0.5871268
 
       NA
 
           NA
  
 
  
 20
 
  60719442
 
  60719824
 
  ENSEMBL   UCSC 
 
  383
 
  5
 
 25484
 
 gene;tss;island
 
 0.010273
 
 0.0183065
 
 Not queried
 
  0.5868999
 
       NA
 
           NA
  
 
  
 15
 
  84115811
 
  84116018
 
  ENSEMBL   UCSC 
 
  208
 
  6
 
 13866
 
 tss
 
 0.013041
 
 0.0225020
 
 Not queried
 
  0.5868268
 
       NA
 
           NA
  
 
  
 6
 
  31645405
 
  31645677
 
  ENSEMBL   UCSC 
 
  273
 
  4
 
 33936
 
 gene;island
 
 0.945891
 
 0.7605484
 
 Not queried
 
  0.5867982
 
       NA
 
           NA
  
 
  
 15
 
  63449340
 
  63449725
 
  ENSEMBL   UCSC 
 
  386
 
  5
 
 13373
 
 gene
 
 0.006644
 
 0.0083948
 
 Not queried
 
  0.5867431
 
       NA
 
           NA
  
 
  
 6
 
 114178192
 
 114178507
 
  ENSEMBL   UCSC 
 
  316
 
  9
 
 35185
 
 tss;island
 
 0.002326
 
 0.0035539
 
 Not queried
 
  0.5867210
 
       NA
 
           NA
  
 
  
 1
 
 172501940
 
 172502422
 
  ENSEMBL   UCSC 
 
  483
 
 10
 
 3968
 
 island
 
 0.008361
 
 0.0050714
 
 Not queried
 
  0.5866799
 
       NA
 
           NA
  
 
  
 6
 
  33133329
 
  33133675
 
  ENSEMBL   UCSC 
 
  347
 
  8
 
 35964
 
 island
 
 0.624488
 
 0.7448578
 
 Not queried
 
  0.5866465
 
       NA
 
           NA
  
 
  
 3
 
 179065417
 
 179065847
 
  ENSEMBL   UCSC 
 
  431
 
  8
 
 29679
 
 island
 
 0.004906
 
 0.0060936
 
 Not queried
 
  0.5865519
 
       NA
 
           NA
  
 
  
 22
 
  19512712
 
  19513176
 
  ENSEMBL   UCSC 
 
  465
 
  9
 
 27298
 
 island
 
 0.750328
 
 0.8374327
 
 Not queried
 
  0.5864309
 
       NA
 
           NA
  
 
  
 20
 
  43991346
 
  43991695
 
  ENSEMBL   UCSC 
 
  350
 
  6
 
 25773
 
 tss;island
 
 0.013468
 
 0.0105891
 
 Not queried
 
  0.5862204
 
       NA
 
           NA
  
 
  
 14
 
 102829091
 
 102829219
 
  ENSEMBL   UCSC 
 
  129
 
  5
 
 12231
 
 gene
 
 0.002139
 
 0.0010876
 
 Not queried
 
  0.5860784
 
       NA
 
           NA
  
 
  
 2
 
  98852704
 
  98852938
 
  ENSEMBL   UCSC 
 
  235
 
  5
 
 22799
 
 gene
 
 0.855562
 
 0.8990873
 
 Not queried
 
  0.5859925
 
       NA
 
           NA
  
 
  
 18
 
  54318324
 
  54318940
 
  ENSEMBL   UCSC 
 
  617
 
 10
 
 19530
 
 island
 
 0.004780
 
 0.0062224
 
 Not queried
 
  0.5858922
 
       NA
 
           NA
  
 
  
 11
 
   7272653
 
   7273154
 
  ENSEMBL   UCSC 
 
  502
 
  8
 
 7120
 
 tss;island
 
 0.055127
 
 0.2529641
 
 Not queried
 
  0.5858738
 
       NA
 
           NA
  
 
  
 17
 
  74381133
 
  74381280
 
  ENSEMBL   UCSC 
 
  148
 
  4
 
 17677
 
 tss
 
 0.040749
 
 0.0483239
 
 Not queried
 
  0.5856313
 
       NA
 
           NA
  
 
  
 19
 
  56904901
 
  56905152
 
  ENSEMBL   UCSC 
 
  252
 
  9
 
 21096
 
 tss
 
 0.045890
 
 0.0093059
 
 Not queried
 
  0.5855186
 
       NA
 
           NA
  
 
  
 1
 
 204120607
 
 204120951
 
  ENSEMBL   UCSC 
 
  345
 
  5
 
 956
 
 gene;island
 
 0.077338
 
 0.0512257
 
 Not queried
 
  0.5854118
 
       NA
 
           NA
  
 
  
 8
 
 143869138
 
 143869473
 
  ENSEMBL   UCSC 
 
  336
 
  5
 
 39940
 
 tss
 
 0.420602
 
 0.5390259
 
 Not queried
 
  0.5852875
 
       NA
 
           NA
  
 
  
 8
 
 146078724
 
 146079007
 
  ENSEMBL   UCSC 
 
  284
 
  5
 
 39994
 
 tss;island
 
 0.013460
 
 0.0062041
 
 Not queried
 
  0.5852250
 
       NA
 
           NA
  
 
  
 3
 
  11078979
 
  11079879
 
  ENSEMBL   UCSC 
 
  901
 
  8
 
 27666
 
 gene
 
 0.237834
 
 0.4909619
 
 Not queried
 
  0.5851512
 
       NA
 
           NA
  
 
  
 11
 
 115375341
 
 115375432
 
  ENSEMBL   UCSC 
 
   92
 
  4
 
 7709
 
 tss
 
 0.022536
 
 0.0390078
 
 Not queried
 
  0.5850961
 
       NA
 
           NA
  
 
  
 6
 
  26188969
 
  26189234
 
  ENSEMBL   UCSC 
 
  266
 
  4
 
 33604
 
 gene
 
 0.019989
 
 0.0163889
 
 Not queried
 
  0.5850499
 
       NA
 
           NA
  
 
  
 4
 
   6271485
 
   6271658
 
  ENSEMBL   UCSC 
 
  174
 
  6
 
 30887
 
 island
 
 0.006321
 
 0.0118467
 
 Not queried
 
  0.5850431
 
       NA
 
           NA
  
 
  
 1
 
 155197384
 
 155197843
 
  ENSEMBL   UCSC 
 
  460
 
  7
 
 1987
 
 tss
 
 0.008654
 
 0.0070165
 
 Not queried
 
  0.5850188
 
       NA
 
           NA
  
 
  
 14
 
  92588234
 
  92588364
 
  ENSEMBL   UCSC 
 
  131
 
  5
 
 12565
 
 tss;island
 
 0.018803
 
 0.0140286
 
 Not queried
 
  0.5850171
 
       NA
 
           NA
  
 
  
 10
 
 131505762
 
 131505869
 
  ENSEMBL   UCSC 
 
  108
 
  4
 
 4830
 
 gene
 
 0.505958
 
 0.6023263
 
 Not queried
 
  0.5848455
 
       NA
 
           NA
  
 
  
 12
 
 110841169
 
 110841667
 
  ENSEMBL   UCSC 
 
  499
 
 12
 
 10794
 
 island
 
 0.005475
 
 0.0034966
 
 Not queried
 
  0.5847543
 
       NA
 
           NA
  
 
  
 2
 
 210674029
 
 210674232
 
  ENSEMBL   UCSC 
 
  204
 
  4
 
 23002
 
 gene;island
 
 0.866578
 
 0.9101179
 
 Not queried
 
  0.5845954
 
       NA
 
           NA
  
 
  
 7
 
  26191458
 
  26191726
 
  ENSEMBL   UCSC 
 
  269
 
  6
 
 37481
 
 tss;island
 
 0.032885
 
 0.0215494
 
 Not queried
 
  0.5845871
 
       NA
 
           NA
  
 
  
 10
 
  52499747
 
  52500187
 
  ENSEMBL   UCSC 
 
  441
 
  5
 
 4588
 
 gene
 
 0.005319
 
 0.0026508
 
 Not queried
 
  0.5845182
 
       NA
 
           NA
  
 
  
 10
 
  52499393
 
  52500187
 
  ENSEMBL   UCSC 
 
  795
 
  9
 
 5745
 
 island
 
 0.005319
 
 0.0027800
 
 Not queried
 
  0.5845182
 
       NA
 
           NA
  
 
  
 12
 
 114029248
 
 114029659
 
  ENSEMBL   UCSC 
 
  412
 
  5
 
 10836
 
 island
 
 0.240080
 
 0.0774689
 
 Not queried
 
  0.5844884
 
       NA
 
           NA
  
 
  
 2
 
  26101343
 
  26101831
 
  ENSEMBL   UCSC 
 
  489
 
  8
 
 23235
 
 tss;island
 
 0.009353
 
 0.0096814
 
 Not queried
 
  0.5843651
 
       NA
 
           NA
  
 
  
 2
 
  86790614
 
  86790844
 
  ENSEMBL   UCSC 
 
  231
 
  6
 
 23495
 
 tss
 
 0.012696
 
 0.0085283
 
 Not queried
 
  0.5842714
 
       NA
 
           NA
  
 
  
 18
 
  60382519
 
  60382673
 
  ENSEMBL   UCSC 
 
  155
 
  7
 
 19305
 
 tss;island
 
 0.009344
 
 0.0069337
 
 Not queried
 
  0.5842169
 
       NA
 
           NA
  
 
  
 6
 
 108145539
 
 108145930
 
  ENSEMBL   UCSC 
 
  392
 
  6
 
 35155
 
 tss
 
 0.021728
 
 0.0422491
 
 Not queried
 
  0.5840155
 
       NA
 
           NA
  
 
  
 2
 
 220379635
 
 220379968
 
  ENSEMBL   UCSC 
 
  334
 
  4
 
 23037
 
 gene;island
 
 0.714320
 
 0.7868247
 
 Not queried
 
  0.5839914
 
       NA
 
           NA
  
 
  
 19
 
   5334979
 
   5335260
 
  ENSEMBL   UCSC 
 
  282
 
  5
 
 19750
 
 gene;island
 
 0.962931
 
 0.9387784
 
 Not queried
 
  0.5839697
 
       NA
 
           NA
  
 
  
 8
 
   2242024
 
   2242304
 
  ENSEMBL   UCSC 
 
  281
 
  4
 
 40050
 
 island
 
 0.580768
 
 0.7053519
 
 Not queried
 
  0.5837932
 
       NA
 
           NA
  
 
  
 4
 
 140216957
 
 140217098
 
  ENSEMBL   UCSC 
 
  142
 
  5
 
 30598
 
 tss
 
 0.014925
 
 0.0174784
 
 Not queried
 
  0.5837928
 
       NA
 
           NA
  
 
  
 4
 
 157996959
 
 157997526
 
  ENSEMBL   UCSC 
 
  568
 
 12
 
 30653
 
 tss
 
 0.036797
 
 0.0530382
 
 Not queried
 
  0.5837176
 
       NA
 
           NA
  
 
  
 10
 
  75173848
 
  75174282
 
  ENSEMBL   UCSC 
 
  435
 
  9
 
 5159
 
 tss
 
 0.011365
 
 0.0052196
 
 Not queried
 
  0.5834152
 
       NA
 
           NA
  
 
  
 10
 
 105110944
 
 105111158
 
  ENSEMBL   UCSC 
 
  215
 
  8
 
 5335
 
 tss
 
 0.016404
 
 0.0105420
 
 Not queried
 
  0.5834005
 
       NA
 
           NA
  
 
  
 1
 
 145039638
 
 145039929
 
  ENSEMBL   UCSC 
 
  292
 
  5
 
 721
 
 gene;island
 
 0.051256
 
 0.0522942
 
 Not queried
 
  0.5832263
 
       NA
 
           NA
  
 
  
 1
 
 154947991
 
 154948305
 
  ENSEMBL   UCSC 
 
  315
 
  4
 
 786
 
 gene;tss;island
 
 0.003551
 
 0.0037008
 
 Not queried
 
  0.5831652
 
       NA
 
           NA
  
 
  
 19
 
  10221818
 
  10222175
 
  ENSEMBL   UCSC 
 
  358
 
  4
 
 19809
 
 gene;tss;island
 
 0.443459
 
 0.4930970
 
 Not queried
 
  0.5831334
 
       NA
 
           NA
  
 
  
 11
 
 123066108
 
 123066252
 
  ENSEMBL   UCSC 
 
  145
 
  4
 
 7777
 
 tss;island
 
 0.016550
 
 0.0170063
 
 Not queried
 
  0.5831284
 
       NA
 
           NA
  
 
  
 4
 
 108745557
 
 108745771
 
  ENSEMBL   UCSC 
 
  215
 
  4
 
 31226
 
 island
 
 0.053322
 
 0.0372575
 
 Not queried
 
  0.5829866
 
       NA
 
           NA
  
 
  
 7
 
  90032535
 
  90032717
 
  ENSEMBL   UCSC 
 
  183
 
  5
 
 37699
 
 tss
 
 0.004035
 
 0.0035527
 
 Not queried
 
  0.5829438
 
       NA
 
           NA
  
 
  
 11
 
 117747689
 
 117748132
 
  ENSEMBL   UCSC 
 
  444
 
  6
 
 6923
 
 gene
 
 0.439846
 
 0.4579305
 
 Not queried
 
  0.5828716
 
       NA
 
           NA
  
 
  
 11
 
 117747689
 
 117748236
 
  ENSEMBL   UCSC 
 
  548
 
 10
 
 8852
 
 island
 
 0.473965
 
 0.7064411
 
 Not queried
 
  0.5828716
 
       NA
 
           NA
  
 
  
 7
 
  45961772
 
  45962236
 
  ENSEMBL   UCSC 
 
  465
 
 13
 
 37588
 
 tss;island
 
 0.009976
 
 0.0282485
 
 Not queried
 
  0.5828515
 
       NA
 
           NA
  
 
  
 14
 
  39572202
 
  39572780
 
  ENSEMBL   UCSC 
 
  579
 
  9
 
 12803
 
 island
 
 0.014337
 
 0.0079367
 
 Not queried
 
  0.5827891
 
       NA
 
           NA
  
 
  
 22
 
  32870857
 
  32871215
 
  ENSEMBL   UCSC 
 
  359
 
  6
 
 27093
 
 tss
 
 0.003733
 
 0.0048417
 
 Not queried
 
  0.5827694
 
       NA
 
           NA
  
 
  
 17
 
  17105857
 
  17106312
 
  ENSEMBL   UCSC 
 
  456
 
  7
 
 16461
 
 gene;island
 
 0.985540
 
 0.9681607
 
 Not queried
 
  0.5826708
 
       NA
 
           NA
  
 
  
 7
 
  12443478
 
  12443704
 
  ENSEMBL   UCSC 
 
  227
 
  4
 
 36895
 
 gene
 
 0.017481
 
 0.0127172
 
 Not queried
 
  0.5826451
 
       NA
 
           NA
  
 
  
 7
 
 100076964
 
 100077394
 
  ENSEMBL   UCSC 
 
  431
 
  5
 
 37783
 
 tss;island
 
 0.024980
 
 0.0333478
 
 Not queried
 
  0.5825424
 
       NA
 
           NA
  
 
  
 1
 
  43312353
 
  43312644
 
  ENSEMBL   UCSC 
 
  292
 
  4
 
 469
 
 gene
 
 0.006117
 
 0.0088428
 
 Not queried
 
  0.5824862
 
       NA
 
           NA
  
 
  
 4
 
 141489963
 
 141490428
 
  ENSEMBL   UCSC 
 
  466
 
 10
 
 30608
 
 tss
 
 0.011606
 
 0.0100333
 
 Not queried
 
  0.5824756
 
       NA
 
           NA
  
 
  
 15
 
  77577491
 
  77577655
 
  ENSEMBL   UCSC 
 
  165
 
  4
 
 13825
 
 tss
 
 0.979129
 
 0.9681955
 
 Not queried
 
  0.5823875
 
       NA
 
           NA
  
 
  
 12
 
 115124584
 
 115125098
 
  ENSEMBL   UCSC 
 
  515
 
  5
 
 10848
 
 island
 
 0.078554
 
 0.1247850
 
 Not queried
 
  0.5823808
 
       NA
 
           NA
  
 
  
 5
 
  31193340
 
  31194800
 
  ENSEMBL   UCSC 
 
 1461
 
 13
 
 32713
 
 island
 
 0.020435
 
 0.0199470
 
 Not queried
 
  0.5822964
 
       NA
 
           NA
  
 
  
 12
 
  24715864
 
  24716204
 
  ENSEMBL   UCSC 
 
  341
 
  4
 
 9606
 
 tss;island
 
 0.014137
 
 0.0211599
 
 Not queried
 
  0.5822833
 
       NA
 
           NA
  
 
  
 7
 
  72847862
 
  72848049
 
  ENSEMBL   UCSC 
 
  188
 
  4
 
 37633
 
 tss;island
 
 0.017057
 
 0.0098513
 
 Not queried
 
  0.5821196
 
       NA
 
           NA
  
 
  
 6
 
 159421281
 
 159421528
 
  ENSEMBL   UCSC 
 
  248
 
  6
 
 35318
 
 tss;island
 
 0.028591
 
 0.0124169
 
 Not queried
 
  0.5820893
 
       NA
 
           NA
  
 
  
 19
 
  59067160
 
  59067560
 
  ENSEMBL   UCSC 
 
  401
 
 10
 
 20264
 
 gene;tss;island
 
 0.849174
 
 0.9210523
 
 Not queried
 
  0.5820543
 
       NA
 
           NA
  
 
  
 17
 
  19290503
 
  19290762
 
  ENSEMBL   UCSC 
 
  260
 
  6
 
 17190
 
 tss
 
 0.047747
 
 0.0815038
 
 Not queried
 
  0.5820394
 
       NA
 
           NA
  
 
  
 19
 
  54663461
 
  54664028
 
  ENSEMBL   UCSC 
 
  568
 
  9
 
 21054
 
 tss
 
 0.005852
 
 0.0096282
 
 Not queried
 
  0.5819523
 
       NA
 
           NA
  
 
  
 7
 
  18535072
 
  18535499
 
  ENSEMBL   UCSC 
 
  428
 
  6
 
 37458
 
 tss
 
 0.003471
 
 0.0076764
 
 Not queried
 
  0.5819472
 
       NA
 
           NA
  
 
  
 3
 
 184870685
 
 184871070
 
  ENSEMBL   UCSC 
 
  386
 
  8
 
 29736
 
 island
 
 0.011481
 
 0.0247025
 
 Not queried
 
  0.5818159
 
       NA
 
           NA
  
 
  
 1
 
   6845123
 
   6845484
 
  ENSEMBL   UCSC 
 
  362
 
 12
 
 2620
 
 island
 
 0.010955
 
 0.0069819
 
 Not queried
 
  0.5818028
 
       NA
 
           NA
  
 
  
 12
 
  54426498
 
  54426772
 
  ENSEMBL   UCSC 
 
  275
 
  8
 
 9755
 
 tss
 
 0.005761
 
 0.0110383
 
 Not queried
 
  0.5817771
 
       NA
 
           NA
  
 
  
 14
 
  58893672
 
  58893942
 
  ENSEMBL   UCSC 
 
  271
 
  4
 
 12097
 
 gene;tss;island
 
 0.001960
 
 0.0017482
 
 Not queried
 
  0.5816688
 
       NA
 
           NA
  
 
  
 15
 
  49462118
 
  49462405
 
  ENSEMBL   UCSC 
 
  288
 
  7
 
 13663
 
 tss
 
 0.028268
 
 0.0420495
 
 Not queried
 
  0.5816593
 
       NA
 
           NA
  
 
  
 13
 
  26828738
 
  26828880
 
  ENSEMBL   UCSC 
 
  143
 
  4
 
 11620
 
 island
 
 0.002077
 
 0.0032875
 
 Not queried
 
  0.5816095
 
       NA
 
           NA
  
 
  
 3
 
 127770926
 
 127771091
 
  ENSEMBL   UCSC 
 
  166
 
  5
 
 28566
 
 tss;island
 
 0.004604
 
 0.0049299
 
 Not queried
 
  0.5815428
 
       NA
 
           NA
  
 
  
 6
 
   8086756
 
   8086870
 
  ENSEMBL   UCSC 
 
  115
 
  4
 
 33555
 
 gene
 
 0.987674
 
 0.9899142
 
 Not queried
 
  0.5815262
 
       NA
 
           NA
  
 
  
 15
 
  63334355
 
  63334761
 
  ENSEMBL   UCSC 
 
  407
 
  8
 
 13717
 
 tss
 
 0.024145
 
 0.0243820
 
 Not queried
 
  0.5813787
 
       NA
 
           NA
  
 
  
 15
 
  63334355
 
  63335016
 
  ENSEMBL   UCSC 
 
  662
 
 10
 
 14201
 
 island
 
 0.024145
 
 0.0243820
 
 Not queried
 
  0.5813787
 
       NA
 
           NA
  
 
  
 11
 
 123172362
 
 123172864
 
  ENSEMBL   UCSC 
 
  503
 
  5
 
 8907
 
 island
 
 0.014245
 
 0.0082866
 
 Not queried
 
  0.5812293
 
       NA
 
           NA
  
 
  
 10
 
 114135847
 
 114136073
 
  ENSEMBL   UCSC 
 
  227
 
  6
 
 4762
 
 gene
 
 0.009038
 
 0.0107553
 
 Not queried
 
  0.5811921
 
       NA
 
           NA
  
 
  
 3
 
  42632375
 
  42632622
 
  ENSEMBL   UCSC 
 
  248
 
  4
 
 27733
 
 gene
 
 0.012670
 
 0.0123893
 
 Not queried
 
  0.5811620
 
       NA
 
           NA
  
 
  
 16
 
  31724470
 
  31724534
 
  ENSEMBL   UCSC 
 
   65
 
  4
 
 15216
 
 tss
 
 0.014133
 
 0.0066768
 
 Not queried
 
  0.5810607
 
       NA
 
           NA
  
 
  
 19
 
  12807371
 
  12807673
 
  ENSEMBL   UCSC 
 
  303
 
 10
 
 21614
 
 island
 
 0.008461
 
 0.0076441
 
 Not queried
 
  0.5809695
 
       NA
 
           NA
  
 
  
 3
 
  44596178
 
  44596770
 
  ENSEMBL   UCSC 
 
  593
 
  9
 
 29035
 
 island
 
 0.857327
 
 0.2325455
 
 Not queried
 
  0.5809300
 
       NA
 
           NA
  
 
  
 16
 
  82661421
 
  82661725
 
  ENSEMBL   UCSC 
 
  305
 
  4
 
 14859
 
 gene;island
 
 0.093786
 
 0.1691872
 
 Not queried
 
  0.5808010
 
       NA
 
           NA
  
 
  
 1
 
 230203041
 
 230203164
 
  ENSEMBL   UCSC 
 
  124
 
  5
 
 1068
 
 gene;island
 
 0.008223
 
 0.0150162
 
 Not queried
 
  0.5805351
 
       NA
 
           NA
  
 
  
 1
 
 221051758
 
 221052143
 
  ENSEMBL   UCSC 
 
  386
 
  4
 
 2263
 
 tss;island
 
 0.027624
 
 0.0382098
 
 Not queried
 
  0.5804691
 
       NA
 
           NA
  
 
  
 18
 
  47088234
 
  47088432
 
  ENSEMBL   UCSC 
 
  199
 
  8
 
 19506
 
 island
 
 0.057068
 
 0.0356293
 
 Not queried
 
  0.5803288
 
       NA
 
           NA
  
 
  
 8
 
  54164296
 
  54164442
 
  ENSEMBL   UCSC 
 
  147
 
  5
 
 39701
 
 tss
 
 0.058973
 
 0.0309037
 
 Not queried
 
  0.5803052
 
       NA
 
           NA
  
 
  
 1
 
  87793364
 
  87793607
 
  ENSEMBL   UCSC 
 
  244
 
  4
 
 1733
 
 tss;island
 
 0.665043
 
 0.7569171
 
 Not queried
 
  0.5802518
 
       NA
 
           NA
  
 
  
 20
 
  16553772
 
  16554047
 
  ENSEMBL   UCSC 
 
  276
 
  5
 
 25363
 
 gene
 
 0.003459
 
 0.0030235
 
 Not queried
 
  0.5802400
 
       NA
 
           NA
  
 
  
 2
 
    662602
 
    663024
 
  ENSEMBL   UCSC 
 
  423
 
  4
 
 24010
 
 island
 
 0.366477
 
 0.0962803
 
 Not queried
 
  0.5802371
 
       NA
 
           NA
  
 
  
 19
 
  13135514
 
  13135808
 
  ENSEMBL   UCSC 
 
  295
 
  4
 
 19877
 
 gene;island
 
 0.377429
 
 0.2757919
 
 Not queried
 
  0.5802220
 
       NA
 
           NA
  
 
  
 20
 
    623089
 
    623421
 
  ENSEMBL   UCSC 
 
  333
 
  4
 
 25926
 
 island
 
 0.031357
 
 0.0776917
 
 Not queried
 
  0.5801898
 
       NA
 
           NA
  
 
  
 8
 
  59465767
 
  59465971
 
  ENSEMBL   UCSC 
 
  205
 
  4
 
 39316
 
 gene
 
 0.014176
 
 0.0086998
 
 Not queried
 
  0.5801067
 
       NA
 
           NA
  
 
  
 14
 
 104583048
 
 104583245
 
  ENSEMBL   UCSC 
 
  198
 
  4
 
 12657
 
 tss;island
 
 0.053382
 
 0.0373208
 
 Not queried
 
  0.5800967
 
       NA
 
           NA
  
 
  
 6
 
  74363775
 
  74363913
 
  ENSEMBL   UCSC 
 
  139
 
  6
 
 35073
 
 tss
 
 0.014284
 
 0.0103630
 
 Not queried
 
  0.5800598
 
       NA
 
           NA
  
 
  
 2
 
  95537044
 
  95537475
 
  ENSEMBL   UCSC 
 
  432
 
  8
 
 24510
 
 island
 
 0.366444
 
 0.5342165
 
 Not queried
 
  0.5800231
 
       NA
 
           NA
  
 
  
 6
 
  32028831
 
  32029216
 
  ENSEMBL   UCSC 
 
  386
 
  8
 
 34025
 
 gene
 
 0.295474
 
 0.4262744
 
 Not queried
 
  0.5798757
 
       NA
 
           NA
  
 
  
 6
 
  13574034
 
  13574371
 
  ENSEMBL   UCSC 
 
  338
 
  5
 
 34644
 
 tss;island
 
 0.014915
 
 0.0057017
 
 Not queried
 
  0.5798653
 
       NA
 
           NA
  
 
  
 10
 
 134188633
 
 134188873
 
  ENSEMBL   UCSC 
 
  241
 
  5
 
 4863
 
 gene
 
 0.918119
 
 0.9431338
 
 Not queried
 
  0.5797941
 
       NA
 
           NA
  
 
  
 19
 
  39217605
 
  39218041
 
  ENSEMBL   UCSC 
 
  437
 
  4
 
 20019
 
 gene;island
 
 0.958169
 
 0.9583008
 
 Not queried
 
  0.5797940
 
       NA
 
           NA
  
 
  
 14
 
  65171673
 
  65172195
 
  ENSEMBL   UCSC 
 
  523
 
  5
 
 12112
 
 gene;island
 
 0.028468
 
 0.0192107
 
 Not queried
 
  0.5797697
 
       NA
 
           NA
  
 
  
 13
 
  53422691
 
  53423262
 
  ENSEMBL   UCSC 
 
  572
 
 11
 
 11762
 
 island
 
 0.073831
 
 0.0506119
 
 Not queried
 
  0.5797489
 
       NA
 
           NA
  
 
  
 22
 
  42228699
 
  42229068
 
  ENSEMBL   UCSC 
 
  370
 
  9
 
 27181
 
 tss
 
 0.004942
 
 0.0086247
 
 Not queried
 
  0.5796846
 
       NA
 
           NA
  
 
  
 22
 
  42228699
 
  42229324
 
  ENSEMBL   UCSC 
 
  626
 
 11
 
 27531
 
 island
 
 0.004942
 
 0.0086247
 
 Not queried
 
  0.5796846
 
       NA
 
           NA
  
 
  
 6
 
  28493019
 
  28493664
 
  ENSEMBL   UCSC 
 
  646
 
  9
 
 34743
 
 tss
 
 0.017015
 
 0.2531556
 
 Not queried
 
  0.5796613
 
       NA
 
           NA
  
 
  
 19
 
  34111400
 
  34111733
 
  ENSEMBL   UCSC 
 
  334
 
  5
 
 20686
 
 tss;island
 
 0.313920
 
 0.4355722
 
 Not queried
 
  0.5796170
 
       NA
 
           NA
  
 
  
 1
 
 183441292
 
 183441464
 
  ENSEMBL   UCSC 
 
  173
 
  4
 
 2135
 
 tss;island
 
 0.013093
 
 0.0116977
 
 Not queried
 
  0.5794956
 
       NA
 
           NA
  
 
  
 8
 
  33330294
 
  33330940
 
  ENSEMBL   UCSC 
 
  647
 
 13
 
 40210
 
 island
 
 0.010740
 
 0.0069665
 
 Not queried
 
  0.5794828
 
       NA
 
           NA
  
 
  
 8
 
  24857432
 
  24857894
 
  ENSEMBL   UCSC 
 
  463
 
  5
 
 40163
 
 island
 
 0.016328
 
 0.0544818
 
 Not queried
 
  0.5794039
 
       NA
 
           NA
  
 
  
 19
 
  57831544
 
  57831909
 
  ENSEMBL   UCSC 
 
  366
 
 12
 
 22465
 
 island
 
 0.006918
 
 0.0088128
 
 Not queried
 
  0.5793636
 
       NA
 
           NA
  
 
  
 12
 
 111358446
 
 111358639
 
  ENSEMBL   UCSC 
 
  194
 
  7
 
 10012
 
 tss
 
 0.763085
 
 0.6498704
 
 Not queried
 
  0.5793599
 
       NA
 
           NA
  
 
  
 7
 
  30492035
 
  30492171
 
  ENSEMBL   UCSC 
 
  137
 
  4
 
 36954
 
 gene
 
 0.899872
 
 0.9407221
 
 Not queried
 
  0.5793066
 
       NA
 
           NA
  
 
  
 18
 
  34340585
 
  34340941
 
  ENSEMBL   UCSC 
 
  357
 
  4
 
 19092
 
 gene;island
 
 0.874775
 
 0.8689692
 
 Not queried
 
  0.5793006
 
       NA
 
           NA
  
 
  
 11
 
  70963994
 
  70964256
 
  ENSEMBL   UCSC 
 
  263
 
  4
 
 8617
 
 island
 
 0.027950
 
 0.1210608
 
 Not queried
 
  0.5792479
 
       NA
 
           NA
  
 
  
 5
 
 175788872
 
 175789238
 
  ENSEMBL   UCSC 
 
  367
 
  8
 
 32509
 
 tss
 
 0.008502
 
 0.0108289
 
 Not queried
 
  0.5791150
 
       NA
 
           NA
  
 
  
 1
 
 207925081
 
 207925400
 
  ENSEMBL   UCSC 
 
  320
 
  6
 
 2225
 
 tss
 
 0.005370
 
 0.0042874
 
 Not queried
 
  0.5790841
 
       NA
 
           NA
  
 
  
 1
 
  46632363
 
  46632871
 
  ENSEMBL   UCSC 
 
  509
 
  7
 
 3213
 
 island
 
 0.024420
 
 0.0215495
 
 Not queried
 
  0.5790243
 
       NA
 
           NA
  
 
  
 11
 
 110583377
 
 110583662
 
  ENSEMBL   UCSC 
 
  286
 
  7
 
 8794
 
 island
 
 0.052116
 
 0.0386392
 
 Not queried
 
  0.5790097
 
       NA
 
           NA
  
 
  
 16
 
   3085571
 
   3085982
 
  ENSEMBL   UCSC 
 
  412
 
  4
 
 15048
 
 tss
 
 0.016920
 
 0.1343856
 
 Not queried
 
  0.5789781
 
       NA
 
           NA
  
 
  
 1
 
  52870280
 
  52870655
 
  ENSEMBL   UCSC 
 
  376
 
  6
 
 533
 
 gene
 
 0.012499
 
 0.0185586
 
 Not queried
 
  0.5789345
 
       NA
 
           NA
  
 
  
 18
 
  30352782
 
  30353283
 
  ENSEMBL   UCSC 
 
  502
 
  8
 
 19454
 
 island
 
 0.004905
 
 0.0029872
 
 Not queried
 
  0.5787899
 
       NA
 
           NA
  
 
  
 12
 
   4140624
 
   4141193
 
  ENSEMBL   UCSC 
 
  570
 
  6
 
 10168
 
 island
 
 0.284642
 
 0.2152122
 
 Not queried
 
  0.5787221
 
       NA
 
           NA
  
 
  
 20
 
  18039663
 
  18040012
 
  ENSEMBL   UCSC 
 
  350
 
  5
 
 25612
 
 tss;island
 
 0.055798
 
 0.0780317
 
 Not queried
 
  0.5787160
 
       NA
 
           NA
  
 
  
 1
 
  15852955
 
  15853278
 
  ENSEMBL   UCSC 
 
  324
 
  9
 
 1273
 
 tss;island
 
 0.002535
 
 0.0059811
 
 Not queried
 
  0.5786879
 
       NA
 
           NA
  
 
  
 15
 
  90792609
 
  90793056
 
  ENSEMBL   UCSC 
 
  448
 
  7
 
 14450
 
 island
 
 0.026881
 
 0.0323448
 
 Not queried
 
  0.5786177
 
       NA
 
           NA
  
 
  
 12
 
 121177142
 
 121177413
 
  ENSEMBL   UCSC 
 
  272
 
  5
 
 9360
 
 gene;island
 
 0.867030
 
 0.8915534
 
 Not queried
 
  0.5785988
 
       NA
 
           NA
  
 
  
 11
 
  60623415
 
  60623918
 
  ENSEMBL   UCSC 
 
  504
 
  7
 
 8314
 
 island
 
 0.126133
 
 0.0658166
 
 Not queried
 
  0.5784572
 
       NA
 
           NA
  
 
  
 17
 
  76989451
 
  76989965
 
  ENSEMBL   UCSC 
 
  515
 
  6
 
 16831
 
 gene;island
 
 0.937845
 
 0.8535303
 
 Not queried
 
  0.5783770
 
       NA
 
           NA
  
 
  
 16
 
  68481049
 
  68481543
 
  ENSEMBL   UCSC 
 
  495
 
  6
 
 14815
 
 gene;island
 
 0.584117
 
 0.4880807
 
 Not queried
 
  0.5783361
 
       NA
 
           NA
  
 
  
 21
 
  46056847
 
  46057082
 
  ENSEMBL   UCSC 
 
  236
 
  4
 
 26458
 
 gene;tss
 
 0.286758
 
 0.3434943
 
 Not queried
 
  0.5782797
 
       NA
 
           NA
  
 
  
 10
 
  50969997
 
  50970358
 
  ENSEMBL   UCSC 
 
  362
 
  5
 
 4586
 
 gene
 
 0.093009
 
 0.1303325
 
 Not queried
 
  0.5781283
 
       NA
 
           NA
  
 
  
 2
 
   1426068
 
   1426328
 
  ENSEMBL   UCSC 
 
  261
 
  5
 
 22530
 
 gene
 
 0.469186
 
 0.7943808
 
 Not queried
 
  0.5781175
 
       NA
 
           NA
  
 
  
 10
 
  64564161
 
  64564556
 
  ENSEMBL   UCSC 
 
  396
 
  8
 
 5773
 
 island
 
 0.006234
 
 0.0063060
 
 Not queried
 
  0.5780197
 
       NA
 
           NA
  
 
  
 18
 
  77125738
 
  77125858
 
  ENSEMBL   UCSC 
 
  121
 
  4
 
 19135
 
 gene;island
 
 0.974498
 
 0.9559381
 
 Not queried
 
  0.5779507
 
       NA
 
           NA
  
 
  
 17
 
  59494040
 
  59494444
 
  ENSEMBL   UCSC 
 
  405
 
  4
 
 18630
 
 island
 
 0.162810
 
 0.1737838
 
 Not queried
 
  0.5779403
 
       NA
 
           NA
  
 
  
 5
 
 158533621
 
 158533942
 
  ENSEMBL   UCSC 
 
  322
 
  5
 
 33352
 
 island
 
 0.021122
 
 0.0085577
 
 Not queried
 
  0.5778163
 
       NA
 
           NA
  
 
  
 13
 
 111957387
 
 111957848
 
  ENSEMBL   UCSC 
 
  462
 
  4
 
 11244
 
 gene
 
 0.985772
 
 0.9741239
 
 Not queried
 
  0.5777912
 
       NA
 
           NA
  
 
  
 22
 
  38577768
 
  38578138
 
  ENSEMBL   UCSC 
 
  371
 
  9
 
 27132
 
 tss
 
 0.004106
 
 0.0032426
 
 Not queried
 
  0.5777589
 
       NA
 
           NA
  
 
  
 22
 
  38577578
 
  38578138
 
  ENSEMBL   UCSC 
 
  561
 
 11
 
 27481
 
 island
 
 0.004106
 
 0.0032426
 
 Not queried
 
  0.5777589
 
       NA
 
           NA
  
 
  
 16
 
  87894334
 
  87894690
 
  ENSEMBL   UCSC 
 
  357
 
  4
 
 14893
 
 gene;island
 
 0.591591
 
 0.7333246
 
 Not queried
 
  0.5776529
 
       NA
 
           NA
  
 
  
 8
 
  99956467
 
  99956585
 
  ENSEMBL   UCSC 
 
  119
 
  5
 
 39837
 
 tss
 
 0.013138
 
 0.0067530
 
 Not queried
 
  0.5775768
 
       NA
 
           NA
  
 
  
 6
 
  33170692
 
  33170838
 
  ENSEMBL   UCSC 
 
  147
 
  6
 
 34224
 
 gene;island
 
 0.972038
 
 0.9687973
 
 Not queried
 
  0.5775323
 
       NA
 
           NA
  
 
  
 14
 
  89020645
 
  89021477
 
  ENSEMBL   UCSC 
 
  833
 
 11
 
 13059
 
 island
 
 0.011412
 
 0.0114779
 
 Not queried
 
  0.5775170
 
       NA
 
           NA
  
 
  
 19
 
   1449857
 
   1450023
 
  ENSEMBL   UCSC 
 
  167
 
  5
 
 20306
 
 tss;island
 
 0.028015
 
 0.0131111
 
 Not queried
 
  0.5773114
 
       NA
 
           NA
  
 
  
 6
 
  49431117
 
  49431433
 
  ENSEMBL   UCSC 
 
  317
 
  6
 
 34362
 
 gene
 
 0.009595
 
 0.0063233
 
 Not queried
 
  0.5772504
 
       NA
 
           NA
  
 
  
 19
 
   5340582
 
   5340832
 
  ENSEMBL   UCSC 
 
  251
 
  4
 
 21403
 
 island
 
 0.011463
 
 0.0197614
 
 Not queried
 
  0.5771523
 
       NA
 
           NA
  
 
  
 7
 
 100612725
 
 100612937
 
  ENSEMBL   UCSC 
 
  213
 
  5
 
 38786
 
 island
 
 0.311243
 
 0.4594080
 
 Not queried
 
  0.5769313
 
       NA
 
           NA
  
 
  
 11
 
  63885584
 
  63885769
 
  ENSEMBL   UCSC 
 
  186
 
  4
 
 6664
 
 gene;island
 
 0.870028
 
 0.8877452
 
 Not queried
 
  0.5769310
 
       NA
 
           NA
  
 
  
 2
 
  56150255
 
  56150675
 
  ENSEMBL   UCSC 
 
  421
 
  6
 
 22691
 
 gene;island
 
 0.205021
 
 0.2364469
 
 Not queried
 
  0.5767127
 
       NA
 
           NA
  
 
  
 19
 
  14606622
 
  14607052
 
  ENSEMBL   UCSC 
 
  431
 
  7
 
 21685
 
 island
 
 0.004975
 
 0.0051122
 
 Not queried
 
  0.5766918
 
       NA
 
           NA
  
 
  
 13
 
  21678461
 
  21678647
 
  ENSEMBL   UCSC 
 
  187
 
  4
 
 11584
 
 island
 
 0.977894
 
 0.9785430
 
 Not queried
 
  0.5766782
 
       NA
 
           NA
  
 
  
 8
 
 143763326
 
 143763565
 
  ENSEMBL   UCSC 
 
  240
 
  4
 
 39466
 
 gene
 
 0.638943
 
 0.7194317
 
 Not queried
 
  0.5766596
 
       NA
 
           NA
  
 
  
 12
 
 122985348
 
 122985803
 
  ENSEMBL   UCSC 
 
  456
 
  7
 
 10930
 
 island
 
 0.008968
 
 0.0083309
 
 Not queried
 
  0.5765278
 
       NA
 
           NA
  
 
  
 7
 
 152373310
 
 152373553
 
  ENSEMBL   UCSC 
 
  244
 
  6
 
 38006
 
 tss
 
 0.005819
 
 0.0039462
 
 Not queried
 
  0.5765259
 
       NA
 
           NA
  
 
  
 7
 
  94537716
 
  94537893
 
  ENSEMBL   UCSC 
 
  178
 
  8
 
 37723
 
 tss
 
 0.022627
 
 0.0334385
 
 Not queried
 
  0.5764792
 
       NA
 
           NA
  
 
  
 19
 
  11924686
 
  11925094
 
  ENSEMBL   UCSC 
 
  409
 
  8
 
 20483
 
 tss
 
 0.013627
 
 0.0147064
 
 Not queried
 
  0.5763573
 
       NA
 
           NA
  
 
  
 11
 
   1974132
 
   1974332
 
  ENSEMBL   UCSC 
 
  201
 
  4
 
 6440
 
 gene;island
 
 0.945462
 
 0.9638329
 
 Not queried
 
  0.5762965
 
       NA
 
           NA
  
 
  
 3
 
  36422059
 
  36422406
 
  ENSEMBL   UCSC 
 
  348
 
  7
 
 28978
 
 island
 
 0.012929
 
 0.0191173
 
 Not queried
 
  0.5762694
 
       NA
 
           NA
  
 
  
 6
 
  30584596
 
  30584980
 
  ENSEMBL   UCSC 
 
  385
 
  8
 
 33786
 
 gene
 
 0.007051
 
 0.0055955
 
 Not queried
 
  0.5762599
 
       NA
 
           NA
  
 
  
 11
 
 120081497
 
 120081645
 
  ENSEMBL   UCSC 
 
  149
 
  4
 
 7760
 
 tss
 
 0.012829
 
 0.0093894
 
 Not queried
 
  0.5762462
 
       NA
 
           NA
  
 
  
 5
 
 137911500
 
 137911699
 
  ENSEMBL   UCSC 
 
  200
 
  4
 
 32325
 
 tss;island
 
 0.005888
 
 0.0079217
 
 Not queried
 
  0.5761922
 
       NA
 
           NA
  
 
  
 16
 
  50775288
 
  50776026
 
  ENSEMBL   UCSC 
 
  739
 
 11
 
 15230
 
 tss
 
 0.002830
 
 0.0077182
 
 Not queried
 
  0.5760855
 
       NA
 
           NA
  
 
  
 2
 
  97426158
 
  97426627
 
  ENSEMBL   UCSC 
 
  470
 
  9
 
 23523
 
 tss;island
 
 0.010499
 
 0.0096215
 
 Not queried
 
  0.5760683
 
       NA
 
           NA
  
 
  
 1
 
 236017128
 
 236017325
 
  ENSEMBL   UCSC 
 
  198
 
  5
 
 1094
 
 gene;tss
 
 0.976522
 
 0.9633903
 
 Not queried
 
  0.5760641
 
       NA
 
           NA
  
 
  
 2
 
  63278026
 
  63278211
 
  ENSEMBL   UCSC 
 
  186
 
  4
 
 22703
 
 gene
 
 0.003740
 
 0.0021483
 
 Not queried
 
  0.5760152
 
       NA
 
           NA
  
 
  
 17
 
  76498808
 
  76499012
 
  ENSEMBL   UCSC 
 
  205
 
  4
 
 16822
 
 gene;island
 
 0.718204
 
 0.7720800
 
 Not queried
 
  0.5759496
 
       NA
 
           NA
  
 
  
 14
 
  36003367
 
  36003528
 
  ENSEMBL   UCSC 
 
  162
 
  4
 
 12065
 
 gene
 
 0.011299
 
 0.0083815
 
 Not queried
 
  0.5758966
 
       NA
 
           NA
  
 
  
 3
 
 147110229
 
 147110667
 
  ENSEMBL   UCSC 
 
  439
 
  8
 
 27986
 
 gene;tss;island
 
 0.050897
 
 0.0834906
 
 Not queried
 
  0.5758907
 
       NA
 
           NA
  
 
  
 12
 
 101673672
 
 101673815
 
  ENSEMBL   UCSC 
 
  144
 
  5
 
 9944
 
 tss
 
 0.012622
 
 0.0061196
 
 Not queried
 
  0.5758893
 
       NA
 
           NA
  
 
  
 8
 
  86157735
 
  86157935
 
  ENSEMBL   UCSC 
 
  201
 
  4
 
 39353
 
 gene
 
 0.009618
 
 0.0080668
 
 Not queried
 
  0.5756891
 
       NA
 
           NA
  
 
  
 10
 
  31609882
 
  31609960
 
  ENSEMBL   UCSC 
 
   79
 
  6
 
 5018
 
 tss
 
 0.002276
 
 0.0060356
 
 Not queried
 
  0.5756746
 
       NA
 
           NA
  
 
  
 14
 
  89259551
 
  89259748
 
  ENSEMBL   UCSC 
 
  198
 
  4
 
 12547
 
 tss;island
 
 0.041926
 
 0.0314571
 
 Not queried
 
  0.5756303
 
       NA
 
           NA
  
 
  
 12
 
  70636482
 
  70636659
 
  ENSEMBL   UCSC 
 
  178
 
  9
 
 9866
 
 tss;island
 
 0.002832
 
 0.0056963
 
 Not queried
 
  0.5756169
 
       NA
 
           NA
  
 
  
 13
 
  28712342
 
  28712670
 
  ENSEMBL   UCSC 
 
  329
 
  4
 
 11118
 
 gene;island
 
 0.003919
 
 0.0102584
 
 Not queried
 
  0.5755958
 
       NA
 
           NA
  
 
  
 1
 
 241587493
 
 241587783
 
  ENSEMBL   UCSC 
 
  291
 
  4
 
 4377
 
 island
 
 0.050520
 
 0.0333971
 
 Not queried
 
  0.5755801
 
       NA
 
           NA
  
 
  
 17
 
  42401753
 
  42402188
 
  ENSEMBL   UCSC 
 
  436
 
  5
 
 16616
 
 gene
 
 0.001761
 
 0.0028227
 
 Not queried
 
  0.5755162
 
       NA
 
           NA
  
 
  
 8
 
  63951642
 
  63952060
 
  ENSEMBL   UCSC 
 
  419
 
  8
 
 39730
 
 tss
 
 0.013587
 
 0.0091019
 
 Not queried
 
  0.5754976
 
       NA
 
           NA
  
 
  
 1
 
  94883469
 
  94883754
 
  ENSEMBL   UCSC 
 
  286
 
  7
 
 1764
 
 tss;island
 
 0.015484
 
 0.0106832
 
 Not queried
 
  0.5754754
 
       NA
 
           NA
  
 
  
 12
 
   1100377
 
   1100598
 
  ENSEMBL   UCSC 
 
  222
 
  7
 
 10148
 
 island
 
 0.004596
 
 0.0068820
 
 Not queried
 
  0.5754365
 
       NA
 
           NA
  
 
  
 2
 
 128568865
 
 128569347
 
  ENSEMBL   UCSC 
 
  483
 
  8
 
 23617
 
 tss;island
 
 0.006316
 
 0.0063097
 
 Not queried
 
  0.5754312
 
       NA
 
           NA
  
 
  
 14
 
  51706677
 
  51706820
 
  ENSEMBL   UCSC 
 
  144
 
  4
 
 12392
 
 tss
 
 0.007379
 
 0.0036675
 
 Not queried
 
  0.5753497
 
       NA
 
           NA
  
 
  
 6
 
  20534695
 
  20534796
 
  ENSEMBL   UCSC 
 
  102
 
  6
 
 33588
 
 gene
 
 0.010616
 
 0.0129000
 
 Not queried
 
  0.5753304
 
       NA
 
           NA
  
 
  
 14
 
  21979464
 
  21979675
 
  ENSEMBL   UCSC 
 
  212
 
  8
 
 12285
 
 tss
 
 0.004352
 
 0.0034795
 
 Not queried
 
  0.5752939
 
       NA
 
           NA
  
 
  
 16
 
   4466639
 
   4466888
 
  ENSEMBL   UCSC 
 
  250
 
  4
 
 15067
 
 tss
 
 0.007344
 
 0.0092970
 
 Not queried
 
  0.5751365
 
       NA
 
           NA
  
 
  
 14
 
  20811627
 
  20811808
 
  ENSEMBL   UCSC 
 
  182
 
  4
 
 12268
 
 tss
 
 0.002034
 
 0.0010091
 
 Not queried
 
  0.5750687
 
       NA
 
           NA
  
 
  
 8
 
 145137100
 
 145137346
 
  ENSEMBL   UCSC 
 
  247
 
  7
 
 39971
 
 tss;island
 
 0.016289
 
 0.0202812
 
 Not queried
 
  0.5749444
 
       NA
 
           NA
  
 
  
 16
 
   9857475
 
   9857925
 
  ENSEMBL   UCSC 
 
  451
 
  4
 
 14657
 
 gene
 
 0.690817
 
 0.5913653
 
 Not queried
 
  0.5748895
 
       NA
 
           NA
  
 
  
 8
 
  74884436
 
  74884794
 
  ENSEMBL   UCSC 
 
  359
 
  8
 
 39771
 
 tss
 
 0.004893
 
 0.0038209
 
 Not queried
 
  0.5747937
 
       NA
 
           NA
  
 
  
 1
 
  19536960
 
  19537115
 
  ENSEMBL   UCSC 
 
  156
 
  4
 
 1298
 
 tss;island
 
 0.002343
 
 0.0012813
 
 Not queried
 
  0.5746649
 
       NA
 
           NA
  
 
  
 2
 
  25142117
 
  25142523
 
  ENSEMBL   UCSC 
 
  407
 
  6
 
 23228
 
 tss
 
 0.014670
 
 0.0080111
 
 Not queried
 
  0.5745829
 
       NA
 
           NA
  
 
  
 12
 
   6756671
 
   6756815
 
  ENSEMBL   UCSC 
 
  145
 
  4
 
 9519
 
 tss
 
 0.314880
 
 0.4588648
 
 Not queried
 
  0.5745486
 
       NA
 
           NA
  
 
  
 22
 
  35695625
 
  35695944
 
  ENSEMBL   UCSC 
 
  320
 
  5
 
 26851
 
 gene;island
 
 0.003189
 
 0.0052576
 
 Not queried
 
  0.5745281
 
       NA
 
           NA
  
 
  
 16
 
  75599896
 
  75600069
 
  ENSEMBL   UCSC 
 
  174
 
  4
 
 15364
 
 tss;island
 
 0.021353
 
 0.0097884
 
 Not queried
 
  0.5744324
 
       NA
 
           NA
  
 
  
 17
 
  80621636
 
  80621858
 
  ENSEMBL   UCSC 
 
  223
 
  4
 
 16955
 
 gene;island
 
 0.831057
 
 0.8681570
 
 Not queried
 
  0.5743696
 
       NA
 
           NA
  
 
  
 11
 
   9635889
 
   9636089
 
  ENSEMBL   UCSC 
 
  201
 
  4
 
 8050
 
 island
 
 0.012114
 
 0.0114890
 
 Not queried
 
  0.5743391
 
       NA
 
           NA
  
 
  
 5
 
 139780509
 
 139780877
 
  ENSEMBL   UCSC 
 
  369
 
  4
 
 32340
 
 tss;island
 
 0.001292
 
 0.0011468
 
 Not queried
 
  0.5743222
 
       NA
 
           NA
  
 
  
 11
 
  67888858
 
  67889237
 
  ENSEMBL   UCSC 
 
  380
 
  8
 
 7507
 
 tss
 
 0.015902
 
 0.0095765
 
 Not queried
 
  0.5742939
 
       NA
 
           NA
  
 
  
 17
 
  66243584
 
  66243669
 
  ENSEMBL   UCSC 
 
   86
 
  4
 
 17604
 
 tss;island
 
 0.025411
 
 0.0158416
 
 Not queried
 
  0.5742557
 
       NA
 
           NA
  
 
  
 6
 
  26501812
 
  26502192
 
  ENSEMBL   UCSC 
 
  381
 
  5
 
 33613
 
 gene;island
 
 0.036494
 
 0.0545267
 
 Not queried
 
  0.5742166
 
       NA
 
           NA
  
 
  
 22
 
  18893811
 
  18894001
 
  ENSEMBL   UCSC 
 
  191
 
  4
 
 26785
 
 gene
 
 0.016026
 
 0.0105489
 
 Not queried
 
  0.5740263
 
       NA
 
           NA
  
 
  
 22
 
  22987059
 
  22987126
 
  ENSEMBL   UCSC 
 
   68
 
  5
 
 27005
 
 tss;island
 
 0.971544
 
 0.9770884
 
 Not queried
 
  0.5739134
 
       NA
 
           NA
  
 
  
 6
 
  41888549
 
  41889049
 
  ENSEMBL   UCSC 
 
  501
 
 11
 
 34975
 
 tss;island
 
 0.005724
 
 0.0070984
 
 Not queried
 
  0.5739032
 
       NA
 
           NA
  
 
  
 4
 
 174291930
 
 174292144
 
  ENSEMBL   UCSC 
 
  215
 
  8
 
 31423
 
 island
 
 0.010048
 
 0.0096501
 
 Not queried
 
  0.5738946
 
       NA
 
           NA
  
 
  
 13
 
  43148684
 
  43149281
 
  ENSEMBL   UCSC 
 
  598
 
  7
 
 11147
 
 gene;island
 
 0.013635
 
 0.0070351
 
 Not queried
 
  0.5738482
 
       NA
 
           NA
  
 
  
 17
 
  79026049
 
  79026354
 
  ENSEMBL   UCSC 
 
  306
 
  5
 
 16889
 
 gene;island
 
 0.490673
 
 0.5990449
 
 Not queried
 
  0.5738056
 
       NA
 
           NA
  
 
  
 10
 
 124402673
 
 124403063
 
  ENSEMBL   UCSC 
 
  391
 
  5
 
 4798
 
 gene;island
 
 0.462032
 
 0.4707786
 
 Not queried
 
  0.5737921
 
       NA
 
           NA
  
 
  
 10
 
  90967612
 
  90968091
 
  ENSEMBL   UCSC 
 
  480
 
  6
 
 5212
 
 tss;island
 
 0.018309
 
 0.0270509
 
 Not queried
 
  0.5737420
 
       NA
 
           NA
  
 
  
 9
 
  74061171
 
  74061514
 
  ENSEMBL   UCSC 
 
  344
 
  4
 
 40892
 
 island
 
 0.252270
 
 0.2957400
 
 Not queried
 
  0.5737399
 
       NA
 
           NA
  
 
  
 6
 
 100062488
 
 100062855
 
  ENSEMBL   UCSC 
 
  368
 
  5
 
 34430
 
 gene;island
 
 0.081526
 
 0.0516213
 
 Not queried
 
  0.5737189
 
       NA
 
           NA
  
 
  
 1
 
  35442212
 
  35442255
 
  ENSEMBL   UCSC 
 
   44
 
  4
 
 3031
 
 island
 
 0.980283
 
 0.9859351
 
 Not queried
 
  0.5734475
 
       NA
 
           NA
  
 
  
 10
 
  28287388
 
  28288192
 
  ENSEMBL   UCSC 
 
  805
 
 13
 
 5636
 
 island
 
 0.028591
 
 0.0138460
 
 Not queried
 
  0.5734283
 
       NA
 
           NA
  
 
  
 1
 
   1322461
 
   1322923
 
  ENSEMBL   UCSC 
 
  463
 
  5
 
 34
 
 gene;island
 
 0.988606
 
 0.9829325
 
 Not queried
 
  0.5731086
 
       NA
 
           NA
  
 
  
 3
 
  46037408
 
  46037608
 
  ENSEMBL   UCSC 
 
  201
 
  6
 
 28273
 
 tss;island
 
 0.148758
 
 0.1276927
 
 Not queried
 
  0.5730260
 
       NA
 
           NA
  
 
  
 3
 
  47844180
 
  47844363
 
  ENSEMBL   UCSC 
 
  184
 
  8
 
 28291
 
 tss;island
 
 0.004414
 
 0.0092168
 
 Not queried
 
  0.5729367
 
       NA
 
           NA
  
 
  
 5
 
  54469363
 
  54469551
 
  ENSEMBL   UCSC 
 
  189
 
  4
 
 32064
 
 tss;island
 
 0.007736
 
 0.0158733
 
 Not queried
 
  0.5727768
 
       NA
 
           NA
  
 
  
 3
 
   8693749
 
   8693859
 
  ENSEMBL   UCSC 
 
  111
 
  4
 
 28121
 
 tss
 
 0.440812
 
 0.5310356
 
 Not queried
 
  0.5727705
 
       NA
 
           NA
  
 
  
 18
 
  19192099
 
  19192502
 
  ENSEMBL   UCSC 
 
  404
 
  7
 
 19418
 
 island
 
 0.019097
 
 0.0141359
 
 Not queried
 
  0.5727212
 
       NA
 
           NA
  
 
  
 19
 
  18303849
 
  18304032
 
  ENSEMBL   UCSC 
 
  184
 
  7
 
 20612
 
 tss
 
 0.003368
 
 0.0020753
 
 Not queried
 
  0.5726927
 
       NA
 
           NA
  
 
  
 19
 
  18303849
 
  18304045
 
  ENSEMBL   UCSC 
 
  197
 
  9
 
 21771
 
 island
 
 0.003368
 
 0.0020753
 
 Not queried
 
  0.5726927
 
       NA
 
           NA
  
 
  
 7
 
   2595255
 
   2595589
 
  ENSEMBL   UCSC 
 
  335
 
  8
 
 37412
 
 tss
 
 0.005738
 
 0.0081510
 
 Not queried
 
  0.5726619
 
       NA
 
           NA
  
 
  
 22
 
  18923787
 
  18923986
 
  ENSEMBL   UCSC 
 
  200
 
  5
 
 27287
 
 island
 
 0.006287
 
 0.0217330
 
 Not queried
 
  0.5725123
 
       NA
 
           NA
  
 
  
 19
 
  50167792
 
  50168129
 
  ENSEMBL   UCSC 
 
  338
 
  4
 
 20158
 
 gene;tss;island
 
 0.003476
 
 0.0027405
 
 Not queried
 
  0.5722699
 
       NA
 
           NA
  
 
  
 7
 
  99678668
 
  99679036
 
  ENSEMBL   UCSC 
 
  369
 
  4
 
 37133
 
 gene;island
 
 0.015012
 
 0.0220880
 
 Not queried
 
  0.5721982
 
       NA
 
           NA
  
 
  
 1
 
 155197152
 
 155197843
 
  ENSEMBL   UCSC 
 
  692
 
 10
 
 3818
 
 island
 
 0.007533
 
 0.0059555
 
 Not queried
 
  0.5721209
 
       NA
 
           NA
  
 
  
 13
 
  80915061
 
  80915130
 
  ENSEMBL   UCSC 
 
   70
 
  4
 
 11801
 
 island
 
 0.012951
 
 0.0272002
 
 Not queried
 
  0.5720111
 
       NA
 
           NA
  
 
  
 19
 
   1313407
 
   1313642
 
  ENSEMBL   UCSC 
 
  236
 
  4
 
 21214
 
 island
 
 0.562385
 
 0.6428501
 
 Not queried
 
  0.5719815
 
       NA
 
           NA
  
 
  
 22
 
  45559239
 
  45559602
 
  ENSEMBL   UCSC 
 
  364
 
  4
 
 27217
 
 tss;island
 
 0.004192
 
 0.0044094
 
 Not queried
 
  0.5718520
 
       NA
 
           NA
  
 
  
 1
 
  77333749
 
  77334256
 
  ENSEMBL   UCSC 
 
  508
 
  7
 
 592
 
 gene;island
 
 0.009374
 
 0.0418037
 
 Not queried
 
  0.5718427
 
       NA
 
           NA
  
 
  
 1
 
 228566548
 
 228566997
 
  ENSEMBL   UCSC 
 
  450
 
  5
 
 4292
 
 island
 
 0.040715
 
 0.0609063
 
 Not queried
 
  0.5718021
 
       NA
 
           NA
  
 
  
 2
 
 132233726
 
 132234134
 
  ENSEMBL   UCSC 
 
  409
 
  5
 
 22871
 
 gene
 
 0.959924
 
 0.9684903
 
 Not queried
 
  0.5717748
 
       NA
 
           NA
  
 
  
 19
 
  52643207
 
  52643487
 
  ENSEMBL   UCSC 
 
  281
 
  6
 
 21007
 
 tss
 
 0.020922
 
 0.0121402
 
 Not queried
 
  0.5717117
 
       NA
 
           NA
  
 
  
 7
 
   4174939
 
   4175403
 
  ENSEMBL   UCSC 
 
  465
 
  5
 
 36830
 
 gene
 
 0.461047
 
 0.5594600
 
 Not queried
 
  0.5716424
 
       NA
 
           NA
  
 
  
 1
 
 241802961
 
 241803367
 
  ENSEMBL   UCSC 
 
  407
 
  4
 
 1109
 
 gene;island
 
 0.026414
 
 0.0155286
 
 Not queried
 
  0.5714357
 
       NA
 
           NA
  
 
  
 6
 
  71666803
 
  71666858
 
  ENSEMBL   UCSC 
 
   56
 
  4
 
 35060
 
 tss
 
 0.030319
 
 0.0348502
 
 Not queried
 
  0.5713996
 
       NA
 
           NA
  
 
  
 16
 
  88730291
 
  88730494
 
  ENSEMBL   UCSC 
 
  204
 
  4
 
 14910
 
 gene;tss;island
 
 0.015393
 
 0.0147030
 
 Not queried
 
  0.5713734
 
       NA
 
           NA
  
 
  
 10
 
  98945025
 
  98946238
 
  ENSEMBL   UCSC 
 
 1214
 
 15
 
 5977
 
 island
 
 0.009006
 
 0.0112166
 
 Not queried
 
  0.5713507
 
       NA
 
           NA
  
 
  
 2
 
 134024093
 
 134024618
 
  ENSEMBL   UCSC 
 
  526
 
  5
 
 22880
 
 gene;island
 
 0.048529
 
 0.0595810
 
 Not queried
 
  0.5711367
 
       NA
 
           NA
  
 
  
 1
 
 224301873
 
 224302585
 
  ENSEMBL   UCSC 
 
  713
 
 10
 
 1026
 
 gene
 
 0.007917
 
 0.0067871
 
 Not queried
 
  0.5711353
 
       NA
 
           NA
  
 
  
 10
 
  73648638
 
  73648991
 
  ENSEMBL   UCSC 
 
  354
 
  4
 
 5834
 
 island
 
 0.005348
 
 0.0059087
 
 Not queried
 
  0.5709226
 
       NA
 
           NA
  
 
  
 2
 
 238600469
 
 238600693
 
  ENSEMBL   UCSC 
 
  225
 
  4
 
 23092
 
 gene;tss;island
 
 0.016149
 
 0.0157814
 
 Not queried
 
  0.5708333
 
       NA
 
           NA
  
 
  
 6
 
  35109242
 
  35109722
 
  ENSEMBL   UCSC 
 
  481
 
  9
 
 34926
 
 tss
 
 0.941698
 
 0.9730406
 
 Not queried
 
  0.5708253
 
       NA
 
           NA
  
 
  
 22
 
  38713874
 
  38714466
 
  ENSEMBL   UCSC 
 
  593
 
 10
 
 27137
 
 tss;island
 
 0.543722
 
 0.5717256
 
 Not queried
 
  0.5707841
 
       NA
 
           NA
  
 
  
 1
 
   6653447
 
   6653634
 
  ENSEMBL   UCSC 
 
  188
 
  4
 
 180
 
 gene;island
 
 0.772676
 
 0.8707085
 
 Not queried
 
  0.5707491
 
       NA
 
           NA
  
 
  
 2
 
  70520884
 
  70521051
 
  ENSEMBL   UCSC 
 
  168
 
  5
 
 23417
 
 tss
 
 0.004716
 
 0.0033391
 
 Not queried
 
  0.5706826
 
       NA
 
           NA
  
 
  
 11
 
  69590282
 
  69590580
 
  ENSEMBL   UCSC 
 
  299
 
  7
 
 7522
 
 tss
 
 0.008009
 
 0.0077935
 
 Not queried
 
  0.5706613
 
       NA
 
           NA
  
 
  
 5
 
  89825494
 
  89825908
 
  ENSEMBL   UCSC 
 
  415
 
  7
 
 32173
 
 tss
 
 0.003728
 
 0.0039306
 
 Not queried
 
  0.5705789
 
       NA
 
           NA
  
 
  
 5
 
  89825117
 
  89825908
 
  ENSEMBL   UCSC 
 
  792
 
  9
 
 32954
 
 island
 
 0.003597
 
 0.0039306
 
 Not queried
 
  0.5705789
 
       NA
 
           NA
  
 
  
 17
 
  75524913
 
  75525151
 
  ENSEMBL   UCSC 
 
  239
 
  4
 
 18811
 
 island
 
 0.049927
 
 0.0395907
 
 Not queried
 
  0.5705631
 
       NA
 
           NA
  
 
  
 19
 
  40949191
 
  40949452
 
  ENSEMBL   UCSC 
 
  262
 
  5
 
 20042
 
 gene;tss;island
 
 0.009297
 
 0.0054313
 
 Not queried
 
  0.5705431
 
       NA
 
           NA
  
 
  
 1
 
  11724238
 
  11724943
 
  ENSEMBL   UCSC 
 
  706
 
  8
 
 223
 
 gene
 
 0.006919
 
 0.0047791
 
 Not queried
 
  0.5704237
 
       NA
 
           NA
  
 
  
 19
 
  46010592
 
  46010836
 
  ENSEMBL   UCSC 
 
  245
 
  4
 
 22143
 
 island
 
 0.003619
 
 0.0031137
 
 Not queried
 
  0.5703620
 
       NA
 
           NA
  
 
  
 5
 
 134210707
 
 134210839
 
  ENSEMBL   UCSC 
 
  133
 
  4
 
 31754
 
 gene;island
 
 0.031864
 
 0.0066162
 
 Not queried
 
  0.5703179
 
       NA
 
           NA
  
 
  
 21
 
  48055212
 
  48055519
 
  ENSEMBL   UCSC 
 
  308
 
  5
 
 26602
 
 tss
 
 0.013119
 
 0.0037912
 
 Not queried
 
  0.5702380
 
       NA
 
           NA
  
 
  
 21
 
  48055212
 
  48055893
 
  ENSEMBL   UCSC 
 
  682
 
  9
 
 26776
 
 island
 
 0.013119
 
 0.0107981
 
 Not queried
 
  0.5702380
 
       NA
 
           NA
  
 
  
 6
 
  26033394
 
  26033735
 
  ENSEMBL   UCSC 
 
  342
 
  5
 
 33598
 
 gene
 
 0.009073
 
 0.0118228
 
 Not queried
 
  0.5701507
 
       NA
 
           NA
  
 
  
 3
 
  74663507
 
  74663796
 
  ENSEMBL   UCSC 
 
  290
 
  4
 
 29287
 
 island
 
 0.029457
 
 0.1099136
 
 Not queried
 
  0.5701368
 
       NA
 
           NA
  
 
  
 19
 
   2085286
 
   2085483
 
  ENSEMBL   UCSC 
 
  198
 
  4
 
 19678
 
 gene
 
 0.003405
 
 0.0031518
 
 Not queried
 
  0.5699556
 
       NA
 
           NA
  
 
  
 1
 
 110754087
 
 110754436
 
  ENSEMBL   UCSC 
 
  350
 
  4
 
 675
 
 gene
 
 0.046393
 
 0.0375020
 
 Not queried
 
  0.5698971
 
       NA
 
           NA
  
 
  
 5
 
  59189375
 
  59189934
 
  ENSEMBL   UCSC 
 
  560
 
 10
 
 31625
 
 gene;island
 
 0.105468
 
 0.2511781
 
 Not queried
 
  0.5698621
 
       NA
 
           NA
  
 
  
 10
 
 101088533
 
 101088828
 
  ENSEMBL   UCSC 
 
  296
 
  4
 
 5280
 
 tss
 
 0.137446
 
 0.5344573
 
 Not queried
 
  0.5698538
 
       NA
 
           NA
  
 
  
 16
 
  89686618
 
  89686913
 
  ENSEMBL   UCSC 
 
  296
 
  4
 
 15428
 
 tss
 
 0.268860
 
 0.3863614
 
 Not queried
 
  0.5698042
 
       NA
 
           NA
  
 
  
 12
 
  12878211
 
  12878440
 
  ENSEMBL   UCSC 
 
  230
 
  4
 
 9575
 
 tss;island
 
 0.026749
 
 0.0186506
 
 Not queried
 
  0.5695394
 
       NA
 
           NA
  
 
  
 7
 
 102389191
 
 102389392
 
  ENSEMBL   UCSC 
 
  202
 
  7
 
 37806
 
 tss
 
 0.006390
 
 0.0019867
 
 Not queried
 
  0.5694598
 
       NA
 
           NA
  
 
  
 13
 
  32605187
 
  32605406
 
  ENSEMBL   UCSC 
 
  220
 
  7
 
 11381
 
 tss;island
 
 0.017947
 
 0.0208304
 
 Not queried
 
  0.5693851
 
       NA
 
           NA
  
 
  
 2
 
  45838293
 
  45838581
 
  ENSEMBL   UCSC 
 
  289
 
  7
 
 24271
 
 island
 
 0.006740
 
 0.0036568
 
 Not queried
 
  0.5693213
 
       NA
 
           NA
  
 
  
 7
 
 150745278
 
 150745709
 
  ENSEMBL   UCSC 
 
  432
 
  7
 
 39020
 
 island
 
 0.874685
 
 0.8344667
 
 Not queried
 
  0.5693042
 
       NA
 
           NA
  
 
  
 5
 
 122971759
 
 122971951
 
  ENSEMBL   UCSC 
 
  193
 
  4
 
 33058
 
 island
 
 0.761427
 
 0.8405341
 
 Not queried
 
  0.5693034
 
       NA
 
           NA
  
 
  
 16
 
  85157762
 
  85158047
 
  ENSEMBL   UCSC 
 
  286
 
  5
 
 16149
 
 island
 
 0.579123
 
 0.6587673
 
 Not queried
 
  0.5691063
 
       NA
 
           NA
  
 
  
 19
 
   2163593
 
   2163832
 
  ENSEMBL   UCSC 
 
  240
 
  4
 
 20320
 
 tss;island
 
 0.004111
 
 0.0037328
 
 Not queried
 
  0.5690117
 
       NA
 
           NA
  
 
  
 6
 
  41470812
 
  41471064
 
  ENSEMBL   UCSC 
 
  253
 
  4
 
 36122
 
 island
 
 0.314446
 
 0.3717717
 
 Not queried
 
  0.5690110
 
       NA
 
           NA
  
 
  
 17
 
  80189962
 
  80190136
 
  ENSEMBL   UCSC 
 
  175
 
  6
 
 17770
 
 tss
 
 0.067126
 
 0.1653950
 
 Not queried
 
  0.5687915
 
       NA
 
           NA
  
 
  
 18
 
  56529784
 
  56529878
 
  ENSEMBL   UCSC 
 
   95
 
  5
 
 19296
 
 tss;island
 
 0.980194
 
 0.9847695
 
 Not queried
 
  0.5687242
 
       NA
 
           NA
  
 
  
 6
 
  32977983
 
  32978775
 
  ENSEMBL   UCSC 
 
  793
 
 16
 
 34887
 
 tss;island
 
 0.981318
 
 0.9674406
 
 Not queried
 
  0.5686917
 
       NA
 
           NA
  
 
  
 4
 
  41753439
 
  41754125
 
  ENSEMBL   UCSC 
 
  687
 
  8
 
 31004
 
 island
 
 0.059501
 
 0.0359108
 
 Not queried
 
  0.5686099
 
       NA
 
           NA
  
 
  
 17
 
  62339926
 
  62340143
 
  ENSEMBL   UCSC 
 
  218
 
  4
 
 16732
 
 gene;island
 
 0.006217
 
 0.0107398
 
 Not queried
 
  0.5685207
 
       NA
 
           NA
  
 
  
 6
 
  34725116
 
  34725359
 
  ENSEMBL   UCSC 
 
  244
 
  5
 
 34305
 
 gene;island
 
 0.002394
 
 0.0018276
 
 Not queried
 
  0.5684625
 
       NA
 
           NA
  
 
  
 7
 
 150725083
 
 150725503
 
  ENSEMBL   UCSC 
 
  421
 
  8
 
 37985
 
 tss
 
 0.011036
 
 0.0048635
 
 Not queried
 
  0.5682399
 
       NA
 
           NA
  
 
  
 1
 
  33592870
 
  33593154
 
  ENSEMBL   UCSC 
 
  285
 
  5
 
 3008
 
 island
 
 0.003106
 
 0.0053285
 
 Not queried
 
  0.5682129
 
       NA
 
           NA
  
 
  
 2
 
 208031734
 
 208031995
 
  ENSEMBL   UCSC 
 
  262
 
  5
 
 23841
 
 tss;island
 
 0.012674
 
 0.0254272
 
 Not queried
 
  0.5681744
 
       NA
 
           NA
  
 
  
 21
 
  34350407
 
  34350934
 
  ENSEMBL   UCSC 
 
  528
 
  6
 
 26641
 
 island
 
 0.627434
 
 0.7171006
 
 Not queried
 
  0.5681168
 
       NA
 
           NA
  
 
  
 7
 
 142960290
 
 142960510
 
  ENSEMBL   UCSC 
 
  221
 
  6
 
 37950
 
 tss
 
 0.009836
 
 0.0073786
 
 Not queried
 
  0.5681137
 
       NA
 
           NA
  
 
  
 2
 
 242295487
 
 242295778
 
  ENSEMBL   UCSC 
 
  292
 
  4
 
 25292
 
 island
 
 0.004190
 
 0.0034140
 
 Not queried
 
  0.5680218
 
       NA
 
           NA
  
 
  
 1
 
   7740243
 
   7740286
 
  ENSEMBL   UCSC 
 
   44
 
  4
 
 187
 
 gene;island
 
 0.011666
 
 0.0117774
 
 Not queried
 
  0.5680195
 
       NA
 
           NA
  
 
  
 8
 
 101117949
 
 101118083
 
  ENSEMBL   UCSC 
 
  135
 
  5
 
 39376
 
 gene;island
 
 0.863599
 
 0.9036906
 
 Not queried
 
  0.5679241
 
       NA
 
           NA
  
 
  
 11
 
  64039065
 
  64039302
 
  ENSEMBL   UCSC 
 
  238
 
  4
 
 6672
 
 gene;island
 
 0.008090
 
 0.0043426
 
 Not queried
 
  0.5678432
 
       NA
 
           NA
  
 
  
 3
 
 138665654
 
 138665957
 
  ENSEMBL   UCSC 
 
  304
 
  4
 
 27966
 
 gene
 
 0.052067
 
 0.0427638
 
 Not queried
 
  0.5676376
 
       NA
 
           NA
  
 
  
 11
 
  65339691
 
  65339797
 
  ENSEMBL   UCSC 
 
  107
 
  5
 
 7434
 
 tss;island
 
 0.009506
 
 0.0040897
 
 Not queried
 
  0.5675933
 
       NA
 
           NA
  
 
  
 1
 
  58715499
 
  58715747
 
  ENSEMBL   UCSC 
 
  249
 
  6
 
 556
 
 gene;island
 
 0.012738
 
 0.0289416
 
 Not queried
 
  0.5675532
 
       NA
 
           NA
  
 
  
 2
 
  70780723
 
  70781325
 
  ENSEMBL   UCSC 
 
  603
 
 11
 
 24383
 
 island
 
 0.016616
 
 0.0082773
 
 Not queried
 
  0.5675104
 
       NA
 
           NA
  
 
  
 10
 
  71562314
 
  71562858
 
  ENSEMBL   UCSC 
 
  545
 
  4
 
 4616
 
 gene;island
 
 0.076603
 
 0.0807164
 
 Not queried
 
  0.5674331
 
       NA
 
           NA
  
 
  
 10
 
    409280
 
    409541
 
  ENSEMBL   UCSC 
 
  262
 
  4
 
 4435
 
 gene;island
 
 0.488893
 
 0.5134470
 
 Not queried
 
  0.5673869
 
       NA
 
           NA
  
 
  
 3
 
  52351355
 
  52351963
 
  ENSEMBL   UCSC 
 
  609
 
  5
 
 27817
 
 gene
 
 0.014782
 
 0.0214430
 
 Not queried
 
  0.5672470
 
       NA
 
           NA
  
 
  
 16
 
   1993336
 
   1993707
 
  ENSEMBL   UCSC 
 
  372
 
  7
 
 15010
 
 tss
 
 0.009763
 
 0.0060497
 
 Not queried
 
  0.5672300
 
       NA
 
           NA
  
 
  
 11
 
  36531754
 
  36532056
 
  ENSEMBL   UCSC 
 
  303
 
  6
 
 8211
 
 island
 
 0.008038
 
 0.0047618
 
 Not queried
 
  0.5670450
 
       NA
 
           NA
  
 
  
 20
 
  19915716
 
  19915874
 
  ENSEMBL   UCSC 
 
  159
 
  6
 
 25369
 
 gene
 
 0.050866
 
 0.0643210
 
 Not queried
 
  0.5669849
 
       NA
 
           NA
  
 
  
 12
 
 110149962
 
 110150261
 
  ENSEMBL   UCSC 
 
  300
 
  4
 
 10783
 
 island
 
 0.331893
 
 0.2901467
 
 Not queried
 
  0.5669693
 
       NA
 
           NA
  
 
  
 10
 
 112326929
 
 112327767
 
  ENSEMBL   UCSC 
 
  839
 
 12
 
 6131
 
 island
 
 0.005240
 
 0.0046528
 
 Not queried
 
  0.5669501
 
       NA
 
           NA
  
 
  
 19
 
  17905589
 
  17905835
 
  ENSEMBL   UCSC 
 
  247
 
  5
 
 20600
 
 tss;island
 
 0.044530
 
 0.0466221
 
 Not queried
 
  0.5668702
 
       NA
 
           NA
  
 
  
 7
 
 130125200
 
 130126637
 
  ENSEMBL   UCSC 
 
 1438
 
 18
 
 38921
 
 island
 
 0.182825
 
 0.1962546
 
 Not queried
 
  0.5668600
 
       NA
 
           NA
  
 
  
 10
 
  23460245
 
  23460646
 
  ENSEMBL   UCSC 
 
  402
 
  4
 
 5607
 
 island
 
 0.483519
 
 0.3739650
 
 Not queried
 
  0.5668207
 
       NA
 
           NA
  
 
  
 10
 
 118083701
 
 118083921
 
  ENSEMBL   UCSC 
 
  221
 
  5
 
 5384
 
 tss
 
 0.850550
 
 0.8870399
 
 Not queried
 
  0.5667936
 
       NA
 
           NA
  
 
  
 17
 
  75136725
 
  75136989
 
  ENSEMBL   UCSC 
 
  265
 
  7
 
 17694
 
 tss
 
 0.004858
 
 0.0020107
 
 Not queried
 
  0.5667835
 
       NA
 
           NA
  
 
  
 17
 
  75136725
 
  75137144
 
  ENSEMBL   UCSC 
 
  420
 
  9
 
 18802
 
 island
 
 0.004858
 
 0.0020107
 
 Not queried
 
  0.5667835
 
       NA
 
           NA
  
 
  
 7
 
   1460673
 
   1460944
 
  ENSEMBL   UCSC 
 
  272
 
  4
 
 38104
 
 island
 
 0.671338
 
 0.7401175
 
 Not queried
 
  0.5667805
 
       NA
 
           NA
  
 
  
 20
 
  61903843
 
  61904111
 
  ENSEMBL   UCSC 
 
  269
 
  8
 
 25894
 
 tss
 
 0.005573
 
 0.0020405
 
 Not queried
 
  0.5667499
 
       NA
 
           NA
  
 
  
 1
 
   1683738
 
   1684095
 
  ENSEMBL   UCSC 
 
  358
 
  5
 
 47
 
 gene;island
 
 0.964344
 
 0.9799619
 
 Not queried
 
  0.5666622
 
       NA
 
           NA
  
 
  
 4
 
   8345155
 
   8345588
 
  ENSEMBL   UCSC 
 
  434
 
  4
 
 30919
 
 island
 
 0.621900
 
 0.6930176
 
 Not queried
 
  0.5663144
 
       NA
 
           NA
  
 
  
 2
 
 108443292
 
 108443511
 
  ENSEMBL   UCSC 
 
  220
 
  7
 
 23561
 
 tss;island
 
 0.894393
 
 0.9261313
 
 Not queried
 
  0.5663063
 
       NA
 
           NA
  
 
  
 3
 
  71802420
 
  71802608
 
  ENSEMBL   UCSC 
 
  189
 
  4
 
 27859
 
 gene;tss;island
 
 0.122400
 
 0.0712210
 
 Not queried
 
  0.5662989
 
       NA
 
           NA
  
 
  
 2
 
 113299370
 
 113299708
 
  ENSEMBL   UCSC 
 
  339
 
  4
 
 24623
 
 island
 
 0.003570
 
 0.0024286
 
 Not queried
 
  0.5662912
 
       NA
 
           NA
  
 
  
 7
 
 150690699
 
 150690969
 
  ENSEMBL   UCSC 
 
  271
 
  4
 
 37280
 
 gene
 
 0.703971
 
 0.8265343
 
 Not queried
 
  0.5662869
 
       NA
 
           NA
  
 
  
 15
 
  52030519
 
  52030746
 
  ENSEMBL   UCSC 
 
  228
 
  4
 
 13352
 
 gene;tss;island
 
 0.926292
 
 0.7101633
 
 Not queried
 
  0.5661987
 
       NA
 
           NA
  
 
  
 1
 
 203830875
 
 203831055
 
  ENSEMBL   UCSC 
 
  181
 
  4
 
 955
 
 gene
 
 0.009089
 
 0.0034479
 
 Not queried
 
  0.5661687
 
       NA
 
           NA
  
 
  
 3
 
 119379414
 
 119379792
 
  ENSEMBL   UCSC 
 
  379
 
  4
 
 28524
 
 tss
 
 0.931268
 
 0.9446845
 
 Not queried
 
  0.5660748
 
       NA
 
           NA
  
 
  
 2
 
 219433438
 
 219433691
 
  ENSEMBL   UCSC 
 
  254
 
  4
 
 23881
 
 tss;island
 
 0.008371
 
 0.0087255
 
 Not queried
 
  0.5660651
 
       NA
 
           NA
  
 
  
 19
 
  49436579
 
  49436866
 
  ENSEMBL   UCSC 
 
  288
 
 10
 
 20932
 
 tss
 
 0.018490
 
 0.0249013
 
 Not queried
 
  0.5659768
 
       NA
 
           NA
  
 
  
 7
 
  72722590
 
  72723152
 
  ENSEMBL   UCSC 
 
  563
 
  8
 
 38551
 
 island
 
 0.007736
 
 0.0042141
 
 Not queried
 
  0.5659410
 
       NA
 
           NA
  
 
  
 18
 
  14132207
 
  14132697
 
  ENSEMBL   UCSC 
 
  491
 
  9
 
 19413
 
 island
 
 0.020238
 
 0.0118685
 
 Not queried
 
  0.5659168
 
       NA
 
           NA
  
 
  
 3
 
  48342880
 
  48343235
 
  ENSEMBL   UCSC 
 
  356
 
  7
 
 28295
 
 tss
 
 0.005809
 
 0.0065209
 
 Not queried
 
  0.5659100
 
       NA
 
           NA
  
 
  
 1
 
  67217673
 
  67218275
 
  ENSEMBL   UCSC 
 
  603
 
 12
 
 3361
 
 island
 
 0.042226
 
 0.0417128
 
 Not queried
 
  0.5659003
 
       NA
 
           NA
  
 
  
 17
 
  36613455
 
  36613704
 
  ENSEMBL   UCSC 
 
  250
 
  4
 
 18252
 
 island
 
 0.682257
 
 0.7945523
 
 Not queried
 
  0.5657833
 
       NA
 
           NA
  
 
  
 7
 
  89874187
 
  89874597
 
  ENSEMBL   UCSC 
 
  411
 
  8
 
 38647
 
 island
 
 0.021277
 
 0.0099676
 
 Not queried
 
  0.5655604
 
       NA
 
           NA
  
 
  
 12
 
 109535838
 
 109535998
 
  ENSEMBL   UCSC 
 
  161
 
  4
 
 9990
 
 tss
 
 0.014547
 
 0.0114897
 
 Not queried
 
  0.5654181
 
       NA
 
           NA
  
 
  
 11
 
  67290850
 
  67291026
 
  ENSEMBL   UCSC 
 
  177
 
  4
 
 8556
 
 island
 
 0.707885
 
 0.7710714
 
 Not queried
 
  0.5654084
 
       NA
 
           NA
  
 
  
 14
 
  55657967
 
  55658398
 
  ENSEMBL   UCSC 
 
  432
 
  5
 
 12865
 
 island
 
 0.020207
 
 0.0157984
 
 Not queried
 
  0.5653784
 
       NA
 
           NA
  
 
  
 6
 
  27356234
 
  27356513
 
  ENSEMBL   UCSC 
 
  280
 
  7
 
 34716
 
 tss
 
 0.006744
 
 0.0161170
 
 Not queried
 
  0.5652860
 
       NA
 
           NA
  
 
  
 8
 
  63951472
 
  63952060
 
  ENSEMBL   UCSC 
 
  589
 
  9
 
 40336
 
 island
 
 0.011095
 
 0.0066990
 
 Not queried
 
  0.5652448
 
       NA
 
           NA
  
 
  
 2
 
 130345044
 
 130345405
 
  ENSEMBL   UCSC 
 
  362
 
  4
 
 24712
 
 island
 
 0.657641
 
 0.8063453
 
 Not queried
 
  0.5651833
 
       NA
 
           NA
  
 
  
 18
 
  43683922
 
  43684181
 
  ENSEMBL   UCSC 
 
  260
 
  8
 
 19100
 
 gene
 
 0.042769
 
 0.0441614
 
 Not queried
 
  0.5649932
 
       NA
 
           NA
  
 
  
 8
 
 104426639
 
 104426919
 
  ENSEMBL   UCSC 
 
  281
 
  5
 
 39859
 
 tss
 
 0.015765
 
 0.0152730
 
 Not queried
 
  0.5649594
 
       NA
 
           NA
  
 
  
 17
 
   5026393
 
   5026840
 
  ENSEMBL   UCSC 
 
  448
 
  8
 
 17890
 
 island
 
 0.321921
 
 0.4838602
 
 Not queried
 
  0.5649256
 
       NA
 
           NA
  
 
  
 3
 
  97540262
 
  97540665
 
  ENSEMBL   UCSC 
 
  404
 
  5
 
 29312
 
 island
 
 0.007775
 
 0.0052981
 
 Not queried
 
  0.5648340
 
       NA
 
           NA
  
 
  
 6
 
  31894990
 
  31895257
 
  ENSEMBL   UCSC 
 
  268
 
  4
 
 34852
 
 tss
 
 0.719395
 
 0.6487908
 
 Not queried
 
  0.5648179
 
       NA
 
           NA
  
 
  
 6
 
  32917979
 
  32918967
 
  ENSEMBL   UCSC 
 
  989
 
  9
 
 34165
 
 gene
 
 0.539758
 
 0.8239351
 
 Not queried
 
  0.5646402
 
       NA
 
           NA
  
 
  
 12
 
   1799875
 
   1800136
 
  ENSEMBL   UCSC 
 
  262
 
  8
 
 9487
 
 tss;island
 
 0.010503
 
 0.0045759
 
 Not queried
 
  0.5644575
 
       NA
 
           NA
  
 
  
 1
 
 231473707
 
 231473791
 
  ENSEMBL   UCSC 
 
   85
 
  4
 
 1079
 
 gene
 
 0.026180
 
 0.0216037
 
 Not queried
 
  0.5644461
 
       NA
 
           NA
  
 
  
 3
 
 155422092
 
 155422145
 
  ENSEMBL   UCSC 
 
   54
 
  4
 
 28671
 
 tss
 
 0.392807
 
 0.4655006
 
 Not queried
 
  0.5643446
 
       NA
 
           NA
  
 
  
 7
 
 100033938
 
 100034454
 
  ENSEMBL   UCSC 
 
  517
 
  8
 
 38760
 
 island
 
 0.018840
 
 0.0116875
 
 Not queried
 
  0.5641300
 
       NA
 
           NA
  
 
  
 8
 
  74205882
 
  74205946
 
  ENSEMBL   UCSC 
 
   65
 
  4
 
 39768
 
 tss;island
 
 0.006837
 
 0.0033510
 
 Not queried
 
  0.5639590
 
       NA
 
           NA
  
 
  
 2
 
  55921045
 
  55921125
 
  ENSEMBL   UCSC 
 
   81
 
  6
 
 23364
 
 tss
 
 0.002281
 
 0.0039270
 
 Not queried
 
  0.5638692
 
       NA
 
           NA
  
 
  
 2
 
  74681787
 
  74682200
 
  ENSEMBL   UCSC 
 
  414
 
 11
 
 24433
 
 island
 
 0.007116
 
 0.0022870
 
 Not queried
 
  0.5637790
 
       NA
 
           NA
  
 
  
 1
 
  15763737
 
  15764095
 
  ENSEMBL   UCSC 
 
  359
 
  5
 
 1271
 
 tss
 
 0.698494
 
 0.8982689
 
 Not queried
 
  0.5637573
 
       NA
 
           NA
  
 
  
 4
 
   2341196
 
   2341463
 
  ENSEMBL   UCSC 
 
  268
 
  5
 
 29888
 
 gene;island
 
 0.731630
 
 0.6900105
 
 Not queried
 
  0.5637523
 
       NA
 
           NA
  
 
  
 7
 
  82073339
 
  82073810
 
  ENSEMBL   UCSC 
 
  472
 
  7
 
 37677
 
 tss;island
 
 0.056056
 
 0.0495270
 
 Not queried
 
  0.5637064
 
       NA
 
           NA
  
 
  
 6
 
  28479225
 
  28479996
 
  ENSEMBL   UCSC 
 
  772
 
 17
 
 33633
 
 gene
 
 0.736618
 
 0.8057537
 
 Not queried
 
  0.5635246
 
       NA
 
           NA
  
 
  
 22
 
  46481603
 
  46481822
 
  ENSEMBL   UCSC 
 
  220
 
  7
 
 27228
 
 tss
 
 0.826302
 
 0.7438367
 
 Not queried
 
  0.5633732
 
       NA
 
           NA
  
 
  
 10
 
 134119682
 
 134120029
 
  ENSEMBL   UCSC 
 
  348
 
  5
 
 4861
 
 gene;island
 
 0.927743
 
 0.9602342
 
 Not queried
 
  0.5633559
 
       NA
 
           NA
  
 
  
 1
 
  53392492
 
  53393034
 
  ENSEMBL   UCSC 
 
  543
 
  9
 
 3277
 
 island
 
 0.030829
 
 0.0473061
 
 Not queried
 
  0.5632826
 
       NA
 
           NA
  
 
  
 13
 
  25745946
 
  25746644
 
  ENSEMBL   UCSC 
 
  699
 
 11
 
 11350
 
 tss
 
 0.164223
 
 0.1259348
 
 Not queried
 
  0.5631769
 
       NA
 
           NA
  
 
  
 1
 
 183155366
 
 183155644
 
  ENSEMBL   UCSC 
 
  279
 
  4
 
 914
 
 gene
 
 0.489089
 
 0.5124013
 
 Not queried
 
  0.5630529
 
       NA
 
           NA
  
 
  
 19
 
   5243839
 
   5244269
 
  ENSEMBL   UCSC 
 
  431
 
  5
 
 19747
 
 gene;island
 
 0.857575
 
 0.9065631
 
 Not queried
 
  0.5629835
 
       NA
 
           NA
  
 
  
 7
 
  81240257
 
  81240667
 
  ENSEMBL   UCSC 
 
  411
 
  5
 
 38623
 
 island
 
 0.723575
 
 0.8171889
 
 Not queried
 
  0.5627560
 
       NA
 
           NA
  
 
  
 13
 
 100607772
 
 100608447
 
  ENSEMBL   UCSC 
 
  676
 
  5
 
 11844
 
 island
 
 0.130445
 
 0.1804474
 
 Not queried
 
  0.5626550
 
       NA
 
           NA
  
 
  
 2
 
 154335014
 
 154335250
 
  ENSEMBL   UCSC 
 
  237
 
  4
 
 22899
 
 gene
 
 0.045325
 
 0.0685009
 
 Not queried
 
  0.5625628
 
       NA
 
           NA
  
 
  
 1
 
 115322961
 
 115323269
 
  ENSEMBL   UCSC 
 
  309
 
  4
 
 702
 
 gene
 
 0.004544
 
 0.0054933
 
 Not queried
 
  0.5625548
 
       NA
 
           NA
  
 
  
 2
 
 206547064
 
 206547237
 
  ENSEMBL   UCSC 
 
  174
 
  5
 
 25028
 
 island
 
 0.017979
 
 0.0164746
 
 Not queried
 
  0.5625029
 
       NA
 
           NA
  
 
  
 17
 
  48585858
 
  48586147
 
  ENSEMBL   UCSC 
 
  290
 
  4
 
 16688
 
 gene;island
 
 0.030899
 
 0.0225019
 
 Not queried
 
  0.5624919
 
       NA
 
           NA
  
 
  
 7
 
  27225058
 
  27225897
 
  ENSEMBL   UCSC 
 
  840
 
 23
 
 36942
 
 gene
 
 0.100908
 
 0.1004160
 
 Not queried
 
  0.5624351
 
       NA
 
           NA
  
 
  
 7
 
 107300939
 
 107301048
 
  ENSEMBL   UCSC 
 
  110
 
  5
 
 37829
 
 tss
 
 0.016802
 
 0.0293265
 
 Not queried
 
  0.5624214
 
       NA
 
           NA
  
 
  
 12
 
  66217779
 
  66218057
 
  ENSEMBL   UCSC 
 
  279
 
  4
 
 9205
 
 gene;tss;island
 
 0.038695
 
 0.0185111
 
 Not queried
 
  0.5623855
 
       NA
 
           NA
  
 
  
 14
 
  89878562
 
  89878733
 
  ENSEMBL   UCSC 
 
  172
 
  6
 
 12170
 
 gene;island
 
 0.964592
 
 0.9397097
 
 Not queried
 
  0.5623286
 
       NA
 
           NA
  
 
  
 3
 
 160283376
 
 160283695
 
  ENSEMBL   UCSC 
 
  320
 
  6
 
 28695
 
 tss
 
 0.005028
 
 0.0135499
 
 Not queried
 
  0.5622714
 
       NA
 
           NA
  
 
  
 3
 
 134369339
 
 134369828
 
  ENSEMBL   UCSC 
 
  490
 
  4
 
 27957
 
 gene
 
 0.251492
 
 0.2983303
 
 Not queried
 
  0.5620788
 
       NA
 
           NA
  
 
  
 14
 
  89029552
 
  89029605
 
  ENSEMBL   UCSC 
 
   54
 
  5
 
 12546
 
 tss
 
 0.006163
 
 0.0095189
 
 Not queried
 
  0.5620406
 
       NA
 
           NA
  
 
  
 14
 
  70655803
 
  70656116
 
  ENSEMBL   UCSC 
 
  314
 
  8
 
 12485
 
 tss
 
 0.009067
 
 0.0151553
 
 Not queried
 
  0.5620141
 
       NA
 
           NA
  
 
  
 3
 
 159942832
 
 159943320
 
  ENSEMBL   UCSC 
 
  489
 
  8
 
 28690
 
 tss
 
 0.008623
 
 0.0071191
 
 Not queried
 
  0.5619310
 
       NA
 
           NA
  
 
  
 17
 
  79133095
 
  79133388
 
  ENSEMBL   UCSC 
 
  294
 
  4
 
 16900
 
 gene;island
 
 0.681402
 
 0.8546024
 
 Not queried
 
  0.5618470
 
       NA
 
           NA
  
 
  
 12
 
  85306323
 
  85307152
 
  ENSEMBL   UCSC 
 
  830
 
 13
 
 10656
 
 island
 
 0.034736
 
 0.0718333
 
 Not queried
 
  0.5617892
 
       NA
 
           NA
  
 
  
 12
 
  54331892
 
  54332530
 
  ENSEMBL   UCSC 
 
  639
 
 10
 
 9748
 
 tss
 
 0.107857
 
 0.0949450
 
 Not queried
 
  0.5617378
 
       NA
 
           NA
  
 
  
 12
 
  54331892
 
  54332695
 
  ENSEMBL   UCSC 
 
  804
 
 12
 
 10478
 
 island
 
 0.107857
 
 0.0987696
 
 Not queried
 
  0.5617378
 
       NA
 
           NA
  
 
  
 6
 
   5996893
 
   5997375
 
  ENSEMBL   UCSC 
 
  483
 
  6
 
 35404
 
 island
 
 0.010900
 
 0.0095812
 
 Not queried
 
  0.5617253
 
       NA
 
           NA
  
 
  
 3
 
 179065561
 
 179065847
 
  ENSEMBL   UCSC 
 
  287
 
  5
 
 28037
 
 gene
 
 0.007387
 
 0.0073348
 
 Not queried
 
  0.5617177
 
       NA
 
           NA
  
 
  
 11
 
  63655748
 
  63656090
 
  ENSEMBL   UCSC 
 
  343
 
  5
 
 6655
 
 gene
 
 0.009410
 
 0.0177958
 
 Not queried
 
  0.5617158
 
       NA
 
           NA
  
 
  
 20
 
  20032981
 
  20033184
 
  ENSEMBL   UCSC 
 
  204
 
  7
 
 25371
 
 gene;island
 
 0.003156
 
 0.0027401
 
 Not queried
 
  0.5616861
 
       NA
 
           NA
  
 
  
 16
 
   9185438
 
   9185541
 
  ENSEMBL   UCSC 
 
  104
 
  5
 
 15691
 
 island
 
 0.009661
 
 0.0089723
 
 Not queried
 
  0.5616504
 
       NA
 
           NA
  
 
  
 6
 
 166666838
 
 166667648
 
  ENSEMBL   UCSC 
 
  811
 
  7
 
 36628
 
 island
 
 0.013837
 
 0.0216117
 
 Not queried
 
  0.5616501
 
       NA
 
           NA
  
 
  
 13
 
  36050844
 
  36051073
 
  ENSEMBL   UCSC 
 
  230
 
  7
 
 11390
 
 tss
 
 0.009500
 
 0.0091596
 
 Not queried
 
  0.5616479
 
       NA
 
           NA
  
 
  
 3
 
 114102565
 
 114102863
 
  ENSEMBL   UCSC 
 
  299
 
  5
 
 27900
 
 gene;tss
 
 0.950380
 
 0.9740389
 
 Not queried
 
  0.5615168
 
       NA
 
           NA
  
 
  
 19
 
  49403890
 
  49404253
 
  ENSEMBL   UCSC 
 
  364
 
  4
 
 22241
 
 island
 
 0.141623
 
 0.0297388
 
 Not queried
 
  0.5613997
 
       NA
 
           NA
  
 
  
 2
 
  25194829
 
  25195213
 
  ENSEMBL   UCSC 
 
  385
 
  6
 
 23229
 
 tss;island
 
 0.007157
 
 0.0052948
 
 Not queried
 
  0.5612946
 
       NA
 
           NA
  
 
  
 11
 
  18415641
 
  18415927
 
  ENSEMBL   UCSC 
 
  287
 
  8
 
 7183
 
 tss
 
 0.011403
 
 0.0159725
 
 Not queried
 
  0.5612647
 
       NA
 
           NA
  
 
  
 4
 
 122791694
 
 122792031
 
  ENSEMBL   UCSC 
 
  338
 
  9
 
 30576
 
 tss;island
 
 0.011767
 
 0.0083897
 
 Not queried
 
  0.5610465
 
       NA
 
           NA
  
 
  
 14
 
  95236539
 
  95236615
 
  ENSEMBL   UCSC 
 
   77
 
  4
 
 12585
 
 tss;island
 
 0.010646
 
 0.0118852
 
 Not queried
 
  0.5610174
 
       NA
 
           NA
  
 
  
 6
 
  43337670
 
  43337791
 
  ENSEMBL   UCSC 
 
  122
 
  5
 
 34997
 
 tss;island
 
 0.002608
 
 0.0072947
 
 Not queried
 
  0.5610162
 
       NA
 
           NA
  
 
  
 4
 
 176986621
 
 176987577
 
  ENSEMBL   UCSC 
 
  957
 
 14
 
 31440
 
 island
 
 0.010180
 
 0.0045092
 
 Not queried
 
  0.5609394
 
       NA
 
           NA
  
 
  
 17
 
  41923945
 
  41924501
 
  ENSEMBL   UCSC 
 
  557
 
 10
 
 17395
 
 tss
 
 0.025823
 
 0.0460703
 
 Not queried
 
  0.5609228
 
       NA
 
           NA
  
 
  
 14
 
  60558421
 
  60558592
 
  ENSEMBL   UCSC 
 
  172
 
  5
 
 12435
 
 tss
 
 0.009427
 
 0.0046092
 
 Not queried
 
  0.5608935
 
       NA
 
           NA
  
 
  
 20
 
  44044727
 
  44044875
 
  ENSEMBL   UCSC 
 
  149
 
  4
 
 25430
 
 gene
 
 0.015202
 
 0.0174757
 
 Not queried
 
  0.5608668
 
       NA
 
           NA
  
 
  
 5
 
  43066798
 
  43067144
 
  ENSEMBL   UCSC 
 
  347
 
  4
 
 32771
 
 island
 
 0.007579
 
 0.0053995
 
 Not queried
 
  0.5607654
 
       NA
 
           NA
  
 
  
 7
 
  96339249
 
  96339613
 
  ENSEMBL   UCSC 
 
  365
 
  8
 
 37732
 
 tss
 
 0.032593
 
 0.0276802
 
 Not queried
 
  0.5607615
 
       NA
 
           NA
  
 
  
 1
 
 229542868
 
 229543254
 
  ENSEMBL   UCSC 
 
  387
 
  4
 
 4308
 
 island
 
 0.042842
 
 0.0256129
 
 Not queried
 
  0.5607017
 
       NA
 
           NA
  
 
  
 15
 
  41624995
 
  41625261
 
  ENSEMBL   UCSC 
 
  267
 
  6
 
 13303
 
 gene
 
 0.005544
 
 0.0035388
 
 Not queried
 
  0.5606914
 
       NA
 
           NA
  
 
  
 8
 
 103424275
 
 103424494
 
  ENSEMBL   UCSC 
 
  220
 
  4
 
 39385
 
 gene;island
 
 0.008567
 
 0.0112959
 
 Not queried
 
  0.5606830
 
       NA
 
           NA
  
 
  
 2
 
 182321354
 
 182321496
 
  ENSEMBL   UCSC 
 
  143
 
  4
 
 23761
 
 tss;island
 
 0.006552
 
 0.0031734
 
 Not queried
 
  0.5606413
 
       NA
 
           NA
  
 
  
 3
 
 169530268
 
 169530621
 
  ENSEMBL   UCSC 
 
  354
 
  6
 
 29650
 
 island
 
 0.019432
 
 0.0170413
 
 Not queried
 
  0.5605948
 
       NA
 
           NA
  
 
  
 8
 
  33370659
 
  33370902
 
  ENSEMBL   UCSC 
 
  244
 
  6
 
 39640
 
 tss
 
 0.012721
 
 0.0068435
 
 Not queried
 
  0.5604346
 
       NA
 
           NA
  
 
  
 10
 
 105677453
 
 105677985
 
  ENSEMBL   UCSC 
 
  533
 
  5
 
 4747
 
 gene
 
 0.009581
 
 0.0057282
 
 Not queried
 
  0.5603498
 
       NA
 
           NA
  
 
  
 8
 
   1830682
 
   1830965
 
  ENSEMBL   UCSC 
 
  284
 
  5
 
 39182
 
 gene;island
 
 0.560206
 
 0.6525318
 
 Not queried
 
  0.5602754
 
       NA
 
           NA
  
 
  
 9
 
  72288069
 
  72288179
 
  ENSEMBL   UCSC 
 
  111
 
  4
 
 40814
 
 tss;island
 
 0.011363
 
 0.0075593
 
 Not queried
 
  0.5601568
 
       NA
 
           NA
  
 
  
 11
 
  64329383
 
  64329824
 
  ENSEMBL   UCSC 
 
  442
 
  6
 
 6688
 
 gene;island
 
 0.459494
 
 0.6184538
 
 Not queried
 
  0.5599264
 
       NA
 
           NA
  
 
  
 6
 
  91004932
 
  91005300
 
  ENSEMBL   UCSC 
 
  369
 
  4
 
 34417
 
 gene;island
 
 0.019372
 
 0.0143755
 
 Not queried
 
  0.5598988
 
       NA
 
           NA
  
 
  
 17
 
  28804287
 
  28804354
 
  ENSEMBL   UCSC 
 
   68
 
  4
 
 17241
 
 tss;island
 
 0.010045
 
 0.0066770
 
 Not queried
 
  0.5598315
 
       NA
 
           NA
  
 
  
 19
 
  17861850
 
  17862210
 
  ENSEMBL   UCSC 
 
  361
 
  6
 
 20599
 
 tss
 
 0.001163
 
 0.0011935
 
 Not queried
 
  0.5598056
 
       NA
 
           NA
  
 
  
 6
 
  84233917
 
  84234463
 
  ENSEMBL   UCSC 
 
  547
 
  8
 
 34403
 
 gene
 
 0.595957
 
 0.7833732
 
 Not queried
 
  0.5597313
 
       NA
 
           NA
  
 
  
 15
 
  89631044
 
  89631334
 
  ENSEMBL   UCSC 
 
  291
 
  5
 
 13883
 
 tss;island
 
 0.028830
 
 0.0216028
 
 Not queried
 
  0.5595693
 
       NA
 
           NA
  
 
  
 17
 
  42297002
 
  42297085
 
  ENSEMBL   UCSC 
 
   84
 
  5
 
 16612
 
 gene;tss;island
 
 0.010191
 
 0.0075377
 
 Not queried
 
  0.5594909
 
       NA
 
           NA
  
 
  
 1
 
   8878173
 
   8878227
 
  ENSEMBL   UCSC 
 
   55
 
  4
 
 1223
 
 tss;island
 
 0.004223
 
 0.0029359
 
 Not queried
 
  0.5594732
 
       NA
 
           NA
  
 
  
 7
 
  84569142
 
  84569518
 
  ENSEMBL   UCSC 
 
  377
 
  4
 
 38627
 
 island
 
 0.288002
 
 0.3899171
 
 Not queried
 
  0.5594667
 
       NA
 
           NA
  
 
  
 17
 
  72209469
 
  72209802
 
  ENSEMBL   UCSC 
 
  334
 
  7
 
 17620
 
 tss;island
 
 0.006125
 
 0.0075905
 
 Not queried
 
  0.5594428
 
       NA
 
           NA
  
 
  
 1
 
 203595048
 
 203595283
 
  ENSEMBL   UCSC 
 
  236
 
  4
 
 2194
 
 tss;island
 
 0.015607
 
 0.0268000
 
 Not queried
 
  0.5592487
 
       NA
 
           NA
  
 
  
 10
 
 102505266
 
 102505483
 
  ENSEMBL   UCSC 
 
  218
 
  7
 
 6034
 
 island
 
 0.011722
 
 0.0064354
 
 Not queried
 
  0.5592312
 
       NA
 
           NA
  
 
  
 1
 
 154530682
 
 154530958
 
  ENSEMBL   UCSC 
 
  277
 
  5
 
 780
 
 gene;island
 
 0.003265
 
 0.0072378
 
 Not queried
 
  0.5591646
 
       NA
 
           NA
  
 
  
 10
 
   5454184
 
   5454480
 
  ENSEMBL   UCSC 
 
  297
 
  7
 
 4923
 
 tss
 
 0.009630
 
 0.0058900
 
 Not queried
 
  0.5591432
 
       NA
 
           NA
  
 
  
 7
 
   4875911
 
   4876228
 
  ENSEMBL   UCSC 
 
  318
 
  4
 
 36861
 
 gene;island
 
 0.805790
 
 0.8552838
 
 Not queried
 
  0.5591187
 
       NA
 
           NA
  
 
  
 19
 
  50412816
 
  50413083
 
  ENSEMBL   UCSC 
 
  268
 
  7
 
 20169
 
 gene
 
 0.926497
 
 0.9435707
 
 Not queried
 
  0.5589696
 
       NA
 
           NA
  
 
  
 2
 
  85108351
 
  85108429
 
  ENSEMBL   UCSC 
 
   79
 
  5
 
 23476
 
 tss
 
 0.006538
 
 0.0094520
 
 Not queried
 
  0.5589208
 
       NA
 
           NA
  
 
  
 16
 
  71843015
 
  71843100
 
  ENSEMBL   UCSC 
 
   86
 
  4
 
 15350
 
 tss;island
 
 0.002028
 
 0.0025912
 
 Not queried
 
  0.5588611
 
       NA
 
           NA
  
 
  
 16
 
  67225915
 
  67226033
 
  ENSEMBL   UCSC 
 
  119
 
  6
 
 15297
 
 tss
 
 0.007034
 
 0.0037940
 
 Not queried
 
  0.5588509
 
       NA
 
           NA
  
 
  
 16
 
   4524103
 
   4524711
 
  ENSEMBL   UCSC 
 
  609
 
  7
 
 15069
 
 tss
 
 0.020468
 
 0.0145445
 
 Not queried
 
  0.5588277
 
       NA
 
           NA
  
 
  
 13
 
 111290667
 
 111290952
 
  ENSEMBL   UCSC 
 
  286
 
  5
 
 11235
 
 gene;island
 
 0.976987
 
 0.9794010
 
 Not queried
 
  0.5587589
 
       NA
 
           NA
  
 
  
 17
 
  40829133
 
  40829241
 
  ENSEMBL   UCSC 
 
  109
 
  6
 
 17370
 
 tss
 
 0.004706
 
 0.0109014
 
 Not queried
 
  0.5587513
 
       NA
 
           NA
  
 
  
 3
 
 160879441
 
 160879721
 
  ENSEMBL   UCSC 
 
  281
 
  5
 
 29636
 
 island
 
 0.900854
 
 0.9491630
 
 Not queried
 
  0.5587373
 
       NA
 
           NA
  
 
  
 1
 
  24882153
 
  24882451
 
  ENSEMBL   UCSC 
 
  299
 
  5
 
 1361
 
 tss
 
 0.075792
 
 0.0551215
 
 Not queried
 
  0.5587080
 
       NA
 
           NA
  
 
  
 11
 
    506809
 
    507555
 
  ENSEMBL   UCSC 
 
  747
 
 16
 
 7857
 
 island
 
 0.005341
 
 0.0030713
 
 Not queried
 
  0.5586877
 
       NA
 
           NA
  
 
  
 4
 
   1807118
 
   1808029
 
  ENSEMBL   UCSC 
 
  912
 
  8
 
 29877
 
 gene;island
 
 0.658480
 
 0.7627498
 
 Not queried
 
  0.5585904
 
       NA
 
           NA
  
 
  
 12
 
 118453982
 
 118454420
 
  ENSEMBL   UCSC 
 
  439
 
  9
 
 10046
 
 tss
 
 0.035149
 
 0.0354192
 
 Not queried
 
  0.5585691
 
       NA
 
           NA
  
 
  
 20
 
  62369445
 
  62369605
 
  ENSEMBL   UCSC 
 
  161
 
  4
 
 25511
 
 gene;island
 
 0.017548
 
 0.0213867
 
 Not queried
 
  0.5585443
 
       NA
 
           NA
  
 
  
 2
 
  70781256
 
  70781325
 
  ENSEMBL   UCSC 
 
   70
 
  6
 
 23419
 
 tss
 
 0.018822
 
 0.0081603
 
 Not queried
 
  0.5584585
 
       NA
 
           NA
  
 
  
 16
 
  66584061
 
  66584358
 
  ENSEMBL   UCSC 
 
  298
 
  4
 
 15987
 
 island
 
 0.242041
 
 0.5155614
 
 Not queried
 
  0.5583850
 
       NA
 
           NA
  
 
  
 8
 
  55014641
 
  55014888
 
  ENSEMBL   UCSC 
 
  248
 
  7
 
 39704
 
 tss;island
 
 0.019356
 
 0.0138091
 
 Not queried
 
  0.5583727
 
       NA
 
           NA
  
 
  
 19
 
  11849362
 
  11850013
 
  ENSEMBL   UCSC 
 
  652
 
 10
 
 21582
 
 island
 
 0.007639
 
 0.0062142
 
 Not queried
 
  0.5583160
 
       NA
 
           NA
  
 
  
 4
 
 168155510
 
 168156080
 
  ENSEMBL   UCSC 
 
  571
 
  6
 
 31411
 
 island
 
 0.041249
 
 0.0541348
 
 Not queried
 
  0.5582533
 
       NA
 
           NA
  
 
  
 15
 
  25199028
 
  25199270
 
  ENSEMBL   UCSC 
 
  243
 
  4
 
 13257
 
 gene;tss;island
 
 0.766761
 
 0.9070881
 
 Not queried
 
  0.5582450
 
       NA
 
           NA
  
 
  
 17
 
   4900630
 
   4900769
 
  ENSEMBL   UCSC 
 
  140
 
  4
 
 16363
 
 gene;tss;island
 
 0.011942
 
 0.0114523
 
 Not queried
 
  0.5581609
 
       NA
 
           NA
  
 
  
 19
 
  21860753
 
  21860916
 
  ENSEMBL   UCSC 
 
  164
 
  4
 
 21837
 
 island
 
 0.409154
 
 0.3065803
 
 Not queried
 
  0.5580558
 
       NA
 
           NA
  
 
  
 2
 
  23913769
 
  23914666
 
  ENSEMBL   UCSC 
 
  898
 
  8
 
 22597
 
 gene;island
 
 0.971055
 
 0.9568412
 
 Not queried
 
  0.5580276
 
       NA
 
           NA
  
 
  
 6
 
  32050905
 
  32051108
 
  ENSEMBL   UCSC 
 
  204
 
  5
 
 34037
 
 gene;island
 
 0.488260
 
 0.5938237
 
 Not queried
 
  0.5580019
 
       NA
 
           NA
  
 
  
 6
 
  42847125
 
  42847646
 
  ENSEMBL   UCSC 
 
  522
 
 10
 
 34982
 
 tss
 
 0.006189
 
 0.0014262
 
 Not queried
 
  0.5579759
 
       NA
 
           NA
  
 
  
 22
 
  45705512
 
  45706060
 
  ENSEMBL   UCSC 
 
  549
 
  9
 
 26902
 
 gene;island
 
 0.003736
 
 0.0046817
 
 Not queried
 
  0.5578947
 
       NA
 
           NA
  
 
  
 19
 
    925787
 
    925914
 
  ENSEMBL   UCSC 
 
  128
 
  4
 
 20286
 
 tss;island
 
 0.008648
 
 0.0097047
 
 Not queried
 
  0.5578573
 
       NA
 
           NA
  
 
  
 19
 
   2945000
 
   2945201
 
  ENSEMBL   UCSC 
 
  202
 
  7
 
 20336
 
 tss
 
 0.012261
 
 0.0098810
 
 Not queried
 
  0.5577718
 
       NA
 
           NA
  
 
  
 22
 
  21995943
 
  21996426
 
  ENSEMBL   UCSC 
 
  484
 
  8
 
 26997
 
 tss;island
 
 0.006920
 
 0.0091545
 
 Not queried
 
  0.5577458
 
       NA
 
           NA
  
 
  
 5
 
  56110761
 
  56110881
 
  ENSEMBL   UCSC 
 
  121
 
  5
 
 32072
 
 tss
 
 0.005183
 
 0.0027324
 
 Not queried
 
  0.5577354
 
       NA
 
           NA
  
 
  
 6
 
  33142936
 
  33143356
 
  ENSEMBL   UCSC 
 
  421
 
  7
 
 34203
 
 gene
 
 0.418067
 
 0.5554817
 
 Not queried
 
  0.5577000
 
       NA
 
           NA
  
 
  
 1
 
 171454351
 
 171454651
 
  ENSEMBL   UCSC 
 
  301
 
  4
 
 2090
 
 tss
 
 0.002776
 
 0.0066640
 
 Not queried
 
  0.5576728
 
       NA
 
           NA
  
 
  
 13
 
  73301900
 
  73302139
 
  ENSEMBL   UCSC 
 
  240
 
 10
 
 11474
 
 tss;island
 
 0.004612
 
 0.0098243
 
 Not queried
 
  0.5576001
 
       NA
 
           NA
  
 
  
 1
 
 235667348
 
 235667680
 
  ENSEMBL   UCSC 
 
  333
 
  4
 
 1090
 
 gene;island
 
 0.006033
 
 0.0051557
 
 Not queried
 
  0.5575831
 
       NA
 
           NA
  
 
  
 1
 
  40943161
 
  40943386
 
  ENSEMBL   UCSC 
 
  226
 
  4
 
 3124
 
 island
 
 0.013184
 
 0.0237713
 
 Not queried
 
  0.5575304
 
       NA
 
           NA
  
 
  
 11
 
  82867879
 
  82868133
 
  ENSEMBL   UCSC 
 
  255
 
  8
 
 7598
 
 tss
 
 0.003684
 
 0.0077652
 
 Not queried
 
  0.5574381
 
       NA
 
           NA
  
 
  
 2
 
 122042939
 
 122043074
 
  ENSEMBL   UCSC 
 
  136
 
  5
 
 23601
 
 tss;island
 
 0.102984
 
 0.0575397
 
 Not queried
 
  0.5573324
 
       NA
 
           NA
  
 
  
 18
 
  74325812
 
  74326081
 
  ENSEMBL   UCSC 
 
  270
 
  4
 
 19580
 
 island
 
 0.810829
 
 0.9176801
 
 Not queried
 
  0.5572928
 
       NA
 
           NA
  
 
  
 19
 
   3721303
 
   3721572
 
  ENSEMBL   UCSC 
 
  270
 
  4
 
 21345
 
 island
 
 0.090248
 
 0.1172429
 
 Not queried
 
  0.5571514
 
       NA
 
           NA
  
 
  
 6
 
  26027513
 
  26027625
 
  ENSEMBL   UCSC 
 
  113
 
  6
 
 34680
 
 tss
 
 0.001361
 
 0.0016022
 
 Not queried
 
  0.5571010
 
       NA
 
           NA
  
 
  
 8
 
  39695788
 
  39696062
 
  ENSEMBL   UCSC 
 
  275
 
  6
 
 39665
 
 tss
 
 0.660742
 
 0.8447910
 
 Not queried
 
  0.5570696
 
       NA
 
           NA
  
 
  
 2
 
 148602278
 
 148602993
 
  ENSEMBL   UCSC 
 
  716
 
  9
 
 24773
 
 island
 
 0.021836
 
 0.0183580
 
 Not queried
 
  0.5570629
 
       NA
 
           NA
  
 
  
 1
 
 151031286
 
 151031323
 
  ENSEMBL   UCSC 
 
   38
 
  4
 
 747
 
 gene;tss;island
 
 0.006339
 
 0.0049101
 
 Not queried
 
  0.5570201
 
       NA
 
           NA
  
 
  
 16
 
   1604980
 
   1605339
 
  ENSEMBL   UCSC 
 
  360
 
  4
 
 14577
 
 gene;island
 
 0.947388
 
 0.9663731
 
 Not queried
 
  0.5569919
 
       NA
 
           NA
  
 
  
 4
 
 140656749
 
 140657110
 
  ENSEMBL   UCSC 
 
  362
 
  4
 
 30133
 
 gene;island
 
 0.011018
 
 0.0109235
 
 Not queried
 
  0.5569448
 
       NA
 
           NA
  
 
  
 1
 
  54411759
 
  54412009
 
  ENSEMBL   UCSC 
 
  251
 
  6
 
 1642
 
 tss;island
 
 0.007376
 
 0.0084826
 
 Not queried
 
  0.5569315
 
       NA
 
           NA
  
 
  
 6
 
  55443757
 
  55444488
 
  ENSEMBL   UCSC 
 
  732
 
 10
 
 36218
 
 island
 
 0.147387
 
 0.2385949
 
 Not queried
 
  0.5568877
 
       NA
 
           NA
  
 
  
 1
 
 207818395
 
 207818449
 
  ENSEMBL   UCSC 
 
   55
 
  4
 
 2224
 
 tss
 
 0.049042
 
 0.0133172
 
 Not queried
 
  0.5568326
 
       NA
 
           NA
  
 
  
 10
 
 102106080
 
 102106757
 
  ENSEMBL   UCSC 
 
  678
 
  8
 
 5292
 
 tss
 
 0.005133
 
 0.0039800
 
 Not queried
 
  0.5567365
 
       NA
 
           NA
  
 
  
 19
 
   2251588
 
   2252069
 
  ENSEMBL   UCSC 
 
  482
 
  5
 
 19683
 
 gene;island
 
 0.652199
 
 0.4920203
 
 Not queried
 
  0.5566973
 
       NA
 
           NA
  
 
  
 4
 
   2733734
 
   2733970
 
  ENSEMBL   UCSC 
 
  237
 
  4
 
 29891
 
 gene;island
 
 0.970975
 
 0.9662958
 
 Not queried
 
  0.5566284
 
       NA
 
           NA
  
 
  
 16
 
  30102919
 
  30103624
 
  ENSEMBL   UCSC 
 
  706
 
  7
 
 15822
 
 island
 
 0.018800
 
 0.0129286
 
 Not queried
 
  0.5565913
 
       NA
 
           NA
  
 
  
 14
 
 104569519
 
 104569653
 
  ENSEMBL   UCSC 
 
  135
 
  4
 
 12245
 
 gene;island
 
 0.406077
 
 0.4889653
 
 Not queried
 
  0.5563256
 
       NA
 
           NA
  
 
  
 6
 
  31623180
 
  31623318
 
  ENSEMBL   UCSC 
 
  139
 
  4
 
 34825
 
 tss;island
 
 0.957792
 
 0.7918163
 
 Not queried
 
  0.5562572
 
       NA
 
           NA
  
 
  
 19
 
   1104476
 
   1104646
 
  ENSEMBL   UCSC 
 
  171
 
  6
 
 19642
 
 gene;tss;island
 
 0.005304
 
 0.0076608
 
 Not queried
 
  0.5562517
 
       NA
 
           NA
  
 
  
 7
 
   2738728
 
   2738961
 
  ENSEMBL   UCSC 
 
  234
 
  4
 
 36812
 
 gene;island
 
 0.002620
 
 0.0038604
 
 Not queried
 
  0.5562162
 
       NA
 
           NA
  
 
  
 22
 
  20236751
 
  20236856
 
  ENSEMBL   UCSC 
 
  106
 
  4
 
 26797
 
 gene;tss
 
 0.618799
 
 0.7084560
 
 Not queried
 
  0.5561807
 
       NA
 
           NA
  
 
  
 5
 
 140018644
 
 140019523
 
  ENSEMBL   UCSC 
 
  880
 
 15
 
 33191
 
 island
 
 0.010604
 
 0.0118908
 
 Not queried
 
  0.5561634
 
       NA
 
           NA
  
 
  
 17
 
  25620944
 
  25621101
 
  ENSEMBL   UCSC 
 
  158
 
  7
 
 17204
 
 tss
 
 0.007302
 
 0.0024424
 
 Not queried
 
  0.5561186
 
       NA
 
           NA
  
 
  
 21
 
  43187083
 
  43187372
 
  ENSEMBL   UCSC 
 
  290
 
  4
 
 26692
 
 island
 
 0.029005
 
 0.0240301
 
 Not queried
 
  0.5561120
 
       NA
 
           NA
  
 
  
 15
 
  99512700
 
  99512842
 
  ENSEMBL   UCSC 
 
  143
 
  4
 
 13500
 
 gene;island
 
 0.620916
 
 0.7656075
 
 Not queried
 
  0.5561105
 
       NA
 
           NA
  
 
  
 2
 
 159824855
 
 159825303
 
  ENSEMBL   UCSC 
 
  449
 
  6
 
 24807
 
 island
 
 0.016971
 
 0.0143019
 
 Not queried
 
  0.5560425
 
       NA
 
           NA
  
 
  
 6
 
 135376137
 
 135376255
 
  ENSEMBL   UCSC 
 
  119
 
  6
 
 35236
 
 tss
 
 0.009410
 
 0.0060538
 
 Not queried
 
  0.5559267
 
       NA
 
           NA
  
 
  
 5
 
  77591420
 
  77591744
 
  ENSEMBL   UCSC 
 
  325
 
  4
 
 32141
 
 tss;island
 
 0.017326
 
 0.0260066
 
 Not queried
 
  0.5559197
 
       NA
 
           NA
  
 
  
 17
 
  25620944
 
  25621539
 
  ENSEMBL   UCSC 
 
  596
 
 10
 
 18133
 
 island
 
 0.009641
 
 0.0054451
 
 Not queried
 
  0.5558646
 
       NA
 
           NA
  
 
  
 2
 
  98703324
 
  98703475
 
  ENSEMBL   UCSC 
 
  152
 
  7
 
 23534
 
 tss;island
 
 0.004510
 
 0.0141347
 
 Not queried
 
  0.5558380
 
       NA
 
           NA
  
 
  
 7
 
   1062669
 
   1062965
 
  ENSEMBL   UCSC 
 
  297
 
  8
 
 37393
 
 tss
 
 0.136022
 
 0.2466158
 
 Not queried
 
  0.5558186
 
       NA
 
           NA
  
 
  
 19
 
  41769929
 
  41770024
 
  ENSEMBL   UCSC 
 
   96
 
  6
 
 20804
 
 tss
 
 0.006244
 
 0.0102083
 
 Not queried
 
  0.5556962
 
       NA
 
           NA
  
 
  
 3
 
  64008736
 
  64008893
 
  ENSEMBL   UCSC 
 
  158
 
  4
 
 27848
 
 gene;island
 
 0.028921
 
 0.1011241
 
 Not queried
 
  0.5556386
 
       NA
 
           NA
  
 
  
 2
 
 139259466
 
 139259770
 
  ENSEMBL   UCSC 
 
  305
 
  4
 
 22887
 
 gene;island
 
 0.008733
 
 0.0064199
 
 Not queried
 
  0.5556330
 
       NA
 
           NA
  
 
  
 6
 
 150326316
 
 150326776
 
  ENSEMBL   UCSC 
 
  461
 
 10
 
 35293
 
 tss;island
 
 0.039944
 
 0.0574578
 
 Not queried
 
  0.5556090
 
       NA
 
           NA
  
 
  
 3
 
 123699145
 
 123699389
 
  ENSEMBL   UCSC 
 
  245
 
  4
 
 27920
 
 gene
 
 0.311068
 
 0.2146770
 
 Not queried
 
  0.5555539
 
       NA
 
           NA
  
 
  
 2
 
  85838641
 
  85839143
 
  ENSEMBL   UCSC 
 
  503
 
  4
 
 22764
 
 gene
 
 0.017772
 
 0.0101873
 
 Not queried
 
  0.5555342
 
       NA
 
           NA
  
 
  
 14
 
  91007720
 
  91008005
 
  ENSEMBL   UCSC 
 
  286
 
  5
 
 12175
 
 gene;island
 
 0.767085
 
 0.6608914
 
 Not queried
 
  0.5554772
 
       NA
 
           NA
  
 
  
 15
 
  75918608
 
  75918757
 
  ENSEMBL   UCSC 
 
  150
 
  4
 
 13815
 
 tss;island
 
 0.033691
 
 0.0408459
 
 Not queried
 
  0.5553837
 
       NA
 
           NA
  
 
  
 1
 
 161008811
 
 161008977
 
  ENSEMBL   UCSC 
 
  167
 
  4
 
 2042
 
 tss
 
 0.007016
 
 0.0032539
 
 Not queried
 
  0.5553608
 
       NA
 
           NA
  
 
  
 2
 
 216176467
 
 216176660
 
  ENSEMBL   UCSC 
 
  194
 
  6
 
 23862
 
 tss
 
 0.007304
 
 0.0064364
 
 Not queried
 
  0.5552572
 
       NA
 
           NA
  
 
  
 16
 
  88262974
 
  88263263
 
  ENSEMBL   UCSC 
 
  290
 
  4
 
 16196
 
 island
 
 0.852682
 
 0.8382654
 
 Not queried
 
  0.5552136
 
       NA
 
           NA
  
 
  
 5
 
 132148706
 
 132149076
 
  ENSEMBL   UCSC 
 
  371
 
  8
 
 33100
 
 island
 
 0.011552
 
 0.0400927
 
 Not queried
 
  0.5551631
 
       NA
 
           NA
  
 
  
 7
 
 150019955
 
 150020401
 
  ENSEMBL   UCSC 
 
  447
 
 10
 
 37270
 
 gene;island
 
 0.020313
 
 0.0118536
 
 Not queried
 
  0.5551396
 
       NA
 
           NA
  
 
  
 6
 
  26457928
 
  26458121
 
  ENSEMBL   UCSC 
 
  194
 
  5
 
 34706
 
 tss
 
 0.004428
 
 0.0023265
 
 Not queried
 
  0.5550019
 
       NA
 
           NA
  
 
  
 2
 
 201170790
 
 201170828
 
  ENSEMBL   UCSC 
 
   39
 
  5
 
 22978
 
 gene;tss;island
 
 0.025268
 
 0.0321783
 
 Not queried
 
  0.5549003
 
       NA
 
           NA
  
 
  
 19
 
  58326288
 
  58326618
 
  ENSEMBL   UCSC 
 
  331
 
  8
 
 21126
 
 tss
 
 0.027414
 
 0.0299014
 
 Not queried
 
  0.5548334
 
       NA
 
           NA
  
 
  
 18
 
   4454189
 
   4454548
 
  ENSEMBL   UCSC 
 
  360
 
  4
 
 19367
 
 island
 
 0.015500
 
 0.0447212
 
 Not queried
 
  0.5548193
 
       NA
 
           NA
  
 
  
 6
 
  88299842
 
  88300029
 
  ENSEMBL   UCSC 
 
  188
 
  5
 
 34411
 
 gene
 
 0.016375
 
 0.0102489
 
 Not queried
 
  0.5547443
 
       NA
 
           NA
  
 
  
 11
 
  17099234
 
  17099458
 
  ENSEMBL   UCSC 
 
  225
 
  5
 
 7172
 
 tss
 
 0.005167
 
 0.0053801
 
 Not queried
 
  0.5546196
 
       NA
 
           NA
  
 
  
 11
 
  17099044
 
  17099458
 
  ENSEMBL   UCSC 
 
  415
 
  7
 
 8094
 
 island
 
 0.006695
 
 0.0121287
 
 Not queried
 
  0.5546196
 
       NA
 
           NA
  
 
  
 4
 
 176986621
 
 176986950
 
  ENSEMBL   UCSC 
 
  330
 
  7
 
 30687
 
 tss
 
 0.005219
 
 0.0035353
 
 Not queried
 
  0.5545646
 
       NA
 
           NA
  
 
  
 6
 
  28778091
 
  28778676
 
  ENSEMBL   UCSC 
 
  586
 
  9
 
 35595
 
 island
 
 0.014662
 
 0.0204016
 
 Not queried
 
  0.5545620
 
       NA
 
           NA
  
 
  
 7
 
  19157193
 
  19157420
 
  ENSEMBL   UCSC 
 
  228
 
  6
 
 38283
 
 island
 
 0.011905
 
 0.0113636
 
 Not queried
 
  0.5544943
 
       NA
 
           NA
  
 
  
 13
 
  32889023
 
  32889539
 
  ENSEMBL   UCSC 
 
  517
 
  9
 
 11382
 
 tss;island
 
 0.009207
 
 0.0140281
 
 Not queried
 
  0.5544729
 
       NA
 
           NA
  
 
  
 17
 
   1395864
 
   1396123
 
  ENSEMBL   UCSC 
 
  260
 
  5
 
 16987
 
 tss;island
 
 0.821287
 
 0.7609587
 
 Not queried
 
  0.5543965
 
       NA
 
           NA
  
 
  
 11
 
  62439250
 
  62439317
 
  ENSEMBL   UCSC 
 
   68
 
  6
 
 7355
 
 tss
 
 0.007648
 
 0.0057685
 
 Not queried
 
  0.5542806
 
       NA
 
           NA
  
 
  
 19
 
  35630106
 
  35630388
 
  ENSEMBL   UCSC 
 
  283
 
  6
 
 20699
 
 tss
 
 0.417028
 
 0.5269488
 
 Not queried
 
  0.5542129
 
       NA
 
           NA
  
 
  
 2
 
  73871782
 
  73871942
 
  ENSEMBL   UCSC 
 
  161
 
  5
 
 23442
 
 tss
 
 0.064066
 
 0.0281095
 
 Not queried
 
  0.5542092
 
       NA
 
           NA
  
 
  
 18
 
  44676656
 
  44676764
 
  ENSEMBL   UCSC 
 
  109
 
  4
 
 19107
 
 gene
 
 0.003166
 
 0.0031345
 
 Not queried
 
  0.5542042
 
       NA
 
           NA
  
 
  
 8
 
  67090581
 
  67090928
 
  ENSEMBL   UCSC 
 
  348
 
  6
 
 40352
 
 island
 
 0.712172
 
 0.8960725
 
 Not queried
 
  0.5541969
 
       NA
 
           NA
  
 
  
 6
 
  31927895
 
  31928395
 
  ENSEMBL   UCSC 
 
  501
 
  6
 
 34001
 
 gene;island
 
 0.915786
 
 0.9298278
 
 Not queried
 
  0.5540711
 
       NA
 
           NA
  
 
  
 11
 
  71163428
 
  71164349
 
  ENSEMBL   UCSC 
 
  922
 
 15
 
 8621
 
 island
 
 0.008164
 
 0.0048308
 
 Not queried
 
  0.5539605
 
       NA
 
           NA
  
 
  
 17
 
  58042029
 
  58042493
 
  ENSEMBL   UCSC 
 
  465
 
 12
 
 18615
 
 island
 
 0.007749
 
 0.0052168
 
 Not queried
 
  0.5539596
 
       NA
 
           NA
  
 
  
 17
 
  40811298
 
  40811580
 
  ENSEMBL   UCSC 
 
  283
 
  4
 
 16584
 
 gene
 
 0.018141
 
 0.0186307
 
 Not queried
 
  0.5539297
 
       NA
 
           NA
  
 
  
 1
 
  34090664
 
  34090941
 
  ENSEMBL   UCSC 
 
  278
 
  5
 
 405
 
 gene;island
 
 0.507228
 
 0.6123991
 
 Not queried
 
  0.5538431
 
       NA
 
           NA
  
 
  
 16
 
  89920501
 
  89921102
 
  ENSEMBL   UCSC 
 
  602
 
  6
 
 14951
 
 gene;island
 
 0.614101
 
 0.7837774
 
 Not queried
 
  0.5537757
 
       NA
 
           NA
  
 
  
 1
 
  53925109
 
  53925368
 
  ENSEMBL   UCSC 
 
  260
 
  4
 
 541
 
 gene
 
 0.750637
 
 0.8135063
 
 Not queried
 
  0.5535586
 
       NA
 
           NA
  
 
  
 1
 
  53924998
 
  53925368
 
  ENSEMBL   UCSC 
 
  371
 
  6
 
 3285
 
 island
 
 0.766147
 
 0.8823720
 
 Not queried
 
  0.5535586
 
       NA
 
           NA
  
 
  
 2
 
 198380137
 
 198380764
 
  ENSEMBL   UCSC 
 
  628
 
 15
 
 23800
 
 tss
 
 0.002108
 
 0.0013478
 
 Not queried
 
  0.5533767
 
       NA
 
           NA
  
 
  
 15
 
  78631878
 
  78632194
 
  ENSEMBL   UCSC 
 
  317
 
  8
 
 13834
 
 tss;island
 
 0.043020
 
 0.1089389
 
 Not queried
 
  0.5533151
 
       NA
 
           NA
  
 
  
 1
 
  78148360
 
  78148604
 
  ENSEMBL   UCSC 
 
  245
 
  7
 
 1706
 
 tss
 
 0.004306
 
 0.0055567
 
 Not queried
 
  0.5533088
 
       NA
 
           NA
  
 
  
 19
 
  41304898
 
  41304979
 
  ENSEMBL   UCSC 
 
   82
 
  4
 
 20801
 
 tss;island
 
 0.012362
 
 0.0177688
 
 Not queried
 
  0.5533081
 
       NA
 
           NA
  
 
  
 13
 
  41635698
 
  41636195
 
  ENSEMBL   UCSC 
 
  498
 
  4
 
 11145
 
 gene
 
 0.008001
 
 0.0071455
 
 Not queried
 
  0.5531399
 
       NA
 
           NA
  
 
  
 11
 
 113659927
 
 113659952
 
  ENSEMBL   UCSC 
 
   26
 
  4
 
 8818
 
 island
 
 0.909014
 
 0.8733837
 
 Not queried
 
  0.5528589
 
       NA
 
           NA
  
 
  
 1
 
 205011906
 
 205012139
 
  ENSEMBL   UCSC 
 
  234
 
  4
 
 2200
 
 tss
 
 0.149507
 
 0.2726567
 
 Not queried
 
  0.5527434
 
       NA
 
           NA
  
 
  
 19
 
  36390904
 
  36391588
 
  ENSEMBL   UCSC 
 
  685
 
  7
 
 21925
 
 island
 
 0.013016
 
 0.0182758
 
 Not queried
 
  0.5527016
 
       NA
 
           NA
  
 
  
 17
 
  77216389
 
  77216699
 
  ENSEMBL   UCSC 
 
  311
 
  4
 
 16838
 
 gene
 
 0.020237
 
 0.0931443
 
 Not queried
 
  0.5526212
 
       NA
 
           NA
  
 
  
 2
 
 170440636
 
 170440819
 
  ENSEMBL   UCSC 
 
  184
 
  6
 
 23703
 
 tss
 
 0.006313
 
 0.0627828
 
 Not queried
 
  0.5525078
 
       NA
 
           NA
  
 
  
 6
 
  29855110
 
  29855347
 
  ENSEMBL   UCSC 
 
  238
 
  7
 
 34756
 
 tss
 
 0.209822
 
 0.0355552
 
 Not queried
 
  0.5524551
 
       NA
 
           NA
  
 
  
 16
 
  33965255
 
  33965573
 
  ENSEMBL   UCSC 
 
  319
 
  4
 
 15889
 
 island
 
 0.528606
 
 0.6686686
 
 Not queried
 
  0.5524313
 
       NA
 
           NA
  
 
  
 11
 
  57117162
 
  57117666
 
  ENSEMBL   UCSC 
 
  505
 
  4
 
 6612
 
 gene;island
 
 0.048230
 
 0.3001877
 
 Not queried
 
  0.5524309
 
       NA
 
           NA
  
 
  
 10
 
  70863693
 
  70863740
 
  ENSEMBL   UCSC 
 
   48
 
  4
 
 4611
 
 gene
 
 0.944497
 
 0.9691947
 
 Not queried
 
  0.5523664
 
       NA
 
           NA
  
 
  
 20
 
   1448419
 
   1448645
 
  ENSEMBL   UCSC 
 
  227
 
  8
 
 25539
 
 tss;island
 
 0.155357
 
 0.1489432
 
 Not queried
 
  0.5523587
 
       NA
 
           NA
  
 
  
 11
 
 110583543
 
 110583662
 
  ENSEMBL   UCSC 
 
  120
 
  6
 
 7679
 
 tss
 
 0.066934
 
 0.0426960
 
 Not queried
 
  0.5523576
 
       NA
 
           NA
  
 
  
 10
 
   8096669
 
   8097354
 
  ENSEMBL   UCSC 
 
  686
 
  6
 
 4489
 
 gene
 
 0.021027
 
 0.0391963
 
 Not queried
 
  0.5523480
 
       NA
 
           NA
  
 
  
 11
 
  94964236
 
  94964516
 
  ENSEMBL   UCSC 
 
  281
 
  7
 
 8747
 
 island
 
 0.005995
 
 0.0067531
 
 Not queried
 
  0.5523447
 
       NA
 
           NA
  
 
  
 3
 
  33138421
 
  33138627
 
  ENSEMBL   UCSC 
 
  207
 
  6
 
 28209
 
 tss
 
 0.017514
 
 0.0119818
 
 Not queried
 
  0.5523271
 
       NA
 
           NA
  
 
  
 11
 
 111385338
 
 111385778
 
  ENSEMBL   UCSC 
 
  441
 
  7
 
 8801
 
 island
 
 0.022049
 
 0.0150263
 
 Not queried
 
  0.5521450
 
       NA
 
           NA
  
 
  
 1
 
 119531625
 
 119532925
 
  ENSEMBL   UCSC 
 
 1301
 
 17
 
 3657
 
 island
 
 0.054377
 
 0.1364610
 
 Not queried
 
  0.5521338
 
       NA
 
           NA
  
 
  
 13
 
  79169714
 
  79170627
 
  ENSEMBL   UCSC 
 
  914
 
 10
 
 11792
 
 island
 
 0.193485
 
 0.2622704
 
 Not queried
 
  0.5521336
 
       NA
 
           NA
  
 
  
 12
 
 132413387
 
 132413779
 
  ENSEMBL   UCSC 
 
  393
 
  5
 
 10125
 
 tss;island
 
 0.016221
 
 0.0066510
 
 Not queried
 
  0.5521213
 
       NA
 
           NA
  
 
  
 20
 
  57581903
 
  57582292
 
  ENSEMBL   UCSC 
 
  390
 
  6
 
 25478
 
 gene
 
 0.005983
 
 0.0142758
 
 Not queried
 
  0.5520059
 
       NA
 
           NA
  
 
  
 1
 
  45205136
 
  45205518
 
  ENSEMBL   UCSC 
 
  383
 
  9
 
 3187
 
 island
 
 0.001451
 
 0.0022985
 
 Not queried
 
  0.5519477
 
       NA
 
           NA
  
 
  
 1
 
  63788184
 
  63788557
 
  ENSEMBL   UCSC 
 
  374
 
  5
 
 1668
 
 tss;island
 
 0.013669
 
 0.0139399
 
 Not queried
 
  0.5518839
 
       NA
 
           NA
  
 
  
 16
 
  55090590
 
  55090946
 
  ENSEMBL   UCSC 
 
  357
 
  5
 
 15933
 
 island
 
 0.085343
 
 0.0897980
 
 Not queried
 
  0.5518620
 
       NA
 
           NA
  
 
  
 14
 
  77422738
 
  77423234
 
  ENSEMBL   UCSC 
 
  497
 
  6
 
 13027
 
 island
 
 0.008197
 
 0.0077826
 
 Not queried
 
  0.5518382
 
       NA
 
           NA
  
 
  
 12
 
 123319894
 
 123320057
 
  ENSEMBL   UCSC 
 
  164
 
  5
 
 10934
 
 island
 
 0.009003
 
 0.0152918
 
 Not queried
 
  0.5517458
 
       NA
 
           NA
  
 
  
 13
 
  73634056
 
  73634670
 
  ENSEMBL   UCSC 
 
  615
 
  7
 
 11180
 
 gene;island
 
 0.006429
 
 0.0120064
 
 Not queried
 
  0.5516996
 
       NA
 
           NA
  
 
  
 13
 
 111214096
 
 111214246
 
  ENSEMBL   UCSC 
 
  151
 
  6
 
 11533
 
 tss;island
 
 0.014270
 
 0.0131946
 
 Not queried
 
  0.5516833
 
       NA
 
           NA
  
 
  
 3
 
  48487808
 
  48488159
 
  ENSEMBL   UCSC 
 
  352
 
  6
 
 28299
 
 tss
 
 0.018636
 
 0.0114108
 
 Not queried
 
  0.5516624
 
       NA
 
           NA
  
 
  
 3
 
 101292685
 
 101293208
 
  ENSEMBL   UCSC 
 
  524
 
 10
 
 29332
 
 island
 
 0.008638
 
 0.0053004
 
 Not queried
 
  0.5514939
 
       NA
 
           NA
  
 
  
 8
 
  86375919
 
  86376489
 
  ENSEMBL   UCSC 
 
  571
 
  5
 
 40433
 
 island
 
 0.009132
 
 0.0097230
 
 Not queried
 
  0.5514353
 
       NA
 
           NA
  
 
  
 13
 
  53422777
 
  53423262
 
  ENSEMBL   UCSC 
 
  486
 
 10
 
 11465
 
 tss
 
 0.067124
 
 0.0418544
 
 Not queried
 
  0.5513162
 
       NA
 
           NA
  
 
  
 11
 
   8932751
 
   8933103
 
  ENSEMBL   UCSC 
 
  353
 
  4
 
 6502
 
 gene
 
 0.035026
 
 0.0270475
 
 Not queried
 
  0.5512026
 
       NA
 
           NA
  
 
  
 2
 
 106810978
 
 106811174
 
  ENSEMBL   UCSC 
 
  197
 
  7
 
 23557
 
 tss;island
 
 0.003863
 
 0.0047813
 
 Not queried
 
  0.5511445
 
       NA
 
           NA
  
 
  
 14
 
  93673175
 
  93673304
 
  ENSEMBL   UCSC 
 
  130
 
  4
 
 12186
 
 gene;tss;island
 
 0.007159
 
 0.0117977
 
 Not queried
 
  0.5510896
 
       NA
 
           NA
  
 
  
 20
 
  30433233
 
  30433673
 
  ENSEMBL   UCSC 
 
  441
 
  5
 
 26082
 
 island
 
 0.537826
 
 0.4733164
 
 Not queried
 
  0.5510863
 
       NA
 
           NA
  
 
  
 8
 
   9413342
 
   9413415
 
  ENSEMBL   UCSC 
 
   74
 
  5
 
 39544
 
 tss
 
 0.010037
 
 0.0035118
 
 Not queried
 
  0.5510206
 
       NA
 
           NA
  
 
  
 8
 
   9413342
 
   9413457
 
  ENSEMBL   UCSC 
 
  116
 
  7
 
 40062
 
 island
 
 0.010037
 
 0.0035118
 
 Not queried
 
  0.5510206
 
       NA
 
           NA
  
 
  
 2
 
 170551086
 
 170551352
 
  ENSEMBL   UCSC 
 
  267
 
  7
 
 22916
 
 gene
 
 0.005172
 
 0.0081939
 
 Not queried
 
  0.5509928
 
       NA
 
           NA
  
 
  
 2
 
 202645908
 
 202646121
 
  ENSEMBL   UCSC 
 
  214
 
  7
 
 23822
 
 tss
 
 0.003482
 
 0.0022007
 
 Not queried
 
  0.5507898
 
       NA
 
           NA
  
 
  
 6
 
  29697700
 
  29697949
 
  ENSEMBL   UCSC 
 
  250
 
  4
 
 33688
 
 gene
 
 0.976334
 
 0.9858344
 
 Not queried
 
  0.5507133
 
       NA
 
           NA
  
 
  
 11
 
  64808285
 
  64808367
 
  ENSEMBL   UCSC 
 
   83
 
  5
 
 7411
 
 tss
 
 0.012231
 
 0.0082924
 
 Not queried
 
  0.5506856
 
       NA
 
           NA
  
 
  
 11
 
  65383415
 
  65383829
 
  ENSEMBL   UCSC 
 
  415
 
  5
 
 8471
 
 island
 
 0.005947
 
 0.0058273
 
 Not queried
 
  0.5506628
 
       NA
 
           NA
  
 
  
 10
 
  70660920
 
  70661008
 
  ENSEMBL   UCSC 
 
   89
 
  6
 
 5118
 
 tss
 
 0.010545
 
 0.0018040
 
 Not queried
 
  0.5505826
 
       NA
 
           NA
  
 
  
 10
 
    387348
 
    387543
 
  ENSEMBL   UCSC 
 
  196
 
  4
 
 4434
 
 gene;island
 
 0.875836
 
 0.9150008
 
 Not queried
 
  0.5505424
 
       NA
 
           NA
  
 
  
 11
 
 111944731
 
 111945650
 
  ENSEMBL   UCSC 
 
  920
 
 14
 
 7697
 
 tss;island
 
 0.004979
 
 0.0035321
 
 Not queried
 
  0.5504915
 
       NA
 
           NA
  
 
  
 22
 
  44568699
 
  44568812
 
  ENSEMBL   UCSC 
 
  114
 
  6
 
 27210
 
 tss
 
 0.053693
 
 0.0910031
 
 Not queried
 
  0.5504623
 
       NA
 
           NA
  
 
  
 8
 
 125486586
 
 125486985
 
  ENSEMBL   UCSC 
 
  400
 
  8
 
 39905
 
 tss
 
 0.021795
 
 0.0157110
 
 Not queried
 
  0.5503422
 
       NA
 
           NA
  
 
  
 8
 
 125486586
 
 125487057
 
  ENSEMBL   UCSC 
 
  472
 
 10
 
 40582
 
 island
 
 0.016256
 
 0.0128149
 
 Not queried
 
  0.5503422
 
       NA
 
           NA
  
 
  
 12
 
 133066471
 
 133066762
 
  ENSEMBL   UCSC 
 
  292
 
 14
 
 10131
 
 tss;island
 
 0.005990
 
 0.0074234
 
 Not queried
 
  0.5503401
 
       NA
 
           NA
  
 
  
 20
 
  60795362
 
  60795465
 
  ENSEMBL   UCSC 
 
  104
 
  4
 
 25866
 
 tss;island
 
 0.034808
 
 0.0229734
 
 Not queried
 
  0.5502610
 
       NA
 
           NA
  
 
  
 6
 
   7313605
 
   7313731
 
  ENSEMBL   UCSC 
 
  127
 
  5
 
 34612
 
 tss
 
 0.014376
 
 0.0079541
 
 Not queried
 
  0.5501562
 
       NA
 
           NA
  
 
  
 12
 
 123464511
 
 123464823
 
  ENSEMBL   UCSC 
 
  313
 
  5
 
 10095
 
 tss
 
 0.019151
 
 0.0188563
 
 Not queried
 
  0.5501282
 
       NA
 
           NA
  
 
  
 11
 
    506809
 
    507177
 
  ENSEMBL   UCSC 
 
  369
 
  8
 
 6378
 
 gene
 
 0.005163
 
 0.0046403
 
 Not queried
 
  0.5500958
 
       NA
 
           NA
  
 
  
 1
 
 178994930
 
 178995133
 
  ENSEMBL   UCSC 
 
  204
 
  6
 
 3991
 
 island
 
 0.008313
 
 0.0059926
 
 Not queried
 
  0.5500639
 
       NA
 
           NA
  
 
  
 10
 
 118429481
 
 118429952
 
  ENSEMBL   UCSC 
 
  472
 
  7
 
 5386
 
 tss
 
 0.135249
 
 0.4315539
 
 Not queried
 
  0.5500495
 
       NA
 
           NA
  
 
  
 1
 
 150336450
 
 150336971
 
  ENSEMBL   UCSC 
 
  522
 
 10
 
 1907
 
 tss
 
 0.010243
 
 0.0061521
 
 Not queried
 
  0.5499390
 
       NA
 
           NA
  
 
  
 17
 
   7761198
 
   7761459
 
  ENSEMBL   UCSC 
 
  262
 
  5
 
 17101
 
 tss
 
 0.011391
 
 0.0059374
 
 Not queried
 
  0.5499300
 
       NA
 
           NA
  
 
  
 17
 
  59940826
 
  59941132
 
  ENSEMBL   UCSC 
 
  307
 
  7
 
 17559
 
 tss
 
 0.005994
 
 0.0039891
 
 Not queried
 
  0.5499037
 
       NA
 
           NA
  
 
  
 18
 
  32847251
 
  32847789
 
  ENSEMBL   UCSC 
 
  539
 
  5
 
 19089
 
 gene;island
 
 0.024822
 
 0.0234007
 
 Not queried
 
  0.5497963
 
       NA
 
           NA
  
 
  
 1
 
  28585612
 
  28586105
 
  ENSEMBL   UCSC 
 
  494
 
  7
 
 2941
 
 island
 
 0.006445
 
 0.0108328
 
 Not queried
 
  0.5497411
 
       NA
 
           NA
  
 
  
 16
 
   4852825
 
   4853175
 
  ENSEMBL   UCSC 
 
  351
 
  7
 
 15077
 
 tss;island
 
 0.006086
 
 0.0036142
 
 Not queried
 
  0.5497247
 
       NA
 
           NA
  
 
  
 1
 
  16482430
 
  16482818
 
  ENSEMBL   UCSC 
 
  389
 
  6
 
 2739
 
 island
 
 0.027555
 
 0.0608084
 
 Not queried
 
  0.5497088
 
       NA
 
           NA
  
 
  
 7
 
 130080846
 
 130081239
 
  ENSEMBL   UCSC 
 
  394
 
 10
 
 38920
 
 island
 
 0.007625
 
 0.0097001
 
 Not queried
 
  0.5496259
 
       NA
 
           NA
  
 
  
 2
 
  85637673
 
  85637851
 
  ENSEMBL   UCSC 
 
  179
 
  4
 
 24477
 
 island
 
 0.511572
 
 0.5134491
 
 Not queried
 
  0.5496246
 
       NA
 
           NA
  
 
  
 17
 
   8868541
 
   8868834
 
  ENSEMBL   UCSC 
 
  294
 
  4
 
 16435
 
 gene;island
 
 0.116069
 
 0.0777985
 
 Not queried
 
  0.5494943
 
       NA
 
           NA
  
 
  
 10
 
  75407372
 
  75407991
 
  ENSEMBL   UCSC 
 
  620
 
  6
 
 4636
 
 gene;island
 
 0.068298
 
 0.1658169
 
 Not queried
 
  0.5494690
 
       NA
 
           NA
  
 
  
 21
 
  47813364
 
  47813491
 
  ENSEMBL   UCSC 
 
  128
 
  4
 
 26476
 
 gene;island
 
 0.960008
 
 0.9632980
 
 Not queried
 
  0.5494611
 
       NA
 
           NA
  
 
  
 17
 
   7516888
 
   7517258
 
  ENSEMBL   UCSC 
 
  371
 
  6
 
 16404
 
 gene;tss;island
 
 0.765447
 
 0.8322024
 
 Not queried
 
  0.5493486
 
       NA
 
           NA
  
 
  
 22
 
  29702766
 
  29702866
 
  ENSEMBL   UCSC 
 
  101
 
  7
 
 27046
 
 tss;island
 
 0.104234
 
 0.0182421
 
 Not queried
 
  0.5492811
 
       NA
 
           NA
  
 
  
 20
 
  45985495
 
  45985741
 
  ENSEMBL   UCSC 
 
  247
 
  7
 
 25799
 
 tss
 
 0.005985
 
 0.0095111
 
 Not queried
 
  0.5491869
 
       NA
 
           NA
  
 
  
 22
 
  51066755
 
  51066924
 
  ENSEMBL   UCSC 
 
  170
 
  7
 
 27263
 
 tss;island
 
 0.006888
 
 0.0063474
 
 Not queried
 
  0.5490618
 
       NA
 
           NA
  
 
  
 6
 
  33244709
 
  33244799
 
  ENSEMBL   UCSC 
 
   91
 
  7
 
 34898
 
 tss
 
 0.007022
 
 0.0058415
 
 Not queried
 
  0.5490210
 
       NA
 
           NA
  
 
  
 12
 
  14926572
 
  14927245
 
  ENSEMBL   UCSC 
 
  674
 
  9
 
 9586
 
 tss
 
 0.012605
 
 0.0120851
 
 Not queried
 
  0.5488166
 
       NA
 
           NA
  
 
  
 17
 
  18011879
 
  18012014
 
  ENSEMBL   UCSC 
 
  136
 
  5
 
 17172
 
 tss
 
 0.911392
 
 0.9073293
 
 Not queried
 
  0.5487263
 
       NA
 
           NA
  
 
  
 14
 
  50087093
 
  50087325
 
  ENSEMBL   UCSC 
 
  233
 
  5
 
 12077
 
 gene
 
 0.005031
 
 0.0055528
 
 Not queried
 
  0.5486171
 
       NA
 
           NA
  
 
  
 15
 
  64679818
 
  64680295
 
  ENSEMBL   UCSC 
 
  478
 
  6
 
 14215
 
 island
 
 0.002281
 
 0.0096891
 
 Not queried
 
  0.5485501
 
       NA
 
           NA
  
 
  
 1
 
  92683403
 
  92683554
 
  ENSEMBL   UCSC 
 
  152
 
  5
 
 1748
 
 tss
 
 0.831758
 
 0.9300409
 
 Not queried
 
  0.5485071
 
       NA
 
           NA
  
 
  
 19
 
   5977966
 
   5978281
 
  ENSEMBL   UCSC 
 
  316
 
  4
 
 19758
 
 gene
 
 0.001160
 
 0.0016964
 
 Not queried
 
  0.5484755
 
       NA
 
           NA
  
 
  
 14
 
  80328091
 
  80328595
 
  ENSEMBL   UCSC 
 
  505
 
  5
 
 12160
 
 gene;island
 
 0.607445
 
 0.8109902
 
 Not queried
 
  0.5484286
 
       NA
 
           NA
  
 
  
 7
 
 122527351
 
 122527522
 
  ENSEMBL   UCSC 
 
  172
 
  5
 
 37868
 
 tss;island
 
 0.028831
 
 0.0764947
 
 Not queried
 
  0.5483178
 
       NA
 
           NA
  
 
  
 10
 
 134460304
 
 134460468
 
  ENSEMBL   UCSC 
 
  165
 
  4
 
 4871
 
 gene;island
 
 0.958650
 
 0.9748895
 
 Not queried
 
  0.5482697
 
       NA
 
           NA
  
 
  
 18
 
  19180767
 
  19181025
 
  ENSEMBL   UCSC 
 
  259
 
  8
 
 19198
 
 tss
 
 0.003178
 
 0.0077842
 
 Not queried
 
  0.5482403
 
       NA
 
           NA
  
 
  
 1
 
   2259065
 
   2259411
 
  ENSEMBL   UCSC 
 
  347
 
  5
 
 74
 
 gene;island
 
 0.485679
 
 0.5799791
 
 Not queried
 
  0.5482305
 
       NA
 
           NA
  
 
  
 12
 
 129308602
 
 129308719
 
  ENSEMBL   UCSC 
 
  118
 
  5
 
 10114
 
 tss
 
 0.003567
 
 0.0031544
 
 Not queried
 
  0.5482235
 
       NA
 
           NA
  
 
  
 1
 
   2322827
 
   2323201
 
  ENSEMBL   UCSC 
 
  375
 
  6
 
 1170
 
 tss;island
 
 0.012789
 
 0.0115228
 
 Not queried
 
  0.5481924
 
       NA
 
           NA
  
 
  
 19
 
   6463949
 
   6464275
 
  ENSEMBL   UCSC 
 
  327
 
 10
 
 21430
 
 island
 
 0.096324
 
 0.1272851
 
 Not queried
 
  0.5481445
 
       NA
 
           NA
  
 
  
 19
 
  40562144
 
  40562354
 
  ENSEMBL   UCSC 
 
  211
 
  5
 
 20780
 
 tss
 
 0.004069
 
 0.0078205
 
 Not queried
 
  0.5481410
 
       NA
 
           NA
  
 
  
 10
 
 121356679
 
 121356865
 
  ENSEMBL   UCSC 
 
  187
 
  8
 
 5401
 
 tss
 
 0.015793
 
 0.0173723
 
 Not queried
 
  0.5481128
 
       NA
 
           NA
  
 
  
 8
 
  79428434
 
  79428725
 
  ENSEMBL   UCSC 
 
  292
 
  4
 
 39346
 
 gene
 
 0.004201
 
 0.0044571
 
 Not queried
 
  0.5481126
 
       NA
 
           NA
  
 
  
 6
 
 134638742
 
 134639482
 
  ENSEMBL   UCSC 
 
  741
 
 10
 
 36495
 
 island
 
 0.037175
 
 0.0400016
 
 Not queried
 
  0.5480779
 
       NA
 
           NA
  
 
  
 15
 
  22955254
 
  22956212
 
  ENSEMBL   UCSC 
 
  959
 
  9
 
 13251
 
 gene
 
 0.942414
 
 0.9599293
 
 Not queried
 
  0.5479379
 
       NA
 
           NA
  
 
  
 5
 
  60627852
 
  60628093
 
  ENSEMBL   UCSC 
 
  242
 
  6
 
 32083
 
 tss;island
 
 0.002448
 
 0.0045490
 
 Not queried
 
  0.5478595
 
       NA
 
           NA
  
 
  
 18
 
  61089605
 
  61090076
 
  ENSEMBL   UCSC 
 
  472
 
  9
 
 19557
 
 island
 
 0.006453
 
 0.0067234
 
 Not queried
 
  0.5478205
 
       NA
 
           NA
  
 
  
 11
 
 125366238
 
 125366590
 
  ENSEMBL   UCSC 
 
  353
 
  9
 
 7791
 
 tss;island
 
 0.103929
 
 0.4729718
 
 Not queried
 
  0.5478035
 
       NA
 
           NA
  
 
  
 22
 
  19467283
 
  19467461
 
  ENSEMBL   UCSC 
 
  179
 
  6
 
 26960
 
 tss;island
 
 0.004874
 
 0.0029075
 
 Not queried
 
  0.5477711
 
       NA
 
           NA
  
 
  
 14
 
  35183905
 
  35184330
 
  ENSEMBL   UCSC 
 
  426
 
  7
 
 12348
 
 tss;island
 
 0.004835
 
 0.0034923
 
 Not queried
 
  0.5475700
 
       NA
 
           NA
  
 
  
 7
 
 101712087
 
 101712363
 
  ENSEMBL   UCSC 
 
  277
 
  4
 
 37160
 
 gene
 
 0.976239
 
 0.8661334
 
 Not queried
 
  0.5475510
 
       NA
 
           NA
  
 
  
 2
 
 109558966
 
 109559185
 
  ENSEMBL   UCSC 
 
  220
 
  4
 
 22821
 
 gene;island
 
 0.650482
 
 0.7155423
 
 Not queried
 
  0.5474907
 
       NA
 
           NA
  
 
  
 11
 
  76777529
 
  76777997
 
  ENSEMBL   UCSC 
 
  469
 
  9
 
 8678
 
 island
 
 0.012895
 
 0.0092293
 
 Not queried
 
  0.5474687
 
       NA
 
           NA
  
 
  
 3
 
 111697479
 
 111697816
 
  ENSEMBL   UCSC 
 
  338
 
  9
 
 28497
 
 tss
 
 0.011788
 
 0.0233493
 
 Not queried
 
  0.5474490
 
       NA
 
           NA
  
 
  
 3
 
 111697479
 
 111698265
 
  ENSEMBL   UCSC 
 
  787
 
 13
 
 29356
 
 island
 
 0.007550
 
 0.0156128
 
 Not queried
 
  0.5474490
 
       NA
 
           NA
  
 
  
 22
 
  39052386
 
  39052759
 
  ENSEMBL   UCSC 
 
  374
 
 11
 
 26871
 
 gene;tss;island
 
 0.003762
 
 0.0047606
 
 Not queried
 
  0.5472406
 
       NA
 
           NA
  
 
  
 7
 
  86689097
 
  86689388
 
  ENSEMBL   UCSC 
 
  292
 
  5
 
 37681
 
 tss
 
 0.004915
 
 0.0109495
 
 Not queried
 
  0.5472249
 
       NA
 
           NA
  
 
  
 13
 
  21635722
 
  21635991
 
  ENSEMBL   UCSC 
 
  270
 
  8
 
 11335
 
 tss
 
 0.010730
 
 0.0082125
 
 Not queried
 
  0.5472102
 
       NA
 
           NA
  
 
  
 11
 
   8986187
 
   8986674
 
  ENSEMBL   UCSC 
 
  488
 
  5
 
 7131
 
 tss;island
 
 0.008951
 
 0.0028879
 
 Not queried
 
  0.5472054
 
       NA
 
           NA
  
 
  
 14
 
  69446504
 
  69446784
 
  ENSEMBL   UCSC 
 
  281
 
  4
 
 12477
 
 tss;island
 
 0.026366
 
 0.0135826
 
 Not queried
 
  0.5470581
 
       NA
 
           NA
  
 
  
 17
 
   1613831
 
   1613939
 
  ENSEMBL   UCSC 
 
  109
 
  5
 
 16992
 
 tss;island
 
 0.107235
 
 0.0448463
 
 Not queried
 
  0.5469965
 
       NA
 
           NA
  
 
  
 4
 
  15704393
 
  15704550
 
  ENSEMBL   UCSC 
 
  158
 
  4
 
 30311
 
 tss
 
 0.035273
 
 0.1691261
 
 Not queried
 
  0.5469567
 
       NA
 
           NA
  
 
  
 6
 
  31549480
 
  31549631
 
  ENSEMBL   UCSC 
 
  152
 
  4
 
 33906
 
 gene;island
 
 0.024938
 
 0.0236049
 
 Not queried
 
  0.5469552
 
       NA
 
           NA
  
 
  
 22
 
  41940355
 
  41940855
 
  ENSEMBL   UCSC 
 
  501
 
  9
 
 27523
 
 island
 
 0.004323
 
 0.0028380
 
 Not queried
 
  0.5469392
 
       NA
 
           NA
  
 
  
 1
 
  27816300
 
  27816661
 
  ENSEMBL   UCSC 
 
  362
 
  7
 
 355
 
 gene
 
 0.006777
 
 0.0056087
 
 Not queried
 
  0.5468853
 
       NA
 
           NA
  
 
  
 5
 
 137474700
 
 137475115
 
  ENSEMBL   UCSC 
 
  416
 
  5
 
 31764
 
 gene
 
 0.018780
 
 0.0178258
 
 Not queried
 
  0.5468827
 
       NA
 
           NA
  
 
  
 1
 
  63782980
 
  63783167
 
  ENSEMBL   UCSC 
 
  188
 
  4
 
 3339
 
 island
 
 0.004680
 
 0.0048765
 
 Not queried
 
  0.5468075
 
       NA
 
           NA
  
 
  
 5
 
  52083512
 
  52084055
 
  ENSEMBL   UCSC 
 
  544
 
 13
 
 32058
 
 tss;island
 
 0.018657
 
 0.0095262
 
 Not queried
 
  0.5467769
 
       NA
 
           NA
  
 
  
 14
 
  74769604
 
  74769859
 
  ENSEMBL   UCSC 
 
  256
 
  5
 
 12999
 
 island
 
 0.003509
 
 0.0028236
 
 Not queried
 
  0.5467734
 
       NA
 
           NA
  
 
  
 17
 
  25798741
 
  25799018
 
  ENSEMBL   UCSC 
 
  278
 
  5
 
 17205
 
 tss
 
 0.020109
 
 0.0143402
 
 Not queried
 
  0.5467136
 
       NA
 
           NA
  
 
  
 2
 
 128615778
 
 128616167
 
  ENSEMBL   UCSC 
 
  390
 
 10
 
 23618
 
 tss
 
 0.013489
 
 0.0130073
 
 Not queried
 
  0.5465663
 
       NA
 
           NA
  
 
  
 5
 
 139227979
 
 139228242
 
  ENSEMBL   UCSC 
 
  264
 
  5
 
 31781
 
 gene;island
 
 0.017236
 
 0.0050034
 
 Not queried
 
  0.5465620
 
       NA
 
           NA
  
 
  
 7
 
  27224092
 
  27225897
 
  ENSEMBL   UCSC 
 
 1806
 
 34
 
 37499
 
 tss;island
 
 0.060116
 
 0.0674490
 
 Not queried
 
  0.5465587
 
       NA
 
           NA
  
 
  
 7
 
  35226450
 
  35226751
 
  ENSEMBL   UCSC 
 
  302
 
  4
 
 38406
 
 island
 
 0.037176
 
 0.2317785
 
 Not queried
 
  0.5464257
 
       NA
 
           NA
  
 
  
 1
 
   2716877
 
   2717193
 
  ENSEMBL   UCSC 
 
  317
 
  4
 
 2517
 
 island
 
 0.837754
 
 0.9132749
 
 Not queried
 
  0.5463978
 
       NA
 
           NA
  
 
  
 11
 
 117747822
 
 117748236
 
  ENSEMBL   UCSC 
 
  415
 
  9
 
 7723
 
 tss
 
 0.476545
 
 0.8448477
 
 Not queried
 
  0.5463040
 
       NA
 
           NA
  
 
  
 11
 
  65188784
 
  65189075
 
  ENSEMBL   UCSC 
 
  292
 
  4
 
 7428
 
 tss;island
 
 0.003931
[truncated: 3,148,006 more chars]
